# Causal Association of Plasma Lipidome With Inflammatory Bowel Diseases and Mediating Role of Circulating Inflammatory Proteins: A Mendelian Randomization Study

**DOI:** 10.1002/fsn3.70916

**Published:** 2025-09-10

**Authors:** Linlin Yin, Yue Zhu, Fang Kong, Hongfei Tu, Bin Zhang

**Affiliations:** ^1^ Department of Digestive Diseases The Second Hospital of Nanjing, Affiliated to Nanjing University of Chinese Medicine Nanjing China

**Keywords:** circulating inflammatory proteins, inflammatory bowel diseases, mendelian randomization, plasma lipidome

## Abstract

The existing studies indicate that the lipidome may be associated with inflammatory bowel disease (IBD). However, most of the results of previous studies are based on epidemiological or observational studies. Meanwhile, the causal association between different lipids and inflammatory bowel diseases, as well as whether inflammatory proteins act as mediators, remains unclear. We employed Mendelian randomized (MR) analysis to investigate the causal relationship between lipidome and IBD (ulcerative colitis and Crohn's disease). We mainly used the IVW method for MR analysis and further evaluated heterogeneity and pleiotropy. Additionally, we explored whether circulating inflammatory proteins play a mediating role in the pathway from the lipidome to IBD. In our MR study, we identified causal relationships between four classes of lipids (sterol ester, phosphatidylcholine, sphingomyelin, and phosphatidylethanolamine) and IBD. The risk of IBD is positively associated with the levels of 6 inflammatory proteins (CCL19, CCL4, CD5, CD6, IL‐10α, and TNFSF12). In the mediation analysis, we identified two circulating inflammatory proteins (CD6, CCL4) that play a mediating role between the lipidome and IBD. Plasma lipidome was causally associated with IBD, and circulating inflammatory proteins serve as mediating factors in the pathway from lipidome to IBD.

## Graphical abstract

A Mendelian randomization study revealed a causal relationship between Sterol Ester, Phosphatidylcholine, Sphingomyelin, Phosphatidylethanolamine, and IBD from a genetic perspective. Additionally, CD6 and CCL4 played mediating roles in the association between lipidome and IBD. These findings may bring new targets and directions for the treatment of IBD.

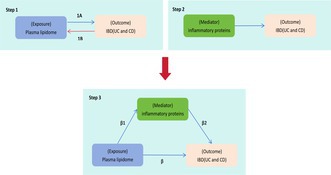

## 1 Introduction

Over the past few decades, the global burden of inflammatory bowel disease (IBD), which includes Crohn's disease (CD) and ulcerative colitis (UC), has risen markedly. Once considered a condition predominantly affecting Western populations, IBD is now experiencing a rapid increase in incidence across newly industrialized regions such as Asia and Latin America (Hracs et al. 2025). This global upward trend is largely attributed to environmental changes associated with industrialization, including urbanization, shifts toward high‐fat, low‐fiber Westernized diets, and reduced microbial exposure (Adolph et al. 2022; Kaplan and Ng 2017). Projections estimate that the prevalence of IBD in developed regions such as Europe and North America will continue to rise by approximately 30% over the next decade (Coward et al. 2019; Jones et al. 2019). Similarly, many non‐Western countries are expected to see a surge in IBD prevalence, potentially approaching the levels historically observed in high‐incidence areas (Kaplan and Windsor 2021). As a chronic, incurable disease, the increasing global prevalence of IBD is expected to impose a significant and growing burden on healthcare systems and society (Kaplan 2015).

The traditional lipid measurements in plasma include total cholesterol, high‐density lipoprotein cholesterol, low‐density lipoprotein cholesterol, and triglycerides. With the development of new lipidomic technologies, our understanding of the plasma lipidome has been enhanced. Currently, lipids are believed to include cholesteryl esters, ceramides, phosphatidylethanolamines, lysophosphatidylethanolamines, lysophosphatidylcholines, phosphatidylcholines, phosphatidylcholine‐ether, phosphatidylethanolamine‐ethers, sphingomyelins, diacylglycerols, and triacylglycerols (Ottensmann et al. 2023). Lipids constitute a vital and highly diverse class of molecules, serving as essential nutrients in the body. They play critical roles in cell structure, intracellular and extracellular signaling, as well as biological energy provision (Hornburg et al. 2023). Related studies indicate the relevance of the lipidome in inflammatory bowel diseases, playing crucial roles in the initiation, progression, inflammatory activities, and resolution of the diseases (Yang et al. 2022; Fan et al. 2015; Iwatani et al. 2020). Similarly, lipids are integral in modulating inflammatory cytokines within the body, demonstrating distinct interactions with a variety of intracellular cytokines, chemokines, and growth factors (Hornburg et al. 2023).

IBD refers to a nonspecific inflammation of the intestinal tract, and its occurrence is associated with the body's autoimmune response. Currently, various inflammatory cytokines have been implicated in the pathophysiological mechanisms and progression of IBD (Korta et al. 2023; da Silva Júnior et al. 2023). Lipids and inflammatory cytokines both play certain roles in the initiation and progression of IBD. Additionally, lipids have the ability to regulate various levels of cytokines within the body. Therefore, we hypothesized that inflammatory cytokines are the mediators of the lipidome regulating the occurrence and development of IBD.

In the past, the correlation between lipids and IBD was limited to the analysis of patients' lipidomics. This kind of observational analysis is difficult to avoid the influence of selection bias, bidirectional causality, and potential confounding variables. Therefore, we chose a new and more reliable method (MR analysis) to study the relationship between plasma lipids, circulating inflammatory proteins, and inflammatory bowel disease.

MR is a genetic epidemiological research tool that helps investigate the causes of disease. It uses the genetic variants identified from the GWAS summary data as instrumental variables (IVs) to assess the causal link between exposure factors and disease outcomes (Emdin et al. 2017). Mendelian randomization relies on the random allocation of genetic variations during sperm and ovum formation, which is similar to randomized controlled trials (Birney 2022). Besides, in the two‐sample MR analysis, the genetic variation data for both exposure and outcome are obtained from separate GWAS data sets, and the analysis results have a greater statistical effect and less bias. In our study, we employed two‐sample MR analysis to investigate the causal association between the lipidome and IBD (including CD and UC), while also examining the potential mediating role of inflammatory proteins in this relationship.

## 2 Methods

### 2.1 Study Design

**FIGURE 1:**
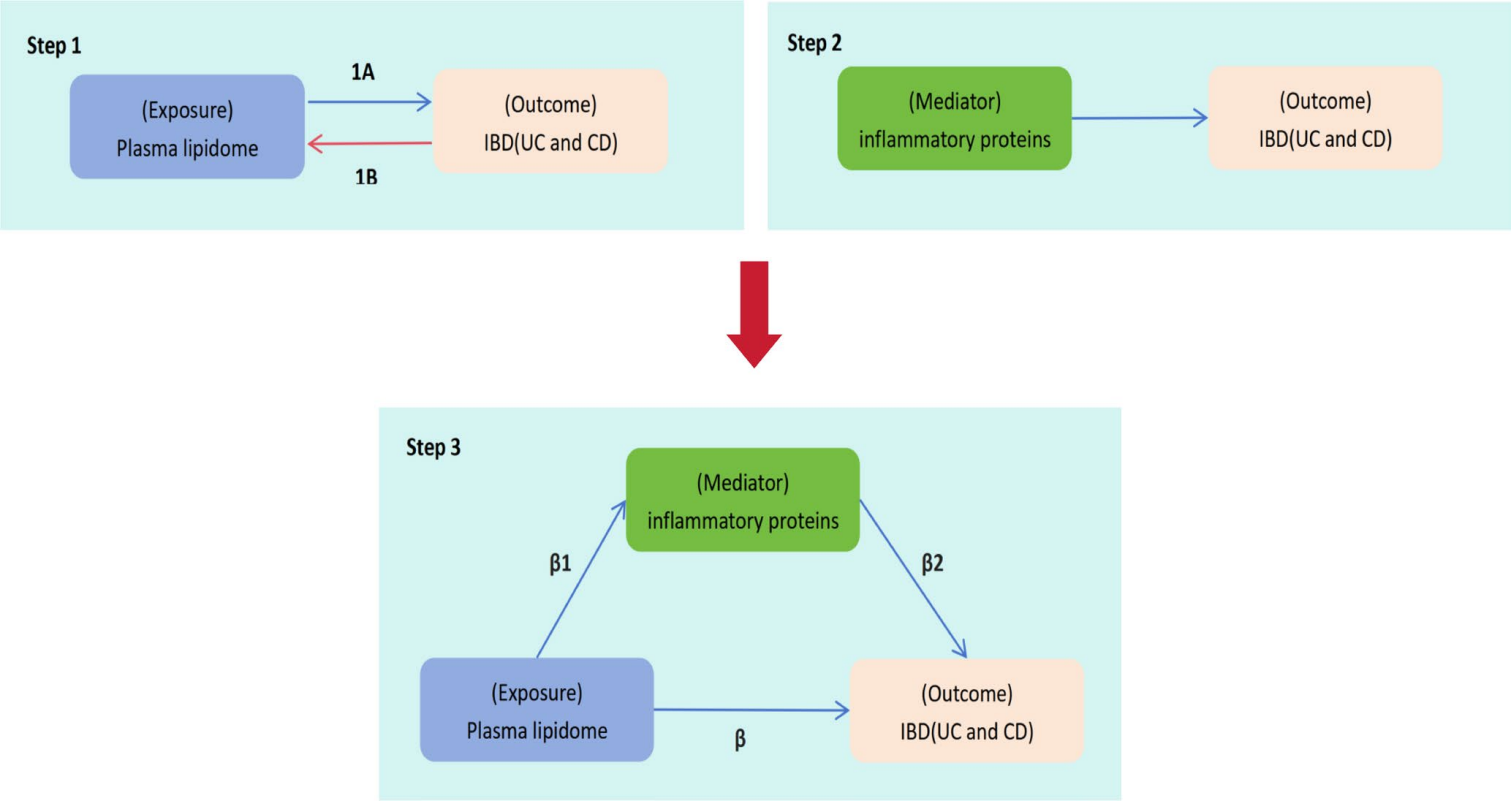
Design of MR analyses. Step 1A and B represents the bidirectional causal associations between lipidome (exposure) and IBD (outcome). Step 2 represents the causal effects of circulating inflammatory proteins (mediator) on IBD. Step 3 represents the mediation analysis of circulating inflammatory proteins in the pathway from the plasma lipidome to IBD.

Our research is mainly divided into the following steps (Figure 1): Step 1: identify the plasma lipidome that has a causal relationship with IBD, and analyze whether there is a reverse causal relationship between IBD and the plasma lipidome; Step 2: analyze the causal association between 91 inflammatory proteins and IBD, searching for potential mediating factors; Step 3: investigate the association between the lipidome and potential mediating inflammatory proteins to identify mediating factors in the lipidome‐to‐IBD pathway.

### 2.2 Data Source

The lipidome genetic data were sourced from the most recent GWAS summary statistics of the Finnish GeneRISK cohort, encompassing 13 lipid classes and 179 lipid species profiled across 7174 participants (Ottensmann et al. 2023). Genetic associations for 91 circulating inflammatory proteins were sourced from GWAS summary statistics across 11 independent cohorts, aggregating data from 14,824 participants (Zhao et al. 2023). GWAS summary statistics for IBD (IBD, CD, UC) are identified from the FinnGen study. Finally, our study comprises IBD (9083 cases vs. 403,098 controls), ulcerative colitis (5931 cases vs. 405,386 controls), and Crohn's disease (2033 cases vs. 409,940 controls).

### 2.3 Instrumental Variable Screening

We initially selected SNPs meeting the following selection criteria (*p* < 1 × 10^−5^; linkage disequilibrium (LD): *R* ^2^ < 0.001, kb = 10,000). Additionally, we excluded palindromic SNPs with intermediate allele frequencies. We also excluded IVs with *F*‐statistics < 10 to minimize potential bias from weak instruments (Burgess and Thompson 2011; Shim et al. 2015). To further validate the robustness of our MR results and minimize the likelihood of false‐positive associations, we repeated the MR analyses using a more stringent selection threshold of *p* < 1 × 10^−6^.

### 2.4 MR Analysis

#### 2.4.1 Preliminary Analysis

We initially assessed the causal relationship between the lipidome and IBD, as well as between inflammatory proteins and IBD, aiming to identify lipidome exposures closely associated with IBD and potentially mediating inflammatory proteins. The MR results were primarily analyzed using the inverse variance weighted (IVW) method. Furthermore, we implemented complementary analytical approaches, including weighted mode, MR‐Egger, simple mode, and weighted median, to rigorously evaluate the robustness of our causal estimates. To further corroborate causal associations with IVW *p*‐values < 0.05, we applied Bayesian Weighted Mendelian Randomization (BWMR) to reinforce the credibility of our causal inferences (Zhao et al. 2020). Meanwhile to account for multiple comparisons, *p*‐values derived from IVW analyses were adjusted using the Benjamini–Hochberg procedure to control the false discovery rate (FDR). A corrected FDR < 0.05 was considered statistically significant. When the *p*‐values of both FDR and BWMR were less than 0.05, and the directions were consistent across five MR analysis methods, it was considered statistically significant evidence for potential causal effects.

#### 2.4.2 Bidirectional MR

To evaluate bidirectional causality between IBD and the lipidome, bidirectional MR analyses were performed, treating IBD as the exposure and lipidome components associated with IBD as outcomes. We selected SNPs significantly associated with IBD (*p* < 5 × 10^−8^) as IVs.

#### 2.4.3 Mediation Analysis

A two‐step analytical framework was implemented to assess mediation effects, systematically evaluating potential intermediary pathways within the causal associations (Carter et al. 2021). First, we analyzed the associations between the lipidome and the mediator variable (inflammatory proteins) and calculated the corresponding causal effect (*β*1). Subsequently, we examined the relationship between the mediator variable (inflammatory proteins) and IBD, with a causal effect denoted as *β*2. The total causal effect between the lipidome and IBD is defined as *β*. At the same time, we calculated the mediation effect (*β*1 * *β*2), direct effect (*β*–*β*1 * *β*2), and the proportion of mediated effect (*β*1*β2/*β*) based on the values of *β*, *β*1, and *β*2.

#### 2.4.4 Sensitivity Analysis

We utilized Cochran's *Q* test to assess heterogeneity within IVs. We also assessed horizontal pleiotropy through the MR‐Egger intercept method and the MR‐PRESSO method. Considering that significant heterogeneity and horizontal pleiotropy can impact MR analysis results, to obtain more rigorous and reliable conclusions, we excluded results exhibiting heterogeneity and horizontal pleiotropy (*p* < 0.05).

We apply “TwoSampleMR” package and “MRPRESSO” package for MR analysis. All MR analyses are based on R (version 4.1.2).

## 3 Results

### 3.1 Instrumental Variables for MR Analysis

For the lipidome, through screening, we obtained 4499 significantly associated SNPs (*p* < 1 × 10^−5^) (Table S1). For mediating variables, 2842 SNPs associated with 91 inflammatory proteins (*p* < 1 × 10^−5^) were identified for MR analysis (Table S2). When IBD was considered as the exposure, we identified 58, 43, and 9 associated SNPs for IBD, UC, and CD, respectively, for conducting bidirectional causal analysis between IBD and the lipidome (*p* < 5 × 10^−8^) (Table S3).

Using a significance threshold of *p* < 1 × 10^−5^, we identified 19 lipids and 8 inflammatory proteins that exhibited causal associations with IBD, including UC and CD. Considering that the relatively lenient threshold of *p* < 1 × 10^−5^ may lead to spurious associations, we further validated our MR results using SNPs selected under a more stringent threshold of *p* < 1 × 10^−6^. After applying the threshold of *p* < 1 × 10^−6^, a total of 184 SNPs from 19 lipids and 61 SNPs from 8 inflammatory proteins were identified for MR analysis (Tables S4 and S5).

### 3.2 Causal Analysis Between Lipidome, Inflammatory Proteins, and IBD

#### 3.2.1 Inflammatory Bowel Disease

##### 3.2.1.1 Lipidome and IBD

**FIGURE 2:**
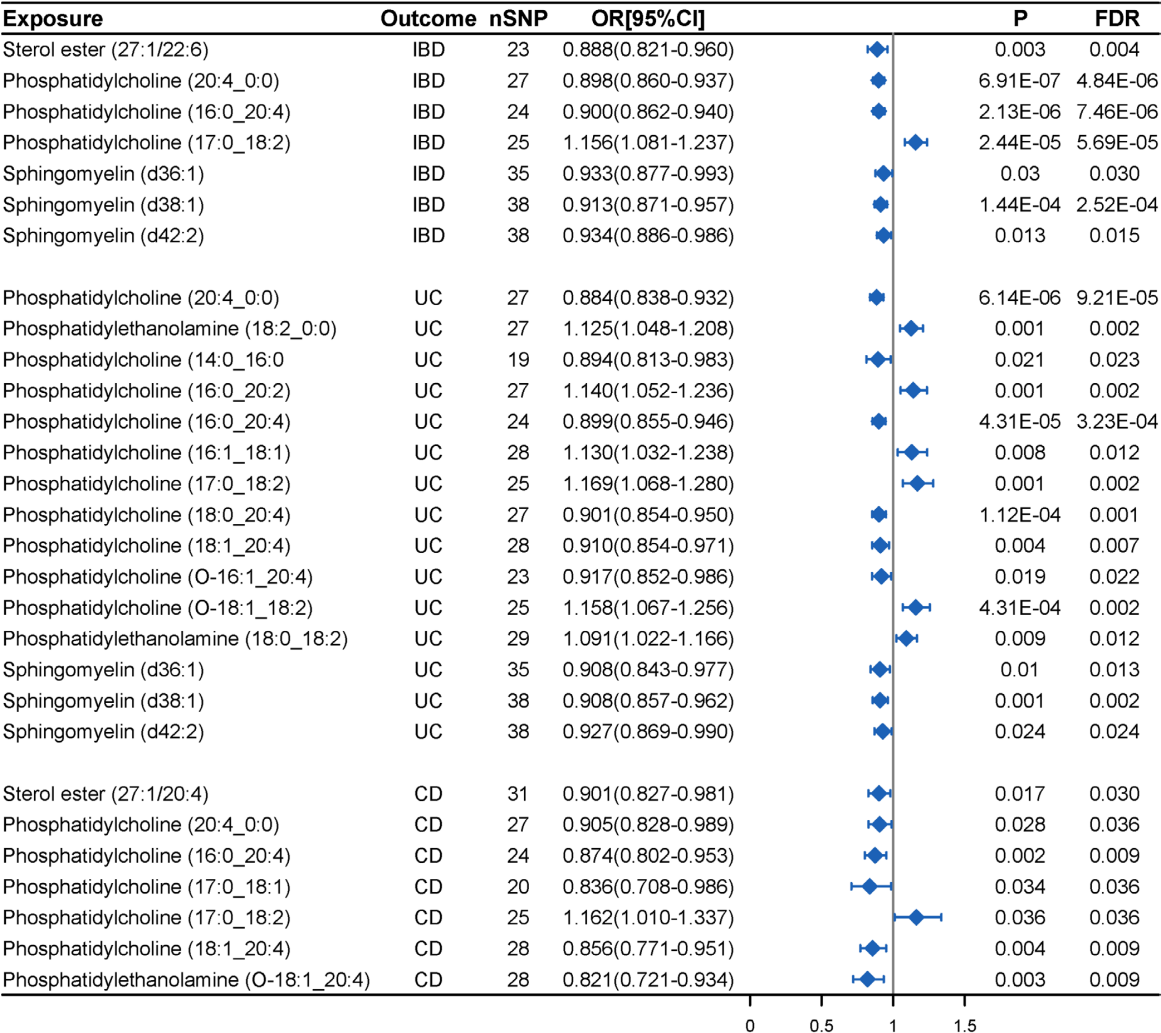
MR results of causal effects between plasma lipidome and IBD (UC and CD).

After the rigorous IVW screening, 36 lipids exhibited statistically significant associations with inflammatory bowel disease (IBD) (Table S6). Details on the association of relevant SNPs with IBD are given in Table S7. After further excluding lipids with heterogeneity, horizontal pleiotropy, inconsistent directions across the five MR analysis methods, and BWMR *p* > 0.05, we finally identified 7 lipids associated with IBD (Table S8). The MR analysis results revealed that 6 lipids [Sterol ester (27:1/22:6); Phosphatidylcholine (20:4_0:0); Phosphatidylcholine (16:0_20:4); Sphingomyelin (d36:1); Sphingomyelin (d38:1); Sphingomyelin (d42:2)] were associated with a reduced risk of IBD, while 1 lipid [Phosphatidylcholine (17:0_18:2)] was correlated with an increased risk of IBD (Figure 2, Table S8).

In the further validation analysis (SNP selection threshold: *p* < 1 × 10^−6^), 6 lipids [Sterol ester (27:1/22:6); Phosphatidylcholine (20:4_0:0); Phosphatidylcholine (16:0_20:4); Phosphatidylcholine (17:0_18:2); Sphingomyelin (d38:1); Sphingomyelin (d42:2)] remained significantly associated with IBD. However, no significant causal relationship was observed between Sphingomyelin (d36:1) and IBD (Figure S1, Table S9).

**FIGURE 3:**
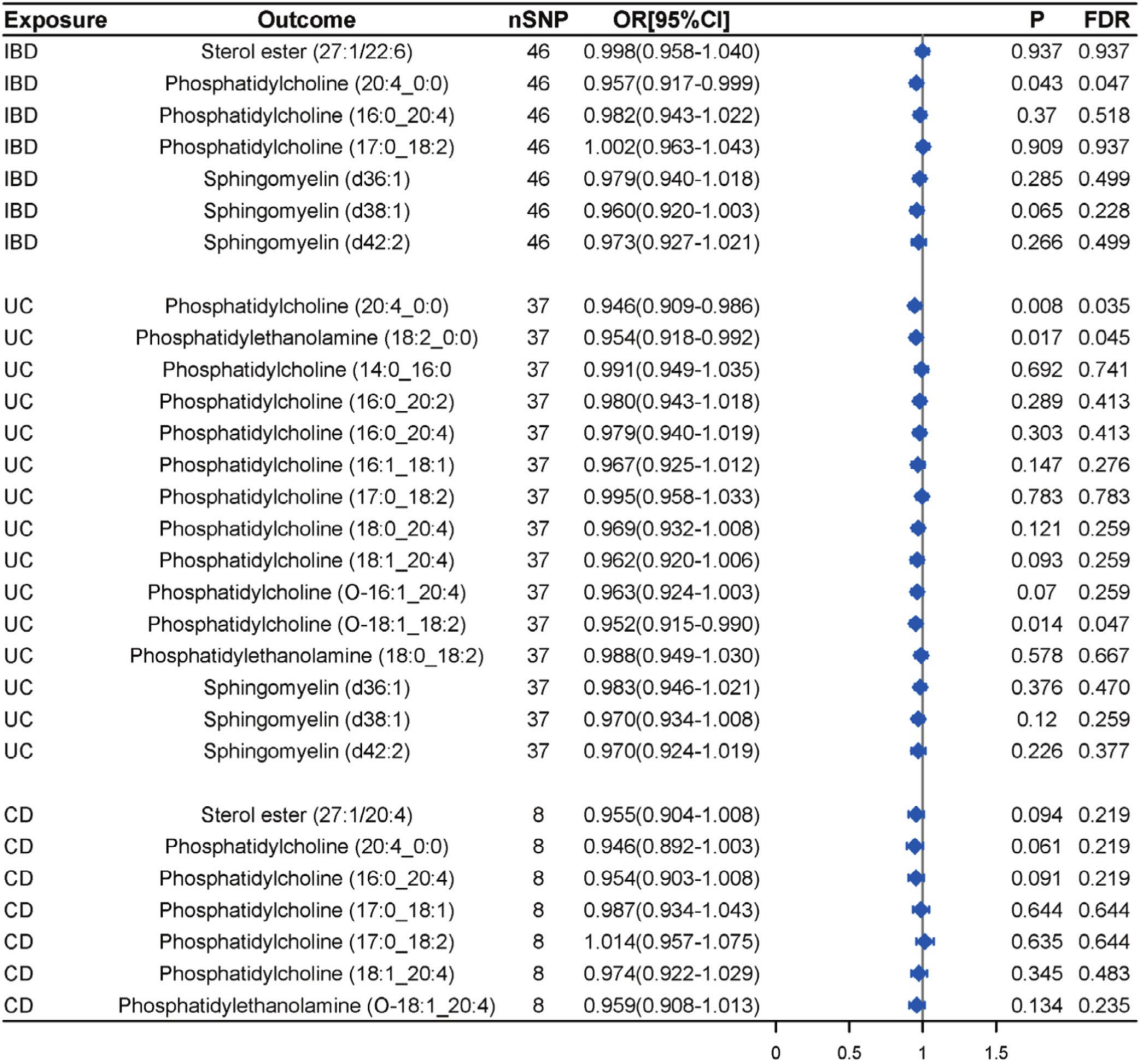
MR results of reverse associations between IBD (UC and CD) and plasma lipidome.

Subsequently, a reverse causal analysis was conducted for IBD with the 7 lipids. The association between IBD and the lipidome is presented in Table S10. The MR results indicate a reverse association between IBD and Phosphatidylcholine (20:4_0:0) (*p* = 0.043), while no reverse causal effects were observed between other lipids and IBD (Figure 3, Table S11).

#### 3.2.2 Inflammatory Proteins and IBD

**FIGURE 4:**
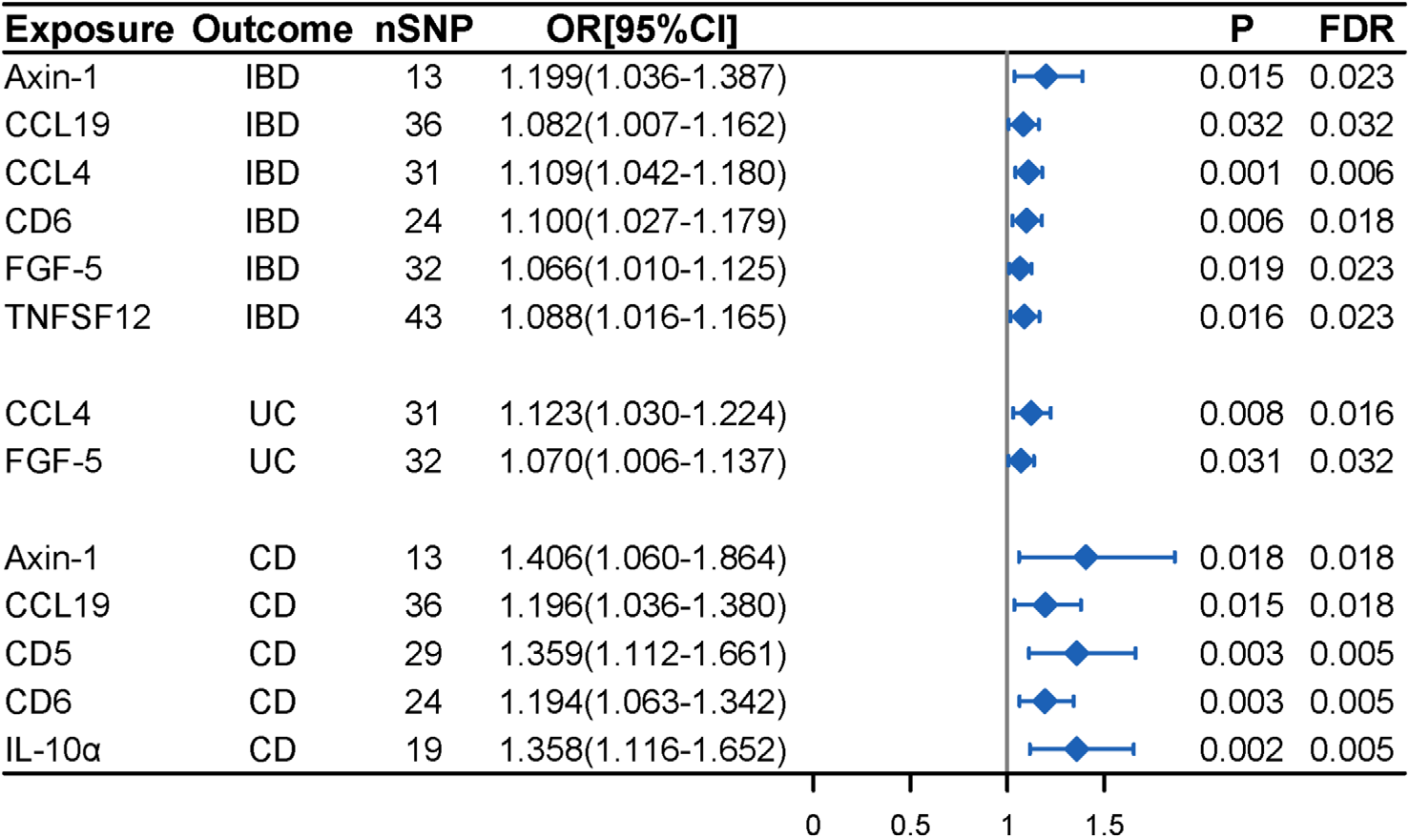
MR analysis results of associations between circulating inflammatory proteins and IBD (UC and CD).

We identified 12 inflammatory proteins associated with IBD after initial screening (IVW *p* < 0.05) (Table S12). Detailed associations between the inflammatory proteins and IBD are presented in Table S13. After further screening, 6 inflammatory proteins (Axin‐1, CCL19, CCL4, CD6, FGF‐5, and TNFSF12) were ultimately identified as being positively associated with the risk of IBD (Figure 4, Table S14).

After further validation analysis, 4 inflammatory proteins (CCL19, CCL4, CD6, and TNFSF12) remained significantly associated with IBD, whereas no significant causal relationship was observed for Axin‐1 and FGF‐5 (Figure S2, Table S15).

#### 3.2.3 Ulcerative Colitis

##### 3.2.3.1 Lipidome and UC

We initially identified 37 lipids associated with UC (Table S6). The association between relevant SNPs and UC is detailed in Table S7. After screening, a total of 15 lipids were ultimately identified as having a significant causal relationship with UC (Table S8). Among them, 9 lipids [Phosphatidylcholine (20:4_0:0); Phosphatidylcholine (14:0_16:0); Phosphatidylcholine (16:0_20:4); Phosphatidylcholine (18:0_20:4); Phosphatidylcholine (18:1_20:4); Phosphatidylcholine (O‐16:1_20:4); Sphingomyelin (d36:1); Sphingomyelin (d38:1); Sphingomyelin (d42:2)] were negatively correlated with UC risk, while 6 lipids [Phosphatidylethanolamine (18:2_0:0); Phosphatidylcholine (16:0_20:2); Phosphatidylcholine (16:1_18:1); Phosphatidylcholine (17:0_18:2); Phosphatidylcholine (O‐18:1_18:2); Phosphatidylethanolamine (18:0_18:2)] were positively correlated with the risk of UC (Figure 2, Table S8).

After further validation, Phosphatidylcholine (14:0_16:0) was excluded from the MR analysis for UC due to an insufficient number of associated SNPs. Among the remaining 14 lipids, 13 [excluding only Phosphatidylcholine (O‐16:1_20:4)] remained significantly associated with UC (Figure S1, Table S9).

The associations between UC and the lipidome are outlined in Table S10. In the causal relationship analysis between UC and the lipidome, bidirectional causal relationships were identified for Phosphatidylcholine (20:4_0:0), Phosphatidylethanolamine (18:2_0:0), and Phosphatidylcholine (O‐18:1_18:2), whereas no significant reverse causal effects were observed for the remaining 12 lipids (Figure 3, Table S11).

#### 3.2.4 Inflammatory Proteins and UC

In the MR analysis examining the relationship between inflammatory proteins and UC, we initially identified 7 inflammatory proteins associated with UC (Table S12). Detailed SNP information for 7 inflammatory proteins associated with UC is provided in Table S13. After screening, we observed that the increased risk of UC was significantly associated with genetically predicted higher levels of circulating inflammatory proteins (CCL4 and FGF‐5) (Figure 4, Table S14).

In a validation analysis using a more stringent SNP selection threshold (*p* < 1 × 10^−6^), CCL4 showed a significant causal association with UC, whereas FGF‐5 was not significantly associated with UC (Figure S2, Table S15).

#### 3.2.5 Crohn's Disease

##### 3.2.5.1 Lipidome and CD

We initially identified 20 lipids potentially associated with CD (Table S6). Detailed SNP information for the lipidome associated with CD is provided in Table S7. After multiple rounds of screening, we finally identified 7 lipids associated with CD (Table S8). The MR analysis results indicated a protective effect of the genetically driven increase in 6 lipids [Sterol ester (27:1/20:4); Phosphatidylcholine (20:4_0:0); Phosphatidylcholine (16:0_20:4); Phosphatidylcholine (17:0_18:1); Phosphatidylcholine (18:1_20:4); Phosphatidylethanolamine (O‐18:1_20:4)], while Phosphatidylcholine (17:0_18:2) was the risk factor for CD (Figure 2, Table S8).

When applying a more stringent SNP selection threshold (*p* < 1 × 10^−6^) to further validate the robustness of the findings, we observed that 5 lipids [Sterol ester (27:1/20:4); Phosphatidylcholine (20:4_0:0); Phosphatidylcholine (16:0_20:4); Phosphatidylcholine (18:1_20:4); Phosphatidylethanolamine (O‐18:1_20:4)] remained significantly associated with CD. However, no significant causal relationship was observed for Phosphatidylcholine (17:0_18:1) and Phosphatidylcholine (17:0_18:2) under this more conservative threshold (Figure S1, Table S9).

Similarly, subsequent bidirectional causal analysis showed no reverse causal effects between CD and these 7 lipids (Figure 3, Table S11).

##### 3.2.5.2 Inflammatory Proteins and CD

We initially screened out 9 inflammatory proteins associated with CD (Table S12). Detailed SNP information for inflammatory proteins associated with CD is shown in Table S13. Ultimately, the MR analysis results demonstrated that 5 circulating inflammatory proteins (Axin‐1, CCL19, CD5, CD6, and IL‐10α) were positively correlated with the risk of CD (Figure 4, Table S14).

Using a more stringent SNP selection threshold (*p* < 1 × 10^−6^), IL‐10α could not be included in the MR analysis due to an insufficient number of associated SNPs. Among the remaining 4 inflammatory proteins, three (CCL19, CD5, and CD6) were significantly associated with CD, while no significant causal relationship was found for Axin‐1 (Figure S2, Table S15).

### 3.3 Mediation Analysis

**FIGURE 5:**
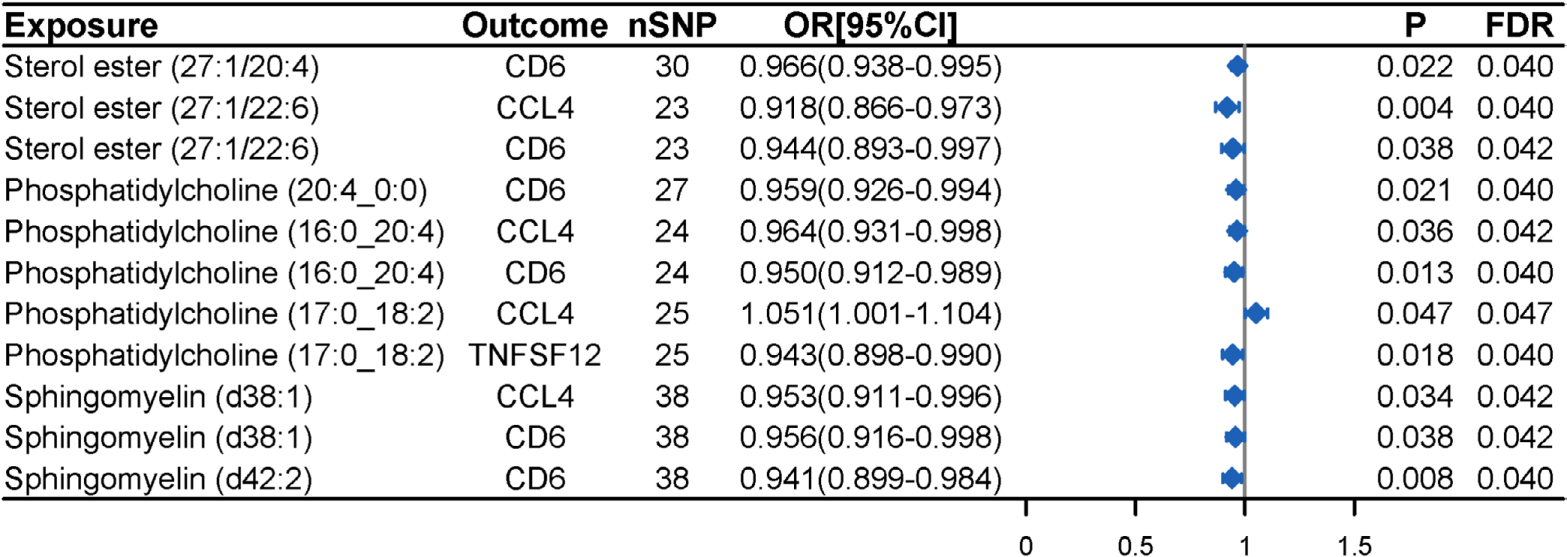
MR analysis results of associations between plasma lipidome and circulating inflammatory proteins.

After excluding lipids that have a bidirectional causal relationship with inflammatory bowel disease, we further analyzed the causal relationship between the lipidome and inflammatory proteins. We initially identified 12 causal relationships between 8 lipids and 3 inflammatory proteins (Table S16). Detailed SNP information for the lipidome associated with inflammatory proteins is shown in Table S17. After excluding causal relationships with heterogeneity, horizontal pleiotropy, and different directions of the five MR analysis methods, and BWMR *p* > 0.05, we ultimately identified 11 causal relationships between 7 lipids and 3 inflammatory proteins (Figure 5, Table S18). Under a more stringent SNP selection threshold (*p* < 1 × 10^−6^), we found that 4 lipids [Sterol ester (27:1/22:6); Phosphatidylcholine (16:0_20:4); Phosphatidylcholine (17:0_18:2); Sphingomyelin (d38:1)] were significantly associated with CCL4. We found that 6 lipids [Sterol ester (27:1/20:4); Sterol ester (27:1/22:6); Phosphatidylcholine (20:4_0:0); Phosphatidylcholine (16:0_20:4); Sphingomyelin (d38:1); Sphingomyelin (d42:2)] showed significant causal associations with CD6. Under the more stringent threshold (*p* < 1 × 10^−6^), no significant causal relationship was found between Phosphatidylcholine (17:0_18:2) and TNFSF12 (Figure S3, Table S19).

In the subsequent mediation analysis using a threshold of *p* < 1 × 10^−5^, we found that CCL4 played a mediating effect in the associations between 4 lipids [Sterol ester (27:1/22:6), Phosphatidylcholine (16:0_20:4), Phosphatidylcholine (17:0_18:2), Sphingomyelin (d38:1)] and IBD, with mediated proportions of 7.46%, 3.61%, 3.53%, and 5.46%, respectively (Table 1). The mediated proportions of CD6 in the associations between 5 lipids [Sterol ester (27:1/22:6), Phosphatidylcholine (20:4_0:0), Phosphatidylcholine (16:0_20:4), Sphingomyelin (d38:1), Sphingomyelin (d42:2)] and IBD were 4.67%, 3.69%, 4.70%, 4.74%, and 8.65%, respectively (Table 1). In the association analysis between lipidome and UC, we observed the mediating effect of CCL4 in the associations of Phosphatidylcholine (16:0_20:4), Phosphatidylcholine (17:0_18:2), and Sphingomyelin (d38:1) with UC, with mediated proportions of 3.20%, 3.69%, and 5.82%, respectively (Table 1). In the association analysis between lipidome and CD, we found that CD6 had the mediating effect in the associations of 3 lipids [Sterol ester (27:1/20:4), Phosphatidylcholine (20:4_0:0), and Phosphatidylcholine (16:0_20:4)] with CD, with mediated proportions of 5.87%, 7.39%, and 6.82%, respectively (Table 1). To validate these findings under a more stringent SNP threshold (*p* < 1 × 10^−6^), we repeated the mediation analyses. Consistent trends were observed: CCL4 and CD6 remained significant mediators in the lipidome‐IBD associations (Table S20).

**TABLE 1:** Results of mediation analysis.

Exposure	Mediation	Outcome	*β*	*β*1	*β*2	Mediation effect	Direct effect	Mediation effect/*β*
Sterol ester (27:1/22:6)	CCL4	IBD	−0.119	−0.086	0.103	−0.009	−0.110	0.0746
Sterol ester (27:1/22:6)	CD6	IBD	−0.119	−0.058	0.096	−0.006	−0.113	0.0467
Phosphatidylcholine (20:4_0:0)	CD6	IBD	−0.108	−0.042	0.096	−0.004	−0.104	0.0369
Phosphatidylcholine (16:0_20:4)	CCL4	IBD	−0.105	−0.037	0.103	−0.004	−0.101	0.0361
Phosphatidylcholine (16:0_20:4)	CD6	IBD	−0.105	−0.052	0.096	−0.005	−0.100	0.0470
Phosphatidylcholine (17:0_18:2)	CCL4	IBD	0.145	0.050	0.103	0.005	0.140	0.0353
Sphingomyelin (d38:1)	CCL4	IBD	−0.091	−0.048	0.103	−0.005	−0.086	0.0546
Sphingomyelin (d38:1)	CD6	IBD	−0.091	−0.045	0.096	−0.004	−0.087	0.0474
Sphingomyelin (d42:2)	CD6	IBD	−0.068	−0.061	0.096	−0.006	−0.062	0.0865
Phosphatidylcholine (16:0_20:4)	CCL4	UC	−0.134	−0.037	0.116	−0.004	−0.130	0.0318
Phosphatidylcholine (17:0_18:2)	CCL4	UC	0.156	0.050	0.116	0.006	0.150	0.0369
Sphingomyelin (d38:1)	CCL4	UC	−0.096	−0.048	0.116	−0.006	−0.091	0.0582
Sterol ester (27:1/20:4)	CD6	CD	−0.104	−0.035	0.177	−0.006	−0.098	0.0587
Phosphatidylcholine (20:4_0:0)	CD6	CD	−0.100	−0.042	0.177	−0.007	−0.092	0.0739
Phosphatidylcholine (16:0_20:4)	CD6	CD	−0.134	−0.052	0.177	−0.009	−0.125	0.0682

## 4 Discussion

Our MR study identified causal relationships between four classes of lipidome (Sterol ester, Phosphatidylcholine, Sphingomyelin, and Phosphatidylethanolamine) and IBD, UC, and CD. The increased levels of 6 circulating inflammatory proteins (CCL19, CCL4, CD5, CD6, IL‐10α, and TNFSF12) were associated with an elevated risk of IBD, UC, and CD. In the mediation analysis, we identified two circulating inflammatory proteins (CD6, CCL4) playing a mediating role between lipidome and inflammatory bowel disease. We proposed that Sterol ester [Sterol ester (27:1/20:4), Sterol ester (27:1/22:6)], Phosphatidylcholine [Phosphatidylcholine (20:4_0:0), Phosphatidylcholine (16:0_20:4)], and Sphingomyelin [Sphingomyelin (d38:1), Sphingomyelin (d42:2)] exerted a protective effect against IBD, UC, and CD by reducing CD6 and CCL4 levels. Additionally, phosphatidylcholine (17:0_18:2) was suggested to increase the risk of UC and IBD by elevating CCL4.

Our study found that Sterol ester plays a protective role in the occurrence of IBD. Previous studies also suggest that Sterol ester may improve intestinal inflammation in IBD by reducing inflammatory cell infiltration and the expression of inflammatory factors (Mencarelli et al. 2009; te Velde et al. 2015; Cheon et al. 2006). In our study, it was observed that the majority of phosphatidylcholine can reduce the risk of IBD. Previous studies have indicated that the level of Phosphatidylcholine in patients with IBD is decreased, and phosphatidylcholine can reduce the damage to the intestinal mucosal barrier caused by toxic substances, and it is also involved in a variety of anti‐inflammatory‐related signal pathways. (Tan et al. 2020; Ai et al. 2022). Meanwhile, a multicenter study showed that oral supplementation of Phosphatidylcholine can improve impaired intestinal mucosal barrier function and reduce disease activity in patients with IBD (Karner et al. 2014). However, our study found that some Phosphatidylcholine is positively associated with the risk of IBD. Phosphatidylcholine can be hydrolyzed in the body to saturated lysophosphatidylcholine, thereby increasing the production of pro‐inflammatory cytokines, inducing oxidative stress, and promoting inflammation, which may partially explain why some Phosphatidylcholine contributes to the risk of IBD (Huang et al. 2005; Liu et al. 2020). Our study suggests that an elevated level of Sphingomyelin in plasma is associated with a reduced risk of developing IBD (especially UC). Furuya et al.'s study indicates that oral administration of Sphingomyelin prevents the increase in MPO activity, alleviating intestinal inflammation in mice with IBD (Furuya et al. 2008). Meanwhile, a review suggests that increasing dietary Sphingomyelin is associated with a reduction in intestinal inflammation (Yamashita et al. 2021). Similarly, in our study, some Phosphatidylethanolamine showed an inverse correlation with the risk of IBD. Previous studies have not elucidated the relationship between Phosphatidylethanolamine and IBD. Wei et al.'s study shows that supplementing Phosphatidylethanolamine can inhibit endoplasmic reticulum stress and reduce intestinal cell damage through the p38 MAPK‐p65 pathway (Fang et al. 2022). Our study similarly demonstrates a positive correlation between certain Phosphatidylethanolamine levels and the risk of CD and UC. We speculate that it may be associated with the hydrolysis of Phosphatidylethanolamine into lyphosphatidylethanolamine by phospholipase A2 in the body. Current literature indicates a positive correlation between Lyphosphatidylethanolamine and the risk of Alzheimer's disease and fatty liver (Llano and Devanarayan 2021; Yamamoto et al. 2022). However, there is no literature reporting the association between Lyphosphatidylethanolamine and IBD.

Our MR study provides genetic evidence supporting the role of several inflammatory cytokines (CD6, CCL4) in mediating the impact of lipidome on inflammatory bowel diseases. Previous studies have indicated that Phosphatidylcholine can reduce the expression of various inflammatory factors in the body (Treede et al. 2007; Baek et al. 2022). However, few studies explored the association between Sterol ester, Phosphatidylcholine, and Sphingomyelin with CCL4 and CD6. In our study, Phosphatidylcholine (16:0_20:4), Sphingomyelin (d38:1), and Sterol ester (27:1/22:6) were found to reduce the risk of UC and IBD by lowering the levels of CCL4 in the body. Conversely, Phosphatidylcholine (17:0_18:2) increased the risk of UC and IBD by elevating the levels of CCL4 in the body. Previous studies have indicated an association between CCL4 and the body's inflammatory response and a significant correlation between decreased CCL4 levels and symptom relief in UC patients (Maurer and von Stebut 2004; Sato et al. 2009). Sterol ester (27:1/20:4), sterol ester (27:1/22:6), phosphatidylcholine (20:4_0:0), phosphatidylcholine (16:0_20:4), sphingomyelin (d38:1), and sphingomyelin (d42:2) were found to reduce the risk of IBD and CD by decreasing CD6. Sergi et al. revealed the association between CD6 and the prognosis of CD patients from a genetic perspective (Casadó‐Llombart et al. 2022).

### 4.1 Our Study Has Several Strengths

First, it included a total of 434,179 participants and utilized the largest and most up‐to‐date GWAS datasets to date on lipidome, inflammatory proteins, and IBD, ensuring broad population representation and robust statistical power. Second, we employed five different MR methods—IVW, MR‐Egger, weighted median, weighted mode, and simple mode—to estimate the causal relationships between exposures and outcomes. The consistent directionality of the effect estimates across these methods further strengthened the robustness of our conclusions. In addition, we incorporated the Bayesian weighted MR (BWMR) method as a sensitivity analysis to enhance the reliability of our findings. To further validate the robustness of the MR results, we selected IVs using two different significance thresholds (*p* < 1 × 10^−5^ and *p* < 1 × 10^−6^) and repeated the analyses under the more stringent threshold to reduce the risk of false‐positive associations. Finally, we integrated mediation MR analyses to explore the potential mediating roles of inflammatory proteins in the associations between lipids and IBD, providing new insights into the biological pathways underlying disease development. However, our study also has some limitations. First, all summary statistics were derived from GWAS datasets based on European populations, which limits the generalizability of our findings to other ancestries. Genetic architecture, environmental exposures, dietary patterns, and immune responses may differ across populations, potentially influencing the mechanisms linking lipid metabolism, inflammation, and IBD. Therefore, the applicability of our findings to Asian, African, or other non‐European populations remains to be confirmed in future multi‐ethnic studies. Second, all GWAS data used in this study were based on peripheral blood samples, and the lack of genetic and transcriptomic data from intestinal tissues limited our ability to investigate gut‐specific mechanisms. Third, due to the constraints of publicly available summary data, we were unable to perform stratified analyses based on sex, age, or disease severity, which may have obscured potential subgroup‐specific heterogeneity. Fourth, although MR analysis effectively mitigates confounding bias inherent in observational studies, it cannot fully rule out the influence of pleiotropy or unmeasured confounders. Finally, we adopted relatively lenient P‐value thresholds (*p* < 1 × 10^−5^ and *p* < 1 × 10^−6^) for selecting instrumental variables, which may have increased the risk of false positives, despite the application of multiple sensitivity analyses to address this issue.

Our study revealed a causal relationship between Sterol Ester, Phosphatidylcholine, Sphingomyelin, Phosphatidylethanolamine, and IBD, including UC and CD. Additionally, CD6 and CCL4 played mediating roles in the association between lipidome and IBD. These results and findings may bring new targets and directions for the treatment of IBD.

## Author Contributions

**Linlin Yin:** conceptualization (equal), data curation (equal), formal analysis (equal), investigation (equal), methodology (equal), validation (equal), visualization (equal), writing – original draft (equal), writing – review and editing (equal). **Yue Zhu:** data curation (equal), formal analysis (equal), investigation (equal), validation (equal), visualization (equal), writing – review and editing (equal). **Fang Kong:** data curation (equal), formal analysis (equal), writing – review and editing (equal). **Hongfei Tu:** data curation (equal), formal analysis (equal), writing – review and editing (equal). **Bin Zhang:** data curation (equal), formal analysis (equal), investigation (equal), methodology (equal), validation (equal), writing – original draft (equal).

## Ethics Statement

The study used only public data and did not require additional informed consent or ethical applications.

## Conflicts of Interest

The authors declare no conflicts of interest.

## Supporting information


**Figures S1–S3:** fsn370916‐sup‐0001‐FiguresS1‐S3.docx.

**Tables S1–S20:** fsn370916‐sup‐0002‐TablesS1‐S20.docx.

## Data Availability

The summary data of FinnGen can be downloaded from the website https://www.finngen.fi/en/access_results. The summary data of plasma lipidome and circulating inflammatory proteins can be downloaded from the website https://www.ebi.ac.uk/gwas/downloads/summary‐statistics.
