# Supplementary material for: Causal Association of Plasma Lipidome With Inflammatory Bowel Diseases and Mediating Role of Circulating Inflammatory Proteins: A Mendelian Randomization Study
Source: Food Sci Nutr. 2025 Sep 10;13(9):e70916. doi: 10.1002/fsn3.70916 (PMC12421312; doi:10.1002/fsn3.70916)
Supplement: Supplementary file 1 — Figures S1–S3: fsn370916‐sup‐0001‐FiguresS1‐S3.docx. [file FSN3-13-e70916-s002.docx]

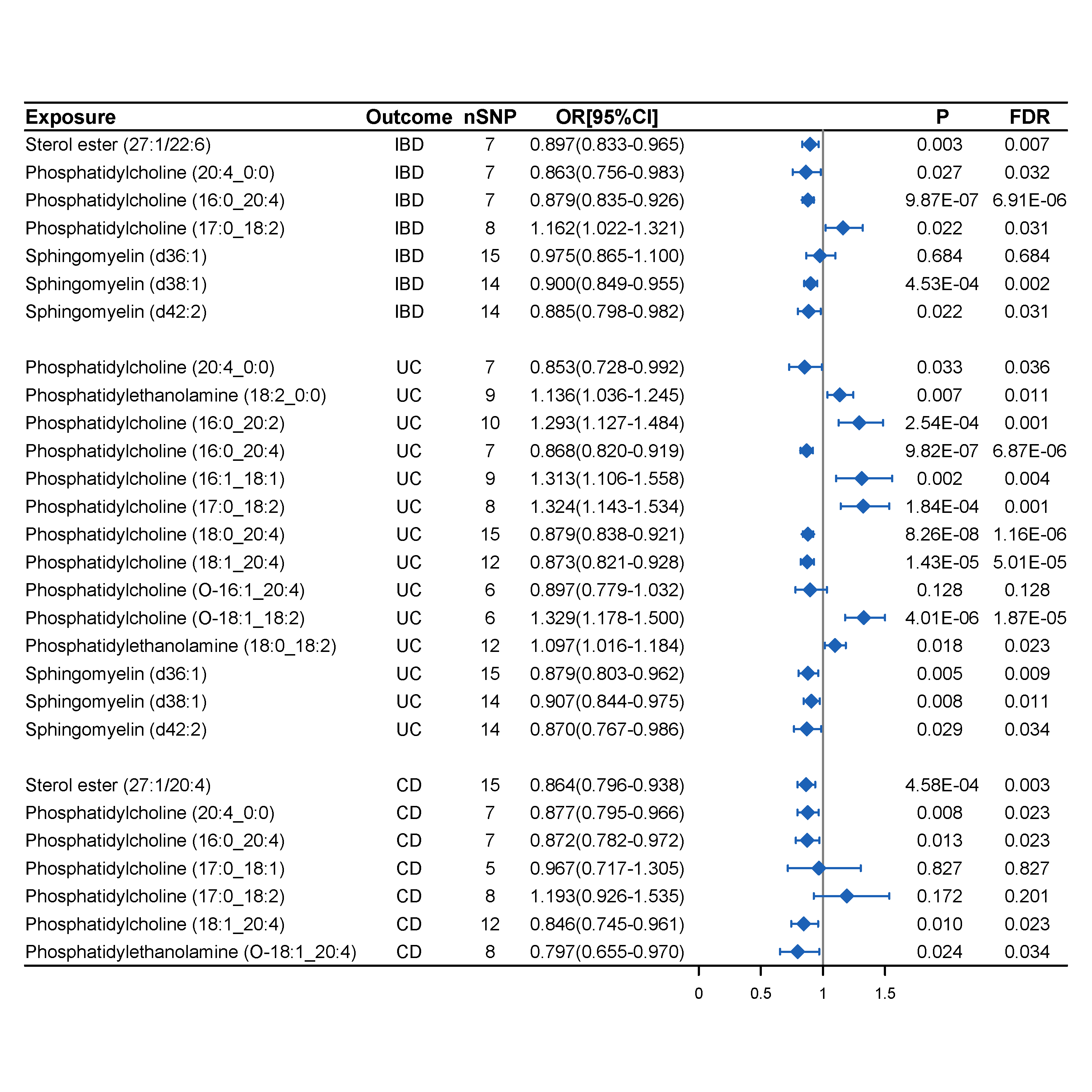


**Supplementary Figure 1:** MR results of causal effects between plasma lipidome and IBD (UC and CD) after further validation analysis.


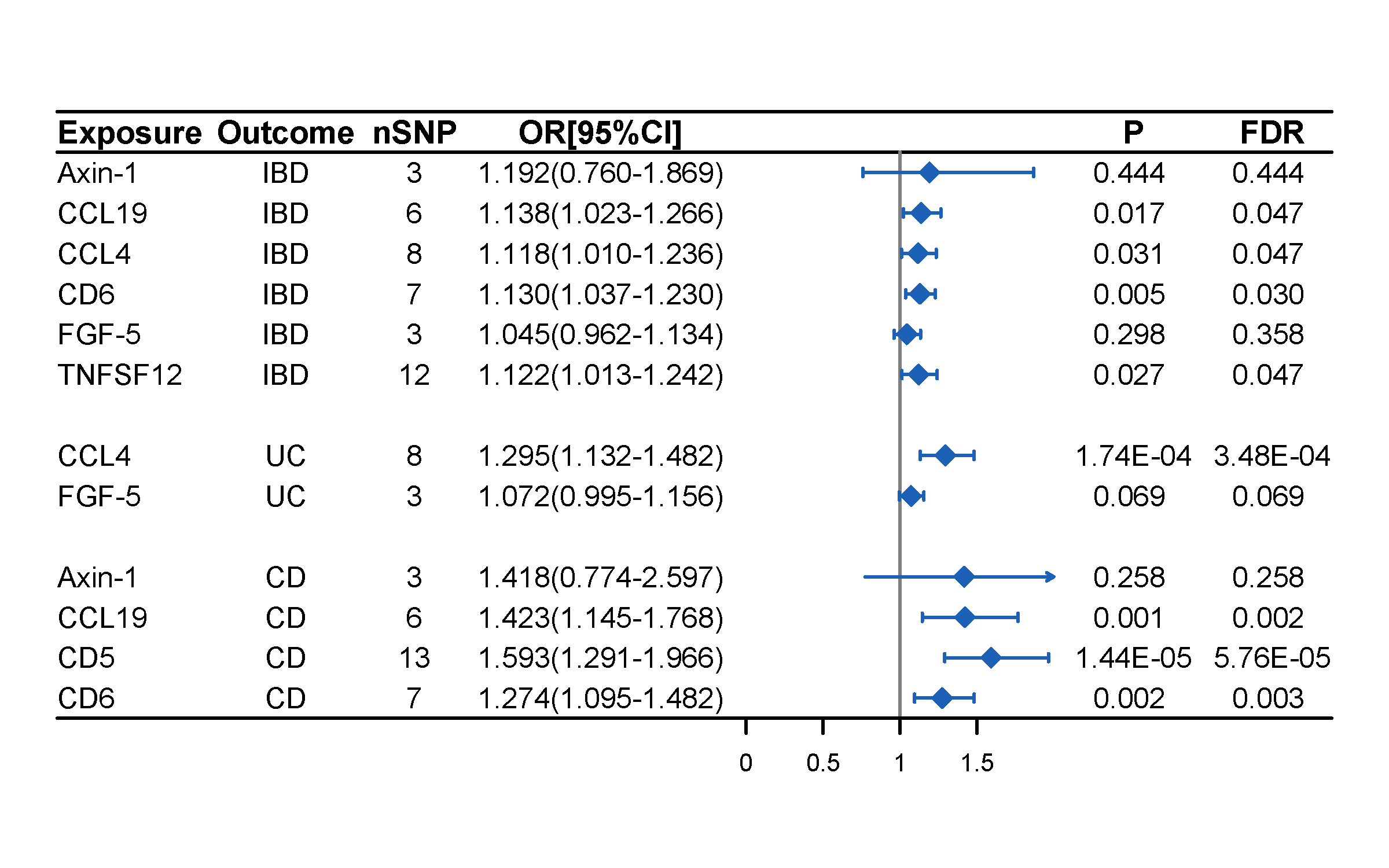


**Supplementary Figure 2:** MR analysis results of associations between circulating inflammatory proteins and IBD (UC and CD) after further validation analysis.


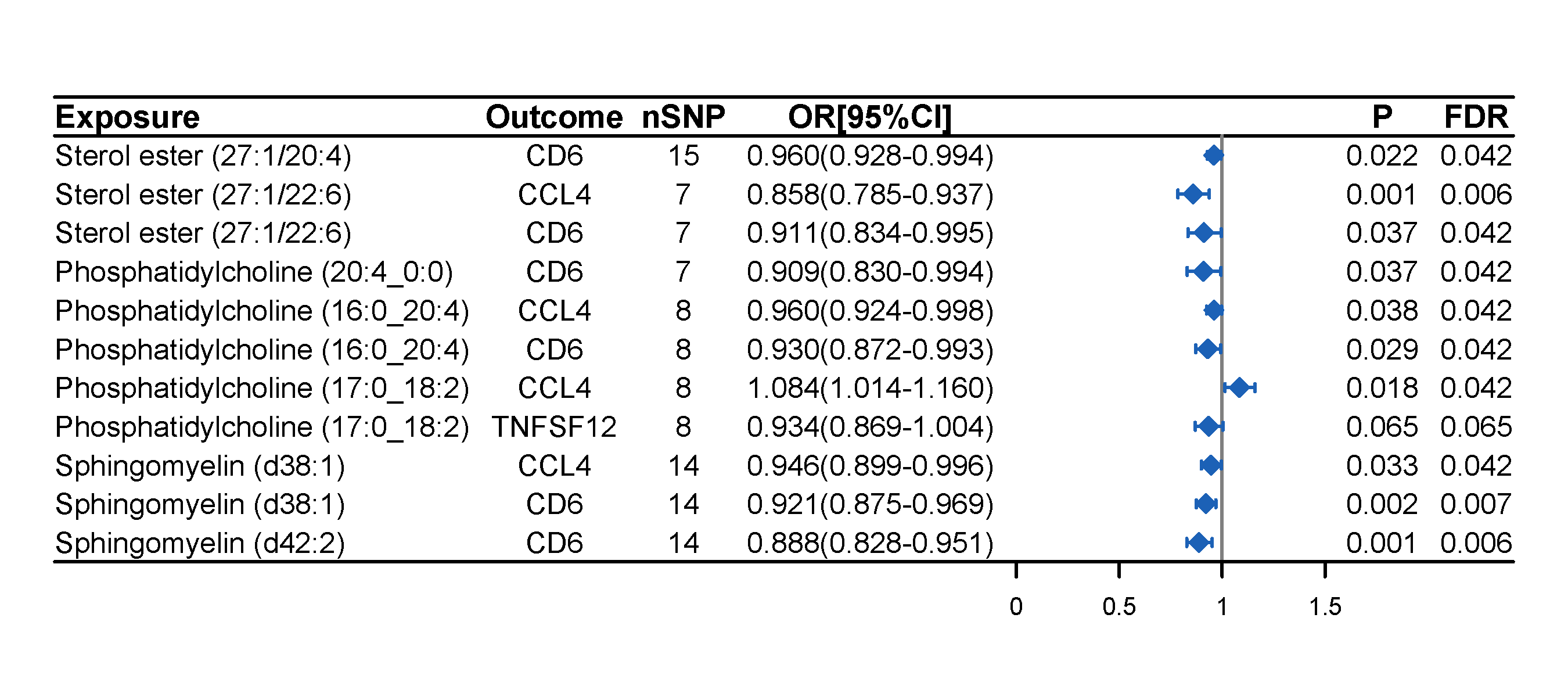


**Supplementary Figure 3:** MR analysis results of associations between plasma lipidome and circulating inflammatory proteins after further validation analysis.
