# Supplementary material for: Causal Association of Plasma Lipidome With Inflammatory Bowel Diseases and Mediating Role of Circulating Inflammatory Proteins: A Mendelian Randomization Study
Source: Food Sci Nutr. 2025 Sep 10;13(9):e70916. doi: 10.1002/fsn3.70916 (PMC12421312; doi:10.1002/fsn3.70916)
Supplement: Supplementary file 2 — Tables S1–S20: fsn370916‐sup‐0002‐TablesS1‐S20.docx. [file FSN3-13-e70916-s001.docx]

| **Supplementary Table S1. Summary information for SNPs that were used as genetic instruments for Mendelian randomization analyses of plasma lipidome.** | | | | | | | | | | |
| --- | --- | --- | --- | --- | --- | --- | --- | --- | --- | --- |
| **Plasma lipidome** | **SNPs** | **Chromosome** | **Location** | **EA** | **OA** | **Beta** | **SE** | **EAF** | **P** | **N** |
| Sterol ester (27:1/14:0) | rs41289170 | 1 | 72791134 | A | G | 0.799 | 0.179 | 0.003 | 8.30E-06 | 7174 |
|  | rs11608 | 2 | 27212506 | A | G | 0.088 | 0.019 | 0.723 | 2.53E-06 | 7174 |
|  | rs116778355 | 2 | 224951925 | T | A | -0.380 | 0.078 | 0.012 | 1.00E-06 | 7174 |
|  | rs2686446 | 3 | 195507255 | C | G | 0.090 | 0.018 | 0.711 | 1.28E-06 | 7174 |
|  | rs2964094 | 5 | 174326694 | C | T | 0.087 | 0.019 | 0.753 | 5.94E-06 | 7174 |
|  | rs55764039 | 7 | 17920067 | C | G | -0.181 | 0.037 | 0.053 | 1.41E-06 | 7174 |
|  | rs28601761 | 8 | 125487789 | G | C | -0.084 | 0.017 | 0.410 | 7.71E-07 | 7174 |
|  | rs10812660 | 9 | 27742537 | A | T | 0.079 | 0.018 | 0.375 | 9.81E-06 | 7174 |
|  | rs80195215 | 10 | 122133760 | G | A | -0.187 | 0.041 | 0.047 | 5.46E-06 | 7174 |
|  | rs12787616 | 11 | 26008522 | C | T | 0.077 | 0.017 | 0.373 | 9.98E-06 | 7174 |
|  | rs4246215 | 11 | 61796827 | T | G | -0.081 | 0.017 | 0.436 | 1.80E-06 | 7174 |
|  | rs7957768 | 12 | 101754541 | T | G | -0.087 | 0.017 | 0.521 | 2.21E-07 | 7174 |
|  | rs1696359 | 12 | 120950756 | C | T | -0.080 | 0.017 | 0.585 | 2.84E-06 | 7174 |
|  | rs2479959 | 13 | 111318086 | A | G | -0.080 | 0.018 | 0.300 | 9.95E-06 | 7174 |
|  | rs75922719 | 15 | 93906166 | C | T | 0.481 | 0.103 | 0.007 | 2.79E-06 | 7174 |
|  | rs7198817 | 16 | 11851921 | A | C | 0.076 | 0.017 | 0.578 | 7.55E-06 | 7174 |
|  | rs187429064 | 19 | 19269704 | G | A | -0.234 | 0.038 | 0.054 | 6.89E-10 | 7174 |
|  | rs429358 | 19 | 44908684 | C | T | 0.142 | 0.021 | 0.189 | 4.33E-11 | 7174 |
|  | rs6045698 | 20 | 1965583 | C | A | 0.090 | 0.019 | 0.264 | 2.96E-06 | 7174 |
|  | rs1800961 | 20 | 44413724 | T | C | -0.186 | 0.037 | 0.052 | 5.99E-07 | 7174 |
|  | rs62232045 | 22 | 43503538 | C | T | 0.086 | 0.018 | 0.344 | 1.04E-06 | 7174 |
| Sterol ester (27:1/15:0) | rs1999811 | 1 | 30991480 | T | C | -0.086 | 0.019 | 0.691 | 7.28E-06 | 6428 |
|  | rs17014799 | 1 | 209576196 | C | T | 0.462 | 0.102 | 0.008 | 6.37E-06 | 6428 |
|  | rs139857433 | 2 | 98354059 | T | A | -0.460 | 0.100 | 0.009 | 4.49E-06 | 6428 |
|  | rs930917 | 3 | 39848105 | G | A | 0.082 | 0.018 | 0.373 | 7.39E-06 | 6428 |
|  | rs113844909 | 4 | 26516933 | C | G | -0.330 | 0.070 | 0.017 | 2.77E-06 | 6428 |
|  | rs115686032 | 5 | 40282763 | T | C | -0.232 | 0.046 | 0.039 | 3.88E-07 | 6428 |
|  | rs150680623 | 5 | 63595335 | G | T | 0.332 | 0.074 | 0.014 | 7.08E-06 | 6428 |
|  | rs2100040 | 5 | 65839152 | A | G | 0.092 | 0.020 | 0.259 | 5.57E-06 | 6428 |
|  | rs55662279 | 5 | 175777765 | T | C | -0.780 | 0.166 | 0.004 | 2.72E-06 | 6428 |
|  | rs2237478 | 7 | 50678097 | T | C | -0.105 | 0.022 | 0.198 | 2.55E-06 | 6428 |
|  | rs146236963 | 7 | 104180833 | C | T | -0.327 | 0.071 | 0.016 | 4.49E-06 | 6428 |
|  | rs62502692 | 8 | 4035643 | C | G | -0.128 | 0.029 | 0.109 | 8.94E-06 | 6428 |
|  | rs985679 | 8 | 138441693 | G | A | 0.080 | 0.018 | 0.531 | 6.64E-06 | 6428 |
|  | rs61859797 | 10 | 129461219 | T | C | 0.109 | 0.023 | 0.178 | 2.63E-06 | 6428 |
|  | rs4246215 | 11 | 61796827 | T | G | -0.084 | 0.018 | 0.436 | 2.52E-06 | 6428 |
|  | rs12589429 | 14 | 48229919 | T | C | -0.103 | 0.023 | 0.182 | 8.08E-06 | 6428 |
|  | rs77201364 | 17 | 14664504 | A | G | 0.367 | 0.082 | 0.013 | 6.93E-06 | 6428 |
|  | rs140283718 | 19 | 30685856 | A | T | -0.254 | 0.057 | 0.026 | 7.08E-06 | 6428 |
|  | rs2104810 | 21 | 41477170 | A | G | 0.084 | 0.019 | 0.636 | 6.19E-06 | 6428 |
|  | rs28582101 | 22 | 36797580 | G | C | -0.105 | 0.024 | 0.823 | 9.75E-06 | 6428 |
| Sterol ester (27:1/16:0) | rs11591147 | 1 | 55039974 | T | G | -0.390 | 0.046 | 0.033 | 5.36E-17 | 7174 |
|  | rs3005923 | 1 | 56335870 | A | G | -0.286 | 0.053 | 0.028 | 6.58E-08 | 7174 |
|  | rs7517801 | 1 | 154043206 | A | G | -0.256 | 0.057 | 0.022 | 6.62E-06 | 7174 |
|  | rs1367117 | 2 | 21041028 | A | G | 0.109 | 0.018 | 0.285 | 2.74E-09 | 7174 |
|  | rs56767222 | 3 | 3342886 | C | T | 0.667 | 0.142 | 0.003 | 2.84E-06 | 7174 |
|  | rs13085650 | 3 | 76900144 | C | T | -0.087 | 0.019 | 0.252 | 7.31E-06 | 7174 |
|  | rs10031375 | 4 | 37043069 | G | C | 0.076 | 0.016 | 0.558 | 4.14E-06 | 7174 |
|  | rs74915447 | 4 | 72899760 | A | G | 0.247 | 0.044 | 0.038 | 2.55E-08 | 7174 |
|  | rs79146711 | 4 | 73424911 | T | C | 0.224 | 0.041 | 0.045 | 4.45E-08 | 7174 |
|  | rs138270540 | 4 | 74487710 | C | A | 0.330 | 0.051 | 0.028 | 8.36E-11 | 7174 |
|  | rs28627829 | 4 | 164639807 | G | T | -0.119 | 0.025 | 0.134 | 1.63E-06 | 7174 |
|  | rs10078182 | 5 | 118775347 | C | T | 0.268 | 0.059 | 0.022 | 5.22E-06 | 7174 |
|  | rs10943045 | 6 | 72631607 | T | G | -0.089 | 0.020 | 0.217 | 9.57E-06 | 7174 |
|  | rs1336770 | 6 | 124502870 | G | A | 0.080 | 0.017 | 0.586 | 3.07E-06 | 7174 |
|  | rs141836086 | 6 | 152650134 | T | C | 0.223 | 0.046 | 0.033 | 1.48E-06 | 7174 |
|  | rs2236514 | 9 | 136677616 | G | C | -0.086 | 0.018 | 0.647 | 1.38E-06 | 7174 |
|  | rs10795530 | 10 | 7162916 | G | A | -0.076 | 0.017 | 0.580 | 7.08E-06 | 7174 |
|  | rs117010856 | 10 | 52079201 | T | C | 0.751 | 0.165 | 0.003 | 5.80E-06 | 7174 |
|  | rs7932326 | 11 | 43971474 | A | G | 0.079 | 0.017 | 0.617 | 4.13E-06 | 7174 |
|  | rs174527 | 11 | 61770929 | C | G | -0.083 | 0.017 | 0.444 | 1.24E-06 | 7174 |
|  | rs174547 | 11 | 61803311 | C | T | -0.206 | 0.017 | 0.407 | 4.77E-34 | 7174 |
|  | rs3017103 | 11 | 62406721 | G | A | 0.094 | 0.021 | 0.804 | 8.15E-06 | 7174 |
|  | rs538047 | 11 | 88314021 | A | G | -0.073 | 0.017 | 0.463 | 9.75E-06 | 7174 |
|  | rs11604424 | 11 | 116780399 | T | C | -0.105 | 0.019 | 0.753 | 5.06E-08 | 7174 |
|  | rs9788072 | 12 | 4796048 | G | A | 0.170 | 0.037 | 0.056 | 3.41E-06 | 7174 |
|  | rs10841310 | 12 | 19677962 | T | G | -0.096 | 0.019 | 0.246 | 7.87E-07 | 7174 |
|  | rs7487904 | 12 | 53231170 | G | C | 0.109 | 0.024 | 0.147 | 3.94E-06 | 7174 |
|  | rs10860778 | 12 | 101714807 | T | C | -0.085 | 0.017 | 0.491 | 3.85E-07 | 7174 |
|  | rs7167665 | 15 | 53458377 | C | T | 0.086 | 0.017 | 0.538 | 2.37E-07 | 7174 |
|  | rs9924448 | 16 | 73349244 | T | C | -0.085 | 0.017 | 0.557 | 7.15E-07 | 7174 |
|  | rs75708826 | 18 | 65789123 | G | A | -0.313 | 0.069 | 0.015 | 6.16E-06 | 7174 |
|  | rs118068660 | 19 | 11079868 | T | C | -0.209 | 0.028 | 0.095 | 1.02E-13 | 7174 |
|  | rs73005445 | 19 | 15830645 | G | A | -0.096 | 0.019 | 0.253 | 5.59E-07 | 7174 |
|  | rs7412 | 19 | 44908822 | T | C | -0.407 | 0.037 | 0.053 | 4.75E-28 | 7174 |
|  | rs56336136 | 19 | 48224239 | T | C | 0.130 | 0.028 | 0.104 | 2.32E-06 | 7174 |
|  | rs1800961 | 20 | 44413724 | T | C | -0.222 | 0.037 | 0.052 | 2.60E-09 | 7174 |
| Sterol ester (27:1/16:1) | rs142738732 | 1 | 1329726 | A | T | -0.166 | 0.035 | 0.063 | 2.20E-06 | 7172 |
|  | rs111656006 | 1 | 38245682 | T | C | 0.185 | 0.040 | 0.048 | 3.82E-06 | 7172 |
|  | rs11119973 | 1 | 212561372 | A | C | 0.111 | 0.023 | 0.153 | 1.84E-06 | 7172 |
|  | rs1260326 | 2 | 27508073 | C | T | -0.090 | 0.017 | 0.651 | 2.33E-07 | 7172 |
|  | rs7355269 | 2 | 62072939 | C | T | 0.243 | 0.053 | 0.026 | 4.35E-06 | 7172 |
|  | rs80227620 | 2 | 167832629 | T | C | 0.117 | 0.024 | 0.144 | 1.73E-06 | 7172 |
|  | rs116522970 | 4 | 14330096 | G | T | 0.558 | 0.124 | 0.005 | 7.01E-06 | 7172 |
|  | rs5022521 | 4 | 110207431 | T | C | -0.108 | 0.021 | 0.797 | 3.39E-07 | 7172 |
|  | rs274673 | 5 | 6743310 | C | T | 0.186 | 0.039 | 0.952 | 2.24E-06 | 7172 |
|  | rs17603855 | 7 | 138187033 | T | C | 0.169 | 0.036 | 0.058 | 2.13E-06 | 7172 |
|  | rs10812660 | 9 | 27742537 | A | T | 0.080 | 0.018 | 0.375 | 7.26E-06 | 7172 |
|  | rs855500 | 9 | 33614216 | C | T | 0.076 | 0.017 | 0.582 | 6.24E-06 | 7172 |
|  | rs10760286 | 9 | 123419309 | C | T | 0.083 | 0.017 | 0.510 | 1.06E-06 | 7172 |
|  | rs603424 | 10 | 100315722 | A | G | -0.156 | 0.026 | 0.121 | 2.07E-09 | 7172 |
|  | rs4447106 | 10 | 100600003 | A | G | 0.118 | 0.024 | 0.141 | 1.01E-06 | 7172 |
|  | rs7313803 | 12 | 87990673 | T | C | 0.087 | 0.019 | 0.260 | 6.97E-06 | 7172 |
|  | rs2464190 | 12 | 120977587 | C | T | 0.076 | 0.017 | 0.457 | 5.25E-06 | 7172 |
|  | rs113130033 | 14 | 89719965 | G | A | -0.194 | 0.041 | 0.043 | 2.22E-06 | 7172 |
|  | rs11647818 | 16 | 11833509 | T | C | 0.084 | 0.017 | 0.575 | 8.02E-07 | 7172 |
|  | rs201714080 | 16 | 71095785 | A | C | -0.188 | 0.038 | 0.052 | 7.60E-07 | 7172 |
|  | rs150352030 | 17 | 34678243 | C | A | -0.645 | 0.142 | 0.004 | 5.67E-06 | 7172 |
|  | rs116917626 | 17 | 51561964 | T | C | -0.259 | 0.052 | 0.028 | 7.87E-07 | 7172 |
|  | rs117952876 | 19 | 9621826 | C | T | 0.219 | 0.050 | 0.031 | 9.64E-06 | 7172 |
|  | rs187429064 | 19 | 19269704 | G | A | -0.217 | 0.038 | 0.054 | 9.82E-09 | 7172 |
|  | rs58489806 | 19 | 19346108 | T | C | -0.142 | 0.032 | 0.076 | 6.59E-06 | 7172 |
|  | rs1800961 | 20 | 44413724 | T | C | -0.204 | 0.037 | 0.052 | 4.34E-08 | 7172 |
| Sterol ester (27:1/17:0) | rs11265455 | 1 | 160650361 | G | A | 0.119 | 0.026 | 0.141 | 3.30E-06 | 7047 |
|  | rs12735602 | 1 | 210320724 | A | C | 0.076 | 0.017 | 0.541 | 9.09E-06 | 7047 |
|  | rs1868072 | 2 | 30180840 | C | T | -0.083 | 0.019 | 0.705 | 8.47E-06 | 7047 |
|  | rs72945021 | 2 | 211666173 | A | T | -0.100 | 0.022 | 0.183 | 5.01E-06 | 7047 |
|  | rs9827613 | 3 | 11833970 | G | A | -0.075 | 0.017 | 0.461 | 8.51E-06 | 7047 |
|  | rs13125535 | 4 | 12263787 | G | T | 0.086 | 0.017 | 0.598 | 5.61E-07 | 7047 |
|  | rs79568254 | 4 | 136648128 | A | T | -0.310 | 0.069 | 0.015 | 7.56E-06 | 7047 |
|  | rs17791425 | 6 | 84260623 | A | G | 0.180 | 0.039 | 0.050 | 4.90E-06 | 7047 |
|  | rs77943189 | 6 | 122284176 | G | A | 0.336 | 0.071 | 0.016 | 2.05E-06 | 7047 |
|  | rs10241012 | 7 | 104334784 | G | A | 0.118 | 0.026 | 0.123 | 4.98E-06 | 7047 |
|  | rs2382335 | 9 | 12243520 | C | T | 0.102 | 0.023 | 0.835 | 7.21E-06 | 7047 |
|  | rs111566713 | 9 | 86181820 | T | C | -0.238 | 0.054 | 0.025 | 8.77E-06 | 7047 |
|  | rs635634 | 9 | 133279427 | C | T | -0.100 | 0.021 | 0.798 | 1.84E-06 | 7047 |
|  | rs10881589 | 9 | 134243611 | T | C | 0.079 | 0.018 | 0.433 | 8.37E-06 | 7047 |
|  | rs4246215 | 11 | 61796827 | T | G | -0.085 | 0.017 | 0.436 | 6.70E-07 | 7047 |
|  | rs140015624 | 11 | 71313656 | C | T | 0.323 | 0.069 | 0.016 | 2.96E-06 | 7047 |
|  | rs4503592 | 12 | 8854565 | G | T | -0.090 | 0.020 | 0.222 | 9.63E-06 | 7047 |
|  | rs116859210 | 12 | 46735737 | G | A | 0.351 | 0.077 | 0.012 | 5.04E-06 | 7047 |
|  | rs2280696 | 12 | 53118972 | T | A | 0.204 | 0.025 | 0.126 | 6.37E-16 | 7047 |
|  | rs113505726 | 12 | 54038501 | A | G | -0.192 | 0.042 | 0.042 | 5.11E-06 | 7047 |
|  | rs12296850 | 12 | 100426307 | G | A | 0.173 | 0.038 | 0.054 | 4.26E-06 | 7047 |
|  | rs79528439 | 15 | 48709000 | A | T | -0.132 | 0.027 | 0.118 | 7.60E-07 | 7047 |
|  | rs12921107 | 16 | 292437 | C | T | -0.125 | 0.024 | 0.142 | 2.38E-07 | 7047 |
|  | rs10775274 | 16 | 25212475 | C | T | 0.170 | 0.036 | 0.938 | 1.93E-06 | 7047 |
|  | rs2358779 | 16 | 31850641 | A | G | -0.243 | 0.053 | 0.971 | 5.03E-06 | 7047 |
|  | rs1002079 | 16 | 76465100 | G | C | -0.142 | 0.028 | 0.101 | 3.89E-07 | 7047 |
|  | rs429358 | 19 | 44908684 | C | T | 0.122 | 0.022 | 0.189 | 1.91E-08 | 7047 |
|  | rs28582101 | 22 | 36797580 | G | C | -0.108 | 0.023 | 0.823 | 2.22E-06 | 7047 |
| Sterol ester (27:1/17:1) | rs116778355 | 2 | 224951925 | T | A | -0.357 | 0.078 | 0.012 | 4.61E-06 | 7166 |
|  | rs40435 | 3 | 134998561 | T | A | -0.079 | 0.017 | 0.406 | 3.81E-06 | 7166 |
|  | rs72723967 | 4 | 137812361 | A | C | 0.103 | 0.023 | 0.163 | 5.81E-06 | 7166 |
|  | rs79101108 | 5 | 105868026 | A | G | 0.218 | 0.049 | 0.031 | 9.05E-06 | 7166 |
|  | rs6469013 | 8 | 105734207 | G | A | 0.082 | 0.018 | 0.299 | 7.16E-06 | 7166 |
|  | rs10812660 | 9 | 27742537 | A | T | 0.093 | 0.018 | 0.375 | 1.91E-07 | 7166 |
|  | rs77392374 | 10 | 71286664 | C | T | -0.243 | 0.053 | 0.027 | 5.17E-06 | 7166 |
|  | rs11614909 | 12 | 68935916 | A | C | -0.097 | 0.022 | 0.186 | 7.04E-06 | 7166 |
|  | rs10746106 | 12 | 79317580 | G | A | -0.085 | 0.019 | 0.272 | 7.24E-06 | 7166 |
|  | rs7957655 | 12 | 101754513 | A | G | -0.078 | 0.017 | 0.521 | 3.41E-06 | 7166 |
|  | rs7151696 | 14 | 22802228 | T | C | 0.078 | 0.017 | 0.570 | 4.31E-06 | 7166 |
|  | rs217184 | 16 | 72072066 | C | T | -0.093 | 0.020 | 0.228 | 3.24E-06 | 7166 |
|  | rs117691424 | 17 | 34703734 | A | G | -0.634 | 0.142 | 0.004 | 8.36E-06 | 7166 |
|  | rs141647056 | 18 | 55127832 | G | A | -0.905 | 0.202 | 0.002 | 7.80E-06 | 7166 |
|  | rs76492272 | 18 | 70340195 | A | G | 0.258 | 0.054 | 0.029 | 1.50E-06 | 7166 |
|  | rs187429064 | 19 | 19269704 | G | A | -0.211 | 0.038 | 0.054 | 3.10E-08 | 7166 |
|  | rs193228861 | 19 | 41730113 | C | A | -0.423 | 0.092 | 0.008 | 4.14E-06 | 7166 |
|  | rs1800961 | 20 | 44413724 | T | C | -0.198 | 0.037 | 0.052 | 1.08E-07 | 7166 |
| Sterol ester (27:1/18:0) | rs113270487 | 1 | 5333063 | A | G | 0.175 | 0.038 | 0.052 | 5.00E-06 | 7157 |
|  | rs144944992 | 1 | 164790578 | T | C | 0.495 | 0.090 | 0.009 | 3.58E-08 | 7157 |
|  | rs6738602 | 2 | 131901250 | G | T | -0.324 | 0.063 | 0.021 | 2.56E-07 | 7157 |
|  | rs7425339 | 2 | 162800402 | A | T | 0.079 | 0.017 | 0.623 | 6.17E-06 | 7157 |
|  | rs141162511 | 2 | 172761437 | T | C | 0.267 | 0.060 | 0.020 | 8.21E-06 | 7157 |
|  | rs13405349 | 2 | 211659523 | T | G | -0.101 | 0.022 | 0.185 | 3.35E-06 | 7157 |
|  | rs55721138 | 3 | 13583299 | T | C | -0.181 | 0.038 | 0.053 | 1.53E-06 | 7157 |
|  | rs11130797 | 3 | 60928407 | A | G | 0.083 | 0.018 | 0.300 | 4.54E-06 | 7157 |
|  | rs17482800 | 3 | 137801985 | A | G | -0.131 | 0.028 | 0.098 | 2.99E-06 | 7157 |
|  | rs10031375 | 4 | 37043069 | G | C | 0.075 | 0.017 | 0.558 | 5.46E-06 | 7157 |
|  | rs30743 | 5 | 135996882 | C | T | -0.095 | 0.020 | 0.763 | 2.99E-06 | 7157 |
|  | rs11154240 | 6 | 124457214 | C | T | 0.094 | 0.020 | 0.783 | 3.12E-06 | 7157 |
|  | rs17181296 | 6 | 154117109 | A | G | 0.122 | 0.027 | 0.114 | 6.76E-06 | 7157 |
|  | rs17725246 | 7 | 44542387 | C | T | 0.097 | 0.018 | 0.339 | 4.14E-08 | 7157 |
|  | rs17242295 | 7 | 136137436 | C | G | 0.081 | 0.018 | 0.319 | 6.63E-06 | 7157 |
|  | rs635634 | 9 | 133279427 | C | T | -0.154 | 0.021 | 0.798 | 1.31E-13 | 7157 |
|  | rs366590 | 11 | 16850893 | A | G | 0.084 | 0.019 | 0.288 | 7.83E-06 | 7157 |
|  | rs7130086 | 11 | 48217630 | A | C | -0.103 | 0.023 | 0.160 | 4.68E-06 | 7157 |
|  | rs4246215 | 11 | 61796827 | T | G | -0.111 | 0.017 | 0.436 | 4.47E-11 | 7157 |
|  | rs3741298 | 11 | 116786845 | T | C | -0.109 | 0.020 | 0.765 | 3.09E-08 | 7157 |
|  | rs2280696 | 12 | 53118972 | T | A | 0.254 | 0.025 | 0.126 | 3.69E-24 | 7157 |
|  | rs10735853 | 12 | 53924362 | C | T | -0.079 | 0.017 | 0.482 | 2.61E-06 | 7157 |
|  | rs10879574 | 12 | 73198257 | A | G | 0.089 | 0.020 | 0.237 | 7.40E-06 | 7157 |
|  | rs11646327 | 16 | 29117117 | G | A | 0.093 | 0.019 | 0.255 | 1.24E-06 | 7157 |
|  | rs56228609 | 16 | 56953853 | T | C | -0.104 | 0.019 | 0.276 | 2.73E-08 | 7157 |
|  | rs73005445 | 19 | 15830645 | G | A | -0.104 | 0.019 | 0.253 | 5.71E-08 | 7157 |
|  | rs429358 | 19 | 44908684 | C | T | 0.134 | 0.021 | 0.189 | 5.26E-10 | 7157 |
|  | rs134783 | 22 | 27255754 | C | T | 0.081 | 0.017 | 0.398 | 2.12E-06 | 7157 |
| Sterol ester (27:1/18:1) | rs12127700 | 1 | 38265767 | G | A | 0.079 | 0.018 | 0.455 | 9.36E-06 | 7174 |
|  | rs11591147 | 1 | 55039974 | T | G | -0.298 | 0.047 | 0.033 | 1.63E-10 | 7174 |
|  | rs11588501 | 1 | 56532951 | G | C | 0.242 | 0.055 | 0.024 | 9.06E-06 | 7174 |
|  | rs4845593 | 1 | 154078023 | C | T | -0.290 | 0.057 | 0.022 | 3.80E-07 | 7174 |
|  | rs11687710 | 2 | 21148196 | C | T | 0.089 | 0.019 | 0.257 | 3.09E-06 | 7174 |
|  | rs115419570 | 2 | 156522444 | T | C | 0.295 | 0.060 | 0.020 | 7.75E-07 | 7174 |
|  | rs74915447 | 4 | 72899760 | A | G | 0.263 | 0.044 | 0.038 | 2.80E-09 | 7174 |
|  | rs114483871 | 4 | 73124451 | T | C | 0.234 | 0.045 | 0.038 | 1.85E-07 | 7174 |
|  | rs182695896 | 4 | 73947510 | C | A | 0.338 | 0.053 | 0.025 | 1.78E-10 | 7174 |
|  | rs41441450 | 5 | 86717236 | T | C | -0.384 | 0.085 | 0.010 | 6.70E-06 | 7174 |
|  | rs10078182 | 5 | 118775347 | C | T | 0.268 | 0.059 | 0.022 | 4.93E-06 | 7174 |
|  | rs1061808 | 6 | 32168770 | G | T | -0.081 | 0.017 | 0.584 | 1.47E-06 | 7174 |
|  | rs7804103 | 7 | 19940796 | A | T | -0.159 | 0.035 | 0.062 | 5.05E-06 | 7174 |
|  | rs10812660 | 9 | 27742537 | A | T | 0.080 | 0.018 | 0.375 | 7.44E-06 | 7174 |
|  | rs151274583 | 9 | 116950689 | A | C | 0.288 | 0.063 | 0.018 | 4.99E-06 | 7174 |
|  | rs7932326 | 11 | 43971474 | A | G | 0.077 | 0.017 | 0.617 | 6.56E-06 | 7174 |
|  | rs10841310 | 12 | 19677962 | T | G | -0.106 | 0.019 | 0.246 | 5.70E-08 | 7174 |
|  | rs7487904 | 12 | 53231170 | G | C | 0.128 | 0.024 | 0.147 | 7.01E-08 | 7174 |
|  | rs10860778 | 12 | 101714807 | T | C | -0.082 | 0.017 | 0.491 | 1.16E-06 | 7174 |
|  | rs701081 | 12 | 124663423 | G | A | -0.084 | 0.017 | 0.603 | 1.09E-06 | 7174 |
|  | rs140055410 | 13 | 26901722 | T | C | 0.723 | 0.162 | 0.003 | 8.50E-06 | 7174 |
|  | rs73176681 | 13 | 31500828 | G | A | 0.191 | 0.040 | 0.046 | 2.33E-06 | 7174 |
|  | rs4906111 | 14 | 101393317 | C | T | -0.246 | 0.054 | 0.024 | 5.51E-06 | 7174 |
|  | rs9924448 | 16 | 73349244 | T | C | -0.077 | 0.017 | 0.557 | 5.93E-06 | 7174 |
|  | rs55833830 | 17 | 80953542 | A | C | -0.107 | 0.020 | 0.229 | 6.21E-08 | 7174 |
|  | rs117765824 | 18 | 53959046 | C | G | 0.318 | 0.067 | 0.016 | 2.32E-06 | 7174 |
|  | rs118068660 | 19 | 11079868 | T | C | -0.172 | 0.028 | 0.095 | 1.07E-09 | 7174 |
|  | rs73005445 | 19 | 15830645 | G | A | -0.087 | 0.019 | 0.253 | 5.51E-06 | 7174 |
|  | rs10404959 | 19 | 41031633 | C | T | -0.079 | 0.017 | 0.630 | 5.25E-06 | 7174 |
|  | rs7412 | 19 | 44908822 | T | C | -0.281 | 0.037 | 0.053 | 4.01E-14 | 7174 |
|  | rs1800961 | 20 | 44413724 | T | C | -0.210 | 0.037 | 0.052 | 1.71E-08 | 7174 |
| Sterol ester (27:1/18:2) | rs10796911 | 1 | 37622895 | C | G | 0.117 | 0.025 | 0.865 | 2.06E-06 | 7174 |
|  | rs11591147 | 1 | 55039974 | T | G | -0.358 | 0.047 | 0.033 | 1.69E-14 | 7174 |
|  | rs17395333 | 2 | 21100497 | G | A | 0.104 | 0.019 | 0.278 | 3.06E-08 | 7174 |
|  | rs61796597 | 4 | 36228194 | T | C | -0.209 | 0.045 | 0.038 | 2.97E-06 | 7174 |
|  | rs144064722 | 4 | 72540456 | G | A | 0.239 | 0.047 | 0.032 | 4.81E-07 | 7174 |
|  | rs79146711 | 4 | 73424911 | T | C | 0.284 | 0.041 | 0.045 | 4.02E-12 | 7174 |
|  | rs116302332 | 4 | 74505174 | T | C | 0.346 | 0.051 | 0.028 | 1.17E-11 | 7174 |
|  | rs73327386 | 5 | 172483791 | C | T | -0.638 | 0.138 | 0.004 | 3.66E-06 | 7174 |
|  | rs9405846 | 6 | 5542174 | G | A | 0.078 | 0.017 | 0.585 | 4.63E-06 | 7174 |
|  | rs7012814 | 8 | 9315848 | A | G | 0.079 | 0.017 | 0.512 | 3.22E-06 | 7174 |
|  | rs9330455 | 9 | 89816177 | A | C | 0.106 | 0.023 | 0.828 | 4.30E-06 | 7174 |
|  | rs117806675 | 10 | 5384541 | A | G | -0.463 | 0.098 | 0.008 | 2.58E-06 | 7174 |
|  | rs7932326 | 11 | 43971474 | A | G | 0.076 | 0.017 | 0.617 | 9.93E-06 | 7174 |
|  | rs2524299 | 11 | 61837310 | T | A | 0.139 | 0.019 | 0.259 | 3.25E-13 | 7174 |
|  | rs11604424 | 11 | 116780399 | T | C | -0.097 | 0.019 | 0.753 | 4.37E-07 | 7174 |
|  | rs80339463 | 12 | 19768671 | T | C | 0.325 | 0.073 | 0.014 | 7.90E-06 | 7174 |
|  | rs61941338 | 12 | 112377818 | T | C | 0.224 | 0.049 | 0.032 | 5.44E-06 | 7174 |
|  | rs144863685 | 16 | 88768837 | C | A | 0.500 | 0.110 | 0.006 | 6.12E-06 | 7174 |
|  | rs2521811 | 17 | 45856678 | C | T | -0.083 | 0.018 | 0.714 | 7.51E-06 | 7174 |
|  | rs281417 | 19 | 10308454 | T | C | -0.082 | 0.018 | 0.312 | 6.21E-06 | 7174 |
|  | rs118068660 | 19 | 11079868 | T | C | -0.194 | 0.028 | 0.095 | 6.81E-12 | 7174 |
|  | rs7412 | 19 | 44908822 | T | C | -0.464 | 0.037 | 0.053 | 7.79E-36 | 7174 |
|  | rs9304645 | 19 | 44947071 | A | G | -0.123 | 0.022 | 0.166 | 4.20E-08 | 7174 |
|  | rs1800961 | 20 | 44413724 | T | C | -0.216 | 0.037 | 0.052 | 7.42E-09 | 7174 |
| Sterol ester (27:1/18:3) | rs11591147 | 1 | 55039974 | T | G | -0.225 | 0.047 | 0.033 | 1.47E-06 | 7174 |
|  | rs1260326 | 2 | 27508073 | C | T | -0.105 | 0.017 | 0.651 | 1.64E-09 | 7174 |
|  | rs7355269 | 2 | 62072939 | C | T | 0.259 | 0.053 | 0.026 | 9.93E-07 | 7174 |
|  | rs137900618 | 2 | 136640311 | G | T | 0.605 | 0.132 | 0.004 | 5.00E-06 | 7174 |
|  | rs62294915 | 3 | 182509337 | C | T | 0.188 | 0.042 | 0.048 | 8.97E-06 | 7174 |
|  | rs13172653 | 5 | 50889130 | G | A | 0.095 | 0.020 | 0.228 | 2.62E-06 | 7174 |
|  | rs12199598 | 6 | 94453722 | T | C | 0.501 | 0.113 | 0.006 | 8.72E-06 | 7174 |
|  | rs34500711 | 9 | 130101985 | T | C | 0.210 | 0.044 | 0.038 | 1.59E-06 | 7174 |
|  | rs174527 | 11 | 61770929 | C | G | -0.086 | 0.017 | 0.444 | 5.00E-07 | 7174 |
|  | rs174584 | 11 | 61843278 | A | G | -0.183 | 0.017 | 0.410 | 4.02E-27 | 7174 |
|  | rs7957655 | 12 | 101754513 | A | G | -0.085 | 0.017 | 0.521 | 3.88E-07 | 7174 |
|  | rs2255531 | 12 | 120977112 | A | G | 0.093 | 0.017 | 0.395 | 4.25E-08 | 7174 |
|  | rs146997505 | 13 | 51210597 | G | A | 0.297 | 0.066 | 0.017 | 7.42E-06 | 7174 |
|  | rs319552 | 13 | 93173799 | C | G | 0.085 | 0.019 | 0.255 | 9.76E-06 | 7174 |
|  | rs113130033 | 14 | 89719965 | G | A | -0.217 | 0.041 | 0.043 | 1.22E-07 | 7174 |
|  | rs1980102 | 15 | 33326741 | A | C | 0.081 | 0.017 | 0.375 | 2.27E-06 | 7174 |
|  | rs58542926 | 19 | 19268740 | T | C | -0.169 | 0.034 | 0.063 | 1.01E-06 | 7174 |
|  | rs182611493 | 19 | 19347579 | G | A | -0.282 | 0.038 | 0.054 | 8.55E-14 | 7174 |
|  | rs7412 | 19 | 44908822 | T | C | -0.191 | 0.037 | 0.053 | 3.07E-07 | 7174 |
|  | rs1800961 | 20 | 44413724 | T | C | -0.240 | 0.037 | 0.052 | 1.30E-10 | 7174 |
| Sterol ester (27:1/20:2) | rs6755057 | 2 | 25435229 | C | G | -0.180 | 0.037 | 0.935 | 1.33E-06 | 5848 |
|  | rs7933 | 2 | 43225044 | G | A | 0.106 | 0.020 | 0.329 | 9.14E-08 | 5848 |
|  | rs4245791 | 2 | 43847292 | T | C | -0.256 | 0.022 | 0.773 | 9.13E-32 | 5848 |
|  | rs6709904 | 2 | 43853185 | G | A | -0.211 | 0.032 | 0.100 | 3.77E-11 | 5848 |
|  | rs79849855 | 2 | 60821224 | C | T | -0.311 | 0.067 | 0.020 | 4.07E-06 | 5848 |
|  | rs77590808 | 2 | 165115925 | A | G | -0.388 | 0.087 | 0.012 | 9.32E-06 | 5848 |
|  | rs36194627 | 2 | 225135785 | A | G | 0.293 | 0.064 | 0.023 | 4.70E-06 | 5848 |
|  | rs75823353 | 3 | 7487632 | G | A | 0.169 | 0.036 | 0.073 | 2.39E-06 | 5848 |
|  | rs182695896 | 4 | 73947510 | C | A | 0.286 | 0.059 | 0.025 | 1.52E-06 | 5848 |
|  | rs13121156 | 4 | 96997008 | C | T | -0.087 | 0.020 | 0.320 | 9.25E-06 | 5848 |
|  | rs74726976 | 5 | 90954717 | T | C | 0.227 | 0.051 | 0.034 | 9.64E-06 | 5848 |
|  | rs114838148 | 5 | 180649403 | T | C | 0.247 | 0.055 | 0.031 | 7.99E-06 | 5848 |
|  | rs9504426 | 6 | 5531009 | G | C | -0.092 | 0.019 | 0.378 | 1.16E-06 | 5848 |
|  | rs12527805 | 6 | 138623575 | C | T | 0.131 | 0.029 | 0.119 | 6.29E-06 | 5848 |
|  | rs1404999 | 7 | 39081865 | T | C | -0.102 | 0.020 | 0.314 | 3.99E-07 | 5848 |
|  | rs55837134 | 7 | 44532058 | A | G | 0.099 | 0.022 | 0.239 | 4.95E-06 | 5848 |
|  | rs700320 | 7 | 147774030 | T | C | -0.096 | 0.021 | 0.269 | 5.31E-06 | 5848 |
|  | rs56276984 | 8 | 23548281 | T | C | -0.168 | 0.036 | 0.069 | 3.57E-06 | 5848 |
|  | rs7853989 | 9 | 133256205 | C | G | -0.133 | 0.026 | 0.156 | 2.41E-07 | 5848 |
|  | rs118137821 | 10 | 7348594 | T | C | -0.269 | 0.059 | 0.026 | 4.91E-06 | 5848 |
|  | rs10826704 | 10 | 29809803 | T | C | -0.173 | 0.038 | 0.067 | 5.50E-06 | 5848 |
|  | rs1903973 | 10 | 53105888 | A | G | 0.086 | 0.018 | 0.556 | 2.62E-06 | 5848 |
|  | rs2244334 | 10 | 69935534 | C | T | -0.191 | 0.043 | 0.055 | 7.39E-06 | 5848 |
|  | rs6490297 | 12 | 120617356 | C | T | -0.094 | 0.020 | 0.313 | 2.04E-06 | 5848 |
|  | rs4778971 | 15 | 79180180 | G | A | -0.090 | 0.020 | 0.609 | 4.61E-06 | 5848 |
|  | rs7202845 | 16 | 26717568 | C | A | 0.099 | 0.020 | 0.670 | 6.41E-07 | 5848 |
|  | rs429358 | 19 | 44908684 | C | T | 0.162 | 0.024 | 0.189 | 9.20E-12 | 5848 |
| Sterol ester (27:1/20:3) | rs72660594 | 1 | 55170567 | C | T | -0.245 | 0.042 | 0.042 | 4.38E-09 | 7171 |
|  | rs79047909 | 2 | 10043013 | G | C | -0.516 | 0.107 | 0.006 | 1.38E-06 | 7171 |
|  | rs6761859 | 2 | 173386140 | A | G | 0.253 | 0.056 | 0.025 | 7.01E-06 | 7171 |
|  | rs112478356 | 2 | 173688057 | C | G | 0.316 | 0.067 | 0.017 | 2.29E-06 | 7171 |
|  | rs9310455 | 3 | 14644646 | A | C | -0.152 | 0.034 | 0.067 | 5.99E-06 | 7171 |
|  | rs79771052 | 3 | 72433728 | G | A | 0.392 | 0.081 | 0.012 | 1.25E-06 | 7171 |
|  | rs77645768 | 4 | 72803111 | A | G | 0.272 | 0.054 | 0.026 | 4.39E-07 | 7171 |
|  | rs79146711 | 4 | 73424911 | T | C | 0.186 | 0.041 | 0.045 | 5.45E-06 | 7171 |
|  | rs182695896 | 4 | 73947510 | C | A | 0.285 | 0.053 | 0.025 | 8.01E-08 | 7171 |
|  | rs1428498 | 5 | 38698557 | G | A | -0.081 | 0.017 | 0.351 | 2.48E-06 | 7171 |
|  | rs117754141 | 6 | 109151613 | G | C | 0.291 | 0.060 | 0.023 | 1.12E-06 | 7171 |
|  | rs4977342 | 9 | 19882242 | G | C | -0.093 | 0.020 | 0.251 | 2.47E-06 | 7171 |
|  | rs117079226 | 9 | 109704483 | A | G | 0.376 | 0.078 | 0.011 | 1.47E-06 | 7171 |
|  | rs80325645 | 9 | 131892383 | C | T | -0.465 | 0.097 | 0.008 | 1.53E-06 | 7171 |
|  | rs7036889 | 9 | 132336145 | C | A | -0.134 | 0.029 | 0.091 | 5.37E-06 | 7171 |
|  | rs2727271 | 11 | 61835886 | T | A | -0.170 | 0.019 | 0.258 | 3.98E-19 | 7171 |
|  | rs624605 | 11 | 76043576 | T | G | -0.084 | 0.017 | 0.444 | 1.35E-06 | 7171 |
|  | rs570929 | 11 | 88318708 | C | G | -0.082 | 0.017 | 0.548 | 9.70E-07 | 7171 |
|  | rs111575978 | 14 | 46693947 | A | G | 0.289 | 0.059 | 0.021 | 9.70E-07 | 7171 |
|  | rs2352991 | 14 | 95283517 | G | A | -0.089 | 0.019 | 0.735 | 3.61E-06 | 7171 |
|  | rs685850 | 15 | 53459505 | G | A | 0.083 | 0.017 | 0.557 | 7.01E-07 | 7171 |
|  | rs1135999 | 16 | 15038105 | G | A | -0.226 | 0.018 | 0.315 | 1.90E-36 | 7171 |
|  | rs56374730 | 16 | 15773795 | G | T | 0.112 | 0.018 | 0.296 | 1.15E-09 | 7171 |
|  | rs116917626 | 17 | 51561964 | T | C | -0.238 | 0.053 | 0.028 | 5.66E-06 | 7171 |
|  | rs199568207 | 17 | 82619804 | T | G | -0.192 | 0.041 | 0.044 | 2.99E-06 | 7171 |
|  | rs3752149 | 19 | 15729265 | C | A | -0.092 | 0.019 | 0.259 | 2.25E-06 | 7171 |
|  | rs187429064 | 19 | 19269704 | G | A | -0.236 | 0.038 | 0.054 | 4.92E-10 | 7171 |
|  | rs7412 | 19 | 44908822 | T | C | -0.203 | 0.037 | 0.053 | 5.69E-08 | 7171 |
|  | rs62217148 | 20 | 18988572 | T | C | -0.195 | 0.040 | 0.049 | 1.43E-06 | 7171 |
|  | rs7020 | 20 | 25297964 | A | G | 0.085 | 0.017 | 0.446 | 4.67E-07 | 7171 |
|  | rs1800961 | 20 | 44413724 | T | C | -0.239 | 0.037 | 0.052 | 1.55E-10 | 7171 |
| Sterol ester (27:1/20:4) | rs3767941 | 1 | 40824563 | T | C | 0.105 | 0.022 | 0.166 | 2.01E-06 | 7174 |
|  | rs11591147 | 1 | 55039974 | T | G | -0.297 | 0.046 | 0.033 | 1.30E-10 | 7174 |
|  | rs3820438 | 1 | 201955621 | C | G | 0.084 | 0.018 | 0.291 | 3.54E-06 | 7174 |
|  | rs186913978 | 2 | 17147264 | T | G | -0.414 | 0.077 | 0.012 | 8.60E-08 | 7174 |
|  | rs74915447 | 4 | 72899760 | A | G | 0.196 | 0.044 | 0.038 | 7.81E-06 | 7174 |
|  | rs182695896 | 4 | 73947510 | C | A | 0.296 | 0.052 | 0.025 | 1.82E-08 | 7174 |
|  | rs6812370 | 4 | 174026533 | A | G | -0.344 | 0.066 | 0.017 | 1.55E-07 | 7174 |
|  | rs6860806 | 5 | 132304843 | G | A | 0.076 | 0.017 | 0.420 | 7.76E-06 | 7174 |
|  | rs9388335 | 6 | 124438655 | A | T | 0.078 | 0.017 | 0.532 | 3.35E-06 | 7174 |
|  | rs7837272 | 8 | 22193452 | C | G | 0.890 | 0.194 | 0.002 | 4.47E-06 | 7174 |
|  | rs117825513 | 9 | 22932648 | T | C | 0.385 | 0.085 | 0.011 | 6.64E-06 | 7174 |
|  | rs113603971 | 10 | 13808836 | C | T | -0.161 | 0.036 | 0.057 | 9.89E-06 | 7174 |
|  | rs11022403 | 11 | 12542442 | T | C | 0.084 | 0.018 | 0.304 | 4.15E-06 | 7174 |
|  | rs79136768 | 11 | 61594967 | G | A | -0.220 | 0.045 | 0.037 | 9.10E-07 | 7174 |
|  | rs3741252 | 11 | 61744026 | T | C | 0.212 | 0.025 | 0.134 | 6.46E-18 | 7174 |
|  | rs174528 | 11 | 61776027 | C | T | -0.648 | 0.015 | 0.414 | 0 | 7174 |
|  | rs147981159 | 11 | 62056826 | A | G | -0.327 | 0.042 | 0.043 | 6.76E-15 | 7174 |
|  | rs113248417 | 11 | 62059462 | G | A | -0.268 | 0.045 | 0.037 | 2.59E-09 | 7174 |
|  | rs4382917 | 11 | 62454004 | A | G | 0.146 | 0.020 | 0.256 | 1.24E-13 | 7174 |
|  | rs2229738 | 11 | 68794860 | T | C | -0.153 | 0.023 | 0.156 | 7.01E-11 | 7174 |
|  | rs646299 | 13 | 38255030 | T | A | -0.075 | 0.017 | 0.601 | 9.30E-06 | 7174 |
|  | rs9555673 | 13 | 110137139 | T | A | -0.077 | 0.017 | 0.431 | 5.31E-06 | 7174 |
|  | rs117084735 | 14 | 98823032 | T | C | 0.320 | 0.071 | 0.014 | 6.51E-06 | 7174 |
|  | rs1135999 | 16 | 15038105 | G | A | -0.101 | 0.018 | 0.315 | 1.23E-08 | 7174 |
|  | rs223841 | 16 | 57423359 | G | A | -0.101 | 0.022 | 0.172 | 4.23E-06 | 7174 |
|  | rs117186386 | 16 | 87676514 | C | T | 0.178 | 0.035 | 0.060 | 4.60E-07 | 7174 |
|  | rs635196 | 18 | 44179195 | C | T | 0.105 | 0.021 | 0.184 | 1.05E-06 | 7174 |
|  | rs2469034 | 18 | 73089879 | C | A | -0.091 | 0.020 | 0.788 | 7.35E-06 | 7174 |
|  | rs138482835 | 19 | 7662522 | A | G | -0.172 | 0.037 | 0.061 | 2.39E-06 | 7174 |
|  | rs187429064 | 19 | 19269704 | G | A | -0.254 | 0.037 | 0.054 | 1.21E-11 | 7174 |
|  | rs7412 | 19 | 44908822 | T | C | -0.310 | 0.037 | 0.053 | 4.29E-17 | 7174 |
|  | rs7020 | 20 | 25297964 | A | G | 0.075 | 0.017 | 0.446 | 8.25E-06 | 7174 |
|  | rs1800961 | 20 | 44413724 | T | C | -0.167 | 0.037 | 0.052 | 5.95E-06 | 7174 |
| Sterol ester (27:1/20:5) | rs4908782 | 1 | 8828055 | T | C | -0.079 | 0.017 | 0.354 | 5.59E-06 | 7174 |
|  | rs34343838 | 1 | 169678964 | A | G | 0.124 | 0.026 | 0.120 | 1.33E-06 | 7174 |
|  | rs2625222 | 1 | 204934830 | C | T | -0.125 | 0.027 | 0.107 | 3.84E-06 | 7174 |
|  | rs71629271 | 3 | 176095628 | C | T | 0.160 | 0.036 | 0.063 | 8.36E-06 | 7174 |
|  | rs2054498 | 4 | 37392086 | G | A | -0.085 | 0.017 | 0.538 | 2.88E-07 | 7174 |
|  | rs59807465 | 4 | 189640546 | G | C | -0.307 | 0.069 | 0.017 | 7.85E-06 | 7174 |
|  | rs4490551 | 5 | 23468036 | A | G | -0.094 | 0.021 | 0.205 | 9.25E-06 | 7174 |
|  | rs143678376 | 5 | 85519569 | A | C | 0.152 | 0.031 | 0.078 | 1.12E-06 | 7174 |
|  | rs12515163 | 5 | 113784003 | G | A | -0.082 | 0.018 | 0.400 | 3.21E-06 | 7174 |
|  | rs980814 | 6 | 156440204 | G | T | 0.079 | 0.017 | 0.455 | 3.55E-06 | 7174 |
|  | rs7780677 | 7 | 132693734 | A | C | -0.076 | 0.017 | 0.409 | 7.21E-06 | 7174 |
|  | rs75897070 | 8 | 3077293 | C | G | 0.169 | 0.038 | 0.051 | 8.62E-06 | 7174 |
|  | rs11986253 | 8 | 132397243 | C | G | -0.106 | 0.023 | 0.175 | 3.00E-06 | 7174 |
|  | rs5896690 | 9 | 16421864 | T | C | -0.076 | 0.017 | 0.464 | 5.48E-06 | 7174 |
|  | rs7947954 | 11 | 42657198 | C | T | 0.111 | 0.023 | 0.155 | 1.55E-06 | 7174 |
|  | rs3019200 | 11 | 61481911 | A | C | -0.101 | 0.021 | 0.803 | 2.37E-06 | 7174 |
|  | rs174527 | 11 | 61770929 | C | G | -0.129 | 0.017 | 0.444 | 4.44E-14 | 7174 |
|  | rs174581 | 11 | 61839211 | A | G | -0.411 | 0.016 | 0.410 | 3.56E-133 | 7174 |
|  | rs7936002 | 11 | 62451557 | G | A | 0.110 | 0.020 | 0.257 | 2.53E-08 | 7174 |
|  | rs508049 | 11 | 68908029 | T | C | -0.158 | 0.029 | 0.098 | 3.35E-08 | 7174 |
|  | rs1481977 | 11 | 103598670 | G | A | 0.113 | 0.024 | 0.861 | 3.79E-06 | 7174 |
|  | rs10860778 | 12 | 101714807 | T | C | -0.086 | 0.017 | 0.491 | 3.23E-07 | 7174 |
|  | rs17548303 | 15 | 53447803 | G | C | 0.086 | 0.019 | 0.262 | 6.25E-06 | 7174 |
|  | rs62025936 | 16 | 10434179 | G | A | 0.107 | 0.023 | 0.165 | 2.75E-06 | 7174 |
|  | rs182611493 | 19 | 19347579 | G | A | -0.181 | 0.038 | 0.054 | 1.87E-06 | 7174 |
|  | rs449647 | 19 | 44905307 | T | A | -0.133 | 0.025 | 0.136 | 1.07E-07 | 7174 |
|  | rs8132104 | 21 | 41847687 | A | G | 0.086 | 0.019 | 0.268 | 6.98E-06 | 7174 |
| Sterol ester (27:1/22:6) | rs17110517 | 1 | 54369167 | T | C | -0.175 | 0.035 | 0.061 | 7.85E-07 | 7173 |
|  | rs11591147 | 1 | 55039974 | T | G | -0.238 | 0.047 | 0.033 | 3.90E-07 | 7173 |
|  | rs77708260 | 1 | 119349977 | A | G | 0.245 | 0.055 | 0.025 | 7.41E-06 | 7173 |
|  | rs149229417 | 3 | 68666055 | A | C | 0.318 | 0.066 | 0.017 | 1.44E-06 | 7173 |
|  | rs113651041 | 3 | 185542441 | T | C | 0.076 | 0.017 | 0.385 | 9.97E-06 | 7173 |
|  | rs116794590 | 4 | 13024335 | C | T | -0.248 | 0.053 | 0.026 | 3.28E-06 | 7173 |
|  | rs72657952 | 4 | 87901015 | G | C | 0.245 | 0.055 | 0.024 | 8.06E-06 | 7173 |
|  | rs115647337 | 4 | 91336094 | T | G | -0.198 | 0.043 | 0.041 | 3.24E-06 | 7173 |
|  | rs75101588 | 6 | 24387133 | C | T | 0.259 | 0.050 | 0.030 | 2.23E-07 | 7173 |
|  | rs73769662 | 6 | 120046093 | A | G | -0.130 | 0.029 | 0.089 | 9.33E-06 | 7173 |
|  | rs2075083 | 7 | 17680332 | T | G | 0.074 | 0.017 | 0.581 | 9.42E-06 | 7173 |
|  | rs35473591 | 11 | 61818856 | T | C | -0.201 | 0.017 | 0.406 | 2.14E-32 | 7173 |
|  | rs2229738 | 11 | 68794860 | T | C | -0.114 | 0.023 | 0.156 | 1.28E-06 | 7173 |
|  | rs9559135 | 13 | 107677268 | G | A | 0.086 | 0.018 | 0.284 | 3.17E-06 | 7173 |
|  | rs111751059 | 14 | 20010775 | T | C | -0.164 | 0.037 | 0.055 | 7.18E-06 | 7173 |
|  | rs10150075 | 14 | 72571809 | T | C | -0.205 | 0.038 | 0.053 | 8.96E-08 | 7173 |
|  | rs77456635 | 15 | 64865848 | A | G | -0.268 | 0.054 | 0.024 | 5.68E-07 | 7173 |
|  | rs11633878 | 15 | 93239374 | A | G | -0.097 | 0.020 | 0.719 | 1.97E-06 | 7173 |
|  | rs117061362 | 15 | 101196347 | T | G | 0.199 | 0.041 | 0.045 | 1.26E-06 | 7173 |
|  | rs72852391 | 17 | 78966695 | T | C | 0.213 | 0.045 | 0.039 | 2.18E-06 | 7173 |
|  | rs4072727 | 18 | 48039184 | C | T | -0.093 | 0.017 | 0.385 | 7.11E-08 | 7173 |
|  | rs7412 | 19 | 44908822 | T | C | -0.269 | 0.037 | 0.053 | 5.61E-13 | 7173 |
|  | rs11696517 | 20 | 57125699 | A | G | -0.101 | 0.023 | 0.160 | 8.19E-06 | 7173 |
| Ceramide (d40:1) | rs12477218 | 2 | 12194447 | G | A | 0.075 | 0.017 | 0.492 | 9.41E-06 | 7117 |
|  | rs1260326 | 2 | 27508073 | C | T | -0.099 | 0.018 | 0.651 | 1.40E-08 | 7117 |
|  | rs114910162 | 2 | 141867363 | T | C | 0.195 | 0.040 | 0.049 | 9.74E-07 | 7117 |
|  | rs78573518 | 2 | 200379367 | G | A | -0.479 | 0.108 | 0.007 | 9.82E-06 | 7117 |
|  | rs10025139 | 4 | 36311616 | C | T | 0.090 | 0.020 | 0.222 | 7.10E-06 | 7117 |
|  | rs6535831 | 4 | 151996582 | T | G | 0.114 | 0.024 | 0.861 | 2.85E-06 | 7117 |
|  | rs4704352 | 5 | 76672693 | A | G | 0.089 | 0.020 | 0.773 | 8.80E-06 | 7117 |
|  | rs2255744 | 7 | 47368432 | G | A | -0.088 | 0.020 | 0.768 | 9.31E-06 | 7117 |
|  | rs11982486 | 7 | 95426286 | C | T | 0.089 | 0.020 | 0.241 | 4.77E-06 | 7117 |
|  | rs28505920 | 8 | 118268727 | A | T | 0.123 | 0.022 | 0.171 | 3.58E-08 | 7117 |
|  | rs1346910 | 9 | 71460729 | T | C | -0.103 | 0.022 | 0.823 | 3.46E-06 | 7117 |
|  | rs56143221 | 10 | 73731455 | A | G | -0.111 | 0.024 | 0.144 | 4.14E-06 | 7117 |
|  | rs12366015 | 11 | 117120135 | G | A | -0.088 | 0.019 | 0.717 | 2.25E-06 | 7117 |
|  | rs11064535 | 12 | 111854 | A | G | -0.159 | 0.036 | 0.059 | 9.98E-06 | 7117 |
|  | rs117873718 | 12 | 67485111 | C | T | -0.223 | 0.048 | 0.033 | 4.03E-06 | 7117 |
|  | rs7157785 | 14 | 63768838 | T | G | 0.148 | 0.025 | 0.125 | 5.10E-09 | 7117 |
|  | rs865934 | 14 | 98057258 | G | T | -0.283 | 0.063 | 0.018 | 8.11E-06 | 7117 |
|  | rs12927793 | 16 | 89171782 | A | G | -0.079 | 0.017 | 0.530 | 2.13E-06 | 7117 |
|  | rs11868222 | 17 | 81124244 | C | T | -0.084 | 0.019 | 0.266 | 9.25E-06 | 7117 |
|  | rs1917921 | 18 | 5754635 | T | A | -0.085 | 0.019 | 0.267 | 8.91E-06 | 7117 |
|  | rs187429064 | 19 | 19269704 | G | A | -0.232 | 0.038 | 0.054 | 1.02E-09 | 7117 |
|  | rs58489806 | 19 | 19346108 | T | C | -0.178 | 0.032 | 0.076 | 1.72E-08 | 7117 |
|  | rs2945048 | 19 | 38556464 | A | T | 0.088 | 0.019 | 0.241 | 7.16E-06 | 7117 |
|  | rs364585 | 20 | 12982070 | G | A | -0.263 | 0.017 | 0.661 | 1.02E-50 | 7117 |
|  | rs2236124 | 20 | 13126078 | C | T | 0.128 | 0.023 | 0.146 | 5.60E-08 | 7117 |
|  | rs56406916 | 20 | 13295543 | A | G | 0.173 | 0.037 | 0.057 | 3.96E-06 | 7117 |
|  | rs293566 | 20 | 32510074 | C | T | -0.085 | 0.017 | 0.467 | 5.58E-07 | 7117 |
|  | rs5767288 | 22 | 46600357 | A | G | -0.113 | 0.022 | 0.190 | 1.42E-07 | 7117 |
| Ceramide (d40:2) | rs61834063 | 1 | 240877505 | G | A | -0.113 | 0.024 | 0.209 | 2.10E-06 | 5287 |
|  | rs114316759 | 2 | 132139262 | A | G | -0.198 | 0.045 | 0.059 | 8.85E-06 | 5287 |
|  | rs115833524 | 2 | 167928158 | T | C | 0.231 | 0.049 | 0.042 | 2.92E-06 | 5287 |
|  | rs147060788 | 2 | 240329826 | C | T | 0.249 | 0.053 | 0.034 | 3.08E-06 | 5287 |
|  | rs6537481 | 4 | 147474942 | G | A | -0.114 | 0.025 | 0.189 | 6.59E-06 | 5287 |
|  | rs79320828 | 7 | 151291137 | A | C | 0.425 | 0.095 | 0.013 | 7.88E-06 | 5287 |
|  | rs6559102 | 8 | 6166747 | A | G | -0.418 | 0.088 | 0.013 | 2.12E-06 | 5287 |
|  | rs10869023 | 9 | 71444815 | T | C | 0.160 | 0.035 | 0.083 | 6.84E-06 | 5287 |
|  | rs117064201 | 9 | 76271174 | A | C | -0.302 | 0.067 | 0.022 | 7.66E-06 | 5287 |
|  | rs139456652 | 11 | 6300702 | A | G | 0.214 | 0.043 | 0.059 | 8.01E-07 | 5287 |
|  | rs117671667 | 11 | 36075190 | T | C | 0.267 | 0.057 | 0.034 | 2.83E-06 | 5287 |
|  | rs629047 | 11 | 97717674 | G | A | -0.089 | 0.020 | 0.434 | 5.44E-06 | 5287 |
|  | rs964184 | 11 | 116778201 | C | G | -0.140 | 0.027 | 0.849 | 1.64E-07 | 5287 |
|  | rs17748108 | 13 | 76834291 | C | T | 0.145 | 0.030 | 0.123 | 1.72E-06 | 5287 |
|  | rs12878001 | 14 | 63772911 | G | T | 0.216 | 0.029 | 0.124 | 5.67E-14 | 5287 |
|  | rs111745969 | 15 | 92566992 | A | G | -0.135 | 0.029 | 0.135 | 2.59E-06 | 5287 |
|  | rs111984437 | 19 | 1150087 | T | C | -0.425 | 0.095 | 0.013 | 7.43E-06 | 5287 |
|  | rs187429064 | 19 | 19269704 | G | A | -0.220 | 0.044 | 0.054 | 7.10E-07 | 5287 |
|  | rs12974674 | 19 | 38559208 | C | G | 0.106 | 0.022 | 0.241 | 2.54E-06 | 5287 |
|  | rs61751862 | 19 | 48629610 | C | G | 0.250 | 0.054 | 0.031 | 3.75E-06 | 5287 |
|  | rs438568 | 20 | 12978039 | G | A | -0.228 | 0.020 | 0.666 | 3.66E-29 | 5287 |
| Ceramide (d42:1) | rs62621917 | 1 | 114581582 | C | T | 0.569 | 0.128 | 0.005 | 9.12E-06 | 7174 |
|  | rs3923037 | 2 | 20948514 | C | A | -0.077 | 0.017 | 0.393 | 5.55E-06 | 7174 |
|  | rs1260326 | 2 | 27508073 | C | T | -0.089 | 0.017 | 0.651 | 3.64E-07 | 7174 |
|  | rs10201832 | 2 | 62042163 | T | A | 0.262 | 0.058 | 0.021 | 7.31E-06 | 7174 |
|  | rs114910162 | 2 | 141867363 | T | C | 0.186 | 0.040 | 0.049 | 2.99E-06 | 7174 |
|  | rs78573518 | 2 | 200379367 | G | A | -0.500 | 0.108 | 0.007 | 3.38E-06 | 7174 |
|  | rs11130463 | 3 | 55192986 | C | A | 0.115 | 0.026 | 0.881 | 8.35E-06 | 7174 |
|  | rs10033592 | 4 | 40237784 | G | T | -0.082 | 0.018 | 0.655 | 3.36E-06 | 7174 |
|  | rs114822153 | 4 | 72372827 | A | G | 0.237 | 0.051 | 0.030 | 3.21E-06 | 7174 |
|  | rs72941422 | 4 | 131441209 | C | T | 0.084 | 0.019 | 0.277 | 6.55E-06 | 7174 |
|  | rs57099041 | 4 | 175907374 | T | C | 0.154 | 0.034 | 0.064 | 5.50E-06 | 7174 |
|  | rs186176314 | 5 | 16573000 | C | T | 0.269 | 0.058 | 0.023 | 3.13E-06 | 7174 |
|  | rs6977389 | 7 | 95390043 | A | G | -0.078 | 0.017 | 0.560 | 4.10E-06 | 7174 |
|  | rs112631157 | 8 | 17632332 | T | C | -0.538 | 0.120 | 0.006 | 7.37E-06 | 7174 |
|  | rs28505920 | 8 | 118268727 | A | T | 0.101 | 0.022 | 0.171 | 5.57E-06 | 7174 |
|  | rs117888980 | 9 | 107021609 | G | T | -0.294 | 0.064 | 0.019 | 3.74E-06 | 7174 |
|  | rs7123454 | 11 | 116833462 | A | C | -0.111 | 0.022 | 0.811 | 2.87E-07 | 7174 |
|  | rs7970512 | 12 | 67513820 | A | C | -0.212 | 0.048 | 0.035 | 9.54E-06 | 7174 |
|  | rs11105228 | 12 | 89259912 | A | G | -0.086 | 0.018 | 0.337 | 1.13E-06 | 7174 |
|  | rs56368930 | 14 | 33070106 | C | G | 0.380 | 0.083 | 0.012 | 4.44E-06 | 7174 |
|  | rs12927793 | 16 | 89171782 | A | G | -0.079 | 0.017 | 0.530 | 2.34E-06 | 7174 |
|  | rs72954979 | 18 | 71270815 | A | G | -0.209 | 0.047 | 0.034 | 7.77E-06 | 7174 |
|  | rs7252628 | 19 | 18958653 | A | G | 0.084 | 0.019 | 0.303 | 9.98E-06 | 7174 |
|  | rs8100204 | 19 | 19282905 | A | G | -0.166 | 0.022 | 0.171 | 1.29E-13 | 7174 |
|  | rs2945048 | 19 | 38556464 | A | T | 0.093 | 0.019 | 0.241 | 1.88E-06 | 7174 |
|  | rs364585 | 20 | 12982070 | G | A | -0.223 | 0.017 | 0.661 | 6.11E-37 | 7174 |
|  | rs2236124 | 20 | 13126078 | C | T | 0.148 | 0.023 | 0.146 | 2.53E-10 | 7174 |
| Ceramide (d42:2) | rs1042034 | 2 | 21002409 | T | C | 0.103 | 0.019 | 0.728 | 3.54E-08 | 7173 |
|  | rs1260326 | 2 | 27508073 | C | T | -0.095 | 0.017 | 0.651 | 5.58E-08 | 7173 |
|  | rs55817533 | 2 | 120863835 | C | A | 0.130 | 0.026 | 0.116 | 7.05E-07 | 7173 |
|  | rs116327692 | 2 | 137364592 | T | C | 0.347 | 0.072 | 0.014 | 1.28E-06 | 7173 |
|  | rs779714 | 3 | 7468297 | T | C | 0.121 | 0.026 | 0.870 | 4.03E-06 | 7173 |
|  | rs9839625 | 3 | 31247248 | A | C | -0.087 | 0.019 | 0.260 | 5.88E-06 | 7173 |
|  | rs11713732 | 3 | 54513590 | T | C | 0.112 | 0.024 | 0.160 | 2.12E-06 | 7173 |
|  | rs142985829 | 3 | 102651475 | T | C | 0.362 | 0.079 | 0.012 | 4.79E-06 | 7173 |
|  | rs6535831 | 4 | 151996582 | T | G | 0.121 | 0.024 | 0.861 | 6.38E-07 | 7173 |
|  | rs6837202 | 4 | 168325002 | A | G | -0.209 | 0.046 | 0.033 | 6.22E-06 | 7173 |
|  | rs7769878 | 6 | 56110664 | G | C | 0.116 | 0.025 | 0.134 | 5.19E-06 | 7173 |
|  | rs28505920 | 8 | 118268727 | A | T | 0.117 | 0.022 | 0.171 | 1.66E-07 | 7173 |
|  | rs11507232 | 9 | 71470570 | A | G | 0.127 | 0.029 | 0.095 | 9.99E-06 | 7173 |
|  | rs1572595 | 10 | 8406642 | T | C | -0.084 | 0.017 | 0.382 | 1.24E-06 | 7173 |
|  | rs61836115 | 10 | 14273933 | G | T | 0.123 | 0.028 | 0.105 | 8.54E-06 | 7173 |
|  | rs6479877 | 10 | 63118358 | G | C | -0.079 | 0.017 | 0.461 | 2.17E-06 | 7173 |
|  | rs12763964 | 10 | 70843134 | C | T | -0.132 | 0.021 | 0.206 | 1.88E-10 | 7173 |
|  | rs12356523 | 10 | 130351351 | A | G | 0.098 | 0.021 | 0.191 | 3.31E-06 | 7173 |
|  | rs5141 | 11 | 116831407 | C | T | -0.125 | 0.025 | 0.864 | 3.87E-07 | 7173 |
|  | rs1169306 | 12 | 121000508 | T | C | -0.098 | 0.017 | 0.360 | 1.51E-08 | 7173 |
|  | rs7493416 | 14 | 63888972 | C | T | 0.081 | 0.018 | 0.691 | 7.57E-06 | 7173 |
|  | rs79098941 | 16 | 52635159 | C | T | 0.264 | 0.056 | 0.024 | 2.39E-06 | 7173 |
|  | rs117704733 | 17 | 37499294 | T | C | 0.137 | 0.030 | 0.088 | 4.39E-06 | 7173 |
|  | rs6503695 | 17 | 42347515 | C | T | 0.084 | 0.018 | 0.314 | 3.28E-06 | 7173 |
|  | rs72999033 | 19 | 19255823 | T | C | -0.199 | 0.034 | 0.062 | 7.12E-09 | 7173 |
|  | rs187429064 | 19 | 19269704 | G | A | -0.288 | 0.038 | 0.054 | 2.75E-14 | 7173 |
|  | rs2945048 | 19 | 38556464 | A | T | 0.086 | 0.019 | 0.241 | 9.80E-06 | 7173 |
|  | rs364585 | 20 | 12982070 | G | A | -0.265 | 0.017 | 0.661 | 7.12E-52 | 7173 |
|  | rs2236124 | 20 | 13126078 | C | T | 0.170 | 0.023 | 0.146 | 4.26E-13 | 7173 |
|  | rs78709083 | 21 | 28580399 | T | C | 0.204 | 0.044 | 0.039 | 3.19E-06 | 7173 |
|  | rs5767288 | 22 | 46600357 | A | G | -0.141 | 0.021 | 0.190 | 4.31E-11 | 7173 |
| Cholesterol | rs11591147 | 1 | 55039974 | T | G | -0.214 | 0.047 | 0.033 | 5.51E-06 | 7166 |
|  | rs12562141 | 1 | 181952433 | C | T | -0.139 | 0.030 | 0.083 | 4.52E-06 | 7166 |
|  | rs180958041 | 2 | 170149397 | T | C | -0.365 | 0.077 | 0.012 | 2.22E-06 | 7166 |
|  | rs34462361 | 2 | 215511513 | G | A | 0.085 | 0.019 | 0.256 | 8.05E-06 | 7166 |
|  | rs669552 | 3 | 172335874 | G | A | -0.108 | 0.023 | 0.156 | 3.40E-06 | 7166 |
|  | rs143214193 | 3 | 194964435 | A | G | -0.495 | 0.109 | 0.008 | 5.35E-06 | 7166 |
|  | rs11730766 | 4 | 72366511 | T | C | 0.253 | 0.052 | 0.029 | 9.67E-07 | 7166 |
|  | rs77645768 | 4 | 72803111 | A | G | 0.242 | 0.054 | 0.026 | 7.16E-06 | 7166 |
|  | rs182695896 | 4 | 73947510 | C | A | 0.268 | 0.053 | 0.025 | 5.02E-07 | 7166 |
|  | rs75059546 | 5 | 126190702 | A | C | -0.278 | 0.061 | 0.020 | 5.45E-06 | 7166 |
|  | rs2237478 | 7 | 50678097 | T | C | -0.099 | 0.021 | 0.198 | 2.52E-06 | 7166 |
|  | rs11781607 | 8 | 11644899 | A | G | -0.081 | 0.018 | 0.322 | 7.89E-06 | 7166 |
|  | rs112411704 | 9 | 2958367 | G | A | 0.303 | 0.066 | 0.018 | 4.51E-06 | 7166 |
|  | rs10812660 | 9 | 27742537 | A | T | 0.086 | 0.018 | 0.375 | 1.57E-06 | 7166 |
|  | rs78721266 | 9 | 96213656 | G | A | 0.191 | 0.041 | 0.044 | 3.90E-06 | 7166 |
|  | rs12552499 | 9 | 121135217 | G | A | -0.189 | 0.040 | 0.047 | 2.50E-06 | 7166 |
|  | rs2879630 | 10 | 51918584 | A | G | -0.101 | 0.021 | 0.189 | 1.80E-06 | 7166 |
|  | rs7909144 | 10 | 52240944 | G | A | 0.076 | 0.017 | 0.593 | 7.66E-06 | 7166 |
|  | rs12366015 | 11 | 117120135 | G | A | -0.082 | 0.019 | 0.717 | 9.91E-06 | 7166 |
|  | rs1271671 | 12 | 31810895 | A | G | 0.235 | 0.051 | 0.028 | 4.70E-06 | 7166 |
|  | rs1259751 | 12 | 31822701 | T | C | 0.238 | 0.051 | 0.028 | 4.02E-06 | 7166 |
|  | rs75611814 | 13 | 23150340 | C | T | 0.124 | 0.027 | 0.103 | 5.82E-06 | 7166 |
|  | rs11858279 | 15 | 58420774 | C | T | 0.088 | 0.018 | 0.303 | 1.43E-06 | 7166 |
|  | rs11857386 | 15 | 58428969 | C | G | -0.079 | 0.017 | 0.471 | 4.57E-06 | 7166 |
|  | rs16963800 | 16 | 14576910 | C | T | 0.110 | 0.023 | 0.160 | 1.36E-06 | 7166 |
|  | rs223841 | 16 | 57423359 | G | A | -0.098 | 0.022 | 0.172 | 9.81E-06 | 7166 |
|  | rs141401656 | 18 | 35844265 | C | T | -0.303 | 0.068 | 0.015 | 9.94E-06 | 7166 |
|  | rs17702736 | 18 | 39850559 | C | T | -0.077 | 0.017 | 0.389 | 8.24E-06 | 7166 |
|  | rs118068660 | 19 | 11079868 | T | C | -0.161 | 0.028 | 0.095 | 1.16E-08 | 7166 |
|  | rs73005445 | 19 | 15830645 | G | A | -0.090 | 0.019 | 0.253 | 2.94E-06 | 7166 |
|  | rs429358 | 19 | 44908684 | C | T | 0.106 | 0.022 | 0.189 | 9.26E-07 | 7166 |
|  | rs114243848 | 19 | 49823108 | C | A | -0.228 | 0.042 | 0.042 | 7.17E-08 | 7166 |
|  | rs57405165 | 20 | 40665923 | T | C | -0.107 | 0.023 | 0.155 | 3.52E-06 | 7166 |
|  | rs1800961 | 20 | 44413724 | T | C | -0.180 | 0.037 | 0.052 | 1.57E-06 | 7166 |
|  | rs76093749 | 22 | 50075106 | T | C | 0.395 | 0.087 | 0.010 | 5.10E-06 | 7166 |
| Diacylglycerol (16:0_18:1) | rs6699688 | 1 | 77136442 | T | C | -0.081 | 0.018 | 0.563 | 9.29E-06 | 6293 |
|  | rs78056622 | 1 | 184416563 | G | C | 0.417 | 0.092 | 0.011 | 5.51E-06 | 6293 |
|  | rs55857911 | 1 | 230598232 | T | C | 0.148 | 0.032 | 0.093 | 3.66E-06 | 6293 |
|  | rs1260326 | 2 | 27508073 | C | T | -0.099 | 0.019 | 0.651 | 9.81E-08 | 6293 |
|  | rs17033194 | 3 | 10598526 | C | G | 0.102 | 0.023 | 0.213 | 5.69E-06 | 6293 |
|  | rs115473344 | 5 | 120107843 | A | G | 0.374 | 0.084 | 0.011 | 8.89E-06 | 6293 |
|  | rs93 | 7 | 24401906 | C | T | 0.082 | 0.018 | 0.656 | 9.99E-06 | 6293 |
|  | rs117905902 | 7 | 76683164 | G | A | 0.191 | 0.043 | 0.048 | 7.21E-06 | 6293 |
|  | rs180999317 | 7 | 157355450 | A | G | -0.654 | 0.137 | 0.005 | 2.04E-06 | 6293 |
|  | rs11775636 | 8 | 142752432 | A | G | -0.122 | 0.024 | 0.158 | 4.60E-07 | 6293 |
|  | rs117730598 | 9 | 2770506 | C | T | 0.239 | 0.053 | 0.030 | 6.90E-06 | 6293 |
|  | rs72753379 | 9 | 120350676 | T | C | 0.231 | 0.048 | 0.039 | 1.28E-06 | 6293 |
|  | rs111568723 | 9 | 132470502 | G | A | 0.259 | 0.058 | 0.026 | 8.97E-06 | 6293 |
|  | rs964184 | 11 | 116778201 | C | G | -0.186 | 0.025 | 0.849 | 4.04E-14 | 6293 |
|  | rs118099052 | 12 | 44462630 | T | A | 0.379 | 0.076 | 0.015 | 7.22E-07 | 6293 |
|  | rs36094040 | 12 | 72431183 | C | T | -0.087 | 0.019 | 0.348 | 4.64E-06 | 6293 |
|  | rs1884216 | 14 | 36679967 | A | C | -0.085 | 0.018 | 0.623 | 4.29E-06 | 6293 |
|  | rs117614422 | 15 | 40252330 | G | C | -0.173 | 0.038 | 0.065 | 4.70E-06 | 6293 |
|  | rs28689929 | 15 | 93154632 | T | C | -0.082 | 0.018 | 0.378 | 8.05E-06 | 6293 |
|  | rs9796793 | 16 | 30484331 | A | G | -0.123 | 0.028 | 0.125 | 7.82E-06 | 6293 |
|  | rs76606887 | 16 | 78596301 | C | T | 0.218 | 0.049 | 0.035 | 8.68E-06 | 6293 |
|  | rs447117 | 18 | 5149977 | G | C | -0.083 | 0.018 | 0.651 | 7.43E-06 | 6293 |
| Diacylglycerol (16:0_18:2) | rs76587848 | 1 | 186849811 | T | G | 0.178 | 0.040 | 0.058 | 9.36E-06 | 6075 |
|  | rs6687291 | 1 | 230586281 | A | G | 0.098 | 0.020 | 0.320 | 7.54E-07 | 6075 |
|  | rs2004776 | 1 | 230712956 | T | C | -0.108 | 0.023 | 0.196 | 2.83E-06 | 6075 |
|  | rs1260326 | 2 | 27508073 | C | T | -0.102 | 0.019 | 0.651 | 6.21E-08 | 6075 |
|  | rs7569544 | 2 | 42290376 | A | C | -0.091 | 0.021 | 0.265 | 9.64E-06 | 6075 |
|  | rs78504564 | 2 | 42953102 | T | C | -0.630 | 0.134 | 0.005 | 2.49E-06 | 6075 |
|  | rs140314539 | 2 | 220215369 | A | G | 0.308 | 0.064 | 0.021 | 1.38E-06 | 6075 |
|  | rs149992851 | 3 | 55269076 | G | A | 0.293 | 0.065 | 0.020 | 7.72E-06 | 6075 |
|  | rs6810359 | 3 | 188792426 | T | G | -0.192 | 0.041 | 0.053 | 3.61E-06 | 6075 |
|  | rs34606692 | 5 | 149755706 | T | C | -0.114 | 0.023 | 0.196 | 7.38E-07 | 6075 |
|  | rs758398 | 6 | 28352897 | T | C | -0.116 | 0.023 | 0.183 | 7.42E-07 | 6075 |
|  | rs11756450 | 6 | 64409802 | A | C | 0.094 | 0.019 | 0.324 | 1.61E-06 | 6075 |
|  | rs17145750 | 7 | 73612048 | T | C | -0.134 | 0.026 | 0.141 | 3.14E-07 | 6075 |
|  | rs111934503 | 8 | 130543708 | C | T | 0.669 | 0.140 | 0.005 | 1.66E-06 | 6075 |
|  | rs34792804 | 9 | 8556190 | G | T | -0.173 | 0.039 | 0.060 | 8.70E-06 | 6075 |
|  | rs148163011 | 9 | 71279650 | G | C | -0.281 | 0.060 | 0.024 | 2.83E-06 | 6075 |
|  | rs112122274 | 9 | 136257994 | G | A | -0.253 | 0.053 | 0.036 | 1.68E-06 | 6075 |
|  | rs4262724 | 11 | 23915220 | A | C | 0.092 | 0.020 | 0.717 | 6.46E-06 | 6075 |
|  | rs964184 | 11 | 116778201 | C | G | -0.225 | 0.025 | 0.849 | 1.64E-19 | 6075 |
|  | rs57747810 | 12 | 19924761 | A | C | -0.404 | 0.084 | 0.012 | 1.70E-06 | 6075 |
|  | rs11182480 | 12 | 44386579 | C | T | 0.341 | 0.071 | 0.018 | 1.78E-06 | 6075 |
|  | rs7165355 | 15 | 38675336 | C | G | -0.107 | 0.023 | 0.192 | 3.92E-06 | 6075 |
|  | rs1032956 | 15 | 97687850 | C | T | 0.083 | 0.019 | 0.451 | 7.88E-06 | 6075 |
|  | rs2868424 | 16 | 11584433 | A | T | 0.111 | 0.025 | 0.165 | 8.47E-06 | 6075 |
|  | rs35986054 | 17 | 1623049 | C | T | 0.107 | 0.023 | 0.196 | 4.30E-06 | 6075 |
|  | rs4806901 | 19 | 3057222 | C | T | 0.121 | 0.026 | 0.151 | 4.18E-06 | 6075 |
|  | rs7254036 | 19 | 41202334 | T | C | 0.082 | 0.018 | 0.532 | 7.01E-06 | 6075 |
|  | rs144838649 | 21 | 44845081 | T | C | -0.580 | 0.125 | 0.005 | 3.52E-06 | 6075 |
| Diacylglycerol (16:1_18:1) | rs143808674 | 1 | 81671654 | G | A | -0.186 | 0.042 | 0.048 | 8.76E-06 | 6215 |
|  | rs1260326 | 2 | 27508073 | C | T | -0.112 | 0.019 | 0.651 | 2.15E-09 | 6215 |
|  | rs10022344 | 4 | 124943164 | T | C | 0.106 | 0.024 | 0.181 | 8.41E-06 | 6215 |
|  | rs2524075 | 6 | 31276082 | T | C | -0.107 | 0.022 | 0.208 | 9.22E-07 | 6215 |
|  | rs2182380 | 6 | 43900875 | G | A | 0.261 | 0.054 | 0.031 | 1.65E-06 | 6215 |
|  | rs77134026 | 7 | 36495127 | A | G | 0.258 | 0.055 | 0.031 | 3.20E-06 | 6215 |
|  | rs11775636 | 8 | 142752432 | A | G | -0.113 | 0.024 | 0.158 | 3.05E-06 | 6215 |
|  | rs3829088 | 9 | 23713682 | C | T | 0.102 | 0.022 | 0.203 | 5.65E-06 | 6215 |
|  | rs112122274 | 9 | 136257994 | G | A | -0.260 | 0.053 | 0.036 | 7.58E-07 | 6215 |
|  | rs35162925 | 10 | 62375635 | A | C | -0.285 | 0.061 | 0.023 | 3.12E-06 | 6215 |
|  | rs964184 | 11 | 116778201 | C | G | -0.164 | 0.025 | 0.849 | 3.17E-11 | 6215 |
|  | rs11182480 | 12 | 44386579 | C | T | 0.345 | 0.070 | 0.018 | 7.16E-07 | 6215 |
|  | rs7955732 | 12 | 72331215 | T | G | -0.089 | 0.018 | 0.423 | 1.24E-06 | 6215 |
|  | rs111750990 | 12 | 96298692 | C | A | -0.214 | 0.046 | 0.041 | 2.65E-06 | 6215 |
|  | rs12050879 | 15 | 39983732 | A | G | -0.278 | 0.062 | 0.021 | 7.99E-06 | 6215 |
|  | rs4419068 | 16 | 71309806 | A | G | -0.093 | 0.021 | 0.237 | 9.89E-06 | 6215 |
|  | rs908150 | 17 | 66540315 | C | T | 0.081 | 0.018 | 0.428 | 9.56E-06 | 6215 |
|  | rs10502551 | 18 | 30887893 | G | C | -0.213 | 0.042 | 0.051 | 3.21E-07 | 6215 |
|  | rs147782497 | 18 | 78352143 | A | C | 0.267 | 0.060 | 0.024 | 9.11E-06 | 6215 |
|  | rs140283718 | 19 | 30685856 | A | T | -0.266 | 0.057 | 0.026 | 3.29E-06 | 6215 |
|  | rs78263224 | 19 | 52961172 | C | T | 0.206 | 0.046 | 0.043 | 7.61E-06 | 6215 |
|  | rs117771237 | 22 | 40189878 | A | C | 0.384 | 0.085 | 0.012 | 7.02E-06 | 6215 |
| Diacylglycerol (18:1_18:1) | rs17596144 | 1 | 220805303 | T | C | 0.131 | 0.024 | 0.152 | 4.26E-08 | 6884 |
|  | rs1042034 | 2 | 21002409 | T | C | 0.104 | 0.019 | 0.728 | 4.97E-08 | 6884 |
|  | rs1260326 | 2 | 27508073 | C | T | -0.123 | 0.018 | 0.651 | 5.88E-12 | 6884 |
|  | rs3770813 | 2 | 36549412 | T | G | -0.246 | 0.052 | 0.028 | 2.37E-06 | 6884 |
|  | rs1060743 | 2 | 127068957 | G | A | -0.083 | 0.019 | 0.291 | 8.86E-06 | 6884 |
|  | rs76950187 | 2 | 128427075 | G | A | 0.160 | 0.033 | 0.074 | 9.58E-07 | 6884 |
|  | rs10169217 | 2 | 212010621 | G | A | -0.098 | 0.018 | 0.676 | 7.18E-08 | 6884 |
|  | rs116011373 | 3 | 87298641 | A | G | -0.186 | 0.040 | 0.049 | 3.06E-06 | 6884 |
|  | rs9865586 | 3 | 132015999 | G | A | -0.107 | 0.023 | 0.165 | 2.33E-06 | 6884 |
|  | rs62291573 | 4 | 4999649 | C | T | 0.107 | 0.021 | 0.214 | 6.91E-07 | 6884 |
|  | rs116796527 | 4 | 62344980 | T | C | -0.337 | 0.072 | 0.014 | 2.68E-06 | 6884 |
|  | rs7782961 | 7 | 6295952 | T | A | 0.095 | 0.020 | 0.232 | 3.51E-06 | 6884 |
|  | rs35332062 | 7 | 73597712 | A | G | -0.144 | 0.026 | 0.122 | 3.11E-08 | 6884 |
|  | rs268 | 8 | 19956018 | G | A | 0.281 | 0.058 | 0.023 | 1.26E-06 | 6884 |
|  | rs2954021 | 8 | 125469835 | G | A | -0.076 | 0.017 | 0.538 | 9.05E-06 | 6884 |
|  | rs12365864 | 11 | 116618319 | G | A | 0.105 | 0.023 | 0.166 | 5.55E-06 | 6884 |
|  | rs964184 | 11 | 116778201 | C | G | -0.264 | 0.023 | 0.849 | 3.74E-29 | 6884 |
|  | rs36094040 | 12 | 72431183 | C | T | -0.083 | 0.018 | 0.348 | 4.96E-06 | 6884 |
|  | rs139500046 | 12 | 96850670 | C | A | -0.393 | 0.081 | 0.011 | 1.13E-06 | 6884 |
|  | rs137871178 | 14 | 58138012 | C | T | 0.299 | 0.067 | 0.017 | 8.42E-06 | 6884 |
|  | rs62046303 | 16 | 82018393 | C | T | 0.325 | 0.070 | 0.017 | 3.80E-06 | 6884 |
|  | rs147782497 | 18 | 78352143 | A | C | 0.259 | 0.056 | 0.024 | 4.75E-06 | 6884 |
|  | rs157582 | 19 | 44892962 | T | C | 0.094 | 0.021 | 0.223 | 4.43E-06 | 6884 |
| Diacylglycerol (18:1_18:2) | rs72694990 | 1 | 150251390 | A | G | -0.121 | 0.026 | 0.129 | 4.00E-06 | 6613 |
|  | rs34621709 | 1 | 220806736 | A | C | 0.112 | 0.022 | 0.185 | 6.40E-07 | 6613 |
|  | rs2352723 | 1 | 230156121 | C | T | -0.112 | 0.020 | 0.769 | 3.76E-08 | 6613 |
|  | rs1042034 | 2 | 21002409 | T | C | 0.093 | 0.019 | 0.728 | 1.65E-06 | 6613 |
|  | rs1260326 | 2 | 27508073 | C | T | -0.129 | 0.018 | 0.651 | 9.98E-13 | 6613 |
|  | rs11684166 | 2 | 111060064 | A | G | 0.106 | 0.023 | 0.177 | 3.49E-06 | 6613 |
|  | rs9973939 | 2 | 212078809 | G | A | -0.082 | 0.018 | 0.660 | 8.26E-06 | 6613 |
|  | rs116011373 | 3 | 87298641 | A | G | -0.182 | 0.041 | 0.049 | 7.38E-06 | 6613 |
|  | rs57500102 | 3 | 171536246 | A | G | -0.499 | 0.111 | 0.007 | 7.12E-06 | 6613 |
|  | rs114444157 | 3 | 176180291 | C | T | 0.149 | 0.033 | 0.078 | 7.23E-06 | 6613 |
|  | rs62291573 | 4 | 4999649 | C | T | 0.100 | 0.022 | 0.214 | 4.75E-06 | 6613 |
|  | rs17007519 | 4 | 141672748 | T | C | -0.301 | 0.060 | 0.022 | 6.24E-07 | 6613 |
|  | rs4691907 | 4 | 163302506 | T | G | 0.078 | 0.017 | 0.532 | 7.60E-06 | 6613 |
|  | rs7745829 | 6 | 14146172 | G | A | -0.112 | 0.025 | 0.147 | 5.66E-06 | 6613 |
|  | rs1233660 | 6 | 28292472 | A | T | -0.098 | 0.021 | 0.221 | 3.00E-06 | 6613 |
|  | rs75008187 | 6 | 64441454 | A | G | 0.129 | 0.026 | 0.128 | 9.80E-07 | 6613 |
|  | rs141116593 | 6 | 154880792 | G | C | 0.155 | 0.033 | 0.074 | 2.82E-06 | 6613 |
|  | rs7782961 | 7 | 6295952 | T | A | 0.092 | 0.021 | 0.232 | 9.94E-06 | 6613 |
|  | rs79624003 | 7 | 73598455 | G | A | -0.172 | 0.027 | 0.122 | 8.98E-11 | 6613 |
|  | rs268 | 8 | 19956018 | G | A | 0.336 | 0.058 | 0.023 | 8.26E-09 | 6613 |
|  | rs75596627 | 8 | 43650502 | A | G | 0.128 | 0.028 | 0.114 | 4.62E-06 | 6613 |
|  | rs117416257 | 10 | 5793091 | T | C | -0.604 | 0.131 | 0.005 | 3.83E-06 | 6613 |
|  | rs75082203 | 10 | 88606986 | A | G | 0.349 | 0.078 | 0.013 | 8.40E-06 | 6613 |
|  | rs72836628 | 10 | 112142696 | T | C | -0.090 | 0.019 | 0.297 | 2.11E-06 | 6613 |
|  | rs138578034 | 11 | 17897155 | T | G | -0.689 | 0.154 | 0.003 | 7.80E-06 | 6613 |
|  | rs181215046 | 11 | 32530936 | G | T | -0.477 | 0.105 | 0.007 | 5.32E-06 | 6613 |
|  | rs142868058 | 11 | 62717059 | T | C | -0.559 | 0.125 | 0.005 | 8.45E-06 | 6613 |
|  | rs12365864 | 11 | 116618319 | G | A | 0.124 | 0.024 | 0.166 | 1.58E-07 | 6613 |
|  | rs964184 | 11 | 116778201 | C | G | -0.291 | 0.024 | 0.849 | 3.26E-34 | 6613 |
|  | rs10773708 | 12 | 129796667 | A | G | -0.087 | 0.019 | 0.683 | 3.71E-06 | 6613 |
|  | rs55788915 | 16 | 23267056 | A | C | -0.251 | 0.053 | 0.031 | 1.92E-06 | 6613 |
|  | rs7239207 | 18 | 62009100 | C | T | -0.086 | 0.019 | 0.694 | 4.99E-06 | 6613 |
|  | rs4608457 | 19 | 20106360 | T | C | -0.200 | 0.042 | 0.045 | 1.81E-06 | 6613 |
|  | rs11670344 | 19 | 33144357 | C | A | 0.087 | 0.019 | 0.278 | 5.24E-06 | 6613 |
|  | rs157582 | 19 | 44892962 | T | C | 0.118 | 0.021 | 0.223 | 1.66E-08 | 6613 |
|  | rs11668758 | 19 | 44971423 | T | C | 0.096 | 0.019 | 0.307 | 4.54E-07 | 6613 |
| Diacylglycerol (18:1_18:3) | rs6583035 | 1 | 107643461 | C | T | -0.112 | 0.024 | 0.162 | 2.46E-06 | 6572 |
|  | rs141217537 | 1 | 186241818 | C | T | 0.219 | 0.049 | 0.033 | 6.59E-06 | 6572 |
|  | rs55865364 | 1 | 196932199 | G | A | 0.231 | 0.050 | 0.032 | 4.22E-06 | 6572 |
|  | rs1260326 | 2 | 27508073 | C | T | -0.090 | 0.018 | 0.651 | 8.83E-07 | 6572 |
|  | rs7599400 | 2 | 36315434 | A | C | 0.490 | 0.096 | 0.008 | 3.46E-07 | 6572 |
|  | rs116011373 | 3 | 87298641 | A | G | -0.191 | 0.041 | 0.049 | 3.06E-06 | 6572 |
|  | rs117518401 | 3 | 133895761 | T | C | 0.112 | 0.023 | 0.173 | 1.48E-06 | 6572 |
|  | rs114441702 | 4 | 141972167 | G | A | -0.257 | 0.056 | 0.027 | 4.52E-06 | 6572 |
|  | rs77442856 | 5 | 153405520 | A | G | -0.123 | 0.027 | 0.122 | 4.21E-06 | 6572 |
|  | rs112374014 | 5 | 178403016 | A | G | 0.318 | 0.071 | 0.016 | 7.33E-06 | 6572 |
|  | rs6958772 | 7 | 10662626 | C | G | 0.096 | 0.020 | 0.244 | 2.27E-06 | 6572 |
|  | rs13237829 | 7 | 74460551 | A | G | -0.081 | 0.017 | 0.503 | 3.03E-06 | 6572 |
|  | rs268 | 8 | 19956018 | G | A | 0.281 | 0.059 | 0.023 | 2.39E-06 | 6572 |
|  | rs180761622 | 8 | 32454379 | T | C | 0.235 | 0.049 | 0.033 | 1.73E-06 | 6572 |
|  | rs75224949 | 10 | 24164242 | T | C | -0.189 | 0.042 | 0.049 | 7.27E-06 | 6572 |
|  | rs10749053 | 10 | 110816937 | C | T | -0.142 | 0.028 | 0.874 | 2.57E-07 | 6572 |
|  | rs964184 | 11 | 116778201 | C | G | -0.171 | 0.024 | 0.849 | 1.43E-12 | 6572 |
|  | rs61950149 | 13 | 20969363 | C | T | 0.101 | 0.023 | 0.185 | 7.99E-06 | 6572 |
|  | rs112075774 | 16 | 23263670 | A | G | -0.165 | 0.034 | 0.077 | 1.21E-06 | 6572 |
|  | rs151145497 | 18 | 78520543 | G | C | 0.186 | 0.041 | 0.046 | 6.35E-06 | 6572 |
|  | rs4608457 | 19 | 20106360 | T | C | -0.193 | 0.043 | 0.045 | 6.51E-06 | 6572 |
|  | rs12463051 | 19 | 54419778 | A | G | 0.086 | 0.019 | 0.353 | 5.58E-06 | 6572 |
| Phosphatidylcholine (16:0_0:0) | rs7529794 | 1 | 39937698 | T | G | 0.134 | 0.019 | 0.250 | 4.32E-12 | 7174 |
|  | rs571344 | 1 | 98498507 | C | T | 0.082 | 0.018 | 0.630 | 4.23E-06 | 7174 |
|  | rs12116819 | 1 | 163474114 | A | G | -0.246 | 0.051 | 0.030 | 1.24E-06 | 7174 |
|  | rs1260326 | 2 | 27508073 | C | T | -0.099 | 0.017 | 0.651 | 1.56E-08 | 7174 |
|  | rs2691773 | 2 | 155599766 | T | C | 0.075 | 0.017 | 0.556 | 8.10E-06 | 7174 |
|  | rs62231613 | 3 | 4762993 | A | G | -0.442 | 0.093 | 0.009 | 2.10E-06 | 7174 |
|  | rs4955144 | 3 | 32353724 | A | C | 0.082 | 0.018 | 0.708 | 5.91E-06 | 7174 |
|  | rs76530357 | 3 | 77008436 | T | G | 0.256 | 0.055 | 0.025 | 2.70E-06 | 7174 |
|  | rs34782816 | 3 | 120300479 | T | C | -0.094 | 0.021 | 0.203 | 5.65E-06 | 7174 |
|  | rs75563920 | 4 | 80232181 | C | T | -0.126 | 0.026 | 0.117 | 1.29E-06 | 7174 |
|  | rs17029364 | 4 | 152971920 | G | A | -0.097 | 0.022 | 0.189 | 6.68E-06 | 7174 |
|  | rs75668753 | 5 | 117857598 | G | A | -0.297 | 0.059 | 0.021 | 5.49E-07 | 7174 |
|  | rs2714309 | 6 | 7298054 | A | G | 0.080 | 0.017 | 0.391 | 3.69E-06 | 7174 |
|  | rs76309137 | 7 | 95573476 | T | C | -0.340 | 0.076 | 0.013 | 7.34E-06 | 7174 |
|  | rs73200277 | 8 | 18126210 | T | C | 0.284 | 0.059 | 0.022 | 1.45E-06 | 7174 |
|  | rs73693512 | 8 | 79831072 | A | G | 0.238 | 0.053 | 0.026 | 6.97E-06 | 7174 |
|  | rs117001547 | 8 | 100059985 | G | C | 0.277 | 0.062 | 0.020 | 8.34E-06 | 7174 |
|  | rs117646116 | 10 | 20803487 | T | C | 0.161 | 0.036 | 0.058 | 8.59E-06 | 7174 |
|  | rs191695863 | 11 | 40267436 | A | C | -0.546 | 0.119 | 0.006 | 4.41E-06 | 7174 |
|  | rs149105878 | 12 | 17851399 | C | A | -0.733 | 0.163 | 0.003 | 7.12E-06 | 7174 |
|  | rs17477122 | 12 | 26619127 | G | A | -0.110 | 0.024 | 0.142 | 4.77E-06 | 7174 |
|  | rs11051913 | 12 | 32302096 | C | T | -0.088 | 0.019 | 0.250 | 6.29E-06 | 7174 |
|  | rs9565669 | 13 | 81460606 | A | G | -0.076 | 0.017 | 0.483 | 6.32E-06 | 7174 |
|  | rs77116141 | 13 | 111120067 | C | T | 0.110 | 0.024 | 0.137 | 5.99E-06 | 7174 |
|  | rs7168957 | 15 | 61598332 | T | C | -0.169 | 0.034 | 0.937 | 9.02E-07 | 7174 |
|  | rs12926135 | 16 | 23623429 | A | C | -0.080 | 0.018 | 0.377 | 9.11E-06 | 7174 |
|  | rs62067063 | 17 | 9294060 | C | T | 0.086 | 0.018 | 0.580 | 1.50E-06 | 7174 |
|  | rs181353915 | 17 | 49398516 | G | A | 0.290 | 0.065 | 0.017 | 9.48E-06 | 7174 |
|  | rs78117248 | 19 | 1052854 | G | A | -0.493 | 0.110 | 0.006 | 7.96E-06 | 7174 |
|  | rs148313235 | 19 | 54065411 | G | T | -0.345 | 0.067 | 0.017 | 3.16E-07 | 7174 |
| Phosphatidylcholine (18:0_0:0) | rs7529794 | 1 | 39937698 | T | G | 0.101 | 0.019 | 0.250 | 1.65E-07 | 7173 |
|  | rs1146461 | 1 | 94942936 | A | G | -0.080 | 0.017 | 0.463 | 1.57E-06 | 7173 |
|  | rs11582724 | 1 | 201132736 | T | C | -0.078 | 0.017 | 0.575 | 4.64E-06 | 7173 |
|  | rs73040346 | 2 | 178827066 | C | T | -0.217 | 0.049 | 0.031 | 8.28E-06 | 7173 |
|  | rs56121911 | 3 | 45251126 | G | A | 0.131 | 0.029 | 0.097 | 5.24E-06 | 7173 |
|  | rs76530357 | 3 | 77008436 | T | G | 0.266 | 0.054 | 0.025 | 1.05E-06 | 7173 |
|  | rs143183807 | 5 | 51956035 | A | C | 0.140 | 0.031 | 0.084 | 6.94E-06 | 7173 |
|  | rs75668753 | 5 | 117857598 | G | A | -0.268 | 0.059 | 0.021 | 6.12E-06 | 7173 |
|  | rs78392466 | 6 | 101082967 | T | A | -0.209 | 0.044 | 0.038 | 1.98E-06 | 7173 |
|  | rs797821 | 7 | 83961536 | C | T | -0.075 | 0.017 | 0.561 | 7.21E-06 | 7173 |
|  | rs76309137 | 7 | 95573476 | T | C | -0.351 | 0.076 | 0.013 | 3.63E-06 | 7173 |
|  | rs186790715 | 7 | 124474397 | C | T | 0.339 | 0.066 | 0.017 | 2.45E-07 | 7173 |
|  | rs11780085 | 8 | 11680104 | T | A | 0.452 | 0.102 | 0.008 | 8.92E-06 | 7173 |
|  | rs1018070 | 8 | 18416117 | A | T | 0.177 | 0.035 | 0.061 | 5.83E-07 | 7173 |
|  | rs10095602 | 8 | 53669039 | T | C | 0.176 | 0.038 | 0.052 | 3.79E-06 | 7173 |
|  | rs145366219 | 9 | 33013634 | G | A | -0.158 | 0.034 | 0.067 | 2.68E-06 | 7173 |
|  | rs7048143 | 9 | 91806878 | T | C | -0.088 | 0.020 | 0.247 | 9.79E-06 | 7173 |
|  | rs76179782 | 10 | 70458313 | A | C | 0.199 | 0.044 | 0.044 | 5.97E-06 | 7173 |
|  | rs117140365 | 10 | 74861163 | C | G | -0.329 | 0.067 | 0.016 | 9.83E-07 | 7173 |
|  | rs77751297 | 12 | 95853480 | T | G | 0.113 | 0.023 | 0.165 | 6.41E-07 | 7173 |
|  | rs7322909 | 13 | 81451428 | A | C | -0.079 | 0.017 | 0.531 | 3.00E-06 | 7173 |
|  | rs4000217 | 13 | 110757928 | G | T | 0.075 | 0.017 | 0.439 | 8.23E-06 | 7173 |
|  | rs79713567 | 14 | 36841591 | T | C | -0.187 | 0.041 | 0.050 | 4.75E-06 | 7173 |
|  | rs6496477 | 15 | 88358204 | A | G | 0.078 | 0.018 | 0.407 | 8.70E-06 | 7173 |
|  | rs59634655 | 16 | 78457680 | T | G | -0.108 | 0.023 | 0.163 | 2.46E-06 | 7173 |
|  | rs140427655 | 19 | 54124989 | T | C | -0.301 | 0.067 | 0.016 | 7.53E-06 | 7173 |
|  | rs76023792 | 20 | 9964250 | A | T | 0.217 | 0.047 | 0.034 | 3.87E-06 | 7173 |
|  | rs77900286 | 21 | 40397531 | C | T | -0.500 | 0.097 | 0.008 | 2.88E-07 | 7173 |
| Phosphatidylcholine (18:1_0:0) | rs7529794 | 1 | 39937698 | T | G | 0.126 | 0.019 | 0.250 | 8.15E-11 | 7174 |
|  | rs61852444 | 1 | 246692321 | C | G | -0.093 | 0.018 | 0.314 | 2.50E-07 | 7174 |
|  | rs76881617 | 2 | 27256675 | T | C | 0.172 | 0.038 | 0.049 | 7.48E-06 | 7174 |
|  | rs11720139 | 3 | 46995266 | G | C | 0.078 | 0.017 | 0.384 | 4.97E-06 | 7174 |
|  | rs11727825 | 4 | 24133433 | A | G | 0.093 | 0.019 | 0.263 | 1.43E-06 | 7174 |
|  | rs115972339 | 4 | 77085694 | G | A | -0.211 | 0.046 | 0.035 | 5.16E-06 | 7174 |
|  | rs146435288 | 5 | 2069974 | C | A | 0.153 | 0.033 | 0.070 | 4.37E-06 | 7174 |
|  | rs2714309 | 6 | 7298054 | A | G | 0.080 | 0.017 | 0.391 | 3.76E-06 | 7174 |
|  | rs6455722 | 6 | 161340057 | G | T | -0.081 | 0.018 | 0.335 | 4.26E-06 | 7174 |
|  | rs9691107 | 7 | 100920067 | C | T | -0.076 | 0.017 | 0.436 | 7.83E-06 | 7174 |
|  | rs35583283 | 8 | 18396999 | C | G | 0.102 | 0.021 | 0.193 | 1.70E-06 | 7174 |
|  | rs10833360 | 11 | 20577887 | T | C | -0.084 | 0.018 | 0.670 | 2.38E-06 | 7174 |
|  | rs7935110 | 11 | 41087025 | G | C | 0.082 | 0.017 | 0.633 | 1.70E-06 | 7174 |
|  | rs11212840 | 11 | 109005691 | C | T | -0.080 | 0.018 | 0.317 | 8.07E-06 | 7174 |
|  | rs6539823 | 12 | 84167129 | C | T | 0.079 | 0.017 | 0.650 | 6.07E-06 | 7174 |
|  | rs9527967 | 13 | 59324563 | G | A | -0.086 | 0.019 | 0.277 | 5.07E-06 | 7174 |
|  | rs6496477 | 15 | 88358204 | A | G | 0.079 | 0.018 | 0.407 | 7.32E-06 | 7174 |
|  | rs71379351 | 17 | 49270778 | C | T | -0.081 | 0.017 | 0.483 | 1.54E-06 | 7174 |
|  | rs142604671 | 17 | 61552499 | T | C | 0.147 | 0.031 | 0.093 | 1.56E-06 | 7174 |
|  | rs55908410 | 17 | 61714452 | C | T | -0.314 | 0.070 | 0.015 | 7.15E-06 | 7174 |
|  | rs72845685 | 17 | 68496359 | C | G | 0.095 | 0.021 | 0.190 | 6.52E-06 | 7174 |
|  | rs138778351 | 20 | 16819023 | A | C | -0.468 | 0.105 | 0.007 | 9.37E-06 | 7174 |
| Phosphatidylcholine (18:2_0:0) | rs7529794 | 1 | 39937698 | T | G | 0.117 | 0.019 | 0.250 | 1.76E-09 | 7174 |
|  | rs6685742 | 1 | 171705634 | A | G | 0.088 | 0.019 | 0.257 | 5.73E-06 | 7174 |
|  | rs59319733 | 1 | 246693388 | A | G | -0.085 | 0.018 | 0.339 | 1.20E-06 | 7174 |
|  | rs149768482 | 2 | 204687512 | T | C | 0.198 | 0.035 | 0.067 | 1.46E-08 | 7174 |
|  | rs73218874 | 3 | 110722896 | G | A | -0.207 | 0.047 | 0.038 | 9.92E-06 | 7174 |
|  | rs114796420 | 3 | 122279149 | A | G | 0.275 | 0.061 | 0.019 | 7.90E-06 | 7174 |
|  | rs115972339 | 4 | 77085694 | G | A | -0.225 | 0.046 | 0.035 | 1.04E-06 | 7174 |
|  | rs150917153 | 4 | 182275394 | T | C | 0.176 | 0.037 | 0.055 | 1.45E-06 | 7174 |
|  | rs72707125 | 4 | 184839680 | G | A | 0.222 | 0.050 | 0.030 | 7.93E-06 | 7174 |
|  | rs17126576 | 8 | 18381562 | G | C | -0.075 | 0.017 | 0.424 | 8.21E-06 | 7174 |
|  | rs2610042 | 8 | 56509388 | G | A | 0.274 | 0.056 | 0.977 | 1.19E-06 | 7174 |
|  | rs10833360 | 11 | 20577887 | T | C | -0.088 | 0.018 | 0.670 | 6.25E-07 | 7174 |
|  | rs139683825 | 11 | 30336511 | G | A | -0.209 | 0.044 | 0.038 | 2.52E-06 | 7174 |
|  | rs174560 | 11 | 61814292 | C | T | 0.131 | 0.017 | 0.383 | 3.22E-14 | 7174 |
|  | rs414383 | 17 | 31622748 | T | A | -0.081 | 0.018 | 0.671 | 4.37E-06 | 7174 |
|  | rs71379351 | 17 | 49270778 | C | T | -0.077 | 0.017 | 0.483 | 4.95E-06 | 7174 |
|  | rs8069148 | 17 | 68491696 | C | A | 0.089 | 0.019 | 0.252 | 3.14E-06 | 7174 |
|  | rs111336143 | 18 | 57256007 | A | T | 0.260 | 0.055 | 0.027 | 1.85E-06 | 7174 |
| Phosphatidylcholine (20:4_0:0) | rs76469829 | 1 | 29962031 | T | C | -0.216 | 0.048 | 0.037 | 5.69E-06 | 6235 |
|  | rs76513766 | 1 | 172130754 | A | G | 0.378 | 0.084 | 0.012 | 7.01E-06 | 6235 |
|  | rs9967725 | 2 | 56382464 | T | A | -0.101 | 0.019 | 0.350 | 6.79E-08 | 6235 |
|  | rs200932481 | 4 | 150117372 | C | T | 0.489 | 0.102 | 0.008 | 1.66E-06 | 6235 |
|  | rs4708901 | 6 | 161271441 | A | G | -0.099 | 0.022 | 0.215 | 5.37E-06 | 6235 |
|  | rs191895023 | 7 | 2289982 | A | C | 0.805 | 0.177 | 0.002 | 5.63E-06 | 6235 |
|  | rs142999489 | 7 | 95974040 | G | C | -0.194 | 0.040 | 0.056 | 1.09E-06 | 6235 |
|  | rs62511275 | 8 | 116151644 | G | C | 0.220 | 0.046 | 0.042 | 2.08E-06 | 6235 |
|  | rs10733608 | 9 | 114386150 | T | G | 0.083 | 0.018 | 0.485 | 3.34E-06 | 6235 |
|  | rs10884272 | 10 | 106220847 | A | G | -0.083 | 0.018 | 0.365 | 6.46E-06 | 6235 |
|  | rs3741252 | 11 | 61744026 | T | C | 0.166 | 0.026 | 0.134 | 3.34E-10 | 6235 |
|  | rs35473591 | 11 | 61818856 | T | C | -0.535 | 0.017 | 0.406 | 4.23E-202 | 6235 |
|  | rs12226389 | 11 | 62056158 | C | T | -0.176 | 0.024 | 0.173 | 2.15E-13 | 6235 |
|  | rs7936002 | 11 | 62451557 | G | A | 0.134 | 0.021 | 0.257 | 1.65E-10 | 6235 |
|  | rs474901 | 11 | 64357915 | T | G | 0.080 | 0.018 | 0.473 | 8.54E-06 | 6235 |
|  | rs600626 | 11 | 75744264 | G | A | -0.121 | 0.021 | 0.232 | 1.23E-08 | 6235 |
|  | rs529446 | 12 | 5112153 | C | T | 0.097 | 0.022 | 0.784 | 7.85E-06 | 6235 |
|  | rs35599386 | 12 | 98288399 | A | G | 0.178 | 0.040 | 0.054 | 7.32E-06 | 6235 |
|  | rs79995028 | 14 | 41287117 | T | G | -0.653 | 0.144 | 0.004 | 5.86E-06 | 6235 |
|  | rs7179330 | 15 | 23837617 | T | A | 0.313 | 0.064 | 0.980 | 1.15E-06 | 6235 |
|  | rs12905466 | 15 | 71480547 | T | C | -0.102 | 0.022 | 0.204 | 5.01E-06 | 6235 |
|  | rs6564238 | 16 | 75219782 | G | C | 0.086 | 0.019 | 0.329 | 7.56E-06 | 6235 |
|  | rs150158785 | 17 | 49470511 | A | G | -0.148 | 0.033 | 0.080 | 7.51E-06 | 6235 |
|  | rs1363011 | 18 | 64269741 | A | G | 0.118 | 0.027 | 0.130 | 1.00E-05 | 6235 |
|  | rs11669519 | 19 | 614967 | C | T | 0.091 | 0.020 | 0.341 | 6.45E-06 | 6235 |
|  | rs182611493 | 19 | 19347579 | G | A | -0.208 | 0.040 | 0.054 | 2.78E-07 | 6235 |
|  | rs12303 | 20 | 64047793 | A | G | -0.104 | 0.023 | 0.196 | 4.56E-06 | 6235 |
|  | rs139516883 | 22 | 23818551 | C | T | -0.353 | 0.071 | 0.016 | 7.61E-07 | 6235 |
| Phosphatidylethanolamine (18:0_0:0) | rs7529794 | 1 | 39937698 | T | G | 0.151 | 0.021 | 0.250 | 3.79E-13 | 6258 |
|  | rs12726330 | 1 | 155135691 | A | G | -0.232 | 0.045 | 0.043 | 2.54E-07 | 6258 |
|  | rs6713691 | 2 | 9689106 | G | A | -0.110 | 0.023 | 0.197 | 1.76E-06 | 6258 |
|  | rs1260326 | 2 | 27508073 | C | T | -0.094 | 0.019 | 0.651 | 5.12E-07 | 6258 |
|  | rs4663191 | 2 | 235266114 | G | A | 0.097 | 0.021 | 0.746 | 2.64E-06 | 6258 |
|  | rs3135062 | 4 | 3285732 | G | C | 0.104 | 0.023 | 0.191 | 5.91E-06 | 6258 |
|  | rs72757442 | 5 | 54771412 | T | C | 0.217 | 0.043 | 0.049 | 5.83E-07 | 6258 |
|  | rs62411861 | 6 | 49191020 | T | C | -0.738 | 0.164 | 0.003 | 6.88E-06 | 6258 |
|  | rs12196051 | 6 | 142892562 | A | G | -0.292 | 0.059 | 0.024 | 8.50E-07 | 6258 |
|  | rs149182005 | 6 | 159557473 | C | T | 0.277 | 0.061 | 0.024 | 5.17E-06 | 6258 |
|  | rs116987919 | 7 | 70998624 | G | C | 0.340 | 0.073 | 0.016 | 2.98E-06 | 6258 |
|  | rs189518615 | 7 | 116377385 | T | A | 0.206 | 0.046 | 0.040 | 9.08E-06 | 6258 |
|  | rs35152003 | 8 | 18433891 | A | T | -0.097 | 0.019 | 0.662 | 4.05E-07 | 6258 |
|  | rs16918167 | 9 | 99053057 | T | G | -0.119 | 0.025 | 0.151 | 1.96E-06 | 6258 |
|  | rs1532085 | 15 | 58391167 | G | A | -0.101 | 0.018 | 0.573 | 1.93E-08 | 6258 |
|  | rs1800588 | 15 | 58431476 | T | C | 0.109 | 0.021 | 0.258 | 1.76E-07 | 6258 |
|  | rs758186 | 17 | 12597341 | T | C | 0.081 | 0.018 | 0.593 | 7.19E-06 | 6258 |
|  | rs79577600 | 17 | 49966298 | T | G | 0.146 | 0.031 | 0.096 | 2.89E-06 | 6258 |
|  | rs62220410 | 21 | 44325499 | T | G | 0.674 | 0.151 | 0.004 | 8.58E-06 | 6258 |
| Phosphatidylethanolamine (18:1_0:0) | rs116537941 | 1 | 230124135 | G | A | -0.198 | 0.044 | 0.054 | 6.84E-06 | 5172 |
|  | rs75388820 | 2 | 27102273 | A | G | 0.228 | 0.047 | 0.047 | 1.19E-06 | 5172 |
|  | rs6434115 | 2 | 152605833 | A | G | 0.093 | 0.021 | 0.613 | 6.92E-06 | 5172 |
|  | rs9826895 | 3 | 23781447 | A | G | -0.103 | 0.022 | 0.272 | 2.95E-06 | 5172 |
|  | rs11917269 | 3 | 50443604 | T | G | 0.109 | 0.022 | 0.277 | 6.75E-07 | 5172 |
|  | rs7636653 | 3 | 73256915 | A | T | -0.100 | 0.020 | 0.476 | 4.13E-07 | 5172 |
|  | rs6768813 | 3 | 142947149 | C | T | 0.110 | 0.020 | 0.629 | 7.74E-08 | 5172 |
|  | rs10015043 | 4 | 168564086 | G | A | 0.089 | 0.020 | 0.463 | 7.91E-06 | 5172 |
|  | rs115554476 | 5 | 4129081 | T | C | -0.355 | 0.080 | 0.015 | 9.06E-06 | 5172 |
|  | rs149492888 | 5 | 99192949 | A | G | -0.525 | 0.113 | 0.009 | 3.40E-06 | 5172 |
|  | rs35584640 | 6 | 73616238 | A | G | -0.108 | 0.024 | 0.218 | 4.89E-06 | 5172 |
|  | rs151000252 | 6 | 85323731 | T | G | 0.873 | 0.195 | 0.002 | 7.49E-06 | 5172 |
|  | rs7776911 | 7 | 50816756 | A | G | -0.137 | 0.027 | 0.841 | 3.22E-07 | 5172 |
|  | rs2296637 | 10 | 127361837 | T | C | -0.145 | 0.032 | 0.109 | 7.14E-06 | 5172 |
|  | rs192233385 | 14 | 46165338 | T | C | -0.914 | 0.206 | 0.003 | 9.20E-06 | 5172 |
|  | rs117398262 | 15 | 38411586 | A | C | 0.266 | 0.059 | 0.029 | 6.72E-06 | 5172 |
|  | rs2585266 | 17 | 5310096 | T | C | -0.241 | 0.049 | 0.044 | 7.67E-07 | 5172 |
|  | rs113318060 | 17 | 83090952 | A | G | -0.443 | 0.100 | 0.011 | 8.86E-06 | 5172 |
| Phosphatidylethanolamine (18:2_0:0) | rs6413913 | 1 | 189909464 | A | G | -0.088 | 0.018 | 0.309 | 1.50E-06 | 6905 |
|  | rs111877698 | 1 | 201480758 | C | A | 0.423 | 0.087 | 0.013 | 1.10E-06 | 6905 |
|  | rs1338449 | 1 | 218177981 | C | T | 0.082 | 0.018 | 0.349 | 4.98E-06 | 6905 |
|  | rs1260326 | 2 | 27508073 | C | T | -0.115 | 0.018 | 0.651 | 1.12E-10 | 6905 |
|  | rs2236950 | 3 | 50383123 | A | C | -0.087 | 0.019 | 0.265 | 5.47E-06 | 6905 |
|  | rs2290053 | 3 | 126114417 | T | C | 0.076 | 0.017 | 0.497 | 7.24E-06 | 6905 |
|  | rs114899411 | 4 | 152031948 | A | G | -0.189 | 0.037 | 0.057 | 5.08E-07 | 6905 |
|  | rs326048 | 5 | 996057 | G | A | -0.090 | 0.020 | 0.285 | 4.60E-06 | 6905 |
|  | rs115359603 | 5 | 21867829 | G | A | -0.302 | 0.066 | 0.017 | 5.26E-06 | 6905 |
|  | rs791631 | 5 | 173674912 | C | G | -0.080 | 0.018 | 0.628 | 5.58E-06 | 6905 |
|  | rs79211442 | 6 | 22879225 | C | T | 0.111 | 0.025 | 0.136 | 9.23E-06 | 6905 |
|  | rs7776911 | 7 | 50816756 | A | G | -0.104 | 0.023 | 0.841 | 9.36E-06 | 6905 |
|  | rs4726320 | 7 | 153641664 | A | T | 0.128 | 0.028 | 0.895 | 5.80E-06 | 6905 |
|  | rs146330404 | 8 | 2533493 | C | G | 0.560 | 0.115 | 0.006 | 1.14E-06 | 6905 |
|  | rs1442407 | 8 | 3234062 | T | G | -0.116 | 0.022 | 0.179 | 1.60E-07 | 6905 |
|  | rs71508993 | 10 | 64197077 | T | C | 0.245 | 0.054 | 0.026 | 5.81E-06 | 6905 |
|  | rs3741259 | 11 | 61514878 | T | C | 0.139 | 0.027 | 0.116 | 1.86E-07 | 6905 |
|  | rs174560 | 11 | 61814292 | C | T | 0.365 | 0.017 | 0.383 | 7.02E-98 | 6905 |
|  | rs1792909 | 11 | 62170073 | A | G | 0.090 | 0.017 | 0.387 | 2.60E-07 | 6905 |
|  | rs11063393 | 12 | 4892157 | G | A | -0.134 | 0.029 | 0.102 | 3.22E-06 | 6905 |
|  | rs11045856 | 12 | 21197755 | G | T | -0.118 | 0.023 | 0.166 | 2.49E-07 | 6905 |
|  | rs188404615 | 12 | 99719293 | C | T | 0.544 | 0.104 | 0.007 | 1.53E-07 | 6905 |
|  | rs8034802 | 15 | 58432593 | A | T | 0.088 | 0.019 | 0.311 | 2.31E-06 | 6905 |
|  | rs62076866 | 17 | 38791988 | T | C | 0.388 | 0.077 | 0.013 | 4.38E-07 | 6905 |
|  | rs35140833 | 18 | 5841984 | G | A | -0.115 | 0.024 | 0.154 | 2.30E-06 | 6905 |
|  | rs12459249 | 19 | 40833991 | C | T | 0.087 | 0.019 | 0.714 | 5.89E-06 | 6905 |
|  | rs62242707 | 21 | 17470749 | A | G | 0.091 | 0.020 | 0.272 | 6.33E-06 | 6905 |
| Phosphatidylcholine (14:0_16:0) | rs72666927 | 4 | 93196475 | T | C | -0.229 | 0.051 | 0.040 | 7.34E-06 | 5497 |
|  | rs6050 | 4 | 154586438 | C | T | 0.095 | 0.020 | 0.323 | 3.24E-06 | 5497 |
|  | rs148426771 | 5 | 21444102 | G | T | -0.212 | 0.047 | 0.049 | 6.49E-06 | 5497 |
|  | rs77191266 | 6 | 86624181 | T | C | 0.242 | 0.053 | 0.037 | 4.43E-06 | 5497 |
|  | rs60984271 | 7 | 102708677 | T | A | 0.103 | 0.023 | 0.220 | 8.34E-06 | 5497 |
|  | rs55654962 | 8 | 27843092 | A | G | 0.134 | 0.030 | 0.109 | 8.32E-06 | 5497 |
|  | rs74939791 | 8 | 82931796 | G | T | 0.270 | 0.057 | 0.030 | 2.03E-06 | 5497 |
|  | rs72764354 | 9 | 127026955 | A | G | 0.210 | 0.046 | 0.045 | 5.86E-06 | 5497 |
|  | rs7075480 | 10 | 98416870 | A | G | 0.527 | 0.117 | 0.007 | 7.18E-06 | 5497 |
|  | rs9507859 | 13 | 27228661 | T | C | 0.111 | 0.022 | 0.765 | 7.13E-07 | 5497 |
|  | rs28416404 | 15 | 57731057 | C | T | -0.166 | 0.035 | 0.079 | 2.84E-06 | 5497 |
|  | rs1553262 | 15 | 97625949 | T | C | 0.517 | 0.115 | 0.008 | 6.57E-06 | 5497 |
|  | rs7230887 | 18 | 11445795 | G | A | -0.106 | 0.024 | 0.207 | 9.29E-06 | 5497 |
|  | rs7239464 | 18 | 33463076 | T | C | 0.099 | 0.021 | 0.289 | 2.43E-06 | 5497 |
|  | rs145618222 | 18 | 68848836 | G | T | -0.318 | 0.072 | 0.019 | 9.34E-06 | 5497 |
|  | rs75414245 | 18 | 80062512 | A | G | 0.352 | 0.078 | 0.015 | 7.28E-06 | 5497 |
|  | rs1230353 | 19 | 20083148 | T | C | -0.114 | 0.025 | 0.185 | 4.92E-06 | 5497 |
|  | rs2014300 | 21 | 34985564 | G | A | 0.141 | 0.030 | 0.888 | 3.42E-06 | 5497 |
|  | rs17749540 | 22 | 36971495 | T | C | -0.121 | 0.027 | 0.152 | 7.39E-06 | 5497 |
| Phosphatidylcholine (14:0_18:1) | rs4846905 | 1 | 230143835 | G | A | 0.088 | 0.020 | 0.768 | 8.68E-06 | 7064 |
|  | rs1260326 | 2 | 27508073 | C | T | -0.105 | 0.018 | 0.651 | 2.39E-09 | 7064 |
|  | rs62189362 | 2 | 227401238 | C | T | 0.124 | 0.027 | 0.108 | 4.45E-06 | 7064 |
|  | rs4564909 | 3 | 138147591 | G | A | -0.081 | 0.018 | 0.653 | 5.94E-06 | 7064 |
|  | rs2686446 | 3 | 195507255 | C | G | 0.090 | 0.019 | 0.711 | 1.41E-06 | 7064 |
|  | rs6531968 | 4 | 87278332 | G | T | -0.079 | 0.017 | 0.544 | 3.79E-06 | 7064 |
|  | rs79066364 | 5 | 141674766 | T | C | -0.241 | 0.054 | 0.025 | 8.80E-06 | 7064 |
|  | rs2946493 | 8 | 12966162 | C | T | 0.099 | 0.021 | 0.206 | 2.91E-06 | 7064 |
|  | rs113668788 | 9 | 137711224 | A | G | 0.135 | 0.028 | 0.108 | 1.81E-06 | 7064 |
|  | rs7484827 | 12 | 47729780 | G | A | 0.090 | 0.020 | 0.241 | 4.21E-06 | 7064 |
|  | rs78414457 | 13 | 37381120 | T | G | 0.155 | 0.033 | 0.074 | 2.43E-06 | 7064 |
|  | rs11857386 | 15 | 58428969 | C | G | -0.081 | 0.017 | 0.471 | 3.12E-06 | 7064 |
|  | rs12446515 | 16 | 56953103 | T | C | 0.097 | 0.019 | 0.279 | 2.30E-07 | 7064 |
|  | rs3816689 | 17 | 4480495 | T | C | -0.121 | 0.027 | 0.111 | 7.13E-06 | 7064 |
|  | rs146934967 | 18 | 15033501 | T | C | 0.372 | 0.074 | 0.013 | 5.92E-07 | 7064 |
|  | rs12960229 | 18 | 21542677 | T | G | -0.121 | 0.020 | 0.781 | 1.87E-09 | 7064 |
|  | rs182611493 | 19 | 19347579 | G | A | -0.169 | 0.038 | 0.054 | 9.04E-06 | 7064 |
|  | rs2379129 | 20 | 62310086 | A | C | -0.090 | 0.017 | 0.399 | 1.65E-07 | 7064 |
|  | rs144849989 | 22 | 19662612 | T | C | -0.532 | 0.120 | 0.006 | 9.33E-06 | 7064 |
|  | rs2070474 | 22 | 24495324 | G | C | 0.085 | 0.017 | 0.549 | 5.10E-07 | 7064 |
| Phosphatidylcholine (14:0_18:2) | rs116105185 | 1 | 63400788 | C | T | 0.222 | 0.050 | 0.030 | 9.97E-06 | 6866 |
|  | rs12137781 | 1 | 79358390 | A | G | -0.170 | 0.037 | 0.059 | 3.83E-06 | 6866 |
|  | rs1262295 | 1 | 201408310 | G | A | 0.117 | 0.025 | 0.864 | 3.86E-06 | 6866 |
|  | rs4665972 | 2 | 27375230 | C | T | -0.115 | 0.018 | 0.620 | 6.24E-11 | 6866 |
|  | rs4952487 | 2 | 41532438 | T | A | -0.079 | 0.018 | 0.366 | 8.30E-06 | 6866 |
|  | rs13418209 | 2 | 163870869 | T | G | 0.080 | 0.018 | 0.364 | 7.65E-06 | 6866 |
|  | rs78880935 | 3 | 68279920 | C | T | 0.277 | 0.063 | 0.020 | 9.75E-06 | 6866 |
|  | rs4683767 | 3 | 139875064 | C | G | -0.097 | 0.022 | 0.194 | 6.52E-06 | 6866 |
|  | rs74920877 | 6 | 8749035 | A | G | 0.220 | 0.049 | 0.031 | 8.64E-06 | 6866 |
|  | rs12113381 | 7 | 1103491 | T | C | 0.123 | 0.026 | 0.120 | 3.24E-06 | 6866 |
|  | rs500246 | 8 | 37124499 | G | A | -0.131 | 0.028 | 0.895 | 2.98E-06 | 6866 |
|  | rs9663572 | 10 | 41829153 | G | C | -0.125 | 0.028 | 0.110 | 8.13E-06 | 6866 |
|  | rs174548 | 11 | 61803876 | G | C | 0.184 | 0.018 | 0.384 | 1.15E-25 | 6866 |
|  | rs144154975 | 13 | 65750016 | A | C | 0.248 | 0.055 | 0.025 | 6.56E-06 | 6866 |
|  | rs8015793 | 14 | 95471324 | C | G | 0.088 | 0.020 | 0.274 | 8.53E-06 | 6866 |
|  | rs950668 | 15 | 71629500 | A | G | -0.081 | 0.018 | 0.483 | 4.50E-06 | 6866 |
|  | rs11853492 | 15 | 97139522 | T | C | -0.083 | 0.019 | 0.660 | 7.13E-06 | 6866 |
|  | rs55771840 | 17 | 79455291 | T | C | 0.110 | 0.025 | 0.140 | 8.48E-06 | 6866 |
|  | rs17202368 | 18 | 21040292 | A | C | 0.331 | 0.051 | 0.029 | 1.29E-10 | 6866 |
|  | rs150543432 | 18 | 21887951 | T | A | 0.354 | 0.076 | 0.013 | 3.05E-06 | 6866 |
|  | rs2044350 | 18 | 52193653 | G | C | 0.095 | 0.021 | 0.788 | 7.87E-06 | 6866 |
|  | rs11668274 | 19 | 47816733 | T | C | -0.398 | 0.089 | 0.010 | 7.89E-06 | 6866 |
|  | rs6045639 | 20 | 1953902 | G | A | 0.092 | 0.018 | 0.324 | 5.20E-07 | 6866 |
|  | rs1800961 | 20 | 44413724 | T | C | -0.171 | 0.038 | 0.052 | 8.08E-06 | 6866 |
|  | rs6089695 | 20 | 62232970 | T | C | -0.083 | 0.017 | 0.419 | 1.79E-06 | 6866 |
|  | rs7281206 | 21 | 37359926 | C | T | 0.088 | 0.017 | 0.506 | 1.93E-07 | 6866 |
| Phosphatidylcholine (15:0_18:1) | rs10917404 | 1 | 23639339 | T | C | -0.098 | 0.021 | 0.227 | 3.32E-06 | 6468 |
|  | rs1260326 | 2 | 27508073 | C | T | -0.097 | 0.018 | 0.651 | 1.22E-07 | 6468 |
|  | rs41441450 | 5 | 86717236 | T | C | -0.408 | 0.089 | 0.010 | 5.08E-06 | 6468 |
|  | rs13161129 | 5 | 133494602 | A | G | -0.126 | 0.028 | 0.114 | 6.63E-06 | 6468 |
|  | rs6866162 | 5 | 174328650 | C | T | 0.134 | 0.028 | 0.127 | 1.34E-06 | 6468 |
|  | rs142477396 | 6 | 21056212 | A | G | -0.107 | 0.023 | 0.175 | 3.77E-06 | 6468 |
|  | rs1044043 | 6 | 32826204 | C | A | -0.098 | 0.022 | 0.808 | 8.88E-06 | 6468 |
|  | rs581080 | 9 | 15305380 | C | G | 0.117 | 0.025 | 0.856 | 2.75E-06 | 6468 |
|  | rs77392374 | 10 | 71286664 | C | T | -0.252 | 0.057 | 0.027 | 9.28E-06 | 6468 |
|  | rs4238010 | 12 | 4009151 | A | G | -0.115 | 0.026 | 0.866 | 9.44E-06 | 6468 |
|  | rs117207178 | 12 | 34480655 | T | C | 0.134 | 0.030 | 0.097 | 8.39E-06 | 6468 |
|  | rs12916925 | 15 | 38647674 | A | T | 0.081 | 0.018 | 0.461 | 4.41E-06 | 6468 |
|  | rs1532085 | 15 | 58391167 | G | A | -0.089 | 0.018 | 0.573 | 4.67E-07 | 6468 |
|  | rs75922719 | 15 | 93906166 | C | T | 0.476 | 0.107 | 0.007 | 8.01E-06 | 6468 |
|  | rs11075022 | 16 | 11827406 | C | T | 0.086 | 0.018 | 0.570 | 1.60E-06 | 6468 |
|  | rs138445434 | 16 | 19425616 | A | G | -0.247 | 0.055 | 0.026 | 7.74E-06 | 6468 |
|  | rs72815248 | 16 | 54459960 | T | C | -0.130 | 0.027 | 0.122 | 1.66E-06 | 6468 |
|  | rs12926159 | 16 | 88735092 | A | G | -0.431 | 0.080 | 0.013 | 6.10E-08 | 6468 |
|  | rs2379129 | 20 | 62310086 | A | C | -0.095 | 0.018 | 0.399 | 1.58E-07 | 6468 |
|  | rs73897494 | 21 | 23717899 | A | G | -0.337 | 0.075 | 0.016 | 7.88E-06 | 6468 |
| Phosphatidylcholine (15:0_18:2) | rs1168036 | 1 | 62497063 | G | A | 0.107 | 0.020 | 0.738 | 4.78E-08 | 6639 |
|  | rs1911704 | 2 | 41471833 | C | G | -0.084 | 0.018 | 0.349 | 4.65E-06 | 6639 |
|  | rs115852609 | 2 | 156288948 | A | G | 0.255 | 0.057 | 0.023 | 8.90E-06 | 6639 |
|  | rs114498622 | 2 | 213698079 | C | A | 0.347 | 0.075 | 0.016 | 4.32E-06 | 6639 |
|  | rs13063467 | 3 | 11166089 | A | C | 0.212 | 0.048 | 0.035 | 9.06E-06 | 6639 |
|  | rs6769712 | 3 | 24997572 | A | T | -0.084 | 0.017 | 0.479 | 1.07E-06 | 6639 |
|  | rs1488201 | 3 | 26848389 | G | T | 0.085 | 0.018 | 0.350 | 3.60E-06 | 6639 |
|  | rs11722429 | 4 | 136284098 | A | G | 0.132 | 0.029 | 0.099 | 5.94E-06 | 6639 |
|  | rs13101957 | 4 | 169278135 | A | C | -0.122 | 0.026 | 0.130 | 2.89E-06 | 6639 |
|  | rs17614174 | 5 | 16862310 | C | T | -0.187 | 0.040 | 0.052 | 3.75E-06 | 6639 |
|  | rs56144603 | 6 | 73220880 | A | G | 0.247 | 0.051 | 0.032 | 1.42E-06 | 6639 |
|  | rs139075653 | 6 | 114209369 | C | T | 0.646 | 0.138 | 0.004 | 3.04E-06 | 6639 |
|  | rs184289628 | 6 | 139585552 | G | T | 0.299 | 0.065 | 0.018 | 3.67E-06 | 6639 |
|  | rs12113214 | 7 | 6530143 | A | T | 0.093 | 0.019 | 0.323 | 7.34E-07 | 6639 |
|  | rs6558177 | 8 | 22701002 | G | A | -0.080 | 0.018 | 0.622 | 8.36E-06 | 6639 |
|  | rs142336498 | 8 | 51606091 | T | G | 0.408 | 0.090 | 0.009 | 6.36E-06 | 6639 |
|  | rs77404354 | 8 | 64641039 | T | C | -0.478 | 0.100 | 0.008 | 1.84E-06 | 6639 |
|  | rs13281002 | 8 | 65661212 | A | G | -0.262 | 0.053 | 0.027 | 9.59E-07 | 6639 |
|  | rs551412 | 9 | 132450788 | T | A | 0.088 | 0.019 | 0.286 | 4.64E-06 | 6639 |
|  | rs4335452 | 10 | 5872836 | A | G | -0.103 | 0.022 | 0.800 | 3.88E-06 | 6639 |
|  | rs35960238 | 10 | 26331627 | G | A | -0.131 | 0.026 | 0.127 | 5.37E-07 | 6639 |
|  | rs76916318 | 10 | 44350663 | C | T | 0.293 | 0.065 | 0.018 | 5.76E-06 | 6639 |
|  | rs7920171 | 10 | 63094343 | A | G | 0.095 | 0.021 | 0.208 | 8.96E-06 | 6639 |
|  | rs174527 | 11 | 61770929 | C | G | 0.102 | 0.018 | 0.444 | 9.33E-09 | 6639 |
|  | rs174566 | 11 | 61824890 | G | A | 0.317 | 0.017 | 0.408 | 1.46E-73 | 6639 |
|  | rs4382917 | 11 | 62454004 | A | G | -0.101 | 0.021 | 0.256 | 9.84E-07 | 6639 |
|  | rs639956 | 11 | 79640107 | G | A | -0.078 | 0.017 | 0.561 | 8.06E-06 | 6639 |
|  | rs964184 | 11 | 116778201 | C | G | -0.141 | 0.024 | 0.849 | 5.34E-09 | 6639 |
|  | rs11174583 | 12 | 62657284 | C | T | -0.121 | 0.027 | 0.113 | 9.96E-06 | 6639 |
|  | rs75097472 | 12 | 130443132 | A | G | -0.383 | 0.084 | 0.011 | 4.65E-06 | 6639 |
|  | rs16968712 | 13 | 106475436 | C | T | -0.132 | 0.028 | 0.119 | 2.63E-06 | 6639 |
|  | rs10468017 | 15 | 58386313 | T | C | 0.112 | 0.018 | 0.338 | 7.41E-10 | 6639 |
|  | rs633695 | 15 | 58433640 | G | A | 0.093 | 0.019 | 0.314 | 9.28E-07 | 6639 |
|  | rs325406 | 15 | 99707535 | T | C | 0.098 | 0.021 | 0.791 | 5.19E-06 | 6639 |
|  | rs60071820 | 16 | 14348932 | T | C | -0.079 | 0.018 | 0.426 | 8.51E-06 | 6639 |
|  | rs55727637 | 16 | 15051395 | T | C | 0.111 | 0.019 | 0.299 | 5.22E-09 | 6639 |
|  | rs17231506 | 16 | 56960616 | T | C | 0.143 | 0.019 | 0.278 | 1.20E-13 | 6639 |
|  | rs12938196 | 17 | 2780136 | T | C | 0.107 | 0.024 | 0.159 | 9.69E-06 | 6639 |
|  | rs146108252 | 20 | 7847799 | T | C | 0.568 | 0.128 | 0.005 | 9.11E-06 | 6639 |
|  | rs483528 | 22 | 33757979 | T | C | 0.171 | 0.038 | 0.946 | 9.00E-06 | 6639 |
| Phosphatidylcholine (16:0_16:0) | rs56270325 | 1 | 5899241 | T | A | 0.091 | 0.019 | 0.259 | 1.49E-06 | 7174 |
|  | rs11591147 | 1 | 55039974 | T | G | -0.234 | 0.047 | 0.033 | 4.94E-07 | 7174 |
|  | rs148444916 | 1 | 92970296 | C | T | 0.289 | 0.058 | 0.021 | 5.67E-07 | 7174 |
|  | rs75023439 | 1 | 158505761 | C | T | -0.173 | 0.039 | 0.048 | 8.49E-06 | 7174 |
|  | rs1367117 | 2 | 21041028 | A | G | 0.084 | 0.018 | 0.285 | 5.33E-06 | 7174 |
|  | rs13060992 | 3 | 186944804 | G | T | 0.186 | 0.038 | 0.948 | 7.57E-07 | 7174 |
|  | rs5022521 | 4 | 110207431 | T | C | -0.097 | 0.021 | 0.797 | 4.26E-06 | 7174 |
|  | rs3936021 | 5 | 85099087 | G | A | -0.107 | 0.023 | 0.158 | 4.96E-06 | 7174 |
|  | rs12522374 | 5 | 159237696 | A | G | 0.081 | 0.017 | 0.347 | 3.11E-06 | 7174 |
|  | rs3134950 | 6 | 32159700 | A | C | 0.097 | 0.017 | 0.583 | 9.96E-09 | 7174 |
|  | rs72974647 | 6 | 123327335 | G | A | 0.144 | 0.030 | 0.082 | 1.61E-06 | 7174 |
|  | rs7780296 | 7 | 17674637 | G | C | 0.084 | 0.019 | 0.719 | 5.57E-06 | 7174 |
|  | rs3807226 | 7 | 154679558 | G | A | -0.082 | 0.019 | 0.276 | 9.57E-06 | 7174 |
|  | rs643531 | 9 | 15296036 | A | C | 0.125 | 0.026 | 0.890 | 1.91E-06 | 7174 |
|  | rs4744927 | 9 | 69669802 | C | A | 0.907 | 0.192 | 0.998 | 2.35E-06 | 7174 |
|  | rs72733996 | 9 | 80936784 | G | A | 0.101 | 0.021 | 0.198 | 1.23E-06 | 7174 |
|  | rs2236514 | 9 | 136677616 | G | C | -0.084 | 0.018 | 0.647 | 2.32E-06 | 7174 |
|  | rs174546 | 11 | 61802358 | T | C | -0.149 | 0.017 | 0.407 | 1.69E-18 | 7174 |
|  | rs1187583 | 14 | 54570145 | A | G | -0.142 | 0.030 | 0.916 | 2.41E-06 | 7174 |
|  | rs261290 | 15 | 58386521 | C | T | -0.139 | 0.017 | 0.612 | 2.37E-16 | 7174 |
|  | rs633695 | 15 | 58433640 | G | A | 0.091 | 0.018 | 0.314 | 5.23E-07 | 7174 |
|  | rs17231506 | 16 | 56960616 | T | C | 0.142 | 0.018 | 0.278 | 1.54E-14 | 7174 |
|  | rs9966610 | 18 | 72301960 | C | A | 0.079 | 0.017 | 0.511 | 5.38E-06 | 7174 |
|  | rs73015021 | 19 | 11082239 | G | A | -0.158 | 0.029 | 0.092 | 3.49E-08 | 7174 |
|  | rs78909142 | 22 | 40993330 | A | G | -0.433 | 0.094 | 0.009 | 4.56E-06 | 7174 |
| Phosphatidylcholine (16:0_16:1) | rs1260326 | 2 | 27508073 | C | T | -0.108 | 0.017 | 0.651 | 6.12E-10 | 7172 |
|  | rs80227620 | 2 | 167832629 | T | C | 0.119 | 0.024 | 0.144 | 1.19E-06 | 7172 |
|  | rs28524821 | 4 | 17068686 | C | A | 0.109 | 0.024 | 0.146 | 3.98E-06 | 7172 |
|  | rs2597486 | 4 | 162453799 | G | A | 0.076 | 0.017 | 0.554 | 8.12E-06 | 7172 |
|  | rs116681793 | 4 | 180227646 | A | G | -0.185 | 0.041 | 0.045 | 7.01E-06 | 7172 |
|  | rs4629629 | 5 | 86296114 | T | C | 0.088 | 0.020 | 0.765 | 7.25E-06 | 7172 |
|  | rs113366516 | 6 | 138148100 | T | G | 0.308 | 0.066 | 0.017 | 3.14E-06 | 7172 |
|  | rs117726731 | 8 | 303655 | T | C | 0.186 | 0.042 | 0.042 | 9.99E-06 | 7172 |
|  | rs1854434 | 9 | 32796762 | G | A | -0.110 | 0.023 | 0.154 | 2.15E-06 | 7172 |
|  | rs10760286 | 9 | 123419309 | C | T | 0.079 | 0.017 | 0.510 | 3.32E-06 | 7172 |
|  | rs35235479 | 10 | 92767403 | G | A | -0.096 | 0.020 | 0.214 | 2.24E-06 | 7172 |
|  | rs62015468 | 16 | 6538604 | C | G | 0.194 | 0.043 | 0.042 | 6.47E-06 | 7172 |
|  | rs11647818 | 16 | 11833509 | T | C | 0.079 | 0.017 | 0.575 | 3.68E-06 | 7172 |
|  | rs77007318 | 16 | 59856268 | C | G | -0.241 | 0.052 | 0.029 | 3.88E-06 | 7172 |
|  | rs34832413 | 16 | 71310208 | G | A | -0.119 | 0.026 | 0.112 | 6.08E-06 | 7172 |
|  | rs117571757 | 17 | 16458446 | A | G | -0.334 | 0.073 | 0.013 | 4.44E-06 | 7172 |
|  | rs62098276 | 18 | 63512537 | C | T | -0.142 | 0.031 | 0.080 | 3.80E-06 | 7172 |
|  | rs12960635 | 18 | 78257046 | C | T | 0.124 | 0.028 | 0.105 | 7.73E-06 | 7172 |
| Phosphatidylcholine (16:0_17:1) | rs138107736 | 1 | 61714683 | G | T | -0.343 | 0.077 | 0.013 | 8.58E-06 | 7076 |
|  | rs12749808 | 1 | 158095972 | T | C | -0.084 | 0.018 | 0.356 | 2.75E-06 | 7076 |
|  | rs12475699 | 2 | 30022181 | C | T | 0.081 | 0.018 | 0.663 | 4.86E-06 | 7076 |
|  | rs78395462 | 4 | 122641012 | T | G | 0.258 | 0.055 | 0.024 | 3.17E-06 | 7076 |
|  | rs162448 | 5 | 7954664 | G | C | 0.077 | 0.017 | 0.505 | 6.82E-06 | 7076 |
|  | rs818276 | 6 | 78337820 | T | C | 0.074 | 0.017 | 0.476 | 8.73E-06 | 7076 |
|  | rs80201535 | 7 | 10826150 | A | G | -0.220 | 0.049 | 0.031 | 7.45E-06 | 7076 |
|  | rs2122922 | 8 | 101050430 | T | C | -0.098 | 0.021 | 0.202 | 4.61E-06 | 7076 |
|  | rs116911263 | 10 | 95941250 | T | C | -0.352 | 0.073 | 0.014 | 1.47E-06 | 7076 |
|  | rs117085626 | 11 | 1607718 | A | G | 0.120 | 0.027 | 0.113 | 6.77E-06 | 7076 |
|  | rs10500613 | 11 | 4687754 | G | A | -0.247 | 0.055 | 0.024 | 7.81E-06 | 7076 |
|  | rs2317091 | 11 | 63193288 | A | T | 0.191 | 0.043 | 0.040 | 9.83E-06 | 7076 |
|  | rs10783517 | 12 | 52522822 | T | C | -0.092 | 0.021 | 0.213 | 7.89E-06 | 7076 |
|  | rs78418403 | 15 | 23752030 | T | G | 0.157 | 0.035 | 0.062 | 6.23E-06 | 7076 |
|  | rs7177289 | 15 | 58387985 | T | C | 0.082 | 0.017 | 0.393 | 1.53E-06 | 7076 |
|  | rs6496787 | 15 | 91344956 | G | T | 0.093 | 0.017 | 0.608 | 8.27E-08 | 7076 |
|  | rs12602611 | 17 | 11337105 | A | G | 0.099 | 0.022 | 0.173 | 8.10E-06 | 7076 |
|  | rs41488845 | 17 | 38380397 | A | G | -0.496 | 0.104 | 0.007 | 2.04E-06 | 7076 |
|  | rs7248028 | 19 | 55189474 | C | T | 0.091 | 0.020 | 0.245 | 5.47E-06 | 7076 |
|  | rs16986523 | 20 | 24390991 | A | G | 0.268 | 0.058 | 0.022 | 3.78E-06 | 7076 |
|  | rs2075937 | 22 | 36934040 | C | T | -0.075 | 0.017 | 0.586 | 9.73E-06 | 7076 |
|  | rs13054328 | 22 | 44080894 | T | C | 0.185 | 0.041 | 0.049 | 6.81E-06 | 7076 |
| Phosphatidylcholine (16:0_18:0) | rs1168036 | 1 | 62497063 | G | A | 0.090 | 0.019 | 0.738 | 1.99E-06 | 7154 |
|  | rs2391389 | 1 | 95020123 | G | A | -0.077 | 0.017 | 0.379 | 5.98E-06 | 7154 |
|  | rs4659619 | 1 | 236035687 | C | T | 0.085 | 0.017 | 0.338 | 1.21E-06 | 7154 |
|  | rs11903018 | 2 | 37366100 | C | T | -0.098 | 0.022 | 0.181 | 7.88E-06 | 7154 |
|  | rs12052409 | 2 | 68074558 | C | A | -0.193 | 0.039 | 0.052 | 5.57E-07 | 7154 |
|  | rs72723967 | 4 | 137812361 | A | C | 0.111 | 0.023 | 0.163 | 1.18E-06 | 7154 |
|  | rs11723712 | 4 | 169304745 | A | G | 0.103 | 0.021 | 0.806 | 1.11E-06 | 7154 |
|  | rs13119269 | 4 | 188521987 | T | C | 0.100 | 0.023 | 0.167 | 9.47E-06 | 7154 |
|  | rs62330208 | 5 | 983129 | C | G | -0.103 | 0.020 | 0.240 | 4.69E-07 | 7154 |
|  | rs113053792 | 5 | 63116092 | G | A | -0.356 | 0.079 | 0.012 | 5.97E-06 | 7154 |
|  | rs3924 | 5 | 151030574 | A | G | -0.076 | 0.017 | 0.604 | 9.52E-06 | 7154 |
|  | rs139644986 | 6 | 18721099 | G | C | 0.549 | 0.124 | 0.005 | 9.93E-06 | 7154 |
|  | rs2854275 | 6 | 32660651 | A | C | -0.123 | 0.027 | 0.105 | 6.23E-06 | 7154 |
|  | rs72974647 | 6 | 123327335 | G | A | 0.143 | 0.030 | 0.082 | 1.91E-06 | 7154 |
|  | rs117495065 | 6 | 147174911 | G | C | 0.302 | 0.065 | 0.017 | 4.02E-06 | 7154 |
|  | rs141165989 | 6 | 150183580 | T | C | 0.203 | 0.045 | 0.037 | 5.59E-06 | 7154 |
|  | rs76779498 | 7 | 17925463 | T | C | 0.326 | 0.073 | 0.013 | 8.13E-06 | 7154 |
|  | rs2214660 | 7 | 122869647 | A | G | -0.102 | 0.022 | 0.185 | 2.56E-06 | 7154 |
|  | rs113076792 | 7 | 153170994 | A | G | -0.338 | 0.074 | 0.014 | 4.84E-06 | 7154 |
|  | rs2126263 | 8 | 9324101 | A | G | 0.111 | 0.023 | 0.850 | 2.30E-06 | 7154 |
|  | rs10738267 | 9 | 12222686 | G | C | 0.104 | 0.023 | 0.837 | 5.61E-06 | 7154 |
|  | rs7046115 | 9 | 94130323 | G | C | -0.080 | 0.018 | 0.321 | 9.93E-06 | 7154 |
|  | rs2274855 | 9 | 132230557 | A | G | -0.103 | 0.023 | 0.171 | 9.51E-06 | 7154 |
|  | rs2724782 | 10 | 12379924 | A | G | -0.215 | 0.048 | 0.034 | 8.99E-06 | 7154 |
|  | rs118179558 | 11 | 58479658 | C | T | -0.704 | 0.158 | 0.003 | 8.26E-06 | 7154 |
|  | rs174601 | 11 | 61855668 | T | C | 0.146 | 0.017 | 0.419 | 6.00E-18 | 7154 |
|  | rs10890773 | 11 | 107889276 | A | G | -0.117 | 0.024 | 0.860 | 1.02E-06 | 7154 |
|  | rs143975147 | 12 | 4779414 | T | C | 0.220 | 0.049 | 0.030 | 8.09E-06 | 7154 |
|  | rs10468017 | 15 | 58386313 | T | C | 0.088 | 0.018 | 0.338 | 6.14E-07 | 7154 |
|  | rs633695 | 15 | 58433640 | G | A | 0.081 | 0.018 | 0.314 | 8.31E-06 | 7154 |
|  | rs76413658 | 16 | 6589736 | C | G | -0.112 | 0.024 | 0.133 | 4.73E-06 | 7154 |
|  | rs247617 | 16 | 56956804 | A | C | 0.092 | 0.019 | 0.278 | 7.77E-07 | 7154 |
|  | rs11870991 | 17 | 44758756 | A | C | 0.129 | 0.029 | 0.092 | 8.31E-06 | 7154 |
|  | rs62080593 | 18 | 14429629 | G | A | 0.093 | 0.021 | 0.249 | 8.02E-06 | 7154 |
|  | rs117528775 | 18 | 49649558 | C | T | 0.594 | 0.125 | 0.005 | 2.25E-06 | 7154 |
|  | rs10853781 | 19 | 47242784 | G | A | 0.079 | 0.017 | 0.496 | 3.32E-06 | 7154 |
|  | rs662846 | 20 | 819559 | A | C | -0.272 | 0.057 | 0.023 | 1.66E-06 | 7154 |
|  | rs2833577 | 21 | 31954469 | A | G | -0.080 | 0.018 | 0.372 | 8.64E-06 | 7154 |
|  | rs6005648 | 22 | 27828806 | G | A | -0.105 | 0.024 | 0.150 | 8.65E-06 | 7154 |
| Phosphatidylcholine (16:0_18:1) | rs10779836 | 1 | 230167404 | T | C | 0.090 | 0.020 | 0.777 | 6.13E-06 | 7174 |
|  | rs1260326 | 2 | 27508073 | C | T | -0.101 | 0.017 | 0.651 | 6.89E-09 | 7174 |
|  | rs13060992 | 3 | 186944804 | G | T | 0.173 | 0.038 | 0.948 | 4.50E-06 | 7174 |
|  | rs12650992 | 4 | 123415224 | T | C | 0.089 | 0.020 | 0.234 | 5.70E-06 | 7174 |
|  | rs114925849 | 5 | 109218889 | A | C | 0.577 | 0.127 | 0.005 | 5.74E-06 | 7174 |
|  | rs28807772 | 5 | 165822116 | G | A | 0.095 | 0.021 | 0.202 | 4.72E-06 | 7174 |
|  | rs76546858 | 5 | 181070075 | T | C | -0.121 | 0.027 | 0.131 | 7.80E-06 | 7174 |
|  | rs3134937 | 6 | 32238762 | T | C | -0.101 | 0.021 | 0.200 | 1.17E-06 | 7174 |
|  | rs117726731 | 8 | 303655 | T | C | 0.186 | 0.042 | 0.042 | 9.94E-06 | 7174 |
|  | rs4538911 | 8 | 6228839 | C | G | -0.091 | 0.018 | 0.703 | 8.54E-07 | 7174 |
|  | rs7004301 | 8 | 129226534 | G | A | 0.077 | 0.017 | 0.619 | 7.95E-06 | 7174 |
|  | rs7912885 | 10 | 127538724 | T | G | -0.082 | 0.018 | 0.699 | 9.28E-06 | 7174 |
|  | rs116997061 | 12 | 19869115 | T | C | -0.170 | 0.036 | 0.062 | 2.35E-06 | 7174 |
|  | rs1541361 | 12 | 47105688 | T | C | -0.077 | 0.017 | 0.430 | 5.63E-06 | 7174 |
|  | rs1532085 | 15 | 58391167 | G | A | -0.098 | 0.017 | 0.573 | 6.25E-09 | 7174 |
|  | rs633695 | 15 | 58433640 | G | A | 0.090 | 0.018 | 0.314 | 6.82E-07 | 7174 |
|  | rs247617 | 16 | 56956804 | A | C | 0.098 | 0.019 | 0.278 | 1.56E-07 | 7174 |
|  | rs1593135 | 16 | 71259459 | G | C | -0.119 | 0.026 | 0.112 | 6.52E-06 | 7174 |
|  | rs77027238 | 17 | 43849174 | A | G | 0.185 | 0.038 | 0.050 | 1.54E-06 | 7174 |
| Phosphatidylcholine (16:0_18:2) | rs187681244 | 1 | 16249217 | T | C | -0.439 | 0.098 | 0.008 | 7.01E-06 | 7174 |
|  | rs75833961 | 1 | 33445467 | C | T | 0.272 | 0.061 | 0.020 | 8.43E-06 | 7174 |
|  | rs9436221 | 1 | 62437688 | C | T | -0.127 | 0.019 | 0.261 | 1.82E-11 | 7174 |
|  | rs6673582 | 1 | 189911359 | A | T | -0.080 | 0.018 | 0.309 | 7.04E-06 | 7174 |
|  | rs13402814 | 2 | 137186441 | T | A | 0.164 | 0.035 | 0.060 | 2.89E-06 | 7174 |
|  | rs2629180 | 3 | 3947204 | A | G | -0.695 | 0.148 | 0.004 | 2.56E-06 | 7174 |
|  | rs150352222 | 3 | 10308480 | T | A | -0.303 | 0.068 | 0.015 | 8.89E-06 | 7174 |
|  | rs1488201 | 3 | 26848389 | G | T | 0.078 | 0.018 | 0.350 | 8.86E-06 | 7174 |
|  | rs11722429 | 4 | 136284098 | A | G | 0.133 | 0.028 | 0.099 | 2.18E-06 | 7174 |
|  | rs13150924 | 4 | 169277659 | A | G | -0.135 | 0.025 | 0.130 | 7.20E-08 | 7174 |
|  | rs62330208 | 5 | 983129 | C | G | -0.090 | 0.020 | 0.240 | 9.97E-06 | 7174 |
|  | rs7717591 | 5 | 52415905 | G | A | -0.081 | 0.018 | 0.679 | 6.26E-06 | 7174 |
|  | rs78882831 | 5 | 96128576 | T | C | 0.247 | 0.055 | 0.025 | 6.35E-06 | 7174 |
|  | rs4415100 | 5 | 165816602 | A | T | 0.093 | 0.020 | 0.211 | 5.24E-06 | 7174 |
|  | rs112569946 | 6 | 124913409 | A | G | 0.716 | 0.156 | 0.003 | 4.34E-06 | 7174 |
|  | rs10961143 | 9 | 13564220 | G | A | 0.195 | 0.039 | 0.048 | 4.88E-07 | 7174 |
|  | rs4412689 | 10 | 57406164 | A | G | -0.093 | 0.021 | 0.200 | 7.42E-06 | 7174 |
|  | rs10787711 | 10 | 116647273 | A | G | -0.090 | 0.017 | 0.648 | 2.74E-07 | 7174 |
|  | rs6578729 | 11 | 6068510 | A | G | -0.139 | 0.031 | 0.079 | 7.32E-06 | 7174 |
|  | rs174527 | 11 | 61770929 | C | G | 0.107 | 0.017 | 0.444 | 3.51E-10 | 7174 |
|  | rs174574 | 11 | 61832870 | C | A | -0.331 | 0.017 | 0.591 | 5.66E-86 | 7174 |
|  | rs7936002 | 11 | 62451557 | G | A | -0.099 | 0.020 | 0.257 | 4.27E-07 | 7174 |
|  | rs56075928 | 11 | 63135902 | A | G | 0.245 | 0.053 | 0.026 | 3.85E-06 | 7174 |
|  | rs10890773 | 11 | 107889276 | A | G | -0.126 | 0.024 | 0.860 | 1.42E-07 | 7174 |
|  | rs964184 | 11 | 116778201 | C | G | -0.158 | 0.023 | 0.849 | 9.56E-12 | 7174 |
|  | rs150376922 | 12 | 29702911 | A | G | -0.290 | 0.064 | 0.018 | 6.88E-06 | 7174 |
|  | rs17126640 | 12 | 52311207 | A | G | -0.099 | 0.021 | 0.203 | 1.89E-06 | 7174 |
|  | rs143066473 | 12 | 99432175 | A | G | 0.254 | 0.055 | 0.025 | 4.29E-06 | 7174 |
|  | rs73205054 | 12 | 110067189 | C | A | 0.196 | 0.043 | 0.038 | 6.17E-06 | 7174 |
|  | rs1532085 | 15 | 58391167 | G | A | -0.111 | 0.017 | 0.573 | 4.61E-11 | 7174 |
|  | rs1800588 | 15 | 58431476 | T | C | 0.138 | 0.019 | 0.258 | 1.09E-12 | 7174 |
|  | rs1833172 | 16 | 6626446 | T | C | 0.157 | 0.033 | 0.072 | 1.60E-06 | 7174 |
|  | rs62039480 | 16 | 15043593 | A | G | 0.116 | 0.018 | 0.332 | 6.52E-11 | 7174 |
|  | rs17231506 | 16 | 56960616 | T | C | 0.144 | 0.018 | 0.278 | 8.35E-15 | 7174 |
|  | rs117528775 | 18 | 49649558 | C | T | 0.597 | 0.125 | 0.005 | 1.92E-06 | 7174 |
|  | rs35925446 | 19 | 3325829 | C | T | -0.159 | 0.035 | 0.067 | 5.28E-06 | 7174 |
|  | rs16985573 | 22 | 27818698 | T | C | -0.150 | 0.033 | 0.073 | 5.43E-06 | 7174 |
| Phosphatidylcholine (16:0_18:3) | rs114797690 | 1 | 98597981 | T | C | 0.263 | 0.059 | 0.023 | 7.71E-06 | 7174 |
|  | rs1260326 | 2 | 27508073 | C | T | -0.115 | 0.017 | 0.651 | 4.02E-11 | 7174 |
|  | rs147507697 | 3 | 76641189 | G | A | 0.209 | 0.045 | 0.036 | 3.46E-06 | 7174 |
|  | rs1474236 | 3 | 117112654 | C | T | -0.112 | 0.023 | 0.818 | 8.21E-07 | 7174 |
|  | rs79189815 | 5 | 62191215 | G | A | 0.431 | 0.091 | 0.009 | 2.19E-06 | 7174 |
|  | rs115079643 | 5 | 176924035 | C | T | -0.282 | 0.056 | 0.024 | 4.60E-07 | 7174 |
|  | rs55841968 | 6 | 123212261 | T | C | 0.077 | 0.017 | 0.493 | 5.99E-06 | 7174 |
|  | rs9397785 | 6 | 155156506 | G | T | -0.079 | 0.018 | 0.655 | 7.36E-06 | 7174 |
|  | rs5025214 | 8 | 6245562 | T | G | -0.082 | 0.018 | 0.680 | 5.20E-06 | 7174 |
|  | rs3125786 | 9 | 137735506 | A | G | -0.114 | 0.026 | 0.869 | 9.39E-06 | 7174 |
|  | rs12255873 | 10 | 60834796 | G | A | -0.139 | 0.028 | 0.901 | 9.11E-07 | 7174 |
|  | rs60847460 | 10 | 112224000 | T | C | -0.108 | 0.024 | 0.140 | 7.20E-06 | 7174 |
|  | rs174598 | 11 | 61853722 | A | G | 0.112 | 0.018 | 0.346 | 2.24E-10 | 7174 |
|  | rs7139079 | 12 | 120977490 | A | G | -0.077 | 0.017 | 0.547 | 5.11E-06 | 7174 |
|  | rs1800588 | 15 | 58431476 | T | C | 0.089 | 0.019 | 0.258 | 3.95E-06 | 7174 |
|  | rs247617 | 16 | 56956804 | A | C | 0.107 | 0.019 | 0.278 | 9.57E-09 | 7174 |
|  | rs192646452 | 17 | 27754096 | T | A | -0.255 | 0.056 | 0.023 | 4.52E-06 | 7174 |
|  | rs345182 | 17 | 60163403 | G | A | 0.098 | 0.022 | 0.189 | 7.79E-06 | 7174 |
|  | rs138477772 | 18 | 62042253 | A | G | -0.543 | 0.117 | 0.006 | 3.27E-06 | 7174 |
|  | rs182611493 | 19 | 19347579 | G | A | -0.189 | 0.038 | 0.054 | 6.28E-07 | 7174 |
|  | rs144573714 | 19 | 24009974 | T | C | -0.198 | 0.044 | 0.038 | 6.05E-06 | 7174 |
|  | rs111506410 | 20 | 1141294 | C | T | 0.243 | 0.052 | 0.028 | 2.62E-06 | 7174 |
|  | rs57021772 | 22 | 23430310 | A | G | 0.281 | 0.063 | 0.019 | 9.16E-06 | 7174 |
| Phosphatidylcholine (16:0_20:1) | rs147707008 | 2 | 13150132 | A | G | -0.198 | 0.044 | 0.043 | 5.91E-06 | 6882 |
|  | rs2268493 | 3 | 8759154 | C | T | 0.084 | 0.018 | 0.392 | 1.60E-06 | 6882 |
|  | rs150608914 | 5 | 137344967 | A | G | 0.195 | 0.040 | 0.050 | 8.25E-07 | 6882 |
|  | rs73728261 | 6 | 18747694 | T | C | 0.117 | 0.025 | 0.129 | 3.11E-06 | 6882 |
|  | rs1409892 | 6 | 51400810 | A | G | 0.079 | 0.017 | 0.448 | 4.54E-06 | 6882 |
|  | rs12707188 | 7 | 134906346 | T | C | 0.092 | 0.018 | 0.417 | 1.66E-07 | 6882 |
|  | rs2499085 | 10 | 19302248 | C | T | 0.110 | 0.024 | 0.846 | 6.30E-06 | 6882 |
|  | rs10995430 | 10 | 63087988 | T | C | -0.084 | 0.018 | 0.650 | 2.61E-06 | 6882 |
|  | rs10490936 | 10 | 82089541 | G | A | -0.427 | 0.095 | 0.010 | 7.47E-06 | 6882 |
|  | rs17715191 | 10 | 114348244 | G | A | 0.106 | 0.023 | 0.183 | 4.77E-06 | 6882 |
|  | rs174594 | 11 | 61852357 | A | C | -0.115 | 0.017 | 0.582 | 3.58E-11 | 6882 |
|  | rs6007233 | 22 | 44667863 | T | C | 0.085 | 0.018 | 0.678 | 4.06E-06 | 6882 |
| Phosphatidylcholine (16:0_20:2) | rs1168041 | 1 | 62494579 | C | T | 0.093 | 0.019 | 0.733 | 9.29E-07 | 7135 |
|  | rs1260326 | 2 | 27508073 | C | T | -0.097 | 0.018 | 0.651 | 3.00E-08 | 7135 |
|  | rs17268633 | 2 | 56194921 | G | A | -0.285 | 0.060 | 0.020 | 2.07E-06 | 7135 |
|  | rs78230994 | 4 | 26558575 | A | G | 0.595 | 0.123 | 0.006 | 1.25E-06 | 7135 |
|  | rs142392117 | 4 | 176627941 | C | T | -0.277 | 0.057 | 0.022 | 1.40E-06 | 7135 |
|  | rs3132935 | 6 | 32203298 | G | A | -0.097 | 0.022 | 0.177 | 7.80E-06 | 7135 |
|  | rs34516410 | 6 | 99968458 | T | G | -0.114 | 0.025 | 0.133 | 4.16E-06 | 7135 |
|  | rs12700446 | 7 | 23532125 | T | C | 0.309 | 0.067 | 0.016 | 4.73E-06 | 7135 |
|  | rs2236514 | 9 | 136677616 | G | C | -0.091 | 0.018 | 0.647 | 3.85E-07 | 7135 |
|  | rs148069564 | 10 | 51792352 | A | T | -0.777 | 0.167 | 0.003 | 3.28E-06 | 7135 |
|  | rs72800299 | 10 | 65395218 | A | T | -0.085 | 0.019 | 0.274 | 4.69E-06 | 7135 |
|  | rs12761749 | 10 | 90294727 | C | T | -0.105 | 0.024 | 0.150 | 9.93E-06 | 7135 |
|  | rs174592 | 11 | 61851136 | G | A | 0.262 | 0.017 | 0.418 | 4.96E-54 | 7135 |
|  | rs2317091 | 11 | 63193288 | A | T | 0.218 | 0.043 | 0.040 | 4.11E-07 | 7135 |
|  | rs1815763 | 11 | 67075266 | A | G | -0.366 | 0.080 | 0.011 | 4.39E-06 | 7135 |
|  | rs10160784 | 11 | 75745010 | C | T | -0.110 | 0.020 | 0.232 | 3.61E-08 | 7135 |
|  | rs79718024 | 11 | 107569619 | G | A | -0.113 | 0.024 | 0.155 | 1.76E-06 | 7135 |
|  | rs17128436 | 11 | 124146158 | C | G | 0.309 | 0.069 | 0.016 | 7.29E-06 | 7135 |
|  | rs17126640 | 12 | 52311207 | A | G | -0.098 | 0.021 | 0.203 | 2.70E-06 | 7135 |
|  | rs1169289 | 12 | 120978819 | G | C | 0.075 | 0.017 | 0.470 | 7.32E-06 | 7135 |
|  | rs113130033 | 14 | 89719965 | G | A | -0.197 | 0.041 | 0.043 | 1.73E-06 | 7135 |
|  | rs1532085 | 15 | 58391167 | G | A | -0.094 | 0.017 | 0.573 | 2.69E-08 | 7135 |
|  | rs1800588 | 15 | 58431476 | T | C | 0.107 | 0.019 | 0.258 | 3.85E-08 | 7135 |
|  | rs1640963 | 16 | 6506441 | C | G | -0.211 | 0.048 | 0.032 | 9.76E-06 | 7135 |
|  | rs113550786 | 16 | 13890509 | G | A | 0.393 | 0.083 | 0.012 | 2.06E-06 | 7135 |
|  | rs76711488 | 18 | 8971803 | A | G | -0.260 | 0.056 | 0.023 | 3.60E-06 | 7135 |
|  | rs62081852 | 18 | 26802732 | T | C | 0.389 | 0.068 | 0.018 | 9.35E-09 | 7135 |
|  | rs150057262 | 19 | 19210016 | G | C | -0.202 | 0.039 | 0.052 | 1.77E-07 | 7135 |
|  | rs138597 | 22 | 44572650 | A | G | 0.081 | 0.018 | 0.709 | 9.82E-06 | 7135 |
| Phosphatidylcholine (16:0_20:3) | rs1168041 | 1 | 62494579 | C | T | 0.089 | 0.019 | 0.733 | 2.10E-06 | 7174 |
|  | rs1260326 | 2 | 27508073 | C | T | -0.077 | 0.017 | 0.651 | 9.67E-06 | 7174 |
|  | rs77990460 | 2 | 126127979 | C | A | -0.307 | 0.067 | 0.017 | 5.35E-06 | 7174 |
|  | rs143838492 | 5 | 114892988 | A | G | 0.678 | 0.149 | 0.003 | 5.19E-06 | 7174 |
|  | rs141883259 | 5 | 120061007 | G | A | 0.380 | 0.083 | 0.010 | 4.89E-06 | 7174 |
|  | rs151287892 | 6 | 156361355 | A | G | -0.132 | 0.029 | 0.093 | 6.19E-06 | 7174 |
|  | rs372466687 | 7 | 146970602 | T | C | 0.314 | 0.069 | 0.015 | 5.58E-06 | 7174 |
|  | rs10102672 | 8 | 39183266 | G | C | 0.086 | 0.018 | 0.293 | 2.57E-06 | 7174 |
|  | rs117372257 | 9 | 71015708 | T | C | -0.402 | 0.088 | 0.009 | 5.23E-06 | 7174 |
|  | rs1410901 | 10 | 90245408 | T | C | 0.079 | 0.017 | 0.505 | 3.24E-06 | 7174 |
|  | rs968567 | 11 | 61828092 | T | C | 0.257 | 0.026 | 0.113 | 3.05E-22 | 7174 |
|  | rs138705401 | 12 | 43610337 | T | C | 0.183 | 0.040 | 0.047 | 4.48E-06 | 7174 |
|  | rs77419701 | 13 | 66958436 | C | T | -0.200 | 0.044 | 0.037 | 5.36E-06 | 7174 |
|  | rs319552 | 13 | 93173799 | C | G | 0.087 | 0.019 | 0.255 | 6.23E-06 | 7174 |
|  | rs7173306 | 15 | 92337647 | C | T | -0.084 | 0.018 | 0.674 | 3.78E-06 | 7174 |
|  | rs6498540 | 16 | 15036737 | G | A | -0.192 | 0.018 | 0.334 | 1.90E-27 | 7174 |
|  | rs56374730 | 16 | 15773795 | G | T | 0.083 | 0.018 | 0.296 | 5.80E-06 | 7174 |
|  | rs116917626 | 17 | 51561964 | T | C | -0.234 | 0.052 | 0.028 | 8.10E-06 | 7174 |
|  | rs150057262 | 19 | 19210016 | G | C | -0.200 | 0.039 | 0.052 | 2.39E-07 | 7174 |
|  | rs2379129 | 20 | 62310086 | A | C | -0.077 | 0.017 | 0.399 | 6.76E-06 | 7174 |
| Phosphatidylcholine (16:0_20:4) | rs116643980 | 1 | 4847659 | T | G | 0.167 | 0.037 | 0.056 | 4.84E-06 | 7174 |
|  | rs3820438 | 1 | 201955621 | C | G | 0.089 | 0.018 | 0.291 | 1.15E-06 | 7174 |
|  | rs17366743 | 3 | 186854300 | C | T | -0.203 | 0.046 | 0.034 | 9.68E-06 | 7174 |
|  | rs17017629 | 4 | 143352205 | T | C | -0.231 | 0.051 | 0.027 | 6.78E-06 | 7174 |
|  | rs116438525 | 4 | 181645150 | T | C | -0.470 | 0.102 | 0.007 | 4.05E-06 | 7174 |
|  | rs149130695 | 4 | 186464194 | C | A | 0.154 | 0.035 | 0.065 | 9.36E-06 | 7174 |
|  | rs2966427 | 7 | 110566718 | G | C | -0.147 | 0.033 | 0.932 | 7.78E-06 | 7174 |
|  | rs58160859 | 7 | 156099813 | T | C | 0.093 | 0.020 | 0.221 | 5.72E-06 | 7174 |
|  | rs1251003 | 8 | 8854096 | A | T | -0.144 | 0.030 | 0.090 | 1.23E-06 | 7174 |
|  | rs4756323 | 11 | 36469520 | T | C | -0.078 | 0.017 | 0.456 | 3.22E-06 | 7174 |
|  | rs2943813 | 11 | 61490996 | C | T | -0.139 | 0.020 | 0.783 | 9.96E-12 | 7174 |
|  | rs174527 | 11 | 61770929 | C | G | -0.179 | 0.017 | 0.444 | 8.38E-26 | 7174 |
|  | rs174533 | 11 | 61781553 | A | G | -0.598 | 0.016 | 0.409 | 6.29E-295 | 7174 |
|  | rs74754540 | 11 | 61948885 | A | G | -0.179 | 0.039 | 0.049 | 4.19E-06 | 7174 |
|  | rs113394924 | 11 | 62314821 | G | T | 0.214 | 0.030 | 0.087 | 6.09E-13 | 7174 |
|  | rs3018617 | 11 | 62435462 | G | A | -0.108 | 0.019 | 0.302 | 8.62E-09 | 7174 |
|  | rs499974 | 11 | 75743976 | A | C | -0.103 | 0.020 | 0.232 | 2.26E-07 | 7174 |
|  | rs11062107 | 12 | 2077875 | G | A | -0.089 | 0.020 | 0.223 | 9.12E-06 | 7174 |
|  | rs72709529 | 14 | 101397558 | A | C | -0.272 | 0.056 | 0.023 | 1.13E-06 | 7174 |
|  | rs147549994 | 16 | 1939264 | T | G | 0.246 | 0.045 | 0.036 | 5.51E-08 | 7174 |
|  | rs6498540 | 16 | 15036737 | G | A | -0.079 | 0.018 | 0.334 | 8.47E-06 | 7174 |
|  | rs12720922 | 16 | 56966973 | A | G | -0.096 | 0.022 | 0.179 | 9.09E-06 | 7174 |
|  | rs13329893 | 16 | 87672930 | C | A | 0.158 | 0.036 | 0.060 | 9.16E-06 | 7174 |
|  | rs141391095 | 17 | 10763022 | G | A | -0.281 | 0.060 | 0.021 | 2.58E-06 | 7174 |
|  | rs187429064 | 19 | 19269704 | G | A | -0.226 | 0.038 | 0.054 | 2.04E-09 | 7174 |
| Phosphatidylcholine (16:0_20:5) | rs4908782 | 1 | 8828055 | T | C | -0.080 | 0.017 | 0.354 | 5.17E-06 | 7173 |
|  | rs34822092 | 2 | 52123601 | A | T | -0.283 | 0.056 | 0.023 | 5.21E-07 | 7173 |
|  | rs3936196 | 2 | 239878629 | A | G | -0.077 | 0.017 | 0.609 | 9.25E-06 | 7173 |
|  | rs59839417 | 3 | 39249420 | A | G | -0.099 | 0.021 | 0.206 | 2.02E-06 | 7173 |
|  | rs2733412 | 3 | 114428196 | C | T | -0.096 | 0.021 | 0.189 | 8.72E-06 | 7173 |
|  | rs10936925 | 3 | 177015370 | T | G | 0.079 | 0.018 | 0.340 | 8.89E-06 | 7173 |
|  | rs3796575 | 4 | 155800587 | T | C | -0.092 | 0.020 | 0.228 | 6.46E-06 | 7173 |
|  | rs146489454 | 5 | 115424684 | T | C | -0.217 | 0.049 | 0.031 | 9.32E-06 | 7173 |
|  | rs113917790 | 6 | 21142482 | T | C | 0.350 | 0.074 | 0.013 | 2.55E-06 | 7173 |
|  | rs7780677 | 7 | 132693734 | A | C | -0.086 | 0.017 | 0.409 | 4.33E-07 | 7173 |
|  | rs7947954 | 11 | 42657198 | C | T | 0.108 | 0.023 | 0.155 | 2.80E-06 | 7173 |
|  | rs174527 | 11 | 61770929 | C | G | -0.101 | 0.017 | 0.444 | 3.21E-09 | 7173 |
|  | rs102274 | 11 | 61790354 | C | T | -0.373 | 0.017 | 0.409 | 4.34E-109 | 7173 |
|  | rs74754540 | 11 | 61948885 | A | G | -0.189 | 0.039 | 0.049 | 1.18E-06 | 7173 |
|  | rs113394924 | 11 | 62314821 | G | T | 0.141 | 0.030 | 0.087 | 2.28E-06 | 7173 |
|  | rs3018617 | 11 | 62435462 | G | A | -0.093 | 0.019 | 0.302 | 7.08E-07 | 7173 |
|  | rs531117 | 11 | 75745089 | T | C | -0.088 | 0.020 | 0.232 | 8.73E-06 | 7173 |
|  | rs28569162 | 11 | 87975724 | G | A | 0.115 | 0.025 | 0.131 | 4.68E-06 | 7173 |
|  | rs1481977 | 11 | 103598670 | G | A | 0.109 | 0.024 | 0.861 | 7.66E-06 | 7173 |
|  | rs2041149 | 12 | 101716327 | G | A | -0.079 | 0.017 | 0.477 | 2.73E-06 | 7173 |
|  | rs137945273 | 14 | 71270831 | T | C | -0.204 | 0.045 | 0.037 | 7.22E-06 | 7173 |
|  | rs80083325 | 14 | 104397326 | A | G | -0.214 | 0.048 | 0.035 | 8.69E-06 | 7173 |
|  | rs62025936 | 16 | 10434179 | G | A | 0.121 | 0.023 | 0.165 | 1.18E-07 | 7173 |
|  | rs61653779 | 16 | 20568904 | C | T | 0.274 | 0.060 | 0.020 | 5.99E-06 | 7173 |
|  | rs2833287 | 21 | 31127778 | G | A | 0.460 | 0.103 | 0.007 | 8.62E-06 | 7173 |
| Phosphatidylcholine (16:0_22:4) | rs75679298 | 1 | 102472833 | T | C | 0.423 | 0.095 | 0.009 | 9.08E-06 | 6874 |
|  | rs1260326 | 2 | 27508073 | C | T | -0.080 | 0.018 | 0.651 | 6.72E-06 | 6874 |
|  | rs6713301 | 2 | 30362772 | T | C | 0.421 | 0.087 | 0.010 | 1.25E-06 | 6874 |
|  | rs61008604 | 2 | 101652331 | T | G | -0.116 | 0.020 | 0.249 | 5.24E-09 | 6874 |
|  | rs4856561 | 3 | 85098584 | A | C | 0.212 | 0.040 | 0.047 | 1.46E-07 | 6874 |
|  | rs4686798 | 3 | 186727647 | T | C | -0.086 | 0.018 | 0.358 | 9.65E-07 | 6874 |
|  | rs116522970 | 4 | 14330096 | G | T | 0.565 | 0.127 | 0.005 | 8.98E-06 | 6874 |
|  | rs4693564 | 4 | 83110085 | C | A | 0.115 | 0.023 | 0.831 | 9.19E-07 | 6874 |
|  | rs115448758 | 4 | 136353788 | C | T | 0.200 | 0.040 | 0.049 | 7.11E-07 | 6874 |
|  | rs35040911 | 5 | 83594473 | T | C | -0.112 | 0.023 | 0.161 | 1.34E-06 | 6874 |
|  | rs1393144 | 5 | 126004183 | A | C | -0.104 | 0.022 | 0.172 | 3.99E-06 | 6874 |
|  | rs12528390 | 6 | 148952703 | A | G | 0.182 | 0.037 | 0.056 | 1.22E-06 | 6874 |
|  | rs2730612 | 7 | 43872241 | G | T | 0.082 | 0.017 | 0.596 | 2.94E-06 | 6874 |
|  | rs7007401 | 8 | 84225835 | C | T | -0.089 | 0.019 | 0.282 | 2.95E-06 | 6874 |
|  | rs148506839 | 8 | 118116184 | A | T | -0.233 | 0.052 | 0.028 | 8.18E-06 | 6874 |
|  | rs140028072 | 9 | 6890384 | T | C | -0.217 | 0.047 | 0.034 | 4.65E-06 | 6874 |
|  | rs1360225 | 9 | 23713150 | C | T | 0.102 | 0.021 | 0.202 | 1.66E-06 | 6874 |
|  | rs2236514 | 9 | 136677616 | G | C | -0.092 | 0.018 | 0.647 | 4.04E-07 | 6874 |
|  | rs10741214 | 10 | 130397790 | T | C | -0.087 | 0.019 | 0.710 | 7.65E-06 | 6874 |
|  | rs3741252 | 11 | 61744026 | T | C | 0.121 | 0.025 | 0.134 | 1.53E-06 | 6874 |
|  | rs174551 | 11 | 61806212 | C | T | -0.290 | 0.017 | 0.406 | 3.67E-63 | 6874 |
|  | rs570929 | 11 | 88318708 | C | G | -0.079 | 0.017 | 0.548 | 3.98E-06 | 6874 |
|  | rs192523409 | 12 | 107709655 | C | T | 0.277 | 0.056 | 0.025 | 6.89E-07 | 6874 |
|  | rs61979278 | 14 | 98833387 | C | T | -0.131 | 0.029 | 0.096 | 7.79E-06 | 6874 |
|  | rs10138425 | 14 | 100137997 | G | A | -0.102 | 0.023 | 0.165 | 8.73E-06 | 6874 |
|  | rs77730345 | 16 | 2352549 | A | G | 0.213 | 0.045 | 0.039 | 2.53E-06 | 6874 |
|  | rs72807394 | 17 | 10024173 | T | C | 0.111 | 0.025 | 0.136 | 6.60E-06 | 6874 |
|  | rs111622683 | 20 | 937096 | T | C | 0.123 | 0.028 | 0.119 | 7.96E-06 | 6874 |
| Phosphatidylcholine (16:0_22:5) | rs147057045 | 1 | 34932549 | T | C | -0.341 | 0.073 | 0.015 | 2.63E-06 | 7172 |
|  | rs3009985 | 1 | 212018649 | A | G | 0.083 | 0.018 | 0.716 | 6.56E-06 | 7172 |
|  | rs1260326 | 2 | 27508073 | C | T | -0.090 | 0.017 | 0.651 | 2.20E-07 | 7172 |
|  | rs62142428 | 2 | 35106778 | A | C | 0.236 | 0.049 | 0.031 | 1.77E-06 | 7172 |
|  | rs4016738 | 3 | 39250756 | C | G | -0.093 | 0.021 | 0.206 | 7.04E-06 | 7172 |
|  | rs62280086 | 4 | 7382480 | A | G | 0.103 | 0.023 | 0.178 | 5.38E-06 | 7172 |
|  | rs2613167 | 4 | 30161140 | T | G | 0.081 | 0.018 | 0.373 | 4.83E-06 | 7172 |
|  | rs139606388 | 5 | 134851174 | T | C | -0.221 | 0.049 | 0.029 | 6.78E-06 | 7172 |
|  | rs9918362 | 6 | 10999417 | C | T | 0.085 | 0.017 | 0.458 | 5.09E-07 | 7172 |
|  | rs3096698 | 6 | 32139362 | C | T | -0.084 | 0.017 | 0.631 | 1.39E-06 | 7172 |
|  | rs73019765 | 6 | 160908659 | C | T | 0.112 | 0.024 | 0.142 | 2.31E-06 | 7172 |
|  | rs7805803 | 7 | 50185795 | A | G | 0.081 | 0.017 | 0.402 | 2.33E-06 | 7172 |
|  | rs73225965 | 8 | 25975855 | A | G | 0.117 | 0.026 | 0.121 | 6.97E-06 | 7172 |
|  | rs58967664 | 8 | 93993037 | C | T | 0.150 | 0.034 | 0.069 | 9.44E-06 | 7172 |
|  | rs2236514 | 9 | 136677616 | G | C | -0.123 | 0.018 | 0.647 | 4.04E-12 | 7172 |
|  | rs3019200 | 11 | 61481911 | A | C | -0.109 | 0.021 | 0.803 | 3.77E-07 | 7172 |
|  | rs174527 | 11 | 61770929 | C | G | -0.113 | 0.017 | 0.444 | 3.69E-11 | 7172 |
|  | rs174533 | 11 | 61781553 | A | G | -0.413 | 0.016 | 0.409 | 5.08E-135 | 7172 |
|  | rs7936002 | 11 | 62451557 | G | A | 0.089 | 0.020 | 0.257 | 6.17E-06 | 7172 |
|  | rs508049 | 11 | 68908029 | T | C | -0.167 | 0.029 | 0.098 | 5.62E-09 | 7172 |
|  | rs80197029 | 12 | 78247210 | T | G | 0.392 | 0.081 | 0.011 | 1.48E-06 | 7172 |
|  | rs1800574 | 12 | 120979061 | T | C | 0.208 | 0.040 | 0.046 | 1.51E-07 | 7172 |
|  | rs11072257 | 15 | 71171261 | C | T | -0.120 | 0.027 | 0.111 | 8.46E-06 | 7172 |
|  | rs61653779 | 16 | 20568904 | C | T | 0.276 | 0.060 | 0.020 | 4.69E-06 | 7172 |
|  | rs12598390 | 16 | 54140373 | G | A | 0.079 | 0.017 | 0.355 | 5.89E-06 | 7172 |
|  | rs1242492 | 17 | 17471077 | C | T | -0.075 | 0.017 | 0.501 | 8.58E-06 | 7172 |
|  | rs147797702 | 18 | 52743009 | C | T | -0.477 | 0.089 | 0.009 | 8.64E-08 | 7172 |
|  | rs146509239 | 18 | 54253269 | C | G | 0.316 | 0.067 | 0.016 | 2.60E-06 | 7172 |
|  | rs56336136 | 19 | 48224239 | T | C | 0.125 | 0.028 | 0.104 | 5.35E-06 | 7172 |
|  | rs62229686 | 21 | 43971391 | T | C | -0.246 | 0.042 | 0.039 | 4.54E-09 | 7172 |
| Phosphatidylcholine (16:0_22:6) | rs57634784 | 2 | 158238814 | T | C | -0.087 | 0.018 | 0.331 | 6.93E-07 | 7170 |
|  | rs76218130 | 4 | 11829414 | C | A | 0.249 | 0.056 | 0.024 | 8.52E-06 | 7170 |
|  | rs3775850 | 4 | 119529863 | A | G | -0.096 | 0.021 | 0.204 | 4.12E-06 | 7170 |
|  | rs6864719 | 5 | 54649287 | G | A | -0.171 | 0.038 | 0.949 | 8.12E-06 | 7170 |
|  | rs143699848 | 5 | 82984713 | C | T | -0.340 | 0.074 | 0.013 | 5.15E-06 | 7170 |
|  | rs77304558 | 5 | 153118411 | C | T | -0.915 | 0.196 | 0.002 | 3.14E-06 | 7170 |
|  | rs10498720 | 6 | 24353208 | C | A | 0.262 | 0.050 | 0.029 | 2.01E-07 | 7170 |
|  | rs2075083 | 7 | 17680332 | T | G | 0.081 | 0.017 | 0.581 | 1.42E-06 | 7170 |
|  | rs73188528 | 7 | 106395713 | A | G | 0.098 | 0.022 | 0.200 | 8.35E-06 | 7170 |
|  | rs17173596 | 7 | 150780296 | A | G | 0.082 | 0.018 | 0.304 | 6.93E-06 | 7170 |
|  | rs117398583 | 10 | 125205699 | A | G | -0.243 | 0.051 | 0.032 | 1.88E-06 | 7170 |
|  | rs117185962 | 10 | 126057886 | C | A | -0.268 | 0.059 | 0.022 | 5.62E-06 | 7170 |
|  | rs61900787 | 11 | 42654689 | C | T | 0.079 | 0.017 | 0.415 | 3.34E-06 | 7170 |
|  | rs174544 | 11 | 61800281 | A | C | -0.127 | 0.017 | 0.380 | 2.30E-13 | 7170 |
|  | rs964184 | 11 | 116778201 | C | G | -0.122 | 0.023 | 0.849 | 1.38E-07 | 7170 |
|  | rs4937784 | 11 | 133501713 | T | C | -0.119 | 0.025 | 0.136 | 1.56E-06 | 7170 |
|  | rs1935135 | 13 | 107675829 | C | T | 0.083 | 0.018 | 0.287 | 6.42E-06 | 7170 |
|  | rs10150075 | 14 | 72571809 | T | C | -0.191 | 0.038 | 0.053 | 5.88E-07 | 7170 |
|  | rs79508872 | 14 | 78360286 | A | C | -0.276 | 0.060 | 0.020 | 4.14E-06 | 7170 |
|  | rs77456635 | 15 | 64865848 | A | G | -0.239 | 0.054 | 0.024 | 8.62E-06 | 7170 |
|  | rs117061362 | 15 | 101196347 | T | G | 0.215 | 0.041 | 0.045 | 1.81E-07 | 7170 |
|  | rs74883996 | 16 | 24973770 | T | C | 0.382 | 0.084 | 0.011 | 5.82E-06 | 7170 |
|  | rs76744655 | 20 | 57590477 | C | T | -0.247 | 0.055 | 0.026 | 7.72E-06 | 7170 |
| Phosphatidylcholine (16:1_18:0) | rs2609122 | 2 | 6080522 | C | G | -0.275 | 0.057 | 0.024 | 1.55E-06 | 6524 |
|  | rs145028924 | 2 | 15232755 | G | A | -0.449 | 0.100 | 0.009 | 6.87E-06 | 6524 |
|  | rs1260326 | 2 | 27508073 | C | T | -0.108 | 0.018 | 0.651 | 3.75E-09 | 6524 |
|  | rs114728714 | 2 | 172180344 | A | G | 0.204 | 0.045 | 0.041 | 6.82E-06 | 6524 |
|  | rs62239496 | 3 | 37451054 | G | A | -0.098 | 0.022 | 0.201 | 8.56E-06 | 6524 |
|  | rs1533362 | 4 | 17072136 | A | C | 0.114 | 0.025 | 0.145 | 3.89E-06 | 6524 |
|  | rs76712623 | 4 | 131189906 | A | G | -0.416 | 0.084 | 0.011 | 6.84E-07 | 6524 |
|  | rs28807772 | 5 | 165822116 | G | A | 0.101 | 0.022 | 0.202 | 3.33E-06 | 6524 |
|  | rs78275150 | 7 | 137280208 | C | T | 0.280 | 0.062 | 0.020 | 7.25E-06 | 6524 |
|  | rs4332435 | 10 | 128061194 | T | A | 0.186 | 0.041 | 0.951 | 5.56E-06 | 6524 |
|  | rs174559 | 11 | 61814184 | A | G | 0.095 | 0.019 | 0.314 | 5.39E-07 | 6524 |
|  | rs11112689 | 12 | 105707476 | C | G | -0.088 | 0.019 | 0.273 | 5.84E-06 | 6524 |
|  | rs7991657 | 13 | 37372860 | T | C | 0.157 | 0.035 | 0.929 | 5.95E-06 | 6524 |
|  | rs10520096 | 15 | 38611953 | C | G | 0.151 | 0.033 | 0.075 | 5.82E-06 | 6524 |
|  | rs77007318 | 16 | 59856268 | C | G | -0.279 | 0.055 | 0.029 | 3.19E-07 | 6524 |
|  | rs9934078 | 16 | 78478626 | C | A | -0.095 | 0.020 | 0.271 | 1.74E-06 | 6524 |
|  | rs76447935 | 19 | 58583007 | T | C | 0.307 | 0.061 | 0.023 | 4.94E-07 | 6524 |
|  | rs2379129 | 20 | 62310086 | A | C | -0.087 | 0.018 | 0.399 | 1.44E-06 | 6524 |
|  | rs117402498 | 21 | 14678032 | T | C | 0.199 | 0.041 | 0.047 | 1.59E-06 | 6524 |
| Phosphatidylcholine (16:1_18:1) | rs701919 | 1 | 211687024 | C | G | -0.094 | 0.020 | 0.790 | 5.04E-06 | 7166 |
|  | rs1260326 | 2 | 27508073 | C | T | -0.110 | 0.017 | 0.651 | 2.95E-10 | 7166 |
|  | rs5865 | 2 | 97756543 | T | C | 0.087 | 0.018 | 0.712 | 2.13E-06 | 7166 |
|  | rs13402814 | 2 | 137186441 | T | A | 0.169 | 0.035 | 0.060 | 1.68E-06 | 7166 |
|  | rs80160469 | 2 | 169154895 | A | G | -0.225 | 0.049 | 0.032 | 5.50E-06 | 7166 |
|  | rs56316064 | 3 | 187394534 | G | T | -0.090 | 0.019 | 0.292 | 1.58E-06 | 7166 |
|  | rs74357965 | 5 | 20149566 | C | A | -0.126 | 0.028 | 0.102 | 7.10E-06 | 7166 |
|  | rs113366516 | 6 | 138148100 | T | G | 0.311 | 0.066 | 0.017 | 2.65E-06 | 7166 |
|  | rs75102979 | 7 | 81204492 | A | G | 0.183 | 0.041 | 0.047 | 8.63E-06 | 7166 |
|  | rs145551604 | 7 | 107240375 | T | C | 0.155 | 0.033 | 0.073 | 2.03E-06 | 7166 |
|  | rs141084476 | 8 | 5187127 | T | G | 0.188 | 0.042 | 0.043 | 6.02E-06 | 7166 |
|  | rs7007401 | 8 | 84225835 | C | T | -0.082 | 0.019 | 0.282 | 9.16E-06 | 7166 |
|  | rs72834725 | 10 | 62688203 | T | C | 0.196 | 0.042 | 0.043 | 2.92E-06 | 7166 |
|  | rs11189347 | 10 | 81316583 | A | G | -0.116 | 0.025 | 0.133 | 2.57E-06 | 7166 |
|  | rs603424 | 10 | 100315722 | A | G | -0.176 | 0.026 | 0.121 | 1.21E-11 | 7166 |
|  | rs4447106 | 10 | 100600003 | A | G | 0.128 | 0.024 | 0.141 | 1.29E-07 | 7166 |
|  | rs139935251 | 11 | 30070603 | C | T | -0.723 | 0.158 | 0.003 | 4.78E-06 | 7166 |
|  | rs174560 | 11 | 61814292 | C | T | 0.176 | 0.017 | 0.383 | 2.28E-24 | 7166 |
|  | rs7968329 | 12 | 4996173 | G | A | -0.075 | 0.017 | 0.440 | 6.45E-06 | 7166 |
|  | rs116997061 | 12 | 19869115 | T | C | -0.168 | 0.036 | 0.062 | 3.00E-06 | 7166 |
|  | rs1532085 | 15 | 58391167 | G | A | -0.093 | 0.017 | 0.573 | 3.79E-08 | 7166 |
|  | rs8033940 | 15 | 58432643 | A | G | 0.094 | 0.018 | 0.337 | 2.02E-07 | 7166 |
|  | rs117217513 | 16 | 6532848 | T | G | -0.231 | 0.051 | 0.028 | 5.76E-06 | 7166 |
|  | rs56156922 | 16 | 56953457 | C | T | 0.101 | 0.019 | 0.279 | 5.95E-08 | 7166 |
|  | rs185867215 | 17 | 18969312 | A | C | 0.565 | 0.124 | 0.005 | 5.40E-06 | 7166 |
|  | rs146934967 | 18 | 15033501 | T | C | 0.415 | 0.074 | 0.013 | 1.83E-08 | 7166 |
|  | rs36106314 | 18 | 61427576 | G | A | -0.088 | 0.020 | 0.253 | 9.22E-06 | 7166 |
|  | rs8100204 | 19 | 19282905 | A | G | -0.114 | 0.022 | 0.171 | 4.12E-07 | 7166 |
| Phosphatidylcholine (16:1_18:2) | rs12031139 | 1 | 213668292 | G | T | 0.074 | 0.017 | 0.489 | 8.07E-06 | 7165 |
|  | rs1260326 | 2 | 27508073 | C | T | -0.098 | 0.017 | 0.651 | 1.66E-08 | 7165 |
|  | rs10201921 | 2 | 137159225 | A | G | 0.157 | 0.035 | 0.060 | 8.15E-06 | 7165 |
|  | rs9839380 | 3 | 26850125 | G | A | 0.096 | 0.018 | 0.335 | 6.37E-08 | 7165 |
|  | rs13150924 | 4 | 169277659 | A | G | -0.121 | 0.025 | 0.130 | 1.37E-06 | 7165 |
|  | rs6876235 | 5 | 86303978 | C | T | 0.131 | 0.028 | 0.905 | 3.88E-06 | 7165 |
|  | rs184289628 | 6 | 139585552 | G | T | 0.284 | 0.062 | 0.018 | 5.40E-06 | 7165 |
|  | rs9480322 | 6 | 156383631 | C | T | -0.220 | 0.045 | 0.037 | 8.16E-07 | 7165 |
|  | rs7783799 | 7 | 6533456 | G | T | 0.086 | 0.018 | 0.308 | 2.63E-06 | 7165 |
|  | rs77949981 | 7 | 10911524 | G | T | -0.275 | 0.061 | 0.020 | 6.23E-06 | 7165 |
|  | rs7036554 | 9 | 14382621 | C | T | 0.078 | 0.018 | 0.350 | 9.12E-06 | 7165 |
|  | rs603424 | 10 | 100315722 | A | G | -0.161 | 0.026 | 0.121 | 5.15E-10 | 7165 |
|  | rs174527 | 11 | 61770929 | C | G | 0.118 | 0.017 | 0.444 | 4.23E-12 | 7165 |
|  | rs174574 | 11 | 61832870 | C | A | -0.366 | 0.016 | 0.591 | 2.11E-105 | 7165 |
|  | rs7936002 | 11 | 62451557 | G | A | -0.115 | 0.020 | 0.257 | 4.22E-09 | 7165 |
|  | rs11602429 | 11 | 92398043 | G | A | 0.112 | 0.025 | 0.132 | 5.49E-06 | 7165 |
|  | rs10890773 | 11 | 107889276 | A | G | -0.109 | 0.024 | 0.860 | 4.82E-06 | 7165 |
|  | rs1532085 | 15 | 58391167 | G | A | -0.097 | 0.017 | 0.573 | 7.02E-09 | 7165 |
|  | rs1800588 | 15 | 58431476 | T | C | 0.116 | 0.019 | 0.258 | 2.21E-09 | 7165 |
|  | rs4122352 | 16 | 15080714 | A | G | -0.088 | 0.018 | 0.281 | 1.96E-06 | 7165 |
|  | rs56156922 | 16 | 56953457 | C | T | 0.114 | 0.019 | 0.279 | 8.30E-10 | 7165 |
|  | rs216958 | 16 | 60201094 | G | A | -0.079 | 0.017 | 0.617 | 4.47E-06 | 7165 |
|  | rs146934967 | 18 | 15033501 | T | C | 0.443 | 0.074 | 0.013 | 1.88E-09 | 7165 |
|  | rs62104079 | 18 | 71672897 | G | C | 0.104 | 0.020 | 0.232 | 2.34E-07 | 7165 |
|  | rs150057262 | 19 | 19210016 | G | C | -0.197 | 0.039 | 0.052 | 3.57E-07 | 7165 |
|  | rs78575494 | 20 | 1014797 | A | G | 0.207 | 0.046 | 0.037 | 7.79E-06 | 7165 |
| Phosphatidylcholine (16:1_20:4) | rs2446624 | 1 | 161649571 | T | C | 0.163 | 0.035 | 0.085 | 2.80E-06 | 5564 |
|  | rs75878220 | 1 | 163867608 | C | G | -0.306 | 0.062 | 0.025 | 7.32E-07 | 5564 |
|  | rs6545883 | 2 | 61545122 | G | A | 0.095 | 0.020 | 0.620 | 1.17E-06 | 5564 |
|  | rs9809733 | 3 | 43486710 | A | C | -0.084 | 0.019 | 0.562 | 9.73E-06 | 5564 |
|  | rs2197750 | 3 | 60495677 | T | C | 0.164 | 0.037 | 0.071 | 7.74E-06 | 5564 |
|  | rs35811605 | 3 | 159910270 | T | A | 0.138 | 0.029 | 0.122 | 2.25E-06 | 5564 |
|  | rs4539990 | 4 | 144171704 | C | T | -0.103 | 0.023 | 0.769 | 9.31E-06 | 5564 |
|  | rs2085061 | 4 | 176373256 | G | T | 0.188 | 0.041 | 0.934 | 6.19E-06 | 5564 |
|  | rs1757973 | 6 | 168992667 | A | G | 0.093 | 0.021 | 0.723 | 9.54E-06 | 5564 |
|  | rs7787163 | 7 | 89538067 | T | C | 0.605 | 0.121 | 0.007 | 5.65E-07 | 5564 |
|  | rs56317095 | 7 | 150431359 | A | G | 0.269 | 0.056 | 0.031 | 1.62E-06 | 5564 |
|  | rs174527 | 11 | 61770929 | C | G | -0.125 | 0.019 | 0.444 | 1.09E-10 | 5564 |
|  | rs174535 | 11 | 61783884 | C | T | -0.328 | 0.019 | 0.409 | 5.33E-64 | 5564 |
|  | rs695112 | 11 | 75745535 | T | A | -0.109 | 0.022 | 0.241 | 1.33E-06 | 5564 |
|  | rs75781049 | 11 | 84737810 | G | T | 0.169 | 0.038 | 0.067 | 9.13E-06 | 5564 |
|  | rs591931 | 11 | 124824361 | T | C | 0.110 | 0.024 | 0.192 | 5.55E-06 | 5564 |
|  | rs117505687 | 12 | 96871603 | A | G | -0.226 | 0.051 | 0.038 | 9.36E-06 | 5564 |
|  | rs10848122 | 12 | 130508437 | C | G | -0.110 | 0.023 | 0.778 | 2.54E-06 | 5564 |
|  | rs8068175 | 17 | 17971172 | T | C | 0.089 | 0.019 | 0.421 | 3.32E-06 | 5564 |
|  | rs11650558 | 17 | 21397913 | T | C | 0.092 | 0.019 | 0.537 | 1.49E-06 | 5564 |
|  | rs11659363 | 18 | 22904677 | G | T | 0.104 | 0.022 | 0.242 | 2.60E-06 | 5564 |
|  | rs8100204 | 19 | 19282905 | A | G | -0.126 | 0.026 | 0.171 | 9.87E-07 | 5564 |
|  | rs192436652 | 19 | 54449569 | T | C | 0.203 | 0.043 | 0.053 | 2.80E-06 | 5564 |
| Phosphatidylcholine (17:0_18:1) | rs17030373 | 1 | 7149275 | G | A | 0.083 | 0.019 | 0.290 | 9.60E-06 | 7073 |
|  | rs139070595 | 1 | 247024993 | A | T | 0.592 | 0.122 | 0.005 | 1.35E-06 | 7073 |
|  | rs1257173 | 2 | 134188357 | C | G | -0.134 | 0.030 | 0.088 | 7.90E-06 | 7073 |
|  | rs145967151 | 2 | 241739239 | T | C | -0.114 | 0.026 | 0.129 | 9.92E-06 | 7073 |
|  | rs5022521 | 4 | 110207431 | T | C | 0.096 | 0.021 | 0.797 | 6.55E-06 | 7073 |
|  | rs77534789 | 4 | 130689035 | A | C | -0.208 | 0.045 | 0.038 | 4.12E-06 | 7073 |
|  | rs4692808 | 4 | 170196755 | A | G | 0.080 | 0.017 | 0.436 | 2.08E-06 | 7073 |
|  | rs1106933 | 6 | 166616136 | G | A | 0.125 | 0.024 | 0.136 | 3.44E-07 | 7073 |
|  | rs2126263 | 8 | 9324101 | A | G | 0.123 | 0.024 | 0.850 | 1.95E-07 | 7073 |
|  | rs187552376 | 8 | 93920504 | C | T | -0.318 | 0.070 | 0.016 | 6.09E-06 | 7073 |
|  | rs78308012 | 9 | 137586795 | G | T | 0.153 | 0.029 | 0.103 | 1.11E-07 | 7073 |
|  | rs143506888 | 12 | 14115139 | T | A | 0.296 | 0.064 | 0.018 | 4.13E-06 | 7073 |
|  | rs76157703 | 13 | 78203799 | T | G | -0.324 | 0.071 | 0.015 | 5.78E-06 | 7073 |
|  | rs7350789 | 15 | 58387469 | A | G | 0.113 | 0.017 | 0.393 | 4.46E-11 | 7073 |
|  | rs17231506 | 16 | 56960616 | T | C | 0.094 | 0.019 | 0.278 | 5.82E-07 | 7073 |
|  | rs12373089 | 16 | 79172686 | A | G | 0.112 | 0.025 | 0.135 | 8.44E-06 | 7073 |
|  | rs61737926 | 16 | 87644718 | T | C | -0.754 | 0.164 | 0.003 | 4.10E-06 | 7073 |
|  | rs141245990 | 18 | 44024688 | T | C | -0.247 | 0.054 | 0.026 | 3.97E-06 | 7073 |
|  | rs2003890 | 20 | 61233003 | G | A | -0.093 | 0.020 | 0.223 | 4.57E-06 | 7073 |
|  | rs140419225 | 20 | 61849802 | A | G | 0.646 | 0.139 | 0.004 | 3.40E-06 | 7073 |
| Phosphatidylcholine (17:0_18:2) | rs1168104 | 1 | 62662654 | G | A | 0.086 | 0.019 | 0.738 | 4.96E-06 | 7170 |
|  | rs1253283 | 1 | 99137108 | T | C | 0.226 | 0.051 | 0.028 | 9.36E-06 | 7170 |
|  | rs72747028 | 1 | 211733797 | C | T | -0.151 | 0.032 | 0.073 | 3.25E-06 | 7170 |
|  | rs57518551 | 2 | 42156698 | C | T | -0.125 | 0.025 | 0.132 | 3.79E-07 | 7170 |
|  | rs192537901 | 4 | 113463133 | C | T | -0.257 | 0.053 | 0.026 | 1.10E-06 | 7170 |
|  | rs7437408 | 4 | 149246245 | G | A | 0.077 | 0.017 | 0.478 | 4.54E-06 | 7170 |
|  | rs4478142 | 4 | 169401962 | T | C | -0.100 | 0.022 | 0.166 | 7.67E-06 | 7170 |
|  | rs1609446 | 5 | 3220235 | A | C | 0.143 | 0.032 | 0.078 | 7.97E-06 | 7170 |
|  | rs115686032 | 5 | 40282763 | T | C | -0.199 | 0.043 | 0.039 | 3.39E-06 | 7170 |
|  | rs111233850 | 5 | 107005641 | G | C | 0.224 | 0.050 | 0.030 | 7.06E-06 | 7170 |
|  | rs73058910 | 7 | 14912136 | C | T | 0.169 | 0.037 | 0.063 | 3.80E-06 | 7170 |
|  | rs34489224 | 7 | 100927939 | G | C | 0.088 | 0.019 | 0.266 | 3.75E-06 | 7170 |
|  | rs4840981 | 8 | 8532922 | G | C | 0.339 | 0.073 | 0.987 | 4.05E-06 | 7170 |
|  | rs2126260 | 8 | 9327571 | C | T | 0.091 | 0.019 | 0.749 | 1.72E-06 | 7170 |
|  | rs12548274 | 8 | 133696200 | G | A | 0.104 | 0.022 | 0.170 | 2.63E-06 | 7170 |
|  | rs11027519 | 11 | 23815041 | T | C | -0.132 | 0.030 | 0.089 | 8.04E-06 | 7170 |
|  | rs174566 | 11 | 61824890 | G | A | 0.259 | 0.017 | 0.408 | 9.03E-53 | 7170 |
|  | rs964184 | 11 | 116778201 | C | G | -0.154 | 0.023 | 0.849 | 2.76E-11 | 7170 |
|  | rs422137 | 15 | 58416983 | A | G | 0.085 | 0.017 | 0.493 | 3.66E-07 | 7170 |
|  | rs1800588 | 15 | 58431476 | T | C | 0.115 | 0.019 | 0.258 | 3.31E-09 | 7170 |
|  | rs141509040 | 15 | 73731833 | T | C | -0.143 | 0.032 | 0.074 | 7.49E-06 | 7170 |
|  | rs6498540 | 16 | 15036737 | G | A | 0.090 | 0.018 | 0.334 | 3.81E-07 | 7170 |
|  | rs17231506 | 16 | 56960616 | T | C | 0.097 | 0.019 | 0.278 | 1.89E-07 | 7170 |
|  | rs78592970 | 19 | 44938026 | T | G | -0.128 | 0.026 | 0.127 | 5.18E-07 | 7170 |
|  | rs111314022 | 22 | 20410911 | C | T | 0.225 | 0.050 | 0.029 | 6.54E-06 | 7170 |
| Phosphatidylcholine (17:0_20:4) | rs116643980 | 1 | 4847659 | T | G | 0.169 | 0.037 | 0.056 | 4.65E-06 | 7106 |
|  | rs143309746 | 1 | 27599934 | G | A | 0.270 | 0.059 | 0.023 | 4.94E-06 | 7106 |
|  | rs1168041 | 1 | 62494579 | C | T | 0.088 | 0.019 | 0.733 | 2.75E-06 | 7106 |
|  | rs35788378 | 1 | 236035822 | T | A | 0.085 | 0.019 | 0.262 | 5.52E-06 | 7106 |
|  | rs190664666 | 2 | 142929648 | A | G | -0.158 | 0.035 | 0.064 | 5.01E-06 | 7106 |
|  | rs55725503 | 3 | 66040764 | A | G | -0.248 | 0.055 | 0.025 | 5.71E-06 | 7106 |
|  | rs3110971 | 5 | 38490169 | G | A | 0.099 | 0.021 | 0.208 | 1.78E-06 | 7106 |
|  | rs56399423 | 5 | 132336964 | C | T | 0.084 | 0.018 | 0.323 | 2.49E-06 | 7106 |
|  | rs142082962 | 6 | 158517411 | A | C | -0.144 | 0.031 | 0.078 | 3.46E-06 | 7106 |
|  | rs73161879 | 7 | 152050140 | A | G | -0.103 | 0.022 | 0.181 | 3.77E-06 | 7106 |
|  | rs143060591 | 8 | 85171256 | T | C | -0.150 | 0.032 | 0.074 | 3.72E-06 | 7106 |
|  | rs585002 | 9 | 15293515 | C | T | 0.115 | 0.024 | 0.862 | 2.33E-06 | 7106 |
|  | rs117580248 | 9 | 137477951 | A | G | 0.131 | 0.028 | 0.110 | 2.78E-06 | 7106 |
|  | rs174527 | 11 | 61770929 | C | G | -0.176 | 0.017 | 0.444 | 1.14E-24 | 7106 |
|  | rs174533 | 11 | 61781553 | A | G | -0.543 | 0.016 | 0.409 | 9.69E-236 | 7106 |
|  | rs57815521 | 11 | 61960675 | T | C | -0.295 | 0.053 | 0.027 | 2.07E-08 | 7106 |
|  | rs140619100 | 11 | 62195501 | G | A | 0.218 | 0.035 | 0.062 | 3.15E-10 | 7106 |
|  | rs61896563 | 11 | 62267340 | T | C | -0.188 | 0.040 | 0.048 | 2.47E-06 | 7106 |
|  | rs113394924 | 11 | 62314821 | G | T | 0.194 | 0.030 | 0.087 | 8.01E-11 | 7106 |
|  | rs600518 | 11 | 75744328 | T | A | -0.113 | 0.020 | 0.232 | 1.42E-08 | 7106 |
|  | rs74452899 | 12 | 88630462 | C | T | -0.446 | 0.088 | 0.011 | 4.64E-07 | 7106 |
|  | rs2398428 | 12 | 128751021 | G | A | -0.097 | 0.020 | 0.263 | 8.96E-07 | 7106 |
|  | rs12429208 | 13 | 37767109 | T | C | 0.088 | 0.019 | 0.274 | 3.76E-06 | 7106 |
|  | rs11626405 | 14 | 81838369 | A | G | -0.081 | 0.018 | 0.307 | 7.26E-06 | 7106 |
|  | rs12928399 | 16 | 14611144 | C | G | -0.086 | 0.018 | 0.361 | 1.08E-06 | 7106 |
|  | rs635196 | 18 | 44179195 | C | T | 0.100 | 0.022 | 0.184 | 3.91E-06 | 7106 |
|  | rs2469035 | 18 | 73095106 | G | A | -0.092 | 0.020 | 0.787 | 6.69E-06 | 7106 |
|  | rs187429064 | 19 | 19269704 | G | A | -0.240 | 0.038 | 0.054 | 3.18E-10 | 7106 |
|  | rs11673120 | 19 | 47696062 | A | G | 0.076 | 0.017 | 0.419 | 7.45E-06 | 7106 |
|  | rs6083838 | 20 | 25397682 | G | A | -0.078 | 0.017 | 0.536 | 4.51E-06 | 7106 |
|  | rs11088781 | 21 | 20132472 | A | G | 0.145 | 0.031 | 0.914 | 2.27E-06 | 7106 |
| Phosphatidylcholine (18:0_18:1) | rs2797620 | 1 | 94975736 | T | C | -0.102 | 0.018 | 0.333 | 6.23E-09 | 7173 |
|  | rs34833702 | 1 | 218198038 | T | C | 0.186 | 0.038 | 0.052 | 9.96E-07 | 7173 |
|  | rs612577 | 1 | 230188618 | C | T | -0.128 | 0.022 | 0.180 | 3.63E-09 | 7173 |
|  | rs2183640 | 1 | 232142866 | A | G | -0.107 | 0.023 | 0.162 | 2.30E-06 | 7173 |
|  | rs11123861 | 2 | 97693419 | T | C | 0.084 | 0.019 | 0.723 | 8.22E-06 | 7173 |
|  | rs535686 | 3 | 155568069 | C | T | -0.221 | 0.048 | 0.034 | 4.00E-06 | 7173 |
|  | rs115754811 | 3 | 180551409 | T | G | -0.296 | 0.066 | 0.017 | 7.11E-06 | 7173 |
|  | rs67835422 | 4 | 71877919 | T | C | -0.082 | 0.018 | 0.277 | 9.60E-06 | 7173 |
|  | rs4629629 | 5 | 86296114 | T | C | 0.094 | 0.020 | 0.765 | 1.83E-06 | 7173 |
|  | rs140116915 | 6 | 11511662 | A | G | -0.515 | 0.115 | 0.006 | 7.24E-06 | 7173 |
|  | rs10457811 | 6 | 147266089 | G | A | -0.153 | 0.034 | 0.067 | 6.19E-06 | 7173 |
|  | rs181751380 | 7 | 50773538 | A | G | 0.228 | 0.047 | 0.034 | 1.34E-06 | 7173 |
|  | rs2169387 | 8 | 9323885 | G | A | 0.107 | 0.023 | 0.850 | 4.82E-06 | 7173 |
|  | rs12335533 | 9 | 111192133 | A | G | -0.076 | 0.017 | 0.413 | 9.10E-06 | 7173 |
|  | rs11197775 | 10 | 116636627 | C | T | 0.077 | 0.017 | 0.532 | 4.55E-06 | 7173 |
|  | rs150862068 | 11 | 44029081 | T | C | -0.742 | 0.161 | 0.003 | 3.97E-06 | 7173 |
|  | rs16916055 | 11 | 91426863 | C | T | 0.421 | 0.092 | 0.009 | 4.28E-06 | 7173 |
|  | rs117207178 | 12 | 34480655 | T | C | 0.138 | 0.029 | 0.097 | 1.38E-06 | 7173 |
|  | rs11113650 | 12 | 79313979 | C | T | -0.082 | 0.019 | 0.288 | 9.14E-06 | 7173 |
|  | rs73215839 | 12 | 92131416 | T | C | 0.241 | 0.047 | 0.033 | 3.62E-07 | 7173 |
|  | rs2708081 | 12 | 121025485 | C | T | -0.083 | 0.017 | 0.490 | 8.38E-07 | 7173 |
|  | rs8034802 | 15 | 58432593 | A | T | 0.086 | 0.018 | 0.311 | 2.94E-06 | 7173 |
|  | rs151331635 | 16 | 32012667 | T | A | 0.302 | 0.064 | 0.021 | 2.43E-06 | 7173 |
|  | rs77191869 | 16 | 70566761 | C | T | -0.207 | 0.044 | 0.037 | 3.15E-06 | 7173 |
|  | rs34832413 | 16 | 71310208 | G | A | -0.118 | 0.026 | 0.112 | 7.68E-06 | 7173 |
|  | rs1034642 | 17 | 31609275 | T | C | -0.078 | 0.018 | 0.336 | 9.86E-06 | 7173 |
|  | rs77027238 | 17 | 43849174 | A | G | 0.172 | 0.039 | 0.050 | 8.24E-06 | 7173 |
|  | rs113179719 | 18 | 48681930 | T | C | 0.151 | 0.034 | 0.067 | 9.41E-06 | 7173 |
|  | rs7252513 | 19 | 1991747 | A | G | 0.172 | 0.037 | 0.056 | 3.48E-06 | 7173 |
|  | rs5769111 | 22 | 46721676 | T | C | 0.097 | 0.022 | 0.196 | 7.44E-06 | 7173 |
| Phosphatidylcholine (18:0_18:2) | rs10889352 | 1 | 62633352 | C | T | -0.101 | 0.019 | 0.262 | 8.49E-08 | 7174 |
|  | rs6678964 | 1 | 95228712 | G | A | 0.200 | 0.028 | 0.902 | 6.84E-13 | 7174 |
|  | rs12122878 | 1 | 189927375 | T | C | -0.083 | 0.018 | 0.289 | 7.96E-06 | 7174 |
|  | rs55917360 | 1 | 211735543 | T | C | -0.173 | 0.032 | 0.073 | 6.90E-08 | 7174 |
|  | rs150457719 | 1 | 212247960 | G | A | -0.218 | 0.046 | 0.034 | 1.74E-06 | 7174 |
|  | rs12620952 | 2 | 5749013 | A | G | 0.463 | 0.102 | 0.007 | 5.72E-06 | 7174 |
|  | rs72840078 | 2 | 85365218 | G | A | 0.124 | 0.027 | 0.102 | 4.49E-06 | 7174 |
|  | rs77588674 | 2 | 121313828 | T | G | 0.100 | 0.023 | 0.169 | 8.96E-06 | 7174 |
|  | rs6432738 | 2 | 162918693 | G | A | 0.075 | 0.017 | 0.474 | 7.24E-06 | 7174 |
|  | rs16838403 | 2 | 206629893 | C | G | -0.270 | 0.061 | 0.019 | 8.74E-06 | 7174 |
|  | rs73126869 | 4 | 14739231 | G | A | 0.133 | 0.029 | 0.099 | 3.48E-06 | 7174 |
|  | rs5022521 | 4 | 110207431 | T | C | 0.094 | 0.021 | 0.797 | 9.45E-06 | 7174 |
|  | rs6878903 | 5 | 86295297 | T | G | 0.088 | 0.020 | 0.764 | 8.02E-06 | 7174 |
|  | rs10040989 | 5 | 138238036 | A | G | 0.094 | 0.021 | 0.192 | 6.86E-06 | 7174 |
|  | rs12211498 | 6 | 46779152 | A | G | -0.130 | 0.029 | 0.095 | 6.54E-06 | 7174 |
|  | rs9987289 | 8 | 9325848 | G | A | 0.126 | 0.023 | 0.852 | 8.75E-08 | 7174 |
|  | rs675849 | 9 | 15298668 | C | T | 0.127 | 0.027 | 0.899 | 3.50E-06 | 7174 |
|  | rs192934615 | 9 | 80727726 | C | T | -0.338 | 0.072 | 0.014 | 3.07E-06 | 7174 |
|  | rs2274855 | 9 | 132230557 | A | G | -0.105 | 0.023 | 0.171 | 5.43E-06 | 7174 |
|  | rs7932326 | 11 | 43971474 | A | G | 0.077 | 0.017 | 0.617 | 6.29E-06 | 7174 |
|  | rs174594 | 11 | 61852357 | A | C | -0.247 | 0.017 | 0.582 | 8.13E-49 | 7174 |
|  | rs10890773 | 11 | 107889276 | A | G | -0.109 | 0.024 | 0.860 | 4.83E-06 | 7174 |
|  | rs964184 | 11 | 116778201 | C | G | -0.128 | 0.023 | 0.849 | 3.04E-08 | 7174 |
|  | rs564216 | 11 | 126095437 | C | T | -0.165 | 0.037 | 0.055 | 6.38E-06 | 7174 |
|  | rs1440724 | 12 | 62017823 | A | G | 0.080 | 0.017 | 0.403 | 2.18E-06 | 7174 |
|  | rs422137 | 15 | 58416983 | A | G | 0.091 | 0.017 | 0.493 | 5.70E-08 | 7174 |
|  | rs1800588 | 15 | 58431476 | T | C | 0.135 | 0.019 | 0.258 | 2.52E-12 | 7174 |
|  | rs62039480 | 16 | 15043593 | A | G | 0.096 | 0.018 | 0.332 | 6.94E-08 | 7174 |
|  | rs17231506 | 16 | 56960616 | T | C | 0.107 | 0.018 | 0.278 | 6.78E-09 | 7174 |
|  | rs77008693 | 17 | 44755436 | A | G | 0.111 | 0.024 | 0.139 | 3.40E-06 | 7174 |
|  | rs34031934 | 18 | 13728480 | T | C | -0.084 | 0.017 | 0.326 | 1.43E-06 | 7174 |
|  | rs117528775 | 18 | 49649558 | C | T | 0.600 | 0.125 | 0.005 | 1.65E-06 | 7174 |
|  | rs112121830 | 21 | 32410680 | T | C | -0.164 | 0.035 | 0.064 | 3.10E-06 | 7174 |
| Phosphatidylcholine (18:0_18:3) | rs10874911 | 1 | 91728966 | A | G | -0.083 | 0.018 | 0.394 | 2.41E-06 | 6692 |
|  | rs2797620 | 1 | 94975736 | T | C | -0.097 | 0.018 | 0.333 | 1.07E-07 | 6692 |
|  | rs4846841 | 1 | 230166915 | G | A | 0.091 | 0.020 | 0.747 | 3.79E-06 | 6692 |
|  | rs1260326 | 2 | 27508073 | C | T | -0.085 | 0.018 | 0.651 | 2.52E-06 | 6692 |
|  | rs79831249 | 4 | 94538445 | C | G | 0.380 | 0.083 | 0.013 | 5.18E-06 | 6692 |
|  | rs75593273 | 5 | 44740041 | T | C | 0.138 | 0.030 | 0.095 | 3.91E-06 | 6692 |
|  | rs181264690 | 5 | 122510180 | T | G | -0.090 | 0.019 | 0.281 | 3.53E-06 | 6692 |
|  | rs183478874 | 5 | 176998593 | A | C | -0.295 | 0.059 | 0.024 | 6.57E-07 | 6692 |
|  | rs72867166 | 6 | 41878684 | A | T | 0.124 | 0.027 | 0.118 | 4.87E-06 | 6692 |
|  | rs3729986 | 11 | 47350047 | T | C | -0.125 | 0.028 | 0.105 | 8.87E-06 | 6692 |
|  | rs11222413 | 11 | 130971030 | A | T | -0.294 | 0.066 | 0.018 | 9.56E-06 | 6692 |
|  | rs116844659 | 12 | 12204586 | G | A | 0.614 | 0.135 | 0.005 | 5.67E-06 | 6692 |
|  | rs118094862 | 12 | 30829729 | T | G | -0.448 | 0.101 | 0.008 | 9.08E-06 | 6692 |
|  | rs73215839 | 12 | 92131416 | T | C | 0.269 | 0.049 | 0.033 | 4.33E-08 | 6692 |
|  | rs7139079 | 12 | 120977490 | A | G | -0.081 | 0.017 | 0.547 | 3.50E-06 | 6692 |
|  | rs16949722 | 15 | 95071963 | G | T | 0.316 | 0.071 | 0.016 | 8.83E-06 | 6692 |
|  | rs12448528 | 16 | 56951643 | G | A | 0.096 | 0.022 | 0.792 | 9.70E-06 | 6692 |
|  | rs4888557 | 16 | 76816258 | A | T | 0.306 | 0.064 | 0.019 | 2.00E-06 | 6692 |
|  | rs143029300 | 17 | 13574441 | A | G | 0.304 | 0.067 | 0.018 | 6.21E-06 | 6692 |
|  | rs62122127 | 19 | 14102503 | A | G | 0.290 | 0.063 | 0.021 | 4.65E-06 | 6692 |
|  | rs145856648 | 19 | 21959621 | T | C | 0.515 | 0.115 | 0.006 | 7.78E-06 | 6692 |
|  | rs16987303 | 19 | 56525734 | G | C | -0.097 | 0.020 | 0.234 | 2.11E-06 | 6692 |
|  | rs2834084 | 21 | 33084026 | T | C | 0.081 | 0.018 | 0.623 | 6.43E-06 | 6692 |
| Phosphatidylcholine (18:0_20:2) | rs111595890 | 1 | 214966966 | A | G | -0.294 | 0.065 | 0.022 | 6.17E-06 | 5778 |
|  | rs6744368 | 2 | 68444883 | T | C | 0.188 | 0.043 | 0.948 | 9.85E-06 | 5778 |
|  | rs12513382 | 4 | 43912586 | T | C | -0.088 | 0.019 | 0.621 | 3.68E-06 | 5778 |
|  | rs112997491 | 5 | 86253956 | A | G | -0.199 | 0.044 | 0.048 | 8.03E-06 | 5778 |
|  | rs1422819 | 5 | 166671878 | G | T | -0.090 | 0.019 | 0.593 | 1.97E-06 | 5778 |
|  | rs67207258 | 8 | 5603995 | T | C | -0.105 | 0.024 | 0.190 | 9.22E-06 | 5778 |
|  | rs112483795 | 8 | 12981507 | G | A | -0.290 | 0.062 | 0.024 | 3.37E-06 | 5778 |
|  | rs174527 | 11 | 61770929 | C | G | 0.102 | 0.019 | 0.444 | 8.17E-08 | 5778 |
|  | rs174547 | 11 | 61803311 | C | T | 0.282 | 0.019 | 0.407 | 4.05E-51 | 5778 |
|  | rs7959861 | 12 | 90339893 | G | A | 0.087 | 0.019 | 0.348 | 8.23E-06 | 5778 |
|  | rs7139079 | 12 | 120977490 | A | G | -0.099 | 0.019 | 0.547 | 1.17E-07 | 5778 |
|  | rs17238104 | 13 | 37429471 | T | C | -0.123 | 0.027 | 0.138 | 7.01E-06 | 5778 |
|  | rs147220276 | 13 | 73360255 | G | T | 0.427 | 0.095 | 0.010 | 7.84E-06 | 5778 |
|  | rs9671254 | 14 | 24946787 | A | T | -0.095 | 0.021 | 0.711 | 3.88E-06 | 5778 |
|  | rs34955778 | 16 | 15045737 | C | T | 0.098 | 0.019 | 0.436 | 2.01E-07 | 5778 |
|  | rs8068923 | 17 | 54741041 | A | G | 0.190 | 0.040 | 0.940 | 1.81E-06 | 5778 |
|  | rs8183765 | 20 | 6660791 | T | C | 0.182 | 0.041 | 0.059 | 8.04E-06 | 5778 |
|  | rs6005648 | 22 | 27828806 | G | A | -0.123 | 0.026 | 0.150 | 3.25E-06 | 5778 |
| Phosphatidylcholine (18:0_20:3) | rs116259186 | 1 | 74502464 | C | T | 0.227 | 0.051 | 0.029 | 7.49E-06 | 7169 |
|  | rs2797620 | 1 | 94975736 | T | C | -0.116 | 0.018 | 0.333 | 3.27E-11 | 7169 |
|  | rs11577288 | 1 | 242606205 | A | G | 0.089 | 0.020 | 0.234 | 7.14E-06 | 7169 |
|  | rs13001426 | 2 | 169055662 | A | C | 0.335 | 0.074 | 0.014 | 6.79E-06 | 7169 |
|  | rs62239496 | 3 | 37451054 | G | A | -0.094 | 0.021 | 0.201 | 7.30E-06 | 7169 |
|  | rs114162005 | 3 | 148308352 | C | A | -0.179 | 0.039 | 0.053 | 5.96E-06 | 7169 |
|  | rs139676091 | 4 | 16403106 | A | T | 0.371 | 0.083 | 0.011 | 7.59E-06 | 7169 |
|  | rs78009561 | 4 | 158046658 | G | C | -0.127 | 0.029 | 0.096 | 9.94E-06 | 7169 |
|  | rs140123208 | 5 | 16863519 | G | A | -0.204 | 0.044 | 0.038 | 3.45E-06 | 7169 |
|  | rs192117305 | 5 | 33853083 | T | C | -0.197 | 0.043 | 0.041 | 4.38E-06 | 7169 |
|  | rs72827678 | 5 | 168832803 | G | T | 0.143 | 0.030 | 0.086 | 2.15E-06 | 7169 |
|  | rs3134937 | 6 | 32238762 | T | C | -0.095 | 0.021 | 0.200 | 4.78E-06 | 7169 |
|  | rs151178379 | 9 | 130575137 | A | G | 0.289 | 0.061 | 0.019 | 2.48E-06 | 7169 |
|  | rs56205329 | 9 | 133667068 | A | G | -0.299 | 0.067 | 0.016 | 8.55E-06 | 7169 |
|  | rs7943728 | 11 | 61779596 | A | G | 0.236 | 0.027 | 0.112 | 6.87E-19 | 7169 |
|  | rs140588245 | 11 | 124328766 | A | G | 0.364 | 0.082 | 0.011 | 8.52E-06 | 7169 |
|  | rs112534228 | 12 | 6260820 | C | A | 0.210 | 0.045 | 0.038 | 2.78E-06 | 7169 |
|  | rs1169288 | 12 | 120978847 | C | A | 0.077 | 0.017 | 0.377 | 6.39E-06 | 7169 |
|  | rs11847009 | 14 | 33476504 | A | T | -0.111 | 0.022 | 0.174 | 6.87E-07 | 7169 |
|  | rs59739041 | 15 | 58368884 | C | T | 0.183 | 0.039 | 0.048 | 2.32E-06 | 7169 |
|  | rs6498540 | 16 | 15036737 | G | A | -0.199 | 0.018 | 0.334 | 2.09E-29 | 7169 |
|  | rs56374730 | 16 | 15773795 | G | T | 0.089 | 0.018 | 0.296 | 1.44E-06 | 7169 |
|  | rs11648397 | 16 | 78589971 | C | G | 0.152 | 0.032 | 0.078 | 1.59E-06 | 7169 |
|  | rs116917626 | 17 | 51561964 | T | C | -0.236 | 0.052 | 0.028 | 6.79E-06 | 7169 |
|  | rs4808779 | 19 | 18294126 | G | A | -0.088 | 0.018 | 0.666 | 5.93E-07 | 7169 |
|  | rs6141277 | 20 | 32243354 | C | G | -0.075 | 0.017 | 0.493 | 7.41E-06 | 7169 |
|  | rs1800961 | 20 | 44413724 | T | C | -0.184 | 0.037 | 0.052 | 7.41E-07 | 7169 |
|  | rs2379129 | 20 | 62310086 | A | C | -0.081 | 0.017 | 0.399 | 2.15E-06 | 7169 |
| Phosphatidylcholine (18:0_20:4) | rs2797620 | 1 | 94975736 | T | C | -0.101 | 0.017 | 0.333 | 6.11E-09 | 7174 |
|  | rs938822 | 3 | 64950744 | C | T | -0.106 | 0.023 | 0.146 | 4.66E-06 | 7174 |
|  | rs3110971 | 5 | 38490169 | G | A | 0.096 | 0.020 | 0.208 | 2.72E-06 | 7174 |
|  | rs6860806 | 5 | 132304843 | G | A | 0.079 | 0.017 | 0.420 | 2.78E-06 | 7174 |
|  | rs9462860 | 6 | 42979275 | C | G | 0.076 | 0.017 | 0.458 | 4.66E-06 | 7174 |
|  | rs2990536 | 10 | 36620136 | T | C | -0.078 | 0.017 | 0.578 | 3.48E-06 | 7174 |
|  | rs61873274 | 10 | 124924165 | G | A | 0.146 | 0.032 | 0.072 | 5.07E-06 | 7174 |
|  | rs12275418 | 11 | 61012351 | A | G | 0.124 | 0.025 | 0.134 | 4.66E-07 | 7174 |
|  | rs3019200 | 11 | 61481911 | A | C | -0.163 | 0.021 | 0.803 | 2.57E-14 | 7174 |
|  | rs79136768 | 11 | 61594967 | G | A | -0.249 | 0.045 | 0.037 | 2.71E-08 | 7174 |
|  | rs3741252 | 11 | 61744026 | T | C | 0.243 | 0.024 | 0.134 | 5.54E-23 | 7174 |
|  | rs174528 | 11 | 61776027 | C | T | -0.666 | 0.015 | 0.414 | 0 | 7174 |
|  | rs147981159 | 11 | 62056826 | A | G | -0.346 | 0.042 | 0.043 | 1.54E-16 | 7174 |
|  | rs4382917 | 11 | 62454004 | A | G | 0.149 | 0.020 | 0.256 | 4.68E-14 | 7174 |
|  | rs1219539 | 11 | 75722099 | A | G | -0.088 | 0.017 | 0.493 | 1.74E-07 | 7174 |
|  | rs600518 | 11 | 75744328 | T | A | -0.101 | 0.020 | 0.232 | 3.49E-07 | 7174 |
|  | rs144719617 | 12 | 91627546 | A | C | 0.126 | 0.027 | 0.107 | 2.69E-06 | 7174 |
|  | rs2802390 | 13 | 98690376 | T | C | -0.075 | 0.017 | 0.465 | 7.51E-06 | 7174 |
|  | rs139102782 | 14 | 83647468 | A | G | -0.206 | 0.046 | 0.034 | 9.06E-06 | 7174 |
|  | rs2070895 | 15 | 58431740 | A | G | 0.094 | 0.019 | 0.267 | 8.73E-07 | 7174 |
|  | rs147549994 | 16 | 1939264 | T | G | 0.256 | 0.045 | 0.036 | 1.35E-08 | 7174 |
|  | rs72789541 | 16 | 15033677 | A | T | -0.097 | 0.018 | 0.315 | 6.36E-08 | 7174 |
|  | rs7499892 | 16 | 56972678 | T | C | -0.096 | 0.022 | 0.177 | 9.21E-06 | 7174 |
|  | rs223841 | 16 | 57423359 | G | A | -0.103 | 0.022 | 0.172 | 2.89E-06 | 7174 |
|  | rs1477491 | 18 | 73092300 | G | A | -0.102 | 0.020 | 0.789 | 5.06E-07 | 7174 |
|  | rs793864 | 19 | 7552489 | C | G | -0.104 | 0.022 | 0.818 | 1.50E-06 | 7174 |
|  | rs73005469 | 19 | 15845766 | A | G | -0.077 | 0.017 | 0.357 | 9.40E-06 | 7174 |
|  | rs187429064 | 19 | 19269704 | G | A | -0.202 | 0.037 | 0.054 | 7.42E-08 | 7174 |
| Phosphatidylcholine (18:0_20:5) | rs4908782 | 1 | 8828055 | T | C | -0.078 | 0.018 | 0.354 | 8.94E-06 | 7121 |
|  | rs138053477 | 1 | 169897372 | T | C | 0.212 | 0.042 | 0.042 | 4.26E-07 | 7121 |
|  | rs1076708 | 1 | 210394086 | G | A | -0.076 | 0.017 | 0.427 | 9.92E-06 | 7121 |
|  | rs35226034 | 3 | 139980152 | G | A | -0.202 | 0.043 | 0.040 | 2.57E-06 | 7121 |
|  | rs12487951 | 3 | 169305300 | C | T | -0.228 | 0.051 | 0.028 | 9.17E-06 | 7121 |
|  | rs71629271 | 3 | 176095628 | C | T | 0.168 | 0.036 | 0.063 | 2.97E-06 | 7121 |
|  | rs2054498 | 4 | 37392086 | G | A | -0.078 | 0.017 | 0.538 | 2.78E-06 | 7121 |
|  | rs12656795 | 5 | 113783266 | C | G | -0.079 | 0.018 | 0.387 | 8.21E-06 | 7121 |
|  | rs2431163 | 5 | 174893516 | A | G | 0.264 | 0.056 | 0.027 | 2.81E-06 | 7121 |
|  | rs9380073 | 6 | 11061917 | T | C | 0.119 | 0.025 | 0.123 | 2.65E-06 | 7121 |
|  | rs187698886 | 6 | 67555567 | T | G | -0.200 | 0.045 | 0.037 | 9.12E-06 | 7121 |
|  | rs2023988 | 7 | 71849296 | T | C | -0.077 | 0.017 | 0.435 | 6.28E-06 | 7121 |
|  | rs7780677 | 7 | 132693734 | A | C | -0.081 | 0.017 | 0.409 | 2.52E-06 | 7121 |
|  | rs145259113 | 7 | 147506779 | C | T | -0.528 | 0.118 | 0.006 | 7.30E-06 | 7121 |
|  | rs5896690 | 9 | 16421864 | T | C | -0.078 | 0.017 | 0.464 | 3.08E-06 | 7121 |
|  | rs146438407 | 10 | 116536092 | T | C | -0.441 | 0.098 | 0.009 | 6.21E-06 | 7121 |
|  | rs3019200 | 11 | 61481911 | A | C | -0.108 | 0.021 | 0.803 | 4.74E-07 | 7121 |
|  | rs174527 | 11 | 61770929 | C | G | -0.116 | 0.017 | 0.444 | 1.73E-11 | 7121 |
|  | rs102275 | 11 | 61790331 | C | T | -0.420 | 0.016 | 0.410 | 3.30E-137 | 7121 |
|  | rs7936002 | 11 | 62451557 | G | A | 0.111 | 0.020 | 0.257 | 2.22E-08 | 7121 |
|  | rs508049 | 11 | 68908029 | T | C | -0.141 | 0.029 | 0.098 | 1.04E-06 | 7121 |
|  | rs9587408 | 13 | 107595697 | C | T | 0.092 | 0.020 | 0.219 | 4.86E-06 | 7121 |
|  | rs80116939 | 15 | 94356658 | A | G | -0.765 | 0.156 | 0.004 | 1.00E-06 | 7121 |
|  | rs62025936 | 16 | 10434179 | G | A | 0.123 | 0.023 | 0.165 | 6.27E-08 | 7121 |
|  | rs61653779 | 16 | 20568904 | C | T | 0.276 | 0.060 | 0.020 | 5.09E-06 | 7121 |
|  | rs2135279 | 18 | 71740925 | A | T | 0.075 | 0.017 | 0.581 | 9.45E-06 | 7121 |
| Phosphatidylcholine (18:0_22:5) | rs2797620 | 1 | 94975736 | T | C | -0.086 | 0.018 | 0.333 | 2.16E-06 | 6805 |
|  | rs34833702 | 1 | 218198038 | T | C | 0.197 | 0.039 | 0.052 | 4.44E-07 | 6805 |
|  | rs628035 | 1 | 230188399 | T | G | -0.097 | 0.021 | 0.214 | 2.72E-06 | 6805 |
|  | rs2304401 | 2 | 11621183 | C | T | -0.227 | 0.051 | 0.968 | 7.43E-06 | 6805 |
|  | rs1260326 | 2 | 27508073 | C | T | -0.101 | 0.018 | 0.651 | 1.64E-08 | 6805 |
|  | rs1220131 | 2 | 157711265 | A | T | 0.108 | 0.021 | 0.200 | 4.43E-07 | 6805 |
|  | rs115247383 | 3 | 148282274 | T | C | -0.203 | 0.042 | 0.049 | 1.84E-06 | 6805 |
|  | rs2613167 | 4 | 30161140 | T | G | 0.083 | 0.018 | 0.373 | 5.19E-06 | 6805 |
|  | rs13182955 | 5 | 52103978 | C | T | 0.078 | 0.018 | 0.378 | 9.35E-06 | 6805 |
|  | rs1465411 | 5 | 170155391 | G | A | 0.079 | 0.017 | 0.506 | 3.70E-06 | 6805 |
|  | rs9918362 | 6 | 10999417 | C | T | 0.082 | 0.017 | 0.458 | 2.24E-06 | 6805 |
|  | rs9374192 | 6 | 110673493 | C | T | -0.102 | 0.019 | 0.272 | 1.69E-07 | 6805 |
|  | rs183410044 | 7 | 54771130 | C | T | -0.329 | 0.069 | 0.016 | 1.90E-06 | 6805 |
|  | rs143380352 | 7 | 136096416 | C | G | -0.286 | 0.063 | 0.021 | 6.34E-06 | 6805 |
|  | rs4841133 | 8 | 9326154 | G | A | 0.121 | 0.024 | 0.852 | 5.19E-07 | 6805 |
|  | rs113847854 | 8 | 69019004 | T | C | 0.231 | 0.049 | 0.030 | 2.85E-06 | 6805 |
|  | rs58967664 | 8 | 93993037 | C | T | 0.153 | 0.035 | 0.069 | 9.11E-06 | 6805 |
|  | rs34415160 | 9 | 33767894 | A | G | -0.079 | 0.018 | 0.383 | 6.71E-06 | 6805 |
|  | rs2236514 | 9 | 136677616 | G | C | -0.089 | 0.018 | 0.647 | 1.28E-06 | 6805 |
|  | rs174527 | 11 | 61770929 | C | G | -0.100 | 0.018 | 0.444 | 1.07E-08 | 6805 |
|  | rs102275 | 11 | 61790331 | C | T | -0.285 | 0.017 | 0.410 | 5.38E-61 | 6805 |
|  | rs41352345 | 11 | 88482589 | T | A | -0.127 | 0.029 | 0.098 | 9.19E-06 | 6805 |
|  | rs11178277 | 12 | 70541856 | G | A | -0.077 | 0.017 | 0.472 | 6.47E-06 | 6805 |
|  | rs7968201 | 12 | 93538379 | T | G | 0.100 | 0.021 | 0.215 | 1.98E-06 | 6805 |
|  | rs1169289 | 12 | 120978819 | G | C | 0.108 | 0.017 | 0.470 | 3.73E-10 | 6805 |
|  | rs7146460 | 14 | 100895541 | T | C | -0.096 | 0.022 | 0.223 | 8.96E-06 | 6805 |
|  | rs55982271 | 15 | 70670258 | C | A | -0.245 | 0.055 | 0.024 | 9.21E-06 | 6805 |
|  | rs8068517 | 17 | 18192664 | A | G | -0.105 | 0.022 | 0.814 | 1.74E-06 | 6805 |
|  | rs147797702 | 18 | 52743009 | C | T | -0.462 | 0.092 | 0.009 | 4.97E-07 | 6805 |
|  | rs117135394 | 18 | 54154713 | T | C | 0.492 | 0.102 | 0.007 | 1.45E-06 | 6805 |
| Phosphatidylcholine (18:0_22:6) | rs7539908 | 1 | 53711606 | C | T | 0.098 | 0.022 | 0.178 | 8.56E-06 | 7004 |
|  | rs7542307 | 1 | 222093891 | T | C | 0.084 | 0.018 | 0.347 | 1.96E-06 | 7004 |
|  | rs2619330 | 3 | 124802671 | T | G | 0.126 | 0.027 | 0.112 | 3.26E-06 | 7004 |
|  | rs2067613 | 3 | 176189554 | C | T | -0.090 | 0.019 | 0.707 | 1.67E-06 | 7004 |
|  | rs62345669 | 4 | 147476374 | A | G | -0.472 | 0.100 | 0.008 | 2.36E-06 | 7004 |
|  | rs3110235 | 5 | 38486310 | T | G | 0.095 | 0.021 | 0.207 | 5.40E-06 | 7004 |
|  | rs139606388 | 5 | 134851174 | T | C | -0.244 | 0.050 | 0.029 | 1.04E-06 | 7004 |
|  | rs4987173 | 6 | 42963486 | A | G | 0.102 | 0.017 | 0.393 | 3.48E-09 | 7004 |
|  | rs9719746 | 7 | 155293878 | T | C | -0.250 | 0.055 | 0.025 | 6.55E-06 | 7004 |
|  | rs10503355 | 8 | 6096074 | T | G | -0.192 | 0.042 | 0.042 | 5.43E-06 | 7004 |
|  | rs4841133 | 8 | 9326154 | G | A | 0.106 | 0.024 | 0.852 | 8.17E-06 | 7004 |
|  | rs3779874 | 8 | 64610547 | T | C | 0.101 | 0.022 | 0.828 | 6.12E-06 | 7004 |
|  | rs11792256 | 9 | 874977 | G | T | -0.133 | 0.028 | 0.103 | 1.73E-06 | 7004 |
|  | rs61900787 | 11 | 42654689 | C | T | 0.079 | 0.017 | 0.415 | 4.34E-06 | 7004 |
|  | rs174530 | 11 | 61779120 | G | A | -0.167 | 0.017 | 0.414 | 2.21E-22 | 7004 |
|  | rs11045913 | 12 | 21263479 | G | A | -0.080 | 0.018 | 0.647 | 7.32E-06 | 7004 |
|  | rs143578075 | 12 | 46987694 | A | G | -0.177 | 0.038 | 0.050 | 3.92E-06 | 7004 |
|  | rs7974440 | 12 | 59019021 | A | G | -0.116 | 0.025 | 0.137 | 2.57E-06 | 7004 |
|  | rs10150075 | 14 | 72571809 | T | C | -0.186 | 0.039 | 0.053 | 1.56E-06 | 7004 |
|  | rs12050889 | 15 | 62205752 | A | T | -0.083 | 0.019 | 0.296 | 8.75E-06 | 7004 |
|  | rs117061362 | 15 | 101196347 | T | G | 0.189 | 0.042 | 0.045 | 5.13E-06 | 7004 |
|  | rs72827432 | 17 | 18166314 | T | G | 0.111 | 0.022 | 0.181 | 3.38E-07 | 7004 |
|  | rs6030870 | 20 | 43445166 | G | C | -0.148 | 0.033 | 0.075 | 5.83E-06 | 7004 |
|  | rs2426606 | 20 | 56297803 | T | C | 0.086 | 0.019 | 0.721 | 6.52E-06 | 7004 |
| Phosphatidylcholine (18:1_18:1) | rs10889352 | 1 | 62633352 | C | T | -0.112 | 0.019 | 0.262 | 2.61E-09 | 7174 |
|  | rs41391244 | 1 | 111314654 | C | T | -0.093 | 0.020 | 0.220 | 3.80E-06 | 7174 |
|  | rs10779836 | 1 | 230167404 | T | C | 0.092 | 0.020 | 0.777 | 4.12E-06 | 7174 |
|  | rs4848460 | 2 | 117641775 | T | C | 0.175 | 0.038 | 0.050 | 5.05E-06 | 7174 |
|  | rs56057593 | 2 | 204579749 | T | C | -0.227 | 0.049 | 0.032 | 4.30E-06 | 7174 |
|  | rs140268092 | 4 | 29977517 | T | C | 0.582 | 0.128 | 0.005 | 5.51E-06 | 7174 |
|  | rs73241068 | 4 | 38036537 | T | C | -0.218 | 0.046 | 0.034 | 1.85E-06 | 7174 |
|  | rs79008894 | 4 | 140097358 | T | C | 0.209 | 0.046 | 0.039 | 5.56E-06 | 7174 |
|  | rs41441450 | 5 | 86717236 | T | C | -0.394 | 0.085 | 0.010 | 3.92E-06 | 7174 |
|  | rs113277188 | 5 | 151601186 | C | G | -0.291 | 0.066 | 0.018 | 9.15E-06 | 7174 |
|  | rs144512980 | 6 | 18685527 | C | T | 0.370 | 0.069 | 0.015 | 8.17E-08 | 7174 |
|  | rs2126263 | 8 | 9324101 | A | G | 0.112 | 0.023 | 0.850 | 1.93E-06 | 7174 |
|  | rs13278111 | 8 | 22702678 | T | C | -0.077 | 0.017 | 0.430 | 7.00E-06 | 7174 |
|  | rs174574 | 11 | 61832870 | C | A | -0.202 | 0.017 | 0.591 | 1.28E-32 | 7174 |
|  | rs3741298 | 11 | 116786845 | T | C | -0.089 | 0.020 | 0.765 | 5.19E-06 | 7174 |
|  | rs10850057 | 12 | 112553648 | T | C | -0.125 | 0.028 | 0.100 | 8.27E-06 | 7174 |
|  | rs7300883 | 12 | 121021637 | G | C | -0.076 | 0.017 | 0.438 | 6.70E-06 | 7174 |
|  | rs10145681 | 14 | 68688212 | G | A | -0.267 | 0.057 | 0.976 | 2.32E-06 | 7174 |
|  | rs1532085 | 15 | 58391167 | G | A | -0.110 | 0.017 | 0.573 | 7.42E-11 | 7174 |
|  | rs1077834 | 15 | 58431280 | C | T | 0.093 | 0.019 | 0.266 | 1.56E-06 | 7174 |
|  | rs17231506 | 16 | 56960616 | T | C | 0.109 | 0.019 | 0.278 | 4.04E-09 | 7174 |
|  | rs2000827 | 18 | 49695288 | A | T | 0.122 | 0.027 | 0.894 | 7.27E-06 | 7174 |
|  | rs136061 | 22 | 46945349 | G | C | 0.089 | 0.019 | 0.263 | 2.41E-06 | 7174 |
| Phosphatidylcholine (18:1_18:2) | rs10889352 | 1 | 62633352 | C | T | -0.126 | 0.019 | 0.262 | 1.75E-11 | 7174 |
|  | rs9789400 | 2 | 57690087 | T | C | -0.089 | 0.019 | 0.265 | 2.74E-06 | 7174 |
|  | rs28844909 | 4 | 145707221 | A | G | -0.153 | 0.034 | 0.067 | 7.07E-06 | 7174 |
|  | rs13128807 | 4 | 169311729 | A | G | -0.101 | 0.021 | 0.196 | 1.81E-06 | 7174 |
|  | rs78882831 | 5 | 96128576 | T | C | 0.243 | 0.054 | 0.025 | 8.43E-06 | 7174 |
|  | rs142689129 | 6 | 18587903 | T | C | 0.580 | 0.116 | 0.005 | 6.42E-07 | 7174 |
|  | rs11757990 | 6 | 29796264 | T | C | 0.457 | 0.103 | 0.007 | 9.55E-06 | 7174 |
|  | rs147761802 | 7 | 5810475 | C | G | -0.181 | 0.039 | 0.050 | 4.04E-06 | 7174 |
|  | rs7036554 | 9 | 14382621 | C | T | 0.091 | 0.018 | 0.350 | 2.43E-07 | 7174 |
|  | rs174527 | 11 | 61770929 | C | G | 0.091 | 0.017 | 0.444 | 1.11E-07 | 7174 |
|  | rs174574 | 11 | 61832870 | C | A | -0.380 | 0.016 | 0.591 | 2.79E-114 | 7174 |
|  | rs118108980 | 11 | 62211023 | G | A | -0.100 | 0.021 | 0.196 | 1.93E-06 | 7174 |
|  | rs4944732 | 11 | 72735137 | T | C | 0.109 | 0.024 | 0.135 | 8.27E-06 | 7174 |
|  | rs12798268 | 11 | 107869185 | C | A | -0.107 | 0.024 | 0.859 | 7.19E-06 | 7174 |
|  | rs613808 | 11 | 116840252 | G | A | -0.091 | 0.017 | 0.619 | 1.43E-07 | 7174 |
|  | rs564216 | 11 | 126095437 | C | T | -0.168 | 0.037 | 0.055 | 4.51E-06 | 7174 |
|  | rs1601935 | 15 | 58379566 | T | G | -0.106 | 0.017 | 0.611 | 5.31E-10 | 7174 |
|  | rs1077835 | 15 | 58431227 | G | A | 0.125 | 0.019 | 0.266 | 7.94E-11 | 7174 |
|  | rs62004153 | 15 | 59198971 | A | G | -0.131 | 0.030 | 0.913 | 9.20E-06 | 7174 |
|  | rs6498540 | 16 | 15036737 | G | A | 0.079 | 0.018 | 0.334 | 7.68E-06 | 7174 |
|  | rs17231506 | 16 | 56960616 | T | C | 0.120 | 0.018 | 0.278 | 1.06E-10 | 7174 |
|  | rs948727 | 18 | 13715335 | G | A | -0.082 | 0.017 | 0.325 | 2.78E-06 | 7174 |
|  | rs6023985 | 20 | 55260213 | C | T | -0.333 | 0.072 | 0.014 | 4.10E-06 | 7174 |
|  | rs2236605 | 21 | 37563977 | C | G | 0.084 | 0.017 | 0.432 | 5.14E-07 | 7174 |
| Phosphatidylcholine (18:1_18:3) | rs114797690 | 1 | 98597981 | T | C | 0.285 | 0.062 | 0.023 | 4.84E-06 | 6339 |
|  | rs149851240 | 2 | 41556996 | C | G | 0.195 | 0.043 | 0.046 | 5.55E-06 | 6339 |
|  | rs1116931 | 3 | 100870486 | A | C | -0.093 | 0.020 | 0.272 | 2.78E-06 | 6339 |
|  | rs751841 | 4 | 5640434 | G | A | 0.088 | 0.020 | 0.645 | 6.49E-06 | 6339 |
|  | rs17208010 | 5 | 139952900 | T | C | 0.144 | 0.032 | 0.084 | 8.83E-06 | 6339 |
|  | rs2774509 | 6 | 6411842 | C | G | -0.084 | 0.019 | 0.664 | 7.22E-06 | 6339 |
|  | rs144128487 | 6 | 97082368 | G | C | -0.399 | 0.082 | 0.012 | 1.23E-06 | 6339 |
|  | rs9480321 | 6 | 156378387 | T | A | -0.204 | 0.041 | 0.050 | 6.16E-07 | 6339 |
|  | rs10769099 | 11 | 5586584 | A | G | 0.088 | 0.019 | 0.337 | 2.73E-06 | 6339 |
|  | rs174601 | 11 | 61855668 | T | C | 0.080 | 0.018 | 0.419 | 8.29E-06 | 6339 |
|  | rs116910056 | 11 | 80166090 | T | A | -0.418 | 0.095 | 0.009 | 9.84E-06 | 6339 |
|  | rs148879093 | 12 | 96120840 | A | G | -0.153 | 0.034 | 0.079 | 7.84E-06 | 6339 |
|  | rs9559745 | 13 | 110209642 | C | T | 0.426 | 0.096 | 0.009 | 8.58E-06 | 6339 |
|  | rs422137 | 15 | 58416983 | A | G | 0.081 | 0.018 | 0.493 | 6.73E-06 | 6339 |
|  | rs8033940 | 15 | 58432643 | A | G | 0.091 | 0.019 | 0.337 | 2.32E-06 | 6339 |
|  | rs17231506 | 16 | 56960616 | T | C | 0.089 | 0.020 | 0.278 | 6.76E-06 | 6339 |
|  | rs140885981 | 16 | 75302415 | A | G | -0.477 | 0.103 | 0.008 | 3.89E-06 | 6339 |
|  | rs2965212 | 19 | 2561737 | C | T | 0.103 | 0.023 | 0.820 | 9.04E-06 | 6339 |
| Phosphatidylcholine (18:1_20:2) | rs114139553 | 1 | 103864588 | A | G | -0.373 | 0.081 | 0.014 | 4.63E-06 | 5852 |
|  | rs265114 | 1 | 217457286 | T | C | 0.178 | 0.036 | 0.074 | 1.06E-06 | 5852 |
|  | rs17543793 | 5 | 17302034 | T | A | 0.301 | 0.067 | 0.020 | 6.64E-06 | 5852 |
|  | rs9384216 | 6 | 154419744 | C | T | -0.139 | 0.031 | 0.097 | 7.05E-06 | 5852 |
|  | rs6944011 | 7 | 6332316 | G | A | 0.102 | 0.023 | 0.205 | 7.50E-06 | 5852 |
|  | rs80201535 | 7 | 10826150 | A | G | -0.247 | 0.053 | 0.031 | 4.14E-06 | 5852 |
|  | rs116922907 | 7 | 106989575 | A | G | 0.214 | 0.047 | 0.042 | 5.03E-06 | 5852 |
|  | rs12113068 | 7 | 155435171 | T | G | -0.275 | 0.062 | 0.025 | 9.34E-06 | 5852 |
|  | rs6984305 | 8 | 9320758 | T | A | 0.122 | 0.025 | 0.837 | 1.30E-06 | 5852 |
|  | rs7041283 | 9 | 78442148 | C | T | -0.116 | 0.021 | 0.245 | 5.79E-08 | 5852 |
|  | rs35195855 | 11 | 36094558 | T | C | 0.130 | 0.028 | 0.148 | 3.10E-06 | 5852 |
|  | rs174527 | 11 | 61770929 | C | G | 0.094 | 0.019 | 0.444 | 7.37E-07 | 5852 |
|  | rs174580 | 11 | 61839170 | G | A | 0.431 | 0.018 | 0.410 | 1.49E-122 | 5852 |
|  | rs12417747 | 11 | 62060922 | A | G | 0.190 | 0.029 | 0.115 | 5.83E-11 | 5852 |
|  | rs61893829 | 11 | 63036470 | T | C | -0.157 | 0.034 | 0.081 | 3.96E-06 | 5852 |
|  | rs656095 | 11 | 75734293 | G | A | -0.130 | 0.022 | 0.227 | 4.24E-09 | 5852 |
|  | rs74089903 | 12 | 54160565 | A | G | -0.539 | 0.103 | 0.009 | 1.57E-07 | 5852 |
|  | rs10846753 | 12 | 124836915 | G | T | 0.106 | 0.021 | 0.235 | 8.21E-07 | 5852 |
|  | rs1800588 | 15 | 58431476 | T | C | 0.113 | 0.021 | 0.258 | 1.58E-07 | 5852 |
|  | rs117534325 | 15 | 70406039 | G | A | 0.639 | 0.135 | 0.005 | 2.47E-06 | 5852 |
|  | rs7176661 | 15 | 95538680 | C | T | 0.084 | 0.019 | 0.596 | 8.55E-06 | 5852 |
|  | rs56208677 | 16 | 56976320 | T | C | 0.182 | 0.035 | 0.078 | 2.63E-07 | 5852 |
|  | rs118150981 | 18 | 68219549 | A | G | -0.306 | 0.067 | 0.019 | 4.54E-06 | 5852 |
|  | rs73292027 | 20 | 40441104 | G | T | 0.151 | 0.030 | 0.112 | 6.31E-07 | 5852 |
| Phosphatidylcholine (18:1_20:3) | rs10889335 | 1 | 62494430 | G | A | -0.098 | 0.018 | 0.285 | 1.06E-07 | 7171 |
|  | rs2797620 | 1 | 94975736 | T | C | -0.093 | 0.018 | 0.333 | 1.49E-07 | 7171 |
|  | rs79047909 | 2 | 10043013 | G | C | -0.497 | 0.107 | 0.006 | 3.20E-06 | 7171 |
|  | rs822798 | 3 | 22971838 | G | A | 0.085 | 0.017 | 0.416 | 4.93E-07 | 7171 |
|  | rs182828998 | 4 | 115193660 | C | A | -0.492 | 0.105 | 0.007 | 2.52E-06 | 7171 |
|  | rs116647148 | 4 | 172429853 | G | A | 0.178 | 0.039 | 0.052 | 6.14E-06 | 7171 |
|  | rs12198590 | 6 | 18489429 | C | G | 0.513 | 0.114 | 0.006 | 7.34E-06 | 7171 |
|  | rs72974647 | 6 | 123327335 | G | A | 0.136 | 0.030 | 0.082 | 6.80E-06 | 7171 |
|  | rs113366516 | 6 | 138148100 | T | G | 0.297 | 0.066 | 0.017 | 7.07E-06 | 7171 |
|  | rs6985657 | 8 | 59010827 | G | A | -0.137 | 0.029 | 0.090 | 2.77E-06 | 7171 |
|  | rs10504795 | 8 | 84222419 | G | C | -0.095 | 0.019 | 0.258 | 6.32E-07 | 7171 |
|  | rs7897746 | 10 | 5578436 | T | C | 0.133 | 0.029 | 0.903 | 3.44E-06 | 7171 |
|  | rs61896141 | 11 | 61788567 | C | A | 0.224 | 0.026 | 0.113 | 3.04E-17 | 7171 |
|  | rs695112 | 11 | 75745535 | T | A | -0.100 | 0.020 | 0.241 | 3.92E-07 | 7171 |
|  | rs75926193 | 11 | 119000408 | G | A | 0.260 | 0.052 | 0.028 | 4.98E-07 | 7171 |
|  | rs7139079 | 12 | 120977490 | A | G | -0.083 | 0.017 | 0.547 | 7.63E-07 | 7171 |
|  | rs17092227 | 14 | 95434157 | C | T | 0.194 | 0.041 | 0.044 | 2.53E-06 | 7171 |
|  | rs59739041 | 15 | 58368884 | C | T | 0.173 | 0.039 | 0.048 | 8.86E-06 | 7171 |
|  | rs8033940 | 15 | 58432643 | A | G | 0.095 | 0.018 | 0.337 | 1.38E-07 | 7171 |
|  | rs6498540 | 16 | 15036737 | G | A | -0.155 | 0.018 | 0.334 | 3.06E-18 | 7171 |
|  | rs56374730 | 16 | 15773795 | G | T | 0.081 | 0.018 | 0.296 | 9.83E-06 | 7171 |
|  | rs10423742 | 19 | 19117327 | A | G | -0.093 | 0.020 | 0.753 | 1.71E-06 | 7171 |
|  | rs150057262 | 19 | 19210016 | G | C | -0.247 | 0.039 | 0.052 | 1.82E-10 | 7171 |
|  | rs641738 | 19 | 54173068 | C | T | -0.092 | 0.017 | 0.593 | 6.97E-08 | 7171 |
|  | rs6083838 | 20 | 25397682 | G | A | -0.079 | 0.017 | 0.536 | 2.60E-06 | 7171 |
| Phosphatidylcholine (18:1_20:4) | rs10889353 | 1 | 62652525 | C | A | -0.085 | 0.019 | 0.262 | 5.95E-06 | 7173 |
|  | rs4846905 | 1 | 230143835 | G | A | 0.094 | 0.020 | 0.768 | 1.50E-06 | 7173 |
|  | rs79113397 | 2 | 101570369 | C | T | 0.100 | 0.020 | 0.229 | 7.59E-07 | 7173 |
|  | rs1917701 | 2 | 141078979 | A | T | 0.156 | 0.035 | 0.929 | 7.78E-06 | 7173 |
|  | rs76582138 | 4 | 115324589 | G | A | -0.458 | 0.102 | 0.007 | 6.56E-06 | 7173 |
|  | rs6850591 | 4 | 140028398 | C | T | -0.081 | 0.017 | 0.541 | 1.64E-06 | 7173 |
|  | rs116814948 | 6 | 2822782 | C | T | -0.425 | 0.094 | 0.009 | 6.20E-06 | 7173 |
|  | rs4628166 | 7 | 39265042 | G | C | -0.076 | 0.017 | 0.634 | 9.16E-06 | 7173 |
|  | rs2943813 | 11 | 61490996 | C | T | -0.130 | 0.020 | 0.783 | 2.26E-10 | 7173 |
|  | rs174527 | 11 | 61770929 | C | G | -0.160 | 0.017 | 0.444 | 5.01E-21 | 7173 |
|  | rs174537 | 11 | 61785208 | T | G | -0.508 | 0.016 | 0.408 | 5.48E-207 | 7173 |
|  | rs113394924 | 11 | 62314821 | G | T | 0.187 | 0.030 | 0.087 | 3.52E-10 | 7173 |
|  | rs3018617 | 11 | 62435462 | G | A | -0.092 | 0.019 | 0.302 | 1.08E-06 | 7173 |
|  | rs10793123 | 11 | 75723045 | A | T | -0.077 | 0.017 | 0.485 | 5.11E-06 | 7173 |
|  | rs531117 | 11 | 75745089 | T | C | -0.114 | 0.020 | 0.232 | 9.96E-09 | 7173 |
|  | rs113913626 | 12 | 76255614 | T | C | -0.213 | 0.048 | 0.032 | 9.99E-06 | 7173 |
|  | rs73176681 | 13 | 31500828 | G | A | 0.184 | 0.040 | 0.046 | 5.15E-06 | 7173 |
|  | rs75976069 | 14 | 79220260 | C | T | -0.261 | 0.054 | 0.026 | 1.31E-06 | 7173 |
|  | rs61978272 | 14 | 94523321 | T | C | -0.098 | 0.019 | 0.282 | 1.41E-07 | 7173 |
|  | rs1077834 | 15 | 58431280 | C | T | 0.099 | 0.019 | 0.266 | 2.88E-07 | 7173 |
|  | rs147549994 | 16 | 1939264 | T | G | 0.232 | 0.045 | 0.036 | 3.16E-07 | 7173 |
|  | rs173539 | 16 | 56954132 | T | C | 0.085 | 0.018 | 0.283 | 4.26E-06 | 7173 |
|  | rs113049169 | 16 | 78573129 | A | C | 0.215 | 0.049 | 0.030 | 9.26E-06 | 7173 |
|  | rs147797702 | 18 | 52743009 | C | T | -0.489 | 0.089 | 0.009 | 4.03E-08 | 7173 |
|  | rs793864 | 19 | 7552489 | C | G | -0.102 | 0.022 | 0.818 | 2.95E-06 | 7173 |
|  | rs187429064 | 19 | 19269704 | G | A | -0.276 | 0.038 | 0.054 | 2.68E-13 | 7173 |
|  | rs8107974 | 19 | 19277691 | T | A | -0.191 | 0.034 | 0.062 | 3.00E-08 | 7173 |
|  | rs641738 | 19 | 54173068 | C | T | -0.085 | 0.017 | 0.593 | 5.94E-07 | 7173 |
|  | rs6083838 | 20 | 25397682 | G | A | -0.075 | 0.017 | 0.536 | 9.53E-06 | 7173 |
|  | rs73618588 | 20 | 36497804 | A | G | -0.130 | 0.029 | 0.093 | 6.01E-06 | 7173 |
| Phosphatidylcholine (18:2_18:2) | rs1168127 | 1 | 62670407 | A | C | 0.092 | 0.019 | 0.737 | 1.17E-06 | 7174 |
|  | rs148498852 | 2 | 204695970 | G | A | 0.157 | 0.033 | 0.077 | 1.73E-06 | 7174 |
|  | rs59256568 | 2 | 227805415 | G | A | -0.297 | 0.063 | 0.020 | 2.34E-06 | 7174 |
|  | rs62238391 | 3 | 10450834 | T | C | 0.086 | 0.018 | 0.320 | 1.38E-06 | 7174 |
|  | rs13078306 | 3 | 80053426 | C | A | -0.082 | 0.018 | 0.328 | 3.51E-06 | 7174 |
|  | rs56158036 | 3 | 95414411 | A | G | 0.296 | 0.064 | 0.017 | 3.58E-06 | 7174 |
|  | rs4271965 | 4 | 159948553 | G | A | 0.080 | 0.017 | 0.567 | 2.16E-06 | 7174 |
|  | rs13150924 | 4 | 169277659 | A | G | -0.112 | 0.025 | 0.130 | 8.25E-06 | 7174 |
|  | rs139598970 | 4 | 185838932 | T | G | 0.422 | 0.090 | 0.010 | 2.54E-06 | 7174 |
|  | rs7717591 | 5 | 52415905 | G | A | -0.096 | 0.018 | 0.679 | 8.41E-08 | 7174 |
|  | rs7798734 | 7 | 91299442 | T | G | -0.082 | 0.018 | 0.339 | 3.70E-06 | 7174 |
|  | rs35958132 | 7 | 120336033 | G | A | 0.158 | 0.034 | 0.070 | 4.47E-06 | 7174 |
|  | rs117017186 | 10 | 93203138 | T | C | 0.362 | 0.080 | 0.012 | 5.33E-06 | 7174 |
|  | rs174574 | 11 | 61832870 | C | A | -0.222 | 0.017 | 0.591 | 4.16E-39 | 7174 |
|  | rs7122803 | 11 | 107883610 | G | C | -0.108 | 0.024 | 0.860 | 6.32E-06 | 7174 |
|  | rs147002062 | 13 | 79574011 | T | C | -0.839 | 0.183 | 0.003 | 4.77E-06 | 7174 |
|  | rs1601935 | 15 | 58379566 | T | G | -0.094 | 0.017 | 0.611 | 3.75E-08 | 7174 |
|  | rs1077835 | 15 | 58431227 | G | A | 0.115 | 0.019 | 0.266 | 2.66E-09 | 7174 |
|  | rs6498540 | 16 | 15036737 | G | A | 0.093 | 0.018 | 0.334 | 1.44E-07 | 7174 |
|  | rs173539 | 16 | 56954132 | T | C | 0.098 | 0.018 | 0.283 | 1.01E-07 | 7174 |
|  | rs11869356 | 17 | 34718046 | T | C | -0.077 | 0.017 | 0.585 | 7.72E-06 | 7174 |
|  | rs117675848 | 20 | 46799183 | A | G | 0.321 | 0.065 | 0.018 | 9.54E-07 | 7174 |
|  | rs80315588 | 21 | 25877514 | A | G | -0.077 | 0.017 | 0.418 | 5.15E-06 | 7174 |
|  | rs2236605 | 21 | 37563977 | C | G | 0.076 | 0.017 | 0.432 | 6.19E-06 | 7174 |
| Phosphatidylcholine (18:2_20:1) | rs4622847 | 3 | 38782852 | G | A | -0.094 | 0.021 | 0.710 | 9.74E-06 | 5521 |
|  | rs56128146 | 3 | 87312021 | T | C | 0.089 | 0.019 | 0.423 | 3.25E-06 | 5521 |
|  | rs17484 | 3 | 116723503 | C | T | 0.091 | 0.020 | 0.615 | 3.72E-06 | 5521 |
|  | rs9994217 | 4 | 14321016 | A | G | 0.086 | 0.019 | 0.425 | 7.26E-06 | 5521 |
|  | rs78098253 | 4 | 74104898 | A | C | 0.266 | 0.056 | 0.030 | 2.53E-06 | 5521 |
|  | rs62315647 | 4 | 85577702 | C | T | 0.360 | 0.078 | 0.018 | 3.96E-06 | 5521 |
|  | rs11950850 | 5 | 33202141 | A | C | -0.093 | 0.021 | 0.290 | 9.19E-06 | 5521 |
|  | rs72795807 | 5 | 112580236 | T | C | -0.158 | 0.035 | 0.080 | 7.34E-06 | 5521 |
|  | rs9402974 | 6 | 138435656 | T | A | -0.131 | 0.028 | 0.154 | 2.55E-06 | 5521 |
|  | rs200921913 | 8 | 128749313 | T | C | -0.097 | 0.020 | 0.374 | 1.12E-06 | 5521 |
|  | rs10740329 | 10 | 70181497 | T | A | 0.085 | 0.019 | 0.439 | 8.42E-06 | 5521 |
|  | rs79136768 | 11 | 61594967 | G | A | 0.241 | 0.051 | 0.037 | 2.05E-06 | 5521 |
|  | rs182173873 | 11 | 61678244 | C | A | -0.242 | 0.045 | 0.049 | 9.15E-08 | 5521 |
|  | rs174599 | 11 | 61854084 | C | G | 0.323 | 0.019 | 0.419 | 3.92E-65 | 5521 |
|  | rs2302263 | 11 | 64254349 | T | C | 0.151 | 0.028 | 0.130 | 6.10E-08 | 5521 |
|  | rs72956488 | 11 | 97015272 | A | G | 0.277 | 0.061 | 0.030 | 5.44E-06 | 5521 |
|  | rs117210128 | 12 | 31290266 | T | C | 0.254 | 0.057 | 0.030 | 7.49E-06 | 5521 |
|  | rs211991 | 17 | 36639734 | T | C | -0.103 | 0.023 | 0.261 | 6.83E-06 | 5521 |
|  | rs4147976 | 17 | 68929782 | T | C | -0.094 | 0.019 | 0.550 | 1.28E-06 | 5521 |
| Phosphatidylcholine (18:2_20:3) | rs11208004 | 1 | 62679768 | A | G | -0.092 | 0.020 | 0.262 | 3.11E-06 | 6615 |
|  | rs60417255 | 1 | 87589957 | T | G | 0.164 | 0.037 | 0.056 | 9.89E-06 | 6615 |
|  | rs2489189 | 1 | 91866179 | A | T | -0.158 | 0.035 | 0.064 | 7.09E-06 | 6615 |
|  | rs10779836 | 1 | 230167404 | T | C | 0.104 | 0.021 | 0.777 | 6.56E-07 | 6615 |
|  | rs75662719 | 3 | 23041410 | G | A | -0.200 | 0.045 | 0.038 | 8.25E-06 | 6615 |
|  | rs2347672 | 4 | 43914879 | C | A | -0.093 | 0.018 | 0.674 | 4.46E-07 | 6615 |
|  | rs138827081 | 5 | 99176070 | A | G | -0.583 | 0.122 | 0.005 | 1.97E-06 | 6615 |
|  | rs11757990 | 6 | 29796264 | T | C | 0.471 | 0.105 | 0.007 | 7.54E-06 | 6615 |
|  | rs146084531 | 6 | 98413921 | C | G | -0.277 | 0.061 | 0.021 | 5.82E-06 | 6615 |
|  | rs76833802 | 7 | 155488156 | T | C | -0.154 | 0.033 | 0.084 | 2.58E-06 | 6615 |
|  | rs35480480 | 10 | 58741730 | C | T | -0.131 | 0.029 | 0.101 | 8.14E-06 | 6615 |
|  | rs117663514 | 11 | 15133256 | A | C | -0.337 | 0.075 | 0.014 | 7.48E-06 | 6615 |
|  | rs174548 | 11 | 61803876 | G | C | 0.210 | 0.018 | 0.384 | 1.40E-31 | 6615 |
|  | rs10896384 | 11 | 68952542 | A | G | 0.088 | 0.018 | 0.382 | 9.23E-07 | 6615 |
|  | rs695112 | 11 | 75745535 | T | A | -0.109 | 0.020 | 0.241 | 9.51E-08 | 6615 |
|  | rs16916055 | 11 | 91426863 | C | T | 0.425 | 0.094 | 0.009 | 6.42E-06 | 6615 |
|  | rs4130016 | 11 | 133528221 | G | A | 0.108 | 0.024 | 0.156 | 7.13E-06 | 6615 |
|  | rs75719904 | 12 | 26133418 | G | A | 0.209 | 0.044 | 0.043 | 2.21E-06 | 6615 |
|  | rs10774859 | 12 | 116425661 | T | G | 0.078 | 0.017 | 0.531 | 6.74E-06 | 6615 |
|  | rs2096994 | 18 | 52821119 | A | C | -0.105 | 0.021 | 0.232 | 4.59E-07 | 6615 |
|  | rs150057262 | 19 | 19210016 | G | C | -0.204 | 0.041 | 0.052 | 5.10E-07 | 6615 |
| Phosphatidylcholine (18:2_20:4) | rs2419125 | 1 | 168675568 | A | G | -0.082 | 0.018 | 0.704 | 8.59E-06 | 7049 |
|  | rs114076093 | 1 | 202937186 | T | C | -0.307 | 0.069 | 0.016 | 8.05E-06 | 7049 |
|  | rs72830074 | 2 | 68091692 | T | G | -0.181 | 0.039 | 0.051 | 3.53E-06 | 7049 |
|  | rs10446372 | 3 | 76875178 | C | T | -0.077 | 0.017 | 0.437 | 6.17E-06 | 7049 |
|  | rs34689885 | 3 | 138128558 | T | A | 0.096 | 0.019 | 0.279 | 3.76E-07 | 7049 |
|  | rs77985607 | 4 | 7394662 | A | G | -0.173 | 0.038 | 0.056 | 4.18E-06 | 7049 |
|  | rs2973222 | 4 | 37584805 | T | C | 0.079 | 0.017 | 0.506 | 3.39E-06 | 7049 |
|  | rs4695858 | 4 | 173581856 | G | A | 0.217 | 0.049 | 0.032 | 9.96E-06 | 7049 |
|  | rs117919959 | 6 | 104035109 | C | G | -0.358 | 0.080 | 0.012 | 8.83E-06 | 7049 |
|  | rs117352906 | 7 | 18606087 | T | C | 0.432 | 0.093 | 0.009 | 3.47E-06 | 7049 |
|  | rs62482552 | 7 | 100924735 | A | G | -0.082 | 0.017 | 0.608 | 2.81E-06 | 7049 |
|  | rs139923737 | 7 | 153908913 | T | C | -0.304 | 0.057 | 0.023 | 1.24E-07 | 7049 |
|  | rs4237708 | 11 | 15423060 | A | G | -0.122 | 0.027 | 0.890 | 7.26E-06 | 7049 |
|  | rs76780983 | 11 | 43360418 | G | T | 0.235 | 0.049 | 0.030 | 2.11E-06 | 7049 |
|  | rs102274 | 11 | 61790354 | C | T | -0.174 | 0.017 | 0.409 | 2.66E-24 | 7049 |
|  | rs673335 | 11 | 75739531 | C | T | -0.097 | 0.020 | 0.245 | 7.43E-07 | 7049 |
|  | rs71425785 | 13 | 40081407 | G | A | -0.134 | 0.026 | 0.120 | 3.66E-07 | 7049 |
|  | rs1601935 | 15 | 58379566 | T | G | -0.082 | 0.017 | 0.611 | 2.32E-06 | 7049 |
|  | rs1077834 | 15 | 58431280 | C | T | 0.123 | 0.019 | 0.266 | 2.67E-10 | 7049 |
|  | rs1033155 | 15 | 89110533 | G | A | -0.097 | 0.017 | 0.398 | 2.50E-08 | 7049 |
|  | rs75528309 | 16 | 23549438 | T | C | 0.203 | 0.043 | 0.048 | 1.82E-06 | 7049 |
|  | rs173539 | 16 | 56954132 | T | C | 0.089 | 0.019 | 0.283 | 1.92E-06 | 7049 |
|  | rs79450037 | 16 | 81367012 | A | G | -0.221 | 0.045 | 0.039 | 7.72E-07 | 7049 |
|  | rs712265 | 17 | 18105133 | A | G | -0.074 | 0.017 | 0.502 | 9.65E-06 | 7049 |
|  | rs9889746 | 17 | 44595295 | C | T | 0.146 | 0.032 | 0.073 | 6.66E-06 | 7049 |
|  | rs55942656 | 19 | 8084803 | T | G | -0.077 | 0.017 | 0.440 | 6.88E-06 | 7049 |
|  | rs8100204 | 19 | 19282905 | A | G | -0.141 | 0.023 | 0.171 | 4.82E-10 | 7049 |
|  | rs248968 | 19 | 19760295 | C | T | 0.080 | 0.017 | 0.523 | 2.38E-06 | 7049 |
|  | rs836912 | 19 | 20102422 | T | C | -0.084 | 0.018 | 0.343 | 3.12E-06 | 7049 |
|  | rs8736 | 19 | 54173495 | C | T | -0.084 | 0.017 | 0.595 | 9.99E-07 | 7049 |
|  | rs8131052 | 21 | 18184728 | T | C | -0.140 | 0.029 | 0.094 | 1.25E-06 | 7049 |
| Phosphatidylcholine (O-16:0_16:0) | rs6675844 | 1 | 95674018 | A | T | -0.149 | 0.032 | 0.081 | 3.50E-06 | 6605 |
|  | rs6686021 | 1 | 155011063 | T | C | 0.080 | 0.018 | 0.400 | 7.97E-06 | 6605 |
|  | rs150651997 | 2 | 49428827 | C | T | 0.281 | 0.063 | 0.020 | 9.15E-06 | 6605 |
|  | rs148625143 | 2 | 227808434 | G | C | -0.206 | 0.044 | 0.043 | 3.54E-06 | 6605 |
|  | rs17586917 | 3 | 2923966 | A | G | -0.084 | 0.019 | 0.323 | 6.69E-06 | 6605 |
|  | rs115294982 | 4 | 144919778 | C | A | -0.223 | 0.046 | 0.037 | 1.08E-06 | 6605 |
|  | rs76494046 | 7 | 82989938 | C | A | -0.189 | 0.041 | 0.048 | 4.97E-06 | 6605 |
|  | rs80134854 | 7 | 97045603 | T | C | 0.253 | 0.057 | 0.026 | 9.65E-06 | 6605 |
|  | rs6987890 | 8 | 71111290 | G | A | 0.100 | 0.022 | 0.194 | 7.34E-06 | 6605 |
|  | rs72739734 | 9 | 81097537 | A | G | -0.292 | 0.064 | 0.019 | 5.02E-06 | 6605 |
|  | rs112728288 | 12 | 114634472 | C | T | 0.149 | 0.033 | 0.078 | 5.53E-06 | 6605 |
|  | rs17592511 | 13 | 67207825 | A | T | 0.390 | 0.086 | 0.011 | 5.43E-06 | 6605 |
|  | rs12440833 | 15 | 88328568 | G | A | -0.130 | 0.029 | 0.111 | 5.88E-06 | 6605 |
|  | rs7170186 | 15 | 94708362 | A | C | 0.083 | 0.018 | 0.670 | 6.71E-06 | 6605 |
|  | rs74591482 | 16 | 10794552 | C | G | -0.152 | 0.034 | 0.091 | 7.86E-06 | 6605 |
|  | rs17292238 | 17 | 1884243 | C | G | 0.151 | 0.034 | 0.077 | 8.61E-06 | 6605 |
|  | rs1160027 | 17 | 55740741 | T | G | 0.095 | 0.021 | 0.250 | 7.44E-06 | 6605 |
|  | rs75248478 | 17 | 82135397 | A | G | 0.238 | 0.048 | 0.036 | 7.88E-07 | 6605 |
|  | rs34115485 | 18 | 8859056 | G | A | 0.086 | 0.019 | 0.338 | 3.75E-06 | 6605 |
|  | rs117091735 | 19 | 42262816 | A | G | -0.704 | 0.155 | 0.004 | 5.89E-06 | 6605 |
| Phosphatidylcholine (O-16:0_16:1) | rs114541469 | 2 | 47577655 | C | G | 0.150 | 0.034 | 0.090 | 8.33E-06 | 5658 |
|  | rs13394815 | 2 | 70336870 | A | C | -0.094 | 0.019 | 0.389 | 1.29E-06 | 5658 |
|  | rs77494629 | 2 | 101572416 | A | C | 0.107 | 0.021 | 0.268 | 6.54E-07 | 5658 |
|  | rs2356187 | 2 | 158513491 | A | G | 0.126 | 0.028 | 0.869 | 6.54E-06 | 5658 |
|  | rs62311841 | 4 | 133202073 | A | G | -0.162 | 0.029 | 0.124 | 2.88E-08 | 5658 |
|  | rs6453462 | 5 | 79597004 | G | A | 0.194 | 0.039 | 0.934 | 6.61E-07 | 5658 |
|  | rs10248190 | 7 | 131005149 | A | T | 0.144 | 0.032 | 0.100 | 7.48E-06 | 5658 |
|  | rs16916332 | 8 | 51454913 | A | G | 0.210 | 0.046 | 0.044 | 6.24E-06 | 5658 |
|  | rs146085506 | 9 | 31384831 | C | T | 0.202 | 0.041 | 0.058 | 1.20E-06 | 5658 |
|  | rs4744927 | 9 | 69669802 | C | A | 0.953 | 0.212 | 0.998 | 7.11E-06 | 5658 |
|  | rs603424 | 10 | 100315722 | A | G | -0.143 | 0.029 | 0.121 | 1.10E-06 | 5658 |
|  | rs728516 | 11 | 45579256 | A | G | -0.085 | 0.019 | 0.526 | 5.97E-06 | 5658 |
|  | rs72973984 | 11 | 105208627 | G | A | -0.145 | 0.031 | 0.109 | 2.43E-06 | 5658 |
|  | rs117421874 | 13 | 62695542 | T | C | -0.521 | 0.110 | 0.007 | 2.36E-06 | 5658 |
|  | rs28609532 | 14 | 62141305 | G | A | -0.166 | 0.036 | 0.073 | 3.61E-06 | 5658 |
|  | rs1800588 | 15 | 58431476 | T | C | 0.110 | 0.022 | 0.258 | 4.86E-07 | 5658 |
|  | rs17231506 | 16 | 56960616 | T | C | 0.133 | 0.021 | 0.278 | 1.90E-10 | 5658 |
| Phosphatidylcholine (O-16:0_18:1) | rs606587 | 1 | 230180443 | G | A | -0.082 | 0.017 | 0.374 | 2.49E-06 | 6956 |
|  | rs876969 | 2 | 48551738 | T | C | -0.111 | 0.024 | 0.150 | 2.40E-06 | 6956 |
|  | rs9682490 | 3 | 4949091 | C | T | -0.080 | 0.017 | 0.435 | 3.82E-06 | 6956 |
|  | rs7677028 | 4 | 137022381 | G | C | 0.393 | 0.072 | 0.014 | 5.29E-08 | 6956 |
|  | rs2963775 | 5 | 115413799 | C | G | 0.079 | 0.018 | 0.581 | 9.87E-06 | 6956 |
|  | rs13194791 | 6 | 31307500 | G | A | 0.112 | 0.024 | 0.145 | 3.55E-06 | 6956 |
|  | rs7845596 | 8 | 2845634 | G | C | 0.154 | 0.032 | 0.077 | 1.59E-06 | 6956 |
|  | rs34861396 | 8 | 31640951 | A | G | 0.087 | 0.018 | 0.299 | 2.52E-06 | 6956 |
|  | rs138038919 | 8 | 118920650 | T | C | 0.089 | 0.020 | 0.248 | 7.10E-06 | 6956 |
|  | rs10120342 | 9 | 26919609 | G | T | 0.092 | 0.021 | 0.208 | 8.42E-06 | 6956 |
|  | rs77211823 | 11 | 12450600 | C | T | 0.189 | 0.041 | 0.049 | 4.83E-06 | 6956 |
|  | rs72923423 | 11 | 35044759 | T | G | -0.152 | 0.033 | 0.074 | 4.17E-06 | 6956 |
|  | rs11057853 | 12 | 124844767 | C | T | -0.077 | 0.017 | 0.454 | 6.20E-06 | 6956 |
|  | rs9538944 | 13 | 60820833 | A | C | 0.143 | 0.031 | 0.082 | 5.62E-06 | 6956 |
|  | rs1532085 | 15 | 58391167 | G | A | -0.098 | 0.017 | 0.573 | 9.47E-09 | 6956 |
|  | rs1800588 | 15 | 58431476 | T | C | 0.119 | 0.020 | 0.258 | 1.29E-09 | 6956 |
|  | rs17231506 | 16 | 56960616 | T | C | 0.129 | 0.019 | 0.278 | 6.16E-12 | 6956 |
|  | rs117449635 | 16 | 71063316 | A | G | 0.333 | 0.072 | 0.015 | 3.83E-06 | 6956 |
|  | rs79037957 | 16 | 72797212 | C | G | 0.219 | 0.048 | 0.035 | 4.45E-06 | 6956 |
|  | rs113746682 | 16 | 85344145 | T | C | 0.273 | 0.059 | 0.023 | 4.12E-06 | 6956 |
|  | rs73015021 | 19 | 11082239 | G | A | -0.137 | 0.029 | 0.092 | 2.92E-06 | 6956 |
|  | rs7260891 | 20 | 11603340 | A | G | -0.079 | 0.017 | 0.394 | 5.06E-06 | 6956 |
|  | rs6023985 | 20 | 55260213 | C | T | -0.326 | 0.073 | 0.014 | 8.23E-06 | 6956 |
| Phosphatidylcholine (O-16:0_18:2) | rs6741227 | 2 | 207017121 | T | C | 0.085 | 0.018 | 0.329 | 2.78E-06 | 7158 |
|  | rs11918522 | 3 | 112816210 | A | G | 0.106 | 0.023 | 0.159 | 4.10E-06 | 7158 |
|  | rs6764912 | 3 | 128653644 | A | G | -0.134 | 0.030 | 0.087 | 6.76E-06 | 7158 |
|  | rs11936543 | 4 | 151270229 | G | A | -0.126 | 0.028 | 0.100 | 8.95E-06 | 7158 |
|  | rs17086339 | 5 | 96599571 | C | G | -0.117 | 0.025 | 0.130 | 2.67E-06 | 7158 |
|  | rs4579316 | 6 | 75047845 | C | T | -0.108 | 0.022 | 0.834 | 1.66E-06 | 7158 |
|  | rs149918547 | 6 | 107204231 | A | G | 0.566 | 0.128 | 0.004 | 9.97E-06 | 7158 |
|  | rs79195215 | 7 | 10960253 | G | C | -0.237 | 0.054 | 0.026 | 9.81E-06 | 7158 |
|  | rs2537611 | 7 | 17693175 | T | C | 0.078 | 0.017 | 0.372 | 6.90E-06 | 7158 |
|  | rs75863117 | 7 | 69316302 | A | G | -0.132 | 0.030 | 0.096 | 8.85E-06 | 7158 |
|  | rs76888052 | 7 | 117677562 | T | C | 0.676 | 0.151 | 0.004 | 8.00E-06 | 7158 |
|  | rs11777584 | 8 | 22971029 | T | C | -0.077 | 0.017 | 0.345 | 9.95E-06 | 7158 |
|  | rs923920 | 9 | 14779673 | T | A | 0.077 | 0.017 | 0.531 | 5.10E-06 | 7158 |
|  | rs174574 | 11 | 61832870 | C | A | -0.181 | 0.017 | 0.591 | 1.55E-26 | 7158 |
|  | rs6582326 | 12 | 76023289 | T | A | -0.214 | 0.047 | 0.965 | 5.85E-06 | 7158 |
|  | rs1532085 | 15 | 58391167 | G | A | -0.086 | 0.017 | 0.573 | 3.40E-07 | 7158 |
|  | rs181750334 | 16 | 14645161 | T | A | -0.470 | 0.103 | 0.007 | 5.09E-06 | 7158 |
|  | rs17231506 | 16 | 56960616 | T | C | 0.122 | 0.019 | 0.278 | 6.06E-11 | 7158 |
|  | rs78285907 | 18 | 24042039 | T | A | 0.131 | 0.030 | 0.090 | 8.60E-06 | 7158 |
|  | rs75804573 | 20 | 44186250 | A | G | 0.109 | 0.024 | 0.150 | 4.52E-06 | 7158 |
|  | rs140661870 | 22 | 32427941 | T | C | -0.321 | 0.070 | 0.015 | 4.84E-06 | 7158 |
| Phosphatidylcholine (O-16:0_20:3) | rs1833350 | 2 | 71103693 | T | C | 0.091 | 0.020 | 0.252 | 7.62E-06 | 6709 |
|  | rs75111872 | 3 | 134061016 | A | G | -0.109 | 0.023 | 0.162 | 2.93E-06 | 6709 |
|  | rs10951096 | 7 | 3168526 | G | A | -0.087 | 0.020 | 0.725 | 8.90E-06 | 6709 |
|  | rs12671763 | 7 | 28483085 | A | C | 0.311 | 0.069 | 0.017 | 6.85E-06 | 6709 |
|  | rs968742 | 7 | 29412521 | G | A | 0.081 | 0.017 | 0.519 | 2.68E-06 | 6709 |
|  | rs62501991 | 8 | 15196227 | T | C | -0.101 | 0.022 | 0.180 | 7.35E-06 | 6709 |
|  | rs2360706 | 9 | 420203 | C | G | 0.091 | 0.020 | 0.766 | 7.69E-06 | 6709 |
|  | rs117435540 | 10 | 124011070 | A | G | 0.529 | 0.114 | 0.007 | 3.69E-06 | 6709 |
|  | rs968567 | 11 | 61828092 | T | C | 0.271 | 0.027 | 0.113 | 2.17E-23 | 6709 |
|  | rs75085081 | 11 | 118930336 | A | G | 0.238 | 0.053 | 0.028 | 8.47E-06 | 6709 |
|  | rs9552958 | 13 | 23426939 | T | C | -0.136 | 0.030 | 0.095 | 5.66E-06 | 6709 |
|  | rs79036443 | 13 | 52780240 | C | A | -0.197 | 0.043 | 0.042 | 5.65E-06 | 6709 |
|  | rs1800588 | 15 | 58431476 | T | C | 0.096 | 0.020 | 0.258 | 1.73E-06 | 6709 |
|  | rs6498540 | 16 | 15036737 | G | A | -0.154 | 0.018 | 0.334 | 5.73E-17 | 6709 |
|  | rs7412 | 19 | 44908822 | T | C | 0.225 | 0.038 | 0.053 | 4.52E-09 | 6709 |
|  | rs6015087 | 20 | 57822739 | C | T | 0.078 | 0.018 | 0.421 | 9.71E-06 | 6709 |
| Phosphatidylcholine (O-16:0_20:4) | rs77277881 | 1 | 19722845 | A | G | -0.498 | 0.106 | 0.007 | 2.89E-06 | 7174 |
|  | rs7554873 | 1 | 161642443 | C | T | 0.186 | 0.039 | 0.050 | 2.13E-06 | 7174 |
|  | rs115076819 | 1 | 245444784 | T | C | -0.222 | 0.050 | 0.030 | 9.02E-06 | 7174 |
|  | rs6709442 | 2 | 123584690 | A | G | 0.095 | 0.017 | 0.416 | 3.00E-08 | 7174 |
|  | rs2678932 | 2 | 180057085 | C | T | -0.081 | 0.017 | 0.468 | 1.08E-06 | 7174 |
|  | rs698909 | 2 | 205689820 | A | G | 0.082 | 0.018 | 0.686 | 6.00E-06 | 7174 |
|  | rs114653320 | 4 | 42283844 | G | A | 0.278 | 0.062 | 0.021 | 7.51E-06 | 7174 |
|  | rs114373179 | 4 | 66818143 | G | A | -0.425 | 0.096 | 0.008 | 9.46E-06 | 7174 |
|  | rs34881711 | 5 | 154465674 | A | G | 0.107 | 0.020 | 0.229 | 6.84E-08 | 7174 |
|  | rs10487877 | 7 | 80837985 | G | C | 0.177 | 0.040 | 0.047 | 8.71E-06 | 7174 |
|  | rs76798309 | 7 | 110642820 | A | G | 0.298 | 0.065 | 0.017 | 4.36E-06 | 7174 |
|  | rs6601694 | 8 | 8390548 | A | G | 0.083 | 0.018 | 0.334 | 3.04E-06 | 7174 |
|  | rs75385811 | 8 | 66145639 | G | A | -0.095 | 0.021 | 0.202 | 5.77E-06 | 7174 |
|  | rs73422593 | 9 | 21616162 | G | A | -0.130 | 0.027 | 0.112 | 1.68E-06 | 7174 |
|  | rs10867645 | 9 | 80921135 | T | C | -0.078 | 0.017 | 0.609 | 5.22E-06 | 7174 |
|  | rs658580 | 9 | 113637327 | A | C | 0.090 | 0.019 | 0.727 | 3.18E-06 | 7174 |
|  | rs642327 | 11 | 35494674 | G | C | -0.077 | 0.017 | 0.394 | 9.68E-06 | 7174 |
|  | rs3741252 | 11 | 61744026 | T | C | 0.139 | 0.025 | 0.134 | 1.70E-08 | 7174 |
|  | rs174568 | 11 | 61826344 | T | C | -0.382 | 0.016 | 0.405 | 1.16E-114 | 7174 |
|  | rs2903910 | 11 | 62267882 | A | T | 0.202 | 0.039 | 0.952 | 3.17E-07 | 7174 |
|  | rs146605342 | 11 | 103100479 | A | G | 0.197 | 0.044 | 0.037 | 8.18E-06 | 7174 |
|  | rs11173828 | 12 | 61209189 | A | G | -0.160 | 0.033 | 0.069 | 1.17E-06 | 7174 |
|  | rs11057853 | 12 | 124844767 | C | T | -0.088 | 0.017 | 0.454 | 1.69E-07 | 7174 |
|  | rs7982529 | 13 | 40337326 | C | T | 0.092 | 0.020 | 0.214 | 4.30E-06 | 7174 |
|  | rs79995028 | 14 | 41287117 | T | G | -0.635 | 0.132 | 0.004 | 1.62E-06 | 7174 |
|  | rs12928189 | 16 | 7546177 | G | A | -0.078 | 0.018 | 0.650 | 8.41E-06 | 7174 |
|  | rs173539 | 16 | 56954132 | T | C | 0.089 | 0.018 | 0.283 | 1.63E-06 | 7174 |
|  | rs145755646 | 17 | 59341291 | G | C | 0.184 | 0.034 | 0.065 | 7.23E-08 | 7174 |
|  | rs117215929 | 18 | 27258198 | C | T | -0.200 | 0.043 | 0.042 | 3.27E-06 | 7174 |
|  | rs2829725 | 21 | 25391715 | T | A | 0.081 | 0.018 | 0.314 | 7.18E-06 | 7174 |
| Phosphatidylcholine (O-16:0_22:5) | rs55709923 | 1 | 16964634 | G | C | -0.123 | 0.026 | 0.144 | 3.10E-06 | 5988 |
|  | rs187264161 | 3 | 68831568 | T | G | 0.340 | 0.076 | 0.017 | 7.55E-06 | 5988 |
|  | rs80261753 | 4 | 27344507 | A | G | -0.340 | 0.075 | 0.017 | 5.12E-06 | 5988 |
|  | rs145866244 | 5 | 76222760 | A | T | -0.251 | 0.052 | 0.031 | 1.60E-06 | 5988 |
|  | rs140792636 | 6 | 45929408 | A | C | -0.673 | 0.143 | 0.004 | 2.47E-06 | 5988 |
|  | rs9458511 | 6 | 162315657 | G | A | -0.121 | 0.027 | 0.127 | 9.41E-06 | 5988 |
|  | rs79318457 | 9 | 5604987 | T | C | 0.186 | 0.041 | 0.054 | 5.82E-06 | 5988 |
|  | rs150885037 | 10 | 131373745 | T | C | -0.232 | 0.051 | 0.033 | 5.08E-06 | 5988 |
|  | rs174548 | 11 | 61803876 | G | C | -0.168 | 0.019 | 0.384 | 7.57E-19 | 5988 |
|  | rs7103356 | 11 | 134357507 | G | A | 0.088 | 0.019 | 0.644 | 4.83E-06 | 5988 |
|  | rs56358871 | 15 | 58387188 | C | G | -0.087 | 0.018 | 0.492 | 1.53E-06 | 5988 |
|  | rs142015595 | 16 | 83721684 | A | T | -0.294 | 0.066 | 0.022 | 8.76E-06 | 5988 |
|  | rs79157443 | 18 | 76150476 | T | C | 0.168 | 0.038 | 0.062 | 8.43E-06 | 5988 |
|  | rs10414438 | 19 | 55193090 | G | A | -0.107 | 0.023 | 0.195 | 4.98E-06 | 5988 |
|  | rs4815298 | 20 | 2539461 | G | A | -0.084 | 0.018 | 0.536 | 4.88E-06 | 5988 |
|  | rs118036933 | 20 | 18282575 | T | G | -0.575 | 0.116 | 0.006 | 6.95E-07 | 5988 |
|  | rs184099437 | 22 | 43089231 | G | C | -0.244 | 0.051 | 0.035 | 2.24E-06 | 5988 |
| Phosphatidylcholine (O-16:1_16:0) | rs74843639 | 2 | 178137573 | G | T | -0.241 | 0.054 | 0.030 | 8.02E-06 | 5868 |
|  | rs79425159 | 3 | 178540293 | C | T | -0.218 | 0.047 | 0.039 | 4.26E-06 | 5868 |
|  | rs13104164 | 4 | 160356985 | T | A | -0.124 | 0.027 | 0.130 | 5.35E-06 | 5868 |
|  | rs1023679 | 5 | 144386603 | G | A | 0.102 | 0.021 | 0.751 | 2.25E-06 | 5868 |
|  | rs670210 | 6 | 20072349 | T | C | -0.089 | 0.020 | 0.668 | 7.72E-06 | 5868 |
|  | rs73125516 | 7 | 39384981 | C | G | 0.104 | 0.023 | 0.202 | 5.05E-06 | 5868 |
|  | rs75066420 | 9 | 24151529 | A | G | 0.185 | 0.041 | 0.056 | 6.65E-06 | 5868 |
|  | rs2740488 | 9 | 104899461 | C | A | -0.108 | 0.024 | 0.185 | 5.18E-06 | 5868 |
|  | rs1247489 | 10 | 76243822 | G | T | 0.093 | 0.019 | 0.549 | 4.76E-07 | 5868 |
|  | rs34160235 | 10 | 133100099 | T | C | 0.272 | 0.060 | 0.025 | 6.76E-06 | 5868 |
|  | rs79367190 | 12 | 69475924 | T | C | 0.217 | 0.046 | 0.041 | 1.85E-06 | 5868 |
|  | rs78682716 | 12 | 95184329 | A | G | 0.465 | 0.093 | 0.010 | 5.82E-07 | 5868 |
|  | rs77351278 | 13 | 36635371 | C | T | 0.306 | 0.068 | 0.021 | 6.71E-06 | 5868 |
|  | rs1077989 | 14 | 67509105 | C | A | -0.183 | 0.018 | 0.468 | 2.91E-23 | 5868 |
|  | rs71423392 | 14 | 70230276 | C | G | -0.200 | 0.043 | 0.053 | 4.18E-06 | 5868 |
|  | rs76014869 | 15 | 40795879 | A | G | 0.423 | 0.093 | 0.011 | 5.88E-06 | 5868 |
|  | rs11858902 | 15 | 88324326 | C | A | -0.097 | 0.021 | 0.304 | 4.76E-06 | 5868 |
|  | rs72942772 | 18 | 9003268 | C | T | -0.107 | 0.023 | 0.195 | 5.25E-06 | 5868 |
| Phosphatidylcholine (O-16:1_18:0) | rs115012576 | 3 | 150051286 | G | A | -0.507 | 0.114 | 0.006 | 8.66E-06 | 6751 |
|  | rs9997698 | 4 | 141036586 | C | T | -0.177 | 0.038 | 0.056 | 2.73E-06 | 6751 |
|  | rs74997551 | 6 | 109308440 | C | T | -0.131 | 0.029 | 0.097 | 5.75E-06 | 6751 |
|  | rs143594930 | 8 | 26457702 | T | C | -0.402 | 0.091 | 0.010 | 9.56E-06 | 6751 |
|  | rs1397106 | 8 | 77676794 | G | T | -0.078 | 0.017 | 0.411 | 6.65E-06 | 6751 |
|  | rs11187130 | 10 | 80809816 | T | C | -0.452 | 0.094 | 0.009 | 1.78E-06 | 6751 |
|  | rs1093316 | 10 | 94349017 | C | T | -0.147 | 0.029 | 0.098 | 4.68E-07 | 6751 |
|  | rs4565878 | 11 | 3204472 | A | G | -0.087 | 0.019 | 0.259 | 8.27E-06 | 6751 |
|  | rs12270739 | 11 | 36410769 | A | G | -0.123 | 0.026 | 0.139 | 1.60E-06 | 6751 |
|  | rs77512406 | 11 | 66971292 | T | C | -0.134 | 0.026 | 0.130 | 2.65E-07 | 6751 |
|  | rs181154147 | 12 | 69626170 | T | C | 0.221 | 0.049 | 0.032 | 7.02E-06 | 6751 |
|  | rs116883951 | 13 | 102563170 | A | G | 0.439 | 0.098 | 0.008 | 8.38E-06 | 6751 |
|  | rs1440391 | 14 | 48425602 | A | G | 0.080 | 0.018 | 0.630 | 9.17E-06 | 6751 |
|  | rs1667515 | 14 | 85227560 | A | G | -0.087 | 0.019 | 0.701 | 3.27E-06 | 6751 |
|  | rs1355105 | 17 | 53585624 | C | T | 0.081 | 0.018 | 0.595 | 4.95E-06 | 6751 |
|  | rs1941562 | 18 | 41193890 | G | A | -0.086 | 0.019 | 0.282 | 6.51E-06 | 6751 |
|  | rs448313 | 22 | 22014247 | C | T | 0.172 | 0.034 | 0.082 | 6.67E-07 | 6751 |
| Phosphatidylcholine (O-16:1_18:1) | rs6672758 | 1 | 230167766 | T | C | 0.119 | 0.019 | 0.742 | 3.78E-10 | 7154 |
|  | rs1441162 | 2 | 181590050 | T | G | -0.126 | 0.026 | 0.120 | 8.95E-07 | 7154 |
|  | rs116614673 | 3 | 30168901 | A | G | -0.126 | 0.028 | 0.096 | 9.54E-06 | 7154 |
|  | rs150860836 | 3 | 73177331 | G | A | -0.204 | 0.045 | 0.038 | 5.22E-06 | 7154 |
|  | rs7677028 | 4 | 137022381 | G | C | 0.328 | 0.071 | 0.014 | 3.28E-06 | 7154 |
|  | rs116438463 | 4 | 150864359 | A | T | -0.179 | 0.036 | 0.057 | 7.28E-07 | 7154 |
|  | rs117331519 | 7 | 44312910 | T | G | -0.428 | 0.095 | 0.008 | 6.37E-06 | 7154 |
|  | rs35293298 | 7 | 104770675 | G | A | -0.105 | 0.023 | 0.150 | 7.35E-06 | 7154 |
|  | rs73162481 | 7 | 137203256 | C | T | 0.109 | 0.023 | 0.164 | 1.64E-06 | 7154 |
|  | rs74629446 | 9 | 70909853 | T | A | -0.453 | 0.101 | 0.007 | 8.26E-06 | 7154 |
|  | rs3012735 | 9 | 131776174 | A | G | 0.077 | 0.017 | 0.398 | 5.58E-06 | 7154 |
|  | rs34040476 | 10 | 110932051 | T | C | 0.193 | 0.043 | 0.043 | 6.59E-06 | 7154 |
|  | rs158633 | 11 | 30952568 | A | C | 0.132 | 0.027 | 0.112 | 6.58E-07 | 7154 |
|  | rs964184 | 11 | 116778201 | C | G | 0.153 | 0.023 | 0.849 | 4.01E-11 | 7154 |
|  | rs117722620 | 11 | 131747965 | A | G | 0.274 | 0.056 | 0.025 | 9.31E-07 | 7154 |
|  | rs12426108 | 12 | 68489126 | C | T | -0.156 | 0.030 | 0.083 | 3.18E-07 | 7154 |
|  | rs60426605 | 12 | 129441623 | G | A | 0.250 | 0.056 | 0.023 | 8.77E-06 | 7154 |
|  | rs116948429 | 14 | 45836784 | T | G | -0.208 | 0.046 | 0.034 | 6.36E-06 | 7154 |
|  | rs116943761 | 14 | 67507335 | A | C | -0.160 | 0.031 | 0.082 | 1.74E-07 | 7154 |
|  | rs143615441 | 14 | 95480964 | C | A | -0.443 | 0.090 | 0.010 | 7.69E-07 | 7154 |
|  | rs1532085 | 15 | 58391167 | G | A | -0.095 | 0.017 | 0.573 | 1.60E-08 | 7154 |
|  | rs1800588 | 15 | 58431476 | T | C | 0.089 | 0.019 | 0.258 | 4.79E-06 | 7154 |
|  | rs17231506 | 16 | 56960616 | T | C | 0.094 | 0.019 | 0.278 | 3.84E-07 | 7154 |
|  | rs7190500 | 16 | 76864528 | C | A | -0.098 | 0.022 | 0.814 | 5.62E-06 | 7154 |
|  | rs62087966 | 18 | 369878 | G | A | 0.486 | 0.105 | 0.007 | 3.47E-06 | 7154 |
| Phosphatidylcholine (O-16:1_18:2) | rs627108 | 1 | 230174822 | T | C | -0.086 | 0.017 | 0.396 | 5.20E-07 | 7096 |
|  | rs114517755 | 2 | 181779530 | C | A | 0.653 | 0.129 | 0.005 | 4.31E-07 | 7096 |
|  | rs13403377 | 2 | 205953957 | T | C | 0.085 | 0.018 | 0.356 | 1.36E-06 | 7096 |
|  | rs143533648 | 2 | 214577935 | T | C | 0.252 | 0.054 | 0.026 | 2.71E-06 | 7096 |
|  | rs11918522 | 3 | 112816210 | A | G | 0.109 | 0.023 | 0.159 | 2.08E-06 | 7096 |
|  | rs2670896 | 3 | 129984722 | A | G | -0.234 | 0.050 | 0.030 | 2.76E-06 | 7096 |
|  | rs138542536 | 4 | 25796122 | C | A | -0.357 | 0.079 | 0.013 | 6.61E-06 | 7096 |
|  | rs61999264 | 5 | 149246504 | A | G | -0.344 | 0.075 | 0.013 | 3.86E-06 | 7096 |
|  | rs17194174 | 6 | 30094133 | A | G | 0.165 | 0.036 | 0.059 | 5.74E-06 | 7096 |
|  | rs2977197 | 8 | 1742309 | C | G | 0.144 | 0.031 | 0.918 | 3.75E-06 | 7096 |
|  | rs151236898 | 8 | 74189562 | C | G | 0.266 | 0.058 | 0.021 | 5.16E-06 | 7096 |
|  | rs76103105 | 10 | 4699577 | A | G | -0.212 | 0.046 | 0.036 | 4.23E-06 | 7096 |
|  | rs4237708 | 11 | 15423060 | A | G | -0.126 | 0.027 | 0.890 | 3.02E-06 | 7096 |
|  | rs9666651 | 11 | 34810889 | T | C | 0.085 | 0.019 | 0.263 | 9.86E-06 | 7096 |
|  | rs180360 | 11 | 116728272 | G | A | 0.086 | 0.018 | 0.324 | 1.69E-06 | 7096 |
|  | rs77172221 | 12 | 23079509 | C | T | -0.137 | 0.030 | 0.090 | 4.53E-06 | 7096 |
|  | rs117002628 | 12 | 46480289 | A | G | 0.511 | 0.111 | 0.006 | 4.41E-06 | 7096 |
|  | rs12426108 | 12 | 68489126 | C | T | -0.148 | 0.031 | 0.083 | 1.52E-06 | 7096 |
|  | rs10859677 | 12 | 94142171 | G | A | -0.081 | 0.018 | 0.605 | 5.27E-06 | 7096 |
|  | rs186412131 | 12 | 124792520 | A | G | -0.207 | 0.046 | 0.036 | 6.79E-06 | 7096 |
|  | rs1532085 | 15 | 58391167 | G | A | -0.115 | 0.017 | 0.573 | 8.88E-12 | 7096 |
|  | rs1077834 | 15 | 58431280 | C | T | 0.100 | 0.019 | 0.266 | 2.10E-07 | 7096 |
|  | rs17231506 | 16 | 56960616 | T | C | 0.128 | 0.019 | 0.278 | 5.42E-12 | 7096 |
|  | rs139013784 | 17 | 50256987 | A | G | -0.361 | 0.081 | 0.012 | 9.33E-06 | 7096 |
|  | rs62087966 | 18 | 369878 | G | A | 0.499 | 0.105 | 0.007 | 1.89E-06 | 7096 |
|  | rs77099066 | 21 | 45199268 | A | G | -0.580 | 0.131 | 0.005 | 9.25E-06 | 7096 |
| Phosphatidylcholine (O-16:1_20:3) | rs1203148 | 1 | 35503298 | G | A | 0.093 | 0.021 | 0.240 | 8.17E-06 | 6770 |
|  | rs686843 | 1 | 60908464 | A | G | 0.082 | 0.018 | 0.480 | 3.83E-06 | 6770 |
|  | rs11883812 | 2 | 215392258 | C | T | -0.220 | 0.041 | 0.049 | 6.86E-08 | 6770 |
|  | rs9872756 | 3 | 87569181 | A | G | 0.099 | 0.020 | 0.749 | 6.28E-07 | 6770 |
|  | rs9880183 | 3 | 188687875 | C | T | -0.078 | 0.018 | 0.390 | 9.86E-06 | 6770 |
|  | rs3795169 | 4 | 37858054 | T | C | 0.119 | 0.025 | 0.144 | 1.51E-06 | 6770 |
|  | rs6817929 | 4 | 56204082 | G | A | 0.092 | 0.018 | 0.588 | 1.90E-07 | 6770 |
|  | rs6815866 | 4 | 95119717 | C | G | -0.134 | 0.027 | 0.891 | 1.03E-06 | 6770 |
|  | rs58105955 | 7 | 74470522 | G | A | 0.134 | 0.029 | 0.095 | 6.06E-06 | 6770 |
|  | rs254868 | 7 | 112024749 | G | T | -0.102 | 0.023 | 0.172 | 8.91E-06 | 6770 |
|  | rs34455876 | 7 | 130154739 | A | G | -0.087 | 0.019 | 0.313 | 3.40E-06 | 6770 |
|  | rs7874842 | 9 | 16476973 | A | G | 0.099 | 0.022 | 0.190 | 5.08E-06 | 6770 |
|  | rs61896141 | 11 | 61788567 | C | A | 0.220 | 0.027 | 0.113 | 3.61E-16 | 6770 |
|  | rs55665216 | 12 | 7036085 | T | C | 0.152 | 0.032 | 0.080 | 1.59E-06 | 6770 |
|  | rs35556451 | 12 | 28686870 | T | C | -0.226 | 0.047 | 0.036 | 1.28E-06 | 6770 |
|  | rs80320850 | 12 | 61219406 | A | G | -0.179 | 0.039 | 0.052 | 3.99E-06 | 6770 |
|  | rs112516243 | 13 | 27037828 | G | T | 0.759 | 0.170 | 0.003 | 8.27E-06 | 6770 |
|  | rs9602749 | 13 | 85434789 | G | T | 0.114 | 0.025 | 0.147 | 3.30E-06 | 6770 |
|  | rs56142013 | 14 | 56431229 | T | C | -0.349 | 0.076 | 0.014 | 4.31E-06 | 6770 |
|  | rs1077989 | 14 | 67509105 | C | A | -0.121 | 0.017 | 0.468 | 1.70E-12 | 6770 |
|  | rs8033940 | 15 | 58432643 | A | G | 0.089 | 0.019 | 0.337 | 1.62E-06 | 6770 |
|  | rs6498540 | 16 | 15036737 | G | A | -0.147 | 0.018 | 0.334 | 8.85E-16 | 6770 |
|  | rs144750125 | 17 | 15541920 | T | C | 0.259 | 0.055 | 0.024 | 3.17E-06 | 6770 |
|  | rs28399664 | 19 | 44821499 | G | C | -0.214 | 0.046 | 0.038 | 3.68E-06 | 6770 |
|  | rs1800961 | 20 | 44413724 | T | C | -0.174 | 0.038 | 0.052 | 6.40E-06 | 6770 |
| Phosphatidylcholine (O-16:1_20:4) | rs114325590 | 1 | 107442367 | T | C | -0.458 | 0.091 | 0.011 | 4.70E-07 | 6025 |
|  | rs17268903 | 2 | 56518805 | G | A | -0.126 | 0.028 | 0.121 | 8.32E-06 | 6025 |
|  | rs56303140 | 2 | 157798940 | A | G | 0.233 | 0.051 | 0.035 | 4.65E-06 | 6025 |
|  | rs6752137 | 2 | 181604439 | A | G | -0.129 | 0.028 | 0.120 | 3.46E-06 | 6025 |
|  | rs5028238 | 2 | 217342965 | A | G | -0.096 | 0.022 | 0.765 | 7.68E-06 | 6025 |
|  | rs138018897 | 3 | 19181916 | G | A | -0.299 | 0.065 | 0.022 | 4.74E-06 | 6025 |
|  | rs6776300 | 3 | 153579259 | A | C | 0.082 | 0.018 | 0.534 | 8.20E-06 | 6025 |
|  | rs10487877 | 7 | 80837985 | G | C | 0.209 | 0.043 | 0.047 | 1.29E-06 | 6025 |
|  | rs74854572 | 8 | 9367590 | G | C | -0.394 | 0.087 | 0.012 | 6.39E-06 | 6025 |
|  | rs174527 | 11 | 61770929 | C | G | -0.121 | 0.019 | 0.444 | 7.84E-11 | 6025 |
|  | rs174535 | 11 | 61783884 | C | T | -0.404 | 0.018 | 0.409 | 1.18E-107 | 6025 |
|  | rs2015950 | 11 | 62058058 | A | G | -0.134 | 0.027 | 0.137 | 5.32E-07 | 6025 |
|  | rs180360 | 11 | 116728272 | G | A | 0.088 | 0.019 | 0.324 | 6.34E-06 | 6025 |
|  | rs12579775 | 12 | 6976009 | A | G | 0.160 | 0.033 | 0.082 | 1.05E-06 | 6025 |
|  | rs71452092 | 12 | 28633010 | T | C | -0.222 | 0.050 | 0.036 | 8.89E-06 | 6025 |
|  | rs7963111 | 12 | 124935624 | T | G | -0.193 | 0.043 | 0.049 | 5.86E-06 | 6025 |
|  | rs1316133 | 14 | 67471921 | A | G | -0.090 | 0.020 | 0.299 | 8.71E-06 | 6025 |
|  | rs1077989 | 14 | 67509105 | C | A | -0.192 | 0.018 | 0.468 | 3.43E-26 | 6025 |
|  | rs1037782 | 15 | 29614744 | C | T | -0.173 | 0.038 | 0.061 | 5.26E-06 | 6025 |
|  | rs2043085 | 15 | 58388755 | C | T | -0.100 | 0.018 | 0.576 | 4.75E-08 | 6025 |
|  | rs12920974 | 16 | 56959113 | T | G | -0.104 | 0.020 | 0.330 | 1.16E-07 | 6025 |
|  | rs71366199 | 17 | 15393870 | G | A | 0.118 | 0.026 | 0.148 | 6.50E-06 | 6025 |
|  | rs2431867 | 19 | 7570522 | A | G | 0.105 | 0.023 | 0.199 | 7.79E-06 | 6025 |
|  | rs73531625 | 19 | 18613744 | T | C | -0.091 | 0.020 | 0.289 | 4.83E-06 | 6025 |
|  | rs79740876 | 20 | 1463662 | A | G | 0.197 | 0.043 | 0.048 | 4.27E-06 | 6025 |
| Phosphatidylcholine (O-16:2_18:0) | rs141656454 | 1 | 82430121 | A | G | 0.392 | 0.088 | 0.010 | 8.63E-06 | 6672 |
|  | rs13433708 | 3 | 4383597 | T | A | -0.094 | 0.019 | 0.329 | 5.61E-07 | 6672 |
|  | rs1145605 | 5 | 158277324 | G | A | -0.136 | 0.028 | 0.116 | 1.18E-06 | 6672 |
|  | rs184729252 | 6 | 24525754 | A | G | 0.836 | 0.176 | 0.003 | 2.14E-06 | 6672 |
|  | rs6455474 | 6 | 167990307 | C | G | 0.299 | 0.065 | 0.018 | 4.54E-06 | 6672 |
|  | rs61703937 | 8 | 76625718 | A | G | -0.143 | 0.031 | 0.088 | 4.26E-06 | 6672 |
|  | rs10869152 | 9 | 68622132 | G | A | 0.082 | 0.018 | 0.342 | 8.30E-06 | 6672 |
|  | rs3814533 | 9 | 81637892 | T | C | -0.151 | 0.032 | 0.915 | 1.77E-06 | 6672 |
|  | rs35807440 | 9 | 131354630 | G | A | 0.085 | 0.018 | 0.348 | 4.04E-06 | 6672 |
|  | rs74684016 | 12 | 118561421 | G | A | 0.100 | 0.022 | 0.192 | 5.96E-06 | 6672 |
|  | rs55816615 | 13 | 41576909 | A | G | 0.093 | 0.021 | 0.238 | 7.22E-06 | 6672 |
|  | rs1532085 | 15 | 58391167 | G | A | -0.174 | 0.017 | 0.573 | 1.26E-23 | 6672 |
|  | rs588136 | 15 | 58438299 | T | C | -0.191 | 0.020 | 0.758 | 4.89E-21 | 6672 |
| Phosphatidylcholine (O-17:0_15:0) | rs4949186 | 1 | 30929301 | C | T | -0.097 | 0.020 | 0.700 | 7.41E-07 | 6140 |
|  | rs145150348 | 1 | 62121729 | A | G | 0.337 | 0.069 | 0.017 | 1.05E-06 | 6140 |
|  | rs144941484 | 2 | 158921003 | T | C | 0.592 | 0.122 | 0.006 | 1.28E-06 | 6140 |
|  | rs149897605 | 2 | 197373815 | C | T | -0.282 | 0.062 | 0.024 | 5.62E-06 | 6140 |
|  | rs150669352 | 2 | 238794542 | T | C | -0.504 | 0.110 | 0.008 | 4.34E-06 | 6140 |
|  | rs76913081 | 3 | 104655275 | T | G | -0.171 | 0.038 | 0.061 | 6.58E-06 | 6140 |
|  | rs74648736 | 3 | 173729514 | T | A | -0.328 | 0.072 | 0.022 | 4.79E-06 | 6140 |
|  | rs748301 | 6 | 23380540 | G | A | -0.084 | 0.019 | 0.608 | 7.53E-06 | 6140 |
|  | rs10225730 | 7 | 17648098 | T | G | -0.238 | 0.054 | 0.030 | 9.76E-06 | 6140 |
|  | rs417916 | 7 | 41274867 | T | C | -0.088 | 0.019 | 0.440 | 3.09E-06 | 6140 |
|  | rs146967253 | 7 | 81169417 | T | C | 0.285 | 0.060 | 0.026 | 2.05E-06 | 6140 |
|  | rs72967793 | 11 | 95940105 | T | A | -0.180 | 0.039 | 0.060 | 2.99E-06 | 6140 |
|  | rs141417792 | 11 | 114838676 | G | T | 0.266 | 0.060 | 0.024 | 8.26E-06 | 6140 |
|  | rs76292244 | 12 | 42408584 | C | A | -0.261 | 0.057 | 0.027 | 5.52E-06 | 6140 |
|  | rs112686420 | 13 | 42902737 | G | A | 0.470 | 0.099 | 0.009 | 2.02E-06 | 6140 |
|  | rs252277 | 16 | 29082111 | G | A | 0.082 | 0.018 | 0.424 | 7.53E-06 | 6140 |
|  | rs116984749 | 16 | 48346132 | G | A | 0.243 | 0.055 | 0.027 | 9.83E-06 | 6140 |
|  | rs7204112 | 16 | 86938606 | G | A | 0.238 | 0.053 | 0.032 | 6.35E-06 | 6140 |
|  | rs139939351 | 22 | 49966644 | G | T | 0.739 | 0.160 | 0.004 | 3.64E-06 | 6140 |
| Phosphatidylcholine (O-17:0_17:1) | rs115823613 | 1 | 109494012 | T | C | 0.146 | 0.033 | 0.072 | 8.99E-06 | 6880 |
|  | rs612577 | 1 | 230188618 | C | T | -0.114 | 0.022 | 0.180 | 2.01E-07 | 6880 |
|  | rs11691441 | 2 | 100856837 | C | G | -0.183 | 0.040 | 0.047 | 4.67E-06 | 6880 |
|  | rs7575011 | 2 | 113675313 | G | T | -0.082 | 0.018 | 0.662 | 4.63E-06 | 6880 |
|  | rs7597998 | 2 | 192167629 | C | T | 0.090 | 0.020 | 0.765 | 7.99E-06 | 6880 |
|  | rs72975671 | 3 | 135812971 | T | A | 0.095 | 0.021 | 0.215 | 5.24E-06 | 6880 |
|  | rs11947730 | 4 | 6203208 | C | T | 0.093 | 0.020 | 0.737 | 5.19E-06 | 6880 |
|  | rs17611110 | 4 | 168219402 | C | T | -0.100 | 0.020 | 0.241 | 5.86E-07 | 6880 |
|  | rs12186559 | 5 | 3950508 | T | C | 0.227 | 0.047 | 0.036 | 1.19E-06 | 6880 |
|  | rs3765259 | 6 | 136669145 | C | T | -0.079 | 0.018 | 0.427 | 9.73E-06 | 6880 |
|  | rs112832771 | 6 | 151101373 | G | A | 0.359 | 0.080 | 0.012 | 6.61E-06 | 6880 |
|  | rs17513056 | 6 | 158732893 | C | T | 0.104 | 0.021 | 0.212 | 8.11E-07 | 6880 |
|  | rs79648513 | 7 | 18311602 | G | A | 0.097 | 0.021 | 0.229 | 2.69E-06 | 6880 |
|  | rs38820 | 7 | 118070482 | C | G | 0.140 | 0.031 | 0.914 | 4.96E-06 | 6880 |
|  | rs2725087 | 8 | 4601984 | A | T | -0.255 | 0.057 | 0.024 | 7.80E-06 | 6880 |
|  | rs7819125 | 8 | 22926841 | T | C | 0.082 | 0.018 | 0.344 | 6.93E-06 | 6880 |
|  | rs4517092 | 8 | 97974998 | C | T | 0.290 | 0.065 | 0.018 | 9.06E-06 | 6880 |
|  | rs28403152 | 9 | 16670164 | C | G | -0.385 | 0.087 | 0.010 | 8.72E-06 | 6880 |
|  | rs2784923 | 10 | 97150097 | A | C | 0.166 | 0.037 | 0.941 | 6.83E-06 | 6880 |
|  | rs174601 | 11 | 61855668 | T | C | 0.089 | 0.017 | 0.419 | 2.59E-07 | 6880 |
|  | rs1893460 | 11 | 116732961 | A | G | 0.090 | 0.020 | 0.236 | 7.94E-06 | 6880 |
|  | rs151297234 | 12 | 16652538 | C | G | 0.240 | 0.053 | 0.027 | 6.18E-06 | 6880 |
|  | rs11068152 | 12 | 116703206 | A | G | -0.094 | 0.021 | 0.223 | 4.89E-06 | 6880 |
|  | rs61644965 | 13 | 27479289 | T | C | -0.235 | 0.052 | 0.027 | 6.56E-06 | 6880 |
|  | rs9516264 | 13 | 93646525 | T | C | -0.081 | 0.018 | 0.330 | 8.59E-06 | 6880 |
|  | rs709981 | 14 | 56937249 | A | C | 0.077 | 0.017 | 0.467 | 8.76E-06 | 6880 |
|  | rs10468017 | 15 | 58386313 | T | C | 0.087 | 0.018 | 0.338 | 1.43E-06 | 6880 |
|  | rs247617 | 16 | 56956804 | A | C | 0.120 | 0.019 | 0.278 | 2.69E-10 | 6880 |
|  | rs75206639 | 16 | 79069483 | A | G | -0.335 | 0.075 | 0.015 | 9.02E-06 | 6880 |
|  | rs62072490 | 17 | 5010850 | T | G | -0.108 | 0.022 | 0.194 | 8.69E-07 | 6880 |
|  | rs7223101 | 17 | 66571589 | A | C | 0.082 | 0.018 | 0.382 | 4.39E-06 | 6880 |
|  | rs150269921 | 19 | 45759621 | C | T | -0.464 | 0.101 | 0.007 | 4.15E-06 | 6880 |
|  | rs72495602 | 21 | 42137794 | A | G | 0.122 | 0.026 | 0.125 | 2.57E-06 | 6880 |
| Phosphatidylcholine (O-18:0_14:0) | rs7566488 | 2 | 11801863 | G | A | -0.118 | 0.026 | 0.140 | 7.91E-06 | 6462 |
|  | rs77444850 | 3 | 127976198 | C | A | -0.271 | 0.059 | 0.024 | 3.83E-06 | 6462 |
|  | rs60635506 | 4 | 121735493 | C | A | -0.321 | 0.072 | 0.017 | 7.54E-06 | 6462 |
|  | rs116304326 | 4 | 158808075 | T | C | 0.308 | 0.069 | 0.017 | 7.93E-06 | 6462 |
|  | rs10516113 | 5 | 174236186 | G | A | -0.089 | 0.020 | 0.733 | 8.99E-06 | 6462 |
|  | rs9365258 | 6 | 161187390 | C | G | -0.120 | 0.024 | 0.837 | 5.76E-07 | 6462 |
|  | rs117246644 | 10 | 58404019 | T | C | -0.442 | 0.090 | 0.010 | 9.60E-07 | 6462 |
|  | rs72835302 | 10 | 86168949 | A | C | 0.142 | 0.030 | 0.102 | 3.09E-06 | 6462 |
|  | rs79139024 | 14 | 95941109 | C | G | -0.487 | 0.107 | 0.008 | 4.96E-06 | 6462 |
|  | rs111316958 | 15 | 76487930 | T | C | -0.303 | 0.068 | 0.016 | 8.95E-06 | 6462 |
|  | rs146726414 | 16 | 78809113 | T | G | 0.312 | 0.063 | 0.020 | 8.16E-07 | 6462 |
|  | rs73975572 | 17 | 851904 | G | A | -0.200 | 0.045 | 0.042 | 7.98E-06 | 6462 |
|  | rs72637380 | 17 | 16925824 | T | C | -0.169 | 0.035 | 0.067 | 1.84E-06 | 6462 |
|  | rs116907632 | 17 | 81307290 | A | G | 0.706 | 0.150 | 0.004 | 2.58E-06 | 6462 |
|  | rs2115108 | 19 | 7899091 | C | T | 0.098 | 0.021 | 0.243 | 1.73E-06 | 6462 |
|  | rs35728084 | 20 | 17196017 | T | C | -0.174 | 0.039 | 0.053 | 7.53E-06 | 6462 |
|  | rs8129049 | 21 | 38363308 | G | A | 0.096 | 0.020 | 0.252 | 2.40E-06 | 6462 |
| Phosphatidylcholine (O-18:0_16:1) | rs79014169 | 1 | 65877338 | A | G | 0.114 | 0.026 | 0.155 | 9.29E-06 | 5959 |
|  | rs55969585 | 1 | 117639417 | T | G | 0.209 | 0.046 | 0.046 | 4.75E-06 | 5959 |
|  | rs149012657 | 2 | 229375003 | C | T | -0.188 | 0.037 | 0.065 | 3.82E-07 | 5959 |
|  | rs4547920 | 5 | 124738074 | A | G | 0.126 | 0.025 | 0.166 | 4.26E-07 | 5959 |
|  | rs10243115 | 7 | 49798317 | T | C | 0.088 | 0.019 | 0.392 | 3.01E-06 | 5959 |
|  | rs11009204 | 10 | 33061164 | A | G | 0.143 | 0.031 | 0.098 | 4.56E-06 | 5959 |
|  | rs603424 | 10 | 100315722 | A | G | -0.181 | 0.029 | 0.121 | 5.21E-10 | 5959 |
|  | rs639060 | 10 | 100348766 | G | A | 0.219 | 0.049 | 0.037 | 7.25E-06 | 5959 |
|  | rs12575589 | 11 | 96017012 | T | C | 0.112 | 0.023 | 0.205 | 1.17E-06 | 5959 |
|  | rs10859795 | 12 | 94924181 | G | A | -0.084 | 0.018 | 0.494 | 5.50E-06 | 5959 |
|  | rs74644051 | 13 | 27642024 | A | G | -0.344 | 0.072 | 0.017 | 2.04E-06 | 5959 |
|  | rs1257588 | 14 | 99061905 | G | A | -0.084 | 0.019 | 0.538 | 6.86E-06 | 5959 |
|  | rs12903358 | 15 | 97398226 | A | G | -0.117 | 0.026 | 0.146 | 9.03E-06 | 5959 |
|  | rs2911458 | 16 | 88778601 | T | C | -0.090 | 0.019 | 0.389 | 1.54E-06 | 5959 |
|  | rs13044736 | 20 | 48803368 | G | A | 0.085 | 0.019 | 0.398 | 5.96E-06 | 5959 |
| Phosphatidylcholine (O-18:0_20:4) | rs34737685 | 1 | 164956995 | C | A | -0.078 | 0.017 | 0.538 | 5.63E-06 | 6956 |
|  | rs138202451 | 2 | 66991489 | A | G | -0.576 | 0.111 | 0.006 | 2.32E-07 | 6956 |
|  | rs148462060 | 2 | 130169813 | G | A | -0.261 | 0.056 | 0.024 | 3.30E-06 | 6956 |
|  | rs79856406 | 2 | 163582301 | G | T | -0.125 | 0.028 | 0.102 | 9.96E-06 | 6956 |
|  | rs148840717 | 3 | 16036318 | T | G | -0.198 | 0.045 | 0.037 | 8.98E-06 | 6956 |
|  | rs11954170 | 5 | 106783090 | G | A | 0.083 | 0.018 | 0.324 | 6.07E-06 | 6956 |
|  | rs4706511 | 6 | 72738363 | G | A | -0.098 | 0.022 | 0.191 | 9.96E-06 | 6956 |
|  | rs143233558 | 7 | 64330243 | A | G | 0.149 | 0.028 | 0.102 | 1.55E-07 | 6956 |
|  | rs6992951 | 8 | 18172468 | A | C | -0.082 | 0.017 | 0.390 | 2.05E-06 | 6956 |
|  | rs78711160 | 9 | 79743146 | A | G | 0.182 | 0.040 | 0.050 | 4.51E-06 | 6956 |
|  | rs3741252 | 11 | 61744026 | T | C | 0.150 | 0.025 | 0.134 | 1.99E-09 | 6956 |
|  | rs174536 | 11 | 61784455 | C | A | -0.308 | 0.017 | 0.408 | 2.28E-72 | 6956 |
|  | rs117870830 | 11 | 120590088 | G | A | -0.306 | 0.067 | 0.017 | 5.47E-06 | 6956 |
|  | rs34382810 | 12 | 2231632 | C | A | 0.086 | 0.018 | 0.365 | 1.12E-06 | 6956 |
|  | rs73074943 | 12 | 21873844 | T | C | 0.251 | 0.055 | 0.024 | 5.54E-06 | 6956 |
|  | rs11057853 | 12 | 124844767 | C | T | -0.077 | 0.017 | 0.454 | 6.11E-06 | 6956 |
|  | rs9591400 | 13 | 51261013 | T | A | 0.120 | 0.026 | 0.118 | 5.67E-06 | 6956 |
|  | rs1800588 | 15 | 58431476 | T | C | 0.103 | 0.020 | 0.258 | 1.56E-07 | 6956 |
|  | rs4782783 | 16 | 83381927 | C | T | 0.125 | 0.027 | 0.888 | 3.64E-06 | 6956 |
|  | rs376516 | 17 | 4978481 | G | C | -0.075 | 0.017 | 0.473 | 8.56E-06 | 6956 |
|  | rs145755646 | 17 | 59341291 | G | C | 0.160 | 0.035 | 0.065 | 4.21E-06 | 6956 |
|  | rs7412 | 19 | 44908822 | T | C | 0.194 | 0.038 | 0.053 | 2.93E-07 | 6956 |
| Phosphatidylcholine (O-18:1_16:0) | rs606587 | 1 | 230180443 | G | A | -0.079 | 0.017 | 0.374 | 7.17E-06 | 6956 |
|  | rs13030345 | 2 | 27780307 | T | G | -0.108 | 0.022 | 0.186 | 6.73E-07 | 6956 |
|  | rs60331765 | 2 | 181582854 | G | A | -0.103 | 0.022 | 0.180 | 2.80E-06 | 6956 |
|  | rs142774676 | 2 | 200469214 | A | G | 0.296 | 0.067 | 0.016 | 9.42E-06 | 6956 |
|  | rs9682490 | 3 | 4949091 | C | T | -0.086 | 0.017 | 0.435 | 6.24E-07 | 6956 |
|  | rs138542536 | 4 | 25796122 | C | A | -0.387 | 0.079 | 0.013 | 1.14E-06 | 6956 |
|  | rs189970593 | 4 | 110407918 | A | C | -0.157 | 0.035 | 0.063 | 9.50E-06 | 6956 |
|  | rs72683831 | 4 | 124795272 | C | T | 0.159 | 0.034 | 0.071 | 2.23E-06 | 6956 |
|  | rs7677028 | 4 | 137022381 | G | C | 0.382 | 0.072 | 0.014 | 1.30E-07 | 6956 |
|  | rs56868865 | 5 | 82667322 | A | G | 0.125 | 0.028 | 0.101 | 9.97E-06 | 6956 |
|  | rs258736 | 5 | 115795209 | T | C | -0.098 | 0.022 | 0.807 | 6.54E-06 | 6956 |
|  | rs7450458 | 6 | 31450839 | A | G | -0.114 | 0.024 | 0.144 | 1.99E-06 | 6956 |
|  | rs57493997 | 6 | 34475746 | C | T | -0.144 | 0.031 | 0.081 | 3.57E-06 | 6956 |
|  | rs6568686 | 6 | 111551279 | C | T | -0.103 | 0.023 | 0.839 | 7.96E-06 | 6956 |
|  | rs6908815 | 6 | 167330921 | C | T | 0.087 | 0.020 | 0.259 | 8.67E-06 | 6956 |
|  | rs34861396 | 8 | 31640951 | A | G | 0.093 | 0.018 | 0.299 | 4.51E-07 | 6956 |
|  | rs1503311 | 11 | 13766154 | T | C | 0.098 | 0.021 | 0.197 | 3.43E-06 | 6956 |
|  | rs78194510 | 12 | 124853790 | C | T | 0.135 | 0.026 | 0.117 | 3.32E-07 | 6956 |
|  | rs1049302 | 13 | 27424632 | C | T | 0.088 | 0.019 | 0.742 | 5.84E-06 | 6956 |
|  | rs7327319 | 13 | 40354480 | A | C | 0.117 | 0.024 | 0.150 | 7.43E-07 | 6956 |
|  | rs7330980 | 13 | 60824670 | A | G | 0.148 | 0.032 | 0.076 | 4.90E-06 | 6956 |
|  | rs2043082 | 15 | 58382109 | A | G | 0.097 | 0.017 | 0.411 | 1.61E-08 | 6956 |
|  | rs1800588 | 15 | 58431476 | T | C | 0.123 | 0.020 | 0.258 | 4.58E-10 | 6956 |
|  | rs118138240 | 16 | 760921 | T | C | -0.230 | 0.049 | 0.033 | 2.89E-06 | 6956 |
|  | rs246168 | 16 | 14276763 | A | C | 0.158 | 0.034 | 0.067 | 4.05E-06 | 6956 |
|  | rs17231506 | 16 | 56960616 | T | C | 0.135 | 0.019 | 0.278 | 7.74E-13 | 6956 |
|  | rs113746682 | 16 | 85344145 | T | C | 0.292 | 0.059 | 0.023 | 8.63E-07 | 6956 |
|  | rs73015021 | 19 | 11082239 | G | A | -0.147 | 0.029 | 0.092 | 5.36E-07 | 6956 |
|  | rs79674727 | 19 | 28504762 | C | T | 0.129 | 0.027 | 0.111 | 2.72E-06 | 6956 |
|  | rs187861964 | 20 | 61244195 | G | C | 0.260 | 0.056 | 0.026 | 3.54E-06 | 6956 |
| Phosphatidylcholine (O-18:1_18:2) | rs17875991 | 1 | 11749358 | T | C | -0.202 | 0.046 | 0.040 | 9.75E-06 | 6718 |
|  | rs4655970 | 1 | 87571263 | C | T | 0.085 | 0.019 | 0.649 | 6.73E-06 | 6718 |
|  | rs72709643 | 1 | 181004059 | C | T | -0.213 | 0.046 | 0.036 | 4.74E-06 | 6718 |
|  | rs111306887 | 1 | 183933905 | A | G | -0.236 | 0.052 | 0.028 | 5.62E-06 | 6718 |
|  | rs606587 | 1 | 230180443 | G | A | -0.091 | 0.018 | 0.374 | 3.41E-07 | 6718 |
|  | rs61833681 | 1 | 240595182 | A | G | -0.091 | 0.020 | 0.243 | 7.76E-06 | 6718 |
|  | rs11918522 | 3 | 112816210 | A | G | 0.127 | 0.024 | 0.159 | 7.40E-08 | 6718 |
|  | rs62294908 | 3 | 182474482 | G | A | 0.134 | 0.030 | 0.110 | 5.72E-06 | 6718 |
|  | rs62331262 | 4 | 104599304 | C | G | 0.107 | 0.023 | 0.173 | 3.11E-06 | 6718 |
|  | rs115600841 | 4 | 173654939 | G | T | 0.303 | 0.068 | 0.017 | 9.46E-06 | 6718 |
|  | rs17086339 | 5 | 96599571 | C | G | -0.114 | 0.026 | 0.130 | 8.41E-06 | 6718 |
|  | rs2537611 | 7 | 17693175 | T | C | 0.087 | 0.018 | 0.372 | 9.77E-07 | 6718 |
|  | rs74864573 | 7 | 31320724 | A | G | -0.223 | 0.049 | 0.033 | 5.58E-06 | 6718 |
|  | rs117145701 | 7 | 109154252 | A | C | -0.439 | 0.098 | 0.008 | 8.00E-06 | 6718 |
|  | rs11022354 | 11 | 12425660 | A | G | 0.077 | 0.017 | 0.535 | 9.48E-06 | 6718 |
|  | rs2985394 | 11 | 34887145 | G | A | 0.087 | 0.018 | 0.669 | 2.63E-06 | 6718 |
|  | rs174551 | 11 | 61806212 | C | T | 0.229 | 0.017 | 0.406 | 3.59E-39 | 6718 |
|  | rs79040967 | 11 | 97125581 | A | G | 0.201 | 0.043 | 0.044 | 2.75E-06 | 6718 |
|  | rs147996419 | 15 | 45403250 | T | G | -0.322 | 0.073 | 0.015 | 1.00E-05 | 6718 |
|  | rs1601934 | 15 | 58379522 | A | G | -0.097 | 0.018 | 0.645 | 8.40E-08 | 6718 |
|  | rs1800588 | 15 | 58431476 | T | C | 0.090 | 0.020 | 0.258 | 6.89E-06 | 6718 |
|  | rs17231506 | 16 | 56960616 | T | C | 0.095 | 0.019 | 0.278 | 6.14E-07 | 6718 |
|  | rs58828781 | 19 | 2786104 | A | T | 0.197 | 0.043 | 0.048 | 4.42E-06 | 6718 |
|  | rs73133304 | 20 | 21155899 | C | A | 0.552 | 0.121 | 0.005 | 4.81E-06 | 6718 |
|  | rs2133619 | 20 | 55033469 | C | G | -0.246 | 0.054 | 0.027 | 5.38E-06 | 6718 |
| Phosphatidylcholine (O-18:1_20:3) | rs1925669 | 1 | 20394196 | A | G | -0.113 | 0.025 | 0.128 | 9.76E-06 | 6836 |
|  | rs149022879 | 1 | 212057557 | A | G | -0.228 | 0.051 | 0.030 | 6.44E-06 | 6836 |
|  | rs12239821 | 1 | 245438211 | A | G | -0.130 | 0.028 | 0.108 | 4.62E-06 | 6836 |
|  | rs9843297 | 3 | 22449600 | G | A | 0.100 | 0.022 | 0.196 | 4.75E-06 | 6836 |
|  | rs2170790 | 3 | 25418634 | C | T | 0.628 | 0.130 | 0.005 | 1.33E-06 | 6836 |
|  | rs12659734 | 5 | 9303860 | C | T | 0.107 | 0.023 | 0.155 | 5.21E-06 | 6836 |
|  | rs1540881 | 7 | 18082952 | T | C | 0.083 | 0.018 | 0.540 | 3.73E-06 | 6836 |
|  | rs62493656 | 7 | 158353254 | T | C | -0.214 | 0.046 | 0.036 | 3.89E-06 | 6836 |
|  | rs139594872 | 8 | 140933062 | A | G | -0.206 | 0.046 | 0.035 | 9.50E-06 | 6836 |
|  | rs78183891 | 9 | 70856641 | T | C | 0.223 | 0.050 | 0.030 | 7.43E-06 | 6836 |
|  | rs1978125 | 11 | 2436436 | A | G | 0.103 | 0.023 | 0.196 | 9.23E-06 | 6836 |
|  | rs174548 | 11 | 61803876 | G | C | 0.259 | 0.017 | 0.384 | 2.68E-49 | 6836 |
|  | rs11236557 | 11 | 75841084 | T | C | 0.092 | 0.018 | 0.582 | 2.63E-07 | 6836 |
|  | rs188004353 | 15 | 68561088 | G | C | -0.230 | 0.051 | 0.032 | 6.36E-06 | 6836 |
|  | rs6498540 | 16 | 15036737 | G | A | -0.109 | 0.018 | 0.334 | 1.93E-09 | 6836 |
|  | rs12444786 | 16 | 82643141 | G | A | 0.108 | 0.023 | 0.821 | 3.29E-06 | 6836 |
|  | rs62131325 | 19 | 770714 | T | C | -0.102 | 0.022 | 0.209 | 5.29E-06 | 6836 |
|  | rs71351740 | 19 | 33752643 | G | A | -0.085 | 0.019 | 0.297 | 6.11E-06 | 6836 |
|  | rs7412 | 19 | 44908822 | T | C | 0.205 | 0.038 | 0.053 | 9.45E-08 | 6836 |
|  | rs111260004 | 20 | 35695325 | C | G | -0.256 | 0.056 | 0.026 | 4.57E-06 | 6836 |
| Phosphatidylcholine (O-18:1_20:4) | rs12760720 | 1 | 234273597 | G | T | -0.077 | 0.017 | 0.524 | 5.43E-06 | 7173 |
|  | rs6721307 | 2 | 123649367 | C | T | 0.076 | 0.017 | 0.428 | 8.63E-06 | 7173 |
|  | rs2332690 | 3 | 124049075 | G | T | -0.092 | 0.021 | 0.790 | 7.30E-06 | 7173 |
|  | rs2522051 | 5 | 132461886 | C | T | -0.076 | 0.017 | 0.576 | 8.47E-06 | 7173 |
|  | rs34881711 | 5 | 154465674 | A | G | 0.090 | 0.020 | 0.229 | 5.67E-06 | 7173 |
|  | rs116814948 | 6 | 2822782 | C | T | -0.421 | 0.094 | 0.009 | 7.98E-06 | 7173 |
|  | rs4142412 | 6 | 34451115 | A | G | 0.094 | 0.020 | 0.762 | 1.66E-06 | 7173 |
|  | rs9800437 | 6 | 169319631 | T | C | -0.117 | 0.026 | 0.122 | 5.97E-06 | 7173 |
|  | rs149490626 | 8 | 55496716 | G | A | 0.277 | 0.061 | 0.019 | 6.41E-06 | 7173 |
|  | rs4618752 | 9 | 1294441 | T | G | 0.077 | 0.017 | 0.506 | 3.57E-06 | 7173 |
|  | rs638491 | 9 | 15290014 | G | A | 0.114 | 0.026 | 0.881 | 8.31E-06 | 7173 |
|  | rs174527 | 11 | 61770929 | C | G | -0.090 | 0.017 | 0.444 | 1.22E-07 | 7173 |
|  | rs174536 | 11 | 61784455 | C | A | -0.284 | 0.017 | 0.408 | 3.74E-63 | 7173 |
|  | rs3017103 | 11 | 62406721 | G | A | 0.101 | 0.021 | 0.804 | 1.91E-06 | 7173 |
|  | rs11057853 | 12 | 124844767 | C | T | -0.094 | 0.017 | 0.454 | 2.45E-08 | 7173 |
|  | rs4942185 | 13 | 42984710 | C | T | -0.081 | 0.018 | 0.687 | 5.59E-06 | 7173 |
|  | rs79995028 | 14 | 41287117 | T | G | -0.604 | 0.133 | 0.004 | 5.32E-06 | 7173 |
|  | rs77199708 | 14 | 61648205 | A | G | 0.188 | 0.042 | 0.043 | 9.19E-06 | 7173 |
|  | rs1532085 | 15 | 58391167 | G | A | -0.082 | 0.017 | 0.573 | 1.05E-06 | 7173 |
|  | rs1800588 | 15 | 58431476 | T | C | 0.107 | 0.019 | 0.258 | 3.09E-08 | 7173 |
|  | rs173539 | 16 | 56954132 | T | C | 0.121 | 0.018 | 0.283 | 6.68E-11 | 7173 |
|  | rs117215929 | 18 | 27258198 | C | T | -0.226 | 0.043 | 0.042 | 1.52E-07 | 7173 |
|  | rs6090437 | 20 | 63507672 | T | C | 0.085 | 0.019 | 0.733 | 9.23E-06 | 7173 |
| Phosphatidylcholine (O-18:2_16:0) | rs34446132 | 1 | 14405165 | A | G | -0.221 | 0.049 | 0.033 | 6.08E-06 | 7158 |
|  | rs185625951 | 1 | 49528668 | T | G | -0.293 | 0.062 | 0.019 | 2.52E-06 | 7158 |
|  | rs12123955 | 1 | 200136295 | T | C | -0.161 | 0.035 | 0.061 | 3.59E-06 | 7158 |
|  | rs11572749 | 1 | 216582264 | A | C | -0.143 | 0.032 | 0.078 | 9.02E-06 | 7158 |
|  | rs76437425 | 1 | 245514505 | T | G | 0.106 | 0.022 | 0.173 | 1.96E-06 | 7158 |
|  | rs114061227 | 2 | 12892879 | G | A | 0.795 | 0.179 | 0.002 | 9.34E-06 | 7158 |
|  | rs7563526 | 2 | 81754844 | T | C | 0.243 | 0.050 | 0.031 | 1.17E-06 | 7158 |
|  | rs55904113 | 3 | 82448350 | T | C | 0.096 | 0.021 | 0.187 | 6.11E-06 | 7158 |
|  | rs142235374 | 4 | 7350426 | A | G | 0.347 | 0.064 | 0.018 | 7.44E-08 | 7158 |
|  | rs188033349 | 4 | 28252716 | A | G | -0.449 | 0.094 | 0.009 | 1.88E-06 | 7158 |
|  | rs77219173 | 4 | 78357277 | T | C | -0.264 | 0.059 | 0.020 | 8.10E-06 | 7158 |
|  | rs7677028 | 4 | 137022381 | G | C | 0.327 | 0.071 | 0.014 | 3.79E-06 | 7158 |
|  | rs11936543 | 4 | 151270229 | G | A | -0.129 | 0.028 | 0.100 | 5.25E-06 | 7158 |
|  | rs9341454 | 6 | 74169888 | G | A | -0.082 | 0.018 | 0.671 | 4.37E-06 | 7158 |
|  | rs75957793 | 7 | 52644681 | T | G | 0.107 | 0.023 | 0.152 | 5.10E-06 | 7158 |
|  | rs149897686 | 8 | 31250608 | T | C | 0.301 | 0.063 | 0.020 | 1.46E-06 | 7158 |
|  | rs7870035 | 9 | 135771210 | A | G | 0.078 | 0.017 | 0.484 | 7.81E-06 | 7158 |
|  | rs7091107 | 10 | 55141172 | T | C | 0.366 | 0.080 | 0.011 | 5.09E-06 | 7158 |
|  | rs10768424 | 11 | 4976404 | G | A | 0.079 | 0.017 | 0.573 | 3.98E-06 | 7158 |
|  | rs174566 | 11 | 61824890 | G | A | 0.148 | 0.017 | 0.408 | 4.33E-18 | 7158 |
|  | rs113016188 | 11 | 100294047 | T | C | -0.796 | 0.165 | 0.003 | 1.56E-06 | 7158 |
|  | rs149389892 | 16 | 47545501 | T | C | -0.325 | 0.072 | 0.014 | 7.22E-06 | 7158 |
|  | rs173539 | 16 | 56954132 | T | C | 0.109 | 0.019 | 0.283 | 3.53E-09 | 7158 |
|  | rs73263508 | 17 | 13886556 | C | G | -0.210 | 0.046 | 0.034 | 5.60E-06 | 7158 |
|  | rs146468726 | 18 | 49199328 | A | G | -0.211 | 0.047 | 0.033 | 6.14E-06 | 7158 |
|  | rs10775658 | 21 | 37607127 | A | G | -0.078 | 0.017 | 0.621 | 6.06E-06 | 7158 |
| Phosphatidylcholine (O-18:2_18:1) | rs72709643 | 1 | 181004059 | C | T | -0.217 | 0.047 | 0.036 | 3.21E-06 | 6677 |
|  | rs55975242 | 2 | 4201657 | A | G | -0.216 | 0.046 | 0.037 | 2.58E-06 | 6677 |
|  | rs148835629 | 2 | 125728700 | G | C | 0.224 | 0.050 | 0.033 | 6.67E-06 | 6677 |
|  | rs7642984 | 3 | 112839107 | T | C | 0.130 | 0.024 | 0.157 | 5.05E-08 | 6677 |
|  | rs56734149 | 3 | 143232607 | A | C | 0.103 | 0.023 | 0.180 | 5.56E-06 | 6677 |
|  | rs73189920 | 3 | 182320787 | C | T | 0.133 | 0.029 | 0.108 | 6.13E-06 | 6677 |
|  | rs79847391 | 6 | 69804495 | T | A | 0.168 | 0.038 | 0.056 | 8.04E-06 | 6677 |
|  | rs370796571 | 7 | 53119301 | C | A | -0.237 | 0.048 | 0.034 | 7.72E-07 | 6677 |
|  | rs1982433 | 8 | 77170491 | G | A | -0.135 | 0.030 | 0.092 | 6.46E-06 | 6677 |
|  | rs3110417 | 8 | 88464222 | G | T | 0.089 | 0.020 | 0.257 | 8.10E-06 | 6677 |
|  | rs10868635 | 9 | 87561451 | A | G | -0.089 | 0.019 | 0.299 | 2.59E-06 | 6677 |
|  | rs72840788 | 10 | 119656173 | A | G | 0.097 | 0.021 | 0.229 | 3.16E-06 | 6677 |
|  | rs117376487 | 11 | 35071655 | A | G | -0.417 | 0.090 | 0.009 | 3.31E-06 | 6677 |
|  | rs174564 | 11 | 61820833 | G | A | 0.147 | 0.018 | 0.409 | 7.47E-17 | 6677 |
|  | rs79713419 | 14 | 73445684 | C | A | 0.088 | 0.020 | 0.258 | 9.10E-06 | 6677 |
|  | rs1077834 | 15 | 58431280 | C | T | 0.089 | 0.020 | 0.266 | 7.47E-06 | 6677 |
|  | rs12441343 | 15 | 59939767 | A | G | 0.079 | 0.017 | 0.539 | 5.58E-06 | 6677 |
|  | rs62621679 | 17 | 66778240 | G | C | 0.175 | 0.036 | 0.061 | 1.61E-06 | 6677 |
|  | rs77664718 | 19 | 2942174 | T | C | -0.099 | 0.022 | 0.236 | 9.55E-06 | 6677 |
| Phosphatidylcholine (O-18:2_18:2) | rs191784724 | 1 | 43502581 | C | A | 0.234 | 0.049 | 0.031 | 2.38E-06 | 7153 |
|  | rs114930909 | 3 | 60237477 | A | G | 0.369 | 0.078 | 0.012 | 2.57E-06 | 7153 |
|  | rs113370662 | 4 | 3535970 | T | C | 0.158 | 0.035 | 0.064 | 8.15E-06 | 7153 |
|  | rs7677028 | 4 | 137022381 | G | C | 0.324 | 0.071 | 0.014 | 4.38E-06 | 7153 |
|  | rs149058290 | 5 | 38235392 | T | C | -0.358 | 0.079 | 0.012 | 6.50E-06 | 7153 |
|  | rs114401161 | 5 | 154241592 | G | T | 0.195 | 0.042 | 0.042 | 3.14E-06 | 7153 |
|  | rs9366277 | 6 | 10147800 | T | A | -0.078 | 0.017 | 0.636 | 8.27E-06 | 7153 |
|  | rs61448283 | 6 | 169316922 | T | C | -0.175 | 0.038 | 0.054 | 3.69E-06 | 7153 |
|  | rs375094094 | 9 | 20097890 | C | T | -0.344 | 0.076 | 0.013 | 6.53E-06 | 7153 |
|  | rs784655 | 9 | 107072174 | G | A | -0.081 | 0.017 | 0.550 | 1.59E-06 | 7153 |
|  | rs150046733 | 11 | 732354 | C | T | -0.175 | 0.039 | 0.049 | 7.84E-06 | 7153 |
|  | rs174574 | 11 | 61832870 | C | A | -0.156 | 0.017 | 0.591 | 6.55E-20 | 7153 |
|  | rs35874447 | 12 | 124967043 | G | A | 0.096 | 0.019 | 0.288 | 2.80E-07 | 7153 |
|  | rs28489182 | 14 | 77828999 | A | G | 0.112 | 0.024 | 0.145 | 2.46E-06 | 7153 |
|  | rs1601934 | 15 | 58379522 | A | G | -0.087 | 0.017 | 0.645 | 5.69E-07 | 7153 |
|  | rs1800588 | 15 | 58431476 | T | C | 0.103 | 0.019 | 0.258 | 1.03E-07 | 7153 |
|  | rs17231506 | 16 | 56960616 | T | C | 0.117 | 0.019 | 0.278 | 2.96E-10 | 7153 |
|  | rs12922657 | 16 | 57284962 | G | A | 0.079 | 0.017 | 0.358 | 6.13E-06 | 7153 |
|  | rs140484966 | 21 | 30352436 | C | T | -0.246 | 0.055 | 0.028 | 7.38E-06 | 7153 |
| Phosphatidylcholine (O-18:2_20:4) | rs12046045 | 1 | 47591044 | A | G | -0.156 | 0.032 | 0.089 | 1.12E-06 | 6127 |
|  | rs1936090 | 1 | 164952845 | A | G | -0.082 | 0.018 | 0.534 | 6.91E-06 | 6127 |
|  | rs3820438 | 1 | 201955621 | C | G | 0.088 | 0.020 | 0.291 | 9.13E-06 | 6127 |
|  | rs13401553 | 2 | 123610457 | C | T | 0.087 | 0.018 | 0.428 | 2.18E-06 | 6127 |
|  | rs6722606 | 2 | 191752537 | C | A | -0.452 | 0.096 | 0.009 | 2.52E-06 | 6127 |
|  | rs7660654 | 4 | 179908526 | T | A | -0.092 | 0.021 | 0.751 | 9.70E-06 | 6127 |
|  | rs112718171 | 7 | 41177033 | C | T | 0.517 | 0.113 | 0.007 | 5.32E-06 | 6127 |
|  | rs2189991 | 7 | 82556403 | A | G | 0.087 | 0.018 | 0.587 | 1.53E-06 | 6127 |
|  | rs6467318 | 7 | 130802167 | G | A | -0.088 | 0.019 | 0.574 | 2.82E-06 | 6127 |
|  | rs73691232 | 8 | 76925244 | A | C | -0.154 | 0.034 | 0.075 | 7.59E-06 | 6127 |
|  | rs76375697 | 9 | 32337256 | G | T | 0.340 | 0.071 | 0.018 | 1.77E-06 | 6127 |
|  | rs174537 | 11 | 61785208 | T | G | -0.176 | 0.018 | 0.408 | 1.97E-21 | 6127 |
|  | rs7118021 | 11 | 89058340 | A | G | -0.117 | 0.025 | 0.159 | 1.95E-06 | 6127 |
|  | rs113023745 | 13 | 66979217 | A | G | -0.254 | 0.047 | 0.037 | 9.21E-08 | 6127 |
|  | rs1980615 | 14 | 67491655 | C | A | -0.103 | 0.018 | 0.455 | 1.12E-08 | 6127 |
|  | rs17778967 | 15 | 43258624 | A | G | 0.250 | 0.053 | 0.032 | 2.46E-06 | 6127 |
|  | rs7203057 | 16 | 57439704 | G | T | -0.200 | 0.044 | 0.041 | 6.77E-06 | 6127 |
|  | rs16975847 | 16 | 75555692 | A | G | -0.124 | 0.026 | 0.134 | 2.76E-06 | 6127 |
|  | rs145525514 | 17 | 13263077 | T | C | 0.177 | 0.039 | 0.061 | 5.59E-06 | 6127 |
|  | rs141198865 | 17 | 17676043 | T | C | -0.349 | 0.076 | 0.018 | 4.02E-06 | 6127 |
|  | rs117215929 | 18 | 27258198 | C | T | -0.229 | 0.047 | 0.042 | 1.00E-06 | 6127 |
| Phosphatidylethanolamine (16:0_18:2) | rs7550598 | 1 | 15301756 | G | T | 0.171 | 0.038 | 0.072 | 5.20E-06 | 5462 |
|  | rs4129186 | 1 | 214395764 | C | T | -0.153 | 0.033 | 0.096 | 3.37E-06 | 5462 |
|  | rs140904588 | 2 | 11679202 | A | G | 0.197 | 0.042 | 0.061 | 2.89E-06 | 5462 |
|  | rs1260326 | 2 | 27508073 | C | T | -0.116 | 0.020 | 0.651 | 5.70E-09 | 5462 |
|  | rs35319363 | 5 | 163344530 | T | C | 0.173 | 0.038 | 0.068 | 6.97E-06 | 5462 |
|  | rs6415128 | 6 | 32363117 | A | G | 0.090 | 0.020 | 0.663 | 7.65E-06 | 5462 |
|  | rs436509 | 6 | 169389898 | C | T | 0.122 | 0.027 | 0.851 | 6.32E-06 | 5462 |
|  | rs61696472 | 7 | 43464229 | G | A | 0.092 | 0.019 | 0.390 | 1.99E-06 | 5462 |
|  | rs4269529 | 8 | 66311811 | C | T | -0.504 | 0.098 | 0.010 | 2.76E-07 | 5462 |
|  | rs117099105 | 9 | 116846849 | C | G | 0.459 | 0.098 | 0.011 | 2.73E-06 | 5462 |
|  | rs1891637 | 9 | 123321244 | T | C | -0.102 | 0.023 | 0.771 | 7.64E-06 | 5462 |
|  | rs4363488 | 10 | 63102554 | G | T | -0.109 | 0.024 | 0.811 | 8.20E-06 | 5462 |
|  | rs174564 | 11 | 61820833 | G | A | 0.308 | 0.019 | 0.409 | 5.58E-58 | 5462 |
|  | rs12226389 | 11 | 62056158 | C | T | 0.118 | 0.025 | 0.173 | 3.11E-06 | 5462 |
|  | rs510988 | 11 | 116943088 | T | C | -0.146 | 0.025 | 0.829 | 7.40E-09 | 5462 |
|  | rs894342 | 15 | 57885393 | T | C | 0.096 | 0.019 | 0.530 | 7.35E-07 | 5462 |
|  | rs2043085 | 15 | 58388755 | C | T | -0.263 | 0.019 | 0.576 | 1.18E-43 | 5462 |
|  | rs1077834 | 15 | 58431280 | C | T | 0.316 | 0.021 | 0.266 | 1.42E-49 | 5462 |
|  | rs544282 | 15 | 58729903 | A | G | 0.106 | 0.021 | 0.280 | 5.18E-07 | 5462 |
|  | rs34278620 | 15 | 59032191 | G | A | 0.225 | 0.028 | 0.125 | 2.95E-15 | 5462 |
|  | rs7180275 | 15 | 74379820 | G | A | -0.142 | 0.027 | 0.153 | 2.05E-07 | 5462 |
| Phosphatidylethanolamine (16:0_20:4) | rs1193168 | 1 | 7531062 | C | A | 0.085 | 0.018 | 0.655 | 4.64E-06 | 6666 |
|  | rs1260326 | 2 | 27508073 | C | T | -0.086 | 0.018 | 0.651 | 2.11E-06 | 6666 |
|  | rs62155727 | 2 | 101740535 | G | A | -0.121 | 0.024 | 0.151 | 4.42E-07 | 6666 |
|  | rs79450270 | 2 | 138745944 | A | G | 0.383 | 0.082 | 0.013 | 3.03E-06 | 6666 |
|  | rs74929979 | 3 | 8868967 | C | T | -0.187 | 0.042 | 0.047 | 9.13E-06 | 6666 |
|  | rs115265221 | 3 | 141158645 | G | C | -0.467 | 0.097 | 0.008 | 1.37E-06 | 6666 |
|  | rs72679383 | 4 | 100929253 | T | G | 0.313 | 0.061 | 0.022 | 2.65E-07 | 6666 |
|  | rs4370152 | 4 | 121541914 | A | G | -0.083 | 0.018 | 0.417 | 2.70E-06 | 6666 |
|  | rs76435838 | 7 | 35570399 | A | C | 0.147 | 0.033 | 0.075 | 7.46E-06 | 6666 |
|  | rs75344234 | 8 | 138164717 | C | T | -0.178 | 0.037 | 0.066 | 1.98E-06 | 6666 |
|  | rs149460121 | 9 | 137858500 | A | T | 0.145 | 0.029 | 0.109 | 5.91E-07 | 6666 |
|  | rs139848079 | 10 | 59368742 | T | C | -0.254 | 0.053 | 0.030 | 1.66E-06 | 6666 |
|  | rs9943332 | 10 | 128061505 | A | G | 0.120 | 0.026 | 0.135 | 3.01E-06 | 6666 |
|  | rs55864504 | 11 | 98131421 | T | G | 0.135 | 0.030 | 0.089 | 9.93E-06 | 6666 |
|  | rs1815786 | 11 | 117050674 | C | T | -0.134 | 0.025 | 0.866 | 1.43E-07 | 6666 |
|  | rs10892181 | 11 | 117828871 | G | T | 0.080 | 0.017 | 0.466 | 4.17E-06 | 6666 |
|  | rs34733845 | 12 | 60861641 | T | C | 0.167 | 0.037 | 0.059 | 5.96E-06 | 6666 |
|  | rs192384799 | 14 | 41753127 | G | T | 0.402 | 0.091 | 0.010 | 9.43E-06 | 6666 |
|  | rs142259144 | 14 | 57331615 | C | T | 0.148 | 0.033 | 0.078 | 8.74E-06 | 6666 |
|  | rs1690333 | 15 | 57616988 | C | T | 0.100 | 0.017 | 0.439 | 1.20E-08 | 6666 |
|  | rs61999891 | 15 | 58007401 | A | G | 0.161 | 0.029 | 0.104 | 3.10E-08 | 6666 |
|  | rs2043085 | 15 | 58388755 | C | T | -0.372 | 0.017 | 0.576 | 3.62E-104 | 6666 |
|  | rs1800588 | 15 | 58431476 | T | C | 0.393 | 0.019 | 0.258 | 1.26E-88 | 6666 |
|  | rs34278620 | 15 | 59032191 | G | A | 0.213 | 0.026 | 0.125 | 4.39E-16 | 6666 |
|  | rs2684847 | 18 | 47243946 | A | C | -0.118 | 0.025 | 0.853 | 2.02E-06 | 6666 |
| Phosphatidylethanolamine (18:0_18:2) | rs1260326 | 2 | 27508073 | C | T | -0.122 | 0.017 | 0.651 | 2.57E-12 | 7128 |
|  | rs4296402 | 2 | 174696563 | A | G | 0.106 | 0.023 | 0.160 | 4.49E-06 | 7128 |
|  | rs62291572 | 4 | 4996200 | T | C | 0.100 | 0.021 | 0.214 | 1.96E-06 | 7128 |
|  | rs13110318 | 4 | 38137235 | A | G | -0.121 | 0.026 | 0.114 | 3.74E-06 | 7128 |
|  | rs72679383 | 4 | 100929253 | T | G | 0.286 | 0.059 | 0.022 | 1.04E-06 | 7128 |
|  | rs149431055 | 4 | 115874677 | T | C | -0.243 | 0.055 | 0.025 | 9.10E-06 | 7128 |
|  | rs10069895 | 5 | 86297284 | C | T | 0.097 | 0.020 | 0.764 | 7.18E-07 | 7128 |
|  | rs141883259 | 5 | 120061007 | G | A | 0.373 | 0.083 | 0.010 | 6.59E-06 | 7128 |
|  | rs805292 | 6 | 31722232 | A | G | 0.085 | 0.017 | 0.378 | 1.10E-06 | 7128 |
|  | rs419482 | 6 | 94336592 | T | A | -0.078 | 0.017 | 0.408 | 4.12E-06 | 7128 |
|  | rs62434228 | 6 | 154681357 | A | G | 0.109 | 0.023 | 0.160 | 2.35E-06 | 7128 |
|  | rs10276778 | 7 | 146994417 | C | T | -0.182 | 0.040 | 0.044 | 6.58E-06 | 7128 |
|  | rs2946493 | 8 | 12966162 | C | T | 0.105 | 0.021 | 0.206 | 4.35E-07 | 7128 |
|  | rs10761714 | 10 | 63110944 | A | G | -0.104 | 0.023 | 0.845 | 6.47E-06 | 7128 |
|  | rs174527 | 11 | 61770929 | C | G | 0.102 | 0.017 | 0.444 | 2.73E-09 | 7128 |
|  | rs174567 | 11 | 61825533 | G | A | 0.343 | 0.017 | 0.409 | 1.25E-92 | 7128 |
|  | rs117900629 | 11 | 62205328 | A | G | -0.213 | 0.034 | 0.062 | 6.25E-10 | 7128 |
|  | rs964184 | 11 | 116778201 | C | G | -0.154 | 0.023 | 0.849 | 2.75E-11 | 7128 |
|  | rs35828755 | 12 | 17547405 | C | T | -0.092 | 0.021 | 0.195 | 9.29E-06 | 7128 |
|  | rs34733845 | 12 | 60861641 | T | C | 0.165 | 0.036 | 0.059 | 3.86E-06 | 7128 |
|  | rs138283783 | 15 | 58244328 | C | T | 0.352 | 0.054 | 0.027 | 5.56E-11 | 7128 |
|  | rs10468017 | 15 | 58386313 | T | C | 0.298 | 0.017 | 0.338 | 9.03E-66 | 7128 |
|  | rs633695 | 15 | 58433640 | G | A | 0.255 | 0.018 | 0.314 | 5.56E-45 | 7128 |
|  | rs117376818 | 15 | 58506762 | A | G | 0.630 | 0.070 | 0.016 | 4.16E-19 | 7128 |
|  | rs73424597 | 15 | 58607410 | C | T | 0.355 | 0.045 | 0.035 | 5.88E-15 | 7128 |
|  | rs112129861 | 15 | 59047450 | C | G | 0.156 | 0.034 | 0.065 | 3.91E-06 | 7128 |
|  | rs111543310 | 15 | 59239619 | C | T | 0.280 | 0.060 | 0.022 | 3.55E-06 | 7128 |
|  | rs12928099 | 16 | 15056648 | A | C | -0.113 | 0.019 | 0.274 | 1.50E-09 | 7128 |
|  | rs449480 | 16 | 84499954 | C | G | 0.076 | 0.017 | 0.541 | 5.89E-06 | 7128 |
|  | rs7226158 | 17 | 79394643 | C | T | -0.081 | 0.018 | 0.684 | 6.06E-06 | 7128 |
|  | rs2840005 | 19 | 55803533 | C | T | -0.111 | 0.025 | 0.133 | 8.44E-06 | 7128 |
| Phosphatidylethanolamine (18:0_20:4) | rs1260326 | 2 | 27508073 | C | T | -0.088 | 0.017 | 0.651 | 5.43E-07 | 7164 |
|  | rs113887618 | 3 | 71834170 | A | G | -0.082 | 0.018 | 0.301 | 6.81E-06 | 7164 |
|  | rs143607354 | 4 | 61547490 | T | C | -0.188 | 0.041 | 0.042 | 5.48E-06 | 7164 |
|  | rs116866162 | 8 | 11624851 | A | G | -0.260 | 0.055 | 0.025 | 2.07E-06 | 7164 |
|  | rs62549032 | 9 | 74092748 | A | G | 0.226 | 0.049 | 0.031 | 3.71E-06 | 7164 |
|  | rs72753379 | 9 | 120350676 | T | C | 0.221 | 0.045 | 0.039 | 8.09E-07 | 7164 |
|  | rs11031419 | 11 | 31584349 | T | A | 0.177 | 0.035 | 0.059 | 6.13E-07 | 7164 |
|  | rs174537 | 11 | 61785208 | T | G | -0.188 | 0.017 | 0.408 | 1.33E-28 | 7164 |
|  | rs10750098 | 11 | 116834852 | T | G | -0.149 | 0.024 | 0.863 | 1.09E-09 | 7164 |
|  | rs77144832 | 13 | 76058703 | A | G | 0.277 | 0.062 | 0.019 | 8.93E-06 | 7164 |
|  | rs138283783 | 15 | 58244328 | C | T | 0.365 | 0.053 | 0.027 | 9.22E-12 | 7164 |
|  | rs10468017 | 15 | 58386313 | T | C | 0.354 | 0.017 | 0.338 | 7.59E-93 | 7164 |
|  | rs633695 | 15 | 58433640 | G | A | 0.288 | 0.018 | 0.314 | 2.75E-57 | 7164 |
|  | rs117376818 | 15 | 58506762 | A | G | 0.664 | 0.070 | 0.016 | 3.43E-21 | 7164 |
|  | rs73424577 | 15 | 58576986 | G | A | 0.346 | 0.046 | 0.034 | 3.66E-14 | 7164 |
|  | rs112129861 | 15 | 59047450 | C | G | 0.174 | 0.034 | 0.065 | 2.51E-07 | 7164 |
|  | rs111543310 | 15 | 59239619 | C | T | 0.271 | 0.060 | 0.022 | 6.69E-06 | 7164 |
|  | rs17798605 | 18 | 47575930 | G | A | 0.215 | 0.046 | 0.038 | 2.43E-06 | 7164 |
|  | rs73964403 | 18 | 67212380 | G | C | -0.227 | 0.048 | 0.032 | 2.55E-06 | 7164 |
|  | rs2876109 | 20 | 7958929 | T | G | -0.102 | 0.022 | 0.170 | 6.32E-06 | 7164 |
| Phosphatidylethanolamine (18:1_18:1) | rs10915321 | 1 | 4046086 | A | G | 0.169 | 0.036 | 0.069 | 2.38E-06 | 7045 |
|  | rs74899875 | 2 | 11870210 | T | C | 0.184 | 0.040 | 0.052 | 3.60E-06 | 7045 |
|  | rs78564380 | 2 | 13358463 | A | G | 0.346 | 0.072 | 0.016 | 1.46E-06 | 7045 |
|  | rs1260326 | 2 | 27508073 | C | T | -0.083 | 0.018 | 0.651 | 2.56E-06 | 7045 |
|  | rs2946819 | 3 | 118500064 | T | G | -0.143 | 0.030 | 0.912 | 2.38E-06 | 7045 |
|  | rs9837048 | 3 | 133061273 | G | A | -0.076 | 0.017 | 0.558 | 7.59E-06 | 7045 |
|  | rs16874868 | 5 | 5149248 | T | A | -0.127 | 0.028 | 0.106 | 4.62E-06 | 7045 |
|  | rs2503980 | 6 | 67129565 | T | A | -0.083 | 0.018 | 0.325 | 5.68E-06 | 7045 |
|  | rs151000252 | 6 | 85323731 | T | G | 0.789 | 0.178 | 0.002 | 9.22E-06 | 7045 |
|  | rs112314129 | 6 | 138010882 | T | C | 0.147 | 0.033 | 0.074 | 7.26E-06 | 7045 |
|  | rs17151028 | 7 | 43504908 | G | A | 0.091 | 0.020 | 0.214 | 7.40E-06 | 7045 |
|  | rs7779208 | 7 | 146504425 | A | G | -0.422 | 0.087 | 0.010 | 1.22E-06 | 7045 |
|  | rs7009708 | 8 | 22695176 | C | G | -0.076 | 0.017 | 0.432 | 9.35E-06 | 7045 |
|  | rs140764699 | 9 | 92627750 | A | G | -0.476 | 0.102 | 0.007 | 3.08E-06 | 7045 |
|  | rs10751670 | 11 | 944466 | G | A | -0.098 | 0.022 | 0.814 | 5.99E-06 | 7045 |
|  | rs174548 | 11 | 61803876 | G | C | 0.190 | 0.017 | 0.384 | 1.12E-27 | 7045 |
|  | rs17174502 | 11 | 116888939 | A | G | 0.136 | 0.022 | 0.174 | 1.14E-09 | 7045 |
|  | rs9920308 | 15 | 57610033 | A | C | 0.087 | 0.017 | 0.418 | 4.26E-07 | 7045 |
|  | rs1532085 | 15 | 58391167 | G | A | -0.207 | 0.017 | 0.573 | 3.29E-34 | 7045 |
|  | rs2070895 | 15 | 58431740 | A | G | 0.259 | 0.019 | 0.267 | 3.11E-41 | 7045 |
|  | rs11633043 | 15 | 58545523 | A | G | 0.135 | 0.022 | 0.191 | 4.34E-10 | 7045 |
|  | rs112129861 | 15 | 59047450 | C | G | 0.194 | 0.034 | 0.065 | 1.08E-08 | 7045 |
|  | rs11651494 | 17 | 65074311 | A | G | 0.081 | 0.018 | 0.398 | 5.58E-06 | 7045 |
|  | rs72880096 | 17 | 77018065 | C | T | 0.136 | 0.029 | 0.099 | 1.75E-06 | 7045 |
|  | rs139689930 | 20 | 45958142 | T | G | -0.305 | 0.068 | 0.016 | 7.95E-06 | 7045 |
| Phosphatidylethanolamine (O-16:1_18:2) | rs115261885 | 1 | 66604062 | A | G | 0.185 | 0.041 | 0.058 | 7.78E-06 | 5430 |
|  | rs78655391 | 1 | 110525431 | C | T | 0.190 | 0.040 | 0.066 | 1.97E-06 | 5430 |
|  | rs78816612 | 1 | 110840034 | T | C | -0.591 | 0.133 | 0.006 | 9.31E-06 | 5430 |
|  | rs12997068 | 2 | 42197717 | A | G | 0.273 | 0.057 | 0.029 | 1.36E-06 | 5430 |
|  | rs62294904 | 3 | 182462564 | T | A | 0.162 | 0.035 | 0.098 | 3.30E-06 | 5430 |
|  | rs35485513 | 4 | 28423082 | C | T | -0.356 | 0.079 | 0.017 | 7.74E-06 | 5430 |
|  | rs602307 | 5 | 73116953 | A | T | 0.091 | 0.020 | 0.403 | 3.87E-06 | 5430 |
|  | rs10949554 | 6 | 10113293 | G | A | -0.098 | 0.022 | 0.300 | 6.67E-06 | 5430 |
|  | rs311415 | 8 | 54198654 | G | C | -0.092 | 0.020 | 0.588 | 2.65E-06 | 5430 |
|  | rs71520868 | 8 | 104150496 | G | A | 0.459 | 0.104 | 0.010 | 9.92E-06 | 5430 |
|  | rs111680982 | 8 | 121336542 | T | C | -0.564 | 0.126 | 0.007 | 8.44E-06 | 5430 |
|  | rs174530 | 11 | 61779120 | G | A | 0.149 | 0.019 | 0.414 | 1.33E-14 | 5430 |
|  | rs111761575 | 13 | 27121538 | C | T | 0.564 | 0.127 | 0.006 | 8.76E-06 | 5430 |
|  | rs9590678 | 13 | 42237414 | A | T | 0.098 | 0.020 | 0.410 | 7.54E-07 | 5430 |
|  | rs145863254 | 15 | 69696241 | T | C | 0.273 | 0.061 | 0.027 | 9.08E-06 | 5430 |
|  | rs75480840 | 16 | 79902520 | G | C | 0.369 | 0.081 | 0.014 | 5.87E-06 | 5430 |
|  | rs3826884 | 19 | 55226813 | A | G | -0.126 | 0.028 | 0.149 | 5.24E-06 | 5430 |
|  | rs5757363 | 22 | 38910264 | C | G | -0.103 | 0.021 | 0.322 | 7.93E-07 | 5430 |
| Phosphatidylethanolamine (O-16:1_20:4) | rs143745495 | 1 | 119172555 | T | C | -0.098 | 0.022 | 0.172 | 8.71E-06 | 7169 |
|  | rs10494405 | 1 | 163979236 | C | T | -0.127 | 0.028 | 0.106 | 4.62E-06 | 7169 |
|  | rs3820438 | 1 | 201955621 | C | G | 0.090 | 0.018 | 0.291 | 1.03E-06 | 7169 |
|  | rs2573210 | 2 | 232520315 | A | G | 0.113 | 0.023 | 0.838 | 6.34E-07 | 7169 |
|  | rs11725298 | 4 | 52717255 | C | T | 0.259 | 0.057 | 0.022 | 4.96E-06 | 7169 |
|  | rs13123798 | 4 | 183919435 | C | G | 0.085 | 0.017 | 0.589 | 8.11E-07 | 7169 |
|  | rs7445920 | 5 | 74678000 | T | C | 0.097 | 0.021 | 0.193 | 4.82E-06 | 7169 |
|  | rs9265944 | 6 | 31347332 | C | T | 0.084 | 0.017 | 0.391 | 1.13E-06 | 7169 |
|  | rs1034755 | 7 | 8811155 | T | C | 0.085 | 0.018 | 0.319 | 2.13E-06 | 7169 |
|  | rs10487877 | 7 | 80837985 | G | C | 0.182 | 0.040 | 0.047 | 5.12E-06 | 7169 |
|  | rs10234951 | 7 | 94419222 | G | T | -0.151 | 0.031 | 0.080 | 1.17E-06 | 7169 |
|  | rs79337987 | 7 | 138155111 | A | T | -0.294 | 0.061 | 0.020 | 1.32E-06 | 7169 |
|  | rs11136432 | 8 | 1843472 | G | T | -0.080 | 0.017 | 0.602 | 2.74E-06 | 7169 |
|  | rs6601694 | 8 | 8390548 | A | G | 0.086 | 0.018 | 0.334 | 1.47E-06 | 7169 |
|  | rs97384 | 11 | 61856709 | C | T | 0.210 | 0.017 | 0.580 | 4.34E-35 | 7169 |
|  | rs2277289 | 11 | 118311945 | C | T | 0.080 | 0.018 | 0.326 | 6.69E-06 | 7169 |
|  | rs74677222 | 12 | 121783011 | A | G | 0.146 | 0.030 | 0.083 | 1.79E-06 | 7169 |
|  | rs2070895 | 15 | 58431740 | A | G | 0.085 | 0.019 | 0.267 | 9.89E-06 | 7169 |
|  | rs12923459 | 16 | 56955918 | A | G | -0.075 | 0.017 | 0.457 | 8.79E-06 | 7169 |
|  | rs9946720 | 18 | 4552061 | G | T | -0.717 | 0.151 | 0.003 | 1.93E-06 | 7169 |
|  | rs7232502 | 18 | 77015662 | T | C | -0.084 | 0.019 | 0.279 | 6.81E-06 | 7169 |
|  | rs204474 | 19 | 44983921 | T | C | -0.084 | 0.017 | 0.598 | 9.15E-07 | 7169 |
|  | rs3826884 | 19 | 55226813 | A | G | -0.179 | 0.024 | 0.149 | 3.56E-14 | 7169 |
|  | rs78802873 | 20 | 37995099 | A | G | 0.148 | 0.033 | 0.067 | 8.88E-06 | 7169 |
| Phosphatidylethanolamine (O-16:1_22:5) | rs41264497 | 1 | 39624750 | G | A | -0.634 | 0.127 | 0.006 | 5.96E-07 | 5609 |
|  | rs55892660 | 1 | 98370123 | T | C | -0.120 | 0.027 | 0.148 | 7.05E-06 | 5609 |
|  | rs115002881 | 2 | 62271952 | T | G | 0.243 | 0.055 | 0.031 | 9.44E-06 | 5609 |
|  | rs10187082 | 2 | 140761432 | G | C | -0.527 | 0.115 | 0.007 | 4.40E-06 | 5609 |
|  | rs72993205 | 2 | 229625649 | G | A | 0.311 | 0.068 | 0.024 | 5.07E-06 | 5609 |
|  | rs4974228 | 3 | 60777864 | C | T | 0.154 | 0.033 | 0.092 | 2.43E-06 | 5609 |
|  | rs73199626 | 3 | 191170774 | C | T | -0.104 | 0.023 | 0.209 | 6.31E-06 | 5609 |
|  | rs115536895 | 3 | 193209944 | A | G | 0.271 | 0.060 | 0.027 | 6.09E-06 | 5609 |
|  | rs150942220 | 5 | 32055261 | C | G | 0.181 | 0.041 | 0.062 | 9.64E-06 | 5609 |
|  | rs2940919 | 5 | 42470628 | G | A | 0.133 | 0.029 | 0.115 | 5.21E-06 | 5609 |
|  | rs116250572 | 5 | 107567832 | C | G | 0.579 | 0.129 | 0.005 | 6.85E-06 | 5609 |
|  | rs7761485 | 6 | 85404250 | C | T | -0.087 | 0.020 | 0.385 | 9.05E-06 | 5609 |
|  | rs190716231 | 7 | 133098676 | C | G | -0.309 | 0.067 | 0.021 | 3.97E-06 | 5609 |
|  | rs76268189 | 8 | 62982677 | T | C | 0.323 | 0.072 | 0.019 | 8.04E-06 | 5609 |
|  | rs7028232 | 9 | 114255513 | T | C | 0.093 | 0.020 | 0.374 | 2.95E-06 | 5609 |
|  | rs111698799 | 10 | 109515997 | T | C | -0.391 | 0.086 | 0.012 | 5.47E-06 | 5609 |
|  | rs116937344 | 19 | 41215233 | A | G | -0.313 | 0.070 | 0.020 | 7.36E-06 | 5609 |
| Phosphatidylethanolamine (O-18:1_18:2) | rs182992622 | 1 | 153820721 | T | C | 0.638 | 0.143 | 0.004 | 8.01E-06 | 6870 |
|  | rs76085699 | 1 | 206738030 | A | G | -0.122 | 0.025 | 0.134 | 1.42E-06 | 6870 |
|  | rs10926263 | 1 | 240448048 | T | G | -0.086 | 0.019 | 0.698 | 5.29E-06 | 6870 |
|  | rs149968069 | 1 | 242074452 | T | C | -0.250 | 0.055 | 0.025 | 5.34E-06 | 6870 |
|  | rs111703312 | 2 | 204509075 | G | A | 0.334 | 0.071 | 0.016 | 2.19E-06 | 6870 |
|  | rs709166 | 3 | 12470153 | A | G | -0.078 | 0.017 | 0.473 | 5.08E-06 | 6870 |
|  | rs187748847 | 3 | 181603334 | G | A | -0.587 | 0.124 | 0.005 | 2.29E-06 | 6870 |
|  | rs4696957 | 4 | 18851049 | A | G | -0.167 | 0.037 | 0.058 | 7.14E-06 | 6870 |
|  | rs55683433 | 4 | 74887304 | A | C | -0.210 | 0.044 | 0.042 | 1.52E-06 | 6870 |
|  | rs17019568 | 4 | 92558738 | G | C | 0.151 | 0.030 | 0.088 | 7.10E-07 | 6870 |
|  | rs28844909 | 4 | 145707221 | A | G | -0.167 | 0.035 | 0.067 | 1.69E-06 | 6870 |
|  | rs11936543 | 4 | 151270229 | G | A | -0.143 | 0.029 | 0.100 | 7.70E-07 | 6870 |
|  | rs17138200 | 7 | 17697283 | G | C | 0.091 | 0.021 | 0.214 | 8.84E-06 | 6870 |
|  | rs68103316 | 7 | 81690761 | C | T | 0.098 | 0.022 | 0.184 | 9.14E-06 | 6870 |
|  | rs7003304 | 8 | 14334458 | T | C | -0.120 | 0.025 | 0.139 | 1.54E-06 | 6870 |
|  | rs9644026 | 8 | 22936374 | C | T | 0.082 | 0.019 | 0.310 | 9.06E-06 | 6870 |
|  | rs118048608 | 8 | 114265886 | C | T | 0.247 | 0.055 | 0.026 | 6.92E-06 | 6870 |
|  | rs10811102 | 9 | 1911291 | T | G | -0.107 | 0.023 | 0.195 | 2.28E-06 | 6870 |
|  | rs174547 | 11 | 61803311 | C | T | 0.194 | 0.017 | 0.407 | 3.60E-29 | 6870 |
|  | rs10848158 | 12 | 130607421 | C | T | 0.093 | 0.017 | 0.434 | 7.17E-08 | 6870 |
|  | rs1532085 | 15 | 58391167 | G | A | -0.081 | 0.017 | 0.573 | 2.64E-06 | 6870 |
|  | rs261334 | 15 | 58434545 | C | G | -0.104 | 0.020 | 0.758 | 1.91E-07 | 6870 |
|  | rs9652580 | 16 | 24297504 | T | C | -0.080 | 0.018 | 0.423 | 5.02E-06 | 6870 |
|  | rs28415525 | 17 | 70147293 | G | A | -0.129 | 0.029 | 0.095 | 8.81E-06 | 6870 |
|  | rs117511403 | 18 | 35300850 | A | G | 0.697 | 0.143 | 0.004 | 1.12E-06 | 6870 |
|  | rs57684876 | 19 | 44710429 | T | C | 0.102 | 0.022 | 0.187 | 2.64E-06 | 6870 |
|  | rs190712692 | 19 | 44921921 | A | G | 0.245 | 0.051 | 0.030 | 1.51E-06 | 6870 |
|  | rs78605935 | 20 | 45423037 | T | C | 0.157 | 0.035 | 0.066 | 8.09E-06 | 6870 |
| Phosphatidylethanolamine (O-18:1_20:4) | rs1203148 | 1 | 35503298 | G | A | 0.091 | 0.020 | 0.240 | 7.34E-06 | 7172 |
|  | rs114325590 | 1 | 107442367 | T | C | -0.379 | 0.085 | 0.011 | 9.41E-06 | 7172 |
|  | rs3820438 | 1 | 201955621 | C | G | 0.082 | 0.018 | 0.291 | 8.42E-06 | 7172 |
|  | rs72635955 | 1 | 244982806 | T | C | 0.126 | 0.025 | 0.125 | 5.16E-07 | 7172 |
|  | rs71420341 | 2 | 59638953 | G | A | -0.146 | 0.030 | 0.089 | 7.54E-07 | 7172 |
|  | rs1513795 | 2 | 123647385 | C | T | 0.080 | 0.017 | 0.429 | 2.47E-06 | 7172 |
|  | rs11725298 | 4 | 52717255 | C | T | 0.256 | 0.057 | 0.022 | 6.50E-06 | 7172 |
|  | rs10520189 | 4 | 170720084 | A | G | 0.122 | 0.027 | 0.114 | 7.55E-06 | 7172 |
|  | rs10234951 | 7 | 94419222 | G | T | -0.149 | 0.031 | 0.080 | 1.69E-06 | 7172 |
|  | rs2705007 | 7 | 106360522 | G | C | -0.361 | 0.079 | 0.987 | 5.12E-06 | 7172 |
|  | rs6601694 | 8 | 8390548 | A | G | 0.088 | 0.018 | 0.334 | 8.85E-07 | 7172 |
|  | rs311416 | 8 | 54198249 | T | C | -0.079 | 0.018 | 0.649 | 6.58E-06 | 7172 |
|  | rs13269712 | 8 | 69966863 | A | G | 0.105 | 0.023 | 0.160 | 6.82E-06 | 7172 |
|  | rs10756627 | 9 | 14928596 | C | T | 0.075 | 0.017 | 0.551 | 8.04E-06 | 7172 |
|  | rs117002537 | 9 | 130943915 | T | C | 0.291 | 0.065 | 0.018 | 8.52E-06 | 7172 |
|  | rs174527 | 11 | 61770929 | C | G | -0.079 | 0.017 | 0.444 | 3.97E-06 | 7172 |
|  | rs174536 | 11 | 61784455 | C | A | -0.251 | 0.017 | 0.408 | 1.09E-49 | 7172 |
|  | rs2277289 | 11 | 118311945 | C | T | 0.091 | 0.018 | 0.326 | 3.31E-07 | 7172 |
|  | rs11062287 | 12 | 2630538 | A | G | 0.255 | 0.050 | 0.034 | 4.45E-07 | 7172 |
|  | rs113913626 | 12 | 76255614 | T | C | -0.225 | 0.048 | 0.032 | 3.27E-06 | 7172 |
|  | rs17703316 | 13 | 75596558 | T | G | 0.179 | 0.039 | 0.050 | 4.25E-06 | 7172 |
|  | rs4525452 | 15 | 96625932 | A | G | 0.077 | 0.017 | 0.393 | 7.24E-06 | 7172 |
|  | rs117565387 | 16 | 5924670 | T | C | 0.175 | 0.038 | 0.053 | 3.51E-06 | 7172 |
|  | rs35427954 | 16 | 76463097 | C | T | -0.140 | 0.030 | 0.087 | 2.30E-06 | 7172 |
|  | rs145926488 | 18 | 27805383 | T | A | 0.402 | 0.091 | 0.008 | 9.11E-06 | 7172 |
|  | rs1456608 | 18 | 44272134 | G | T | -0.087 | 0.019 | 0.755 | 5.93E-06 | 7172 |
|  | rs141622900 | 19 | 44923535 | A | G | 0.250 | 0.050 | 0.030 | 6.52E-07 | 7172 |
|  | rs204468 | 19 | 44987378 | C | T | -0.095 | 0.017 | 0.595 | 2.35E-08 | 7172 |
|  | rs41376249 | 22 | 36714714 | A | G | -0.112 | 0.024 | 0.146 | 2.08E-06 | 7172 |
| Phosphatidylethanolamine (O-18:2_18:1) | rs17368528 | 1 | 9264154 | T | C | 0.161 | 0.031 | 0.084 | 2.04E-07 | 6717 |
|  | rs148727465 | 1 | 167181238 | A | G | 0.209 | 0.046 | 0.040 | 6.67E-06 | 6717 |
|  | rs112243303 | 1 | 224192928 | T | C | 0.138 | 0.028 | 0.112 | 5.86E-07 | 6717 |
|  | rs116075306 | 1 | 241342846 | G | C | -0.371 | 0.084 | 0.011 | 9.30E-06 | 6717 |
|  | rs79878108 | 1 | 245122916 | T | C | -0.261 | 0.052 | 0.029 | 5.08E-07 | 6717 |
|  | rs35253870 | 2 | 12232463 | G | A | 0.118 | 0.024 | 0.151 | 1.39E-06 | 6717 |
|  | rs112779958 | 2 | 14315428 | C | T | -0.234 | 0.052 | 0.030 | 6.07E-06 | 6717 |
|  | rs192913427 | 2 | 63521077 | A | G | 0.718 | 0.152 | 0.003 | 2.42E-06 | 6717 |
|  | rs6749797 | 2 | 120398486 | A | G | -0.098 | 0.020 | 0.229 | 1.74E-06 | 6717 |
|  | rs4674246 | 2 | 218075695 | T | C | 0.102 | 0.023 | 0.186 | 7.63E-06 | 6717 |
|  | rs12496575 | 3 | 236587 | G | A | -0.090 | 0.019 | 0.693 | 1.65E-06 | 6717 |
|  | rs2271489 | 3 | 39235168 | G | C | -0.172 | 0.032 | 0.081 | 6.67E-08 | 6717 |
|  | rs116140603 | 5 | 180049450 | C | T | 0.576 | 0.130 | 0.004 | 9.21E-06 | 6717 |
|  | rs3752651 | 7 | 55161850 | C | T | 0.115 | 0.025 | 0.136 | 5.07E-06 | 6717 |
|  | rs13257713 | 8 | 112417407 | T | C | -0.119 | 0.025 | 0.139 | 2.06E-06 | 6717 |
|  | rs117049823 | 9 | 2495519 | G | A | 0.489 | 0.110 | 0.006 | 8.55E-06 | 6717 |
|  | rs140484398 | 9 | 102668496 | A | G | 0.519 | 0.106 | 0.007 | 1.02E-06 | 6717 |
|  | rs150046733 | 11 | 732354 | C | T | -0.181 | 0.041 | 0.049 | 8.64E-06 | 6717 |
|  | rs11174276 | 12 | 62005843 | A | G | 0.083 | 0.019 | 0.318 | 7.22E-06 | 6717 |
|  | rs1800588 | 15 | 58431476 | T | C | 0.095 | 0.020 | 0.258 | 1.94E-06 | 6717 |
|  | rs28606541 | 19 | 6934007 | T | C | -0.207 | 0.043 | 0.043 | 1.71E-06 | 6717 |
|  | rs8108277 | 19 | 44702607 | C | T | 0.106 | 0.022 | 0.189 | 1.13E-06 | 6717 |
| Phosphatidylethanolamine (O-18:2_18:2) | rs34683829 | 2 | 216708722 | T | C | -0.104 | 0.022 | 0.206 | 2.36E-06 | 6337 |
|  | rs79087995 | 3 | 114457667 | G | A | -0.271 | 0.061 | 0.022 | 9.55E-06 | 6337 |
|  | rs11936543 | 4 | 151270229 | G | A | -0.136 | 0.030 | 0.100 | 6.52E-06 | 6337 |
|  | rs9686220 | 5 | 178795278 | A | G | -0.564 | 0.125 | 0.006 | 6.50E-06 | 6337 |
|  | rs7459809 | 8 | 139128322 | T | A | -0.686 | 0.144 | 0.004 | 2.08E-06 | 6337 |
|  | rs497849 | 10 | 67993863 | T | C | -0.107 | 0.024 | 0.190 | 5.60E-06 | 6337 |
|  | rs72840788 | 10 | 119656173 | A | G | 0.095 | 0.021 | 0.229 | 8.98E-06 | 6337 |
|  | rs4603290 | 11 | 15301207 | C | G | 0.153 | 0.031 | 0.099 | 5.94E-07 | 6337 |
|  | rs2727270 | 11 | 61835765 | T | C | 0.177 | 0.020 | 0.258 | 2.43E-18 | 6337 |
|  | rs79998109 | 12 | 109070443 | T | C | 0.540 | 0.114 | 0.007 | 2.24E-06 | 6337 |
|  | rs181750334 | 16 | 14645161 | T | A | -0.492 | 0.110 | 0.007 | 8.28E-06 | 6337 |
|  | rs2550882 | 16 | 75515447 | G | C | -0.085 | 0.018 | 0.414 | 2.36E-06 | 6337 |
|  | rs238237 | 17 | 4999663 | A | G | -0.093 | 0.020 | 0.731 | 3.70E-06 | 6337 |
|  | rs11870168 | 17 | 76481609 | T | C | -0.083 | 0.018 | 0.420 | 6.62E-06 | 6337 |
|  | rs186111880 | 19 | 5868331 | T | C | -0.259 | 0.053 | 0.031 | 1.16E-06 | 6337 |
| Phosphatidylethanolamine (O-18:2_20:4) | rs143745495 | 1 | 119172555 | T | C | -0.104 | 0.022 | 0.172 | 2.38E-06 | 7154 |
|  | rs3820438 | 1 | 201955621 | C | G | 0.089 | 0.018 | 0.291 | 1.38E-06 | 7154 |
|  | rs71420341 | 2 | 59638953 | G | A | -0.138 | 0.030 | 0.089 | 3.15E-06 | 7154 |
|  | rs72627458 | 2 | 106808276 | G | T | -0.231 | 0.051 | 0.029 | 6.48E-06 | 7154 |
|  | rs115069241 | 2 | 146766791 | C | T | -0.247 | 0.054 | 0.025 | 5.54E-06 | 7154 |
|  | rs2323249 | 3 | 5474437 | G | A | -0.241 | 0.054 | 0.028 | 8.02E-06 | 7154 |
|  | rs115623452 | 3 | 106127782 | A | G | -0.803 | 0.174 | 0.003 | 4.14E-06 | 7154 |
|  | rs61792775 | 3 | 132262400 | T | C | -0.153 | 0.033 | 0.073 | 3.23E-06 | 7154 |
|  | rs55853808 | 3 | 182336158 | G | A | 0.111 | 0.025 | 0.142 | 9.77E-06 | 7154 |
|  | rs1034755 | 7 | 8811155 | T | C | 0.080 | 0.018 | 0.319 | 7.79E-06 | 7154 |
|  | rs17864186 | 7 | 127303602 | A | C | 0.147 | 0.032 | 0.074 | 4.97E-06 | 7154 |
|  | rs79337987 | 7 | 138155111 | A | T | -0.276 | 0.061 | 0.020 | 5.74E-06 | 7154 |
|  | rs13280051 | 8 | 8374175 | G | A | 0.093 | 0.019 | 0.247 | 1.88E-06 | 7154 |
|  | rs77754835 | 8 | 69988547 | A | G | 0.103 | 0.023 | 0.156 | 9.77E-06 | 7154 |
|  | rs4877665 | 9 | 81622002 | C | A | 0.079 | 0.018 | 0.600 | 7.61E-06 | 7154 |
|  | rs145522126 | 10 | 36183817 | T | C | 0.267 | 0.059 | 0.023 | 6.81E-06 | 7154 |
|  | rs11522703 | 11 | 56943668 | A | G | 0.102 | 0.023 | 0.166 | 7.85E-06 | 7154 |
|  | rs174556 | 11 | 61813163 | T | C | -0.183 | 0.017 | 0.380 | 4.16E-26 | 7154 |
|  | rs9564073 | 13 | 34119843 | A | G | -0.193 | 0.043 | 0.041 | 8.13E-06 | 7154 |
|  | rs17703316 | 13 | 75596558 | T | G | 0.175 | 0.039 | 0.050 | 7.40E-06 | 7154 |
|  | rs181750334 | 16 | 14645161 | T | A | -0.537 | 0.103 | 0.007 | 1.91E-07 | 7154 |
|  | rs80299509 | 16 | 75555515 | A | C | -0.112 | 0.025 | 0.128 | 5.91E-06 | 7154 |
|  | rs71366199 | 17 | 15393870 | G | A | 0.121 | 0.024 | 0.148 | 4.87E-07 | 7154 |
|  | rs9946720 | 18 | 4552061 | G | T | -0.718 | 0.151 | 0.003 | 1.91E-06 | 7154 |
|  | rs7228147 | 18 | 46589554 | C | T | 0.119 | 0.026 | 0.114 | 6.68E-06 | 7154 |
|  | rs204474 | 19 | 44983921 | T | C | -0.095 | 0.017 | 0.598 | 2.58E-08 | 7154 |
|  | rs141451859 | 20 | 47368051 | C | T | -0.182 | 0.040 | 0.047 | 6.90E-06 | 7154 |
| Phosphatidylinositol (16:0_18:1) | rs11208007 | 1 | 62710412 | T | A | -0.086 | 0.019 | 0.249 | 8.47E-06 | 7157 |
|  | rs1918704 | 2 | 10553201 | A | G | 0.103 | 0.023 | 0.840 | 9.15E-06 | 7157 |
|  | rs10490343 | 2 | 45617902 | A | T | -0.079 | 0.017 | 0.362 | 5.90E-06 | 7157 |
|  | rs13113877 | 4 | 59111539 | A | C | -0.078 | 0.017 | 0.378 | 7.13E-06 | 7157 |
|  | rs79107687 | 4 | 80070812 | G | A | -0.145 | 0.032 | 0.082 | 8.22E-06 | 7157 |
|  | rs3103985 | 8 | 118730631 | A | G | -0.119 | 0.027 | 0.882 | 8.20E-06 | 7157 |
|  | rs80310784 | 10 | 52382881 | A | G | 0.168 | 0.036 | 0.060 | 3.44E-06 | 7157 |
|  | rs75678679 | 10 | 62558401 | T | C | 0.243 | 0.055 | 0.025 | 9.53E-06 | 7157 |
|  | rs174556 | 11 | 61813163 | T | C | 0.100 | 0.017 | 0.380 | 8.51E-09 | 7157 |
|  | rs964184 | 11 | 116778201 | C | G | -0.123 | 0.023 | 0.849 | 1.20E-07 | 7157 |
|  | rs76902173 | 12 | 29000949 | G | C | -0.293 | 0.059 | 0.022 | 5.81E-07 | 7157 |
|  | rs9564991 | 13 | 73455183 | T | C | -0.119 | 0.025 | 0.130 | 2.06E-06 | 7157 |
|  | rs1471435 | 16 | 8941267 | C | T | -0.077 | 0.017 | 0.406 | 7.98E-06 | 7157 |
|  | rs117226992 | 16 | 51313598 | G | C | 0.734 | 0.159 | 0.003 | 3.78E-06 | 7157 |
|  | rs6507838 | 18 | 48309616 | C | T | 0.076 | 0.017 | 0.412 | 7.44E-06 | 7157 |
|  | rs1540037 | 18 | 49656294 | G | A | 0.099 | 0.020 | 0.757 | 5.07E-07 | 7157 |
|  | rs641738 | 19 | 54173068 | C | T | -0.117 | 0.017 | 0.593 | 9.74E-12 | 7157 |
|  | rs11704468 | 22 | 47263264 | T | C | 0.165 | 0.036 | 0.059 | 5.05E-06 | 7157 |
| Phosphatidylinositol (16:0_18:2) | rs1748199 | 1 | 62591465 | C | T | -0.113 | 0.019 | 0.262 | 3.20E-09 | 6998 |
|  | rs4665972 | 2 | 27375230 | C | T | -0.089 | 0.017 | 0.620 | 3.19E-07 | 6998 |
|  | rs115506950 | 2 | 36921090 | A | T | 0.222 | 0.046 | 0.036 | 1.46E-06 | 6998 |
|  | rs28675901 | 4 | 108839211 | C | T | 0.124 | 0.025 | 0.128 | 1.11E-06 | 6998 |
|  | rs73127058 | 5 | 77835346 | A | G | -0.313 | 0.068 | 0.016 | 4.47E-06 | 6998 |
|  | rs9344829 | 6 | 64177173 | T | C | 0.086 | 0.017 | 0.373 | 9.08E-07 | 6998 |
|  | rs17427389 | 6 | 150841979 | A | G | 0.108 | 0.021 | 0.191 | 4.96E-07 | 6998 |
|  | rs34777947 | 7 | 105583433 | C | T | 0.103 | 0.022 | 0.189 | 1.99E-06 | 6998 |
|  | rs143705793 | 8 | 226933 | T | C | 0.244 | 0.055 | 0.024 | 8.49E-06 | 6998 |
|  | rs9666759 | 11 | 11262071 | G | T | -0.078 | 0.017 | 0.420 | 5.86E-06 | 6998 |
|  | rs174549 | 11 | 61803910 | A | G | 0.164 | 0.017 | 0.382 | 5.75E-21 | 6998 |
|  | rs10466588 | 11 | 116739533 | G | A | 0.151 | 0.028 | 0.096 | 1.11E-07 | 6998 |
|  | rs2264782 | 12 | 120994800 | T | C | 0.085 | 0.018 | 0.359 | 1.65E-06 | 6998 |
|  | rs184003290 | 13 | 25953992 | C | T | 0.610 | 0.136 | 0.005 | 7.26E-06 | 6998 |
|  | rs57107736 | 16 | 76627797 | T | C | 0.115 | 0.025 | 0.141 | 2.94E-06 | 6998 |
|  | rs141824100 | 16 | 88985800 | T | C | -0.185 | 0.039 | 0.051 | 1.93E-06 | 6998 |
|  | rs514614 | 18 | 362173 | A | G | 0.076 | 0.017 | 0.576 | 9.43E-06 | 6998 |
|  | rs3786247 | 18 | 49592553 | G | T | 0.158 | 0.029 | 0.096 | 5.25E-08 | 6998 |
|  | rs7241918 | 18 | 49634583 | T | G | 0.102 | 0.022 | 0.821 | 3.42E-06 | 6998 |
|  | rs11669648 | 19 | 54038742 | A | G | 0.107 | 0.023 | 0.172 | 2.80E-06 | 6998 |
|  | rs641738 | 19 | 54173068 | C | T | -0.242 | 0.017 | 0.593 | 3.64E-45 | 6998 |
|  | rs2274890 | 20 | 25320386 | A | G | 0.102 | 0.019 | 0.265 | 8.32E-08 | 6998 |
|  | rs117113213 | 20 | 40537052 | A | G | 0.167 | 0.034 | 0.071 | 1.12E-06 | 6998 |
| Phosphatidylinositol (16:0_20:4) | rs111243657 | 1 | 239331091 | C | T | 0.319 | 0.072 | 0.018 | 8.88E-06 | 5519 |
|  | rs115609318 | 2 | 23397292 | T | C | -0.397 | 0.089 | 0.012 | 7.63E-06 | 5519 |
|  | rs1260326 | 2 | 27508073 | C | T | -0.110 | 0.020 | 0.651 | 3.99E-08 | 5519 |
|  | rs114728714 | 2 | 172180344 | A | G | 0.225 | 0.049 | 0.041 | 5.35E-06 | 5519 |
|  | rs7562707 | 2 | 205962661 | G | A | -0.100 | 0.022 | 0.754 | 5.51E-06 | 5519 |
|  | rs12494536 | 3 | 27225555 | G | C | 0.091 | 0.020 | 0.340 | 7.09E-06 | 5519 |
|  | rs73197087 | 3 | 108882807 | C | A | -0.162 | 0.032 | 0.094 | 4.68E-07 | 5519 |
|  | rs9653945 | 3 | 142941864 | A | G | -0.111 | 0.020 | 0.364 | 2.72E-08 | 5519 |
|  | rs6884150 | 5 | 1767659 | G | A | -0.095 | 0.021 | 0.298 | 6.88E-06 | 5519 |
|  | rs141039423 | 6 | 37305084 | A | G | -0.398 | 0.088 | 0.013 | 5.75E-06 | 5519 |
|  | rs144018078 | 6 | 116375468 | G | C | 0.287 | 0.061 | 0.026 | 3.03E-06 | 5519 |
|  | rs7782358 | 7 | 67820 | G | A | -0.098 | 0.020 | 0.593 | 1.25E-06 | 5519 |
|  | rs149878915 | 8 | 541839 | A | G | 0.240 | 0.048 | 0.040 | 6.91E-07 | 5519 |
|  | rs12545907 | 8 | 40568584 | A | G | 0.106 | 0.024 | 0.205 | 9.10E-06 | 5519 |
|  | rs112184984 | 11 | 1669042 | T | C | 0.202 | 0.044 | 0.053 | 5.26E-06 | 5519 |
|  | rs174537 | 11 | 61785208 | T | G | -0.136 | 0.019 | 0.408 | 2.51E-12 | 5519 |
|  | rs145012974 | 11 | 127595711 | A | G | 0.860 | 0.183 | 0.004 | 2.83E-06 | 5519 |
|  | rs76609522 | 12 | 6138757 | T | C | 0.310 | 0.066 | 0.023 | 2.67E-06 | 5519 |
|  | rs4759322 | 12 | 54071880 | G | A | -0.096 | 0.019 | 0.400 | 7.70E-07 | 5519 |
|  | rs148590777 | 16 | 31911781 | A | G | 0.311 | 0.070 | 0.020 | 8.04E-06 | 5519 |
|  | rs12449710 | 17 | 52850260 | C | A | -0.096 | 0.019 | 0.582 | 7.40E-07 | 5519 |
|  | rs11085758 | 19 | 11074338 | G | A | 0.089 | 0.020 | 0.352 | 8.54E-06 | 5519 |
|  | rs8736 | 19 | 54173495 | C | T | 0.104 | 0.020 | 0.595 | 1.17E-07 | 5519 |
| Phosphatidylinositol (18:0_18:1) | rs10889352 | 1 | 62633352 | C | T | -0.160 | 0.019 | 0.262 | 2.22E-17 | 7162 |
|  | rs1340414 | 1 | 102480786 | G | A | 0.083 | 0.018 | 0.646 | 2.24E-06 | 7162 |
|  | rs12122878 | 1 | 189927375 | T | C | -0.086 | 0.019 | 0.289 | 3.98E-06 | 7162 |
|  | rs4846841 | 1 | 230166915 | G | A | 0.091 | 0.019 | 0.747 | 1.85E-06 | 7162 |
|  | rs10490343 | 2 | 45617902 | A | T | -0.077 | 0.017 | 0.362 | 9.45E-06 | 7162 |
|  | rs115510074 | 2 | 141967352 | A | G | -0.210 | 0.047 | 0.034 | 7.06E-06 | 7162 |
|  | rs12638256 | 3 | 142936448 | G | A | 0.160 | 0.017 | 0.632 | 4.88E-20 | 7162 |
|  | rs6813524 | 4 | 27126056 | G | A | -0.077 | 0.017 | 0.392 | 9.64E-06 | 7162 |
|  | rs67835422 | 4 | 71877919 | T | C | -0.088 | 0.018 | 0.277 | 1.72E-06 | 7162 |
|  | rs1560063 | 5 | 5209937 | G | A | 0.082 | 0.018 | 0.328 | 4.51E-06 | 7162 |
|  | rs183478874 | 5 | 176998593 | A | C | -0.248 | 0.056 | 0.024 | 9.41E-06 | 7162 |
|  | rs7742350 | 6 | 14803818 | T | C | -0.190 | 0.042 | 0.044 | 4.85E-06 | 7162 |
|  | rs11783515 | 8 | 58407830 | G | A | -0.088 | 0.017 | 0.626 | 3.02E-07 | 7162 |
|  | rs174556 | 11 | 61813163 | T | C | 0.135 | 0.017 | 0.380 | 5.98E-15 | 7162 |
|  | rs7122803 | 11 | 107883610 | G | C | -0.116 | 0.024 | 0.860 | 1.18E-06 | 7162 |
|  | rs964184 | 11 | 116778201 | C | G | -0.195 | 0.023 | 0.849 | 3.67E-17 | 7162 |
|  | rs2264782 | 12 | 120994800 | T | C | 0.094 | 0.017 | 0.359 | 7.47E-08 | 7162 |
|  | rs12910401 | 15 | 99799692 | C | T | -0.083 | 0.018 | 0.286 | 7.99E-06 | 7162 |
|  | rs149615216 | 18 | 49579658 | T | C | 0.518 | 0.116 | 0.005 | 8.26E-06 | 7162 |
|  | rs1540037 | 18 | 49656294 | G | A | 0.146 | 0.020 | 0.757 | 1.36E-13 | 7162 |
|  | rs4804576 | 19 | 11220678 | T | G | -0.167 | 0.035 | 0.062 | 1.40E-06 | 7162 |
|  | rs641738 | 19 | 54173068 | C | T | -0.225 | 0.017 | 0.593 | 7.56E-40 | 7162 |
|  | rs204015 | 21 | 18058617 | T | C | -0.137 | 0.031 | 0.080 | 8.93E-06 | 7162 |
| Phosphatidylinositol (18:0_18:2) | rs1748199 | 1 | 62591465 | C | T | -0.187 | 0.019 | 0.262 | 1.99E-23 | 7170 |
|  | rs780093 | 2 | 27519736 | C | T | -0.091 | 0.017 | 0.646 | 1.52E-07 | 7170 |
|  | rs7561644 | 2 | 78784774 | T | G | 0.086 | 0.017 | 0.462 | 3.63E-07 | 7170 |
|  | rs7687811 | 4 | 39404797 | G | C | 0.076 | 0.017 | 0.574 | 7.30E-06 | 7170 |
|  | rs72676136 | 4 | 115592282 | G | A | -0.284 | 0.063 | 0.018 | 6.00E-06 | 7170 |
|  | rs146893850 | 4 | 133227651 | T | G | -0.351 | 0.079 | 0.012 | 8.84E-06 | 7170 |
|  | rs72830461 | 6 | 10040346 | C | T | 0.638 | 0.133 | 0.005 | 1.56E-06 | 7170 |
|  | rs915894 | 6 | 32222613 | G | T | -0.086 | 0.018 | 0.326 | 1.07E-06 | 7170 |
|  | rs139001006 | 6 | 160179246 | C | T | 0.738 | 0.163 | 0.003 | 6.26E-06 | 7170 |
|  | rs2713348 | 7 | 9471070 | G | A | 0.081 | 0.018 | 0.698 | 9.26E-06 | 7170 |
|  | rs73107473 | 7 | 44538023 | A | G | 0.094 | 0.021 | 0.197 | 6.83E-06 | 7170 |
|  | rs34777947 | 7 | 105583433 | C | T | 0.102 | 0.021 | 0.189 | 1.74E-06 | 7170 |
|  | rs112713750 | 7 | 135205323 | T | C | -0.267 | 0.058 | 0.020 | 4.58E-06 | 7170 |
|  | rs34372369 | 7 | 143395176 | A | G | -0.132 | 0.029 | 0.090 | 5.79E-06 | 7170 |
|  | rs2081687 | 8 | 58476006 | C | T | -0.080 | 0.017 | 0.618 | 2.67E-06 | 7170 |
|  | rs11780222 | 8 | 66293006 | C | A | 0.079 | 0.018 | 0.318 | 9.98E-06 | 7170 |
|  | rs114488287 | 8 | 84010633 | A | T | -0.263 | 0.057 | 0.022 | 4.11E-06 | 7170 |
|  | rs77470505 | 11 | 61458997 | G | T | 0.311 | 0.063 | 0.018 | 8.70E-07 | 7170 |
|  | rs150684478 | 11 | 61545254 | T | G | 0.308 | 0.057 | 0.024 | 7.46E-08 | 7170 |
|  | rs174566 | 11 | 61824890 | G | A | 0.292 | 0.017 | 0.408 | 3.90E-67 | 7170 |
|  | rs976947 | 11 | 115593068 | A | G | -0.078 | 0.017 | 0.635 | 8.04E-06 | 7170 |
|  | rs964184 | 11 | 116778201 | C | G | -0.239 | 0.023 | 0.849 | 5.59E-25 | 7170 |
|  | rs7979473 | 12 | 120982457 | G | A | -0.110 | 0.017 | 0.577 | 7.38E-11 | 7170 |
|  | rs184003290 | 13 | 25953992 | C | T | 0.608 | 0.135 | 0.005 | 7.25E-06 | 7170 |
|  | rs9551866 | 13 | 30195598 | A | G | -0.084 | 0.018 | 0.302 | 5.27E-06 | 7170 |
|  | rs12147624 | 14 | 73791596 | A | G | 0.135 | 0.030 | 0.082 | 9.03E-06 | 7170 |
|  | rs8033940 | 15 | 58432643 | A | G | 0.091 | 0.018 | 0.337 | 4.14E-07 | 7170 |
|  | rs41429348 | 15 | 73927659 | T | C | 0.183 | 0.039 | 0.049 | 2.29E-06 | 7170 |
|  | rs62039480 | 16 | 15043593 | A | G | 0.085 | 0.018 | 0.332 | 1.50E-06 | 7170 |
|  | rs149615216 | 18 | 49579658 | T | C | 0.642 | 0.116 | 0.005 | 2.88E-08 | 7170 |
|  | rs1540037 | 18 | 49656294 | G | A | 0.155 | 0.020 | 0.757 | 4.09E-15 | 7170 |
|  | rs11669648 | 19 | 54038742 | A | G | 0.113 | 0.023 | 0.172 | 5.71E-07 | 7170 |
|  | rs8736 | 19 | 54173495 | C | T | -0.369 | 0.017 | 0.595 | 4.82E-107 | 7170 |
|  | rs6115094 | 20 | 25230191 | G | A | 0.079 | 0.017 | 0.502 | 2.26E-06 | 7170 |
|  | rs117113213 | 20 | 40537052 | A | G | 0.174 | 0.034 | 0.071 | 2.85E-07 | 7170 |
| Phosphatidylinositol (18:0_20:3) | rs1168104 | 1 | 62662654 | G | A | 0.151 | 0.019 | 0.738 | 6.66E-15 | 6784 |
|  | rs7355249 | 2 | 26361322 | T | C | 0.477 | 0.106 | 0.007 | 7.50E-06 | 6784 |
|  | rs1260326 | 2 | 27508073 | C | T | -0.090 | 0.018 | 0.651 | 5.42E-07 | 6784 |
|  | rs6820980 | 4 | 4988836 | A | G | -0.085 | 0.018 | 0.572 | 1.98E-06 | 6784 |
|  | rs76457276 | 4 | 37896323 | G | A | -0.257 | 0.056 | 0.026 | 4.21E-06 | 6784 |
|  | rs67945876 | 4 | 78054777 | C | T | -0.095 | 0.020 | 0.234 | 3.28E-06 | 6784 |
|  | rs13147779 | 4 | 169287018 | C | T | -0.104 | 0.022 | 0.189 | 2.03E-06 | 6784 |
|  | rs2819082 | 6 | 156379168 | C | T | -0.088 | 0.019 | 0.295 | 2.97E-06 | 6784 |
|  | rs7782961 | 7 | 6295952 | T | A | 0.094 | 0.020 | 0.232 | 4.21E-06 | 6784 |
|  | rs11769084 | 7 | 72363703 | A | G | 0.130 | 0.028 | 0.105 | 3.96E-06 | 6784 |
|  | rs7845664 | 8 | 40712078 | G | A | -0.082 | 0.017 | 0.576 | 2.26E-06 | 6784 |
|  | rs28514538 | 8 | 58485873 | G | A | -0.092 | 0.020 | 0.246 | 3.67E-06 | 6784 |
|  | rs117761170 | 9 | 34672503 | G | A | -0.198 | 0.044 | 0.039 | 7.29E-06 | 6784 |
|  | rs17085148 | 9 | 81886311 | C | T | -0.193 | 0.043 | 0.050 | 6.95E-06 | 6784 |
|  | rs1937711 | 10 | 51855519 | G | C | 0.091 | 0.019 | 0.651 | 1.84E-06 | 6784 |
|  | rs72914920 | 11 | 60782852 | A | G | -0.096 | 0.019 | 0.306 | 3.98E-07 | 6784 |
|  | rs28456 | 11 | 61822009 | G | A | 0.349 | 0.017 | 0.384 | 1.70E-88 | 6784 |
|  | rs10897211 | 11 | 62056104 | A | G | 0.099 | 0.018 | 0.335 | 5.84E-08 | 6784 |
|  | rs16916055 | 11 | 91426863 | C | T | 0.462 | 0.095 | 0.009 | 1.10E-06 | 6784 |
|  | rs976947 | 11 | 115593068 | A | G | -0.081 | 0.018 | 0.635 | 6.42E-06 | 6784 |
|  | rs964184 | 11 | 116778201 | C | G | -0.162 | 0.024 | 0.849 | 1.05E-11 | 6784 |
|  | rs59382088 | 12 | 5886232 | A | G | 0.272 | 0.061 | 0.020 | 8.23E-06 | 6784 |
|  | rs9530145 | 13 | 73071162 | T | C | 0.125 | 0.028 | 0.105 | 8.04E-06 | 6784 |
|  | rs319552 | 13 | 93173799 | C | G | 0.090 | 0.020 | 0.255 | 5.02E-06 | 6784 |
|  | rs59739041 | 15 | 58368884 | C | T | 0.176 | 0.040 | 0.048 | 8.81E-06 | 6784 |
|  | rs6498540 | 16 | 15036737 | G | A | -0.126 | 0.018 | 0.334 | 5.24E-12 | 6784 |
|  | rs12598816 | 16 | 80253481 | T | G | -0.091 | 0.019 | 0.268 | 3.12E-06 | 6784 |
|  | rs80011585 | 16 | 84256448 | T | C | -0.151 | 0.034 | 0.073 | 7.28E-06 | 6784 |
|  | rs145740269 | 17 | 53197241 | C | T | -0.183 | 0.040 | 0.049 | 6.00E-06 | 6784 |
|  | rs12458441 | 18 | 49595788 | C | A | 0.147 | 0.030 | 0.093 | 8.74E-07 | 6784 |
|  | rs117006704 | 18 | 68982283 | G | T | 0.321 | 0.071 | 0.016 | 6.03E-06 | 6784 |
|  | rs117511121 | 19 | 18072285 | T | C | -0.365 | 0.077 | 0.012 | 2.28E-06 | 6784 |
|  | rs8736 | 19 | 54173495 | C | T | 0.222 | 0.017 | 0.595 | 4.35E-37 | 6784 |
| Phosphatidylinositol (18:0_20:4) | rs4912056 | 1 | 18918184 | T | C | 0.114 | 0.023 | 0.152 | 1.02E-06 | 7174 |
|  | rs1168104 | 1 | 62662654 | G | A | 0.194 | 0.019 | 0.738 | 6.30E-25 | 7174 |
|  | rs112944184 | 1 | 239312130 | T | C | 0.303 | 0.064 | 0.017 | 1.92E-06 | 7174 |
|  | rs1260326 | 2 | 27508073 | C | T | -0.096 | 0.017 | 0.651 | 3.06E-08 | 7174 |
|  | rs4629190 | 2 | 68915026 | C | T | 0.083 | 0.017 | 0.487 | 6.30E-07 | 7174 |
|  | rs11899922 | 2 | 137161796 | G | A | 0.163 | 0.035 | 0.060 | 3.46E-06 | 7174 |
|  | rs9848779 | 3 | 142933227 | G | C | -0.094 | 0.017 | 0.631 | 6.90E-08 | 7174 |
|  | rs13068826 | 3 | 171396003 | A | G | -0.080 | 0.017 | 0.587 | 2.02E-06 | 7174 |
|  | rs67945876 | 4 | 78054777 | C | T | -0.094 | 0.020 | 0.234 | 2.11E-06 | 7174 |
|  | rs116730430 | 5 | 35798066 | T | C | -0.261 | 0.058 | 0.022 | 7.11E-06 | 7174 |
|  | rs186923696 | 5 | 106005784 | T | C | 0.276 | 0.062 | 0.020 | 9.94E-06 | 7174 |
|  | rs62396086 | 6 | 26710895 | G | A | 0.082 | 0.018 | 0.351 | 3.15E-06 | 7174 |
|  | rs12213531 | 6 | 156391192 | T | C | 0.076 | 0.017 | 0.508 | 5.56E-06 | 7174 |
|  | rs111265437 | 7 | 28245096 | A | T | 0.391 | 0.087 | 0.010 | 7.87E-06 | 7174 |
|  | rs35332062 | 7 | 73597712 | A | G | -0.129 | 0.025 | 0.122 | 4.08E-07 | 7174 |
|  | rs187491194 | 7 | 80900239 | A | G | -0.206 | 0.046 | 0.036 | 6.85E-06 | 7174 |
|  | rs2725021 | 8 | 4623841 | G | C | 0.084 | 0.017 | 0.507 | 9.12E-07 | 7174 |
|  | rs11780610 | 8 | 18402366 | T | C | -0.092 | 0.018 | 0.680 | 2.25E-07 | 7174 |
|  | rs114051008 | 9 | 69555144 | C | T | 0.351 | 0.079 | 0.012 | 8.37E-06 | 7174 |
|  | rs61872308 | 10 | 112296190 | C | G | -0.144 | 0.031 | 0.085 | 2.65E-06 | 7174 |
|  | rs174533 | 11 | 61781553 | A | G | -0.249 | 0.017 | 0.409 | 3.40E-49 | 7174 |
|  | rs964184 | 11 | 116778201 | C | G | -0.267 | 0.023 | 0.849 | 5.97E-31 | 7174 |
|  | rs2149773 | 13 | 114124757 | T | C | 0.118 | 0.027 | 0.109 | 9.48E-06 | 7174 |
|  | rs10138449 | 14 | 61547116 | C | G | 0.117 | 0.025 | 0.133 | 2.05E-06 | 7174 |
|  | rs9910521 | 17 | 11562403 | A | G | -0.134 | 0.029 | 0.091 | 4.48E-06 | 7174 |
|  | rs12052014 | 18 | 22676206 | T | G | 0.075 | 0.017 | 0.554 | 8.65E-06 | 7174 |
|  | rs56339947 | 18 | 49628409 | C | T | 0.188 | 0.027 | 0.114 | 1.36E-12 | 7174 |
|  | rs6507942 | 18 | 49666690 | A | T | 0.079 | 0.017 | 0.499 | 1.98E-06 | 7174 |
|  | rs17699030 | 19 | 11220266 | G | A | -0.231 | 0.039 | 0.047 | 4.48E-09 | 7174 |
|  | rs10423460 | 19 | 31666796 | G | A | -0.120 | 0.026 | 0.874 | 3.22E-06 | 7174 |
|  | rs144215405 | 19 | 53935764 | T | C | -0.308 | 0.066 | 0.019 | 2.72E-06 | 7174 |
|  | rs8736 | 19 | 54173495 | C | T | 0.275 | 0.017 | 0.595 | 1.26E-59 | 7174 |
|  | rs2425225 | 20 | 36376425 | C | T | 0.078 | 0.017 | 0.630 | 4.87E-06 | 7174 |
|  | rs2032107 | 21 | 34102305 | C | T | 0.155 | 0.034 | 0.064 | 6.31E-06 | 7174 |
| Phosphatidylinositol (18:1_18:1) | rs10889352 | 1 | 62633352 | C | T | -0.154 | 0.019 | 0.262 | 2.63E-16 | 7164 |
|  | rs627108 | 1 | 230174822 | T | C | -0.085 | 0.017 | 0.396 | 6.88E-07 | 7164 |
|  | rs72846591 | 2 | 141712670 | C | T | 0.080 | 0.017 | 0.398 | 3.00E-06 | 7164 |
|  | rs149540646 | 3 | 74470951 | G | A | -0.086 | 0.019 | 0.258 | 6.60E-06 | 7164 |
|  | rs62409317 | 4 | 25950031 | A | G | 0.195 | 0.042 | 0.046 | 3.65E-06 | 7164 |
|  | rs11783515 | 8 | 58407830 | G | A | -0.089 | 0.017 | 0.626 | 2.39E-07 | 7164 |
|  | rs145993592 | 9 | 91769704 | A | C | 0.472 | 0.104 | 0.007 | 5.87E-06 | 7164 |
|  | rs78572804 | 10 | 74877945 | G | A | 0.406 | 0.089 | 0.010 | 5.15E-06 | 7164 |
|  | rs174568 | 11 | 61826344 | T | C | 0.157 | 0.017 | 0.405 | 3.37E-20 | 7164 |
|  | rs964184 | 11 | 116778201 | C | G | -0.166 | 0.023 | 0.849 | 7.65E-13 | 7164 |
|  | rs12428337 | 13 | 22601154 | A | G | 0.089 | 0.019 | 0.238 | 4.56E-06 | 7164 |
|  | rs1751064 | 13 | 95125494 | G | A | -0.088 | 0.020 | 0.760 | 7.79E-06 | 7164 |
|  | rs116845732 | 14 | 38415942 | C | T | 0.715 | 0.139 | 0.004 | 2.86E-07 | 7164 |
|  | rs186422572 | 14 | 103616633 | A | G | 0.347 | 0.075 | 0.013 | 4.08E-06 | 7164 |
|  | rs10468017 | 15 | 58386313 | T | C | 0.089 | 0.018 | 0.338 | 4.59E-07 | 7164 |
|  | rs9652580 | 16 | 24297504 | T | C | -0.080 | 0.017 | 0.423 | 3.30E-06 | 7164 |
|  | rs149615216 | 18 | 49579658 | T | C | 0.525 | 0.116 | 0.005 | 6.17E-06 | 7164 |
|  | rs1540037 | 18 | 49656294 | G | A | 0.165 | 0.020 | 0.757 | 7.66E-17 | 7164 |
|  | rs62095282 | 18 | 69102156 | G | A | 0.121 | 0.027 | 0.121 | 5.08E-06 | 7164 |
|  | rs4804576 | 19 | 11220678 | T | G | -0.178 | 0.035 | 0.062 | 2.66E-07 | 7164 |
|  | rs36634 | 19 | 54142105 | A | G | -0.122 | 0.017 | 0.593 | 6.21E-13 | 7164 |
|  | rs6023985 | 20 | 55260213 | C | T | -0.321 | 0.073 | 0.014 | 9.62E-06 | 7164 |
|  | rs41418546 | 21 | 36261009 | G | T | 0.148 | 0.032 | 0.076 | 4.75E-06 | 7164 |
| Phosphatidylinositol (18:1_18:2) | rs10889352 | 1 | 62633352 | C | T | -0.124 | 0.020 | 0.262 | 4.53E-10 | 6489 |
|  | rs10911365 | 1 | 183589970 | A | G | 0.144 | 0.031 | 0.090 | 3.29E-06 | 6489 |
|  | rs11803708 | 1 | 236637819 | A | G | 0.197 | 0.041 | 0.049 | 1.69E-06 | 6489 |
|  | rs150683971 | 2 | 2513104 | T | C | -0.170 | 0.038 | 0.058 | 9.18E-06 | 6489 |
|  | rs79507869 | 2 | 42173936 | A | C | -0.125 | 0.026 | 0.129 | 1.79E-06 | 6489 |
|  | rs114782597 | 2 | 52445173 | A | G | -0.466 | 0.104 | 0.008 | 8.06E-06 | 6489 |
|  | rs4848460 | 2 | 117641775 | T | C | 0.187 | 0.040 | 0.050 | 3.94E-06 | 6489 |
|  | rs72887719 | 3 | 62442977 | C | G | -0.251 | 0.056 | 0.027 | 8.62E-06 | 6489 |
|  | rs73109128 | 3 | 74310731 | G | C | 0.259 | 0.055 | 0.028 | 2.20E-06 | 6489 |
|  | rs75519325 | 3 | 196572573 | A | G | 0.162 | 0.035 | 0.069 | 5.23E-06 | 6489 |
|  | rs17074477 | 5 | 172222516 | G | A | -0.140 | 0.028 | 0.109 | 6.51E-07 | 6489 |
|  | rs117234964 | 8 | 99590810 | T | C | 0.134 | 0.030 | 0.099 | 7.08E-06 | 6489 |
|  | rs174568 | 11 | 61826344 | T | C | 0.161 | 0.018 | 0.405 | 1.47E-19 | 6489 |
|  | rs964184 | 11 | 116778201 | C | G | -0.123 | 0.024 | 0.849 | 4.28E-07 | 6489 |
|  | rs144480172 | 12 | 32548599 | T | C | 0.279 | 0.057 | 0.025 | 8.07E-07 | 6489 |
|  | rs2701185 | 12 | 120915171 | G | A | 0.080 | 0.018 | 0.539 | 5.94E-06 | 6489 |
|  | rs72753955 | 15 | 82771066 | G | A | -0.417 | 0.088 | 0.011 | 2.09E-06 | 6489 |
|  | rs1544531 | 16 | 5592867 | A | C | 0.082 | 0.017 | 0.517 | 2.37E-06 | 6489 |
|  | rs12103149 | 16 | 24662577 | A | G | -0.139 | 0.030 | 0.091 | 5.11E-06 | 6489 |
|  | rs12943438 | 17 | 21216552 | C | G | 0.256 | 0.057 | 0.026 | 6.16E-06 | 6489 |
|  | rs211991 | 17 | 36639734 | T | C | -0.095 | 0.021 | 0.261 | 6.38E-06 | 6489 |
|  | rs72884421 | 18 | 13582870 | C | G | -0.168 | 0.036 | 0.066 | 2.40E-06 | 6489 |
|  | rs9953437 | 18 | 49594230 | A | G | 0.154 | 0.030 | 0.096 | 2.91E-07 | 6489 |
|  | rs17699030 | 19 | 11220266 | G | A | -0.188 | 0.042 | 0.047 | 6.79E-06 | 6489 |
|  | rs365653 | 19 | 44858389 | G | A | -0.141 | 0.031 | 0.091 | 4.80E-06 | 6489 |
|  | rs8736 | 19 | 54173495 | C | T | -0.131 | 0.018 | 0.595 | 2.45E-13 | 6489 |
| Phosphatidylinositol (18:1_20:4) | rs1168127 | 1 | 62670407 | A | C | 0.105 | 0.021 | 0.737 | 5.26E-07 | 5882 |
|  | rs117960039 | 1 | 97483748 | G | A | 0.250 | 0.056 | 0.029 | 7.25E-06 | 5882 |
|  | rs4920209 | 1 | 234028770 | A | G | 0.088 | 0.018 | 0.475 | 1.63E-06 | 5882 |
|  | rs12467458 | 2 | 213973289 | G | T | 0.126 | 0.028 | 0.126 | 6.52E-06 | 5882 |
|  | rs140854080 | 4 | 87407252 | A | G | 0.370 | 0.078 | 0.014 | 2.47E-06 | 5882 |
|  | rs2379249 | 5 | 17631738 | T | G | -0.162 | 0.036 | 0.927 | 8.22E-06 | 5882 |
|  | rs114918906 | 5 | 174699125 | T | C | 0.193 | 0.044 | 0.049 | 9.38E-06 | 5882 |
|  | rs55976852 | 6 | 94237667 | C | A | -0.099 | 0.021 | 0.274 | 1.92E-06 | 5882 |
|  | rs2819083 | 6 | 156379682 | A | G | -0.092 | 0.018 | 0.486 | 4.92E-07 | 5882 |
|  | rs36061340 | 8 | 100795002 | T | C | 0.194 | 0.041 | 0.052 | 2.94E-06 | 5882 |
|  | rs12554791 | 9 | 31860328 | G | T | 0.136 | 0.030 | 0.110 | 6.69E-06 | 5882 |
|  | rs13291383 | 9 | 133897690 | C | T | 0.096 | 0.021 | 0.276 | 7.48E-06 | 5882 |
|  | rs74559802 | 10 | 6185479 | A | G | -0.432 | 0.097 | 0.011 | 9.56E-06 | 5882 |
|  | rs670975 | 11 | 76222144 | T | A | 0.173 | 0.038 | 0.067 | 4.20E-06 | 5882 |
|  | rs3741298 | 11 | 116786845 | T | C | -0.127 | 0.022 | 0.765 | 4.35E-09 | 5882 |
|  | rs61915733 | 12 | 18171023 | A | G | 0.152 | 0.032 | 0.090 | 2.09E-06 | 5882 |
|  | rs111645166 | 13 | 31552770 | A | G | -0.465 | 0.105 | 0.009 | 9.76E-06 | 5882 |
|  | rs74572381 | 14 | 35052659 | A | G | 0.173 | 0.039 | 0.062 | 8.84E-06 | 5882 |
|  | rs117598891 | 14 | 95031978 | C | T | -0.482 | 0.105 | 0.008 | 4.91E-06 | 5882 |
|  | rs10775270 | 16 | 10136125 | C | A | -0.096 | 0.022 | 0.753 | 9.48E-06 | 5882 |
|  | rs117210444 | 16 | 73859072 | G | A | -0.287 | 0.050 | 0.034 | 9.19E-09 | 5882 |
|  | rs12938892 | 17 | 10103775 | G | A | -0.087 | 0.019 | 0.437 | 6.54E-06 | 5882 |
|  | rs957142 | 18 | 49619347 | A | G | 0.152 | 0.032 | 0.094 | 2.24E-06 | 5882 |
|  | rs75166839 | 18 | 73825578 | G | A | 0.301 | 0.065 | 0.022 | 3.70E-06 | 5882 |
|  | rs146290629 | 19 | 2402297 | T | C | -0.302 | 0.068 | 0.021 | 8.49E-06 | 5882 |
|  | rs599859 | 19 | 52584863 | C | A | -0.089 | 0.019 | 0.394 | 4.86E-06 | 5882 |
|  | rs4822502 | 22 | 24485813 | T | C | 0.084 | 0.019 | 0.545 | 5.54E-06 | 5882 |
| Sphingomyelin (d32:1) | rs11591147 | 1 | 55039974 | T | G | -0.233 | 0.047 | 0.033 | 5.44E-07 | 7174 |
|  | rs16851199 | 1 | 230244207 | G | A | -0.188 | 0.042 | 0.043 | 9.37E-06 | 7174 |
|  | rs12622488 | 2 | 47216823 | T | C | -0.120 | 0.026 | 0.113 | 5.02E-06 | 7174 |
|  | rs10192465 | 2 | 57531720 | C | G | -0.086 | 0.019 | 0.721 | 5.41E-06 | 7174 |
|  | rs2553631 | 2 | 124364198 | T | A | -0.151 | 0.033 | 0.070 | 4.24E-06 | 7174 |
|  | rs7594469 | 2 | 209438053 | C | G | 0.074 | 0.017 | 0.501 | 7.05E-06 | 7174 |
|  | rs6768493 | 3 | 4819419 | C | G | 0.079 | 0.017 | 0.525 | 2.27E-06 | 7174 |
|  | rs6444176 | 3 | 186868271 | T | C | 0.180 | 0.038 | 0.052 | 1.59E-06 | 7174 |
|  | rs75418091 | 4 | 4501634 | T | C | 0.406 | 0.079 | 0.012 | 3.03E-07 | 7174 |
|  | rs2604631 | 4 | 60937521 | A | G | -0.090 | 0.019 | 0.754 | 4.03E-06 | 7174 |
|  | rs12192157 | 6 | 169320808 | T | C | 0.079 | 0.017 | 0.465 | 2.78E-06 | 7174 |
|  | rs10975312 | 9 | 5837780 | A | G | 0.246 | 0.049 | 0.031 | 4.55E-07 | 7174 |
|  | rs58001441 | 9 | 6764828 | A | G | -0.104 | 0.023 | 0.165 | 4.21E-06 | 7174 |
|  | rs10968815 | 9 | 28896200 | G | A | 0.130 | 0.029 | 0.092 | 8.82E-06 | 7174 |
|  | rs1218344 | 10 | 14269684 | C | A | 0.100 | 0.022 | 0.170 | 7.92E-06 | 7174 |
|  | rs3741111 | 11 | 124076588 | G | A | 0.082 | 0.017 | 0.483 | 1.02E-06 | 7174 |
|  | rs61927425 | 12 | 79722154 | T | C | -0.221 | 0.049 | 0.033 | 5.53E-06 | 7174 |
|  | rs10717199 | 12 | 106336256 | T | C | -0.095 | 0.021 | 0.197 | 7.30E-06 | 7174 |
|  | rs61977690 | 14 | 22820083 | G | C | 0.282 | 0.063 | 0.020 | 7.03E-06 | 7174 |
|  | rs7157785 | 14 | 63768838 | T | G | 0.513 | 0.024 | 0.125 | 3.08E-95 | 7174 |
|  | rs8017356 | 14 | 65115063 | G | A | -0.348 | 0.072 | 0.986 | 1.29E-06 | 7174 |
|  | rs10468017 | 15 | 58386313 | T | C | 0.083 | 0.017 | 0.338 | 1.82E-06 | 7174 |
|  | rs72649550 | 15 | 93611333 | C | G | 0.082 | 0.018 | 0.332 | 4.73E-06 | 7174 |
|  | rs117510063 | 16 | 55539036 | G | A | -0.584 | 0.127 | 0.005 | 4.59E-06 | 7174 |
|  | rs217184 | 16 | 72072066 | C | T | -0.096 | 0.020 | 0.228 | 1.49E-06 | 7174 |
|  | rs75679663 | 17 | 4764677 | A | C | -0.256 | 0.054 | 0.026 | 1.86E-06 | 7174 |
|  | rs3026120 | 17 | 5384095 | A | G | -0.175 | 0.031 | 0.075 | 2.14E-08 | 7174 |
|  | rs118068660 | 19 | 11079868 | T | C | -0.153 | 0.028 | 0.095 | 5.34E-08 | 7174 |
|  | rs7247433 | 19 | 19684751 | A | C | -0.080 | 0.017 | 0.394 | 4.21E-06 | 7174 |
|  | rs364585 | 20 | 12982070 | G | A | -0.110 | 0.018 | 0.661 | 4.03E-10 | 7174 |
|  | rs138440819 | 20 | 33730205 | A | G | -0.328 | 0.070 | 0.015 | 2.89E-06 | 7174 |
|  | rs76596580 | 21 | 34990612 | T | C | 0.119 | 0.026 | 0.115 | 6.91E-06 | 7174 |
| Sphingomyelin (d34:0) | rs11591147 | 1 | 55039974 | T | G | -0.256 | 0.053 | 0.033 | 1.33E-06 | 6207 |
|  | rs6683774 | 1 | 198625465 | T | C | 0.178 | 0.037 | 0.934 | 1.17E-06 | 6207 |
|  | rs17497891 | 1 | 222184468 | G | A | 0.119 | 0.025 | 0.150 | 2.51E-06 | 6207 |
|  | rs934198 | 2 | 21055901 | T | G | 0.098 | 0.020 | 0.297 | 8.40E-07 | 6207 |
|  | rs114372832 | 2 | 75724208 | C | G | -0.300 | 0.067 | 0.020 | 7.69E-06 | 6207 |
|  | rs55811539 | 3 | 113313552 | C | T | -0.399 | 0.084 | 0.012 | 1.83E-06 | 6207 |
|  | rs112585713 | 4 | 72876701 | A | G | 0.262 | 0.051 | 0.032 | 2.96E-07 | 6207 |
|  | rs16850360 | 4 | 74006728 | G | A | 0.227 | 0.050 | 0.033 | 6.51E-06 | 6207 |
|  | rs140560087 | 7 | 11194365 | G | A | -0.377 | 0.085 | 0.012 | 9.31E-06 | 6207 |
|  | rs7814780 | 8 | 122227701 | T | G | -0.087 | 0.019 | 0.328 | 6.75E-06 | 6207 |
|  | rs35275531 | 8 | 142178314 | A | C | -0.269 | 0.059 | 0.026 | 5.38E-06 | 6207 |
|  | rs1324162 | 9 | 80858018 | G | T | 0.090 | 0.020 | 0.271 | 7.81E-06 | 6207 |
|  | rs2808569 | 9 | 98537277 | C | A | -0.098 | 0.021 | 0.242 | 3.51E-06 | 6207 |
|  | rs80130206 | 10 | 106229406 | T | C | -0.182 | 0.039 | 0.055 | 2.99E-06 | 6207 |
|  | rs113312978 | 11 | 59731240 | A | G | -0.645 | 0.146 | 0.004 | 9.79E-06 | 6207 |
|  | rs174535 | 11 | 61783884 | C | T | -0.098 | 0.018 | 0.409 | 1.14E-07 | 6207 |
|  | rs12880341 | 14 | 63769473 | C | T | -0.327 | 0.027 | 0.125 | 3.94E-33 | 6207 |
|  | rs11631073 | 15 | 57322347 | T | C | -0.091 | 0.019 | 0.618 | 1.11E-06 | 6207 |
|  | rs55985238 | 17 | 3769209 | A | T | -0.560 | 0.101 | 0.009 | 2.72E-08 | 6207 |
|  | rs75679663 | 17 | 4764677 | A | C | -0.689 | 0.062 | 0.026 | 3.31E-28 | 6207 |
|  | rs3026120 | 17 | 5384095 | A | G | -0.207 | 0.034 | 0.075 | 1.44E-09 | 6207 |
|  | rs8071514 | 17 | 80990283 | A | C | 0.085 | 0.018 | 0.569 | 2.80E-06 | 6207 |
|  | rs73015021 | 19 | 11082239 | G | A | -0.153 | 0.032 | 0.092 | 1.32E-06 | 6207 |
|  | rs61751862 | 19 | 48629610 | C | G | -0.241 | 0.053 | 0.031 | 5.67E-06 | 6207 |
|  | rs1073042 | 20 | 11012603 | G | A | -0.082 | 0.018 | 0.397 | 6.87E-06 | 6207 |
|  | rs595587 | 21 | 43371358 | C | T | 0.090 | 0.019 | 0.340 | 2.56E-06 | 6207 |
|  | rs1989780 | 22 | 19694202 | A | G | -0.301 | 0.068 | 0.020 | 9.27E-06 | 6207 |
| Sphingomyelin (d34:1) | rs11591147 | 1 | 55039974 | T | G | -0.419 | 0.046 | 0.033 | 2.32E-19 | 7174 |
|  | rs1934410 | 1 | 180552628 | T | G | 0.082 | 0.017 | 0.627 | 2.86E-06 | 7174 |
|  | rs7575840 | 2 | 21050618 | T | G | 0.100 | 0.018 | 0.296 | 5.50E-08 | 7174 |
|  | rs4954960 | 2 | 138816573 | C | T | 0.084 | 0.019 | 0.738 | 9.90E-06 | 7174 |
|  | rs34087767 | 2 | 181902451 | G | C | 0.598 | 0.125 | 0.005 | 1.78E-06 | 7174 |
|  | rs74915447 | 4 | 72899760 | A | G | 0.288 | 0.044 | 0.038 | 7.50E-11 | 7174 |
|  | rs114483871 | 4 | 73124451 | T | C | 0.282 | 0.045 | 0.038 | 3.24E-10 | 7174 |
|  | rs116302332 | 4 | 74505174 | T | C | 0.377 | 0.051 | 0.028 | 1.28E-13 | 7174 |
|  | rs35577358 | 4 | 162543049 | G | A | 0.126 | 0.025 | 0.135 | 2.84E-07 | 7174 |
|  | rs2306619 | 5 | 126552164 | G | A | -0.101 | 0.021 | 0.183 | 2.14E-06 | 7174 |
|  | rs7012814 | 8 | 9315848 | A | G | 0.080 | 0.017 | 0.512 | 2.09E-06 | 7174 |
|  | rs78143047 | 9 | 5527351 | G | A | 0.343 | 0.070 | 0.015 | 1.19E-06 | 7174 |
|  | rs7032795 | 9 | 114372665 | C | T | 0.084 | 0.017 | 0.481 | 4.47E-07 | 7174 |
|  | rs600038 | 9 | 133276354 | T | C | -0.089 | 0.020 | 0.778 | 8.70E-06 | 7174 |
|  | rs2894147 | 10 | 71241186 | G | A | -0.076 | 0.017 | 0.374 | 9.47E-06 | 7174 |
|  | rs4246215 | 11 | 61796827 | T | G | -0.075 | 0.017 | 0.436 | 8.47E-06 | 7174 |
|  | rs3912622 | 11 | 103250744 | C | A | -0.093 | 0.021 | 0.200 | 9.57E-06 | 7174 |
|  | rs117279275 | 12 | 52892255 | A | G | -0.334 | 0.069 | 0.014 | 1.52E-06 | 7174 |
|  | rs191640693 | 13 | 47201342 | T | C | -0.244 | 0.053 | 0.027 | 4.13E-06 | 7174 |
|  | rs722814 | 14 | 69889451 | A | C | 0.142 | 0.031 | 0.079 | 4.74E-06 | 7174 |
|  | rs56108088 | 14 | 86739725 | C | T | 0.077 | 0.017 | 0.563 | 4.11E-06 | 7174 |
|  | rs7350789 | 15 | 58387469 | A | G | 0.110 | 0.017 | 0.393 | 6.71E-11 | 7174 |
|  | rs141286087 | 16 | 51029992 | G | A | 0.219 | 0.045 | 0.041 | 1.31E-06 | 7174 |
|  | rs72794954 | 16 | 76342662 | T | C | -0.499 | 0.105 | 0.007 | 1.93E-06 | 7174 |
|  | rs75679663 | 17 | 4764677 | A | C | -0.304 | 0.054 | 0.026 | 1.50E-08 | 7174 |
|  | rs3026120 | 17 | 5384095 | A | G | -0.162 | 0.031 | 0.075 | 2.01E-07 | 7174 |
|  | rs118068660 | 19 | 11079868 | T | C | -0.244 | 0.028 | 0.095 | 4.10E-18 | 7174 |
|  | rs7257703 | 19 | 41030717 | G | A | -0.077 | 0.017 | 0.630 | 9.08E-06 | 7174 |
|  | rs7412 | 19 | 44908822 | T | C | -0.199 | 0.037 | 0.053 | 9.41E-08 | 7174 |
|  | rs1800961 | 20 | 44413724 | T | C | -0.189 | 0.037 | 0.052 | 3.93E-07 | 7174 |
|  | rs2836524 | 21 | 38573905 | A | G | 0.167 | 0.036 | 0.060 | 2.90E-06 | 7174 |
| Sphingomyelin (d34:2) | rs56163454 | 1 | 44173695 | G | C | 0.267 | 0.059 | 0.020 | 5.58E-06 | 7174 |
|  | rs11591147 | 1 | 55039974 | T | G | -0.246 | 0.047 | 0.033 | 1.27E-07 | 7174 |
|  | rs9835223 | 3 | 186868495 | G | C | -0.095 | 0.021 | 0.801 | 6.54E-06 | 7174 |
|  | rs2531191 | 4 | 10615596 | T | C | 0.371 | 0.083 | 0.011 | 8.23E-06 | 7174 |
|  | rs74915447 | 4 | 72899760 | A | G | 0.196 | 0.044 | 0.038 | 9.99E-06 | 7174 |
|  | rs116302332 | 4 | 74505174 | T | C | 0.298 | 0.051 | 0.028 | 4.74E-09 | 7174 |
|  | rs10471676 | 5 | 66284563 | G | C | -0.092 | 0.020 | 0.226 | 6.75E-06 | 7174 |
|  | rs146136464 | 5 | 147384423 | T | C | 0.793 | 0.176 | 0.003 | 6.79E-06 | 7174 |
|  | rs62412280 | 6 | 67724705 | T | A | -0.656 | 0.140 | 0.004 | 2.97E-06 | 7174 |
|  | rs7787163 | 7 | 89538067 | T | C | 0.477 | 0.103 | 0.007 | 3.33E-06 | 7174 |
|  | rs1404846 | 7 | 110130812 | C | T | 0.696 | 0.154 | 0.003 | 6.24E-06 | 7174 |
|  | rs7012814 | 8 | 9315848 | A | G | 0.087 | 0.017 | 0.512 | 2.67E-07 | 7174 |
|  | rs10975312 | 9 | 5837780 | A | G | 0.237 | 0.049 | 0.031 | 1.18E-06 | 7174 |
|  | rs10867632 | 9 | 80862163 | G | C | 0.092 | 0.021 | 0.207 | 8.84E-06 | 7174 |
|  | rs113127305 | 10 | 70837164 | A | G | -0.217 | 0.038 | 0.050 | 1.46E-08 | 7174 |
|  | rs174561 | 11 | 61815236 | C | T | -0.170 | 0.017 | 0.381 | 7.26E-23 | 7174 |
|  | rs3912622 | 11 | 103250744 | C | A | -0.093 | 0.021 | 0.200 | 9.05E-06 | 7174 |
|  | rs117009181 | 12 | 124846478 | C | T | 0.110 | 0.023 | 0.150 | 2.03E-06 | 7174 |
|  | rs111507730 | 13 | 33753876 | C | T | 0.171 | 0.037 | 0.054 | 3.33E-06 | 7174 |
|  | rs17276940 | 14 | 20017956 | T | C | -0.214 | 0.046 | 0.034 | 3.48E-06 | 7174 |
|  | rs1033030 | 14 | 99309314 | A | G | -0.105 | 0.022 | 0.176 | 2.16E-06 | 7174 |
|  | rs1532085 | 15 | 58391167 | G | A | -0.109 | 0.017 | 0.573 | 7.57E-11 | 7174 |
|  | rs939971 | 15 | 80029927 | A | C | -0.096 | 0.020 | 0.211 | 2.43E-06 | 7174 |
|  | rs12952341 | 17 | 4482208 | T | C | 0.085 | 0.019 | 0.326 | 4.84E-06 | 7174 |
|  | rs113722226 | 19 | 11078596 | C | T | -0.186 | 0.028 | 0.094 | 3.70E-11 | 7174 |
|  | rs7247433 | 19 | 19684751 | A | C | -0.078 | 0.017 | 0.394 | 7.82E-06 | 7174 |
|  | rs61751862 | 19 | 48629610 | C | G | 0.253 | 0.048 | 0.031 | 1.39E-07 | 7174 |
|  | rs1800961 | 20 | 44413724 | T | C | -0.168 | 0.037 | 0.052 | 5.97E-06 | 7174 |
| Sphingomyelin (d36:1) | rs11591147 | 1 | 55039974 | T | G | -0.259 | 0.047 | 0.033 | 2.96E-08 | 7174 |
|  | rs1452773 | 2 | 50050811 | G | T | -0.117 | 0.024 | 0.138 | 1.10E-06 | 7174 |
|  | rs12464353 | 2 | 60982893 | A | G | 0.229 | 0.048 | 0.033 | 1.74E-06 | 7174 |
|  | rs886813 | 2 | 241457278 | T | G | 0.081 | 0.017 | 0.468 | 1.61E-06 | 7174 |
|  | rs73073606 | 3 | 194176577 | C | A | -0.090 | 0.020 | 0.241 | 8.25E-06 | 7174 |
|  | rs112585713 | 4 | 72876701 | A | G | 0.282 | 0.048 | 0.032 | 4.41E-09 | 7174 |
|  | rs79146711 | 4 | 73424911 | T | C | 0.234 | 0.041 | 0.045 | 1.02E-08 | 7174 |
|  | rs182695896 | 4 | 73947510 | C | A | 0.339 | 0.053 | 0.025 | 1.58E-10 | 7174 |
|  | rs6829637 | 4 | 110405753 | G | A | -0.079 | 0.017 | 0.416 | 4.77E-06 | 7174 |
|  | rs114839471 | 5 | 90249218 | G | A | -0.445 | 0.095 | 0.008 | 2.59E-06 | 7174 |
|  | rs884532 | 5 | 174239481 | T | C | -0.075 | 0.017 | 0.510 | 6.95E-06 | 7174 |
|  | rs4144237 | 6 | 2365034 | G | A | -0.113 | 0.025 | 0.126 | 8.50E-06 | 7174 |
|  | rs17664981 | 6 | 13187371 | C | T | 0.167 | 0.037 | 0.057 | 5.34E-06 | 7174 |
|  | rs374071816 | 6 | 160682773 | A | T | 0.161 | 0.036 | 0.057 | 9.69E-06 | 7174 |
|  | rs12675806 | 8 | 4789612 | A | G | 0.126 | 0.026 | 0.112 | 1.90E-06 | 7174 |
|  | rs6558122 | 8 | 29518250 | C | T | -0.140 | 0.028 | 0.101 | 4.58E-07 | 7174 |
|  | rs11779209 | 8 | 71073538 | G | T | 0.114 | 0.026 | 0.126 | 9.39E-06 | 7174 |
|  | rs581080 | 9 | 15305380 | C | G | 0.114 | 0.024 | 0.856 | 1.47E-06 | 7174 |
|  | rs117442205 | 11 | 4966841 | A | G | 0.609 | 0.138 | 0.004 | 9.71E-06 | 7174 |
|  | rs10838826 | 11 | 48198260 | T | G | 0.092 | 0.020 | 0.783 | 5.13E-06 | 7174 |
|  | rs4246215 | 11 | 61796827 | T | G | -0.075 | 0.017 | 0.436 | 8.88E-06 | 7174 |
|  | rs3742023 | 12 | 109256177 | T | C | -0.077 | 0.017 | 0.423 | 5.63E-06 | 7174 |
|  | rs1169306 | 12 | 121000508 | T | C | -0.085 | 0.017 | 0.360 | 9.27E-07 | 7174 |
|  | rs4981443 | 14 | 22831454 | A | G | -0.077 | 0.017 | 0.360 | 9.63E-06 | 7174 |
|  | rs7157785 | 14 | 63768838 | T | G | 0.136 | 0.025 | 0.125 | 6.56E-08 | 7174 |
|  | rs138524673 | 15 | 68380799 | A | G | 0.193 | 0.042 | 0.045 | 5.75E-06 | 7174 |
|  | rs186039163 | 17 | 3547599 | G | T | -0.416 | 0.084 | 0.010 | 7.74E-07 | 7174 |
|  | rs75679663 | 17 | 4764677 | A | C | -0.325 | 0.054 | 0.026 | 1.42E-09 | 7174 |
|  | rs117643293 | 17 | 5065669 | T | C | -0.171 | 0.034 | 0.062 | 5.50E-07 | 7174 |
|  | rs72898481 | 17 | 77755768 | T | C | 0.286 | 0.064 | 0.019 | 6.72E-06 | 7174 |
|  | rs2336171 | 19 | 8209156 | C | G | 0.195 | 0.017 | 0.399 | 2.94E-30 | 7174 |
|  | rs7248003 | 19 | 8236164 | T | C | 0.145 | 0.019 | 0.721 | 2.69E-14 | 7174 |
|  | rs12462619 | 19 | 8607097 | C | T | -0.108 | 0.019 | 0.261 | 2.32E-08 | 7174 |
|  | rs364585 | 20 | 12982070 | G | A | -0.109 | 0.018 | 0.661 | 5.55E-10 | 7174 |
|  | rs4809845 | 20 | 51460945 | C | T | 0.129 | 0.025 | 0.134 | 2.41E-07 | 7174 |
|  | rs8139868 | 22 | 17172477 | T | C | -0.088 | 0.019 | 0.257 | 5.04E-06 | 7174 |
| Sphingomyelin (d36:2) | rs1452773 | 2 | 50050811 | G | T | -0.109 | 0.024 | 0.138 | 5.98E-06 | 7173 |
|  | rs12464353 | 2 | 60982893 | A | G | 0.221 | 0.048 | 0.033 | 4.04E-06 | 7173 |
|  | rs13005731 | 2 | 64114191 | G | T | -0.504 | 0.113 | 0.006 | 7.86E-06 | 7173 |
|  | rs78999781 | 2 | 203425314 | C | T | -0.148 | 0.029 | 0.091 | 3.95E-07 | 7173 |
|  | rs146465816 | 3 | 19323998 | A | G | -0.361 | 0.081 | 0.011 | 8.07E-06 | 7173 |
|  | rs6444176 | 3 | 186868271 | T | C | 0.175 | 0.038 | 0.052 | 3.22E-06 | 7173 |
|  | rs77645768 | 4 | 72803111 | A | G | 0.243 | 0.054 | 0.026 | 6.45E-06 | 7173 |
|  | rs182695896 | 4 | 73947510 | C | A | 0.303 | 0.053 | 0.025 | 1.12E-08 | 7173 |
|  | rs313950 | 4 | 113100013 | A | G | -0.076 | 0.017 | 0.396 | 7.32E-06 | 7173 |
|  | rs146253188 | 5 | 35225964 | A | G | -0.288 | 0.064 | 0.017 | 8.07E-06 | 7173 |
|  | rs7700432 | 5 | 130859001 | A | G | -0.079 | 0.017 | 0.354 | 4.55E-06 | 7173 |
|  | rs12207488 | 6 | 10952103 | A | G | -0.096 | 0.022 | 0.182 | 8.21E-06 | 7173 |
|  | rs117546336 | 6 | 98098087 | G | T | -0.190 | 0.040 | 0.047 | 2.00E-06 | 7173 |
|  | rs581080 | 9 | 15305380 | C | G | 0.108 | 0.024 | 0.856 | 5.20E-06 | 7173 |
|  | rs7920200 | 10 | 70899547 | A | G | -0.188 | 0.042 | 0.042 | 8.75E-06 | 7173 |
|  | rs174544 | 11 | 61800281 | A | C | -0.178 | 0.017 | 0.380 | 5.30E-25 | 7173 |
|  | rs9738226 | 12 | 120985856 | G | A | 0.101 | 0.017 | 0.579 | 2.37E-09 | 7173 |
|  | rs17276940 | 14 | 20017956 | T | C | -0.219 | 0.046 | 0.034 | 2.12E-06 | 7173 |
|  | rs72879665 | 18 | 13203131 | A | G | 0.382 | 0.083 | 0.011 | 4.73E-06 | 7173 |
|  | rs7253584 | 19 | 8222034 | C | T | 0.181 | 0.018 | 0.675 | 2.48E-24 | 7173 |
|  | rs7247304 | 19 | 8602475 | T | C | -0.103 | 0.019 | 0.267 | 9.07E-08 | 7173 |
|  | rs68137603 | 20 | 37965215 | T | C | 0.153 | 0.035 | 0.062 | 9.80E-06 | 7173 |
|  | rs148860058 | 22 | 48612019 | A | G | 0.401 | 0.087 | 0.011 | 4.37E-06 | 7173 |
| Sphingomyelin (d38:1) | rs76174849 | 1 | 8738706 | T | C | 0.334 | 0.072 | 0.014 | 3.44E-06 | 7174 |
|  | rs11591147 | 1 | 55039974 | T | G | -0.301 | 0.046 | 0.033 | 1.07E-10 | 7174 |
|  | rs1452773 | 2 | 50050811 | G | T | -0.110 | 0.024 | 0.138 | 4.26E-06 | 7174 |
|  | rs200949196 | 2 | 146363675 | T | G | 0.303 | 0.064 | 0.018 | 2.03E-06 | 7174 |
|  | rs188346987 | 2 | 157097431 | G | C | -0.178 | 0.039 | 0.051 | 6.67E-06 | 7174 |
|  | rs73193962 | 3 | 105974935 | A | C | -0.243 | 0.054 | 0.026 | 6.41E-06 | 7174 |
|  | rs73073606 | 3 | 194176577 | C | A | -0.096 | 0.020 | 0.241 | 1.68E-06 | 7174 |
|  | rs77645768 | 4 | 72803111 | A | G | 0.304 | 0.054 | 0.026 | 1.53E-08 | 7174 |
|  | rs114483871 | 4 | 73124451 | T | C | 0.268 | 0.045 | 0.038 | 2.19E-09 | 7174 |
|  | rs182695896 | 4 | 73947510 | C | A | 0.371 | 0.053 | 0.025 | 2.53E-12 | 7174 |
|  | rs146127446 | 4 | 114762666 | T | A | -0.076 | 0.017 | 0.406 | 8.13E-06 | 7174 |
|  | rs374071816 | 6 | 160682773 | A | T | 0.190 | 0.036 | 0.057 | 1.62E-07 | 7174 |
|  | rs12675806 | 8 | 4789612 | A | G | 0.121 | 0.026 | 0.112 | 4.82E-06 | 7174 |
|  | rs147426822 | 8 | 23959870 | T | C | 0.359 | 0.079 | 0.013 | 4.97E-06 | 7174 |
|  | rs79231873 | 8 | 53151614 | G | A | 0.294 | 0.066 | 0.020 | 7.37E-06 | 7174 |
|  | rs13258507 | 8 | 125480355 | G | T | 0.093 | 0.019 | 0.241 | 1.92E-06 | 7174 |
|  | rs715119 | 9 | 104975112 | T | C | -0.092 | 0.020 | 0.233 | 3.11E-06 | 7174 |
|  | rs7478167 | 10 | 101716501 | A | T | -0.083 | 0.018 | 0.669 | 3.36E-06 | 7174 |
|  | rs75740927 | 11 | 95614411 | G | C | 0.230 | 0.051 | 0.031 | 7.25E-06 | 7174 |
|  | rs3741111 | 11 | 124076588 | G | A | 0.086 | 0.017 | 0.483 | 2.19E-07 | 7174 |
|  | rs118070067 | 12 | 61250942 | A | C | 0.258 | 0.057 | 0.022 | 7.31E-06 | 7174 |
|  | rs7970512 | 12 | 67513820 | A | C | -0.225 | 0.048 | 0.035 | 2.46E-06 | 7174 |
|  | rs117146578 | 12 | 101922985 | C | T | 0.183 | 0.039 | 0.048 | 3.27E-06 | 7174 |
|  | rs7157785 | 14 | 63768838 | T | G | 0.370 | 0.025 | 0.125 | 1.11E-49 | 7174 |
|  | rs7172051 | 15 | 69995976 | G | A | 0.077 | 0.017 | 0.373 | 9.32E-06 | 7174 |
|  | rs2867985 | 15 | 78071433 | A | G | 0.082 | 0.018 | 0.305 | 6.90E-06 | 7174 |
|  | rs186039163 | 17 | 3547599 | G | T | -0.381 | 0.084 | 0.010 | 5.68E-06 | 7174 |
|  | rs75679663 | 17 | 4764677 | A | C | -0.474 | 0.053 | 0.026 | 9.06E-19 | 7174 |
|  | rs117643293 | 17 | 5065669 | T | C | -0.243 | 0.034 | 0.062 | 8.25E-13 | 7174 |
|  | rs144440646 | 18 | 71848979 | C | T | -0.394 | 0.082 | 0.011 | 1.54E-06 | 7174 |
|  | rs7258249 | 19 | 8206837 | A | G | 0.217 | 0.017 | 0.399 | 2.81E-37 | 7174 |
|  | rs7248003 | 19 | 8236164 | T | C | 0.109 | 0.019 | 0.721 | 9.84E-09 | 7174 |
|  | rs12462619 | 19 | 8607097 | C | T | -0.096 | 0.019 | 0.261 | 6.04E-07 | 7174 |
|  | rs7247433 | 19 | 19684751 | A | C | -0.085 | 0.017 | 0.394 | 9.72E-07 | 7174 |
|  | rs7412 | 19 | 44908822 | T | C | -0.176 | 0.037 | 0.053 | 2.28E-06 | 7174 |
|  | rs364585 | 20 | 12982070 | G | A | -0.164 | 0.018 | 0.661 | 7.39E-21 | 7174 |
|  | rs12480916 | 20 | 41303716 | G | T | 0.076 | 0.017 | 0.474 | 5.52E-06 | 7174 |
|  | rs76596580 | 21 | 34990612 | T | C | 0.121 | 0.026 | 0.115 | 4.58E-06 | 7174 |
| Sphingomyelin (d38:2) | rs11591147 | 1 | 55039974 | T | G | -0.219 | 0.047 | 0.033 | 2.84E-06 | 7150 |
|  | rs116411822 | 2 | 46494121 | G | A | -0.420 | 0.094 | 0.008 | 7.29E-06 | 7150 |
|  | rs10206976 | 2 | 210749914 | T | G | 0.091 | 0.019 | 0.268 | 1.88E-06 | 7150 |
|  | rs3936109 | 3 | 184623650 | G | A | -0.104 | 0.022 | 0.180 | 2.72E-06 | 7150 |
|  | rs77645768 | 4 | 72803111 | A | G | 0.268 | 0.054 | 0.026 | 6.16E-07 | 7150 |
|  | rs114483871 | 4 | 73124451 | T | C | 0.220 | 0.045 | 0.038 | 9.38E-07 | 7150 |
|  | rs182695896 | 4 | 73947510 | C | A | 0.326 | 0.053 | 0.025 | 8.57E-10 | 7150 |
|  | rs114717988 | 6 | 48788044 | A | T | 0.197 | 0.040 | 0.046 | 6.37E-07 | 7150 |
|  | rs581080 | 9 | 15305380 | C | G | 0.115 | 0.024 | 0.856 | 1.19E-06 | 7150 |
|  | rs672959 | 10 | 14418295 | G | A | -0.459 | 0.100 | 0.008 | 4.67E-06 | 7150 |
|  | rs139150589 | 10 | 70459848 | T | C | 0.521 | 0.100 | 0.008 | 1.87E-07 | 7150 |
|  | rs72839716 | 10 | 122833455 | A | G | -0.108 | 0.023 | 0.159 | 2.53E-06 | 7150 |
|  | rs174556 | 11 | 61813163 | T | C | -0.144 | 0.017 | 0.380 | 6.46E-17 | 7150 |
|  | rs657315 | 11 | 69517944 | T | C | 0.082 | 0.018 | 0.284 | 8.75E-06 | 7150 |
|  | rs75038600 | 12 | 12341682 | C | G | 0.726 | 0.160 | 0.003 | 5.94E-06 | 7150 |
|  | rs1169306 | 12 | 121000508 | T | C | -0.121 | 0.017 | 0.360 | 4.14E-12 | 7150 |
|  | rs75098294 | 13 | 62988645 | G | A | 0.527 | 0.107 | 0.006 | 9.06E-07 | 7150 |
|  | rs17276940 | 14 | 20017956 | T | C | -0.245 | 0.046 | 0.034 | 1.02E-07 | 7150 |
|  | rs113118892 | 15 | 59274186 | T | C | 0.383 | 0.080 | 0.012 | 1.58E-06 | 7150 |
|  | rs146723536 | 16 | 12147559 | A | G | -0.256 | 0.057 | 0.022 | 8.40E-06 | 7150 |
|  | rs72794954 | 16 | 76342662 | T | C | -0.490 | 0.105 | 0.007 | 3.02E-06 | 7150 |
|  | rs142118024 | 18 | 67530560 | G | A | -0.303 | 0.068 | 0.019 | 8.02E-06 | 7150 |
|  | rs2336171 | 19 | 8209156 | C | G | 0.261 | 0.017 | 0.399 | 5.70E-53 | 7150 |
|  | rs7248003 | 19 | 8236164 | T | C | 0.152 | 0.019 | 0.721 | 2.09E-15 | 7150 |
|  | rs250507 | 19 | 8268177 | C | T | -0.109 | 0.020 | 0.229 | 7.49E-08 | 7150 |
|  | rs10405061 | 19 | 8341496 | T | C | -0.095 | 0.018 | 0.309 | 2.01E-07 | 7150 |
|  | rs12462619 | 19 | 8607097 | C | T | -0.131 | 0.019 | 0.261 | 1.44E-11 | 7150 |
|  | rs4804319 | 19 | 8628548 | A | C | -0.087 | 0.018 | 0.691 | 1.74E-06 | 7150 |
|  | rs61751862 | 19 | 48629610 | C | G | 0.230 | 0.048 | 0.031 | 1.70E-06 | 7150 |
|  | rs142551539 | 21 | 29991071 | T | C | 0.545 | 0.122 | 0.005 | 7.56E-06 | 7150 |
|  | rs148683988 | 22 | 48622225 | G | C | 0.339 | 0.073 | 0.014 | 4.04E-06 | 7150 |
| Sphingomyelin (d40:1) | rs76174849 | 1 | 8738706 | T | C | 0.367 | 0.072 | 0.014 | 3.41E-07 | 7174 |
|  | rs4949190 | 1 | 31012510 | T | C | -0.099 | 0.020 | 0.773 | 7.78E-07 | 7174 |
|  | rs11591147 | 1 | 55039974 | T | G | -0.332 | 0.046 | 0.033 | 1.05E-12 | 7174 |
|  | rs80019356 | 1 | 155912046 | G | C | 0.252 | 0.056 | 0.023 | 7.92E-06 | 7174 |
|  | rs10927285 | 1 | 236024983 | G | C | 0.082 | 0.018 | 0.327 | 2.86E-06 | 7174 |
|  | rs283819 | 2 | 79000117 | C | A | -0.106 | 0.022 | 0.816 | 1.01E-06 | 7174 |
|  | rs4954960 | 2 | 138816573 | C | T | 0.091 | 0.019 | 0.738 | 1.90E-06 | 7174 |
|  | rs188346987 | 2 | 157097431 | G | C | -0.176 | 0.040 | 0.051 | 8.76E-06 | 7174 |
|  | rs9835223 | 3 | 186868495 | G | C | -0.104 | 0.021 | 0.801 | 9.29E-07 | 7174 |
|  | rs80101718 | 4 | 72375030 | A | G | 0.334 | 0.051 | 0.030 | 4.97E-11 | 7174 |
|  | rs77645768 | 4 | 72803111 | A | G | 0.320 | 0.054 | 0.026 | 2.56E-09 | 7174 |
|  | rs182695896 | 4 | 73947510 | C | A | 0.377 | 0.053 | 0.025 | 1.11E-12 | 7174 |
|  | rs77030061 | 4 | 168898979 | T | C | 0.225 | 0.048 | 0.038 | 2.68E-06 | 7174 |
|  | rs66626762 | 5 | 101638460 | G | A | 0.141 | 0.032 | 0.075 | 9.67E-06 | 7174 |
|  | rs374071816 | 6 | 160682773 | A | T | 0.175 | 0.036 | 0.057 | 1.38E-06 | 7174 |
|  | rs2552134 | 8 | 4075730 | T | G | -0.093 | 0.020 | 0.221 | 3.88E-06 | 7174 |
|  | rs279967 | 8 | 93048045 | G | A | -0.157 | 0.034 | 0.938 | 5.45E-06 | 7174 |
|  | rs2275544 | 9 | 104888931 | C | T | -0.123 | 0.025 | 0.125 | 1.13E-06 | 7174 |
|  | rs115478735 | 9 | 133274295 | T | A | 0.093 | 0.021 | 0.199 | 7.41E-06 | 7174 |
|  | rs7478167 | 10 | 101716501 | A | T | -0.081 | 0.018 | 0.669 | 6.39E-06 | 7174 |
|  | rs4962634 | 10 | 124492922 | G | A | 0.075 | 0.017 | 0.511 | 6.80E-06 | 7174 |
|  | rs138255545 | 11 | 22327737 | A | G | 0.211 | 0.047 | 0.033 | 8.58E-06 | 7174 |
|  | rs113318071 | 11 | 46848527 | T | C | 0.162 | 0.034 | 0.063 | 2.33E-06 | 7174 |
|  | rs8189230 | 11 | 48806055 | A | G | 0.156 | 0.034 | 0.070 | 3.88E-06 | 7174 |
|  | rs11050950 | 12 | 30557703 | T | G | 0.147 | 0.032 | 0.070 | 6.29E-06 | 7174 |
|  | rs7970512 | 12 | 67513820 | A | C | -0.229 | 0.048 | 0.035 | 1.65E-06 | 7174 |
|  | rs117146578 | 12 | 101922985 | C | T | 0.179 | 0.039 | 0.048 | 5.15E-06 | 7174 |
|  | rs4767003 | 12 | 112753113 | C | T | 0.087 | 0.018 | 0.665 | 6.52E-07 | 7174 |
|  | rs34359547 | 13 | 96476320 | A | G | 0.177 | 0.040 | 0.045 | 9.05E-06 | 7174 |
|  | rs145797759 | 13 | 103929015 | A | T | 0.829 | 0.185 | 0.002 | 7.39E-06 | 7174 |
|  | rs113118892 | 15 | 59274186 | T | C | 0.359 | 0.079 | 0.012 | 6.26E-06 | 7174 |
|  | rs186039163 | 17 | 3547599 | G | T | -0.399 | 0.084 | 0.010 | 2.09E-06 | 7174 |
|  | rs76790474 | 17 | 4498679 | A | G | -0.137 | 0.019 | 0.253 | 1.64E-12 | 7174 |
|  | rs75679663 | 17 | 4764677 | A | C | -0.774 | 0.053 | 0.026 | 1.26E-47 | 7174 |
|  | rs192913735 | 17 | 4779345 | T | C | -0.562 | 0.087 | 0.009 | 1.36E-10 | 7174 |
|  | rs117643293 | 17 | 5065669 | T | C | -0.325 | 0.034 | 0.062 | 1.08E-21 | 7174 |
|  | rs8067235 | 17 | 81050837 | A | G | 0.079 | 0.017 | 0.396 | 4.10E-06 | 7174 |
|  | rs118068660 | 19 | 11079868 | T | C | -0.143 | 0.028 | 0.095 | 3.46E-07 | 7174 |
|  | rs7412 | 19 | 44908822 | T | C | -0.200 | 0.037 | 0.053 | 7.36E-08 | 7174 |
|  | rs111997200 | 19 | 44963068 | C | T | -0.080 | 0.017 | 0.501 | 1.99E-06 | 7174 |
|  | rs56671692 | 20 | 4931633 | A | G | 0.241 | 0.054 | 0.026 | 7.09E-06 | 7174 |
|  | rs59596298 | 20 | 41301845 | C | G | 0.089 | 0.017 | 0.475 | 1.27E-07 | 7174 |
|  | rs1800961 | 20 | 44413724 | T | C | -0.178 | 0.037 | 0.052 | 1.71E-06 | 7174 |
|  | rs140202522 | 21 | 38913660 | T | G | 0.403 | 0.090 | 0.010 | 6.98E-06 | 7174 |
| Sphingomyelin (d40:2) | rs79984432 | 1 | 8809221 | C | A | 0.343 | 0.076 | 0.013 | 5.94E-06 | 7174 |
|  | rs11591147 | 1 | 55039974 | T | G | -0.259 | 0.047 | 0.033 | 2.73E-08 | 7174 |
|  | rs1237196 | 1 | 164890921 | A | C | -0.089 | 0.020 | 0.782 | 9.98E-06 | 7174 |
|  | rs7536561 | 1 | 180274389 | G | A | 0.076 | 0.017 | 0.578 | 6.94E-06 | 7174 |
|  | rs2760512 | 1 | 192592415 | G | A | 0.084 | 0.018 | 0.333 | 3.31E-06 | 7174 |
|  | rs16851199 | 1 | 230244207 | G | A | -0.189 | 0.042 | 0.043 | 8.33E-06 | 7174 |
|  | rs10490233 | 2 | 50201990 | A | T | -0.131 | 0.026 | 0.111 | 7.65E-07 | 7174 |
|  | rs148316008 | 2 | 128299827 | A | G | -0.352 | 0.077 | 0.012 | 5.30E-06 | 7174 |
|  | rs56298236 | 2 | 210741300 | T | C | 0.096 | 0.019 | 0.268 | 4.61E-07 | 7174 |
|  | rs115880518 | 3 | 20089961 | G | A | 0.468 | 0.102 | 0.007 | 4.20E-06 | 7174 |
|  | rs9839427 | 3 | 50264152 | G | A | -0.081 | 0.018 | 0.325 | 4.75E-06 | 7174 |
|  | rs9835223 | 3 | 186868495 | G | C | -0.105 | 0.021 | 0.801 | 6.33E-07 | 7174 |
|  | rs73073606 | 3 | 194176577 | C | A | -0.095 | 0.020 | 0.241 | 2.59E-06 | 7174 |
|  | rs77645768 | 4 | 72803111 | A | G | 0.304 | 0.054 | 0.026 | 1.55E-08 | 7174 |
|  | rs114483871 | 4 | 73124451 | T | C | 0.236 | 0.045 | 0.038 | 1.41E-07 | 7174 |
|  | rs182695896 | 4 | 73947510 | C | A | 0.353 | 0.053 | 0.025 | 2.77E-11 | 7174 |
|  | rs79402526 | 4 | 168864851 | C | T | 0.214 | 0.047 | 0.039 | 4.43E-06 | 7174 |
|  | rs187144380 | 6 | 151920771 | G | A | 0.380 | 0.082 | 0.011 | 3.36E-06 | 7174 |
|  | rs73641617 | 9 | 5454500 | T | A | 0.294 | 0.064 | 0.018 | 3.84E-06 | 7174 |
|  | rs7478167 | 10 | 101716501 | A | T | -0.082 | 0.018 | 0.669 | 5.02E-06 | 7174 |
|  | rs11522703 | 11 | 56943668 | A | G | 0.100 | 0.023 | 0.166 | 8.88E-06 | 7174 |
|  | rs174561 | 11 | 61815236 | C | T | -0.121 | 0.017 | 0.381 | 2.13E-12 | 7174 |
|  | rs646488 | 11 | 125920827 | C | T | 0.082 | 0.018 | 0.679 | 5.12E-06 | 7174 |
|  | rs1797734 | 12 | 31794260 | A | G | 0.079 | 0.018 | 0.656 | 7.57E-06 | 7174 |
|  | rs17604972 | 12 | 45576938 | A | G | 0.107 | 0.024 | 0.144 | 9.37E-06 | 7174 |
|  | rs17276933 | 14 | 20017746 | A | T | -0.165 | 0.036 | 0.057 | 4.64E-06 | 7174 |
|  | rs146855398 | 14 | 56720725 | T | C | -0.237 | 0.053 | 0.028 | 7.06E-06 | 7174 |
|  | rs7157785 | 14 | 63768838 | T | G | 0.337 | 0.025 | 0.125 | 2.10E-41 | 7174 |
|  | rs12595641 | 15 | 31376503 | A | G | 0.098 | 0.022 | 0.183 | 6.00E-06 | 7174 |
|  | rs113118892 | 15 | 59274186 | T | C | 0.362 | 0.079 | 0.012 | 5.09E-06 | 7174 |
|  | rs75679663 | 17 | 4764677 | A | C | -0.277 | 0.054 | 0.026 | 2.59E-07 | 7174 |
|  | rs3026120 | 17 | 5384095 | A | G | -0.188 | 0.031 | 0.075 | 1.61E-09 | 7174 |
|  | rs113722226 | 19 | 11078596 | C | T | -0.141 | 0.028 | 0.094 | 5.58E-07 | 7174 |
|  | rs7247433 | 19 | 19684751 | A | C | -0.078 | 0.017 | 0.394 | 7.99E-06 | 7174 |
|  | rs61751862 | 19 | 48629610 | C | G | 0.238 | 0.048 | 0.031 | 7.20E-07 | 7174 |
|  | rs364585 | 20 | 12982070 | G | A | -0.122 | 0.018 | 0.661 | 4.33E-12 | 7174 |
| Sphingomyelin (d42:2) | rs17110517 | 1 | 54369167 | T | C | -0.167 | 0.035 | 0.061 | 2.26E-06 | 7174 |
|  | rs11591147 | 1 | 55039974 | T | G | -0.362 | 0.047 | 0.033 | 8.29E-15 | 7174 |
|  | rs17018185 | 1 | 107036673 | C | A | -0.082 | 0.018 | 0.279 | 8.28E-06 | 7174 |
|  | rs6680361 | 1 | 180277126 | T | C | 0.097 | 0.021 | 0.795 | 4.53E-06 | 7174 |
|  | rs1367117 | 2 | 21041028 | A | G | 0.100 | 0.018 | 0.285 | 6.88E-08 | 7174 |
|  | rs17261772 | 2 | 135153852 | C | T | 0.090 | 0.018 | 0.326 | 3.94E-07 | 7174 |
|  | rs59916403 | 2 | 241431336 | T | G | 0.078 | 0.017 | 0.413 | 3.85E-06 | 7174 |
|  | rs2034007 | 3 | 19330366 | G | A | -0.082 | 0.018 | 0.300 | 7.52E-06 | 7174 |
|  | rs62274367 | 3 | 103515604 | C | T | -0.680 | 0.146 | 0.004 | 3.03E-06 | 7174 |
|  | rs2177333 | 3 | 153576128 | A | G | 0.075 | 0.017 | 0.534 | 7.76E-06 | 7174 |
|  | rs6843498 | 4 | 16520100 | G | T | 0.095 | 0.019 | 0.312 | 5.00E-07 | 7174 |
|  | rs112585713 | 4 | 72876701 | A | G | 0.288 | 0.048 | 0.032 | 1.86E-09 | 7174 |
|  | rs114483871 | 4 | 73124451 | T | C | 0.279 | 0.045 | 0.038 | 4.78E-10 | 7174 |
|  | rs182695896 | 4 | 73947510 | C | A | 0.382 | 0.053 | 0.025 | 5.36E-13 | 7174 |
|  | rs115350073 | 5 | 90870095 | T | C | 0.294 | 0.063 | 0.018 | 3.56E-06 | 7174 |
|  | rs67718253 | 5 | 108641417 | C | A | -0.102 | 0.022 | 0.176 | 3.65E-06 | 7174 |
|  | rs12530474 | 6 | 14104359 | G | A | -0.111 | 0.024 | 0.133 | 4.56E-06 | 7174 |
|  | rs17145750 | 7 | 73612048 | T | C | 0.132 | 0.024 | 0.141 | 3.07E-08 | 7174 |
|  | rs10252648 | 7 | 151941718 | C | G | 0.257 | 0.058 | 0.978 | 9.95E-06 | 7174 |
|  | rs75444922 | 8 | 46957856 | G | A | 0.236 | 0.053 | 0.027 | 9.45E-06 | 7174 |
|  | rs4100654 | 9 | 104906960 | C | T | -0.156 | 0.034 | 0.066 | 3.76E-06 | 7174 |
|  | rs184845520 | 10 | 45824374 | G | C | 0.564 | 0.123 | 0.005 | 4.49E-06 | 7174 |
|  | rs139474488 | 10 | 128742890 | C | A | 0.344 | 0.076 | 0.014 | 6.01E-06 | 7174 |
|  | rs116893213 | 11 | 101739572 | C | G | 0.227 | 0.045 | 0.037 | 4.17E-07 | 7174 |
|  | rs3912622 | 11 | 103250744 | C | A | -0.109 | 0.021 | 0.200 | 2.00E-07 | 7174 |
|  | rs7104343 | 11 | 116658307 | T | C | 0.093 | 0.021 | 0.205 | 6.92E-06 | 7174 |
|  | rs11610836 | 12 | 112765162 | T | C | 0.079 | 0.017 | 0.422 | 1.75E-06 | 7174 |
|  | rs17276933 | 14 | 20017746 | A | T | -0.171 | 0.036 | 0.057 | 2.11E-06 | 7174 |
|  | rs4283208 | 15 | 38953699 | A | G | -0.104 | 0.023 | 0.840 | 6.55E-06 | 7174 |
|  | rs515081 | 15 | 58424058 | G | A | -0.092 | 0.019 | 0.688 | 1.31E-06 | 7174 |
|  | rs116919723 | 17 | 3531540 | A | G | -0.334 | 0.075 | 0.013 | 7.56E-06 | 7174 |
|  | rs75679663 | 17 | 4764677 | A | C | -0.538 | 0.053 | 0.026 | 1.09E-23 | 7174 |
|  | rs146331166 | 17 | 5464230 | C | T | -0.219 | 0.035 | 0.060 | 2.56E-10 | 7174 |
|  | rs3751934 | 17 | 80964698 | A | C | 0.077 | 0.017 | 0.434 | 5.32E-06 | 7174 |
|  | rs118068660 | 19 | 11079868 | T | C | -0.219 | 0.028 | 0.095 | 6.77E-15 | 7174 |
|  | rs7247433 | 19 | 19684751 | A | C | -0.079 | 0.017 | 0.394 | 5.43E-06 | 7174 |
|  | rs7412 | 19 | 44908822 | T | C | -0.211 | 0.037 | 0.053 | 1.45E-08 | 7174 |
|  | rs73047641 | 19 | 44954120 | A | G | -0.080 | 0.017 | 0.498 | 1.73E-06 | 7174 |
| Triacylglycerol (46:1) | rs78056622 | 1 | 184416563 | G | C | 0.406 | 0.090 | 0.011 | 6.91E-06 | 6070 |
|  | rs1260326 | 2 | 27508073 | C | T | -0.101 | 0.019 | 0.651 | 8.99E-08 | 6070 |
|  | rs478562 | 2 | 136406475 | C | T | 0.788 | 0.178 | 0.997 | 9.57E-06 | 6070 |
|  | rs16853823 | 2 | 215338573 | G | A | 0.100 | 0.022 | 0.223 | 4.54E-06 | 6070 |
|  | rs9837572 | 3 | 72260449 | T | G | -0.097 | 0.021 | 0.236 | 6.10E-06 | 6070 |
|  | rs4318703 | 4 | 135989116 | G | A | 0.089 | 0.020 | 0.300 | 8.71E-06 | 6070 |
|  | rs117591451 | 8 | 40367808 | A | G | -0.402 | 0.085 | 0.013 | 2.27E-06 | 6070 |
|  | rs16923137 | 8 | 58158358 | T | A | 0.186 | 0.040 | 0.052 | 4.37E-06 | 6070 |
|  | rs117730598 | 9 | 2770506 | C | T | 0.263 | 0.054 | 0.030 | 1.16E-06 | 6070 |
|  | rs111568723 | 9 | 132470502 | G | A | 0.279 | 0.059 | 0.026 | 2.63E-06 | 6070 |
|  | rs12225049 | 11 | 110379615 | T | C | -0.117 | 0.024 | 0.169 | 1.44E-06 | 6070 |
|  | rs12301917 | 12 | 81672829 | T | C | 0.113 | 0.023 | 0.189 | 1.16E-06 | 6070 |
|  | rs73259585 | 14 | 29818391 | G | A | -0.216 | 0.048 | 0.037 | 8.36E-06 | 6070 |
|  | rs11160050 | 14 | 92187131 | G | A | -0.133 | 0.029 | 0.116 | 4.42E-06 | 6070 |
|  | rs9796793 | 16 | 30484331 | A | G | -0.131 | 0.028 | 0.125 | 3.81E-06 | 6070 |
|  | rs10521320 | 16 | 55542249 | C | A | 0.107 | 0.023 | 0.195 | 5.44E-06 | 6070 |
|  | rs1553368 | 17 | 53571810 | G | A | -0.091 | 0.019 | 0.614 | 1.17E-06 | 6070 |
|  | rs35840156 | 17 | 77816410 | T | C | 0.273 | 0.058 | 0.026 | 2.14E-06 | 6070 |
|  | rs10502551 | 18 | 30887893 | G | C | -0.202 | 0.042 | 0.051 | 1.83E-06 | 6070 |
|  | rs79152531 | 19 | 55802466 | C | T | -0.195 | 0.039 | 0.060 | 5.89E-07 | 6070 |
|  | rs998182 | 20 | 13847007 | T | C | -0.082 | 0.018 | 0.465 | 5.07E-06 | 6070 |
|  | rs2205135 | 21 | 40708914 | C | G | 0.093 | 0.020 | 0.277 | 5.49E-06 | 6070 |
| Triacylglycerol (46:2) | rs78056622 | 1 | 184416563 | G | C | 0.472 | 0.096 | 0.011 | 8.72E-07 | 5448 |
|  | rs1260326 | 2 | 27508073 | C | T | -0.114 | 0.020 | 0.651 | 1.35E-08 | 5448 |
|  | rs7560313 | 2 | 238572357 | G | C | -0.087 | 0.019 | 0.452 | 7.53E-06 | 5448 |
|  | rs778957 | 4 | 62386287 | C | G | -0.143 | 0.030 | 0.120 | 1.30E-06 | 5448 |
|  | rs17189819 | 6 | 30972348 | T | C | -0.142 | 0.032 | 0.103 | 7.30E-06 | 5448 |
|  | rs141935215 | 6 | 33985737 | A | G | 0.298 | 0.066 | 0.024 | 6.61E-06 | 5448 |
|  | rs117591451 | 8 | 40367808 | A | G | -0.408 | 0.091 | 0.013 | 7.07E-06 | 5448 |
|  | rs6474079 | 8 | 56711024 | T | C | -0.115 | 0.025 | 0.817 | 2.88E-06 | 5448 |
|  | rs191616475 | 8 | 58330047 | G | A | 0.225 | 0.044 | 0.048 | 3.64E-07 | 5448 |
|  | rs140796055 | 9 | 2283431 | C | T | -0.228 | 0.051 | 0.039 | 6.76E-06 | 5448 |
|  | rs117529982 | 9 | 99655889 | T | A | -0.477 | 0.106 | 0.009 | 6.46E-06 | 5448 |
|  | rs116923150 | 9 | 112114209 | A | G | 0.830 | 0.183 | 0.003 | 5.75E-06 | 5448 |
|  | rs2084594 | 11 | 26783827 | A | G | 0.091 | 0.019 | 0.461 | 1.79E-06 | 5448 |
|  | rs12225049 | 11 | 110379615 | T | C | -0.118 | 0.026 | 0.169 | 4.24E-06 | 5448 |
|  | rs11613792 | 12 | 8608014 | G | A | -0.118 | 0.027 | 0.163 | 8.83E-06 | 5448 |
|  | rs12301917 | 12 | 81672829 | T | C | 0.110 | 0.025 | 0.189 | 7.62E-06 | 5448 |
|  | rs7323268 | 13 | 33335601 | T | C | 0.105 | 0.023 | 0.220 | 5.24E-06 | 5448 |
|  | rs73226143 | 13 | 76166574 | C | T | 0.130 | 0.028 | 0.136 | 3.52E-06 | 5448 |
|  | rs9796793 | 16 | 30484331 | A | G | -0.150 | 0.030 | 0.125 | 4.57E-07 | 5448 |
|  | rs6502975 | 17 | 6764031 | T | C | 0.148 | 0.033 | 0.103 | 5.20E-06 | 5448 |
|  | rs6504835 | 17 | 53554728 | A | T | -0.094 | 0.020 | 0.412 | 1.53E-06 | 5448 |
|  | rs10502551 | 18 | 30887893 | G | C | -0.229 | 0.045 | 0.051 | 3.43E-07 | 5448 |
|  | rs2018873 | 19 | 15080523 | T | C | 0.093 | 0.020 | 0.603 | 4.28E-06 | 5448 |
|  | rs35305910 | 19 | 32508986 | T | C | 0.154 | 0.034 | 0.086 | 7.60E-06 | 5448 |
|  | rs79152531 | 19 | 55802466 | C | T | -0.191 | 0.042 | 0.060 | 4.47E-06 | 5448 |
| Triacylglycerol (48:0) | rs10218823 | 1 | 6029418 | T | G | -0.352 | 0.076 | 0.017 | 3.63E-06 | 5463 |
|  | rs969816 | 2 | 210779037 | G | A | 0.133 | 0.030 | 0.120 | 9.09E-06 | 5463 |
|  | rs4955801 | 3 | 179092711 | C | T | -0.107 | 0.023 | 0.785 | 4.09E-06 | 5463 |
|  | rs140828820 | 4 | 34767471 | G | A | 0.424 | 0.095 | 0.011 | 7.93E-06 | 5463 |
|  | rs62348866 | 4 | 186453244 | A | G | -0.141 | 0.030 | 0.118 | 2.01E-06 | 5463 |
|  | rs7721676 | 5 | 149703644 | C | T | -0.104 | 0.023 | 0.218 | 8.45E-06 | 5463 |
|  | rs79159286 | 7 | 31677697 | G | T | 0.163 | 0.036 | 0.080 | 5.20E-06 | 5463 |
|  | rs117945317 | 8 | 11538263 | A | T | -0.475 | 0.107 | 0.009 | 9.91E-06 | 5463 |
|  | rs2242104 | 9 | 2640492 | G | A | -0.103 | 0.022 | 0.752 | 4.16E-06 | 5463 |
|  | rs117264209 | 9 | 135992678 | G | C | -0.331 | 0.072 | 0.019 | 4.84E-06 | 5463 |
|  | rs73302224 | 10 | 48593367 | C | T | 0.518 | 0.116 | 0.007 | 7.88E-06 | 5463 |
|  | rs60243822 | 12 | 81637055 | C | A | 0.114 | 0.026 | 0.172 | 7.93E-06 | 5463 |
|  | rs222507 | 12 | 101725095 | T | G | -0.323 | 0.070 | 0.979 | 3.68E-06 | 5463 |
|  | rs10141087 | 14 | 36678136 | C | G | -0.089 | 0.020 | 0.615 | 5.80E-06 | 5463 |
|  | rs769432 | 17 | 3420961 | T | C | 0.091 | 0.019 | 0.509 | 2.04E-06 | 5463 |
|  | rs6504835 | 17 | 53554728 | A | T | -0.087 | 0.020 | 0.412 | 9.96E-06 | 5463 |
|  | rs3894049 | 18 | 79524930 | G | C | 0.088 | 0.020 | 0.522 | 8.06E-06 | 5463 |
|  | rs10425025 | 19 | 9009145 | A | T | 0.305 | 0.066 | 0.023 | 4.55E-06 | 5463 |
|  | rs76375876 | 19 | 49524378 | T | C | 0.296 | 0.063 | 0.026 | 2.78E-06 | 5463 |
|  | rs2180528 | 20 | 15445391 | T | C | 0.096 | 0.020 | 0.389 | 9.31E-07 | 5463 |
|  | rs62235973 | 22 | 37593713 | A | G | 0.241 | 0.051 | 0.037 | 2.54E-06 | 5463 |
| Triacylglycerol (48:1) | rs12565526 | 1 | 77362636 | G | A | 0.080 | 0.018 | 0.624 | 6.17E-06 | 7019 |
|  | rs79416109 | 1 | 102847102 | G | T | 0.126 | 0.028 | 0.104 | 8.66E-06 | 7019 |
|  | rs6664147 | 1 | 227429818 | T | C | -0.097 | 0.022 | 0.190 | 7.94E-06 | 7019 |
|  | rs1260326 | 2 | 27508073 | C | T | -0.119 | 0.018 | 0.651 | 1.89E-11 | 7019 |
|  | rs67093838 | 2 | 62122935 | G | A | -0.220 | 0.049 | 0.030 | 8.76E-06 | 7019 |
|  | rs17192812 | 2 | 163555501 | A | G | 0.166 | 0.037 | 0.059 | 6.03E-06 | 7019 |
|  | rs6721083 | 2 | 170225142 | C | G | 0.158 | 0.035 | 0.937 | 8.31E-06 | 7019 |
|  | rs1515104 | 2 | 226227689 | A | T | 0.078 | 0.018 | 0.631 | 8.22E-06 | 7019 |
|  | rs6804331 | 3 | 104035543 | G | T | -0.080 | 0.018 | 0.337 | 8.25E-06 | 7019 |
|  | rs147463852 | 3 | 171488096 | T | C | -0.440 | 0.097 | 0.008 | 5.62E-06 | 7019 |
|  | rs7721676 | 5 | 149703644 | C | T | -0.091 | 0.020 | 0.218 | 8.92E-06 | 7019 |
|  | rs75178403 | 8 | 2825696 | C | T | -0.414 | 0.090 | 0.010 | 4.19E-06 | 7019 |
|  | rs16923137 | 8 | 58158358 | T | A | 0.172 | 0.038 | 0.052 | 5.08E-06 | 7019 |
|  | rs117730598 | 9 | 2770506 | C | T | 0.257 | 0.051 | 0.030 | 4.40E-07 | 7019 |
|  | rs72753379 | 9 | 120350676 | T | C | 0.207 | 0.045 | 0.039 | 4.90E-06 | 7019 |
|  | rs964184 | 11 | 116778201 | C | G | -0.129 | 0.023 | 0.849 | 3.48E-08 | 7019 |
|  | rs56082136 | 12 | 52243392 | T | C | 0.173 | 0.039 | 0.053 | 8.47E-06 | 7019 |
|  | rs10861498 | 12 | 105758014 | A | G | -0.095 | 0.019 | 0.242 | 9.10E-07 | 7019 |
|  | rs117387197 | 12 | 131622906 | C | G | 0.141 | 0.032 | 0.084 | 9.39E-06 | 7019 |
|  | rs3127050 | 13 | 60832603 | A | G | 0.088 | 0.019 | 0.718 | 2.18E-06 | 7019 |
|  | rs1884216 | 14 | 36679967 | A | C | -0.085 | 0.017 | 0.623 | 1.18E-06 | 7019 |
|  | rs16970164 | 15 | 39999889 | C | A | -0.202 | 0.043 | 0.039 | 2.04E-06 | 7019 |
|  | rs79152531 | 19 | 55802466 | C | T | -0.188 | 0.036 | 0.060 | 1.80E-07 | 7019 |
|  | rs6079205 | 20 | 13891447 | T | C | -0.077 | 0.017 | 0.639 | 9.42E-06 | 7019 |
| Triacylglycerol (48:2) | rs6664147 | 1 | 227429818 | T | C | -0.096 | 0.022 | 0.190 | 8.72E-06 | 7071 |
|  | rs1260326 | 2 | 27508073 | C | T | -0.138 | 0.018 | 0.651 | 4.46E-15 | 7071 |
|  | rs10084264 | 2 | 163558771 | T | G | 0.172 | 0.036 | 0.059 | 2.26E-06 | 7071 |
|  | rs6804331 | 3 | 104035543 | G | T | -0.081 | 0.018 | 0.337 | 5.87E-06 | 7071 |
|  | rs79934453 | 6 | 23919006 | G | A | -0.610 | 0.137 | 0.004 | 8.65E-06 | 7071 |
|  | rs173848 | 7 | 30277547 | T | A | 0.083 | 0.018 | 0.663 | 7.34E-06 | 7071 |
|  | rs117591451 | 8 | 40367808 | A | G | -0.360 | 0.078 | 0.013 | 3.43E-06 | 7071 |
|  | rs191616475 | 8 | 58330047 | G | A | 0.189 | 0.039 | 0.048 | 1.15E-06 | 7071 |
|  | rs138672261 | 8 | 85466785 | A | G | 0.282 | 0.063 | 0.020 | 7.50E-06 | 7071 |
|  | rs117730598 | 9 | 2770506 | C | T | 0.236 | 0.051 | 0.030 | 2.97E-06 | 7071 |
|  | rs1832326 | 9 | 33656919 | C | A | 0.083 | 0.019 | 0.725 | 9.60E-06 | 7071 |
|  | rs72738698 | 9 | 80090365 | A | G | 0.102 | 0.022 | 0.179 | 4.16E-06 | 7071 |
|  | rs72753379 | 9 | 120350676 | T | C | 0.208 | 0.045 | 0.039 | 3.94E-06 | 7071 |
|  | rs964184 | 11 | 116778201 | C | G | -0.143 | 0.023 | 0.849 | 9.60E-10 | 7071 |
|  | rs56082136 | 12 | 52243392 | T | C | 0.174 | 0.039 | 0.053 | 6.20E-06 | 7071 |
|  | rs10861498 | 12 | 105758014 | A | G | -0.088 | 0.019 | 0.242 | 5.21E-06 | 7071 |
|  | rs3127050 | 13 | 60832603 | A | G | 0.089 | 0.019 | 0.718 | 1.87E-06 | 7071 |
|  | rs113777805 | 18 | 23684659 | C | G | 0.238 | 0.050 | 0.031 | 1.78E-06 | 7071 |
|  | rs10502551 | 18 | 30887893 | G | C | -0.185 | 0.039 | 0.051 | 2.01E-06 | 7071 |
|  | rs2546043 | 19 | 34971287 | G | A | -0.080 | 0.017 | 0.533 | 1.66E-06 | 7071 |
|  | rs117882070 | 19 | 43181186 | T | C | 0.300 | 0.068 | 0.018 | 9.40E-06 | 7071 |
|  | rs79152531 | 19 | 55802466 | C | T | -0.194 | 0.036 | 0.060 | 6.80E-08 | 7071 |
|  | rs13042732 | 20 | 56580207 | T | C | -0.180 | 0.040 | 0.046 | 8.96E-06 | 7071 |
|  | rs79213832 | 22 | 23971765 | G | A | 0.171 | 0.036 | 0.060 | 1.68E-06 | 7071 |
| Triacylglycerol (48:3) | rs1042034 | 2 | 21002409 | T | C | 0.090 | 0.019 | 0.728 | 2.44E-06 | 6855 |
|  | rs1260326 | 2 | 27508073 | C | T | -0.142 | 0.018 | 0.651 | 1.98E-15 | 6855 |
|  | rs111430300 | 2 | 36390910 | G | A | -0.111 | 0.025 | 0.135 | 6.78E-06 | 6855 |
|  | rs9837572 | 3 | 72260449 | T | G | -0.093 | 0.020 | 0.236 | 4.82E-06 | 6855 |
|  | rs62410903 | 4 | 44884393 | T | C | 0.082 | 0.018 | 0.335 | 5.78E-06 | 6855 |
|  | rs117591451 | 8 | 40367808 | A | G | -0.387 | 0.078 | 0.013 | 6.57E-07 | 6855 |
|  | rs191616475 | 8 | 58330047 | G | A | 0.205 | 0.039 | 0.048 | 2.08E-07 | 6855 |
|  | rs117730598 | 9 | 2770506 | C | T | 0.238 | 0.051 | 0.030 | 3.37E-06 | 6855 |
|  | rs72738698 | 9 | 80090365 | A | G | 0.109 | 0.022 | 0.179 | 1.25E-06 | 6855 |
|  | rs72846885 | 11 | 7455964 | G | A | 0.140 | 0.031 | 0.086 | 4.92E-06 | 6855 |
|  | rs820903 | 11 | 38161116 | G | A | 0.146 | 0.032 | 0.917 | 5.39E-06 | 6855 |
|  | rs964184 | 11 | 116778201 | C | G | -0.163 | 0.024 | 0.849 | 6.67E-12 | 6855 |
|  | rs56082136 | 12 | 52243392 | T | C | 0.189 | 0.039 | 0.053 | 1.49E-06 | 6855 |
|  | rs10861498 | 12 | 105758014 | A | G | -0.091 | 0.020 | 0.242 | 3.26E-06 | 6855 |
|  | rs77745982 | 13 | 21074873 | T | C | -0.141 | 0.032 | 0.081 | 8.49E-06 | 6855 |
|  | rs112578388 | 13 | 25886443 | T | C | -0.561 | 0.124 | 0.005 | 6.36E-06 | 6855 |
|  | rs4353433 | 14 | 93951456 | A | C | 0.076 | 0.017 | 0.578 | 9.85E-06 | 6855 |
|  | rs2280065 | 15 | 57470542 | A | G | -0.138 | 0.031 | 0.086 | 9.58E-06 | 6855 |
|  | rs9796793 | 16 | 30484331 | A | G | -0.126 | 0.026 | 0.125 | 1.80E-06 | 6855 |
|  | rs8047793 | 16 | 84544387 | T | C | -0.109 | 0.025 | 0.144 | 9.34E-06 | 6855 |
|  | rs113777805 | 18 | 23684659 | C | G | 0.249 | 0.051 | 0.031 | 8.55E-07 | 6855 |
|  | rs10502551 | 18 | 30887893 | G | C | -0.199 | 0.040 | 0.051 | 4.90E-07 | 6855 |
|  | rs187429064 | 19 | 19269704 | G | A | -0.188 | 0.039 | 0.054 | 1.44E-06 | 6855 |
|  | rs2546043 | 19 | 34971287 | G | A | -0.082 | 0.017 | 0.533 | 1.24E-06 | 6855 |
|  | rs79152531 | 19 | 55802466 | C | T | -0.174 | 0.037 | 0.060 | 2.28E-06 | 6855 |
|  | rs13042732 | 20 | 56580207 | T | C | -0.183 | 0.041 | 0.046 | 9.32E-06 | 6855 |
|  | rs79213832 | 22 | 23971765 | G | A | 0.160 | 0.036 | 0.060 | 9.53E-06 | 6855 |
| Triacylglycerol (49:1) | rs80278227 | 1 | 6096167 | T | C | -0.497 | 0.110 | 0.007 | 6.05E-06 | 5843 |
|  | rs2004776 | 1 | 230712956 | T | C | -0.105 | 0.023 | 0.196 | 8.53E-06 | 5843 |
|  | rs6429254 | 1 | 241221572 | T | C | 0.085 | 0.019 | 0.365 | 8.53E-06 | 5843 |
|  | rs34379959 | 2 | 12497377 | C | T | 0.150 | 0.034 | 0.088 | 9.49E-06 | 5843 |
|  | rs1260326 | 2 | 27508073 | C | T | -0.094 | 0.019 | 0.651 | 1.02E-06 | 5843 |
|  | rs149371861 | 2 | 241698370 | A | G | 0.231 | 0.051 | 0.036 | 5.13E-06 | 5843 |
|  | rs73216924 | 4 | 35018175 | G | T | -0.130 | 0.029 | 0.118 | 6.80E-06 | 5843 |
|  | rs71620944 | 4 | 182736762 | T | G | 0.190 | 0.043 | 0.051 | 9.58E-06 | 5843 |
|  | rs79725259 | 5 | 108626779 | G | C | -0.313 | 0.069 | 0.018 | 6.43E-06 | 5843 |
|  | rs112902435 | 5 | 149735907 | T | C | 0.183 | 0.040 | 0.056 | 5.99E-06 | 5843 |
|  | rs1233660 | 6 | 28292472 | A | T | -0.102 | 0.022 | 0.221 | 4.45E-06 | 5843 |
|  | rs33951980 | 7 | 73615107 | T | C | -0.130 | 0.028 | 0.122 | 4.91E-06 | 5843 |
|  | rs117591451 | 8 | 40367808 | A | G | -0.408 | 0.085 | 0.013 | 1.87E-06 | 5843 |
|  | rs138672261 | 8 | 85466785 | A | G | 0.308 | 0.069 | 0.020 | 7.60E-06 | 5843 |
|  | rs117730598 | 9 | 2770506 | C | T | 0.280 | 0.055 | 0.030 | 4.31E-07 | 5843 |
|  | rs10812660 | 9 | 27742537 | A | T | 0.089 | 0.020 | 0.375 | 7.02E-06 | 5843 |
|  | rs72753379 | 9 | 120350676 | T | C | 0.232 | 0.050 | 0.039 | 2.96E-06 | 5843 |
|  | rs964184 | 11 | 116778201 | C | G | -0.131 | 0.025 | 0.849 | 2.24E-07 | 5843 |
|  | rs146238333 | 12 | 83707933 | T | A | 0.627 | 0.134 | 0.005 | 2.75E-06 | 5843 |
|  | rs10861498 | 12 | 105758014 | A | G | -0.100 | 0.021 | 0.242 | 3.17E-06 | 5843 |
|  | rs2031531 | 13 | 49437013 | T | C | -0.102 | 0.023 | 0.206 | 9.14E-06 | 5843 |
|  | rs73259585 | 14 | 29818391 | G | A | -0.227 | 0.050 | 0.037 | 5.85E-06 | 5843 |
|  | rs16970164 | 15 | 39999889 | C | A | -0.211 | 0.048 | 0.039 | 9.81E-06 | 5843 |
|  | rs8055088 | 16 | 6818646 | T | A | -0.085 | 0.019 | 0.510 | 4.97E-06 | 5843 |
|  | rs113777805 | 18 | 23684659 | C | G | 0.244 | 0.054 | 0.031 | 5.40E-06 | 5843 |
|  | rs7412 | 19 | 44908822 | T | C | 0.199 | 0.041 | 0.053 | 1.63E-06 | 5843 |
| Triacylglycerol (49:2) | rs1260326 | 2 | 27508073 | C | T | -0.120 | 0.019 | 0.651 | 2.23E-10 | 6076 |
|  | rs6846577 | 4 | 64367871 | A | G | -0.101 | 0.022 | 0.215 | 4.27E-06 | 6076 |
|  | rs143838492 | 5 | 114892988 | A | G | 0.758 | 0.159 | 0.003 | 1.88E-06 | 6076 |
|  | rs145055632 | 6 | 50783096 | G | A | -0.516 | 0.112 | 0.006 | 4.36E-06 | 6076 |
|  | rs71556492 | 6 | 107854775 | C | T | 0.114 | 0.025 | 0.165 | 4.51E-06 | 6076 |
|  | rs2736863 | 8 | 133451589 | G | T | 0.463 | 0.101 | 0.992 | 4.81E-06 | 6076 |
|  | rs117730598 | 9 | 2770506 | C | T | 0.273 | 0.054 | 0.030 | 5.54E-07 | 6076 |
|  | rs964184 | 11 | 116778201 | C | G | -0.155 | 0.025 | 0.849 | 5.55E-10 | 6076 |
|  | rs4937417 | 11 | 129348709 | G | A | -0.085 | 0.018 | 0.467 | 3.39E-06 | 6076 |
|  | rs10861498 | 12 | 105758014 | A | G | -0.100 | 0.021 | 0.242 | 2.27E-06 | 6076 |
|  | rs76411296 | 15 | 59517420 | T | G | -0.136 | 0.029 | 0.116 | 2.28E-06 | 6076 |
|  | rs10468471 | 17 | 12222568 | C | T | -0.164 | 0.037 | 0.065 | 9.83E-06 | 6076 |
|  | rs1810258 | 17 | 66539562 | C | T | 0.085 | 0.018 | 0.428 | 4.49E-06 | 6076 |
|  | rs113777805 | 18 | 23684659 | C | G | 0.233 | 0.052 | 0.031 | 9.18E-06 | 6076 |
|  | rs10502551 | 18 | 30887893 | G | C | -0.199 | 0.042 | 0.051 | 2.76E-06 | 6076 |
|  | rs187429064 | 19 | 19269704 | G | A | -0.220 | 0.041 | 0.054 | 8.65E-08 | 6076 |
|  | rs140283718 | 19 | 30685856 | A | T | -0.297 | 0.059 | 0.026 | 5.75E-07 | 6076 |
| Triacylglycerol (50:1) | rs79416109 | 1 | 102847102 | G | T | 0.133 | 0.028 | 0.104 | 2.05E-06 | 7143 |
|  | rs78275146 | 1 | 196028959 | T | C | 0.337 | 0.076 | 0.014 | 8.42E-06 | 7143 |
|  | rs55857911 | 1 | 230598232 | T | C | 0.148 | 0.030 | 0.093 | 9.79E-07 | 7143 |
|  | rs1260326 | 2 | 27508073 | C | T | -0.109 | 0.017 | 0.651 | 4.18E-10 | 7143 |
|  | rs1399627 | 2 | 226218695 | G | A | 0.083 | 0.017 | 0.632 | 1.76E-06 | 7143 |
|  | rs6804331 | 3 | 104035543 | G | T | -0.079 | 0.018 | 0.337 | 9.07E-06 | 7143 |
|  | rs144960920 | 3 | 122660865 | T | C | -0.234 | 0.052 | 0.027 | 6.84E-06 | 7143 |
|  | rs268983 | 5 | 22651038 | A | T | 0.078 | 0.017 | 0.350 | 7.56E-06 | 7143 |
|  | rs143838492 | 5 | 114892988 | A | G | 0.673 | 0.149 | 0.003 | 6.07E-06 | 7143 |
|  | rs9324675 | 5 | 151110256 | T | C | 0.101 | 0.023 | 0.166 | 8.16E-06 | 7143 |
|  | rs79934453 | 6 | 23919006 | G | A | -0.616 | 0.137 | 0.004 | 7.04E-06 | 7143 |
|  | rs1061808 | 6 | 32168770 | G | T | 0.100 | 0.017 | 0.584 | 3.91E-09 | 7143 |
|  | rs113739902 | 6 | 66453262 | C | A | -0.572 | 0.128 | 0.005 | 7.97E-06 | 7143 |
|  | rs75178403 | 8 | 2825696 | C | T | -0.398 | 0.090 | 0.010 | 9.44E-06 | 7143 |
|  | rs117730598 | 9 | 2770506 | C | T | 0.235 | 0.050 | 0.030 | 2.99E-06 | 7143 |
|  | rs72753379 | 9 | 120350676 | T | C | 0.204 | 0.045 | 0.039 | 5.18E-06 | 7143 |
|  | rs480021 | 9 | 132455588 | A | C | 0.079 | 0.018 | 0.340 | 7.35E-06 | 7143 |
|  | rs112122274 | 9 | 136257994 | G | A | -0.240 | 0.049 | 0.036 | 7.85E-07 | 7143 |
|  | rs964184 | 11 | 116778201 | C | G | -0.162 | 0.023 | 0.849 | 3.12E-12 | 7143 |
|  | rs61906420 | 11 | 132660922 | A | G | 0.675 | 0.141 | 0.004 | 1.64E-06 | 7143 |
|  | rs10861498 | 12 | 105758014 | A | G | -0.089 | 0.019 | 0.242 | 3.20E-06 | 7143 |
|  | rs1884216 | 14 | 36679967 | A | C | -0.082 | 0.017 | 0.623 | 2.03E-06 | 7143 |
|  | rs75931581 | 15 | 39994335 | C | T | -0.202 | 0.041 | 0.042 | 1.13E-06 | 7143 |
|  | rs62016118 | 16 | 6791691 | C | A | -0.177 | 0.038 | 0.051 | 4.08E-06 | 7143 |
|  | rs187952596 | 17 | 78119940 | G | A | 0.214 | 0.048 | 0.033 | 8.37E-06 | 7143 |
|  | rs447117 | 18 | 5149977 | G | C | -0.078 | 0.017 | 0.651 | 6.56E-06 | 7143 |
| Triacylglycerol (50:2) | rs79416109 | 1 | 102847102 | G | T | 0.128 | 0.028 | 0.104 | 5.23E-06 | 7172 |
|  | rs78275146 | 1 | 196028959 | T | C | 0.340 | 0.075 | 0.014 | 6.59E-06 | 7172 |
|  | rs1260326 | 2 | 27508073 | C | T | -0.139 | 0.017 | 0.651 | 1.53E-15 | 7172 |
|  | rs111430300 | 2 | 36390910 | G | A | -0.112 | 0.024 | 0.135 | 3.36E-06 | 7172 |
|  | rs10213073 | 4 | 81524180 | G | T | -0.081 | 0.018 | 0.306 | 9.29E-06 | 7172 |
|  | rs143838492 | 5 | 114892988 | A | G | 0.723 | 0.148 | 0.003 | 1.15E-06 | 7172 |
|  | rs79934453 | 6 | 23919006 | G | A | -0.643 | 0.137 | 0.004 | 2.72E-06 | 7172 |
|  | rs15285 | 8 | 19967156 | T | C | -0.086 | 0.019 | 0.261 | 5.17E-06 | 7172 |
|  | rs1010313 | 8 | 59089983 | T | C | 0.093 | 0.021 | 0.198 | 8.23E-06 | 7172 |
|  | rs4615671 | 9 | 24032283 | T | C | 0.162 | 0.036 | 0.058 | 7.13E-06 | 7172 |
|  | rs72753379 | 9 | 120350676 | T | C | 0.216 | 0.045 | 0.039 | 1.42E-06 | 7172 |
|  | rs112122274 | 9 | 136257994 | G | A | -0.219 | 0.049 | 0.036 | 6.84E-06 | 7172 |
|  | rs964184 | 11 | 116778201 | C | G | -0.184 | 0.023 | 0.849 | 2.10E-15 | 7172 |
|  | rs10861498 | 12 | 105758014 | A | G | -0.085 | 0.019 | 0.242 | 9.89E-06 | 7172 |
|  | rs17704001 | 15 | 92583935 | T | G | 0.089 | 0.020 | 0.226 | 9.24E-06 | 7172 |
|  | rs187429064 | 19 | 19269704 | G | A | -0.172 | 0.038 | 0.054 | 5.47E-06 | 7172 |
|  | rs6033833 | 20 | 13842673 | G | C | -0.078 | 0.017 | 0.640 | 4.48E-06 | 7172 |
|  | rs79213832 | 22 | 23971765 | G | A | 0.164 | 0.036 | 0.060 | 4.31E-06 | 7172 |
| Triacylglycerol (50:3) | rs12120691 | 1 | 227027789 | C | T | -0.115 | 0.026 | 0.123 | 6.66E-06 | 7161 |
|  | rs1042034 | 2 | 21002409 | T | C | 0.099 | 0.019 | 0.728 | 1.25E-07 | 7161 |
|  | rs1260326 | 2 | 27508073 | C | T | -0.159 | 0.017 | 0.651 | 7.41E-20 | 7161 |
|  | rs111430300 | 2 | 36390910 | G | A | -0.111 | 0.024 | 0.135 | 4.66E-06 | 7161 |
|  | rs79771052 | 3 | 72433728 | G | A | 0.359 | 0.081 | 0.012 | 8.91E-06 | 7161 |
|  | rs116011373 | 3 | 87298641 | A | G | -0.186 | 0.039 | 0.049 | 2.25E-06 | 7161 |
|  | rs139499638 | 4 | 120830005 | C | G | -0.643 | 0.136 | 0.004 | 2.35E-06 | 7161 |
|  | rs143838492 | 5 | 114892988 | A | G | 0.676 | 0.148 | 0.003 | 5.34E-06 | 7161 |
|  | rs79934453 | 6 | 23919006 | G | A | -0.637 | 0.137 | 0.004 | 3.41E-06 | 7161 |
|  | rs79954170 | 6 | 49123122 | A | C | -0.440 | 0.096 | 0.008 | 4.32E-06 | 7161 |
|  | rs9344829 | 6 | 64177173 | T | C | 0.080 | 0.017 | 0.373 | 3.39E-06 | 7161 |
|  | rs35368205 | 7 | 73603327 | T | C | -0.103 | 0.022 | 0.167 | 4.03E-06 | 7161 |
|  | rs10105606 | 8 | 19970337 | A | C | -0.101 | 0.019 | 0.279 | 5.10E-08 | 7161 |
|  | rs1428264 | 8 | 59082215 | C | A | 0.099 | 0.021 | 0.197 | 2.24E-06 | 7161 |
|  | rs72738698 | 9 | 80090365 | A | G | 0.104 | 0.022 | 0.179 | 1.95E-06 | 7161 |
|  | rs72753379 | 9 | 120350676 | T | C | 0.208 | 0.045 | 0.039 | 3.22E-06 | 7161 |
|  | rs12344868 | 9 | 130572514 | A | C | 0.294 | 0.066 | 0.017 | 9.56E-06 | 7161 |
|  | rs10741876 | 11 | 3228978 | G | A | -0.101 | 0.022 | 0.832 | 6.10E-06 | 7161 |
|  | rs820903 | 11 | 38161116 | G | A | 0.140 | 0.031 | 0.917 | 7.51E-06 | 7161 |
|  | rs964184 | 11 | 116778201 | C | G | -0.213 | 0.023 | 0.849 | 4.05E-20 | 7161 |
|  | rs111526079 | 14 | 76971123 | A | G | 0.121 | 0.027 | 0.105 | 9.51E-06 | 7161 |
|  | rs72697234 | 14 | 92538920 | G | A | 0.211 | 0.046 | 0.036 | 5.49E-06 | 7161 |
|  | rs11849573 | 14 | 95566739 | A | G | -0.075 | 0.017 | 0.487 | 5.58E-06 | 7161 |
|  | rs17704001 | 15 | 92583935 | T | G | 0.090 | 0.020 | 0.226 | 7.71E-06 | 7161 |
|  | rs35986054 | 17 | 1623049 | C | T | 0.096 | 0.021 | 0.196 | 7.59E-06 | 7161 |
|  | rs113777805 | 18 | 23684659 | C | G | 0.219 | 0.049 | 0.031 | 8.15E-06 | 7161 |
|  | rs10502551 | 18 | 30887893 | G | C | -0.170 | 0.039 | 0.051 | 9.80E-06 | 7161 |
|  | rs187429064 | 19 | 19269704 | G | A | -0.235 | 0.038 | 0.054 | 5.20E-10 | 7161 |
|  | rs79152531 | 19 | 55802466 | C | T | -0.162 | 0.036 | 0.060 | 5.94E-06 | 7161 |
|  | rs79213832 | 22 | 23971765 | G | A | 0.166 | 0.036 | 0.060 | 3.16E-06 | 7161 |
| Triacylglycerol (50:4) | rs12120691 | 1 | 227027789 | C | T | -0.118 | 0.026 | 0.123 | 3.71E-06 | 7166 |
|  | rs1042034 | 2 | 21002409 | T | C | 0.093 | 0.019 | 0.728 | 5.96E-07 | 7166 |
|  | rs1260326 | 2 | 27508073 | C | T | -0.169 | 0.017 | 0.651 | 3.53E-22 | 7166 |
|  | rs301555 | 3 | 11749984 | G | A | 0.081 | 0.018 | 0.340 | 4.36E-06 | 7166 |
|  | rs116497138 | 3 | 87180483 | T | G | -0.184 | 0.039 | 0.050 | 2.68E-06 | 7166 |
|  | rs116635711 | 3 | 151540066 | A | G | 0.230 | 0.051 | 0.028 | 7.33E-06 | 7166 |
|  | rs17054866 | 5 | 157774262 | C | T | 0.116 | 0.026 | 0.119 | 6.86E-06 | 7166 |
|  | rs9344829 | 6 | 64177173 | T | C | 0.077 | 0.017 | 0.373 | 7.85E-06 | 7166 |
|  | rs11783556 | 8 | 764450 | G | A | -0.100 | 0.021 | 0.200 | 2.35E-06 | 7166 |
|  | rs10105606 | 8 | 19970337 | A | C | -0.097 | 0.019 | 0.279 | 1.54E-07 | 7166 |
|  | rs191616475 | 8 | 58330047 | G | A | 0.185 | 0.039 | 0.048 | 1.61E-06 | 7166 |
|  | rs117730598 | 9 | 2770506 | C | T | 0.236 | 0.050 | 0.030 | 2.61E-06 | 7166 |
|  | rs1832326 | 9 | 33656919 | C | A | 0.086 | 0.019 | 0.725 | 4.51E-06 | 7166 |
|  | rs72738698 | 9 | 80090365 | A | G | 0.105 | 0.022 | 0.179 | 1.44E-06 | 7166 |
|  | rs759126 | 9 | 120284301 | G | A | -0.214 | 0.047 | 0.964 | 5.56E-06 | 7166 |
|  | rs190411877 | 10 | 7871139 | C | T | 0.416 | 0.088 | 0.009 | 2.38E-06 | 7166 |
|  | rs72846885 | 11 | 7455964 | G | A | 0.135 | 0.030 | 0.086 | 6.30E-06 | 7166 |
|  | rs820903 | 11 | 38161116 | G | A | 0.140 | 0.031 | 0.917 | 7.48E-06 | 7166 |
|  | rs964184 | 11 | 116778201 | C | G | -0.203 | 0.023 | 0.849 | 2.01E-18 | 7166 |
|  | rs72697234 | 14 | 92538920 | G | A | 0.207 | 0.046 | 0.036 | 7.93E-06 | 7166 |
|  | rs2249682 | 15 | 61669154 | A | G | 0.074 | 0.017 | 0.525 | 7.64E-06 | 7166 |
|  | rs113777805 | 18 | 23684659 | C | G | 0.221 | 0.049 | 0.031 | 6.34E-06 | 7166 |
|  | rs10502551 | 18 | 30887893 | G | C | -0.172 | 0.039 | 0.051 | 8.00E-06 | 7166 |
|  | rs4453607 | 18 | 69122883 | T | C | -0.241 | 0.054 | 0.976 | 9.71E-06 | 7166 |
|  | rs668013 | 18 | 79647516 | A | G | 0.078 | 0.017 | 0.545 | 4.34E-06 | 7166 |
|  | rs187429064 | 19 | 19269704 | G | A | -0.247 | 0.038 | 0.054 | 7.59E-11 | 7166 |
|  | rs2546043 | 19 | 34971287 | G | A | -0.076 | 0.017 | 0.533 | 4.65E-06 | 7166 |
|  | rs79152531 | 19 | 55802466 | C | T | -0.177 | 0.036 | 0.060 | 7.55E-07 | 7166 |
|  | rs79213832 | 22 | 23971765 | G | A | 0.168 | 0.036 | 0.060 | 2.30E-06 | 7166 |
| Triacylglycerol (50:5) | rs3767910 | 1 | 25641071 | C | T | 0.105 | 0.024 | 0.211 | 9.28E-06 | 5491 |
|  | rs11208199 | 1 | 63377301 | A | G | -0.097 | 0.022 | 0.267 | 9.03E-06 | 5491 |
|  | rs1260326 | 2 | 27508073 | C | T | -0.157 | 0.020 | 0.651 | 2.77E-15 | 5491 |
|  | rs115914883 | 4 | 28034565 | T | G | 0.304 | 0.068 | 0.022 | 7.61E-06 | 5491 |
|  | rs3906419 | 5 | 108951913 | T | A | 0.108 | 0.023 | 0.220 | 2.28E-06 | 5491 |
|  | rs254668 | 5 | 157845455 | A | G | 0.101 | 0.019 | 0.442 | 1.28E-07 | 5491 |
|  | rs187087759 | 5 | 176875666 | G | C | -0.422 | 0.092 | 0.012 | 4.65E-06 | 5491 |
|  | rs78571096 | 7 | 71777939 | C | G | 0.526 | 0.118 | 0.007 | 7.87E-06 | 5491 |
|  | rs117906663 | 8 | 3605480 | A | G | -0.610 | 0.137 | 0.006 | 8.78E-06 | 5491 |
|  | rs143248091 | 8 | 9820239 | G | C | -0.837 | 0.184 | 0.003 | 5.73E-06 | 5491 |
|  | rs2954038 | 8 | 125495147 | A | C | -0.100 | 0.022 | 0.743 | 5.22E-06 | 5491 |
|  | rs67080320 | 8 | 139794453 | C | T | -0.203 | 0.044 | 0.051 | 3.25E-06 | 5491 |
|  | rs117730598 | 9 | 2770506 | C | T | 0.266 | 0.056 | 0.030 | 2.10E-06 | 5491 |
|  | rs11257904 | 10 | 12582085 | T | C | 0.573 | 0.126 | 0.006 | 5.45E-06 | 5491 |
|  | rs150889723 | 11 | 21740959 | C | T | -0.222 | 0.045 | 0.050 | 7.81E-07 | 5491 |
|  | rs181240184 | 11 | 78029938 | T | C | -0.305 | 0.068 | 0.021 | 7.15E-06 | 5491 |
|  | rs964184 | 11 | 116778201 | C | G | -0.184 | 0.026 | 0.849 | 1.16E-12 | 5491 |
|  | rs112578388 | 13 | 25886443 | T | C | -0.672 | 0.148 | 0.005 | 5.82E-06 | 5491 |
|  | rs116876951 | 16 | 27125614 | A | G | 0.495 | 0.110 | 0.008 | 7.37E-06 | 5491 |
|  | rs2278983 | 16 | 70692892 | A | C | 0.088 | 0.019 | 0.536 | 4.76E-06 | 5491 |
|  | rs7241605 | 18 | 44974706 | G | A | 0.148 | 0.032 | 0.104 | 2.82E-06 | 5491 |
|  | rs62125455 | 19 | 3058543 | T | C | 0.154 | 0.035 | 0.093 | 8.72E-06 | 5491 |
|  | rs187429064 | 19 | 19269704 | G | A | -0.269 | 0.044 | 0.054 | 1.62E-09 | 5491 |
|  | rs888860 | 19 | 34974283 | C | A | -0.094 | 0.020 | 0.621 | 1.39E-06 | 5491 |
|  | rs2915945 | 19 | 38522685 | C | T | 0.100 | 0.022 | 0.260 | 3.90E-06 | 5491 |
|  | rs72646948 | 22 | 19759510 | G | C | 0.849 | 0.163 | 0.004 | 1.85E-07 | 5491 |
| Triacylglycerol (51:1) | rs11102221 | 1 | 111242194 | A | G | -0.110 | 0.024 | 0.198 | 3.73E-06 | 5495 |
|  | rs1260326 | 2 | 27508073 | C | T | -0.102 | 0.020 | 0.651 | 2.96E-07 | 5495 |
|  | rs12613782 | 2 | 49039715 | C | T | 0.214 | 0.048 | 0.042 | 9.64E-06 | 5495 |
|  | rs67093838 | 2 | 62122935 | G | A | -0.251 | 0.057 | 0.030 | 9.88E-06 | 5495 |
|  | rs140828820 | 4 | 34767471 | G | A | 0.451 | 0.095 | 0.011 | 2.18E-06 | 5495 |
|  | rs2169099 | 4 | 62360675 | C | T | -0.117 | 0.024 | 0.199 | 1.21E-06 | 5495 |
|  | rs72801474 | 5 | 133108436 | A | G | -0.197 | 0.044 | 0.051 | 8.77E-06 | 5495 |
|  | rs71556711 | 7 | 73440219 | T | C | -0.147 | 0.033 | 0.095 | 6.93E-06 | 5495 |
|  | rs2242104 | 9 | 2640492 | G | A | -0.101 | 0.022 | 0.752 | 5.25E-06 | 5495 |
|  | rs832231 | 9 | 33414022 | G | A | -0.088 | 0.020 | 0.599 | 9.78E-06 | 5495 |
|  | rs1928423 | 9 | 71635471 | C | A | -0.102 | 0.021 | 0.647 | 8.56E-07 | 5495 |
|  | rs4979284 | 9 | 113789023 | A | G | -0.097 | 0.020 | 0.677 | 2.13E-06 | 5495 |
|  | rs111568723 | 9 | 132470502 | G | A | 0.338 | 0.062 | 0.026 | 5.37E-08 | 5495 |
|  | rs4448624 | 10 | 85979448 | A | G | -0.088 | 0.019 | 0.470 | 4.82E-06 | 5495 |
|  | rs7076657 | 10 | 132832356 | G | C | 0.180 | 0.039 | 0.065 | 4.21E-06 | 5495 |
|  | rs10767577 | 11 | 26777276 | A | T | 0.085 | 0.019 | 0.428 | 8.81E-06 | 5495 |
|  | rs143186626 | 11 | 96250298 | T | C | -0.173 | 0.038 | 0.070 | 6.47E-06 | 5495 |
|  | rs964184 | 11 | 116778201 | C | G | -0.140 | 0.026 | 0.849 | 9.62E-08 | 5495 |
|  | rs146238333 | 12 | 83707933 | T | A | 0.621 | 0.134 | 0.005 | 3.60E-06 | 5495 |
|  | rs34496841 | 17 | 51439207 | A | G | -0.112 | 0.022 | 0.237 | 5.15E-07 | 5495 |
|  | rs116845319 | 22 | 45070829 | A | G | 0.174 | 0.038 | 0.079 | 4.11E-06 | 5495 |
| Triacylglycerol (51:2) | rs4266911 | 1 | 27100305 | C | T | 0.160 | 0.036 | 0.941 | 9.76E-06 | 7149 |
|  | rs144241030 | 1 | 103243167 | T | C | 0.147 | 0.032 | 0.077 | 5.05E-06 | 7149 |
|  | rs1260326 | 2 | 27508073 | C | T | -0.128 | 0.017 | 0.651 | 2.46E-13 | 7149 |
|  | rs76950187 | 2 | 128427075 | G | A | 0.163 | 0.032 | 0.074 | 3.65E-07 | 7149 |
|  | rs6721083 | 2 | 170225142 | C | G | 0.161 | 0.035 | 0.937 | 4.43E-06 | 7149 |
|  | rs17282355 | 3 | 134977489 | T | C | -0.116 | 0.026 | 0.119 | 1.00E-05 | 7149 |
|  | rs139499638 | 4 | 120830005 | C | G | -0.617 | 0.136 | 0.004 | 6.00E-06 | 7149 |
|  | rs28437809 | 4 | 182501447 | G | T | 0.075 | 0.017 | 0.516 | 8.29E-06 | 7149 |
|  | rs143838492 | 5 | 114892988 | A | G | 0.681 | 0.148 | 0.003 | 4.53E-06 | 7149 |
|  | rs316343 | 6 | 2839825 | T | C | -0.076 | 0.017 | 0.468 | 9.37E-06 | 7149 |
|  | rs33951980 | 7 | 73615107 | T | C | -0.136 | 0.025 | 0.122 | 9.09E-08 | 7149 |
|  | rs117730598 | 9 | 2770506 | C | T | 0.231 | 0.050 | 0.030 | 4.35E-06 | 7149 |
|  | rs72753379 | 9 | 120350676 | T | C | 0.207 | 0.045 | 0.039 | 3.94E-06 | 7149 |
|  | rs28368829 | 9 | 136001733 | A | G | -0.088 | 0.020 | 0.240 | 8.07E-06 | 7149 |
|  | rs112122274 | 9 | 136257994 | G | A | -0.221 | 0.049 | 0.036 | 5.64E-06 | 7149 |
|  | rs174556 | 11 | 61813163 | T | C | 0.084 | 0.017 | 0.380 | 1.08E-06 | 7149 |
|  | rs964184 | 11 | 116778201 | C | G | -0.212 | 0.023 | 0.849 | 5.60E-20 | 7149 |
|  | rs75573052 | 13 | 21038010 | T | C | -0.135 | 0.030 | 0.085 | 9.70E-06 | 7149 |
|  | rs10147474 | 14 | 29823442 | G | A | -0.227 | 0.045 | 0.037 | 3.98E-07 | 7149 |
|  | rs62043594 | 15 | 93154154 | A | C | -0.080 | 0.017 | 0.418 | 2.16E-06 | 7149 |
|  | rs7412 | 19 | 44908822 | T | C | 0.168 | 0.037 | 0.053 | 7.41E-06 | 7149 |
| Triacylglycerol (51:3) | rs6692319 | 1 | 230148628 | T | C | -0.088 | 0.019 | 0.740 | 2.93E-06 | 7119 |
|  | rs1042034 | 2 | 21002409 | T | C | 0.107 | 0.019 | 0.728 | 1.01E-08 | 7119 |
|  | rs1260326 | 2 | 27508073 | C | T | -0.138 | 0.017 | 0.651 | 3.28E-15 | 7119 |
|  | rs12635725 | 3 | 1895465 | G | A | 0.075 | 0.017 | 0.560 | 7.13E-06 | 7119 |
|  | rs116011373 | 3 | 87298641 | A | G | -0.191 | 0.039 | 0.049 | 1.29E-06 | 7119 |
|  | rs35397574 | 3 | 148395586 | G | A | -0.080 | 0.017 | 0.483 | 2.72E-06 | 7119 |
|  | rs139278484 | 3 | 177023858 | C | A | 0.252 | 0.056 | 0.022 | 7.54E-06 | 7119 |
|  | rs11728165 | 4 | 107886862 | T | C | -0.148 | 0.032 | 0.080 | 4.08E-06 | 7119 |
|  | rs139499638 | 4 | 120830005 | C | G | -0.659 | 0.136 | 0.004 | 1.28E-06 | 7119 |
|  | rs138427786 | 4 | 163552148 | G | A | 0.302 | 0.067 | 0.016 | 5.76E-06 | 7119 |
|  | rs146131769 | 6 | 49493994 | G | A | -0.448 | 0.095 | 0.008 | 2.58E-06 | 7119 |
|  | rs7782961 | 7 | 6295952 | T | A | 0.090 | 0.020 | 0.232 | 7.66E-06 | 7119 |
|  | rs35332062 | 7 | 73597712 | A | G | -0.151 | 0.025 | 0.122 | 3.41E-09 | 7119 |
|  | rs15285 | 8 | 19967156 | T | C | -0.106 | 0.019 | 0.261 | 2.62E-08 | 7119 |
|  | rs7846649 | 8 | 59088496 | C | T | 0.095 | 0.021 | 0.191 | 8.14E-06 | 7119 |
|  | rs117730598 | 9 | 2770506 | C | T | 0.243 | 0.051 | 0.030 | 1.60E-06 | 7119 |
|  | rs111568723 | 9 | 132470502 | G | A | 0.242 | 0.055 | 0.026 | 9.58E-06 | 7119 |
|  | rs10767577 | 11 | 26777276 | A | T | 0.078 | 0.017 | 0.428 | 3.68E-06 | 7119 |
|  | rs964184 | 11 | 116778201 | C | G | -0.249 | 0.023 | 0.849 | 5.67E-27 | 7119 |
|  | rs117428564 | 12 | 20960665 | T | C | 0.466 | 0.102 | 0.007 | 5.38E-06 | 7119 |
|  | rs139500046 | 12 | 96850670 | C | A | -0.373 | 0.080 | 0.011 | 3.04E-06 | 7119 |
|  | rs1047974 | 12 | 108520819 | A | G | 0.143 | 0.032 | 0.076 | 6.08E-06 | 7119 |
|  | rs10147474 | 14 | 29823442 | G | A | -0.205 | 0.045 | 0.037 | 4.99E-06 | 7119 |
|  | rs111526079 | 14 | 76971123 | A | G | 0.125 | 0.027 | 0.105 | 5.14E-06 | 7119 |
|  | rs4791050 | 17 | 66543378 | A | T | 0.077 | 0.017 | 0.432 | 5.36E-06 | 7119 |
|  | rs187429064 | 19 | 19269704 | G | A | -0.226 | 0.038 | 0.054 | 3.17E-09 | 7119 |
|  | rs16996148 | 19 | 19547663 | T | G | -0.148 | 0.033 | 0.066 | 9.94E-06 | 7119 |
|  | rs390082 | 19 | 44913574 | G | T | 0.151 | 0.031 | 0.077 | 1.51E-06 | 7119 |
| Triacylglycerol (51:4) | rs2151252 | 1 | 170382059 | C | G | 0.101 | 0.022 | 0.250 | 7.12E-06 | 5237 |
|  | rs1260326 | 2 | 27508073 | C | T | -0.135 | 0.020 | 0.651 | 3.13E-11 | 5237 |
|  | rs149992851 | 3 | 55269076 | G | A | 0.338 | 0.069 | 0.020 | 1.17E-06 | 5237 |
|  | rs966039 | 3 | 66778832 | G | A | -0.127 | 0.027 | 0.164 | 2.09E-06 | 5237 |
|  | rs76308170 | 3 | 188269372 | C | T | 0.163 | 0.036 | 0.078 | 6.45E-06 | 5237 |
|  | rs62291572 | 4 | 4996200 | T | C | 0.119 | 0.024 | 0.214 | 1.05E-06 | 5237 |
|  | rs71605604 | 4 | 86536048 | T | C | 0.262 | 0.057 | 0.030 | 4.16E-06 | 5237 |
|  | rs2853923 | 6 | 31297960 | G | A | 0.095 | 0.019 | 0.505 | 1.03E-06 | 5237 |
|  | rs4709886 | 6 | 164722387 | C | A | 0.108 | 0.024 | 0.794 | 7.67E-06 | 5237 |
|  | rs11782705 | 8 | 4633421 | C | T | -0.200 | 0.042 | 0.059 | 2.02E-06 | 5237 |
|  | rs116923150 | 9 | 112114209 | A | G | 0.955 | 0.192 | 0.003 | 6.84E-07 | 5237 |
|  | rs10159864 | 10 | 17398917 | T | G | -0.778 | 0.174 | 0.003 | 7.89E-06 | 5237 |
|  | rs182295482 | 10 | 71191707 | C | T | -0.547 | 0.119 | 0.008 | 4.54E-06 | 5237 |
|  | rs964184 | 11 | 116778201 | C | G | -0.240 | 0.026 | 0.849 | 6.65E-20 | 5237 |
|  | rs7112577 | 11 | 117173887 | G | C | -0.190 | 0.042 | 0.058 | 7.28E-06 | 5237 |
|  | rs112421492 | 13 | 24038710 | T | A | -0.971 | 0.197 | 0.002 | 8.22E-07 | 5237 |
|  | rs17514333 | 13 | 105939568 | G | A | 0.110 | 0.024 | 0.209 | 3.41E-06 | 5237 |
|  | rs62002515 | 15 | 59004639 | C | T | -0.328 | 0.071 | 0.019 | 3.46E-06 | 5237 |
|  | rs62111901 | 19 | 13789855 | A | G | -0.125 | 0.027 | 0.171 | 2.57E-06 | 5237 |
|  | rs78327363 | 19 | 33235567 | A | G | -0.134 | 0.030 | 0.134 | 6.34E-06 | 5237 |
|  | rs438811 | 19 | 44913484 | T | C | 0.115 | 0.023 | 0.242 | 5.09E-07 | 5237 |
| Triacylglycerol (52:2) | rs144241030 | 1 | 103243167 | T | C | 0.150 | 0.032 | 0.077 | 3.26E-06 | 7168 |
|  | rs78275146 | 1 | 196028959 | T | C | 0.350 | 0.075 | 0.014 | 3.49E-06 | 7168 |
|  | rs34621709 | 1 | 220806736 | A | C | 0.108 | 0.022 | 0.185 | 5.87E-07 | 7168 |
|  | rs1042034 | 2 | 21002409 | T | C | 0.090 | 0.019 | 0.728 | 1.28E-06 | 7168 |
|  | rs1260326 | 2 | 27508073 | C | T | -0.122 | 0.017 | 0.651 | 2.79E-12 | 7168 |
|  | rs3770813 | 2 | 36549412 | T | G | -0.237 | 0.051 | 0.028 | 3.47E-06 | 7168 |
|  | rs147987331 | 2 | 104244849 | A | T | -0.261 | 0.057 | 0.023 | 3.96E-06 | 7168 |
|  | rs76950187 | 2 | 128427075 | G | A | 0.154 | 0.032 | 0.074 | 1.54E-06 | 7168 |
|  | rs10169217 | 2 | 212010621 | G | A | -0.084 | 0.018 | 0.676 | 2.59E-06 | 7168 |
|  | rs62291572 | 4 | 4996200 | T | C | 0.095 | 0.021 | 0.214 | 5.90E-06 | 7168 |
|  | rs501474 | 5 | 6523892 | G | A | 0.074 | 0.017 | 0.425 | 8.81E-06 | 7168 |
|  | rs150741556 | 5 | 83807154 | A | G | 0.238 | 0.052 | 0.026 | 5.63E-06 | 7168 |
|  | rs79934453 | 6 | 23919006 | G | A | -0.644 | 0.137 | 0.004 | 2.67E-06 | 7168 |
|  | rs79954170 | 6 | 49123122 | A | C | -0.443 | 0.096 | 0.008 | 3.85E-06 | 7168 |
|  | rs35332062 | 7 | 73597712 | A | G | -0.141 | 0.025 | 0.122 | 3.06E-08 | 7168 |
|  | rs15285 | 8 | 19967156 | T | C | -0.106 | 0.019 | 0.261 | 2.15E-08 | 7168 |
|  | rs1010313 | 8 | 59089983 | T | C | 0.097 | 0.021 | 0.198 | 3.42E-06 | 7168 |
|  | rs2980888 | 8 | 125495066 | C | T | -0.087 | 0.019 | 0.743 | 5.32E-06 | 7168 |
|  | rs72753379 | 9 | 120350676 | T | C | 0.206 | 0.045 | 0.039 | 4.08E-06 | 7168 |
|  | rs112122274 | 9 | 136257994 | G | A | -0.239 | 0.049 | 0.036 | 8.47E-07 | 7168 |
|  | rs35162925 | 10 | 62375635 | CA | C | -0.257 | 0.056 | 0.023 | 5.24E-06 | 7168 |
|  | rs11017845 | 10 | 131254190 | C | T | -0.176 | 0.040 | 0.047 | 9.65E-06 | 7168 |
|  | rs964184 | 11 | 116778201 | C | G | -0.248 | 0.023 | 0.849 | 7.83E-27 | 7168 |
|  | rs10147474 | 14 | 29823442 | G | A | -0.216 | 0.045 | 0.037 | 1.32E-06 | 7168 |
| Triacylglycerol (52:3) | rs55724405 | 1 | 8199214 | C | T | -0.082 | 0.018 | 0.283 | 9.56E-06 | 7173 |
|  | rs34621709 | 1 | 220806736 | A | C | 0.112 | 0.022 | 0.185 | 2.27E-07 | 7173 |
|  | rs2352723 | 1 | 230156121 | C | T | -0.114 | 0.020 | 0.769 | 5.93E-09 | 7173 |
|  | rs1042034 | 2 | 21002409 | T | C | 0.110 | 0.019 | 0.728 | 3.90E-09 | 7173 |
|  | rs1260326 | 2 | 27508073 | C | T | -0.137 | 0.017 | 0.651 | 4.16E-15 | 7173 |
|  | rs147987331 | 2 | 104244849 | A | T | -0.260 | 0.057 | 0.023 | 4.27E-06 | 7173 |
|  | rs116011373 | 3 | 87298641 | A | G | -0.176 | 0.039 | 0.049 | 7.27E-06 | 7173 |
|  | rs57500102 | 3 | 171536246 | A | G | -0.499 | 0.107 | 0.007 | 3.10E-06 | 7173 |
|  | rs76078494 | 3 | 189123018 | A | G | 0.209 | 0.046 | 0.035 | 6.73E-06 | 7173 |
|  | rs6554273 | 4 | 55360008 | T | C | 0.075 | 0.017 | 0.467 | 8.50E-06 | 7173 |
|  | rs9997195 | 4 | 113004554 | C | G | -0.081 | 0.017 | 0.613 | 2.61E-06 | 7173 |
|  | rs144313866 | 4 | 141550641 | A | G | -0.135 | 0.030 | 0.084 | 8.80E-06 | 7173 |
|  | rs79954170 | 6 | 49123122 | A | C | -0.511 | 0.096 | 0.008 | 9.54E-08 | 7173 |
|  | rs144072769 | 6 | 154916245 | C | T | 0.157 | 0.034 | 0.068 | 2.84E-06 | 7173 |
|  | rs35332062 | 7 | 73597712 | A | G | -0.160 | 0.025 | 0.122 | 3.11E-10 | 7173 |
|  | rs10105606 | 8 | 19970337 | A | C | -0.129 | 0.018 | 0.279 | 2.84E-12 | 7173 |
|  | rs1010313 | 8 | 59089983 | T | C | 0.103 | 0.021 | 0.198 | 6.95E-07 | 7173 |
|  | rs2980888 | 8 | 125495066 | C | T | -0.089 | 0.019 | 0.743 | 3.51E-06 | 7173 |
|  | rs16913024 | 10 | 59062519 | A | G | -0.457 | 0.103 | 0.008 | 9.81E-06 | 7173 |
|  | rs72836628 | 10 | 112142696 | T | C | -0.081 | 0.018 | 0.297 | 9.11E-06 | 7173 |
|  | rs174560 | 11 | 61814292 | C | T | 0.077 | 0.017 | 0.383 | 8.73E-06 | 7173 |
|  | rs12365864 | 11 | 116618319 | G | A | 0.112 | 0.023 | 0.166 | 6.71E-07 | 7173 |
|  | rs964184 | 11 | 116778201 | C | G | -0.299 | 0.023 | 0.849 | 2.31E-38 | 7173 |
|  | rs75573052 | 13 | 21038010 | T | C | -0.138 | 0.030 | 0.085 | 5.67E-06 | 7173 |
|  | rs61702480 | 14 | 95218464 | A | G | -0.084 | 0.019 | 0.275 | 8.15E-06 | 7173 |
|  | rs35986054 | 17 | 1623049 | C | T | 0.095 | 0.021 | 0.196 | 9.56E-06 | 7173 |
|  | rs75237020 | 17 | 72177274 | C | G | -0.216 | 0.047 | 0.035 | 4.96E-06 | 7173 |
|  | rs116843064 | 19 | 8364439 | A | G | -0.241 | 0.051 | 0.026 | 2.70E-06 | 7173 |
|  | rs187429064 | 19 | 19269704 | G | A | -0.185 | 0.038 | 0.054 | 9.80E-07 | 7173 |
|  | rs117950471 | 22 | 32729795 | T | C | 0.177 | 0.038 | 0.051 | 3.24E-06 | 7173 |
| Triacylglycerol (52:4) | rs34621709 | 1 | 220806736 | A | C | 0.110 | 0.022 | 0.185 | 3.33E-07 | 7174 |
|  | rs10779836 | 1 | 230167404 | T | C | -0.117 | 0.020 | 0.777 | 4.90E-09 | 7174 |
|  | rs1042034 | 2 | 21002409 | T | C | 0.106 | 0.019 | 0.728 | 1.28E-08 | 7174 |
|  | rs1260326 | 2 | 27508073 | C | T | -0.139 | 0.017 | 0.651 | 1.80E-15 | 7174 |
|  | rs76330538 | 2 | 212061574 | C | T | 0.257 | 0.058 | 0.022 | 9.76E-06 | 7174 |
|  | rs116497138 | 3 | 87180483 | T | G | -0.183 | 0.039 | 0.050 | 3.01E-06 | 7174 |
|  | rs56376607 | 4 | 99807593 | T | C | -0.220 | 0.050 | 0.030 | 9.24E-06 | 7174 |
|  | rs79954170 | 6 | 49123122 | A | C | -0.466 | 0.096 | 0.008 | 1.15E-06 | 7174 |
|  | rs9344829 | 6 | 64177173 | T | C | 0.078 | 0.017 | 0.373 | 5.25E-06 | 7174 |
|  | rs144072769 | 6 | 154916245 | C | T | 0.163 | 0.034 | 0.068 | 1.17E-06 | 7174 |
|  | rs79624003 | 7 | 73598455 | G | A | -0.141 | 0.025 | 0.122 | 2.70E-08 | 7174 |
|  | rs11783556 | 8 | 764450 | G | A | -0.095 | 0.021 | 0.200 | 6.06E-06 | 7174 |
|  | rs10105606 | 8 | 19970337 | A | C | -0.125 | 0.018 | 0.279 | 1.44E-11 | 7174 |
|  | rs13259370 | 8 | 59090254 | A | G | 0.099 | 0.021 | 0.197 | 2.11E-06 | 7174 |
|  | rs13261274 | 8 | 97226039 | G | A | -0.074 | 0.017 | 0.531 | 7.82E-06 | 7174 |
|  | rs142146849 | 10 | 101966464 | C | A | 0.160 | 0.036 | 0.055 | 9.83E-06 | 7174 |
|  | rs72900657 | 11 | 45826596 | A | G | -0.261 | 0.058 | 0.023 | 6.06E-06 | 7174 |
|  | rs12365864 | 11 | 116618319 | G | A | 0.105 | 0.023 | 0.166 | 3.23E-06 | 7174 |
|  | rs964184 | 11 | 116778201 | C | G | -0.284 | 0.023 | 0.849 | 6.53E-35 | 7174 |
|  | rs10773708 | 12 | 129796667 | A | G | -0.082 | 0.018 | 0.683 | 6.20E-06 | 7174 |
|  | rs61702480 | 14 | 95218464 | A | G | -0.083 | 0.019 | 0.275 | 9.36E-06 | 7174 |
|  | rs56051325 | 17 | 1615646 | G | A | 0.088 | 0.018 | 0.302 | 1.72E-06 | 7174 |
|  | rs61156392 | 17 | 49154037 | T | G | 0.114 | 0.025 | 0.121 | 6.83E-06 | 7174 |
|  | rs187429064 | 19 | 19269704 | G | A | -0.206 | 0.038 | 0.054 | 4.86E-08 | 7174 |
|  | rs113672231 | 19 | 33135815 | C | G | 0.139 | 0.031 | 0.078 | 7.46E-06 | 7174 |
|  | rs157582 | 19 | 44892962 | T | C | 0.095 | 0.020 | 0.223 | 2.29E-06 | 7174 |
| Triacylglycerol (52:5) | rs17596144 | 1 | 220805303 | T | C | 0.110 | 0.023 | 0.152 | 2.60E-06 | 7172 |
|  | rs10779836 | 1 | 230167404 | T | C | -0.094 | 0.020 | 0.777 | 2.30E-06 | 7172 |
|  | rs1042034 | 2 | 21002409 | T | C | 0.090 | 0.019 | 0.728 | 1.22E-06 | 7172 |
|  | rs1260326 | 2 | 27508073 | C | T | -0.154 | 0.017 | 0.651 | 1.01E-18 | 7172 |
|  | rs6753694 | 2 | 42239140 | G | C | -0.082 | 0.018 | 0.284 | 8.14E-06 | 7172 |
|  | rs301555 | 3 | 11749984 | G | A | 0.081 | 0.018 | 0.340 | 4.18E-06 | 7172 |
|  | rs116497138 | 3 | 87180483 | T | G | -0.174 | 0.039 | 0.050 | 8.43E-06 | 7172 |
|  | rs9344829 | 6 | 64177173 | T | C | 0.080 | 0.017 | 0.373 | 3.41E-06 | 7172 |
|  | rs144072769 | 6 | 154916245 | C | T | 0.157 | 0.034 | 0.068 | 2.86E-06 | 7172 |
|  | rs33951980 | 7 | 73615107 | T | C | -0.113 | 0.025 | 0.122 | 8.70E-06 | 7172 |
|  | rs11783556 | 8 | 764450 | G | A | -0.098 | 0.021 | 0.200 | 3.05E-06 | 7172 |
|  | rs10105606 | 8 | 19970337 | A | C | -0.112 | 0.018 | 0.279 | 1.58E-09 | 7172 |
|  | rs13259370 | 8 | 59090254 | A | G | 0.093 | 0.021 | 0.197 | 7.84E-06 | 7172 |
|  | rs4529433 | 8 | 100382190 | G | T | 0.084 | 0.019 | 0.268 | 9.02E-06 | 7172 |
|  | rs111568723 | 9 | 132470502 | G | A | 0.241 | 0.054 | 0.026 | 1.00E-05 | 7172 |
|  | rs190411877 | 10 | 7871139 | C | T | 0.428 | 0.088 | 0.009 | 1.15E-06 | 7172 |
|  | rs142146849 | 10 | 101966464 | C | A | 0.171 | 0.036 | 0.055 | 2.46E-06 | 7172 |
|  | rs72900657 | 11 | 45826596 | A | G | -0.273 | 0.058 | 0.023 | 2.21E-06 | 7172 |
|  | rs964184 | 11 | 116778201 | C | G | -0.251 | 0.023 | 0.849 | 1.59E-27 | 7172 |
|  | rs7996305 | 13 | 50548374 | T | C | 0.086 | 0.019 | 0.250 | 7.99E-06 | 7172 |
|  | rs35986054 | 17 | 1623049 | C | T | 0.100 | 0.021 | 0.196 | 2.77E-06 | 7172 |
|  | rs61156392 | 17 | 49154037 | C | G | 0.119 | 0.025 | 0.121 | 2.54E-06 | 7172 |
|  | rs75560495 | 17 | 79406653 | G | A | -0.121 | 0.027 | 0.128 | 6.03E-06 | 7172 |
|  | rs143467207 | 19 | 17375221 | A | G | 0.178 | 0.040 | 0.049 | 7.42E-06 | 7172 |
|  | rs187429064 | 19 | 19269704 | G | A | -0.241 | 0.038 | 0.054 | 1.76E-10 | 7172 |
|  | rs113672231 | 19 | 33135815 | C | G | 0.142 | 0.031 | 0.078 | 4.95E-06 | 7172 |
|  | rs157582 | 19 | 44892962 | T | C | 0.094 | 0.020 | 0.223 | 2.92E-06 | 7172 |
| Triacylglycerol (52:6) | rs78095833 | 1 | 237378917 | T | C | -0.306 | 0.064 | 0.020 | 2.01E-06 | 6554 |
|  | rs1260326 | 2 | 27508073 | C | T | -0.132 | 0.018 | 0.651 | 4.04E-13 | 6554 |
|  | rs6752909 | 2 | 45482958 | T | C | -0.104 | 0.023 | 0.826 | 7.19E-06 | 6554 |
|  | rs114314612 | 2 | 193027049 | A | C | 0.413 | 0.092 | 0.010 | 6.97E-06 | 6554 |
|  | rs4508726 | 3 | 37387931 | G | A | -0.087 | 0.018 | 0.365 | 1.77E-06 | 6554 |
|  | rs117518401 | 3 | 133895761 | T | C | 0.112 | 0.023 | 0.173 | 1.70E-06 | 6554 |
|  | rs78178542 | 4 | 179015062 | C | T | -0.313 | 0.065 | 0.020 | 1.59E-06 | 6554 |
|  | rs1017216 | 4 | 180460689 | A | G | 0.081 | 0.017 | 0.459 | 2.73E-06 | 6554 |
|  | rs56257715 | 7 | 71530474 | A | G | 0.428 | 0.093 | 0.009 | 3.70E-06 | 6554 |
|  | rs10105606 | 8 | 19970337 | A | C | -0.095 | 0.019 | 0.279 | 9.51E-07 | 6554 |
|  | rs7815372 | 8 | 64695903 | G | A | 0.098 | 0.022 | 0.792 | 5.52E-06 | 6554 |
|  | rs117082735 | 9 | 79867479 | T | C | -0.264 | 0.059 | 0.022 | 6.72E-06 | 6554 |
|  | rs140620869 | 10 | 94617201 | T | A | -0.384 | 0.086 | 0.010 | 7.67E-06 | 6554 |
|  | rs102274 | 11 | 61790354 | C | T | -0.132 | 0.018 | 0.409 | 1.00E-13 | 6554 |
|  | rs61905503 | 11 | 89627134 | C | G | -0.190 | 0.040 | 0.050 | 2.23E-06 | 6554 |
|  | rs964184 | 11 | 116778201 | C | G | -0.203 | 0.024 | 0.849 | 3.70E-17 | 6554 |
|  | rs112578388 | 13 | 25886443 | T | C | -0.576 | 0.126 | 0.005 | 5.22E-06 | 6554 |
|  | rs9591384 | 13 | 51065337 | A | T | -0.128 | 0.026 | 0.124 | 1.25E-06 | 6554 |
|  | rs7232381 | 18 | 79621226 | G | A | -0.082 | 0.018 | 0.373 | 6.68E-06 | 6554 |
|  | rs143467207 | 19 | 17375221 | A | G | 0.185 | 0.042 | 0.049 | 9.39E-06 | 6554 |
|  | rs182611493 | 19 | 19347579 | G | A | -0.275 | 0.040 | 0.054 | 6.08E-12 | 6554 |
|  | rs2546044 | 19 | 34971620 | G | A | -0.080 | 0.017 | 0.533 | 4.10E-06 | 6554 |
|  | rs445925 | 19 | 44912383 | A | G | 0.158 | 0.032 | 0.077 | 1.15E-06 | 6554 |
| Triacylglycerol (53:2) | rs4266911 | 1 | 27100305 | C | T | 0.165 | 0.037 | 0.941 | 6.96E-06 | 6996 |
|  | rs34621709 | 1 | 220806736 | A | C | 0.113 | 0.022 | 0.185 | 2.40E-07 | 6996 |
|  | rs1042034 | 2 | 21002409 | T | C | 0.084 | 0.019 | 0.728 | 9.40E-06 | 6996 |
|  | rs1260326 | 2 | 27508073 | C | T | -0.119 | 0.018 | 0.651 | 1.73E-11 | 6996 |
|  | rs76950187 | 2 | 128427075 | G | A | 0.157 | 0.032 | 0.074 | 1.17E-06 | 6996 |
|  | rs12635725 | 3 | 1895465 | G | A | 0.076 | 0.017 | 0.560 | 7.41E-06 | 6996 |
|  | rs116497138 | 3 | 87180483 | T | G | -0.184 | 0.040 | 0.050 | 3.54E-06 | 6996 |
|  | rs11926107 | 3 | 134995496 | G | A | -0.083 | 0.019 | 0.284 | 8.32E-06 | 6996 |
|  | rs111241638 | 3 | 168112642 | C | T | -0.222 | 0.050 | 0.033 | 8.97E-06 | 6996 |
|  | rs55870249 | 3 | 177504180 | G | C | 0.091 | 0.020 | 0.225 | 7.99E-06 | 6996 |
|  | rs62291572 | 4 | 4996200 | T | C | 0.103 | 0.021 | 0.214 | 1.20E-06 | 6996 |
|  | rs72679249 | 4 | 110201006 | G | A | 0.092 | 0.019 | 0.270 | 2.06E-06 | 6996 |
|  | rs150741556 | 5 | 83807154 | A | G | 0.235 | 0.053 | 0.026 | 8.45E-06 | 6996 |
|  | rs34021847 | 7 | 1998126 | A | G | -0.088 | 0.018 | 0.320 | 1.03E-06 | 6996 |
|  | rs35332062 | 7 | 73597712 | A | G | -0.134 | 0.026 | 0.122 | 2.21E-07 | 6996 |
|  | rs15285 | 8 | 19967156 | T | C | -0.097 | 0.019 | 0.261 | 4.10E-07 | 6996 |
|  | rs174556 | 11 | 61813163 | T | C | 0.095 | 0.017 | 0.380 | 5.17E-08 | 6996 |
|  | rs964184 | 11 | 116778201 | C | G | -0.233 | 0.023 | 0.849 | 2.38E-23 | 6996 |
|  | rs75573052 | 13 | 21038010 | T | C | -0.142 | 0.031 | 0.085 | 4.79E-06 | 6996 |
|  | rs10147474 | 14 | 29823442 | G | A | -0.219 | 0.045 | 0.037 | 1.54E-06 | 6996 |
|  | rs62043594 | 15 | 93154154 | A | C | -0.091 | 0.017 | 0.418 | 9.63E-08 | 6996 |
|  | rs390082 | 19 | 44913574 | G | T | 0.144 | 0.032 | 0.077 | 5.53E-06 | 6996 |
| Triacylglycerol (53:3) | rs11584070 | 1 | 150322476 | G | A | -0.114 | 0.024 | 0.137 | 2.83E-06 | 7113 |
|  | rs34621709 | 1 | 220806736 | A | C | 0.124 | 0.022 | 0.185 | 9.47E-09 | 7113 |
|  | rs6692319 | 1 | 230148628 | T | C | -0.095 | 0.019 | 0.740 | 4.41E-07 | 7113 |
|  | rs1042034 | 2 | 21002409 | T | C | 0.097 | 0.019 | 0.728 | 2.12E-07 | 7113 |
|  | rs1260326 | 2 | 27508073 | C | T | -0.137 | 0.017 | 0.651 | 4.37E-15 | 7113 |
|  | rs76950187 | 2 | 128427075 | G | A | 0.148 | 0.032 | 0.074 | 4.23E-06 | 7113 |
|  | rs12635725 | 3 | 1895465 | G | A | 0.081 | 0.017 | 0.560 | 1.46E-06 | 7113 |
|  | rs28450659 | 3 | 15332543 | T | C | -0.119 | 0.026 | 0.112 | 6.66E-06 | 7113 |
|  | rs116011373 | 3 | 87298641 | A | G | -0.192 | 0.039 | 0.049 | 1.02E-06 | 7113 |
|  | rs62291574 | 4 | 5003811 | A | G | 0.099 | 0.021 | 0.214 | 2.61E-06 | 7113 |
|  | rs501474 | 5 | 6523892 | G | A | 0.077 | 0.017 | 0.425 | 5.14E-06 | 7113 |
|  | rs150741556 | 5 | 83807154 | A | G | 0.258 | 0.052 | 0.026 | 9.24E-07 | 7113 |
|  | rs35332062 | 7 | 73597712 | A | G | -0.148 | 0.026 | 0.122 | 7.43E-09 | 7113 |
|  | rs15285 | 8 | 19967156 | T | C | -0.111 | 0.019 | 0.261 | 4.62E-09 | 7113 |
|  | rs3829088 | 9 | 23713682 | C | T | 0.092 | 0.021 | 0.203 | 9.16E-06 | 7113 |
|  | rs111568723 | 9 | 132470502 | G | A | 0.250 | 0.055 | 0.026 | 4.67E-06 | 7113 |
|  | rs174560 | 11 | 61814292 | C | T | 0.104 | 0.017 | 0.383 | 1.96E-09 | 7113 |
|  | rs964184 | 11 | 116778201 | C | G | -0.283 | 0.023 | 0.849 | 2.83E-34 | 7113 |
|  | rs138622921 | 12 | 26757135 | C | A | -0.291 | 0.065 | 0.017 | 6.86E-06 | 7113 |
|  | rs139500046 | 12 | 96850670 | C | A | -0.381 | 0.079 | 0.011 | 1.56E-06 | 7113 |
|  | rs935833 | 12 | 129791836 | A | G | -0.079 | 0.018 | 0.635 | 7.19E-06 | 7113 |
|  | rs1003966 | 13 | 21038020 | T | C | -0.150 | 0.030 | 0.085 | 9.16E-07 | 7113 |
|  | rs10147474 | 14 | 29823442 | G | A | -0.211 | 0.045 | 0.037 | 2.67E-06 | 7113 |
|  | rs12448223 | 16 | 4104550 | C | T | 0.077 | 0.017 | 0.443 | 3.77E-06 | 7113 |
|  | rs1989995 | 16 | 71133811 | T | C | -0.086 | 0.019 | 0.254 | 6.83E-06 | 7113 |
| Triacylglycerol (53:4) | rs351596 | 1 | 4317851 | T | C | -0.262 | 0.058 | 0.975 | 7.43E-06 | 6479 |
|  | rs116014876 | 1 | 109657509 | T | C | 0.420 | 0.094 | 0.009 | 7.33E-06 | 6479 |
|  | rs34621709 | 1 | 220806736 | A | C | 0.102 | 0.023 | 0.185 | 5.63E-06 | 6479 |
|  | rs1260326 | 2 | 27508073 | C | T | -0.136 | 0.018 | 0.651 | 1.14E-13 | 6479 |
|  | rs1949011 | 2 | 141741698 | A | C | -0.087 | 0.018 | 0.429 | 1.19E-06 | 6479 |
|  | rs12635725 | 3 | 1895465 | G | A | 0.079 | 0.018 | 0.560 | 6.57E-06 | 6479 |
|  | rs79235240 | 3 | 42816309 | A | C | 0.218 | 0.047 | 0.038 | 3.14E-06 | 6479 |
|  | rs138987797 | 3 | 190557173 | T | A | 0.776 | 0.159 | 0.003 | 1.04E-06 | 6479 |
|  | rs62291573 | 4 | 4999649 | C | T | 0.100 | 0.022 | 0.214 | 6.00E-06 | 6479 |
|  | rs144500033 | 4 | 27061504 | T | A | -0.765 | 0.170 | 0.003 | 7.15E-06 | 6479 |
|  | rs2546584 | 4 | 180456342 | T | C | 0.077 | 0.017 | 0.463 | 9.37E-06 | 6479 |
|  | rs55672000 | 5 | 66234813 | A | G | -0.255 | 0.057 | 0.027 | 7.10E-06 | 6479 |
|  | rs247164 | 5 | 128702919 | C | T | 0.297 | 0.066 | 0.983 | 7.48E-06 | 6479 |
|  | rs28362364 | 6 | 31202352 | A | T | 0.110 | 0.024 | 0.159 | 5.48E-06 | 6479 |
|  | rs71556711 | 7 | 73440219 | T | C | -0.141 | 0.030 | 0.095 | 2.40E-06 | 6479 |
|  | rs117829392 | 8 | 771605 | C | G | 0.233 | 0.051 | 0.032 | 5.50E-06 | 6479 |
|  | rs10105606 | 8 | 19970337 | A | C | -0.112 | 0.020 | 0.279 | 1.29E-08 | 6479 |
|  | rs7846649 | 8 | 59088496 | C | T | 0.126 | 0.022 | 0.191 | 1.58E-08 | 6479 |
|  | rs13288401 | 9 | 1147365 | T | C | -0.236 | 0.052 | 0.032 | 6.82E-06 | 6479 |
|  | rs3829088 | 9 | 23713682 | C | T | 0.097 | 0.022 | 0.203 | 9.59E-06 | 6479 |
|  | rs4837740 | 9 | 120256064 | G | A | -0.202 | 0.045 | 0.955 | 6.30E-06 | 6479 |
|  | rs36174561 | 11 | 38197193 | A | G | -0.149 | 0.032 | 0.087 | 3.64E-06 | 6479 |
|  | rs964184 | 11 | 116778201 | C | G | -0.274 | 0.024 | 0.849 | 5.39E-30 | 6479 |
|  | rs2370511 | 12 | 78389910 | C | T | 0.116 | 0.024 | 0.157 | 1.44E-06 | 6479 |
|  | rs139500046 | 12 | 96850670 | C | A | -0.402 | 0.083 | 0.011 | 1.52E-06 | 6479 |
|  | rs17081481 | 13 | 34561042 | G | A | 0.087 | 0.019 | 0.334 | 7.33E-06 | 6479 |
|  | rs7152096 | 14 | 97793198 | A | G | 0.104 | 0.023 | 0.190 | 7.83E-06 | 6479 |
|  | rs825692 | 16 | 73556815 | A | G | -0.139 | 0.029 | 0.110 | 1.65E-06 | 6479 |
|  | rs35832030 | 17 | 1624088 | T | G | 0.104 | 0.023 | 0.196 | 4.30E-06 | 6479 |
|  | rs117922651 | 17 | 41828494 | A | G | -0.177 | 0.039 | 0.057 | 4.79E-06 | 6479 |
|  | rs8100204 | 19 | 19282905 | A | G | -0.114 | 0.024 | 0.171 | 1.43E-06 | 6479 |
|  | rs338593 | 19 | 41198399 | C | T | 0.079 | 0.018 | 0.577 | 9.36E-06 | 6479 |
|  | rs157582 | 19 | 44892962 | T | C | 0.099 | 0.021 | 0.223 | 3.00E-06 | 6479 |
|  | rs11570257 | 20 | 59301745 | A | G | 0.316 | 0.068 | 0.019 | 4.00E-06 | 6479 |
| Triacylglycerol (54:3) | rs2643912 | 1 | 2352403 | T | G | -0.079 | 0.017 | 0.451 | 2.67E-06 | 7162 |
|  | rs2642442 | 1 | 220800221 | T | C | -0.117 | 0.018 | 0.693 | 1.42E-10 | 7162 |
|  | rs2281721 | 1 | 230161390 | T | C | -0.078 | 0.017 | 0.567 | 4.41E-06 | 7162 |
|  | rs1042034 | 2 | 21002409 | T | C | 0.095 | 0.019 | 0.728 | 3.97E-07 | 7162 |
|  | rs1260326 | 2 | 27508073 | C | T | -0.095 | 0.017 | 0.651 | 5.61E-08 | 7162 |
|  | rs147987331 | 2 | 104244849 | A | T | -0.252 | 0.057 | 0.023 | 8.45E-06 | 7162 |
|  | rs76950187 | 2 | 128427075 | G | A | 0.150 | 0.032 | 0.074 | 2.79E-06 | 7162 |
|  | rs10169217 | 2 | 212010621 | G | A | -0.083 | 0.018 | 0.676 | 3.95E-06 | 7162 |
|  | rs7638534 | 3 | 1898506 | T | G | 0.078 | 0.017 | 0.349 | 8.59E-06 | 7162 |
|  | rs62291572 | 4 | 4996200 | T | C | 0.110 | 0.021 | 0.214 | 1.63E-07 | 7162 |
|  | rs3934642 | 4 | 81683582 | G | C | -0.126 | 0.027 | 0.115 | 2.55E-06 | 7162 |
|  | rs78540742 | 5 | 114571608 | A | T | 0.246 | 0.055 | 0.024 | 8.35E-06 | 7162 |
|  | rs141116593 | 6 | 154880792 | G | C | 0.150 | 0.032 | 0.074 | 3.15E-06 | 7162 |
|  | rs3812316 | 7 | 73606007 | G | C | -0.120 | 0.025 | 0.121 | 2.41E-06 | 7162 |
|  | rs13237172 | 7 | 155371896 | A | G | -0.172 | 0.038 | 0.054 | 6.67E-06 | 7162 |
|  | rs144679576 | 8 | 2896369 | C | T | -0.374 | 0.080 | 0.012 | 3.18E-06 | 7162 |
|  | rs10105606 | 8 | 19970337 | A | C | -0.113 | 0.019 | 0.279 | 9.69E-10 | 7162 |
|  | rs2980888 | 8 | 125495066 | C | T | -0.088 | 0.019 | 0.743 | 4.60E-06 | 7162 |
|  | rs112122274 | 9 | 136257994 | G | A | -0.217 | 0.049 | 0.036 | 7.89E-06 | 7162 |
|  | rs17333900 | 10 | 88675523 | T | G | 0.118 | 0.026 | 0.123 | 3.84E-06 | 7162 |
|  | rs72836628 | 10 | 112142696 | T | C | -0.087 | 0.018 | 0.297 | 1.79E-06 | 7162 |
|  | rs174560 | 11 | 61814292 | C | T | 0.133 | 0.017 | 0.383 | 1.13E-14 | 7162 |
|  | rs56252769 | 11 | 65951162 | C | T | 0.090 | 0.020 | 0.229 | 7.50E-06 | 7162 |
|  | rs12365864 | 11 | 116618319 | G | A | 0.116 | 0.023 | 0.166 | 3.12E-07 | 7162 |
|  | rs964184 | 11 | 116778201 | C | G | -0.286 | 0.023 | 0.849 | 3.91E-35 | 7162 |
|  | rs112465335 | 12 | 10312813 | C | A | 0.182 | 0.041 | 0.045 | 7.99E-06 | 7162 |
|  | rs112576575 | 12 | 20732159 | G | A | -0.408 | 0.090 | 0.010 | 6.74E-06 | 7162 |
|  | rs11068322 | 12 | 117079732 | C | G | 0.094 | 0.021 | 0.192 | 7.76E-06 | 7162 |
|  | rs9578336 | 13 | 20972374 | G | C | 0.098 | 0.022 | 0.182 | 5.84E-06 | 7162 |
|  | rs137871178 | 14 | 58138012 | C | T | 0.350 | 0.066 | 0.017 | 1.15E-07 | 7162 |
| Triacylglycerol (54:4) | rs2643912 | 1 | 2352403 | T | G | -0.075 | 0.017 | 0.451 | 9.42E-06 | 7165 |
|  | rs351596 | 1 | 4317851 | T | C | -0.247 | 0.056 | 0.975 | 9.46E-06 | 7165 |
|  | rs59017319 | 1 | 74445900 | A | G | 0.184 | 0.041 | 0.047 | 5.56E-06 | 7165 |
|  | rs2642442 | 1 | 220800221 | T | C | -0.120 | 0.018 | 0.693 | 4.79E-11 | 7165 |
|  | rs10779833 | 1 | 230157290 | G | A | -0.093 | 0.018 | 0.657 | 1.21E-07 | 7165 |
|  | rs1042034 | 2 | 21002409 | T | C | 0.102 | 0.019 | 0.728 | 4.38E-08 | 7165 |
|  | rs1260326 | 2 | 27508073 | C | T | -0.105 | 0.017 | 0.651 | 1.95E-09 | 7165 |
|  | rs2941755 | 2 | 79833869 | C | T | -0.169 | 0.037 | 0.943 | 4.22E-06 | 7165 |
|  | rs62291573 | 4 | 4999649 | C | T | 0.098 | 0.021 | 0.214 | 3.24E-06 | 7165 |
|  | rs17160706 | 5 | 107893061 | C | A | 0.098 | 0.022 | 0.180 | 5.77E-06 | 7165 |
|  | rs144072769 | 6 | 154916245 | C | T | 0.167 | 0.034 | 0.068 | 6.72E-07 | 7165 |
|  | rs73022136 | 6 | 165581086 | T | C | -0.196 | 0.043 | 0.044 | 4.96E-06 | 7165 |
|  | rs78433420 | 7 | 44922206 | T | C | -0.268 | 0.059 | 0.021 | 5.58E-06 | 7165 |
|  | rs3812316 | 7 | 73606007 | G | C | -0.124 | 0.025 | 0.121 | 1.09E-06 | 7165 |
|  | rs10105606 | 8 | 19970337 | A | C | -0.124 | 0.018 | 0.279 | 2.21E-11 | 7165 |
|  | rs72836628 | 10 | 112142696 | T | C | -0.090 | 0.018 | 0.297 | 8.55E-07 | 7165 |
|  | rs189281327 | 11 | 40018396 | A | G | -0.149 | 0.033 | 0.071 | 7.91E-06 | 7165 |
|  | rs174604 | 11 | 61858798 | G | C | 0.098 | 0.017 | 0.372 | 1.71E-08 | 7165 |
|  | rs12365864 | 11 | 116618319 | G | A | 0.122 | 0.023 | 0.166 | 7.80E-08 | 7165 |
|  | rs964184 | 11 | 116778201 | C | G | -0.301 | 0.023 | 0.849 | 9.12E-39 | 7165 |
|  | rs10773708 | 12 | 129796667 | A | G | -0.089 | 0.018 | 0.683 | 9.43E-07 | 7165 |
|  | rs137871178 | 14 | 58138012 | C | T | 0.337 | 0.066 | 0.017 | 3.40E-07 | 7165 |
|  | rs61702480 | 14 | 95218464 | A | G | -0.083 | 0.019 | 0.275 | 8.61E-06 | 7165 |
|  | rs56051325 | 17 | 1615646 | G | A | 0.085 | 0.018 | 0.302 | 3.91E-06 | 7165 |
|  | rs116843064 | 19 | 8364439 | A | G | -0.237 | 0.051 | 0.026 | 4.18E-06 | 7165 |
|  | rs115215688 | 19 | 33148516 | G | C | 0.168 | 0.037 | 0.055 | 4.31E-06 | 7165 |
|  | rs438811 | 19 | 44913484 | T | C | 0.091 | 0.020 | 0.242 | 3.66E-06 | 7165 |
| Triacylglycerol (54:5) | rs351596 | 1 | 4317851 | T | C | -0.252 | 0.056 | 0.975 | 5.89E-06 | 7146 |
|  | rs59017319 | 1 | 74445900 | A | G | 0.187 | 0.041 | 0.047 | 4.20E-06 | 7146 |
|  | rs17596144 | 1 | 220805303 | T | C | 0.130 | 0.023 | 0.152 | 2.84E-08 | 7146 |
|  | rs2352723 | 1 | 230156121 | C | T | -0.097 | 0.020 | 0.769 | 7.46E-07 | 7146 |
|  | rs1042034 | 2 | 21002409 | T | C | 0.092 | 0.019 | 0.728 | 8.05E-07 | 7146 |
|  | rs1260326 | 2 | 27508073 | C | T | -0.122 | 0.017 | 0.651 | 3.24E-12 | 7146 |
|  | rs115614772 | 2 | 87205327 | T | C | 0.479 | 0.106 | 0.007 | 6.30E-06 | 7146 |
|  | rs301555 | 3 | 11749984 | G | A | 0.079 | 0.018 | 0.340 | 8.98E-06 | 7146 |
|  | rs1456674 | 3 | 148163041 | C | T | -0.451 | 0.095 | 0.009 | 2.10E-06 | 7146 |
|  | rs17160706 | 5 | 107893061 | C | A | 0.097 | 0.022 | 0.180 | 8.00E-06 | 7146 |
|  | rs13178172 | 5 | 118341860 | G | A | 0.090 | 0.019 | 0.739 | 1.53E-06 | 7146 |
|  | rs147151674 | 6 | 98110337 | T | A | 0.357 | 0.079 | 0.012 | 5.61E-06 | 7146 |
|  | rs144072769 | 6 | 154916245 | C | T | 0.164 | 0.034 | 0.068 | 1.03E-06 | 7146 |
|  | rs13246993 | 7 | 73608416 | A | G | -0.122 | 0.025 | 0.121 | 1.57E-06 | 7146 |
|  | rs139245678 | 8 | 16479793 | T | C | -0.230 | 0.051 | 0.030 | 7.90E-06 | 7146 |
|  | rs10105606 | 8 | 19970337 | A | C | -0.116 | 0.019 | 0.279 | 4.47E-10 | 7146 |
|  | rs664178 | 9 | 33414691 | G | A | -0.076 | 0.017 | 0.438 | 8.74E-06 | 7146 |
|  | rs12365864 | 11 | 116618319 | G | A | 0.113 | 0.023 | 0.166 | 6.92E-07 | 7146 |
|  | rs964184 | 11 | 116778201 | C | G | -0.268 | 0.023 | 0.849 | 5.22E-31 | 7146 |
|  | rs10773708 | 12 | 129796667 | A | G | -0.087 | 0.018 | 0.683 | 1.43E-06 | 7146 |
|  | rs9578336 | 13 | 20972374 | G | C | 0.097 | 0.022 | 0.182 | 7.48E-06 | 7146 |
|  | rs79380657 | 13 | 89358643 | A | G | -0.261 | 0.058 | 0.023 | 5.97E-06 | 7146 |
|  | rs72671922 | 14 | 21794664 | G | A | -0.150 | 0.034 | 0.067 | 9.57E-06 | 7146 |
|  | rs137871178 | 14 | 58138012 | C | T | 0.298 | 0.066 | 0.017 | 6.67E-06 | 7146 |
|  | rs56051325 | 17 | 1615646 | G | A | 0.091 | 0.018 | 0.302 | 8.23E-07 | 7146 |
|  | rs74994260 | 19 | 33147680 | G | C | 0.191 | 0.039 | 0.048 | 1.28E-06 | 7146 |
|  | rs438811 | 19 | 44913484 | T | C | 0.092 | 0.020 | 0.242 | 2.59E-06 | 7146 |
| Triacylglycerol (54:6) | rs12405870 | 1 | 187918746 | T | A | -0.083 | 0.018 | 0.315 | 5.13E-06 | 7066 |
|  | rs17596144 | 1 | 220805303 | T | C | 0.110 | 0.024 | 0.152 | 3.22E-06 | 7066 |
|  | rs417237 | 1 | 228344494 | T | G | 0.076 | 0.017 | 0.590 | 7.77E-06 | 7066 |
|  | rs1260326 | 2 | 27508073 | C | T | -0.112 | 0.018 | 0.651 | 1.83E-10 | 7066 |
|  | rs114561185 | 2 | 204066660 | G | A | 0.617 | 0.129 | 0.005 | 1.70E-06 | 7066 |
|  | rs1456674 | 3 | 148163041 | C | T | -0.437 | 0.096 | 0.009 | 5.16E-06 | 7066 |
|  | rs4057791 | 4 | 163303980 | C | A | 0.081 | 0.017 | 0.546 | 1.72E-06 | 7066 |
|  | rs13178172 | 5 | 118341860 | G | A | 0.091 | 0.019 | 0.739 | 1.30E-06 | 7066 |
|  | rs9344829 | 6 | 64177173 | T | C | 0.077 | 0.017 | 0.373 | 8.10E-06 | 7066 |
|  | rs181609235 | 6 | 164617090 | G | A | -0.351 | 0.078 | 0.012 | 6.54E-06 | 7066 |
|  | rs268 | 8 | 19956018 | G | A | 0.294 | 0.057 | 0.023 | 3.09E-07 | 7066 |
|  | rs140647506 | 9 | 31005163 | A | G | 0.467 | 0.103 | 0.007 | 5.83E-06 | 7066 |
|  | rs190411877 | 10 | 7871139 | C | T | 0.432 | 0.089 | 0.009 | 1.08E-06 | 7066 |
|  | rs174554 | 11 | 61811991 | G | A | -0.098 | 0.017 | 0.406 | 9.75E-09 | 7066 |
|  | rs964184 | 11 | 116778201 | C | G | -0.218 | 0.023 | 0.849 | 6.27E-21 | 7066 |
|  | rs56051325 | 17 | 1615646 | G | A | 0.092 | 0.019 | 0.302 | 8.01E-07 | 7066 |
|  | rs6501273 | 17 | 78966282 | T | C | 0.096 | 0.021 | 0.793 | 5.34E-06 | 7066 |
|  | rs182611493 | 19 | 19347579 | G | A | -0.195 | 0.038 | 0.054 | 2.97E-07 | 7066 |
|  | rs445925 | 19 | 44912383 | A | G | 0.155 | 0.031 | 0.077 | 7.83E-07 | 7066 |
|  | rs8123587 | 20 | 46633119 | A | C | 0.122 | 0.027 | 0.111 | 6.47E-06 | 7066 |
| Triacylglycerol (54:7) | rs4970780 | 1 | 109791954 | A | T | 0.089 | 0.019 | 0.327 | 3.47E-06 | 6828 |
|  | rs76481684 | 1 | 217318646 | G | A | 0.265 | 0.057 | 0.023 | 3.18E-06 | 6828 |
|  | rs417237 | 1 | 228344494 | T | G | 0.081 | 0.017 | 0.590 | 3.02E-06 | 6828 |
|  | rs111853669 | 1 | 230959661 | A | G | -0.158 | 0.034 | 0.071 | 2.58E-06 | 6828 |
|  | rs12120498 | 1 | 246000079 | C | T | 0.094 | 0.019 | 0.697 | 4.10E-07 | 6828 |
|  | rs1260326 | 2 | 27508073 | C | T | -0.103 | 0.018 | 0.651 | 8.72E-09 | 6828 |
|  | rs4858271 | 3 | 20815484 | C | T | -0.079 | 0.017 | 0.424 | 6.08E-06 | 6828 |
|  | rs73192177 | 3 | 122621089 | T | C | -0.319 | 0.070 | 0.015 | 5.59E-06 | 6828 |
|  | rs138383428 | 4 | 185010479 | T | G | -0.585 | 0.131 | 0.005 | 8.67E-06 | 6828 |
|  | rs12210453 | 6 | 129737300 | G | A | -0.188 | 0.042 | 0.047 | 7.46E-06 | 6828 |
|  | rs377037955 | 7 | 4768282 | A | G | 0.792 | 0.172 | 0.003 | 3.97E-06 | 6828 |
|  | rs74516326 | 7 | 71293164 | T | C | -0.098 | 0.022 | 0.196 | 6.58E-06 | 6828 |
|  | rs73660137 | 8 | 5570990 | G | C | -0.365 | 0.083 | 0.011 | 9.79E-06 | 6828 |
|  | rs34942551 | 8 | 20012504 | G | T | -0.092 | 0.020 | 0.253 | 3.11E-06 | 6828 |
|  | rs62534834 | 9 | 1496121 | G | A | -0.167 | 0.036 | 0.061 | 3.72E-06 | 6828 |
|  | rs140647506 | 9 | 31005163 | A | G | 0.464 | 0.103 | 0.007 | 6.41E-06 | 6828 |
|  | rs190411877 | 10 | 7871139 | C | T | 0.423 | 0.089 | 0.009 | 1.91E-06 | 6828 |
|  | rs2192081 | 10 | 57923245 | A | G | 0.089 | 0.019 | 0.269 | 4.07E-06 | 6828 |
|  | rs184609620 | 10 | 94386362 | T | C | -0.395 | 0.089 | 0.009 | 8.31E-06 | 6828 |
|  | rs174584 | 11 | 61843278 | A | G | -0.126 | 0.017 | 0.410 | 5.09E-13 | 6828 |
|  | rs9645690 | 11 | 62438816 | T | C | -0.085 | 0.018 | 0.322 | 2.74E-06 | 6828 |
|  | rs964184 | 11 | 116778201 | C | G | -0.174 | 0.024 | 0.849 | 1.70E-13 | 6828 |
|  | rs56870907 | 14 | 104290084 | C | G | 0.122 | 0.026 | 0.135 | 3.61E-06 | 6828 |
|  | rs35986054 | 17 | 1623049 | C | T | 0.097 | 0.022 | 0.196 | 9.38E-06 | 6828 |
|  | rs182611493 | 19 | 19347579 | G | A | -0.227 | 0.039 | 0.054 | 5.05E-09 | 6828 |
|  | rs16996148 | 19 | 19547663 | T | G | -0.161 | 0.034 | 0.066 | 3.16E-06 | 6828 |
| Triacylglycerol (56:3) | rs11399 | 1 | 27886295 | T | C | 0.146 | 0.030 | 0.100 | 1.19E-06 | 6172 |
|  | rs4908327 | 1 | 99527521 | T | C | -0.127 | 0.027 | 0.866 | 2.03E-06 | 6172 |
|  | rs1539733 | 1 | 162720456 | C | T | 0.098 | 0.022 | 0.794 | 7.49E-06 | 6172 |
|  | rs17596144 | 1 | 220805303 | T | C | 0.162 | 0.025 | 0.152 | 1.11E-10 | 6172 |
|  | rs10169217 | 2 | 212010621 | G | A | -0.099 | 0.019 | 0.676 | 2.63E-07 | 6172 |
|  | rs9851701 | 3 | 103282265 | A | G | 0.119 | 0.026 | 0.148 | 3.58E-06 | 6172 |
|  | rs62291572 | 4 | 4996200 | T | C | 0.108 | 0.023 | 0.214 | 1.86E-06 | 6172 |
|  | rs10058653 | 5 | 109602550 | T | G | -0.089 | 0.019 | 0.302 | 4.77E-06 | 6172 |
|  | rs75008187 | 6 | 64441454 | A | G | 0.136 | 0.027 | 0.128 | 6.10E-07 | 6172 |
|  | rs2980862 | 8 | 125472396 | C | G | -0.101 | 0.021 | 0.743 | 8.68E-07 | 6172 |
|  | rs145737110 | 9 | 122973735 | T | C | 0.387 | 0.084 | 0.014 | 4.77E-06 | 6172 |
|  | rs111568723 | 9 | 132470502 | G | A | 0.287 | 0.059 | 0.026 | 1.05E-06 | 6172 |
|  | rs174560 | 11 | 61814292 | C | T | 0.170 | 0.018 | 0.383 | 3.95E-20 | 6172 |
|  | rs149199066 | 11 | 65863732 | A | G | 0.208 | 0.044 | 0.044 | 2.92E-06 | 6172 |
|  | rs116846412 | 11 | 79066210 | A | G | -0.244 | 0.053 | 0.032 | 3.92E-06 | 6172 |
|  | rs964184 | 11 | 116778201 | C | G | -0.238 | 0.024 | 0.849 | 4.19E-22 | 6172 |
|  | rs117786044 | 11 | 128757016 | A | G | -0.238 | 0.051 | 0.034 | 2.83E-06 | 6172 |
|  | rs58355654 | 13 | 22883681 | A | G | 0.119 | 0.027 | 0.129 | 9.76E-06 | 6172 |
|  | rs62016118 | 16 | 6791691 | C | A | -0.185 | 0.042 | 0.051 | 9.92E-06 | 6172 |
|  | rs117819583 | 16 | 23267355 | A | G | -0.251 | 0.055 | 0.029 | 5.16E-06 | 6172 |
|  | rs181023072 | 16 | 62982329 | A | C | -0.850 | 0.178 | 0.002 | 1.72E-06 | 6172 |
|  | rs11151739 | 18 | 72273093 | G | A | 0.086 | 0.019 | 0.346 | 5.08E-06 | 6172 |
|  | rs7412 | 19 | 44908822 | T | C | 0.220 | 0.040 | 0.053 | 3.91E-08 | 6172 |
|  | rs138845231 | 21 | 24358586 | G | A | -0.202 | 0.046 | 0.041 | 9.89E-06 | 6172 |
| Triacylglycerol (56:4) | rs34621709 | 1 | 220806736 | A | C | 0.148 | 0.024 | 0.185 | 5.58E-10 | 5820 |
|  | rs10169217 | 2 | 212010621 | G | A | -0.095 | 0.020 | 0.676 | 1.53E-06 | 5820 |
|  | rs62291572 | 4 | 4996200 | T | C | 0.110 | 0.023 | 0.214 | 2.19E-06 | 5820 |
|  | rs35120448 | 4 | 95245119 | T | C | -0.235 | 0.053 | 0.034 | 9.11E-06 | 5820 |
|  | rs75533654 | 4 | 142175079 | C | A | -0.336 | 0.076 | 0.015 | 9.90E-06 | 5820 |
|  | rs141116593 | 6 | 154880792 | G | C | 0.161 | 0.035 | 0.074 | 4.83E-06 | 5820 |
|  | rs62475137 | 7 | 115692953 | A | G | 0.192 | 0.043 | 0.052 | 9.80E-06 | 5820 |
|  | rs2978946 | 8 | 6766077 | G | T | 0.085 | 0.019 | 0.551 | 7.07E-06 | 5820 |
|  | rs117883640 | 8 | 56921257 | G | T | 0.203 | 0.042 | 0.052 | 1.35E-06 | 5820 |
|  | rs10966037 | 9 | 23714477 | T | A | 0.109 | 0.023 | 0.202 | 1.94E-06 | 5820 |
|  | rs10430989 | 11 | 43542682 | A | G | -0.116 | 0.023 | 0.202 | 6.63E-07 | 5820 |
|  | rs174592 | 11 | 61851136 | G | A | 0.136 | 0.019 | 0.418 | 4.89E-13 | 5820 |
|  | rs964184 | 11 | 116778201 | C | G | -0.242 | 0.025 | 0.849 | 2.14E-21 | 5820 |
|  | rs36094040 | 12 | 72431183 | C | T | -0.093 | 0.020 | 0.348 | 2.67E-06 | 5820 |
|  | rs61942065 | 12 | 129794726 | T | C | 0.109 | 0.022 | 0.216 | 1.15E-06 | 5820 |
|  | rs12448223 | 16 | 4104550 | C | T | 0.082 | 0.018 | 0.443 | 1.00E-05 | 5820 |
|  | rs390082 | 19 | 44913574 | G | T | 0.173 | 0.034 | 0.077 | 5.27E-07 | 5820 |
|  | rs73082153 | 20 | 983640 | T | C | -0.391 | 0.086 | 0.013 | 5.27E-06 | 5820 |
|  | rs1883350 | 22 | 43932163 | C | T | 0.099 | 0.019 | 0.391 | 2.12E-07 | 5820 |
| Triacylglycerol (56:5) | rs182009884 | 1 | 182723688 | C | G | -0.237 | 0.052 | 0.035 | 4.98E-06 | 5490 |
|  | rs34621709 | 1 | 220806736 | A | C | 0.125 | 0.025 | 0.185 | 3.71E-07 | 5490 |
|  | rs10169217 | 2 | 212010621 | G | A | -0.104 | 0.020 | 0.676 | 3.51E-07 | 5490 |
|  | rs149473233 | 3 | 6179893 | A | G | 0.699 | 0.149 | 0.005 | 2.64E-06 | 5490 |
|  | rs11127989 | 3 | 87531960 | A | G | 0.152 | 0.034 | 0.913 | 7.06E-06 | 5490 |
|  | rs9682946 | 3 | 189075054 | G | A | 0.171 | 0.034 | 0.088 | 5.66E-07 | 5490 |
|  | rs78081886 | 4 | 27694435 | A | G | 0.358 | 0.077 | 0.015 | 3.52E-06 | 5490 |
|  | rs73125564 | 4 | 32591008 | T | A | 0.105 | 0.022 | 0.278 | 2.68E-06 | 5490 |
|  | rs4073968 | 4 | 38490214 | T | C | -0.102 | 0.023 | 0.234 | 9.99E-06 | 5490 |
|  | rs9997979 | 4 | 99524202 | T | C | 0.285 | 0.062 | 0.024 | 4.44E-06 | 5490 |
|  | rs75768973 | 6 | 109778182 | T | C | -0.325 | 0.074 | 0.018 | 9.80E-06 | 5490 |
|  | rs12193589 | 6 | 163939775 | A | G | 0.091 | 0.020 | 0.450 | 7.08E-06 | 5490 |
|  | rs1441764 | 8 | 20005513 | T | C | -0.133 | 0.021 | 0.280 | 3.60E-10 | 5490 |
|  | rs34527930 | 9 | 114264016 | T | C | -0.127 | 0.028 | 0.138 | 5.54E-06 | 5490 |
|  | rs2038844 | 10 | 112611346 | T | G | 0.242 | 0.053 | 0.964 | 6.10E-06 | 5490 |
|  | rs1472230 | 11 | 4653826 | A | G | 0.088 | 0.019 | 0.422 | 6.60E-06 | 5490 |
|  | rs97384 | 11 | 61856709 | C | T | 0.154 | 0.019 | 0.580 | 3.45E-15 | 5490 |
|  | rs964184 | 11 | 116778201 | C | G | -0.210 | 0.026 | 0.849 | 4.58E-16 | 5490 |
|  | rs61960432 | 13 | 83982225 | G | A | -0.151 | 0.031 | 0.115 | 8.22E-07 | 5490 |
|  | rs11623520 | 14 | 95282764 | A | G | -0.092 | 0.020 | 0.650 | 4.02E-06 | 5490 |
|  | rs60102597 | 17 | 78155616 | C | T | 0.121 | 0.027 | 0.154 | 6.74E-06 | 5490 |
|  | rs4608457 | 19 | 20106360 | T | C | -0.218 | 0.047 | 0.045 | 3.98E-06 | 5490 |
|  | rs390082 | 19 | 44913574 | G | T | 0.178 | 0.035 | 0.077 | 3.18E-07 | 5490 |
|  | rs738409 | 22 | 43928847 | G | C | 0.145 | 0.023 | 0.224 | 1.65E-10 | 5490 |
| Triacylglycerol (56:6) | rs17596144 | 1 | 220805303 | T | C | 0.111 | 0.023 | 0.152 | 2.05E-06 | 7151 |
|  | rs56296027 | 2 | 20934251 | C | T | -0.088 | 0.018 | 0.285 | 1.60E-06 | 7151 |
|  | rs1260326 | 2 | 27508073 | C | T | -0.118 | 0.017 | 0.651 | 1.60E-11 | 7151 |
|  | rs114971431 | 2 | 56529283 | G | A | 0.348 | 0.076 | 0.013 | 4.45E-06 | 7151 |
|  | rs197730 | 3 | 37455234 | G | A | -0.087 | 0.017 | 0.402 | 3.38E-07 | 7151 |
|  | rs187665388 | 3 | 176326805 | T | C | -0.248 | 0.052 | 0.027 | 1.93E-06 | 7151 |
|  | rs3096698 | 6 | 32139362 | C | T | -0.077 | 0.017 | 0.631 | 9.21E-06 | 7151 |
|  | rs115007623 | 6 | 44048675 | C | T | 0.402 | 0.089 | 0.010 | 5.98E-06 | 7151 |
|  | rs4277987 | 6 | 168930674 | G | A | -0.095 | 0.020 | 0.241 | 1.14E-06 | 7151 |
|  | rs33951980 | 7 | 73615107 | T | C | -0.128 | 0.025 | 0.122 | 4.81E-07 | 7151 |
|  | rs1089588 | 7 | 86663619 | G | T | 0.083 | 0.019 | 0.264 | 9.81E-06 | 7151 |
|  | rs10105606 | 8 | 19970337 | A | C | -0.130 | 0.018 | 0.279 | 2.19E-12 | 7151 |
|  | rs2954021 | 8 | 125469835 | G | A | -0.080 | 0.017 | 0.538 | 1.66E-06 | 7151 |
|  | rs7846376 | 8 | 130143197 | A | G | -0.080 | 0.018 | 0.637 | 5.53E-06 | 7151 |
|  | rs7863337 | 9 | 71634286 | T | C | -0.137 | 0.030 | 0.095 | 3.65E-06 | 7151 |
|  | rs10739560 | 9 | 120268566 | C | T | -0.219 | 0.046 | 0.962 | 2.07E-06 | 7151 |
|  | rs56013388 | 10 | 74276153 | T | C | -0.379 | 0.083 | 0.010 | 4.59E-06 | 7151 |
|  | rs174527 | 11 | 61770929 | C | G | -0.078 | 0.017 | 0.444 | 5.30E-06 | 7151 |
|  | rs174533 | 11 | 61781553 | A | G | -0.233 | 0.017 | 0.409 | 8.17E-43 | 7151 |
|  | rs964184 | 11 | 116778201 | C | G | -0.241 | 0.023 | 0.849 | 2.07E-25 | 7151 |
|  | rs9578336 | 13 | 20972374 | G | C | 0.100 | 0.022 | 0.182 | 4.07E-06 | 7151 |
|  | rs187032204 | 16 | 82427963 | C | T | 0.280 | 0.062 | 0.019 | 6.51E-06 | 7151 |
|  | rs2968430 | 16 | 85292036 | G | C | -0.104 | 0.023 | 0.155 | 8.42E-06 | 7151 |
|  | rs187429064 | 19 | 19269704 | G | A | -0.297 | 0.038 | 0.054 | 5.28E-15 | 7151 |
|  | rs16996148 | 19 | 19547663 | T | G | -0.171 | 0.033 | 0.066 | 3.15E-07 | 7151 |
|  | rs445925 | 19 | 44912383 | A | G | 0.194 | 0.031 | 0.077 | 4.64E-10 | 7151 |
|  | rs114160087 | 20 | 15006201 | C | A | 0.520 | 0.116 | 0.005 | 7.95E-06 | 7151 |
|  | rs738409 | 22 | 43928847 | G | C | 0.178 | 0.020 | 0.224 | 5.90E-19 | 7151 |
| Triacylglycerol (56:7) | rs6699175 | 1 | 72049213 | T | C | 0.124 | 0.027 | 0.114 | 3.27E-06 | 7148 |
|  | rs592586 | 1 | 109650768 | G | A | 0.080 | 0.017 | 0.595 | 2.10E-06 | 7148 |
|  | rs1260326 | 2 | 27508073 | C | T | -0.099 | 0.017 | 0.651 | 1.79E-08 | 7148 |
|  | rs7608044 | 2 | 74005215 | A | G | -0.127 | 0.028 | 0.104 | 7.11E-06 | 7148 |
|  | rs73095198 | 3 | 72073972 | A | G | -0.228 | 0.051 | 0.029 | 8.52E-06 | 7148 |
|  | rs595400 | 3 | 166723434 | C | A | -0.076 | 0.017 | 0.525 | 6.59E-06 | 7148 |
|  | rs112214356 | 3 | 196197114 | C | T | 0.164 | 0.035 | 0.061 | 3.46E-06 | 7148 |
|  | rs80329613 | 4 | 75629568 | A | G | -0.164 | 0.037 | 0.054 | 8.55E-06 | 7148 |
|  | rs1902483 | 4 | 155035692 | G | T | 0.076 | 0.017 | 0.505 | 5.63E-06 | 7148 |
|  | rs72767411 | 5 | 57685337 | A | T | 0.296 | 0.066 | 0.017 | 6.92E-06 | 7148 |
|  | rs115007623 | 6 | 44048675 | C | T | 0.403 | 0.089 | 0.010 | 6.41E-06 | 7148 |
|  | rs62419698 | 6 | 105463186 | G | C | -0.172 | 0.037 | 0.061 | 3.81E-06 | 7148 |
|  | rs6904245 | 6 | 134421023 | A | G | 0.145 | 0.031 | 0.081 | 4.28E-06 | 7148 |
|  | rs3916027 | 8 | 19967357 | A | G | -0.126 | 0.019 | 0.253 | 6.22E-11 | 7148 |
|  | rs7002672 | 8 | 64694359 | A | G | 0.095 | 0.021 | 0.805 | 6.36E-06 | 7148 |
|  | rs75402627 | 9 | 130364541 | C | T | 0.156 | 0.032 | 0.075 | 1.59E-06 | 7148 |
|  | rs174554 | 11 | 61811991 | G | A | -0.161 | 0.017 | 0.406 | 2.72E-21 | 7148 |
|  | rs2229738 | 11 | 68794860 | T | C | -0.121 | 0.023 | 0.156 | 2.81E-07 | 7148 |
|  | rs964184 | 11 | 116778201 | C | G | -0.216 | 0.023 | 0.849 | 1.24E-20 | 7148 |
|  | rs513272 | 12 | 129053394 | C | T | 0.079 | 0.017 | 0.564 | 4.97E-06 | 7148 |
|  | rs72623355 | 13 | 55008165 | C | T | -0.133 | 0.028 | 0.097 | 2.64E-06 | 7148 |
|  | rs7156531 | 14 | 36233601 | G | C | 0.122 | 0.027 | 0.114 | 6.40E-06 | 7148 |
|  | rs148132478 | 14 | 74300719 | T | C | -0.736 | 0.163 | 0.003 | 6.59E-06 | 7148 |
|  | rs10149017 | 14 | 95049182 | G | A | 0.093 | 0.021 | 0.796 | 9.44E-06 | 7148 |
|  | rs2626606 | 14 | 97502998 | A | G | -0.110 | 0.024 | 0.862 | 5.80E-06 | 7148 |
|  | rs7170012 | 15 | 59864454 | G | A | -0.076 | 0.017 | 0.579 | 7.03E-06 | 7148 |
|  | rs34001747 | 17 | 16960373 | G | C | 0.358 | 0.081 | 0.012 | 1.00E-05 | 7148 |
|  | rs182611493 | 19 | 19347579 | G | A | -0.271 | 0.038 | 0.054 | 8.42E-13 | 7148 |
|  | rs16996148 | 19 | 19547663 | T | G | -0.151 | 0.034 | 0.066 | 7.18E-06 | 7148 |
|  | rs57706029 | 19 | 21770359 | T | C | 0.079 | 0.017 | 0.605 | 3.93E-06 | 7148 |
|  | rs483082 | 19 | 44912921 | T | G | 0.105 | 0.020 | 0.242 | 7.96E-08 | 7148 |
|  | rs150578762 | 22 | 28083968 | A | G | 0.133 | 0.029 | 0.094 | 4.11E-06 | 7148 |
|  | rs1883350 | 22 | 43932163 | C | T | 0.088 | 0.017 | 0.391 | 3.37E-07 | 7148 |
| Triacylglycerol (56:8) | rs592586 | 1 | 109650768 | G | A | 0.082 | 0.017 | 0.595 | 2.57E-06 | 6800 |
|  | rs78095833 | 1 | 237378917 | T | C | -0.285 | 0.064 | 0.020 | 8.05E-06 | 6800 |
|  | rs1260326 | 2 | 27508073 | C | T | -0.086 | 0.018 | 0.651 | 1.90E-06 | 6800 |
|  | rs11892100 | 2 | 40100036 | C | G | -0.170 | 0.038 | 0.056 | 5.67E-06 | 6800 |
|  | rs62258666 | 3 | 35239965 | A | G | 0.499 | 0.108 | 0.007 | 3.69E-06 | 6800 |
|  | rs7621165 | 3 | 166672944 | A | G | -0.081 | 0.017 | 0.450 | 2.75E-06 | 6800 |
|  | rs2647110 | 4 | 124768047 | C | G | 0.093 | 0.020 | 0.240 | 4.68E-06 | 6800 |
|  | rs4711782 | 6 | 44395493 | T | A | 0.144 | 0.032 | 0.079 | 8.44E-06 | 6800 |
|  | rs62419698 | 6 | 105463186 | G | C | -0.184 | 0.038 | 0.061 | 1.62E-06 | 6800 |
|  | rs13205789 | 6 | 137580166 | C | T | -0.120 | 0.026 | 0.120 | 5.38E-06 | 6800 |
|  | rs10480656 | 7 | 104552272 | G | C | 0.092 | 0.019 | 0.266 | 1.63E-06 | 6800 |
|  | rs141329532 | 7 | 144901272 | G | C | 0.686 | 0.152 | 0.003 | 7.02E-06 | 6800 |
|  | rs4726178 | 7 | 152575262 | T | C | -0.097 | 0.022 | 0.201 | 6.81E-06 | 6800 |
|  | rs2165558 | 8 | 19998749 | G | T | -0.111 | 0.019 | 0.271 | 6.70E-09 | 6800 |
|  | rs7815372 | 8 | 64695903 | G | A | 0.099 | 0.021 | 0.792 | 2.68E-06 | 6800 |
|  | rs57588467 | 8 | 92429509 | A | G | -0.079 | 0.018 | 0.368 | 7.59E-06 | 6800 |
|  | rs11142645 | 9 | 70932129 | A | G | -0.193 | 0.043 | 0.043 | 6.61E-06 | 6800 |
|  | rs7031579 | 9 | 130374888 | T | C | -0.147 | 0.032 | 0.920 | 4.18E-06 | 6800 |
|  | rs174530 | 11 | 61779120 | G | A | -0.141 | 0.017 | 0.414 | 9.01E-16 | 6800 |
|  | rs2229738 | 11 | 68794860 | T | C | -0.137 | 0.024 | 0.156 | 1.48E-08 | 6800 |
|  | rs964184 | 11 | 116778201 | C | G | -0.194 | 0.024 | 0.849 | 4.73E-16 | 6800 |
|  | rs72623355 | 13 | 55008165 | C | T | -0.132 | 0.029 | 0.097 | 6.70E-06 | 6800 |
|  | rs61967369 | 13 | 73092472 | C | A | -0.161 | 0.035 | 0.064 | 3.71E-06 | 6800 |
|  | rs60543421 | 13 | 107454127 | A | G | -0.292 | 0.065 | 0.018 | 7.35E-06 | 6800 |
|  | rs61994001 | 14 | 78906415 | A | T | -0.610 | 0.120 | 0.006 | 4.12E-07 | 6800 |
|  | rs8028182 | 15 | 75426328 | T | G | -0.103 | 0.022 | 0.188 | 3.45E-06 | 6800 |
|  | rs117564891 | 15 | 82134529 | G | A | -0.212 | 0.046 | 0.037 | 4.80E-06 | 6800 |
|  | rs11649869 | 17 | 49540307 | G | A | 0.105 | 0.023 | 0.182 | 3.75E-06 | 6800 |
|  | rs182611493 | 19 | 19347579 | G | A | -0.259 | 0.039 | 0.054 | 5.15E-11 | 6800 |
|  | rs16996148 | 19 | 19547663 | T | G | -0.153 | 0.035 | 0.066 | 9.22E-06 | 6800 |
|  | rs57706029 | 19 | 21770359 | T | C | 0.080 | 0.018 | 0.605 | 5.72E-06 | 6800 |
|  | rs438811 | 19 | 44913484 | T | C | 0.108 | 0.020 | 0.242 | 8.66E-08 | 6800 |
| Triacylglycerol (58:7) | rs592586 | 1 | 109650768 | G | A | 0.091 | 0.019 | 0.595 | 2.91E-06 | 5403 |
|  | rs34621709 | 1 | 220806736 | A | C | 0.111 | 0.025 | 0.185 | 8.13E-06 | 5403 |
|  | rs1260326 | 2 | 27508073 | C | T | -0.119 | 0.020 | 0.651 | 2.66E-09 | 5403 |
|  | rs7764213 | 6 | 112137404 | C | T | 0.108 | 0.024 | 0.195 | 7.83E-06 | 5403 |
|  | rs117926062 | 6 | 138930517 | T | G | -0.454 | 0.101 | 0.009 | 7.65E-06 | 5403 |
|  | rs12700064 | 7 | 19617082 | A | G | 0.158 | 0.033 | 0.900 | 2.12E-06 | 5403 |
|  | rs15285 | 8 | 19967156 | T | C | -0.123 | 0.022 | 0.261 | 1.99E-08 | 5403 |
|  | rs3011337 | 10 | 120774281 | G | A | 0.099 | 0.021 | 0.673 | 1.85E-06 | 5403 |
|  | rs102275 | 11 | 61790331 | C | T | -0.159 | 0.020 | 0.410 | 5.95E-16 | 5403 |
|  | rs145956593 | 11 | 88458623 | A | G | -0.122 | 0.028 | 0.141 | 8.97E-06 | 5403 |
|  | rs964184 | 11 | 116778201 | C | G | -0.210 | 0.026 | 0.849 | 1.38E-15 | 5403 |
|  | rs1258712 | 12 | 31313616 | T | G | 0.180 | 0.039 | 0.931 | 4.08E-06 | 5403 |
|  | rs75214296 | 12 | 43836589 | G | A | 0.125 | 0.028 | 0.138 | 5.94E-06 | 5403 |
|  | rs141765432 | 14 | 76516128 | T | G | -0.263 | 0.053 | 0.035 | 8.36E-07 | 5403 |
|  | rs67076780 | 18 | 78936828 | T | C | -0.282 | 0.063 | 0.024 | 6.79E-06 | 5403 |
|  | rs187429064 | 19 | 19269704 | G | A | -0.216 | 0.045 | 0.054 | 1.60E-06 | 5403 |
|  | rs111473971 | 19 | 21652047 | A | G | 0.138 | 0.030 | 0.121 | 5.11E-06 | 5403 |
|  | rs445925 | 19 | 44912383 | A | G | 0.221 | 0.035 | 0.077 | 5.08E-10 | 5403 |
|  | rs62209231 | 20 | 13843242 | C | T | 0.088 | 0.020 | 0.354 | 9.08E-06 | 5403 |
|  | rs371927 | 20 | 44634395 | A | G | -0.177 | 0.037 | 0.915 | 1.50E-06 | 5403 |
|  | rs150578762 | 22 | 28083968 | A | G | 0.155 | 0.033 | 0.094 | 2.52E-06 | 5403 |
|  | rs1883350 | 22 | 43932163 | C | T | 0.114 | 0.020 | 0.391 | 6.13E-09 | 5403 |
| Triacylglycerol (58:8) | rs506912 | 1 | 109649355 | A | C | 0.093 | 0.019 | 0.705 | 1.46E-06 | 6384 |
|  | rs6429013 | 1 | 237429307 | A | C | 0.116 | 0.026 | 0.860 | 6.87E-06 | 6384 |
|  | rs12476545 | 2 | 29060519 | G | C | 0.126 | 0.028 | 0.115 | 6.61E-06 | 6384 |
|  | rs79294676 | 2 | 67277529 | A | G | 0.280 | 0.062 | 0.022 | 6.81E-06 | 6384 |
|  | rs116095614 | 3 | 2139069 | A | G | 0.495 | 0.107 | 0.007 | 3.78E-06 | 6384 |
|  | rs67872791 | 3 | 65548155 | G | T | 0.505 | 0.107 | 0.007 | 2.40E-06 | 6384 |
|  | rs62321577 | 4 | 75860202 | T | G | -0.206 | 0.040 | 0.050 | 3.13E-07 | 6384 |
|  | rs62419698 | 6 | 105463186 | G | C | -0.178 | 0.039 | 0.061 | 6.66E-06 | 6384 |
|  | rs34718616 | 6 | 137598572 | G | A | -0.124 | 0.027 | 0.120 | 5.68E-06 | 6384 |
|  | rs151093994 | 6 | 162733959 | A | G | -0.284 | 0.064 | 0.020 | 9.38E-06 | 6384 |
|  | rs141329532 | 7 | 144901272 | G | C | 0.711 | 0.160 | 0.003 | 9.39E-06 | 6384 |
|  | rs4726178 | 7 | 152575262 | T | C | -0.106 | 0.022 | 0.201 | 1.54E-06 | 6384 |
|  | rs3916027 | 8 | 19967357 | A | G | -0.108 | 0.020 | 0.253 | 1.32E-07 | 6384 |
|  | rs11776355 | 8 | 92462316 | T | C | -0.087 | 0.020 | 0.288 | 8.51E-06 | 6384 |
|  | rs112792028 | 9 | 19072292 | A | C | -0.093 | 0.019 | 0.322 | 1.34E-06 | 6384 |
|  | rs10521440 | 9 | 70920558 | T | C | -0.183 | 0.041 | 0.049 | 9.72E-06 | 6384 |
|  | rs143453636 | 9 | 126866252 | G | C | -0.435 | 0.098 | 0.009 | 8.52E-06 | 6384 |
|  | rs138578034 | 11 | 17897155 | T | G | -0.678 | 0.153 | 0.003 | 9.37E-06 | 6384 |
|  | rs174544 | 11 | 61800281 | A | C | -0.096 | 0.018 | 0.380 | 1.74E-07 | 6384 |
|  | rs2229738 | 11 | 68794860 | T | C | -0.119 | 0.025 | 0.156 | 1.92E-06 | 6384 |
|  | rs964184 | 11 | 116778201 | C | G | -0.171 | 0.024 | 0.849 | 2.38E-12 | 6384 |
|  | rs7336059 | 13 | 21648634 | G | A | -0.280 | 0.063 | 0.021 | 9.87E-06 | 6384 |
|  | rs61967369 | 13 | 73092472 | C | A | -0.173 | 0.036 | 0.064 | 1.55E-06 | 6384 |
|  | rs17765709 | 14 | 36553367 | T | C | -0.114 | 0.025 | 0.140 | 6.02E-06 | 6384 |
|  | rs261291 | 15 | 58387979 | C | T | 0.081 | 0.018 | 0.395 | 6.05E-06 | 6384 |
|  | rs182611493 | 19 | 19347579 | G | A | -0.244 | 0.041 | 0.054 | 2.40E-09 | 6384 |
|  | rs445925 | 19 | 44912383 | A | G | 0.159 | 0.033 | 0.077 | 1.36E-06 | 6384 |
|  | rs11880266 | 19 | 55460024 | G | A | -0.089 | 0.019 | 0.393 | 2.00E-06 | 6384 |
| Abbreviations: SNPs: single nucleotide polymorphisms; EA: effect allele; OA: other allele; EAF: effect allele frequency; SE: standard error. | | | | | | | | | | |

| **Supplementary Table S2. Summary information for SNPs that were used as genetic instruments for  Mendelian randomization analyses of circulating inflammatory proteins.** | | | | | | | | | | |
| --- | --- | --- | --- | --- | --- | --- | --- | --- | --- | --- |
| **Inflammatory proteins** | **SNPs** | **Chromosome** | **Location** | **EA** | **OA** | **Beta** | **SE** | **EAF** | **P** | **N** |
| 4EBP1 | rs356283 | 1 | 80975190 | T | G | 0.0588 | 0.0123 | 0.6475 | 1.75E-06 | 14730 |
|  | rs115454951 | 1 | 108736338 | A | C | -0.1537 | 0.0344 | 0.0384 | 7.89E-06 | 11982 |
|  | rs10926313 | 1 | 240804510 | A | G | -0.0884 | 0.0175 | 0.8334 | 4.39E-07 | 14288 |
|  | rs76686678 | 2 | 38198906 | T | G | 0.2607 | 0.0521 | 0.015 | 5.62E-07 | 14730 |
|  | rs139951113 | 3 | 6066466 | T | C | 0.3201 | 0.0722 | 0.0087 | 9.27E-06 | 12935 |
|  | rs10513018 | 3 | 136674468 | A | G | -0.0875 | 0.0197 | 0.8986 | 8.93E-06 | 14288 |
|  | rs5030072 | 3 | 186455546 | T | C | 0.0594 | 0.0119 | 0.5711 | 5.99E-07 | 14716 |
|  | rs112020430 | 4 | 117584424 | T | C | 0.1784 | 0.0403 | 0.0251 | 9.56E-06 | 14735 |
|  | rs3733402 | 4 | 187158034 | A | G | -0.1305 | 0.0116 | 0.5102 | 2.32E-29 | 14736 |
|  | rs2731672 | 5 | 176842474 | T | C | 0.0926 | 0.0152 | 0.2679 | 1.11E-09 | 11783 |
|  | rs1034059 | 6 | 158193698 | A | G | 0.066 | 0.0146 | 0.6982 | 6.17E-06 | 14288 |
|  | rs6963617 | 7 | 17594314 | T | C | 0.0754 | 0.0142 | 0.2938 | 1.10E-07 | 14288 |
|  | rs78948504 | 8 | 3936170 | C | G | -0.1269 | 0.0281 | 0.9352 | 6.30E-06 | 11781 |
|  | rs10792025 | 11 | 68921211 | T | C | -0.0593 | 0.0124 | 0.3383 | 1.73E-06 | 14727 |
|  | rs181095999 | 12 | 23923166 | T | C | -0.2226 | 0.0474 | 0.0227 | 2.65E-06 | 14736 |
|  | rs7490744 | 13 | 84976969 | T | G | 0.0705 | 0.0159 | 0.2217 | 9.25E-06 | 11783 |
|  | rs114302245 | 16 | 84586319 | T | C | 0.0791 | 0.0169 | 0.358 | 2.86E-06 | 10894 |
|  | rs2856226 | 17 | 15470414 | T | C | -0.0995 | 0.0214 | 0.8975 | 3.33E-06 | 12404 |
|  | rs60037565 | 17 | 74618280 | A | T | 0.0772 | 0.0172 | 0.8011 | 7.18E-06 | 11337 |
|  | rs137887001 | 20 | 44566861 | C | G | 0.2235 | 0.0486 | 0.9827 | 4.25E-06 | 14288 |
| ADA | rs115657851 | 1 | 57353848 | A | G | 0.139 | 0.0293 | 0.0462 | 2.10E-06 | 14715 |
|  | rs75492374 | 1 | 150902178 | A | G | 0.3552 | 0.077 | 0.9905 | 3.97E-06 | 11139 |
|  | rs184103539 | 1 | 222149663 | T | C | -0.1524 | 0.0339 | 0.0397 | 6.94E-06 | 12833 |
|  | rs6544381 | 2 | 41276504 | A | C | 0.0643 | 0.0143 | 0.7983 | 6.91E-06 | 14732 |
|  | rs111478357 | 2 | 144650434 | T | C | 0.2044 | 0.044 | 0.0243 | 3.39E-06 | 13801 |
|  | rs62188709 | 2 | 225565760 | A | T | -0.1012 | 0.0222 | 0.9174 | 5.15E-06 | 14288 |
|  | rs6441227 | 3 | 158534618 | A | C | -0.087 | 0.0194 | 0.1824 | 7.31E-06 | 10894 |
|  | rs10024178 | 4 | 5801734 | T | C | -0.0925 | 0.0199 | 0.905 | 3.35E-06 | 14716 |
|  | rs189104742 | 4 | 75665562 | A | T | 0.2754 | 0.0584 | 0.9857 | 2.41E-06 | 14729 |
|  | rs62319741 | 4 | 124571895 | T | C | 0.1281 | 0.0258 | 0.9456 | 6.87E-07 | 14733 |
|  | rs6463679 | 7 | 7345002 | A | G | 0.0552 | 0.0122 | 0.3628 | 6.05E-06 | 14288 |
|  | rs1608554 | 7 | 22807634 | A | G | 0.0598 | 0.0122 | 0.656 | 9.50E-07 | 14733 |
|  | rs2527856 | 7 | 80678923 | T | C | 0.0562 | 0.0122 | 0.5989 | 4.09E-06 | 14288 |
|  | rs73701916 | 8 | 102530192 | A | G | -0.1301 | 0.0292 | 0.9512 | 8.37E-06 | 14726 |
|  | rs4749761 | 10 | 8502517 | A | T | 0.5548 | 0.1204 | 0.0104 | 4.07E-06 | 6862 |
|  | rs11512688 | 11 | 102613285 | T | G | -0.0606 | 0.0133 | 0.6677 | 5.20E-06 | 12193 |
|  | rs2620728 | 12 | 65118323 | C | G | -0.0551 | 0.0119 | 0.5267 | 3.65E-06 | 14288 |
|  | rs79226097 | 14 | 91715447 | T | C | 0.1608 | 0.0357 | 0.03 | 6.66E-06 | 14732 |
|  | rs739998 | 16 | 316890 | T | C | 0.0594 | 0.0132 | 0.5782 | 6.80E-06 | 13662 |
|  | rs56139330 | 16 | 58197178 | A | G | 0.0777 | 0.0173 | 0.8369 | 7.08E-06 | 12197 |
|  | rs117645472 | 18 | 917376 | A | T | 0.3494 | 0.0754 | 0.0125 | 3.59E-06 | 11095 |
|  | rs73966257 | 18 | 70229342 | A | G | -0.2927 | 0.0654 | 0.0126 | 7.62E-06 | 12935 |
|  | rs151018349 | 19 | 561872 | A | T | 0.1025 | 0.0231 | 0.1817 | 9.11E-06 | 10474 |
|  | rs76418401 | 20 | 43233080 | A | C | -0.907 | 0.0214 | 0.9299 | 0.00E+00 | 14727 |
|  | rs2007720 | 20 | 43276688 | A | G | -0.1698 | 0.0136 | 0.7345 | 8.98E-36 | 14288 |
|  | rs73617409 | 20 | 43300800 | A | G | -0.1313 | 0.0164 | 0.2474 | 1.18E-15 | 12404 |
|  | rs147052185 | 21 | 19074306 | A | G | 0.2127 | 0.0473 | 0.9793 | 6.90E-06 | 14244 |
|  | rs7292320 | 22 | 37921425 | A | G | -0.0599 | 0.0131 | 0.2529 | 4.82E-06 | 14729 |
| ARTN | rs13303278 | 1 | 921716 | A | C | -0.0664 | 0.0144 | 0.5539 | 4.01E-06 | 10265 |
|  | rs3003450 | 1 | 17628383 | T | G | 0.0632 | 0.0137 | 0.6627 | 3.97E-06 | 11762 |
|  | rs10799609 | 1 | 20418323 | T | C | -0.1321 | 0.0293 | 0.0561 | 6.53E-06 | 11778 |
|  | rs79664530 | 1 | 197189391 | C | G | -0.3211 | 0.0718 | 0.0125 | 7.74E-06 | 10843 |
|  | rs76672219 | 1 | 225057589 | A | C | -0.3226 | 0.0682 | 0.9882 | 2.24E-06 | 11291 |
|  | rs141625055 | 2 | 142183922 | A | G | 0.1852 | 0.0418 | 0.0327 | 9.40E-06 | 11765 |
|  | rs9682166 | 3 | 93520803 | T | C | -0.1481 | 0.0334 | 0.0532 | 9.24E-06 | 9453 |
|  | rs62277657 | 3 | 111720183 | A | G | 0.0833 | 0.0166 | 0.1972 | 5.22E-07 | 11777 |
|  | rs17491469 | 3 | 189164574 | A | G | -0.0897 | 0.0197 | 0.1327 | 5.28E-06 | 11777 |
|  | rs138311590 | 4 | 4424044 | A | G | -0.0811 | 0.0176 | 0.84 | 4.07E-06 | 11758 |
|  | rs79259958 | 4 | 40380879 | T | C | -0.3099 | 0.0697 | 0.0147 | 8.74E-06 | 9945 |
|  | rs9684768 | 4 | 156930775 | C | G | 0.0692 | 0.014 | 0.626 | 7.70E-07 | 11330 |
|  | rs17340161 | 5 | 103466564 | A | G | -0.0873 | 0.0189 | 0.8494 | 3.86E-06 | 11330 |
|  | rs118087756 | 6 | 94484664 | A | G | -0.4237 | 0.094 | 0.0108 | 6.56E-06 | 8024 |
|  | rs9496445 | 6 | 143047898 | T | C | 0.0853 | 0.0181 | 0.1545 | 2.44E-06 | 11768 |
|  | rs12699544 | 7 | 13780997 | A | G | 0.0803 | 0.0181 | 0.1536 | 9.14E-06 | 11777 |
|  | rs142970662 | 7 | 106961661 | A | G | 0.311 | 0.0639 | 0.0191 | 1.13E-06 | 9834 |
|  | rs4993886 | 8 | 3686552 | A | T | -0.079 | 0.0162 | 0.6759 | 1.08E-06 | 8371 |
|  | rs55885546 | 8 | 19493177 | A | G | -0.2047 | 0.044 | 0.0245 | 3.28E-06 | 11778 |
|  | rs141429639 | 8 | 53903686 | A | G | -0.293 | 0.0644 | 0.0147 | 5.37E-06 | 11330 |
|  | rs74383279 | 8 | 112086697 | C | G | -0.1899 | 0.0421 | 0.9634 | 6.46E-06 | 11761 |
|  | rs1329733 | 9 | 92208227 | A | G | -0.0674 | 0.0136 | 0.5323 | 7.20E-07 | 11330 |
|  | rs4740350 | 9 | 133454106 | A | G | 0.0877 | 0.0194 | 0.1326 | 6.17E-06 | 11778 |
|  | rs142585779 | 9 | 140239588 | T | C | 0.2119 | 0.0435 | 0.038 | 1.11E-06 | 10266 |
|  | rs3001873 | 10 | 52449020 | T | C | -0.0852 | 0.0185 | 0.1749 | 4.12E-06 | 11330 |
|  | rs4561132 | 10 | 97678022 | A | G | -0.0716 | 0.0159 | 0.2386 | 6.70E-06 | 11329 |
|  | rs77502336 | 11 | 123394636 | C | G | 0.0679 | 0.0141 | 0.318 | 1.47E-06 | 11775 |
|  | rs118115625 | 12 | 81724190 | T | C | 0.1703 | 0.0362 | 0.0344 | 2.55E-06 | 11778 |
|  | rs148226005 | 12 | 130004146 | A | G | 0.2112 | 0.0451 | 0.9758 | 2.83E-06 | 11765 |
|  | rs114907684 | 16 | 81582982 | T | C | -0.3648 | 0.0818 | 0.9881 | 8.21E-06 | 8283 |
|  | rs1370215 | 18 | 54688939 | T | C | -0.07 | 0.0153 | 0.7417 | 4.76E-06 | 11330 |
|  | rs6509909 | 19 | 55422034 | C | G | -0.0881 | 0.0176 | 0.8122 | 5.57E-07 | 11330 |
|  | rs9941952 | 22 | 25646493 | T | C | 0.0765 | 0.0167 | 0.7151 | 4.63E-06 | 11774 |
| Axin-1 | rs2712427 | 3 | 128431622 | A | G | 0.0626 | 0.0134 | 0.4935 | 2.99E-06 | 11345 |
|  | rs115455827 | 5 | 78787853 | A | G | 0.1447 | 0.0305 | 0.9489 | 2.09E-06 | 11792 |
|  | rs117097664 | 6 | 151334530 | A | C | 0.2444 | 0.0522 | 0.0197 | 2.84E-06 | 11345 |
|  | rs2691530 | 7 | 77960243 | T | C | -0.067 | 0.0133 | 0.5969 | 4.71E-07 | 11793 |
|  | rs2470622 | 8 | 102489704 | A | G | -0.1229 | 0.0242 | 0.9127 | 3.80E-07 | 11345 |
|  | rs11202576 | 10 | 89584284 | A | G | -0.0767 | 0.0172 | 0.7946 | 8.22E-06 | 11345 |
|  | rs72984228 | 11 | 101331132 | A | G | -0.1557 | 0.035 | 0.9611 | 8.64E-06 | 11792 |
|  | rs113946880 | 11 | 134706712 | T | C | 0.1331 | 0.0293 | 0.0539 | 5.55E-06 | 11792 |
|  | rs10860902 | 12 | 103075047 | C | G | 0.0659 | 0.0149 | 0.7403 | 9.74E-06 | 11778 |
|  | rs7171268 | 15 | 81221771 | T | C | 0.1554 | 0.0329 | 0.9514 | 2.32E-06 | 11345 |
|  | rs148317708 | 18 | 50513818 | A | G | -0.212 | 0.042 | 0.0295 | 4.47E-07 | 10919 |
|  | rs9962377 | 18 | 70105531 | T | C | -0.0843 | 0.0186 | 0.1481 | 5.84E-06 | 11793 |
|  | rs428324 | 18 | 77253338 | T | C | 0.0692 | 0.0148 | 0.6047 | 2.93E-06 | 10281 |
| Beta-NGF | rs6328 | 1 | 115829943 | A | C | -0.0797 | 0.0122 | 0.3498 | 6.46E-11 | 13224 |
|  | rs72673086 | 4 | 96824143 | A | G | 0.0584 | 0.0126 | 0.3152 | 3.57E-06 | 12858 |
|  | rs78167655 | 4 | 119266088 | T | C | -0.1646 | 0.0365 | 0.0262 | 6.50E-06 | 14743 |
|  | rs4017 | 4 | 164064099 | T | C | -0.1187 | 0.0258 | 0.9491 | 4.21E-06 | 14742 |
|  | rs17689534 | 4 | 166807640 | A | G | 0.2208 | 0.0462 | 0.0229 | 1.76E-06 | 13421 |
|  | rs12188775 | 5 | 132971762 | T | G | 0.1213 | 0.0247 | 0.9435 | 9.06E-07 | 14737 |
|  | rs77493302 | 6 | 838092 | T | C | 0.0853 | 0.0185 | 0.8904 | 4.01E-06 | 14743 |
|  | rs2237172 | 6 | 16601587 | A | G | 0.0619 | 0.0136 | 0.7375 | 5.33E-06 | 14295 |
|  | rs2904780 | 6 | 31457874 | A | C | -0.0685 | 0.0143 | 0.3918 | 1.67E-06 | 11538 |
|  | rs397081 | 6 | 32192617 | T | C | 0.1203 | 0.0262 | 0.9514 | 4.40E-06 | 14743 |
|  | rs73782207 | 6 | 150452471 | A | G | 0.0925 | 0.0205 | 0.0825 | 6.42E-06 | 14734 |
|  | rs9479501 | 6 | 153400418 | T | G | 0.0629 | 0.0137 | 0.2135 | 4.41E-06 | 14729 |
|  | rs12533747 | 7 | 30944899 | A | G | 0.1389 | 0.0276 | 0.0802 | 4.84E-07 | 14295 |
|  | rs17647697 | 7 | 130302087 | A | C | -0.1617 | 0.0352 | 0.9727 | 4.35E-06 | 14741 |
|  | rs12539643 | 7 | 151019021 | C | G | 0.1867 | 0.0408 | 0.966 | 4.74E-06 | 11344 |
|  | rs62494116 | 8 | 13332042 | T | C | 0.0578 | 0.0117 | 0.6636 | 7.81E-07 | 14729 |
|  | rs1878359 | 8 | 122948812 | A | G | 0.0626 | 0.0126 | 0.719 | 6.76E-07 | 14740 |
|  | rs10961630 | 9 | 14636946 | C | G | 0.053 | 0.0118 | 0.55 | 7.07E-06 | 14295 |
|  | rs3128517 | 9 | 90362040 | T | C | -0.0775 | 0.0118 | 0.4965 | 5.11E-11 | 14295 |
|  | rs12259196 | 10 | 89839430 | A | G | -0.075 | 0.0148 | 0.1928 | 4.03E-07 | 13244 |
|  | rs4980184 | 10 | 125058869 | T | C | 0.2352 | 0.0521 | 0.9836 | 6.35E-06 | 14295 |
|  | rs183185212 | 11 | 9434918 | A | C | -0.2977 | 0.0657 | 0.9883 | 5.86E-06 | 13808 |
|  | rs11025264 | 11 | 19837758 | T | C | 0.1739 | 0.0384 | 0.9748 | 5.94E-06 | 14743 |
|  | rs9599159 | 13 | 67428046 | T | C | -0.175 | 0.0387 | 0.9756 | 6.13E-06 | 14730 |
|  | rs4899264 | 14 | 69285337 | C | G | -0.055 | 0.0117 | 0.5283 | 2.59E-06 | 14295 |
|  | rs7149605 | 14 | 94854041 | A | G | 0.0862 | 0.0195 | 0.0949 | 9.85E-06 | 14731 |
|  | rs140072192 | 16 | 3327943 | A | G | 0.2773 | 0.0602 | 0.0196 | 4.10E-06 | 11925 |
|  | rs140882208 | 17 | 47365286 | T | C | -0.1722 | 0.0388 | 0.0258 | 9.07E-06 | 14731 |
|  | rs4087847 | 18 | 2455418 | T | G | -0.1546 | 0.0343 | 0.0301 | 6.57E-06 | 14743 |
|  | rs74639669 | 18 | 5679360 | A | T | -0.1748 | 0.0386 | 0.0281 | 5.94E-06 | 14733 |
|  | rs62092633 | 18 | 55441626 | T | C | -0.1249 | 0.0259 | 0.1005 | 1.42E-06 | 11344 |
|  | rs34559191 | 19 | 16434937 | T | C | 0.0607 | 0.0124 | 0.3088 | 9.82E-07 | 13675 |
|  | rs117164089 | 19 | 43780396 | T | C | -0.271 | 0.0565 | 0.014 | 1.61E-06 | 13421 |
|  | rs148834134 | 21 | 43942626 | T | C | 0.2541 | 0.0556 | 0.0235 | 4.87E-06 | 13421 |
|  | rs13058191 | 22 | 49775973 | C | G | -0.2164 | 0.0467 | 0.0256 | 3.59E-06 | 14295 |
| CASP-8 | rs34154371 | 1 | 2338015 | T | C | -0.2016 | 0.0422 | 0.9714 | 1.78E-06 | 13232 |
|  | rs12073499 | 1 | 157270515 | A | C | -0.0812 | 0.0167 | 0.8466 | 1.16E-06 | 14295 |
|  | rs7418913 | 1 | 203643268 | A | G | -0.0614 | 0.0138 | 0.2714 | 8.62E-06 | 14296 |
|  | rs56328050 | 2 | 202164805 | C | G | -0.1987 | 0.0173 | 0.8696 | 1.56E-30 | 14743 |
|  | rs11695193 | 2 | 231090015 | A | T | -0.0806 | 0.0162 | 0.1582 | 6.51E-07 | 14743 |
|  | rs11709039 | 3 | 152332673 | T | C | 0.0781 | 0.0168 | 0.856 | 3.34E-06 | 14743 |
|  | rs10024203 | 4 | 5786594 | A | C | 0.0922 | 0.0203 | 0.094 | 5.58E-06 | 14735 |
|  | rs13188908 | 5 | 179923576 | T | C | 0.0668 | 0.015 | 0.1902 | 8.45E-06 | 14736 |
|  | rs148608719 | 5 | 180339648 | A | G | 0.3474 | 0.0683 | 0.0145 | 3.65E-07 | 12745 |
|  | rs1034059 | 6 | 158193698 | A | G | 0.0681 | 0.0145 | 0.6981 | 2.65E-06 | 14296 |
|  | rs187405204 | 7 | 61833768 | T | C | 0.4195 | 0.0912 | 0.9851 | 4.23E-06 | 6257 |
|  | rs111764025 | 8 | 5557775 | C | G | 0.1095 | 0.0238 | 0.9304 | 4.21E-06 | 14731 |
|  | rs78449258 | 9 | 22742536 | A | G | -0.2001 | 0.0428 | 0.9804 | 2.94E-06 | 14744 |
|  | rs73306603 | 10 | 84027526 | T | C | 0.2044 | 0.0412 | 0.978 | 7.01E-07 | 14741 |
|  | rs1467702 | 10 | 129961901 | A | G | -0.0962 | 0.0215 | 0.0863 | 7.66E-06 | 14743 |
|  | rs140844269 | 11 | 63817395 | C | G | -0.3615 | 0.0776 | 0.0099 | 3.19E-06 | 12330 |
|  | rs11216018 | 11 | 98455719 | A | G | -0.0539 | 0.0121 | 0.3708 | 8.41E-06 | 14296 |
|  | rs1032417 | 12 | 62645486 | C | G | -0.0858 | 0.0165 | 0.1545 | 1.99E-07 | 14296 |
|  | rs56097553 | 12 | 82467039 | A | G | 0.1384 | 0.0312 | 0.0405 | 9.17E-06 | 14744 |
|  | rs7146962 | 14 | 54145317 | T | C | -0.0613 | 0.0135 | 0.7483 | 5.61E-06 | 14295 |
|  | rs2411067 | 17 | 75033876 | A | G | -0.0706 | 0.0157 | 0.685 | 6.90E-06 | 11345 |
|  | rs137887001 | 20 | 44566861 | C | G | 0.2318 | 0.0486 | 0.9827 | 1.85E-06 | 14296 |
|  | rs7287303 | 22 | 48714112 | T | C | 0.0985 | 0.0222 | 0.1249 | 9.12E-06 | 11791 |
| CCL11 | rs12075 | 1 | 159175354 | A | G | 0.0749 | 0.0114 | 0.5547 | 5.03E-11 | 14731 |
|  | rs10911109 | 1 | 172117232 | A | G | -0.0767 | 0.0172 | 0.1696 | 8.22E-06 | 11774 |
|  | rs2228467 | 3 | 42906116 | T | C | -0.3316 | 0.0225 | 0.9289 | 3.69E-49 | 14733 |
|  | rs79684468 | 3 | 43179320 | T | C | 0.1502 | 0.0303 | 0.0433 | 7.16E-07 | 14286 |
|  | rs2201150 | 3 | 46258902 | T | C | 0.0785 | 0.0123 | 0.3561 | 1.75E-10 | 14286 |
|  | rs148554043 | 3 | 175964549 | T | C | 0.4043 | 0.0898 | 0.0083 | 6.72E-06 | 11726 |
|  | rs139597204 | 4 | 104507060 | A | C | -0.3374 | 0.0714 | 0.0128 | 2.30E-06 | 12303 |
|  | rs6894241 | 5 | 118058126 | A | C | -0.0656 | 0.0137 | 0.2333 | 1.68E-06 | 14715 |
|  | rs151240414 | 5 | 130672061 | A | G | 0.2299 | 0.0508 | 0.0222 | 6.02E-06 | 11335 |
|  | rs757973 | 7 | 75495667 | A | G | 0.2002 | 0.0205 | 0.09 | 1.58E-22 | 14713 |
|  | rs73209015 | 8 | 11700967 | A | T | -0.0913 | 0.0189 | 0.1104 | 1.36E-06 | 14728 |
|  | rs140965534 | 8 | 49300287 | T | C | -0.2283 | 0.0514 | 0.0172 | 8.93E-06 | 14238 |
|  | rs79432525 | 10 | 128183195 | T | G | 0.1929 | 0.0422 | 0.9754 | 4.85E-06 | 14247 |
|  | rs1564995 | 11 | 24773107 | A | G | -0.0609 | 0.0131 | 0.6155 | 3.34E-06 | 14286 |
|  | rs34536806 | 11 | 95274487 | T | G | 0.1017 | 0.0208 | 0.9023 | 1.01E-06 | 14286 |
|  | rs35543857 | 12 | 105757994 | A | G | -0.1138 | 0.0244 | 0.9282 | 3.10E-06 | 14286 |
|  | rs114744647 | 16 | 24006879 | T | C | 0.2107 | 0.0451 | 0.9774 | 2.99E-06 | 14730 |
|  | rs9940390 | 16 | 80051931 | T | C | -0.0818 | 0.0133 | 0.7358 | 7.73E-10 | 14286 |
|  | rs8068185 | 17 | 12248581 | T | C | 0.0732 | 0.016 | 0.8434 | 4.76E-06 | 14727 |
|  | rs79722574 | 17 | 32619052 | T | C | -0.1215 | 0.0156 | 0.167 | 6.78E-15 | 14720 |
|  | rs9303380 | 17 | 54117492 | A | G | 0.1213 | 0.0272 | 0.0494 | 8.21E-06 | 14286 |
|  | rs12453306 | 17 | 77253324 | C | G | -0.1089 | 0.0244 | 0.9022 | 8.08E-06 | 11335 |
|  | rs55843637 | 18 | 1508223 | C | G | 0.0555 | 0.0121 | 0.5296 | 4.50E-06 | 14286 |
|  | rs170520 | 18 | 38337850 | A | G | 0.0554 | 0.0125 | 0.3264 | 9.34E-06 | 14285 |
|  | rs2112920 | 19 | 37955248 | C | G | 0.0817 | 0.016 | 0.8362 | 3.29E-07 | 14286 |
|  | rs139476527 | 20 | 49033630 | T | G | 0.2826 | 0.0606 | 0.0125 | 3.11E-06 | 12933 |
|  | rs9976282 | 21 | 39593226 | A | G | -0.1678 | 0.0345 | 0.0333 | 1.15E-06 | 14722 |
|  | rs73171545 | 22 | 45003109 | A | G | -0.0861 | 0.0187 | 0.1199 | 4.14E-06 | 14286 |
| CCL19 | rs7595241 | 2 | 7425475 | C | G | 0.0615 | 0.013 | 0.6941 | 2.24E-06 | 14288 |
|  | rs113139367 | 2 | 66609925 | A | C | -0.2101 | 0.043 | 0.021 | 1.03E-06 | 14242 |
|  | rs13010492 | 2 | 204776176 | C | G | -0.0803 | 0.0123 | 0.4754 | 6.65E-11 | 13422 |
|  | rs192686691 | 3 | 12700866 | C | G | 0.2648 | 0.0589 | 0.0195 | 6.93E-06 | 10795 |
|  | rs113539352 | 3 | 58516854 | C | G | 0.4485 | 0.0922 | 0.992 | 1.15E-06 | 11439 |
|  | rs1654018 | 3 | 71575288 | T | C | 0.2391 | 0.0538 | 0.9738 | 8.82E-06 | 10894 |
|  | rs62292952 | 3 | 132200719 | T | G | 0.2346 | 0.0185 | 0.8822 | 7.53E-37 | 14728 |
|  | rs4356912 | 4 | 7444846 | T | C | -0.0592 | 0.0133 | 0.6909 | 8.54E-06 | 14288 |
|  | rs6535499 | 4 | 76428761 | T | C | -0.1216 | 0.0272 | 0.0583 | 7.80E-06 | 14288 |
|  | rs374784992 | 4 | 119585039 | A | G | 0.1335 | 0.0298 | 0.9401 | 7.47E-06 | 11760 |
|  | rs79086127 | 4 | 123839263 | T | C | -0.2096 | 0.045 | 0.0195 | 3.20E-06 | 14247 |
|  | rs4554017 | 4 | 145124024 | T | C | 0.0596 | 0.0122 | 0.3904 | 1.03E-06 | 14288 |
|  | rs115109204 | 5 | 23807519 | T | C | 0.3784 | 0.0842 | 0.0116 | 6.99E-06 | 10795 |
|  | rs12521515 | 5 | 123121314 | A | T | 0.086 | 0.0192 | 0.861 | 7.49E-06 | 11780 |
|  | rs3792790 | 5 | 150442171 | A | C | 0.0551 | 0.012 | 0.4736 | 4.40E-06 | 14288 |
|  | rs6870560 | 5 | 174187919 | T | C | 0.0863 | 0.0169 | 0.8559 | 3.28E-07 | 14732 |
|  | rs1328133 | 6 | 9710127 | A | T | 0.3112 | 0.0695 | 0.01 | 7.55E-06 | 13422 |
|  | rs9469127 | 6 | 32444093 | C | G | -0.4884 | 0.0277 | 0.9365 | 1.41E-69 | 14735 |
|  | rs28635588 | 6 | 32519041 | A | G | -0.1775 | 0.0206 | 0.8408 | 6.90E-18 | 9182 |
|  | rs11961362 | 6 | 81004579 | T | C | -0.1185 | 0.0264 | 0.0713 | 7.17E-06 | 12851 |
|  | rs1415763 | 6 | 125027776 | C | G | 0.0757 | 0.0157 | 0.1687 | 1.42E-06 | 14731 |
|  | rs13212719 | 6 | 133835436 | A | G | 0.0671 | 0.015 | 0.8005 | 7.70E-06 | 14288 |
|  | rs10242459 | 7 | 3911280 | A | G | 0.0729 | 0.0154 | 0.8219 | 2.20E-06 | 14729 |
|  | rs73257451 | 8 | 61418130 | T | C | -0.3645 | 0.0756 | 0.989 | 1.43E-06 | 11337 |
|  | rs454265 | 8 | 81339142 | A | G | -0.0561 | 0.0124 | 0.6505 | 6.06E-06 | 14728 |
|  | rs11574915 | 9 | 34710084 | A | C | -0.1464 | 0.017 | 0.8639 | 7.19E-18 | 14733 |
|  | rs34791102 | 9 | 35612978 | A | G | 0.1238 | 0.0265 | 0.0534 | 2.99E-06 | 14735 |
|  | rs7894965 | 10 | 93652211 | A | G | -0.0644 | 0.0142 | 0.6255 | 5.75E-06 | 11538 |
|  | rs6486995 | 12 | 19813981 | A | T | -0.0697 | 0.0135 | 0.2452 | 2.43E-07 | 14735 |
|  | rs11174279 | 12 | 62401393 | A | T | -0.0694 | 0.0156 | 0.1741 | 8.64E-06 | 14730 |
|  | rs4766578 | 12 | 111904371 | A | T | -0.0801 | 0.0132 | 0.5095 | 1.29E-09 | 11785 |
|  | rs76295077 | 13 | 23257952 | T | C | 0.0928 | 0.0208 | 0.8962 | 8.14E-06 | 14288 |
|  | rs4907572 | 13 | 113647321 | A | G | -0.0671 | 0.0137 | 0.2653 | 9.69E-07 | 14722 |
|  | rs111317412 | 14 | 48619204 | A | C | -0.1236 | 0.0274 | 0.9429 | 6.45E-06 | 14288 |
|  | rs138108512 | 14 | 76855291 | A | G | -0.2939 | 0.0616 | 0.0119 | 1.83E-06 | 13422 |
|  | rs138618048 | 14 | 100142541 | A | G | -0.2066 | 0.046 | 0.0209 | 7.08E-06 | 14247 |
|  | rs74764632 | 16 | 57300587 | A | T | 0.1349 | 0.0294 | 0.9557 | 4.47E-06 | 14734 |
|  | rs7185085 | 16 | 82146285 | T | C | -0.3558 | 0.0803 | 0.9931 | 9.38E-06 | 12291 |
|  | rs147708384 | 17 | 14918425 | T | C | -0.2707 | 0.0574 | 0.0151 | 2.40E-06 | 13801 |
|  | rs11877886 | 18 | 69226577 | A | G | -0.1065 | 0.0239 | 0.0669 | 8.35E-06 | 14722 |
|  | rs2070746 | 19 | 52249702 | T | G | -0.0575 | 0.0128 | 0.307 | 7.05E-06 | 14288 |
|  | rs138440733 | 22 | 17731243 | A | G | -0.1724 | 0.036 | 0.0585 | 1.68E-06 | 12404 |
| CCL20 | rs11582772 | 1 | 32260562 | T | C | 0.2585 | 0.0548 | 0.9859 | 2.39E-06 | 14243 |
|  | rs111674022 | 1 | 238606022 | T | C | -0.0953 | 0.0208 | 0.0967 | 4.61E-06 | 14288 |
|  | rs10207134 | 2 | 228661828 | T | C | -0.0996 | 0.0128 | 0.326 | 7.18E-15 | 14287 |
|  | rs35757792 | 3 | 45157203 | A | T | -0.053 | 0.0119 | 0.4995 | 8.44E-06 | 14720 |
|  | rs55891514 | 5 | 40408939 | T | C | 0.0955 | 0.0213 | 0.9122 | 7.34E-06 | 14735 |
|  | rs13185268 | 5 | 133019542 | T | C | 0.1091 | 0.0239 | 0.9296 | 5.00E-06 | 14724 |
|  | rs11242693 | 6 | 1448190 | T | C | 0.0637 | 0.0135 | 0.3674 | 2.38E-06 | 12404 |
|  | rs9274623 | 6 | 32635998 | T | G | -0.076 | 0.0148 | 0.215 | 2.82E-07 | 14288 |
|  | rs742493 | 6 | 40998167 | T | C | 0.1459 | 0.0185 | 0.883 | 3.11E-15 | 14735 |
|  | rs9357723 | 6 | 51953097 | A | G | 0.0522 | 0.0116 | 0.4744 | 6.80E-06 | 14735 |
|  | rs7749768 | 6 | 107477601 | T | G | -0.0622 | 0.0138 | 0.754 | 6.57E-06 | 14709 |
|  | rs34729167 | 6 | 162943133 | T | C | 0.0801 | 0.0179 | 0.136 | 7.65E-06 | 14288 |
|  | rs3798304 | 6 | 167363434 | C | G | -0.0624 | 0.0138 | 0.7473 | 6.13E-06 | 14287 |
|  | rs62456601 | 7 | 55791153 | T | C | 0.0611 | 0.0137 | 0.6697 | 8.20E-06 | 12836 |
|  | rs1129474 | 8 | 22584718 | T | C | 0.0573 | 0.012 | 0.5396 | 1.80E-06 | 14288 |
|  | rs2796032 | 9 | 115519904 | T | C | 0.0636 | 0.0127 | 0.3159 | 5.50E-07 | 14718 |
|  | rs748503 | 10 | 73509465 | T | C | -0.0722 | 0.0162 | 0.1993 | 8.32E-06 | 14288 |
|  | rs41302923 | 10 | 127693479 | A | G | 0.1939 | 0.0414 | 0.9774 | 2.82E-06 | 14726 |
|  | rs10837780 | 11 | 42142787 | T | C | 0.1191 | 0.026 | 0.9413 | 4.63E-06 | 14288 |
|  | rs73072893 | 12 | 19657540 | A | G | 0.2057 | 0.0422 | 0.9739 | 1.09E-06 | 13230 |
|  | rs4981022 | 12 | 104149874 | A | G | 0.0635 | 0.0128 | 0.6933 | 7.02E-07 | 14721 |
|  | rs61945278 | 13 | 24524337 | T | C | -0.2 | 0.0451 | 0.028 | 9.22E-06 | 13386 |
|  | rs118164045 | 14 | 92792639 | T | C | 0.1297 | 0.0289 | 0.9526 | 7.19E-06 | 14288 |
|  | rs151092565 | 14 | 94625543 | T | C | -0.1512 | 0.0328 | 0.0376 | 4.03E-06 | 14728 |
|  | rs2003431 | 14 | 106782532 | A | T | -0.1602 | 0.0344 | 0.0632 | 3.21E-06 | 9786 |
|  | rs1027232 | 15 | 40077948 | A | C | 0.1072 | 0.0238 | 0.0762 | 6.66E-06 | 14730 |
|  | rs6498925 | 16 | 63795821 | A | T | -0.0568 | 0.0125 | 0.6585 | 5.52E-06 | 14729 |
|  | rs117753190 | 17 | 67136325 | C | G | -0.185 | 0.0359 | 0.9688 | 2.56E-07 | 14243 |
|  | rs117900576 | 17 | 72761195 | C | G | -0.2256 | 0.0429 | 0.9694 | 1.45E-07 | 13224 |
|  | rs6420556 | 18 | 56487606 | T | C | -0.0736 | 0.0158 | 0.1755 | 3.19E-06 | 14288 |
|  | rs348389 | 19 | 6535637 | T | C | -0.055 | 0.0124 | 0.3574 | 9.19E-06 | 14288 |
|  | rs35833676 | 21 | 32961256 | T | C | 0.0804 | 0.0166 | 0.1452 | 1.28E-06 | 14732 |
|  | rs13049624 | 21 | 44005002 | A | G | -0.1315 | 0.0276 | 0.9476 | 1.89E-06 | 14288 |
|  | rs5769672 | 22 | 49877754 | T | G | -0.1184 | 0.0257 | 0.9406 | 4.09E-06 | 14732 |
| CCL23 | rs74127049 | 1 | 161582200 | C | G | 0.0603 | 0.0132 | 0.608 | 4.92E-06 | 13422 |
|  | rs146074782 | 1 | 203441260 | A | G | -0.3061 | 0.0626 | 0.0116 | 1.01E-06 | 12935 |
|  | rs55755820 | 1 | 245606006 | T | G | -0.1035 | 0.0228 | 0.899 | 5.64E-06 | 11538 |
|  | rs1001495 | 2 | 241647342 | T | C | 0.0983 | 0.0215 | 0.9113 | 4.83E-06 | 13650 |
|  | rs143910182 | 3 | 6127208 | T | C | 0.2157 | 0.0449 | 0.0255 | 1.56E-06 | 14730 |
|  | rs116683909 | 3 | 14445911 | A | G | -0.21 | 0.0426 | 0.0278 | 8.24E-07 | 14724 |
|  | rs74491711 | 3 | 40054358 | A | G | 0.1273 | 0.0278 | 0.048 | 4.67E-06 | 14735 |
|  | rs4317138 | 3 | 46462144 | T | C | 0.0585 | 0.0127 | 0.6736 | 4.10E-06 | 14288 |
|  | rs2870238 | 4 | 77373079 | T | C | 0.0574 | 0.0114 | 0.4869 | 4.78E-07 | 14736 |
|  | rs2263318 | 6 | 31432006 | A | G | -0.1027 | 0.0232 | 0.0765 | 9.57E-06 | 14735 |
|  | rs12204524 | 6 | 86144743 | C | G | 0.1164 | 0.0237 | 0.9291 | 9.04E-07 | 14727 |
|  | rs4719850 | 7 | 3238978 | T | C | -0.0548 | 0.0118 | 0.5907 | 3.42E-06 | 14726 |
|  | rs73071153 | 7 | 17545964 | A | G | -0.1497 | 0.0319 | 0.0393 | 2.70E-06 | 14728 |
|  | rs11285223 | 7 | 34711511 | T | C | -0.0671 | 0.0149 | 0.6941 | 6.69E-06 | 10894 |
|  | rs80014265 | 9 | 94180109 | T | C | 0.1307 | 0.0289 | 0.0514 | 6.11E-06 | 14736 |
|  | rs2905095 | 9 | 135902793 | T | C | 0.0664 | 0.0148 | 0.3078 | 7.24E-06 | 11337 |
|  | rs1926742 | 10 | 18295482 | A | G | 0.0574 | 0.0125 | 0.3093 | 4.39E-06 | 14725 |
|  | rs77354000 | 10 | 34433796 | A | G | -0.2064 | 0.0437 | 0.9788 | 2.32E-06 | 14735 |
|  | rs17092563 | 10 | 116653091 | C | G | -0.202 | 0.0445 | 0.9774 | 5.64E-06 | 14733 |
|  | rs11017721 | 10 | 132905425 | A | G | 0.0971 | 0.0186 | 0.1154 | 1.79E-07 | 14288 |
|  | rs112492 | 11 | 1989274 | A | G | -0.0692 | 0.014 | 0.7293 | 7.70E-07 | 13224 |
|  | rs17735284 | 11 | 84372512 | A | T | 0.0927 | 0.0202 | 0.0922 | 4.45E-06 | 14725 |
|  | rs77083410 | 11 | 115081786 | C | G | 0.224 | 0.0416 | 0.0223 | 7.26E-08 | 14734 |
|  | rs79960240 | 14 | 94811056 | T | G | -0.2384 | 0.0537 | 0.0143 | 9.02E-06 | 14249 |
|  | rs6497967 | 16 | 26890471 | T | C | -0.0547 | 0.0123 | 0.3198 | 8.70E-06 | 14729 |
|  | rs12949647 | 17 | 21386459 | A | G | -0.0643 | 0.0137 | 0.7287 | 2.69E-06 | 14288 |
|  | rs72625914 | 17 | 33992028 | T | C | -0.2589 | 0.0342 | 0.035 | 3.73E-14 | 14726 |
|  | rs117639761 | 17 | 34195180 | A | C | -0.3246 | 0.0413 | 0.9561 | 3.85E-15 | 14736 |
|  | rs143566774 | 17 | 34276072 | A | G | 0.1738 | 0.0372 | 0.0369 | 2.98E-06 | 14288 |
|  | rs712048 | 17 | 34326215 | A | C | -0.5147 | 0.0158 | 0.1497 | 8.99E-233 | 14723 |
|  | rs12103669 | 17 | 47298101 | A | G | -0.1778 | 0.0402 | 0.0234 | 9.74E-06 | 14734 |
|  | rs376974828 | 19 | 11626019 | T | C | -0.1724 | 0.039 | 0.0385 | 9.85E-06 | 10696 |
|  | rs438811 | 19 | 45416741 | T | C | 0.0669 | 0.0138 | 0.2405 | 1.25E-06 | 14720 |
|  | rs113959305 | 22 | 51076281 | T | C | -0.1397 | 0.0285 | 0.0559 | 9.50E-07 | 13224 |
| CCL25 | rs11589432 | 1 | 23748852 | C | G | 0.0566 | 0.0119 | 0.4781 | 1.97E-06 | 14287 |
|  | rs143132666 | 1 | 160762078 | A | C | -0.1934 | 0.0406 | 0.0225 | 1.90E-06 | 14734 |
|  | rs142015555 | 1 | 243141624 | A | T | -0.2379 | 0.0524 | 0.0212 | 5.62E-06 | 12935 |
|  | rs6744934 | 2 | 115179913 | T | C | -0.0544 | 0.0122 | 0.6208 | 8.23E-06 | 14288 |
|  | rs149395110 | 2 | 130959219 | T | C | -0.2782 | 0.0609 | 0.0129 | 4.92E-06 | 12880 |
|  | rs9825982 | 3 | 132612790 | A | G | 0.059 | 0.0133 | 0.356 | 9.16E-06 | 12196 |
|  | rs7611785 | 3 | 144213414 | A | G | 0.0941 | 0.0198 | 0.103 | 2.01E-06 | 14736 |
|  | rs851850 | 6 | 67085077 | A | G | -0.0777 | 0.0163 | 0.846 | 1.87E-06 | 14288 |
|  | rs7806274 | 7 | 155146782 | T | C | -0.0755 | 0.0161 | 0.1668 | 2.74E-06 | 13667 |
|  | rs111437511 | 8 | 10694001 | T | C | 0.1437 | 0.0297 | 0.0428 | 1.31E-06 | 14733 |
|  | rs1705699 | 8 | 23781453 | T | C | -0.0546 | 0.0116 | 0.5447 | 2.52E-06 | 14714 |
|  | rs12114935 | 8 | 42100809 | A | G | 0.0769 | 0.0142 | 0.7856 | 6.11E-08 | 14732 |
|  | rs141247883 | 8 | 144986071 | A | G | -0.5803 | 0.13 | 0.9919 | 8.05E-06 | 6780 |
|  | rs11140450 | 9 | 86838024 | A | T | -0.2681 | 0.0579 | 0.9693 | 3.65E-06 | 10797 |
|  | rs116534774 | 9 | 125200862 | T | C | 0.3717 | 0.0777 | 0.9916 | 1.72E-06 | 11139 |
|  | rs635634 | 9 | 136155000 | T | C | -0.2482 | 0.0166 | 0.1875 | 1.52E-50 | 11785 |
|  | rs79990760 | 10 | 29446793 | A | G | 0.2192 | 0.0478 | 0.9825 | 4.52E-06 | 14725 |
|  | rs11028036 | 11 | 24600146 | A | G | 0.0546 | 0.0117 | 0.5707 | 3.06E-06 | 14728 |
|  | rs200184122 | 11 | 50675038 | T | C | -0.0822 | 0.018 | 0.6982 | 4.96E-06 | 9830 |
|  | rs10792444 | 11 | 64434151 | T | C | -0.0865 | 0.0181 | 0.8838 | 1.76E-06 | 14732 |
|  | rs636479 | 11 | 75419643 | A | G | 0.0548 | 0.0119 | 0.3757 | 4.12E-06 | 14731 |
|  | rs7296588 | 12 | 578100 | A | G | -0.1159 | 0.0116 | 0.4365 | 1.66E-23 | 14725 |
|  | rs7137942 | 12 | 81177765 | A | G | -0.0637 | 0.0133 | 0.6502 | 1.67E-06 | 12404 |
|  | rs34520165 | 12 | 119526244 | T | C | 0.055 | 0.0122 | 0.4114 | 6.54E-06 | 14727 |
|  | rs521795 | 13 | 22284926 | T | C | -0.0624 | 0.0122 | 0.3844 | 3.14E-07 | 14288 |
|  | rs9530655 | 13 | 78006196 | A | G | 0.1539 | 0.0343 | 0.0337 | 7.23E-06 | 14724 |
|  | rs146498677 | 13 | 94811031 | C | G | 0.2285 | 0.0507 | 0.0168 | 6.58E-06 | 13422 |
|  | rs147851492 | 15 | 56638156 | A | G | 0.2127 | 0.0449 | 0.0215 | 2.17E-06 | 14727 |
|  | rs28494158 | 15 | 82219411 | T | C | -0.0733 | 0.0161 | 0.8338 | 5.29E-06 | 13867 |
|  | rs12606983 | 18 | 2511738 | T | C | -0.0673 | 0.0134 | 0.2475 | 5.10E-07 | 14715 |
|  | rs7252764 | 19 | 7827137 | T | C | 0.2321 | 0.0295 | 0.0471 | 3.61E-15 | 14736 |
|  | rs149462828 | 19 | 7901618 | A | G | 0.3017 | 0.0521 | 0.0169 | 7.01E-09 | 14288 |
|  | rs78865157 | 19 | 8107212 | A | G | -0.392 | 0.0448 | 0.0207 | 2.13E-18 | 14726 |
|  | rs3136647 | 19 | 8115457 | A | G | 0.7681 | 0.0147 | 0.1703 | 0.00E+00 | 14288 |
|  | rs61345716 | 19 | 8259301 | T | C | -0.1048 | 0.0212 | 0.1035 | 7.68E-07 | 14288 |
|  | rs516316 | 19 | 49206145 | C | G | -0.3279 | 0.0114 | 0.4445 | 6.20E-182 | 14734 |
|  | rs2317999 | 20 | 1689636 | A | G | -0.0577 | 0.013 | 0.4566 | 9.06E-06 | 11760 |
| CCL28 | rs1514964 | 1 | 64836646 | T | C | -0.1263 | 0.0264 | 0.9486 | 1.72E-06 | 14734 |
|  | rs17098842 | 1 | 76835048 | A | G | 0.1458 | 0.0321 | 0.9674 | 5.57E-06 | 14732 |
|  | rs6671688 | 1 | 154994265 | T | C | 0.0534 | 0.0116 | 0.5411 | 4.16E-06 | 14286 |
|  | rs291084 | 1 | 207095623 | C | G | 0.0638 | 0.0111 | 0.5279 | 9.04E-09 | 14726 |
|  | rs76291845 | 2 | 111889358 | A | G | 0.2696 | 0.0542 | 0.9859 | 6.55E-07 | 14244 |
|  | rs112641086 | 2 | 207076122 | T | C | -0.1056 | 0.0198 | 0.0895 | 9.64E-08 | 14731 |
|  | rs74855027 | 2 | 225265371 | T | C | 0.1215 | 0.0271 | 0.0639 | 7.35E-06 | 14286 |
|  | rs79205279 | 3 | 124901642 | A | G | 0.1063 | 0.024 | 0.0645 | 9.46E-06 | 13854 |
|  | rs2866626 | 4 | 102569209 | C | G | -0.0597 | 0.0121 | 0.6079 | 8.06E-07 | 14285 |
|  | rs76679915 | 4 | 125828318 | T | C | 0.0624 | 0.0137 | 0.7035 | 5.24E-06 | 11962 |
|  | rs374794081 | 4 | 138284908 | A | C | 0.1182 | 0.0263 | 0.9197 | 6.98E-06 | 11339 |
|  | rs7661151 | 4 | 157065076 | T | C | 0.0531 | 0.0116 | 0.6323 | 4.70E-06 | 14730 |
|  | rs4866742 | 5 | 43405459 | T | C | 0.0702 | 0.0122 | 0.4126 | 8.71E-09 | 12829 |
|  | rs116782490 | 5 | 84924989 | A | C | 0.2298 | 0.0488 | 0.9815 | 2.49E-06 | 12633 |
|  | rs78843608 | 5 | 102824347 | A | G | -0.1684 | 0.0323 | 0.0376 | 1.85E-07 | 14725 |
|  | rs2631360 | 5 | 131707429 | A | G | 0.0597 | 0.0128 | 0.5481 | 3.10E-06 | 11780 |
|  | rs141430371 | 6 | 29556379 | A | G | -0.4948 | 0.112 | 0.9936 | 9.97E-06 | 6941 |
|  | rs11754249 | 6 | 145107700 | A | G | 0.0934 | 0.0205 | 0.1058 | 5.21E-06 | 11779 |
|  | rs118111688 | 7 | 12554637 | A | T | 0.1874 | 0.0424 | 0.9776 | 9.88E-06 | 13374 |
|  | rs801520 | 7 | 18702222 | A | C | 0.0779 | 0.0171 | 0.1364 | 5.22E-06 | 14732 |
|  | rs3823797 | 7 | 77694740 | A | G | -0.058 | 0.0128 | 0.7423 | 5.86E-06 | 14732 |
|  | rs143400203 | 7 | 115560778 | T | C | 0.1812 | 0.0392 | 0.0244 | 3.79E-06 | 14722 |
|  | rs76669782 | 9 | 90419900 | A | C | -0.2853 | 0.0513 | 0.0176 | 2.68E-08 | 12933 |
|  | rs2519093 | 9 | 136141870 | T | C | -0.078 | 0.0162 | 0.1883 | 1.47E-06 | 11783 |
|  | rs2477954 | 10 | 90264140 | T | C | -0.058 | 0.012 | 0.6211 | 1.34E-06 | 14286 |
|  | rs78552613 | 10 | 105492625 | T | C | -0.1844 | 0.0395 | 0.0271 | 3.04E-06 | 14734 |
|  | rs371663005 | 11 | 25452442 | A | G | -0.0959 | 0.0208 | 0.8571 | 4.02E-06 | 10894 |
|  | rs73000929 | 11 | 113953622 | A | G | -0.1935 | 0.0355 | 0.0347 | 5.02E-08 | 14734 |
|  | rs6580980 | 12 | 54692061 | A | G | -0.0688 | 0.0118 | 0.4015 | 5.53E-09 | 14285 |
|  | rs80328354 | 12 | 55670876 | A | T | -0.2841 | 0.0599 | 0.9884 | 2.11E-06 | 14734 |
|  | rs7958187 | 12 | 69466941 | A | G | -0.055 | 0.0122 | 0.3872 | 6.54E-06 | 13420 |
|  | rs140404587 | 12 | 106453419 | T | G | -0.2617 | 0.0587 | 0.0119 | 8.26E-06 | 14241 |
|  | rs77569365 | 13 | 106647340 | T | G | 0.129 | 0.0282 | 0.9561 | 4.77E-06 | 14734 |
|  | rs12589600 | 14 | 86936828 | A | G | 0.051 | 0.0114 | 0.5556 | 7.69E-06 | 14711 |
|  | rs28384995 | 15 | 91295829 | A | T | -0.0866 | 0.0194 | 0.8668 | 8.05E-06 | 14286 |
|  | rs11078696 | 17 | 7459299 | T | G | -0.0855 | 0.017 | 0.7824 | 4.92E-07 | 10894 |
|  | rs4346241 | 17 | 8580609 | A | G | 0.0907 | 0.0204 | 0.9021 | 8.75E-06 | 14286 |
|  | rs55843637 | 18 | 1508223 | C | G | 0.0549 | 0.0119 | 0.5287 | 3.96E-06 | 14286 |
|  | rs71363004 | 18 | 74179338 | C | G | 0.2181 | 0.0466 | 0.9802 | 2.87E-06 | 11982 |
|  | rs150193318 | 19 | 31385455 | A | G | 0.2249 | 0.0501 | 0.0212 | 7.16E-06 | 14286 |
|  | rs113582651 | 19 | 55238516 | T | G | 0.242 | 0.0523 | 0.0489 | 3.71E-06 | 8911 |
|  | rs3064568 | 21 | 22059297 | C | G | -0.0585 | 0.0131 | 0.5068 | 7.98E-06 | 11538 |
|  | rs139902 | 22 | 38406782 | A | C | 0.0684 | 0.0121 | 0.3659 | 1.58E-08 | 14285 |
| CCL4 | rs148518028 | 2 | 79469060 | T | C | 0.1989 | 0.044 | 0.0317 | 6.17E-06 | 14296 |
|  | rs143922263 | 2 | 162240222 | A | G | 0.2198 | 0.0497 | 0.0203 | 9.75E-06 | 14296 |
|  | rs116193969 | 2 | 236974412 | A | T | 0.2915 | 0.0617 | 0.0134 | 2.31E-06 | 14296 |
|  | rs116309267 | 3 | 756117 | C | G | 0.1956 | 0.0405 | 0.0299 | 1.37E-06 | 14723 |
|  | rs13100427 | 3 | 22458640 | A | G | 0.0533 | 0.012 | 0.56 | 8.93E-06 | 14296 |
|  | rs5019353 | 3 | 45755730 | T | G | -0.0598 | 0.0134 | 0.5219 | 8.09E-06 | 11743 |
|  | rs113010081 | 3 | 46457412 | T | C | -0.5767 | 0.0184 | 0.8759 | 1.24E-215 | 14296 |
|  | rs140505982 | 3 | 46459749 | T | C | -0.2294 | 0.0415 | 0.0248 | 3.24E-08 | 13865 |
|  | rs66822987 | 3 | 63106417 | T | C | 0.0648 | 0.0146 | 0.2289 | 9.06E-06 | 14296 |
|  | rs9818901 | 3 | 152777012 | A | T | -0.1185 | 0.0265 | 0.9445 | 7.76E-06 | 14742 |
|  | rs112846979 | 3 | 190832842 | T | C | -0.188 | 0.0414 | 0.0228 | 5.60E-06 | 14296 |
|  | rs5743614 | 4 | 38798935 | T | C | 0.0728 | 0.0143 | 0.2565 | 3.56E-07 | 12859 |
|  | rs111476793 | 4 | 53951496 | A | G | 0.0869 | 0.019 | 0.1344 | 4.79E-06 | 12800 |
|  | rs78044056 | 4 | 157055604 | A | G | -0.2607 | 0.0552 | 0.0138 | 2.33E-06 | 13809 |
|  | rs42279 | 5 | 11317698 | T | C | 0.0743 | 0.0162 | 0.1598 | 4.51E-06 | 14738 |
|  | rs112315344 | 5 | 33775793 | T | G | 0.2123 | 0.0455 | 0.0284 | 3.07E-06 | 14255 |
|  | rs17792183 | 6 | 22899548 | T | C | 0.0763 | 0.017 | 0.8565 | 7.18E-06 | 14296 |
|  | rs141958785 | 6 | 124666261 | T | G | 0.0547 | 0.0123 | 0.3884 | 8.70E-06 | 14296 |
|  | rs112356872 | 7 | 22212109 | T | C | -0.2165 | 0.0478 | 0.9805 | 5.92E-06 | 14743 |
|  | rs3860989 | 9 | 33460141 | A | C | -0.0631 | 0.0142 | 0.6988 | 8.84E-06 | 11984 |
|  | rs10990301 | 9 | 105528047 | A | T | 0.085 | 0.0186 | 0.8839 | 4.88E-06 | 14296 |
|  | rs56131468 | 10 | 132716137 | A | G | 0.0833 | 0.0178 | 0.1323 | 2.87E-06 | 14296 |
|  | rs200664920 | 11 | 30476151 | T | A | 0.1906 | 0.0364 | 0.965 | 1.64E-07 | 11342 |
|  | rs143960014 | 12 | 52285090 | A | G | 0.1953 | 0.0416 | 0.0228 | 2.67E-06 | 14257 |
|  | rs1161092 | 12 | 67722012 | A | G | -0.0635 | 0.0125 | 0.6742 | 3.77E-07 | 14296 |
|  | rs11619402 | 13 | 43555687 | A | G | 0.0672 | 0.015 | 0.7907 | 7.46E-06 | 14296 |
|  | rs72800563 | 16 | 79338459 | T | G | -0.1765 | 0.0374 | 0.9693 | 2.37E-06 | 14741 |
|  | rs1634503 | 17 | 34421139 | A | G | -0.0745 | 0.0138 | 0.2416 | 6.72E-08 | 14295 |
|  | rs8064426 | 17 | 34819750 | A | G | -0.4104 | 0.0173 | 0.1459 | 2.11E-124 | 14295 |
|  | rs17692864 | 17 | 34945214 | T | C | 0.2321 | 0.0283 | 0.9406 | 2.38E-16 | 13662 |
|  | rs137896358 | 19 | 18917908 | T | C | 0.2797 | 0.0551 | 0.0186 | 3.85E-07 | 12745 |
|  | rs183450854 | 20 | 50372747 | C | G | 0.2133 | 0.0475 | 0.9802 | 7.10E-06 | 14737 |
|  | rs6100282 | 20 | 57507885 | A | G | -0.0715 | 0.0159 | 0.198 | 6.90E-06 | 12799 |
|  | rs4817032 | 21 | 26966560 | A | T | -0.0899 | 0.0196 | 0.8283 | 4.50E-06 | 10894 |
| CD244 | rs10903115 | 1 | 25261696 | A | G | -0.0559 | 0.0122 | 0.4429 | 4.61E-06 | 14286 |
|  | rs3828139 | 1 | 44253015 | T | C | 0.0744 | 0.0119 | 0.4677 | 4.05E-10 | 14286 |
|  | rs147033439 | 1 | 88677329 | A | G | 0.2053 | 0.0411 | 0.0249 | 5.88E-07 | 14731 |
|  | rs11265488 | 1 | 160773957 | A | G | -0.0624 | 0.0118 | 0.4675 | 1.24E-07 | 14287 |
|  | rs11265493 | 1 | 160803802 | A | G | 0.2122 | 0.0119 | 0.4066 | 3.99E-71 | 14287 |
|  | rs113798825 | 1 | 233431752 | T | C | -0.3154 | 0.0695 | 0.9868 | 5.68E-06 | 11273 |
|  | rs114344979 | 2 | 49094515 | T | C | 0.1066 | 0.024 | 0.0652 | 8.93E-06 | 14724 |
|  | rs4672486 | 2 | 62495209 | A | G | 0.0661 | 0.0117 | 0.4329 | 1.61E-08 | 14720 |
|  | rs1434219 | 2 | 79554556 | T | C | -0.0589 | 0.0123 | 0.4388 | 1.68E-06 | 14287 |
|  | rs56272634 | 2 | 111846068 | T | C | 0.0704 | 0.0126 | 0.6994 | 2.31E-08 | 14729 |
|  | rs148214660 | 3 | 112589458 | A | G | -0.2705 | 0.0612 | 0.0131 | 9.87E-06 | 14287 |
|  | rs73173072 | 3 | 167513752 | A | T | 0.1576 | 0.0353 | 0.9675 | 8.02E-06 | 14718 |
|  | rs1574847 | 4 | 27611759 | C | G | -0.0637 | 0.0139 | 0.2366 | 4.59E-06 | 14287 |
|  | rs114758826 | 4 | 174530615 | A | G | -0.1804 | 0.0337 | 0.0393 | 8.65E-08 | 14728 |
|  | rs72756898 | 5 | 64185695 | C | G | 0.2056 | 0.0454 | 0.0192 | 5.94E-06 | 14734 |
|  | rs78142752 | 5 | 96078542 | T | C | 0.2288 | 0.0497 | 0.0184 | 4.15E-06 | 14734 |
|  | rs140189694 | 5 | 113042679 | A | T | 0.2783 | 0.0621 | 0.9887 | 7.41E-06 | 12934 |
|  | rs6928352 | 6 | 614064 | A | G | -0.1074 | 0.0215 | 0.0794 | 5.87E-07 | 14731 |
|  | rs149826608 | 7 | 109234908 | A | G | -0.3587 | 0.0807 | 0.007 | 8.80E-06 | 13156 |
|  | rs2554697 | 8 | 3921805 | A | G | 0.0627 | 0.0134 | 0.6347 | 2.88E-06 | 11782 |
|  | rs145175118 | 8 | 92927069 | T | C | 0.2308 | 0.052 | 0.9818 | 9.06E-06 | 14287 |
|  | rs34826779 | 8 | 106570964 | T | G | 0.063 | 0.0138 | 0.248 | 4.99E-06 | 14287 |
|  | rs10992054 | 9 | 92856642 | T | C | -0.3705 | 0.0763 | 0.9873 | 1.20E-06 | 12304 |
|  | rs145111742 | 9 | 129557272 | T | C | 0.3046 | 0.0689 | 0.0304 | 9.83E-06 | 12304 |
|  | rs61865960 | 10 | 95662408 | T | C | 0.2604 | 0.0584 | 0.9697 | 8.24E-06 | 11810 |
|  | rs11020961 | 11 | 94575608 | A | G | 0.0768 | 0.0161 | 0.8167 | 1.84E-06 | 14287 |
|  | rs11220506 | 11 | 126335329 | A | G | -0.0702 | 0.0147 | 0.1876 | 1.79E-06 | 14729 |
|  | rs3184504 | 12 | 111884608 | T | C | 0.116 | 0.0128 | 0.4764 | 1.27E-19 | 11784 |
|  | rs149458216 | 16 | 48902293 | A | C | -0.2146 | 0.047 | 0.0182 | 4.97E-06 | 14725 |
|  | rs72837690 | 17 | 7088423 | T | C | -0.0776 | 0.0125 | 0.6405 | 5.37E-10 | 14287 |
|  | rs149212182 | 20 | 60283624 | T | C | 0.2642 | 0.059 | 0.0173 | 7.54E-06 | 12321 |
|  | rs60006581 | 21 | 38144075 | A | G | 0.0905 | 0.0203 | 0.9052 | 8.27E-06 | 14724 |
|  | rs139924614 | 22 | 17214005 | A | G | 0.1618 | 0.0348 | 0.9666 | 3.33E-06 | 14725 |
| CD40 | rs17372980 | 1 | 59140291 | T | C | -0.0611 | 0.0134 | 0.7421 | 5.12E-06 | 14288 |
|  | rs4278412 | 1 | 211450157 | C | G | 0.1978 | 0.0413 | 0.0203 | 1.67E-06 | 14725 |
|  | rs138897138 | 2 | 122731427 | A | G | 0.109 | 0.0216 | 0.914 | 4.50E-07 | 14288 |
|  | rs4664334 | 2 | 161368905 | T | C | 0.0541 | 0.0118 | 0.5003 | 4.55E-06 | 14288 |
|  | rs3967170 | 3 | 3773438 | A | G | 0.0789 | 0.017 | 0.1353 | 3.46E-06 | 14721 |
|  | rs144949926 | 3 | 20921481 | A | G | -0.2655 | 0.0591 | 0.9784 | 7.04E-06 | 14288 |
|  | rs12500563 | 4 | 7304426 | T | G | 0.3143 | 0.0687 | 0.9875 | 4.76E-06 | 10795 |
|  | rs9993874 | 4 | 55046724 | A | G | -0.0582 | 0.0125 | 0.4883 | 3.22E-06 | 12403 |
|  | rs74794857 | 5 | 22143588 | T | C | 0.0707 | 0.0138 | 0.4128 | 3.00E-07 | 11760 |
|  | rs11755766 | 6 | 10178951 | T | C | -0.0504 | 0.0112 | 0.5049 | 6.80E-06 | 14735 |
|  | rs200824954 | 7 | 29807677 | T | G | 0.1568 | 0.0335 | 0.0642 | 2.86E-06 | 10894 |
|  | rs7798473 | 7 | 108543387 | A | C | -0.1041 | 0.0235 | 0.0609 | 9.43E-06 | 14734 |
|  | rs149900704 | 8 | 111799116 | A | G | -0.1956 | 0.0442 | 0.0203 | 9.63E-06 | 14732 |
|  | rs75626299 | 9 | 29426599 | C | G | 0.1458 | 0.0316 | 0.9638 | 3.95E-06 | 14728 |
|  | rs9526031 | 13 | 45595067 | A | C | 0.064 | 0.012 | 0.4882 | 9.64E-08 | 14288 |
|  | rs8024256 | 15 | 26829521 | A | G | -0.0562 | 0.0121 | 0.3092 | 3.41E-06 | 14736 |
|  | rs404842 | 16 | 84304113 | A | G | -0.0599 | 0.0123 | 0.6433 | 1.12E-06 | 14287 |
|  | rs35864161 | 17 | 66365155 | T | G | -0.1269 | 0.0284 | 0.9543 | 7.88E-06 | 14735 |
|  | rs34755276 | 19 | 57755822 | A | G | 0.0587 | 0.0131 | 0.3475 | 7.43E-06 | 12403 |
|  | rs7250371 | 19 | 57861085 | T | C | 0.0686 | 0.0116 | 0.3648 | 3.34E-09 | 14722 |
|  | rs187640246 | 20 | 30007295 | T | C | -0.2717 | 0.0604 | 0.0134 | 6.85E-06 | 12792 |
|  | rs117191103 | 20 | 44464356 | T | C | 0.3553 | 0.0437 | 0.0208 | 4.28E-16 | 14736 |
|  | rs73128569 | 20 | 44606446 | A | T | -0.1788 | 0.0268 | 0.9406 | 2.53E-11 | 14288 |
|  | rs1883832 | 20 | 44746982 | T | C | -0.4519 | 0.0126 | 0.2575 | 1.07E-281 | 14736 |
|  | rs138739860 | 21 | 18103398 | T | G | 0.1632 | 0.0351 | 0.9648 | 3.33E-06 | 11979 |
|  | rs12152184 | 22 | 45002809 | A | G | 0.1085 | 0.0205 | 0.8849 | 1.21E-07 | 12402 |
| CD5 | rs61768480 | 1 | 1103150 | A | G | 0.0695 | 0.0147 | 0.2776 | 2.27E-06 | 13670 |
|  | rs61747728 | 1 | 179526214 | T | C | 0.1841 | 0.0313 | 0.0399 | 4.06E-09 | 14287 |
|  | rs13010492 | 2 | 204776176 | C | G | -0.0589 | 0.0122 | 0.4754 | 1.38E-06 | 13421 |
|  | rs138861398 | 4 | 77211819 | A | C | -0.0803 | 0.0151 | 0.2321 | 1.05E-07 | 11967 |
|  | rs74874989 | 4 | 164762615 | A | G | 0.141 | 0.0292 | 0.1033 | 1.37E-06 | 9398 |
|  | rs141298297 | 5 | 17157345 | G | T | 0.1034 | 0.0232 | 0.8877 | 8.32E-06 | 10894 |
|  | rs4073745 | 5 | 176733341 | A | G | -0.0683 | 0.0131 | 0.7003 | 1.85E-07 | 14287 |
|  | rs367364 | 6 | 32019946 | T | C | -0.0828 | 0.016 | 0.1538 | 2.28E-07 | 14730 |
|  | rs3104373 | 6 | 32600375 | T | C | 0.0973 | 0.0165 | 0.1469 | 3.70E-09 | 14735 |
|  | rs2237450 | 7 | 50716017 | T | C | 0.0623 | 0.0124 | 0.6389 | 5.06E-07 | 14287 |
|  | rs17345759 | 7 | 97499410 | A | C | -0.1134 | 0.0245 | 0.9301 | 3.68E-06 | 14287 |
|  | rs7012814 | 8 | 9173358 | A | G | -0.0533 | 0.0119 | 0.4819 | 7.50E-06 | 14287 |
|  | rs74796205 | 8 | 67386869 | A | G | 0.2987 | 0.0657 | 0.9884 | 5.46E-06 | 13800 |
|  | rs17769062 | 9 | 27496604 | T | C | 0.1638 | 0.0334 | 0.9589 | 9.38E-07 | 14287 |
|  | rs192582121 | 9 | 105614669 | A | T | -0.4169 | 0.0806 | 0.9916 | 2.31E-07 | 11438 |
|  | rs117276991 | 10 | 6251691 | A | G | -0.1261 | 0.0256 | 0.0751 | 8.40E-07 | 14287 |
|  | rs12416520 | 10 | 71211945 | A | G | -0.0629 | 0.0132 | 0.7492 | 1.89E-06 | 14709 |
|  | rs60562286 | 10 | 114454187 | A | T | -0.1681 | 0.0352 | 0.0327 | 1.79E-06 | 14726 |
|  | rs4910962 | 11 | 4281522 | A | C | 0.1465 | 0.0312 | 0.9341 | 2.66E-06 | 11760 |
|  | rs4939490 | 11 | 60793651 | C | G | 0.1083 | 0.012 | 0.6328 | 1.80E-19 | 14735 |
|  | rs674379 | 11 | 60922561 | C | G | 0.1193 | 0.0126 | 0.6395 | 2.85E-21 | 12835 |
|  | rs193245191 | 11 | 102724025 | A | G | -0.3109 | 0.0696 | 0.0109 | 7.93E-06 | 12290 |
|  | rs78808574 | 11 | 127558155 | A | G | -0.118 | 0.0265 | 0.9449 | 8.47E-06 | 14287 |
|  | rs1259355 | 12 | 31688535 | A | G | 0.0569 | 0.0119 | 0.4361 | 1.74E-06 | 14287 |
|  | rs7295476 | 12 | 74680262 | A | T | -0.118 | 0.0265 | 0.0501 | 8.47E-06 | 14735 |
|  | rs3184504 | 12 | 111884608 | T | C | 0.1427 | 0.0128 | 0.4764 | 7.29E-29 | 11784 |
|  | rs6488973 | 12 | 125487245 | A | G | -0.0613 | 0.0135 | 0.2804 | 5.61E-06 | 14722 |
|  | rs7298384 | 12 | 129141888 | A | G | -0.1418 | 0.032 | 0.9625 | 9.37E-06 | 14718 |
|  | rs117516652 | 13 | 39003323 | A | G | 0.2374 | 0.0476 | 0.9826 | 6.12E-07 | 14248 |
|  | rs11622090 | 14 | 93329692 | T | C | -0.0588 | 0.0128 | 0.3206 | 4.35E-06 | 13236 |
|  | rs16541 | 17 | 37365138 | A | G | -0.0679 | 0.015 | 0.7871 | 5.99E-06 | 14287 |
|  | rs7242462 | 18 | 45545720 | A | G | -0.1199 | 0.0187 | 0.8853 | 1.44E-10 | 14287 |
|  | rs4893 | 19 | 48833608 | A | G | 0.1471 | 0.0313 | 0.9627 | 2.61E-06 | 14735 |
| CD6 | rs143374617 | 1 | 4327524 | T | C | 0.1766 | 0.0329 | 0.0397 | 7.97E-08 | 14733 |
|  | rs841332 | 1 | 95171460 | A | G | -0.0721 | 0.0154 | 0.8286 | 2.84E-06 | 14727 |
|  | rs12074680 | 1 | 194299592 | A | G | -0.1855 | 0.0392 | 0.0301 | 2.22E-06 | 12404 |
|  | rs200729584 | 2 | 112870516 | A | T | 0.3331 | 0.0646 | 0.9834 | 2.52E-07 | 10407 |
|  | rs35086654 | 2 | 173467755 | T | C | -0.2416 | 0.0517 | 0.017 | 2.97E-06 | 13800 |
|  | rs72969761 | 2 | 227938476 | A | T | -0.1796 | 0.0376 | 0.97 | 1.78E-06 | 14710 |
|  | rs116557290 | 3 | 26765764 | A | G | 0.1439 | 0.0325 | 0.0401 | 9.52E-06 | 14735 |
|  | rs150354514 | 4 | 7067069 | C | G | -0.3633 | 0.0813 | 0.9897 | 7.87E-06 | 11336 |
|  | rs56294840 | 4 | 56646059 | A | G | 0.0776 | 0.0171 | 0.858 | 5.68E-06 | 14287 |
|  | rs74886615 | 4 | 83099601 | A | T | 0.0688 | 0.0129 | 0.6149 | 9.64E-08 | 12404 |
|  | rs74874989 | 4 | 164762615 | A | G | 0.1283 | 0.029 | 0.1032 | 9.68E-06 | 9398 |
|  | rs78007268 | 7 | 29401094 | A | G | 0.2645 | 0.0593 | 0.0124 | 8.18E-06 | 14248 |
|  | rs9691968 | 7 | 153635380 | A | C | 0.0646 | 0.0134 | 0.2505 | 1.43E-06 | 14735 |
|  | rs1483572 | 8 | 79635243 | A | C | 0.0602 | 0.0124 | 0.3374 | 1.20E-06 | 14287 |
|  | rs60988380 | 9 | 33117954 | T | C | 0.0955 | 0.0193 | 0.1058 | 7.49E-07 | 14735 |
|  | rs28509311 | 9 | 137135257 | A | G | -0.2416 | 0.0533 | 0.9722 | 5.82E-06 | 9406 |
|  | rs2237995 | 11 | 60749256 | A | G | -0.3633 | 0.0218 | 0.0784 | 2.35E-62 | 14725 |
|  | rs11230555 | 11 | 60763134 | A | C | -0.6032 | 0.0134 | 0.2215 | 0.00E+00 | 14287 |
|  | rs10792322 | 11 | 61761552 | T | C | -0.0662 | 0.0126 | 0.3002 | 1.49E-07 | 14734 |
|  | rs10843957 | 12 | 31647305 | A | T | 0.0529 | 0.0117 | 0.5922 | 6.14E-06 | 14725 |
|  | rs597808 | 12 | 111973358 | A | G | 0.116 | 0.0134 | 0.4779 | 4.86E-18 | 11335 |
|  | rs28741066 | 14 | 20317880 | T | C | 0.1266 | 0.0283 | 0.1022 | 7.70E-06 | 12404 |
|  | rs77020403 | 14 | 24430996 | A | G | -0.0632 | 0.014 | 0.4175 | 6.35E-06 | 12404 |
|  | rs4902621 | 14 | 69103560 | C | G | 0.0685 | 0.0154 | 0.1762 | 8.67E-06 | 14725 |
|  | rs144764321 | 16 | 60666212 | A | G | -0.2823 | 0.0621 | 0.0168 | 5.47E-06 | 14287 |
|  | rs8051133 | 16 | 86486037 | A | G | -0.1079 | 0.0236 | 0.0691 | 4.83E-06 | 14729 |
|  | rs35634144 | 22 | 37638221 | C | G | 0.1752 | 0.0376 | 0.9711 | 3.17E-06 | 14237 |
| CDCP1 | rs5010503 | 1 | 19875930 | A | T | -0.1578 | 0.0352 | 0.0675 | 7.36E-06 | 12404 |
|  | rs61782461 | 1 | 101804329 | T | C | -0.2176 | 0.0471 | 0.9829 | 3.84E-06 | 14286 |
|  | rs2990246 | 1 | 155197602 | C | G | -0.0566 | 0.012 | 0.4028 | 2.40E-06 | 14286 |
|  | rs59872649 | 2 | 148430383 | A | G | -0.1093 | 0.0232 | 0.0652 | 2.46E-06 | 14734 |
|  | rs34027772 | 3 | 43423959 | T | C | 0.0757 | 0.0143 | 0.2607 | 1.20E-07 | 12403 |
|  | rs7618923 | 3 | 45130725 | T | C | -0.1738 | 0.0151 | 0.1811 | 1.18E-30 | 14286 |
|  | rs2276862 | 3 | 45187785 | C | G | 0.303 | 0.0146 | 0.8212 | 1.14E-95 | 14730 |
|  | rs35478654 | 3 | 58453757 | T | C | -0.148 | 0.0312 | 0.0367 | 2.10E-06 | 14725 |
|  | rs111476793 | 4 | 53951496 | A | G | 0.0895 | 0.0182 | 0.1339 | 8.76E-07 | 12790 |
|  | rs10044847 | 5 | 2103391 | C | G | -0.0781 | 0.0156 | 0.8283 | 5.55E-07 | 14719 |
|  | rs6452820 | 5 | 72411335 | A | G | -0.0738 | 0.0159 | 0.8367 | 3.46E-06 | 14286 |
|  | rs115009171 | 6 | 622484 | A | G | 0.4223 | 0.0932 | 0.0078 | 5.87E-06 | 10300 |
|  | rs9272226 | 6 | 32602396 | T | C | -0.0933 | 0.0118 | 0.6505 | 2.64E-15 | 14733 |
|  | rs118116993 | 6 | 162326838 | A | T | 0.0957 | 0.0197 | 0.0969 | 1.19E-06 | 14286 |
|  | rs184033839 | 7 | 89952215 | A | C | -0.2509 | 0.0514 | 0.0133 | 1.05E-06 | 14246 |
|  | rs59803571 | 8 | 27678341 | A | T | 0.1022 | 0.0227 | 0.8817 | 6.73E-06 | 10894 |
|  | rs6991332 | 8 | 61937553 | T | C | -0.0579 | 0.0119 | 0.6455 | 1.14E-06 | 14286 |
|  | rs1463919 | 8 | 91776995 | A | C | 0.0507 | 0.0113 | 0.4982 | 7.23E-06 | 14285 |
|  | rs10964983 | 9 | 21413803 | C | G | -0.0616 | 0.0114 | 0.5862 | 6.53E-08 | 14724 |
|  | rs10465082 | 9 | 35993938 | T | C | -0.0504 | 0.0112 | 0.5023 | 6.80E-06 | 14733 |
|  | rs35176543 | 11 | 17556914 | T | G | 0.0894 | 0.0201 | 0.0924 | 8.68E-06 | 14286 |
|  | rs672806 | 11 | 126188405 | A | G | 0.0816 | 0.0116 | 0.4097 | 2.00E-12 | 14286 |
|  | rs12290068 | 11 | 126261564 | A | G | 0.1221 | 0.0122 | 0.3187 | 1.40E-23 | 14726 |
|  | rs111984955 | 12 | 15991375 | C | G | 0.2058 | 0.0461 | 0.9817 | 8.04E-06 | 14725 |
|  | rs17607930 | 12 | 60829749 | A | G | 0.1654 | 0.037 | 0.9669 | 7.81E-06 | 14286 |
|  | rs17078000 | 13 | 84915900 | A | G | 0.0609 | 0.0128 | 0.7179 | 1.96E-06 | 14286 |
|  | rs28535751 | 14 | 59010649 | T | C | -0.0541 | 0.0118 | 0.6294 | 4.55E-06 | 14733 |
|  | rs59752567 | 15 | 58791416 | T | G | -0.0923 | 0.0204 | 0.0938 | 6.05E-06 | 14730 |
|  | rs11864674 | 16 | 66391749 | A | C | 0.2029 | 0.0458 | 0.0186 | 9.42E-06 | 14246 |
|  | rs12928188 | 16 | 79183747 | A | T | -0.0658 | 0.0134 | 0.2312 | 9.09E-07 | 14715 |
|  | rs75892552 | 17 | 36021431 | T | G | -0.1259 | 0.0279 | 0.0544 | 6.41E-06 | 14286 |
|  | rs74811341 | 17 | 38720826 | T | C | -0.1323 | 0.0277 | 0.0513 | 1.79E-06 | 14734 |
|  | rs7506429 | 18 | 19991319 | A | C | 0.0737 | 0.0144 | 0.7965 | 3.09E-07 | 14286 |
|  | rs4804148 | 19 | 11270867 | T | C | -0.0619 | 0.0125 | 0.7187 | 7.35E-07 | 14734 |
|  | rs146583535 | 20 | 18761853 | A | G | 0.4557 | 0.0968 | 0.0265 | 2.51E-06 | 8372 |
|  | rs2742145 | 21 | 37270018 | A | G | 0.2325 | 0.051 | 0.0213 | 5.14E-06 | 11538 |
|  | rs191929998 | 22 | 31852311 | A | G | -0.1938 | 0.0409 | 0.9581 | 2.15E-06 | 9844 |
| CSF-1 | rs7538029 | 1 | 110474695 | A | C | 0.0775 | 0.0165 | 0.2211 | 2.64E-06 | 11335 |
|  | rs17610659 | 1 | 110503296 | T | C | 0.1684 | 0.0118 | 0.4821 | 3.31E-46 | 14286 |
|  | rs142421172 | 1 | 179682087 | A | G | 0.1439 | 0.031 | 0.044 | 3.45E-06 | 12840 |
|  | rs11806838 | 1 | 190519358 | T | C | 0.0674 | 0.0152 | 0.185 | 9.24E-06 | 14286 |
|  | rs11685487 | 2 | 8775320 | T | C | 0.0944 | 0.0196 | 0.1197 | 1.46E-06 | 14286 |
|  | rs846654 | 2 | 10646611 | T | C | -0.0519 | 0.0114 | 0.5786 | 5.30E-06 | 14707 |
|  | rs116443177 | 2 | 27764122 | T | C | 0.0827 | 0.0173 | 0.1595 | 1.75E-06 | 12403 |
|  | rs115370595 | 2 | 140484970 | A | G | -0.2277 | 0.0496 | 0.9815 | 4.42E-06 | 13799 |
|  | rs72997796 | 3 | 1456080 | C | G | 0.0888 | 0.0192 | 0.1075 | 3.75E-06 | 14286 |
|  | rs188348611 | 3 | 47563522 | A | T | 0.1847 | 0.0412 | 0.9719 | 7.36E-06 | 12354 |
|  | rs76421506 | 3 | 115295680 | A | G | 0.0778 | 0.0168 | 0.853 | 3.64E-06 | 14286 |
|  | rs74849049 | 3 | 171430935 | T | G | -0.0914 | 0.02 | 0.8992 | 4.88E-06 | 14286 |
|  | rs78652121 | 4 | 13713646 | C | G | -0.1189 | 0.026 | 0.0697 | 4.81E-06 | 12838 |
|  | rs116039112 | 4 | 154438727 | A | G | 0.2238 | 0.0504 | 0.016 | 8.98E-06 | 14286 |
|  | rs75305583 | 4 | 183461339 | T | C | -0.1493 | 0.0329 | 0.0382 | 5.68E-06 | 14286 |
|  | rs10042665 | 5 | 78775099 | C | G | 0.0583 | 0.0122 | 0.3043 | 1.76E-06 | 14727 |
|  | rs3934571 | 5 | 174229899 | T | C | -0.0802 | 0.018 | 0.1143 | 8.37E-06 | 14732 |
|  | rs2523992 | 6 | 30075103 | T | G | -0.075 | 0.0152 | 0.8323 | 8.05E-07 | 14734 |
|  | rs7825914 | 8 | 19590012 | A | G | 0.054 | 0.0122 | 0.3028 | 9.59E-06 | 14732 |
|  | rs12549430 | 8 | 80715263 | A | G | -0.061 | 0.0135 | 0.738 | 6.23E-06 | 14286 |
|  | rs1605333 | 12 | 58388232 | C | G | -0.0601 | 0.0124 | 0.6484 | 1.25E-06 | 13237 |
|  | rs142902162 | 13 | 75660663 | T | C | 0.3234 | 0.0723 | 0.0106 | 7.71E-06 | 12898 |
|  | rs17734417 | 14 | 58461604 | T | C | -0.1225 | 0.0265 | 0.9466 | 3.79E-06 | 14723 |
|  | rs75869070 | 14 | 78693608 | A | G | 0.1151 | 0.0239 | 0.936 | 1.47E-06 | 14715 |
|  | rs28673875 | 15 | 45690795 | A | T | -0.2096 | 0.046 | 0.9801 | 5.20E-06 | 14236 |
|  | rs34674345 | 15 | 63787216 | T | C | -0.2243 | 0.0503 | 0.9814 | 8.22E-06 | 14286 |
|  | rs72783071 | 16 | 60437963 | T | C | -0.199 | 0.0414 | 0.0231 | 1.53E-06 | 14247 |
|  | rs17638424 | 18 | 3048453 | A | T | 0.1034 | 0.023 | 0.9329 | 6.94E-06 | 14715 |
|  | rs7227917 | 18 | 45546185 | A | G | -0.0872 | 0.0185 | 0.8823 | 2.43E-06 | 14286 |
|  | rs117591817 | 19 | 58875573 | C | G | -0.1989 | 0.0435 | 0.0348 | 4.82E-06 | 14286 |
| CST5 | rs1536565 | 1 | 102420542 | A | G | -0.1244 | 0.0264 | 0.9481 | 2.45E-06 | 14735 |
|  | rs930735 | 1 | 202185979 | A | G | 0.0579 | 0.0114 | 0.547 | 3.80E-07 | 14734 |
|  | rs2791713 | 1 | 207153881 | A | G | 0.0532 | 0.0117 | 0.4536 | 5.44E-06 | 14288 |
|  | rs76753488 | 2 | 36609059 | A | C | 0.1171 | 0.0263 | 0.9478 | 8.49E-06 | 14736 |
|  | rs13397003 | 2 | 62640887 | T | G | 0.0701 | 0.0141 | 0.7748 | 6.64E-07 | 14732 |
|  | rs138248604 | 2 | 173718684 | A | G | 0.2257 | 0.0508 | 0.0336 | 8.87E-06 | 14731 |
|  | rs72980022 | 2 | 233853187 | A | G | 0.0607 | 0.0126 | 0.2813 | 1.45E-06 | 14721 |
|  | rs145735210 | 4 | 22724745 | T | C | 0.2031 | 0.0454 | 0.0213 | 7.69E-06 | 14242 |
|  | rs13127297 | 4 | 23899510 | A | G | 0.0545 | 0.0117 | 0.4095 | 3.19E-06 | 14730 |
|  | rs35936034 | 4 | 71466227 | T | C | 0.0628 | 0.0142 | 0.2106 | 9.75E-06 | 14718 |
|  | rs10065995 | 5 | 10215009 | T | C | -0.0536 | 0.0121 | 0.6595 | 9.43E-06 | 14723 |
|  | rs150547 | 5 | 59193431 | A | C | 0.0548 | 0.0119 | 0.4071 | 4.12E-06 | 14288 |
|  | rs139918398 | 5 | 96079646 | T | C | 0.3373 | 0.0676 | 0.0111 | 6.05E-07 | 13457 |
|  | rs79359257 | 5 | 109366574 | T | C | 0.073 | 0.0156 | 0.8152 | 2.88E-06 | 14288 |
|  | rs9327707 | 5 | 134512655 | T | C | 0.059 | 0.0118 | 0.3955 | 5.73E-07 | 14710 |
|  | rs3889401 | 6 | 7051402 | A | G | 0.0885 | 0.0191 | 0.8624 | 3.60E-06 | 11759 |
|  | rs62436191 | 6 | 146809314 | A | G | 0.1194 | 0.0234 | 0.0762 | 3.35E-07 | 14288 |
|  | rs9455923 | 6 | 168345756 | A | G | 0.0537 | 0.0115 | 0.4261 | 3.02E-06 | 14734 |
|  | rs111613686 | 7 | 5003387 | A | G | -0.2439 | 0.0537 | 0.0187 | 5.57E-06 | 13801 |
|  | rs1477229 | 7 | 131455573 | T | C | -0.0612 | 0.0136 | 0.2323 | 6.80E-06 | 14735 |
|  | rs17154027 | 8 | 11713852 | A | G | -0.0558 | 0.0121 | 0.6302 | 4.00E-06 | 14288 |
|  | rs77556255 | 8 | 43501515 | T | C | -0.3228 | 0.0723 | 0.98 | 8.02E-06 | 9731 |
|  | rs4507745 | 8 | 56143053 | T | C | 0.057 | 0.0122 | 0.6482 | 2.98E-06 | 13866 |
|  | rs962720 | 9 | 109490400 | T | C | 0.0558 | 0.0119 | 0.5837 | 2.74E-06 | 14287 |
|  | rs78295068 | 10 | 63140238 | A | G | 0.0777 | 0.0173 | 0.167 | 7.08E-06 | 12404 |
|  | rs7069904 | 10 | 89649838 | A | G | 0.1309 | 0.0293 | 0.0427 | 7.91E-06 | 14733 |
|  | rs140505821 | 11 | 21963583 | A | G | 0.3276 | 0.0691 | 0.0089 | 2.13E-06 | 13381 |
|  | rs4756151 | 11 | 34495358 | A | C | -0.094 | 0.0172 | 0.1273 | 4.63E-08 | 14736 |
|  | rs12294339 | 11 | 34860199 | T | C | 0.0702 | 0.0118 | 0.3671 | 2.70E-09 | 14726 |
|  | rs145343977 | 11 | 97836152 | T | C | 0.2679 | 0.0597 | 0.0156 | 7.21E-06 | 14288 |
|  | rs11054069 | 12 | 11058117 | T | C | 0.1674 | 0.013 | 0.7421 | 6.07E-38 | 14724 |
|  | rs141192864 | 12 | 31381343 | T | C | 0.1016 | 0.0213 | 0.0812 | 1.84E-06 | 14728 |
|  | rs4766797 | 12 | 117050587 | A | G | -0.0574 | 0.0117 | 0.4935 | 9.30E-07 | 14288 |
|  | rs67020211 | 15 | 63639644 | T | G | 0.079 | 0.0115 | 0.4609 | 6.44E-12 | 14734 |
|  | rs12924891 | 16 | 66817975 | T | C | 0.2573 | 0.0571 | 0.0141 | 6.60E-06 | 13422 |
|  | rs58679828 | 17 | 64340405 | A | G | 0.1808 | 0.0407 | 0.0225 | 8.90E-06 | 14736 |
|  | rs60586299 | 18 | 3479423 | T | C | -0.0654 | 0.0134 | 0.3077 | 1.06E-06 | 14288 |
|  | rs470841 | 18 | 24286681 | T | C | 0.0568 | 0.0119 | 0.6369 | 1.81E-06 | 14736 |
|  | rs516316 | 19 | 49206145 | C | G | 0.0845 | 0.0115 | 0.4441 | 2.01E-13 | 14734 |
|  | rs34074361 | 20 | 23464311 | A | G | 0.0814 | 0.0145 | 0.1953 | 1.98E-08 | 14736 |
|  | rs11696133 | 20 | 23810508 | T | C | -0.6554 | 0.0149 | 0.8166 | 0.00E+00 | 14288 |
|  | rs1555472 | 20 | 23957181 | A | C | 0.1411 | 0.0155 | 0.2118 | 8.77E-20 | 12403 |
|  | rs141179525 | 20 | 24071731 | T | C | -0.2583 | 0.0447 | 0.9784 | 7.54E-09 | 14735 |
|  | rs4813529 | 20 | 24691928 | T | G | -0.0726 | 0.012 | 0.3362 | 1.45E-09 | 14714 |
|  | rs697699 | 20 | 51363052 | A | C | 0.1098 | 0.0237 | 0.0675 | 3.61E-06 | 14288 |
|  | rs7275493 | 21 | 18049996 | T | C | -0.0572 | 0.0119 | 0.6243 | 1.53E-06 | 14727 |
|  | rs77121303 | 22 | 37630079 | A | G | -0.2516 | 0.0532 | 0.983 | 2.25E-06 | 13383 |
| CX3CL1 | rs114359340 | 1 | 6532317 | A | G | -0.1881 | 0.0405 | 0.9747 | 3.41E-06 | 13651 |
|  | rs4047522 | 1 | 161608256 | T | C | -0.0639 | 0.0142 | 0.4952 | 6.80E-06 | 11768 |
|  | rs77915710 | 1 | 172099857 | A | G | -0.1436 | 0.0304 | 0.0402 | 2.32E-06 | 14742 |
|  | rs72704449 | 1 | 179473273 | T | C | -0.1358 | 0.0298 | 0.9547 | 5.19E-06 | 14295 |
|  | rs71451021 | 2 | 19059082 | A | C | -0.165 | 0.0362 | 0.9728 | 5.16E-06 | 14740 |
|  | rs111472651 | 2 | 44117788 | C | G | -0.1191 | 0.0248 | 0.9355 | 1.57E-06 | 14728 |
|  | rs11682107 | 2 | 113835522 | A | G | -0.0675 | 0.0126 | 0.5568 | 8.45E-08 | 12412 |
|  | rs72985859 | 2 | 232254374 | T | C | 0.1351 | 0.0292 | 0.0806 | 3.72E-06 | 8297 |
|  | rs1976172 | 3 | 1260482 | C | G | -0.0569 | 0.012 | 0.3525 | 2.12E-06 | 14732 |
|  | rs3732378 | 3 | 39307162 | A | G | 0.0941 | 0.0154 | 0.1671 | 9.94E-10 | 14740 |
|  | rs35026817 | 3 | 70136592 | T | G | 0.0633 | 0.0122 | 0.3327 | 2.12E-07 | 14740 |
|  | rs980364 | 4 | 179539497 | A | G | 0.1479 | 0.0301 | 0.9343 | 8.94E-07 | 9902 |
|  | rs12331618 | 4 | 187139939 | A | G | -0.0582 | 0.0114 | 0.4914 | 3.30E-07 | 14729 |
|  | rs13167439 | 5 | 120396066 | T | C | -0.0903 | 0.0193 | 0.89 | 2.89E-06 | 14295 |
|  | rs2731674 | 5 | 176839890 | T | G | -0.0681 | 0.0128 | 0.274 | 1.04E-07 | 14740 |
|  | rs2442752 | 6 | 31351764 | T | C | -0.0589 | 0.0117 | 0.6177 | 4.80E-07 | 14743 |
|  | rs6937696 | 6 | 32418018 | T | C | -0.0811 | 0.012 | 0.651 | 1.40E-11 | 14742 |
|  | rs2736723 | 7 | 30524015 | A | T | 0.0597 | 0.012 | 0.4243 | 6.52E-07 | 14295 |
|  | rs56397903 | 8 | 26741279 | A | G | 0.1968 | 0.0424 | 0.9744 | 3.46E-06 | 14743 |
|  | rs16904321 | 8 | 131635787 | A | G | -0.0867 | 0.0193 | 0.8632 | 7.05E-06 | 14295 |
|  | rs77819349 | 9 | 81886673 | C | G | 0.1196 | 0.0263 | 0.9371 | 5.43E-06 | 14295 |
|  | rs635634 | 9 | 136155000 | T | C | -0.1145 | 0.0166 | 0.1884 | 5.29E-12 | 11792 |
|  | rs56675723 | 11 | 120941125 | T | C | 0.0666 | 0.0149 | 0.7791 | 7.83E-06 | 12412 |
|  | rs712137 | 12 | 41381284 | A | C | 0.0563 | 0.012 | 0.6601 | 2.71E-06 | 14737 |
|  | rs10778675 | 12 | 79905558 | A | C | 0.0792 | 0.0158 | 0.1687 | 5.37E-07 | 14295 |
|  | rs36067349 | 15 | 23001299 | T | C | 0.0666 | 0.015 | 0.8038 | 9.00E-06 | 14294 |
|  | rs117698194 | 15 | 85657494 | A | G | -0.1996 | 0.0432 | 0.0224 | 3.83E-06 | 13859 |
|  | rs8052631 | 16 | 57167230 | A | G | -0.1511 | 0.027 | 0.9463 | 2.19E-08 | 14295 |
|  | rs78751841 | 16 | 57183274 | A | G | -0.1935 | 0.0433 | 0.9764 | 7.87E-06 | 14723 |
|  | rs671623 | 16 | 57412802 | C | G | 0.2041 | 0.0118 | 0.424 | 4.99E-67 | 14295 |
|  | rs55896747 | 16 | 57508595 | T | C | 0.1129 | 0.0244 | 0.9372 | 3.71E-06 | 14734 |
|  | rs73007244 | 18 | 59374764 | T | C | 0.0813 | 0.0179 | 0.1321 | 5.57E-06 | 14295 |
|  | rs73137770 | 20 | 52569781 | C | G | 0.2442 | 0.0529 | 0.9812 | 3.91E-06 | 13808 |
|  | rs9981489 | 21 | 25498266 | T | C | 0.1127 | 0.0254 | 0.0652 | 9.12E-06 | 14295 |
| CXCL1 | rs863004 | 1 | 159176831 | T | C | 0.0661 | 0.0119 | 0.3809 | 2.78E-08 | 14722 |
|  | rs75883619 | 1 | 191712620 | T | C | -0.2241 | 0.0502 | 0.9769 | 8.04E-06 | 14736 |
|  | rs6700633 | 1 | 211439817 | T | C | -0.1886 | 0.042 | 0.9791 | 7.11E-06 | 14726 |
|  | rs6807587 | 3 | 45270029 | A | G | 0.0577 | 0.0128 | 0.2947 | 6.55E-06 | 14733 |
|  | rs2122155 | 4 | 59689961 | A | G | -0.0538 | 0.0117 | 0.5637 | 4.26E-06 | 14717 |
|  | rs1366946 | 4 | 74738469 | T | C | -0.3134 | 0.0134 | 0.7638 | 5.65E-121 | 14730 |
|  | rs7700661 | 5 | 122405522 | A | G | 0.0556 | 0.0124 | 0.3196 | 7.33E-06 | 14719 |
|  | rs144747179 | 5 | 159394723 | T | C | 0.2626 | 0.0582 | 0.0157 | 6.42E-06 | 12591 |
|  | rs374636704 | 5 | 177372616 | T | G | 0.3935 | 0.0869 | 0.009 | 5.95E-06 | 8911 |
|  | rs77914035 | 6 | 54641812 | A | G | -0.1047 | 0.0206 | 0.8675 | 3.72E-07 | 10893 |
|  | rs144620567 | 7 | 115483444 | T | C | 0.1891 | 0.0426 | 0.9761 | 9.04E-06 | 14730 |
|  | rs67797811 | 9 | 4396102 | C | G | 0.0822 | 0.0179 | 0.1691 | 4.39E-06 | 14288 |
|  | rs146204850 | 10 | 13896867 | T | C | 0.2706 | 0.0601 | 0.0138 | 6.72E-06 | 13801 |
|  | rs10508556 | 10 | 18285342 | T | C | 0.0518 | 0.0116 | 0.4633 | 7.99E-06 | 14735 |
|  | rs12770839 | 10 | 65297720 | A | C | 0.0648 | 0.0126 | 0.321 | 2.71E-07 | 14724 |
|  | rs10734292 | 11 | 20486401 | A | G | 0.123 | 0.0267 | 0.067 | 4.09E-06 | 11776 |
|  | rs118090102 | 12 | 28799395 | T | G | -0.2573 | 0.0582 | 0.0166 | 9.83E-06 | 12935 |
|  | rs141276323 | 12 | 90180208 | C | G | 0.1982 | 0.0444 | 0.0196 | 8.05E-06 | 14728 |
|  | rs937341 | 12 | 125093430 | T | G | 0.066 | 0.0146 | 0.7719 | 6.17E-06 | 14288 |
|  | rs4474574 | 13 | 37854253 | T | C | -0.365 | 0.0779 | 0.0146 | 2.79E-06 | 9555 |
|  | rs150441545 | 14 | 35091527 | A | C | -0.0719 | 0.0158 | 0.8268 | 5.35E-06 | 14288 |
|  | rs2280441 | 15 | 81240669 | A | G | -0.1443 | 0.0312 | 0.0405 | 3.75E-06 | 14735 |
|  | rs11880837 | 19 | 14808453 | A | G | 0.0583 | 0.0127 | 0.3161 | 4.42E-06 | 14288 |
|  | rs2835916 | 21 | 39102421 | C | G | -0.0564 | 0.012 | 0.3876 | 2.60E-06 | 14735 |
|  | rs188111670 | 22 | 16564036 | A | G | 0.0976 | 0.0208 | 0.3429 | 2.70E-06 | 7743 |
|  | rs8136931 | 22 | 33031339 | A | G | -0.1533 | 0.0344 | 0.0337 | 8.33E-06 | 14736 |
| CXCL10 | rs112202639 | 1 | 44566391 | A | G | -0.1609 | 0.0336 | 0.9671 | 1.68E-06 | 14744 |
|  | rs61811733 | 1 | 181143447 | C | G | -0.1436 | 0.0324 | 0.9616 | 9.33E-06 | 14738 |
|  | rs1563171 | 2 | 3891773 | A | G | 0.0592 | 0.0127 | 0.7042 | 3.14E-06 | 14740 |
|  | rs12476448 | 2 | 14400474 | T | C | 0.0536 | 0.0115 | 0.4864 | 3.15E-06 | 14737 |
|  | rs820963 | 2 | 57744868 | C | G | -0.0539 | 0.0122 | 0.3617 | 9.96E-06 | 14732 |
|  | rs2164693 | 2 | 139887164 | A | G | -0.1736 | 0.0393 | 0.9625 | 9.99E-06 | 14296 |
|  | rs76484468 | 2 | 227559824 | T | C | -0.2168 | 0.0482 | 0.9792 | 6.86E-06 | 14744 |
|  | rs12629593 | 3 | 54104178 | T | G | -0.0879 | 0.0182 | 0.1209 | 1.37E-06 | 14744 |
|  | rs3846056 | 3 | 134227292 | A | G | -0.0559 | 0.0124 | 0.3248 | 6.54E-06 | 14296 |
|  | rs72651343 | 4 | 76808216 | T | C | -0.6096 | 0.0619 | 0.9892 | 6.98E-23 | 14257 |
|  | rs115140093 | 4 | 76910673 | T | C | -0.5186 | 0.042 | 0.9732 | 5.02E-35 | 14744 |
|  | rs12646113 | 4 | 77175245 | T | C | 0.0977 | 0.0136 | 0.246 | 6.78E-13 | 14743 |
|  | rs72682331 | 4 | 112904535 | A | G | 0.1089 | 0.0209 | 0.0979 | 1.88E-07 | 14731 |
|  | rs59465157 | 5 | 115740808 | A | G | 0.1295 | 0.0287 | 0.0442 | 6.42E-06 | 14744 |
|  | rs2523495 | 6 | 31377978 | T | C | 0.1095 | 0.0196 | 0.1048 | 2.31E-08 | 14743 |
|  | rs71534595 | 6 | 32516855 | T | C | -0.2991 | 0.0606 | 0.9813 | 7.99E-07 | 11719 |
|  | rs56283616 | 6 | 55774512 | T | C | 0.0677 | 0.0149 | 0.352 | 5.53E-06 | 10894 |
|  | rs12174864 | 6 | 88557407 | A | G | -0.0582 | 0.0121 | 0.6254 | 1.51E-06 | 14295 |
|  | rs117298099 | 6 | 152726625 | A | G | 0.1456 | 0.0325 | 0.0363 | 7.46E-06 | 14725 |
|  | rs12155428 | 7 | 3425301 | T | C | -0.1765 | 0.0375 | 0.0284 | 2.52E-06 | 14733 |
|  | rs2349689 | 8 | 62003730 | A | G | -0.0789 | 0.0164 | 0.2555 | 1.50E-06 | 14296 |
|  | rs143796249 | 9 | 4150061 | C | G | -0.2858 | 0.062 | 0.012 | 4.03E-06 | 14296 |
|  | rs10481754 | 9 | 84196490 | T | C | -0.0548 | 0.0119 | 0.4567 | 4.12E-06 | 14296 |
|  | rs4367871 | 10 | 1845059 | T | C | -0.0567 | 0.0119 | 0.5587 | 1.89E-06 | 14296 |
|  | rs7115485 | 11 | 33788499 | A | G | 0.1157 | 0.0254 | 0.0613 | 5.24E-06 | 14252 |
|  | rs55732300 | 11 | 73731422 | A | G | 0.1175 | 0.025 | 0.929 | 2.60E-06 | 14730 |
|  | rs11825923 | 11 | 116006514 | T | C | 0.1921 | 0.0421 | 0.0237 | 5.04E-06 | 14247 |
|  | rs741344 | 12 | 68597086 | A | G | -0.06 | 0.013 | 0.7036 | 3.92E-06 | 14296 |
|  | rs3184504 | 12 | 111884608 | T | C | 0.1138 | 0.0131 | 0.4756 | 3.72E-18 | 11793 |
|  | rs1077965 | 15 | 80065337 | A | G | -0.0546 | 0.0118 | 0.5648 | 3.71E-06 | 14731 |
|  | rs11645285 | 16 | 5063233 | C | G | -0.1096 | 0.0242 | 0.9073 | 5.93E-06 | 11345 |
|  | rs144219691 | 17 | 33794704 | A | C | 0.0645 | 0.0145 | 0.6875 | 8.66E-06 | 10894 |
|  | rs74956615 | 19 | 10427721 | A | T | -0.1358 | 0.0294 | 0.0495 | 3.86E-06 | 13671 |
|  | rs4804148 | 19 | 11270867 | T | C | -0.0629 | 0.013 | 0.7227 | 1.31E-06 | 14744 |
|  | rs62208309 | 20 | 51942555 | T | C | -0.1371 | 0.0309 | 0.0542 | 9.13E-06 | 14296 |
|  | rs150519056 | 22 | 50774821 | T | C | -0.1841 | 0.0376 | 0.9688 | 9.77E-07 | 13661 |
| CXCL11 | rs60141220 | 1 | 26395741 | A | G | -0.0777 | 0.0165 | 0.1703 | 2.49E-06 | 13221 |
|  | rs185516256 | 1 | 51541675 | T | C | 0.1566 | 0.0349 | 0.9575 | 7.22E-06 | 14288 |
|  | rs2013836 | 1 | 181142796 | A | C | 0.1383 | 0.0309 | 0.0402 | 7.62E-06 | 14732 |
|  | rs17466177 | 1 | 222852472 | T | C | -0.1822 | 0.0409 | 0.9741 | 8.40E-06 | 14723 |
|  | rs115344667 | 2 | 78400313 | T | C | -0.1344 | 0.0302 | 0.9568 | 8.57E-06 | 14736 |
|  | rs114013730 | 2 | 112444622 | A | G | 0.3893 | 0.0853 | 0.9892 | 5.02E-06 | 11961 |
|  | rs12998222 | 2 | 153497193 | T | C | 0.2339 | 0.0528 | 0.9855 | 9.43E-06 | 13801 |
|  | rs148828177 | 2 | 240244812 | T | C | -0.1509 | 0.034 | 0.0385 | 9.07E-06 | 14732 |
|  | rs79378014 | 3 | 114380762 | A | T | -0.1549 | 0.0341 | 0.0325 | 5.56E-06 | 14734 |
|  | rs62291421 | 3 | 188015332 | C | G | 0.2674 | 0.0518 | 0.9763 | 2.44E-07 | 12305 |
|  | rs6827617 | 4 | 76916146 | A | G | -0.1724 | 0.0118 | 0.6041 | 2.42E-48 | 14718 |
|  | rs59148364 | 4 | 77130802 | A | G | 0.1821 | 0.0378 | 0.0278 | 1.45E-06 | 14721 |
|  | rs116470923 | 4 | 77374393 | T | C | -0.1975 | 0.0427 | 0.0216 | 3.74E-06 | 14736 |
|  | rs374784992 | 4 | 119585039 | A | G | 0.1349 | 0.0297 | 0.9402 | 5.57E-06 | 11760 |
|  | rs112884138 | 4 | 134362360 | T | C | -0.0976 | 0.0209 | 0.0892 | 3.01E-06 | 14728 |
|  | rs72747175 | 5 | 18009856 | A | G | 0.1535 | 0.0326 | 0.9608 | 2.49E-06 | 14730 |
|  | rs2894233 | 6 | 32011678 | A | G | -0.2236 | 0.047 | 0.9724 | 1.96E-06 | 11917 |
|  | rs72925566 | 6 | 89498971 | A | G | -0.0973 | 0.0185 | 0.1651 | 1.44E-07 | 11337 |
|  | rs7750544 | 6 | 154177484 | T | C | -0.0773 | 0.0164 | 0.165 | 2.44E-06 | 13644 |
|  | rs75612655 | 7 | 101594247 | T | C | 0.5352 | 0.0705 | 0.0084 | 3.16E-14 | 12935 |
|  | rs111787182 | 7 | 157911633 | A | C | -0.1445 | 0.0314 | 0.951 | 4.19E-06 | 13224 |
|  | rs78632565 | 8 | 70982905 | T | C | 0.1082 | 0.0214 | 0.9152 | 4.28E-07 | 14722 |
|  | rs7009937 | 8 | 96176796 | T | C | 0.1175 | 0.025 | 0.9321 | 2.60E-06 | 14288 |
|  | rs4748380 | 10 | 17590681 | T | C | 0.0901 | 0.019 | 0.1188 | 2.12E-06 | 14732 |
|  | rs77516076 | 10 | 50895131 | C | G | 0.1915 | 0.0432 | 0.0233 | 9.30E-06 | 14730 |
|  | rs10733789 | 10 | 64948684 | T | C | -0.0815 | 0.0127 | 0.6875 | 1.39E-10 | 14287 |
|  | rs7475335 | 10 | 104343428 | A | C | -0.0646 | 0.0124 | 0.3172 | 1.89E-07 | 14735 |
|  | rs111848253 | 11 | 22351553 | A | G | -0.1162 | 0.025 | 0.9044 | 3.35E-06 | 11538 |
|  | rs10898158 | 11 | 83554112 | A | G | 0.0609 | 0.0121 | 0.6028 | 4.83E-07 | 14288 |
|  | rs2678131 | 12 | 43091649 | T | C | -0.1497 | 0.033 | 0.0348 | 5.72E-06 | 14720 |
|  | rs10876335 | 12 | 53150540 | C | G | 0.0607 | 0.0133 | 0.3069 | 5.02E-06 | 13240 |
|  | rs3184504 | 12 | 111884608 | T | C | 0.1236 | 0.0132 | 0.4764 | 7.70E-21 | 11785 |
|  | rs371789589 | 13 | 53082648 | A | G | 0.154 | 0.034 | 0.0398 | 5.91E-06 | 12189 |
|  | rs12433317 | 14 | 50775476 | A | G | 0.0571 | 0.012 | 0.4282 | 1.95E-06 | 14288 |
|  | rs147096628 | 16 | 55366068 | A | C | -0.239 | 0.0529 | 0.0224 | 6.24E-06 | 12935 |
|  | rs222772 | 17 | 3584167 | T | C | -0.0654 | 0.0144 | 0.428 | 5.58E-06 | 11538 |
|  | rs7222922 | 17 | 34335694 | T | C | -0.0882 | 0.0182 | 0.1205 | 1.26E-06 | 14726 |
|  | rs144908342 | 17 | 71105507 | A | G | -0.1763 | 0.0394 | 0.0292 | 7.65E-06 | 14727 |
|  | rs2245786 | 20 | 4795382 | A | G | -0.0599 | 0.0131 | 0.6525 | 4.82E-06 | 14721 |
|  | rs191536201 | 20 | 17839484 | A | G | -0.3323 | 0.0684 | 0.0117 | 1.18E-06 | 11194 |
|  | rs17824652 | 20 | 41987913 | T | C | 0.1168 | 0.0258 | 0.9406 | 5.98E-06 | 14288 |
| CXCL5 | rs9429422 | 1 | 112467126 | T | C | 0.0601 | 0.0135 | 0.2633 | 8.51E-06 | 14288 |
|  | rs4279906 | 1 | 211477063 | T | C | 0.2082 | 0.042 | 0.0209 | 7.15E-07 | 14717 |
|  | rs7636889 | 3 | 56874033 | A | G | -0.0768 | 0.0137 | 0.7593 | 2.07E-08 | 14733 |
|  | rs146553494 | 3 | 183850210 | A | G | 0.1844 | 0.0377 | 0.0265 | 1.00E-06 | 14733 |
|  | rs11734099 | 4 | 6891435 | A | G | 0.0769 | 0.0153 | 0.1845 | 5.00E-07 | 14731 |
|  | rs28750249 | 4 | 23807061 | T | G | -0.118 | 0.0266 | 0.945 | 9.16E-06 | 14735 |
|  | rs425535 | 4 | 74863997 | T | C | 0.5063 | 0.0181 | 0.1127 | 3.52E-172 | 14731 |
|  | rs79649467 | 4 | 74880852 | T | G | -0.1721 | 0.0276 | 0.0605 | 4.50E-10 | 14288 |
|  | rs77914035 | 6 | 54641812 | A | G | -0.1174 | 0.0206 | 0.8675 | 1.20E-08 | 10893 |
|  | rs2647400 | 6 | 76351999 | C | G | 0.0631 | 0.013 | 0.2784 | 1.21E-06 | 14734 |
|  | rs7781261 | 7 | 111992684 | A | G | -0.0675 | 0.0151 | 0.8155 | 7.81E-06 | 14728 |
|  | rs11779495 | 8 | 81858024 | A | G | 0.1153 | 0.0253 | 0.0709 | 5.18E-06 | 14288 |
|  | rs6993770 | 8 | 106581528 | A | T | -0.1058 | 0.0135 | 0.7312 | 4.61E-15 | 14288 |
|  | rs2004100 | 8 | 144554731 | T | C | 0.0675 | 0.0147 | 0.2492 | 4.39E-06 | 13224 |
|  | rs3183927 | 9 | 100684757 | A | G | -0.0619 | 0.0121 | 0.6436 | 3.13E-07 | 14725 |
|  | rs10821552 | 9 | 136939992 | A | C | -0.1132 | 0.0156 | 0.3353 | 3.97E-13 | 9841 |
|  | rs7090111 | 10 | 65077994 | C | G | -0.1734 | 0.0117 | 0.5834 | 1.08E-49 | 14735 |
|  | rs111848253 | 11 | 22351553 | A | G | -0.1111 | 0.0249 | 0.9045 | 8.13E-06 | 11538 |
|  | rs140828666 | 13 | 53109423 | A | G | -0.1474 | 0.0322 | 0.9544 | 4.70E-06 | 12401 |
|  | rs916776 | 16 | 26812516 | T | G | 0.0546 | 0.0122 | 0.3883 | 7.63E-06 | 14287 |
|  | rs7197943 | 16 | 64226202 | A | C | 0.0552 | 0.0123 | 0.6522 | 7.20E-06 | 14719 |
|  | rs59312158 | 16 | 88582345 | A | G | -0.19 | 0.0385 | 0.0309 | 8.01E-07 | 13672 |
|  | rs10403201 | 19 | 14764810 | A | G | -0.0601 | 0.0127 | 0.2925 | 2.22E-06 | 14730 |
|  | rs188111670 | 22 | 16564036 | A | G | 0.0949 | 0.0209 | 0.3428 | 5.61E-06 | 7743 |
| CXCL6 | rs77336315 | 1 | 97287518 | A | C | 0.2417 | 0.0532 | 0.9838 | 5.54E-06 | 13809 |
|  | rs12075 | 1 | 159175354 | A | G | 0.0816 | 0.0115 | 0.5554 | 1.29E-12 | 14741 |
|  | rs6540677 | 1 | 211474259 | T | C | -0.185 | 0.0416 | 0.9711 | 8.70E-06 | 12840 |
|  | rs184103539 | 1 | 222149663 | T | C | -0.1603 | 0.0351 | 0.039 | 4.95E-06 | 12841 |
|  | rs545114 | 3 | 15082196 | A | G | -0.0558 | 0.0125 | 0.6634 | 8.04E-06 | 14743 |
|  | rs16850073 | 4 | 74703999 | T | C | 0.4978 | 0.0118 | 0.3722 | 0.00E+00 | 14296 |
|  | rs116381873 | 4 | 74825095 | T | C | -0.264 | 0.0531 | 0.9852 | 6.64E-07 | 13379 |
|  | rs191180456 | 4 | 74826316 | T | C | -0.406 | 0.0648 | 0.0112 | 3.72E-10 | 13809 |
|  | rs35096759 | 4 | 75172482 | T | C | 0.2109 | 0.0427 | 0.0308 | 7.85E-07 | 14296 |
|  | rs374784992 | 4 | 119585039 | A | G | 0.1333 | 0.0298 | 0.9401 | 7.71E-06 | 11768 |
|  | rs77914035 | 6 | 54641812 | A | G | -0.0916 | 0.0207 | 0.8674 | 9.64E-06 | 10893 |
|  | rs17147002 | 7 | 88295512 | A | G | -0.0538 | 0.0119 | 0.5826 | 6.15E-06 | 14734 |
|  | rs144620567 | 7 | 115483444 | T | C | 0.1887 | 0.0426 | 0.9761 | 9.44E-06 | 14738 |
|  | rs182381042 | 8 | 93838957 | A | G | -0.3831 | 0.0799 | 0.0074 | 1.63E-06 | 12935 |
|  | rs4877818 | 9 | 86683575 | T | G | 0.0625 | 0.0134 | 0.5188 | 3.10E-06 | 11538 |
|  | rs10984957 | 9 | 123330336 | A | C | -0.1024 | 0.0228 | 0.0754 | 7.08E-06 | 14740 |
|  | rs111836168 | 10 | 24373882 | T | G | -0.2018 | 0.0443 | 0.0323 | 5.23E-06 | 12412 |
|  | rs11598331 | 10 | 121351367 | A | G | 0.0791 | 0.0179 | 0.1233 | 9.92E-06 | 14737 |
|  | rs142702207 | 11 | 86628253 | T | C | -0.1882 | 0.0419 | 0.0296 | 7.07E-06 | 14249 |
|  | rs118177380 | 11 | 106531034 | A | G | 0.2275 | 0.051 | 0.0188 | 8.17E-06 | 14296 |
|  | rs3184504 | 12 | 111884608 | T | C | 0.0679 | 0.0131 | 0.4766 | 2.18E-07 | 11793 |
|  | rs34080302 | 14 | 89402542 | T | G | 0.0605 | 0.0131 | 0.715 | 3.87E-06 | 14296 |
|  | rs11547311 | 16 | 2222286 | T | C | 0.1285 | 0.0272 | 0.0649 | 2.31E-06 | 13232 |
|  | rs8049795 | 16 | 86395119 | T | C | -0.0538 | 0.0119 | 0.4107 | 6.15E-06 | 14744 |
|  | rs12480971 | 20 | 45697196 | T | C | -0.1011 | 0.0225 | 0.0744 | 7.01E-06 | 14729 |
|  | rs117109857 | 21 | 39099887 | C | G | 0.1897 | 0.0415 | 0.0242 | 4.85E-06 | 14743 |
|  | rs377588320 | 22 | 35739167 | A | C | -0.3485 | 0.0719 | 0.9837 | 1.25E-06 | 10271 |
| CXCL9 | rs192119373 | 1 | 3823464 | T | G | 0.6177 | 0.1287 | 0.0057 | 1.59E-06 | 8829 |
|  | rs4047522 | 1 | 161608256 | T | C | -0.0645 | 0.0137 | 0.4962 | 2.50E-06 | 11760 |
|  | rs10921271 | 1 | 192799276 | A | G | 0.0626 | 0.0138 | 0.6904 | 5.73E-06 | 12404 |
|  | rs114033894 | 2 | 86977227 | T | G | -0.2245 | 0.0488 | 0.981 | 4.22E-06 | 14287 |
|  | rs7600322 | 2 | 204754353 | T | C | 0.057 | 0.0118 | 0.5214 | 1.36E-06 | 13849 |
|  | rs79551873 | 2 | 231423737 | T | C | 0.0533 | 0.0119 | 0.3563 | 7.50E-06 | 14287 |
|  | rs72991127 | 2 | 236906094 | C | G | -0.0583 | 0.0131 | 0.264 | 8.57E-06 | 14287 |
|  | rs35435997 | 2 | 242665455 | A | G | 0.0915 | 0.02 | 0.1125 | 4.76E-06 | 12791 |
|  | rs7429588 | 3 | 46835250 | T | C | 0.0592 | 0.0134 | 0.706 | 9.97E-06 | 14285 |
|  | rs11130569 | 3 | 57075894 | A | G | -0.051 | 0.0114 | 0.4547 | 7.69E-06 | 14727 |
|  | rs6548432 | 3 | 76525063 | A | G | -0.0514 | 0.0114 | 0.4276 | 6.52E-06 | 14724 |
|  | rs62272916 | 3 | 151541803 | A | G | -0.0834 | 0.0176 | 0.1182 | 2.15E-06 | 14710 |
|  | rs10008912 | 4 | 7148069 | A | G | 0.0586 | 0.0131 | 0.5255 | 7.70E-06 | 14287 |
|  | rs4241577 | 4 | 76930776 | A | C | -0.1419 | 0.0128 | 0.2629 | 1.47E-28 | 14724 |
|  | rs80348644 | 4 | 160574480 | T | C | 0.1546 | 0.0332 | 0.0336 | 3.21E-06 | 14734 |
|  | rs145607633 | 5 | 19287867 | T | C | 0.0728 | 0.0154 | 0.1625 | 2.28E-06 | 14287 |
|  | rs2535260 | 6 | 29628883 | C | G | 0.0642 | 0.0144 | 0.8128 | 8.26E-06 | 14735 |
|  | rs9366778 | 6 | 31269173 | A | G | -0.0605 | 0.0115 | 0.4075 | 1.43E-07 | 14735 |
|  | rs181816009 | 6 | 31423412 | A | G | -0.1603 | 0.0342 | 0.0378 | 2.77E-06 | 11985 |
|  | rs783149 | 6 | 161088918 | A | C | -0.0805 | 0.0144 | 0.1958 | 2.27E-08 | 14287 |
|  | rs12191307 | 6 | 161256529 | A | G | -0.1452 | 0.0197 | 0.098 | 1.70E-13 | 14287 |
|  | rs656739 | 7 | 29049949 | A | G | 0.0562 | 0.0119 | 0.6566 | 2.33E-06 | 14735 |
|  | rs142119726 | 7 | 48859798 | T | C | 0.2705 | 0.0551 | 0.9851 | 9.14E-07 | 13800 |
|  | rs1758695 | 9 | 94165889 | T | G | 0.0511 | 0.0115 | 0.5681 | 8.85E-06 | 14732 |
|  | rs113504136 | 10 | 13400390 | T | G | -0.1494 | 0.0338 | 0.0325 | 9.86E-06 | 14723 |
|  | rs6586167 | 10 | 90756146 | T | G | -0.052 | 0.0114 | 0.5056 | 5.08E-06 | 14735 |
|  | rs61875680 | 10 | 107966626 | A | G | -0.0758 | 0.0158 | 0.1611 | 1.61E-06 | 14287 |
|  | rs192488790 | 11 | 7767109 | T | C | 0.2369 | 0.0532 | 0.0168 | 8.47E-06 | 13421 |
|  | rs368022534 | 11 | 102612924 | A | G | 0.1686 | 0.0371 | 0.9563 | 5.51E-06 | 9256 |
|  | rs58524610 | 12 | 11768143 | T | G | 0.223 | 0.0491 | 0.0204 | 5.58E-06 | 14287 |
|  | rs3184504 | 12 | 111884608 | T | C | 0.1008 | 0.013 | 0.4744 | 8.92E-15 | 11784 |
|  | rs11059314 | 12 | 128159229 | T | C | -0.0559 | 0.0124 | 0.6535 | 6.54E-06 | 14287 |
|  | rs118106660 | 14 | 54798313 | A | G | 0.259 | 0.0573 | 0.0138 | 6.18E-06 | 13800 |
|  | rs7149776 | 14 | 74739091 | A | T | -0.0574 | 0.0115 | 0.5236 | 6.00E-07 | 14286 |
|  | rs182152471 | 16 | 1803589 | T | C | 0.1861 | 0.0385 | 0.028 | 1.34E-06 | 13667 |
|  | rs4888966 | 16 | 79431853 | C | G | -0.157 | 0.0343 | 0.9683 | 4.71E-06 | 14287 |
|  | rs75525140 | 18 | 28624793 | C | G | -0.2062 | 0.0465 | 0.9819 | 9.23E-06 | 14725 |
|  | rs74956615 | 19 | 10427721 | A | T | -0.1309 | 0.0286 | 0.049 | 4.72E-06 | 13662 |
|  | rs73606147 | 20 | 2538467 | A | C | 0.2792 | 0.059 | 0.0133 | 2.22E-06 | 13379 |
|  | rs62200304 | 20 | 20119931 | A | C | -0.115 | 0.0244 | 0.9336 | 2.44E-06 | 14287 |
|  | rs73194812 | 21 | 18734554 | A | G | 0.1821 | 0.0391 | 0.9758 | 3.20E-06 | 14730 |
|  | rs73367822 | 21 | 45024252 | T | C | -0.0588 | 0.0126 | 0.5804 | 3.06E-06 | 11760 |
| DNER | rs10191831 | 2 | 41864408 | T | C | -0.0591 | 0.0125 | 0.4361 | 2.27E-06 | 12403 |
|  | rs35107184 | 2 | 113834459 | T | G | 0.0586 | 0.013 | 0.4047 | 6.55E-06 | 11759 |
|  | rs116540722 | 2 | 168312525 | A | G | -0.1726 | 0.0381 | 0.0313 | 5.89E-06 | 14735 |
|  | rs207671 | 2 | 230256586 | A | G | 0.1016 | 0.0117 | 0.5359 | 3.83E-18 | 14287 |
|  | rs35032874 | 2 | 230309360 | T | G | 0.132 | 0.0132 | 0.7027 | 1.52E-23 | 14287 |
|  | rs62193248 | 2 | 230596917 | A | T | 0.213 | 0.0123 | 0.6507 | 3.50E-67 | 14287 |
|  | rs62253189 | 3 | 69959680 | A | G | -0.108 | 0.0244 | 0.9404 | 9.59E-06 | 14718 |
|  | rs116794986 | 3 | 99306038 | A | G | 0.2476 | 0.0554 | 0.9884 | 7.85E-06 | 14735 |
|  | rs9289590 | 3 | 139639117 | T | C | -0.0652 | 0.0142 | 0.7033 | 4.40E-06 | 12404 |
|  | rs13107325 | 4 | 103188709 | T | C | 0.1066 | 0.0233 | 0.0697 | 4.76E-06 | 14732 |
|  | rs11725578 | 4 | 160466825 | C | G | -0.0584 | 0.0128 | 0.697 | 5.05E-06 | 14287 |
|  | rs74890918 | 6 | 32953148 | A | G | 0.2266 | 0.0502 | 0.0157 | 6.36E-06 | 14287 |
|  | rs7809355 | 7 | 25386238 | A | C | -0.0734 | 0.0164 | 0.1553 | 7.62E-06 | 14287 |
|  | rs117343254 | 7 | 152385669 | C | G | -0.1281 | 0.0274 | 0.942 | 2.94E-06 | 14287 |
|  | rs77209370 | 9 | 113330834 | A | C | 0.1346 | 0.0298 | 0.9589 | 6.28E-06 | 14231 |
|  | rs7123587 | 11 | 1681922 | A | C | -0.0552 | 0.0124 | 0.6266 | 8.52E-06 | 13222 |
|  | rs10892466 | 11 | 119661317 | C | G | 0.0808 | 0.0175 | 0.1236 | 3.89E-06 | 14732 |
|  | rs116179073 | 12 | 31458615 | T | C | -0.2481 | 0.0537 | 0.0138 | 3.84E-06 | 13800 |
|  | rs773112 | 12 | 56375881 | T | C | 0.0566 | 0.0125 | 0.3128 | 5.95E-06 | 14287 |
|  | rs9597690 | 13 | 58788135 | A | G | -0.0531 | 0.0118 | 0.646 | 6.80E-06 | 14720 |
|  | rs194746 | 14 | 69282887 | T | C | 0.0528 | 0.0112 | 0.4849 | 2.43E-06 | 14723 |
|  | rs75685422 | 14 | 95752666 | T | C | 0.0879 | 0.0186 | 0.1139 | 2.29E-06 | 14287 |
|  | rs28689968 | 17 | 47247257 | T | C | 0.0918 | 0.0184 | 0.1074 | 6.07E-07 | 14733 |
|  | rs1133851 | 18 | 3722038 | A | G | -0.0664 | 0.0142 | 0.2533 | 2.92E-06 | 14286 |
|  | rs116927267 | 20 | 4054373 | A | G | -0.1742 | 0.0389 | 0.0257 | 7.53E-06 | 14734 |
|  | rs9967959 | 21 | 28101979 | T | G | 0.0718 | 0.0152 | 0.1718 | 2.32E-06 | 14731 |
|  | rs6517655 | 21 | 42536246 | C | G | 0.0757 | 0.0129 | 0.289 | 4.41E-09 | 14287 |
|  | rs2837988 | 21 | 42619544 | A | C | 0.0576 | 0.0118 | 0.3578 | 1.05E-06 | 14733 |
| EN-RAGE | rs2742666 | 1 | 3058233 | T | C | 0.0629 | 0.0138 | 0.4364 | 5.17E-06 | 11348 |
|  | rs3014874 | 1 | 153337943 | A | G | -0.1726 | 0.0137 | 0.2623 | 2.15E-36 | 14294 |
|  | rs61803119 | 1 | 153390202 | T | G | 0.1501 | 0.0194 | 0.1069 | 1.02E-14 | 14295 |
|  | rs59538998 | 1 | 245480237 | C | G | -0.1188 | 0.0258 | 0.0622 | 4.13E-06 | 14295 |
|  | rs13384799 | 2 | 125135589 | T | C | -0.0622 | 0.0133 | 0.7266 | 2.92E-06 | 14295 |
|  | rs150886675 | 2 | 227300526 | A | G | -0.411 | 0.0911 | 0.0085 | 6.44E-06 | 9876 |
|  | rs17325940 | 3 | 60071129 | T | C | -0.0784 | 0.0174 | 0.8611 | 6.61E-06 | 14735 |
|  | rs56268787 | 3 | 152573197 | T | C | -0.0936 | 0.0203 | 0.0955 | 4.01E-06 | 14737 |
|  | rs150015576 | 3 | 174824333 | A | C | 0.194 | 0.0423 | 0.0217 | 4.51E-06 | 14740 |
|  | rs2056031 | 4 | 12920594 | T | C | -0.0604 | 0.0119 | 0.613 | 3.86E-07 | 14739 |
|  | rs1846881 | 4 | 16409999 | A | G | -0.0737 | 0.0165 | 0.1925 | 7.94E-06 | 12212 |
|  | rs140298956 | 5 | 39597187 | A | C | -0.2114 | 0.0467 | 0.0217 | 5.99E-06 | 14295 |
|  | rs191808657 | 7 | 75133433 | A | C | 0.3059 | 0.0665 | 0.983 | 4.22E-06 | 12412 |
|  | rs117923478 | 7 | 81211709 | T | C | 0.1795 | 0.0395 | 0.9721 | 5.51E-06 | 14295 |
|  | rs2946493 | 8 | 12823671 | T | C | 0.0664 | 0.0148 | 0.7902 | 7.24E-06 | 14295 |
|  | rs3802266 | 8 | 123985708 | A | G | 0.0555 | 0.0118 | 0.5633 | 2.56E-06 | 14740 |
|  | rs369132765 | 9 | 27275548 | A | C | 0.1301 | 0.0294 | 0.0558 | 9.64E-06 | 12194 |
|  | rs78800546 | 11 | 31879143 | A | G | -0.2529 | 0.0534 | 0.0156 | 2.18E-06 | 14295 |
|  | rs112956934 | 12 | 96571503 | T | C | 0.1884 | 0.0407 | 0.0246 | 3.67E-06 | 14737 |
|  | rs112988802 | 14 | 59990514 | T | C | -0.1396 | 0.0293 | 0.9492 | 1.89E-06 | 12851 |
|  | rs147302029 | 16 | 4602209 | A | G | 0.1806 | 0.0345 | 0.0379 | 1.65E-07 | 13649 |
|  | rs113955692 | 16 | 79653201 | T | C | 0.1621 | 0.0366 | 0.0315 | 9.47E-06 | 14295 |
|  | rs61742155 | 18 | 3896518 | A | C | 0.0805 | 0.018 | 0.129 | 7.74E-06 | 14729 |
|  | rs138439507 | 19 | 4247567 | C | G | 0.0865 | 0.0183 | 0.191 | 2.28E-06 | 11537 |
|  | rs2425640 | 20 | 43028037 | A | G | -0.0583 | 0.0123 | 0.3511 | 2.14E-06 | 14741 |
|  | rs55783131 | 22 | 17406250 | T | C | 0.2064 | 0.0425 | 0.0239 | 1.19E-06 | 14728 |
|  | rs174344 | 22 | 18032226 | A | G | 0.0617 | 0.0125 | 0.6296 | 7.97E-07 | 14295 |
| FGF-19 | rs12407505 | 1 | 85295202 | A | G | -0.1614 | 0.0352 | 0.9664 | 4.53E-06 | 14735 |
|  | rs834971 | 1 | 100098055 | A | G | -0.0614 | 0.0128 | 0.3002 | 1.61E-06 | 14728 |
|  | rs79439490 | 1 | 193891487 | T | C | -0.3528 | 0.079 | 0.984 | 7.98E-06 | 8334 |
|  | rs142585344 | 2 | 32584586 | A | G | 0.2133 | 0.0482 | 0.0218 | 9.63E-06 | 13809 |
|  | rs75715636 | 3 | 7913293 | A | G | 0.0838 | 0.0187 | 0.1237 | 7.42E-06 | 14296 |
|  | rs112126444 | 3 | 132162249 | A | G | -0.0927 | 0.0199 | 0.1012 | 3.19E-06 | 14735 |
|  | rs3846346 | 4 | 27730465 | T | G | 0.1372 | 0.0305 | 0.0459 | 6.85E-06 | 14729 |
|  | rs13103023 | 4 | 39457617 | A | G | 0.0837 | 0.0124 | 0.3415 | 1.48E-11 | 14296 |
|  | rs140276495 | 4 | 61879934 | A | G | 0.0586 | 0.0129 | 0.3096 | 5.56E-06 | 14296 |
|  | rs12520287 | 5 | 54847098 | T | C | -0.1014 | 0.0221 | 0.9175 | 4.47E-06 | 14296 |
|  | rs12520033 | 5 | 161543116 | A | G | -0.0802 | 0.0178 | 0.8026 | 6.62E-06 | 11341 |
|  | rs4483036 | 6 | 8777463 | A | C | -0.0675 | 0.0148 | 0.2516 | 5.10E-06 | 12412 |
|  | rs12110436 | 6 | 168606201 | T | C | -0.136 | 0.0297 | 0.0418 | 4.67E-06 | 14743 |
|  | rs145274424 | 7 | 17445553 | T | C | 0.1992 | 0.0421 | 0.9777 | 2.23E-06 | 14735 |
|  | rs2906655 | 7 | 101569302 | T | C | -0.0623 | 0.0135 | 0.2503 | 3.93E-06 | 14743 |
|  | rs7005978 | 8 | 59382715 | A | G | -0.1055 | 0.0123 | 0.3458 | 9.72E-18 | 14744 |
|  | rs79110990 | 9 | 18148302 | A | G | 0.073 | 0.0156 | 0.1762 | 2.88E-06 | 14295 |
|  | rs12342925 | 9 | 82082569 | A | T | -0.1352 | 0.0299 | 0.0429 | 6.13E-06 | 14744 |
|  | rs35836706 | 9 | 84435749 | A | G | 0.2151 | 0.0456 | 0.0203 | 2.39E-06 | 14734 |
|  | rs11244038 | 9 | 136095770 | C | G | 0.1199 | 0.0247 | 0.0898 | 1.21E-06 | 11786 |
|  | rs141140791 | 10 | 61186227 | T | C | -0.1293 | 0.0286 | 0.0497 | 6.16E-06 | 14733 |
|  | rs191522330 | 11 | 5610048 | T | C | 0.2325 | 0.0488 | 0.9812 | 1.89E-06 | 14738 |
|  | rs1671812 | 11 | 114495920 | T | C | 0.0621 | 0.0132 | 0.6316 | 2.54E-06 | 12412 |
|  | rs80023023 | 12 | 20656675 | A | G | -0.2706 | 0.0607 | 0.0119 | 8.27E-06 | 12935 |
|  | rs142067135 | 12 | 76644195 | A | G | -0.2295 | 0.0492 | 0.0198 | 3.09E-06 | 13809 |
|  | rs10777251 | 12 | 91080475 | A | G | -0.0767 | 0.0166 | 0.1923 | 3.83E-06 | 12411 |
|  | rs76708593 | 12 | 108147245 | T | C | 0.1024 | 0.0222 | 0.9158 | 3.98E-06 | 14296 |
|  | rs17326790 | 13 | 85357827 | T | C | 0.2161 | 0.0471 | 0.0209 | 4.47E-06 | 14741 |
|  | rs117438391 | 14 | 38084604 | T | C | -0.2079 | 0.0466 | 0.0225 | 8.14E-06 | 13868 |
|  | rs145434248 | 14 | 64892172 | A | G | -0.1442 | 0.0323 | 0.0425 | 8.03E-06 | 14296 |
|  | rs117398067 | 15 | 59188192 | T | C | 0.2583 | 0.0564 | 0.0178 | 4.65E-06 | 14744 |
|  | rs2306969 | 19 | 5851801 | A | G | 0.0674 | 0.0141 | 0.2338 | 1.75E-06 | 14739 |
|  | rs516246 | 19 | 49206172 | T | C | -0.1661 | 0.0118 | 0.445 | 5.31E-45 | 14744 |
|  | rs111914842 | 22 | 46332463 | T | C | -0.1634 | 0.0362 | 0.0368 | 6.37E-06 | 13667 |
| FGF-21 | rs145319387 | 1 | 188302601 | G | T | -0.0756 | 0.0162 | 0.769 | 3.06E-06 | 10893 |
|  | rs145127946 | 1 | 203746507 | A | G | -0.1747 | 0.036 | 0.037 | 1.22E-06 | 14295 |
|  | rs10495032 | 1 | 216837226 | T | G | -0.1557 | 0.0318 | 0.0383 | 9.77E-07 | 14720 |
|  | rs1260326 | 2 | 27730940 | T | C | 0.1323 | 0.0119 | 0.3977 | 1.03E-28 | 14730 |
|  | rs183222959 | 2 | 40402664 | A | G | 0.1419 | 0.0313 | 0.8998 | 5.80E-06 | 11538 |
|  | rs7610704 | 3 | 11689679 | T | C | -0.0568 | 0.012 | 0.4644 | 2.21E-06 | 14295 |
|  | rs13062487 | 3 | 71944243 | A | G | 0.0571 | 0.0126 | 0.3387 | 5.85E-06 | 14295 |
|  | rs17284047 | 3 | 100519020 | T | C | -0.2149 | 0.0479 | 0.9794 | 7.24E-06 | 14743 |
|  | rs7442192 | 4 | 45023201 | A | G | 0.0619 | 0.0138 | 0.7357 | 7.27E-06 | 14295 |
|  | rs4700382 | 5 | 55882435 | T | C | -0.063 | 0.0136 | 0.2698 | 3.62E-06 | 14295 |
|  | rs60277384 | 5 | 84356011 | T | C | -0.0881 | 0.019 | 0.8922 | 3.54E-06 | 14742 |
|  | rs35852631 | 5 | 107955452 | C | G | -0.0608 | 0.0135 | 0.2555 | 6.68E-06 | 14730 |
|  | rs12532101 | 7 | 1194526 | T | C | 0.0659 | 0.0149 | 0.203 | 9.74E-06 | 14295 |
|  | rs13229619 | 7 | 73030175 | A | G | -0.1609 | 0.0176 | 0.1297 | 6.13E-20 | 14743 |
|  | rs79789860 | 7 | 114309511 | T | C | -0.2003 | 0.0448 | 0.0209 | 7.79E-06 | 14729 |
|  | rs7012637 | 8 | 9173209 | A | G | -0.0605 | 0.012 | 0.4812 | 4.61E-07 | 14295 |
|  | rs8191009 | 8 | 30545827 | A | G | 0.0678 | 0.0151 | 0.1998 | 7.12E-06 | 14295 |
|  | rs7834283 | 8 | 135756557 | A | G | -0.3467 | 0.0784 | 0.01 | 9.77E-06 | 9555 |
|  | rs374354803 | 9 | 132338678 | A | G | 0.1208 | 0.0266 | 0.9316 | 5.59E-06 | 12198 |
|  | rs7083293 | 10 | 11862075 | C | G | -0.0595 | 0.0134 | 0.5772 | 8.98E-06 | 12412 |
|  | rs188758663 | 10 | 127847758 | A | G | -0.3801 | 0.0749 | 0.0104 | 3.88E-07 | 11668 |
|  | rs12290350 | 11 | 76480255 | T | C | -0.0689 | 0.0145 | 0.2168 | 2.02E-06 | 14295 |
|  | rs2429473 | 12 | 47198899 | A | C | -0.0812 | 0.0147 | 0.795 | 3.32E-08 | 14295 |
|  | rs72965996 | 18 | 72826607 | A | G | -0.2215 | 0.0444 | 0.031 | 6.08E-07 | 13808 |
|  | rs838131 | 19 | 49260677 | A | C | 0.1627 | 0.013 | 0.501 | 6.15E-36 | 14294 |
|  | rs142603673 | 21 | 33860969 | T | C | -0.2352 | 0.0455 | 0.0232 | 2.35E-07 | 14252 |
|  | rs9974115 | 21 | 37867322 | A | C | 0.0758 | 0.0169 | 0.8308 | 7.28E-06 | 14295 |
|  | rs149561599 | 22 | 20883740 | T | G | 0.1978 | 0.0444 | 0.9786 | 8.39E-06 | 13869 |
| FGF-23 | rs671205 | 1 | 146522722 | T | C | 0.0708 | 0.0136 | 0.7502 | 1.93E-07 | 14287 |
|  | rs72720704 | 1 | 176974037 | T | C | 0.0725 | 0.0161 | 0.8402 | 6.70E-06 | 14287 |
|  | rs10737349 | 1 | 180583036 | A | G | 0.0746 | 0.0167 | 0.1462 | 7.93E-06 | 14286 |
|  | rs138865481 | 1 | 205812500 | A | G | -0.2386 | 0.0534 | 0.0154 | 7.89E-06 | 13800 |
|  | rs6753743 | 2 | 121962947 | T | G | 0.0941 | 0.0185 | 0.8733 | 3.65E-07 | 14287 |
|  | rs3811621 | 2 | 190446541 | C | G | 0.1163 | 0.0131 | 0.2897 | 6.82E-19 | 14286 |
|  | rs9859317 | 3 | 64683150 | A | G | -0.0549 | 0.0124 | 0.3489 | 9.54E-06 | 14287 |
|  | rs794007 | 4 | 40142535 | T | C | 0.0685 | 0.0144 | 0.2406 | 1.97E-06 | 12844 |
|  | rs62307586 | 4 | 61224008 | T | G | -0.0552 | 0.0124 | 0.2859 | 8.52E-06 | 14735 |
|  | rs11930290 | 4 | 124325226 | A | T | -0.1845 | 0.0389 | 0.9748 | 2.11E-06 | 12848 |
|  | rs4958089 | 5 | 132484429 | T | C | 0.0513 | 0.0115 | 0.4217 | 8.16E-06 | 14735 |
|  | rs34551523 | 5 | 146629145 | A | G | 0.1704 | 0.0348 | 0.0295 | 9.75E-07 | 14730 |
|  | rs33921462 | 5 | 176814656 | A | G | -0.0653 | 0.0135 | 0.2766 | 1.32E-06 | 14287 |
|  | rs181409232 | 6 | 31175118 | A | C | -0.2224 | 0.0479 | 0.0191 | 3.43E-06 | 12846 |
|  | rs12195292 | 6 | 81343345 | A | G | -0.0768 | 0.0166 | 0.8634 | 3.72E-06 | 14710 |
|  | rs370203222 | 7 | 12540548 | C | G | 0.1059 | 0.0222 | 0.1002 | 1.84E-06 | 11759 |
|  | rs4730018 | 7 | 104141309 | A | C | 0.0936 | 0.0206 | 0.9039 | 5.53E-06 | 14287 |
|  | rs117963256 | 8 | 19386357 | A | T | -0.291 | 0.0653 | 0.0117 | 8.34E-06 | 12304 |
|  | rs118177380 | 11 | 106531034 | A | G | 0.2362 | 0.0507 | 0.0187 | 3.18E-06 | 14287 |
|  | rs57479285 | 11 | 111082782 | T | C | 0.4343 | 0.0981 | 0.0113 | 9.55E-06 | 7424 |
|  | rs75271339 | 11 | 115871001 | A | T | -0.415 | 0.089 | 0.0141 | 3.12E-06 | 9947 |
|  | rs145628723 | 13 | 103962376 | A | G | 0.1957 | 0.0437 | 0.022 | 7.53E-06 | 14238 |
|  | rs11150539 | 16 | 83137317 | T | C | -0.056 | 0.0119 | 0.4938 | 2.53E-06 | 14287 |
|  | rs9950230 | 18 | 56254394 | C | G | -0.0559 | 0.0123 | 0.7034 | 5.50E-06 | 14726 |
|  | rs6127099 | 20 | 52731402 | A | T | 0.0907 | 0.0134 | 0.706 | 1.30E-11 | 14287 |
|  | rs2825747 | 21 | 21059864 | A | G | 0.0693 | 0.0153 | 0.8386 | 5.91E-06 | 14735 |
|  | rs368215473 | 22 | 18002038 | A | G | 0.1228 | 0.0257 | 0.0941 | 1.77E-06 | 9397 |
|  | rs35590112 | 22 | 22238107 | T | C | 0.0803 | 0.0166 | 0.1851 | 1.32E-06 | 12386 |
| FGF-5 | rs9433760 | 1 | 101733215 | A | G | 0.1023 | 0.0224 | 0.0841 | 4.95E-06 | 11788 |
|  | rs4970834 | 1 | 109814880 | T | C | -0.0729 | 0.0155 | 0.2041 | 2.56E-06 | 11786 |
|  | rs11120686 | 1 | 216050785 | T | C | -0.0599 | 0.0127 | 0.6038 | 2.40E-06 | 11786 |
|  | rs11674185 | 2 | 45095097 | A | G | -0.0586 | 0.0128 | 0.364 | 4.69E-06 | 11787 |
|  | rs188323153 | 2 | 103579729 | T | C | -0.4381 | 0.0966 | 0.9875 | 5.75E-06 | 10360 |
|  | rs148332905 | 2 | 144875473 | T | C | 0.2883 | 0.0608 | 0.0166 | 2.12E-06 | 11341 |
|  | rs189846846 | 2 | 229871311 | T | C | -0.3042 | 0.0659 | 0.0146 | 3.91E-06 | 9980 |
|  | rs369482528 | 2 | 241614719 | A | G | -0.0988 | 0.0213 | 0.1585 | 3.51E-06 | 8813 |
|  | rs4326078 | 4 | 70058335 | A | C | -0.0728 | 0.015 | 0.2193 | 1.21E-06 | 11789 |
|  | rs1458038 | 4 | 81164723 | T | C | 0.5159 | 0.0126 | 0.3173 | 0.00E+00 | 11788 |
|  | rs3796605 | 4 | 81192363 | A | G | 0.1629 | 0.0283 | 0.9341 | 8.60E-09 | 11782 |
|  | rs200900616 | 4 | 81234749 | A | C | -0.6078 | 0.0627 | 0.0206 | 3.20E-22 | 7943 |
|  | rs11742072 | 5 | 122595513 | C | G | -0.1437 | 0.0315 | 0.9471 | 5.07E-06 | 11341 |
|  | rs305897 | 6 | 39710570 | T | C | 0.1185 | 0.0249 | 0.0828 | 1.95E-06 | 11341 |
|  | rs148797749 | 6 | 89057163 | A | G | -0.1705 | 0.0361 | 0.036 | 2.32E-06 | 11785 |
|  | rs150524893 | 8 | 106471400 | A | G | 0.2554 | 0.0552 | 0.9848 | 3.71E-06 | 11298 |
|  | rs77210320 | 8 | 143464103 | A | T | 0.1673 | 0.0356 | 0.9648 | 2.61E-06 | 11786 |
|  | rs1412253 | 9 | 1354486 | A | G | -0.0909 | 0.0192 | 0.8798 | 2.20E-06 | 11778 |
|  | rs117253850 | 9 | 7476426 | T | C | 0.2266 | 0.0453 | 0.0223 | 5.67E-07 | 11781 |
|  | rs2767000 | 9 | 113552214 | A | G | 0.1197 | 0.0263 | 0.9276 | 5.33E-06 | 11341 |
|  | rs12005489 | 9 | 133305614 | T | C | 0.1509 | 0.0322 | 0.0458 | 2.78E-06 | 10725 |
|  | rs112819708 | 10 | 4599492 | A | G | -0.1941 | 0.0435 | 0.9715 | 8.12E-06 | 11779 |
|  | rs10788164 | 10 | 123042845 | T | C | -0.0579 | 0.0131 | 0.3425 | 9.88E-06 | 11766 |
|  | rs4910278 | 11 | 11232260 | T | C | -0.0556 | 0.0124 | 0.4757 | 7.33E-06 | 11789 |
|  | rs146840556 | 11 | 15208397 | T | C | 0.1933 | 0.0434 | 0.0244 | 8.43E-06 | 11773 |
|  | rs624605 | 11 | 75754620 | T | G | 0.0619 | 0.0133 | 0.4196 | 3.25E-06 | 11341 |
|  | rs112790107 | 12 | 48680454 | A | G | 0.509 | 0.1062 | 0.006 | 1.64E-06 | 9845 |
|  | rs75924419 | 13 | 48055229 | A | G | 0.1613 | 0.0362 | 0.0312 | 8.36E-06 | 11788 |
|  | rs112371527 | 13 | 62077906 | A | T | 0.1018 | 0.02 | 0.8396 | 3.58E-07 | 8389 |
|  | rs12907074 | 15 | 52478472 | A | T | -0.0934 | 0.0207 | 0.1471 | 6.42E-06 | 9461 |
|  | rs56391958 | 16 | 62005709 | A | G | -0.1197 | 0.0249 | 0.0816 | 1.53E-06 | 11341 |
|  | rs192746810 | 18 | 14708913 | A | G | -0.1842 | 0.0395 | 0.0464 | 3.11E-06 | 11341 |
|  | rs62211039 | 21 | 24140005 | A | G | 0.3703 | 0.083 | 0.9894 | 8.14E-06 | 9358 |
| FIt3L | rs67811337 | 1 | 6546418 | A | C | 0.1083 | 0.024 | 0.081 | 6.41E-06 | 13222 |
|  | rs77584520 | 1 | 78494817 | A | T | 0.1313 | 0.0293 | 0.9568 | 7.42E-06 | 14731 |
|  | rs55633025 | 1 | 159890969 | A | G | 0.0725 | 0.0129 | 0.4738 | 1.91E-08 | 12404 |
|  | rs112538528 | 1 | 236377861 | A | C | -0.2426 | 0.0547 | 0.9835 | 9.20E-06 | 13305 |
|  | rs186975 | 2 | 8442736 | C | G | 0.0554 | 0.0119 | 0.382 | 3.23E-06 | 14703 |
|  | rs113877570 | 2 | 36270546 | A | G | -0.1207 | 0.0266 | 0.0513 | 5.69E-06 | 14729 |
|  | rs1866051 | 2 | 65602149 | T | C | -0.0746 | 0.0115 | 0.4771 | 8.76E-11 | 14732 |
|  | rs7624160 | 3 | 128381886 | T | G | -0.1061 | 0.0119 | 0.6093 | 4.84E-19 | 14719 |
|  | rs7625643 | 3 | 141150026 | A | G | 0.0611 | 0.0129 | 0.5605 | 2.18E-06 | 12404 |
|  | rs61792394 | 3 | 170851052 | A | G | -0.1569 | 0.0338 | 0.9624 | 3.45E-06 | 14286 |
|  | rs57080649 | 4 | 442232 | A | C | 0.0986 | 0.022 | 0.8422 | 7.40E-06 | 10894 |
|  | rs218265 | 4 | 55408999 | T | C | 0.0833 | 0.016 | 0.8361 | 1.93E-07 | 14731 |
|  | rs1277312 | 4 | 57902945 | A | T | 0.0539 | 0.0117 | 0.4587 | 4.09E-06 | 14719 |
|  | rs144317085 | 4 | 105806108 | A | T | 0.2206 | 0.0324 | 0.9647 | 9.85E-12 | 14722 |
|  | rs184038883 | 4 | 185989605 | A | C | -0.2276 | 0.0504 | 0.0242 | 6.31E-06 | 12790 |
|  | rs7726159 | 5 | 1282319 | A | C | -0.1191 | 0.0128 | 0.3326 | 1.34E-20 | 14733 |
|  | rs1379304 | 5 | 6289394 | T | C | 0.0603 | 0.0127 | 0.3741 | 2.05E-06 | 14286 |
|  | rs139721800 | 6 | 18758144 | T | C | 0.0959 | 0.0202 | 0.9089 | 2.06E-06 | 14715 |
|  | rs9381118 | 6 | 41985436 | T | C | 0.0643 | 0.0136 | 0.2577 | 2.27E-06 | 14285 |
|  | rs6453962 | 6 | 77907355 | T | C | -0.0559 | 0.0117 | 0.4699 | 1.77E-06 | 14732 |
|  | rs6925032 | 6 | 91008027 | A | C | 0.0676 | 0.0128 | 0.326 | 1.28E-07 | 14286 |
|  | rs11961049 | 6 | 155496339 | A | G | 0.1031 | 0.0232 | 0.0686 | 8.83E-06 | 14724 |
|  | rs28533776 | 7 | 3914756 | A | G | -0.0749 | 0.0147 | 0.1924 | 3.48E-07 | 14731 |
|  | rs117040659 | 8 | 29348124 | T | C | 0.3667 | 0.0815 | 0.0086 | 6.81E-06 | 14286 |
|  | rs192857133 | 9 | 243950 | T | C | 0.1812 | 0.0386 | 0.026 | 2.68E-06 | 14727 |
|  | rs35977476 | 9 | 9191493 | C | G | 0.1102 | 0.0244 | 0.0668 | 6.29E-06 | 14725 |
|  | rs57191857 | 9 | 10640144 | A | G | -0.1429 | 0.0299 | 0.9571 | 1.76E-06 | 14734 |
|  | rs12338549 | 9 | 94348687 | T | C | -0.0851 | 0.0186 | 0.1096 | 4.76E-06 | 14719 |
|  | rs55646869 | 9 | 129753010 | A | G | 0.0529 | 0.0116 | 0.4437 | 5.11E-06 | 14703 |
|  | rs185979634 | 10 | 101403323 | T | C | 0.1483 | 0.0306 | 0.9513 | 1.26E-06 | 12847 |
|  | rs4919613 | 10 | 103905779 | A | G | -0.0662 | 0.0148 | 0.1973 | 7.71E-06 | 14286 |
|  | rs74722523 | 10 | 105351013 | T | C | 0.3529 | 0.076 | 0.9914 | 3.43E-06 | 11437 |
|  | rs141117375 | 11 | 62294309 | T | C | -0.1519 | 0.0331 | 0.0364 | 4.45E-06 | 14286 |
|  | rs11212636 | 11 | 108311965 | A | G | -0.0772 | 0.0117 | 0.4171 | 4.16E-11 | 14723 |
|  | rs73000965 | 11 | 113982321 | A | T | 0.0588 | 0.0125 | 0.3111 | 2.55E-06 | 14731 |
|  | rs9554186 | 13 | 28455266 | A | T | 0.0821 | 0.0128 | 0.3602 | 1.42E-10 | 14286 |
|  | rs76428106 | 13 | 28604007 | T | C | -1.1019 | 0.0524 | 0.9832 | 3.59E-98 | 13799 |
|  | rs2325555 | 13 | 74312013 | A | G | -0.0653 | 0.0146 | 0.8002 | 7.73E-06 | 14720 |
|  | rs871051 | 13 | 95950210 | T | G | 0.1342 | 0.0301 | 0.0401 | 8.25E-06 | 14726 |
|  | rs72681653 | 14 | 51156734 | A | G | 0.1302 | 0.0266 | 0.9447 | 9.84E-07 | 14734 |
|  | rs78723322 | 14 | 87871738 | T | C | -0.1487 | 0.0329 | 0.0403 | 6.19E-06 | 14734 |
|  | rs35340580 | 16 | 1526820 | A | G | -0.1383 | 0.0303 | 0.0449 | 5.01E-06 | 13653 |
|  | rs78154669 | 16 | 81591243 | T | G | 0.0923 | 0.0207 | 0.0938 | 8.24E-06 | 14721 |
|  | rs10164255 | 18 | 70532840 | T | G | 0.0556 | 0.0121 | 0.408 | 4.33E-06 | 14729 |
|  | rs374309 | 19 | 22332298 | A | G | 0.0786 | 0.0174 | 0.8629 | 6.27E-06 | 14286 |
|  | rs17272847 | 19 | 49979398 | A | G | 0.1179 | 0.022 | 0.0907 | 8.36E-08 | 13222 |
|  | rs12609918 | 19 | 56755467 | A | G | -0.1863 | 0.0412 | 0.9451 | 6.13E-06 | 9453 |
|  | rs142870956 | 20 | 4261712 | T | C | -0.4307 | 0.0883 | 0.0075 | 1.07E-06 | 11952 |
| hGDNF | rs12739417 | 1 | 199872770 | A | G | 0.0579 | 0.0129 | 0.4066 | 7.18E-06 | 12404 |
|  | rs17353853 | 2 | 165822047 | A | G | 0.1022 | 0.0229 | 0.9255 | 8.09E-06 | 14288 |
|  | rs144181640 | 2 | 208746333 | A | T | 0.3366 | 0.0697 | 0.9898 | 1.37E-06 | 13988 |
|  | rs147483239 | 3 | 11787496 | T | C | -0.2148 | 0.0485 | 0.019 | 9.47E-06 | 14726 |
|  | rs62243355 | 3 | 36072052 | T | C | -0.0981 | 0.0196 | 0.0997 | 5.58E-07 | 14731 |
|  | rs11740708 | 5 | 37868088 | A | G | -0.3888 | 0.0198 | 0.0906 | 7.56E-86 | 14710 |
|  | rs2194229 | 5 | 37918839 | A | T | -0.1683 | 0.0179 | 0.1296 | 5.34E-21 | 14288 |
|  | rs2453338 | 5 | 38094871 | A | T | 0.0829 | 0.0124 | 0.352 | 2.30E-11 | 14734 |
|  | rs112594435 | 6 | 134688249 | T | C | 0.1531 | 0.0333 | 0.0371 | 4.27E-06 | 14721 |
|  | rs150807191 | 8 | 34985123 | A | G | -0.1719 | 0.0387 | 0.9717 | 8.92E-06 | 14725 |
|  | rs2093446 | 9 | 5230371 | C | G | 0.0606 | 0.0129 | 0.3 | 2.63E-06 | 14288 |
|  | rs9409461 | 9 | 94530989 | A | G | 0.0528 | 0.0119 | 0.4811 | 9.12E-06 | 14288 |
|  | rs72743928 | 9 | 98815653 | T | C | 0.0871 | 0.0192 | 0.1091 | 5.72E-06 | 14288 |
|  | rs10982156 | 9 | 117088064 | A | T | -0.1073 | 0.0236 | 0.0803 | 5.45E-06 | 14288 |
|  | rs117195704 | 10 | 61379709 | A | G | -0.1377 | 0.0309 | 0.0386 | 8.34E-06 | 14723 |
|  | rs77671113 | 10 | 87694408 | A | C | -0.1194 | 0.0208 | 0.0859 | 9.45E-09 | 14725 |
|  | rs138691533 | 12 | 98629454 | A | G | 0.2584 | 0.0539 | 0.0148 | 1.63E-06 | 14731 |
|  | rs57104839 | 13 | 28147359 | A | G | 0.0915 | 0.0196 | 0.903 | 3.04E-06 | 14736 |
|  | rs79953230 | 13 | 67293674 | T | C | 0.1918 | 0.042 | 0.0254 | 4.96E-06 | 14719 |
|  | rs77124379 | 16 | 48955894 | T | C | -0.1117 | 0.0245 | 0.9259 | 5.14E-06 | 14288 |
|  | rs35740453 | 17 | 1490782 | A | G | -0.0718 | 0.0154 | 0.7373 | 3.13E-06 | 13224 |
|  | rs16945776 | 17 | 12160288 | A | G | 0.0871 | 0.0196 | 0.9042 | 8.84E-06 | 14731 |
|  | rs9303102 | 17 | 14221290 | T | G | 0.2096 | 0.0453 | 0.0231 | 3.71E-06 | 12935 |
|  | rs55850217 | 19 | 14805280 | T | C | 0.0816 | 0.014 | 0.3119 | 5.59E-09 | 11755 |
|  | rs12329721 | 21 | 22101949 | A | G | 0.0862 | 0.0188 | 0.8974 | 4.54E-06 | 14733 |
|  | rs116928328 | 21 | 38984857 | T | C | 0.3727 | 0.0821 | 0.9927 | 5.64E-06 | 13801 |
| HGF | rs34219417 | 1 | 38949303 | T | C | 0.0992 | 0.0217 | 0.8959 | 4.84E-06 | 14286 |
|  | rs138848271 | 1 | 83864840 | T | C | -0.405 | 0.0832 | 0.9907 | 1.13E-06 | 10793 |
|  | rs114581807 | 1 | 190171653 | A | G | 0.2031 | 0.0452 | 0.0214 | 7.01E-06 | 14734 |
|  | rs111239033 | 2 | 43842712 | A | G | 0.1021 | 0.0228 | 0.0699 | 7.53E-06 | 14732 |
|  | rs6709159 | 2 | 108946178 | A | G | 0.0564 | 0.0114 | 0.4434 | 7.52E-07 | 14734 |
|  | rs2737756 | 3 | 128064791 | A | G | 0.2047 | 0.0391 | 0.0276 | 1.65E-07 | 14718 |
|  | rs59950280 | 4 | 3452345 | A | G | 0.1219 | 0.0131 | 0.328 | 1.34E-20 | 13222 |
|  | rs183596950 | 4 | 31819358 | A | G | -0.0699 | 0.0152 | 0.6438 | 4.25E-06 | 11538 |
|  | rs80308275 | 4 | 153539149 | T | C | -0.1177 | 0.026 | 0.0698 | 5.98E-06 | 14731 |
|  | rs189111993 | 5 | 175563330 | T | C | 0.266 | 0.0595 | 0.0183 | 7.80E-06 | 11382 |
|  | rs115009171 | 6 | 622484 | A | G | 0.4297 | 0.0934 | 0.0078 | 4.21E-06 | 10300 |
|  | rs201840950 | 6 | 32487091 | T | G | 0.0993 | 0.0215 | 0.7957 | 3.86E-06 | 10216 |
|  | rs79246709 | 7 | 19933976 | T | G | 0.1375 | 0.0305 | 0.0427 | 6.54E-06 | 14719 |
|  | rs5745692 | 7 | 81358266 | C | G | -0.1545 | 0.0332 | 0.964 | 3.26E-06 | 14716 |
|  | rs5745687 | 7 | 81359051 | T | C | -0.1943 | 0.0242 | 0.0608 | 9.83E-16 | 14733 |
|  | rs1963492 | 8 | 2653987 | T | C | -0.0749 | 0.0168 | 0.1917 | 8.26E-06 | 11335 |
|  | rs113903858 | 8 | 72362254 | A | G | 0.3666 | 0.0747 | 0.014 | 9.22E-07 | 11137 |
|  | rs5007363 | 9 | 107570128 | T | C | 0.0736 | 0.0165 | 0.7969 | 8.17E-06 | 11751 |
|  | rs74130123 | 10 | 30042437 | T | C | -0.2429 | 0.0489 | 0.9809 | 6.79E-07 | 14734 |
|  | rs61897389 | 11 | 47380087 | T | G | -0.0761 | 0.0171 | 0.1322 | 8.58E-06 | 14723 |
|  | rs4963955 | 12 | 26274153 | T | C | 0.1359 | 0.0307 | 0.9623 | 9.57E-06 | 14722 |
|  | rs144638564 | 12 | 114913966 | A | G | -0.3746 | 0.0839 | 0.0114 | 8.01E-06 | 12031 |
|  | rs145015846 | 14 | 24292539 | T | C | 0.2333 | 0.0525 | 0.0213 | 8.84E-06 | 14286 |
|  | rs57193877 | 14 | 65654346 | T | C | 0.1572 | 0.0311 | 0.9611 | 4.31E-07 | 14725 |
|  | rs11631440 | 15 | 101649383 | A | T | -0.0638 | 0.012 | 0.6021 | 1.06E-07 | 14709 |
|  | rs72778371 | 16 | 57045498 | A | G | -0.0717 | 0.0161 | 0.2021 | 8.45E-06 | 12789 |
|  | rs4290523 | 17 | 7014969 | T | C | -0.0992 | 0.0217 | 0.0942 | 4.84E-06 | 12846 |
|  | rs1986719 | 17 | 45959996 | T | C | -0.1954 | 0.039 | 0.9572 | 5.44E-07 | 14718 |
|  | rs150498795 | 18 | 76506666 | A | T | 0.1566 | 0.0327 | 0.9642 | 1.68E-06 | 14732 |
|  | rs851612 | 19 | 42769693 | A | G | -0.1182 | 0.0257 | 0.9405 | 4.24E-06 | 14286 |
|  | rs8106345 | 19 | 58888317 | A | G | 0.0592 | 0.0127 | 0.5179 | 3.14E-06 | 12404 |
|  | rs2281532 | 20 | 6103587 | T | C | -0.0698 | 0.0151 | 0.7983 | 3.79E-06 | 14286 |
|  | rs739269 | 22 | 38212068 | T | C | -0.0556 | 0.0125 | 0.6561 | 8.67E-06 | 14286 |
| IFN-γ | rs12408527 | 1 | 6305053 | T | C | -0.0663 | 0.015 | 0.6507 | 9.87E-06 | 10279 |
|  | rs80064136 | 1 | 46963687 | A | G | 0.2305 | 0.0521 | 0.9818 | 9.68E-06 | 11791 |
|  | rs67101138 | 2 | 19234534 | T | G | 0.1461 | 0.0307 | 0.9482 | 1.95E-06 | 11793 |
|  | rs6442763 | 3 | 2921515 | A | C | 0.0648 | 0.014 | 0.4253 | 3.68E-06 | 11345 |
|  | rs1392570 | 3 | 20774445 | A | G | -0.0627 | 0.0139 | 0.6157 | 6.46E-06 | 11345 |
|  | rs34658335 | 7 | 10535996 | T | C | 0.1163 | 0.0261 | 0.0793 | 8.35E-06 | 11345 |
|  | rs7909512 | 10 | 3683786 | A | G | 0.0567 | 0.0128 | 0.5207 | 9.44E-06 | 11775 |
|  | rs61863416 | 10 | 130545142 | A | G | 0.136 | 0.0283 | 0.9427 | 1.54E-06 | 11774 |
|  | rs140562194 | 11 | 110972327 | T | C | 0.4666 | 0.0897 | 0.0122 | 1.97E-07 | 7396 |
|  | rs61904946 | 11 | 116241126 | T | G | 0.1257 | 0.0282 | 0.0613 | 8.29E-06 | 11792 |
|  | rs74903999 | 13 | 27473585 | T | C | 0.3485 | 0.0708 | 0.9882 | 8.55E-07 | 10558 |
|  | rs146527076 | 13 | 90334795 | C | G | 0.1935 | 0.0384 | 0.9683 | 4.68E-07 | 11781 |
|  | rs1953463 | 14 | 44797800 | A | G | -0.0801 | 0.0176 | 0.8282 | 5.34E-06 | 11345 |
|  | rs78042482 | 15 | 58694863 | A | G | 0.2688 | 0.0606 | 0.0145 | 9.18E-06 | 10890 |
|  | rs74245341 | 15 | 93585096 | A | C | 0.2414 | 0.0533 | 0.0232 | 5.92E-06 | 10720 |
|  | rs704 | 17 | 26694861 | A | G | -0.0635 | 0.0127 | 0.4705 | 5.73E-07 | 11793 |
|  | rs4810686 | 20 | 46408844 | T | C | 0.0671 | 0.0137 | 0.5585 | 9.69E-07 | 11344 |
|  | rs62230781 | 21 | 37335521 | A | G | 0.2184 | 0.0403 | 0.0412 | 5.98E-08 | 11345 |
| IL-10 | rs4655953 | 1 | 87832695 | A | G | 0.0565 | 0.0121 | 0.3683 | 3.02E-06 | 14722 |
|  | rs12123181 | 1 | 206954566 | A | G | -0.0996 | 0.0151 | 0.2042 | 4.22E-11 | 14296 |
|  | rs202092984 | 2 | 87276729 | A | G | 0.1563 | 0.0341 | 0.0498 | 4.57E-06 | 11768 |
|  | rs138203589 | 2 | 125939274 | T | G | 0.1937 | 0.0418 | 0.0455 | 3.59E-06 | 14730 |
|  | rs3769274 | 2 | 173757986 | A | G | 0.0771 | 0.0162 | 0.8403 | 1.94E-06 | 14742 |
|  | rs188535859 | 2 | 201728383 | T | C | -0.2351 | 0.0486 | 0.0174 | 1.32E-06 | 14741 |
|  | rs398518 | 3 | 8954959 | A | G | -0.1293 | 0.0218 | 0.9146 | 3.01E-09 | 14296 |
|  | rs1370717 | 3 | 32354387 | A | G | -0.0534 | 0.0118 | 0.4388 | 6.03E-06 | 14730 |
|  | rs73226323 | 3 | 111531258 | T | C | 0.1847 | 0.0415 | 0.9755 | 8.56E-06 | 14738 |
|  | rs28651780 | 4 | 68058232 | T | C | -0.0746 | 0.0167 | 0.8437 | 7.93E-06 | 14296 |
|  | rs28419759 | 4 | 78929167 | A | C | -0.0542 | 0.0121 | 0.5556 | 7.49E-06 | 13851 |
|  | rs72701782 | 4 | 185304872 | A | G | 0.0981 | 0.0211 | 0.0999 | 3.33E-06 | 14296 |
|  | rs10067443 | 5 | 19244332 | T | C | -0.0753 | 0.0149 | 0.8011 | 4.33E-07 | 14296 |
|  | rs116018601 | 5 | 132022975 | T | C | 0.1763 | 0.0398 | 0.9715 | 9.44E-06 | 14727 |
|  | rs385697 | 6 | 7880027 | T | C | 0.0555 | 0.0119 | 0.6126 | 3.10E-06 | 14727 |
|  | rs17654493 | 6 | 18825879 | T | C | 0.2951 | 0.0626 | 0.014 | 2.43E-06 | 12313 |
|  | rs28377109 | 6 | 32434716 | A | C | 0.1586 | 0.02 | 0.1118 | 2.19E-15 | 13383 |
|  | rs67037615 | 6 | 32453163 | T | C | -0.1528 | 0.0196 | 0.8893 | 6.39E-15 | 14296 |
|  | rs150408090 | 6 | 122709508 | A | G | 0.3666 | 0.0761 | 0.9898 | 1.45E-06 | 12313 |
|  | rs389239 | 6 | 169465075 | A | G | -0.0574 | 0.0125 | 0.6162 | 4.39E-06 | 14296 |
|  | rs148332061 | 7 | 590757 | C | G | 0.1112 | 0.0239 | 0.1005 | 3.28E-06 | 13232 |
|  | rs34059323 | 7 | 148496619 | A | G | -0.1789 | 0.0401 | 0.0275 | 8.14E-06 | 14744 |
|  | rs66624860 | 8 | 61861039 | T | C | 0.0681 | 0.0143 | 0.2791 | 1.91E-06 | 12799 |
|  | rs79438518 | 9 | 18625666 | A | T | -0.2845 | 0.0618 | 0.0122 | 4.15E-06 | 13809 |
|  | rs3135932 | 11 | 117864063 | A | G | -0.1597 | 0.0154 | 0.823 | 3.39E-25 | 14744 |
|  | rs147027610 | 13 | 52566577 | T | C | 0.1876 | 0.0416 | 0.9747 | 6.49E-06 | 14250 |
|  | rs72661933 | 13 | 113612154 | A | C | -0.2388 | 0.0535 | 0.9811 | 8.06E-06 | 13809 |
|  | rs76932862 | 14 | 20318041 | T | C | 0.1401 | 0.0291 | 0.1028 | 1.48E-06 | 12412 |
|  | rs35898626 | 14 | 83509406 | C | G | -0.0519 | 0.0117 | 0.4552 | 9.17E-06 | 14729 |
|  | rs28929474 | 14 | 94844947 | T | C | 0.2027 | 0.0451 | 0.0203 | 6.97E-06 | 14744 |
|  | rs17632797 | 15 | 95207832 | A | G | -0.0772 | 0.0173 | 0.8561 | 8.10E-06 | 14296 |
|  | rs7500869 | 16 | 11009587 | A | C | -0.0714 | 0.0159 | 0.1848 | 7.10E-06 | 14743 |
|  | rs17747934 | 20 | 60272196 | A | G | -0.1551 | 0.0342 | 0.9375 | 5.76E-06 | 13232 |
|  | rs9975090 | 21 | 43024340 | A | G | 0.0551 | 0.0123 | 0.5972 | 7.48E-06 | 14296 |
| IL-10 α | rs6670799 | 1 | 56566352 | A | G | -0.1247 | 0.0252 | 0.0839 | 7.48E-07 | 11345 |
|  | rs12026492 | 1 | 226676794 | T | C | 0.0709 | 0.0156 | 0.2527 | 5.50E-06 | 11345 |
|  | rs28408877 | 1 | 247347252 | C | G | -0.069 | 0.0153 | 0.7429 | 6.49E-06 | 11345 |
|  | rs62262707 | 3 | 83110489 | A | C | 0.2156 | 0.0483 | 0.0253 | 8.05E-06 | 11345 |
|  | rs41272625 | 3 | 158540291 | T | G | 0.082 | 0.0179 | 0.1526 | 4.63E-06 | 11784 |
|  | rs286605 | 5 | 154443639 | T | G | 0.0623 | 0.0131 | 0.5891 | 1.98E-06 | 11793 |
|  | rs9472032 | 6 | 43294041 | A | C | 0.0968 | 0.0214 | 0.1006 | 6.09E-06 | 11793 |
|  | rs180893431 | 6 | 78703747 | A | C | 0.4057 | 0.0865 | 0.9896 | 2.73E-06 | 9340 |
|  | rs492616 | 6 | 116712455 | A | G | -0.0835 | 0.0189 | 0.8497 | 9.96E-06 | 11344 |
|  | rs3777726 | 6 | 167359279 | T | C | -0.075 | 0.0165 | 0.2995 | 5.48E-06 | 9461 |
|  | rs10091841 | 8 | 9104264 | T | G | 0.064 | 0.0135 | 0.4945 | 2.13E-06 | 11345 |
|  | rs79202194 | 8 | 27864161 | T | C | -0.0993 | 0.0224 | 0.1001 | 9.29E-06 | 11792 |
|  | rs958402 | 8 | 97437587 | T | C | 0.06 | 0.0134 | 0.4233 | 7.55E-06 | 11345 |
|  | rs80137559 | 10 | 18335103 | T | C | 0.2347 | 0.0511 | 0.9822 | 4.37E-06 | 11791 |
|  | rs3135932 | 11 | 117864063 | A | G | -0.0844 | 0.0168 | 0.8171 | 5.07E-07 | 11793 |
|  | rs9548767 | 13 | 31511360 | T | G | -0.0686 | 0.0155 | 0.2251 | 9.61E-06 | 11781 |
|  | rs17832137 | 14 | 55005535 | A | G | 0.1676 | 0.0343 | 0.0481 | 1.03E-06 | 10289 |
|  | rs74719858 | 14 | 66407688 | A | G | 0.3915 | 0.0823 | 0.9853 | 1.97E-06 | 10858 |
|  | rs1406714 | 15 | 93461371 | C | G | 0.0623 | 0.0131 | 0.4267 | 1.98E-06 | 11783 |
|  | rs143313574 | 16 | 5247086 | T | C | -0.3868 | 0.0841 | 0.0107 | 4.24E-06 | 9956 |
|  | rs201449583 | 18 | 9085066 | A | T | -0.0782 | 0.0172 | 0.5926 | 5.45E-06 | 7943 |
|  | rs1377036 | 20 | 12662915 | A | G | -0.0791 | 0.0179 | 0.8076 | 9.92E-06 | 9907 |
| IL-10 β | rs142421172 | 1 | 179682087 | A | G | 0.208 | 0.031 | 0.0438 | 1.95E-11 | 12840 |
|  | rs114288486 | 1 | 212796908 | T | C | 0.1449 | 0.0311 | 0.0363 | 3.17E-06 | 14730 |
|  | rs12087642 | 1 | 216769382 | A | G | -0.0533 | 0.0118 | 0.3491 | 6.27E-06 | 14719 |
|  | rs112798338 | 1 | 228433772 | T | C | 0.1926 | 0.0411 | 0.026 | 2.78E-06 | 14286 |
|  | rs7574167 | 2 | 43150924 | A | G | 0.0637 | 0.0135 | 0.2684 | 2.38E-06 | 12832 |
|  | rs116315732 | 2 | 176630651 | T | C | 0.1988 | 0.0402 | 0.0212 | 7.60E-07 | 14734 |
|  | rs1153572 | 3 | 4275803 | T | C | -0.0548 | 0.0119 | 0.5866 | 4.12E-06 | 14286 |
|  | rs4686528 | 3 | 189486023 | A | G | -0.1435 | 0.0317 | 0.9488 | 5.99E-06 | 14286 |
|  | rs186756775 | 4 | 40034728 | A | C | -0.0632 | 0.0142 | 0.4693 | 8.56E-06 | 11538 |
|  | rs9685374 | 4 | 119751261 | A | G | -0.0618 | 0.0134 | 0.2721 | 3.99E-06 | 14286 |
|  | rs115242284 | 4 | 183615376 | T | C | -0.1662 | 0.0373 | 0.027 | 8.36E-06 | 14724 |
|  | rs11731108 | 4 | 185140403 | C | G | -0.0592 | 0.0132 | 0.7134 | 7.30E-06 | 14286 |
|  | rs34361138 | 6 | 41174158 | A | G | 0.0532 | 0.0113 | 0.5488 | 2.50E-06 | 14732 |
|  | rs16887395 | 6 | 55785369 | A | G | -0.0547 | 0.012 | 0.6068 | 5.16E-06 | 14286 |
|  | rs117092856 | 6 | 150298636 | A | G | -0.3461 | 0.0783 | 0.0088 | 9.86E-06 | 13420 |
|  | rs111850948 | 7 | 25740142 | A | G | -0.0843 | 0.0181 | 0.1348 | 3.20E-06 | 14286 |
|  | rs7815666 | 8 | 18274205 | T | C | 0.0512 | 0.0113 | 0.5351 | 5.87E-06 | 14732 |
|  | rs116898411 | 8 | 128868102 | A | G | -0.1593 | 0.0349 | 0.96 | 5.01E-06 | 14286 |
|  | rs7855466 | 9 | 136121303 | T | C | 0.0861 | 0.0169 | 0.1723 | 3.49E-07 | 11783 |
|  | rs192325200 | 10 | 71477178 | A | G | -0.2327 | 0.0505 | 0.9827 | 4.07E-06 | 13867 |
|  | rs6584485 | 10 | 103937244 | T | C | 0.0743 | 0.0161 | 0.8362 | 3.93E-06 | 14286 |
|  | rs145144288 | 10 | 121606887 | T | G | -0.3019 | 0.0671 | 0.9879 | 6.82E-06 | 14286 |
|  | rs1605333 | 12 | 58388232 | C | G | -0.0601 | 0.0124 | 0.6484 | 1.25E-06 | 13237 |
|  | rs11110586 | 12 | 101321963 | A | G | 0.0667 | 0.0151 | 0.1973 | 1.00E-05 | 12846 |
|  | rs55780441 | 16 | 13209083 | T | C | 0.1783 | 0.0348 | 0.9674 | 3.00E-07 | 14734 |
|  | rs8064909 | 17 | 74093726 | T | C | -0.0608 | 0.0135 | 0.4413 | 6.68E-06 | 10281 |
|  | rs117883662 | 19 | 49553051 | C | G | -0.4295 | 0.0907 | 0.9904 | 2.19E-06 | 8938 |
|  | rs149156511 | 20 | 39437913 | A | G | 0.2066 | 0.0444 | 0.0323 | 3.27E-06 | 14286 |
|  | rs75710908 | 21 | 34510084 | A | G | 0.1586 | 0.0341 | 0.9665 | 3.30E-06 | 14708 |
|  | rs6517157 | 21 | 34634808 | T | C | 0.1509 | 0.0251 | 0.9258 | 1.83E-09 | 14286 |
|  | rs2266590 | 21 | 34659396 | A | G | -0.4105 | 0.0108 | 0.5426 | 4.06000000776829e-316 | 14714 |
| IL12 β | rs6750065 | 2 | 64889908 | T | C | 0.0628 | 0.0134 | 0.7333 | 2.78E-06 | 14286 |
|  | rs149993822 | 2 | 112046960 | T | C | 0.0984 | 0.0194 | 0.1662 | 3.93E-07 | 14287 |
|  | rs201331149 | 2 | 180262342 | T | C | 0.0663 | 0.015 | 0.487 | 9.87E-06 | 10893 |
|  | rs11130215 | 3 | 5026008 | A | G | -0.1009 | 0.0151 | 0.8154 | 2.36E-11 | 14732 |
|  | rs77331970 | 3 | 23011632 | A | G | -0.1039 | 0.0229 | 0.0757 | 5.70E-06 | 14286 |
|  | rs34169305 | 3 | 141071293 | T | C | 0.0561 | 0.0126 | 0.3099 | 8.49E-06 | 14722 |
|  | rs148549227 | 3 | 171348710 | T | C | 0.1088 | 0.0228 | 0.0889 | 1.82E-06 | 12403 |
|  | rs9815073 | 3 | 188115682 | A | C | 0.2861 | 0.013 | 0.3539 | 2.43E-107 | 14287 |
|  | rs13095341 | 3 | 188238286 | T | C | 0.1904 | 0.0429 | 0.027 | 9.07E-06 | 14287 |
|  | rs3822030 | 4 | 987343 | T | G | 0.0751 | 0.0166 | 0.5622 | 6.07E-06 | 7424 |
|  | rs12510419 | 4 | 66148918 | A | G | 0.2664 | 0.0595 | 0.019 | 7.56E-06 | 11272 |
|  | rs76322730 | 4 | 96638784 | A | G | 0.1511 | 0.0335 | 0.0343 | 6.47E-06 | 14735 |
|  | rs5014495 | 4 | 190527932 | T | C | 0.207 | 0.0442 | 0.0363 | 2.82E-06 | 10208 |
|  | rs144850787 | 5 | 158693364 | T | G | 0.3161 | 0.056 | 0.9869 | 1.66E-08 | 14287 |
|  | rs4244437 | 5 | 158773117 | A | G | 0.4912 | 0.0121 | 0.6981 | 0.00E+00 | 14719 |
|  | rs17056751 | 5 | 158852200 | A | G | 0.1694 | 0.0325 | 0.9592 | 1.87E-07 | 14735 |
|  | rs17722688 | 5 | 158907737 | T | C | -0.0857 | 0.0131 | 0.7246 | 6.07E-11 | 14729 |
|  | rs3130510 | 6 | 31154493 | A | G | 0.1155 | 0.014 | 0.2275 | 1.58E-16 | 14735 |
|  | rs144411658 | 6 | 31305502 | T | G | -0.0658 | 0.0145 | 0.3108 | 5.68E-06 | 11327 |
|  | rs6968201 | 7 | 6408796 | T | G | -0.0622 | 0.0119 | 0.5425 | 1.72E-07 | 14287 |
|  | rs117888068 | 8 | 4597842 | T | C | 0.219 | 0.0472 | 0.9787 | 3.49E-06 | 11783 |
|  | rs10100578 | 8 | 106471906 | A | G | -0.0523 | 0.0117 | 0.5034 | 7.82E-06 | 14723 |
|  | rs150590615 | 9 | 19034087 | T | C | -0.5077 | 0.1141 | 0.0046 | 8.60E-06 | 10678 |
|  | rs17500902 | 10 | 56466912 | A | G | -0.0727 | 0.0164 | 0.8535 | 9.30E-06 | 14726 |
|  | rs780679 | 10 | 73114509 | T | C | -0.0695 | 0.0139 | 0.2389 | 5.73E-07 | 14729 |
|  | rs201503938 | 10 | 94215014 | A | C | -0.1064 | 0.0219 | 0.1117 | 1.18E-06 | 10893 |
|  | rs144811230 | 10 | 108564105 | A | G | 0.0787 | 0.0177 | 0.8682 | 8.74E-06 | 13869 |
|  | rs11039216 | 11 | 47406592 | T | C | 0.0588 | 0.0116 | 0.5207 | 4.00E-07 | 14730 |
|  | rs539598 | 11 | 96026074 | T | C | -0.0672 | 0.0129 | 0.2822 | 1.90E-07 | 14731 |
|  | rs72993671 | 11 | 115767583 | A | G | -0.204 | 0.0435 | 0.0216 | 2.74E-06 | 14246 |
|  | rs1398590 | 12 | 17314071 | A | G | -0.197 | 0.0363 | 0.0298 | 5.73E-08 | 14726 |
|  | rs705705 | 12 | 56435504 | C | G | 0.0785 | 0.0133 | 0.323 | 3.59E-09 | 12841 |
|  | rs3184504 | 12 | 111884608 | T | C | 0.1332 | 0.0131 | 0.4766 | 2.76E-24 | 11785 |
|  | rs516978 | 12 | 121197553 | C | G | 0.0721 | 0.0122 | 0.3473 | 3.42E-09 | 14725 |
|  | rs76428106 | 13 | 28604007 | T | C | -0.3406 | 0.0535 | 0.9832 | 1.94E-10 | 13800 |
|  | rs1950897 | 14 | 68760141 | T | C | 0.0798 | 0.0128 | 0.7151 | 4.54E-10 | 14735 |
|  | rs12588969 | 14 | 103230758 | C | G | -0.1297 | 0.0127 | 0.6875 | 1.74E-24 | 14287 |
|  | rs8026767 | 15 | 80260554 | A | G | -0.0743 | 0.0132 | 0.2595 | 1.81E-08 | 14734 |
|  | rs4783410 | 16 | 20634309 | A | G | -0.0874 | 0.0196 | 0.097 | 8.23E-06 | 14722 |
|  | rs9965402 | 18 | 26624891 | A | G | 0.0918 | 0.0201 | 0.9081 | 4.94E-06 | 14729 |
|  | rs73416208 | 22 | 37881211 | A | G | -0.1477 | 0.0246 | 0.0654 | 1.92E-09 | 14729 |
| IL-13 | rs10917637 | 1 | 162878651 | A | G | 0.0692 | 0.0136 | 0.3447 | 3.61E-07 | 11772 |
|  | rs181829077 | 1 | 210429399 | A | C | 0.448 | 0.0997 | 0.9937 | 7.01E-06 | 9956 |
|  | rs10752781 | 1 | 234425332 | T | G | 0.0595 | 0.0134 | 0.4904 | 8.98E-06 | 11344 |
|  | rs13021250 | 2 | 8361137 | C | G | 0.1112 | 0.0248 | 0.9154 | 7.33E-06 | 10915 |
|  | rs4535095 | 2 | 98857893 | T | C | 0.0594 | 0.0134 | 0.5305 | 9.30E-06 | 11344 |
|  | rs36038515 | 3 | 8912627 | A | G | 0.0892 | 0.0197 | 0.1389 | 5.96E-06 | 11344 |
|  | rs9881117 | 3 | 123702960 | A | G | -0.0656 | 0.0141 | 0.3094 | 3.28E-06 | 11791 |
|  | rs10446322 | 3 | 143068250 | A | G | 0.064 | 0.0132 | 0.3961 | 1.24E-06 | 11777 |
|  | rs141538472 | 3 | 162592726 | T | C | 0.3156 | 0.0692 | 0.9803 | 5.10E-06 | 9093 |
|  | rs13119159 | 4 | 40938582 | A | G | 0.0739 | 0.0132 | 0.4958 | 2.16E-08 | 11344 |
|  | rs183684201 | 5 | 9974643 | C | G | -0.2968 | 0.0628 | 0.9858 | 2.29E-06 | 11782 |
|  | rs4260678 | 5 | 33630839 | A | G | 0.0713 | 0.016 | 0.7649 | 8.34E-06 | 11786 |
|  | rs74647203 | 6 | 16610802 | A | G | -0.2845 | 0.0629 | 0.0136 | 6.10E-06 | 11792 |
|  | rs11757926 | 6 | 41584211 | C | G | 0.1458 | 0.0327 | 0.9259 | 8.25E-06 | 7943 |
|  | rs1472639 | 6 | 55271616 | A | C | 0.0677 | 0.015 | 0.2405 | 6.38E-06 | 11790 |
|  | rs2485365 | 6 | 159512373 | A | G | 0.0579 | 0.0129 | 0.4703 | 7.18E-06 | 11776 |
|  | rs149979178 | 7 | 61999529 | A | G | 0.1832 | 0.039 | 0.9229 | 2.63E-06 | 6901 |
|  | rs76219628 | 7 | 154418309 | A | G | 0.169 | 0.0357 | 0.0397 | 2.20E-06 | 11344 |
|  | rs2073696 | 8 | 17271300 | A | G | -0.0992 | 0.0219 | 0.112 | 5.91E-06 | 11782 |
|  | rs909357 | 8 | 133902602 | A | G | -0.1099 | 0.0247 | 0.0772 | 8.61E-06 | 11777 |
|  | rs13297881 | 9 | 4934758 | T | G | 0.1562 | 0.035 | 0.9494 | 8.09E-06 | 11344 |
|  | rs11251952 | 10 | 3623961 | A | G | -0.1845 | 0.0397 | 0.0751 | 3.36E-06 | 11344 |
|  | rs12146346 | 10 | 53942324 | T | G | 0.0734 | 0.0155 | 0.3902 | 2.19E-06 | 8388 |
|  | rs36107074 | 11 | 11427220 | T | C | -0.0706 | 0.0159 | 0.2438 | 8.99E-06 | 11343 |
|  | rs10893042 | 11 | 123332970 | C | G | -0.0702 | 0.015 | 0.6744 | 2.87E-06 | 11344 |
|  | rs141726773 | 13 | 41810092 | T | C | 0.2093 | 0.0446 | 0.9682 | 2.69E-06 | 11344 |
|  | rs55924857 | 15 | 57830694 | T | C | -0.0934 | 0.0183 | 0.8535 | 3.33E-07 | 11792 |
|  | rs59235092 | 16 | 79977921 | A | G | 0.2122 | 0.0439 | 0.0274 | 1.34E-06 | 11784 |
|  | rs72477067 | 17 | 1645221 | T | C | 0.0999 | 0.0226 | 0.6877 | 9.85E-06 | 6879 |
|  | rs141637150 | 19 | 48502692 | T | C | 0.206 | 0.0466 | 0.0279 | 9.84E-06 | 11779 |
|  | rs3119485 | 21 | 14817382 | A | T | -0.1308 | 0.0293 | 0.094 | 8.04E-06 | 7523 |
|  | rs5756680 | 22 | 37842933 | T | C | 0.0848 | 0.0186 | 0.177 | 5.14E-06 | 9905 |
|  | rs117322078 | 22 | 37976432 | T | C | 0.2081 | 0.046 | 0.9743 | 6.07E-06 | 11781 |
| IL-15 α | rs79638163 | 1 | 235799305 | T | G | 0.176 | 0.0398 | 0.9701 | 9.77E-06 | 11792 |
|  | rs62144964 | 2 | 31652761 | A | G | 0.0852 | 0.019 | 0.1223 | 7.32E-06 | 11782 |
|  | rs6751509 | 2 | 52233445 | T | C | 0.0577 | 0.0128 | 0.3704 | 6.55E-06 | 11782 |
|  | rs75485361 | 3 | 174432353 | A | G | -0.07 | 0.0158 | 0.2414 | 9.41E-06 | 11343 |
|  | rs56240706 | 4 | 20169693 | A | T | 0.0919 | 0.0193 | 0.8082 | 1.92E-06 | 8861 |
|  | rs6845172 | 4 | 140657353 | C | G | 0.0778 | 0.0162 | 0.8203 | 1.57E-06 | 11772 |
|  | rs9270551 | 6 | 32560501 | A | G | 0.0749 | 0.0153 | 0.7077 | 9.81E-07 | 11344 |
|  | rs679874 | 6 | 153065996 | A | G | 0.0629 | 0.0139 | 0.7114 | 6.03E-06 | 11779 |
|  | rs17635883 | 7 | 51409710 | A | G | 0.0937 | 0.0211 | 0.122 | 8.96E-06 | 11344 |
|  | rs117428573 | 8 | 27696981 | A | C | -0.3132 | 0.0684 | 0.0119 | 4.67E-06 | 10857 |
|  | rs151041734 | 8 | 79506727 | T | C | -0.3825 | 0.0746 | 0.0139 | 2.94E-07 | 8297 |
|  | rs10125254 | 9 | 1673918 | A | C | 0.0651 | 0.0138 | 0.5937 | 2.39E-06 | 10856 |
|  | rs1004308 | 9 | 124977274 | T | C | 0.0727 | 0.0162 | 0.1931 | 7.20E-06 | 11783 |
|  | rs649129 | 9 | 136154304 | T | C | -0.0726 | 0.0158 | 0.2166 | 4.33E-06 | 10918 |
|  | rs12570881 | 10 | 5815194 | A | T | -0.0867 | 0.0174 | 0.8028 | 6.27E-07 | 11344 |
|  | rs2228059 | 10 | 6002368 | T | G | 0.3692 | 0.0127 | 0.5146 | 8.39E-186 | 11344 |
|  | rs7898286 | 10 | 6009462 | T | G | -0.1967 | 0.0135 | 0.3975 | 4.34E-48 | 11344 |
|  | rs145793996 | 11 | 37594094 | A | G | -0.2278 | 0.0492 | 0.9801 | 3.66E-06 | 11779 |
|  | rs17129907 | 11 | 97047987 | A | G | -0.1381 | 0.0303 | 0.9503 | 5.17E-06 | 11790 |
|  | rs142180961 | 12 | 38055500 | T | C | 0.2488 | 0.0553 | 0.0166 | 6.82E-06 | 10724 |
|  | rs7137828 | 12 | 111932800 | T | C | -0.0748 | 0.0133 | 0.5243 | 1.87E-08 | 11344 |
|  | rs4772056 | 13 | 98893318 | C | G | 0.0894 | 0.0197 | 0.8608 | 5.68E-06 | 11344 |
|  | rs117046642 | 15 | 94729209 | A | T | -0.0849 | 0.017 | 0.4367 | 5.91E-07 | 7943 |
|  | rs4459616 | 18 | 12175379 | T | C | 0.14 | 0.0294 | 0.0658 | 1.92E-06 | 9899 |
|  | rs140982443 | 19 | 14884642 | T | C | 0.1794 | 0.0395 | 0.963 | 5.58E-06 | 9906 |
|  | rs200873126 | 19 | 35402241 | A | G | -0.1 | 0.0226 | 0.8163 | 9.65E-06 | 7942 |
|  | rs192538756 | 20 | 2629394 | A | T | 0.1159 | 0.0241 | 0.8743 | 1.52E-06 | 9461 |
|  | rs117484888 | 21 | 15303777 | A | G | 0.3794 | 0.0799 | 0.0158 | 2.05E-06 | 7423 |
|  | rs2836464 | 21 | 39896592 | A | G | 0.0796 | 0.0169 | 0.8384 | 2.48E-06 | 11791 |
| IL-17A | rs33998516 | 1 | 96622380 | A | C | -0.0802 | 0.0164 | 0.7781 | 1.01E-06 | 11336 |
|  | rs75788494 | 2 | 13468626 | T | C | -0.2432 | 0.0525 | 0.021 | 3.61E-06 | 10574 |
|  | rs13028360 | 2 | 193404884 | A | T | 0.0597 | 0.0135 | 0.4446 | 9.77E-06 | 11336 |
|  | rs115193412 | 3 | 63484616 | T | C | 0.1688 | 0.0349 | 0.0418 | 1.32E-06 | 11778 |
|  | rs13092332 | 3 | 163713211 | T | C | 0.1668 | 0.0373 | 0.0383 | 7.75E-06 | 11776 |
|  | rs17448036 | 3 | 189579511 | A | G | -0.0585 | 0.0128 | 0.5609 | 4.87E-06 | 11783 |
|  | rs10024009 | 4 | 108605133 | A | C | -0.1038 | 0.0234 | 0.1174 | 9.17E-06 | 10274 |
|  | rs2731803 | 5 | 17221637 | T | C | -0.0713 | 0.0161 | 0.3974 | 9.49E-06 | 11782 |
|  | rs41270871 | 6 | 32628224 | A | T | 0.0923 | 0.0189 | 0.221 | 1.04E-06 | 8742 |
|  | rs28852438 | 7 | 6380998 | A | G | -0.068 | 0.0148 | 0.2897 | 4.34E-06 | 11336 |
|  | rs146730540 | 7 | 158133754 | T | C | -0.0903 | 0.0202 | 0.7451 | 7.81E-06 | 7943 |
|  | rs56702825 | 8 | 504034 | A | G | 0.1383 | 0.0303 | 0.9451 | 5.01E-06 | 11766 |
|  | rs1462017 | 8 | 70106392 | C | G | -0.0893 | 0.017 | 0.1776 | 1.50E-07 | 11774 |
|  | rs145734140 | 8 | 82998811 | A | G | -0.3662 | 0.0815 | 0.985 | 7.01E-06 | 8187 |
|  | rs77529534 | 9 | 27215497 | T | C | -0.1279 | 0.0272 | 0.9323 | 2.57E-06 | 11775 |
|  | rs79982350 | 9 | 31560030 | C | G | -0.1467 | 0.0295 | 0.0555 | 6.60E-07 | 11336 |
|  | rs4877075 | 9 | 91976386 | A | G | 0.0908 | 0.0192 | 0.1411 | 2.25E-06 | 11336 |
|  | rs34557923 | 10 | 67955765 | T | G | 0.2446 | 0.0517 | 0.9819 | 2.23E-06 | 10913 |
|  | rs3895928 | 12 | 89686567 | T | C | -0.1893 | 0.0418 | 0.9682 | 5.93E-06 | 11783 |
|  | rs77595989 | 12 | 118130693 | T | G | 0.1696 | 0.0371 | 0.9648 | 4.84E-06 | 11767 |
|  | rs57876893 | 14 | 23580861 | T | G | 0.1915 | 0.043 | 0.0279 | 8.45E-06 | 11776 |
|  | rs12599620 | 16 | 8011846 | T | C | -0.0726 | 0.0153 | 0.7391 | 2.08E-06 | 11336 |
|  | rs4985487 | 16 | 70109850 | T | G | -0.0615 | 0.0138 | 0.6045 | 8.33E-06 | 11336 |
|  | rs2429911 | 17 | 2272057 | T | C | 0.2309 | 0.0486 | 0.0251 | 2.02E-06 | 11294 |
|  | rs2693361 | 17 | 43658721 | T | G | 0.0884 | 0.0185 | 0.517 | 1.77E-06 | 7943 |
|  | rs146575213 | 19 | 16896917 | A | T | -0.4006 | 0.0904 | 0.0103 | 9.36E-06 | 8776 |
| IL-17C | rs114882743 | 1 | 176930254 | A | T | -0.3259 | 0.0548 | 0.9804 | 2.73E-09 | 11345 |
|  | rs12092228 | 1 | 242536005 | T | G | 0.1508 | 0.0327 | 0.0512 | 4.00E-06 | 9892 |
|  | rs139637682 | 2 | 111999407 | T | C | -0.3301 | 0.0737 | 0.9817 | 7.50E-06 | 8488 |
|  | rs2584966 | 2 | 117363487 | T | C | 0.0625 | 0.0139 | 0.4639 | 6.91E-06 | 9906 |
|  | rs77654242 | 2 | 136675328 | T | G | -0.2174 | 0.0469 | 0.9707 | 3.56E-06 | 10294 |
|  | rs7559734 | 2 | 212465664 | T | C | -0.0915 | 0.0206 | 0.8803 | 8.92E-06 | 11345 |
|  | rs149756404 | 2 | 239715033 | A | C | -0.2749 | 0.0524 | 0.9809 | 1.55E-07 | 11302 |
|  | rs113334480 | 3 | 184205438 | T | C | -0.0962 | 0.0217 | 0.8992 | 9.29E-06 | 11788 |
|  | rs1072074 | 4 | 24758319 | A | G | -0.0649 | 0.0141 | 0.5847 | 4.17E-06 | 11785 |
|  | rs11931996 | 4 | 152291909 | T | C | -0.1219 | 0.0262 | 0.9068 | 3.28E-06 | 11345 |
|  | rs6874610 | 5 | 39436460 | T | G | -0.0649 | 0.0144 | 0.2795 | 6.58E-06 | 11783 |
|  | rs59356989 | 5 | 107423766 | A | G | -0.0882 | 0.0177 | 0.8432 | 6.26E-07 | 11790 |
|  | rs2408771 | 5 | 126053775 | T | G | 0.0625 | 0.0138 | 0.4358 | 5.93E-06 | 11345 |
|  | rs75246422 | 5 | 174179760 | T | C | -0.0951 | 0.0201 | 0.1233 | 2.23E-06 | 11793 |
|  | rs72815428 | 5 | 174836306 | T | C | -0.1673 | 0.0366 | 0.0423 | 4.85E-06 | 11775 |
|  | rs2814092 | 6 | 66547346 | C | G | 0.0624 | 0.0135 | 0.6437 | 3.80E-06 | 11792 |
|  | rs2770113 | 6 | 158194819 | A | G | 0.0604 | 0.0135 | 0.4998 | 7.67E-06 | 11345 |
|  | rs1920106 | 7 | 21240100 | T | C | 0.0633 | 0.0132 | 0.3948 | 1.62E-06 | 11792 |
|  | rs75360344 | 7 | 35414652 | A | G | -0.1414 | 0.031 | 0.0498 | 5.08E-06 | 11774 |
|  | rs78289993 | 8 | 14156196 | A | G | 0.1577 | 0.0351 | 0.961 | 7.03E-06 | 11785 |
|  | rs28552890 | 8 | 23691952 | A | T | -0.096 | 0.0215 | 0.8859 | 8.00E-06 | 11345 |
|  | rs113702571 | 8 | 118629302 | T | C | -0.2558 | 0.0564 | 0.9848 | 5.75E-06 | 11786 |
|  | rs79340054 | 10 | 19368604 | T | C | 0.138 | 0.0309 | 0.9508 | 7.97E-06 | 11780 |
|  | rs139152046 | 12 | 23245149 | T | G | -0.2421 | 0.0536 | 0.0211 | 6.28E-06 | 9422 |
|  | rs7324443 | 13 | 30065064 | C | G | 0.0637 | 0.014 | 0.3575 | 5.36E-06 | 11345 |
|  | rs10870773 | 13 | 44396409 | A | G | -0.0816 | 0.0171 | 0.8025 | 1.82E-06 | 11345 |
|  | rs12863903 | 13 | 96638651 | T | C | -0.0857 | 0.018 | 0.1597 | 1.93E-06 | 11345 |
|  | rs8015296 | 14 | 92763907 | C | G | -0.1162 | 0.0253 | 0.0991 | 4.37E-06 | 9461 |
|  | rs12895022 | 14 | 102333234 | T | C | 0.1091 | 0.0236 | 0.0906 | 3.78E-06 | 11774 |
|  | rs79599491 | 15 | 42484321 | T | C | -0.1905 | 0.0419 | 0.9711 | 5.45E-06 | 11306 |
|  | rs77223587 | 15 | 46123423 | T | G | -0.3175 | 0.0705 | 0.9893 | 6.68E-06 | 10324 |
|  | rs39779 | 16 | 8393906 | C | G | 0.1077 | 0.0227 | 0.1032 | 2.09E-06 | 11345 |
|  | rs12445031 | 16 | 76626873 | C | G | -0.09 | 0.0195 | 0.1382 | 3.92E-06 | 11339 |
|  | rs17700884 | 16 | 88684495 | T | G | -0.105 | 0.0136 | 0.3783 | 1.16E-14 | 11775 |
|  | rs5992909 | 22 | 17005246 | T | C | 0.0828 | 0.0186 | 0.4646 | 8.52E-06 | 6879 |
|  | rs73181003 | 22 | 46922217 | A | G | -0.1122 | 0.0246 | 0.093 | 5.09E-06 | 11345 |
| IL-18 | rs115120333 | 1 | 78975194 | T | C | -0.1967 | 0.0443 | 0.9795 | 8.99E-06 | 14743 |
|  | rs385076 | 2 | 32489851 | T | C | -0.1857 | 0.0123 | 0.3734 | 1.68E-51 | 14296 |
|  | rs144626837 | 2 | 125030834 | T | C | 0.2026 | 0.0458 | 0.9792 | 9.71E-06 | 14741 |
|  | rs12711729 | 2 | 126751021 | T | C | -0.0702 | 0.0153 | 0.7336 | 4.47E-06 | 11345 |
|  | rs139760701 | 3 | 115001900 | A | T | -0.3049 | 0.0604 | 0.989 | 4.46E-07 | 13809 |
|  | rs11918804 | 3 | 196038580 | A | C | -0.1184 | 0.0266 | 0.9337 | 8.54E-06 | 11791 |
|  | rs12648080 | 4 | 121893301 | A | G | 0.072 | 0.0146 | 0.8077 | 8.16E-07 | 14743 |
|  | rs62351325 | 5 | 19698240 | T | C | 0.1557 | 0.0344 | 0.9627 | 6.01E-06 | 14744 |
|  | rs17229943 | 5 | 68682536 | A | C | -0.16 | 0.0267 | 0.9351 | 2.07E-09 | 14744 |
|  | rs72766437 | 5 | 83710370 | T | C | 0.0637 | 0.0143 | 0.2088 | 8.41E-06 | 14734 |
|  | rs4140444 | 6 | 38300402 | A | G | 0.2157 | 0.0461 | 0.0204 | 2.88E-06 | 14741 |
|  | rs4612182 | 6 | 138336137 | A | G | -0.0547 | 0.0123 | 0.5914 | 8.70E-06 | 14743 |
|  | rs7755771 | 6 | 158786921 | T | C | -0.0861 | 0.0181 | 0.2688 | 1.97E-06 | 10893 |
|  | rs12201187 | 6 | 162785078 | A | T | 0.0676 | 0.0138 | 0.2767 | 9.65E-07 | 13245 |
|  | rs67424072 | 7 | 11104167 | T | G | -0.082 | 0.0165 | 0.8341 | 6.71E-07 | 14296 |
|  | rs56836641 | 7 | 155637974 | T | C | -0.163 | 0.0301 | 0.0477 | 6.12E-08 | 14743 |
|  | rs12681624 | 8 | 144606172 | C | G | -0.0935 | 0.0157 | 0.8179 | 2.59E-09 | 13657 |
|  | rs112338272 | 9 | 20069041 | A | C | -0.1954 | 0.0425 | 0.0236 | 4.27E-06 | 14721 |
|  | rs56266057 | 10 | 17082153 | T | G | 0.0796 | 0.018 | 0.8662 | 9.77E-06 | 14295 |
|  | rs11605127 | 11 | 268430 | C | G | -0.0629 | 0.0137 | 0.7543 | 4.41E-06 | 14730 |
|  | rs4758106 | 11 | 6482713 | C | G | 0.0595 | 0.0133 | 0.5639 | 7.69E-06 | 12411 |
|  | rs55858715 | 11 | 86685696 | T | G | -0.117 | 0.0254 | 0.0649 | 4.10E-06 | 13233 |
|  | rs60235102 | 11 | 105060888 | T | C | 0.1154 | 0.0225 | 0.9237 | 2.91E-07 | 14722 |
|  | rs5744249 | 11 | 112025306 | A | C | 0.2039 | 0.0135 | 0.7618 | 1.53E-51 | 14742 |
|  | rs35495214 | 11 | 112433719 | T | C | 0.1374 | 0.0291 | 0.0465 | 2.34E-06 | 14735 |
|  | rs113841145 | 11 | 116331456 | A | G | 0.2866 | 0.0629 | 0.9893 | 5.20E-06 | 13809 |
|  | rs7990672 | 13 | 90838056 | T | G | -0.0568 | 0.0128 | 0.69 | 9.10E-06 | 14295 |
|  | rs35656694 | 16 | 52032393 | A | C | 0.0746 | 0.0165 | 0.851 | 6.15E-06 | 14744 |
|  | rs148504635 | 16 | 54161464 | A | C | 0.2532 | 0.0525 | 0.9857 | 1.42E-06 | 14737 |
|  | rs113552426 | 16 | 84730435 | C | G | -0.278 | 0.0581 | 0.9792 | 1.71E-06 | 11249 |
|  | rs77994606 | 18 | 28763724 | A | G | -0.2314 | 0.0472 | 0.9814 | 9.46E-07 | 14743 |
|  | rs2108621 | 19 | 5344989 | T | C | 0.0581 | 0.0126 | 0.3565 | 4.01E-06 | 14296 |
|  | rs1403742 | 20 | 51289816 | A | T | -0.0567 | 0.0124 | 0.371 | 4.82E-06 | 14296 |
| IL-18R1 | rs7512350 | 1 | 15404794 | A | G | 0.0921 | 0.0201 | 0.1213 | 4.60E-06 | 11771 |
|  | rs6693634 | 1 | 162509685 | A | C | 0.0537 | 0.012 | 0.4791 | 7.64E-06 | 14295 |
|  | rs3010065 | 1 | 183830781 | A | G | -0.0644 | 0.0145 | 0.7923 | 8.94E-06 | 14724 |
|  | rs78714574 | 2 | 102637685 | C | G | 0.4727 | 0.0474 | 0.0198 | 2.01E-23 | 14735 |
|  | rs951774 | 2 | 102912664 | A | C | 0.5719 | 0.0148 | 0.1798 | 0.00E+00 | 14728 |
|  | rs75366565 | 2 | 103166288 | T | C | -0.401 | 0.0142 | 0.1988 | 1.92E-175 | 14735 |
|  | rs2732865 | 2 | 103401780 | A | C | 0.3635 | 0.0303 | 0.0405 | 3.70E-33 | 14743 |
|  | rs113070302 | 2 | 103454352 | T | G | -0.1397 | 0.0284 | 0.0535 | 8.70E-07 | 14743 |
|  | rs114696908 | 2 | 236845654 | A | G | 0.1235 | 0.0278 | 0.0625 | 8.89E-06 | 14295 |
|  | rs7642750 | 3 | 10580281 | A | C | 0.0935 | 0.02 | 0.8764 | 2.94E-06 | 11788 |
|  | rs35021151 | 3 | 58414784 | A | G | -0.1646 | 0.0342 | 0.0317 | 1.49E-06 | 14735 |
|  | rs62257659 | 3 | 79589418 | A | G | -0.2015 | 0.042 | 0.9745 | 1.61E-06 | 14727 |
|  | rs78199195 | 3 | 146882115 | A | G | -0.0956 | 0.0196 | 0.8928 | 1.07E-06 | 14295 |
|  | rs34333927 | 5 | 9342797 | A | G | 0.0658 | 0.0147 | 0.2237 | 7.60E-06 | 14741 |
|  | rs139290695 | 5 | 120319225 | A | T | -0.1505 | 0.0336 | 0.9589 | 7.49E-06 | 11791 |
|  | rs78035061 | 6 | 801754 | A | C | 0.1039 | 0.0222 | 0.0787 | 2.87E-06 | 14295 |
|  | rs2267951 | 6 | 133019858 | T | C | 0.056 | 0.0125 | 0.3421 | 7.46E-06 | 14295 |
|  | rs117145942 | 7 | 29690677 | A | G | -0.3006 | 0.0649 | 0.0131 | 3.63E-06 | 13164 |
|  | rs80317525 | 7 | 47517191 | A | G | 0.1408 | 0.0299 | 0.0468 | 2.49E-06 | 14730 |
|  | rs73176827 | 7 | 156048777 | T | C | -0.1045 | 0.022 | 0.0762 | 2.03E-06 | 14733 |
|  | rs190615956 | 8 | 67827730 | A | T | -0.3331 | 0.0754 | 0.0269 | 9.97E-06 | 7424 |
|  | rs148530961 | 9 | 1057984 | A | G | 0.2415 | 0.0532 | 0.017 | 5.64E-06 | 14295 |
|  | rs62554051 | 9 | 29175150 | C | G | -0.1084 | 0.0243 | 0.0677 | 8.16E-06 | 14735 |
|  | rs60148804 | 10 | 47584857 | C | G | -0.2162 | 0.0479 | 0.0325 | 6.37E-06 | 9829 |
|  | rs117195704 | 10 | 61379709 | A | G | -0.1505 | 0.0313 | 0.0389 | 1.52E-06 | 14730 |
|  | rs139990768 | 11 | 37732931 | T | C | -0.1848 | 0.0415 | 0.0239 | 8.47E-06 | 13860 |
|  | rs7398357 | 12 | 99887674 | A | G | 0.0662 | 0.0147 | 0.1899 | 6.69E-06 | 14735 |
|  | rs1760941 | 14 | 20930038 | A | C | 0.0657 | 0.0142 | 0.7591 | 3.71E-06 | 14295 |
|  | rs68109559 | 14 | 62549256 | T | C | -0.0553 | 0.0124 | 0.3212 | 8.21E-06 | 14732 |
|  | rs28929474 | 14 | 94844947 | T | C | 0.2643 | 0.0445 | 0.0204 | 2.86E-09 | 14743 |
|  | rs75969720 | 15 | 70052155 | A | G | 0.1945 | 0.0439 | 0.021 | 9.40E-06 | 14743 |
|  | rs1477086 | 16 | 54743526 | A | G | -0.1103 | 0.0243 | 0.0692 | 5.65E-06 | 14295 |
|  | rs117599131 | 16 | 87926882 | A | C | -0.1483 | 0.0317 | 0.0536 | 2.89E-06 | 13231 |
|  | rs78357146 | 17 | 64305051 | A | G | -0.3382 | 0.0378 | 0.9715 | 3.65E-19 | 14743 |
|  | rs10401247 | 19 | 52051784 | A | G | 0.095 | 0.0211 | 0.8846 | 6.72E-06 | 11344 |
|  | rs34174304 | 20 | 51193977 | A | G | 0.073 | 0.0149 | 0.8157 | 9.62E-07 | 14725 |
|  | rs140199696 | 21 | 24054958 | T | C | -0.2071 | 0.0454 | 0.9784 | 5.07E-06 | 14295 |
|  | rs79097546 | 22 | 19662319 | T | C | -0.2754 | 0.056 | 0.9865 | 8.75E-07 | 14256 |
|  | rs116935210 | 22 | 34904594 | A | G | 0.2387 | 0.0512 | 0.9848 | 3.13E-06 | 14733 |
| IL-1 α | rs6702801 | 1 | 89516076 | C | G | -0.077 | 0.0171 | 0.6598 | 6.70E-06 | 7943 |
|  | rs12047801 | 1 | 105113404 | T | C | 0.0783 | 0.0171 | 0.174 | 4.67E-06 | 11785 |
|  | rs115494443 | 1 | 116784131 | A | G | 0.2658 | 0.0565 | 0.0186 | 2.55E-06 | 10853 |
|  | rs115602471 | 2 | 170964850 | T | C | 0.2215 | 0.0469 | 0.0311 | 2.33E-06 | 11340 |
|  | rs190042298 | 3 | 147628690 | A | G | 0.1502 | 0.0339 | 0.0497 | 9.39E-06 | 9897 |
|  | rs10063171 | 5 | 67352803 | T | G | 0.0775 | 0.0175 | 0.1932 | 9.49E-06 | 11340 |
|  | rs10057700 | 5 | 161958436 | A | G | -0.0822 | 0.0181 | 0.4388 | 5.59E-06 | 8587 |
|  | rs11759846 | 6 | 32586222 | A | G | -0.3263 | 0.0266 | 0.0799 | 1.36E-34 | 11788 |
|  | rs9272305 | 6 | 32603879 | A | C | -0.2444 | 0.0462 | 0.0316 | 1.22E-07 | 8587 |
|  | rs13195317 | 6 | 32656094 | T | C | -0.3251 | 0.0554 | 0.0358 | 4.40E-09 | 11301 |
|  | rs12529944 | 6 | 84066908 | T | C | -0.0845 | 0.0189 | 0.178 | 7.79E-06 | 11340 |
|  | rs10233998 | 7 | 89060931 | T | G | 0.131 | 0.0286 | 0.0604 | 4.64E-06 | 11785 |
|  | rs16886503 | 8 | 89744017 | A | T | 0.2438 | 0.0524 | 0.9824 | 3.28E-06 | 11786 |
|  | rs72937918 | 11 | 80672314 | T | C | -0.2056 | 0.0452 | 0.0233 | 5.40E-06 | 11772 |
|  | rs523604 | 11 | 118755738 | A | G | 0.0754 | 0.0142 | 0.5264 | 1.10E-07 | 10290 |
|  | rs76012752 | 12 | 28953348 | A | G | 0.1568 | 0.0352 | 0.9527 | 8.41E-06 | 11340 |
|  | rs7138689 | 12 | 103893494 | T | G | 0.0961 | 0.0208 | 0.8877 | 3.83E-06 | 11777 |
|  | rs4767214 | 12 | 114539621 | T | C | 0.062 | 0.013 | 0.5326 | 1.85E-06 | 11772 |
|  | rs76544453 | 14 | 81651382 | T | C | -0.1609 | 0.0361 | 0.9585 | 8.31E-06 | 11788 |
|  | rs78267257 | 14 | 83490781 | A | G | -0.1825 | 0.0406 | 0.0323 | 6.95E-06 | 10903 |
|  | rs117537822 | 17 | 32073614 | T | C | 0.2176 | 0.0439 | 0.9755 | 7.17E-07 | 11788 |
|  | rs61098036 | 17 | 53509585 | A | G | -0.0856 | 0.0187 | 0.8232 | 4.70E-06 | 9891 |
|  | rs11878153 | 18 | 308555 | A | G | 0.1116 | 0.0221 | 0.9015 | 4.42E-07 | 11787 |
|  | rs2426634 | 20 | 55282711 | A | G | -0.0896 | 0.0198 | 0.1376 | 6.03E-06 | 11787 |
| IL-2 | rs79419497 | 2 | 192579384 | A | G | 0.2016 | 0.0424 | 0.9733 | 1.99E-06 | 11789 |
|  | rs142641125 | 3 | 12396234 | T | C | 0.2095 | 0.0419 | 0.0287 | 5.73E-07 | 11789 |
|  | rs77853517 | 3 | 64865615 | T | C | -0.1048 | 0.0217 | 0.8961 | 1.37E-06 | 11787 |
|  | rs7638939 | 3 | 125991040 | A | T | 0.0854 | 0.0174 | 0.8293 | 9.20E-07 | 11769 |
|  | rs75739108 | 3 | 137247468 | T | C | 0.1737 | 0.038 | 0.039 | 4.85E-06 | 11341 |
|  | rs111783480 | 4 | 14756774 | A | G | -0.1462 | 0.0329 | 0.1075 | 8.84E-06 | 9265 |
|  | rs79925007 | 4 | 34713434 | T | G | 0.1176 | 0.0255 | 0.9036 | 3.99E-06 | 11341 |
|  | rs77880908 | 4 | 47700645 | T | C | -0.0886 | 0.0196 | 0.1258 | 6.17E-06 | 11770 |
|  | rs6535212 | 4 | 138235156 | T | G | -0.0646 | 0.0139 | 0.6861 | 3.36E-06 | 11784 |
|  | rs4703508 | 5 | 35188802 | T | C | -0.1216 | 0.026 | 0.9334 | 2.91E-06 | 11789 |
|  | rs72659067 | 8 | 62639773 | A | G | 0.3327 | 0.0736 | 0.0111 | 6.17E-06 | 9980 |
|  | rs11141276 | 9 | 88777064 | T | C | 0.1434 | 0.0306 | 0.0611 | 2.78E-06 | 9265 |
|  | rs74852566 | 10 | 2293803 | T | G | -0.1999 | 0.0431 | 0.0244 | 3.52E-06 | 11789 |
|  | rs1657222 | 10 | 34837139 | A | G | -0.0621 | 0.0137 | 0.4037 | 5.82E-06 | 11340 |
|  | rs10996419 | 10 | 67202938 | T | C | -0.1222 | 0.0259 | 0.9188 | 2.38E-06 | 11784 |
|  | rs55813957 | 11 | 128560199 | C | G | 0.0728 | 0.016 | 0.7076 | 5.36E-06 | 11341 |
|  | rs77832761 | 13 | 85868678 | A | G | 0.1922 | 0.0414 | 0.0318 | 3.44E-06 | 11785 |
|  | rs144129701 | 14 | 55563894 | C | G | -0.2699 | 0.0576 | 0.9727 | 2.79E-06 | 11341 |
|  | rs36006983 | 16 | 77431739 | T | C | -0.0728 | 0.0153 | 0.7384 | 1.95E-06 | 11341 |
|  | rs28515912 | 17 | 103299 | A | C | -0.0723 | 0.0149 | 0.6969 | 1.22E-06 | 10723 |
|  | rs1943222 | 18 | 58028057 | A | G | -0.0622 | 0.0138 | 0.6878 | 6.57E-06 | 11789 |
| IL-20 | rs72633862 | 1 | 9746065 | T | G | -0.1722 | 0.0376 | 0.0444 | 4.65E-06 | 11774 |
|  | rs114363239 | 1 | 41433965 | T | C | -0.2372 | 0.0487 | 0.0189 | 1.11E-06 | 11782 |
|  | rs112421516 | 1 | 67400701 | A | G | 0.1935 | 0.0435 | 0.9708 | 8.66E-06 | 11336 |
|  | rs115191082 | 1 | 104640701 | T | G | -0.2543 | 0.0561 | 0.0149 | 5.82E-06 | 11294 |
|  | rs2285212 | 1 | 169461481 | T | C | -0.1112 | 0.0248 | 0.9158 | 7.33E-06 | 11336 |
|  | rs383039 | 1 | 242399370 | A | G | -0.0698 | 0.0156 | 0.2136 | 7.66E-06 | 11783 |
|  | rs13421547 | 2 | 13940522 | T | C | 0.147 | 0.0325 | 0.044 | 6.09E-06 | 11784 |
|  | rs114209791 | 2 | 52215130 | A | G | -0.2134 | 0.046 | 0.9756 | 3.50E-06 | 11784 |
|  | rs146918864 | 2 | 141913966 | T | C | -0.2551 | 0.0515 | 0.0228 | 7.29E-07 | 11438 |
|  | rs35508685 | 2 | 213241261 | T | C | 0.1688 | 0.0373 | 0.9672 | 6.03E-06 | 11782 |
|  | rs73192044 | 3 | 119431497 | A | G | -0.1066 | 0.0237 | 0.9032 | 6.86E-06 | 11336 |
|  | rs12498781 | 4 | 133183457 | A | G | 0.0635 | 0.0137 | 0.3202 | 3.57E-06 | 11763 |
|  | rs34172193 | 6 | 112451601 | A | G | -0.2175 | 0.0488 | 0.02 | 8.31E-06 | 10918 |
|  | rs143181662 | 7 | 17753237 | T | C | 0.0719 | 0.0156 | 0.5043 | 4.05E-06 | 8587 |
|  | rs6952803 | 7 | 54991055 | A | G | 0.2776 | 0.0597 | 0.9804 | 3.32E-06 | 10849 |
|  | rs138771212 | 8 | 68839107 | A | G | -0.3596 | 0.0778 | 0.9909 | 3.80E-06 | 11336 |
|  | rs188205021 | 11 | 100596026 | T | C | -0.222 | 0.0427 | 0.0255 | 2.00E-07 | 11774 |
|  | rs143409618 | 13 | 112575760 | T | C | -0.2247 | 0.0504 | 0.0541 | 8.26E-06 | 11784 |
|  | rs72659995 | 14 | 21169862 | T | C | -0.1232 | 0.0267 | 0.9142 | 3.95E-06 | 11336 |
|  | rs61992201 | 14 | 48507353 | T | G | 0.0888 | 0.0201 | 0.1397 | 9.97E-06 | 10267 |
|  | rs28542561 | 15 | 23769949 | T | C | -0.1753 | 0.0372 | 0.965 | 2.45E-06 | 11774 |
|  | rs117977288 | 16 | 80689313 | T | G | -0.1546 | 0.0316 | 0.9553 | 9.96E-07 | 11784 |
|  | rs12969831 | 18 | 74247282 | A | G | 0.1474 | 0.0331 | 0.9399 | 8.46E-06 | 11336 |
|  | rs34691354 | 18 | 75094435 | C | G | 0.0651 | 0.0143 | 0.6659 | 5.30E-06 | 11336 |
| IL-20RA | rs28846828 | 1 | 153082810 | T | C | -0.0765 | 0.0154 | 0.4228 | 6.78E-07 | 8817 |
|  | rs12138611 | 1 | 168180874 | C | G | 0.119 | 0.0256 | 0.1039 | 3.34E-06 | 11344 |
|  | rs2064077 | 1 | 171216897 | T | C | 0.1072 | 0.0241 | 0.083 | 8.66E-06 | 11790 |
|  | rs2883991 | 2 | 53038599 | T | C | 0.08 | 0.0181 | 0.8477 | 9.88E-06 | 11789 |
|  | rs4667792 | 2 | 166018354 | T | C | -0.0606 | 0.0135 | 0.5471 | 7.16E-06 | 11344 |
|  | rs9817113 | 3 | 151178393 | T | C | 0.1541 | 0.0346 | 0.0446 | 8.44E-06 | 11790 |
|  | rs6842116 | 4 | 6909807 | C | G | -0.0925 | 0.0204 | 0.8324 | 5.78E-06 | 11344 |
|  | rs115680168 | 4 | 164735206 | T | C | -0.3364 | 0.0757 | 0.0177 | 8.84E-06 | 7586 |
|  | rs188200783 | 5 | 22203180 | T | C | -0.2004 | 0.0449 | 0.0429 | 8.07E-06 | 8974 |
|  | rs143067549 | 5 | 46131995 | T | C | -0.4166 | 0.091 | 0.0114 | 4.69E-06 | 7315 |
|  | rs59446445 | 5 | 154577444 | A | G | 0.1741 | 0.038 | 0.0321 | 4.61E-06 | 11790 |
|  | rs145607970 | 6 | 32624017 | A | C | -0.2162 | 0.048 | 0.0367 | 6.66E-06 | 7523 |
|  | rs6920753 | 6 | 158071161 | A | G | -0.0664 | 0.0144 | 0.704 | 4.01E-06 | 11762 |
|  | rs113563938 | 7 | 12560554 | A | T | 0.3176 | 0.0676 | 0.9871 | 2.62E-06 | 11344 |
|  | rs11987533 | 8 | 35154239 | A | G | 0.1171 | 0.0198 | 0.1374 | 3.34E-09 | 11344 |
|  | rs12056343 | 8 | 79840734 | T | G | -0.0761 | 0.0166 | 0.7494 | 4.55E-06 | 9894 |
|  | rs113153423 | 8 | 139987300 | A | G | 0.1913 | 0.0407 | 0.972 | 2.60E-06 | 11792 |
|  | rs113105910 | 11 | 5315973 | A | G | -0.2425 | 0.0538 | 0.0165 | 6.56E-06 | 11786 |
|  | rs71476058 | 11 | 109386685 | A | T | -0.2711 | 0.0606 | 0.0278 | 7.69E-06 | 7943 |
|  | rs72785422 | 16 | 14288564 | T | C | 0.0897 | 0.0195 | 0.8313 | 4.22E-06 | 11343 |
|  | rs74032581 | 16 | 83519341 | A | T | -0.1343 | 0.0298 | 0.9366 | 6.58E-06 | 11344 |
|  | rs149111746 | 17 | 59389022 | A | G | 0.2357 | 0.0511 | 0.0208 | 3.98E-06 | 11783 |
| IL-22RA1 | rs187651556 | 1 | 29484787 | T | C | -0.3827 | 0.0859 | 0.9838 | 8.38E-06 | 6752 |
|  | rs78594373 | 2 | 85320044 | T | C | -0.1271 | 0.0273 | 0.0671 | 3.23E-06 | 11767 |
|  | rs114416525 | 3 | 99744651 | A | G | 0.2819 | 0.0619 | 0.0142 | 5.26E-06 | 11345 |
|  | rs1473089 | 4 | 26143082 | A | C | 0.2679 | 0.0561 | 0.0253 | 1.79E-06 | 10916 |
|  | rs75590335 | 4 | 157031745 | A | G | 0.1722 | 0.037 | 0.0376 | 3.25E-06 | 11345 |
|  | rs13169282 | 5 | 144256718 | A | G | -0.0709 | 0.0158 | 0.2216 | 7.21E-06 | 11783 |
|  | rs79229049 | 7 | 81154741 | A | T | -0.2102 | 0.0412 | 0.9695 | 3.36E-07 | 11781 |
|  | rs11777386 | 8 | 52767675 | A | G | -0.1003 | 0.0224 | 0.0953 | 7.55E-06 | 11779 |
|  | rs56282088 | 8 | 81502336 | A | G | 0.2815 | 0.0571 | 0.0165 | 8.23E-07 | 10919 |
|  | rs13257574 | 8 | 87485765 | T | C | 0.2714 | 0.0584 | 0.0167 | 3.36E-06 | 11306 |
|  | rs3181361 | 9 | 117667449 | T | C | 0.27 | 0.0582 | 0.0163 | 3.50E-06 | 11345 |
|  | rs12240324 | 10 | 44669337 | A | G | 0.2203 | 0.047 | 0.9713 | 2.77E-06 | 11793 |
|  | rs11607326 | 11 | 83564435 | A | T | -0.0919 | 0.0206 | 0.1246 | 8.15E-06 | 11345 |
|  | rs369913010 | 12 | 131925456 | T | C | -0.2141 | 0.0484 | 0.0264 | 9.71E-06 | 9246 |
|  | rs187050106 | 13 | 56206816 | T | C | 0.4278 | 0.0964 | 0.0081 | 9.09E-06 | 8488 |
|  | rs17256492 | 14 | 62394907 | T | C | -0.0751 | 0.0162 | 0.7694 | 3.56E-06 | 11345 |
|  | rs7158900 | 14 | 94197473 | A | G | -0.0674 | 0.0138 | 0.6092 | 1.04E-06 | 11344 |
|  | rs143949731 | 18 | 22333712 | T | C | 0.1655 | 0.0366 | 0.0384 | 6.13E-06 | 11782 |
|  | rs117636462 | 20 | 16761823 | A | G | 0.3671 | 0.0828 | 0.9915 | 9.27E-06 | 10858 |
|  | rs4811167 | 20 | 50001752 | T | C | 0.0632 | 0.0134 | 0.5504 | 2.40E-06 | 11793 |
|  | rs117263973 | 21 | 42295154 | A | G | 0.3467 | 0.0752 | 0.0109 | 4.02E-06 | 10858 |
|  | rs4466 | 22 | 35723831 | A | G | 0.0906 | 0.0203 | 0.1324 | 8.08E-06 | 11792 |
| IL-24 | rs116790447 | 1 | 18931322 | T | C | 0.1729 | 0.0371 | 0.0521 | 3.16E-06 | 9453 |
|  | rs199770642 | 1 | 153060770 | A | T | 0.0762 | 0.0162 | 0.4768 | 2.55E-06 | 7943 |
|  | rs36183478 | 1 | 236592040 | T | C | 0.0815 | 0.0172 | 0.4915 | 2.15E-06 | 8587 |
|  | rs61760504 | 4 | 47684375 | C | G | 0.12 | 0.0265 | 0.9234 | 5.95E-06 | 11337 |
|  | rs2341294 | 5 | 149232308 | A | G | -0.1318 | 0.028 | 0.0716 | 2.51E-06 | 9900 |
|  | rs145295031 | 7 | 9959098 | A | T | 0.3063 | 0.0647 | 0.014 | 2.20E-06 | 11287 |
|  | rs167018 | 7 | 155623090 | A | G | 0.0657 | 0.0136 | 0.4443 | 1.36E-06 | 11336 |
|  | rs76088721 | 9 | 23373327 | T | C | 0.1847 | 0.0417 | 0.9596 | 9.46E-06 | 8587 |
|  | rs11256610 | 10 | 6122343 | A | G | 0.3005 | 0.0641 | 0.0145 | 2.76E-06 | 10850 |
|  | rs145450029 | 11 | 76932037 | T | G | -0.2153 | 0.0484 | 0.0246 | 8.65E-06 | 11784 |
|  | rs145660089 | 11 | 94477313 | T | C | 0.2004 | 0.0446 | 0.0274 | 7.01E-06 | 11779 |
|  | rs12275648 | 11 | 129057999 | T | C | -0.0696 | 0.0155 | 0.3114 | 7.11E-06 | 10277 |
|  | rs145891540 | 12 | 93849398 | T | G | 0.1913 | 0.0422 | 0.0315 | 5.81E-06 | 11772 |
|  | rs61997688 | 14 | 57398908 | T | C | -0.0757 | 0.017 | 0.8019 | 8.47E-06 | 11772 |
|  | rs7401175 | 14 | 86024172 | A | T | 0.0892 | 0.0195 | 0.2392 | 4.78E-06 | 7943 |
|  | rs117613902 | 14 | 97741799 | A | G | -0.3 | 0.0657 | 0.9862 | 4.97E-06 | 10850 |
|  | rs12588414 | 14 | 104576816 | A | G | 0.0753 | 0.0169 | 0.2361 | 8.37E-06 | 10273 |
|  | rs60306270 | 16 | 49575164 | A | G | -0.0886 | 0.0191 | 0.1398 | 3.51E-06 | 11781 |
|  | rs2165910 | 18 | 7919506 | A | G | 0.0674 | 0.015 | 0.7371 | 7.01E-06 | 11768 |
|  | rs113974014 | 19 | 2576036 | C | G | -0.1197 | 0.0268 | 0.8687 | 7.95E-06 | 7943 |
|  | rs143986178 | 21 | 26067535 | A | G | 0.2969 | 0.0644 | 0.0144 | 4.02E-06 | 10850 |
|  | rs73169406 | 22 | 48661109 | A | G | 0.111 | 0.0207 | 0.1327 | 8.22E-08 | 11337 |
| IL-2RB | rs116068903 | 1 | 67498721 | T | C | 0.2831 | 0.0636 | 0.0151 | 8.54E-06 | 10470 |
|  | rs116381951 | 1 | 177439129 | T | C | 0.2794 | 0.063 | 0.9817 | 9.21E-06 | 9983 |
|  | rs13001788 | 2 | 31896839 | T | C | 0.2769 | 0.0557 | 0.9745 | 6.65E-07 | 8243 |
|  | rs115890206 | 2 | 171243489 | A | G | 0.232 | 0.0502 | 0.9786 | 3.81E-06 | 10403 |
|  | rs111805691 | 4 | 13099601 | A | G | -0.1278 | 0.0286 | 0.0648 | 7.88E-06 | 11344 |
|  | rs17549820 | 4 | 108118301 | T | C | 0.0631 | 0.0129 | 0.5622 | 1.00E-06 | 11791 |
|  | rs116007066 | 4 | 136755243 | A | G | -0.2101 | 0.0441 | 0.025 | 1.90E-06 | 10295 |
|  | rs11153066 | 6 | 107846644 | T | C | 0.0615 | 0.0137 | 0.5833 | 7.15E-06 | 11343 |
|  | rs9321271 | 6 | 131393771 | A | C | -0.157 | 0.0325 | 0.9549 | 1.36E-06 | 11779 |
|  | rs1472270 | 7 | 98121843 | C | G | 0.0704 | 0.0144 | 0.346 | 1.01E-06 | 11344 |
|  | rs147955591 | 8 | 120536724 | A | C | -0.0739 | 0.0166 | 0.2186 | 8.52E-06 | 11344 |
|  | rs301439 | 9 | 4589802 | C | G | -0.0643 | 0.0144 | 0.6412 | 8.00E-06 | 11344 |
|  | rs34246740 | 11 | 36198233 | T | C | -0.0721 | 0.0156 | 0.2619 | 3.80E-06 | 11344 |
|  | rs141460846 | 11 | 40470032 | T | C | -0.2471 | 0.0535 | 0.9783 | 3.86E-06 | 11304 |
|  | rs71476282 | 11 | 115220909 | T | C | -0.1842 | 0.0412 | 0.0363 | 7.79E-06 | 11344 |
|  | rs56353341 | 12 | 13099229 | T | C | -0.0984 | 0.021 | 0.7599 | 2.79E-06 | 7943 |
|  | rs2157876 | 12 | 111592380 | T | G | -0.1133 | 0.0239 | 0.0879 | 2.13E-06 | 11343 |
|  | rs111358562 | 13 | 30976443 | A | C | -0.1638 | 0.0368 | 0.3346 | 8.54E-06 | 8817 |
|  | rs55757846 | 14 | 25692351 | A | G | -0.1021 | 0.0231 | 0.8947 | 9.87E-06 | 11344 |
|  | rs11630708 | 15 | 84665328 | T | C | -0.2102 | 0.0443 | 0.9766 | 2.09E-06 | 11788 |
|  | rs55987136 | 16 | 12220199 | T | C | -0.1967 | 0.0443 | 0.9753 | 8.99E-06 | 11781 |
|  | rs9921181 | 16 | 80775714 | A | G | -0.0606 | 0.0128 | 0.4465 | 2.20E-06 | 11786 |
|  | rs117538197 | 17 | 4507431 | T | C | 0.1213 | 0.0261 | 0.0695 | 3.36E-06 | 11774 |
|  | rs117953283 | 17 | 74316443 | T | G | 0.1688 | 0.0374 | 0.0635 | 6.38E-06 | 8397 |
|  | rs2281533 | 20 | 6103768 | C | G | -0.1844 | 0.0404 | 0.0481 | 5.01E-06 | 11776 |
| IL-33 | rs1273797 | 2 | 227438887 | A | G | -0.0652 | 0.0144 | 0.4698 | 5.96E-06 | 9252 |
|  | rs1427438 | 3 | 69721499 | T | C | 0.3362 | 0.0761 | 0.0144 | 9.97E-06 | 10891 |
|  | rs28658099 | 4 | 45839898 | A | T | 0.0848 | 0.0188 | 0.8568 | 6.46E-06 | 11773 |
|  | rs35370544 | 4 | 189492967 | T | G | 0.1741 | 0.0364 | 0.047 | 1.73E-06 | 8385 |
|  | rs10941408 | 5 | 38788544 | T | C | -0.0666 | 0.0148 | 0.2923 | 6.80E-06 | 11345 |
|  | rs7742625 | 6 | 38719850 | C | G | 0.0691 | 0.0148 | 0.6997 | 3.03E-06 | 11345 |
|  | rs60781061 | 6 | 85285875 | A | G | 0.1773 | 0.0398 | 0.9431 | 8.40E-06 | 9265 |
|  | rs77792454 | 6 | 156872261 | T | C | -0.1166 | 0.0255 | 0.1064 | 4.82E-06 | 8817 |
|  | rs17149396 | 7 | 24423445 | C | G | 0.1833 | 0.04 | 0.0307 | 4.59E-06 | 11789 |
|  | rs75671803 | 7 | 83421169 | A | G | 0.2055 | 0.0451 | 0.9763 | 5.20E-06 | 11785 |
|  | rs66755903 | 8 | 87995921 | A | G | -0.0752 | 0.0162 | 0.2321 | 3.45E-06 | 11345 |
|  | rs62556318 | 9 | 21663051 | C | G | 0.1308 | 0.0277 | 0.9172 | 2.34E-06 | 11345 |
|  | rs79417946 | 9 | 31779365 | T | C | -0.1259 | 0.0285 | 0.0665 | 9.98E-06 | 11345 |
|  | rs17147023 | 9 | 133361010 | T | C | 0.0689 | 0.0154 | 0.7409 | 7.68E-06 | 11344 |
|  | rs78455659 | 12 | 96439814 | A | G | 0.1259 | 0.0273 | 0.9273 | 3.99E-06 | 11345 |
|  | rs8042883 | 15 | 66970271 | T | C | -0.0681 | 0.0135 | 0.5496 | 4.55E-07 | 11345 |
|  | rs77248338 | 15 | 98506292 | T | G | 0.2314 | 0.0518 | 0.9759 | 7.93E-06 | 10471 |
|  | rs13336496 | 16 | 79491576 | A | C | 0.0936 | 0.0203 | 0.8755 | 4.01E-06 | 11780 |
|  | rs78103000 | 16 | 83303070 | T | C | -0.0889 | 0.0201 | 0.8647 | 9.74E-06 | 11345 |
|  | rs12453636 | 17 | 64288475 | A | G | -0.1028 | 0.0231 | 0.1006 | 8.58E-06 | 9892 |
|  | rs112495884 | 18 | 73359115 | A | G | 0.3432 | 0.0748 | 0.0112 | 4.47E-06 | 10858 |
|  | rs10415966 | 19 | 14299739 | A | C | -0.1184 | 0.0233 | 0.1177 | 3.74E-07 | 10281 |
|  | rs6099755 | 20 | 56364589 | A | G | 0.095 | 0.0212 | 0.1263 | 7.42E-06 | 11345 |
|  | rs1013434 | 21 | 40484010 | T | C | -0.0732 | 0.0158 | 0.2201 | 3.61E-06 | 11783 |
| IL-4 | rs10910814 | 1 | 145427960 | C | G | 0.1919 | 0.0417 | 0.0407 | 4.19E-06 | 7953 |
|  | rs9425728 | 1 | 173492229 | T | G | 0.0664 | 0.015 | 0.2727 | 9.57E-06 | 11345 |
|  | rs2502347 | 1 | 243071390 | T | C | -0.077 | 0.0167 | 0.2195 | 4.01E-06 | 11345 |
|  | rs6712163 | 2 | 43501970 | T | C | 0.0609 | 0.0136 | 0.546 | 7.54E-06 | 11344 |
|  | rs13418255 | 2 | 107668175 | A | G | 0.0701 | 0.0153 | 0.7233 | 4.61E-06 | 11345 |
|  | rs7587695 | 2 | 172513639 | A | C | 0.0598 | 0.0134 | 0.6086 | 8.09E-06 | 11793 |
|  | rs13130198 | 4 | 40156881 | A | G | -0.0773 | 0.0159 | 0.2374 | 1.16E-06 | 11345 |
|  | rs6552795 | 4 | 185535085 | T | G | 0.0753 | 0.0157 | 0.482 | 1.62E-06 | 8100 |
|  | rs9270229 | 6 | 32556783 | T | C | 0.1212 | 0.0255 | 0.1501 | 2.00E-06 | 6252 |
|  | rs2766594 | 6 | 35764540 | A | G | 0.085 | 0.0179 | 0.5892 | 2.05E-06 | 7943 |
|  | rs71552590 | 6 | 62665592 | T | C | 0.0782 | 0.0173 | 0.6589 | 6.18E-06 | 7943 |
|  | rs802281 | 6 | 124516990 | T | G | -0.1305 | 0.0294 | 0.9368 | 9.05E-06 | 11786 |
|  | rs11989681 | 8 | 22649912 | A | G | 0.0678 | 0.0136 | 0.5982 | 6.19E-07 | 11345 |
|  | rs79359966 | 8 | 71370392 | A | C | -0.1552 | 0.0345 | 0.9596 | 6.84E-06 | 11775 |
|  | rs4876407 | 8 | 119274249 | T | G | -0.1391 | 0.0299 | 0.9451 | 3.28E-06 | 11788 |
|  | rs1490743 | 9 | 117036047 | T | G | -0.1338 | 0.0278 | 0.0752 | 1.49E-06 | 10281 |
|  | rs75610895 | 10 | 6800809 | T | C | -0.2354 | 0.045 | 0.0253 | 1.68E-07 | 11787 |
|  | rs116963481 | 10 | 34284406 | A | G | 0.2805 | 0.0592 | 0.9846 | 2.16E-06 | 11793 |
|  | rs371539 | 11 | 131406108 | T | G | 0.0797 | 0.0177 | 0.8324 | 6.71E-06 | 11775 |
|  | rs61984458 | 14 | 64861479 | T | C | -0.1955 | 0.043 | 0.0285 | 5.45E-06 | 11345 |
|  | rs10152568 | 15 | 39160069 | T | C | -0.0682 | 0.0154 | 0.6072 | 9.49E-06 | 11784 |
|  | rs12900168 | 15 | 79066932 | A | G | 0.0716 | 0.0152 | 0.4842 | 2.47E-06 | 10471 |
|  | rs76678067 | 15 | 79597917 | T | G | 0.1045 | 0.0231 | 0.9046 | 6.07E-06 | 11345 |
|  | rs669029 | 18 | 10579314 | A | G | -0.0699 | 0.0158 | 0.7524 | 9.69E-06 | 11345 |
|  | rs12481726 | 20 | 51651490 | T | C | -0.313 | 0.0706 | 0.0162 | 9.27E-06 | 9849 |
| IL-5 | rs114829303 | 1 | 37473314 | T | C | 0.1661 | 0.0361 | 0.0436 | 4.20E-06 | 11344 |
|  | rs139303816 | 1 | 62157502 | A | G | 0.1942 | 0.0435 | 0.0315 | 8.03E-06 | 9895 |
|  | rs41264833 | 1 | 159897328 | A | G | -0.2117 | 0.0468 | 0.0254 | 6.08E-06 | 11790 |
|  | rs17009223 | 1 | 208050360 | A | G | -0.1169 | 0.0236 | 0.0881 | 7.29E-07 | 11789 |
|  | rs10182644 | 2 | 46461654 | T | C | -0.0828 | 0.0186 | 0.1671 | 8.52E-06 | 11344 |
|  | rs72873944 | 2 | 139572732 | T | C | -0.1649 | 0.0368 | 0.9609 | 7.43E-06 | 11792 |
|  | rs112289272 | 3 | 126910721 | T | C | -0.213 | 0.0471 | 0.9743 | 6.12E-06 | 10905 |
|  | rs706716 | 5 | 67548442 | T | C | -0.0708 | 0.0158 | 0.2459 | 7.43E-06 | 11344 |
|  | rs34639000 | 5 | 95530542 | A | G | 0.0765 | 0.0147 | 0.7289 | 1.95E-07 | 11773 |
|  | rs141941146 | 6 | 124474284 | A | G | 0.3833 | 0.0825 | 0.9859 | 3.38E-06 | 10280 |
|  | rs9457226 | 6 | 167250912 | A | G | 0.1911 | 0.0429 | 0.0282 | 8.41E-06 | 11786 |
|  | rs10235012 | 7 | 138588598 | T | C | -0.3402 | 0.075 | 0.0162 | 5.73E-06 | 7943 |
|  | rs2995998 | 9 | 96094846 | T | C | -0.0623 | 0.0137 | 0.3561 | 5.43E-06 | 11792 |
|  | rs10988407 | 9 | 132281601 | A | G | 0.0777 | 0.0167 | 0.2002 | 3.28E-06 | 11791 |
|  | rs4277106 | 11 | 10380223 | A | G | -0.2747 | 0.0592 | 0.9858 | 3.48E-06 | 11790 |
|  | rs2212484 | 11 | 132275295 | T | G | -0.0732 | 0.0162 | 0.2044 | 6.23E-06 | 11792 |
|  | rs12864018 | 13 | 78234635 | A | C | -0.2292 | 0.0499 | 0.0477 | 4.37E-06 | 7943 |
|  | rs7142197 | 14 | 93316119 | A | G | -0.0924 | 0.0188 | 0.1442 | 8.88E-07 | 11773 |
|  | rs144124166 | 15 | 56343222 | T | G | 0.211 | 0.0465 | 0.0271 | 5.69E-06 | 11768 |
|  | rs12592185 | 15 | 70546132 | T | C | 0.0838 | 0.0173 | 0.8068 | 1.27E-06 | 11344 |
|  | rs67927317 | 16 | 6155800 | T | C | -0.114 | 0.0255 | 0.0784 | 7.80E-06 | 11344 |
|  | rs7193141 | 16 | 86933894 | C | G | 0.2407 | 0.0521 | 0.0351 | 3.84E-06 | 9377 |
|  | rs62069649 | 17 | 59402478 | A | G | 0.2206 | 0.0482 | 0.0267 | 4.72E-06 | 10431 |
|  | rs4815018 | 20 | 2159791 | A | G | 0.0615 | 0.0139 | 0.363 | 9.67E-06 | 11344 |
|  | rs132210 | 22 | 48143913 | T | C | -0.0686 | 0.0141 | 0.3408 | 1.14E-06 | 11344 |
| IL-6 | rs2228145 | 1 | 154426970 | A | C | -0.1684 | 0.0119 | 0.6128 | 1.83E-45 | 14742 |
|  | rs17486819 | 2 | 113871806 | T | G | 0.0975 | 0.0211 | 0.9152 | 3.82E-06 | 14739 |
|  | rs112669397 | 4 | 7449788 | T | C | -0.3592 | 0.0787 | 0.0119 | 5.01E-06 | 10217 |
|  | rs248536 | 5 | 141146747 | T | G | -0.0671 | 0.0143 | 0.2276 | 2.70E-06 | 14295 |
|  | rs116457146 | 6 | 32578632 | T | C | 0.064 | 0.0131 | 0.2766 | 1.03E-06 | 14743 |
|  | rs10234370 | 7 | 28486167 | T | C | 0.0582 | 0.0127 | 0.6371 | 4.59E-06 | 14295 |
|  | rs187416244 | 7 | 76792340 | T | C | -0.2244 | 0.0469 | 0.0182 | 1.71E-06 | 14726 |
|  | rs1346147 | 8 | 21260780 | A | G | -0.0659 | 0.0135 | 0.7459 | 1.05E-06 | 14295 |
|  | rs10977774 | 9 | 954741 | A | C | -0.3418 | 0.0701 | 0.9865 | 1.08E-06 | 12312 |
|  | rs76203502 | 10 | 127845552 | A | G | -0.2492 | 0.0557 | 0.017 | 7.68E-06 | 12634 |
|  | rs185969953 | 13 | 68942611 | T | G | -0.3427 | 0.0682 | 0.0105 | 5.04E-07 | 12934 |
|  | rs1642826 | 14 | 77078350 | A | T | 0.1165 | 0.0244 | 0.118 | 1.80E-06 | 10893 |
|  | rs12433894 | 14 | 85619189 | C | G | 0.063 | 0.0139 | 0.2257 | 5.83E-06 | 14726 |
|  | rs35441559 | 18 | 73432128 | A | C | -0.2484 | 0.0553 | 0.9868 | 7.06E-06 | 14740 |
|  | rs79254378 | 19 | 58504085 | A | C | 0.1173 | 0.0262 | 0.0664 | 7.57E-06 | 14295 |
| IL-7 | rs77336315 | 1 | 97287518 | A | C | 0.2512 | 0.0531 | 0.9836 | 2.24E-06 | 13801 |
|  | rs56889120 | 1 | 174141356 | A | G | 0.097 | 0.021 | 0.8744 | 3.86E-06 | 11340 |
|  | rs74131483 | 1 | 194276216 | A | G | 0.1372 | 0.0274 | 0.9451 | 5.52E-07 | 13240 |
|  | rs17161952 | 1 | 223649878 | T | C | 0.0929 | 0.0204 | 0.9031 | 5.27E-06 | 14734 |
|  | rs144728120 | 3 | 35502404 | T | C | 0.1495 | 0.0316 | 0.0436 | 2.23E-06 | 14717 |
|  | rs116415547 | 4 | 110732727 | T | G | 0.2093 | 0.046 | 0.0198 | 5.36E-06 | 13870 |
|  | rs139102025 | 4 | 147814894 | C | G | -0.1493 | 0.0337 | 0.0376 | 9.41E-06 | 12846 |
|  | rs62338232 | 4 | 185760185 | A | G | -0.0813 | 0.018 | 0.1357 | 6.28E-06 | 14288 |
|  | rs11742240 | 5 | 35881376 | T | G | -0.0647 | 0.0131 | 0.2796 | 7.86E-07 | 14736 |
|  | rs1050152 | 5 | 131676320 | T | C | -0.0651 | 0.0133 | 0.4122 | 9.84E-07 | 11785 |
|  | rs202212924 | 5 | 133893338 | A | G | -0.0713 | 0.0159 | 0.2092 | 7.32E-06 | 12202 |
|  | rs62387962 | 5 | 157298830 | T | G | -0.078 | 0.0176 | 0.1347 | 9.34E-06 | 14288 |
|  | rs12699881 | 7 | 2401569 | A | G | 0.068 | 0.0138 | 0.2517 | 8.33E-07 | 14288 |
|  | rs78939185 | 7 | 129478199 | C | G | 0.2134 | 0.0481 | 0.0211 | 9.14E-06 | 11775 |
|  | rs1375955 | 8 | 106555039 | T | G | 0.0613 | 0.0121 | 0.4013 | 4.06E-07 | 14288 |
|  | rs7026771 | 9 | 133854336 | A | G | 0.0691 | 0.0148 | 0.5682 | 3.03E-06 | 12403 |
|  | rs12217463 | 10 | 16318694 | T | C | -0.1374 | 0.031 | 0.9572 | 9.33E-06 | 14736 |
|  | rs10734292 | 11 | 20486401 | A | G | 0.1208 | 0.0267 | 0.067 | 6.06E-06 | 11776 |
|  | rs76213792 | 12 | 17798114 | T | G | -0.2791 | 0.0616 | 0.0113 | 5.87E-06 | 14288 |
|  | rs11171132 | 12 | 55251385 | T | C | -0.0724 | 0.0163 | 0.8342 | 8.92E-06 | 14288 |
|  | rs12321566 | 12 | 97807415 | A | G | 0.0698 | 0.0139 | 0.237 | 5.12E-07 | 14287 |
|  | rs11158239 | 14 | 59452945 | T | G | 0.0965 | 0.0217 | 0.0814 | 8.71E-06 | 14288 |
|  | rs79072837 | 15 | 81321256 | T | C | -0.136 | 0.028 | 0.0497 | 1.19E-06 | 14715 |
|  | rs10403201 | 19 | 14764810 | A | G | -0.0584 | 0.0127 | 0.2923 | 4.26E-06 | 14730 |
| IL-8 | rs75579654 | 1 | 46373240 | A | G | 0.1025 | 0.0231 | 0.0739 | 9.11E-06 | 14728 |
|  | rs12075 | 1 | 159175354 | A | G | 0.0667 | 0.0116 | 0.5552 | 8.92E-09 | 14741 |
|  | rs144815207 | 1 | 233542010 | T | C | 0.3186 | 0.0622 | 0.9879 | 3.02E-07 | 13394 |
|  | rs6709159 | 2 | 108946178 | A | G | 0.0562 | 0.0117 | 0.4424 | 1.56E-06 | 14744 |
|  | rs13064825 | 3 | 16705058 | T | C | 0.056 | 0.012 | 0.4104 | 3.06E-06 | 14296 |
|  | rs1516478 | 3 | 76218188 | T | C | 0.1479 | 0.0322 | 0.0386 | 4.37E-06 | 14741 |
|  | rs11712240 | 3 | 132203683 | T | C | 0.0932 | 0.0206 | 0.1151 | 6.06E-06 | 11777 |
|  | rs141979722 | 3 | 172877373 | T | C | 0.2829 | 0.0608 | 0.0146 | 3.27E-06 | 11669 |
|  | rs6446951 | 4 | 74574265 | A | G | -0.1894 | 0.0241 | 0.0665 | 3.87E-15 | 14729 |
|  | rs72710634 | 5 | 2106405 | T | C | 0.1432 | 0.0322 | 0.0447 | 8.70E-06 | 14744 |
|  | rs972492 | 5 | 37903927 | A | C | -0.0936 | 0.0184 | 0.1404 | 3.64E-07 | 12412 |
|  | rs12188297 | 5 | 120608739 | A | T | -0.0755 | 0.0147 | 0.2006 | 2.81E-07 | 14740 |
|  | rs141384565 | 6 | 2140801 | A | G | -0.3943 | 0.0886 | 0.0095 | 8.57E-06 | 11331 |
|  | rs145125644 | 7 | 17688229 | A | G | 0.2687 | 0.0555 | 0.9866 | 1.29E-06 | 14733 |
|  | rs7458637 | 7 | 57105100 | A | G | 0.0948 | 0.0213 | 0.7166 | 8.56E-06 | 9830 |
|  | rs2466352 | 8 | 133415401 | C | G | -0.0957 | 0.0212 | 0.9125 | 6.36E-06 | 14730 |
|  | rs12217463 | 10 | 16318694 | T | C | -0.1365 | 0.0308 | 0.9559 | 9.34E-06 | 14744 |
|  | rs6585420 | 10 | 118629449 | T | C | 0.1545 | 0.0325 | 0.9598 | 2.00E-06 | 14744 |
|  | rs4769882 | 13 | 31352538 | T | C | -0.1103 | 0.0249 | 0.94 | 9.44E-06 | 14744 |
|  | rs72633250 | 15 | 101649801 | A | C | 0.0574 | 0.0124 | 0.4025 | 3.67E-06 | 14296 |
|  | rs76149515 | 16 | 4804879 | T | C | -0.1546 | 0.0348 | 0.9689 | 8.89E-06 | 14741 |
|  | rs370192906 | 16 | 22653353 | A | G | 0.389 | 0.0875 | 0.9886 | 8.76E-06 | 9893 |
|  | rs1582581 | 16 | 60551242 | A | G | -0.0541 | 0.0119 | 0.393 | 5.46E-06 | 14730 |
|  | rs1988503 | 17 | 11189278 | T | C | 0.0548 | 0.0121 | 0.5814 | 5.93E-06 | 14296 |
|  | rs8069322 | 17 | 54450134 | A | G | 0.0668 | 0.0131 | 0.7222 | 3.41E-07 | 14726 |
|  | rs7216538 | 17 | 73572798 | A | G | -0.1324 | 0.0279 | 0.0541 | 2.08E-06 | 13672 |
|  | rs34519991 | 18 | 12258051 | T | C | 0.0555 | 0.012 | 0.5073 | 3.75E-06 | 14296 |
|  | rs72886502 | 18 | 36692976 | T | G | -0.3895 | 0.076 | 0.0095 | 2.98E-07 | 12291 |
|  | rs2612387 | 18 | 73584415 | T | G | 0.2945 | 0.0651 | 0.0101 | 6.07E-06 | 12935 |
|  | rs906401 | 18 | 73700336 | T | C | 0.0941 | 0.02 | 0.9034 | 2.54E-06 | 14729 |
|  | rs71337505 | 19 | 34248514 | A | G | 0.0948 | 0.0209 | 0.1019 | 5.74E-06 | 14563 |
| LAP TGF β1 | rs4659810 | 1 | 237983229 | A | G | -0.0551 | 0.0121 | 0.3438 | 5.27E-06 | 14710 |
|  | rs7570818 | 2 | 107310081 | A | G | -0.0849 | 0.0183 | 0.8857 | 3.50E-06 | 14729 |
|  | rs7656674 | 4 | 5793052 | A | T | 0.0626 | 0.0121 | 0.5957 | 2.30E-07 | 14288 |
|  | rs6827317 | 4 | 10683051 | A | G | 0.2159 | 0.048 | 0.0172 | 6.86E-06 | 14736 |
|  | rs73112514 | 4 | 26142254 | C | G | 0.2053 | 0.0433 | 0.9689 | 2.12E-06 | 12393 |
|  | rs1921885 | 4 | 84952443 | A | C | 0.1265 | 0.0263 | 0.9391 | 1.51E-06 | 14288 |
|  | rs114152855 | 4 | 175537640 | T | C | -0.299 | 0.0613 | 0.0112 | 1.07E-06 | 14249 |
|  | rs58801460 | 5 | 11362066 | A | G | 0.0964 | 0.0216 | 0.081 | 8.08E-06 | 14736 |
|  | rs2631367 | 5 | 131705458 | C | G | -0.0605 | 0.013 | 0.4529 | 3.26E-06 | 11785 |
|  | rs34992551 | 7 | 16406570 | T | C | -0.0931 | 0.0199 | 0.1103 | 2.89E-06 | 14734 |
|  | rs201799031 | 7 | 113796203 | C | G | 0.1918 | 0.0427 | 0.9725 | 7.06E-06 | 11760 |
|  | rs2525703 | 7 | 144100595 | T | C | 0.0668 | 0.0151 | 0.1815 | 9.70E-06 | 14723 |
|  | rs2365502 | 7 | 156395495 | A | G | 0.1321 | 0.029 | 0.9369 | 5.23E-06 | 11777 |
|  | rs143420877 | 8 | 51696312 | T | C | 0.1447 | 0.0303 | 0.0388 | 1.79E-06 | 14729 |
|  | rs7820135 | 8 | 68719454 | T | C | -0.0747 | 0.0155 | 0.8321 | 1.44E-06 | 14730 |
|  | rs7013321 | 8 | 106593558 | A | G | -0.0608 | 0.0121 | 0.4469 | 5.04E-07 | 14288 |
|  | rs183319799 | 9 | 87111083 | T | G | 0.2185 | 0.0461 | 0.0182 | 2.14E-06 | 14733 |
|  | rs10508556 | 10 | 18285342 | T | C | 0.0523 | 0.0114 | 0.4627 | 4.48E-06 | 14735 |
|  | rs111848253 | 11 | 22351553 | A | G | -0.1111 | 0.0251 | 0.9044 | 9.59E-06 | 11538 |
|  | rs34536806 | 11 | 95274487 | T | G | 0.0989 | 0.021 | 0.9023 | 2.48E-06 | 14288 |
|  | rs144834546 | 12 | 24809427 | T | C | -0.2662 | 0.0526 | 0.0227 | 4.17E-07 | 12935 |
|  | rs60821320 | 12 | 95312882 | A | C | -0.1083 | 0.0232 | 0.0779 | 3.04E-06 | 12852 |
|  | rs4911055 | 13 | 85826913 | T | C | -0.0607 | 0.0137 | 0.6556 | 9.39E-06 | 11783 |
|  | rs371119188 | 13 | 112876202 | T | C | 0.1588 | 0.0358 | 0.0406 | 9.18E-06 | 12189 |
|  | rs149458216 | 16 | 48902293 | A | C | -0.2301 | 0.0472 | 0.0182 | 1.09E-06 | 14726 |
|  | rs28535005 | 16 | 86330952 | A | T | -0.0548 | 0.0122 | 0.4227 | 7.06E-06 | 14288 |
|  | rs734515 | 17 | 9112300 | A | G | -0.0594 | 0.0133 | 0.7486 | 7.96E-06 | 14715 |
|  | rs56025355 | 17 | 62499163 | T | C | 0.3006 | 0.0666 | 0.0106 | 6.38E-06 | 12968 |
|  | rs10403201 | 19 | 14764810 | A | G | -0.0597 | 0.0125 | 0.292 | 1.79E-06 | 14730 |
|  | rs73045256 | 19 | 41811072 | A | G | -0.1921 | 0.0276 | 0.9378 | 3.40E-12 | 14288 |
|  | rs1800472 | 19 | 41847860 | A | G | -0.4623 | 0.0335 | 0.0345 | 2.55E-43 | 14736 |
|  | rs7247630 | 19 | 41981195 | T | C | 0.1378 | 0.0271 | 0.0525 | 3.68E-07 | 14729 |
|  | rs4919925 | 21 | 43119625 | T | C | 0.0561 | 0.0124 | 0.3696 | 6.06E-06 | 14287 |
|  | rs2281119 | 22 | 45002724 | T | C | -0.0873 | 0.019 | 0.1204 | 4.33E-06 | 14284 |
| LIF | rs4326609 | 1 | 244347370 | A | T | -0.0707 | 0.0155 | 0.7435 | 5.08E-06 | 11345 |
|  | rs11127419 | 2 | 3091471 | T | C | 0.0707 | 0.0153 | 0.3559 | 3.82E-06 | 9252 |
|  | rs80092539 | 2 | 46125903 | A | T | 0.0993 | 0.0213 | 0.8758 | 3.13E-06 | 11345 |
|  | rs825264 | 2 | 222585468 | A | G | -0.2119 | 0.0453 | 0.0321 | 2.90E-06 | 10291 |
|  | rs116794364 | 2 | 224292526 | C | G | -0.3015 | 0.0631 | 0.9851 | 1.77E-06 | 10858 |
|  | rs145344087 | 3 | 58954742 | C | G | 0.2548 | 0.0556 | 0.021 | 4.59E-06 | 10281 |
|  | rs190222173 | 3 | 121028150 | A | C | -0.3456 | 0.0781 | 0.9882 | 9.64E-06 | 9984 |
|  | rs13105239 | 4 | 23472007 | A | C | 0.0575 | 0.013 | 0.5333 | 9.73E-06 | 11766 |
|  | rs4699986 | 5 | 61150440 | T | G | 0.0629 | 0.0139 | 0.6927 | 6.03E-06 | 11792 |
|  | rs142330159 | 5 | 171514639 | A | T | 0.1155 | 0.0258 | 0.0986 | 7.58E-06 | 9436 |
|  | rs9482537 | 6 | 124651086 | C | G | 0.1084 | 0.0243 | 0.9095 | 8.16E-06 | 11345 |
|  | rs9648464 | 7 | 4467714 | T | C | 0.0606 | 0.0135 | 0.4384 | 7.16E-06 | 11345 |
|  | rs111417524 | 7 | 52054682 | T | C | 0.3026 | 0.0674 | 0.0139 | 7.14E-06 | 10701 |
|  | rs117675814 | 7 | 67523386 | T | C | -0.2448 | 0.0544 | 0.018 | 6.80E-06 | 11345 |
|  | rs13264315 | 8 | 109432178 | A | T | 0.0757 | 0.0158 | 0.6575 | 1.66E-06 | 9460 |
|  | rs1534627 | 10 | 13969539 | T | C | 0.06 | 0.0132 | 0.5978 | 5.48E-06 | 11791 |
|  | rs74992559 | 10 | 31490202 | A | G | 0.1725 | 0.0367 | 0.0351 | 2.60E-06 | 11784 |
|  | rs11608349 | 12 | 68026044 | A | T | -0.3084 | 0.0661 | 0.0168 | 3.08E-06 | 9362 |
|  | rs1348602 | 13 | 35704767 | T | G | -0.0782 | 0.0173 | 0.2297 | 6.18E-06 | 9023 |
|  | rs117844161 | 13 | 88387973 | A | G | 0.1842 | 0.0408 | 0.9695 | 6.34E-06 | 11781 |
|  | rs234592 | 14 | 97087097 | A | G | -0.0635 | 0.0136 | 0.5456 | 3.02E-06 | 11345 |
|  | rs12595568 | 15 | 38990803 | T | G | -0.0652 | 0.0146 | 0.2544 | 7.98E-06 | 11792 |
|  | rs10152968 | 15 | 60604436 | A | G | 0.0678 | 0.0153 | 0.7664 | 9.36E-06 | 11792 |
|  | rs147461732 | 15 | 80114266 | A | G | 0.2606 | 0.0583 | 0.0179 | 7.82E-06 | 11345 |
|  | rs150843753 | 16 | 78805709 | T | G | 0.1742 | 0.0385 | 0.9676 | 6.05E-06 | 11779 |
|  | rs7225483 | 17 | 28341679 | T | G | 0.0671 | 0.0143 | 0.486 | 2.70E-06 | 9849 |
|  | rs332426 | 19 | 6801966 | A | G | 0.112 | 0.0238 | 0.0899 | 2.53E-06 | 11789 |
|  | rs11883375 | 19 | 54851842 | A | G | -0.1011 | 0.0197 | 0.1385 | 2.87E-07 | 11776 |
|  | rs62213023 | 21 | 43995327 | T | C | -0.1676 | 0.0374 | 0.0399 | 7.42E-06 | 11345 |
| LIF-R | rs6671847 | 1 | 161478810 | A | G | -0.0604 | 0.0135 | 0.4812 | 7.67E-06 | 11336 |
|  | rs12754806 | 1 | 238821453 | A | G | 0.0563 | 0.0122 | 0.4817 | 3.94E-06 | 11768 |
|  | rs78981063 | 2 | 84195883 | A | G | -0.1377 | 0.0302 | 0.9558 | 5.13E-06 | 11784 |
|  | rs112883732 | 2 | 174802976 | A | G | 0.0639 | 0.0144 | 0.2266 | 9.10E-06 | 11784 |
|  | rs3963364 | 3 | 12385357 | A | C | -0.0951 | 0.0203 | 0.1784 | 2.80E-06 | 9453 |
|  | rs62258077 | 3 | 58363371 | T | C | -0.063 | 0.013 | 0.6603 | 1.26E-06 | 11762 |
|  | rs4689540 | 4 | 6753471 | T | C | 0.0611 | 0.0138 | 0.5834 | 9.53E-06 | 11336 |
|  | rs9313148 | 5 | 6378192 | C | G | -0.0824 | 0.0173 | 0.1414 | 1.91E-06 | 11777 |
|  | rs72730728 | 5 | 36424241 | A | T | 0.1414 | 0.0302 | 0.9403 | 2.84E-06 | 11336 |
|  | rs2289779 | 5 | 38527228 | T | G | 0.0788 | 0.0134 | 0.5419 | 4.09E-09 | 11336 |
|  | rs452976 | 5 | 84611579 | T | C | 0.0692 | 0.0153 | 0.8025 | 6.10E-06 | 11770 |
|  | rs1645151 | 5 | 95049215 | C | G | 0.0745 | 0.0164 | 0.6059 | 5.55E-06 | 8809 |
|  | rs115938660 | 5 | 110784241 | T | C | -0.2181 | 0.0467 | 0.0215 | 3.01E-06 | 11770 |
|  | rs2569006 | 5 | 145246085 | T | G | -0.0553 | 0.0125 | 0.4142 | 9.69E-06 | 11780 |
|  | rs778016 | 6 | 68918580 | A | G | -0.0681 | 0.0145 | 0.2875 | 2.65E-06 | 9883 |
|  | rs73157388 | 7 | 79349730 | T | C | -0.1306 | 0.0285 | 0.9229 | 4.60E-06 | 11336 |
|  | rs1604713 | 8 | 107256388 | A | C | -0.1451 | 0.0322 | 0.9399 | 6.60E-06 | 11336 |
|  | rs1275374 | 8 | 118638630 | A | G | -0.0611 | 0.0137 | 0.4056 | 8.20E-06 | 11336 |
|  | rs117117000 | 8 | 119917062 | A | G | 0.1586 | 0.0333 | 0.961 | 1.91E-06 | 11784 |
|  | rs7047277 | 9 | 14698077 | T | C | 0.0558 | 0.0126 | 0.6218 | 9.49E-06 | 11774 |
|  | rs635634 | 9 | 136155000 | T | C | -0.2885 | 0.0153 | 0.1918 | 2.61E-79 | 11784 |
|  | rs41307428 | 9 | 136336804 | T | C | 0.2027 | 0.0299 | 0.067 | 1.21E-11 | 11774 |
|  | rs1690678 | 10 | 27930818 | A | C | 0.0904 | 0.0192 | 0.1109 | 2.50E-06 | 11784 |
|  | rs1549488 | 11 | 4431867 | C | G | -0.06 | 0.0134 | 0.4942 | 7.55E-06 | 11335 |
|  | rs4937122 | 11 | 126228659 | T | G | 0.1262 | 0.0248 | 0.9129 | 3.61E-07 | 11336 |
|  | rs143212977 | 12 | 115088443 | A | G | 0.2467 | 0.0558 | 0.0184 | 9.82E-06 | 11336 |
|  | rs8059299 | 16 | 9246057 | A | G | 0.1051 | 0.0232 | 0.8941 | 5.89E-06 | 11336 |
|  | rs72837682 | 17 | 7063650 | T | C | -0.08 | 0.0172 | 0.7807 | 3.30E-06 | 11336 |
|  | rs141933232 | 17 | 36964094 | A | G | -0.2724 | 0.0546 | 0.0169 | 6.07E-07 | 11724 |
|  | rs147006060 | 21 | 43780807 | A | G | -0.2602 | 0.0566 | 0.0179 | 4.28E-06 | 11775 |
|  | rs6002001 | 22 | 41054967 | A | G | 0.6489 | 0.1417 | 0.0043 | 4.66E-06 | 6861 |
| MCP-1 | rs12075 | 1 | 159175354 | A | G | 0.1694 | 0.0113 | 0.5552 | 8.39E-51 | 14730 |
|  | rs142018247 | 2 | 114120803 | T | C | 0.1966 | 0.0442 | 0.02 | 8.67E-06 | 14726 |
|  | rs4682862 | 3 | 42877413 | A | G | 0.0576 | 0.0122 | 0.6154 | 2.34E-06 | 14285 |
|  | rs1846616 | 3 | 46032441 | A | T | 0.0856 | 0.0119 | 0.362 | 6.33E-13 | 14719 |
|  | rs35728689 | 3 | 46390228 | A | G | 0.3004 | 0.0198 | 0.0959 | 5.44E-52 | 14732 |
|  | rs181998213 | 3 | 46519788 | T | C | 0.3723 | 0.0695 | 0.9872 | 8.47E-08 | 14285 |
|  | rs9811380 | 3 | 128321226 | A | G | -0.0584 | 0.0131 | 0.7146 | 8.27E-06 | 14284 |
|  | rs7619350 | 3 | 132269435 | A | G | 0.0986 | 0.0201 | 0.118 | 9.32E-07 | 11764 |
|  | rs149604038 | 3 | 185463545 | C | G | -0.2813 | 0.0556 | 0.9851 | 4.21E-07 | 13798 |
|  | rs207311 | 4 | 19075878 | T | C | 0.0579 | 0.0126 | 0.6697 | 4.32E-06 | 14285 |
|  | rs148307902 | 5 | 2885014 | A | G | 0.2553 | 0.0538 | 0.0243 | 2.08E-06 | 13798 |
|  | rs74794857 | 5 | 22143588 | T | C | 0.0614 | 0.0139 | 0.4123 | 1.00E-05 | 11760 |
|  | rs72730728 | 5 | 36424241 | A | T | 0.1238 | 0.0272 | 0.9416 | 5.33E-06 | 14285 |
|  | rs2544916 | 5 | 100158495 | A | T | 0.1653 | 0.0366 | 0.9698 | 6.29E-06 | 14720 |
|  | rs146186562 | 6 | 20408729 | T | G | -0.2293 | 0.0497 | 0.9789 | 3.96E-06 | 14285 |
|  | rs352082 | 6 | 114180646 | C | G | 0.0788 | 0.0178 | 0.1269 | 9.56E-06 | 14284 |
|  | rs9942602 | 7 | 17977054 | T | C | 0.0618 | 0.0117 | 0.4922 | 1.28E-07 | 14728 |
|  | rs182599222 | 8 | 115072929 | A | T | -0.4028 | 0.0864 | 0.991 | 3.13E-06 | 11334 |
|  | rs77344300 | 9 | 78838039 | T | C | -0.1381 | 0.0293 | 0.0491 | 2.44E-06 | 14733 |
|  | rs117510656 | 11 | 87277670 | T | C | -0.2587 | 0.0562 | 0.9806 | 4.16E-06 | 14285 |
|  | rs11214549 | 11 | 113108311 | A | G | -0.1673 | 0.0376 | 0.0305 | 8.61E-06 | 14732 |
|  | rs12367122 | 12 | 30998369 | A | G | 0.0739 | 0.0159 | 0.1756 | 3.35E-06 | 14284 |
|  | rs34292320 | 13 | 43553825 | T | C | -0.0684 | 0.0134 | 0.2742 | 3.32E-07 | 14285 |
|  | rs9324105 | 14 | 29273195 | T | C | -0.0775 | 0.0175 | 0.1395 | 9.49E-06 | 14285 |
|  | rs117265389 | 16 | 4938452 | C | G | 0.1734 | 0.0377 | 0.0256 | 4.24E-06 | 14733 |
|  | rs11870164 | 17 | 32567679 | T | C | 0.0751 | 0.013 | 0.2772 | 7.61E-09 | 14733 |
|  | rs190108694 | 18 | 75133468 | A | G | -0.2084 | 0.0455 | 0.0286 | 4.64E-06 | 11325 |
|  | rs79697698 | 20 | 9086080 | T | C | -0.348 | 0.0653 | 0.0136 | 9.86E-08 | 13752 |
|  | rs77128226 | 20 | 15213518 | T | C | -0.1063 | 0.0218 | 0.0875 | 1.08E-06 | 14285 |
|  | rs1788443 | 21 | 43981957 | T | C | -0.0537 | 0.0119 | 0.5055 | 6.40E-06 | 14285 |
|  | rs117317477 | 22 | 21921920 | T | G | -0.3227 | 0.0704 | 0.0125 | 4.57E-06 | 11725 |
| MCP-2 | rs78743764 | 1 | 3301962 | A | C | -0.2226 | 0.048 | 0.019 | 3.53E-06 | 13656 |
|  | rs12075 | 1 | 159175354 | A | G | -0.0973 | 0.0116 | 0.5553 | 4.95E-17 | 14733 |
|  | rs6690229 | 1 | 175086461 | A | G | 0.0563 | 0.0125 | 0.405 | 6.67E-06 | 13240 |
|  | rs6685547 | 1 | 224882124 | A | G | 0.1462 | 0.0289 | 0.0505 | 4.22E-07 | 13239 |
|  | rs7538845 | 1 | 238880980 | C | G | 0.0566 | 0.0122 | 0.6032 | 3.50E-06 | 14288 |
|  | rs79965992 | 2 | 43748079 | A | G | 0.1092 | 0.0233 | 0.0823 | 2.78E-06 | 12851 |
|  | rs7557474 | 2 | 230344423 | T | C | -0.0805 | 0.0179 | 0.8519 | 6.89E-06 | 12851 |
|  | rs138884871 | 3 | 47071457 | T | C | 0.2198 | 0.0473 | 0.0188 | 3.37E-06 | 13672 |
|  | rs62320291 | 4 | 106578139 | C | G | 0.1198 | 0.027 | 0.0668 | 9.12E-06 | 14288 |
|  | rs77635208 | 4 | 139812379 | T | C | 0.1467 | 0.0331 | 0.9627 | 9.34E-06 | 14717 |
|  | rs2548023 | 5 | 33831973 | A | G | -0.076 | 0.0169 | 0.2077 | 6.89E-06 | 11773 |
|  | rs407595 | 5 | 125099527 | A | C | 0.0697 | 0.0147 | 0.2073 | 2.12E-06 | 14287 |
|  | rs145516279 | 5 | 147351915 | A | G | 0.2225 | 0.048 | 0.979 | 3.56E-06 | 14288 |
|  | rs33190 | 5 | 158103877 | A | T | 0.0636 | 0.0142 | 0.5779 | 7.50E-06 | 12403 |
|  | rs12523126 | 5 | 165649355 | A | T | 0.1121 | 0.0232 | 0.0746 | 1.35E-06 | 14288 |
|  | rs145066788 | 6 | 77535889 | T | C | -0.3035 | 0.0685 | 0.0089 | 9.39E-06 | 13801 |
|  | rs142168130 | 6 | 157080026 | A | G | 0.2289 | 0.0502 | 0.0176 | 5.12E-06 | 14288 |
|  | rs79567569 | 6 | 167056258 | A | C | -0.3284 | 0.0669 | 0.9877 | 9.16E-07 | 13386 |
|  | rs4722985 | 7 | 30472091 | T | C | -0.0626 | 0.0133 | 0.709 | 2.52E-06 | 14288 |
|  | rs200967074 | 8 | 14666417 | T | G | 0.1113 | 0.0251 | 0.922 | 9.24E-06 | 12201 |
|  | rs181726805 | 9 | 95657986 | A | G | -0.2479 | 0.0536 | 0.9838 | 3.75E-06 | 14736 |
|  | rs4639861 | 10 | 1806711 | A | G | 0.0796 | 0.0177 | 0.1379 | 6.89E-06 | 14287 |
|  | rs72804061 | 10 | 73686196 | A | G | 0.2066 | 0.0461 | 0.9801 | 7.41E-06 | 13870 |
|  | rs4881851 | 11 | 51591666 | T | C | 0.127 | 0.0286 | 0.1154 | 8.97E-06 | 8333 |
|  | rs948962 | 11 | 76919478 | A | C | 0.0544 | 0.0119 | 0.4764 | 4.84E-06 | 14736 |
|  | rs143648571 | 11 | 92627436 | C | G | -0.2479 | 0.0554 | 0.0172 | 7.65E-06 | 13801 |
|  | rs75573205 | 11 | 101424282 | C | G | 0.1906 | 0.0408 | 0.976 | 2.99E-06 | 14725 |
|  | rs73178812 | 13 | 37832221 | T | C | 0.1047 | 0.0232 | 0.0744 | 6.39E-06 | 14288 |
|  | rs147984424 | 14 | 105233374 | A | G | -0.4143 | 0.0934 | 0.9888 | 9.17E-06 | 8829 |
|  | rs2001616 | 15 | 45404066 | A | G | 0.089 | 0.0197 | 0.8982 | 6.25E-06 | 14735 |
|  | rs112168535 | 16 | 4704769 | T | C | -0.1553 | 0.0316 | 0.9555 | 8.90E-07 | 13670 |
|  | rs11861137 | 16 | 17250485 | T | C | -0.0537 | 0.0121 | 0.3884 | 9.08E-06 | 14729 |
|  | rs9889517 | 17 | 4704175 | A | G | 0.0609 | 0.0121 | 0.4783 | 4.83E-07 | 14288 |
|  | rs55653410 | 17 | 32505814 | A | T | -0.1736 | 0.0333 | 0.042 | 1.86E-07 | 14288 |
|  | rs41274294 | 17 | 32615171 | T | C | 0.8816 | 0.0356 | 0.9682 | 2.19E-135 | 14732 |
|  | rs12451323 | 17 | 32634098 | T | C | 1.0249 | 0.0174 | 0.8839 | 0.00E+00 | 14288 |
|  | rs144618566 | 17 | 32652307 | T | C | 0.5171 | 0.0538 | 0.0169 | 7.15E-22 | 13801 |
|  | rs72827004 | 17 | 32669907 | C | G | 0.2617 | 0.0317 | 0.0456 | 1.51E-16 | 14728 |
|  | rs62062305 | 17 | 32751484 | T | G | 0.0817 | 0.0168 | 0.8553 | 1.16E-06 | 14288 |
|  | rs35904471 | 17 | 76944702 | T | C | 0.0761 | 0.0165 | 0.2662 | 3.99E-06 | 11337 |
|  | rs12326052 | 18 | 6588865 | T | C | -0.1199 | 0.0267 | 0.9372 | 7.10E-06 | 14288 |
|  | rs80131163 | 18 | 31677370 | A | G | 0.1962 | 0.0399 | 0.9686 | 8.77E-07 | 14730 |
|  | rs141574903 | 18 | 65682390 | A | C | -0.2158 | 0.0487 | 0.981 | 9.37E-06 | 14243 |
|  | rs75580960 | 19 | 2151761 | T | C | -0.0904 | 0.0188 | 0.8703 | 1.52E-06 | 13224 |
|  | rs74350159 | 21 | 36741897 | A | G | 0.2798 | 0.0608 | 0.012 | 4.19E-06 | 13801 |
|  | rs79097546 | 22 | 19662319 | T | C | -0.258 | 0.0569 | 0.9864 | 5.78E-06 | 14249 |
| MCP-3 | rs481267 | 1 | 108717820 | A | G | -0.0754 | 0.015 | 0.3263 | 4.99E-07 | 9889 |
|  | rs12075 | 1 | 159175354 | A | G | -0.0899 | 0.013 | 0.5624 | 4.67E-12 | 11780 |
|  | rs12142504 | 1 | 192799485 | T | C | 0.0778 | 0.0166 | 0.6951 | 2.78E-06 | 9448 |
|  | rs147793446 | 1 | 201769310 | A | G | 0.1941 | 0.0413 | 0.0345 | 2.60E-06 | 11770 |
|  | rs72793575 | 2 | 23771966 | A | G | 0.1043 | 0.0235 | 0.1037 | 9.07E-06 | 11335 |
|  | rs146174820 | 2 | 68614232 | A | G | 0.2663 | 0.0547 | 0.0247 | 1.13E-06 | 10469 |
|  | rs2228467 | 3 | 42906116 | T | C | -0.3169 | 0.0252 | 0.9268 | 2.88E-36 | 11782 |
|  | rs4146770 | 3 | 151048544 | T | C | -0.0933 | 0.0165 | 0.7978 | 1.56E-08 | 11760 |
|  | rs11276535 | 3 | 177062154 | T | C | -0.0849 | 0.0167 | 0.5118 | 3.70E-07 | 7943 |
|  | rs116170440 | 4 | 36138371 | A | G | -0.257 | 0.0579 | 0.0167 | 9.05E-06 | 10848 |
|  | rs28667091 | 4 | 47682997 | T | G | -0.0613 | 0.0133 | 0.4117 | 4.05E-06 | 11782 |
|  | rs182239847 | 4 | 144892144 | T | G | -0.1716 | 0.0359 | 0.9096 | 1.75E-06 | 9453 |
|  | rs72689930 | 4 | 158925763 | T | G | 0.1624 | 0.0363 | 0.9529 | 7.68E-06 | 11783 |
|  | rs153473 | 5 | 150634915 | C | G | -0.0638 | 0.0134 | 0.4563 | 1.92E-06 | 11335 |
|  | rs9968876 | 6 | 100087282 | T | G | -0.1046 | 0.0221 | 0.8991 | 2.21E-06 | 11774 |
|  | rs12661848 | 6 | 123906471 | T | G | 0.0655 | 0.0147 | 0.5602 | 8.36E-06 | 9889 |
|  | rs28571854 | 9 | 16472947 | T | C | -0.1098 | 0.0247 | 0.0785 | 8.78E-06 | 11775 |
|  | rs139796674 | 9 | 110584352 | A | G | 0.2334 | 0.0492 | 0.0249 | 2.10E-06 | 11335 |
|  | rs74157668 | 10 | 121143312 | T | C | 0.1574 | 0.0343 | 0.0404 | 4.46E-06 | 11781 |
|  | rs148859911 | 11 | 42245173 | A | G | 0.1507 | 0.0328 | 0.05 | 4.34E-06 | 11771 |
|  | rs7116054 | 11 | 68891695 | T | C | -0.0998 | 0.0179 | 0.1822 | 2.47E-08 | 11335 |
|  | rs143052555 | 12 | 117637971 | T | C | 0.1051 | 0.0233 | 0.8213 | 6.46E-06 | 8587 |
|  | rs7327202 | 13 | 58931726 | C | G | -0.0969 | 0.0194 | 0.1497 | 5.89E-07 | 10903 |
|  | rs117550373 | 14 | 76638061 | T | G | -0.1405 | 0.0314 | 0.0501 | 7.66E-06 | 11762 |
|  | rs28439856 | 15 | 53643049 | A | G | 0.1646 | 0.0339 | 0.9169 | 1.20E-06 | 7943 |
|  | rs16947053 | 15 | 93092575 | A | G | 0.2264 | 0.0487 | 0.9779 | 3.34E-06 | 11783 |
|  | rs187435509 | 16 | 4844729 | T | G | 0.171 | 0.0381 | 0.0356 | 7.18E-06 | 11780 |
|  | rs7213460 | 17 | 32522613 | A | G | 0.0942 | 0.0132 | 0.4161 | 9.58E-13 | 11780 |
|  | rs72939501 | 18 | 60781974 | A | G | -0.1014 | 0.0226 | 0.1082 | 7.23E-06 | 11335 |
|  | rs200447680 | 20 | 15607887 | A | C | 0.1863 | 0.041 | 0.9612 | 5.52E-06 | 10235 |
|  | rs78848134 | 20 | 41126657 | T | C | 0.2441 | 0.0514 | 0.0198 | 2.04E-06 | 11296 |
|  | rs8129542 | 21 | 45846823 | A | T | -0.1022 | 0.0228 | 0.1218 | 7.38E-06 | 10271 |
| MCP-4 | rs12075 | 1 | 159175354 | A | G | 0.189 | 0.0114 | 0.5553 | 9.89E-62 | 14733 |
|  | rs148945704 | 2 | 160090389 | C | G | -0.2016 | 0.0456 | 0.0179 | 9.82E-06 | 14232 |
|  | rs56003568 | 2 | 203491699 | C | G | 0.0569 | 0.0126 | 0.2956 | 6.31E-06 | 14734 |
|  | rs7612912 | 3 | 42910621 | T | C | 0.1281 | 0.012 | 0.6276 | 1.33E-26 | 14714 |
|  | rs55842014 | 3 | 46289113 | A | G | -0.0638 | 0.0131 | 0.3598 | 1.11E-06 | 12404 |
|  | rs1995234 | 3 | 157473371 | A | T | -0.058 | 0.0129 | 0.4227 | 6.92E-06 | 12404 |
|  | rs2192410 | 3 | 172370658 | C | G | 0.072 | 0.0158 | 0.1934 | 5.19E-06 | 12836 |
|  | rs59093248 | 4 | 14288151 | T | C | -0.0664 | 0.0148 | 0.4726 | 7.24E-06 | 10894 |
|  | rs115511138 | 4 | 115566991 | A | G | 0.1956 | 0.042 | 0.9694 | 3.21E-06 | 13944 |
|  | rs144982290 | 4 | 153541434 | T | C | 0.2271 | 0.05 | 0.025 | 5.57E-06 | 13801 |
|  | rs62336911 | 4 | 176433708 | T | C | 0.1569 | 0.0332 | 0.0417 | 2.29E-06 | 14288 |
|  | rs74794857 | 5 | 22143588 | T | C | 0.0674 | 0.0139 | 0.4123 | 1.24E-06 | 11760 |
|  | rs2631360 | 5 | 131707429 | A | G | 0.0609 | 0.0131 | 0.5448 | 3.34E-06 | 11782 |
|  | rs144118870 | 6 | 31214807 | A | G | 0.1749 | 0.0368 | 0.963 | 2.01E-06 | 14735 |
|  | rs847767 | 6 | 40051588 | A | G | 0.077 | 0.0168 | 0.8533 | 4.58E-06 | 14733 |
|  | rs1120252 | 6 | 57282260 | A | T | 0.0873 | 0.019 | 0.5592 | 4.33E-06 | 9830 |
|  | rs2721961 | 8 | 116657911 | T | G | -0.1201 | 0.0133 | 0.7293 | 1.72E-19 | 14288 |
|  | rs438824 | 9 | 102016891 | A | G | -0.0548 | 0.0121 | 0.4985 | 5.93E-06 | 14287 |
|  | rs2497351 | 10 | 94520346 | T | C | 0.0668 | 0.0145 | 0.2018 | 4.09E-06 | 14736 |
|  | rs72832833 | 10 | 117669867 | A | G | -0.0635 | 0.0143 | 0.7811 | 8.97E-06 | 14734 |
|  | rs4980395 | 11 | 1876594 | T | C | 0.0748 | 0.0164 | 0.184 | 5.09E-06 | 14288 |
|  | rs113911480 | 14 | 29113167 | T | G | -0.1502 | 0.0333 | 0.9632 | 6.47E-06 | 14709 |
|  | rs10140592 | 14 | 70451473 | A | G | 0.2704 | 0.0553 | 0.0209 | 1.01E-06 | 13753 |
|  | rs114438927 | 15 | 30072182 | A | G | 0.2261 | 0.05 | 0.0163 | 6.13E-06 | 12852 |
|  | rs34202150 | 16 | 24019294 | T | C | 0.08 | 0.0181 | 0.8698 | 9.88E-06 | 14288 |
|  | rs62055193 | 16 | 74410477 | C | G | 0.093 | 0.0196 | 0.2384 | 2.09E-06 | 9830 |
|  | rs61578494 | 17 | 32535620 | C | G | -0.1675 | 0.0238 | 0.0674 | 1.95E-12 | 14728 |
|  | rs3917900 | 17 | 32587314 | A | G | 0.1557 | 0.012 | 0.6401 | 1.70E-38 | 14730 |
|  | rs3136676 | 17 | 32683289 | A | G | 0.3903 | 0.0251 | 0.9384 | 1.60E-54 | 14728 |
|  | rs181250 | 17 | 56210459 | T | C | -0.1032 | 0.0208 | 0.8805 | 6.99E-07 | 11538 |
|  | rs62201455 | 20 | 12689462 | A | C | -0.2136 | 0.0451 | 0.9807 | 2.18E-06 | 14722 |
|  | rs926646 | 20 | 20023326 | T | C | 0.1432 | 0.0304 | 0.9545 | 2.47E-06 | 14732 |
| MIP-1 α | rs7537266 | 1 | 238391155 | T | C | 0.0615 | 0.0137 | 0.2426 | 7.15E-06 | 14294 |
|  | rs66822987 | 3 | 63106417 | T | C | 0.0653 | 0.0144 | 0.2284 | 5.77E-06 | 14295 |
|  | rs5743604 | 4 | 38801285 | A | G | -0.0736 | 0.0135 | 0.7503 | 4.98E-08 | 14737 |
|  | rs4861445 | 4 | 181734829 | A | G | 0.0588 | 0.013 | 0.3744 | 6.09E-06 | 14295 |
|  | rs2853926 | 6 | 31263051 | A | G | -0.0608 | 0.0133 | 0.7392 | 4.84E-06 | 14743 |
|  | rs113448118 | 7 | 1108954 | A | G | 0.0818 | 0.0179 | 0.1528 | 4.88E-06 | 11793 |
|  | rs188443128 | 8 | 56763360 | T | C | -0.0718 | 0.0151 | 0.4638 | 1.98E-06 | 10893 |
|  | rs1295911 | 10 | 12075632 | A | G | -0.087 | 0.0193 | 0.8805 | 6.55E-06 | 12799 |
|  | rs7917198 | 10 | 93675891 | T | G | -0.0706 | 0.0159 | 0.8397 | 8.99E-06 | 14731 |
|  | rs1125560 | 12 | 67790676 | T | C | -0.0644 | 0.0124 | 0.632 | 2.06E-07 | 13863 |
|  | rs7137828 | 12 | 111932800 | T | C | -0.0837 | 0.0133 | 0.5247 | 3.11E-10 | 11344 |
|  | rs112449949 | 12 | 130276202 | T | C | -0.1932 | 0.0434 | 0.0345 | 8.52E-06 | 13394 |
|  | rs9540456 | 13 | 66085374 | A | G | 0.0926 | 0.0181 | 0.8776 | 3.12E-07 | 14295 |
|  | rs147065959 | 15 | 28291907 | A | C | 0.302 | 0.068 | 0.985 | 8.95E-06 | 11438 |
|  | rs78871521 | 16 | 4774017 | T | C | 0.1812 | 0.0371 | 0.0304 | 1.04E-06 | 13246 |
|  | rs1143687 | 16 | 81922813 | T | C | -0.1229 | 0.024 | 0.0627 | 3.04E-07 | 14743 |
|  | rs677925 | 17 | 15741465 | T | C | -0.0668 | 0.0143 | 0.7284 | 2.99E-06 | 12411 |
|  | rs56675286 | 17 | 34358060 | A | C | -0.1806 | 0.0325 | 0.0383 | 2.75E-08 | 14743 |
|  | rs8951 | 17 | 34415720 | T | C | -0.4102 | 0.0135 | 0.7695 | 8.62E-203 | 14295 |
|  | rs76522981 | 17 | 34450780 | T | C | -0.1462 | 0.0253 | 0.076 | 7.53E-09 | 14295 |
|  | rs9911791 | 17 | 34820996 | A | G | 0.176 | 0.0162 | 0.8208 | 1.71E-27 | 14295 |
|  | rs2847249 | 18 | 12747235 | T | C | -0.0621 | 0.0136 | 0.2605 | 4.97E-06 | 14294 |
| MMP-1 | rs149819211 | 1 | 21336393 | A | G | -0.1601 | 0.0351 | 0.0417 | 5.08E-06 | 14744 |
|  | rs12122036 | 1 | 109101434 | A | T | -0.1077 | 0.024 | 0.0741 | 7.21E-06 | 14296 |
|  | rs12141791 | 1 | 156419786 | A | G | 0.0859 | 0.013 | 0.6981 | 3.90E-11 | 14296 |
|  | rs72777396 | 2 | 10923106 | A | G | 0.1657 | 0.0364 | 0.9671 | 5.31E-06 | 14744 |
|  | rs6717478 | 2 | 63657053 | A | T | 0.1548 | 0.0338 | 0.966 | 4.65E-06 | 14742 |
|  | rs2229629 | 2 | 75116527 | A | G | -0.3153 | 0.0673 | 0.0135 | 2.80E-06 | 10887 |
|  | rs189173367 | 3 | 8761961 | T | C | 0.3179 | 0.0714 | 0.0131 | 8.49E-06 | 11925 |
|  | rs873675 | 3 | 64991445 | T | C | 0.1234 | 0.0234 | 0.0684 | 1.34E-07 | 14742 |
|  | rs188626385 | 7 | 120591120 | T | C | -0.2509 | 0.053 | 0.0177 | 2.20E-06 | 12935 |
|  | rs4734879 | 8 | 106583124 | A | G | 0.1136 | 0.0137 | 0.7422 | 1.11E-16 | 14296 |
|  | rs12380451 | 9 | 9191014 | T | C | -0.0593 | 0.0133 | 0.7098 | 8.25E-06 | 14296 |
|  | rs372555709 | 9 | 87142351 | T | C | 0.2514 | 0.0566 | 0.0194 | 8.93E-06 | 11282 |
|  | rs10990535 | 9 | 99091009 | T | C | 0.0703 | 0.0135 | 0.253 | 1.91E-07 | 14734 |
|  | rs10978641 | 9 | 109554196 | A | T | 0.0649 | 0.014 | 0.7462 | 3.56E-06 | 14296 |
|  | rs10732827 | 10 | 85891907 | A | G | 0.0558 | 0.0124 | 0.3498 | 6.80E-06 | 14742 |
|  | rs141782294 | 11 | 102354473 | A | G | 0.3767 | 0.0445 | 0.9777 | 2.56E-17 | 14731 |
|  | rs77095316 | 11 | 102572180 | T | G | -0.1416 | 0.0238 | 0.0705 | 2.69E-09 | 14744 |
|  | rs2155053 | 11 | 102604607 | T | C | -0.3212 | 0.0264 | 0.9461 | 4.68E-34 | 14741 |
|  | rs484915 | 11 | 102673248 | A | T | -0.3517 | 0.0119 | 0.5613 | 5.73E-192 | 14295 |
|  | rs116889819 | 11 | 102806398 | A | G | -0.176 | 0.03 | 0.0455 | 4.45E-09 | 14744 |
|  | rs148908824 | 12 | 24806965 | A | G | -0.2306 | 0.0517 | 0.0214 | 8.18E-06 | 12935 |
|  | rs2926741 | 12 | 57098597 | T | C | -0.0704 | 0.0126 | 0.3436 | 2.31E-08 | 14296 |
|  | rs12367368 | 12 | 83594043 | T | G | 0.0861 | 0.0186 | 0.1146 | 3.67E-06 | 14744 |
|  | rs116350236 | 12 | 124668322 | A | T | 0.0639 | 0.0142 | 0.3845 | 6.80E-06 | 10717 |
|  | rs9524612 | 13 | 95429892 | T | C | -0.072 | 0.0162 | 0.177 | 8.81E-06 | 14296 |
|  | rs7219205 | 17 | 4351312 | A | G | 0.0569 | 0.0121 | 0.5023 | 2.57E-06 | 14296 |
|  | rs56215832 | 18 | 11644433 | C | G | -0.0671 | 0.015 | 0.5715 | 7.70E-06 | 11767 |
|  | rs11673125 | 19 | 14763748 | T | C | -0.0585 | 0.0127 | 0.296 | 4.10E-06 | 14741 |
|  | rs58811245 | 20 | 60732102 | T | C | -0.1051 | 0.0234 | 0.9266 | 7.07E-06 | 14723 |
| MMP-10 | rs12567824 | 1 | 159990022 | A | C | 0.07 | 0.0153 | 0.822 | 4.76E-06 | 14296 |
|  | rs1356799 | 2 | 140567666 | T | G | -0.069 | 0.015 | 0.7974 | 4.22E-06 | 13869 |
|  | rs7683424 | 4 | 187115945 | A | G | 0.054 | 0.0121 | 0.4046 | 8.09E-06 | 14296 |
|  | rs80346512 | 5 | 668045 | A | G | -0.4022 | 0.0779 | 0.982 | 2.43E-07 | 8534 |
|  | rs181816009 | 6 | 31423412 | A | G | -0.1769 | 0.0358 | 0.0368 | 7.76E-07 | 11985 |
|  | rs41285646 | 6 | 32551603 | A | G | 0.106 | 0.0208 | 0.2799 | 3.47E-07 | 8978 |
|  | rs112062276 | 6 | 134160498 | T | G | 0.1203 | 0.0249 | 0.0601 | 1.36E-06 | 14733 |
|  | rs138847931 | 6 | 167150835 | T | C | -0.3484 | 0.0769 | 0.9866 | 5.88E-06 | 11439 |
|  | rs11771706 | 7 | 13720685 | A | C | 0.2157 | 0.0482 | 0.9813 | 7.64E-06 | 14744 |
|  | rs112327130 | 8 | 77953661 | C | G | -0.2862 | 0.0608 | 0.0153 | 2.51E-06 | 12456 |
|  | rs55942631 | 8 | 99434029 | T | G | 0.1032 | 0.023 | 0.9147 | 7.22E-06 | 14296 |
|  | rs72699188 | 9 | 6176871 | C | G | -0.0931 | 0.0166 | 0.8507 | 2.04E-08 | 14744 |
|  | rs10971521 | 9 | 33470408 | A | G | -0.0585 | 0.0132 | 0.2739 | 9.34E-06 | 14725 |
|  | rs112966738 | 9 | 81249363 | T | C | 0.2201 | 0.0486 | 0.9801 | 5.93E-06 | 14296 |
|  | rs34387740 | 9 | 136375396 | A | G | 0.0845 | 0.019 | 0.1751 | 8.69E-06 | 11345 |
|  | rs55804377 | 10 | 1063892 | A | G | -0.2185 | 0.0469 | 0.0185 | 3.18E-06 | 14741 |
|  | rs11225354 | 11 | 102510046 | T | C | 0.2957 | 0.0181 | 0.879 | 5.38E-60 | 14296 |
|  | rs17860955 | 11 | 102649482 | T | C | 1.1371 | 0.047 | 0.983 | 2.60E-129 | 14256 |
|  | rs17099622 | 11 | 102700352 | A | G | -0.2064 | 0.0233 | 0.9296 | 8.12E-19 | 14296 |
|  | rs142915220 | 11 | 102881952 | A | G | -0.3256 | 0.0494 | 0.9794 | 4.37E-11 | 13809 |
|  | rs7966257 | 12 | 111251437 | C | G | -0.0819 | 0.0178 | 0.1335 | 4.20E-06 | 14295 |
|  | rs192820441 | 12 | 117560675 | A | G | 0.2701 | 0.0603 | 0.9874 | 7.49E-06 | 13394 |
|  | rs3742926 | 14 | 33014869 | T | C | -0.0912 | 0.0196 | 0.0978 | 3.27E-06 | 14744 |
|  | rs2584662 | 17 | 47470487 | A | C | -0.0597 | 0.0122 | 0.585 | 9.91E-07 | 14296 |
|  | rs516316 | 19 | 49206145 | C | G | 0.1003 | 0.0117 | 0.4452 | 1.01E-17 | 14742 |
|  | rs113958571 | 20 | 20711695 | A | G | -0.2315 | 0.0503 | 0.0188 | 4.18E-06 | 14296 |
|  | rs139747874 | 20 | 37234290 | T | C | -0.2523 | 0.0567 | 0.9867 | 8.60E-06 | 13422 |
| NRTN | rs4360502 | 1 | 214886642 | A | G | -0.0652 | 0.0141 | 0.3445 | 3.76E-06 | 11342 |
|  | rs62120726 | 2 | 15734798 | A | G | 0.0786 | 0.0172 | 0.8077 | 4.88E-06 | 11343 |
|  | rs908372 | 2 | 172434373 | C | G | -0.0622 | 0.0136 | 0.3324 | 4.80E-06 | 11773 |
|  | rs48221 | 3 | 8648067 | T | C | -0.0596 | 0.0134 | 0.3995 | 8.68E-06 | 11784 |
|  | rs76777800 | 3 | 73067086 | T | C | 0.3387 | 0.0738 | 0.0176 | 4.44E-06 | 6941 |
|  | rs143131035 | 4 | 43313220 | T | C | -0.4361 | 0.0867 | 0.0118 | 4.91E-07 | 7585 |
|  | rs7712604 | 5 | 21508959 | C | G | -0.0879 | 0.0176 | 0.6738 | 5.90E-07 | 8587 |
|  | rs75066660 | 5 | 92157390 | A | G | 0.2148 | 0.0476 | 0.0197 | 6.40E-06 | 11789 |
|  | rs29630 | 5 | 171026588 | T | C | -0.074 | 0.0159 | 0.2493 | 3.25E-06 | 11343 |
|  | rs61589891 | 6 | 73859555 | A | G | -0.1563 | 0.0348 | 0.0545 | 7.08E-06 | 9021 |
|  | rs76807861 | 6 | 153460965 | A | G | -0.2154 | 0.0486 | 0.023 | 9.33E-06 | 10901 |
|  | rs4723879 | 7 | 4596494 | A | G | -0.0722 | 0.0163 | 0.2241 | 9.45E-06 | 11343 |
|  | rs11766761 | 7 | 41670710 | T | C | 0.0729 | 0.0165 | 0.8184 | 9.95E-06 | 11783 |
|  | rs192427410 | 8 | 43185781 | T | G | 0.3743 | 0.0786 | 0.0252 | 1.92E-06 | 8783 |
|  | rs148658700 | 9 | 138510992 | A | T | -0.3205 | 0.0713 | 0.0145 | 6.95E-06 | 11343 |
|  | rs77216559 | 11 | 4475910 | T | C | 0.1667 | 0.034 | 0.958 | 9.44E-07 | 11791 |
|  | rs182274799 | 11 | 10358788 | A | G | 0.1565 | 0.031 | 0.0646 | 4.46E-07 | 9461 |
|  | rs4753678 | 11 | 95201232 | A | G | 0.087 | 0.0178 | 0.5434 | 1.02E-06 | 7942 |
|  | rs140212763 | 13 | 19290907 | T | G | -0.1599 | 0.0358 | 0.9347 | 7.95E-06 | 8783 |
|  | rs8033310 | 15 | 24046105 | T | C | 0.1332 | 0.0286 | 0.9133 | 3.20E-06 | 11343 |
|  | rs117216753 | 15 | 28183982 | T | C | 0.2114 | 0.0475 | 0.0291 | 8.57E-06 | 9339 |
|  | rs8030121 | 15 | 51931074 | A | G | 0.0601 | 0.0136 | 0.4519 | 9.91E-06 | 11343 |
|  | rs28572049 | 15 | 95039717 | A | G | 0.0707 | 0.016 | 0.2983 | 9.93E-06 | 9461 |
|  | rs7202385 | 16 | 26080883 | A | T | 0.0746 | 0.0157 | 0.6725 | 2.02E-06 | 9460 |
|  | rs10412462 | 19 | 14691684 | A | C | -0.1731 | 0.0366 | 0.0363 | 2.25E-06 | 11790 |
|  | rs113580784 | 20 | 55469957 | A | G | -0.2288 | 0.0495 | 0.0208 | 3.80E-06 | 11768 |
| NT-3 | rs1017982 | 1 | 238095957 | A | G | 0.0623 | 0.014 | 0.2606 | 8.59E-06 | 14296 |
|  | rs145686993 | 2 | 46306174 | C | G | -0.2464 | 0.0539 | 0.0161 | 4.84E-06 | 14257 |
|  | rs77109322 | 2 | 240277019 | T | C | 0.1398 | 0.0313 | 0.0423 | 7.95E-06 | 14744 |
|  | rs185208766 | 3 | 73021223 | T | C | 0.3291 | 0.0722 | 0.0142 | 5.16E-06 | 9893 |
|  | rs79759094 | 4 | 7533099 | A | G | -0.1163 | 0.0251 | 0.0638 | 3.60E-06 | 14728 |
|  | rs111981019 | 4 | 158274860 | A | C | -0.2188 | 0.0495 | 0.9635 | 9.86E-06 | 12800 |
|  | rs7718539 | 5 | 1730637 | T | C | -0.0539 | 0.0122 | 0.353 | 9.96E-06 | 14740 |
|  | rs255889 | 5 | 111111113 | A | G | -0.0937 | 0.0212 | 0.9203 | 9.88E-06 | 14731 |
|  | rs2034246 | 5 | 114451587 | A | C | 0.0582 | 0.012 | 0.5677 | 1.23E-06 | 14296 |
|  | rs188129302 | 5 | 146781995 | T | C | -0.2607 | 0.0577 | 0.018 | 6.24E-06 | 12412 |
|  | rs11756802 | 6 | 7170084 | A | T | -0.1414 | 0.0319 | 0.0444 | 9.31E-06 | 14296 |
|  | rs3218587 | 6 | 111688927 | A | G | 0.1364 | 0.0294 | 0.0429 | 3.49E-06 | 14744 |
|  | rs112418830 | 6 | 151293888 | T | C | 0.1366 | 0.0263 | 0.0567 | 2.06E-07 | 14296 |
|  | rs2107473 | 7 | 8762956 | A | G | -0.2393 | 0.0507 | 0.0175 | 2.36E-06 | 13394 |
|  | rs17537796 | 7 | 40026172 | A | C | -0.2571 | 0.0553 | 0.9866 | 3.33E-06 | 14254 |
|  | rs73166403 | 7 | 152298228 | T | C | -0.092 | 0.02 | 0.1114 | 4.22E-06 | 14296 |
|  | rs72652073 | 8 | 72031901 | C | G | 0.058 | 0.0125 | 0.3811 | 3.48E-06 | 14296 |
|  | rs77065501 | 8 | 143406020 | T | C | 0.326 | 0.0721 | 0.0135 | 6.14E-06 | 10754 |
|  | rs10121142 | 9 | 32900305 | A | T | -0.0633 | 0.0139 | 0.263 | 5.26E-06 | 12844 |
|  | rs10780690 | 9 | 87478172 | T | G | 0.0557 | 0.012 | 0.5265 | 3.46E-06 | 14296 |
|  | rs871910 | 10 | 7026672 | T | C | -0.0632 | 0.0141 | 0.2256 | 7.39E-06 | 14296 |
|  | rs146532158 | 10 | 115833862 | T | C | -0.1859 | 0.0386 | 0.0249 | 1.46E-06 | 14741 |
|  | rs11225042 | 11 | 101727684 | A | C | -0.0973 | 0.0203 | 0.8737 | 1.64E-06 | 12412 |
|  | rs2846299 | 11 | 123375056 | A | G | 0.0582 | 0.0125 | 0.3022 | 3.22E-06 | 14721 |
|  | rs58399805 | 12 | 5680104 | A | T | 0.1106 | 0.022 | 0.0799 | 4.98E-07 | 14737 |
|  | rs116906009 | 12 | 118360925 | T | C | -0.1839 | 0.041 | 0.9745 | 7.28E-06 | 14727 |
|  | rs1009409 | 13 | 69789253 | T | G | 0.0552 | 0.0121 | 0.3857 | 5.07E-06 | 14295 |
|  | rs28735437 | 15 | 88514855 | C | G | -0.1168 | 0.0164 | 0.1435 | 1.06E-12 | 14737 |
|  | rs376150779 | 18 | 55310060 | C | G | -0.0819 | 0.0172 | 0.7735 | 1.92E-06 | 10894 |
|  | rs11151953 | 18 | 72123860 | C | G | 0.0734 | 0.0124 | 0.5258 | 3.23E-09 | 14296 |
|  | rs7250584 | 19 | 8335689 | T | C | -0.117 | 0.026 | 0.0529 | 6.80E-06 | 14743 |
|  | rs2213143 | 22 | 22401216 | T | C | -0.1139 | 0.0253 | 0.0687 | 6.73E-06 | 14296 |
|  | rs5751454 | 22 | 43649715 | T | C | 0.1823 | 0.0398 | 0.0287 | 4.64E-06 | 13680 |
| OPG | rs114165349 | 1 | 27021913 | C | G | -0.1948 | 0.034 | 0.0337 | 1.01E-08 | 14727 |
|  | rs56009454 | 1 | 40045085 | T | G | -0.0637 | 0.0139 | 0.3753 | 4.59E-06 | 10894 |
|  | rs17396131 | 1 | 55145897 | A | G | 0.0562 | 0.0125 | 0.3327 | 6.92E-06 | 14285 |
|  | rs74627924 | 2 | 145820726 | A | G | -0.0827 | 0.0183 | 0.8883 | 6.21E-06 | 14710 |
|  | rs114805200 | 2 | 155956833 | C | G | -0.2032 | 0.0455 | 0.9827 | 7.97E-06 | 14285 |
|  | rs75064915 | 2 | 223274212 | A | T | -0.1311 | 0.0295 | 0.0452 | 8.83E-06 | 14730 |
|  | rs12637074 | 3 | 32324460 | T | C | -0.0712 | 0.0158 | 0.8414 | 6.60E-06 | 14708 |
|  | rs17600346 | 3 | 172223982 | T | C | -0.1569 | 0.0299 | 0.9631 | 1.54E-07 | 14723 |
|  | rs28454196 | 4 | 32870458 | C | G | -0.2242 | 0.0486 | 0.9758 | 3.97E-06 | 14285 |
|  | rs1230164 | 4 | 99986023 | C | G | -0.0671 | 0.0129 | 0.7288 | 1.98E-07 | 14285 |
|  | rs10000065 | 4 | 190051252 | C | G | 0.0775 | 0.0171 | 0.1683 | 5.84E-06 | 11538 |
|  | rs7703177 | 5 | 52799456 | T | C | 0.053 | 0.0117 | 0.5883 | 5.90E-06 | 14285 |
|  | rs192337992 | 6 | 130506773 | A | G | -0.1555 | 0.0334 | 0.962 | 3.23E-06 | 11978 |
|  | rs144471361 | 6 | 134519823 | T | C | 0.3067 | 0.0628 | 0.0154 | 1.04E-06 | 12145 |
|  | rs4540346 | 7 | 116067883 | T | C | 0.0518 | 0.0117 | 0.3382 | 9.54E-06 | 14715 |
|  | rs6985207 | 8 | 8604423 | A | C | 0.0522 | 0.0114 | 0.5556 | 4.67E-06 | 14285 |
|  | rs9643126 | 8 | 119443927 | C | G | 0.0837 | 0.0146 | 0.4689 | 9.87E-09 | 10893 |
|  | rs2247769 | 8 | 120081031 | T | C | -0.1705 | 0.0114 | 0.5577 | 1.42E-50 | 14285 |
|  | rs77507094 | 10 | 55029035 | A | G | 0.1965 | 0.0429 | 0.9791 | 4.64E-06 | 14726 |
|  | rs1437633 | 11 | 16272199 | A | C | -0.079 | 0.0147 | 0.2072 | 7.69E-08 | 12851 |
|  | rs376613127 | 12 | 71789031 | A | G | -0.2692 | 0.0568 | 0.0133 | 2.14E-06 | 13154 |
|  | rs74111611 | 12 | 85607051 | T | C | -0.1471 | 0.0298 | 0.9603 | 7.96E-07 | 14732 |
|  | rs138557324 | 12 | 119984130 | T | C | -0.2253 | 0.0473 | 0.0196 | 1.91E-06 | 14285 |
|  | rs61968587 | 13 | 109537182 | A | G | -0.2671 | 0.0567 | 0.9843 | 2.47E-06 | 13798 |
|  | rs10142466 | 14 | 69271784 | A | G | 0.057 | 0.0109 | 0.49 | 1.70E-07 | 14721 |
|  | rs112635299 | 14 | 94838142 | T | G | 0.2618 | 0.0433 | 0.0205 | 1.48E-09 | 14733 |
|  | rs138408096 | 15 | 84250648 | A | G | -0.2007 | 0.0423 | 0.0206 | 2.09E-06 | 14721 |
|  | rs704 | 17 | 26694861 | A | G | -0.1404 | 0.0111 | 0.4726 | 1.14E-36 | 14733 |
|  | rs271665 | 17 | 47938521 | T | C | -0.0672 | 0.0129 | 0.748 | 1.90E-07 | 14710 |
|  | rs6506157 | 18 | 4040656 | A | G | 0.0642 | 0.0144 | 0.7937 | 8.26E-06 | 14285 |
|  | rs9946923 | 18 | 76523646 | T | C | 0.172 | 0.0376 | 0.9707 | 4.77E-06 | 13230 |
|  | rs928835 | 21 | 21116259 | A | T | 0.1047 | 0.0237 | 0.934 | 9.97E-06 | 14717 |
| OSM | rs141451728 | 2 | 8181069 | T | C | 0.0908 | 0.02 | 0.1152 | 5.63E-06 | 12824 |
|  | rs200034863 | 2 | 149143446 | A | G | 0.3067 | 0.0678 | 0.9871 | 6.08E-06 | 11273 |
|  | rs114931601 | 3 | 26561369 | A | G | 0.1125 | 0.0254 | 0.9343 | 9.46E-06 | 14288 |
|  | rs7432752 | 3 | 155531845 | A | G | 0.0882 | 0.0167 | 0.5176 | 1.28E-07 | 10894 |
|  | rs7714366 | 5 | 169867337 | A | G | 0.0619 | 0.0137 | 0.7535 | 6.24E-06 | 14734 |
|  | rs9264931 | 6 | 31274196 | T | C | -0.1048 | 0.0191 | 0.8812 | 4.09E-08 | 14736 |
|  | rs6932879 | 6 | 162256951 | T | C | -0.162 | 0.0356 | 0.0307 | 5.35E-06 | 14728 |
|  | rs559651 | 8 | 41555366 | T | C | -0.0594 | 0.0125 | 0.6496 | 2.01E-06 | 14715 |
|  | rs117867445 | 9 | 137876508 | C | G | 0.2926 | 0.0621 | 0.0172 | 2.46E-06 | 10471 |
|  | rs76275710 | 10 | 30032133 | A | G | 0.2665 | 0.0525 | 0.0176 | 3.85E-07 | 14735 |
|  | rs61897429 | 11 | 47424864 | A | G | -0.0847 | 0.017 | 0.139 | 6.28E-07 | 14735 |
|  | rs2511241 | 11 | 72945341 | T | C | 0.177 | 0.0236 | 0.9163 | 6.38E-14 | 13668 |
|  | rs7102957 | 11 | 102334152 | T | C | -0.0803 | 0.0173 | 0.8631 | 3.46E-06 | 14288 |
|  | rs653178 | 12 | 112007756 | T | C | -0.0728 | 0.0132 | 0.5224 | 3.48E-08 | 11785 |
|  | rs111827930 | 13 | 51453936 | A | G | -0.061 | 0.0138 | 0.2479 | 9.86E-06 | 14288 |
|  | rs111865607 | 14 | 88422929 | T | C | -0.1077 | 0.0178 | 0.2079 | 1.44E-09 | 10894 |
|  | rs4779338 | 15 | 22508018 | A | G | 0.1274 | 0.0276 | 0.211 | 3.91E-06 | 8491 |
|  | rs76036744 | 15 | 82216867 | T | C | 0.1893 | 0.0419 | 0.9776 | 6.25E-06 | 14248 |
|  | rs4598859 | 15 | 94565995 | A | G | -0.0534 | 0.0118 | 0.494 | 6.03E-06 | 14727 |
|  | rs3859189 | 17 | 38137033 | A | G | 0.1101 | 0.0117 | 0.5367 | 4.95E-21 | 14729 |
|  | rs116928468 | 17 | 75178814 | A | G | -0.2566 | 0.0536 | 0.019 | 1.69E-06 | 11770 |
|  | rs4818145 | 21 | 42005707 | A | G | 0.0619 | 0.0138 | 0.7439 | 7.27E-06 | 14288 |
|  | rs80303768 | 21 | 42594678 | A | G | -0.152 | 0.0327 | 0.0371 | 3.35E-06 | 14722 |
|  | rs4823082 | 22 | 30600105 | C | G | -0.159 | 0.0346 | 0.0363 | 4.32E-06 | 14724 |
|  | rs2285179 | 22 | 38211954 | A | G | -0.0663 | 0.0122 | 0.5586 | 5.50E-08 | 14288 |
|  | rs28444486 | 22 | 49231540 | A | G | 0.0636 | 0.0136 | 0.7378 | 2.92E-06 | 14735 |
| PD-L1 | rs146328951 | 1 | 27646512 | A | G | -0.4926 | 0.0993 | 0.0079 | 7.02E-07 | 9354 |
|  | rs113302946 | 1 | 77828069 | A | G | -0.2866 | 0.0637 | 0.014 | 6.82E-06 | 13801 |
|  | rs184265227 | 1 | 238609559 | A | G | 0.0961 | 0.0192 | 0.868 | 5.58E-07 | 14288 |
|  | rs72823987 | 2 | 67288067 | A | G | -0.1005 | 0.0223 | 0.9271 | 6.58E-06 | 14736 |
|  | rs6726087 | 2 | 85109126 | A | G | 0.1464 | 0.0326 | 0.0408 | 7.10E-06 | 11978 |
|  | rs2019137 | 2 | 113973632 | A | G | -0.0507 | 0.0114 | 0.4683 | 8.69E-06 | 14735 |
|  | rs72563738 | 2 | 202897188 | T | G | 0.2507 | 0.056 | 0.9828 | 7.58E-06 | 12805 |
|  | rs4974444 | 3 | 127280969 | A | G | -0.0816 | 0.0176 | 0.8717 | 3.55E-06 | 14288 |
|  | rs181758547 | 4 | 68657948 | T | C | -0.3071 | 0.069 | 0.0116 | 8.56E-06 | 11917 |
|  | rs11750804 | 5 | 41603985 | T | C | -0.0742 | 0.0164 | 0.8494 | 6.06E-06 | 14723 |
|  | rs62373966 | 5 | 90058982 | T | G | 0.1407 | 0.0273 | 0.9234 | 2.55E-07 | 11777 |
|  | rs10043030 | 5 | 159936536 | T | C | -0.1179 | 0.0248 | 0.0613 | 1.99E-06 | 14091 |
|  | rs6917605 | 6 | 19842497 | T | C | 0.0554 | 0.0116 | 0.4457 | 1.79E-06 | 14733 |
|  | rs75510105 | 6 | 134673561 | A | G | 0.1933 | 0.0385 | 0.0292 | 5.15E-07 | 14727 |
|  | rs73072534 | 7 | 28144510 | T | C | -0.1306 | 0.0294 | 0.0469 | 8.91E-06 | 14288 |
|  | rs151255930 | 7 | 105979524 | T | C | -0.2518 | 0.0561 | 0.0137 | 7.18E-06 | 14243 |
|  | rs12539643 | 7 | 151019021 | C | G | 0.1913 | 0.0407 | 0.966 | 2.60E-06 | 11337 |
|  | rs113674769 | 8 | 68297799 | T | G | -0.1142 | 0.025 | 0.074 | 4.92E-06 | 12404 |
|  | rs822335 | 9 | 5448218 | T | C | -0.1295 | 0.012 | 0.3471 | 3.77E-27 | 14736 |
|  | rs60830384 | 9 | 5485387 | A | T | 0.1138 | 0.0213 | 0.916 | 9.16E-08 | 14718 |
|  | rs7026097 | 9 | 34007646 | A | T | 0.0832 | 0.016 | 0.1779 | 1.99E-07 | 12834 |
|  | rs7125631 | 11 | 1948186 | T | C | -0.0609 | 0.0128 | 0.6543 | 1.96E-06 | 14288 |
|  | rs7936674 | 11 | 33845977 | A | G | -0.0546 | 0.0123 | 0.6468 | 9.04E-06 | 14288 |
|  | rs7945680 | 11 | 127683667 | T | G | 0.061 | 0.0114 | 0.5018 | 8.75E-08 | 14728 |
|  | rs7304709 | 12 | 48670152 | T | C | 0.0537 | 0.0117 | 0.5862 | 4.44E-06 | 14727 |
|  | rs7137828 | 12 | 111932800 | T | C | -0.0622 | 0.0133 | 0.5235 | 2.92E-06 | 11337 |
|  | rs17256127 | 14 | 23711425 | T | G | 0.1205 | 0.0264 | 0.7372 | 5.01E-06 | 10407 |
|  | rs1280389 | 15 | 57727725 | A | T | 0.0574 | 0.0126 | 0.3335 | 5.22E-06 | 14288 |
|  | rs2283438 | 15 | 89721461 | T | C | -0.0577 | 0.012 | 0.4351 | 1.52E-06 | 14288 |
|  | rs79045618 | 19 | 5799008 | A | G | 0.1619 | 0.0324 | 0.0521 | 5.83E-07 | 10714 |
| SCF | rs2843154 | 1 | 2244905 | T | C | -0.0576 | 0.0128 | 0.5999 | 6.80E-06 | 13224 |
|  | rs78397068 | 1 | 88911632 | T | C | 0.1202 | 0.0233 | 0.9155 | 2.49E-07 | 14288 |
|  | rs71644719 | 1 | 223111265 | A | G | -0.1584 | 0.0345 | 0.0354 | 4.40E-06 | 14727 |
|  | rs4846838 | 1 | 230281618 | A | G | -0.0721 | 0.0158 | 0.8081 | 5.04E-06 | 12792 |
|  | rs557933 | 1 | 234853268 | A | C | -0.0553 | 0.0118 | 0.4647 | 2.78E-06 | 14287 |
|  | rs11126305 | 2 | 71106068 | A | G | -0.0567 | 0.0116 | 0.4624 | 1.02E-06 | 14718 |
|  | rs7664024 | 4 | 18944656 | T | C | -0.0718 | 0.0153 | 0.185 | 2.69E-06 | 14288 |
|  | rs12501773 | 4 | 88453631 | A | C | 0.0528 | 0.0116 | 0.5618 | 5.32E-06 | 14733 |
|  | rs34813411 | 4 | 183543315 | T | C | -0.1619 | 0.0339 | 0.0373 | 1.79E-06 | 14717 |
|  | rs4389685 | 5 | 124050437 | A | G | 0.053 | 0.0118 | 0.5999 | 7.07E-06 | 14735 |
|  | rs62398898 | 5 | 161746014 | T | C | -0.1161 | 0.0259 | 0.9437 | 7.37E-06 | 14717 |
|  | rs9264796 | 6 | 31270311 | A | G | 0.1407 | 0.0303 | 0.0716 | 3.42E-06 | 12404 |
|  | rs75059384 | 7 | 90786565 | A | G | -0.1782 | 0.0402 | 0.0264 | 9.30E-06 | 14736 |
|  | rs705379 | 7 | 94953895 | A | G | 0.0789 | 0.012 | 0.4598 | 4.87E-11 | 14288 |
|  | rs2133027 | 8 | 47325114 | A | G | 0.4722 | 0.1032 | 0.007 | 4.75E-06 | 8290 |
|  | rs2980888 | 8 | 126507308 | T | C | -0.0683 | 0.0126 | 0.2937 | 5.94E-08 | 14735 |
|  | rs581080 | 9 | 15305378 | C | G | 0.0668 | 0.015 | 0.8123 | 8.45E-06 | 14734 |
|  | rs785900 | 9 | 79147122 | T | C | 0.0575 | 0.0118 | 0.4446 | 1.10E-06 | 14288 |
|  | rs2740488 | 9 | 107661742 | A | C | 0.1156 | 0.0133 | 0.7485 | 3.57E-18 | 14732 |
|  | rs138854302 | 9 | 128807910 | T | C | -0.1391 | 0.0205 | 0.0915 | 1.16E-11 | 14736 |
|  | rs12378537 | 9 | 136065526 | T | C | 0.105 | 0.0219 | 0.1215 | 1.63E-06 | 11337 |
|  | rs11196977 | 10 | 116663513 | A | C | 0.1658 | 0.0366 | 0.0543 | 5.90E-06 | 10282 |
|  | rs11199541 | 10 | 122531747 | T | C | 0.1548 | 0.0339 | 0.0334 | 4.96E-06 | 14736 |
|  | rs72847212 | 11 | 834389 | T | C | -0.0721 | 0.015 | 0.7926 | 1.53E-06 | 13659 |
|  | rs10832027 | 11 | 13357183 | A | G | -0.0568 | 0.012 | 0.6474 | 2.21E-06 | 14734 |
|  | rs174535 | 11 | 61551356 | T | C | -0.0735 | 0.0121 | 0.6459 | 1.24E-09 | 14736 |
|  | rs10894669 | 11 | 133206940 | A | C | -0.0606 | 0.0129 | 0.3129 | 2.63E-06 | 14735 |
|  | rs74820408 | 12 | 40180662 | A | G | -0.0728 | 0.0163 | 0.8399 | 7.96E-06 | 14287 |
|  | rs11608458 | 12 | 89369806 | T | C | -0.0707 | 0.0122 | 0.6366 | 6.83E-09 | 14288 |
|  | rs71431776 | 13 | 26799167 | A | C | -0.0938 | 0.0192 | 0.1062 | 1.03E-06 | 14714 |
|  | rs140884539 | 14 | 55341903 | T | C | 0.0689 | 0.0154 | 0.2111 | 7.68E-06 | 14735 |
|  | rs1950813 | 14 | 92586869 | T | C | -0.0597 | 0.0131 | 0.645 | 5.18E-06 | 12202 |
|  | rs45617141 | 15 | 43623397 | T | G | 0.2803 | 0.0512 | 0.9812 | 4.38E-08 | 12935 |
|  | rs2043084 | 15 | 58682496 | C | G | -0.0642 | 0.0141 | 0.2186 | 5.28E-06 | 14731 |
|  | rs2161465 | 16 | 25205611 | A | T | -0.0715 | 0.0152 | 0.2479 | 2.55E-06 | 11966 |
|  | rs12149545 | 16 | 56993161 | A | G | 0.1146 | 0.0123 | 0.3094 | 1.20E-20 | 14736 |
|  | rs16942887 | 16 | 67928042 | A | G | 0.1658 | 0.0171 | 0.1303 | 3.14E-22 | 14736 |
|  | rs151246947 | 17 | 16356498 | A | G | 0.2116 | 0.0453 | 0.0274 | 3.00E-06 | 13224 |
|  | rs11877350 | 18 | 43867311 | A | G | 0.0538 | 0.0117 | 0.4265 | 4.26E-06 | 14728 |
|  | rs8108748 | 19 | 39286122 | A | G | 0.066 | 0.0148 | 0.1997 | 8.22E-06 | 14288 |
|  | rs798893 | 19 | 54793830 | C | G | 0.095 | 0.0141 | 0.2138 | 1.61E-11 | 14725 |
|  | rs200582457 | 20 | 23572941 | T | C | -0.068 | 0.0153 | 0.3285 | 8.81E-06 | 10894 |
|  | rs146276488 | 20 | 44109198 | A | G | 0.2422 | 0.0516 | 0.0144 | 2.68E-06 | 14728 |
|  | rs6073958 | 20 | 44551855 | T | C | -0.2858 | 0.0144 | 0.8039 | 1.16E-87 | 14730 |
|  | rs2024570 | 20 | 44776543 | T | C | -0.1985 | 0.0341 | 0.0394 | 5.85E-09 | 14288 |
| SIRT2 | rs7418913 | 1 | 203643268 | A | G | -0.0615 | 0.0139 | 0.2713 | 9.67E-06 | 14288 |
|  | rs138897138 | 2 | 122731427 | A | G | 0.104 | 0.0218 | 0.9138 | 1.84E-06 | 14288 |
|  | rs72847333 | 2 | 125508995 | T | C | 0.093 | 0.0204 | 0.8965 | 5.14E-06 | 14288 |
|  | rs116198125 | 3 | 14613224 | T | C | -0.1521 | 0.0329 | 0.9617 | 3.78E-06 | 14727 |
|  | rs60459634 | 4 | 160629137 | A | G | -0.1063 | 0.0229 | 0.0886 | 3.45E-06 | 14288 |
|  | rs115455827 | 5 | 78787853 | A | G | 0.1277 | 0.0277 | 0.9506 | 4.02E-06 | 14735 |
|  | rs7718478 | 5 | 177560339 | A | T | 0.0639 | 0.0138 | 0.7496 | 3.65E-06 | 14288 |
|  | rs72883370 | 6 | 63413043 | A | G | -0.3428 | 0.0737 | 0.989 | 3.30E-06 | 12737 |
|  | rs1034059 | 6 | 158193698 | A | G | 0.076 | 0.0145 | 0.6981 | 1.59E-07 | 14288 |
|  | rs12531843 | 7 | 14143486 | C | G | -0.1286 | 0.0243 | 0.9177 | 1.21E-07 | 12404 |
|  | rs79663946 | 7 | 21752339 | A | G | -0.0901 | 0.0201 | 0.1174 | 7.37E-06 | 14288 |
|  | rs147383134 | 8 | 89590527 | T | C | 0.2573 | 0.0551 | 0.9858 | 3.02E-06 | 13801 |
|  | rs77197291 | 8 | 111320863 | A | T | -0.0947 | 0.0211 | 0.0878 | 7.18E-06 | 14735 |
|  | rs10998051 | 10 | 70041768 | A | T | 0.0642 | 0.0142 | 0.5227 | 6.15E-06 | 10894 |
|  | rs11115639 | 12 | 83600525 | A | C | -0.0841 | 0.0184 | 0.8864 | 4.86E-06 | 14730 |
|  | rs77381020 | 13 | 37413465 | T | C | 0.0862 | 0.0184 | 0.8546 | 2.80E-06 | 14288 |
|  | rs8112891 | 19 | 7434636 | T | C | 0.1179 | 0.0262 | 0.9292 | 6.80E-06 | 14288 |
|  | rs144373891 | 19 | 39379770 | T | C | -0.5739 | 0.0515 | 0.0161 | 7.69E-29 | 14736 |
|  | rs1966647 | 19 | 56709105 | T | C | -0.0574 | 0.0126 | 0.5696 | 5.22E-06 | 14288 |
|  | rs3788463 | 22 | 32978251 | T | G | -0.06 | 0.0133 | 0.2743 | 6.44E-06 | 14288 |
|  | rs12152184 | 22 | 45002809 | A | G | 0.0965 | 0.0208 | 0.885 | 3.49E-06 | 12402 |
| SLAMF1 | rs3856138 | 1 | 38044814 | T | C | 0.0529 | 0.0118 | 0.5429 | 7.36E-06 | 14286 |
|  | rs12058421 | 1 | 160581152 | A | C | -0.1256 | 0.0159 | 0.1616 | 2.80E-15 | 14731 |
|  | rs60094514 | 1 | 160636559 | T | C | 0.2209 | 0.0162 | 0.8506 | 2.45E-42 | 14732 |
|  | rs13014154 | 2 | 62575443 | A | G | -0.0612 | 0.0123 | 0.3695 | 6.50E-07 | 14285 |
|  | rs192245879 | 2 | 216910771 | A | G | 0.3328 | 0.0655 | 0.989 | 3.76E-07 | 13799 |
|  | rs1972434 | 3 | 98605645 | A | C | 0.065 | 0.0132 | 0.45 | 8.47E-07 | 11333 |
|  | rs10212190 | 3 | 186731157 | A | T | 0.0727 | 0.0121 | 0.6151 | 1.88E-09 | 14285 |
|  | rs3806818 | 4 | 6642331 | A | G | 0.0707 | 0.0153 | 0.1909 | 3.82E-06 | 13853 |
|  | rs13147502 | 4 | 106143797 | A | G | -0.0609 | 0.0127 | 0.5964 | 1.62E-06 | 12843 |
|  | rs191575088 | 5 | 63606345 | A | G | 0.212 | 0.0474 | 0.9791 | 7.73E-06 | 12404 |
|  | rs1057964 | 5 | 95235627 | T | C | -0.1122 | 0.0142 | 0.7184 | 2.76E-15 | 11979 |
|  | rs11745675 | 5 | 179240655 | C | G | 0.0553 | 0.0122 | 0.4344 | 5.82E-06 | 14286 |
|  | rs2308527 | 6 | 31239821 | T | G | 0.0568 | 0.0127 | 0.601 | 7.73E-06 | 12852 |
|  | rs114466578 | 6 | 32635501 | A | G | 0.1017 | 0.0168 | 0.796 | 1.42E-09 | 12733 |
|  | rs7745783 | 6 | 42462406 | T | C | -0.0589 | 0.0119 | 0.4969 | 7.44E-07 | 14286 |
|  | rs115154227 | 6 | 135333467 | T | C | -0.1703 | 0.0381 | 0.0275 | 7.83E-06 | 14734 |
|  | rs2623960 | 6 | 152893441 | T | C | 0.0688 | 0.0143 | 0.2204 | 1.50E-06 | 14286 |
|  | rs80174967 | 7 | 10925674 | A | C | -0.0894 | 0.02 | 0.0995 | 7.82E-06 | 14722 |
|  | rs10499604 | 7 | 38626562 | T | C | 0.058 | 0.0125 | 0.3266 | 3.48E-06 | 14730 |
|  | rs79348253 | 7 | 134817203 | A | G | -0.1585 | 0.0295 | 0.0447 | 7.75E-08 | 14719 |
|  | rs17720330 | 9 | 78767497 | C | G | -0.1982 | 0.043 | 0.0213 | 4.04E-06 | 14727 |
|  | rs61833942 | 10 | 13610507 | T | C | 0.2097 | 0.0474 | 0.9803 | 9.69E-06 | 14286 |
|  | rs2790462 | 10 | 28873202 | T | C | -0.0747 | 0.0126 | 0.2977 | 3.06E-09 | 14733 |
|  | rs138657007 | 10 | 66091335 | A | G | -0.2863 | 0.0577 | 0.0181 | 6.98E-07 | 12790 |
|  | rs7918908 | 10 | 128410584 | A | G | -0.1175 | 0.0264 | 0.0754 | 8.56E-06 | 11780 |
|  | rs72902770 | 11 | 43535182 | T | C | -0.0821 | 0.0181 | 0.1184 | 5.74E-06 | 14723 |
|  | rs12297442 | 12 | 6062889 | A | G | 0.061 | 0.0135 | 0.7296 | 6.23E-06 | 14733 |
|  | rs3184504 | 12 | 111884608 | T | C | 0.097 | 0.013 | 0.4758 | 8.55E-14 | 11783 |
|  | rs2393716 | 12 | 121341933 | C | G | 0.0583 | 0.0124 | 0.5241 | 2.58E-06 | 12848 |
|  | rs111865527 | 13 | 26736047 | T | C | 0.2712 | 0.0569 | 0.9863 | 1.88E-06 | 14244 |
|  | rs73231041 | 13 | 78282092 | C | G | -0.2791 | 0.0598 | 0.0148 | 3.05E-06 | 12735 |
|  | rs112742549 | 13 | 107639422 | T | C | 0.2421 | 0.0506 | 0.9791 | 1.71E-06 | 13499 |
|  | rs8011635 | 14 | 76464831 | T | C | -0.0543 | 0.0122 | 0.373 | 8.55E-06 | 14286 |
|  | rs147616029 | 14 | 87822926 | T | C | -0.0644 | 0.0128 | 0.3774 | 4.87E-07 | 12850 |
|  | rs1609310 | 15 | 55294543 | T | G | -0.0557 | 0.012 | 0.5136 | 3.46E-06 | 14286 |
|  | rs56244095 | 17 | 7063899 | A | C | 0.1013 | 0.0156 | 0.2121 | 8.38E-11 | 14286 |
|  | rs2725405 | 17 | 79220224 | C | G | 0.0894 | 0.0141 | 0.4462 | 2.29E-10 | 10271 |
|  | rs149092077 | 18 | 59233474 | A | G | -0.3592 | 0.073 | 0.9888 | 8.63E-07 | 11137 |
|  | rs35271532 | 18 | 72388527 | A | G | -0.1486 | 0.0324 | 0.0449 | 4.51E-06 | 14286 |
|  | rs3745163 | 19 | 17337555 | A | C | 0.0674 | 0.0145 | 0.2299 | 3.35E-06 | 13669 |
|  | rs142352460 | 21 | 14753496 | T | C | 0.2946 | 0.0654 | 0.0246 | 6.65E-06 | 10216 |
|  | rs73151926 | 22 | 22451222 | T | C | 0.1063 | 0.0229 | 0.0805 | 3.45E-06 | 14286 |
| ST1A1 | rs1698742 | 1 | 86468724 | T | C | -0.0984 | 0.0206 | 0.8775 | 1.78E-06 | 11790 |
|  | rs2712255 | 1 | 119273856 | A | G | 0.0702 | 0.0147 | 0.466 | 1.79E-06 | 9849 |
|  | rs76712070 | 1 | 243050615 | A | G | -0.1367 | 0.0302 | 0.9345 | 6.00E-06 | 11345 |
|  | rs115453780 | 2 | 81661503 | A | T | 0.1995 | 0.0448 | 0.0287 | 8.46E-06 | 11303 |
|  | rs9882591 | 3 | 59801480 | C | G | 0.1061 | 0.0234 | 0.1013 | 5.78E-06 | 11779 |
|  | rs710446 | 3 | 186459927 | T | C | -0.0712 | 0.0135 | 0.599 | 1.33E-07 | 11793 |
|  | rs265013 | 4 | 96411517 | A | G | -0.0784 | 0.0177 | 0.821 | 9.45E-06 | 11301 |
|  | rs149869672 | 4 | 127777021 | T | C | -0.3184 | 0.0684 | 0.9871 | 3.24E-06 | 10858 |
|  | rs371387526 | 4 | 142303334 | A | C | 0.1048 | 0.0214 | 0.8462 | 9.72E-07 | 8817 |
|  | rs66530140 | 4 | 187161211 | T | C | -0.1678 | 0.0136 | 0.4926 | 5.64E-35 | 10913 |
|  | rs12163980 | 5 | 18788822 | T | C | -0.0851 | 0.0191 | 0.1679 | 8.37E-06 | 11345 |
|  | rs114343645 | 5 | 42655238 | A | G | 0.2386 | 0.0539 | 0.9813 | 9.57E-06 | 11345 |
|  | rs2731674 | 5 | 176839890 | T | G | -0.083 | 0.0152 | 0.2653 | 4.75E-08 | 11790 |
|  | rs55793562 | 6 | 52231674 | A | G | 0.0924 | 0.0199 | 0.8711 | 3.43E-06 | 11781 |
|  | rs2691531 | 7 | 77960330 | T | C | -0.0645 | 0.0135 | 0.5986 | 1.77E-06 | 11793 |
|  | rs7812322 | 8 | 11410797 | T | C | -0.3483 | 0.0756 | 0.9898 | 4.08E-06 | 10858 |
|  | rs2213907 | 8 | 17377551 | A | G | -0.1504 | 0.0318 | 0.95 | 2.25E-06 | 11790 |
|  | rs55939507 | 8 | 126413022 | A | G | 0.0768 | 0.0166 | 0.4768 | 3.72E-06 | 9460 |
|  | rs147448406 | 9 | 116822808 | T | C | -0.2619 | 0.059 | 0.0239 | 9.04E-06 | 11793 |
|  | rs77919742 | 11 | 19560616 | T | C | -0.1433 | 0.0287 | 0.9287 | 5.94E-07 | 11345 |
|  | rs1943466 | 11 | 123264642 | A | C | -0.067 | 0.0148 | 0.2759 | 5.98E-06 | 11789 |
|  | rs76833948 | 11 | 128596369 | A | T | 0.134 | 0.0285 | 0.0644 | 2.58E-06 | 11345 |
|  | rs72656644 | 12 | 12719921 | C | G | 0.1077 | 0.0224 | 0.8871 | 1.52E-06 | 11345 |
|  | rs10842189 | 12 | 23759406 | T | C | -0.08 | 0.0173 | 0.8156 | 3.76E-06 | 11784 |
|  | rs9804965 | 12 | 74902158 | A | C | -0.1927 | 0.0361 | 0.0662 | 9.40E-08 | 9265 |
|  | rs703595 | 12 | 103074539 | T | C | -0.0715 | 0.0147 | 0.2856 | 1.15E-06 | 11789 |
|  | rs232928 | 12 | 113112650 | T | C | 0.0622 | 0.0136 | 0.4128 | 4.80E-06 | 11345 |
|  | rs112100718 | 14 | 58195463 | T | G | -0.1892 | 0.0419 | 0.0306 | 6.32E-06 | 11789 |
|  | rs11630815 | 15 | 44335192 | A | G | 0.1545 | 0.0346 | 0.0655 | 7.99E-06 | 9461 |
|  | rs11074902 | 16 | 28285775 | A | G | 0.0695 | 0.0139 | 0.561 | 5.73E-07 | 11345 |
|  | rs149278 | 16 | 28561581 | T | C | -0.1882 | 0.0135 | 0.5314 | 3.58E-44 | 11344 |
|  | rs117222566 | 17 | 54851764 | A | G | 0.271 | 0.0581 | 0.9826 | 3.10E-06 | 10403 |
|  | rs72643498 | 21 | 28777897 | A | C | -0.18 | 0.0376 | 0.9587 | 1.69E-06 | 11788 |
| STAMPB | rs7418913 | 1 | 203643268 | A | G | -0.0628 | 0.0139 | 0.2712 | 6.24E-06 | 14288 |
|  | rs851168 | 1 | 223353343 | A | G | 0.0616 | 0.0136 | 0.3247 | 5.91E-06 | 12792 |
|  | rs111872246 | 2 | 74061232 | A | G | 0.2195 | 0.0425 | 0.0229 | 2.41E-07 | 14248 |
|  | rs138897138 | 2 | 122731427 | A | G | 0.1085 | 0.0218 | 0.9138 | 6.46E-07 | 14288 |
|  | rs16847403 | 2 | 142616069 | T | C | 0.0584 | 0.0131 | 0.7326 | 8.27E-06 | 14713 |
|  | rs116198125 | 3 | 14613224 | T | C | -0.1484 | 0.0325 | 0.9619 | 4.97E-06 | 14727 |
|  | rs547553 | 3 | 176213375 | T | C | 0.2004 | 0.0452 | 0.9798 | 9.27E-06 | 14728 |
|  | rs73788059 | 5 | 132669108 | T | C | -0.092 | 0.0204 | 0.0933 | 6.49E-06 | 14716 |
|  | rs116132562 | 6 | 20187207 | A | G | -0.1689 | 0.0372 | 0.0333 | 5.62E-06 | 14247 |
|  | rs1034059 | 6 | 158193698 | A | G | 0.0695 | 0.0145 | 0.698 | 1.64E-06 | 14288 |
|  | rs12531843 | 7 | 14143486 | C | G | -0.1251 | 0.0243 | 0.9177 | 2.63E-07 | 12404 |
|  | rs200824954 | 7 | 29807677 | T | G | 0.1578 | 0.0339 | 0.0643 | 3.24E-06 | 10894 |
|  | rs75610895 | 10 | 6800809 | T | C | -0.1908 | 0.0394 | 0.0247 | 1.28E-06 | 14730 |
|  | rs61962729 | 13 | 77533163 | T | G | -0.0978 | 0.0217 | 0.9054 | 6.58E-06 | 14288 |
|  | rs2584528 | 13 | 101837214 | T | G | 0.056 | 0.0123 | 0.6302 | 5.29E-06 | 14288 |
|  | rs61980337 | 14 | 33446275 | T | C | 0.0793 | 0.0179 | 0.7163 | 9.42E-06 | 11760 |
|  | rs111581172 | 14 | 104363671 | A | G | -0.1722 | 0.0352 | 0.0323 | 9.98E-07 | 14736 |
|  | rs6492915 | 15 | 40037760 | T | G | -0.1086 | 0.024 | 0.9115 | 6.04E-06 | 11785 |
|  | rs2280441 | 15 | 81240669 | A | G | -0.1571 | 0.0309 | 0.0404 | 3.69E-07 | 14735 |
|  | rs114302245 | 16 | 84586319 | T | C | 0.0876 | 0.0169 | 0.3579 | 2.18E-07 | 10894 |
|  | rs1714837 | 17 | 21231711 | T | C | 0.2538 | 0.056 | 0.0345 | 5.84E-06 | 9343 |
|  | rs73327656 | 17 | 60294757 | A | G | 0.235 | 0.0512 | 0.0269 | 4.44E-06 | 13801 |
|  | rs140656901 | 19 | 6195636 | A | G | 0.1288 | 0.0272 | 0.0724 | 2.19E-06 | 13224 |
|  | rs10426867 | 19 | 56709656 | A | G | 0.2031 | 0.0437 | 0.0246 | 3.36E-06 | 12785 |
|  | rs138739860 | 21 | 18103398 | T | G | 0.1613 | 0.0358 | 0.9645 | 6.62E-06 | 11979 |
|  | rs12152184 | 22 | 45002809 | A | G | 0.0961 | 0.0208 | 0.8851 | 3.83E-06 | 12402 |
| TGF-α | rs79050605 | 1 | 92925962 | T | G | -0.0954 | 0.0202 | 0.8166 | 2.33E-06 | 8287 |
|  | rs78912149 | 1 | 166175871 | C | G | -0.2692 | 0.056 | 0.9835 | 1.53E-06 | 13798 |
|  | rs72912115 | 2 | 70774295 | A | T | 0.137 | 0.0184 | 0.114 | 9.65E-14 | 14728 |
|  | rs34007466 | 4 | 16165036 | A | G | -0.2684 | 0.05 | 0.0153 | 7.96E-08 | 14733 |
|  | rs10473116 | 5 | 38712721 | A | G | -0.0717 | 0.016 | 0.1852 | 7.42E-06 | 13235 |
|  | rs6872427 | 5 | 112532750 | C | G | 0.139 | 0.031 | 0.043 | 7.33E-06 | 14285 |
|  | rs2227284 | 5 | 132012725 | T | G | 0.0672 | 0.0152 | 0.2767 | 9.82E-06 | 11334 |
|  | rs60545802 | 5 | 132204577 | T | G | -0.0701 | 0.0154 | 0.8291 | 5.32E-06 | 14716 |
|  | rs9262670 | 6 | 31034990 | T | C | 0.0717 | 0.0146 | 0.2343 | 9.06E-07 | 12842 |
|  | rs12540570 | 7 | 13645174 | A | C | 0.0557 | 0.0125 | 0.6509 | 8.35E-06 | 14285 |
|  | rs1963491 | 8 | 2654153 | C | G | 0.1081 | 0.023 | 0.905 | 2.60E-06 | 11334 |
|  | rs34630685 | 8 | 13724808 | C | G | 0.1678 | 0.0361 | 0.9681 | 3.35E-06 | 14285 |
|  | rs113903858 | 8 | 72362254 | A | G | 0.3368 | 0.0748 | 0.014 | 6.71E-06 | 11136 |
|  | rs74130123 | 10 | 30042437 | T | C | -0.2601 | 0.0508 | 0.9817 | 3.05E-07 | 14733 |
|  | rs118063320 | 10 | 56979254 | A | G | 0.2401 | 0.0531 | 0.0203 | 6.14E-06 | 13798 |
|  | rs4934441 | 10 | 90836825 | A | G | -0.095 | 0.0198 | 0.1322 | 1.60E-06 | 11780 |
|  | rs2478421 | 10 | 122257278 | T | G | 0.1526 | 0.0342 | 0.9672 | 8.12E-06 | 14285 |
|  | rs12270510 | 11 | 119506018 | T | C | 0.0959 | 0.019 | 0.1107 | 4.48E-07 | 14731 |
|  | rs653178 | 12 | 112007756 | T | C | -0.0708 | 0.0131 | 0.5228 | 6.50E-08 | 11782 |
|  | rs2182884 | 13 | 27399388 | A | T | -0.0692 | 0.0155 | 0.8117 | 8.02E-06 | 14285 |
|  | rs7192501 | 16 | 79874322 | C | G | -0.1432 | 0.0321 | 0.0387 | 8.16E-06 | 14727 |
|  | rs3859189 | 17 | 38137033 | A | G | 0.0655 | 0.0116 | 0.5369 | 1.64E-08 | 14726 |
|  | rs35496173 | 17 | 52896367 | T | G | -0.0561 | 0.0122 | 0.4039 | 4.26E-06 | 14285 |
|  | rs72477244 | 18 | 9837013 | A | G | 0.139 | 0.0309 | 0.0436 | 6.85E-06 | 14285 |
|  | rs78464136 | 19 | 15885492 | A | T | 0.143 | 0.0307 | 0.9552 | 3.19E-06 | 14285 |
|  | rs149146289 | 19 | 58857883 | A | G | 0.2856 | 0.0569 | 0.9866 | 5.19E-07 | 14285 |
|  | rs145310376 | 22 | 39846207 | T | G | -0.1656 | 0.0374 | 0.0346 | 9.52E-06 | 14285 |
| TWEAK | rs13403154 | 2 | 8690963 | A | G | 0.1247 | 0.0277 | 0.0708 | 6.74E-06 | 11337 |
|  | rs56304087 | 2 | 47823379 | T | C | -0.0584 | 0.0131 | 0.4948 | 8.27E-06 | 11778 |
|  | rs12328100 | 2 | 86307128 | A | G | -0.0649 | 0.013 | 0.478 | 5.97E-07 | 11784 |
|  | rs333835 | 2 | 117991812 | A | T | 0.1289 | 0.0254 | 0.9163 | 3.88E-07 | 11337 |
|  | rs11903500 | 2 | 205502517 | A | G | 0.0754 | 0.0166 | 0.2352 | 5.57E-06 | 11337 |
|  | rs1179724 | 2 | 217732382 | T | C | -0.0605 | 0.0136 | 0.5818 | 8.65E-06 | 11337 |
|  | rs7621032 | 3 | 108099780 | T | C | 0.0674 | 0.0135 | 0.4514 | 5.96E-07 | 11337 |
|  | rs4835254 | 4 | 146658520 | T | C | -0.1517 | 0.031 | 0.9506 | 9.90E-07 | 11770 |
|  | rs75625106 | 5 | 3388581 | C | G | -0.5898 | 0.1299 | 0.9941 | 5.61E-06 | 8488 |
|  | rs78812934 | 5 | 6760041 | T | C | -0.1445 | 0.0326 | 0.0459 | 9.31E-06 | 11785 |
|  | rs867063 | 6 | 44325149 | T | C | 0.1175 | 0.0255 | 0.078 | 4.07E-06 | 11337 |
|  | rs138684734 | 6 | 127494091 | A | G | 0.2512 | 0.0533 | 0.9796 | 2.44E-06 | 10850 |
|  | rs117427219 | 7 | 80365289 | A | G | -0.3062 | 0.0629 | 0.0173 | 1.13E-06 | 10850 |
|  | rs7806921 | 7 | 150140762 | T | C | -0.0606 | 0.0131 | 0.5366 | 3.73E-06 | 11778 |
|  | rs148560093 | 8 | 136662225 | A | G | -0.298 | 0.0646 | 0.9824 | 3.97E-06 | 9984 |
|  | rs12354434 | 10 | 11469516 | A | G | -0.1207 | 0.0247 | 0.0885 | 1.03E-06 | 11337 |
|  | rs1377015 | 10 | 106909970 | A | G | -0.0653 | 0.0142 | 0.2861 | 4.25E-06 | 11782 |
|  | rs141295666 | 12 | 50824101 | T | C | 0.3405 | 0.066 | 0.0132 | 2.48E-07 | 11337 |
|  | rs72670134 | 13 | 110635160 | T | C | 0.0863 | 0.0195 | 0.1667 | 9.62E-06 | 9030 |
|  | rs1266163 | 17 | 10503956 | A | T | 0.1372 | 0.0288 | 0.0615 | 1.90E-06 | 11337 |
|  | rs9913313 | 17 | 15740146 | T | C | -0.0836 | 0.0183 | 0.344 | 4.92E-06 | 7957 |
|  | rs73997309 | 17 | 64991154 | A | G | 0.1437 | 0.0315 | 0.053 | 5.07E-06 | 11778 |
|  | rs4804258 | 19 | 8134525 | A | G | -0.0785 | 0.0173 | 0.1915 | 5.69E-06 | 11337 |
|  | rs2279448 | 19 | 16757546 | T | C | 0.4103 | 0.091 | 0.9909 | 6.52E-06 | 8372 |
|  | rs77504024 | 19 | 19064191 | A | G | -0.096 | 0.0214 | 0.8264 | 7.26E-06 | 10273 |
|  | rs12975019 | 19 | 33635097 | C | G | -0.1127 | 0.0226 | 0.8939 | 6.14E-07 | 10278 |
|  | rs6138961 | 20 | 2931738 | C | G | 0.51 | 0.1077 | 0.0088 | 2.19E-06 | 7308 |
|  | rs6089517 | 20 | 59990928 | A | G | -0.2154 | 0.0487 | 0.0299 | 9.73E-06 | 11337 |
|  | rs9614693 | 22 | 45932279 | T | C | 0.0826 | 0.0177 | 0.1679 | 3.06E-06 | 11763 |
| TNF-β | rs77897196 | 1 | 63459761 | T | C | -0.1917 | 0.041 | 0.9711 | 2.93E-06 | 11792 |
|  | rs12743015 | 1 | 110256931 | C | G | -0.1918 | 0.0403 | 0.029 | 1.94E-06 | 11784 |
|  | rs113664581 | 1 | 162552552 | A | G | 0.1631 | 0.0367 | 0.9568 | 8.82E-06 | 11771 |
|  | rs58634322 | 2 | 12848518 | T | C | -0.1011 | 0.0226 | 0.8961 | 7.70E-06 | 11344 |
|  | rs7583622 | 2 | 53931190 | A | G | 0.0748 | 0.0161 | 0.2098 | 3.38E-06 | 11769 |
|  | rs140622233 | 2 | 113468006 | T | G | 0.2762 | 0.0622 | 0.9813 | 8.97E-06 | 9983 |
|  | rs112317147 | 2 | 119783156 | T | C | -0.1441 | 0.0309 | 0.0534 | 3.11E-06 | 11344 |
|  | rs75508961 | 2 | 215601711 | C | G | 0.1731 | 0.0362 | 0.0504 | 1.74E-06 | 8297 |
|  | rs192303297 | 3 | 141634940 | T | C | -0.3872 | 0.0842 | 0.0118 | 4.25E-06 | 7843 |
|  | rs1382846 | 3 | 151804807 | A | G | -0.1213 | 0.0274 | 0.0655 | 9.55E-06 | 11792 |
|  | rs113244922 | 4 | 35497008 | T | C | 0.2324 | 0.0499 | 0.0237 | 3.20E-06 | 10470 |
|  | rs139832280 | 4 | 71720357 | A | T | -0.4149 | 0.0939 | 0.9923 | 9.94E-06 | 9983 |
|  | rs114250919 | 5 | 21093728 | T | C | 0.2469 | 0.049 | 0.9743 | 4.69E-07 | 11305 |
|  | rs247096 | 5 | 128093877 | T | C | -0.0691 | 0.0152 | 0.6986 | 5.47E-06 | 10296 |
|  | rs76913603 | 5 | 167809091 | A | G | -0.1741 | 0.0379 | 0.967 | 4.36E-06 | 11782 |
|  | rs72854560 | 6 | 28404463 | C | G | -0.2968 | 0.0602 | 0.0156 | 8.21E-07 | 11344 |
|  | rs2535313 | 6 | 31052447 | A | G | -0.1768 | 0.0135 | 0.367 | 3.46E-39 | 11792 |
|  | rs2229092 | 6 | 31540757 | A | C | 1.3565 | 0.0256 | 0.9407 | 0.00E+00 | 11792 |
|  | rs185091810 | 6 | 32644431 | A | G | 0.2066 | 0.0339 | 0.079 | 1.10E-09 | 8587 |
|  | rs10282173 | 7 | 38651934 | A | C | -0.1516 | 0.034 | 0.0412 | 8.24E-06 | 11792 |
|  | rs74363807 | 7 | 46566236 | T | C | 0.2496 | 0.0532 | 0.0218 | 2.71E-06 | 11344 |
|  | rs118033381 | 10 | 71320318 | T | C | -0.197 | 0.0441 | 0.0273 | 7.93E-06 | 11785 |
|  | rs10740467 | 10 | 79176594 | A | C | -0.0628 | 0.014 | 0.4585 | 7.27E-06 | 11344 |
|  | rs138471526 | 11 | 72666063 | T | C | -0.3418 | 0.0735 | 0.0137 | 3.31E-06 | 9983 |
|  | rs145415430 | 11 | 84197834 | A | T | 0.2755 | 0.0615 | 0.0148 | 7.48E-06 | 11344 |
|  | rs139511528 | 12 | 6429443 | A | G | -0.2485 | 0.0521 | 0.0294 | 1.85E-06 | 10280 |
|  | rs2364485 | 12 | 6514963 | A | C | 0.1759 | 0.0189 | 0.1645 | 1.32E-20 | 11344 |
|  | rs147423408 | 12 | 52132524 | A | G | 0.1732 | 0.035 | 0.0415 | 7.48E-07 | 11344 |
|  | rs7310615 | 12 | 111865049 | C | G | 0.1356 | 0.0134 | 0.4758 | 4.53E-24 | 11344 |
|  | rs143019966 | 13 | 47656376 | T | C | 0.2122 | 0.0451 | 0.9751 | 2.54E-06 | 10913 |
|  | rs12883833 | 14 | 34880659 | A | G | -0.1252 | 0.0259 | 0.0934 | 1.34E-06 | 9259 |
|  | rs28700215 | 15 | 100064767 | T | C | -0.0626 | 0.0136 | 0.5824 | 4.17E-06 | 11344 |
|  | rs112983129 | 16 | 11519052 | T | C | -0.1854 | 0.0409 | 0.0343 | 5.81E-06 | 11768 |
|  | rs78051721 | 17 | 1829809 | A | G | -0.2581 | 0.0578 | 0.0172 | 7.99E-06 | 10913 |
|  | rs7210455 | 17 | 2295118 | T | C | 0.1349 | 0.0304 | 0.0564 | 9.10E-06 | 11790 |
|  | rs55914821 | 17 | 65264208 | A | G | 0.0748 | 0.0159 | 0.7583 | 2.55E-06 | 11344 |
|  | rs113800750 | 18 | 22485775 | A | G | 0.3221 | 0.071 | 0.0171 | 5.72E-06 | 8817 |
|  | rs76481949 | 18 | 39700979 | A | G | 0.1152 | 0.0254 | 0.923 | 5.75E-06 | 11344 |
|  | rs8098045 | 18 | 62141503 | A | T | 0.0894 | 0.0197 | 0.1285 | 5.68E-06 | 11783 |
|  | rs189767513 | 19 | 10721256 | A | G | -0.3355 | 0.0752 | 0.0119 | 8.14E-06 | 9683 |
| TNFRSF9 | rs1776354 | 1 | 7972201 | A | G | -0.1301 | 0.013 | 0.5203 | 1.41E-23 | 11783 |
|  | rs77558588 | 1 | 201306582 | C | G | -0.2033 | 0.0455 | 0.0259 | 7.89E-06 | 11783 |
|  | rs35055143 | 1 | 227472665 | A | C | -0.0668 | 0.0151 | 0.6196 | 9.70E-06 | 9452 |
|  | rs10178845 | 2 | 8443803 | A | G | -0.0682 | 0.0144 | 0.3043 | 2.18E-06 | 11765 |
|  | rs2579649 | 2 | 120226781 | A | C | -0.0968 | 0.0199 | 0.1322 | 1.15E-06 | 11336 |
|  | rs56079234 | 2 | 230367155 | A | G | 0.0647 | 0.0145 | 0.6831 | 8.12E-06 | 11335 |
|  | rs77827048 | 3 | 102282330 | A | C | 0.2584 | 0.0575 | 0.0183 | 6.99E-06 | 10549 |
|  | rs150949259 | 3 | 177868759 | A | G | 0.375 | 0.0809 | 0.0104 | 3.56E-06 | 8674 |
|  | rs140214797 | 4 | 28155085 | T | G | 0.2317 | 0.0504 | 0.9796 | 4.28E-06 | 11769 |
|  | rs72964492 | 4 | 153651229 | A | C | 0.3111 | 0.065 | 0.9869 | 1.70E-06 | 9983 |
|  | rs4016108 | 5 | 67501872 | T | G | 0.0705 | 0.0159 | 0.5159 | 9.25E-06 | 8807 |
|  | rs7737635 | 5 | 124329728 | A | G | 0.0924 | 0.0201 | 0.1947 | 4.29E-06 | 9453 |
|  | rs75246422 | 5 | 174179760 | T | C | -0.0982 | 0.0203 | 0.1231 | 1.32E-06 | 11784 |
|  | rs4976685 | 5 | 176766177 | A | G | -0.0693 | 0.0139 | 0.6695 | 6.18E-07 | 11763 |
|  | rs2854008 | 6 | 31312538 | A | G | 0.0926 | 0.0149 | 0.2629 | 5.14E-10 | 11784 |
|  | rs13229950 | 7 | 82342738 | A | G | -0.1713 | 0.0381 | 0.9665 | 6.92E-06 | 11779 |
|  | rs146380867 | 8 | 50188685 | A | G | 0.3742 | 0.0814 | 0.0105 | 4.29E-06 | 10849 |
|  | rs10101219 | 8 | 99918668 | C | G | -0.4015 | 0.0847 | 0.9895 | 2.13E-06 | 9540 |
|  | rs1846520 | 8 | 118333064 | A | G | -0.1626 | 0.0313 | 0.9533 | 2.05E-07 | 11782 |
|  | rs7858310 | 9 | 87717237 | A | G | 0.0707 | 0.0152 | 0.7165 | 3.30E-06 | 11336 |
|  | rs78639230 | 9 | 137015396 | C | G | 0.3202 | 0.0703 | 0.0119 | 5.24E-06 | 9320 |
|  | rs11202989 | 10 | 90880926 | T | C | -0.0995 | 0.0212 | 0.1219 | 2.69E-06 | 11336 |
|  | rs72850206 | 11 | 2632174 | A | T | -0.1438 | 0.0281 | 0.0736 | 3.10E-07 | 11336 |
|  | rs9669611 | 12 | 6503786 | T | C | 0.0703 | 0.015 | 0.2853 | 2.78E-06 | 11783 |
|  | rs12227836 | 12 | 67215987 | A | G | -0.0669 | 0.014 | 0.3517 | 1.77E-06 | 11336 |
|  | rs3184504 | 12 | 111884608 | T | C | 0.0681 | 0.013 | 0.476 | 1.62E-07 | 11784 |
|  | rs10744409 | 12 | 129783438 | A | G | 0.064 | 0.0131 | 0.4447 | 1.03E-06 | 11764 |
|  | rs76428106 | 13 | 28604007 | T | C | -0.2714 | 0.0593 | 0.9828 | 4.72E-06 | 10849 |
|  | rs143274881 | 13 | 58754621 | A | T | -0.3325 | 0.0719 | 0.9767 | 3.76E-06 | 9353 |
|  | rs12874404 | 13 | 108993494 | A | G | -0.1402 | 0.0296 | 0.9406 | 2.17E-06 | 11336 |
|  | rs8005108 | 14 | 20709993 | C | G | 0.0889 | 0.0182 | 0.2828 | 1.04E-06 | 9453 |
|  | rs57700441 | 14 | 63713358 | C | G | -0.1155 | 0.0228 | 0.901 | 4.07E-07 | 11773 |
|  | rs118085931 | 15 | 26407103 | A | G | -0.182 | 0.0403 | 0.9684 | 6.30E-06 | 11784 |
|  | rs144141891 | 16 | 4732224 | T | C | 0.2076 | 0.0463 | 0.025 | 7.33E-06 | 10288 |
|  | rs118083884 | 17 | 16522922 | A | G | 0.3284 | 0.0668 | 0.019 | 8.83E-07 | 9785 |
|  | rs1645331 | 19 | 49126012 | C | G | 0.097 | 0.0206 | 0.8419 | 2.49E-06 | 9033 |
|  | rs192538756 | 20 | 2629394 | A | T | 0.1189 | 0.0242 | 0.8742 | 8.96E-07 | 9453 |
| TNFSF14 | rs2689177 | 1 | 238931099 | A | C | 0.0596 | 0.0132 | 0.4939 | 6.33E-06 | 11777 |
|  | rs62139794 | 2 | 13487034 | A | G | -0.2717 | 0.0612 | 0.0172 | 9.02E-06 | 10514 |
|  | rs10211598 | 2 | 137066601 | T | C | -0.0628 | 0.0137 | 0.6354 | 4.56E-06 | 11770 |
|  | rs7688836 | 4 | 94769686 | A | G | -0.0818 | 0.0166 | 0.2019 | 8.32E-07 | 11791 |
|  | rs147119146 | 4 | 125709779 | A | C | -0.2421 | 0.0498 | 0.0299 | 1.17E-06 | 11788 |
|  | rs76778362 | 5 | 39861942 | C | G | -0.2117 | 0.043 | 0.0273 | 8.51E-07 | 11791 |
|  | rs146894100 | 5 | 105199680 | A | G | -0.2109 | 0.0469 | 0.0281 | 6.90E-06 | 10919 |
|  | rs11950562 | 5 | 131652529 | A | C | 0.0785 | 0.0132 | 0.5494 | 2.73E-09 | 11793 |
|  | rs9295562 | 6 | 22713651 | T | C | -0.0651 | 0.0141 | 0.3144 | 3.89E-06 | 11771 |
|  | rs976083 | 6 | 46064645 | A | G | 0.06 | 0.0134 | 0.4237 | 7.55E-06 | 11345 |
|  | rs115950744 | 6 | 56788897 | A | G | -0.2574 | 0.0506 | 0.0213 | 3.64E-07 | 11302 |
|  | rs144647892 | 6 | 161103013 | A | G | -0.2121 | 0.0469 | 0.9619 | 6.11E-06 | 8974 |
|  | rs117442125 | 7 | 28896826 | A | T | 0.1692 | 0.0364 | 0.9583 | 3.35E-06 | 11345 |
|  | rs11546674 | 7 | 150497420 | A | C | -0.0612 | 0.0135 | 0.477 | 5.81E-06 | 11345 |
|  | rs78336300 | 8 | 27416526 | A | C | 0.2392 | 0.0522 | 0.0209 | 4.60E-06 | 10917 |
|  | rs117720118 | 8 | 36844946 | T | G | -0.1456 | 0.0303 | 0.0551 | 1.55E-06 | 11773 |
|  | rs79233212 | 8 | 99636603 | A | G | -0.2358 | 0.0506 | 0.023 | 3.16E-06 | 11345 |
|  | rs78847273 | 9 | 20100286 | T | C | 0.218 | 0.0487 | 0.025 | 7.59E-06 | 11345 |
|  | rs12377591 | 9 | 24791718 | A | G | 0.0707 | 0.0155 | 0.4744 | 5.08E-06 | 8817 |
|  | rs59801342 | 9 | 132432461 | A | G | 0.229 | 0.0497 | 0.024 | 4.07E-06 | 10721 |
|  | rs79791117 | 10 | 111146614 | T | C | -0.2336 | 0.0511 | 0.9787 | 4.84E-06 | 10903 |
|  | rs11026443 | 11 | 1442784 | T | C | 0.1578 | 0.0352 | 0.047 | 7.36E-06 | 10281 |
|  | rs78798615 | 11 | 35451538 | A | C | -0.2602 | 0.0579 | 0.0214 | 6.99E-06 | 8975 |
|  | rs118011483 | 11 | 47589106 | A | C | -0.0856 | 0.0191 | 0.1365 | 7.41E-06 | 11772 |
|  | rs1461044 | 12 | 74925803 | T | C | -0.1519 | 0.0316 | 0.0635 | 1.53E-06 | 11792 |
|  | rs11105725 | 12 | 91045724 | T | C | -0.0683 | 0.0147 | 0.3338 | 3.38E-06 | 11345 |
|  | rs4772260 | 13 | 100645723 | T | C | 0.0619 | 0.0135 | 0.5736 | 4.54E-06 | 11790 |
|  | rs184708984 | 15 | 23989218 | T | C | -0.6334 | 0.1109 | 0.0083 | 1.12E-08 | 6942 |
|  | rs74600570 | 15 | 79463978 | T | C | -0.2255 | 0.0472 | 0.0287 | 1.77E-06 | 11778 |
|  | rs2738591 | 16 | 78681855 | T | C | -0.063 | 0.0133 | 0.5474 | 2.17E-06 | 11789 |
|  | rs56025355 | 17 | 62499163 | T | C | 0.328 | 0.074 | 0.0109 | 9.32E-06 | 10017 |
|  | rs9961731 | 18 | 11626563 | A | G | 0.1048 | 0.0208 | 0.1883 | 4.69E-07 | 9461 |
|  | rs9962377 | 18 | 70105531 | T | C | -0.0905 | 0.0186 | 0.1483 | 1.14E-06 | 11793 |
|  | rs11085155 | 19 | 5968688 | A | G | -0.0811 | 0.0176 | 0.1772 | 4.07E-06 | 11774 |
|  | rs344560 | 19 | 6665020 | T | C | -0.4894 | 0.0273 | 0.0625 | 7.29E-72 | 11793 |
|  | rs11569551 | 19 | 6682873 | A | G | 0.2584 | 0.0327 | 0.0429 | 2.74E-15 | 11793 |
|  | rs118073563 | 19 | 28635804 | T | G | 0.3514 | 0.0771 | 0.0098 | 5.17E-06 | 10858 |
|  | rs76688046 | 19 | 29938464 | T | C | 0.1032 | 0.0215 | 0.8112 | 1.59E-06 | 7942 |
| TRAIL | rs7530513 | 1 | 89409911 | A | G | 0.0516 | 0.0114 | 0.4801 | 6.00E-06 | 14733 |
|  | rs78937490 | 1 | 110159876 | A | G | -0.1368 | 0.0302 | 0.044 | 5.90E-06 | 14729 |
|  | rs16840522 | 1 | 196710916 | T | C | -0.1191 | 0.0147 | 0.8073 | 5.40E-16 | 14725 |
|  | rs147647664 | 2 | 1226771 | T | G | 0.0628 | 0.0141 | 0.4351 | 8.43E-06 | 11538 |
|  | rs2138380 | 2 | 203169224 | A | G | 0.0882 | 0.0182 | 0.8717 | 1.26E-06 | 13239 |
|  | rs368805685 | 2 | 209615008 | A | G | -0.2297 | 0.0501 | 0.0323 | 4.54E-06 | 11760 |
|  | rs114316325 | 3 | 31722698 | A | G | 0.1317 | 0.0292 | 0.0477 | 6.47E-06 | 14287 |
|  | rs28424593 | 3 | 113950321 | A | T | -0.1311 | 0.0279 | 0.9501 | 2.62E-06 | 14722 |
|  | rs62284704 | 3 | 155453189 | A | C | 0.089 | 0.0201 | 0.9101 | 9.52E-06 | 14734 |
|  | rs490342 | 3 | 172130799 | T | G | 0.1252 | 0.02 | 0.1051 | 3.85E-10 | 13228 |
|  | rs79287178 | 3 | 172294500 | A | G | -0.4891 | 0.0342 | 0.0333 | 2.15E-46 | 14718 |
|  | rs5030044 | 3 | 186449122 | A | G | 0.285 | 0.0187 | 0.886 | 1.90E-52 | 14287 |
|  | rs78031538 | 4 | 36596252 | A | G | -0.1712 | 0.038 | 0.9724 | 6.63E-06 | 14729 |
|  | rs146922303 | 4 | 57703725 | T | C | 0.1622 | 0.0347 | 0.0376 | 2.95E-06 | 14727 |
|  | rs142889372 | 4 | 67749461 | C | G | -0.2835 | 0.0606 | 0.0106 | 2.89E-06 | 14238 |
|  | rs145386370 | 4 | 69327121 | T | G | 0.2641 | 0.0535 | 0.019 | 7.96E-07 | 13386 |
|  | rs74341217 | 4 | 143620954 | T | C | -0.0823 | 0.0184 | 0.1166 | 7.72E-06 | 14730 |
|  | rs17779404 | 4 | 181271289 | T | G | 0.0674 | 0.0138 | 0.7733 | 1.04E-06 | 14723 |
|  | rs114597610 | 5 | 75017740 | T | C | -0.26 | 0.0564 | 0.0161 | 4.03E-06 | 14735 |
|  | rs111257277 | 5 | 162595921 | A | G | 0.189 | 0.037 | 0.9721 | 3.25E-07 | 14729 |
|  | rs78956561 | 6 | 18023031 | A | T | 0.1694 | 0.0354 | 0.0304 | 1.71E-06 | 14719 |
|  | rs1892247 | 6 | 25887657 | A | G | -0.0756 | 0.0158 | 0.1589 | 1.71E-06 | 14719 |
|  | rs140862652 | 6 | 33692744 | A | C | 0.1342 | 0.0265 | 0.063 | 4.10E-07 | 14287 |
|  | rs2894910 | 6 | 57391519 | T | C | 0.0807 | 0.0175 | 0.5094 | 4.00E-06 | 9830 |
|  | rs1065657 | 6 | 90047013 | A | G | 0.0655 | 0.0118 | 0.4536 | 2.84E-08 | 14287 |
|  | rs6969337 | 7 | 52792820 | T | C | 0.1823 | 0.038 | 0.9624 | 1.61E-06 | 11336 |
|  | rs148390525 | 7 | 93756130 | A | T | -0.2024 | 0.0457 | 0.9793 | 9.47E-06 | 14720 |
|  | rs141011299 | 7 | 135852098 | A | G | -0.5024 | 0.1076 | 0.0051 | 3.02E-06 | 9812 |
|  | rs113805560 | 9 | 777415 | A | G | 0.1173 | 0.0262 | 0.9438 | 7.57E-06 | 14722 |
|  | rs2418781 | 10 | 107586667 | T | G | -0.1452 | 0.0316 | 0.0401 | 4.33E-06 | 14287 |
|  | rs183978373 | 11 | 18290358 | A | C | 0.1014 | 0.0229 | 0.0834 | 9.51E-06 | 12780 |
|  | rs174533 | 11 | 61549025 | A | G | 0.0787 | 0.0121 | 0.3509 | 7.81E-11 | 14732 |
|  | rs180941492 | 11 | 103163928 | A | G | -0.244 | 0.0491 | 0.0227 | 6.71E-07 | 13800 |
|  | rs28929474 | 14 | 94844947 | T | C | 0.6692 | 0.044 | 0.0205 | 3.08E-52 | 14735 |
|  | rs17580 | 14 | 94847262 | A | T | 0.1402 | 0.0316 | 0.0459 | 9.13E-06 | 14287 |
|  | rs2113173 | 16 | 57345733 | T | C | 0.0691 | 0.0145 | 0.2543 | 1.88E-06 | 14287 |
|  | rs77303550 | 16 | 72079657 | T | C | -0.0724 | 0.0146 | 0.1946 | 7.09E-07 | 14728 |
|  | rs8178824 | 17 | 64224775 | T | C | 0.2575 | 0.037 | 0.029 | 3.42E-12 | 14735 |
|  | rs654488 | 18 | 29804863 | A | T | -0.0894 | 0.0115 | 0.4593 | 7.61E-15 | 14735 |
|  | rs4760 | 19 | 44153100 | A | G | 0.3554 | 0.0163 | 0.8425 | 2.14E-105 | 14287 |
|  | rs201992180 | 20 | 15851839 | A | T | -0.121 | 0.0272 | 0.0623 | 8.65E-06 | 12205 |
| TRANCE | rs72636362 | 1 | 1973608 | A | G | 0.302 | 0.0635 | 0.9818 | 1.98E-06 | 11728 |
|  | rs34221978 | 1 | 22143382 | A | G | 0.167 | 0.0344 | 0.0349 | 1.21E-06 | 14734 |
|  | rs57217149 | 1 | 109697809 | A | T | -0.1093 | 0.0244 | 0.9358 | 7.48E-06 | 14724 |
|  | rs72781813 | 2 | 17337409 | T | G | 0.2368 | 0.0486 | 0.9817 | 1.10E-06 | 14735 |
|  | rs113835939 | 2 | 30655453 | A | C | -0.1939 | 0.0416 | 0.976 | 3.15E-06 | 14732 |
|  | rs76488966 | 2 | 104163036 | T | G | -0.1719 | 0.0366 | 0.9693 | 2.64E-06 | 14720 |
|  | rs201831835 | 2 | 136379377 | A | C | 0.1377 | 0.0285 | 0.9227 | 1.35E-06 | 10894 |
|  | rs77980965 | 3 | 83133339 | T | C | -0.2976 | 0.0641 | 0.9853 | 3.44E-06 | 11337 |
|  | rs67702765 | 3 | 126494760 | A | G | 0.0678 | 0.0152 | 0.8145 | 8.18E-06 | 14719 |
|  | rs329079 | 3 | 168233747 | A | C | -0.0914 | 0.0201 | 0.094 | 5.43E-06 | 14736 |
|  | rs507759 | 3 | 172153341 | A | G | -0.0602 | 0.0133 | 0.7426 | 6.00E-06 | 14727 |
|  | rs79287178 | 3 | 172294500 | A | G | -0.4987 | 0.0343 | 0.0334 | 6.82E-48 | 14719 |
|  | rs11713634 | 3 | 194061578 | A | G | -0.0978 | 0.015 | 0.2943 | 7.03E-11 | 11336 |
|  | rs181906998 | 4 | 8958677 | A | T | -0.1043 | 0.0211 | 0.7203 | 7.69E-07 | 9987 |
|  | rs34227934 | 4 | 25729339 | C | G | 0.0857 | 0.0189 | 0.1149 | 5.78E-06 | 14288 |
|  | rs4365726 | 4 | 105828656 | A | G | -0.0777 | 0.0166 | 0.1504 | 2.86E-06 | 14287 |
|  | rs150011668 | 4 | 111911027 | T | C | -0.1376 | 0.0281 | 0.9058 | 9.74E-07 | 11538 |
|  | rs76878863 | 5 | 55435535 | T | C | -0.1274 | 0.0284 | 0.0553 | 7.26E-06 | 14288 |
|  | rs150128402 | 5 | 115874345 | A | G | 0.2386 | 0.0459 | 0.9763 | 2.01E-07 | 14736 |
|  | rs1432791 | 5 | 160174315 | T | C | -0.0549 | 0.0118 | 0.4357 | 3.28E-06 | 14288 |
|  | rs17221073 | 6 | 33048371 | C | G | 0.2646 | 0.0498 | 0.018 | 1.08E-07 | 13801 |
|  | rs62394571 | 6 | 40514231 | T | C | -0.1649 | 0.0363 | 0.0319 | 5.55E-06 | 14736 |
|  | rs12201845 | 6 | 57933508 | A | G | -0.1884 | 0.0426 | 0.972 | 9.75E-06 | 12798 |
|  | rs62415545 | 6 | 69144519 | A | G | -0.0963 | 0.0216 | 0.9079 | 8.26E-06 | 12851 |
|  | rs6932751 | 6 | 90249507 | A | G | 0.054 | 0.0116 | 0.5245 | 3.24E-06 | 14730 |
|  | rs1273754 | 6 | 121479140 | T | C | -0.2296 | 0.0503 | 0.9805 | 5.00E-06 | 10850 |
|  | rs3801000 | 7 | 50811081 | A | G | 0.0686 | 0.013 | 0.2872 | 1.31E-07 | 14288 |
|  | rs17302 | 7 | 142245686 | T | G | -0.0625 | 0.013 | 0.2875 | 1.53E-06 | 14288 |
|  | rs2975421 | 8 | 19490180 | T | G | -0.078 | 0.017 | 0.8529 | 4.47E-06 | 14288 |
|  | rs62501135 | 8 | 23089208 | A | G | -0.1532 | 0.0148 | 0.2051 | 4.13E-25 | 14288 |
|  | rs183217699 | 8 | 83420230 | C | G | -0.1254 | 0.0272 | 0.9313 | 4.02E-06 | 12852 |
|  | rs17327042 | 8 | 100623060 | T | G | 0.2542 | 0.0568 | 0.9834 | 7.63E-06 | 13988 |
|  | rs2468187 | 8 | 120201029 | T | C | -0.1618 | 0.0118 | 0.4162 | 8.62E-43 | 14735 |
|  | rs5001566 | 9 | 16905616 | A | T | 0.1215 | 0.0265 | 0.0705 | 4.54E-06 | 11982 |
|  | rs6479556 | 9 | 93147790 | T | G | 0.07 | 0.0148 | 0.8064 | 2.25E-06 | 14728 |
|  | rs10980859 | 9 | 114062291 | T | C | -0.1271 | 0.0285 | 0.9379 | 8.21E-06 | 11785 |
|  | rs11592666 | 10 | 12566602 | A | G | 0.2268 | 0.0513 | 0.9812 | 9.82E-06 | 13340 |
|  | rs11258162 | 10 | 13097349 | T | G | 0.0891 | 0.0201 | 0.1277 | 9.30E-06 | 11760 |
|  | rs78045189 | 11 | 37130843 | C | G | -0.0695 | 0.0151 | 0.3443 | 4.17E-06 | 11760 |
|  | rs656095 | 11 | 75445338 | A | G | -0.0665 | 0.0149 | 0.8042 | 8.08E-06 | 14288 |
|  | rs79837331 | 12 | 22855883 | T | C | 0.0902 | 0.0204 | 0.104 | 9.80E-06 | 14288 |
|  | rs75991751 | 12 | 129430611 | T | G | -0.2685 | 0.0539 | 0.9818 | 6.31E-07 | 13422 |
|  | rs12874439 | 13 | 40870237 | T | G | -0.0578 | 0.013 | 0.3162 | 8.74E-06 | 14288 |
|  | rs4512994 | 13 | 43039673 | A | C | 0.1172 | 0.0116 | 0.4634 | 5.33E-24 | 14736 |
|  | rs4334157 | 13 | 68202750 | T | C | -0.1684 | 0.0356 | 0.0319 | 2.24E-06 | 14736 |
|  | rs201650130 | 14 | 84106660 | T | C | -0.0822 | 0.0181 | 0.1494 | 5.59E-06 | 12194 |
|  | rs704 | 17 | 26694861 | A | G | -0.1085 | 0.0116 | 0.4755 | 8.48E-21 | 14736 |
|  | rs139464717 | 17 | 64316544 | A | G | -0.289 | 0.0652 | 0.0124 | 9.31E-06 | 13801 |
|  | rs150416959 | 19 | 35131252 | C | G | 0.1462 | 0.0315 | 0.0491 | 3.46E-06 | 13224 |
|  | rs117113213 | 20 | 39165692 | A | G | -0.1646 | 0.03 | 0.0498 | 4.10E-08 | 14723 |
|  | rs6063153 | 20 | 46454945 | T | C | 0.0636 | 0.0126 | 0.3364 | 4.47E-07 | 14287 |
|  | rs151333895 | 22 | 22966430 | A | T | -0.4148 | 0.091 | 0.0108 | 5.16E-06 | 10553 |
| TSLP | rs7550374 | 1 | 240843947 | C | G | -0.0928 | 0.0187 | 0.8143 | 6.96E-07 | 11345 |
|  | rs12472785 | 2 | 2626966 | C | G | -0.074 | 0.0153 | 0.758 | 1.32E-06 | 11792 |
|  | rs4270392 | 2 | 41752458 | T | C | 0.0625 | 0.0137 | 0.3566 | 5.07E-06 | 11777 |
|  | rs13414099 | 2 | 239484610 | T | C | -0.0834 | 0.0178 | 0.4567 | 2.79E-06 | 7943 |
|  | rs145549228 | 3 | 49668933 | A | G | -0.18 | 0.0405 | 0.9664 | 8.81E-06 | 10727 |
|  | rs56384800 | 6 | 371056 | A | G | -0.1073 | 0.0238 | 0.6328 | 6.53E-06 | 6879 |
|  | rs4724126 | 7 | 42730058 | T | G | 0.0846 | 0.0152 | 0.2624 | 2.61E-08 | 11344 |
|  | rs72652144 | 8 | 69813968 | A | G | 0.1629 | 0.0345 | 0.9535 | 2.34E-06 | 11782 |
|  | rs112206094 | 9 | 23277990 | A | T | -0.0934 | 0.0201 | 0.1258 | 3.37E-06 | 11792 |
|  | rs147525049 | 10 | 6676153 | T | C | 0.1808 | 0.0401 | 0.0322 | 6.52E-06 | 11302 |
|  | rs117095006 | 10 | 34395970 | A | G | -0.1569 | 0.0338 | 0.0499 | 3.45E-06 | 11345 |
|  | rs138903443 | 10 | 46165215 | T | C | -0.1271 | 0.0281 | 0.0803 | 6.09E-06 | 11345 |
|  | rs72838667 | 10 | 102720477 | A | C | 0.1469 | 0.0329 | 0.0429 | 8.01E-06 | 11778 |
|  | rs72811179 | 10 | 108726426 | T | C | -0.2956 | 0.063 | 0.0141 | 2.70E-06 | 11345 |
|  | rs36046025 | 10 | 124936058 | A | G | -0.1223 | 0.0268 | 0.0756 | 5.03E-06 | 11345 |
|  | rs145111430 | 11 | 121946210 | A | G | 0.2674 | 0.0575 | 0.9812 | 3.31E-06 | 11345 |
|  | rs190906347 | 15 | 60209215 | A | C | 0.3219 | 0.066 | 0.0132 | 1.08E-06 | 10858 |
|  | rs140886020 | 15 | 79915029 | A | G | 0.1619 | 0.0351 | 0.0381 | 3.98E-06 | 11786 |
|  | rs12448125 | 16 | 8805947 | T | G | 0.1784 | 0.0367 | 0.0353 | 1.17E-06 | 11792 |
|  | rs9928464 | 16 | 77742082 | T | G | 0.1233 | 0.0278 | 0.0736 | 9.20E-06 | 11345 |
|  | rs71366574 | 17 | 3661052 | A | G | 0.1886 | 0.0416 | 0.0299 | 5.80E-06 | 11793 |
|  | rs118119134 | 17 | 27504563 | A | G | -0.4423 | 0.0943 | 0.0096 | 2.73E-06 | 8576 |
|  | rs7406537 | 17 | 80919387 | A | G | -0.0643 | 0.0139 | 0.4553 | 3.73E-06 | 11149 |
|  | rs185922111 | 20 | 37959633 | A | G | 0.3618 | 0.0747 | 0.9877 | 1.28E-06 | 10858 |
|  | rs9977976 | 21 | 15251501 | T | C | -0.1361 | 0.03 | 0.1902 | 5.72E-06 | 7036 |
| TNFSF12 | rs12145644 | 1 | 162517994 | A | T | -0.0727 | 0.0141 | 0.2484 | 2.52E-07 | 14287 |
|  | rs2738752 | 1 | 221052765 | A | G | -0.0591 | 0.0129 | 0.6653 | 4.62E-06 | 12848 |
|  | rs12726054 | 1 | 233623860 | A | G | 0.109 | 0.0228 | 0.0734 | 1.75E-06 | 14721 |
|  | rs870526 | 2 | 20369562 | T | C | -0.0508 | 0.0114 | 0.5377 | 8.34E-06 | 14731 |
|  | rs2163333 | 2 | 53077754 | A | G | 0.1404 | 0.0273 | 0.9522 | 2.71E-07 | 14719 |
|  | rs201620545 | 2 | 99371601 | A | G | -0.0849 | 0.0168 | 0.8184 | 4.34E-07 | 11327 |
|  | rs147054593 | 2 | 109129667 | C | G | 0.057 | 0.0125 | 0.6122 | 5.12E-06 | 12851 |
|  | rs76854434 | 2 | 140022077 | A | G | 0.2416 | 0.0542 | 0.0173 | 8.29E-06 | 14288 |
|  | rs11676293 | 2 | 153624823 | A | G | 0.0547 | 0.012 | 0.3554 | 5.16E-06 | 14721 |
|  | rs3821473 | 3 | 59625877 | A | G | -0.0622 | 0.0136 | 0.2327 | 4.80E-06 | 14736 |
|  | rs73133996 | 3 | 98429219 | C | G | 0.0812 | 0.012 | 0.4558 | 1.32E-11 | 14288 |
|  | rs57053056 | 3 | 128983680 | T | G | -0.0841 | 0.0187 | 0.1057 | 6.88E-06 | 14730 |
|  | rs1965399 | 3 | 142705695 | A | G | -0.1312 | 0.028 | 0.9544 | 2.79E-06 | 14736 |
|  | rs9842051 | 3 | 143021856 | C | G | 0.1192 | 0.014 | 0.748 | 1.68E-17 | 14288 |
|  | rs76188822 | 3 | 158730534 | A | C | -0.2352 | 0.0525 | 0.9863 | 7.46E-06 | 14736 |
|  | rs78254232 | 3 | 174384187 | T | C | -0.2108 | 0.0456 | 0.9813 | 3.79E-06 | 14248 |
|  | rs13107325 | 4 | 103188709 | T | C | 0.2027 | 0.0236 | 0.0696 | 8.77E-18 | 14733 |
|  | rs10010472 | 4 | 157843781 | A | G | 0.0559 | 0.0126 | 0.7019 | 9.14E-06 | 14732 |
|  | rs11934678 | 4 | 181200669 | C | G | -0.0969 | 0.0218 | 0.0955 | 8.79E-06 | 14287 |
|  | rs32923 | 5 | 141116062 | A | G | -0.0588 | 0.0131 | 0.7435 | 7.17E-06 | 14732 |
|  | rs11738159 | 5 | 167311493 | T | C | 0.0577 | 0.0119 | 0.4339 | 1.24E-06 | 14288 |
|  | rs9470247 | 6 | 36262483 | A | G | -0.1026 | 0.0232 | 0.9246 | 9.76E-06 | 14288 |
|  | rs117799464 | 6 | 70731400 | A | G | 0.2043 | 0.0443 | 0.9767 | 3.99E-06 | 14288 |
|  | rs16879825 | 6 | 88516736 | A | G | -0.2027 | 0.0438 | 0.9577 | 3.69E-06 | 11784 |
|  | rs144115221 | 8 | 9389180 | T | C | 0.2655 | 0.0582 | 0.0153 | 5.07E-06 | 13944 |
|  | rs11136339 | 8 | 145013345 | T | C | 0.0613 | 0.0122 | 0.388 | 5.05E-07 | 13664 |
|  | rs56023600 | 9 | 5636355 | T | C | -0.0856 | 0.0186 | 0.8764 | 4.18E-06 | 14287 |
|  | rs7019909 | 9 | 33113322 | T | C | -0.0897 | 0.0193 | 0.1102 | 3.36E-06 | 14288 |
|  | rs10818768 | 9 | 125709323 | A | G | 0.0522 | 0.0118 | 0.5021 | 9.70E-06 | 14288 |
|  | rs579459 | 9 | 136154168 | T | C | 0.1098 | 0.0157 | 0.7888 | 2.68E-12 | 11785 |
|  | rs201375822 | 10 | 17895807 | T | C | 0.1082 | 0.0193 | 0.2616 | 2.07E-08 | 10692 |
|  | rs111641307 | 10 | 51775971 | C | G | -0.401 | 0.0871 | 0.9859 | 4.15E-06 | 10446 |
|  | rs76047473 | 10 | 132815955 | T | C | 0.1318 | 0.0286 | 0.0482 | 4.06E-06 | 14718 |
|  | rs10831650 | 11 | 11644391 | A | G | 0.0692 | 0.0125 | 0.3558 | 3.09E-08 | 14288 |
|  | rs1564995 | 11 | 24773107 | A | G | -0.06 | 0.0132 | 0.6158 | 5.48E-06 | 14288 |
|  | rs58743186 | 11 | 130975896 | A | G | -0.0717 | 0.0154 | 0.1931 | 3.23E-06 | 12818 |
|  | rs73590361 | 12 | 592399 | T | C | -0.0744 | 0.0161 | 0.1517 | 3.82E-06 | 14726 |
|  | rs117550445 | 12 | 82487578 | C | G | -0.1855 | 0.0377 | 0.9706 | 8.64E-07 | 14721 |
|  | rs4882710 | 12 | 129101566 | T | C | 0.0666 | 0.0131 | 0.3457 | 3.70E-07 | 14288 |
|  | rs145667392 | 13 | 46956927 | T | C | 0.2414 | 0.0531 | 0.0278 | 5.46E-06 | 11728 |
|  | rs2220833 | 13 | 110957507 | T | C | 0.0559 | 0.0126 | 0.6633 | 9.14E-06 | 14731 |
|  | rs7149501 | 14 | 63176023 | A | G | -0.2707 | 0.0611 | 0.987 | 9.40E-06 | 12591 |
|  | rs137861803 | 16 | 60684681 | G | T | -0.0735 | 0.0162 | 0.7665 | 5.71E-06 | 10894 |
|  | rs34790908 | 17 | 7451110 | T | C | 0.211 | 0.0129 | 0.2563 | 3.90E-60 | 14732 |
|  | rs7503751 | 17 | 7789542 | A | G | -0.1386 | 0.0238 | 0.0779 | 5.76E-09 | 14288 |
|  | rs76133364 | 19 | 19706214 | A | C | 0.1896 | 0.0369 | 0.0315 | 2.77E-07 | 13224 |
|  | rs8116972 | 20 | 18299709 | T | C | -0.0953 | 0.0211 | 0.0804 | 6.28E-06 | 14733 |
| uPA | rs59594243 | 1 | 68043857 | A | G | 0.2821 | 0.0634 | 0.0124 | 8.61E-06 | 13799 |
|  | rs333947 | 1 | 110470764 | A | G | -0.0785 | 0.0166 | 0.1578 | 2.26E-06 | 14286 |
|  | rs202063542 | 1 | 152190722 | T | C | 0.2771 | 0.0607 | 0.9835 | 4.99E-06 | 10407 |
|  | rs3021477 | 1 | 158146946 | T | C | -0.0534 | 0.0119 | 0.4143 | 7.21E-06 | 14730 |
|  | rs2579643 | 2 | 120217456 | T | C | -0.0991 | 0.0223 | 0.0812 | 8.83E-06 | 14285 |
|  | rs115346716 | 2 | 130943956 | A | G | -0.0962 | 0.0212 | 0.1038 | 5.69E-06 | 14286 |
|  | rs7564243 | 2 | 160726868 | A | G | -0.0852 | 0.0114 | 0.5375 | 7.80E-14 | 14729 |
|  | rs6805474 | 3 | 56631258 | A | G | 0.0952 | 0.0176 | 0.1456 | 6.33E-08 | 14285 |
|  | rs13075658 | 3 | 120035865 | T | C | 0.051 | 0.0115 | 0.4491 | 9.22E-06 | 14725 |
|  | rs62286554 | 3 | 193665003 | T | C | -0.091 | 0.019 | 0.8498 | 1.67E-06 | 11335 |
|  | rs1074669 | 4 | 35016704 | A | G | -0.0548 | 0.0119 | 0.5246 | 4.12E-06 | 14286 |
|  | rs12510249 | 4 | 131097432 | T | C | -0.1375 | 0.0308 | 0.0414 | 8.03E-06 | 14729 |
|  | rs148356738 | 4 | 189933250 | C | G | -0.2374 | 0.0528 | 0.9849 | 6.92E-06 | 14728 |
|  | rs4272091 | 5 | 53726016 | T | C | -0.068 | 0.0149 | 0.8015 | 5.02E-06 | 14286 |
|  | rs59772299 | 5 | 126921537 | T | G | 0.0627 | 0.0133 | 0.7552 | 2.43E-06 | 14730 |
|  | rs154972 | 6 | 32900651 | A | G | -0.0625 | 0.012 | 0.6344 | 1.91E-07 | 14724 |
|  | rs9385692 | 6 | 134819263 | T | C | -0.0934 | 0.0211 | 0.903 | 9.58E-06 | 14286 |
|  | rs79858331 | 7 | 137186346 | T | C | 0.1425 | 0.0316 | 0.0429 | 6.50E-06 | 14722 |
|  | rs72727847 | 8 | 139662968 | C | G | 0.1968 | 0.0417 | 0.0253 | 2.37E-06 | 14729 |
|  | rs62560864 | 9 | 34105056 | C | G | -0.1607 | 0.0358 | 0.0328 | 7.16E-06 | 13657 |
|  | rs144159840 | 9 | 79131401 | A | C | -0.3959 | 0.0896 | 0.0081 | 9.94E-06 | 10848 |
|  | rs10746571 | 9 | 80912397 | C | G | 0.0643 | 0.0143 | 0.2342 | 6.91E-06 | 14285 |
|  | rs10992204 | 9 | 94784966 | A | G | 0.1061 | 0.0217 | 0.1034 | 1.01E-06 | 14286 |
|  | rs13299342 | 9 | 136141504 | A | G | 0.1 | 0.017 | 0.1744 | 4.04E-09 | 11781 |
|  | rs2227575 | 10 | 75677840 | A | C | -0.0946 | 0.0115 | 0.5624 | 1.93E-16 | 14729 |
|  | rs10840566 | 11 | 11142087 | A | G | 0.0784 | 0.0154 | 0.1671 | 3.56E-07 | 14733 |
|  | rs4078183 | 11 | 27262279 | T | C | -0.0549 | 0.0124 | 0.3046 | 9.54E-06 | 14722 |
|  | rs11220462 | 11 | 126243952 | A | G | -0.1024 | 0.0161 | 0.1509 | 2.01E-10 | 14731 |
|  | rs144470316 | 12 | 105734364 | C | G | 0.3395 | 0.0656 | 0.0119 | 2.28E-07 | 12303 |
|  | rs597808 | 12 | 111973358 | A | G | 0.0662 | 0.0134 | 0.478 | 7.80E-07 | 11334 |
|  | rs12428473 | 13 | 35421790 | T | C | -0.0633 | 0.0141 | 0.207 | 7.14E-06 | 14734 |
|  | rs62004911 | 14 | 74118324 | C | G | -0.106 | 0.0238 | 0.0878 | 8.44E-06 | 11758 |
|  | rs62031354 | 16 | 9111455 | T | C | 0.1034 | 0.023 | 0.1078 | 6.94E-06 | 11335 |
|  | rs35739738 | 17 | 1023740 | T | C | -0.0955 | 0.0216 | 0.0848 | 9.81E-06 | 14721 |
|  | rs7406661 | 17 | 7063667 | T | C | -0.1107 | 0.0156 | 0.7855 | 1.28E-12 | 14286 |
|  | rs704 | 17 | 26694861 | A | G | 0.0567 | 0.0115 | 0.4758 | 8.20E-07 | 14734 |
|  | rs8081644 | 17 | 63476886 | A | C | -0.101 | 0.0223 | 0.9181 | 5.92E-06 | 14286 |
|  | rs4800787 | 18 | 24686365 | T | C | -0.094 | 0.0125 | 0.3008 | 5.48E-14 | 14730 |
|  | rs17882125 | 19 | 4956994 | T | C | 0.1092 | 0.0247 | 0.0649 | 9.82E-06 | 14712 |
|  | rs77076952 | 19 | 30086896 | A | G | -0.2016 | 0.0454 | 0.0227 | 8.97E-06 | 14734 |
|  | rs4760 | 19 | 44153100 | A | G | 0.1408 | 0.0165 | 0.8426 | 1.42E-17 | 14286 |
|  | rs4251805 | 19 | 44174441 | T | C | -0.422 | 0.0311 | 0.0405 | 6.10E-42 | 14734 |
|  | rs3827385 | 22 | 44388817 | T | C | -0.0661 | 0.0146 | 0.8065 | 5.97E-06 | 14727 |
| VEGF_A | rs1256278 | 1 | 21982702 | A | G | 0.0514 | 0.0116 | 0.4743 | 9.38E-06 | 14742 |
|  | rs2050256 | 1 | 32204683 | A | G | -0.0734 | 0.0152 | 0.8126 | 1.37E-06 | 14739 |
|  | rs61818787 | 1 | 190376438 | T | C | -0.0759 | 0.0148 | 0.8114 | 2.92E-07 | 14732 |
|  | rs115245427 | 1 | 201423401 | A | G | -0.2204 | 0.0483 | 0.0257 | 5.04E-06 | 14738 |
|  | rs183596950 | 4 | 31819358 | A | G | -0.07 | 0.0152 | 0.6438 | 4.12E-06 | 11538 |
|  | rs7688836 | 4 | 94769686 | A | G | -0.0685 | 0.0145 | 0.2065 | 2.31E-06 | 14742 |
|  | rs349853 | 4 | 132813569 | A | G | 0.0815 | 0.0182 | 0.1816 | 7.53E-06 | 12412 |
|  | rs16883415 | 5 | 54043585 | T | C | -0.22 | 0.0457 | 0.9797 | 1.48E-06 | 14744 |
|  | rs114694170 | 5 | 88180196 | T | C | -0.1625 | 0.0267 | 0.9462 | 1.16E-09 | 14728 |
|  | rs11953790 | 5 | 117371187 | T | C | -0.1842 | 0.0374 | 0.028 | 8.43E-07 | 14730 |
|  | rs62401205 | 6 | 43855489 | A | C | -0.2112 | 0.0333 | 0.0429 | 2.26E-10 | 14296 |
|  | rs6921438 | 6 | 43925607 | A | G | -0.4343 | 0.0113 | 0.4867 | 3.45e-323 | 14733 |
|  | rs9472184 | 6 | 43940269 | A | G | -0.0602 | 0.0124 | 0.5217 | 1.20E-06 | 14296 |
|  | rs7808444 | 7 | 68565319 | A | C | -0.0628 | 0.0137 | 0.6119 | 4.56E-06 | 14296 |
|  | rs1208181 | 7 | 144108520 | T | C | -0.079 | 0.0166 | 0.8309 | 1.95E-06 | 12855 |
|  | rs4875579 | 8 | 5413008 | C | G | -0.0741 | 0.0158 | 0.8345 | 2.73E-06 | 14296 |
|  | rs62496673 | 8 | 19627714 | T | C | -0.0579 | 0.0129 | 0.2957 | 7.18E-06 | 14728 |
|  | rs143420877 | 8 | 51696312 | T | C | 0.1357 | 0.0304 | 0.039 | 8.05E-06 | 14737 |
|  | rs117976551 | 8 | 73592332 | C | G | -0.2163 | 0.0449 | 0.9739 | 1.45E-06 | 13809 |
|  | rs6993770 | 8 | 106581528 | A | T | 0.1231 | 0.0134 | 0.7319 | 4.06E-20 | 14296 |
|  | rs7852409 | 9 | 2621482 | C | G | 0.0677 | 0.0149 | 0.2413 | 5.53E-06 | 14296 |
|  | rs11789392 | 9 | 2694914 | T | C | 0.1383 | 0.0131 | 0.5728 | 4.70E-26 | 12409 |
|  | rs145703410 | 9 | 3020960 | T | C | -0.2475 | 0.0539 | 0.0153 | 4.39E-06 | 14296 |
|  | rs10822155 | 10 | 65071215 | A | C | 0.0838 | 0.0117 | 0.4155 | 7.93E-13 | 14744 |
|  | rs12784822 | 10 | 104076091 | A | G | -0.3266 | 0.0715 | 0.0091 | 4.93E-06 | 14296 |
|  | rs10840177 | 11 | 2028103 | C | G | -0.0683 | 0.0142 | 0.7087 | 1.51E-06 | 13231 |
|  | rs77998976 | 11 | 19408337 | A | G | 0.1842 | 0.0415 | 0.9765 | 9.06E-06 | 13870 |
|  | rs34536806 | 11 | 95274487 | T | G | 0.0992 | 0.0208 | 0.9024 | 1.85E-06 | 14296 |
|  | rs6582666 | 12 | 46909886 | C | G | -0.1288 | 0.0265 | 0.9341 | 1.17E-06 | 11791 |
|  | rs145846107 | 12 | 75261276 | T | C | 0.2736 | 0.0582 | 0.0162 | 2.59E-06 | 13422 |
|  | rs138648222 | 13 | 29335624 | C | G | 0.1981 | 0.0397 | 0.0248 | 6.04E-07 | 14739 |
|  | rs9603455 | 13 | 39431945 | T | C | 0.1461 | 0.0327 | 0.966 | 7.90E-06 | 14743 |
|  | rs144037665 | 13 | 77462624 | A | C | -0.2779 | 0.0603 | 0.9871 | 4.05E-06 | 11969 |
|  | rs1203834 | 16 | 231541 | T | C | 0.1174 | 0.0253 | 0.069 | 3.48E-06 | 13232 |
|  | rs75756626 | 16 | 26100468 | A | C | 0.0997 | 0.0225 | 0.0763 | 9.37E-06 | 14726 |
|  | rs9893371 | 17 | 46657862 | A | C | 0.142 | 0.0319 | 0.9646 | 8.53E-06 | 14742 |
|  | rs62640435 | 19 | 14759939 | T | C | -0.0671 | 0.0127 | 0.296 | 1.27E-07 | 14292 |
|  | rs13047127 | 21 | 37451947 | A | G | 0.0794 | 0.0175 | 0.8764 | 5.70E-06 | 14743 |
|  | | | | | | | | | | |

|  | **Supplementary Table S3. Summary information for SNPs that were used as genetic instruments for Mendelian randomization analyses of IBD.** | | | | | | | | | | | | | | | | | | |  |
| --- | --- | --- | --- | --- | --- | --- | --- | --- | --- | --- | --- | --- | --- | --- | --- | --- | --- | --- | --- | --- |
|  |  | **SNPs** | **EA** | | **OA** | | **Beta** | | **SE** | | **EAF** | | | **P** | | | | **N** | |  |
|  | IBD | rs61766198 | T | | C | | -0.098 | | 0.017 | | 0.743 | | | 1.07E-08 | | | | 412181 | |  |
|  |  | rs12736494 | A | | G | | -0.113 | | 0.017 | | 0.265 | | | 8.14E-11 | | | | 412181 | |  |
|  |  | rs10737481 | G | | T | | 0.146 | | 0.015 | | 0.509 | | | 2.47E-22 | | | | 412181 | |  |
|  |  | rs148170789 | G | | A | | -0.444 | | 0.041 | | 0.046 | | | 1.38E-27 | | | | 412181 | |  |
|  |  | rs376365394 | T | | C | | 0.254 | | 0.036 | | 0.040 | | | 1.58E-12 | | | | 412181 | |  |
|  |  | rs191615076 | A | | C | | 0.254 | | 0.036 | | 0.040 | | | 1.94E-12 | | | | 412181 | |  |
|  |  | rs12139150 | C | | G | | -0.142 | | 0.015 | | 0.446 | | | 1.01E-20 | | | | 412181 | |  |
|  |  | rs12132298 | C | | T | | -0.150 | | 0.019 | | 0.213 | | | 2.35E-15 | | | | 412181 | |  |
|  |  | rs3024505 | A | | G | | 0.236 | | 0.020 | | 0.157 | | | 2.52E-33 | | | | 412181 | |  |
|  |  | rs6545766 | T | | C | | 0.084 | | 0.015 | | 0.415 | | | 4.37E-08 | | | | 412181 | |  |
|  |  | rs7608910 | G | | A | | 0.100 | | 0.015 | | 0.369 | | | 9.92E-11 | | | | 412181 | |  |
|  |  | rs1921622 | A | | G | | -0.090 | | 0.015 | | 0.503 | | | 2.82E-09 | | | | 412181 | |  |
|  |  | rs842359 | C | | G | | -0.084 | | 0.015 | | 0.460 | | | 4.21E-08 | | | | 412181 | |  |
|  |  | rs1990760 | T | | C | | -0.100 | | 0.015 | | 0.585 | | | 4.62E-11 | | | | 412181 | |  |
|  |  | rs13029501 | T | | G | | -0.085 | | 0.015 | | 0.576 | | | 2.81E-08 | | | | 412181 | |  |
|  |  | rs10931828 | T | | C | | -0.098 | | 0.015 | | 0.441 | | | 1.16E-10 | | | | 412181 | |  |
|  |  | rs4676410 | A | | G | | 0.179 | | 0.017 | | 0.271 | | | 3.00E-27 | | | | 412181 | |  |
|  |  | rs10865941 | C | | T | | 0.088 | | 0.015 | | 0.432 | | | 7.11E-09 | | | | 412181 | |  |
|  |  | rs3197999 | A | | G | | 0.172 | | 0.015 | | 0.392 | | | 1.47E-29 | | | | 412181 | |  |
|  |  | rs6762648 | A | | T | | -0.132 | | 0.018 | | 0.244 | | | 1.97E-13 | | | | 412181 | |  |
|  |  | rs116437514 | C | | T | | 0.129 | | 0.019 | | 0.193 | | | 4.43E-12 | | | | 412181 | |  |
|  |  | rs270610 | A | | G | | 0.088 | | 0.015 | | 0.550 | | | 8.17E-09 | | | | 412181 | |  |
|  |  | rs11745587 | A | | G | | -0.128 | | 0.015 | | 0.405 | | | 1.40E-16 | | | | 412181 | |  |
|  |  | rs7704367 | C | | A | | 0.106 | | 0.016 | | 0.290 | | | 1.30E-10 | | | | 412181 | |  |
|  |  | rs2735075 | G | | T | | -0.132 | | 0.015 | | 0.424 | | | 8.71E-18 | | | | 412181 | |  |
|  |  | rs1265108 | T | | C | | -0.088 | | 0.015 | | 0.469 | | | 6.54E-09 | | | | 412181 | |  |
|  |  | rs371674465 | C | | G | | 0.276 | | 0.044 | | 0.025 | | | 4.49E-10 | | | | 412181 | |  |
|  |  | rs3104371 | G | | C | | -0.232 | | 0.020 | | 0.192 | | | 2.85E-31 | | | | 412181 | |  |
|  |  | rs7747343 | A | | G | | -0.084 | | 0.015 | | 0.601 | | | 4.29E-08 | | | | 412181 | |  |
|  |  | rs12536069 | C | | T | | 0.247 | | 0.028 | | 0.068 | | | 9.50E-19 | | | | 412181 | |  |
|  |  | rs10807943 | C | | T | | -0.344 | | 0.028 | | 0.937 | | | 5.51E-34 | | | | 412181 | |  |
|  |  | rs181316459 | C | | G | | 0.616 | | 0.030 | | 0.048 | | | 1.98E-93 | | | | 412181 | |  |
|  |  | rs142695953 | A | | C | | 0.177 | | 0.019 | | 0.186 | | | 4.19E-21 | | | | 412181 | |  |
|  |  | rs11771806 | T | | C | | 0.134 | | 0.023 | | 0.117 | | | 3.57E-09 | | | | 412181 | |  |
|  |  | rs4730276 | A | | G | | -0.097 | | 0.015 | | 0.430 | | | 1.95E-10 | | | | 412181 | |  |
|  |  | rs3778753 | G | | A | | 0.084 | | 0.015 | | 0.441 | | | 2.67E-08 | | | | 412181 | |  |
|  |  | rs2149560 | T | | G | | 0.113 | | 0.015 | | 0.553 | | | 1.28E-13 | | | | 412181 | |  |
|  |  | rs13300483 | T | | C | | 0.102 | | 0.016 | | 0.327 | | | 1.54E-10 | | | | 412181 | |  |
|  |  | rs10761659 | G | | A | | 0.120 | | 0.015 | | 0.528 | | | 2.69E-15 | | | | 412181 | |  |
|  |  | rs10748781 | A | | C | | -0.169 | | 0.016 | | 0.652 | | | 2.10E-27 | | | | 412181 | |  |
|  |  | rs11190368 | T | | A | | 0.100 | | 0.015 | | 0.444 | | | 4.49E-11 | | | | 412181 | |  |
|  |  | rs7931483 | A | | C | | 0.128 | | 0.015 | | 0.417 | | | 3.13E-17 | | | | 412181 | |  |
|  |  | rs11614309 | T | | C | | 0.096 | | 0.016 | | 0.323 | | | 1.46E-09 | | | | 412181 | |  |
|  |  | rs11064881 | A | | G | | 0.144 | | 0.026 | | 0.087 | | | 2.23E-08 | | | | 412181 | |  |
|  |  | rs17587460 | A | | C | | 0.097 | | 0.017 | | 0.243 | | | 2.14E-08 | | | | 412181 | |  |
|  |  | rs34176982 | G | | A | | -0.124 | | 0.022 | | 0.168 | | | 1.12E-08 | | | | 412181 | |  |
|  |  | rs79973254 | A | | T | | -0.176 | | 0.031 | | 0.069 | | | 1.47E-08 | | | | 412181 | |  |
|  |  | rs62029968 | T | | G | | 0.101 | | 0.017 | | 0.256 | | | 3.46E-09 | | | | 412181 | |  |
|  |  | rs16940186 | C | | T | | 0.117 | | 0.021 | | 0.145 | | | 2.12E-08 | | | | 412181 | |  |
|  |  | rs3091316 | A | | G | | -0.088 | | 0.016 | | 0.336 | | | 4.24E-08 | | | | 412181 | |  |
|  |  | rs12942330 | T | | C | | 0.109 | | 0.015 | | 0.529 | | | 6.32E-13 | | | | 412181 | |  |
|  |  | rs3736161 | G | | C | | -0.103 | | 0.016 | | 0.382 | | | 3.61E-11 | | | | 412181 | |  |
|  |  | rs12967678 | A | | G | | 0.127 | | 0.023 | | 0.119 | | | 1.94E-08 | | | | 412181 | |  |
|  |  | rs6017342 | C | | A | | 0.122 | | 0.015 | | 0.559 | | | 1.58E-15 | | | | 412181 | |  |
|  |  | rs3810491 | G | | C | | -0.140 | | 0.019 | | 0.224 | | | 5.56E-14 | | | | 412181 | |  |
|  |  | rs2836878 | A | | G | | -0.136 | | 0.018 | | 0.247 | | | 3.95E-14 | | | | 412181 | |  |
|  |  | rs7285952 | G | | T | | -0.169 | | 0.025 | | 0.117 | | | 6.32E-12 | | | | 412181 | |  |
|  |  | rs9617090 | T | | C | | -0.121 | | 0.016 | | 0.372 | | | 2.02E-14 | | | | 412181 | |  |
|  | UC | rs12736494 | A | | G | | -0.126 | | 0.021 | | 0.265 | | | 3.70E-09 | | | | 411317 | |  |
|  |  | rs4655159 | T | | C | | -0.108 | | 0.019 | | 0.634 | | | 2.16E-08 | | | | 411317 | |  |
|  |  | rs6674040 | T | | G | | -0.182 | | 0.019 | | 0.491 | | | 8.76E-23 | | | | 411317 | |  |
|  |  | rs34145423 | A | | G | | -0.208 | | 0.037 | | 0.077 | | | 1.68E-08 | | | | 411317 | |  |
|  |  | rs148170789 | G | | A | | -0.369 | | 0.049 | | 0.046 | | | 7.05E-14 | | | | 411317 | |  |
|  |  | rs1801274 | G | | A | | -0.163 | | 0.018 | | 0.503 | | | 1.37E-18 | | | | 411317 | |  |
|  |  | rs12132298 | C | | T | | -0.147 | | 0.023 | | 0.213 | | | 3.05E-10 | | | | 411317 | |  |
|  |  | rs3024495 | T | | C | | 0.265 | | 0.024 | | 0.157 | | | 2.37E-28 | | | | 411317 | |  |
|  |  | rs3732179 | C | | T | | 0.111 | | 0.020 | | 0.672 | | | 2.71E-08 | | | | 411317 | |  |
|  |  | rs1990760 | T | | C | | -0.106 | | 0.019 | | 0.585 | | | 1.66E-08 | | | | 411317 | |  |
|  |  | rs10931828 | T | | C | | -0.109 | | 0.019 | | 0.441 | | | 6.10E-09 | | | | 411317 | |  |
|  |  | rs34236350 | T | | C | | 0.183 | | 0.020 | | 0.267 | | | 2.18E-19 | | | | 411317 | |  |
|  |  | rs113595266 | A | | G | | 0.151 | | 0.025 | | 0.151 | | | 1.58E-09 | | | | 411317 | |  |
|  |  | rs3197999 | A | | G | | 0.194 | | 0.019 | | 0.392 | | | 4.51E-25 | | | | 411317 | |  |
|  |  | rs11745587 | A | | G | | -0.128 | | 0.019 | | 0.405 | | | 1.76E-11 | | | | 411317 | |  |
|  |  | rs12518457 | T | | G | | 0.112 | | 0.020 | | 0.288 | | | 3.32E-08 | | | | 411317 | |  |
|  |  | rs1061537 | A | | G | | -0.147 | | 0.019 | | 0.393 | | | 1.39E-14 | | | | 411317 | |  |
|  |  | rs111432088 | T | | C | | 0.487 | | 0.066 | | 0.015 | | | 1.19E-13 | | | | 411317 | |  |
|  |  | rs4587163 | T | | C | | -0.255 | | 0.021 | | 0.283 | | | 2.32E-33 | | | | 411317 | |  |
|  |  | rs1042131 | A | | C | | -0.139 | | 0.019 | | 0.502 | | | 5.45E-14 | | | | 411317 | |  |
|  |  | rs7761376 | G | | T | | -0.105 | | 0.019 | | 0.601 | | | 2.89E-08 | | | | 411317 | |  |
|  |  | rs12536069 | C | | T | | 0.243 | | 0.034 | | 0.068 | | | 1.57E-12 | | | | 411317 | |  |
|  |  | rs10807943 | C | | T | | -0.327 | | 0.035 | | 0.937 | | | 7.49E-21 | | | | 411317 | |  |
|  |  | rs181316459 | C | | G | | 0.604 | | 0.037 | | 0.048 | | | 7.11E-60 | | | | 411317 | |  |
|  |  | rs113827118 | G | | C | | 0.194 | | 0.023 | | 0.186 | | | 3.30E-17 | | | | 411317 | |  |
|  |  | rs4730276 | A | | G | | -0.156 | | 0.019 | | 0.430 | | | 1.27E-16 | | | | 411317 | |  |
|  |  | rs7865719 | G | | A | | 0.111 | | 0.019 | | 0.557 | | | 3.03E-09 | | | | 411317 | |  |
|  |  | rs7101270 | T | | C | | -0.122 | | 0.022 | | 0.247 | | | 2.50E-08 | | | | 411317 | |  |
|  |  | rs10761659 | G | | A | | 0.111 | | 0.019 | | 0.528 | | | 2.29E-09 | | | | 411317 | |  |
|  |  | rs10748781 | A | | C | | -0.169 | | 0.019 | | 0.652 | | | 1.62E-18 | | | | 411317 | |  |
|  |  | rs11190368 | T | | A | | 0.105 | | 0.019 | | 0.444 | | | 2.03E-08 | | | | 411317 | |  |
|  |  | rs7931483 | A | | C | | 0.119 | | 0.019 | | 0.417 | | | 2.01E-10 | | | | 411317 | |  |
|  |  | rs11614178 | A | | G | | 0.122 | | 0.019 | | 0.337 | | | 3.58E-10 | | | | 411317 | |  |
|  |  | rs13379369 | G | | A | | 0.181 | | 0.031 | | 0.875 | | | 9.60E-09 | | | | 411317 | |  |
|  |  | rs34399539 | G | | C | | -0.185 | | 0.029 | | 0.138 | | | 2.69E-10 | | | | 411317 | |  |
|  |  | rs79973254 | A | | T | | -0.213 | | 0.039 | | 0.069 | | | 3.69E-08 | | | | 411317 | |  |
|  |  | rs16940186 | C | | T | | 0.166 | | 0.025 | | 0.145 | | | 5.22E-11 | | | | 411317 | |  |
|  |  | rs12942330 | T | | C | | 0.128 | | 0.019 | | 0.529 | | | 6.17E-12 | | | | 411317 | |  |
|  |  | rs6017342 | C | | A | | 0.156 | | 0.019 | | 0.559 | | | 9.12E-17 | | | | 411317 | |  |
|  |  | rs6089926 | T | | C | | -0.158 | | 0.024 | | 0.208 | | | 2.84E-11 | | | | 411317 | |  |
|  |  | rs2836883 | A | | G | | -0.158 | | 0.022 | | 0.247 | | | 8.48E-13 | | | | 411317 | |  |
|  |  | rs9607629 | G | | A | | -0.197 | | 0.030 | | 0.125 | | | 3.01E-11 | | | | 411317 | |  |
|  |  | rs9617090 | T | | C | | -0.156 | | 0.019 | | 0.372 | | | 8.58E-16 | | | | 411317 | |  |
|  | CD | rs10889669 | G | | T | | 0.235 | | 0.035 | | 0.245 | | | 1.74E-11 | | | | 411973 | |  |
|  |  | rs10473190 | A | | C | | 0.237 | | 0.032 | | 0.568 | | | 1.17E-13 | | | | 411973 | |  |
|  |  | rs3130980 | T | | C | | 0.195 | | 0.032 | | 0.331 | | | 1.80E-09 | | | | 411973 | |  |
|  |  | rs34434863 | G | | T | | -0.257 | | 0.034 | | 0.336 | | | 3.89E-14 | | | | 411973 | |  |
|  |  | rs10807943 | C | | T | | -0.432 | | 0.057 | | 0.937 | | | 3.19E-14 | | | | 411973 | |  |
|  |  | rs181316459 | C | | G | | 0.659 | | 0.061 | | 0.048 | | | 2.55E-27 | | | | 411973 | |  |
|  |  | rs13300483 | T | | C | | 0.183 | | 0.033 | | 0.327 | | | 2.05E-08 | | | | 411973 | |  |
|  |  | rs7856092 | G | | A | | 0.228 | | 0.039 | | 0.768 | | | 4.24E-09 | | | | 411973 | |  |
|  |  | rs76176364 | G | | A | | 0.644 | | 0.092 | | 0.020 | | | 2.34E-12 | | | | 411973 | |  |
|  | Abbreviations: SNPs: single nucleotide polymorphisms; EA: effect allele; OA: other allele; EAF: effect allele frequency; SE: standard error; IBD: inflammatory bowel disease; UC: ulcerative colitis; CD: Crohn's disease. | | | | | | | | | | | | | | | | | | |  |
| **Supplementary Table S4. Summary information for SNPs (P< 1 × 10^⁻6^) that were used as genetic instruments for Mendelian randomization analyses of plasma lipidome.** | | | | | | | | | | | | | | | | | | | | |
| **Plasma lipidome** | | | | **SNPs** | | **Chromosome** | | **Location** | | **EA** | | **OA** | **Beta** | | **SE** | **EAF** | **P** | | **N** | |
| Sterol ester (27:1/20:4) | | | | rs174528 | | 11 | | 61776027 | | C | | T | -0.648 | | 0.015 | 0.414 | 0 | | 7174 | |
|  | | | | rs3741252 | | 11 | | 61744026 | | T | | C | 0.212 | | 0.025 | 0.134 | 6.46E-18 | | 7174 | |
|  | | | | rs7412 | | 19 | | 44908822 | | T | | C | -0.310 | | 0.037 | 0.053 | 4.29E-17 | | 7174 | |
|  | | | | rs147981159 | | 11 | | 62056826 | | A | | G | -0.327 | | 0.042 | 0.043 | 6.76E-15 | | 7174 | |
|  | | | | rs4382917 | | 11 | | 62454004 | | A | | G | 0.146 | | 0.020 | 0.256 | 1.24E-13 | | 7174 | |
|  | | | | rs187429064 | | 19 | | 19269704 | | G | | A | -0.254 | | 0.037 | 0.054 | 1.21E-11 | | 7174 | |
|  | | | | rs2229738 | | 11 | | 68794860 | | T | | C | -0.153 | | 0.023 | 0.156 | 7.01E-11 | | 7174 | |
|  | | | | rs11591147 | | 1 | | 55039974 | | T | | G | -0.297 | | 0.046 | 0.033 | 1.30E-10 | | 7174 | |
|  | | | | rs113248417 | | 11 | | 62059462 | | G | | A | -0.268 | | 0.045 | 0.037 | 2.59E-09 | | 7174 | |
|  | | | | rs1135999 | | 16 | | 15038105 | | G | | A | -0.101 | | 0.018 | 0.315 | 1.23E-08 | | 7174 | |
|  | | | | rs182695896 | | 4 | | 73947510 | | C | | A | 0.296 | | 0.052 | 0.025 | 1.82E-08 | | 7174 | |
|  | | | | rs186913978 | | 2 | | 17147264 | | T | | G | -0.414 | | 0.077 | 0.012 | 8.60E-08 | | 7174 | |
|  | | | | rs6812370 | | 4 | | 174026533 | | A | | G | -0.344 | | 0.066 | 0.017 | 1.55E-07 | | 7174 | |
|  | | | | rs117186386 | | 16 | | 87676514 | | C | | T | 0.178 | | 0.035 | 0.060 | 4.60E-07 | | 7174 | |
|  | | | | rs79136768 | | 11 | | 61594967 | | G | | A | -0.220 | | 0.045 | 0.037 | 9.10E-07 | | 7174 | |
| Sterol ester (27:1/22:6) | | | | rs7412 | | 19 | | 44908822 | | T | | C | -0.269 | | 0.037 | 0.053 | 5.61E-13 | | 7173 | |
|  | | | | rs4072727 | | 18 | | 48039184 | | C | | T | -0.093 | | 0.017 | 0.385 | 7.11E-08 | | 7173 | |
|  | | | | rs10150075 | | 14 | | 72571809 | | T | | C | -0.205 | | 0.038 | 0.053 | 8.96E-08 | | 7173 | |
|  | | | | rs75101588 | | 6 | | 24387133 | | C | | T | 0.259 | | 0.050 | 0.030 | 2.23E-07 | | 7173 | |
|  | | | | rs11591147 | | 1 | | 55039974 | | T | | G | -0.238 | | 0.047 | 0.033 | 3.90E-07 | | 7173 | |
|  | | | | rs77456635 | | 15 | | 64865848 | | A | | G | -0.268 | | 0.054 | 0.024 | 5.68E-07 | | 7173 | |
|  | | | | rs17110517 | | 1 | | 54369167 | | T | | C | -0.175 | | 0.035 | 0.061 | 7.85E-07 | | 7173 | |
| Phosphatidylcholine (20:4_0:0) | | | | rs12226389 | | 11 | | 62056158 | | C | | T | -0.176 | | 0.024 | 0.173 | 2.15E-13 | | 6235 | |
|  | | | | rs7936002 | | 11 | | 62451557 | | G | | A | 0.134 | | 0.021 | 0.257 | 1.65E-10 | | 6235 | |
|  | | | | rs3741252 | | 11 | | 61744026 | | T | | C | 0.166 | | 0.026 | 0.134 | 3.34E-10 | | 6235 | |
|  | | | | rs600626 | | 11 | | 75744264 | | G | | A | -0.121 | | 0.021 | 0.232 | 1.23E-08 | | 6235 | |
|  | | | | rs9967725 | | 2 | | 56382464 | | T | | A | -0.101 | | 0.019 | 0.350 | 6.79E-08 | | 6235 | |
|  | | | | rs182611493 | | 19 | | 19347579 | | G | | A | -0.208 | | 0.040 | 0.054 | 2.78E-07 | | 6235 | |
|  | | | | rs139516883 | | 22 | | 23818551 | | C | | T | -0.353 | | 0.071 | 0.016 | 7.61E-07 | | 6235 | |
| Phosphatidylethanolamine (18:2_0:0) | | | | rs174560 | | 11 | | 61814292 | | C | | T | 0.365 | | 0.017 | 0.383 | 7.02E-98 | | 6905 | |
|  | | | | rs1260326 | | 2 | | 27508073 | | C | | T | -0.115 | | 0.018 | 0.651 | 1.12E-10 | | 6905 | |
|  | | | | rs188404615 | | 12 | | 99719293 | | C | | T | 0.544 | | 0.104 | 0.007 | 1.53E-07 | | 6905 | |
|  | | | | rs1442407 | | 8 | | 3234062 | | T | | G | -0.116 | | 0.022 | 0.179 | 1.60E-07 | | 6905 | |
|  | | | | rs3741259 | | 11 | | 61514878 | | T | | C | 0.139 | | 0.027 | 0.116 | 1.86E-07 | | 6905 | |
|  | | | | rs11045856 | | 12 | | 21197755 | | G | | T | -0.118 | | 0.023 | 0.166 | 2.49E-07 | | 6905 | |
|  | | | | rs1792909 | | 11 | | 62170073 | | A | | G | 0.090 | | 0.017 | 0.387 | 2.60E-07 | | 6905 | |
|  | | | | rs62076866 | | 17 | | 38791988 | | T | | C | 0.388 | | 0.077 | 0.013 | 4.38E-07 | | 6905 | |
|  | | | | rs114899411 | | 4 | | 152031948 | | A | | G | -0.189 | | 0.037 | 0.057 | 5.08E-07 | | 6905 | |
| Phosphatidylcholine (14:0_16:0 | | | | rs9507859 | | 13 | | 27228661 | | T | | C | 0.111 | | 0.022 | 0.765 | 7.13E-07 | | 5497 | |
| Phosphatidylcholine (16:0_20:2) | | | | rs174592 | | 11 | | 61851136 | | G | | A | 0.262 | | 0.017 | 0.418 | 4.96E-54 | | 7135 | |
|  | | | | rs62081852 | | 18 | | 26802732 | | T | | C | 0.389 | | 0.068 | 0.018 | 9.35E-09 | | 7135 | |
|  | | | | rs1532085 | | 15 | | 58391167 | | G | | A | -0.094 | | 0.017 | 0.573 | 2.69E-08 | | 7135 | |
|  | | | | rs1260326 | | 2 | | 27508073 | | C | | T | -0.097 | | 0.018 | 0.651 | 3.00E-08 | | 7135 | |
|  | | | | rs10160784 | | 11 | | 75745010 | | C | | T | -0.110 | | 0.020 | 0.232 | 3.61E-08 | | 7135 | |
|  | | | | rs1800588 | | 15 | | 58431476 | | T | | C | 0.107 | | 0.019 | 0.258 | 3.85E-08 | | 7135 | |
|  | | | | rs150057262 | | 19 | | 19210016 | | G | | C | -0.202 | | 0.039 | 0.052 | 1.77E-07 | | 7135 | |
|  | | | | rs2236514 | | 9 | | 136677616 | | G | | C | -0.091 | | 0.018 | 0.647 | 3.85E-07 | | 7135 | |
|  | | | | rs2317091 | | 11 | | 63193288 | | A | | T | 0.218 | | 0.043 | 0.040 | 4.11E-07 | | 7135 | |
|  | | | | rs1168041 | | 1 | | 62494579 | | C | | T | 0.093 | | 0.019 | 0.733 | 9.29E-07 | | 7135 | |
| Phosphatidylcholine (16:0_20:4) | | | | rs174533 | | 11 | | 61781553 | | A | | G | -0.598 | | 0.016 | 0.409 | 6.29E-295 | | 7174 | |
|  | | | | rs174527 | | 11 | | 61770929 | | C | | G | -0.179 | | 0.017 | 0.444 | 8.38E-26 | | 7174 | |
|  | | | | rs113394924 | | 11 | | 62314821 | | G | | T | 0.214 | | 0.030 | 0.087 | 6.09E-13 | | 7174 | |
|  | | | | rs2943813 | | 11 | | 61490996 | | C | | T | -0.139 | | 0.020 | 0.783 | 9.96E-12 | | 7174 | |
|  | | | | rs187429064 | | 19 | | 19269704 | | G | | A | -0.226 | | 0.038 | 0.054 | 2.04E-09 | | 7174 | |
|  | | | | rs3018617 | | 11 | | 62435462 | | G | | A | -0.108 | | 0.019 | 0.302 | 8.62E-09 | | 7174 | |
|  | | | | rs147549994 | | 16 | | 1939264 | | T | | G | 0.246 | | 0.045 | 0.036 | 5.51E-08 | | 7174 | |
|  | | | | rs499974 | | 11 | | 75743976 | | A | | C | -0.103 | | 0.020 | 0.232 | 2.26E-07 | | 7174 | |
| Phosphatidylcholine (16:1_18:1) | | | | rs174560 | | 11 | | 61814292 | | C | | T | 0.176 | | 0.017 | 0.383 | 2.28E-24 | | 7166 | |
|  | | | | rs603424 | | 10 | | 100315722 | | A | | G | -0.176 | | 0.026 | 0.121 | 1.21E-11 | | 7166 | |
|  | | | | rs1260326 | | 2 | | 27508073 | | C | | T | -0.110 | | 0.017 | 0.651 | 2.95E-10 | | 7166 | |
|  | | | | rs146934967 | | 18 | | 15033501 | | T | | C | 0.415 | | 0.074 | 0.013 | 1.83E-08 | | 7166 | |
|  | | | | rs1532085 | | 15 | | 58391167 | | G | | A | -0.093 | | 0.017 | 0.573 | 3.79E-08 | | 7166 | |
|  | | | | rs56156922 | | 16 | | 56953457 | | C | | T | 0.101 | | 0.019 | 0.279 | 5.95E-08 | | 7166 | |
|  | | | | rs4447106 | | 10 | | 100600003 | | A | | G | 0.128 | | 0.024 | 0.141 | 1.29E-07 | | 7166 | |
|  | | | | rs8033940 | | 15 | | 58432643 | | A | | G | 0.094 | | 0.018 | 0.337 | 2.02E-07 | | 7166 | |
|  | | | | rs8100204 | | 19 | | 19282905 | | A | | G | -0.114 | | 0.022 | 0.171 | 4.12E-07 | | 7166 | |
| Phosphatidylcholine (17:0_18:1) | | | | rs7350789 | | 15 | | 58387469 | | A | | G | 0.113 | | 0.017 | 0.393 | 4.46E-11 | | 7073 | |
|  | | | | rs78308012 | | 9 | | 137586795 | | G | | T | 0.153 | | 0.029 | 0.103 | 1.11E-07 | | 7073 | |
|  | | | | rs2126263 | | 8 | | 9324101 | | A | | G | 0.123 | | 0.024 | 0.850 | 1.95E-07 | | 7073 | |
|  | | | | rs1106933 | | 6 | | 166616136 | | G | | A | 0.125 | | 0.024 | 0.136 | 3.44E-07 | | 7073 | |
|  | | | | rs17231506 | | 16 | | 56960616 | | T | | C | 0.094 | | 0.019 | 0.278 | 5.82E-07 | | 7073 | |
| Phosphatidylcholine (17:0_18:2) | | | | rs174566 | | 11 | | 61824890 | | G | | A | 0.259 | | 0.017 | 0.408 | 9.03E-53 | | 7170 | |
|  | | | | rs964184 | | 11 | | 116778201 | | C | | G | -0.154 | | 0.023 | 0.849 | 2.76E-11 | | 7170 | |
|  | | | | rs1800588 | | 15 | | 58431476 | | T | | C | 0.115 | | 0.019 | 0.258 | 3.31E-09 | | 7170 | |
|  | | | | rs17231506 | | 16 | | 56960616 | | T | | C | 0.097 | | 0.019 | 0.278 | 1.89E-07 | | 7170 | |
|  | | | | rs422137 | | 15 | | 58416983 | | A | | G | 0.085 | | 0.017 | 0.493 | 3.66E-07 | | 7170 | |
|  | | | | rs57518551 | | 2 | | 42156698 | | C | | T | -0.125 | | 0.025 | 0.132 | 3.79E-07 | | 7170 | |
|  | | | | rs6498540 | | 16 | | 15036737 | | G | | A | 0.090 | | 0.018 | 0.334 | 3.81E-07 | | 7170 | |
|  | | | | rs78592970 | | 19 | | 44938026 | | T | | G | -0.128 | | 0.026 | 0.127 | 5.18E-07 | | 7170 | |
| Phosphatidylcholine (18:0_20:4) | | | | rs174528 | | 11 | | 61776027 | | C | | T | -0.666 | | 0.015 | 0.414 | 0 | | 7174 | |
|  | | | | rs3741252 | | 11 | | 61744026 | | T | | C | 0.243 | | 0.024 | 0.134 | 5.54E-23 | | 7174 | |
|  | | | | rs147981159 | | 11 | | 62056826 | | A | | G | -0.346 | | 0.042 | 0.043 | 1.54E-16 | | 7174 | |
|  | | | | rs3019200 | | 11 | | 61481911 | | A | | C | -0.163 | | 0.021 | 0.803 | 2.57E-14 | | 7174 | |
|  | | | | rs4382917 | | 11 | | 62454004 | | A | | G | 0.149 | | 0.020 | 0.256 | 4.68E-14 | | 7174 | |
|  | | | | rs2797620 | | 1 | | 94975736 | | T | | C | -0.101 | | 0.017 | 0.333 | 6.11E-09 | | 7174 | |
|  | | | | rs147549994 | | 16 | | 1939264 | | T | | G | 0.256 | | 0.045 | 0.036 | 1.35E-08 | | 7174 | |
|  | | | | rs79136768 | | 11 | | 61594967 | | G | | A | -0.249 | | 0.045 | 0.037 | 2.71E-08 | | 7174 | |
|  | | | | rs72789541 | | 16 | | 15033677 | | A | | T | -0.097 | | 0.018 | 0.315 | 6.36E-08 | | 7174 | |
|  | | | | rs187429064 | | 19 | | 19269704 | | G | | A | -0.202 | | 0.037 | 0.054 | 7.42E-08 | | 7174 | |
|  | | | | rs1219539 | | 11 | | 75722099 | | A | | G | -0.088 | | 0.017 | 0.493 | 1.74E-07 | | 7174 | |
|  | | | | rs600518 | | 11 | | 75744328 | | T | | A | -0.101 | | 0.020 | 0.232 | 3.49E-07 | | 7174 | |
|  | | | | rs12275418 | | 11 | | 61012351 | | A | | G | 0.124 | | 0.025 | 0.134 | 4.66E-07 | | 7174 | |
|  | | | | rs1477491 | | 18 | | 73092300 | | G | | A | -0.102 | | 0.020 | 0.789 | 5.06E-07 | | 7174 | |
|  | | | | rs2070895 | | 15 | | 58431740 | | A | | G | 0.094 | | 0.019 | 0.267 | 8.73E-07 | | 7174 | |
| Phosphatidylcholine (18:1_20:4) | | | | rs174537 | | 11 | | 61785208 | | T | | G | -0.508 | | 0.016 | 0.408 | 5.48E-207 | | 7173 | |
|  | | | | rs174527 | | 11 | | 61770929 | | C | | G | -0.160 | | 0.017 | 0.444 | 5.01E-21 | | 7173 | |
|  | | | | rs187429064 | | 19 | | 19269704 | | G | | A | -0.276 | | 0.038 | 0.054 | 2.68E-13 | | 7173 | |
|  | | | | rs2943813 | | 11 | | 61490996 | | C | | T | -0.130 | | 0.020 | 0.783 | 2.26E-10 | | 7173 | |
|  | | | | rs113394924 | | 11 | | 62314821 | | G | | T | 0.187 | | 0.030 | 0.087 | 3.52E-10 | | 7173 | |
|  | | | | rs531117 | | 11 | | 75745089 | | T | | C | -0.114 | | 0.020 | 0.232 | 9.96E-09 | | 7173 | |
|  | | | | rs8107974 | | 19 | | 19277691 | | T | | A | -0.191 | | 0.034 | 0.062 | 3.00E-08 | | 7173 | |
|  | | | | rs147797702 | | 18 | | 52743009 | | C | | T | -0.489 | | 0.089 | 0.009 | 4.03E-08 | | 7173 | |
|  | | | | rs61978272 | | 14 | | 94523321 | | T | | C | -0.098 | | 0.019 | 0.282 | 1.41E-07 | | 7173 | |
|  | | | | rs1077834 | | 15 | | 58431280 | | C | | T | 0.099 | | 0.019 | 0.266 | 2.88E-07 | | 7173 | |
|  | | | | rs147549994 | | 16 | | 1939264 | | T | | G | 0.232 | | 0.045 | 0.036 | 3.16E-07 | | 7173 | |
|  | | | | rs641738 | | 19 | | 54173068 | | C | | T | -0.085 | | 0.017 | 0.593 | 5.94E-07 | | 7173 | |
|  | | | | rs79113397 | | 2 | | 101570369 | | C | | T | 0.100 | | 0.020 | 0.229 | 7.59E-07 | | 7173 | |
| Phosphatidylcholine (O-16:1_20:4) | | | | rs174535 | | 11 | | 61783884 | | C | | T | -0.404 | | 0.018 | 0.409 | 1.18E-107 | | 6025 | |
|  | | | | rs1077989 | | 14 | | 67509105 | | C | | A | -0.192 | | 0.018 | 0.468 | 3.43E-26 | | 6025 | |
|  | | | | rs174527 | | 11 | | 61770929 | | C | | G | -0.121 | | 0.019 | 0.444 | 7.84E-11 | | 6025 | |
|  | | | | rs2043085 | | 15 | | 58388755 | | C | | T | -0.100 | | 0.018 | 0.576 | 4.75E-08 | | 6025 | |
|  | | | | rs12920974 | | 16 | | 56959113 | | T | | G | -0.104 | | 0.020 | 0.330 | 1.16E-07 | | 6025 | |
|  | | | | rs114325590 | | 1 | | 107442367 | | T | | C | -0.458 | | 0.091 | 0.011 | 4.70E-07 | | 6025 | |
|  | | | | rs2015950 | | 11 | | 62058058 | | A | | G | -0.134 | | 0.027 | 0.137 | 5.32E-07 | | 6025 | |
| Phosphatidylcholine (O-18:1_18:2) | | | | rs174551 | | 11 | | 61806212 | | C | | T | 0.229 | | 0.017 | 0.406 | 3.59E-39 | | 6718 | |
|  | | | | rs11918522 | | 3 | | 112816210 | | A | | G | 0.127 | | 0.024 | 0.159 | 7.40E-08 | | 6718 | |
|  | | | | rs1601934 | | 15 | | 58379522 | | A | | G | -0.097 | | 0.018 | 0.645 | 8.40E-08 | | 6718 | |
|  | | | | rs606587 | | 1 | | 230180443 | | G | | A | -0.091 | | 0.018 | 0.374 | 3.41E-07 | | 6718 | |
|  | | | | rs17231506 | | 16 | | 56960616 | | T | | C | 0.095 | | 0.019 | 0.278 | 6.14E-07 | | 6718 | |
|  | | | | rs2537611 | | 7 | | 17693175 | | T | | C | 0.087 | | 0.018 | 0.372 | 9.77E-07 | | 6718 | |
| Phosphatidylethanolamine (18:0_18:2) | | | | rs174567 | | 11 | | 61825533 | | G | | A | 0.343 | | 0.017 | 0.409 | 1.25E-92 | | 7128 | |
|  | | | | rs10468017 | | 15 | | 58386313 | | T | | C | 0.298 | | 0.017 | 0.338 | 9.03E-66 | | 7128 | |
|  | | | | rs633695 | | 15 | | 58433640 | | G | | A | 0.255 | | 0.018 | 0.314 | 5.56E-45 | | 7128 | |
|  | | | | rs117376818 | | 15 | | 58506762 | | A | | G | 0.630 | | 0.070 | 0.016 | 4.16E-19 | | 7128 | |
|  | | | | rs73424597 | | 15 | | 58607410 | | C | | T | 0.355 | | 0.045 | 0.035 | 5.88E-15 | | 7128 | |
|  | | | | rs1260326 | | 2 | | 27508073 | | C | | T | -0.122 | | 0.017 | 0.651 | 2.57E-12 | | 7128 | |
|  | | | | rs964184 | | 11 | | 116778201 | | C | | G | -0.154 | | 0.023 | 0.849 | 2.75E-11 | | 7128 | |
|  | | | | rs138283783 | | 15 | | 58244328 | | C | | T | 0.352 | | 0.054 | 0.027 | 5.56E-11 | | 7128 | |
|  | | | | rs117900629 | | 11 | | 62205328 | | A | | G | -0.213 | | 0.034 | 0.062 | 6.25E-10 | | 7128 | |
|  | | | | rs12928099 | | 16 | | 15056648 | | A | | C | -0.113 | | 0.019 | 0.274 | 1.50E-09 | | 7128 | |
|  | | | | rs174527 | | 11 | | 61770929 | | C | | G | 0.102 | | 0.017 | 0.444 | 2.73E-09 | | 7128 | |
|  | | | | rs2946493 | | 8 | | 12966162 | | C | | T | 0.105 | | 0.021 | 0.206 | 4.35E-07 | | 7128 | |
|  | | | | rs10069895 | | 5 | | 86297284 | | C | | T | 0.097 | | 0.020 | 0.764 | 7.18E-07 | | 7128 | |
| Phosphatidylethanolamine (O-18:1_20:4) | | | | rs174536 | | 11 | | 61784455 | | C | | A | -0.251 | | 0.017 | 0.408 | 1.09E-49 | | 7172 | |
|  | | | | rs204468 | | 19 | | 44987378 | | C | | T | -0.095 | | 0.017 | 0.595 | 2.35E-08 | | 7172 | |
|  | | | | rs2277289 | | 11 | | 118311945 | | C | | T | 0.091 | | 0.018 | 0.326 | 3.31E-07 | | 7172 | |
|  | | | | rs11062287 | | 12 | | 2630538 | | A | | G | 0.255 | | 0.050 | 0.034 | 4.45E-07 | | 7172 | |
|  | | | | rs72635955 | | 1 | | 244982806 | | T | | C | 0.126 | | 0.025 | 0.125 | 5.16E-07 | | 7172 | |
|  | | | | rs141622900 | | 19 | | 44923535 | | A | | G | 0.250 | | 0.050 | 0.030 | 6.52E-07 | | 7172 | |
|  | | | | rs71420341 | | 2 | | 59638953 | | G | | A | -0.146 | | 0.030 | 0.089 | 7.54E-07 | | 7172 | |
|  | | | | rs6601694 | | 8 | | 8390548 | | A | | G | 0.088 | | 0.018 | 0.334 | 8.85E-07 | | 7172 | |
| Sphingomyelin (d36:1) | | | | rs2336171 | | 19 | | 8209156 | | C | | G | 0.195 | | 0.017 | 0.399 | 2.94E-30 | | 7174 | |
|  | | | | rs7248003 | | 19 | | 8236164 | | T | | C | 0.145 | | 0.019 | 0.721 | 2.69E-14 | | 7174 | |
|  | | | | rs182695896 | | 4 | | 73947510 | | C | | A | 0.339 | | 0.053 | 0.025 | 1.58E-10 | | 7174 | |
|  | | | | rs364585 | | 20 | | 12982070 | | G | | A | -0.109 | | 0.018 | 0.661 | 5.55E-10 | | 7174 | |
|  | | | | rs75679663 | | 17 | | 4764677 | | A | | C | -0.325 | | 0.054 | 0.026 | 1.42E-09 | | 7174 | |
|  | | | | rs112585713 | | 4 | | 72876701 | | A | | G | 0.282 | | 0.048 | 0.032 | 4.41E-09 | | 7174 | |
|  | | | | rs79146711 | | 4 | | 73424911 | | T | | C | 0.234 | | 0.041 | 0.045 | 1.02E-08 | | 7174 | |
|  | | | | rs12462619 | | 19 | | 8607097 | | C | | T | -0.108 | | 0.019 | 0.261 | 2.32E-08 | | 7174 | |
|  | | | | rs11591147 | | 1 | | 55039974 | | T | | G | -0.259 | | 0.047 | 0.033 | 2.96E-08 | | 7174 | |
|  | | | | rs7157785 | | 14 | | 63768838 | | T | | G | 0.136 | | 0.025 | 0.125 | 6.56E-08 | | 7174 | |
|  | | | | rs4809845 | | 20 | | 51460945 | | C | | T | 0.129 | | 0.025 | 0.134 | 2.41E-07 | | 7174 | |
|  | | | | rs6558122 | | 8 | | 29518250 | | C | | T | -0.140 | | 0.028 | 0.101 | 4.58E-07 | | 7174 | |
|  | | | | rs117643293 | | 17 | | 5065669 | | T | | C | -0.171 | | 0.034 | 0.062 | 5.50E-07 | | 7174 | |
|  | | | | rs186039163 | | 17 | | 3547599 | | G | | T | -0.416 | | 0.084 | 0.010 | 7.74E-07 | | 7174 | |
|  | | | | rs1169306 | | 12 | | 121000508 | | T | | C | -0.085 | | 0.017 | 0.360 | 9.27E-07 | | 7174 | |
| Sphingomyelin (d38:1) | | | | rs7157785 | | 14 | | 63768838 | | T | | G | 0.370 | | 0.025 | 0.125 | 1.11E-49 | | 7174 | |
|  | | | | rs7258249 | | 19 | | 8206837 | | A | | G | 0.217 | | 0.017 | 0.399 | 2.81E-37 | | 7174 | |
|  | | | | rs364585 | | 20 | | 12982070 | | G | | A | -0.164 | | 0.018 | 0.661 | 7.39E-21 | | 7174 | |
|  | | | | rs75679663 | | 17 | | 4764677 | | A | | C | -0.474 | | 0.053 | 0.026 | 9.06E-19 | | 7174 | |
|  | | | | rs117643293 | | 17 | | 5065669 | | T | | C | -0.243 | | 0.034 | 0.062 | 8.25E-13 | | 7174 | |
|  | | | | rs182695896 | | 4 | | 73947510 | | C | | A | 0.371 | | 0.053 | 0.025 | 2.53E-12 | | 7174 | |
|  | | | | rs11591147 | | 1 | | 55039974 | | T | | G | -0.301 | | 0.046 | 0.033 | 1.07E-10 | | 7174 | |
|  | | | | rs114483871 | | 4 | | 73124451 | | T | | C | 0.268 | | 0.045 | 0.038 | 2.19E-09 | | 7174 | |
|  | | | | rs7248003 | | 19 | | 8236164 | | T | | C | 0.109 | | 0.019 | 0.721 | 9.84E-09 | | 7174 | |
|  | | | | rs77645768 | | 4 | | 72803111 | | A | | G | 0.304 | | 0.054 | 0.026 | 1.53E-08 | | 7174 | |
|  | | | | rs374071816 | | 6 | | 160682773 | | A | | T | 0.190 | | 0.036 | 0.057 | 1.62E-07 | | 7174 | |
|  | | | | rs3741111 | | 11 | | 124076588 | | G | | A | 0.086 | | 0.017 | 0.483 | 2.19E-07 | | 7174 | |
|  | | | | rs12462619 | | 19 | | 8607097 | | C | | T | -0.096 | | 0.019 | 0.261 | 6.04E-07 | | 7174 | |
|  | | | | rs7247433 | | 19 | | 19684751 | | A | | C | -0.085 | | 0.017 | 0.394 | 9.72E-07 | | 7174 | |
| Sphingomyelin (d42:2) | | | | rs75679663 | | 17 | | 4764677 | | A | | C | -0.538 | | 0.053 | 0.026 | 1.09E-23 | | 7174 | |
|  | | | | rs118068660 | | 19 | | 11079868 | | T | | C | -0.219 | | 0.028 | 0.095 | 6.77E-15 | | 7174 | |
|  | | | | rs11591147 | | 1 | | 55039974 | | T | | G | -0.362 | | 0.047 | 0.033 | 8.29E-15 | | 7174 | |
|  | | | | rs182695896 | | 4 | | 73947510 | | C | | A | 0.382 | | 0.053 | 0.025 | 5.36E-13 | | 7174 | |
|  | | | | rs146331166 | | 17 | | 5464230 | | C | | T | -0.219 | | 0.035 | 0.060 | 2.56E-10 | | 7174 | |
|  | | | | rs114483871 | | 4 | | 73124451 | | T | | C | 0.279 | | 0.045 | 0.038 | 4.78E-10 | | 7174 | |
|  | | | | rs112585713 | | 4 | | 72876701 | | A | | G | 0.288 | | 0.048 | 0.032 | 1.86E-09 | | 7174 | |
|  | | | | rs7412 | | 19 | | 44908822 | | T | | C | -0.211 | | 0.037 | 0.053 | 1.45E-08 | | 7174 | |
|  | | | | rs17145750 | | 7 | | 73612048 | | T | | C | 0.132 | | 0.024 | 0.141 | 3.07E-08 | | 7174 | |
|  | | | | rs1367117 | | 2 | | 21041028 | | A | | G | 0.100 | | 0.018 | 0.285 | 6.88E-08 | | 7174 | |
|  | | | | rs3912622 | | 11 | | 103250744 | | C | | A | -0.109 | | 0.021 | 0.200 | 2.00E-07 | | 7174 | |
|  | | | | rs17261772 | | 2 | | 135153852 | | C | | T | 0.090 | | 0.018 | 0.326 | 3.94E-07 | | 7174 | |
|  | | | | rs116893213 | | 11 | | 101739572 | | C | | G | 0.227 | | 0.045 | 0.037 | 4.17E-07 | | 7174 | |
|  | | | | rs6843498 | | 4 | |  | | G | | T | 0.095 | | 0.019 | 0.312 | 5.00E-07 | | 7174 | |
| Abbreviations: SNPs: single nucleotide polymorphisms; EA: effect allele; OA: other allele; EAF: effect allele frequency; SE: standard error. | | | | | | | | | | | | | | | | | | | | |

| **Supplementary Table S5. Summary information for SNPs (P< 1 × 10^⁻6^) that were used as genetic instruments for Mendelian randomization analyses of circulating inflammatory proteins.** | | | | | | | | | | |
| --- | --- | --- | --- | --- | --- | --- | --- | --- | --- | --- |
| **Inflammatory proteins** | **SNPs** | **Chromosome** | **Location** | **EA** | **OA** | **Beta** | **SE** | **EAF** | **P** | **N** |
| Axin-1 | rs2470622 | 8 | 102489704 | A | G | -0.123 | 0.024 | 0.913 | 3.80E-07 | 11345 |
|  | rs148317708 | 18 | 50513818 | A | G | -0.212 | 0.042 | 0.030 | 4.47E-07 | 10919 |
|  | rs2691530 | 7 | 77960243 | T | C | -0.067 | 0.013 | 0.597 | 4.71E-07 | 11793 |
| CCL19 | rs9469127 | 6 | 32444093 | C | G | -0.488 | 0.028 | 0.937 | 1.41E-69 | 14735 |
|  | rs62292952 | 3 | 132200719 | T | G | 0.235 | 0.019 | 0.882 | 7.53E-37 | 14728 |
|  | rs28635588 | 6 | 32519041 | A | G | -0.178 | 0.021 | 0.841 | 6.90E-18 | 9182 |
|  | rs11574915 | 9 | 34710084 | A | C | -0.146 | 0.017 | 0.864 | 7.19E-18 | 14733 |
|  | rs13010492 | 2 | 204776176 | C | G | -0.080 | 0.012 | 0.475 | 6.65E-11 | 13422 |
|  | rs4766578 | 12 | 111904371 | A | T | -0.080 | 0.013 | 0.510 | 1.29E-09 | 11785 |
|  | rs6486995 | 12 | 19813981 | A | T | -0.070 | 0.014 | 0.245 | 2.43E-07 | 14735 |
|  | rs6870560 | 5 | 174187919 | T | C | 0.086 | 0.017 | 0.856 | 3.28E-07 | 14732 |
|  | rs4907572 | 13 | 113647321 | A | G | -0.067 | 0.014 | 0.265 | 9.69E-07 | 14722 |
| CCL4 | rs113010081 | 3 | 46457412 | T | C | -0.577 | 0.018 | 0.876 | 1.24E-215 | 14296 |
|  | rs8064426 | 17 | 34819750 | A | G | -0.410 | 0.017 | 0.146 | 2.11E-124 | 14295 |
|  | rs17692864 | 17 | 34945214 | T | C | 0.232 | 0.028 | 0.941 | 2.38E-16 | 13662 |
|  | rs140505982 | 3 | 46459749 | T | C | -0.229 | 0.042 | 0.025 | 3.24E-08 | 13865 |
|  | rs1634503 | 17 | 34421139 | A | G | -0.075 | 0.014 | 0.242 | 6.72E-08 | 14295 |
|  | rs5743614 | 4 | 38798935 | T | C | 0.073 | 0.014 | 0.257 | 3.56E-07 | 12859 |
|  | rs1161092 | 12 | 67722012 | A | G | -0.064 | 0.013 | 0.674 | 3.77E-07 | 14296 |
|  | rs137896358 | 19 | 18917908 | T | C | 0.280 | 0.055 | 0.019 | 3.85E-07 | 12745 |
| CD5 | rs3184504 | 12 | 111884608 | T | C | 0.143 | 0.013 | 0.476 | 7.29E-29 | 11784 |
|  | rs674379 | 11 | 60922561 | C | G | 0.119 | 0.013 | 0.640 | 2.85E-21 | 12835 |
|  | rs4939490 | 11 | 60793651 | C | G | 0.108 | 0.012 | 0.633 | 1.80E-19 | 14735 |
|  | rs7242462 | 18 | 45545720 | A | G | -0.120 | 0.019 | 0.885 | 1.44E-10 | 14287 |
|  | rs3104373 | 6 | 32600375 | T | C | 0.097 | 0.017 | 0.147 | 3.70E-09 | 14735 |
|  | rs61747728 | 1 | 179526214 | T | C | 0.184 | 0.031 | 0.040 | 4.06E-09 | 14287 |
|  | rs138861398 | 4 | 77211819 | A | C | -0.080 | 0.015 | 0.232 | 1.05E-07 | 11967 |
|  | rs4073745 | 5 | 176733341 | A | G | -0.068 | 0.013 | 0.700 | 1.85E-07 | 14287 |
|  | rs367364 | 6 | 32019946 | T | C | -0.083 | 0.016 | 0.154 | 2.28E-07 | 14730 |
|  | rs192582121 | 9 | 105614669 | A | T | -0.417 | 0.081 | 0.992 | 2.31E-07 | 11438 |
|  | rs2237450 | 7 | 50716017 | T | C | 0.062 | 0.012 | 0.639 | 5.06E-07 | 14287 |
|  | rs117516652 | 13 | 39003323 | A | G | 0.237 | 0.048 | 0.983 | 6.12E-07 | 14248 |
|  | rs117276991 | 10 | 6251691 | A | G | -0.126 | 0.026 | 0.075 | 8.40E-07 | 14287 |
|  | rs17769062 | 9 | 27496604 | T | C | 0.164 | 0.033 | 0.959 | 9.38E-07 | 14287 |
| CD6 | rs11230555 | 11 | 60763134 | A | C | -0.603 | 0.013 | 0.222 | 0 | 14287 |
|  | rs2237995 | 11 | 60749256 | A | G | -0.363 | 0.022 | 0.078 | 2.35E-62 | 14725 |
|  | rs597808 | 12 | 111973358 | A | G | 0.116 | 0.013 | 0.478 | 4.86E-18 | 11335 |
|  | rs143374617 | 1 | 4327524 | T | C | 0.177 | 0.033 | 0.040 | 7.97E-08 | 14733 |
|  | rs74886615 | 4 | 83099601 | A | T | 0.069 | 0.013 | 0.615 | 9.64E-08 | 12404 |
|  | rs10792322 | 11 | 61761552 | T | C | -0.066 | 0.013 | 0.300 | 1.49E-07 | 14734 |
|  | rs60988380 | 9 | 33117954 | T | C | 0.096 | 0.019 | 0.106 | 7.49E-07 | 14735 |
| FGF-5 | rs1458038 | 4 | 81164723 | T | C | 0.516 | 0.013 | 0.317 | 0 | 11788 |
|  | rs3796605 | 4 | 81192363 | A | G | 0.163 | 0.028 | 0.934 | 8.60E-09 | 11782 |
|  | rs117253850 | 9 | 7476426 | T | C | 0.227 | 0.045 | 0.022 | 5.67E-07 | 11781 |
| IL-10α | rs3135932 | 11 | 117864063 | A | G | -0.084 | 0.017 | 0.817 | 5.07E-07 | 11793 |
|  | rs6670799 | 1 | 56566352 | A | G | -0.125 | 0.025 | 0.084 | 7.48E-07 | 11345 |
| TNFSF12 | rs34790908 | 17 | 7451110 | T | C | 0.211 | 0.013 | 0.256 | 3.90E-60 | 14732 |
|  | rs13107325 | 4 | 103188709 | T | C | 0.203 | 0.024 | 0.070 | 8.77E-18 | 14733 |
|  | rs9842051 | 3 | 143021856 | C | G | 0.119 | 0.014 | 0.748 | 1.68E-17 | 14288 |
|  | rs579459 | 9 | 136154168 | T | C | 0.110 | 0.016 | 0.789 | 2.68E-12 | 11785 |
|  | rs73133996 | 3 | 98429219 | C | G | 0.081 | 0.012 | 0.456 | 1.32E-11 | 14288 |
|  | rs7503751 | 17 | 7789542 | A | G | -0.139 | 0.024 | 0.078 | 5.76E-09 | 14288 |
|  | rs201375822 | 10 | 17895807 | T | C | 0.108 | 0.019 | 0.262 | 2.07E-08 | 10692 |
|  | rs10831650 | 11 | 11644391 | A | G | 0.069 | 0.013 | 0.356 | 3.09E-08 | 14288 |
|  | rs12145644 | 1 | 162517994 | A | T | -0.073 | 0.014 | 0.248 | 2.52E-07 | 14287 |
|  | rs2163333 | 2 | 53077754 | A | G | 0.140 | 0.027 | 0.952 | 2.71E-07 | 14719 |
|  | rs76133364 | 19 | 19706214 | A | C | 0.190 | 0.037 | 0.032 | 2.77E-07 | 13224 |
|  | rs4882710 | 12 | 129101566 | T | C | 0.067 | 0.013 | 0.346 | 3.70E-07 | 14288 |
|  | rs201620545 | 2 | 99371601 | A | G | -0.085 | 0.017 | 0.818 | 4.34E-07 | 11327 |
|  | rs11136339 | 8 | 145013345 | T | C | 0.061 | 0.012 | 0.388 | 5.05E-07 | 13664 |
|  | rs117550445 | 12 | 82487578 | C | G | -0.186 | 0.038 | 0.971 | 8.64E-07 | 14721 |
| Abbreviations: SNPs: single nucleotide polymorphisms; EA: effect allele; OA: other allele; EAF: effect allele frequency; SE: standard error. | | | | | | | | | | |

| **Supplementary Table S6. MR analysis results of associations between lipidome and IBD after the initial screening.** | | | | | | | | | | | | | |
| --- | --- | --- | --- | --- | --- | --- | --- | --- | --- | --- | --- | --- | --- |
| **Plasma lipidome** | **Method** | **Nsnp** | **OR** | **OR_lci95** | **OR_uci95** | **P** | **Heterogeneity** | | | | **Pleiotropy** | | |
|  |  |  |  |  |  |  | **MR Egger** | | **IVW** | | **MR-Egger intercep** | | **MR-PRESO** |
|  |  |  |  |  |  |  | **Q** | **P** | **Q** | **P** | **intercept** | **P** | **P** |
| **IBD** |  |  |  |  |  |  |  |  |  |  |  |  |  |
| Sterol ester (27:1/20:3) | MR Egger | 30 | 0.917 | 0.769 | 1.094 | 0.346 | 56.466 | 0.001 | 56.480 | 0.002 | -0.001 | 0.935 | 0.009 |
|  | Weighted median | 30 | 0.906 | 0.824 | 0.997 | 0.043 |  |  |  |  |  |  |  |
|  | IVW | 30 | 0.911 | 0.841 | 0.987 | 0.023 |  |  |  |  |  |  |  |
|  | Simple mode | 30 | 0.919 | 0.776 | 1.088 | 0.333 |  |  |  |  |  |  |  |
|  | Weighted mode | 30 | 0.916 | 0.803 | 1.044 | 0.198 |  |  |  |  |  |  |  |
|  | BWMR | 30 | 0.921 | 0.844 | 1.005 | 0.064 |  |  |  |  |  |  |  |
| Sterol ester (27:1/20:4) | MR Egger | 31 | 0.879 | 0.807 | 0.956 | 0.006 | 72.697 | 0.000 | 75.083 | 0.000 | 0.010 | 0.337 | 0.001 |
|  | Weighted median | 31 | 0.896 | 0.856 | 0.938 | 0.000 |  |  |  |  |  |  |  |
|  | IVW | 31 | 0.906 | 0.856 | 0.960 | 0.001 |  |  |  |  |  |  |  |
|  | Simple mode | 31 | 0.916 | 0.797 | 1.053 | 0.226 |  |  |  |  |  |  |  |
|  | Weighted mode | 31 | 0.899 | 0.861 | 0.940 | 0.000 |  |  |  |  |  |  |  |
|  | BWMR | 31 | 0.909 | 0.853 | 0.969 | 0.003 |  |  |  |  |  |  |  |
| Sterol ester (27:1/20:5) | MR Egger | 26 | 0.812 | 0.721 | 0.915 | 0.002 | 39.450 | 0.024 | 47.795 | 0.004 | 0.020 | 0.034 | 0.016 |
|  | Weighted median | 26 | 0.859 | 0.799 | 0.923 | 0.000 |  |  |  |  |  |  |  |
|  | IVW | 26 | 0.910 | 0.847 | 0.978 | 0.010 |  |  |  |  |  |  |  |
|  | Simple mode | 26 | 1.096 | 0.877 | 1.370 | 0.426 |  |  |  |  |  |  |  |
|  | Weighted mode | 26 | 0.842 | 0.787 | 0.902 | 0.000 |  |  |  |  |  |  |  |
|  | BWMR | 26 | 0.916 | 0.851 | 0.987 | 0.020 |  |  |  |  |  |  |  |
| Sterol ester (27:1/22:6) | MR Egger | 23 | 0.960 | 0.798 | 1.155 | 0.672 | 28.488 | 0.127 | 29.629 | 0.128 | -0.013 | 0.370 | 0.088 |
|  | Weighted median | 23 | 0.963 | 0.866 | 1.071 | 0.486 |  |  |  |  |  |  |  |
|  | IVW | 23 | 0.888 | 0.821 | 0.960 | 0.003 |  |  |  |  |  |  |  |
|  | Simple mode | 23 | 0.990 | 0.813 | 1.206 | 0.922 |  |  |  |  |  |  |  |
|  | Weighted mode | 23 | 0.994 | 0.732 | 1.351 | 0.971 |  |  |  |  |  |  |  |
|  | BWMR | 23 | 0.911 | 0.836 | 0.992 | 0.033 |  |  |  |  |  |  |  |
| Phosphatidylcholine (20:4_0:0) | MR Egger | 27 | 0.860 | 0.803 | 0.920 | 0.000 | 22.955 | 0.580 | 25.557 | 0.488 | 0.011 | 0.119 | 0.484 |
|  | Weighted median | 27 | 0.882 | 0.836 | 0.930 | 0.000 |  |  |  |  |  |  |  |
|  | IVW | 27 | 0.898 | 0.860 | 0.937 | 0.000 |  |  |  |  |  |  |  |
|  | Simple mode | 27 | 0.994 | 0.854 | 1.157 | 0.943 |  |  |  |  |  |  |  |
|  | Weighted mode | 27 | 0.883 | 0.834 | 0.934 | 0.000 |  |  |  |  |  |  |  |
|  | BWMR | 27 | 0.894 | 0.853 | 0.938 | 0.000 |  |  |  |  |  |  |  |
| Phosphatidylethanolamine (18:2_0:0) | MR Egger | 27 | 1.225 | 1.090 | 1.377 | 0.002 | 35.303 | 0.083 | 39.607 | 0.043 | -0.015 | 0.093 | 0.066 |
|  | Weighted median | 27 | 1.168 | 1.084 | 1.260 | 0.000 |  |  |  |  |  |  |  |
|  | IVW | 27 | 1.123 | 1.051 | 1.199 | 0.001 |  |  |  |  |  |  |  |
|  | Simple mode | 27 | 1.106 | 0.911 | 1.344 | 0.318 |  |  |  |  |  |  |  |
|  | Weighted mode | 27 | 1.159 | 1.067 | 1.259 | 0.002 |  |  |  |  |  |  |  |
|  | BWMR | 27 | 1.123 | 1.043 | 1.210 | 0.002 |  |  |  |  |  |  |  |
| Phosphatidylcholine (16:0_18:0) | MR Egger | 39 | 1.135 | 0.949 | 1.359 | 0.174 | 52.951 | 0.043 | 53.367 | 0.050 | -0.006 | 0.593 | 0.037 |
|  | Weighted median | 39 | 1.048 | 0.962 | 1.142 | 0.284 |  |  |  |  |  |  |  |
|  | IVW | 39 | 1.085 | 1.012 | 1.164 | 0.022 |  |  |  |  |  |  |  |
|  | Simple mode | 39 | 0.999 | 0.831 | 1.202 | 0.994 |  |  |  |  |  |  |  |
|  | Weighted mode | 39 | 1.005 | 0.838 | 1.205 | 0.958 |  |  |  |  |  |  |  |
|  | BWMR | 39 | 1.081 | 1.004 | 1.163 | 0.039 |  |  |  |  |  |  |  |
| Phosphatidylcholine (16:0_18:2) | MR Egger | 36 | 1.116 | 0.984 | 1.265 | 0.096 | 48.840 | 0.048 | 49.790 | 0.050 | -0.008 | 0.422 | 0.031 |
|  | Weighted median | 36 | 1.127 | 1.040 | 1.221 | 0.004 |  |  |  |  |  |  |  |
|  | IVW | 36 | 1.065 | 1.005 | 1.129 | 0.033 |  |  |  |  |  |  |  |
|  | Simple mode | 36 | 1.110 | 0.933 | 1.320 | 0.247 |  |  |  |  |  |  |  |
|  | Weighted mode | 36 | 1.188 | 1.090 | 1.295 | 0.000 |  |  |  |  |  |  |  |
|  | BWMR | 36 | 1.054 | 0.991 | 1.121 | 0.096 |  |  |  |  |  |  |  |
| Phosphatidylcholine (16:0_20:2) | MR Egger | 27 | 1.135 | 0.948 | 1.360 | 0.179 | 58.508 | 0.000 | 58.871 | 0.000 | -0.005 | 0.697 | 0.001 |
|  | Weighted median | 27 | 1.111 | 1.003 | 1.231 | 0.044 |  |  |  |  |  |  |  |
|  | IVW | 27 | 1.100 | 1.007 | 1.202 | 0.034 |  |  |  |  |  |  |  |
|  | Simple mode | 27 | 1.043 | 0.808 | 1.345 | 0.751 |  |  |  |  |  |  |  |
|  | Weighted mode | 27 | 1.258 | 1.114 | 1.420 | 0.001 |  |  |  |  |  |  |  |
|  | BWMR | 27 | 1.056 | 0.971 | 1.148 | 0.205 |  |  |  |  |  |  |  |
| Phosphatidylcholine (16:0_20:4) | MR Egger | 24 | 0.879 | 0.823 | 0.938 | 0.001 | 24.428 | 0.325 | 25.456 | 0.327 | 0.007 | 0.346 | 0.390 |
|  | Weighted median | 24 | 0.889 | 0.846 | 0.933 | 0.000 |  |  |  |  |  |  |  |
|  | IVW | 24 | 0.900 | 0.862 | 0.940 | 0.000 |  |  |  |  |  |  |  |
|  | Simple mode | 24 | 0.953 | 0.805 | 1.128 | 0.578 |  |  |  |  |  |  |  |
|  | Weighted mode | 24 | 0.888 | 0.844 | 0.934 | 0.000 |  |  |  |  |  |  |  |
|  | BWMR | 24 | 0.900 | 0.861 | 0.942 | 0.000 |  |  |  |  |  |  |  |
| Phosphatidylcholine (16:0_20:5) | MR Egger | 24 | 0.816 | 0.721 | 0.924 | 0.004 | 32.930 | 0.063 | 37.979 | 0.026 | 0.017 | 0.080 | 0.041 |
|  | Weighted median | 24 | 0.828 | 0.765 | 0.896 | 0.000 |  |  |  |  |  |  |  |
|  | IVW | 24 | 0.899 | 0.836 | 0.966 | 0.004 |  |  |  |  |  |  |  |
|  | Simple mode | 24 | 1.151 | 0.856 | 1.547 | 0.362 |  |  |  |  |  |  |  |
|  | Weighted mode | 24 | 0.827 | 0.761 | 0.899 | 0.000 |  |  |  |  |  |  |  |
|  | BWMR | 24 | 0.906 | 0.839 | 0.977 | 0.011 |  |  |  |  |  |  |  |
| Phosphatidylcholine (16:1_18:1) | MR Egger | 28 | 1.175 | 0.951 | 1.452 | 0.146 | 46.527 | 0.008 | 46.774 | 0.010 | -0.005 | 0.713 | 0.011 |
|  | Weighted median | 28 | 1.125 | 1.013 | 1.250 | 0.028 |  |  |  |  |  |  |  |
|  | IVW | 28 | 1.133 | 1.043 | 1.231 | 0.003 |  |  |  |  |  |  |  |
|  | Simple mode | 28 | 1.041 | 0.794 | 1.365 | 0.771 |  |  |  |  |  |  |  |
|  | Weighted mode | 28 | 1.325 | 1.010 | 1.738 | 0.052 |  |  |  |  |  |  |  |
|  | BWMR | 28 | 1.124 | 1.030 | 1.226 | 0.009 |  |  |  |  |  |  |  |
| Phosphatidylcholine (16:1_18:2) | MR Egger | 25 | 1.179 | 1.001 | 1.388 | 0.060 | 57.231 | 0.000 | 58.549 | 0.000 | -0.009 | 0.474 | 0.002 |
|  | Weighted median | 25 | 1.211 | 1.121 | 1.308 | 0.000 |  |  |  |  |  |  |  |
|  | IVW | 25 | 1.119 | 1.030 | 1.217 | 0.008 |  |  |  |  |  |  |  |
|  | Simple mode | 25 | 1.138 | 0.903 | 1.434 | 0.284 |  |  |  |  |  |  |  |
|  | Weighted mode | 25 | 1.195 | 1.103 | 1.295 | 0.000 |  |  |  |  |  |  |  |
|  | BWMR | 25 | 1.093 | 1.004 | 1.190 | 0.039 |  |  |  |  |  |  |  |
| Phosphatidylcholine (17:0_18:2) | MR Egger | 25 | 1.237 | 1.045 | 1.464 | 0.021 | 27.219 | 0.247 | 28.092 | 0.256 | -0.010 | 0.399 | 0.200 |
|  | Weighted median | 25 | 1.217 | 1.107 | 1.338 | 0.000 |  |  |  |  |  |  |  |
|  | IVW | 25 | 1.156 | 1.081 | 1.237 | 0.000 |  |  |  |  |  |  |  |
|  | Simple mode | 25 | 1.207 | 0.973 | 1.497 | 0.100 |  |  |  |  |  |  |  |
|  | Weighted mode | 25 | 1.284 | 1.149 | 1.434 | 0.000 |  |  |  |  |  |  |  |
|  | BWMR | 25 | 1.157 | 1.077 | 1.243 | 0.000 |  |  |  |  |  |  |  |
| Phosphatidylcholine (17:0_20:4) | MR Egger | 30 | 0.826 | 0.727 | 0.939 | 0.007 | 105.698 | 0.000 | 117.962 | 0.000 | 0.021 | 0.082 | 0.001 |
|  | Weighted median | 30 | 0.877 | 0.829 | 0.928 | 0.000 |  |  |  |  |  |  |  |
|  | IVW | 30 | 0.904 | 0.830 | 0.985 | 0.021 |  |  |  |  |  |  |  |
|  | Simple mode | 30 | 0.925 | 0.794 | 1.077 | 0.323 |  |  |  |  |  |  |  |
|  | Weighted mode | 30 | 0.881 | 0.835 | 0.930 | 0.000 |  |  |  |  |  |  |  |
|  | BWMR | 30 | 0.888 | 0.816 | 0.967 | 0.006 |  |  |  |  |  |  |  |
| Phosphatidylcholine (18:0_18:2) | MR Egger | 33 | 1.262 | 1.066 | 1.495 | 0.011 | 45.349 | 0.046 | 51.269 | 0.017 | -0.022 | 0.053 | 0.007 |
|  | Weighted median | 33 | 1.110 | 1.008 | 1.222 | 0.034 |  |  |  |  |  |  |  |
|  | IVW | 33 | 1.076 | 1.003 | 1.155 | 0.041 |  |  |  |  |  |  |  |
|  | Simple mode | 33 | 1.112 | 0.891 | 1.387 | 0.354 |  |  |  |  |  |  |  |
|  | Weighted mode | 33 | 1.216 | 1.057 | 1.399 | 0.010 |  |  |  |  |  |  |  |
|  | BWMR | 33 | 1.064 | 0.985 | 1.150 | 0.116 |  |  |  |  |  |  |  |
| Phosphatidylcholine (18:0_20:4) | MR Egger | 27 | 0.886 | 0.815 | 0.964 | 0.010 | 64.352 | 0.000 | 65.670 | 0.000 | 0.006 | 0.481 | 0.002 |
|  | Weighted median | 27 | 0.899 | 0.861 | 0.939 | 0.000 |  |  |  |  |  |  |  |
|  | IVW | 27 | 0.906 | 0.855 | 0.960 | 0.001 |  |  |  |  |  |  |  |
|  | Simple mode | 27 | 0.940 | 0.815 | 1.083 | 0.398 |  |  |  |  |  |  |  |
|  | Weighted mode | 27 | 0.896 | 0.858 | 0.937 | 0.000 |  |  |  |  |  |  |  |
|  | BWMR | 27 | 0.908 | 0.855 | 0.965 | 0.002 |  |  |  |  |  |  |  |
| Phosphatidylcholine (18:0_20:5) | MR Egger | 25 | 0.855 | 0.763 | 0.959 | 0.014 | 42.331 | 0.008 | 45.433 | 0.005 | 0.012 | 0.207 | 0.014 |
|  | Weighted median | 25 | 0.856 | 0.798 | 0.919 | 0.000 |  |  |  |  |  |  |  |
|  | IVW | 25 | 0.908 | 0.845 | 0.975 | 0.008 |  |  |  |  |  |  |  |
|  | Simple mode | 25 | 1.083 | 0.862 | 1.361 | 0.501 |  |  |  |  |  |  |  |
|  | Weighted mode | 25 | 0.851 | 0.793 | 0.913 | 0.000 |  |  |  |  |  |  |  |
|  | BWMR | 25 | 0.924 | 0.857 | 0.996 | 0.038 |  |  |  |  |  |  |  |
| Phosphatidylcholine (18:1_18:1) | MR Egger | 22 | 1.227 | 0.956 | 1.575 | 0.124 | 42.986 | 0.002 | 44.638 | 0.002 | -0.015 | 0.391 | 0.001 |
|  | Weighted median | 22 | 1.168 | 1.040 | 1.312 | 0.009 |  |  |  |  |  |  |  |
|  | IVW | 22 | 1.108 | 1.001 | 1.227 | 0.048 |  |  |  |  |  |  |  |
|  | Simple mode | 22 | 1.157 | 0.877 | 1.527 | 0.314 |  |  |  |  |  |  |  |
|  | Weighted mode | 22 | 1.288 | 1.075 | 1.543 | 0.012 |  |  |  |  |  |  |  |
|  | BWMR | 22 | 1.085 | 0.982 | 1.199 | 0.108 |  |  |  |  |  |  |  |
| Phosphatidylcholine (18:1_18:2) | MR Egger | 22 | 1.263 | 1.090 | 1.463 | 0.006 | 40.429 | 0.004 | 48.821 | 0.001 | -0.024 | 0.055 | 0.007 |
|  | Weighted median | 22 | 1.203 | 1.117 | 1.295 | 0.000 |  |  |  |  |  |  |  |
|  | IVW | 22 | 1.109 | 1.020 | 1.206 | 0.016 |  |  |  |  |  |  |  |
|  | Simple mode | 22 | 1.167 | 0.938 | 1.451 | 0.180 |  |  |  |  |  |  |  |
|  | Weighted mode | 22 | 1.202 | 1.111 | 1.301 | 0.000 |  |  |  |  |  |  |  |
|  | BWMR | 22 | 1.091 | 1.000 | 1.190 | 0.051 |  |  |  |  |  |  |  |
| Phosphatidylcholine (18:1_20:2) | MR Egger | 23 | 1.196 | 1.072 | 1.334 | 0.004 | 29.721 | 0.098 | 35.102 | 0.038 | -0.022 | 0.065 | 0.044 |
|  | Weighted median | 23 | 1.143 | 1.070 | 1.221 | 0.000 |  |  |  |  |  |  |  |
|  | IVW | 23 | 1.091 | 1.025 | 1.160 | 0.006 |  |  |  |  |  |  |  |
|  | Simple mode | 23 | 1.034 | 0.853 | 1.253 | 0.736 |  |  |  |  |  |  |  |
|  | Weighted mode | 23 | 1.173 | 1.096 | 1.256 | 0.000 |  |  |  |  |  |  |  |
|  | BWMR | 23 | 1.076 | 1.006 | 1.152 | 0.033 |  |  |  |  |  |  |  |
| Phosphatidylcholine (18:1_20:4) | MR Egger | 28 | 0.869 | 0.794 | 0.952 | 0.005 | 44.085 | 0.015 | 46.446 | 0.011 | 0.010 | 0.249 | 0.040 |
|  | Weighted median | 28 | 0.877 | 0.828 | 0.929 | 0.000 |  |  |  |  |  |  |  |
|  | IVW | 28 | 0.907 | 0.856 | 0.961 | 0.001 |  |  |  |  |  |  |  |
|  | Simple mode | 28 | 1.175 | 0.958 | 1.440 | 0.133 |  |  |  |  |  |  |  |
|  | Weighted mode | 28 | 0.874 | 0.828 | 0.921 | 0.000 |  |  |  |  |  |  |  |
|  | BWMR | 28 | 0.907 | 0.857 | 0.961 | 0.001 |  |  |  |  |  |  |  |
| Phosphatidylcholine (18:2_18:2) | MR Egger | 23 | 1.248 | 1.049 | 1.485 | 0.021 | 30.514 | 0.082 | 33.301 | 0.058 | -0.015 | 0.181 | 0.029 |
|  | Weighted median | 23 | 1.139 | 1.022 | 1.269 | 0.019 |  |  |  |  |  |  |  |
|  | IVW | 23 | 1.119 | 1.032 | 1.214 | 0.007 |  |  |  |  |  |  |  |
|  | Simple mode | 23 | 1.039 | 0.825 | 1.310 | 0.746 |  |  |  |  |  |  |  |
|  | Weighted mode | 23 | 1.305 | 1.112 | 1.530 | 0.004 |  |  |  |  |  |  |  |
|  | BWMR | 23 | 1.097 | 0.999 | 1.205 | 0.052 |  |  |  |  |  |  |  |
| Phosphatidylcholine (O-16:0_18:2) | MR Egger | 20 | 0.975 | 0.767 | 1.239 | 0.837 | 33.150 | 0.016 | 35.844 | 0.011 | 0.019 | 0.242 | 0.008 |
|  | Weighted median | 20 | 1.114 | 0.982 | 1.263 | 0.092 |  |  |  |  |  |  |  |
|  | IVW | 20 | 1.114 | 1.005 | 1.235 | 0.040 |  |  |  |  |  |  |  |
|  | Simple mode | 20 | 1.073 | 0.843 | 1.365 | 0.575 |  |  |  |  |  |  |  |
|  | Weighted mode | 20 | 1.087 | 0.791 | 1.495 | 0.611 |  |  |  |  |  |  |  |
|  | BWMR | 20 | 1.078 | 0.973 | 1.194 | 0.151 |  |  |  |  |  |  |  |
| Phosphatidylcholine (O-16:1_20:4) | MR Egger | 23 | 0.875 | 0.765 | 1.001 | 0.065 | 42.392 | 0.004 | 44.259 | 0.003 | 0.011 | 0.347 | 0.011 |
|  | Weighted median | 23 | 0.842 | 0.782 | 0.907 | 0.000 |  |  |  |  |  |  |  |
|  | IVW | 23 | 0.925 | 0.862 | 0.993 | 0.032 |  |  |  |  |  |  |  |
|  | Simple mode | 23 | 0.976 | 0.800 | 1.191 | 0.812 |  |  |  |  |  |  |  |
|  | Weighted mode | 23 | 0.851 | 0.793 | 0.913 | 0.000 |  |  |  |  |  |  |  |
|  | BWMR | 23 | 0.931 | 0.867 | 0.999 | 0.048 |  |  |  |  |  |  |  |
| Phosphatidylcholine (O-17:0_17:1) | MR Egger | 33 | 1.000 | 0.815 | 1.226 | 0.999 | 47.073 | 0.032 | 48.337 | 0.032 | 0.011 | 0.369 | 0.025 |
|  | Weighted median | 33 | 1.111 | 1.012 | 1.220 | 0.027 |  |  |  |  |  |  |  |
|  | IVW | 33 | 1.091 | 1.010 | 1.180 | 0.028 |  |  |  |  |  |  |  |
|  | Simple mode | 33 | 1.150 | 0.948 | 1.395 | 0.166 |  |  |  |  |  |  |  |
|  | Weighted mode | 33 | 1.138 | 0.947 | 1.369 | 0.178 |  |  |  |  |  |  |  |
|  | BWMR | 33 | 1.085 | 0.998 | 1.179 | 0.056 |  |  |  |  |  |  |  |
| Phosphatidylcholine (O-18:0_20:4) | MR Egger | 21 | 0.713 | 0.623 | 0.816 | 0.000 | 22.963 | 0.239 | 35.090 | 0.020 | 0.033 | 0.005 | 0.026 |
|  | Weighted median | 21 | 0.808 | 0.736 | 0.886 | 0.000 |  |  |  |  |  |  |  |
|  | IVW | 21 | 0.860 | 0.792 | 0.934 | 0.000 |  |  |  |  |  |  |  |
|  | Simple mode | 21 | 0.842 | 0.695 | 1.021 | 0.096 |  |  |  |  |  |  |  |
|  | Weighted mode | 21 | 0.808 | 0.734 | 0.889 | 0.000 |  |  |  |  |  |  |  |
|  | BWMR | 21 | 0.852 | 0.779 | 0.932 | 0.000 |  |  |  |  |  |  |  |
| Phosphatidylcholine (O-18:1_18:2) | MR Egger | 25 | 1.274 | 1.064 | 1.526 | 0.015 | 34.621 | 0.057 | 39.828 | 0.022 | -0.023 | 0.076 | 0.015 |
|  | Weighted median | 25 | 1.162 | 1.048 | 1.289 | 0.004 |  |  |  |  |  |  |  |
|  | IVW | 25 | 1.092 | 1.005 | 1.187 | 0.037 |  |  |  |  |  |  |  |
|  | Simple mode | 25 | 1.188 | 0.898 | 1.570 | 0.239 |  |  |  |  |  |  |  |
|  | Weighted mode | 25 | 1.283 | 1.121 | 1.468 | 0.001 |  |  |  |  |  |  |  |
|  | BWMR | 25 | 1.079 | 0.987 | 1.180 | 0.093 |  |  |  |  |  |  |  |
| Phosphatidylethanolamine (18:0_18:2) | MR Egger | 29 | 1.071 | 0.951 | 1.207 | 0.266 | 52.872 | 0.002 | 53.249 | 0.003 | 0.005 | 0.664 | 0.003 |
|  | Weighted median | 29 | 1.042 | 0.968 | 1.122 | 0.273 |  |  |  |  |  |  |  |
|  | IVW | 29 | 1.096 | 1.032 | 1.164 | 0.003 |  |  |  |  |  |  |  |
|  | Simple mode | 29 | 1.025 | 0.894 | 1.176 | 0.724 |  |  |  |  |  |  |  |
|  | Weighted mode | 29 | 1.056 | 0.950 | 1.174 | 0.323 |  |  |  |  |  |  |  |
|  | BWMR | 29 | 1.086 | 1.024 | 1.152 | 0.006 |  |  |  |  |  |  |  |
| Sphingomyelin (d36:1) | MR Egger | 35 | 0.946 | 0.814 | 1.099 | 0.473 | 42.418 | 0.126 | 42.466 | 0.151 | -0.002 | 0.847 | 0.168 |
|  | Weighted median | 35 | 0.951 | 0.875 | 1.032 | 0.228 |  |  |  |  |  |  |  |
|  | IVW | 35 | 0.933 | 0.877 | 0.993 | 0.030 |  |  |  |  |  |  |  |
|  | Simple mode | 35 | 0.960 | 0.808 | 1.141 | 0.647 |  |  |  |  |  |  |  |
|  | Weighted mode | 35 | 0.929 | 0.822 | 1.049 | 0.241 |  |  |  |  |  |  |  |
|  | BWMR | 35 | 0.932 | 0.872 | 0.996 | 0.038 |  |  |  |  |  |  |  |
| Sphingomyelin (d38:1) | MR Egger | 38 | 0.892 | 0.814 | 0.976 | 0.018 | 26.245 | 0.884 | 26.594 | 0.897 | 0.004 | 0.559 | 0.912 |
|  | Weighted median | 38 | 0.903 | 0.841 | 0.970 | 0.005 |  |  |  |  |  |  |  |
|  | IVW | 38 | 0.913 | 0.871 | 0.957 | 0.000 |  |  |  |  |  |  |  |
|  | Simple mode | 38 | 0.908 | 0.805 | 1.024 | 0.125 |  |  |  |  |  |  |  |
|  | Weighted mode | 38 | 0.902 | 0.833 | 0.977 | 0.016 |  |  |  |  |  |  |  |
|  | BWMR | 38 | 0.911 | 0.868 | 0.957 | 0.000 |  |  |  |  |  |  |  |
| Sphingomyelin (d38:2) | MR Egger | 31 | 1.009 | 0.877 | 1.160 | 0.904 | 45.607 | 0.026 | 48.399 | 0.018 | -0.016 | 0.193 | 0.017 |
|  | Weighted median | 31 | 0.926 | 0.850 | 1.010 | 0.083 |  |  |  |  |  |  |  |
|  | IVW | 31 | 0.928 | 0.868 | 0.993 | 0.030 |  |  |  |  |  |  |  |
|  | Simple mode | 31 | 1.005 | 0.859 | 1.175 | 0.952 |  |  |  |  |  |  |  |
|  | Weighted mode | 31 | 0.944 | 0.853 | 1.045 | 0.274 |  |  |  |  |  |  |  |
|  | BWMR | 31 | 0.937 | 0.877 | 1.001 | 0.053 |  |  |  |  |  |  |  |
| Sphingomyelin (d40:2) | MR Egger | 36 | 1.013 | 0.871 | 1.177 | 0.870 | 63.906 | 0.001 | 69.137 | 0.001 | -0.018 | 0.104 | 0.001 |
|  | Weighted median | 36 | 0.900 | 0.821 | 0.988 | 0.027 |  |  |  |  |  |  |  |
|  | IVW | 36 | 0.906 | 0.840 | 0.977 | 0.011 |  |  |  |  |  |  |  |
|  | Simple mode | 36 | 0.862 | 0.738 | 1.006 | 0.068 |  |  |  |  |  |  |  |
|  | Weighted mode | 36 | 0.896 | 0.807 | 0.995 | 0.049 |  |  |  |  |  |  |  |
|  | BWMR | 36 | 0.927 | 0.860 | 1.000 | 0.049 |  |  |  |  |  |  |  |
| Sphingomyelin (d42:2) | MR Egger | 38 | 0.970 | 0.876 | 1.074 | 0.566 | 35.909 | 0.473 | 36.631 | 0.486 | -0.006 | 0.401 | 0.460 |
|  | Weighted median | 38 | 0.899 | 0.831 | 0.972 | 0.007 |  |  |  |  |  |  |  |
|  | IVW | 38 | 0.934 | 0.886 | 0.986 | 0.013 |  |  |  |  |  |  |  |
|  | Simple mode | 38 | 0.890 | 0.763 | 1.038 | 0.145 |  |  |  |  |  |  |  |
|  | Weighted mode | 38 | 0.878 | 0.766 | 1.006 | 0.069 |  |  |  |  |  |  |  |
|  | BWMR | 38 | 0.930 | 0.878 | 0.985 | 0.013 |  |  |  |  |  |  |  |
| Triacylglycerol (51:2) | MR Egger | 21 | 0.918 | 0.724 | 1.164 | 0.489 | 32.438 | 0.028 | 38.048 | 0.009 | 0.029 | 0.086 | 0.004 |
|  | Weighted median | 21 | 1.081 | 0.962 | 1.216 | 0.191 |  |  |  |  |  |  |  |
|  | IVW | 21 | 1.121 | 1.010 | 1.243 | 0.031 |  |  |  |  |  |  |  |
|  | Simple mode | 21 | 1.085 | 0.873 | 1.349 | 0.469 |  |  |  |  |  |  |  |
|  | Weighted mode | 21 | 0.988 | 0.822 | 1.187 | 0.895 |  |  |  |  |  |  |  |
|  | BWMR | 21 | 1.102 | 0.991 | 1.224 | 0.072 |  |  |  |  |  |  |  |
| Triacylglycerol (52:2) | MR Egger | 24 | 1.072 | 0.901 | 1.275 | 0.443 | 26.983 | 0.212 | 27.009 | 0.256 | 0.002 | 0.886 | 0.233 |
|  | Weighted median | 24 | 1.047 | 0.945 | 1.160 | 0.382 |  |  |  |  |  |  |  |
|  | IVW | 24 | 1.084 | 1.006 | 1.168 | 0.034 |  |  |  |  |  |  |  |
|  | Simple mode | 24 | 1.026 | 0.849 | 1.241 | 0.791 |  |  |  |  |  |  |  |
|  | Weighted mode | 24 | 0.972 | 0.829 | 1.140 | 0.731 |  |  |  |  |  |  |  |
|  | BWMR | 24 | 1.088 | 1.003 | 1.180 | 0.042 |  |  |  |  |  |  |  |
| **UC** |  |  |  |  |  |  |  |  |  |  |  |  |  |
| Sterol ester (27:1/20:4) | MR Egger | 31 | 0.874 | 0.804 | 0.949 | 0.003 | 46.410 | 0.021 | 47.865 | 0.020 | 0.009 | 0.348 | 0.059 |
|  | Weighted median | 31 | 0.883 | 0.836 | 0.933 | 0.000 |  |  |  |  |  |  |  |
|  | IVW | 31 | 0.900 | 0.851 | 0.952 | 0.000 |  |  |  |  |  |  |  |
|  | Simple mode | 31 | 0.954 | 0.817 | 1.113 | 0.552 |  |  |  |  |  |  |  |
|  | Weighted mode | 31 | 0.890 | 0.841 | 0.941 | 0.000 |  |  |  |  |  |  |  |
|  | BWMR | 31 | 0.906 | 0.849 | 0.967 | 0.003 |  |  |  |  |  |  |  |
| Sterol ester (27:1/20:5) | MR Egger | 26 | 0.795 | 0.706 | 0.895 | 0.001 | 25.929 | 0.357 | 32.489 | 0.144 | 0.021 | 0.021 | 0.098 |
|  | Weighted median | 26 | 0.821 | 0.749 | 0.899 | 0.000 |  |  |  |  |  |  |  |
|  | IVW | 26 | 0.900 | 0.837 | 0.968 | 0.004 |  |  |  |  |  |  |  |
|  | Simple mode | 26 | 1.074 | 0.832 | 1.385 | 0.588 |  |  |  |  |  |  |  |
|  | Weighted mode | 26 | 0.827 | 0.759 | 0.901 | 0.000 |  |  |  |  |  |  |  |
|  | BWMR | 26 | 0.901 | 0.836 | 0.971 | 0.007 |  |  |  |  |  |  |  |
| Phosphatidylcholine (20:4_0:0) | MR Egger | 27 | 0.865 | 0.793 | 0.943 | 0.003 | 26.781 | 0.367 | 27.216 | 0.398 | 0.006 | 0.530 | 0.420 |
|  | Weighted median | 27 | 0.872 | 0.815 | 0.933 | 0.000 |  |  |  |  |  |  |  |
|  | IVW | 27 | 0.884 | 0.838 | 0.932 | 0.000 |  |  |  |  |  |  |  |
|  | Simple mode | 27 | 0.923 | 0.758 | 1.124 | 0.434 |  |  |  |  |  |  |  |
|  | Weighted mode | 27 | 0.868 | 0.810 | 0.930 | 0.000 |  |  |  |  |  |  |  |
|  | BWMR | 27 | 0.879 | 0.833 | 0.927 | 0.000 |  |  |  |  |  |  |  |
| Phosphatidylethanolamine (18:2_0:0) | MR Egger | 27 | 1.210 | 1.063 | 1.378 | 0.008 | 28.743 | 0.275 | 30.724 | 0.239 | -0.013 | 0.201 | 0.241 |
|  | Weighted median | 27 | 1.198 | 1.091 | 1.314 | 0.000 |  |  |  |  |  |  |  |
|  | IVW | 27 | 1.125 | 1.048 | 1.208 | 0.001 |  |  |  |  |  |  |  |
|  | Simple mode | 27 | 1.147 | 0.940 | 1.401 | 0.189 |  |  |  |  |  |  |  |
|  | Weighted mode | 27 | 1.187 | 1.080 | 1.305 | 0.002 |  |  |  |  |  |  |  |
|  | BWMR | 27 | 1.124 | 1.038 | 1.217 | 0.004 |  |  |  |  |  |  |  |
| Phosphatidylcholine (14:0_16:0 | MR Egger | 19 | 0.903 | 0.717 | 1.139 | 0.402 | 13.941 | 0.671 | 13.951 | 0.732 | -0.002 | 0.923 | 0.739 |
|  | Weighted median | 19 | 0.949 | 0.832 | 1.082 | 0.432 |  |  |  |  |  |  |  |
|  | IVW | 19 | 0.894 | 0.813 | 0.983 | 0.021 |  |  |  |  |  |  |  |
|  | Simple mode | 19 | 0.974 | 0.763 | 1.243 | 0.833 |  |  |  |  |  |  |  |
|  | Weighted mode | 19 | 0.983 | 0.791 | 1.221 | 0.875 |  |  |  |  |  |  |  |
|  | BWMR | 19 | 0.891 | 0.805 | 0.986 | 0.026 |  |  |  |  |  |  |  |
| Phosphatidylcholine (16:0_18:2) | MR Egger | 36 | 1.190 | 1.035 | 1.368 | 0.020 | 39.896 | 0.224 | 42.995 | 0.166 | -0.018 | 0.113 | 0.084 |
|  | Weighted median | 36 | 1.162 | 1.059 | 1.276 | 0.002 |  |  |  |  |  |  |  |
|  | IVW | 36 | 1.075 | 1.005 | 1.148 | 0.034 |  |  |  |  |  |  |  |
|  | Simple mode | 36 | 0.901 | 0.702 | 1.156 | 0.418 |  |  |  |  |  |  |  |
|  | Weighted mode | 36 | 1.242 | 1.119 | 1.379 | 0.000 |  |  |  |  |  |  |  |
|  | BWMR | 36 | 1.064 | 0.989 | 1.145 | 0.098 |  |  |  |  |  |  |  |
| Phosphatidylcholine (16:0_20:2) | MR Egger | 27 | 1.254 | 1.070 | 1.471 | 0.010 | 30.143 | 0.219 | 32.365 | 0.181 | -0.016 | 0.187 | 0.112 |
|  | Weighted median | 27 | 1.235 | 1.105 | 1.381 | 0.000 |  |  |  |  |  |  |  |
|  | IVW | 27 | 1.140 | 1.052 | 1.236 | 0.001 |  |  |  |  |  |  |  |
|  | Simple mode | 27 | 1.295 | 0.991 | 1.693 | 0.070 |  |  |  |  |  |  |  |
|  | Weighted mode | 27 | 1.332 | 1.167 | 1.520 | 0.000 |  |  |  |  |  |  |  |
|  | BWMR | 27 | 1.121 | 1.030 | 1.220 | 0.008 |  |  |  |  |  |  |  |
| Phosphatidylcholine (16:0_20:4) | MR Egger | 24 | 0.850 | 0.788 | 0.918 | 0.000 | 17.166 | 0.754 | 20.876 | 0.589 | 0.017 | 0.067 | 0.526 |
|  | Weighted median | 24 | 0.876 | 0.822 | 0.935 | 0.000 |  |  |  |  |  |  |  |
|  | IVW | 24 | 0.899 | 0.855 | 0.946 | 0.000 |  |  |  |  |  |  |  |
|  | Simple mode | 24 | 0.917 | 0.733 | 1.148 | 0.459 |  |  |  |  |  |  |  |
|  | Weighted mode | 24 | 0.876 | 0.823 | 0.931 | 0.000 |  |  |  |  |  |  |  |
|  | BWMR | 24 | 0.899 | 0.854 | 0.945 | 0.000 |  |  |  |  |  |  |  |
| Phosphatidylcholine (16:0_20:5) | MR Egger | 24 | 0.802 | 0.703 | 0.914 | 0.003 | 24.287 | 0.332 | 27.150 | 0.250 | 0.016 | 0.122 | 0.162 |
|  | Weighted median | 24 | 0.807 | 0.735 | 0.885 | 0.000 |  |  |  |  |  |  |  |
|  | IVW | 24 | 0.876 | 0.813 | 0.945 | 0.001 |  |  |  |  |  |  |  |
|  | Simple mode | 24 | 1.102 | 0.825 | 1.473 | 0.517 |  |  |  |  |  |  |  |
|  | Weighted mode | 24 | 0.808 | 0.730 | 0.894 | 0.000 |  |  |  |  |  |  |  |
|  | BWMR | 24 | 0.877 | 0.811 | 0.949 | 0.001 |  |  |  |  |  |  |  |
| Phosphatidylcholine (16:1_18:1) | MR Egger | 28 | 1.181 | 0.937 | 1.489 | 0.170 | 36.624 | 0.081 | 36.856 | 0.098 | -0.006 | 0.688 | 0.090 |
|  | Weighted median | 28 | 1.160 | 1.026 | 1.312 | 0.018 |  |  |  |  |  |  |  |
|  | IVW | 28 | 1.130 | 1.032 | 1.238 | 0.008 |  |  |  |  |  |  |  |
|  | Simple mode | 28 | 1.217 | 0.915 | 1.619 | 0.188 |  |  |  |  |  |  |  |
|  | Weighted mode | 28 | 1.341 | 1.055 | 1.705 | 0.024 |  |  |  |  |  |  |  |
|  | BWMR | 28 | 1.125 | 1.023 | 1.238 | 0.015 |  |  |  |  |  |  |  |
| Phosphatidylcholine (16:1_18:2) | MR Egger | 25 | 1.204 | 1.011 | 1.434 | 0.049 | 43.339 | 0.006 | 45.022 | 0.006 | -0.012 | 0.354 | 0.010 |
|  | Weighted median | 25 | 1.233 | 1.123 | 1.354 | 0.000 |  |  |  |  |  |  |  |
|  | IVW | 25 | 1.120 | 1.023 | 1.226 | 0.014 |  |  |  |  |  |  |  |
|  | Simple mode | 25 | 0.905 | 0.670 | 1.222 | 0.520 |  |  |  |  |  |  |  |
|  | Weighted mode | 25 | 1.240 | 1.121 | 1.372 | 0.000 |  |  |  |  |  |  |  |
|  | BWMR | 25 | 1.107 | 1.014 | 1.208 | 0.023 |  |  |  |  |  |  |  |
| Phosphatidylcholine (17:0_18:2) | MR Egger | 25 | 1.298 | 1.036 | 1.626 | 0.033 | 32.175 | 0.097 | 33.552 | 0.093 | -0.016 | 0.331 | 0.073 |
|  | Weighted median | 25 | 1.259 | 1.118 | 1.418 | 0.000 |  |  |  |  |  |  |  |
|  | IVW | 25 | 1.169 | 1.068 | 1.280 | 0.001 |  |  |  |  |  |  |  |
|  | Simple mode | 25 | 1.287 | 0.958 | 1.728 | 0.107 |  |  |  |  |  |  |  |
|  | Weighted mode | 25 | 1.337 | 1.158 | 1.544 | 0.001 |  |  |  |  |  |  |  |
|  | BWMR | 25 | 1.169 | 1.065 | 1.282 | 0.001 |  |  |  |  |  |  |  |
| Phosphatidylcholine (17:0_20:4) | MR Egger | 30 | 0.833 | 0.740 | 0.937 | 0.005 | 59.451 | 0.000 | 62.010 | 0.000 | 0.012 | 0.282 | 0.005 |
|  | Weighted median | 30 | 0.865 | 0.808 | 0.925 | 0.000 |  |  |  |  |  |  |  |
|  | IVW | 30 | 0.876 | 0.812 | 0.945 | 0.001 |  |  |  |  |  |  |  |
|  | Simple mode | 30 | 0.947 | 0.770 | 1.163 | 0.605 |  |  |  |  |  |  |  |
|  | Weighted mode | 30 | 0.867 | 0.810 | 0.929 | 0.000 |  |  |  |  |  |  |  |
|  | BWMR | 30 | 0.873 | 0.807 | 0.944 | 0.001 |  |  |  |  |  |  |  |
| Phosphatidylcholine (18:0_20:4) | MR Egger | 27 | 0.872 | 0.808 | 0.941 | 0.002 | 34.757 | 0.093 | 36.564 | 0.082 | 0.009 | 0.265 | 0.142 |
|  | Weighted median | 27 | 0.886 | 0.840 | 0.935 | 0.000 |  |  |  |  |  |  |  |
|  | IVW | 27 | 0.901 | 0.854 | 0.950 | 0.000 |  |  |  |  |  |  |  |
|  | Simple mode | 27 | 0.854 | 0.726 | 1.006 | 0.070 |  |  |  |  |  |  |  |
|  | Weighted mode | 27 | 0.886 | 0.838 | 0.936 | 0.000 |  |  |  |  |  |  |  |
|  | BWMR | 27 | 0.902 | 0.854 | 0.953 | 0.000 |  |  |  |  |  |  |  |
| Phosphatidylcholine (18:0_20:5) | MR Egger | 25 | 0.831 | 0.746 | 0.926 | 0.003 | 25.108 | 0.345 | 28.104 | 0.256 | 0.014 | 0.111 | 0.178 |
|  | Weighted median | 25 | 0.851 | 0.781 | 0.927 | 0.000 |  |  |  |  |  |  |  |
|  | IVW | 25 | 0.893 | 0.834 | 0.957 | 0.001 |  |  |  |  |  |  |  |
|  | Simple mode | 25 | 1.133 | 0.879 | 1.459 | 0.345 |  |  |  |  |  |  |  |
|  | Weighted mode | 25 | 0.823 | 0.755 | 0.897 | 0.000 |  |  |  |  |  |  |  |
|  | BWMR | 25 | 0.898 | 0.832 | 0.969 | 0.005 |  |  |  |  |  |  |  |
| Phosphatidylcholine (18:1_18:1) | MR Egger | 22 | 1.223 | 0.916 | 1.632 | 0.187 | 38.471 | 0.008 | 39.114 | 0.010 | -0.011 | 0.570 | 0.008 |
|  | Weighted median | 22 | 1.175 | 1.027 | 1.345 | 0.019 |  |  |  |  |  |  |  |
|  | IVW | 22 | 1.131 | 1.007 | 1.272 | 0.038 |  |  |  |  |  |  |  |
|  | Simple mode | 22 | 1.173 | 0.858 | 1.605 | 0.328 |  |  |  |  |  |  |  |
|  | Weighted mode | 22 | 1.307 | 1.080 | 1.583 | 0.012 |  |  |  |  |  |  |  |
|  | BWMR | 22 | 1.117 | 0.999 | 1.250 | 0.053 |  |  |  |  |  |  |  |
| Phosphatidylcholine (18:1_18:2) | MR Egger | 22 | 1.321 | 1.125 | 1.551 | 0.003 | 31.930 | 0.044 | 41.104 | 0.005 | -0.031 | 0.026 | 0.021 |
|  | Weighted median | 22 | 1.174 | 1.067 | 1.293 | 0.001 |  |  |  |  |  |  |  |
|  | IVW | 22 | 1.119 | 1.018 | 1.230 | 0.020 |  |  |  |  |  |  |  |
|  | Simple mode | 22 | 0.991 | 0.747 | 1.315 | 0.951 |  |  |  |  |  |  |  |
|  | Weighted mode | 22 | 1.216 | 1.104 | 1.341 | 0.001 |  |  |  |  |  |  |  |
|  | BWMR | 22 | 1.097 | 0.994 | 1.211 | 0.065 |  |  |  |  |  |  |  |
| Phosphatidylcholine (18:1_20:2) | MR Egger | 23 | 1.225 | 1.078 | 1.392 | 0.005 | 26.806 | 0.177 | 32.478 | 0.070 | -0.028 | 0.047 | 0.060 |
|  | Weighted median | 23 | 1.191 | 1.095 | 1.296 | 0.000 |  |  |  |  |  |  |  |
|  | IVW | 23 | 1.091 | 1.014 | 1.173 | 0.020 |  |  |  |  |  |  |  |
|  | Simple mode | 23 | 1.124 | 0.885 | 1.426 | 0.348 |  |  |  |  |  |  |  |
|  | Weighted mode | 23 | 1.195 | 1.104 | 1.293 | 0.000 |  |  |  |  |  |  |  |
|  | BWMR | 23 | 1.078 | 0.998 | 1.164 | 0.057 |  |  |  |  |  |  |  |
| Phosphatidylcholine (18:1_20:4) | MR Egger | 28 | 0.866 | 0.783 | 0.957 | 0.009 | 35.581 | 0.100 | 37.798 | 0.081 | 0.012 | 0.214 | 0.101 |
|  | Weighted median | 28 | 0.859 | 0.801 | 0.923 | 0.000 |  |  |  |  |  |  |  |
|  | IVW | 28 | 0.910 | 0.854 | 0.971 | 0.004 |  |  |  |  |  |  |  |
|  | Simple mode | 28 | 0.946 | 0.749 | 1.194 | 0.643 |  |  |  |  |  |  |  |
|  | Weighted mode | 28 | 0.867 | 0.805 | 0.935 | 0.001 |  |  |  |  |  |  |  |
|  | BWMR | 28 | 0.910 | 0.854 | 0.969 | 0.003 |  |  |  |  |  |  |  |
| Phosphatidylcholine (18:2_18:2) | MR Egger | 23 | 1.261 | 1.027 | 1.548 | 0.038 | 28.191 | 0.135 | 29.936 | 0.120 | -0.015 | 0.267 | 0.094 |
|  | Weighted median | 23 | 1.061 | 0.932 | 1.209 | 0.370 |  |  |  |  |  |  |  |
|  | IVW | 23 | 1.134 | 1.031 | 1.247 | 0.010 |  |  |  |  |  |  |  |
|  | Simple mode | 23 | 0.959 | 0.721 | 1.277 | 0.778 |  |  |  |  |  |  |  |
|  | Weighted mode | 23 | 1.435 | 1.156 | 1.782 | 0.003 |  |  |  |  |  |  |  |
|  | BWMR | 23 | 1.110 | 0.996 | 1.238 | 0.059 |  |  |  |  |  |  |  |
| Phosphatidylcholine (O-16:0_18:2) | MR Egger | 20 | 1.010 | 0.765 | 1.334 | 0.942 | 29.544 | 0.042 | 31.578 | 0.035 | 0.021 | 0.280 | 0.032 |
|  | Weighted median | 20 | 1.245 | 1.070 | 1.448 | 0.005 |  |  |  |  |  |  |  |
|  | IVW | 20 | 1.165 | 1.035 | 1.313 | 0.012 |  |  |  |  |  |  |  |
|  | Simple mode | 20 | 1.230 | 0.891 | 1.698 | 0.223 |  |  |  |  |  |  |  |
|  | Weighted mode | 20 | 1.389 | 1.028 | 1.878 | 0.046 |  |  |  |  |  |  |  |
|  | BWMR | 20 | 1.143 | 1.016 | 1.287 | 0.026 |  |  |  |  |  |  |  |
| Phosphatidylcholine (O-16:1_20:4) | MR Egger | 23 | 0.823 | 0.723 | 0.937 | 0.008 | 26.286 | 0.196 | 30.877 | 0.099 | 0.022 | 0.069 | 0.069 |
|  | Weighted median | 23 | 0.857 | 0.779 | 0.942 | 0.001 |  |  |  |  |  |  |  |
|  | IVW | 23 | 0.917 | 0.852 | 0.986 | 0.019 |  |  |  |  |  |  |  |
|  | Simple mode | 23 | 0.993 | 0.766 | 1.286 | 0.956 |  |  |  |  |  |  |  |
|  | Weighted mode | 23 | 0.827 | 0.754 | 0.906 | 0.001 |  |  |  |  |  |  |  |
|  | BWMR | 23 | 0.913 | 0.848 | 0.983 | 0.016 |  |  |  |  |  |  |  |
| Phosphatidylcholine (O-18:0_20:4) | MR Egger | 21 | 0.687 | 0.577 | 0.818 | 0.000 | 25.628 | 0.141 | 36.832 | 0.012 | 0.039 | 0.010 | 0.020 |
|  | Weighted median | 21 | 0.820 | 0.733 | 0.919 | 0.001 |  |  |  |  |  |  |  |
|  | IVW | 21 | 0.858 | 0.774 | 0.951 | 0.004 |  |  |  |  |  |  |  |
|  | Simple mode | 21 | 0.876 | 0.686 | 1.120 | 0.304 |  |  |  |  |  |  |  |
|  | Weighted mode | 21 | 0.802 | 0.712 | 0.902 | 0.002 |  |  |  |  |  |  |  |
|  | BWMR | 21 | 0.850 | 0.761 | 0.950 | 0.004 |  |  |  |  |  |  |  |
| Phosphatidylcholine (O-18:1_18:2) | MR Egger | 25 | 1.363 | 1.138 | 1.634 | 0.003 | 21.423 | 0.555 | 25.306 | 0.389 | -0.024 | 0.061 | 0.270 |
|  | Weighted median | 25 | 1.242 | 1.104 | 1.397 | 0.000 |  |  |  |  |  |  |  |
|  | IVW | 25 | 1.158 | 1.067 | 1.256 | 0.000 |  |  |  |  |  |  |  |
|  | Simple mode | 25 | 1.284 | 0.988 | 1.667 | 0.073 |  |  |  |  |  |  |  |
|  | Weighted mode | 25 | 1.345 | 1.149 | 1.573 | 0.001 |  |  |  |  |  |  |  |
|  | BWMR | 25 | 1.163 | 1.061 | 1.275 | 0.001 |  |  |  |  |  |  |  |
| Phosphatidylcholine (O-18:2_16:0) | MR Egger | 26 | 1.031 | 0.837 | 1.269 | 0.779 | 35.525 | 0.061 | 37.251 | 0.055 | 0.016 | 0.291 | 0.034 |
|  | Weighted median | 26 | 1.062 | 0.930 | 1.211 | 0.375 |  |  |  |  |  |  |  |
|  | IVW | 26 | 1.138 | 1.025 | 1.264 | 0.015 |  |  |  |  |  |  |  |
|  | Simple mode | 26 | 1.015 | 0.793 | 1.299 | 0.909 |  |  |  |  |  |  |  |
|  | Weighted mode | 26 | 0.994 | 0.773 | 1.279 | 0.963 |  |  |  |  |  |  |  |
|  | BWMR | 26 | 1.113 | 0.999 | 1.241 | 0.052 |  |  |  |  |  |  |  |
| Phosphatidylcholine (O-18:2_18:1) | MR Egger | 17 | 1.329 | 0.902 | 1.957 | 0.171 | 23.450 | 0.075 | 24.370 | 0.082 | -0.018 | 0.455 | 0.063 |
|  | Weighted median | 17 | 1.135 | 0.967 | 1.333 | 0.120 |  |  |  |  |  |  |  |
|  | IVW | 17 | 1.152 | 1.013 | 1.309 | 0.031 |  |  |  |  |  |  |  |
|  | Simple mode | 17 | 1.117 | 0.827 | 1.509 | 0.482 |  |  |  |  |  |  |  |
|  | Weighted mode | 17 | 1.123 | 0.785 | 1.608 | 0.534 |  |  |  |  |  |  |  |
|  | BWMR | 17 | 1.120 | 0.976 | 1.286 | 0.106 |  |  |  |  |  |  |  |
| Phosphatidylethanolamine (18:0_18:2) | MR Egger | 29 | 1.102 | 0.966 | 1.256 | 0.159 | 23.450 | 0.075 | 24.370 | 0.082 | -0.018 | 0.455 | 0.287 |
|  | Weighted median | 29 | 1.083 | 0.984 | 1.192 | 0.102 |  |  |  |  |  |  |  |
|  | IVW | 29 | 1.091 | 1.022 | 1.166 | 0.009 |  |  |  |  |  |  |  |
|  | Simple mode | 29 | 1.031 | 0.856 | 1.243 | 0.749 |  |  |  |  |  |  |  |
|  | Weighted mode | 29 | 1.097 | 0.986 | 1.220 | 0.101 |  |  |  |  |  |  |  |
|  | BWMR | 29 | 1.084 | 1.013 | 1.160 | 0.019 |  |  |  |  |  |  |  |
| Phosphatidylethanolamine (O-16:1_20:4) | MR Egger | 22 | 0.566 | 0.433 | 0.740 | 0.000 | 27.602 | 0.119 | 43.953 | 0.002 | 0.053 | 0.003 | 0.002 |
|  | Weighted median | 22 | 0.753 | 0.651 | 0.872 | 0.000 |  |  |  |  |  |  |  |
|  | IVW | 22 | 0.876 | 0.775 | 0.990 | 0.034 |  |  |  |  |  |  |  |
|  | Simple mode | 22 | 0.736 | 0.503 | 1.077 | 0.130 |  |  |  |  |  |  |  |
|  | Weighted mode | 22 | 0.705 | 0.601 | 0.828 | 0.000 |  |  |  |  |  |  |  |
|  | BWMR | 22 | 0.909 | 0.816 | 1.014 | 0.087 |  |  |  |  |  |  |  |
| Phosphatidylethanolamine (O-18:2_20:4) | MR Egger | 26 | 0.708 | 0.567 | 0.884 | 0.006 | 28.833 | 0.226 | 35.491 | 0.080 | 0.036 | 0.027 | 0.067 |
|  | Weighted median | 26 | 0.884 | 0.774 | 1.011 | 0.071 |  |  |  |  |  |  |  |
|  | IVW | 26 | 0.903 | 0.817 | 0.998 | 0.045 |  |  |  |  |  |  |  |
|  | Simple mode | 26 | 0.869 | 0.648 | 1.167 | 0.360 |  |  |  |  |  |  |  |
|  | Weighted mode | 26 | 0.779 | 0.596 | 1.019 | 0.080 |  |  |  |  |  |  |  |
|  | BWMR | 26 | 0.943 | 0.864 | 1.029 | 0.185 |  |  |  |  |  |  |  |
| Phosphatidylinositol (18:0_20:3) | MR Egger | 33 | 1.105 | 0.945 | 1.293 | 0.220 | 52.589 | 0.009 | 52.715 | 0.012 | -0.003 | 0.787 | 0.009 |
|  | Weighted median | 33 | 1.128 | 1.034 | 1.230 | 0.006 |  |  |  |  |  |  |  |
|  | IVW | 33 | 1.084 | 1.006 | 1.168 | 0.033 |  |  |  |  |  |  |  |
|  | Simple mode | 33 | 1.014 | 0.818 | 1.257 | 0.902 |  |  |  |  |  |  |  |
|  | Weighted mode | 33 | 1.174 | 1.069 | 1.288 | 0.002 |  |  |  |  |  |  |  |
|  | BWMR | 33 | 1.065 | 0.982 | 1.155 | 0.130 |  |  |  |  |  |  |  |
| Phosphatidylinositol (18:1_18:1) | MR Egger | 23 | 1.029 | 0.829 | 1.278 | 0.795 | 26.964 | 0.172 | 27.517 | 0.192 | 0.009 | 0.519 | 0.168 |
|  | Weighted median | 23 | 1.077 | 0.955 | 1.214 | 0.225 |  |  |  |  |  |  |  |
|  | IVW | 23 | 1.099 | 1.005 | 1.202 | 0.038 |  |  |  |  |  |  |  |
|  | Simple mode | 23 | 1.036 | 0.829 | 1.294 | 0.760 |  |  |  |  |  |  |  |
|  | Weighted mode | 23 | 1.055 | 0.861 | 1.292 | 0.613 |  |  |  |  |  |  |  |
|  | BWMR | 23 | 1.090 | 0.990 | 1.200 | 0.080 |  |  |  |  |  |  |  |
| Sphingomyelin (d36:1) | MR Egger | 35 | 0.986 | 0.829 | 1.174 | 0.876 | 37.565 | 0.268 | 38.777 | 0.263 | -0.012 | 0.310 | 0.298 |
|  | Weighted median | 35 | 0.873 | 0.786 | 0.969 | 0.011 |  |  |  |  |  |  |  |
|  | IVW | 35 | 0.908 | 0.843 | 0.977 | 0.010 |  |  |  |  |  |  |  |
|  | Simple mode | 35 | 0.882 | 0.730 | 1.066 | 0.202 |  |  |  |  |  |  |  |
|  | Weighted mode | 35 | 0.885 | 0.769 | 1.019 | 0.098 |  |  |  |  |  |  |  |
|  | BWMR | 35 | 0.907 | 0.837 | 0.983 | 0.018 |  |  |  |  |  |  |  |
| Sphingomyelin (d38:1) | MR Egger | 38 | 0.944 | 0.844 | 1.055 | 0.315 | 31.896 | 0.664 | 32.513 | 0.679 | -0.007 | 0.437 | 0.693 |
|  | Weighted median | 38 | 0.916 | 0.841 | 0.997 | 0.043 |  |  |  |  |  |  |  |
|  | IVW | 38 | 0.908 | 0.857 | 0.962 | 0.001 |  |  |  |  |  |  |  |
|  | Simple mode | 38 | 0.853 | 0.736 | 0.988 | 0.041 |  |  |  |  |  |  |  |
|  | Weighted mode | 38 | 0.928 | 0.832 | 1.035 | 0.187 |  |  |  |  |  |  |  |
|  | BWMR | 38 | 0.906 | 0.854 | 0.962 | 0.001 |  |  |  |  |  |  |  |
| Sphingomyelin (d38:2) | MR Egger | 31 | 1.072 | 0.926 | 1.241 | 0.358 | 33.084 | 0.274 | 41.582 | 0.078 | -0.034 | 0.011 | 0.073 |
|  | Weighted median | 31 | 0.902 | 0.811 | 1.002 | 0.054 |  |  |  |  |  |  |  |
|  | IVW | 31 | 0.896 | 0.830 | 0.968 | 0.005 |  |  |  |  |  |  |  |
|  | Simple mode | 31 | 0.923 | 0.757 | 1.125 | 0.435 |  |  |  |  |  |  |  |
|  | Weighted mode | 31 | 0.917 | 0.805 | 1.045 | 0.206 |  |  |  |  |  |  |  |
|  | BWMR | 31 | 0.906 | 0.837 | 0.981 | 0.015 |  |  |  |  |  |  |  |
| Sphingomyelin (d40:2) | MR Egger | 36 | 1.055 | 0.894 | 1.245 | 0.530 | 51.488 | 0.028 | 57.274 | 0.010 | -0.023 | 0.059 | 0.009 |
|  | Weighted median | 36 | 0.934 | 0.839 | 1.038 | 0.204 |  |  |  |  |  |  |  |
|  | IVW | 36 | 0.914 | 0.839 | 0.995 | 0.037 |  |  |  |  |  |  |  |
|  | Simple mode | 36 | 0.846 | 0.680 | 1.052 | 0.142 |  |  |  |  |  |  |  |
|  | Weighted mode | 36 | 0.929 | 0.812 | 1.062 | 0.288 |  |  |  |  |  |  |  |
|  | BWMR | 36 | 0.922 | 0.848 | 1.003 | 0.059 |  |  |  |  |  |  |  |
| Sphingomyelin (d42:2) | MR Egger | 38 | 0.954 | 0.842 | 1.082 | 0.469 | 30.838 | 0.712 | 31.113 | 0.741 | -0.004 | 0.603 | 0.718 |
|  | Weighted median | 38 | 0.884 | 0.805 | 0.972 | 0.010 |  |  |  |  |  |  |  |
|  | IVW | 38 | 0.927 | 0.869 | 0.990 | 0.024 |  |  |  |  |  |  |  |
|  | Simple mode | 38 | 0.841 | 0.700 | 1.011 | 0.073 |  |  |  |  |  |  |  |
|  | Weighted mode | 38 | 0.850 | 0.735 | 0.984 | 0.036 |  |  |  |  |  |  |  |
|  | BWMR | 38 | 0.926 | 0.865 | 0.991 | 0.026 |  |  |  |  |  |  |  |
| Triacylglycerol (56:7) | MR Egger | 33 | 0.758 | 0.610 | 0.942 | 0.018 | 44.499 | 0.055 | 48.776 | 0.029 | 0.024 | 0.094 | 0.022 |
|  | Weighted median | 33 | 0.888 | 0.790 | 0.998 | 0.047 |  |  |  |  |  |  |  |
|  | IVW | 33 | 0.903 | 0.826 | 0.987 | 0.025 |  |  |  |  |  |  |  |
|  | Simple mode | 33 | 0.895 | 0.723 | 1.107 | 0.313 |  |  |  |  |  |  |  |
|  | Weighted mode | 33 | 0.895 | 0.729 | 1.098 | 0.295 |  |  |  |  |  |  |  |
|  | BWMR | 33 | 0.902 | 0.821 | 0.992 | 0.033 |  |  |  |  |  |  |  |
| **CD** |  |  |  |  |  |  |  |  |  |  |  |  |  |
| Sterol ester (27:1/20:4) | MR Egger | 31 | 0.878 | 0.773 | 0.998 | 0.056 | 37.509 | 0.134 | 37.868 | 0.153 | 0.008 | 0.602 | 0.562 |
|  | Weighted median | 31 | 0.886 | 0.807 | 0.972 | 0.011 |  |  |  |  |  |  |  |
|  | IVW | 31 | 0.901 | 0.827 | 0.981 | 0.017 |  |  |  |  |  |  |  |
|  | Simple mode | 31 | 0.838 | 0.649 | 1.081 | 0.184 |  |  |  |  |  |  |  |
|  | Weighted mode | 31 | 0.881 | 0.802 | 0.968 | 0.013 |  |  |  |  |  |  |  |
|  | BWMR | 31 | 0.903 | 0.824 | 0.989 | 0.028 |  |  |  |  |  |  |  |
| Sterol ester (27:1/22:6) | MR Egger | 23 | 0.870 | 0.624 | 1.213 | 0.421 | 17.834 | 0.659 | 17.864 | 0.714 | -0.004 | 0.865 | 0.692 |
|  | Weighted median | 23 | 0.816 | 0.666 | 1.000 | 0.050 |  |  |  |  |  |  |  |
|  | IVW | 23 | 0.848 | 0.736 | 0.976 | 0.022 |  |  |  |  |  |  |  |
|  | Simple mode | 23 | 1.070 | 0.725 | 1.579 | 0.736 |  |  |  |  |  |  |  |
|  | Weighted mode | 23 | 0.716 | 0.524 | 0.979 | 0.048 |  |  |  |  |  |  |  |
|  | BWMR | 23 | 0.843 | 0.727 | 0.978 | 0.025 |  |  |  |  |  |  |  |
| Diacylglycerol (16:1_18:1) | MR Egger | 22 | 1.148 | 0.720 | 1.831 | 0.569 | 34.518 | 0.023 | 35.050 | 0.028 | 0.019 | 0.585 | 0.025 |
|  | Weighted median | 22 | 1.099 | 0.871 | 1.389 | 0.426 |  |  |  |  |  |  |  |
|  | IVW | 22 | 1.294 | 1.063 | 1.574 | 0.010 |  |  |  |  |  |  |  |
|  | Simple mode | 22 | 1.617 | 1.011 | 2.585 | 0.058 |  |  |  |  |  |  |  |
|  | Weighted mode | 22 | 1.010 | 0.632 | 1.614 | 0.966 |  |  |  |  |  |  |  |
|  | BWMR | 22 | 1.276 | 1.046 | 1.556 | 0.016 |  |  |  |  |  |  |  |
| Phosphatidylcholine (20:4_0:0) | MR Egger | 27 | 0.835 | 0.724 | 0.962 | 0.020 | 22.100 | 0.630 | 24.164 | 0.567 | 0.020 | 0.163 | 0.541 |
|  | Weighted median | 27 | 0.872 | 0.781 | 0.973 | 0.015 |  |  |  |  |  |  |  |
|  | IVW | 27 | 0.905 | 0.828 | 0.989 | 0.028 |  |  |  |  |  |  |  |
|  | Simple mode | 27 | 0.918 | 0.688 | 1.224 | 0.563 |  |  |  |  |  |  |  |
|  | Weighted mode | 27 | 0.865 | 0.771 | 0.971 | 0.021 |  |  |  |  |  |  |  |
|  | BWMR | 27 | 0.897 | 0.810 | 0.993 | 0.036 |  |  |  |  |  |  |  |
| Phosphatidylcholine (16:0_20:4) | MR Egger | 24 | 0.915 | 0.803 | 1.043 | 0.196 | 21.650 | 0.481 | 22.494 | 0.491 | -0.014 | 0.368 | 0.632 |
|  | Weighted median | 24 | 0.877 | 0.792 | 0.971 | 0.012 |  |  |  |  |  |  |  |
|  | IVW | 24 | 0.874 | 0.802 | 0.953 | 0.002 |  |  |  |  |  |  |  |
|  | Simple mode | 24 | 0.856 | 0.623 | 1.177 | 0.350 |  |  |  |  |  |  |  |
|  | Weighted mode | 24 | 0.877 | 0.800 | 0.963 | 0.011 |  |  |  |  |  |  |  |
|  | BWMR | 24 | 0.872 | 0.801 | 0.950 | 0.002 |  |  |  |  |  |  |  |
| Phosphatidylcholine (17:0_18:1) | MR Egger | 20 | 0.739 | 0.521 | 1.048 | 0.107 | 7.660 | 0.983 | 8.273 | 0.984 | 0.019 | 0.444 | 0.982 |
|  | Weighted median | 20 | 0.854 | 0.691 | 1.056 | 0.146 |  |  |  |  |  |  |  |
|  | IVW | 20 | 0.836 | 0.708 | 0.986 | 0.034 |  |  |  |  |  |  |  |
|  | Simple mode | 20 | 0.857 | 0.583 | 1.259 | 0.440 |  |  |  |  |  |  |  |
|  | Weighted mode | 20 | 0.862 | 0.616 | 1.207 | 0.399 |  |  |  |  |  |  |  |
|  | BWMR | 20 | 0.835 | 0.701 | 0.994 | 0.043 |  |  |  |  |  |  |  |
| Phosphatidylcholine (17:0_18:2) | MR Egger | 25 | 1.414 | 1.001 | 1.998 | 0.062 | 26.040 | 0.299 | 27.718 | 0.272 | -0.029 | 0.236 | 0.239 |
|  | Weighted median | 25 | 1.195 | 0.982 | 1.454 | 0.076 |  |  |  |  |  |  |  |
|  | IVW | 25 | 1.162 | 1.010 | 1.337 | 0.036 |  |  |  |  |  |  |  |
|  | Simple mode | 25 | 1.024 | 0.692 | 1.515 | 0.908 |  |  |  |  |  |  |  |
|  | Weighted mode | 25 | 1.314 | 1.042 | 1.657 | 0.030 |  |  |  |  |  |  |  |
|  | BWMR | 25 | 1.167 | 1.006 | 1.355 | 0.041 |  |  |  |  |  |  |  |
| Phosphatidylcholine (18:0_20:4) | MR Egger | 27 | 0.894 | 0.768 | 1.041 | 0.160 | 47.597 | 0.004 | 47.597 | 0.006 | 0.000 | 0.999 | 0.033 |
|  | Weighted median | 27 | 0.888 | 0.814 | 0.968 | 0.007 |  |  |  |  |  |  |  |
|  | IVW | 27 | 0.894 | 0.806 | 0.991 | 0.033 |  |  |  |  |  |  |  |
|  | Simple mode | 27 | 0.796 | 0.606 | 1.044 | 0.111 |  |  |  |  |  |  |  |
|  | Weighted mode | 27 | 0.885 | 0.808 | 0.969 | 0.014 |  |  |  |  |  |  |  |
|  | BWMR | 27 | 0.900 | 0.808 | 1.002 | 0.055 |  |  |  |  |  |  |  |
| Phosphatidylcholine (18:1_20:2) | MR Egger | 23 | 1.216 | 0.990 | 1.493 | 0.077 | 23.989 | 0.294 | 24.984 | 0.298 | -0.020 | 0.361 | 0.304 |
|  | Weighted median | 23 | 1.195 | 1.043 | 1.370 | 0.011 |  |  |  |  |  |  |  |
|  | IVW | 23 | 1.119 | 1.003 | 1.248 | 0.043 |  |  |  |  |  |  |  |
|  | Simple mode | 23 | 1.031 | 0.751 | 1.416 | 0.851 |  |  |  |  |  |  |  |
|  | Weighted mode | 23 | 1.191 | 1.040 | 1.363 | 0.019 |  |  |  |  |  |  |  |
|  | BWMR | 23 | 1.116 | 0.984 | 1.265 | 0.086 |  |  |  |  |  |  |  |
| Phosphatidylcholine (18:1_20:4) | MR Egger | 28 | 0.829 | 0.700 | 0.981 | 0.038 | 34.610 | 0.120 | 34.928 | 0.141 | 0.007 | 0.629 | 0.214 |
|  | Weighted median | 28 | 0.856 | 0.761 | 0.963 | 0.010 |  |  |  |  |  |  |  |
|  | IVW | 28 | 0.856 | 0.771 | 0.951 | 0.004 |  |  |  |  |  |  |  |
|  | Simple mode | 28 | 0.917 | 0.680 | 1.235 | 0.572 |  |  |  |  |  |  |  |
|  | Weighted mode | 28 | 0.859 | 0.766 | 0.962 | 0.014 |  |  |  |  |  |  |  |
|  | BWMR | 28 | 0.850 | 0.773 | 0.935 | 0.001 |  |  |  |  |  |  |  |
| Phosphatidylcholine (O-18:0_20:4) | MR Egger | 21 | 0.708 | 0.510 | 0.982 | 0.052 | 30.737 | 0.043 | 32.492 | 0.038 | 0.026 | 0.311 | 0.064 |
|  | Weighted median | 21 | 0.775 | 0.640 | 0.939 | 0.009 |  |  |  |  |  |  |  |
|  | IVW | 21 | 0.822 | 0.697 | 0.970 | 0.021 |  |  |  |  |  |  |  |
|  | Simple mode | 21 | 0.777 | 0.542 | 1.112 | 0.182 |  |  |  |  |  |  |  |
|  | Weighted mode | 21 | 0.777 | 0.647 | 0.932 | 0.013 |  |  |  |  |  |  |  |
|  | BWMR | 21 | 0.814 | 0.680 | 0.973 | 0.024 |  |  |  |  |  |  |  |
| Phosphatidylethanolamine (O-18:1_20:4) | MR Egger | 28 | 0.778 | 0.584 | 1.036 | 0.098 | 24.555 | 0.544 | 24.726 | 0.590 | 0.008 | 0.683 | 0.587 |
|  | Weighted median | 28 | 0.750 | 0.613 | 0.919 | 0.005 |  |  |  |  |  |  |  |
|  | IVW | 28 | 0.821 | 0.721 | 0.934 | 0.003 |  |  |  |  |  |  |  |
|  | Simple mode | 28 | 0.790 | 0.533 | 1.172 | 0.251 |  |  |  |  |  |  |  |
|  | Weighted mode | 28 | 0.749 | 0.583 | 0.964 | 0.033 |  |  |  |  |  |  |  |
|  | BWMR | 28 | 0.812 | 0.711 | 0.927 | 0.002 |  |  |  |  |  |  |  |
| Sphingomyelin (d42:2) | MR Egger | 38 | 0.912 | 0.735 | 1.130 | 0.405 | 36.863 | 0.429 | 36.993 | 0.469 | -0.005 | 0.724 | 0.478 |
|  | Weighted median | 38 | 0.915 | 0.777 | 1.078 | 0.289 |  |  |  |  |  |  |  |
|  | IVW | 38 | 0.882 | 0.789 | 0.985 | 0.027 |  |  |  |  |  |  |  |
|  | Simple mode | 38 | 1.033 | 0.740 | 1.444 | 0.849 |  |  |  |  |  |  |  |
|  | Weighted mode | 38 | 1.020 | 0.772 | 1.349 | 0.888 |  |  |  |  |  |  |  |
|  | BWMR | 38 | 0.879 | 0.783 | 0.988 | 0.030 |  |  |  |  |  |  |  |
| Triacylglycerol (52:2) | MR Egger | 24 | 1.278 | 0.857 | 1.905 | 0.242 | 33.016 | 0.062 | 33.139 | 0.079 | -0.008 | 0.777 | 0.075 |
|  | Weighted median | 24 | 1.000 | 0.801 | 1.248 | 0.997 |  |  |  |  |  |  |  |
|  | IVW | 24 | 1.212 | 1.021 | 1.440 | 0.028 |  |  |  |  |  |  |  |
|  | Simple mode | 24 | 0.968 | 0.638 | 1.469 | 0.881 |  |  |  |  |  |  |  |
|  | Weighted mode | 24 | 0.968 | 0.721 | 1.301 | 0.833 |  |  |  |  |  |  |  |
|  | BWMR | 24 | 1.216 | 1.009 | 1.466 | 0.040 |  |  |  |  |  |  |  |
| Triacylglycerol (52:3) | MR Egger | 30 | 1.115 | 0.780 | 1.593 | 0.555 | 43.953 | 0.028 | 44.755 | 0.031 | 0.017 | 0.481 | 0.028 |
|  | Weighted median | 30 | 0.997 | 0.820 | 1.212 | 0.974 |  |  |  |  |  |  |  |
|  | IVW | 30 | 1.254 | 1.075 | 1.462 | 0.004 |  |  |  |  |  |  |  |
|  | Simple mode | 30 | 0.971 | 0.677 | 1.391 | 0.872 |  |  |  |  |  |  |  |
|  | Weighted mode | 30 | 0.976 | 0.746 | 1.276 | 0.859 |  |  |  |  |  |  |  |
|  | BWMR | 30 | 1.251 | 1.065 | 1.470 | 0.006 |  |  |  |  |  |  |  |
| Triacylglycerol (52:4) | MR Egger | 26 | 1.068 | 0.701 | 1.627 | 0.762 | 37.818 | 0.036 | 38.358 | 0.043 | 0.016 | 0.564 | 0.035 |
|  | Weighted median | 26 | 0.997 | 0.818 | 1.216 | 0.979 |  |  |  |  |  |  |  |
|  | IVW | 26 | 1.199 | 1.019 | 1.411 | 0.029 |  |  |  |  |  |  |  |
|  | Simple mode | 26 | 0.963 | 0.637 | 1.456 | 0.860 |  |  |  |  |  |  |  |
|  | Weighted mode | 26 | 0.974 | 0.736 | 1.288 | 0.854 |  |  |  |  |  |  |  |
|  | BWMR | 26 | 1.186 | 1.003 | 1.404 | 0.046 |  |  |  |  |  |  |  |
| Triacylglycerol (52:5) | MR Egger | 27 | 1.152 | 0.715 | 1.857 | 0.566 | 45.268 | 0.008 | 45.443 | 0.011 | 0.009 | 0.758 | 0.006 |
|  | Weighted median | 27 | 1.164 | 0.940 | 1.441 | 0.164 |  |  |  |  |  |  |  |
|  | IVW | 27 | 1.236 | 1.036 | 1.474 | 0.018 |  |  |  |  |  |  |  |
|  | Simple mode | 27 | 1.274 | 0.813 | 1.997 | 0.300 |  |  |  |  |  |  |  |
|  | Weighted mode | 27 | 1.022 | 0.719 | 1.453 | 0.905 |  |  |  |  |  |  |  |
|  | BWMR | 27 | 1.210 | 1.007 | 1.454 | 0.041 |  |  |  |  |  |  |  |
| Triacylglycerol (53:3) | MR Egger | 25 | 1.165 | 0.735 | 1.847 | 0.522 | 43.953 | 0.005 | 44.250 | 0.007 | 0.012 | 0.697 | 0.006 |
|  | Weighted median | 25 | 1.006 | 0.811 | 1.248 | 0.954 |  |  |  |  |  |  |  |
|  | IVW | 25 | 1.268 | 1.059 | 1.519 | 0.010 |  |  |  |  |  |  |  |
|  | Simple mode | 25 | 0.937 | 0.549 | 1.600 | 0.815 |  |  |  |  |  |  |  |
|  | Weighted mode | 25 | 0.963 | 0.721 | 1.288 | 0.803 |  |  |  |  |  |  |  |
|  | BWMR | 25 | 1.256 | 1.047 | 1.507 | 0.014 |  |  |  |  |  |  |  |
| Triacylglycerol (54:4) | MR Egger | 27 | 1.233 | 0.785 | 1.936 | 0.372 | 46.257 | 0.006 | 46.287 | 0.008 | -0.004 | 0.900 | 0.065 |
|  | Weighted median | 27 | 1.035 | 0.853 | 1.255 | 0.728 |  |  |  |  |  |  |  |
|  | IVW | 27 | 1.200 | 1.009 | 1.428 | 0.039 |  |  |  |  |  |  |  |
|  | Simple mode | 27 | 1.014 | 0.685 | 1.500 | 0.947 |  |  |  |  |  |  |  |
|  | Weighted mode | 27 | 1.007 | 0.783 | 1.294 | 0.959 |  |  |  |  |  |  |  |
|  | BWMR | 27 | 1.212 | 1.014 | 1.448 | 0.034 |  |  |  |  |  |  |  |
| Triacylglycerol (54:5) | MR Egger | 27 | 1.088 | 0.699 | 1.693 | 0.712 | 45.165 | 0.008 | 45.780 | 0.010 | 0.017 | 0.565 | 0.010 |
|  | Weighted median | 27 | 1.131 | 0.914 | 1.400 | 0.257 |  |  |  |  |  |  |  |
|  | IVW | 27 | 1.226 | 1.023 | 1.471 | 0.028 |  |  |  |  |  |  |  |
|  | Simple mode | 27 | 1.215 | 0.775 | 1.904 | 0.404 |  |  |  |  |  |  |  |
|  | Weighted mode | 27 | 1.018 | 0.763 | 1.358 | 0.903 |  |  |  |  |  |  |  |
|  | BWMR | 27 | 1.211 | 1.005 | 1.458 | 0.044 |  |  |  |  |  |  |  |
| Abbreviations: IVW: inverse variance weighted; BWMR: Bayesian Weighted Mendelian Randomization; β:beta; OR: odds ratio; IBD: inflammatory bowel disease; UC: ulcerative colitis; CD: Crohn's disease. | | | | | | | | | | | | |  |

| **Supplementary Table S7. Association of genome-wide SNPs for lipidome with IBD** | | | | | | | |
| --- | --- | --- | --- | --- | --- | --- | --- |
| **Plasma lipidome** | **SNP** | **Beta outcome** | **EAF outcome** | **SE outcome** | **P outcome** | **R**^2^ | **F-statistic** |
| **IBD** |  |  |  |  |  |  |  |
| Sterol ester (27:1/20:3) | rs111575978 | 0.009 | 0.028 | 0.046 | 0.844 | 0.003 | 24.019 |
|  | rs112478356 | 0.046 | 0.017 | 0.062 | 0.453 | 0.003 | 22.359 |
|  | rs1135999 | 0.023 | 0.315 | 0.016 | 0.167 | 0.022 | 160.701 |
|  | rs116917626 | 0.039 | 0.022 | 0.052 | 0.453 | 0.003 | 20.622 |
|  | rs117079226 | -0.223 | 0.011 | 0.077 | 0.004 | 0.003 | 23.224 |
|  | rs117754141 | -0.036 | 0.021 | 0.053 | 0.496 | 0.003 | 23.741 |
|  | rs1428498 | -0.014 | 0.351 | 0.016 | 0.370 | 0.003 | 22.210 |
|  | rs1800961 | -0.141 | 0.045 | 0.038 | 0.000 | 0.006 | 41.072 |
|  | rs182695896 | -0.001 | 0.026 | 0.048 | 0.977 | 0.004 | 28.855 |
|  | rs187429064 | 0.048 | 0.051 | 0.034 | 0.164 | 0.005 | 38.802 |
|  | rs199568207 | -0.045 | 0.042 | 0.038 | 0.231 | 0.003 | 21.852 |
|  | rs2352991 | 0.005 | 0.738 | 0.017 | 0.775 | 0.003 | 21.486 |
|  | rs2727271 | 0.076 | 0.268 | 0.017 | 0.000 | 0.011 | 80.303 |
|  | rs3752149 | 0.018 | 0.270 | 0.017 | 0.294 | 0.003 | 22.400 |
|  | rs4977342 | -0.010 | 0.245 | 0.018 | 0.556 | 0.003 | 22.221 |
|  | rs56374730 | -0.001 | 0.303 | 0.016 | 0.928 | 0.005 | 37.145 |
|  | rs570929 | -0.035 | 0.551 | 0.015 | 0.020 | 0.003 | 24.021 |
|  | rs62217148 | 0.057 | 0.047 | 0.037 | 0.120 | 0.003 | 23.267 |
|  | rs624605 | -0.004 | 0.439 | 0.015 | 0.769 | 0.003 | 23.385 |
|  | rs6761859 | 0.022 | 0.026 | 0.047 | 0.641 | 0.003 | 20.214 |
|  | rs685850 | -0.024 | 0.566 | 0.015 | 0.119 | 0.003 | 24.648 |
|  | rs7020 | -0.030 | 0.449 | 0.015 | 0.052 | 0.004 | 25.435 |
|  | rs7036889 | -0.013 | 0.096 | 0.026 | 0.619 | 0.003 | 20.724 |
|  | rs72660594 | 0.062 | 0.044 | 0.037 | 0.092 | 0.005 | 34.525 |
|  | rs7412 | -0.010 | 0.053 | 0.034 | 0.780 | 0.004 | 29.519 |
|  | rs77645768 | -0.024 | 0.027 | 0.047 | 0.611 | 0.004 | 25.555 |
|  | rs79047909 | 0.049 | 0.007 | 0.091 | 0.586 | 0.003 | 23.347 |
|  | rs79146711 | -0.037 | 0.043 | 0.038 | 0.329 | 0.003 | 20.697 |
|  | rs79771052 | -0.052 | 0.012 | 0.072 | 0.471 | 0.003 | 23.537 |
|  | rs80325645 | -0.014 | 0.008 | 0.089 | 0.872 | 0.003 | 23.145 |
|  | rs9310455 | 0.017 | 0.061 | 0.032 | 0.584 | 0.003 | 20.517 |
| Sterol ester (27:1/20:4) | rs11022403 | -0.021 | 0.291 | 0.017 | 0.210 | 0.003 | 21.220 |
|  | rs113248417 | -0.002 | 0.039 | 0.039 | 0.952 | 0.005 | 35.544 |
|  | rs1135999 | 0.023 | 0.315 | 0.016 | 0.167 | 0.005 | 32.506 |
|  | rs113603971 | -0.001 | 0.051 | 0.034 | 0.984 | 0.003 | 19.555 |
|  | rs11591147 | 0.084 | 0.036 | 0.040 | 0.038 | 0.006 | 41.421 |
|  | rs117084735 | -0.031 | 0.016 | 0.061 | 0.605 | 0.003 | 20.353 |
|  | rs117186386 | 0.007 | 0.048 | 0.036 | 0.849 | 0.004 | 25.459 |
|  | rs117825513 | -0.086 | 0.012 | 0.068 | 0.208 | 0.003 | 20.318 |
|  | rs138482835 | -0.025 | 0.052 | 0.035 | 0.472 | 0.003 | 22.278 |
|  | rs147981159 | -0.001 | 0.044 | 0.037 | 0.972 | 0.008 | 60.908 |
|  | rs174528 | 0.071 | 0.420 | 0.015 | 0.000 | 0.202 | 1819.849 |
|  | rs1800961 | -0.141 | 0.045 | 0.038 | 0.000 | 0.003 | 20.532 |
|  | rs182695896 | -0.001 | 0.026 | 0.048 | 0.977 | 0.004 | 31.741 |
|  | rs186913978 | 0.023 | 0.009 | 0.082 | 0.776 | 0.004 | 28.720 |
|  | rs187429064 | 0.048 | 0.051 | 0.034 | 0.164 | 0.006 | 46.088 |
|  | rs2229738 | 0.047 | 0.155 | 0.021 | 0.025 | 0.006 | 42.633 |
|  | rs223841 | 0.043 | 0.169 | 0.020 | 0.034 | 0.003 | 21.183 |
|  | rs2469034 | 0.004 | 0.797 | 0.019 | 0.824 | 0.003 | 20.122 |
|  | rs3741252 | -0.062 | 0.129 | 0.023 | 0.007 | 0.010 | 74.744 |
|  | rs3767941 | 0.032 | 0.173 | 0.020 | 0.105 | 0.003 | 22.617 |
|  | rs3820438 | 0.016 | 0.288 | 0.017 | 0.353 | 0.003 | 21.528 |
|  | rs4382917 | -0.012 | 0.243 | 0.018 | 0.482 | 0.008 | 55.143 |
|  | rs635196 | -0.013 | 0.177 | 0.020 | 0.517 | 0.003 | 23.862 |
|  | rs646299 | 0.014 | 0.606 | 0.016 | 0.384 | 0.003 | 19.672 |
|  | rs6812370 | 0.107 | 0.014 | 0.064 | 0.093 | 0.004 | 27.573 |
|  | rs6860806 | 0.074 | 0.417 | 0.015 | 0.000 | 0.003 | 20.015 |
|  | rs7020 | -0.030 | 0.449 | 0.015 | 0.052 | 0.003 | 19.900 |
|  | rs7412 | -0.010 | 0.053 | 0.034 | 0.780 | 0.010 | 70.977 |
|  | rs74915447 | -0.023 | 0.036 | 0.041 | 0.579 | 0.003 | 20.006 |
|  | rs7837272 | -0.102 | 0.002 | 0.178 | 0.567 | 0.003 | 21.080 |
|  | rs79136768 | 0.057 | 0.036 | 0.041 | 0.160 | 0.003 | 24.145 |
|  | rs9388335 | 0.024 | 0.525 | 0.015 | 0.118 | 0.003 | 21.634 |
|  | rs9555673 | 0.026 | 0.424 | 0.015 | 0.086 | 0.003 | 20.745 |
| Sterol ester (27:1/20:5) | rs10860778 | -0.012 | 0.476 | 0.015 | 0.443 | 0.004 | 26.149 |
|  | rs11986253 | 0.019 | 0.180 | 0.020 | 0.336 | 0.003 | 21.842 |
|  | rs12515163 | -0.025 | 0.415 | 0.016 | 0.106 | 0.003 | 21.714 |
|  | rs143678376 | 0.012 | 0.070 | 0.030 | 0.700 | 0.003 | 23.739 |
|  | rs1481977 | 0.001 | 0.859 | 0.022 | 0.970 | 0.003 | 21.392 |
|  | rs174527 | 0.017 | 0.452 | 0.015 | 0.268 | 0.008 | 57.173 |
|  | rs174581 | 0.074 | 0.415 | 0.015 | 0.000 | 0.081 | 629.075 |
|  | rs17548303 | 0.011 | 0.258 | 0.017 | 0.527 | 0.003 | 20.434 |
|  | rs182611493 | 0.048 | 0.051 | 0.034 | 0.168 | 0.003 | 22.751 |
|  | rs2054498 | -0.009 | 0.538 | 0.015 | 0.558 | 0.004 | 26.371 |
|  | rs2625222 | -0.019 | 0.109 | 0.024 | 0.429 | 0.003 | 21.369 |
|  | rs3019200 | 0.023 | 0.808 | 0.019 | 0.240 | 0.003 | 22.293 |
|  | rs34343838 | -0.033 | 0.123 | 0.023 | 0.149 | 0.003 | 23.409 |
|  | rs4490551 | -0.008 | 0.198 | 0.019 | 0.666 | 0.003 | 19.681 |
|  | rs449647 | -0.009 | 0.134 | 0.022 | 0.700 | 0.004 | 28.288 |
|  | rs4908782 | -0.010 | 0.346 | 0.016 | 0.545 | 0.003 | 20.648 |
|  | rs508049 | 0.020 | 0.093 | 0.026 | 0.447 | 0.004 | 30.551 |
|  | rs5896690 | 0.062 | 0.465 | 0.015 | 0.000 | 0.003 | 20.687 |
|  | rs59807465 | -0.066 | 0.019 | 0.056 | 0.236 | 0.003 | 19.995 |
|  | rs62025936 | -0.009 | 0.156 | 0.021 | 0.655 | 0.003 | 22.008 |
|  | rs71629271 | 0.003 | 0.069 | 0.030 | 0.921 | 0.003 | 19.874 |
|  | rs75897070 | -0.017 | 0.051 | 0.035 | 0.628 | 0.003 | 19.816 |
|  | rs7780677 | -0.009 | 0.410 | 0.015 | 0.543 | 0.003 | 20.158 |
|  | rs7936002 | -0.015 | 0.245 | 0.018 | 0.411 | 0.004 | 31.095 |
|  | rs7947954 | 0.034 | 0.153 | 0.021 | 0.106 | 0.003 | 23.121 |
|  | rs8132104 | 0.027 | 0.264 | 0.017 | 0.119 | 0.003 | 20.221 |
|  | rs980814 | -0.008 | 0.449 | 0.015 | 0.622 | 0.003 | 21.520 |
| Sterol ester (27:1/22:6) | rs10150075 | 0.005 | 0.061 | 0.032 | 0.871 | 0.004 | 28.636 |
|  | rs111751059 | -0.003 | 0.063 | 0.031 | 0.936 | 0.003 | 20.167 |
|  | rs113651041 | -0.020 | 0.380 | 0.016 | 0.197 | 0.003 | 19.538 |
|  | rs115647337 | -0.008 | 0.042 | 0.038 | 0.823 | 0.003 | 21.698 |
|  | rs11591147 | 0.084 | 0.036 | 0.040 | 0.038 | 0.004 | 25.783 |
|  | rs11633878 | 0.003 | 0.717 | 0.017 | 0.867 | 0.003 | 22.651 |
|  | rs116794590 | 0.014 | 0.024 | 0.049 | 0.771 | 0.003 | 21.674 |
|  | rs11696517 | 0.030 | 0.164 | 0.020 | 0.141 | 0.003 | 19.916 |
|  | rs117061362 | 0.063 | 0.048 | 0.036 | 0.080 | 0.003 | 23.519 |
|  | rs149229417 | 0.003 | 0.017 | 0.059 | 0.965 | 0.003 | 23.251 |
|  | rs17110517 | -0.009 | 0.057 | 0.033 | 0.789 | 0.003 | 24.431 |
|  | rs2075083 | -0.022 | 0.566 | 0.015 | 0.157 | 0.003 | 19.647 |
|  | rs2229738 | 0.047 | 0.155 | 0.021 | 0.025 | 0.003 | 23.485 |
|  | rs35473591 | 0.071 | 0.412 | 0.015 | 0.000 | 0.019 | 141.775 |
|  | rs4072727 | -0.002 | 0.369 | 0.016 | 0.883 | 0.004 | 29.085 |
|  | rs72657952 | -0.034 | 0.022 | 0.052 | 0.510 | 0.003 | 19.945 |
|  | rs72852391 | -0.010 | 0.040 | 0.039 | 0.793 | 0.003 | 22.458 |
|  | rs73769662 | 0.004 | 0.093 | 0.026 | 0.875 | 0.003 | 19.666 |
|  | rs7412 | -0.010 | 0.053 | 0.034 | 0.780 | 0.007 | 52.155 |
|  | rs75101588 | -0.009 | 0.029 | 0.045 | 0.834 | 0.004 | 26.866 |
|  | rs77456635 | -0.032 | 0.027 | 0.047 | 0.502 | 0.003 | 25.055 |
|  | rs77708260 | -0.010 | 0.026 | 0.047 | 0.839 | 0.003 | 20.104 |
|  | rs9559135 | -0.012 | 0.282 | 0.017 | 0.492 | 0.003 | 21.738 |
| Phosphatidylcholine (20:4_0:0) | rs10733608 | -0.004 | 0.478 | 0.015 | 0.775 | 0.003 | 21.648 |
|  | rs10884272 | -0.003 | 0.367 | 0.016 | 0.832 | 0.003 | 20.372 |
|  | rs11669519 | -0.016 | 0.343 | 0.016 | 0.302 | 0.003 | 20.381 |
|  | rs12226389 | 0.012 | 0.178 | 0.020 | 0.562 | 0.009 | 54.075 |
|  | rs12303 | 0.008 | 0.199 | 0.019 | 0.668 | 0.003 | 21.040 |
|  | rs12905466 | -0.003 | 0.201 | 0.019 | 0.863 | 0.003 | 20.862 |
|  | rs1363011 | -0.022 | 0.138 | 0.022 | 0.309 | 0.003 | 19.537 |
|  | rs139516883 | 0.001 | 0.014 | 0.065 | 0.991 | 0.004 | 24.495 |
|  | rs142999489 | 0.027 | 0.056 | 0.033 | 0.411 | 0.004 | 23.796 |
|  | rs150158785 | -0.014 | 0.079 | 0.028 | 0.622 | 0.003 | 20.086 |
|  | rs182611493 | 0.048 | 0.051 | 0.034 | 0.168 | 0.004 | 26.444 |
|  | rs191895023 | -0.030 | 0.003 | 0.154 | 0.848 | 0.003 | 20.639 |
|  | rs200932481 | 0.007 | 0.007 | 0.089 | 0.934 | 0.004 | 22.994 |
|  | rs35473591 | 0.071 | 0.412 | 0.015 | 0.000 | 0.137 | 991.669 |
|  | rs35599386 | -0.006 | 0.050 | 0.035 | 0.871 | 0.003 | 20.136 |
|  | rs3741252 | -0.062 | 0.129 | 0.023 | 0.007 | 0.006 | 39.579 |
|  | rs4708901 | -0.006 | 0.206 | 0.019 | 0.759 | 0.003 | 20.729 |
|  | rs474901 | -0.025 | 0.472 | 0.015 | 0.105 | 0.003 | 19.839 |
|  | rs529446 | 0.007 | 0.784 | 0.018 | 0.689 | 0.003 | 20.001 |
|  | rs600626 | 0.043 | 0.230 | 0.018 | 0.016 | 0.005 | 32.513 |
|  | rs62511275 | -0.008 | 0.043 | 0.038 | 0.830 | 0.004 | 22.556 |
|  | rs6564238 | 0.028 | 0.328 | 0.016 | 0.081 | 0.003 | 20.071 |
|  | rs7179330 | 0.021 | 0.982 | 0.057 | 0.714 | 0.004 | 23.702 |
|  | rs76469829 | 0.086 | 0.035 | 0.041 | 0.038 | 0.003 | 20.617 |
|  | rs76513766 | 0.029 | 0.011 | 0.072 | 0.691 | 0.003 | 20.216 |
|  | rs7936002 | -0.015 | 0.245 | 0.018 | 0.411 | 0.007 | 40.967 |
|  | rs79995028 | 0.103 | 0.004 | 0.126 | 0.415 | 0.003 | 20.562 |
|  | rs9967725 | -0.013 | 0.336 | 0.016 | 0.409 | 0.005 | 29.177 |
| Phosphatidylethanolamine (18:2_0:0) | rs11045856 | 0.021 | 0.172 | 0.020 | 0.293 | 0.004 | 26.652 |
|  | rs11063393 | -0.018 | 0.099 | 0.026 | 0.481 | 0.003 | 21.711 |
|  | rs111877698 | 0.025 | 0.012 | 0.073 | 0.737 | 0.003 | 23.786 |
|  | rs114899411 | 0.004 | 0.056 | 0.033 | 0.906 | 0.004 | 25.271 |
|  | rs115359603 | -0.017 | 0.014 | 0.064 | 0.793 | 0.003 | 20.766 |
|  | rs12459249 | -0.009 | 0.707 | 0.017 | 0.609 | 0.003 | 20.549 |
|  | rs1260326 | -0.057 | 0.650 | 0.016 | 0.000 | 0.006 | 41.704 |
|  | rs1338449 | -0.011 | 0.351 | 0.016 | 0.502 | 0.003 | 20.870 |
|  | rs1442407 | 0.034 | 0.183 | 0.020 | 0.085 | 0.004 | 27.510 |
|  | rs146330404 | 0.142 | 0.006 | 0.097 | 0.143 | 0.003 | 23.707 |
|  | rs174560 | 0.061 | 0.388 | 0.015 | 0.000 | 0.062 | 455.116 |
|  | rs1792909 | 0.009 | 0.393 | 0.016 | 0.566 | 0.004 | 26.569 |
|  | rs188404615 | 0.042 | 0.008 | 0.086 | 0.630 | 0.004 | 27.595 |
|  | rs2236950 | 0.021 | 0.259 | 0.017 | 0.220 | 0.003 | 20.689 |
|  | rs2290053 | -0.014 | 0.491 | 0.015 | 0.345 | 0.003 | 20.152 |
|  | rs326048 | 0.004 | 0.289 | 0.017 | 0.806 | 0.003 | 21.024 |
|  | rs35140833 | -0.049 | 0.161 | 0.021 | 0.017 | 0.003 | 22.357 |
|  | rs3741259 | 0.016 | 0.128 | 0.023 | 0.495 | 0.004 | 27.217 |
|  | rs4726320 | 0.028 | 0.896 | 0.025 | 0.256 | 0.003 | 20.578 |
|  | rs62076866 | 0.059 | 0.013 | 0.068 | 0.385 | 0.004 | 25.560 |
|  | rs62242707 | 0.006 | 0.268 | 0.017 | 0.711 | 0.003 | 20.408 |
|  | rs6413913 | -0.023 | 0.309 | 0.016 | 0.166 | 0.003 | 23.185 |
|  | rs71508993 | 0.032 | 0.029 | 0.045 | 0.475 | 0.003 | 20.575 |
|  | rs7776911 | 0.018 | 0.848 | 0.021 | 0.389 | 0.003 | 19.661 |
|  | rs791631 | -0.010 | 0.615 | 0.016 | 0.523 | 0.003 | 20.651 |
|  | rs79211442 | 0.058 | 0.128 | 0.023 | 0.011 | 0.003 | 19.687 |
|  | rs8034802 | -0.006 | 0.301 | 0.016 | 0.693 | 0.003 | 22.345 |
| Phosphatidylcholine (16:0_18:0) | rs10468017 | 0.011 | 0.332 | 0.016 | 0.507 | 0.003 | 24.903 |
|  | rs10738267 | -0.001 | 0.837 | 0.021 | 0.979 | 0.003 | 20.639 |
|  | rs10853781 | 0.000 | 0.489 | 0.015 | 0.988 | 0.003 | 21.647 |
|  | rs10890773 | -0.015 | 0.862 | 0.022 | 0.485 | 0.003 | 23.919 |
|  | rs113053792 | -0.106 | 0.012 | 0.072 | 0.139 | 0.003 | 20.522 |
|  | rs113076792 | 0.005 | 0.017 | 0.059 | 0.936 | 0.003 | 20.926 |
|  | rs1168036 | 0.025 | 0.736 | 0.017 | 0.150 | 0.003 | 22.633 |
|  | rs11723712 | -0.019 | 0.815 | 0.020 | 0.331 | 0.003 | 23.761 |
|  | rs117495065 | -0.106 | 0.020 | 0.055 | 0.054 | 0.003 | 21.282 |
|  | rs117528775 | 0.046 | 0.006 | 0.102 | 0.655 | 0.003 | 22.398 |
|  | rs118179558 | 0.041 | 0.003 | 0.149 | 0.781 | 0.003 | 19.899 |
|  | rs11870991 | -0.030 | 0.092 | 0.026 | 0.255 | 0.003 | 19.887 |
|  | rs11903018 | -0.012 | 0.172 | 0.020 | 0.563 | 0.003 | 19.989 |
|  | rs12052409 | -0.009 | 0.050 | 0.035 | 0.794 | 0.003 | 25.093 |
|  | rs13119269 | -0.019 | 0.161 | 0.021 | 0.356 | 0.003 | 19.636 |
|  | rs139644986 | 0.361 | 0.003 | 0.147 | 0.014 | 0.003 | 19.548 |
|  | rs141165989 | -0.001 | 0.043 | 0.037 | 0.976 | 0.003 | 20.646 |
|  | rs143975147 | -0.012 | 0.028 | 0.046 | 0.792 | 0.003 | 19.938 |
|  | rs174601 | 0.072 | 0.426 | 0.015 | 0.000 | 0.010 | 74.890 |
|  | rs2126263 | 0.046 | 0.854 | 0.021 | 0.033 | 0.003 | 22.357 |
|  | rs2214660 | -0.019 | 0.182 | 0.020 | 0.337 | 0.003 | 22.150 |
|  | rs2274855 | 0.020 | 0.172 | 0.020 | 0.311 | 0.003 | 19.628 |
|  | rs2391389 | -0.014 | 0.373 | 0.016 | 0.376 | 0.003 | 20.518 |
|  | rs247617 | 0.017 | 0.279 | 0.017 | 0.321 | 0.003 | 24.450 |
|  | rs2724782 | -0.010 | 0.033 | 0.042 | 0.815 | 0.003 | 19.738 |
|  | rs2833577 | 0.000 | 0.371 | 0.016 | 0.980 | 0.003 | 19.814 |
|  | rs2854275 | -0.030 | 0.102 | 0.025 | 0.229 | 0.003 | 20.441 |
|  | rs3924 | 0.003 | 0.609 | 0.016 | 0.867 | 0.003 | 19.627 |
|  | rs4659619 | -0.007 | 0.344 | 0.016 | 0.640 | 0.003 | 23.600 |
|  | rs6005648 | -0.036 | 0.159 | 0.021 | 0.082 | 0.003 | 19.811 |
|  | rs62080593 | -0.030 | 0.246 | 0.018 | 0.088 | 0.003 | 19.957 |
|  | rs62330208 | -0.007 | 0.242 | 0.018 | 0.715 | 0.004 | 25.424 |
|  | rs633695 | -0.004 | 0.305 | 0.016 | 0.826 | 0.003 | 19.888 |
|  | rs662846 | -0.056 | 0.019 | 0.056 | 0.316 | 0.003 | 22.988 |
|  | rs7046115 | 0.006 | 0.315 | 0.016 | 0.724 | 0.003 | 19.546 |
|  | rs72723967 | -0.002 | 0.170 | 0.020 | 0.926 | 0.003 | 23.645 |
|  | rs72974647 | 0.013 | 0.085 | 0.027 | 0.642 | 0.003 | 22.708 |
|  | rs76413658 | -0.030 | 0.133 | 0.022 | 0.183 | 0.003 | 20.969 |
|  | rs76779498 | -0.022 | 0.017 | 0.060 | 0.716 | 0.003 | 19.930 |
| Phosphatidylcholine (16:0_18:2) | rs10787711 | -0.009 | 0.641 | 0.016 | 0.588 | 0.004 | 26.467 |
|  | rs10890773 | -0.015 | 0.862 | 0.022 | 0.485 | 0.004 | 27.739 |
|  | rs10961143 | 0.027 | 0.049 | 0.035 | 0.447 | 0.004 | 25.349 |
|  | rs112569946 | -0.062 | 0.002 | 0.156 | 0.692 | 0.003 | 21.137 |
|  | rs11722429 | -0.023 | 0.102 | 0.025 | 0.365 | 0.003 | 22.462 |
|  | rs117528775 | 0.046 | 0.006 | 0.102 | 0.655 | 0.003 | 22.698 |
|  | rs13150924 | 0.036 | 0.122 | 0.023 | 0.123 | 0.004 | 29.063 |
|  | rs13402814 | -0.019 | 0.058 | 0.032 | 0.565 | 0.003 | 21.916 |
|  | rs143066473 | 0.034 | 0.025 | 0.050 | 0.499 | 0.003 | 21.157 |
|  | rs1488201 | -0.020 | 0.351 | 0.016 | 0.196 | 0.003 | 19.765 |
|  | rs150352222 | 0.001 | 0.016 | 0.060 | 0.988 | 0.003 | 19.757 |
|  | rs150376922 | 0.010 | 0.017 | 0.059 | 0.860 | 0.003 | 20.248 |
|  | rs1532085 | -0.010 | 0.578 | 0.015 | 0.512 | 0.006 | 43.461 |
|  | rs16985573 | -0.012 | 0.079 | 0.028 | 0.671 | 0.003 | 20.702 |
|  | rs17126640 | -0.036 | 0.193 | 0.019 | 0.063 | 0.003 | 22.734 |
|  | rs17231506 | 0.018 | 0.279 | 0.017 | 0.285 | 0.008 | 60.485 |
|  | rs174527 | 0.017 | 0.452 | 0.015 | 0.268 | 0.005 | 39.461 |
|  | rs174574 | -0.072 | 0.586 | 0.015 | 0.000 | 0.052 | 396.636 |
|  | rs1800588 | -0.022 | 0.248 | 0.017 | 0.213 | 0.007 | 50.839 |
|  | rs1833172 | -0.013 | 0.078 | 0.028 | 0.651 | 0.003 | 23.054 |
|  | rs187681244 | -0.024 | 0.008 | 0.087 | 0.782 | 0.003 | 20.214 |
|  | rs2629180 | 0.260 | 0.005 | 0.119 | 0.028 | 0.003 | 22.148 |
|  | rs35925446 | 0.006 | 0.069 | 0.030 | 0.844 | 0.003 | 20.755 |
|  | rs4412689 | 0.012 | 0.196 | 0.019 | 0.537 | 0.003 | 20.105 |
|  | rs4415100 | -0.018 | 0.209 | 0.019 | 0.337 | 0.003 | 20.770 |
|  | rs56075928 | -0.003 | 0.028 | 0.046 | 0.957 | 0.003 | 21.363 |
|  | rs62039480 | 0.013 | 0.328 | 0.016 | 0.420 | 0.006 | 42.774 |
|  | rs62330208 | -0.007 | 0.242 | 0.018 | 0.715 | 0.003 | 19.539 |
|  | rs6578729 | -0.018 | 0.084 | 0.027 | 0.505 | 0.003 | 20.131 |
|  | rs6673582 | -0.023 | 0.309 | 0.016 | 0.165 | 0.003 | 20.205 |
|  | rs73205054 | -0.065 | 0.035 | 0.041 | 0.114 | 0.003 | 20.459 |
|  | rs75833961 | -0.096 | 0.017 | 0.059 | 0.105 | 0.003 | 19.860 |
|  | rs7717591 | -0.012 | 0.681 | 0.016 | 0.471 | 0.003 | 20.430 |
|  | rs78882831 | 0.021 | 0.029 | 0.045 | 0.641 | 0.003 | 20.404 |
|  | rs7936002 | -0.015 | 0.245 | 0.018 | 0.411 | 0.004 | 25.605 |
|  | rs9436221 | -0.026 | 0.263 | 0.017 | 0.131 | 0.006 | 45.287 |
|  | rs964184 | 0.026 | 0.855 | 0.022 | 0.219 | 0.006 | 46.555 |
| Phosphatidylcholine (16:0_20:2) | rs10160784 | 0.043 | 0.231 | 0.018 | 0.016 | 0.004 | 30.408 |
|  | rs113130033 | 0.002 | 0.047 | 0.035 | 0.945 | 0.003 | 22.903 |
|  | rs113550786 | 0.051 | 0.014 | 0.066 | 0.438 | 0.003 | 22.568 |
|  | rs1168041 | 0.033 | 0.733 | 0.017 | 0.054 | 0.003 | 24.104 |
|  | rs1169289 | -0.003 | 0.465 | 0.015 | 0.851 | 0.003 | 20.130 |
|  | rs1260326 | -0.057 | 0.650 | 0.016 | 0.000 | 0.004 | 30.766 |
|  | rs12700446 | 0.012 | 0.019 | 0.055 | 0.827 | 0.003 | 20.966 |
|  | rs12761749 | 0.007 | 0.156 | 0.021 | 0.744 | 0.003 | 19.546 |
|  | rs138597 | -0.003 | 0.704 | 0.017 | 0.852 | 0.003 | 19.568 |
|  | rs142392117 | -0.018 | 0.020 | 0.055 | 0.737 | 0.003 | 23.319 |
|  | rs148069564 | -0.001 | 0.003 | 0.132 | 0.994 | 0.003 | 21.673 |
|  | rs150057262 | 0.054 | 0.050 | 0.035 | 0.120 | 0.004 | 27.319 |
|  | rs1532085 | -0.010 | 0.578 | 0.015 | 0.512 | 0.004 | 30.978 |
|  | rs1640963 | 0.013 | 0.188 | 0.019 | 0.510 | 0.003 | 19.580 |
|  | rs17126640 | -0.036 | 0.193 | 0.019 | 0.063 | 0.003 | 22.047 |
|  | rs17128436 | -0.057 | 0.015 | 0.062 | 0.358 | 0.003 | 20.138 |
|  | rs17268633 | -0.064 | 0.024 | 0.050 | 0.201 | 0.003 | 22.560 |
|  | rs174592 | 0.074 | 0.424 | 0.015 | 0.000 | 0.033 | 243.526 |
|  | rs1800588 | -0.022 | 0.248 | 0.017 | 0.213 | 0.004 | 30.277 |
|  | rs1815763 | -0.135 | 0.014 | 0.066 | 0.041 | 0.003 | 21.113 |
|  | rs2236514 | -0.008 | 0.654 | 0.016 | 0.627 | 0.004 | 25.807 |
|  | rs2317091 | -0.045 | 0.044 | 0.037 | 0.224 | 0.004 | 25.683 |
|  | rs3132935 | 0.022 | 0.169 | 0.020 | 0.270 | 0.003 | 20.010 |
|  | rs34516410 | -0.008 | 0.142 | 0.022 | 0.718 | 0.003 | 21.215 |
|  | rs62081852 | 0.071 | 0.020 | 0.055 | 0.195 | 0.005 | 33.040 |
|  | rs72800299 | 0.001 | 0.271 | 0.017 | 0.935 | 0.003 | 20.984 |
|  | rs76711488 | 0.035 | 0.025 | 0.049 | 0.481 | 0.003 | 21.493 |
|  | rs78230994 | -0.150 | 0.007 | 0.097 | 0.121 | 0.003 | 23.532 |
|  | rs79718024 | -0.012 | 0.155 | 0.021 | 0.580 | 0.003 | 22.875 |
| Phosphatidylcholine (16:0_20:4) | rs11062107 | -0.001 | 0.233 | 0.018 | 0.968 | 0.003 | 19.709 |
|  | rs113394924 | -0.057 | 0.083 | 0.028 | 0.038 | 0.007 | 51.998 |
|  | rs116438525 | 0.094 | 0.008 | 0.087 | 0.283 | 0.003 | 21.266 |
|  | rs116643980 | -0.007 | 0.054 | 0.034 | 0.838 | 0.003 | 20.924 |
|  | rs1251003 | -0.010 | 0.090 | 0.027 | 0.714 | 0.003 | 23.562 |
|  | rs12720922 | 0.021 | 0.173 | 0.020 | 0.299 | 0.003 | 19.717 |
|  | rs13329893 | 0.002 | 0.048 | 0.035 | 0.946 | 0.003 | 19.700 |
|  | rs141391095 | 0.021 | 0.025 | 0.048 | 0.667 | 0.003 | 22.132 |
|  | rs147549994 | 0.036 | 0.035 | 0.041 | 0.381 | 0.004 | 29.578 |
|  | rs149130695 | -0.004 | 0.064 | 0.032 | 0.906 | 0.003 | 19.659 |
|  | rs17017629 | 0.007 | 0.027 | 0.047 | 0.876 | 0.003 | 20.281 |
|  | rs17366743 | -0.017 | 0.036 | 0.040 | 0.670 | 0.003 | 19.594 |
|  | rs174527 | 0.017 | 0.452 | 0.015 | 0.268 | 0.015 | 111.162 |
|  | rs174533 | 0.071 | 0.414 | 0.015 | 0.000 | 0.171 | 1480.882 |
|  | rs187429064 | 0.048 | 0.051 | 0.034 | 0.164 | 0.005 | 36.009 |
|  | rs2943813 | 0.019 | 0.792 | 0.019 | 0.309 | 0.006 | 46.464 |
|  | rs2966427 | 0.006 | 0.929 | 0.029 | 0.846 | 0.003 | 20.014 |
|  | rs3018617 | 0.027 | 0.309 | 0.017 | 0.107 | 0.005 | 33.199 |
|  | rs3820438 | 0.016 | 0.288 | 0.017 | 0.353 | 0.003 | 23.703 |
|  | rs4756323 | -0.019 | 0.453 | 0.015 | 0.214 | 0.003 | 21.709 |
|  | rs499974 | 0.043 | 0.230 | 0.018 | 0.016 | 0.004 | 26.845 |
|  | rs58160859 | 0.018 | 0.210 | 0.019 | 0.336 | 0.003 | 20.602 |
|  | rs6498540 | 0.018 | 0.333 | 0.016 | 0.264 | 0.003 | 19.851 |
|  | rs72709529 | -0.079 | 0.022 | 0.052 | 0.128 | 0.003 | 23.734 |
|  | rs74754540 | 0.053 | 0.050 | 0.035 | 0.130 | 0.003 | 21.202 |
| Phosphatidylcholine (16:0_20:5) | rs102274 | 0.071 | 0.415 | 0.015 | 0.000 | 0.066 | 509.724 |
|  | rs10936925 | -0.019 | 0.344 | 0.016 | 0.235 | 0.003 | 19.759 |
|  | rs113394924 | -0.057 | 0.083 | 0.028 | 0.038 | 0.003 | 22.377 |
|  | rs113917790 | -0.065 | 0.011 | 0.073 | 0.373 | 0.003 | 22.160 |
|  | rs137945273 | -0.053 | 0.034 | 0.042 | 0.206 | 0.003 | 20.157 |
|  | rs146489454 | -0.035 | 0.027 | 0.046 | 0.451 | 0.003 | 19.667 |
|  | rs1481977 | 0.001 | 0.859 | 0.022 | 0.970 | 0.003 | 20.043 |
|  | rs174527 | 0.017 | 0.452 | 0.015 | 0.268 | 0.005 | 35.129 |
|  | rs2041149 | -0.019 | 0.464 | 0.015 | 0.207 | 0.003 | 22.029 |
|  | rs2733412 | 0.012 | 0.190 | 0.019 | 0.547 | 0.003 | 19.796 |
|  | rs2833287 | -0.093 | 0.005 | 0.103 | 0.366 | 0.003 | 19.817 |
|  | rs28569162 | 0.016 | 0.128 | 0.023 | 0.481 | 0.003 | 20.988 |
|  | rs3018617 | 0.027 | 0.309 | 0.017 | 0.107 | 0.003 | 24.625 |
|  | rs34822092 | -0.055 | 0.026 | 0.048 | 0.252 | 0.004 | 25.220 |
|  | rs3796575 | 0.018 | 0.233 | 0.018 | 0.327 | 0.003 | 20.371 |
|  | rs3936196 | -0.012 | 0.612 | 0.016 | 0.456 | 0.003 | 19.681 |
|  | rs4908782 | -0.010 | 0.346 | 0.016 | 0.545 | 0.003 | 20.799 |
|  | rs531117 | 0.043 | 0.231 | 0.018 | 0.016 | 0.003 | 19.795 |
|  | rs59839417 | -0.017 | 0.208 | 0.019 | 0.363 | 0.003 | 22.602 |
|  | rs61653779 | -0.008 | 0.016 | 0.060 | 0.897 | 0.003 | 20.516 |
|  | rs62025936 | -0.009 | 0.156 | 0.021 | 0.655 | 0.004 | 28.095 |
|  | rs74754540 | 0.053 | 0.050 | 0.035 | 0.130 | 0.003 | 23.640 |
|  | rs7780677 | -0.009 | 0.410 | 0.015 | 0.543 | 0.004 | 25.579 |
|  | rs7947954 | 0.034 | 0.153 | 0.021 | 0.106 | 0.003 | 21.974 |
|  | rs80083325 | -0.006 | 0.036 | 0.041 | 0.892 | 0.003 | 19.803 |
| Phosphatidylcholine (16:1_18:1) | rs11189347 | -0.014 | 0.135 | 0.022 | 0.532 | 0.003 | 22.141 |
|  | rs113366516 | -0.031 | 0.017 | 0.059 | 0.600 | 0.003 | 22.080 |
|  | rs116997061 | -0.009 | 0.061 | 0.032 | 0.772 | 0.003 | 21.844 |
|  | rs117217513 | -0.076 | 0.028 | 0.046 | 0.096 | 0.003 | 20.591 |
|  | rs1260326 | -0.057 | 0.650 | 0.016 | 0.000 | 0.006 | 39.803 |
|  | rs13402814 | -0.019 | 0.058 | 0.032 | 0.565 | 0.003 | 22.965 |
|  | rs139935251 | 0.006 | 0.003 | 0.151 | 0.971 | 0.003 | 20.947 |
|  | rs141084476 | 0.058 | 0.044 | 0.037 | 0.120 | 0.003 | 20.505 |
|  | rs145551604 | 0.001 | 0.075 | 0.029 | 0.981 | 0.003 | 22.598 |
|  | rs146934967 | -0.076 | 0.015 | 0.064 | 0.235 | 0.004 | 31.725 |
|  | rs1532085 | -0.010 | 0.578 | 0.015 | 0.512 | 0.004 | 30.310 |
|  | rs174560 | 0.061 | 0.388 | 0.015 | 0.000 | 0.014 | 104.489 |
|  | rs185867215 | 0.092 | 0.007 | 0.089 | 0.302 | 0.003 | 20.713 |
|  | rs36106314 | 0.012 | 0.253 | 0.018 | 0.499 | 0.003 | 19.689 |
|  | rs4447106 | -0.030 | 0.142 | 0.022 | 0.170 | 0.004 | 27.925 |
|  | rs56156922 | 0.018 | 0.279 | 0.017 | 0.289 | 0.004 | 29.434 |
|  | rs56316064 | 0.001 | 0.282 | 0.017 | 0.934 | 0.003 | 23.080 |
|  | rs5865 | -0.004 | 0.708 | 0.017 | 0.790 | 0.003 | 22.502 |
|  | rs603424 | -0.072 | 0.117 | 0.024 | 0.003 | 0.006 | 46.092 |
|  | rs7007401 | -0.014 | 0.276 | 0.017 | 0.401 | 0.003 | 19.699 |
|  | rs701919 | -0.028 | 0.784 | 0.018 | 0.127 | 0.003 | 20.845 |
|  | rs72834725 | 0.062 | 0.043 | 0.037 | 0.096 | 0.003 | 21.892 |
|  | rs74357965 | 0.003 | 0.104 | 0.025 | 0.904 | 0.003 | 20.189 |
|  | rs75102979 | 0.040 | 0.044 | 0.038 | 0.299 | 0.003 | 19.814 |
|  | rs7968329 | -0.015 | 0.441 | 0.015 | 0.324 | 0.003 | 20.372 |
|  | rs80160469 | -0.014 | 0.037 | 0.041 | 0.729 | 0.003 | 20.678 |
|  | rs8033940 | -0.016 | 0.327 | 0.016 | 0.323 | 0.004 | 27.057 |
|  | rs8100204 | 0.035 | 0.158 | 0.021 | 0.098 | 0.004 | 25.677 |
| Phosphatidylcholine (16:1_18:2) | rs10201921 | -0.018 | 0.058 | 0.032 | 0.581 | 0.003 | 19.923 |
|  | rs10890773 | -0.015 | 0.862 | 0.022 | 0.485 | 0.003 | 20.933 |
|  | rs11602429 | 0.016 | 0.131 | 0.022 | 0.473 | 0.003 | 20.682 |
|  | rs12031139 | -0.007 | 0.487 | 0.015 | 0.664 | 0.003 | 19.941 |
|  | rs1260326 | -0.057 | 0.650 | 0.016 | 0.000 | 0.004 | 31.914 |
|  | rs13150924 | 0.036 | 0.122 | 0.023 | 0.123 | 0.003 | 23.351 |
|  | rs146934967 | -0.076 | 0.015 | 0.064 | 0.235 | 0.005 | 36.173 |
|  | rs150057262 | 0.054 | 0.050 | 0.035 | 0.120 | 0.004 | 25.955 |
|  | rs1532085 | -0.010 | 0.578 | 0.015 | 0.512 | 0.005 | 33.603 |
|  | rs174527 | 0.017 | 0.452 | 0.015 | 0.268 | 0.007 | 48.162 |
|  | rs174574 | -0.072 | 0.586 | 0.015 | 0.000 | 0.064 | 491.373 |
|  | rs1800588 | -0.022 | 0.248 | 0.017 | 0.213 | 0.005 | 35.855 |
|  | rs184289628 | -0.071 | 0.020 | 0.054 | 0.192 | 0.003 | 20.714 |
|  | rs216958 | -0.001 | 0.622 | 0.016 | 0.974 | 0.003 | 21.076 |
|  | rs4122352 | -0.010 | 0.287 | 0.017 | 0.544 | 0.003 | 22.665 |
|  | rs56156922 | 0.018 | 0.279 | 0.017 | 0.289 | 0.005 | 37.774 |
|  | rs603424 | -0.072 | 0.117 | 0.024 | 0.003 | 0.005 | 38.713 |
|  | rs62104079 | 0.000 | 0.229 | 0.018 | 0.986 | 0.004 | 26.773 |
|  | rs6876235 | 0.012 | 0.897 | 0.025 | 0.639 | 0.003 | 21.352 |
|  | rs7036554 | -0.014 | 0.341 | 0.016 | 0.378 | 0.003 | 19.707 |
|  | rs7783799 | 0.056 | 0.305 | 0.016 | 0.001 | 0.003 | 22.093 |
|  | rs77949981 | -0.053 | 0.022 | 0.052 | 0.311 | 0.003 | 20.439 |
|  | rs78575494 | 0.038 | 0.037 | 0.040 | 0.338 | 0.003 | 20.013 |
|  | rs7936002 | -0.015 | 0.245 | 0.018 | 0.411 | 0.005 | 34.592 |
|  | rs9480322 | 0.007 | 0.031 | 0.044 | 0.878 | 0.003 | 24.354 |
|  | rs9839380 | -0.031 | 0.335 | 0.016 | 0.055 | 0.004 | 29.301 |
| Phosphatidylcholine (17:0_18:2) | rs11027519 | -0.011 | 0.091 | 0.026 | 0.663 | 0.003 | 19.950 |
|  | rs111233850 | 0.034 | 0.032 | 0.043 | 0.432 | 0.003 | 20.199 |
|  | rs111314022 | 0.089 | 0.032 | 0.043 | 0.039 | 0.003 | 20.345 |
|  | rs115686032 | -0.062 | 0.041 | 0.038 | 0.108 | 0.003 | 21.606 |
|  | rs1168104 | 0.024 | 0.736 | 0.017 | 0.156 | 0.003 | 20.877 |
|  | rs1253283 | 0.044 | 0.030 | 0.044 | 0.321 | 0.003 | 19.660 |
|  | rs12548274 | 0.016 | 0.163 | 0.021 | 0.442 | 0.003 | 22.095 |
|  | rs141509040 | -0.025 | 0.073 | 0.029 | 0.387 | 0.003 | 20.086 |
|  | rs1609446 | 0.044 | 0.081 | 0.028 | 0.118 | 0.003 | 19.967 |
|  | rs17231506 | 0.018 | 0.279 | 0.017 | 0.285 | 0.004 | 27.186 |
|  | rs174566 | 0.071 | 0.413 | 0.015 | 0.000 | 0.032 | 237.533 |
|  | rs1800588 | -0.022 | 0.248 | 0.017 | 0.213 | 0.005 | 35.068 |
|  | rs192537901 | 0.054 | 0.028 | 0.046 | 0.241 | 0.003 | 23.785 |
|  | rs2126260 | 0.007 | 0.747 | 0.017 | 0.685 | 0.003 | 22.916 |
|  | rs34489224 | 0.022 | 0.273 | 0.017 | 0.206 | 0.003 | 21.413 |
|  | rs422137 | 0.010 | 0.488 | 0.015 | 0.506 | 0.004 | 25.903 |
|  | rs4478142 | 0.011 | 0.157 | 0.021 | 0.581 | 0.003 | 20.039 |
|  | rs4840981 | -0.029 | 0.988 | 0.070 | 0.683 | 0.003 | 21.265 |
|  | rs57518551 | 0.007 | 0.142 | 0.022 | 0.732 | 0.004 | 25.838 |
|  | rs6498540 | 0.018 | 0.333 | 0.016 | 0.264 | 0.004 | 25.828 |
|  | rs72747028 | -0.023 | 0.068 | 0.030 | 0.439 | 0.003 | 21.691 |
|  | rs73058910 | 0.003 | 0.067 | 0.030 | 0.926 | 0.003 | 21.390 |
|  | rs7437408 | 0.017 | 0.481 | 0.015 | 0.254 | 0.003 | 21.048 |
|  | rs78592970 | -0.025 | 0.123 | 0.023 | 0.281 | 0.004 | 25.234 |
|  | rs964184 | 0.026 | 0.855 | 0.022 | 0.219 | 0.006 | 44.464 |
| Phosphatidylcholine (17:0_20:4) | rs11088781 | -0.024 | 0.910 | 0.026 | 0.356 | 0.003 | 22.380 |
|  | rs113394924 | -0.057 | 0.083 | 0.028 | 0.038 | 0.006 | 42.375 |
|  | rs11626405 | 0.006 | 0.292 | 0.017 | 0.714 | 0.003 | 20.147 |
|  | rs116643980 | -0.007 | 0.054 | 0.034 | 0.838 | 0.003 | 21.001 |
|  | rs11673120 | 0.006 | 0.414 | 0.015 | 0.716 | 0.003 | 20.096 |
|  | rs1168041 | 0.033 | 0.733 | 0.017 | 0.054 | 0.003 | 22.013 |
|  | rs117580248 | -0.013 | 0.115 | 0.024 | 0.596 | 0.003 | 21.992 |
|  | rs12429208 | 0.035 | 0.274 | 0.017 | 0.042 | 0.003 | 21.408 |
|  | rs12928399 | 0.035 | 0.348 | 0.016 | 0.028 | 0.003 | 23.814 |
|  | rs140619100 | -0.009 | 0.055 | 0.033 | 0.782 | 0.006 | 39.675 |
|  | rs142082962 | 0.028 | 0.077 | 0.029 | 0.324 | 0.003 | 21.567 |
|  | rs143060591 | 0.018 | 0.070 | 0.030 | 0.554 | 0.003 | 21.432 |
|  | rs143309746 | -0.198 | 0.023 | 0.054 | 0.000 | 0.003 | 20.883 |
|  | rs174527 | 0.017 | 0.452 | 0.015 | 0.268 | 0.015 | 105.864 |
|  | rs174533 | 0.071 | 0.414 | 0.015 | 0.000 | 0.140 | 1159.576 |
|  | rs187429064 | 0.048 | 0.051 | 0.034 | 0.164 | 0.006 | 39.655 |
|  | rs190664666 | -0.045 | 0.059 | 0.032 | 0.157 | 0.003 | 20.860 |
|  | rs2398428 | 0.001 | 0.261 | 0.017 | 0.932 | 0.003 | 24.175 |
|  | rs2469035 | 0.003 | 0.794 | 0.019 | 0.855 | 0.003 | 20.306 |
|  | rs3110971 | 0.041 | 0.205 | 0.019 | 0.028 | 0.003 | 22.849 |
|  | rs35788378 | 0.000 | 0.267 | 0.017 | 0.995 | 0.003 | 20.673 |
|  | rs55725503 | -0.050 | 0.022 | 0.052 | 0.330 | 0.003 | 20.606 |
|  | rs56399423 | 0.115 | 0.316 | 0.016 | 0.000 | 0.003 | 22.206 |
|  | rs57815521 | 0.111 | 0.029 | 0.045 | 0.014 | 0.004 | 31.489 |
|  | rs585002 | -0.055 | 0.859 | 0.022 | 0.011 | 0.003 | 22.327 |
|  | rs600518 | 0.043 | 0.230 | 0.018 | 0.016 | 0.005 | 32.226 |
|  | rs6083838 | 0.028 | 0.533 | 0.015 | 0.060 | 0.003 | 21.062 |
|  | rs61896563 | -0.014 | 0.052 | 0.034 | 0.673 | 0.003 | 22.214 |
|  | rs635196 | -0.013 | 0.177 | 0.020 | 0.517 | 0.003 | 21.333 |
|  | rs73161879 | -0.010 | 0.180 | 0.020 | 0.605 | 0.003 | 21.407 |
|  | rs74452899 | 0.039 | 0.011 | 0.072 | 0.589 | 0.004 | 25.447 |
| Phosphatidylcholine (18:0_18:2) | rs10040989 | -0.037 | 0.189 | 0.019 | 0.056 | 0.003 | 20.253 |
|  | rs10889352 | -0.025 | 0.264 | 0.017 | 0.138 | 0.004 | 28.743 |
|  | rs10890773 | -0.015 | 0.862 | 0.022 | 0.485 | 0.003 | 20.927 |
|  | rs112121830 | -0.012 | 0.068 | 0.030 | 0.692 | 0.003 | 21.780 |
|  | rs117528775 | 0.046 | 0.006 | 0.102 | 0.655 | 0.003 | 23.000 |
|  | rs12122878 | -0.030 | 0.290 | 0.017 | 0.078 | 0.003 | 19.971 |
|  | rs12211498 | -0.048 | 0.096 | 0.026 | 0.060 | 0.003 | 20.348 |
|  | rs12620952 | 0.017 | 0.006 | 0.101 | 0.867 | 0.003 | 20.601 |
|  | rs1440724 | -0.007 | 0.407 | 0.015 | 0.643 | 0.003 | 22.462 |
|  | rs150457719 | 0.038 | 0.032 | 0.043 | 0.383 | 0.003 | 22.888 |
|  | rs16838403 | 0.052 | 0.018 | 0.058 | 0.364 | 0.003 | 19.792 |
|  | rs17231506 | 0.018 | 0.279 | 0.017 | 0.285 | 0.005 | 33.668 |
|  | rs174594 | -0.073 | 0.575 | 0.015 | 0.000 | 0.030 | 218.849 |
|  | rs1800588 | -0.022 | 0.248 | 0.017 | 0.213 | 0.007 | 49.187 |
|  | rs192934615 | 0.001 | 0.018 | 0.058 | 0.983 | 0.003 | 21.802 |
|  | rs2274855 | 0.020 | 0.172 | 0.020 | 0.311 | 0.003 | 20.703 |
|  | rs34031934 | -0.015 | 0.329 | 0.016 | 0.352 | 0.003 | 23.265 |
|  | rs422137 | 0.010 | 0.488 | 0.015 | 0.506 | 0.004 | 29.519 |
|  | rs5022521 | -0.013 | 0.808 | 0.020 | 0.497 | 0.003 | 19.640 |
|  | rs55917360 | -0.021 | 0.069 | 0.030 | 0.471 | 0.004 | 29.145 |
|  | rs564216 | 0.000 | 0.050 | 0.035 | 0.998 | 0.003 | 20.393 |
|  | rs62039480 | 0.013 | 0.328 | 0.016 | 0.420 | 0.004 | 29.133 |
|  | rs6432738 | -0.014 | 0.483 | 0.015 | 0.365 | 0.003 | 20.152 |
|  | rs6678964 | 0.020 | 0.901 | 0.025 | 0.421 | 0.007 | 51.762 |
|  | rs675849 | -0.013 | 0.898 | 0.025 | 0.603 | 0.003 | 21.547 |
|  | rs6878903 | -0.012 | 0.763 | 0.018 | 0.491 | 0.003 | 19.956 |
|  | rs72840078 | 0.011 | 0.098 | 0.025 | 0.667 | 0.003 | 21.067 |
|  | rs73126869 | 0.028 | 0.101 | 0.025 | 0.263 | 0.003 | 21.561 |
|  | rs77008693 | -0.019 | 0.137 | 0.022 | 0.387 | 0.003 | 21.602 |
|  | rs77588674 | -0.039 | 0.180 | 0.020 | 0.053 | 0.003 | 19.742 |
|  | rs7932326 | -0.007 | 0.619 | 0.016 | 0.668 | 0.003 | 20.422 |
|  | rs964184 | 0.026 | 0.855 | 0.022 | 0.219 | 0.004 | 30.737 |
|  | rs9987289 | 0.040 | 0.857 | 0.022 | 0.062 | 0.004 | 28.684 |
| Phosphatidylcholine (18:0_20:4) | rs1219539 | 0.027 | 0.491 | 0.015 | 0.070 | 0.004 | 27.346 |
|  | rs12275418 | -0.052 | 0.133 | 0.022 | 0.020 | 0.004 | 25.436 |
|  | rs139102782 | -0.014 | 0.034 | 0.042 | 0.735 | 0.003 | 19.724 |
|  | rs144719617 | 0.006 | 0.105 | 0.025 | 0.807 | 0.003 | 22.050 |
|  | rs147549994 | 0.036 | 0.035 | 0.041 | 0.381 | 0.004 | 32.312 |
|  | rs1477491 | 0.004 | 0.798 | 0.019 | 0.828 | 0.004 | 25.279 |
|  | rs147981159 | -0.001 | 0.044 | 0.037 | 0.972 | 0.009 | 68.422 |
|  | rs174528 | 0.071 | 0.420 | 0.015 | 0.000 | 0.213 | 1945.604 |
|  | rs187429064 | 0.048 | 0.051 | 0.034 | 0.164 | 0.004 | 29.000 |
|  | rs2070895 | -0.029 | 0.258 | 0.017 | 0.089 | 0.003 | 24.226 |
|  | rs223841 | 0.043 | 0.169 | 0.020 | 0.034 | 0.003 | 21.924 |
|  | rs2797620 | -0.008 | 0.329 | 0.016 | 0.603 | 0.005 | 33.869 |
|  | rs2802390 | 0.000 | 0.463 | 0.015 | 0.975 | 0.003 | 20.080 |
|  | rs2990536 | 0.018 | 0.581 | 0.015 | 0.246 | 0.003 | 21.561 |
|  | rs3019200 | 0.023 | 0.808 | 0.019 | 0.240 | 0.008 | 58.257 |
|  | rs3110971 | 0.041 | 0.205 | 0.019 | 0.028 | 0.003 | 22.029 |
|  | rs3741252 | -0.062 | 0.129 | 0.023 | 0.007 | 0.013 | 98.083 |
|  | rs4382917 | -0.012 | 0.243 | 0.018 | 0.482 | 0.008 | 57.065 |
|  | rs600518 | 0.043 | 0.230 | 0.018 | 0.016 | 0.004 | 25.996 |
|  | rs61873274 | 0.037 | 0.076 | 0.029 | 0.194 | 0.003 | 20.835 |
|  | rs6860806 | 0.074 | 0.417 | 0.015 | 0.000 | 0.003 | 21.989 |
|  | rs72789541 | 0.023 | 0.316 | 0.016 | 0.153 | 0.004 | 29.299 |
|  | rs73005469 | 0.005 | 0.360 | 0.016 | 0.774 | 0.003 | 19.653 |
|  | rs7499892 | 0.020 | 0.172 | 0.020 | 0.325 | 0.003 | 19.697 |
|  | rs79136768 | 0.057 | 0.036 | 0.041 | 0.160 | 0.004 | 30.967 |
|  | rs793864 | -0.016 | 0.818 | 0.020 | 0.409 | 0.003 | 23.174 |
|  | rs938822 | 0.000 | 0.146 | 0.021 | 0.984 | 0.003 | 20.998 |
|  | rs9462860 | -0.005 | 0.449 | 0.015 | 0.725 | 0.003 | 21.000 |
| Phosphatidylcholine (18:0_20:5) | rs102275 | 0.073 | 0.415 | 0.015 | 0.000 | 0.084 | 649.745 |
|  | rs1076708 | -0.011 | 0.421 | 0.015 | 0.467 | 0.003 | 19.549 |
|  | rs12487951 | -0.022 | 0.027 | 0.047 | 0.649 | 0.003 | 19.697 |
|  | rs12656795 | -0.024 | 0.400 | 0.016 | 0.133 | 0.003 | 19.909 |
|  | rs138053477 | -0.046 | 0.041 | 0.038 | 0.228 | 0.004 | 25.613 |
|  | rs145259113 | 0.074 | 0.006 | 0.102 | 0.466 | 0.003 | 20.135 |
|  | rs146438407 | -0.125 | 0.008 | 0.089 | 0.160 | 0.003 | 20.446 |
|  | rs174527 | 0.017 | 0.452 | 0.015 | 0.268 | 0.006 | 45.386 |
|  | rs187698886 | -0.041 | 0.036 | 0.041 | 0.316 | 0.003 | 19.710 |
|  | rs2023988 | -0.003 | 0.422 | 0.015 | 0.823 | 0.003 | 20.426 |
|  | rs2054498 | -0.009 | 0.538 | 0.015 | 0.558 | 0.003 | 21.989 |
|  | rs2135279 | 0.020 | 0.583 | 0.015 | 0.193 | 0.003 | 19.641 |
|  | rs2431163 | 0.069 | 0.026 | 0.048 | 0.157 | 0.003 | 21.969 |
|  | rs3019200 | 0.023 | 0.808 | 0.019 | 0.240 | 0.004 | 25.403 |
|  | rs35226034 | -0.018 | 0.037 | 0.040 | 0.653 | 0.003 | 22.141 |
|  | rs4908782 | -0.010 | 0.346 | 0.016 | 0.545 | 0.003 | 19.747 |
|  | rs508049 | 0.020 | 0.093 | 0.026 | 0.447 | 0.003 | 23.885 |
|  | rs5896690 | 0.062 | 0.465 | 0.015 | 0.000 | 0.003 | 21.793 |
|  | rs61653779 | -0.008 | 0.016 | 0.060 | 0.897 | 0.003 | 20.827 |
|  | rs62025936 | -0.009 | 0.156 | 0.021 | 0.655 | 0.004 | 29.329 |
|  | rs71629271 | 0.003 | 0.069 | 0.030 | 0.921 | 0.003 | 21.860 |
|  | rs7780677 | -0.009 | 0.410 | 0.015 | 0.543 | 0.003 | 22.178 |
|  | rs7936002 | -0.015 | 0.245 | 0.018 | 0.411 | 0.004 | 31.347 |
|  | rs80116939 | -0.045 | 0.004 | 0.133 | 0.734 | 0.003 | 23.961 |
|  | rs9380073 | 0.001 | 0.127 | 0.023 | 0.976 | 0.003 | 22.080 |
|  | rs9587408 | -0.032 | 0.236 | 0.018 | 0.077 | 0.003 | 20.917 |
| Phosphatidylcholine (18:1_18:1) | rs10145681 | -0.070 | 0.974 | 0.048 | 0.144 | 0.003 | 22.339 |
|  | rs1077834 | -0.030 | 0.257 | 0.017 | 0.087 | 0.003 | 23.102 |
|  | rs10779836 | 0.004 | 0.771 | 0.018 | 0.840 | 0.003 | 21.236 |
|  | rs10850057 | -0.030 | 0.090 | 0.027 | 0.253 | 0.003 | 19.897 |
|  | rs10889352 | -0.025 | 0.264 | 0.017 | 0.138 | 0.005 | 35.528 |
|  | rs113277188 | 0.005 | 0.020 | 0.055 | 0.932 | 0.003 | 19.700 |
|  | rs13278111 | -0.006 | 0.429 | 0.015 | 0.679 | 0.003 | 20.215 |
|  | rs136061 | 0.001 | 0.255 | 0.017 | 0.963 | 0.003 | 22.262 |
|  | rs140268092 | -0.095 | 0.005 | 0.107 | 0.374 | 0.003 | 20.673 |
|  | rs144512980 | 0.136 | 0.011 | 0.072 | 0.058 | 0.004 | 28.815 |
|  | rs1532085 | -0.010 | 0.578 | 0.015 | 0.512 | 0.006 | 42.516 |
|  | rs17231506 | 0.018 | 0.279 | 0.017 | 0.285 | 0.005 | 34.683 |
|  | rs174574 | -0.072 | 0.586 | 0.015 | 0.000 | 0.020 | 142.818 |
|  | rs2000827 | 0.019 | 0.890 | 0.024 | 0.438 | 0.003 | 20.143 |
|  | rs2126263 | 0.046 | 0.854 | 0.021 | 0.033 | 0.003 | 22.694 |
|  | rs3741298 | 0.030 | 0.769 | 0.018 | 0.096 | 0.003 | 20.792 |
|  | rs41391244 | 0.026 | 0.234 | 0.018 | 0.149 | 0.003 | 21.387 |
|  | rs41441450 | 0.098 | 0.010 | 0.077 | 0.203 | 0.003 | 21.327 |
|  | rs4848460 | 0.036 | 0.055 | 0.033 | 0.283 | 0.003 | 20.844 |
|  | rs56057593 | 0.032 | 0.029 | 0.046 | 0.492 | 0.003 | 21.153 |
|  | rs7300883 | -0.001 | 0.439 | 0.015 | 0.930 | 0.003 | 20.300 |
|  | rs73241068 | 0.011 | 0.034 | 0.041 | 0.797 | 0.003 | 22.775 |
|  | rs79008894 | -0.069 | 0.035 | 0.042 | 0.098 | 0.003 | 20.657 |
| Phosphatidylcholine (18:1_18:2) | rs1077835 | -0.030 | 0.257 | 0.017 | 0.086 | 0.006 | 42.386 |
|  | rs10889352 | -0.025 | 0.264 | 0.017 | 0.138 | 0.006 | 45.370 |
|  | rs11757990 | -0.104 | 0.006 | 0.099 | 0.294 | 0.003 | 19.619 |
|  | rs118108980 | -0.013 | 0.188 | 0.019 | 0.501 | 0.003 | 22.696 |
|  | rs12798268 | -0.033 | 0.864 | 0.022 | 0.133 | 0.003 | 20.165 |
|  | rs13128807 | 0.019 | 0.187 | 0.019 | 0.335 | 0.003 | 22.814 |
|  | rs142689129 | 0.338 | 0.003 | 0.134 | 0.011 | 0.003 | 24.817 |
|  | rs147761802 | -0.073 | 0.054 | 0.034 | 0.030 | 0.003 | 21.272 |
|  | rs1601935 | -0.022 | 0.616 | 0.016 | 0.162 | 0.005 | 38.658 |
|  | rs17231506 | 0.018 | 0.279 | 0.017 | 0.285 | 0.006 | 41.820 |
|  | rs174527 | 0.017 | 0.452 | 0.015 | 0.268 | 0.004 | 28.211 |
|  | rs174574 | -0.072 | 0.586 | 0.015 | 0.000 | 0.069 | 535.116 |
|  | rs2236605 | -0.034 | 0.422 | 0.015 | 0.026 | 0.004 | 25.246 |
|  | rs28844909 | -0.014 | 0.066 | 0.031 | 0.658 | 0.003 | 20.197 |
|  | rs4944732 | -0.015 | 0.135 | 0.022 | 0.491 | 0.003 | 19.897 |
|  | rs564216 | 0.000 | 0.050 | 0.035 | 0.998 | 0.003 | 21.058 |
|  | rs6023985 | 0.109 | 0.015 | 0.065 | 0.095 | 0.003 | 21.244 |
|  | rs613808 | 0.014 | 0.625 | 0.016 | 0.367 | 0.004 | 27.730 |
|  | rs62004153 | 0.014 | 0.908 | 0.026 | 0.582 | 0.003 | 19.693 |
|  | rs6498540 | 0.018 | 0.333 | 0.016 | 0.264 | 0.003 | 20.039 |
|  | rs7036554 | -0.014 | 0.341 | 0.016 | 0.378 | 0.004 | 26.696 |
|  | rs78882831 | 0.021 | 0.029 | 0.045 | 0.641 | 0.003 | 19.861 |
|  | rs948727 | -0.015 | 0.329 | 0.016 | 0.363 | 0.003 | 21.994 |
|  | rs9789400 | 0.049 | 0.276 | 0.017 | 0.004 | 0.003 | 22.021 |
| Phosphatidylcholine (18:1_20:2) | rs10846753 | 0.020 | 0.231 | 0.018 | 0.255 | 0.004 | 24.351 |
|  | rs114139553 | 0.053 | 0.013 | 0.066 | 0.424 | 0.004 | 21.015 |
|  | rs116922907 | 0.005 | 0.045 | 0.037 | 0.881 | 0.004 | 20.854 |
|  | rs117534325 | -0.066 | 0.005 | 0.115 | 0.563 | 0.004 | 22.223 |
|  | rs118150981 | 0.072 | 0.020 | 0.055 | 0.188 | 0.004 | 21.051 |
|  | rs12113068 | 0.004 | 0.023 | 0.050 | 0.938 | 0.003 | 19.669 |
|  | rs12417747 | 0.021 | 0.115 | 0.024 | 0.388 | 0.007 | 43.007 |
|  | rs174527 | 0.017 | 0.452 | 0.015 | 0.268 | 0.004 | 24.547 |
|  | rs174580 | 0.073 | 0.415 | 0.015 | 0.000 | 0.090 | 580.696 |
|  | rs17543793 | -0.001 | 0.020 | 0.055 | 0.985 | 0.003 | 20.322 |
|  | rs1800588 | -0.022 | 0.248 | 0.017 | 0.213 | 0.005 | 27.543 |
|  | rs265114 | 0.003 | 0.074 | 0.029 | 0.930 | 0.004 | 23.847 |
|  | rs35195855 | -0.015 | 0.143 | 0.022 | 0.491 | 0.004 | 21.787 |
|  | rs56208677 | -0.005 | 0.076 | 0.028 | 0.850 | 0.005 | 26.554 |
|  | rs61893829 | -0.015 | 0.086 | 0.027 | 0.586 | 0.004 | 21.308 |
|  | rs656095 | 0.049 | 0.222 | 0.018 | 0.006 | 0.006 | 34.601 |
|  | rs6944011 | 0.002 | 0.208 | 0.019 | 0.902 | 0.003 | 20.087 |
|  | rs6984305 | 0.036 | 0.841 | 0.021 | 0.085 | 0.004 | 23.463 |
|  | rs7041283 | -0.025 | 0.246 | 0.018 | 0.157 | 0.005 | 29.495 |
|  | rs7176661 | 0.005 | 0.597 | 0.015 | 0.759 | 0.003 | 19.838 |
|  | rs73292027 | 0.007 | 0.105 | 0.025 | 0.764 | 0.004 | 24.859 |
|  | rs74089903 | -0.154 | 0.009 | 0.081 | 0.057 | 0.005 | 27.553 |
|  | rs80201535 | -0.024 | 0.028 | 0.046 | 0.604 | 0.004 | 21.230 |
|  | rs9384216 | 0.021 | 0.098 | 0.026 | 0.407 | 0.003 | 20.209 |
| Phosphatidylcholine (18:1_20:4) | rs1077834 | -0.030 | 0.257 | 0.017 | 0.087 | 0.004 | 26.370 |
|  | rs10793123 | 0.028 | 0.487 | 0.015 | 0.062 | 0.003 | 20.821 |
|  | rs10889353 | -0.026 | 0.264 | 0.017 | 0.133 | 0.003 | 20.530 |
|  | rs113049169 | -0.121 | 0.032 | 0.045 | 0.007 | 0.003 | 19.680 |
|  | rs113394924 | -0.057 | 0.083 | 0.028 | 0.038 | 0.005 | 39.462 |
|  | rs113913626 | 0.024 | 0.035 | 0.041 | 0.567 | 0.003 | 19.533 |
|  | rs116814948 | 0.047 | 0.010 | 0.077 | 0.542 | 0.003 | 20.447 |
|  | rs147549994 | 0.036 | 0.035 | 0.041 | 0.381 | 0.004 | 26.191 |
|  | rs147797702 | 0.048 | 0.010 | 0.075 | 0.523 | 0.004 | 30.191 |
|  | rs173539 | 0.019 | 0.283 | 0.017 | 0.257 | 0.003 | 21.167 |
|  | rs174527 | 0.017 | 0.452 | 0.015 | 0.268 | 0.012 | 89.070 |
|  | rs174537 | 0.069 | 0.413 | 0.015 | 0.000 | 0.123 | 1006.817 |
|  | rs187429064 | 0.048 | 0.051 | 0.034 | 0.164 | 0.007 | 53.611 |
|  | rs1917701 | 0.035 | 0.945 | 0.033 | 0.288 | 0.003 | 20.013 |
|  | rs2943813 | 0.019 | 0.792 | 0.019 | 0.309 | 0.006 | 40.322 |
|  | rs3018617 | 0.027 | 0.309 | 0.017 | 0.107 | 0.003 | 23.807 |
|  | rs4628166 | -0.002 | 0.631 | 0.016 | 0.916 | 0.003 | 19.699 |
|  | rs4846905 | 0.014 | 0.765 | 0.018 | 0.442 | 0.003 | 23.180 |
|  | rs531117 | 0.043 | 0.231 | 0.018 | 0.016 | 0.005 | 32.924 |
|  | rs6083838 | 0.028 | 0.533 | 0.015 | 0.060 | 0.003 | 19.625 |
|  | rs61978272 | -0.005 | 0.276 | 0.017 | 0.777 | 0.004 | 27.749 |
|  | rs641738 | 0.016 | 0.604 | 0.015 | 0.289 | 0.003 | 24.969 |
|  | rs6850591 | -0.002 | 0.549 | 0.015 | 0.874 | 0.003 | 23.007 |
|  | rs73176681 | 0.040 | 0.047 | 0.036 | 0.255 | 0.003 | 20.807 |
|  | rs73618588 | 0.000 | 0.090 | 0.026 | 0.994 | 0.003 | 20.508 |
|  | rs75976069 | -0.051 | 0.026 | 0.048 | 0.288 | 0.003 | 23.447 |
|  | rs76582138 | -0.105 | 0.009 | 0.083 | 0.206 | 0.003 | 20.340 |
|  | rs79113397 | 0.028 | 0.234 | 0.018 | 0.115 | 0.003 | 24.493 |
|  | rs793864 | -0.016 | 0.818 | 0.020 | 0.409 | 0.003 | 21.873 |
|  | rs8107974 | 0.001 | 0.064 | 0.031 | 0.964 | 0.004 | 30.771 |
| Phosphatidylcholine (18:2_18:2) | rs1077835 | -0.030 | 0.257 | 0.017 | 0.086 | 0.005 | 35.494 |
|  | rs1168127 | 0.025 | 0.736 | 0.017 | 0.139 | 0.003 | 23.658 |
|  | rs117017186 | -0.025 | 0.011 | 0.073 | 0.737 | 0.003 | 20.740 |
|  | rs117675848 | -0.004 | 0.018 | 0.058 | 0.947 | 0.003 | 24.053 |
|  | rs11869356 | -0.018 | 0.589 | 0.015 | 0.239 | 0.003 | 20.029 |
|  | rs13078306 | 0.001 | 0.334 | 0.016 | 0.965 | 0.003 | 21.544 |
|  | rs13150924 | 0.036 | 0.122 | 0.023 | 0.123 | 0.003 | 19.901 |
|  | rs139598970 | -0.011 | 0.014 | 0.065 | 0.861 | 0.003 | 22.161 |
|  | rs147002062 | -0.064 | 0.003 | 0.132 | 0.627 | 0.003 | 20.954 |
|  | rs148498852 | 0.003 | 0.081 | 0.028 | 0.910 | 0.003 | 22.909 |
|  | rs1601935 | -0.022 | 0.616 | 0.016 | 0.162 | 0.004 | 30.334 |
|  | rs173539 | 0.019 | 0.283 | 0.017 | 0.257 | 0.004 | 28.395 |
|  | rs174574 | -0.072 | 0.586 | 0.015 | 0.000 | 0.024 | 173.147 |
|  | rs2236605 | -0.034 | 0.422 | 0.015 | 0.026 | 0.003 | 20.451 |
|  | rs35958132 | -0.004 | 0.065 | 0.031 | 0.909 | 0.003 | 21.078 |
|  | rs4271965 | -0.005 | 0.568 | 0.015 | 0.743 | 0.003 | 22.475 |
|  | rs56158036 | 0.070 | 0.017 | 0.059 | 0.238 | 0.003 | 21.504 |
|  | rs59256568 | -0.102 | 0.020 | 0.054 | 0.061 | 0.003 | 22.320 |
|  | rs62238391 | 0.008 | 0.319 | 0.016 | 0.636 | 0.003 | 23.335 |
|  | rs6498540 | 0.018 | 0.333 | 0.016 | 0.264 | 0.004 | 27.719 |
|  | rs7122803 | -0.015 | 0.862 | 0.022 | 0.487 | 0.003 | 20.412 |
|  | rs7717591 | -0.012 | 0.681 | 0.016 | 0.471 | 0.004 | 28.761 |
|  | rs7798734 | 0.015 | 0.318 | 0.016 | 0.367 | 0.003 | 21.438 |
|  | rs80315588 | 0.018 | 0.427 | 0.015 | 0.234 | 0.003 | 20.805 |
| Phosphatidylcholine (O-16:0_18:2) | rs11777584 | -0.006 | 0.338 | 0.016 | 0.726 | 0.003 | 19.543 |
|  | rs11918522 | 0.031 | 0.162 | 0.021 | 0.135 | 0.003 | 21.241 |
|  | rs11936543 | 0.002 | 0.095 | 0.026 | 0.934 | 0.003 | 19.745 |
|  | rs140661870 | 0.069 | 0.014 | 0.066 | 0.299 | 0.003 | 20.925 |
|  | rs149918547 | -0.029 | 0.005 | 0.106 | 0.785 | 0.003 | 19.538 |
|  | rs1532085 | -0.010 | 0.578 | 0.015 | 0.512 | 0.004 | 26.051 |
|  | rs17086339 | -0.015 | 0.136 | 0.022 | 0.495 | 0.003 | 22.068 |
|  | rs17231506 | 0.018 | 0.279 | 0.017 | 0.285 | 0.006 | 42.917 |
|  | rs174574 | -0.072 | 0.586 | 0.015 | 0.000 | 0.016 | 114.535 |
|  | rs181750334 | 0.082 | 0.009 | 0.083 | 0.321 | 0.003 | 20.828 |
|  | rs2537611 | 0.004 | 0.374 | 0.016 | 0.810 | 0.003 | 20.244 |
|  | rs4579316 | -0.031 | 0.833 | 0.020 | 0.129 | 0.003 | 22.981 |
|  | rs6582326 | 0.073 | 0.965 | 0.042 | 0.083 | 0.003 | 20.560 |
|  | rs6741227 | 0.008 | 0.331 | 0.016 | 0.631 | 0.003 | 21.993 |
|  | rs6764912 | -0.053 | 0.085 | 0.027 | 0.052 | 0.003 | 20.284 |
|  | rs75804573 | 0.028 | 0.151 | 0.021 | 0.189 | 0.003 | 21.057 |
|  | rs75863117 | 0.024 | 0.094 | 0.026 | 0.363 | 0.003 | 19.766 |
|  | rs76888052 | -0.016 | 0.005 | 0.112 | 0.885 | 0.003 | 19.960 |
|  | rs78285907 | 0.006 | 0.088 | 0.027 | 0.826 | 0.003 | 19.822 |
|  | rs79195215 | 0.081 | 0.028 | 0.046 | 0.079 | 0.003 | 19.569 |
|  | rs923920 | 0.008 | 0.537 | 0.015 | 0.583 | 0.003 | 20.825 |
| Phosphatidylcholine (O-16:1_20:4) | rs1037782 | 0.037 | 0.056 | 0.033 | 0.261 | 0.003 | 20.771 |
|  | rs10487877 | -0.001 | 0.201 | 0.019 | 0.950 | 0.004 | 23.469 |
|  | rs1077989 | -0.052 | 0.454 | 0.015 | 0.001 | 0.018 | 113.093 |
|  | rs114325590 | -0.015 | 0.012 | 0.072 | 0.829 | 0.004 | 25.427 |
|  | rs12579775 | -0.002 | 0.087 | 0.027 | 0.954 | 0.004 | 23.878 |
|  | rs12920974 | -0.004 | 0.321 | 0.016 | 0.788 | 0.005 | 28.146 |
|  | rs1316133 | -0.003 | 0.300 | 0.017 | 0.867 | 0.003 | 19.801 |
|  | rs138018897 | 0.051 | 0.024 | 0.051 | 0.313 | 0.003 | 20.974 |
|  | rs17268903 | -0.018 | 0.125 | 0.023 | 0.445 | 0.003 | 19.890 |
|  | rs174527 | 0.017 | 0.452 | 0.015 | 0.268 | 0.007 | 42.432 |
|  | rs174535 | 0.070 | 0.415 | 0.015 | 0.000 | 0.077 | 505.745 |
|  | rs180360 | -0.016 | 0.322 | 0.016 | 0.338 | 0.003 | 20.411 |
|  | rs2015950 | 0.016 | 0.140 | 0.022 | 0.453 | 0.004 | 25.188 |
|  | rs2043085 | -0.008 | 0.580 | 0.015 | 0.588 | 0.005 | 29.882 |
|  | rs2431867 | -0.003 | 0.208 | 0.019 | 0.893 | 0.003 | 20.015 |
|  | rs5028238 | 0.011 | 0.764 | 0.018 | 0.555 | 0.003 | 20.043 |
|  | rs56303140 | -0.012 | 0.033 | 0.043 | 0.782 | 0.003 | 21.005 |
|  | rs6752137 | -0.007 | 0.117 | 0.024 | 0.758 | 0.004 | 21.577 |
|  | rs6776300 | -0.006 | 0.530 | 0.015 | 0.684 | 0.003 | 19.918 |
|  | rs71366199 | -0.047 | 0.150 | 0.021 | 0.025 | 0.003 | 20.361 |
|  | rs71452092 | 0.073 | 0.035 | 0.041 | 0.078 | 0.003 | 19.763 |
|  | rs73531625 | 0.035 | 0.281 | 0.017 | 0.039 | 0.003 | 20.935 |
|  | rs74854572 | -0.040 | 0.011 | 0.074 | 0.584 | 0.003 | 20.394 |
|  | rs7963111 | 0.013 | 0.051 | 0.035 | 0.704 | 0.003 | 20.563 |
|  | rs79740876 | 0.054 | 0.053 | 0.034 | 0.109 | 0.004 | 21.170 |
| Phosphatidylcholine (O-17:0_17:1) | rs10468017 | 0.011 | 0.332 | 0.016 | 0.507 | 0.003 | 23.276 |
|  | rs11068152 | -0.018 | 0.242 | 0.018 | 0.310 | 0.003 | 20.907 |
|  | rs112832771 | 0.055 | 0.016 | 0.062 | 0.377 | 0.003 | 20.328 |
|  | rs115823613 | -0.017 | 0.071 | 0.030 | 0.565 | 0.003 | 19.736 |
|  | rs11691441 | -0.065 | 0.055 | 0.033 | 0.053 | 0.003 | 20.993 |
|  | rs11947730 | -0.013 | 0.735 | 0.017 | 0.439 | 0.003 | 20.787 |
|  | rs12186559 | -0.028 | 0.037 | 0.040 | 0.490 | 0.003 | 23.618 |
|  | rs150269921 | 0.020 | 0.007 | 0.090 | 0.821 | 0.003 | 21.222 |
|  | rs151297234 | -0.017 | 0.025 | 0.048 | 0.719 | 0.003 | 20.458 |
|  | rs174601 | 0.072 | 0.426 | 0.015 | 0.000 | 0.004 | 26.580 |
|  | rs17513056 | 0.016 | 0.221 | 0.018 | 0.385 | 0.004 | 24.365 |
|  | rs17611110 | -0.006 | 0.243 | 0.018 | 0.725 | 0.004 | 24.995 |
|  | rs1893460 | -0.030 | 0.226 | 0.018 | 0.099 | 0.003 | 19.977 |
|  | rs247617 | 0.017 | 0.279 | 0.017 | 0.321 | 0.006 | 40.003 |
|  | rs2725087 | -0.012 | 0.026 | 0.048 | 0.795 | 0.003 | 20.015 |
|  | rs2784923 | -0.003 | 0.941 | 0.032 | 0.936 | 0.003 | 20.264 |
|  | rs28403152 | -0.058 | 0.012 | 0.072 | 0.416 | 0.003 | 19.795 |
|  | rs3765259 | -0.031 | 0.433 | 0.015 | 0.042 | 0.003 | 19.585 |
|  | rs38820 | 0.008 | 0.911 | 0.027 | 0.775 | 0.003 | 20.880 |
|  | rs4517092 | 0.074 | 0.015 | 0.063 | 0.239 | 0.003 | 19.722 |
|  | rs612577 | -0.008 | 0.187 | 0.019 | 0.676 | 0.004 | 27.072 |
|  | rs61644965 | -0.027 | 0.028 | 0.046 | 0.553 | 0.003 | 20.343 |
|  | rs62072490 | 0.018 | 0.202 | 0.019 | 0.356 | 0.004 | 24.235 |
|  | rs709981 | 0.014 | 0.471 | 0.015 | 0.370 | 0.003 | 19.788 |
|  | rs7223101 | -0.017 | 0.375 | 0.016 | 0.272 | 0.003 | 21.118 |
|  | rs72495602 | 0.034 | 0.132 | 0.022 | 0.123 | 0.003 | 22.146 |
|  | rs72975671 | -0.025 | 0.212 | 0.019 | 0.182 | 0.003 | 20.773 |
|  | rs75206639 | 0.052 | 0.016 | 0.061 | 0.394 | 0.003 | 19.726 |
|  | rs7575011 | -0.012 | 0.669 | 0.016 | 0.468 | 0.003 | 21.011 |
|  | rs7597998 | 0.031 | 0.755 | 0.018 | 0.075 | 0.003 | 19.961 |
|  | rs7819125 | 0.019 | 0.337 | 0.016 | 0.246 | 0.003 | 20.237 |
|  | rs79648513 | -0.010 | 0.242 | 0.018 | 0.575 | 0.003 | 22.057 |
|  | rs9516264 | -0.016 | 0.331 | 0.016 | 0.335 | 0.003 | 19.823 |
| Phosphatidylcholine (O-18:0_20:4) | rs11057853 | -0.032 | 0.451 | 0.015 | 0.038 | 0.003 | 20.476 |
|  | rs117870830 | 0.118 | 0.020 | 0.054 | 0.030 | 0.003 | 20.689 |
|  | rs11954170 | -0.002 | 0.314 | 0.016 | 0.901 | 0.003 | 20.489 |
|  | rs138202451 | 0.115 | 0.009 | 0.083 | 0.166 | 0.004 | 26.787 |
|  | rs143233558 | -0.044 | 0.094 | 0.026 | 0.089 | 0.004 | 27.577 |
|  | rs145755646 | -0.027 | 0.063 | 0.031 | 0.381 | 0.003 | 21.195 |
|  | rs148462060 | 0.055 | 0.024 | 0.049 | 0.267 | 0.003 | 21.660 |
|  | rs148840717 | -0.041 | 0.035 | 0.041 | 0.313 | 0.003 | 19.740 |
|  | rs174536 | 0.070 | 0.414 | 0.015 | 0.000 | 0.045 | 331.183 |
|  | rs1800588 | -0.022 | 0.248 | 0.017 | 0.213 | 0.004 | 27.560 |
|  | rs34382810 | 0.019 | 0.363 | 0.016 | 0.217 | 0.003 | 23.749 |
|  | rs34737685 | -0.021 | 0.529 | 0.015 | 0.170 | 0.003 | 20.634 |
|  | rs3741252 | -0.062 | 0.129 | 0.023 | 0.007 | 0.005 | 36.061 |
|  | rs376516 | -0.282 | 0.000 | 0.395 | 0.475 | 0.003 | 19.830 |
|  | rs4706511 | -0.007 | 0.191 | 0.019 | 0.735 | 0.003 | 19.541 |
|  | rs4782783 | -0.014 | 0.886 | 0.024 | 0.547 | 0.003 | 21.473 |
|  | rs6992951 | 0.000 | 0.394 | 0.015 | 0.988 | 0.003 | 22.579 |
|  | rs73074943 | 0.029 | 0.025 | 0.048 | 0.550 | 0.003 | 20.667 |
|  | rs7412 | -0.010 | 0.053 | 0.034 | 0.780 | 0.004 | 26.340 |
|  | rs78711160 | -0.032 | 0.043 | 0.037 | 0.384 | 0.003 | 21.060 |
|  | rs79856406 | 0.059 | 0.102 | 0.025 | 0.019 | 0.003 | 19.540 |
|  | rs9591400 | -0.018 | 0.115 | 0.024 | 0.439 | 0.003 | 20.623 |
| Phosphatidylcholine (O-18:1_18:2) | rs11022354 | -0.010 | 0.528 | 0.015 | 0.522 | 0.003 | 19.637 |
|  | rs111306887 | -0.008 | 0.031 | 0.045 | 0.855 | 0.003 | 20.640 |
|  | rs115600841 | -0.015 | 0.019 | 0.056 | 0.785 | 0.003 | 19.640 |
|  | rs117145701 | -0.068 | 0.010 | 0.078 | 0.381 | 0.003 | 19.960 |
|  | rs11918522 | 0.031 | 0.162 | 0.021 | 0.135 | 0.004 | 29.009 |
|  | rs147996419 | -0.095 | 0.014 | 0.064 | 0.139 | 0.003 | 19.534 |
|  | rs1601934 | -0.014 | 0.654 | 0.016 | 0.364 | 0.004 | 28.766 |
|  | rs17086339 | -0.015 | 0.136 | 0.022 | 0.495 | 0.003 | 19.867 |
|  | rs17231506 | 0.018 | 0.279 | 0.017 | 0.285 | 0.004 | 24.907 |
|  | rs174551 | 0.070 | 0.412 | 0.015 | 0.000 | 0.025 | 173.589 |
|  | rs17875991 | 0.050 | 0.039 | 0.040 | 0.205 | 0.003 | 19.582 |
|  | rs1800588 | -0.022 | 0.248 | 0.017 | 0.213 | 0.003 | 20.248 |
|  | rs2133619 | 0.033 | 0.025 | 0.048 | 0.497 | 0.003 | 20.724 |
|  | rs2537611 | 0.004 | 0.374 | 0.016 | 0.810 | 0.004 | 24.009 |
|  | rs2985394 | -0.026 | 0.652 | 0.016 | 0.097 | 0.003 | 22.096 |
|  | rs4655970 | -0.011 | 0.653 | 0.016 | 0.477 | 0.003 | 20.293 |
|  | rs58828781 | 0.049 | 0.048 | 0.036 | 0.175 | 0.003 | 21.099 |
|  | rs606587 | -0.029 | 0.377 | 0.016 | 0.066 | 0.004 | 26.043 |
|  | rs61833681 | 0.028 | 0.244 | 0.018 | 0.117 | 0.003 | 20.019 |
|  | rs62294908 | 0.005 | 0.093 | 0.026 | 0.858 | 0.003 | 20.605 |
|  | rs62331262 | -0.012 | 0.176 | 0.020 | 0.541 | 0.003 | 21.777 |
|  | rs72709643 | -0.035 | 0.037 | 0.041 | 0.384 | 0.003 | 20.967 |
|  | rs73133304 | -0.097 | 0.004 | 0.114 | 0.395 | 0.003 | 20.939 |
|  | rs74864573 | 0.039 | 0.034 | 0.042 | 0.359 | 0.003 | 20.652 |
|  | rs79040967 | 0.030 | 0.043 | 0.037 | 0.415 | 0.003 | 22.010 |
| Phosphatidylethanolamine (18:0_18:2) | rs10069895 | -0.012 | 0.763 | 0.018 | 0.493 | 0.003 | 24.604 |
|  | rs10276778 | -0.035 | 0.046 | 0.036 | 0.328 | 0.003 | 20.336 |
|  | rs10468017 | 0.011 | 0.332 | 0.016 | 0.507 | 0.040 | 299.556 |
|  | rs10761714 | -0.017 | 0.850 | 0.021 | 0.426 | 0.003 | 20.370 |
|  | rs111543310 | 0.009 | 0.023 | 0.051 | 0.855 | 0.003 | 21.520 |
|  | rs112129861 | 0.033 | 0.066 | 0.030 | 0.279 | 0.003 | 21.335 |
|  | rs117376818 | 0.092 | 0.016 | 0.061 | 0.136 | 0.011 | 80.214 |
|  | rs117900629 | -0.009 | 0.055 | 0.033 | 0.787 | 0.005 | 38.337 |
|  | rs1260326 | -0.057 | 0.650 | 0.016 | 0.000 | 0.007 | 49.139 |
|  | rs12928099 | -0.010 | 0.282 | 0.017 | 0.535 | 0.005 | 36.620 |
|  | rs13110318 | 0.001 | 0.119 | 0.023 | 0.977 | 0.003 | 21.419 |
|  | rs138283783 | 0.030 | 0.024 | 0.050 | 0.542 | 0.006 | 43.088 |
|  | rs141883259 | -0.090 | 0.010 | 0.077 | 0.240 | 0.003 | 20.330 |
|  | rs149431055 | -0.099 | 0.027 | 0.047 | 0.034 | 0.003 | 19.714 |
|  | rs174527 | 0.017 | 0.452 | 0.015 | 0.268 | 0.005 | 35.448 |
|  | rs174567 | 0.073 | 0.414 | 0.015 | 0.000 | 0.057 | 429.030 |
|  | rs2840005 | 0.013 | 0.135 | 0.022 | 0.563 | 0.003 | 19.856 |
|  | rs2946493 | 0.000 | 0.197 | 0.019 | 0.989 | 0.004 | 25.569 |
|  | rs34733845 | -0.019 | 0.060 | 0.032 | 0.544 | 0.003 | 21.360 |
|  | rs35828755 | -0.006 | 0.199 | 0.019 | 0.758 | 0.003 | 19.675 |
|  | rs419482 | -0.035 | 0.394 | 0.015 | 0.025 | 0.003 | 21.233 |
|  | rs4296402 | 0.005 | 0.157 | 0.021 | 0.801 | 0.003 | 21.068 |
|  | rs449480 | 0.007 | 0.541 | 0.015 | 0.624 | 0.003 | 20.549 |
|  | rs62291572 | -0.007 | 0.210 | 0.019 | 0.692 | 0.003 | 22.667 |
|  | rs62434228 | -0.012 | 0.153 | 0.021 | 0.566 | 0.003 | 22.312 |
|  | rs633695 | -0.004 | 0.305 | 0.016 | 0.826 | 0.027 | 200.787 |
|  | rs7226158 | -0.020 | 0.683 | 0.016 | 0.230 | 0.003 | 20.493 |
|  | rs72679383 | -0.116 | 0.023 | 0.051 | 0.023 | 0.003 | 23.879 |
|  | rs73424597 | -0.008 | 0.037 | 0.040 | 0.851 | 0.009 | 61.182 |
|  | rs805292 | 0.043 | 0.362 | 0.016 | 0.006 | 0.003 | 23.781 |
|  | rs964184 | 0.026 | 0.855 | 0.022 | 0.219 | 0.006 | 44.472 |
| Sphingomyelin (d36:1) | rs10838826 | -0.033 | 0.773 | 0.018 | 0.071 | 0.003 | 20.811 |
|  | rs112585713 | -0.030 | 0.030 | 0.045 | 0.502 | 0.005 | 34.504 |
|  | rs114839471 | -0.107 | 0.007 | 0.093 | 0.252 | 0.003 | 22.129 |
|  | rs11591147 | 0.084 | 0.036 | 0.040 | 0.038 | 0.004 | 30.793 |
|  | rs1169306 | -0.012 | 0.356 | 0.016 | 0.451 | 0.003 | 24.107 |
|  | rs117442205 | 0.098 | 0.004 | 0.128 | 0.442 | 0.003 | 19.588 |
|  | rs117643293 | 0.014 | 0.063 | 0.031 | 0.659 | 0.003 | 25.115 |
|  | rs11779209 | -0.011 | 0.129 | 0.023 | 0.637 | 0.003 | 19.653 |
|  | rs12462619 | 0.011 | 0.253 | 0.017 | 0.527 | 0.004 | 31.265 |
|  | rs12464353 | -0.005 | 0.031 | 0.044 | 0.902 | 0.003 | 22.891 |
|  | rs12675806 | 0.020 | 0.113 | 0.024 | 0.395 | 0.003 | 22.719 |
|  | rs138524673 | -0.008 | 0.039 | 0.039 | 0.842 | 0.003 | 20.594 |
|  | rs1452773 | -0.010 | 0.140 | 0.022 | 0.663 | 0.003 | 23.777 |
|  | rs17664981 | -0.003 | 0.054 | 0.034 | 0.937 | 0.003 | 20.735 |
|  | rs182695896 | -0.001 | 0.026 | 0.048 | 0.977 | 0.006 | 41.028 |
|  | rs186039163 | -0.016 | 0.006 | 0.101 | 0.876 | 0.003 | 24.457 |
|  | rs2336171 | -0.026 | 0.400 | 0.016 | 0.091 | 0.018 | 131.824 |
|  | rs364585 | 0.011 | 0.658 | 0.016 | 0.472 | 0.005 | 38.565 |
|  | rs374071816 | 0.013 | 0.063 | 0.031 | 0.677 | 0.003 | 19.593 |
|  | rs3742023 | 0.004 | 0.423 | 0.015 | 0.794 | 0.003 | 20.634 |
|  | rs4144237 | -0.037 | 0.122 | 0.023 | 0.113 | 0.003 | 19.845 |
|  | rs4246215 | 0.056 | 0.443 | 0.015 | 0.000 | 0.003 | 19.761 |
|  | rs4809845 | -0.003 | 0.145 | 0.022 | 0.904 | 0.004 | 26.713 |
|  | rs4981443 | -0.021 | 0.354 | 0.016 | 0.176 | 0.003 | 19.604 |
|  | rs581080 | -0.042 | 0.855 | 0.022 | 0.052 | 0.003 | 23.220 |
|  | rs6558122 | 0.006 | 0.105 | 0.025 | 0.799 | 0.004 | 25.470 |
|  | rs6829637 | -0.003 | 0.410 | 0.015 | 0.854 | 0.003 | 20.950 |
|  | rs7157785 | -0.038 | 0.134 | 0.022 | 0.091 | 0.004 | 29.244 |
|  | rs7248003 | -0.006 | 0.724 | 0.017 | 0.710 | 0.008 | 58.170 |
|  | rs72898481 | 0.051 | 0.023 | 0.051 | 0.325 | 0.003 | 20.296 |
|  | rs73073606 | 0.026 | 0.238 | 0.018 | 0.146 | 0.003 | 19.902 |
|  | rs75679663 | 0.076 | 0.028 | 0.046 | 0.097 | 0.005 | 36.724 |
|  | rs79146711 | -0.037 | 0.043 | 0.038 | 0.329 | 0.005 | 32.871 |
|  | rs8139868 | -0.010 | 0.257 | 0.017 | 0.573 | 0.003 | 20.847 |
|  | rs884532 | -0.009 | 0.510 | 0.015 | 0.545 | 0.003 | 20.230 |
|  | rs886813 | -0.230 | 0.001 | 0.405 | 0.571 | 0.003 | 23.038 |
| Sphingomyelin (d38:1) | rs114483871 | -0.047 | 0.036 | 0.041 | 0.253 | 0.005 | 35.878 |
|  | rs11591147 | 0.084 | 0.036 | 0.040 | 0.038 | 0.006 | 41.800 |
|  | rs117146578 | 0.025 | 0.049 | 0.035 | 0.474 | 0.003 | 21.677 |
|  | rs117643293 | 0.014 | 0.063 | 0.031 | 0.659 | 0.007 | 51.391 |
|  | rs118070067 | 0.029 | 0.020 | 0.054 | 0.598 | 0.003 | 20.131 |
|  | rs12462619 | 0.011 | 0.253 | 0.017 | 0.527 | 0.003 | 24.935 |
|  | rs12480916 | 0.013 | 0.486 | 0.015 | 0.395 | 0.003 | 20.671 |
|  | rs12675806 | 0.020 | 0.113 | 0.024 | 0.395 | 0.003 | 20.931 |
|  | rs13258507 | -0.011 | 0.242 | 0.018 | 0.522 | 0.003 | 22.698 |
|  | rs144440646 | 0.022 | 0.011 | 0.074 | 0.767 | 0.003 | 23.130 |
|  | rs1452773 | -0.010 | 0.140 | 0.022 | 0.663 | 0.003 | 21.171 |
|  | rs146127446 | 0.000 | 0.418 | 0.015 | 0.997 | 0.003 | 19.931 |
|  | rs147426822 | -0.112 | 0.015 | 0.066 | 0.087 | 0.003 | 20.872 |
|  | rs182695896 | -0.001 | 0.026 | 0.048 | 0.977 | 0.007 | 49.183 |
|  | rs186039163 | -0.016 | 0.006 | 0.101 | 0.876 | 0.003 | 20.617 |
|  | rs188346987 | -0.020 | 0.046 | 0.036 | 0.581 | 0.003 | 20.311 |
|  | rs200949196 | 0.008 | 0.014 | 0.064 | 0.902 | 0.003 | 22.596 |
|  | rs2867985 | -0.006 | 0.313 | 0.016 | 0.708 | 0.003 | 20.244 |
|  | rs364585 | 0.011 | 0.658 | 0.016 | 0.472 | 0.012 | 88.272 |
|  | rs374071816 | 0.013 | 0.063 | 0.031 | 0.677 | 0.004 | 27.479 |
|  | rs3741111 | -0.015 | 0.481 | 0.015 | 0.327 | 0.004 | 26.904 |
|  | rs715119 | 0.021 | 0.239 | 0.018 | 0.241 | 0.003 | 21.776 |
|  | rs7157785 | -0.038 | 0.134 | 0.022 | 0.091 | 0.030 | 223.000 |
|  | rs7172051 | 0.011 | 0.371 | 0.016 | 0.498 | 0.003 | 19.669 |
|  | rs7247433 | 0.012 | 0.392 | 0.016 | 0.454 | 0.003 | 24.016 |
|  | rs7248003 | -0.006 | 0.724 | 0.017 | 0.710 | 0.005 | 32.942 |
|  | rs7258249 | -0.024 | 0.399 | 0.016 | 0.117 | 0.022 | 164.594 |
|  | rs73073606 | 0.026 | 0.238 | 0.018 | 0.146 | 0.003 | 22.957 |
|  | rs73193962 | 0.042 | 0.022 | 0.051 | 0.416 | 0.003 | 20.387 |
|  | rs7412 | -0.010 | 0.053 | 0.034 | 0.780 | 0.003 | 22.372 |
|  | rs7478167 | 0.043 | 0.660 | 0.016 | 0.008 | 0.003 | 21.626 |
|  | rs75679663 | 0.076 | 0.028 | 0.046 | 0.097 | 0.011 | 78.660 |
|  | rs75740927 | 0.005 | 0.030 | 0.045 | 0.920 | 0.003 | 20.149 |
|  | rs76174849 | -0.063 | 0.014 | 0.064 | 0.324 | 0.003 | 21.582 |
|  | rs76596580 | 0.013 | 0.109 | 0.024 | 0.580 | 0.003 | 21.031 |
|  | rs77645768 | -0.024 | 0.027 | 0.047 | 0.611 | 0.004 | 32.079 |
|  | rs79231873 | -0.077 | 0.021 | 0.054 | 0.155 | 0.003 | 20.118 |
|  | rs7970512 | 0.070 | 0.034 | 0.042 | 0.095 | 0.003 | 22.221 |
| Sphingomyelin (d38:2) | rs10206976 | -0.023 | 0.264 | 0.017 | 0.172 | 0.003 | 22.744 |
|  | rs10405061 | 0.023 | 0.308 | 0.016 | 0.167 | 0.004 | 27.064 |
|  | rs113118892 | -0.011 | 0.013 | 0.067 | 0.871 | 0.003 | 23.081 |
|  | rs114483871 | -0.047 | 0.036 | 0.041 | 0.253 | 0.003 | 24.085 |
|  | rs114717988 | 0.047 | 0.046 | 0.036 | 0.191 | 0.003 | 24.834 |
|  | rs11591147 | 0.084 | 0.036 | 0.040 | 0.038 | 0.003 | 21.948 |
|  | rs116411822 | -0.028 | 0.009 | 0.082 | 0.737 | 0.003 | 20.139 |
|  | rs1169306 | -0.012 | 0.356 | 0.016 | 0.451 | 0.007 | 48.205 |
|  | rs12462619 | 0.011 | 0.253 | 0.017 | 0.527 | 0.006 | 45.755 |
|  | rs139150589 | 0.053 | 0.007 | 0.092 | 0.564 | 0.004 | 27.202 |
|  | rs142118024 | -0.084 | 0.021 | 0.054 | 0.125 | 0.003 | 19.955 |
|  | rs142551539 | -0.217 | 0.007 | 0.094 | 0.021 | 0.003 | 20.071 |
|  | rs146723536 | -0.094 | 0.020 | 0.054 | 0.084 | 0.003 | 19.868 |
|  | rs148683988 | 0.026 | 0.014 | 0.065 | 0.690 | 0.003 | 21.271 |
|  | rs17276940 | -0.017 | 0.040 | 0.039 | 0.656 | 0.004 | 28.381 |
|  | rs174556 | 0.061 | 0.386 | 0.015 | 0.000 | 0.010 | 70.172 |
|  | rs182695896 | -0.001 | 0.026 | 0.048 | 0.977 | 0.005 | 37.717 |
|  | rs2336171 | -0.026 | 0.400 | 0.016 | 0.091 | 0.032 | 238.502 |
|  | rs250507 | -0.001 | 0.228 | 0.018 | 0.952 | 0.004 | 28.984 |
|  | rs3936109 | -0.006 | 0.172 | 0.020 | 0.762 | 0.003 | 22.033 |
|  | rs4804319 | 0.004 | 0.687 | 0.016 | 0.792 | 0.003 | 22.897 |
|  | rs581080 | -0.042 | 0.855 | 0.022 | 0.052 | 0.003 | 23.627 |
|  | rs61751862 | 0.001 | 0.035 | 0.042 | 0.972 | 0.003 | 22.935 |
|  | rs657315 | -0.012 | 0.293 | 0.017 | 0.459 | 0.003 | 19.788 |
|  | rs672959 | 0.208 | 0.008 | 0.087 | 0.017 | 0.003 | 20.991 |
|  | rs7248003 | -0.006 | 0.724 | 0.017 | 0.710 | 0.009 | 63.243 |
|  | rs72794954 | -0.072 | 0.007 | 0.089 | 0.422 | 0.003 | 21.830 |
|  | rs72839716 | 0.021 | 0.154 | 0.021 | 0.317 | 0.003 | 22.170 |
|  | rs75038600 | 0.161 | 0.004 | 0.117 | 0.169 | 0.003 | 20.534 |
|  | rs75098294 | -0.006 | 0.007 | 0.091 | 0.948 | 0.003 | 24.153 |
|  | rs77645768 | -0.024 | 0.027 | 0.047 | 0.611 | 0.003 | 24.894 |
| Sphingomyelin (d40:2) | rs10490233 | -0.020 | 0.111 | 0.024 | 0.400 | 0.003 | 24.479 |
|  | rs113118892 | -0.011 | 0.013 | 0.067 | 0.871 | 0.003 | 20.826 |
|  | rs113722226 | -0.075 | 0.101 | 0.025 | 0.003 | 0.003 | 25.089 |
|  | rs114483871 | -0.047 | 0.036 | 0.041 | 0.253 | 0.004 | 27.754 |
|  | rs11522703 | -0.018 | 0.169 | 0.020 | 0.365 | 0.003 | 19.762 |
|  | rs115880518 | 0.012 | 0.006 | 0.103 | 0.906 | 0.003 | 21.200 |
|  | rs11591147 | 0.084 | 0.036 | 0.040 | 0.038 | 0.004 | 30.948 |
|  | rs1237196 | 0.023 | 0.781 | 0.018 | 0.202 | 0.003 | 19.536 |
|  | rs12595641 | 0.011 | 0.200 | 0.019 | 0.551 | 0.003 | 20.509 |
|  | rs146855398 | -0.045 | 0.026 | 0.048 | 0.355 | 0.003 | 20.200 |
|  | rs148316008 | -0.025 | 0.013 | 0.068 | 0.713 | 0.003 | 20.750 |
|  | rs16851199 | 0.036 | 0.045 | 0.037 | 0.323 | 0.003 | 19.882 |
|  | rs17276933 | -0.003 | 0.064 | 0.031 | 0.926 | 0.003 | 21.005 |
|  | rs174561 | 0.061 | 0.387 | 0.015 | 0.000 | 0.007 | 49.523 |
|  | rs17604972 | -0.048 | 0.152 | 0.021 | 0.025 | 0.003 | 19.659 |
|  | rs1797734 | -0.012 | 0.648 | 0.016 | 0.464 | 0.003 | 20.067 |
|  | rs182695896 | -0.001 | 0.026 | 0.048 | 0.977 | 0.006 | 44.464 |
|  | rs187144380 | 0.150 | 0.010 | 0.076 | 0.048 | 0.003 | 21.625 |
|  | rs2760512 | -0.001 | 0.327 | 0.016 | 0.933 | 0.003 | 21.653 |
|  | rs3026120 | 0.016 | 0.076 | 0.029 | 0.571 | 0.005 | 36.478 |
|  | rs364585 | 0.011 | 0.658 | 0.016 | 0.472 | 0.007 | 48.114 |
|  | rs56298236 | -0.023 | 0.264 | 0.017 | 0.183 | 0.004 | 25.456 |
|  | rs61751862 | 0.001 | 0.035 | 0.042 | 0.972 | 0.003 | 24.597 |
|  | rs646488 | -0.022 | 0.692 | 0.016 | 0.188 | 0.003 | 20.817 |
|  | rs7157785 | -0.038 | 0.134 | 0.022 | 0.091 | 0.025 | 183.983 |
|  | rs7247433 | 0.012 | 0.392 | 0.016 | 0.454 | 0.003 | 19.961 |
|  | rs73073606 | 0.026 | 0.238 | 0.018 | 0.146 | 0.003 | 22.130 |
|  | rs73641617 | 0.032 | 0.018 | 0.057 | 0.576 | 0.003 | 21.364 |
|  | rs7478167 | 0.043 | 0.660 | 0.016 | 0.008 | 0.003 | 20.855 |
|  | rs7536561 | -0.013 | 0.572 | 0.015 | 0.379 | 0.003 | 20.232 |
|  | rs75679663 | 0.076 | 0.028 | 0.046 | 0.097 | 0.004 | 26.575 |
|  | rs77645768 | -0.024 | 0.027 | 0.047 | 0.611 | 0.004 | 32.052 |
|  | rs79402526 | 0.032 | 0.035 | 0.041 | 0.433 | 0.003 | 21.094 |
|  | rs79984432 | -0.061 | 0.012 | 0.068 | 0.371 | 0.003 | 20.531 |
|  | rs9835223 | -0.049 | 0.798 | 0.019 | 0.009 | 0.003 | 24.844 |
|  | rs9839427 | 0.045 | 0.334 | 0.016 | 0.005 | 0.003 | 20.964 |
| Sphingomyelin (d42:2) | rs10252648 | 0.038 | 0.978 | 0.051 | 0.459 | 0.003 | 19.542 |
|  | rs112585713 | -0.030 | 0.030 | 0.045 | 0.502 | 0.005 | 36.193 |
|  | rs114483871 | -0.047 | 0.036 | 0.041 | 0.253 | 0.005 | 38.862 |
|  | rs115350073 | 0.026 | 0.016 | 0.061 | 0.670 | 0.003 | 21.517 |
|  | rs11591147 | 0.084 | 0.036 | 0.040 | 0.038 | 0.008 | 60.504 |
|  | rs11610836 | -0.033 | 0.419 | 0.015 | 0.031 | 0.003 | 22.885 |
|  | rs116893213 | -0.061 | 0.036 | 0.041 | 0.134 | 0.004 | 25.657 |
|  | rs116919723 | 0.015 | 0.008 | 0.085 | 0.861 | 0.003 | 20.069 |
|  | rs118068660 | -0.072 | 0.102 | 0.025 | 0.005 | 0.008 | 60.902 |
|  | rs12530474 | 0.014 | 0.137 | 0.022 | 0.531 | 0.003 | 21.039 |
|  | rs1367117 | 0.005 | 0.280 | 0.017 | 0.748 | 0.004 | 29.151 |
|  | rs139474488 | -0.043 | 0.016 | 0.061 | 0.485 | 0.003 | 20.509 |
|  | rs146331166 | 0.019 | 0.062 | 0.032 | 0.547 | 0.006 | 40.090 |
|  | rs17018185 | -0.001 | 0.271 | 0.017 | 0.962 | 0.003 | 19.894 |
|  | rs17110517 | -0.009 | 0.057 | 0.033 | 0.789 | 0.003 | 22.389 |
|  | rs17145750 | -0.017 | 0.149 | 0.021 | 0.420 | 0.004 | 30.720 |
|  | rs17261772 | -0.037 | 0.308 | 0.017 | 0.027 | 0.004 | 25.763 |
|  | rs17276933 | -0.003 | 0.064 | 0.031 | 0.926 | 0.003 | 22.524 |
|  | rs182695896 | -0.001 | 0.026 | 0.048 | 0.977 | 0.007 | 52.244 |
|  | rs184845520 | 0.101 | 0.005 | 0.106 | 0.343 | 0.003 | 21.069 |
|  | rs2034007 | -0.020 | 0.305 | 0.016 | 0.224 | 0.003 | 20.080 |
|  | rs2177333 | -0.006 | 0.530 | 0.015 | 0.684 | 0.003 | 20.018 |
|  | rs3751934 | -0.019 | 0.423 | 0.015 | 0.216 | 0.003 | 20.746 |
|  | rs3912622 | 0.024 | 0.199 | 0.019 | 0.201 | 0.004 | 27.078 |
|  | rs4100654 | -0.013 | 0.076 | 0.029 | 0.663 | 0.003 | 21.412 |
|  | rs4283208 | -0.005 | 0.834 | 0.020 | 0.801 | 0.003 | 20.344 |
|  | rs515081 | 0.012 | 0.694 | 0.017 | 0.460 | 0.003 | 23.446 |
|  | rs59916403 | -0.003 | 0.410 | 0.015 | 0.838 | 0.003 | 21.364 |
|  | rs62274367 | 0.094 | 0.004 | 0.123 | 0.445 | 0.003 | 21.826 |
|  | rs6680361 | -0.028 | 0.800 | 0.019 | 0.141 | 0.003 | 21.051 |
|  | rs67718253 | 0.014 | 0.173 | 0.020 | 0.482 | 0.003 | 21.468 |
|  | rs6843498 | 0.001 | 0.309 | 0.016 | 0.941 | 0.004 | 25.306 |
|  | rs7104343 | -0.015 | 0.212 | 0.019 | 0.408 | 0.003 | 20.238 |
|  | rs7247433 | 0.012 | 0.392 | 0.016 | 0.454 | 0.003 | 20.702 |
|  | rs73047641 | 0.005 | 0.495 | 0.015 | 0.764 | 0.003 | 22.905 |
|  | rs7412 | -0.010 | 0.053 | 0.034 | 0.780 | 0.004 | 32.181 |
|  | rs75444922 | 0.023 | 0.022 | 0.053 | 0.667 | 0.003 | 19.641 |
|  | rs75679663 | 0.076 | 0.028 | 0.046 | 0.097 | 0.014 | 101.344 |
| Triacylglycerol (51:2) | rs10147474 | -0.016 | 0.038 | 0.039 | 0.676 | 0.004 | 25.742 |
|  | rs112122274 | -0.070 | 0.031 | 0.044 | 0.116 | 0.003 | 20.630 |
|  | rs117730598 | 0.032 | 0.032 | 0.043 | 0.464 | 0.003 | 21.128 |
|  | rs1260326 | -0.057 | 0.650 | 0.016 | 0.000 | 0.007 | 53.787 |
|  | rs139499638 | -0.116 | 0.004 | 0.122 | 0.344 | 0.003 | 20.512 |
|  | rs143838492 | -0.174 | 0.003 | 0.141 | 0.216 | 0.003 | 21.050 |
|  | rs144241030 | 0.026 | 0.087 | 0.027 | 0.333 | 0.003 | 20.841 |
|  | rs17282355 | 0.021 | 0.118 | 0.024 | 0.369 | 0.003 | 19.533 |
|  | rs174556 | 0.061 | 0.386 | 0.015 | 0.000 | 0.003 | 23.808 |
|  | rs28368829 | 0.004 | 0.235 | 0.018 | 0.838 | 0.003 | 19.943 |
|  | rs28437809 | 0.039 | 0.506 | 0.015 | 0.011 | 0.003 | 19.892 |
|  | rs316343 | -0.004 | 0.465 | 0.015 | 0.811 | 0.003 | 19.659 |
|  | rs33951980 | -0.013 | 0.129 | 0.023 | 0.557 | 0.004 | 28.608 |
|  | rs4266911 | 0.035 | 0.948 | 0.034 | 0.309 | 0.003 | 19.579 |
|  | rs62043594 | 0.007 | 0.421 | 0.015 | 0.670 | 0.003 | 22.479 |
|  | rs6721083 | -0.003 | 0.939 | 0.032 | 0.935 | 0.003 | 21.092 |
|  | rs72753379 | -0.005 | 0.036 | 0.041 | 0.895 | 0.003 | 21.318 |
|  | rs7412 | -0.010 | 0.053 | 0.034 | 0.780 | 0.003 | 20.109 |
|  | rs75573052 | -0.027 | 0.090 | 0.026 | 0.305 | 0.003 | 19.591 |
|  | rs76950187 | 0.016 | 0.078 | 0.028 | 0.575 | 0.004 | 25.911 |
|  | rs964184 | 0.026 | 0.855 | 0.022 | 0.219 | 0.012 | 84.224 |
| Triacylglycerol (52:2) | rs1010313 | 0.005 | 0.199 | 0.019 | 0.781 | 0.003 | 21.593 |
|  | rs10147474 | -0.016 | 0.038 | 0.039 | 0.676 | 0.003 | 23.430 |
|  | rs10169217 | 0.002 | 0.674 | 0.016 | 0.899 | 0.003 | 22.129 |
|  | rs1042034 | 0.003 | 0.732 | 0.017 | 0.859 | 0.003 | 23.485 |
|  | rs11017845 | -0.006 | 0.051 | 0.034 | 0.852 | 0.003 | 19.602 |
|  | rs112122274 | -0.070 | 0.031 | 0.044 | 0.116 | 0.003 | 24.281 |
|  | rs1260326 | -0.057 | 0.650 | 0.016 | 0.000 | 0.007 | 48.984 |
|  | rs144241030 | 0.026 | 0.087 | 0.027 | 0.333 | 0.003 | 21.684 |
|  | rs147987331 | -0.148 | 0.021 | 0.056 | 0.008 | 0.003 | 21.311 |
|  | rs150741556 | 0.049 | 0.025 | 0.049 | 0.314 | 0.003 | 20.635 |
|  | rs15285 | -0.029 | 0.257 | 0.017 | 0.093 | 0.004 | 31.415 |
|  | rs2980888 | 0.015 | 0.741 | 0.017 | 0.396 | 0.003 | 20.742 |
|  | rs34621709 | 0.007 | 0.174 | 0.020 | 0.735 | 0.003 | 24.992 |
|  | rs35162925 | 0.015 | 0.020 | 0.054 | 0.781 | 0.003 | 20.769 |
|  | rs35332062 | -0.015 | 0.129 | 0.023 | 0.512 | 0.004 | 30.725 |
|  | rs3770813 | 0.002 | 0.026 | 0.047 | 0.961 | 0.003 | 21.566 |
|  | rs501474 | -0.002 | 0.434 | 0.015 | 0.908 | 0.003 | 19.776 |
|  | rs62291572 | -0.007 | 0.210 | 0.019 | 0.692 | 0.003 | 20.543 |
|  | rs72753379 | -0.005 | 0.036 | 0.041 | 0.895 | 0.003 | 21.254 |
|  | rs76950187 | 0.016 | 0.078 | 0.028 | 0.575 | 0.003 | 23.127 |
|  | rs78275146 | 0.066 | 0.014 | 0.065 | 0.309 | 0.003 | 21.549 |
|  | rs79934453 | 0.060 | 0.004 | 0.119 | 0.615 | 0.003 | 22.070 |
|  | rs79954170 | -0.039 | 0.009 | 0.080 | 0.621 | 0.003 | 21.366 |
|  | rs964184 | 0.026 | 0.855 | 0.022 | 0.219 | 0.016 | 115.906 |
| **UC** |  |  |  |  |  |  |  |
| Sterol ester (27:1/20:4) | rs11022403 | -0.037 | 0.291 | 0.021 | 0.072 | 0.003 | 21.220 |
|  | rs113248417 | -0.006 | 0.039 | 0.048 | 0.905 | 0.005 | 35.544 |
|  | rs1135999 | -0.001 | 0.315 | 0.020 | 0.947 | 0.005 | 32.506 |
|  | rs113603971 | 0.010 | 0.051 | 0.042 | 0.817 | 0.003 | 19.555 |
|  | rs11591147 | 0.039 | 0.036 | 0.050 | 0.437 | 0.006 | 41.421 |
|  | rs117084735 | -0.007 | 0.016 | 0.074 | 0.924 | 0.003 | 20.353 |
|  | rs117186386 | 0.009 | 0.048 | 0.044 | 0.837 | 0.004 | 25.459 |
|  | rs117825513 | -0.097 | 0.012 | 0.084 | 0.247 | 0.003 | 20.318 |
|  | rs138482835 | -0.010 | 0.052 | 0.043 | 0.819 | 0.003 | 22.278 |
|  | rs147981159 | 0.003 | 0.044 | 0.046 | 0.941 | 0.008 | 60.908 |
|  | rs174528 | 0.081 | 0.420 | 0.019 | 0.000 | 0.202 | 1819.849 |
|  | rs1800961 | -0.149 | 0.045 | 0.046 | 0.001 | 0.003 | 20.532 |
|  | rs182695896 | -0.020 | 0.026 | 0.059 | 0.731 | 0.004 | 31.741 |
|  | rs186913978 | 0.010 | 0.009 | 0.101 | 0.918 | 0.004 | 28.720 |
|  | rs187429064 | 0.024 | 0.051 | 0.043 | 0.573 | 0.006 | 46.088 |
|  | rs2229738 | 0.019 | 0.155 | 0.026 | 0.473 | 0.006 | 42.633 |
|  | rs223841 | 0.038 | 0.169 | 0.025 | 0.124 | 0.003 | 21.183 |
|  | rs2469034 | 0.027 | 0.797 | 0.023 | 0.243 | 0.003 | 20.122 |
|  | rs3741252 | -0.077 | 0.129 | 0.028 | 0.007 | 0.010 | 74.744 |
|  | rs3767941 | 0.039 | 0.173 | 0.025 | 0.112 | 0.003 | 22.617 |
|  | rs3820438 | 0.024 | 0.288 | 0.021 | 0.248 | 0.003 | 21.528 |
|  | rs4382917 | -0.012 | 0.243 | 0.022 | 0.586 | 0.008 | 55.143 |
|  | rs635196 | -0.004 | 0.177 | 0.024 | 0.871 | 0.003 | 23.862 |
|  | rs646299 | 0.027 | 0.606 | 0.019 | 0.160 | 0.003 | 19.672 |
|  | rs6812370 | 0.095 | 0.014 | 0.078 | 0.227 | 0.004 | 27.573 |
|  | rs6860806 | 0.057 | 0.418 | 0.019 | 0.003 | 0.003 | 20.015 |
|  | rs7020 | -0.032 | 0.449 | 0.019 | 0.087 | 0.003 | 19.900 |
|  | rs7412 | -0.013 | 0.053 | 0.042 | 0.762 | 0.010 | 70.977 |
|  | rs74915447 | -0.041 | 0.036 | 0.051 | 0.414 | 0.003 | 20.006 |
|  | rs7837272 | -0.258 | 0.002 | 0.221 | 0.243 | 0.003 | 21.080 |
|  | rs79136768 | 0.057 | 0.036 | 0.050 | 0.258 | 0.003 | 24.145 |
|  | rs9388335 | 0.030 | 0.525 | 0.019 | 0.103 | 0.003 | 21.634 |
|  | rs9555673 | 0.027 | 0.424 | 0.019 | 0.147 | 0.003 | 20.745 |
| Sterol ester (27:1/20:5) | rs10860778 | -0.005 | 0.476 | 0.019 | 0.802 | 0.004 | 26.149 |
|  | rs11986253 | -0.004 | 0.180 | 0.024 | 0.882 | 0.003 | 21.842 |
|  | rs12515163 | -0.017 | 0.415 | 0.019 | 0.383 | 0.003 | 21.714 |
|  | rs143678376 | -0.037 | 0.070 | 0.037 | 0.323 | 0.003 | 23.739 |
|  | rs1481977 | -0.010 | 0.859 | 0.027 | 0.708 | 0.003 | 21.392 |
|  | rs174527 | 0.018 | 0.452 | 0.019 | 0.341 | 0.008 | 57.173 |
|  | rs174581 | 0.083 | 0.415 | 0.019 | 0.000 | 0.081 | 629.075 |
|  | rs17548303 | -0.002 | 0.258 | 0.021 | 0.916 | 0.003 | 20.434 |
|  | rs182611493 | 0.024 | 0.051 | 0.042 | 0.579 | 0.003 | 22.751 |
|  | rs2054498 | -0.003 | 0.538 | 0.019 | 0.856 | 0.004 | 26.371 |
|  | rs2625222 | -0.039 | 0.109 | 0.030 | 0.186 | 0.003 | 21.369 |
|  | rs3019200 | 0.027 | 0.808 | 0.024 | 0.248 | 0.003 | 22.293 |
|  | rs34343838 | -0.034 | 0.123 | 0.028 | 0.233 | 0.003 | 23.409 |
|  | rs4490551 | -0.016 | 0.198 | 0.023 | 0.490 | 0.003 | 19.681 |
|  | rs449647 | -0.012 | 0.134 | 0.028 | 0.663 | 0.004 | 28.288 |
|  | rs4908782 | -0.012 | 0.346 | 0.020 | 0.525 | 0.003 | 20.648 |
|  | rs508049 | 0.019 | 0.093 | 0.032 | 0.556 | 0.004 | 30.551 |
|  | rs5896690 | 0.058 | 0.465 | 0.019 | 0.002 | 0.003 | 20.687 |
|  | rs59807465 | -0.093 | 0.019 | 0.069 | 0.177 | 0.003 | 19.995 |
|  | rs62025936 | 0.009 | 0.156 | 0.026 | 0.734 | 0.003 | 22.008 |
|  | rs71629271 | 0.011 | 0.069 | 0.037 | 0.757 | 0.003 | 19.874 |
|  | rs75897070 | -0.033 | 0.051 | 0.043 | 0.438 | 0.003 | 19.816 |
|  | rs7780677 | -0.010 | 0.410 | 0.019 | 0.583 | 0.003 | 20.158 |
|  | rs7936002 | -0.013 | 0.245 | 0.022 | 0.549 | 0.004 | 31.095 |
|  | rs7947954 | 0.021 | 0.153 | 0.026 | 0.426 | 0.003 | 23.121 |
|  | rs8132104 | 0.025 | 0.264 | 0.021 | 0.231 | 0.003 | 20.221 |
|  | rs980814 | -0.003 | 0.450 | 0.019 | 0.860 | 0.003 | 21.520 |
| Phosphatidylcholine (20:4_0:0) | rs10733608 | -0.036 | 0.478 | 0.019 | 0.055 | 0.003 | 21.648 |
|  | rs10884272 | -0.001 | 0.367 | 0.019 | 0.966 | 0.003 | 20.372 |
|  | rs11669519 | -0.019 | 0.343 | 0.020 | 0.325 | 0.003 | 20.381 |
|  | rs12226389 | 0.020 | 0.178 | 0.024 | 0.422 | 0.009 | 54.075 |
|  | rs12303 | 0.007 | 0.199 | 0.023 | 0.763 | 0.003 | 21.040 |
|  | rs12905466 | -0.017 | 0.201 | 0.023 | 0.478 | 0.003 | 20.862 |
|  | rs1363011 | -0.014 | 0.138 | 0.027 | 0.614 | 0.003 | 19.537 |
|  | rs139516883 | -0.038 | 0.014 | 0.080 | 0.637 | 0.004 | 24.495 |
|  | rs142999489 | 0.015 | 0.056 | 0.040 | 0.712 | 0.004 | 23.796 |
|  | rs150158785 | -0.033 | 0.079 | 0.035 | 0.336 | 0.003 | 20.086 |
|  | rs182611493 | 0.024 | 0.051 | 0.042 | 0.579 | 0.004 | 26.444 |
|  | rs191895023 | 0.030 | 0.003 | 0.189 | 0.876 | 0.003 | 20.639 |
|  | rs200932481 | 0.066 | 0.007 | 0.110 | 0.547 | 0.004 | 22.994 |
|  | rs35473591 | 0.081 | 0.413 | 0.019 | 0.000 | 0.137 | 991.669 |
|  | rs35599386 | 0.011 | 0.050 | 0.043 | 0.799 | 0.003 | 20.136 |
|  | rs3741252 | -0.077 | 0.129 | 0.028 | 0.007 | 0.006 | 39.579 |
|  | rs4708901 | 0.002 | 0.205 | 0.023 | 0.941 | 0.003 | 20.729 |
|  | rs474901 | -0.021 | 0.471 | 0.019 | 0.267 | 0.003 | 19.839 |
|  | rs529446 | -0.006 | 0.784 | 0.023 | 0.776 | 0.003 | 20.001 |
|  | rs600626 | 0.050 | 0.231 | 0.022 | 0.023 | 0.005 | 32.513 |
|  | rs62511275 | 0.002 | 0.043 | 0.047 | 0.969 | 0.004 | 22.556 |
|  | rs6564238 | 0.017 | 0.328 | 0.020 | 0.382 | 0.003 | 20.071 |
|  | rs7179330 | -0.029 | 0.982 | 0.070 | 0.682 | 0.004 | 23.702 |
|  | rs76469829 | 0.123 | 0.035 | 0.051 | 0.016 | 0.003 | 20.617 |
|  | rs76513766 | 0.096 | 0.011 | 0.088 | 0.276 | 0.003 | 20.216 |
|  | rs7936002 | -0.013 | 0.245 | 0.022 | 0.549 | 0.007 | 40.967 |
|  | rs79995028 | 0.110 | 0.004 | 0.155 | 0.479 | 0.003 | 20.562 |
|  | rs9967725 | -0.011 | 0.336 | 0.020 | 0.565 | 0.005 | 29.177 |
| Phosphatidylethanolamine (18:2_0:0) | rs11045856 | 0.025 | 0.172 | 0.025 | 0.319 | 0.004 | 26.652 |
|  | rs11063393 | -0.009 | 0.099 | 0.032 | 0.766 | 0.003 | 21.711 |
|  | rs111877698 | 0.076 | 0.012 | 0.090 | 0.401 | 0.003 | 23.786 |
|  | rs114899411 | 0.015 | 0.056 | 0.040 | 0.720 | 0.004 | 25.271 |
|  | rs115359603 | -0.051 | 0.014 | 0.079 | 0.516 | 0.003 | 20.766 |
|  | rs12459249 | -0.003 | 0.707 | 0.021 | 0.880 | 0.003 | 20.549 |
|  | rs1260326 | -0.030 | 0.650 | 0.020 | 0.124 | 0.006 | 41.704 |
|  | rs1338449 | -0.011 | 0.351 | 0.019 | 0.582 | 0.003 | 20.870 |
|  | rs1442407 | 0.037 | 0.183 | 0.024 | 0.129 | 0.004 | 27.510 |
|  | rs146330404 | 0.106 | 0.006 | 0.120 | 0.378 | 0.003 | 23.707 |
|  | rs174560 | 0.066 | 0.388 | 0.019 | 0.000 | 0.062 | 455.116 |
|  | rs1792909 | 0.018 | 0.393 | 0.019 | 0.359 | 0.004 | 26.569 |
|  | rs188404615 | 0.032 | 0.008 | 0.107 | 0.768 | 0.004 | 27.595 |
|  | rs2236950 | 0.014 | 0.259 | 0.021 | 0.525 | 0.003 | 20.689 |
|  | rs2290053 | -0.011 | 0.491 | 0.019 | 0.555 | 0.003 | 20.152 |
|  | rs326048 | -0.015 | 0.289 | 0.021 | 0.474 | 0.003 | 21.024 |
|  | rs35140833 | -0.059 | 0.162 | 0.025 | 0.021 | 0.003 | 22.357 |
|  | rs3741259 | 0.011 | 0.128 | 0.028 | 0.684 | 0.004 | 27.217 |
|  | rs4726320 | 0.025 | 0.896 | 0.031 | 0.421 | 0.003 | 20.578 |
|  | rs62076866 | 0.033 | 0.013 | 0.083 | 0.690 | 0.004 | 25.560 |
|  | rs62242707 | 0.029 | 0.268 | 0.021 | 0.164 | 0.003 | 20.408 |
|  | rs6413913 | -0.032 | 0.309 | 0.020 | 0.110 | 0.003 | 23.185 |
|  | rs71508993 | -0.018 | 0.029 | 0.055 | 0.750 | 0.003 | 20.575 |
|  | rs7776911 | 0.055 | 0.848 | 0.026 | 0.033 | 0.003 | 19.661 |
|  | rs791631 | -0.009 | 0.615 | 0.019 | 0.624 | 0.003 | 20.651 |
|  | rs79211442 | 0.061 | 0.128 | 0.028 | 0.029 | 0.003 | 19.687 |
|  | rs8034802 | -0.007 | 0.301 | 0.020 | 0.746 | 0.003 | 22.345 |
|  |  |  |  |  |  |  |  |
| Phosphatidylcholine (14:0_16:0) | rs1230353 | -0.020 | 0.181 | 0.024 | 0.416 | 0.004 | 20.902 |
|  | rs145618222 | 0.010 | 0.020 | 0.068 | 0.885 | 0.004 | 19.670 |
|  | rs148426771 | 0.078 | 0.047 | 0.045 | 0.083 | 0.004 | 20.369 |
|  | rs1553262 | -0.023 | 0.007 | 0.113 | 0.838 | 0.004 | 20.348 |
|  | rs17749540 | -0.011 | 0.163 | 0.025 | 0.652 | 0.004 | 20.121 |
|  | rs2014300 | 0.012 | 0.890 | 0.030 | 0.682 | 0.004 | 21.604 |
|  | rs28416404 | -0.006 | 0.080 | 0.035 | 0.852 | 0.004 | 21.959 |
|  | rs55654962 | -0.015 | 0.108 | 0.030 | 0.629 | 0.004 | 19.890 |
|  | rs6050 | -0.026 | 0.314 | 0.020 | 0.203 | 0.004 | 21.701 |
|  | rs60984271 | -0.016 | 0.223 | 0.022 | 0.476 | 0.004 | 19.892 |
|  | rs7075480 | -0.094 | 0.007 | 0.115 | 0.411 | 0.004 | 20.179 |
|  | rs7230887 | 0.006 | 0.196 | 0.024 | 0.788 | 0.004 | 19.677 |
|  | rs7239464 | -0.013 | 0.285 | 0.021 | 0.540 | 0.004 | 22.260 |
|  | rs72666927 | -0.004 | 0.043 | 0.046 | 0.938 | 0.004 | 20.132 |
|  | rs72764354 | 0.007 | 0.046 | 0.044 | 0.878 | 0.004 | 20.568 |
|  | rs74939791 | -0.011 | 0.028 | 0.057 | 0.852 | 0.004 | 22.608 |
|  | rs75414245 | -0.121 | 0.015 | 0.077 | 0.113 | 0.004 | 20.148 |
|  | rs77191266 | -0.067 | 0.034 | 0.052 | 0.197 | 0.004 | 21.104 |
|  | rs9507859 | -0.059 | 0.762 | 0.022 | 0.007 | 0.004 | 24.623 |
| Phosphatidylcholine (16:0_18:2) | rs10787711 | 0.012 | 0.641 | 0.020 | 0.549 | 0.004 | 26.467 |
|  | rs10890773 | 0.012 | 0.862 | 0.027 | 0.665 | 0.004 | 27.739 |
|  | rs10961143 | -0.006 | 0.049 | 0.043 | 0.881 | 0.004 | 25.349 |
|  | rs112569946 | 0.106 | 0.002 | 0.193 | 0.585 | 0.003 | 21.137 |
|  | rs11722429 | -0.044 | 0.102 | 0.031 | 0.158 | 0.003 | 22.462 |
|  | rs117528775 | 0.142 | 0.006 | 0.126 | 0.262 | 0.003 | 22.698 |
|  | rs13150924 | 0.028 | 0.122 | 0.029 | 0.331 | 0.004 | 29.063 |
|  | rs13402814 | -0.017 | 0.058 | 0.039 | 0.673 | 0.003 | 21.916 |
|  | rs143066473 | 0.032 | 0.025 | 0.061 | 0.607 | 0.003 | 21.157 |
|  | rs1488201 | -0.034 | 0.351 | 0.019 | 0.077 | 0.003 | 19.765 |
|  | rs150352222 | 0.016 | 0.016 | 0.074 | 0.832 | 0.003 | 19.757 |
|  | rs150376922 | 0.033 | 0.017 | 0.072 | 0.648 | 0.003 | 20.248 |
|  | rs1532085 | -0.020 | 0.578 | 0.019 | 0.298 | 0.006 | 43.461 |
|  | rs16985573 | 0.029 | 0.079 | 0.034 | 0.399 | 0.003 | 20.702 |
|  | rs17126640 | -0.025 | 0.193 | 0.024 | 0.292 | 0.003 | 22.734 |
|  | rs17231506 | 0.027 | 0.279 | 0.021 | 0.200 | 0.008 | 60.485 |
|  | rs174527 | 0.018 | 0.452 | 0.019 | 0.341 | 0.005 | 39.461 |
|  | rs174574 | -0.079 | 0.586 | 0.019 | 0.000 | 0.052 | 396.636 |
|  | rs1800588 | -0.012 | 0.248 | 0.022 | 0.586 | 0.007 | 50.839 |
|  | rs1833172 | -0.021 | 0.078 | 0.035 | 0.536 | 0.003 | 23.054 |
|  | rs187681244 | -0.070 | 0.008 | 0.107 | 0.511 | 0.003 | 20.214 |
|  | rs2629180 | 0.153 | 0.005 | 0.146 | 0.294 | 0.003 | 22.148 |
|  | rs35925446 | -0.004 | 0.069 | 0.037 | 0.918 | 0.003 | 20.755 |
|  | rs4412689 | 0.005 | 0.196 | 0.023 | 0.816 | 0.003 | 20.105 |
|  | rs4415100 | -0.018 | 0.209 | 0.023 | 0.419 | 0.003 | 20.770 |
|  | rs56075928 | 0.012 | 0.028 | 0.057 | 0.834 | 0.003 | 21.363 |
|  | rs62039480 | -0.007 | 0.328 | 0.020 | 0.712 | 0.006 | 42.774 |
|  | rs62330208 | -0.022 | 0.242 | 0.022 | 0.316 | 0.003 | 19.539 |
|  | rs6578729 | -0.060 | 0.084 | 0.034 | 0.075 | 0.003 | 20.131 |
|  | rs6673582 | -0.033 | 0.309 | 0.020 | 0.106 | 0.003 | 20.205 |
|  | rs73205054 | -0.056 | 0.035 | 0.051 | 0.268 | 0.003 | 20.459 |
|  | rs75833961 | -0.094 | 0.017 | 0.073 | 0.199 | 0.003 | 19.860 |
|  | rs7717591 | -0.007 | 0.681 | 0.020 | 0.744 | 0.003 | 20.430 |
|  | rs78882831 | 0.021 | 0.029 | 0.055 | 0.700 | 0.003 | 20.404 |
|  | rs7936002 | -0.013 | 0.245 | 0.022 | 0.549 | 0.004 | 25.605 |
|  | rs9436221 | -0.036 | 0.263 | 0.021 | 0.087 | 0.006 | 45.287 |
|  | rs964184 | 0.028 | 0.855 | 0.027 | 0.290 | 0.006 | 46.555 |
| Phosphatidylcholine (16:0_20:2) | rs10160784 | 0.050 | 0.231 | 0.022 | 0.023 | 0.004 | 30.408 |
|  | rs113130033 | -0.074 | 0.047 | 0.044 | 0.089 | 0.003 | 22.903 |
|  | rs113550786 | 0.126 | 0.014 | 0.081 | 0.121 | 0.003 | 22.568 |
|  | rs1168041 | 0.032 | 0.733 | 0.021 | 0.135 | 0.003 | 24.104 |
|  | rs1169289 | 0.008 | 0.465 | 0.019 | 0.666 | 0.003 | 20.130 |
|  | rs1260326 | -0.030 | 0.650 | 0.020 | 0.124 | 0.004 | 30.766 |
|  | rs12700446 | 0.043 | 0.019 | 0.068 | 0.524 | 0.003 | 20.966 |
|  | rs12761749 | 0.007 | 0.156 | 0.026 | 0.784 | 0.003 | 19.546 |
|  | rs138597 | 0.000 | 0.704 | 0.020 | 0.985 | 0.003 | 19.568 |
|  | rs142392117 | -0.034 | 0.020 | 0.067 | 0.608 | 0.003 | 23.319 |
|  | rs148069564 | -0.056 | 0.003 | 0.164 | 0.731 | 0.003 | 21.673 |
|  | rs150057262 | 0.035 | 0.050 | 0.043 | 0.415 | 0.004 | 27.319 |
|  | rs1532085 | -0.020 | 0.578 | 0.019 | 0.298 | 0.004 | 30.978 |
|  | rs1640963 | 0.004 | 0.188 | 0.024 | 0.859 | 0.003 | 19.580 |
|  | rs17126640 | -0.025 | 0.193 | 0.024 | 0.292 | 0.003 | 22.047 |
|  | rs17128436 | -0.005 | 0.015 | 0.076 | 0.953 | 0.003 | 20.138 |
|  | rs17268633 | -0.052 | 0.024 | 0.061 | 0.399 | 0.003 | 22.560 |
|  | rs174592 | 0.080 | 0.425 | 0.019 | 0.000 | 0.033 | 243.526 |
|  | rs1800588 | -0.012 | 0.248 | 0.022 | 0.586 | 0.004 | 30.277 |
|  | rs1815763 | -0.109 | 0.014 | 0.081 | 0.176 | 0.003 | 21.113 |
|  | rs2236514 | 0.013 | 0.654 | 0.020 | 0.506 | 0.004 | 25.807 |
|  | rs2317091 | -0.036 | 0.044 | 0.045 | 0.423 | 0.004 | 25.683 |
|  | rs3132935 | 0.005 | 0.169 | 0.025 | 0.828 | 0.003 | 20.010 |
|  | rs34516410 | 0.013 | 0.142 | 0.027 | 0.640 | 0.003 | 21.215 |
|  | rs62081852 | 0.087 | 0.020 | 0.067 | 0.197 | 0.005 | 33.040 |
|  | rs72800299 | -0.026 | 0.271 | 0.021 | 0.214 | 0.003 | 20.984 |
|  | rs76711488 | -0.014 | 0.025 | 0.060 | 0.818 | 0.003 | 21.493 |
|  | rs78230994 | -0.082 | 0.007 | 0.119 | 0.490 | 0.003 | 23.532 |
|  | rs79718024 | -0.002 | 0.155 | 0.026 | 0.948 | 0.003 | 22.875 |
| Phosphatidylcholine (16:0_20:4) | rs11062107 | -0.006 | 0.233 | 0.022 | 0.795 | 0.003 | 19.709 |
|  | rs113394924 | -0.064 | 0.083 | 0.034 | 0.058 | 0.007 | 51.998 |
|  | rs116438525 | 0.117 | 0.008 | 0.108 | 0.277 | 0.003 | 21.266 |
|  | rs116643980 | 0.028 | 0.054 | 0.041 | 0.506 | 0.003 | 20.924 |
|  | rs1251003 | 0.000 | 0.090 | 0.033 | 0.996 | 0.003 | 23.562 |
|  | rs12720922 | 0.013 | 0.173 | 0.025 | 0.588 | 0.003 | 19.717 |
|  | rs13329893 | 0.003 | 0.048 | 0.044 | 0.946 | 0.003 | 19.700 |
|  | rs141391095 | 0.025 | 0.025 | 0.059 | 0.670 | 0.003 | 22.132 |
|  | rs147549994 | 0.034 | 0.035 | 0.051 | 0.501 | 0.004 | 29.578 |
|  | rs149130695 | -0.005 | 0.064 | 0.039 | 0.890 | 0.003 | 19.659 |
|  | rs17017629 | -0.040 | 0.027 | 0.057 | 0.486 | 0.003 | 20.281 |
|  | rs17366743 | 0.020 | 0.036 | 0.050 | 0.695 | 0.003 | 19.594 |
|  | rs174527 | 0.018 | 0.452 | 0.019 | 0.341 | 0.015 | 111.162 |
|  | rs174533 | 0.082 | 0.415 | 0.019 | 0.000 | 0.171 | 1480.882 |
|  | rs187429064 | 0.024 | 0.051 | 0.043 | 0.573 | 0.005 | 36.009 |
|  | rs2943813 | 0.019 | 0.792 | 0.023 | 0.398 | 0.006 | 46.464 |
|  | rs2966427 | -0.041 | 0.929 | 0.036 | 0.263 | 0.003 | 20.014 |
|  | rs3018617 | 0.012 | 0.309 | 0.020 | 0.562 | 0.005 | 33.199 |
|  | rs3820438 | 0.024 | 0.288 | 0.021 | 0.248 | 0.003 | 23.703 |
|  | rs4756323 | -0.016 | 0.453 | 0.019 | 0.406 | 0.003 | 21.709 |
|  | rs499974 | 0.050 | 0.231 | 0.022 | 0.023 | 0.004 | 26.845 |
|  | rs58160859 | 0.017 | 0.210 | 0.023 | 0.471 | 0.003 | 20.602 |
|  | rs6498540 | -0.003 | 0.333 | 0.020 | 0.880 | 0.003 | 19.851 |
|  | rs72709529 | -0.048 | 0.022 | 0.064 | 0.452 | 0.003 | 23.734 |
|  | rs74754540 | 0.040 | 0.050 | 0.043 | 0.350 | 0.003 | 21.202 |
| Phosphatidylcholine (16:0_20:5) | rs102274 | 0.082 | 0.415 | 0.019 | 0.000 | 0.066 | 509.724 |
|  | rs10936925 | -0.027 | 0.344 | 0.020 | 0.162 | 0.003 | 19.759 |
|  | rs113394924 | -0.064 | 0.083 | 0.034 | 0.058 | 0.003 | 22.377 |
|  | rs113917790 | 0.016 | 0.011 | 0.089 | 0.859 | 0.003 | 22.160 |
|  | rs137945273 | -0.023 | 0.034 | 0.052 | 0.665 | 0.003 | 20.157 |
|  | rs146489454 | 0.011 | 0.027 | 0.057 | 0.848 | 0.003 | 19.667 |
|  | rs1481977 | -0.010 | 0.859 | 0.027 | 0.708 | 0.003 | 20.043 |
|  | rs174527 | 0.018 | 0.452 | 0.019 | 0.341 | 0.005 | 35.129 |
|  | rs2041149 | -0.015 | 0.464 | 0.019 | 0.414 | 0.003 | 22.029 |
|  | rs2733412 | -0.007 | 0.189 | 0.024 | 0.753 | 0.003 | 19.796 |
|  | rs2833287 | -0.197 | 0.005 | 0.126 | 0.119 | 0.003 | 19.817 |
|  | rs28569162 | 0.000 | 0.128 | 0.028 | 0.995 | 0.003 | 20.988 |
|  | rs3018617 | 0.012 | 0.309 | 0.020 | 0.562 | 0.003 | 24.625 |
|  | rs34822092 | -0.056 | 0.026 | 0.059 | 0.341 | 0.004 | 25.220 |
|  | rs3796575 | 0.029 | 0.233 | 0.022 | 0.187 | 0.003 | 20.371 |
|  | rs3936196 | 0.015 | 0.613 | 0.019 | 0.425 | 0.003 | 19.681 |
|  | rs4908782 | -0.012 | 0.346 | 0.020 | 0.525 | 0.003 | 20.799 |
|  | rs531117 | 0.050 | 0.231 | 0.022 | 0.023 | 0.003 | 19.795 |
|  | rs59839417 | -0.025 | 0.208 | 0.023 | 0.286 | 0.003 | 22.602 |
|  | rs61653779 | 0.034 | 0.016 | 0.073 | 0.639 | 0.003 | 20.516 |
|  | rs62025936 | 0.009 | 0.156 | 0.026 | 0.734 | 0.004 | 28.095 |
|  | rs74754540 | 0.040 | 0.050 | 0.043 | 0.350 | 0.003 | 23.640 |
|  | rs7780677 | -0.010 | 0.410 | 0.019 | 0.583 | 0.004 | 25.579 |
|  | rs7947954 | 0.021 | 0.153 | 0.026 | 0.426 | 0.003 | 21.974 |
|  | rs80083325 | -0.006 | 0.036 | 0.050 | 0.899 | 0.003 | 19.803 |
| Phosphatidylcholine (16:1_18:1) | rs11189347 | -0.008 | 0.135 | 0.027 | 0.774 | 0.003 | 22.141 |
|  | rs113366516 | -0.086 | 0.017 | 0.073 | 0.241 | 0.003 | 22.080 |
|  | rs116997061 | 0.002 | 0.061 | 0.039 | 0.965 | 0.003 | 21.844 |
|  | rs117217513 | -0.103 | 0.028 | 0.056 | 0.068 | 0.003 | 20.591 |
|  | rs1260326 | -0.030 | 0.650 | 0.020 | 0.124 | 0.006 | 39.803 |
|  | rs13402814 | -0.017 | 0.058 | 0.039 | 0.673 | 0.003 | 22.965 |
|  | rs139935251 | -0.100 | 0.003 | 0.185 | 0.591 | 0.003 | 20.947 |
|  | rs141084476 | 0.028 | 0.044 | 0.046 | 0.540 | 0.003 | 20.505 |
|  | rs145551604 | 0.023 | 0.075 | 0.035 | 0.516 | 0.003 | 22.598 |
|  | rs146934967 | -0.105 | 0.015 | 0.079 | 0.180 | 0.004 | 31.725 |
|  | rs1532085 | -0.020 | 0.578 | 0.019 | 0.298 | 0.004 | 30.310 |
|  | rs174560 | 0.066 | 0.388 | 0.019 | 0.000 | 0.014 | 104.489 |
|  | rs185867215 | 0.147 | 0.007 | 0.111 | 0.183 | 0.003 | 20.713 |
|  | rs36106314 | 0.031 | 0.253 | 0.022 | 0.152 | 0.003 | 19.689 |
|  | rs4447106 | -0.029 | 0.142 | 0.027 | 0.276 | 0.004 | 27.925 |
|  | rs56156922 | 0.027 | 0.279 | 0.021 | 0.188 | 0.004 | 29.434 |
|  | rs56316064 | -0.017 | 0.282 | 0.021 | 0.406 | 0.003 | 23.080 |
|  | rs5865 | -0.007 | 0.708 | 0.020 | 0.748 | 0.003 | 22.502 |
|  | rs603424 | -0.077 | 0.117 | 0.030 | 0.009 | 0.006 | 46.092 |
|  | rs7007401 | -0.025 | 0.276 | 0.021 | 0.225 | 0.003 | 19.699 |
|  | rs701919 | -0.036 | 0.784 | 0.023 | 0.115 | 0.003 | 20.845 |
|  | rs72834725 | 0.064 | 0.043 | 0.046 | 0.164 | 0.003 | 21.892 |
|  | rs74357965 | -0.004 | 0.104 | 0.031 | 0.890 | 0.003 | 20.189 |
|  | rs75102979 | -0.013 | 0.044 | 0.047 | 0.787 | 0.003 | 19.814 |
|  | rs7968329 | -0.002 | 0.441 | 0.019 | 0.923 | 0.003 | 20.372 |
|  | rs80160469 | -0.017 | 0.037 | 0.050 | 0.738 | 0.003 | 20.678 |
|  | rs8033940 | -0.012 | 0.326 | 0.020 | 0.551 | 0.004 | 27.057 |
|  | rs8100204 | 0.037 | 0.158 | 0.026 | 0.148 | 0.004 | 25.677 |
| Phosphatidylcholine (16:1_18:2) | rs10201921 | -0.016 | 0.058 | 0.040 | 0.690 | 0.003 | 19.923 |
|  | rs10890773 | 0.012 | 0.862 | 0.027 | 0.665 | 0.003 | 20.933 |
|  | rs11602429 | 0.036 | 0.131 | 0.028 | 0.195 | 0.003 | 20.682 |
|  | rs12031139 | -0.021 | 0.487 | 0.019 | 0.250 | 0.003 | 19.941 |
|  | rs1260326 | -0.030 | 0.650 | 0.020 | 0.124 | 0.004 | 31.914 |
|  | rs13150924 | 0.028 | 0.122 | 0.029 | 0.331 | 0.003 | 23.351 |
|  | rs146934967 | -0.105 | 0.015 | 0.079 | 0.180 | 0.005 | 36.173 |
|  | rs150057262 | 0.035 | 0.050 | 0.043 | 0.415 | 0.004 | 25.955 |
|  | rs1532085 | -0.020 | 0.578 | 0.019 | 0.298 | 0.005 | 33.603 |
|  | rs174527 | 0.018 | 0.452 | 0.019 | 0.341 | 0.007 | 48.162 |
|  | rs174574 | -0.079 | 0.586 | 0.019 | 0.000 | 0.064 | 491.373 |
|  | rs1800588 | -0.012 | 0.248 | 0.022 | 0.586 | 0.005 | 35.855 |
|  | rs184289628 | -0.141 | 0.020 | 0.067 | 0.034 | 0.003 | 20.714 |
|  | rs216958 | -0.024 | 0.622 | 0.019 | 0.207 | 0.003 | 21.076 |
|  | rs4122352 | 0.007 | 0.287 | 0.021 | 0.729 | 0.003 | 22.665 |
|  | rs56156922 | 0.027 | 0.279 | 0.021 | 0.188 | 0.005 | 37.774 |
|  | rs603424 | -0.077 | 0.117 | 0.030 | 0.009 | 0.005 | 38.713 |
|  | rs62104079 | 0.003 | 0.229 | 0.022 | 0.888 | 0.004 | 26.773 |
|  | rs6876235 | -0.008 | 0.897 | 0.031 | 0.803 | 0.003 | 21.352 |
|  | rs7036554 | -0.005 | 0.341 | 0.020 | 0.783 | 0.003 | 19.707 |
|  | rs7783799 | 0.049 | 0.305 | 0.020 | 0.016 | 0.003 | 22.093 |
|  | rs77949981 | -0.075 | 0.022 | 0.064 | 0.241 | 0.003 | 20.439 |
|  | rs78575494 | 0.058 | 0.037 | 0.049 | 0.238 | 0.003 | 20.013 |
|  | rs7936002 | -0.013 | 0.245 | 0.022 | 0.549 | 0.005 | 34.592 |
|  | rs9480322 | 0.016 | 0.031 | 0.054 | 0.765 | 0.003 | 24.354 |
|  | rs9839380 | -0.041 | 0.335 | 0.020 | 0.035 | 0.004 | 29.301 |
| Phosphatidylcholine (17:0_18:2) | rs11027519 | -0.030 | 0.091 | 0.032 | 0.351 | 0.003 | 19.950 |
|  | rs111233850 | 0.109 | 0.032 | 0.053 | 0.040 | 0.003 | 20.199 |
|  | rs111314022 | 0.053 | 0.032 | 0.053 | 0.317 | 0.003 | 20.345 |
|  | rs115686032 | -0.077 | 0.041 | 0.047 | 0.101 | 0.003 | 21.606 |
|  | rs1168104 | 0.035 | 0.736 | 0.021 | 0.098 | 0.003 | 20.877 |
|  | rs1253283 | 0.101 | 0.030 | 0.055 | 0.064 | 0.003 | 19.660 |
|  | rs12548274 | 0.008 | 0.163 | 0.025 | 0.751 | 0.003 | 22.095 |
|  | rs141509040 | -0.003 | 0.073 | 0.036 | 0.923 | 0.003 | 20.086 |
|  | rs1609446 | 0.069 | 0.081 | 0.034 | 0.045 | 0.003 | 19.967 |
|  | rs17231506 | 0.027 | 0.279 | 0.021 | 0.200 | 0.004 | 27.186 |
|  | rs174566 | 0.079 | 0.413 | 0.019 | 0.000 | 0.032 | 237.533 |
|  | rs1800588 | -0.012 | 0.248 | 0.022 | 0.586 | 0.005 | 35.068 |
|  | rs192537901 | 0.072 | 0.028 | 0.056 | 0.203 | 0.003 | 23.785 |
|  | rs2126260 | -0.005 | 0.747 | 0.021 | 0.810 | 0.003 | 22.916 |
|  | rs34489224 | 0.032 | 0.273 | 0.021 | 0.133 | 0.003 | 21.413 |
|  | rs422137 | 0.016 | 0.487 | 0.019 | 0.389 | 0.004 | 25.903 |
|  | rs4478142 | 0.016 | 0.157 | 0.026 | 0.537 | 0.003 | 20.039 |
|  | rs4840981 | -0.054 | 0.988 | 0.087 | 0.531 | 0.003 | 21.265 |
|  | rs57518551 | 0.040 | 0.142 | 0.027 | 0.130 | 0.004 | 25.838 |
|  | rs6498540 | -0.003 | 0.333 | 0.020 | 0.880 | 0.004 | 25.828 |
|  | rs72747028 | -0.023 | 0.068 | 0.037 | 0.534 | 0.003 | 21.691 |
|  | rs73058910 | -0.009 | 0.067 | 0.037 | 0.807 | 0.003 | 21.390 |
|  | rs7437408 | 0.018 | 0.481 | 0.019 | 0.345 | 0.003 | 21.048 |
|  | rs78592970 | -0.017 | 0.123 | 0.029 | 0.549 | 0.004 | 25.234 |
|  | rs964184 | 0.028 | 0.855 | 0.027 | 0.290 | 0.006 | 44.464 |
| Phosphatidylcholine (17:0_20:4) | rs11088781 | -0.001 | 0.910 | 0.033 | 0.979 | 0.003 | 22.380 |
|  | rs113394924 | -0.064 | 0.083 | 0.034 | 0.058 | 0.006 | 42.375 |
|  | rs11626405 | 0.017 | 0.292 | 0.020 | 0.405 | 0.003 | 20.147 |
|  | rs116643980 | 0.028 | 0.054 | 0.041 | 0.506 | 0.003 | 21.001 |
|  | rs11673120 | -0.003 | 0.414 | 0.019 | 0.894 | 0.003 | 20.096 |
|  | rs1168041 | 0.032 | 0.733 | 0.021 | 0.135 | 0.003 | 22.013 |
|  | rs117580248 | -0.045 | 0.115 | 0.030 | 0.133 | 0.003 | 21.992 |
|  | rs12429208 | 0.014 | 0.274 | 0.021 | 0.512 | 0.003 | 21.408 |
|  | rs12928399 | 0.032 | 0.348 | 0.020 | 0.097 | 0.003 | 23.814 |
|  | rs140619100 | 0.008 | 0.055 | 0.041 | 0.849 | 0.006 | 39.675 |
|  | rs142082962 | -0.002 | 0.077 | 0.035 | 0.951 | 0.003 | 21.567 |
|  | rs143060591 | 0.002 | 0.070 | 0.037 | 0.946 | 0.003 | 21.432 |
|  | rs143309746 | -0.150 | 0.023 | 0.062 | 0.016 | 0.003 | 20.883 |
|  | rs174527 | 0.018 | 0.452 | 0.019 | 0.341 | 0.015 | 105.864 |
|  | rs174533 | 0.082 | 0.415 | 0.019 | 0.000 | 0.140 | 1159.576 |
|  | rs187429064 | 0.024 | 0.051 | 0.043 | 0.573 | 0.006 | 39.655 |
|  | rs190664666 | -0.047 | 0.059 | 0.039 | 0.236 | 0.003 | 20.860 |
|  | rs2398428 | 0.004 | 0.261 | 0.021 | 0.842 | 0.003 | 24.175 |
|  | rs2469035 | 0.024 | 0.794 | 0.023 | 0.297 | 0.003 | 20.306 |
|  | rs3110971 | 0.006 | 0.205 | 0.023 | 0.779 | 0.003 | 22.849 |
|  | rs35788378 | -0.010 | 0.267 | 0.021 | 0.638 | 0.003 | 20.673 |
|  | rs55725503 | -0.063 | 0.022 | 0.064 | 0.326 | 0.003 | 20.606 |
|  | rs56399423 | 0.090 | 0.316 | 0.020 | 0.000 | 0.003 | 22.206 |
|  | rs57815521 | 0.152 | 0.029 | 0.053 | 0.004 | 0.004 | 31.489 |
|  | rs585002 | -0.072 | 0.859 | 0.026 | 0.007 | 0.003 | 22.327 |
|  | rs600518 | 0.050 | 0.231 | 0.022 | 0.023 | 0.005 | 32.226 |
|  | rs6083838 | 0.034 | 0.533 | 0.019 | 0.065 | 0.003 | 21.062 |
|  | rs61896563 | 0.018 | 0.052 | 0.042 | 0.671 | 0.003 | 22.214 |
|  | rs635196 | -0.004 | 0.177 | 0.024 | 0.871 | 0.003 | 21.333 |
|  | rs73161879 | 0.007 | 0.180 | 0.024 | 0.777 | 0.003 | 21.407 |
|  | rs74452899 | 0.077 | 0.011 | 0.089 | 0.388 | 0.004 | 25.447 |
| Phosphatidylcholine (18:0_20:4) | rs1219539 | 0.026 | 0.491 | 0.019 | 0.167 | 0.004 | 27.346 |
|  | rs12275418 | -0.023 | 0.134 | 0.027 | 0.401 | 0.004 | 25.436 |
|  | rs139102782 | -0.047 | 0.034 | 0.052 | 0.366 | 0.003 | 19.724 |
|  | rs144719617 | 0.018 | 0.105 | 0.030 | 0.548 | 0.003 | 22.050 |
|  | rs147549994 | 0.034 | 0.035 | 0.051 | 0.501 | 0.004 | 32.312 |
|  | rs1477491 | 0.027 | 0.798 | 0.023 | 0.241 | 0.004 | 25.279 |
|  | rs147981159 | 0.003 | 0.044 | 0.046 | 0.941 | 0.009 | 68.422 |
|  | rs174528 | 0.081 | 0.420 | 0.019 | 0.000 | 0.213 | 1945.604 |
|  | rs187429064 | 0.024 | 0.051 | 0.043 | 0.573 | 0.004 | 29.000 |
|  | rs2070895 | -0.016 | 0.258 | 0.021 | 0.462 | 0.003 | 24.226 |
|  | rs223841 | 0.038 | 0.169 | 0.025 | 0.124 | 0.003 | 21.924 |
|  | rs2797620 | -0.002 | 0.329 | 0.020 | 0.937 | 0.005 | 33.869 |
|  | rs2802390 | -0.005 | 0.463 | 0.019 | 0.796 | 0.003 | 20.080 |
|  | rs2990536 | 0.021 | 0.581 | 0.019 | 0.260 | 0.003 | 21.561 |
|  | rs3019200 | 0.027 | 0.808 | 0.024 | 0.248 | 0.008 | 58.257 |
|  | rs3110971 | 0.006 | 0.205 | 0.023 | 0.779 | 0.003 | 22.029 |
|  | rs3741252 | -0.077 | 0.129 | 0.028 | 0.007 | 0.013 | 98.083 |
|  | rs4382917 | -0.012 | 0.243 | 0.022 | 0.586 | 0.008 | 57.065 |
|  | rs600518 | 0.050 | 0.231 | 0.022 | 0.023 | 0.004 | 25.996 |
|  | rs61873274 | 0.059 | 0.076 | 0.035 | 0.092 | 0.003 | 20.835 |
|  | rs6860806 | 0.057 | 0.418 | 0.019 | 0.003 | 0.003 | 21.989 |
|  | rs72789541 | 0.000 | 0.316 | 0.020 | 0.983 | 0.004 | 29.299 |
|  | rs73005469 | -0.011 | 0.360 | 0.019 | 0.575 | 0.003 | 19.653 |
|  | rs7499892 | 0.014 | 0.172 | 0.025 | 0.564 | 0.003 | 19.697 |
|  | rs79136768 | 0.057 | 0.036 | 0.050 | 0.258 | 0.004 | 30.967 |
|  | rs793864 | -0.007 | 0.818 | 0.024 | 0.768 | 0.003 | 23.174 |
|  | rs938822 | -0.021 | 0.146 | 0.026 | 0.417 | 0.003 | 20.998 |
|  | rs9462860 | 0.000 | 0.449 | 0.019 | 0.998 | 0.003 | 21.000 |
| Phosphatidylcholine (18:0_20:5) | rs102275 | 0.083 | 0.416 | 0.019 | 0.000 | 0.084 | 649.745 |
|  | rs1076708 | -0.015 | 0.421 | 0.019 | 0.434 | 0.003 | 19.549 |
|  | rs12487951 | -0.027 | 0.027 | 0.058 | 0.638 | 0.003 | 19.697 |
|  | rs12656795 | -0.012 | 0.400 | 0.019 | 0.541 | 0.003 | 19.909 |
|  | rs138053477 | -0.053 | 0.041 | 0.047 | 0.256 | 0.004 | 25.613 |
|  | rs145259113 | 0.068 | 0.006 | 0.126 | 0.591 | 0.003 | 20.135 |
|  | rs146438407 | -0.081 | 0.008 | 0.109 | 0.456 | 0.003 | 20.446 |
|  | rs174527 | 0.018 | 0.452 | 0.019 | 0.341 | 0.006 | 45.386 |
|  | rs187698886 | -0.010 | 0.036 | 0.051 | 0.849 | 0.003 | 19.710 |
|  | rs2023988 | -0.009 | 0.423 | 0.019 | 0.650 | 0.003 | 20.426 |
|  | rs2054498 | -0.003 | 0.538 | 0.019 | 0.856 | 0.003 | 21.989 |
|  | rs2135279 | 0.006 | 0.582 | 0.019 | 0.748 | 0.003 | 19.641 |
|  | rs2431163 | 0.042 | 0.026 | 0.060 | 0.485 | 0.003 | 21.969 |
|  | rs3019200 | 0.027 | 0.808 | 0.024 | 0.248 | 0.004 | 25.403 |
|  | rs35226034 | -0.036 | 0.037 | 0.049 | 0.461 | 0.003 | 22.141 |
|  | rs4908782 | -0.012 | 0.346 | 0.020 | 0.525 | 0.003 | 19.747 |
|  | rs508049 | 0.019 | 0.093 | 0.032 | 0.556 | 0.003 | 23.885 |
|  | rs5896690 | 0.058 | 0.465 | 0.019 | 0.002 | 0.003 | 21.793 |
|  | rs61653779 | 0.034 | 0.016 | 0.073 | 0.639 | 0.003 | 20.827 |
|  | rs62025936 | 0.009 | 0.156 | 0.026 | 0.734 | 0.004 | 29.329 |
|  | rs71629271 | 0.011 | 0.069 | 0.037 | 0.757 | 0.003 | 21.860 |
|  | rs7780677 | -0.010 | 0.410 | 0.019 | 0.583 | 0.003 | 22.178 |
|  | rs7936002 | -0.013 | 0.245 | 0.022 | 0.549 | 0.004 | 31.347 |
|  | rs80116939 | 0.064 | 0.004 | 0.162 | 0.695 | 0.003 | 23.961 |
|  | rs9380073 | 0.015 | 0.127 | 0.028 | 0.596 | 0.003 | 22.080 |
|  | rs9587408 | -0.034 | 0.236 | 0.022 | 0.126 | 0.003 | 20.917 |
| Phosphatidylcholine (18:1_18:1) | rs10145681 | -0.032 | 0.974 | 0.058 | 0.586 | 0.003 | 22.339 |
|  | rs1077834 | -0.016 | 0.257 | 0.021 | 0.453 | 0.003 | 23.102 |
|  | rs10779836 | 0.007 | 0.771 | 0.022 | 0.745 | 0.003 | 21.236 |
|  | rs10850057 | -0.002 | 0.090 | 0.033 | 0.950 | 0.003 | 19.897 |
|  | rs10889352 | -0.036 | 0.264 | 0.021 | 0.091 | 0.005 | 35.528 |
|  | rs113277188 | 0.046 | 0.020 | 0.068 | 0.494 | 0.003 | 19.700 |
|  | rs13278111 | -0.007 | 0.429 | 0.019 | 0.715 | 0.003 | 20.215 |
|  | rs136061 | 0.010 | 0.255 | 0.021 | 0.647 | 0.003 | 22.262 |
|  | rs140268092 | -0.111 | 0.005 | 0.130 | 0.396 | 0.003 | 20.673 |
|  | rs144512980 | 0.219 | 0.011 | 0.083 | 0.008 | 0.004 | 28.815 |
|  | rs1532085 | -0.020 | 0.578 | 0.019 | 0.298 | 0.006 | 42.516 |
|  | rs17231506 | 0.027 | 0.279 | 0.021 | 0.200 | 0.005 | 34.683 |
|  | rs174574 | -0.079 | 0.586 | 0.019 | 0.000 | 0.020 | 142.818 |
|  | rs2000827 | 0.030 | 0.890 | 0.030 | 0.321 | 0.003 | 20.143 |
|  | rs2126263 | 0.039 | 0.854 | 0.026 | 0.143 | 0.003 | 22.694 |
|  | rs3741298 | 0.045 | 0.768 | 0.022 | 0.043 | 0.003 | 20.792 |
|  | rs41391244 | 0.029 | 0.234 | 0.022 | 0.189 | 0.003 | 21.387 |
|  | rs41441450 | 0.152 | 0.010 | 0.095 | 0.109 | 0.003 | 21.327 |
|  | rs4848460 | 0.046 | 0.055 | 0.041 | 0.262 | 0.003 | 20.844 |
|  | rs56057593 | 0.038 | 0.029 | 0.056 | 0.501 | 0.003 | 21.153 |
|  | rs7300883 | -0.017 | 0.439 | 0.019 | 0.377 | 0.003 | 20.300 |
|  | rs73241068 | -0.011 | 0.034 | 0.051 | 0.825 | 0.003 | 22.775 |
|  | rs79008894 | -0.055 | 0.035 | 0.051 | 0.284 | 0.003 | 20.657 |
| Phosphatidylcholine (18:1_18:2) | rs1077835 | -0.016 | 0.257 | 0.021 | 0.452 | 0.006 | 42.386 |
|  | rs10889352 | -0.036 | 0.264 | 0.021 | 0.091 | 0.006 | 45.370 |
|  | rs11757990 | -0.080 | 0.006 | 0.122 | 0.514 | 0.003 | 19.619 |
|  | rs118108980 | -0.004 | 0.188 | 0.024 | 0.857 | 0.003 | 22.696 |
|  | rs12798268 | -0.004 | 0.864 | 0.027 | 0.876 | 0.003 | 20.165 |
|  | rs13128807 | 0.026 | 0.187 | 0.024 | 0.269 | 0.003 | 22.814 |
|  | rs142689129 | 0.372 | 0.003 | 0.147 | 0.011 | 0.003 | 24.817 |
|  | rs147761802 | -0.078 | 0.054 | 0.041 | 0.057 | 0.003 | 21.272 |
|  | rs1601935 | -0.036 | 0.616 | 0.019 | 0.064 | 0.005 | 38.658 |
|  | rs17231506 | 0.027 | 0.279 | 0.021 | 0.200 | 0.006 | 41.820 |
|  | rs174527 | 0.018 | 0.452 | 0.019 | 0.341 | 0.004 | 28.211 |
|  | rs174574 | -0.079 | 0.586 | 0.019 | 0.000 | 0.069 | 535.116 |
|  | rs2236605 | -0.043 | 0.422 | 0.019 | 0.024 | 0.004 | 25.246 |
|  | rs28844909 | -0.007 | 0.066 | 0.038 | 0.851 | 0.003 | 20.197 |
|  | rs4944732 | -0.025 | 0.135 | 0.027 | 0.364 | 0.003 | 19.897 |
|  | rs564216 | -0.008 | 0.050 | 0.043 | 0.851 | 0.003 | 21.058 |
|  | rs6023985 | 0.159 | 0.014 | 0.080 | 0.047 | 0.003 | 21.244 |
|  | rs613808 | 0.012 | 0.625 | 0.019 | 0.522 | 0.004 | 27.730 |
|  | rs62004153 | 0.007 | 0.908 | 0.032 | 0.838 | 0.003 | 19.693 |
|  | rs6498540 | -0.003 | 0.333 | 0.020 | 0.880 | 0.003 | 20.039 |
|  | rs7036554 | -0.005 | 0.341 | 0.020 | 0.783 | 0.004 | 26.696 |
|  | rs78882831 | 0.021 | 0.029 | 0.055 | 0.700 | 0.003 | 19.861 |
|  | rs948727 | -0.001 | 0.329 | 0.020 | 0.948 | 0.003 | 21.994 |
|  | rs9789400 | 0.060 | 0.276 | 0.021 | 0.004 | 0.003 | 22.021 |
| Phosphatidylcholine (18:1_20:2) | rs10846753 | 0.011 | 0.231 | 0.022 | 0.628 | 0.004 | 24.351 |
|  | rs114139553 | 0.111 | 0.013 | 0.082 | 0.175 | 0.004 | 21.015 |
|  | rs116922907 | -0.060 | 0.045 | 0.045 | 0.188 | 0.004 | 20.854 |
|  | rs117534325 | -0.130 | 0.005 | 0.141 | 0.355 | 0.004 | 22.223 |
|  | rs118150981 | 0.118 | 0.020 | 0.068 | 0.081 | 0.004 | 21.051 |
|  | rs12113068 | -0.016 | 0.023 | 0.062 | 0.795 | 0.003 | 19.669 |
|  | rs12417747 | 0.032 | 0.115 | 0.029 | 0.281 | 0.007 | 43.007 |
|  | rs174527 | 0.018 | 0.452 | 0.019 | 0.341 | 0.004 | 24.547 |
|  | rs174580 | 0.082 | 0.415 | 0.019 | 0.000 | 0.090 | 580.696 |
|  | rs17543793 | 0.012 | 0.020 | 0.067 | 0.855 | 0.003 | 20.322 |
|  | rs1800588 | -0.012 | 0.248 | 0.022 | 0.586 | 0.005 | 27.543 |
|  | rs265114 | 0.019 | 0.074 | 0.036 | 0.592 | 0.004 | 23.847 |
|  | rs35195855 | -0.021 | 0.143 | 0.027 | 0.444 | 0.004 | 21.787 |
|  | rs56208677 | 0.010 | 0.076 | 0.035 | 0.776 | 0.005 | 26.554 |
|  | rs61893829 | -0.024 | 0.086 | 0.034 | 0.469 | 0.004 | 21.308 |
|  | rs656095 | 0.051 | 0.222 | 0.022 | 0.023 | 0.006 | 34.601 |
|  | rs6944011 | -0.010 | 0.208 | 0.023 | 0.651 | 0.003 | 20.087 |
|  | rs6984305 | 0.030 | 0.841 | 0.025 | 0.239 | 0.004 | 23.463 |
|  | rs7041283 | -0.025 | 0.246 | 0.022 | 0.248 | 0.005 | 29.495 |
|  | rs7176661 | -0.011 | 0.597 | 0.019 | 0.568 | 0.003 | 19.838 |
|  | rs73292027 | 0.003 | 0.105 | 0.030 | 0.935 | 0.004 | 24.859 |
|  | rs74089903 | -0.133 | 0.009 | 0.100 | 0.183 | 0.005 | 27.553 |
|  | rs80201535 | -0.037 | 0.028 | 0.057 | 0.516 | 0.004 | 21.230 |
|  | rs9384216 | 0.006 | 0.098 | 0.031 | 0.859 | 0.003 | 20.209 |
| Phosphatidylcholine (18:1_20:4) | rs1077834 | -0.016 | 0.257 | 0.021 | 0.453 | 0.004 | 26.370 |
|  | rs10793123 | 0.032 | 0.487 | 0.019 | 0.089 | 0.003 | 20.821 |
|  | rs10889353 | -0.036 | 0.264 | 0.021 | 0.086 | 0.003 | 20.530 |
|  | rs113049169 | -0.095 | 0.032 | 0.053 | 0.073 | 0.003 | 19.680 |
|  | rs113394924 | -0.064 | 0.083 | 0.034 | 0.058 | 0.005 | 39.462 |
|  | rs113913626 | -0.003 | 0.035 | 0.051 | 0.958 | 0.003 | 19.533 |
|  | rs116814948 | -0.012 | 0.010 | 0.095 | 0.899 | 0.003 | 20.447 |
|  | rs147549994 | 0.034 | 0.035 | 0.051 | 0.501 | 0.004 | 26.191 |
|  | rs147797702 | -0.037 | 0.010 | 0.092 | 0.690 | 0.004 | 30.191 |
|  | rs173539 | 0.028 | 0.283 | 0.021 | 0.175 | 0.003 | 21.167 |
|  | rs174527 | 0.018 | 0.452 | 0.019 | 0.341 | 0.012 | 89.070 |
|  | rs174537 | 0.077 | 0.413 | 0.019 | 0.000 | 0.123 | 1006.817 |
|  | rs187429064 | 0.024 | 0.051 | 0.043 | 0.573 | 0.007 | 53.611 |
|  | rs1917701 | 0.064 | 0.945 | 0.041 | 0.121 | 0.003 | 20.013 |
|  | rs2943813 | 0.019 | 0.792 | 0.023 | 0.398 | 0.006 | 40.322 |
|  | rs3018617 | 0.012 | 0.309 | 0.020 | 0.562 | 0.003 | 23.807 |
|  | rs4628166 | 0.000 | 0.631 | 0.019 | 0.979 | 0.003 | 19.699 |
|  | rs4846905 | 0.023 | 0.765 | 0.022 | 0.295 | 0.003 | 23.180 |
|  | rs531117 | 0.050 | 0.231 | 0.022 | 0.023 | 0.005 | 32.924 |
|  | rs6083838 | 0.034 | 0.533 | 0.019 | 0.065 | 0.003 | 19.625 |
|  | rs61978272 | -0.009 | 0.276 | 0.021 | 0.677 | 0.004 | 27.749 |
|  | rs641738 | 0.006 | 0.604 | 0.019 | 0.746 | 0.003 | 24.969 |
|  | rs6850591 | 0.015 | 0.549 | 0.019 | 0.439 | 0.003 | 23.007 |
|  | rs73176681 | 0.019 | 0.047 | 0.044 | 0.661 | 0.003 | 20.807 |
|  | rs73618588 | 0.014 | 0.090 | 0.033 | 0.659 | 0.003 | 20.508 |
|  | rs75976069 | -0.097 | 0.026 | 0.059 | 0.102 | 0.003 | 23.447 |
|  | rs76582138 | -0.104 | 0.009 | 0.102 | 0.307 | 0.003 | 20.340 |
|  | rs79113397 | 0.019 | 0.234 | 0.022 | 0.394 | 0.003 | 24.493 |
|  | rs793864 | -0.007 | 0.818 | 0.024 | 0.768 | 0.003 | 21.873 |
|  | rs8107974 | 0.019 | 0.064 | 0.038 | 0.618 | 0.004 | 30.771 |
| Phosphatidylcholine (18:2_18:2) | rs1077835 | -0.016 | 0.257 | 0.021 | 0.452 | 0.005 | 35.494 |
|  | rs1168127 | 0.035 | 0.736 | 0.021 | 0.094 | 0.003 | 23.658 |
|  | rs117017186 | -0.098 | 0.011 | 0.090 | 0.277 | 0.003 | 20.740 |
|  | rs117675848 | -0.021 | 0.018 | 0.071 | 0.765 | 0.003 | 24.053 |
|  | rs11869356 | -0.028 | 0.589 | 0.019 | 0.139 | 0.003 | 20.029 |
|  | rs13078306 | 0.005 | 0.334 | 0.020 | 0.817 | 0.003 | 21.544 |
|  | rs13150924 | 0.028 | 0.122 | 0.029 | 0.331 | 0.003 | 19.901 |
|  | rs139598970 | -0.008 | 0.014 | 0.080 | 0.922 | 0.003 | 22.161 |
|  | rs147002062 | -0.010 | 0.003 | 0.163 | 0.952 | 0.003 | 20.954 |
|  | rs148498852 | 0.014 | 0.081 | 0.034 | 0.678 | 0.003 | 22.909 |
|  | rs1601935 | -0.036 | 0.616 | 0.019 | 0.064 | 0.004 | 30.334 |
|  | rs173539 | 0.028 | 0.283 | 0.021 | 0.175 | 0.004 | 28.395 |
|  | rs174574 | -0.079 | 0.586 | 0.019 | 0.000 | 0.024 | 173.147 |
|  | rs2236605 | -0.043 | 0.422 | 0.019 | 0.024 | 0.003 | 20.451 |
|  | rs35958132 | 0.008 | 0.065 | 0.038 | 0.831 | 0.003 | 21.078 |
|  | rs4271965 | -0.019 | 0.568 | 0.019 | 0.307 | 0.003 | 22.475 |
|  | rs56158036 | 0.127 | 0.017 | 0.073 | 0.082 | 0.003 | 21.504 |
|  | rs59256568 | -0.122 | 0.020 | 0.067 | 0.069 | 0.003 | 22.320 |
|  | rs62238391 | -0.005 | 0.319 | 0.020 | 0.811 | 0.003 | 23.335 |
|  | rs6498540 | -0.003 | 0.333 | 0.020 | 0.880 | 0.004 | 27.719 |
|  | rs7122803 | 0.012 | 0.862 | 0.027 | 0.668 | 0.003 | 20.412 |
|  | rs7717591 | -0.007 | 0.681 | 0.020 | 0.744 | 0.004 | 28.761 |
|  | rs7798734 | 0.012 | 0.318 | 0.020 | 0.559 | 0.003 | 21.438 |
|  | rs80315588 | 0.002 | 0.427 | 0.019 | 0.930 | 0.003 | 20.805 |
| Phosphatidylcholine (O-16:0_18:2) | rs11777584 | -0.001 | 0.338 | 0.020 | 0.954 | 0.003 | 19.543 |
|  | rs11918522 | 0.037 | 0.162 | 0.025 | 0.141 | 0.003 | 21.241 |
|  | rs11936543 | 0.018 | 0.095 | 0.032 | 0.578 | 0.003 | 19.745 |
|  | rs140661870 | 0.067 | 0.014 | 0.082 | 0.414 | 0.003 | 20.925 |
|  | rs149918547 | 0.049 | 0.005 | 0.129 | 0.703 | 0.003 | 19.538 |
|  | rs1532085 | -0.020 | 0.578 | 0.019 | 0.298 | 0.004 | 26.051 |
|  | rs17086339 | -0.002 | 0.136 | 0.027 | 0.952 | 0.003 | 22.068 |
|  | rs17231506 | 0.027 | 0.279 | 0.021 | 0.200 | 0.006 | 42.917 |
|  | rs174574 | -0.079 | 0.586 | 0.019 | 0.000 | 0.016 | 114.535 |
|  | rs181750334 | 0.066 | 0.009 | 0.102 | 0.516 | 0.003 | 20.828 |
|  | rs2537611 | 0.001 | 0.374 | 0.019 | 0.939 | 0.003 | 20.244 |
|  | rs4579316 | -0.059 | 0.833 | 0.025 | 0.017 | 0.003 | 22.981 |
|  | rs6582326 | 0.057 | 0.965 | 0.052 | 0.271 | 0.003 | 20.560 |
|  | rs6741227 | 0.027 | 0.331 | 0.020 | 0.178 | 0.003 | 21.993 |
|  | rs6764912 | -0.063 | 0.085 | 0.034 | 0.061 | 0.003 | 20.284 |
|  | rs75804573 | 0.024 | 0.151 | 0.026 | 0.349 | 0.003 | 21.057 |
|  | rs75863117 | 0.044 | 0.093 | 0.032 | 0.176 | 0.003 | 19.766 |
|  | rs76888052 | -0.005 | 0.005 | 0.137 | 0.968 | 0.003 | 19.960 |
|  | rs78285907 | 0.037 | 0.088 | 0.033 | 0.264 | 0.003 | 19.822 |
|  | rs79195215 | 0.078 | 0.028 | 0.057 | 0.168 | 0.003 | 19.569 |
|  | rs923920 | 0.013 | 0.537 | 0.019 | 0.473 | 0.003 | 20.825 |
| Phosphatidylcholine (O-16:1_20:4) | rs1037782 | 0.048 | 0.056 | 0.041 | 0.238 | 0.003 | 20.771 |
|  | rs10487877 | -0.025 | 0.201 | 0.023 | 0.279 | 0.004 | 23.469 |
|  | rs1077989 | -0.041 | 0.454 | 0.019 | 0.029 | 0.018 | 113.093 |
|  | rs114325590 | 0.010 | 0.012 | 0.088 | 0.912 | 0.004 | 25.427 |
|  | rs12579775 | -0.008 | 0.087 | 0.033 | 0.808 | 0.004 | 23.878 |
|  | rs12920974 | 0.014 | 0.321 | 0.020 | 0.479 | 0.005 | 28.146 |
|  | rs1316133 | 0.002 | 0.300 | 0.020 | 0.936 | 0.003 | 19.801 |
|  | rs138018897 | 0.006 | 0.024 | 0.063 | 0.922 | 0.003 | 20.974 |
|  | rs17268903 | -0.020 | 0.125 | 0.028 | 0.481 | 0.003 | 19.890 |
|  | rs174527 | 0.018 | 0.452 | 0.019 | 0.341 | 0.007 | 42.432 |
|  | rs174535 | 0.080 | 0.416 | 0.019 | 0.000 | 0.077 | 505.745 |
|  | rs180360 | -0.006 | 0.322 | 0.020 | 0.747 | 0.003 | 20.411 |
|  | rs2015950 | 0.028 | 0.140 | 0.027 | 0.294 | 0.004 | 25.188 |
|  | rs2043085 | -0.018 | 0.580 | 0.019 | 0.338 | 0.005 | 29.882 |
|  | rs2431867 | 0.000 | 0.208 | 0.023 | 0.989 | 0.003 | 20.015 |
|  | rs5028238 | -0.008 | 0.763 | 0.022 | 0.723 | 0.003 | 20.043 |
|  | rs56303140 | -0.075 | 0.033 | 0.053 | 0.156 | 0.003 | 21.005 |
|  | rs6752137 | -0.037 | 0.117 | 0.029 | 0.200 | 0.004 | 21.577 |
|  | rs6776300 | 0.004 | 0.530 | 0.019 | 0.846 | 0.003 | 19.918 |
|  | rs71366199 | -0.017 | 0.150 | 0.026 | 0.522 | 0.003 | 20.361 |
|  | rs71452092 | 0.099 | 0.035 | 0.051 | 0.051 | 0.003 | 19.763 |
|  | rs73531625 | 0.029 | 0.281 | 0.021 | 0.166 | 0.003 | 20.935 |
|  | rs74854572 | -0.021 | 0.011 | 0.090 | 0.820 | 0.003 | 20.394 |
|  | rs7963111 | -0.004 | 0.051 | 0.043 | 0.923 | 0.003 | 20.563 |
|  | rs79740876 | 0.043 | 0.053 | 0.042 | 0.306 | 0.004 | 21.170 |
| Phosphatidylcholine (O-18:0_20:4) | rs11057853 | -0.016 | 0.451 | 0.019 | 0.395 | 0.003 | 20.476 |
|  | rs117870830 | 0.174 | 0.020 | 0.063 | 0.006 | 0.003 | 20.689 |
|  | rs11954170 | 0.016 | 0.314 | 0.020 | 0.432 | 0.003 | 20.489 |
|  | rs138202451 | 0.103 | 0.009 | 0.102 | 0.315 | 0.004 | 26.787 |
|  | rs143233558 | -0.028 | 0.094 | 0.032 | 0.390 | 0.004 | 27.577 |
|  | rs145755646 | -0.006 | 0.063 | 0.038 | 0.880 | 0.003 | 21.195 |
|  | rs148462060 | -0.011 | 0.024 | 0.061 | 0.858 | 0.003 | 21.660 |
|  | rs148840717 | -0.071 | 0.035 | 0.050 | 0.157 | 0.003 | 19.740 |
|  | rs174536 | 0.077 | 0.414 | 0.019 | 0.000 | 0.045 | 331.183 |
|  | rs1800588 | -0.012 | 0.248 | 0.022 | 0.586 | 0.004 | 27.560 |
|  | rs34382810 | 0.050 | 0.364 | 0.019 | 0.009 | 0.003 | 23.749 |
|  | rs34737685 | -0.015 | 0.529 | 0.019 | 0.425 | 0.003 | 20.634 |
|  | rs3741252 | -0.077 | 0.129 | 0.028 | 0.007 | 0.005 | 36.061 |
|  | rs376516 | -0.303 | 0.000 | 0.494 | 0.540 | 0.003 | 19.830 |
|  | rs4706511 | -0.011 | 0.191 | 0.024 | 0.633 | 0.003 | 19.541 |
|  | rs4782783 | 0.002 | 0.886 | 0.029 | 0.940 | 0.003 | 21.473 |
|  | rs6992951 | 0.015 | 0.394 | 0.019 | 0.420 | 0.003 | 22.579 |
|  | rs73074943 | -0.045 | 0.025 | 0.059 | 0.445 | 0.003 | 20.667 |
|  | rs7412 | -0.013 | 0.053 | 0.042 | 0.762 | 0.004 | 26.340 |
|  | rs78711160 | -0.021 | 0.043 | 0.046 | 0.648 | 0.003 | 21.060 |
|  | rs79856406 | 0.037 | 0.101 | 0.031 | 0.228 | 0.003 | 19.540 |
|  | rs9591400 | -0.061 | 0.115 | 0.029 | 0.037 | 0.003 | 20.623 |
| Phosphatidylcholine (O-18:1_18:2) | rs11022354 | -0.008 | 0.529 | 0.019 | 0.651 | 0.003 | 19.637 |
|  | rs111306887 | 0.024 | 0.030 | 0.055 | 0.660 | 0.003 | 20.640 |
|  | rs115600841 | 0.046 | 0.019 | 0.068 | 0.502 | 0.003 | 19.640 |
|  | rs117145701 | -0.060 | 0.010 | 0.096 | 0.534 | 0.003 | 19.960 |
|  | rs11918522 | 0.037 | 0.162 | 0.025 | 0.141 | 0.004 | 29.009 |
|  | rs147996419 | -0.079 | 0.014 | 0.080 | 0.321 | 0.003 | 19.534 |
|  | rs1601934 | -0.025 | 0.654 | 0.020 | 0.201 | 0.004 | 28.766 |
|  | rs17086339 | -0.002 | 0.136 | 0.027 | 0.952 | 0.003 | 19.867 |
|  | rs17231506 | 0.027 | 0.279 | 0.021 | 0.200 | 0.004 | 24.907 |
|  | rs174551 | 0.078 | 0.412 | 0.019 | 0.000 | 0.025 | 173.589 |
|  | rs17875991 | 0.019 | 0.039 | 0.049 | 0.694 | 0.003 | 19.582 |
|  | rs1800588 | -0.012 | 0.248 | 0.022 | 0.586 | 0.003 | 20.248 |
|  | rs2133619 | 0.011 | 0.025 | 0.059 | 0.858 | 0.003 | 20.724 |
|  | rs2537611 | 0.001 | 0.374 | 0.019 | 0.939 | 0.004 | 24.009 |
|  | rs2985394 | -0.030 | 0.653 | 0.020 | 0.126 | 0.003 | 22.096 |
|  | rs4655970 | -0.007 | 0.653 | 0.020 | 0.714 | 0.003 | 20.293 |
|  | rs58828781 | 0.054 | 0.048 | 0.044 | 0.221 | 0.003 | 21.099 |
|  | rs606587 | -0.017 | 0.377 | 0.019 | 0.376 | 0.004 | 26.043 |
|  | rs61833681 | 0.006 | 0.244 | 0.022 | 0.787 | 0.003 | 20.019 |
|  | rs62294908 | 0.020 | 0.093 | 0.032 | 0.527 | 0.003 | 20.605 |
|  | rs62331262 | 0.005 | 0.176 | 0.025 | 0.853 | 0.003 | 21.777 |
|  | rs72709643 | -0.082 | 0.037 | 0.050 | 0.099 | 0.003 | 20.967 |
|  | rs73133304 | -0.178 | 0.004 | 0.139 | 0.201 | 0.003 | 20.939 |
|  | rs74864573 | -0.064 | 0.034 | 0.052 | 0.215 | 0.003 | 20.652 |
|  | rs79040967 | 0.063 | 0.043 | 0.046 | 0.172 | 0.003 | 22.010 |
| Phosphatidylcholine (O-18:2_16:0) | rs10768424 | 0.009 | 0.571 | 0.019 | 0.622 | 0.003 | 21.301 |
|  | rs10775658 | -0.012 | 0.634 | 0.019 | 0.545 | 0.003 | 20.496 |
|  | rs113016188 | -0.026 | 0.003 | 0.187 | 0.888 | 0.003 | 23.108 |
|  | rs114061227 | -0.049 | 0.003 | 0.176 | 0.779 | 0.003 | 19.662 |
|  | rs11572749 | -0.005 | 0.078 | 0.035 | 0.889 | 0.003 | 19.730 |
|  | rs11936543 | 0.018 | 0.095 | 0.032 | 0.578 | 0.003 | 20.769 |
|  | rs12123955 | -0.057 | 0.063 | 0.038 | 0.134 | 0.003 | 21.497 |
|  | rs142235374 | -0.014 | 0.022 | 0.064 | 0.825 | 0.004 | 29.000 |
|  | rs146468726 | -0.056 | 0.026 | 0.058 | 0.340 | 0.003 | 20.469 |
|  | rs149389892 | -0.142 | 0.013 | 0.085 | 0.095 | 0.003 | 20.157 |
|  | rs149897686 | 0.093 | 0.017 | 0.073 | 0.206 | 0.003 | 23.231 |
|  | rs173539 | 0.028 | 0.283 | 0.021 | 0.175 | 0.005 | 34.945 |
|  | rs174566 | 0.079 | 0.413 | 0.019 | 0.000 | 0.010 | 75.535 |
|  | rs185625951 | -0.167 | 0.017 | 0.072 | 0.019 | 0.003 | 22.176 |
|  | rs188033349 | 0.061 | 0.011 | 0.089 | 0.496 | 0.003 | 22.740 |
|  | rs34446132 | -0.047 | 0.030 | 0.055 | 0.391 | 0.003 | 20.488 |
|  | rs55904113 | 0.005 | 0.187 | 0.024 | 0.845 | 0.003 | 20.477 |
|  | rs7091107 | -0.011 | 0.012 | 0.086 | 0.902 | 0.003 | 20.826 |
|  | rs73263508 | 0.093 | 0.033 | 0.053 | 0.078 | 0.003 | 20.645 |
|  | rs7563526 | -0.032 | 0.034 | 0.051 | 0.532 | 0.003 | 23.666 |
|  | rs75957793 | -0.031 | 0.149 | 0.026 | 0.240 | 0.003 | 20.823 |
|  | rs76437425 | 0.011 | 0.176 | 0.024 | 0.658 | 0.003 | 22.666 |
|  | rs7677028 | -0.046 | 0.016 | 0.075 | 0.543 | 0.003 | 21.396 |
|  | rs77219173 | 0.003 | 0.021 | 0.064 | 0.967 | 0.003 | 19.937 |
|  | rs7870035 | 0.006 | 0.489 | 0.019 | 0.760 | 0.003 | 20.003 |
|  | rs9341454 | -0.046 | 0.662 | 0.020 | 0.019 | 0.003 | 21.120 |
| Phosphatidylcholine (O-18:2_18:1) | rs1077834 | -0.016 | 0.257 | 0.021 | 0.453 | 0.003 | 20.091 |
|  | rs10868635 | -0.010 | 0.295 | 0.020 | 0.630 | 0.003 | 22.126 |
|  | rs117376487 | -0.059 | 0.009 | 0.099 | 0.551 | 0.003 | 21.656 |
|  | rs12441343 | 0.006 | 0.546 | 0.019 | 0.749 | 0.003 | 20.652 |
|  | rs148835629 | -0.063 | 0.028 | 0.057 | 0.269 | 0.003 | 20.310 |
|  | rs174564 | 0.081 | 0.414 | 0.019 | 0.000 | 0.010 | 69.884 |
|  | rs1982433 | 0.024 | 0.091 | 0.032 | 0.462 | 0.003 | 20.374 |
|  | rs3110417 | 0.011 | 0.251 | 0.021 | 0.608 | 0.003 | 19.938 |
|  | rs55975242 | -0.031 | 0.040 | 0.047 | 0.506 | 0.003 | 22.140 |
|  | rs56734149 | 0.005 | 0.181 | 0.024 | 0.838 | 0.003 | 20.661 |
|  | rs62621679 | -0.054 | 0.055 | 0.041 | 0.188 | 0.003 | 23.038 |
|  | rs72709643 | -0.082 | 0.037 | 0.050 | 0.099 | 0.003 | 21.714 |
|  | rs72840788 | 0.014 | 0.221 | 0.023 | 0.522 | 0.003 | 21.746 |
|  | rs73189920 | -0.002 | 0.100 | 0.031 | 0.950 | 0.003 | 20.472 |
|  | rs7642984 | 0.034 | 0.161 | 0.025 | 0.185 | 0.004 | 29.754 |
|  | rs77664718 | 0.137 | 0.019 | 0.071 | 0.056 | 0.003 | 19.623 |
|  | rs79713419 | -0.009 | 0.272 | 0.021 | 0.677 | 0.003 | 19.714 |
|  | rs79847391 | 0.060 | 0.053 | 0.042 | 0.153 | 0.003 | 19.952 |
| Phosphatidylethanolamine (18:0_18:2) | rs10069895 | -0.023 | 0.763 | 0.022 | 0.303 | 0.003 | 24.604 |
|  | rs10276778 | -0.007 | 0.045 | 0.044 | 0.866 | 0.003 | 20.336 |
|  | rs10468017 | 0.022 | 0.331 | 0.020 | 0.272 | 0.040 | 299.556 |
|  | rs10761714 | -0.031 | 0.850 | 0.026 | 0.230 | 0.003 | 20.370 |
|  | rs111543310 | 0.055 | 0.023 | 0.062 | 0.377 | 0.003 | 21.520 |
|  | rs112129861 | 0.003 | 0.066 | 0.037 | 0.925 | 0.003 | 21.335 |
|  | rs117376818 | 0.075 | 0.016 | 0.075 | 0.320 | 0.011 | 80.214 |
|  | rs117900629 | 0.008 | 0.055 | 0.041 | 0.846 | 0.005 | 38.337 |
|  | rs1260326 | -0.030 | 0.650 | 0.020 | 0.124 | 0.007 | 49.139 |
|  | rs12928099 | 0.007 | 0.282 | 0.021 | 0.746 | 0.005 | 36.620 |
|  | rs13110318 | -0.012 | 0.119 | 0.029 | 0.676 | 0.003 | 21.419 |
|  | rs138283783 | -0.037 | 0.024 | 0.061 | 0.547 | 0.006 | 43.088 |
|  | rs141883259 | -0.060 | 0.010 | 0.094 | 0.520 | 0.003 | 20.330 |
|  | rs149431055 | -0.095 | 0.027 | 0.057 | 0.099 | 0.003 | 19.714 |
|  | rs174527 | 0.018 | 0.452 | 0.019 | 0.341 | 0.005 | 35.448 |
|  | rs174567 | 0.082 | 0.415 | 0.019 | 0.000 | 0.057 | 429.030 |
|  | rs2840005 | 0.021 | 0.135 | 0.027 | 0.451 | 0.003 | 19.856 |
|  | rs2946493 | 0.000 | 0.197 | 0.024 | 0.989 | 0.004 | 25.569 |
|  | rs34733845 | -0.022 | 0.060 | 0.039 | 0.583 | 0.003 | 21.360 |
|  | rs35828755 | -0.011 | 0.199 | 0.023 | 0.644 | 0.003 | 19.675 |
|  | rs419482 | -0.045 | 0.394 | 0.019 | 0.018 | 0.003 | 21.233 |
|  | rs4296402 | 0.022 | 0.157 | 0.026 | 0.401 | 0.003 | 21.068 |
|  | rs449480 | 0.029 | 0.541 | 0.019 | 0.122 | 0.003 | 20.549 |
|  | rs62291572 | -0.005 | 0.210 | 0.023 | 0.826 | 0.003 | 22.667 |
|  | rs62434228 | -0.047 | 0.153 | 0.026 | 0.075 | 0.003 | 22.312 |
|  | rs633695 | -0.006 | 0.305 | 0.020 | 0.769 | 0.027 | 200.787 |
|  | rs7226158 | -0.012 | 0.683 | 0.020 | 0.561 | 0.003 | 20.493 |
|  | rs72679383 | -0.126 | 0.023 | 0.063 | 0.044 | 0.003 | 23.879 |
|  | rs73424597 | 0.021 | 0.037 | 0.049 | 0.674 | 0.009 | 61.182 |
|  | rs805292 | 0.046 | 0.363 | 0.019 | 0.018 | 0.003 | 23.781 |
|  | rs964184 | 0.028 | 0.855 | 0.027 | 0.290 | 0.006 | 44.472 |
| Phosphatidylethanolamine (O-16:1_20:4) | rs10234951 | 0.042 | 0.082 | 0.034 | 0.213 | 0.003 | 23.661 |
|  | rs1034755 | 0.038 | 0.310 | 0.020 | 0.062 | 0.003 | 22.498 |
|  | rs10487877 | -0.025 | 0.201 | 0.023 | 0.279 | 0.003 | 20.817 |
|  | rs10494405 | 0.039 | 0.104 | 0.031 | 0.207 | 0.003 | 21.013 |
|  | rs11136432 | 0.006 | 0.587 | 0.019 | 0.755 | 0.003 | 22.020 |
|  | rs11725298 | 0.063 | 0.025 | 0.059 | 0.292 | 0.003 | 20.878 |
|  | rs12923459 | -0.008 | 0.451 | 0.019 | 0.660 | 0.003 | 19.779 |
|  | rs13123798 | 0.012 | 0.596 | 0.019 | 0.523 | 0.003 | 24.368 |
|  | rs143745495 | -0.009 | 0.175 | 0.024 | 0.710 | 0.003 | 19.796 |
|  | rs204474 | -0.007 | 0.597 | 0.019 | 0.719 | 0.003 | 24.133 |
|  | rs2070895 | -0.016 | 0.258 | 0.021 | 0.462 | 0.003 | 19.554 |
|  | rs2277289 | -0.033 | 0.325 | 0.020 | 0.093 | 0.003 | 20.303 |
|  | rs2573210 | -0.036 | 0.828 | 0.025 | 0.144 | 0.003 | 24.841 |
|  | rs3820438 | 0.024 | 0.288 | 0.021 | 0.248 | 0.003 | 23.910 |
|  | rs3826884 | 0.065 | 0.156 | 0.025 | 0.010 | 0.008 | 57.616 |
|  | rs6601694 | 0.015 | 0.319 | 0.020 | 0.451 | 0.003 | 23.221 |
|  | rs7232502 | -0.025 | 0.286 | 0.021 | 0.223 | 0.003 | 20.268 |
|  | rs7445920 | -0.048 | 0.194 | 0.024 | 0.045 | 0.003 | 20.932 |
|  | rs74677222 | -0.012 | 0.074 | 0.036 | 0.735 | 0.003 | 22.842 |
|  | rs78802873 | -0.014 | 0.063 | 0.039 | 0.714 | 0.003 | 19.761 |
|  | rs79337987 | 0.103 | 0.021 | 0.065 | 0.115 | 0.003 | 23.426 |
|  | rs9265944 | 0.039 | 0.386 | 0.019 | 0.044 | 0.003 | 23.731 |
|  | rs97384 | -0.080 | 0.574 | 0.019 | 0.000 | 0.021 | 154.348 |
|  | rs9946720 | -0.008 | 0.404 | 0.019 | 0.667 | 0.003 | 22.693 |
| Phosphatidylethanolamine (O-18:2_20:4) | rs1034755 | 0.038 | 0.310 | 0.020 | 0.062 | 0.003 | 20.009 |
|  | rs115069241 | 0.018 | 0.026 | 0.060 | 0.758 | 0.003 | 20.664 |
|  | rs11522703 | -0.010 | 0.169 | 0.025 | 0.698 | 0.003 | 19.995 |
|  | rs115623452 | -0.188 | 0.003 | 0.160 | 0.238 | 0.003 | 21.223 |
|  | rs13280051 | -0.003 | 0.233 | 0.022 | 0.875 | 0.003 | 22.739 |
|  | rs141451859 | 0.056 | 0.043 | 0.046 | 0.226 | 0.003 | 20.244 |
|  | rs143745495 | -0.009 | 0.175 | 0.024 | 0.710 | 0.003 | 22.291 |
|  | rs145522126 | -0.148 | 0.020 | 0.066 | 0.026 | 0.003 | 20.269 |
|  | rs174556 | 0.068 | 0.386 | 0.019 | 0.000 | 0.015 | 112.547 |
|  | rs17703316 | -0.052 | 0.046 | 0.045 | 0.246 | 0.003 | 20.108 |
|  | rs17864186 | -0.046 | 0.071 | 0.037 | 0.205 | 0.003 | 20.873 |
|  | rs181750334 | 0.066 | 0.009 | 0.102 | 0.516 | 0.004 | 27.168 |
|  | rs204474 | -0.007 | 0.597 | 0.019 | 0.719 | 0.004 | 31.056 |
|  | rs2323249 | 0.052 | 0.031 | 0.054 | 0.343 | 0.003 | 19.957 |
|  | rs3820438 | 0.024 | 0.288 | 0.021 | 0.248 | 0.003 | 23.343 |
|  | rs4877665 | -0.003 | 0.587 | 0.019 | 0.872 | 0.003 | 20.057 |
|  | rs55853808 | 0.031 | 0.131 | 0.028 | 0.260 | 0.003 | 19.577 |
|  | rs61792775 | 0.041 | 0.072 | 0.036 | 0.259 | 0.003 | 21.702 |
|  | rs71366199 | -0.017 | 0.150 | 0.026 | 0.522 | 0.004 | 25.351 |
|  | rs71420341 | -0.050 | 0.087 | 0.033 | 0.134 | 0.003 | 21.752 |
|  | rs7228147 | 0.032 | 0.112 | 0.030 | 0.275 | 0.003 | 20.307 |
|  | rs72627458 | 0.002 | 0.029 | 0.055 | 0.968 | 0.003 | 20.366 |
|  | rs77754835 | -0.005 | 0.153 | 0.026 | 0.860 | 0.003 | 19.578 |
|  | rs79337987 | 0.103 | 0.021 | 0.065 | 0.115 | 0.003 | 20.596 |
|  | rs80299509 | 0.019 | 0.124 | 0.028 | 0.504 | 0.003 | 20.541 |
|  | rs9564073 | 0.058 | 0.044 | 0.045 | 0.199 | 0.003 | 19.930 |
|  | rs9946720 | -0.008 | 0.404 | 0.019 | 0.667 | 0.003 | 22.718 |
| Phosphatidylinositol (18:0_20:3) | rs10897211 | 0.011 | 0.342 | 0.020 | 0.566 | 0.004 | 29.473 |
|  | rs1168104 | 0.035 | 0.736 | 0.021 | 0.098 | 0.009 | 60.963 |
|  | rs117006704 | -0.054 | 0.019 | 0.070 | 0.441 | 0.003 | 20.505 |
|  | rs117511121 | 0.084 | 0.013 | 0.083 | 0.310 | 0.003 | 22.374 |
|  | rs11769084 | 0.026 | 0.103 | 0.031 | 0.404 | 0.003 | 21.309 |
|  | rs117761170 | 0.053 | 0.043 | 0.046 | 0.252 | 0.003 | 20.139 |
|  | rs12458441 | -0.009 | 0.090 | 0.032 | 0.786 | 0.004 | 24.223 |
|  | rs12598816 | 0.047 | 0.282 | 0.021 | 0.023 | 0.003 | 21.771 |
|  | rs1260326 | -0.030 | 0.650 | 0.020 | 0.124 | 0.004 | 25.145 |
|  | rs13147779 | 0.026 | 0.181 | 0.024 | 0.293 | 0.003 | 22.601 |
|  | rs145740269 | -0.040 | 0.045 | 0.045 | 0.373 | 0.003 | 20.511 |
|  | rs16916055 | 0.059 | 0.007 | 0.114 | 0.602 | 0.003 | 23.778 |
|  | rs17085148 | -0.015 | 0.046 | 0.045 | 0.732 | 0.003 | 20.232 |
|  | rs1937711 | -0.006 | 0.653 | 0.020 | 0.742 | 0.003 | 22.787 |
|  | rs2819082 | -0.006 | 0.287 | 0.021 | 0.763 | 0.003 | 21.862 |
|  | rs28456 | 0.070 | 0.389 | 0.019 | 0.000 | 0.057 | 409.524 |
|  | rs28514538 | -0.055 | 0.248 | 0.022 | 0.011 | 0.003 | 21.457 |
|  | rs319552 | 0.029 | 0.244 | 0.022 | 0.184 | 0.003 | 20.857 |
|  | rs59382088 | -0.071 | 0.020 | 0.067 | 0.287 | 0.003 | 19.908 |
|  | rs59739041 | -0.015 | 0.048 | 0.043 | 0.721 | 0.003 | 19.777 |
|  | rs6498540 | -0.003 | 0.333 | 0.020 | 0.880 | 0.007 | 47.744 |
|  | rs67945876 | -0.025 | 0.246 | 0.022 | 0.252 | 0.003 | 21.672 |
|  | rs6820980 | 0.004 | 0.575 | 0.019 | 0.834 | 0.003 | 22.643 |
|  | rs72914920 | -0.003 | 0.291 | 0.021 | 0.902 | 0.004 | 25.746 |
|  | rs7355249 | -0.026 | 0.009 | 0.100 | 0.791 | 0.003 | 20.085 |
|  | rs76457276 | -0.078 | 0.024 | 0.062 | 0.209 | 0.003 | 21.195 |
|  | rs7782961 | 0.067 | 0.225 | 0.022 | 0.002 | 0.003 | 21.197 |
|  | rs7845664 | -0.021 | 0.570 | 0.019 | 0.259 | 0.003 | 22.388 |
|  | rs80011585 | 0.058 | 0.070 | 0.036 | 0.112 | 0.003 | 20.143 |
|  | rs8736 | 0.005 | 0.606 | 0.019 | 0.778 | 0.024 | 163.773 |
|  | rs9530145 | -0.047 | 0.099 | 0.031 | 0.133 | 0.003 | 19.952 |
|  | rs964184 | 0.028 | 0.855 | 0.027 | 0.290 | 0.007 | 46.375 |
|  | rs976947 | 0.013 | 0.621 | 0.019 | 0.507 | 0.003 | 20.385 |
| Phosphatidylinositol (18:1_18:1) | rs10468017 | 0.022 | 0.331 | 0.020 | 0.272 | 0.004 | 25.471 |
|  | rs10889352 | -0.036 | 0.264 | 0.021 | 0.091 | 0.009 | 67.360 |
|  | rs116845732 | 0.039 | 0.004 | 0.144 | 0.786 | 0.004 | 26.384 |
|  | rs11783515 | -0.003 | 0.622 | 0.019 | 0.881 | 0.004 | 26.734 |
|  | rs12428337 | 0.014 | 0.243 | 0.022 | 0.531 | 0.003 | 21.037 |
|  | rs145993592 | 0.035 | 0.007 | 0.115 | 0.760 | 0.003 | 20.554 |
|  | rs149540646 | 0.008 | 0.259 | 0.021 | 0.690 | 0.003 | 20.328 |
|  | rs149615216 | -0.014 | 0.005 | 0.132 | 0.916 | 0.003 | 20.457 |
|  | rs1540037 | 0.014 | 0.762 | 0.022 | 0.520 | 0.010 | 69.817 |
|  | rs174568 | 0.079 | 0.411 | 0.019 | 0.000 | 0.012 | 85.237 |
|  | rs1751064 | -0.025 | 0.760 | 0.022 | 0.254 | 0.003 | 20.010 |
|  | rs186422572 | 0.026 | 0.017 | 0.073 | 0.726 | 0.003 | 21.250 |
|  | rs36634 | 0.002 | 0.604 | 0.019 | 0.920 | 0.007 | 51.951 |
|  | rs41418546 | 0.032 | 0.081 | 0.034 | 0.350 | 0.003 | 20.962 |
|  | rs4804576 | -0.035 | 0.063 | 0.038 | 0.358 | 0.004 | 26.525 |
|  | rs6023985 | 0.159 | 0.014 | 0.080 | 0.047 | 0.003 | 19.606 |
|  | rs62095282 | -0.018 | 0.119 | 0.029 | 0.532 | 0.003 | 20.831 |
|  | rs62409317 | -0.014 | 0.051 | 0.042 | 0.743 | 0.003 | 21.469 |
|  | rs627108 | -0.015 | 0.399 | 0.019 | 0.424 | 0.003 | 24.683 |
|  | rs72846591 | -0.001 | 0.389 | 0.019 | 0.971 | 0.003 | 21.841 |
|  | rs78572804 | -0.059 | 0.011 | 0.091 | 0.513 | 0.003 | 20.807 |
|  | rs964184 | 0.028 | 0.855 | 0.027 | 0.290 | 0.007 | 51.541 |
|  | rs9652580 | -0.001 | 0.422 | 0.019 | 0.977 | 0.003 | 21.661 |
| Sphingomyelin (d36:1) | rs10838826 | -0.022 | 0.773 | 0.022 | 0.319 | 0.003 | 20.811 |
|  | rs112585713 | -0.038 | 0.030 | 0.055 | 0.486 | 0.005 | 34.504 |
|  | rs114839471 | -0.092 | 0.007 | 0.116 | 0.425 | 0.003 | 22.129 |
|  | rs11591147 | 0.039 | 0.036 | 0.050 | 0.437 | 0.004 | 30.793 |
|  | rs1169306 | 0.012 | 0.356 | 0.019 | 0.526 | 0.003 | 24.107 |
|  | rs117442205 | 0.036 | 0.004 | 0.159 | 0.820 | 0.003 | 19.588 |
|  | rs117643293 | 0.032 | 0.063 | 0.038 | 0.403 | 0.003 | 25.115 |
|  | rs11779209 | -0.038 | 0.129 | 0.028 | 0.169 | 0.003 | 19.653 |
|  | rs12462619 | 0.046 | 0.253 | 0.021 | 0.033 | 0.004 | 31.265 |
|  | rs12464353 | 0.018 | 0.031 | 0.053 | 0.734 | 0.003 | 22.891 |
|  | rs12675806 | 0.024 | 0.113 | 0.029 | 0.410 | 0.003 | 22.719 |
|  | rs138524673 | -0.052 | 0.039 | 0.048 | 0.277 | 0.003 | 20.594 |
|  | rs1452773 | -0.020 | 0.140 | 0.027 | 0.461 | 0.003 | 23.777 |
|  | rs17664981 | -0.004 | 0.054 | 0.042 | 0.918 | 0.003 | 20.735 |
|  | rs182695896 | -0.020 | 0.026 | 0.059 | 0.731 | 0.006 | 41.028 |
|  | rs186039163 | -0.104 | 0.006 | 0.124 | 0.403 | 0.003 | 24.457 |
|  | rs2336171 | -0.028 | 0.400 | 0.019 | 0.149 | 0.018 | 131.824 |
|  | rs364585 | 0.008 | 0.658 | 0.020 | 0.688 | 0.005 | 38.565 |
|  | rs374071816 | 0.005 | 0.063 | 0.039 | 0.890 | 0.003 | 19.593 |
|  | rs3742023 | -0.020 | 0.422 | 0.019 | 0.291 | 0.003 | 20.634 |
|  | rs4144237 | -0.044 | 0.122 | 0.029 | 0.128 | 0.003 | 19.845 |
|  | rs4246215 | 0.070 | 0.443 | 0.019 | 0.000 | 0.003 | 19.761 |
|  | rs4809845 | -0.018 | 0.145 | 0.027 | 0.508 | 0.004 | 26.713 |
|  | rs4981443 | -0.002 | 0.354 | 0.019 | 0.929 | 0.003 | 19.604 |
|  | rs581080 | -0.061 | 0.855 | 0.027 | 0.022 | 0.003 | 23.220 |
|  | rs6558122 | 0.023 | 0.105 | 0.030 | 0.441 | 0.004 | 25.470 |
|  | rs6829637 | 0.018 | 0.410 | 0.019 | 0.348 | 0.003 | 20.950 |
|  | rs7157785 | -0.005 | 0.134 | 0.027 | 0.848 | 0.004 | 29.244 |
|  | rs7248003 | 0.003 | 0.724 | 0.021 | 0.901 | 0.008 | 58.170 |
|  | rs72898481 | 0.065 | 0.023 | 0.064 | 0.303 | 0.003 | 20.296 |
|  | rs73073606 | 0.026 | 0.239 | 0.022 | 0.240 | 0.003 | 19.902 |
|  | rs75679663 | 0.090 | 0.028 | 0.056 | 0.108 | 0.005 | 36.724 |
|  | rs79146711 | -0.036 | 0.043 | 0.046 | 0.432 | 0.005 | 32.871 |
|  | rs8139868 | -0.008 | 0.257 | 0.021 | 0.713 | 0.003 | 20.847 |
|  | rs884532 | -0.004 | 0.510 | 0.019 | 0.826 | 0.003 | 20.230 |
|  | rs886813 | -0.177 | 0.001 | 0.515 | 0.732 | 0.003 | 23.038 |
| Sphingomyelin (d38:1) | rs114483871 | -0.069 | 0.036 | 0.050 | 0.173 | 0.005 | 35.878 |
|  | rs11591147 | 0.039 | 0.036 | 0.050 | 0.437 | 0.006 | 41.800 |
|  | rs117146578 | -0.013 | 0.049 | 0.044 | 0.763 | 0.003 | 21.677 |
|  | rs117643293 | 0.032 | 0.063 | 0.038 | 0.403 | 0.007 | 51.391 |
|  | rs118070067 | 0.015 | 0.020 | 0.067 | 0.826 | 0.003 | 20.131 |
|  | rs12462619 | 0.046 | 0.253 | 0.021 | 0.033 | 0.003 | 24.935 |
|  | rs12480916 | 0.003 | 0.486 | 0.019 | 0.891 | 0.003 | 20.671 |
|  | rs12675806 | 0.024 | 0.113 | 0.029 | 0.410 | 0.003 | 20.931 |
|  | rs13258507 | -0.018 | 0.242 | 0.022 | 0.403 | 0.003 | 22.698 |
|  | rs144440646 | 0.094 | 0.011 | 0.091 | 0.305 | 0.003 | 23.130 |
|  | rs1452773 | -0.020 | 0.140 | 0.027 | 0.461 | 0.003 | 21.171 |
|  | rs146127446 | 0.012 | 0.418 | 0.019 | 0.522 | 0.003 | 19.931 |
|  | rs147426822 | -0.107 | 0.015 | 0.081 | 0.185 | 0.003 | 20.872 |
|  | rs182695896 | -0.020 | 0.026 | 0.059 | 0.731 | 0.007 | 49.183 |
|  | rs186039163 | -0.104 | 0.006 | 0.124 | 0.403 | 0.003 | 20.617 |
|  | rs188346987 | -0.006 | 0.046 | 0.045 | 0.894 | 0.003 | 20.311 |
|  | rs200949196 | 0.118 | 0.014 | 0.078 | 0.132 | 0.003 | 22.596 |
|  | rs2867985 | -0.020 | 0.313 | 0.020 | 0.320 | 0.003 | 20.244 |
|  | rs364585 | 0.008 | 0.658 | 0.020 | 0.688 | 0.012 | 88.272 |
|  | rs374071816 | 0.005 | 0.063 | 0.039 | 0.890 | 0.004 | 27.479 |
|  | rs3741111 | -0.021 | 0.481 | 0.019 | 0.271 | 0.004 | 26.904 |
|  | rs715119 | 0.024 | 0.239 | 0.022 | 0.272 | 0.003 | 21.776 |
|  | rs7157785 | -0.005 | 0.134 | 0.027 | 0.848 | 0.030 | 223.000 |
|  | rs7172051 | 0.016 | 0.371 | 0.019 | 0.417 | 0.003 | 19.669 |
|  | rs7247433 | 0.015 | 0.392 | 0.019 | 0.431 | 0.003 | 24.016 |
|  | rs7248003 | 0.003 | 0.724 | 0.021 | 0.901 | 0.005 | 32.942 |
|  | rs7258249 | -0.026 | 0.399 | 0.019 | 0.180 | 0.022 | 164.594 |
|  | rs73073606 | 0.026 | 0.239 | 0.022 | 0.240 | 0.003 | 22.957 |
|  | rs73193962 | 0.076 | 0.022 | 0.063 | 0.226 | 0.003 | 20.387 |
|  | rs7412 | -0.013 | 0.053 | 0.042 | 0.762 | 0.003 | 22.372 |
|  | rs7478167 | 0.054 | 0.660 | 0.020 | 0.007 | 0.003 | 21.626 |
|  | rs75679663 | 0.090 | 0.028 | 0.056 | 0.108 | 0.011 | 78.660 |
|  | rs75740927 | 0.020 | 0.030 | 0.055 | 0.716 | 0.003 | 20.149 |
|  | rs76174849 | -0.036 | 0.014 | 0.079 | 0.652 | 0.003 | 21.582 |
|  | rs76596580 | 0.028 | 0.109 | 0.030 | 0.341 | 0.003 | 21.031 |
|  | rs77645768 | -0.009 | 0.027 | 0.058 | 0.870 | 0.004 | 32.079 |
|  | rs79231873 | -0.100 | 0.021 | 0.066 | 0.133 | 0.003 | 20.118 |
|  | rs7970512 | 0.102 | 0.034 | 0.052 | 0.049 | 0.003 | 22.221 |
| Sphingomyelin (d38:2) | rs10206976 | -0.029 | 0.264 | 0.021 | 0.177 | 0.003 | 22.744 |
|  | rs10405061 | 0.023 | 0.308 | 0.020 | 0.252 | 0.004 | 27.064 |
|  | rs113118892 | 0.047 | 0.013 | 0.083 | 0.566 | 0.003 | 23.081 |
|  | rs114483871 | -0.069 | 0.036 | 0.050 | 0.173 | 0.003 | 24.085 |
|  | rs114717988 | 0.016 | 0.046 | 0.044 | 0.725 | 0.003 | 24.834 |
|  | rs11591147 | 0.039 | 0.036 | 0.050 | 0.437 | 0.003 | 21.948 |
|  | rs116411822 | 0.013 | 0.009 | 0.101 | 0.899 | 0.003 | 20.139 |
|  | rs1169306 | 0.012 | 0.356 | 0.019 | 0.526 | 0.007 | 48.205 |
|  | rs12462619 | 0.046 | 0.253 | 0.021 | 0.033 | 0.006 | 45.755 |
|  | rs139150589 | 0.083 | 0.007 | 0.113 | 0.463 | 0.004 | 27.202 |
|  | rs142118024 | -0.144 | 0.021 | 0.067 | 0.030 | 0.003 | 19.955 |
|  | rs142551539 | -0.063 | 0.007 | 0.117 | 0.588 | 0.003 | 20.071 |
|  | rs146723536 | -0.109 | 0.020 | 0.067 | 0.105 | 0.003 | 19.868 |
|  | rs148683988 | 0.099 | 0.014 | 0.080 | 0.213 | 0.003 | 21.271 |
|  | rs17276940 | 0.021 | 0.040 | 0.048 | 0.656 | 0.004 | 28.381 |
|  | rs174556 | 0.068 | 0.386 | 0.019 | 0.000 | 0.010 | 70.172 |
|  | rs182695896 | -0.020 | 0.026 | 0.059 | 0.731 | 0.005 | 37.717 |
|  | rs2336171 | -0.028 | 0.400 | 0.019 | 0.149 | 0.032 | 238.502 |
|  | rs250507 | 0.012 | 0.228 | 0.022 | 0.605 | 0.004 | 28.984 |
|  | rs3936109 | 0.007 | 0.172 | 0.025 | 0.778 | 0.003 | 22.033 |
|  | rs4804319 | 0.002 | 0.687 | 0.020 | 0.913 | 0.003 | 22.897 |
|  | rs581080 | -0.061 | 0.855 | 0.027 | 0.022 | 0.003 | 23.627 |
|  | rs61751862 | -0.045 | 0.035 | 0.051 | 0.376 | 0.003 | 22.935 |
|  | rs657315 | -0.020 | 0.293 | 0.020 | 0.325 | 0.003 | 19.788 |
|  | rs672959 | 0.197 | 0.008 | 0.107 | 0.065 | 0.003 | 20.991 |
|  | rs7248003 | 0.003 | 0.724 | 0.021 | 0.901 | 0.009 | 63.243 |
|  | rs72794954 | -0.062 | 0.007 | 0.110 | 0.572 | 0.003 | 21.830 |
|  | rs72839716 | 0.057 | 0.154 | 0.026 | 0.027 | 0.003 | 22.170 |
|  | rs75038600 | 0.074 | 0.004 | 0.144 | 0.609 | 0.003 | 20.534 |
|  | rs75098294 | -0.051 | 0.007 | 0.112 | 0.648 | 0.003 | 24.153 |
|  | rs77645768 | -0.009 | 0.027 | 0.058 | 0.870 | 0.003 | 24.894 |
| Sphingomyelin (d40:2) | rs10490233 | -0.029 | 0.111 | 0.030 | 0.325 | 0.003 | 24.479 |
|  | rs113118892 | 0.047 | 0.013 | 0.083 | 0.566 | 0.003 | 20.826 |
|  | rs113722226 | -0.063 | 0.101 | 0.031 | 0.041 | 0.003 | 25.089 |
|  | rs114483871 | -0.069 | 0.036 | 0.050 | 0.173 | 0.004 | 27.754 |
|  | rs11522703 | -0.010 | 0.169 | 0.025 | 0.698 | 0.003 | 19.762 |
|  | rs115880518 | -0.156 | 0.006 | 0.127 | 0.221 | 0.003 | 21.200 |
|  | rs11591147 | 0.039 | 0.036 | 0.050 | 0.437 | 0.004 | 30.948 |
|  | rs1237196 | 0.036 | 0.782 | 0.023 | 0.111 | 0.003 | 19.536 |
|  | rs12595641 | 0.021 | 0.200 | 0.023 | 0.378 | 0.003 | 20.509 |
|  | rs146855398 | -0.064 | 0.026 | 0.059 | 0.282 | 0.003 | 20.200 |
|  | rs148316008 | 0.034 | 0.013 | 0.085 | 0.686 | 0.003 | 20.750 |
|  | rs16851199 | 0.058 | 0.045 | 0.045 | 0.199 | 0.003 | 19.882 |
|  | rs17276933 | 0.026 | 0.064 | 0.038 | 0.501 | 0.003 | 21.005 |
|  | rs174561 | 0.067 | 0.387 | 0.019 | 0.000 | 0.007 | 49.523 |
|  | rs17604972 | -0.032 | 0.152 | 0.026 | 0.218 | 0.003 | 19.659 |
|  | rs1797734 | -0.036 | 0.648 | 0.020 | 0.067 | 0.003 | 20.067 |
|  | rs182695896 | -0.020 | 0.026 | 0.059 | 0.731 | 0.006 | 44.464 |
|  | rs187144380 | 0.258 | 0.010 | 0.086 | 0.003 | 0.003 | 21.625 |
|  | rs2760512 | 0.005 | 0.327 | 0.020 | 0.789 | 0.003 | 21.653 |
|  | rs3026120 | 0.020 | 0.076 | 0.035 | 0.579 | 0.005 | 36.478 |
|  | rs364585 | 0.008 | 0.658 | 0.020 | 0.688 | 0.007 | 48.114 |
|  | rs56298236 | -0.028 | 0.264 | 0.021 | 0.185 | 0.004 | 25.456 |
|  | rs61751862 | -0.045 | 0.035 | 0.051 | 0.376 | 0.003 | 24.597 |
|  | rs646488 | -0.005 | 0.692 | 0.020 | 0.822 | 0.003 | 20.817 |
|  | rs7157785 | -0.005 | 0.134 | 0.027 | 0.848 | 0.025 | 183.983 |
|  | rs7247433 | 0.015 | 0.392 | 0.019 | 0.431 | 0.003 | 19.961 |
|  | rs73073606 | 0.026 | 0.239 | 0.022 | 0.240 | 0.003 | 22.130 |
|  | rs73641617 | 0.093 | 0.018 | 0.071 | 0.190 | 0.003 | 21.364 |
|  | rs7478167 | 0.054 | 0.660 | 0.020 | 0.007 | 0.003 | 20.855 |
|  | rs7536561 | -0.017 | 0.572 | 0.019 | 0.366 | 0.003 | 20.232 |
|  | rs75679663 | 0.090 | 0.028 | 0.056 | 0.108 | 0.004 | 26.575 |
|  | rs77645768 | -0.009 | 0.027 | 0.058 | 0.870 | 0.004 | 32.052 |
|  | rs79402526 | -0.016 | 0.035 | 0.051 | 0.750 | 0.003 | 21.094 |
|  | rs79984432 | -0.053 | 0.012 | 0.084 | 0.526 | 0.003 | 20.531 |
|  | rs9835223 | -0.034 | 0.798 | 0.023 | 0.143 | 0.003 | 24.844 |
|  | rs9839427 | 0.024 | 0.334 | 0.020 | 0.233 | 0.003 | 20.964 |
| Sphingomyelin (d42:2) | rs10252648 | 0.096 | 0.978 | 0.064 | 0.129 | 0.003 | 19.542 |
|  | rs112585713 | -0.038 | 0.030 | 0.055 | 0.486 | 0.005 | 36.193 |
|  | rs114483871 | -0.069 | 0.036 | 0.050 | 0.173 | 0.005 | 38.862 |
|  | rs115350073 | 0.036 | 0.016 | 0.075 | 0.629 | 0.003 | 21.517 |
|  | rs11591147 | 0.039 | 0.036 | 0.050 | 0.437 | 0.008 | 60.504 |
|  | rs11610836 | -0.030 | 0.419 | 0.019 | 0.111 | 0.003 | 22.885 |
|  | rs116893213 | -0.037 | 0.036 | 0.050 | 0.463 | 0.004 | 25.657 |
|  | rs116919723 | -0.063 | 0.008 | 0.105 | 0.549 | 0.003 | 20.069 |
|  | rs118068660 | -0.061 | 0.102 | 0.031 | 0.048 | 0.008 | 60.902 |
|  | rs12530474 | -0.010 | 0.137 | 0.027 | 0.723 | 0.003 | 21.039 |
|  | rs1367117 | -0.001 | 0.280 | 0.021 | 0.968 | 0.004 | 29.151 |
|  | rs139474488 | -0.070 | 0.016 | 0.075 | 0.352 | 0.003 | 20.509 |
|  | rs146331166 | 0.045 | 0.062 | 0.039 | 0.244 | 0.006 | 40.090 |
|  | rs17018185 | -0.010 | 0.271 | 0.021 | 0.627 | 0.003 | 19.894 |
|  | rs17110517 | -0.029 | 0.057 | 0.040 | 0.467 | 0.003 | 22.389 |
|  | rs17145750 | 0.016 | 0.148 | 0.026 | 0.544 | 0.004 | 30.720 |
|  | rs17261772 | -0.047 | 0.308 | 0.020 | 0.022 | 0.004 | 25.763 |
|  | rs17276933 | 0.026 | 0.064 | 0.038 | 0.501 | 0.003 | 22.524 |
|  | rs182695896 | -0.020 | 0.026 | 0.059 | 0.731 | 0.007 | 52.244 |
|  | rs184845520 | -0.100 | 0.005 | 0.130 | 0.440 | 0.003 | 21.069 |
|  | rs2034007 | -0.016 | 0.305 | 0.020 | 0.441 | 0.003 | 20.080 |
|  | rs2177333 | 0.004 | 0.530 | 0.019 | 0.846 | 0.003 | 20.018 |
|  | rs3751934 | -0.003 | 0.423 | 0.019 | 0.864 | 0.003 | 20.746 |
|  | rs3912622 | 0.047 | 0.200 | 0.023 | 0.043 | 0.004 | 27.078 |
|  | rs4100654 | -0.017 | 0.076 | 0.036 | 0.631 | 0.003 | 21.412 |
|  | rs4283208 | 0.022 | 0.834 | 0.025 | 0.390 | 0.003 | 20.344 |
|  | rs515081 | -0.004 | 0.694 | 0.021 | 0.846 | 0.003 | 23.446 |
|  | rs59916403 | -0.014 | 0.410 | 0.019 | 0.453 | 0.003 | 21.364 |
|  | rs62274367 | 0.083 | 0.004 | 0.151 | 0.582 | 0.003 | 21.826 |
|  | rs6680361 | -0.021 | 0.800 | 0.023 | 0.369 | 0.003 | 21.051 |
|  | rs67718253 | 0.026 | 0.173 | 0.025 | 0.291 | 0.003 | 21.468 |
|  | rs6843498 | -0.016 | 0.309 | 0.020 | 0.418 | 0.004 | 25.306 |
|  | rs7104343 | -0.019 | 0.212 | 0.023 | 0.415 | 0.003 | 20.238 |
|  | rs7247433 | 0.015 | 0.392 | 0.019 | 0.431 | 0.003 | 20.702 |
|  | rs73047641 | 0.005 | 0.495 | 0.019 | 0.776 | 0.003 | 22.905 |
|  | rs7412 | -0.013 | 0.053 | 0.042 | 0.762 | 0.004 | 32.181 |
|  | rs75444922 | 0.032 | 0.022 | 0.065 | 0.623 | 0.003 | 19.641 |
|  | rs75679663 | 0.090 | 0.028 | 0.056 | 0.108 | 0.014 | 101.344 |
| Triacylglycerol (56:7) | rs10149017 | 0.055 | 0.800 | 0.023 | 0.018 | 0.003 | 19.642 |
|  | rs112214356 | 0.016 | 0.063 | 0.038 | 0.683 | 0.003 | 21.570 |
|  | rs115007623 | -0.067 | 0.010 | 0.095 | 0.483 | 0.003 | 20.386 |
|  | rs1260326 | -0.030 | 0.650 | 0.020 | 0.124 | 0.004 | 31.767 |
|  | rs148132478 | 0.278 | 0.004 | 0.141 | 0.048 | 0.003 | 20.332 |
|  | rs150578762 | 0.042 | 0.098 | 0.031 | 0.179 | 0.003 | 21.240 |
|  | rs16996148 | 0.000 | 0.067 | 0.037 | 0.992 | 0.003 | 20.168 |
|  | rs174554 | 0.082 | 0.412 | 0.019 | 0.000 | 0.012 | 90.275 |
|  | rs182611493 | 0.024 | 0.051 | 0.042 | 0.579 | 0.007 | 51.354 |
|  | rs1883350 | 0.000 | 0.395 | 0.019 | 0.998 | 0.004 | 26.065 |
|  | rs1902483 | -0.009 | 0.494 | 0.019 | 0.630 | 0.003 | 20.635 |
|  | rs2229738 | 0.019 | 0.155 | 0.026 | 0.473 | 0.004 | 26.420 |
|  | rs2626606 | 0.016 | 0.858 | 0.027 | 0.539 | 0.003 | 20.573 |
|  | rs34001747 | 0.015 | 0.011 | 0.089 | 0.869 | 0.003 | 19.534 |
|  | rs3916027 | -0.024 | 0.249 | 0.022 | 0.257 | 0.006 | 42.865 |
|  | rs483082 | 0.024 | 0.235 | 0.022 | 0.277 | 0.004 | 28.866 |
|  | rs513272 | 0.007 | 0.563 | 0.019 | 0.726 | 0.003 | 20.875 |
|  | rs57706029 | -0.003 | 0.611 | 0.019 | 0.883 | 0.003 | 21.326 |
|  | rs592586 | -0.024 | 0.600 | 0.019 | 0.210 | 0.003 | 22.533 |
|  | rs595400 | 0.011 | 0.535 | 0.019 | 0.561 | 0.003 | 20.334 |
|  | rs62419698 | -0.028 | 0.057 | 0.041 | 0.496 | 0.003 | 21.383 |
|  | rs6699175 | -0.031 | 0.107 | 0.030 | 0.302 | 0.003 | 21.677 |
|  | rs6904245 | -0.012 | 0.073 | 0.036 | 0.742 | 0.003 | 21.161 |
|  | rs7002672 | 0.008 | 0.798 | 0.023 | 0.728 | 0.003 | 20.402 |
|  | rs7156531 | -0.031 | 0.116 | 0.029 | 0.290 | 0.003 | 20.389 |
|  | rs7170012 | 0.017 | 0.573 | 0.019 | 0.367 | 0.003 | 20.208 |
|  | rs72623355 | 0.022 | 0.093 | 0.032 | 0.504 | 0.003 | 22.092 |
|  | rs72767411 | -0.187 | 0.013 | 0.084 | 0.027 | 0.003 | 20.237 |
|  | rs73095198 | 0.054 | 0.030 | 0.055 | 0.326 | 0.003 | 19.840 |
|  | rs75402627 | -0.003 | 0.076 | 0.035 | 0.925 | 0.003 | 23.064 |
|  | rs7608044 | 0.078 | 0.099 | 0.032 | 0.014 | 0.003 | 20.186 |
|  | rs80329613 | 0.006 | 0.057 | 0.040 | 0.883 | 0.003 | 19.834 |
|  | rs964184 | 0.028 | 0.855 | 0.027 | 0.290 | 0.012 | 87.242 |
| **CD** |  |  |  |  |  |  |  |
| Sterol ester (27:1/20:4) | rs11022403 | 0.010 | 0.291 | 0.035 | 0.785 | 0.003 | 21.220 |
|  | rs113248417 | 0.022 | 0.039 | 0.082 | 0.786 | 0.005 | 35.544 |
|  | rs1135999 | 0.061 | 0.315 | 0.034 | 0.072 | 0.005 | 32.506 |
|  | rs113603971 | -0.047 | 0.051 | 0.071 | 0.505 | 0.003 | 19.555 |
|  | rs11591147 | 0.081 | 0.036 | 0.085 | 0.338 | 0.006 | 41.421 |
|  | rs117084735 | 0.000 | 0.016 | 0.128 | 0.997 | 0.003 | 20.353 |
|  | rs117186386 | -0.063 | 0.048 | 0.075 | 0.397 | 0.004 | 25.459 |
|  | rs117825513 | -0.071 | 0.012 | 0.143 | 0.620 | 0.003 | 20.318 |
|  | rs138482835 | -0.093 | 0.052 | 0.073 | 0.201 | 0.003 | 22.278 |
|  | rs147981159 | -0.053 | 0.044 | 0.077 | 0.492 | 0.008 | 60.908 |
|  | rs174528 | 0.079 | 0.420 | 0.032 | 0.014 | 0.202 | 1819.849 |
|  | rs1800961 | -0.143 | 0.045 | 0.075 | 0.057 | 0.003 | 20.532 |
|  | rs182695896 | 0.031 | 0.026 | 0.100 | 0.757 | 0.004 | 31.741 |
|  | rs186913978 | 0.051 | 0.009 | 0.170 | 0.765 | 0.004 | 28.720 |
|  | rs187429064 | 0.102 | 0.051 | 0.072 | 0.158 | 0.006 | 46.088 |
|  | rs2229738 | 0.076 | 0.155 | 0.044 | 0.087 | 0.006 | 42.633 |
|  | rs223841 | 0.059 | 0.169 | 0.042 | 0.165 | 0.003 | 21.183 |
|  | rs2469034 | -0.040 | 0.797 | 0.039 | 0.309 | 0.003 | 20.122 |
|  | rs3741252 | -0.076 | 0.129 | 0.047 | 0.109 | 0.010 | 74.744 |
|  | rs3767941 | 0.014 | 0.173 | 0.042 | 0.734 | 0.003 | 22.617 |
|  | rs3820438 | -0.033 | 0.288 | 0.035 | 0.342 | 0.003 | 21.528 |
|  | rs4382917 | -0.035 | 0.243 | 0.037 | 0.350 | 0.008 | 55.143 |
|  | rs635196 | -0.018 | 0.177 | 0.041 | 0.665 | 0.003 | 23.862 |
|  | rs646299 | 0.007 | 0.606 | 0.032 | 0.839 | 0.003 | 19.672 |
|  | rs6812370 | 0.110 | 0.014 | 0.132 | 0.405 | 0.004 | 27.573 |
|  | rs6860806 | 0.101 | 0.418 | 0.032 | 0.001 | 0.003 | 20.015 |
|  | rs7020 | -0.020 | 0.449 | 0.032 | 0.522 | 0.003 | 19.900 |
|  | rs7412 | 0.006 | 0.053 | 0.071 | 0.934 | 0.010 | 70.977 |
|  | rs74915447 | -0.002 | 0.036 | 0.086 | 0.977 | 0.003 | 20.006 |
|  | rs7837272 | 0.682 | 0.002 | 0.376 | 0.070 | 0.003 | 21.080 |
|  | rs79136768 | 0.042 | 0.036 | 0.085 | 0.620 | 0.003 | 24.145 |
|  | rs9388335 | 0.018 | 0.525 | 0.032 | 0.578 | 0.003 | 21.634 |
|  | rs9555673 | 0.018 | 0.424 | 0.032 | 0.586 | 0.003 | 20.745 |
| Sterol ester (27:1/22:6) | rs10150075 | -0.030 | 0.061 | 0.066 | 0.646 | 0.004 | 28.636 |
|  | rs111751059 | -0.011 | 0.063 | 0.066 | 0.863 | 0.003 | 20.167 |
|  | rs113651041 | -0.037 | 0.380 | 0.033 | 0.259 | 0.003 | 19.538 |
|  | rs115647337 | 0.054 | 0.042 | 0.079 | 0.496 | 0.003 | 21.698 |
|  | rs11591147 | 0.081 | 0.036 | 0.085 | 0.338 | 0.004 | 25.783 |
|  | rs11633878 | -0.019 | 0.717 | 0.035 | 0.595 | 0.003 | 22.651 |
|  | rs116794590 | -0.050 | 0.024 | 0.102 | 0.622 | 0.003 | 21.674 |
|  | rs11696517 | 0.095 | 0.164 | 0.043 | 0.026 | 0.003 | 19.916 |
|  | rs117061362 | 0.023 | 0.048 | 0.075 | 0.755 | 0.003 | 23.519 |
|  | rs149229417 | 0.031 | 0.017 | 0.123 | 0.803 | 0.003 | 23.251 |
|  | rs17110517 | -0.016 | 0.057 | 0.069 | 0.820 | 0.003 | 24.431 |
|  | rs2075083 | 0.027 | 0.566 | 0.032 | 0.390 | 0.003 | 19.647 |
|  | rs2229738 | 0.076 | 0.155 | 0.044 | 0.087 | 0.003 | 23.485 |
|  | rs35473591 | 0.080 | 0.413 | 0.032 | 0.013 | 0.019 | 141.775 |
|  | rs4072727 | 0.023 | 0.369 | 0.033 | 0.473 | 0.004 | 29.085 |
|  | rs72657952 | 0.113 | 0.022 | 0.108 | 0.295 | 0.003 | 19.945 |
|  | rs72852391 | -0.069 | 0.040 | 0.081 | 0.397 | 0.003 | 22.458 |
|  | rs73769662 | -0.006 | 0.093 | 0.055 | 0.908 | 0.003 | 19.666 |
|  | rs7412 | 0.006 | 0.053 | 0.071 | 0.934 | 0.007 | 52.155 |
|  | rs75101588 | -0.034 | 0.029 | 0.094 | 0.719 | 0.004 | 26.866 |
|  | rs77456635 | 0.132 | 0.027 | 0.099 | 0.184 | 0.003 | 25.055 |
|  | rs77708260 | -0.068 | 0.026 | 0.099 | 0.493 | 0.003 | 20.104 |
|  | rs9559135 | 0.000 | 0.281 | 0.035 | 0.990 | 0.003 | 21.738 |
| Diacylglycerol (16:1_18:1) | rs10022344 | 0.011 | 0.191 | 0.040 | 0.794 | 0.003 | 19.866 |
|  | rs10502551 | 0.087 | 0.051 | 0.072 | 0.223 | 0.004 | 26.166 |
|  | rs111750990 | 0.005 | 0.040 | 0.080 | 0.955 | 0.004 | 22.084 |
|  | rs11182480 | 0.024 | 0.018 | 0.118 | 0.839 | 0.004 | 24.611 |
|  | rs112122274 | -0.134 | 0.031 | 0.092 | 0.145 | 0.004 | 24.501 |
|  | rs11775636 | -0.050 | 0.152 | 0.044 | 0.253 | 0.003 | 21.815 |
|  | rs117771237 | -0.018 | 0.011 | 0.152 | 0.906 | 0.003 | 20.219 |
|  | rs12050879 | -0.144 | 0.022 | 0.108 | 0.181 | 0.003 | 19.966 |
|  | rs1260326 | -0.146 | 0.650 | 0.032 | 0.000 | 0.006 | 35.922 |
|  | rs140283718 | -0.207 | 0.025 | 0.101 | 0.040 | 0.003 | 21.672 |
|  | rs143808674 | 0.070 | 0.044 | 0.078 | 0.369 | 0.003 | 19.790 |
|  | rs147782497 | -0.118 | 0.022 | 0.108 | 0.275 | 0.003 | 19.715 |
|  | rs2182380 | 0.095 | 0.030 | 0.093 | 0.306 | 0.004 | 23.001 |
|  | rs2524075 | -0.059 | 0.215 | 0.038 | 0.128 | 0.004 | 24.124 |
|  | rs35162925 | -0.018 | 0.020 | 0.112 | 0.874 | 0.003 | 21.771 |
|  | rs3829088 | 0.040 | 0.208 | 0.039 | 0.309 | 0.003 | 20.629 |
|  | rs4419068 | -0.055 | 0.232 | 0.037 | 0.145 | 0.003 | 19.557 |
|  | rs77134026 | 0.141 | 0.032 | 0.092 | 0.126 | 0.003 | 21.724 |
|  | rs78263224 | 0.143 | 0.041 | 0.080 | 0.073 | 0.003 | 20.058 |
|  | rs7955732 | 0.010 | 0.417 | 0.032 | 0.765 | 0.004 | 23.545 |
|  | rs908150 | -0.027 | 0.419 | 0.032 | 0.401 | 0.003 | 19.623 |
|  | rs964184 | 0.007 | 0.855 | 0.045 | 0.870 | 0.007 | 44.215 |
| Phosphatidylcholine (20:4_0:0) | rs10733608 | 0.036 | 0.478 | 0.032 | 0.257 | 0.003 | 21.648 |
|  | rs10884272 | 0.002 | 0.367 | 0.033 | 0.954 | 0.003 | 20.372 |
|  | rs11669519 | 0.001 | 0.343 | 0.033 | 0.971 | 0.003 | 20.381 |
|  | rs12226389 | -0.005 | 0.178 | 0.041 | 0.900 | 0.009 | 54.075 |
|  | rs12303 | 0.033 | 0.199 | 0.040 | 0.411 | 0.003 | 21.040 |
|  | rs12905466 | 0.036 | 0.201 | 0.039 | 0.364 | 0.003 | 20.862 |
|  | rs1363011 | -0.038 | 0.138 | 0.046 | 0.409 | 0.003 | 19.537 |
|  | rs139516883 | -0.058 | 0.014 | 0.136 | 0.670 | 0.004 | 24.495 |
|  | rs142999489 | 0.039 | 0.056 | 0.069 | 0.572 | 0.004 | 23.796 |
|  | rs150158785 | 0.047 | 0.079 | 0.059 | 0.429 | 0.003 | 20.086 |
|  | rs182611493 | 0.105 | 0.051 | 0.072 | 0.143 | 0.004 | 26.444 |
|  | rs191895023 | 0.193 | 0.003 | 0.328 | 0.557 | 0.003 | 20.639 |
|  | rs200932481 | -0.022 | 0.007 | 0.185 | 0.904 | 0.004 | 22.994 |
|  | rs35473591 | 0.080 | 0.413 | 0.032 | 0.013 | 0.137 | 991.669 |
|  | rs35599386 | -0.040 | 0.050 | 0.073 | 0.578 | 0.003 | 20.136 |
|  | rs3741252 | -0.076 | 0.129 | 0.047 | 0.109 | 0.006 | 39.579 |
|  | rs4708901 | -0.029 | 0.205 | 0.039 | 0.458 | 0.003 | 20.729 |
|  | rs474901 | -0.053 | 0.471 | 0.032 | 0.093 | 0.003 | 19.839 |
|  | rs529446 | 0.043 | 0.784 | 0.038 | 0.268 | 0.003 | 20.001 |
|  | rs600626 | 0.030 | 0.231 | 0.037 | 0.422 | 0.005 | 32.513 |
|  | rs62511275 | 0.006 | 0.043 | 0.079 | 0.936 | 0.004 | 22.556 |
|  | rs6564238 | 0.090 | 0.328 | 0.033 | 0.007 | 0.003 | 20.071 |
|  | rs7179330 | 0.166 | 0.982 | 0.119 | 0.165 | 0.004 | 23.702 |
|  | rs76469829 | -0.009 | 0.035 | 0.086 | 0.918 | 0.003 | 20.617 |
|  | rs76513766 | -0.021 | 0.011 | 0.149 | 0.888 | 0.003 | 20.216 |
|  | rs7936002 | -0.039 | 0.245 | 0.037 | 0.290 | 0.007 | 40.967 |
|  | rs79995028 | -0.022 | 0.004 | 0.259 | 0.932 | 0.003 | 20.562 |
|  | rs9967725 | 0.002 | 0.336 | 0.033 | 0.943 | 0.005 | 29.177 |
| Phosphatidylcholine (16:0_20:4) | rs11062107 | 0.014 | 0.233 | 0.037 | 0.709 | 0.003 | 19.709 |
|  | rs113394924 | -0.031 | 0.083 | 0.057 | 0.587 | 0.007 | 51.998 |
|  | rs116438525 | -0.026 | 0.008 | 0.182 | 0.889 | 0.003 | 21.266 |
|  | rs116643980 | -0.049 | 0.054 | 0.070 | 0.487 | 0.003 | 20.924 |
|  | rs1251003 | 0.017 | 0.090 | 0.056 | 0.756 | 0.003 | 23.562 |
|  | rs12720922 | 0.069 | 0.173 | 0.042 | 0.098 | 0.003 | 19.717 |
|  | rs13329893 | -0.067 | 0.048 | 0.074 | 0.367 | 0.003 | 19.700 |
|  | rs141391095 | 0.019 | 0.025 | 0.099 | 0.847 | 0.003 | 22.132 |
|  | rs147549994 | 0.142 | 0.035 | 0.086 | 0.099 | 0.004 | 29.578 |
|  | rs149130695 | 0.020 | 0.064 | 0.066 | 0.766 | 0.003 | 19.659 |
|  | rs17017629 | -0.022 | 0.027 | 0.097 | 0.822 | 0.003 | 20.281 |
|  | rs17366743 | -0.059 | 0.036 | 0.084 | 0.486 | 0.003 | 19.594 |
|  | rs174527 | 0.023 | 0.452 | 0.032 | 0.473 | 0.015 | 111.162 |
|  | rs174533 | 0.078 | 0.415 | 0.032 | 0.015 | 0.171 | 1480.882 |
|  | rs187429064 | 0.102 | 0.051 | 0.072 | 0.158 | 0.005 | 36.009 |
|  | rs2943813 | 0.009 | 0.792 | 0.039 | 0.818 | 0.006 | 46.464 |
|  | rs2966427 | 0.126 | 0.929 | 0.062 | 0.042 | 0.003 | 20.014 |
|  | rs3018617 | 0.065 | 0.309 | 0.035 | 0.059 | 0.005 | 33.199 |
|  | rs3820438 | -0.033 | 0.288 | 0.035 | 0.342 | 0.003 | 23.703 |
|  | rs4756323 | -0.038 | 0.453 | 0.032 | 0.231 | 0.003 | 21.709 |
|  | rs499974 | 0.030 | 0.231 | 0.037 | 0.428 | 0.004 | 26.845 |
|  | rs58160859 | 0.011 | 0.210 | 0.039 | 0.774 | 0.003 | 20.602 |
|  | rs6498540 | 0.052 | 0.333 | 0.034 | 0.123 | 0.003 | 19.851 |
|  | rs72709529 | -0.144 | 0.022 | 0.107 | 0.181 | 0.003 | 23.734 |
|  | rs74754540 | 0.066 | 0.050 | 0.073 | 0.364 | 0.003 | 21.202 |
| Phosphatidylcholine (17:0_18:1) | rs1106933 | -0.021 | 0.136 | 0.046 | 0.648 | 0.004 | 26.027 |
|  | rs12373089 | -0.016 | 0.135 | 0.046 | 0.736 | 0.003 | 19.858 |
|  | rs1257173 | -0.013 | 0.087 | 0.056 | 0.818 | 0.003 | 19.984 |
|  | rs139070595 | -0.039 | 0.006 | 0.210 | 0.852 | 0.003 | 23.379 |
|  | rs140419225 | -0.135 | 0.006 | 0.212 | 0.524 | 0.003 | 21.607 |
|  | rs141245990 | 0.039 | 0.026 | 0.100 | 0.694 | 0.003 | 21.308 |
|  | rs143506888 | -0.052 | 0.018 | 0.122 | 0.668 | 0.003 | 21.230 |
|  | rs145967151 | 0.037 | 0.134 | 0.047 | 0.429 | 0.003 | 19.548 |
|  | rs17030373 | -0.006 | 0.296 | 0.035 | 0.859 | 0.003 | 19.611 |
|  | rs17231506 | -0.004 | 0.279 | 0.035 | 0.899 | 0.004 | 25.009 |
|  | rs187552376 | 0.135 | 0.016 | 0.127 | 0.290 | 0.003 | 20.484 |
|  | rs2003890 | 0.028 | 0.223 | 0.038 | 0.468 | 0.003 | 21.032 |
|  | rs2126263 | 0.013 | 0.854 | 0.045 | 0.773 | 0.004 | 27.121 |
|  | rs4692808 | -0.031 | 0.429 | 0.032 | 0.328 | 0.003 | 22.549 |
|  | rs5022521 | 0.017 | 0.808 | 0.041 | 0.685 | 0.003 | 20.342 |
|  | rs61737926 | 0.173 | 0.004 | 0.257 | 0.501 | 0.003 | 21.245 |
|  | rs7350789 | -0.013 | 0.386 | 0.032 | 0.691 | 0.006 | 43.520 |
|  | rs76157703 | 0.342 | 0.014 | 0.136 | 0.012 | 0.003 | 20.585 |
|  | rs77534789 | 0.086 | 0.041 | 0.081 | 0.288 | 0.003 | 21.233 |
|  | rs78308012 | 0.013 | 0.105 | 0.052 | 0.805 | 0.004 | 28.223 |
| Phosphatidylcholine (17:0_18:2) | rs11027519 | 0.055 | 0.091 | 0.055 | 0.317 | 0.003 | 19.950 |
|  | rs111233850 | -0.126 | 0.032 | 0.091 | 0.164 | 0.003 | 20.199 |
|  | rs111314022 | 0.042 | 0.032 | 0.090 | 0.641 | 0.003 | 20.345 |
|  | rs115686032 | -0.136 | 0.041 | 0.080 | 0.091 | 0.003 | 21.606 |
|  | rs1168104 | 0.007 | 0.736 | 0.036 | 0.835 | 0.003 | 20.877 |
|  | rs1253283 | 0.065 | 0.030 | 0.094 | 0.489 | 0.003 | 19.660 |
|  | rs12548274 | 0.017 | 0.163 | 0.043 | 0.696 | 0.003 | 22.095 |
|  | rs141509040 | -0.057 | 0.073 | 0.060 | 0.342 | 0.003 | 20.086 |
|  | rs1609446 | 0.053 | 0.081 | 0.059 | 0.365 | 0.003 | 19.967 |
|  | rs17231506 | -0.004 | 0.279 | 0.035 | 0.899 | 0.004 | 27.186 |
|  | rs174566 | 0.082 | 0.413 | 0.032 | 0.010 | 0.032 | 237.533 |
|  | rs1800588 | -0.081 | 0.248 | 0.037 | 0.027 | 0.005 | 35.068 |
|  | rs192537901 | 0.026 | 0.028 | 0.096 | 0.785 | 0.003 | 23.785 |
|  | rs2126260 | -0.004 | 0.747 | 0.036 | 0.917 | 0.003 | 22.916 |
|  | rs34489224 | -0.007 | 0.273 | 0.036 | 0.846 | 0.003 | 21.413 |
|  | rs422137 | -0.017 | 0.487 | 0.032 | 0.594 | 0.004 | 25.903 |
|  | rs4478142 | 0.013 | 0.157 | 0.043 | 0.762 | 0.003 | 20.039 |
|  | rs4840981 | -0.004 | 0.988 | 0.147 | 0.979 | 0.003 | 21.265 |
|  | rs57518551 | -0.089 | 0.142 | 0.045 | 0.049 | 0.004 | 25.838 |
|  | rs6498540 | 0.052 | 0.333 | 0.034 | 0.123 | 0.004 | 25.828 |
|  | rs72747028 | -0.152 | 0.068 | 0.063 | 0.016 | 0.003 | 21.691 |
|  | rs73058910 | 0.015 | 0.067 | 0.063 | 0.819 | 0.003 | 21.390 |
|  | rs7437408 | 0.011 | 0.481 | 0.032 | 0.739 | 0.003 | 21.048 |
|  | rs78592970 | -0.013 | 0.123 | 0.049 | 0.797 | 0.004 | 25.234 |
|  | rs964184 | 0.007 | 0.855 | 0.045 | 0.870 | 0.006 | 44.464 |
| Phosphatidylcholine (18:0_20:4) | rs1219539 | 0.040 | 0.491 | 0.032 | 0.203 | 0.004 | 27.346 |
|  | rs12275418 | -0.109 | 0.133 | 0.047 | 0.019 | 0.004 | 25.436 |
|  | rs139102782 | -0.083 | 0.034 | 0.088 | 0.342 | 0.003 | 19.724 |
|  | rs144719617 | -0.008 | 0.105 | 0.051 | 0.881 | 0.003 | 22.050 |
|  | rs147549994 | 0.142 | 0.035 | 0.086 | 0.099 | 0.004 | 32.312 |
|  | rs1477491 | -0.038 | 0.798 | 0.039 | 0.335 | 0.004 | 25.279 |
|  | rs147981159 | -0.053 | 0.044 | 0.077 | 0.492 | 0.009 | 68.422 |
|  | rs174528 | 0.079 | 0.420 | 0.032 | 0.014 | 0.213 | 1945.604 |
|  | rs187429064 | 0.102 | 0.051 | 0.072 | 0.158 | 0.004 | 29.000 |
|  | rs2070895 | -0.092 | 0.258 | 0.036 | 0.010 | 0.003 | 24.226 |
|  | rs223841 | 0.059 | 0.169 | 0.042 | 0.165 | 0.003 | 21.924 |
|  | rs2797620 | -0.003 | 0.329 | 0.034 | 0.935 | 0.005 | 33.869 |
|  | rs2802390 | -0.006 | 0.463 | 0.032 | 0.862 | 0.003 | 20.080 |
|  | rs2990536 | 0.030 | 0.581 | 0.032 | 0.353 | 0.003 | 21.561 |
|  | rs3019200 | 0.017 | 0.808 | 0.040 | 0.676 | 0.008 | 58.257 |
|  | rs3110971 | 0.062 | 0.205 | 0.039 | 0.110 | 0.003 | 22.029 |
|  | rs3741252 | -0.076 | 0.129 | 0.047 | 0.109 | 0.013 | 98.083 |
|  | rs4382917 | -0.035 | 0.243 | 0.037 | 0.350 | 0.008 | 57.065 |
|  | rs600518 | 0.030 | 0.231 | 0.037 | 0.427 | 0.004 | 25.996 |
|  | rs61873274 | 0.017 | 0.076 | 0.060 | 0.777 | 0.003 | 20.835 |
|  | rs6860806 | 0.101 | 0.418 | 0.032 | 0.001 | 0.003 | 21.989 |
|  | rs72789541 | 0.060 | 0.316 | 0.034 | 0.078 | 0.004 | 29.299 |
|  | rs73005469 | 0.029 | 0.360 | 0.033 | 0.371 | 0.003 | 19.653 |
|  | rs7499892 | 0.060 | 0.172 | 0.042 | 0.151 | 0.003 | 19.697 |
|  | rs79136768 | 0.042 | 0.036 | 0.085 | 0.620 | 0.004 | 30.967 |
|  | rs793864 | -0.070 | 0.818 | 0.041 | 0.088 | 0.003 | 23.174 |
|  | rs938822 | -0.015 | 0.146 | 0.045 | 0.738 | 0.003 | 20.998 |
|  | rs9462860 | 0.003 | 0.449 | 0.032 | 0.931 | 0.003 | 21.000 |
| Phosphatidylcholine (18:1_20:2) | rs10846753 | 0.052 | 0.231 | 0.037 | 0.168 | 0.004 | 24.351 |
|  | rs114139553 | -0.144 | 0.013 | 0.137 | 0.294 | 0.004 | 21.015 |
|  | rs116922907 | 0.148 | 0.045 | 0.076 | 0.054 | 0.004 | 20.854 |
|  | rs117534325 | 0.050 | 0.005 | 0.235 | 0.832 | 0.004 | 22.223 |
|  | rs118150981 | -0.030 | 0.020 | 0.114 | 0.793 | 0.004 | 21.051 |
|  | rs12113068 | 0.016 | 0.023 | 0.106 | 0.880 | 0.003 | 19.669 |
|  | rs12417747 | -0.025 | 0.115 | 0.050 | 0.613 | 0.007 | 43.007 |
|  | rs174527 | 0.023 | 0.452 | 0.032 | 0.473 | 0.004 | 24.547 |
|  | rs174580 | 0.079 | 0.415 | 0.032 | 0.013 | 0.090 | 580.696 |
|  | rs17543793 | -0.056 | 0.020 | 0.114 | 0.627 | 0.003 | 20.322 |
|  | rs1800588 | -0.081 | 0.248 | 0.037 | 0.027 | 0.005 | 27.543 |
|  | rs265114 | -0.026 | 0.074 | 0.061 | 0.670 | 0.004 | 23.847 |
|  | rs35195855 | 0.025 | 0.143 | 0.046 | 0.589 | 0.004 | 21.787 |
|  | rs56208677 | -0.042 | 0.076 | 0.060 | 0.482 | 0.005 | 26.554 |
|  | rs61893829 | 0.033 | 0.086 | 0.057 | 0.557 | 0.004 | 21.308 |
|  | rs656095 | 0.070 | 0.222 | 0.038 | 0.064 | 0.006 | 34.601 |
|  | rs6944011 | 0.030 | 0.208 | 0.039 | 0.441 | 0.003 | 20.087 |
|  | rs6984305 | -0.002 | 0.841 | 0.043 | 0.961 | 0.004 | 23.463 |
|  | rs7041283 | -0.042 | 0.246 | 0.037 | 0.253 | 0.005 | 29.495 |
|  | rs7176661 | 0.057 | 0.597 | 0.032 | 0.075 | 0.003 | 19.838 |
|  | rs73292027 | 0.012 | 0.105 | 0.052 | 0.813 | 0.004 | 24.859 |
|  | rs74089903 | -0.177 | 0.009 | 0.167 | 0.289 | 0.005 | 27.553 |
|  | rs80201535 | -0.041 | 0.028 | 0.097 | 0.674 | 0.004 | 21.230 |
|  | rs9384216 | 0.013 | 0.098 | 0.053 | 0.805 | 0.003 | 20.209 |
| Phosphatidylcholine (18:1_20:4) | rs1077834 | -0.093 | 0.257 | 0.036 | 0.010 | 0.004 | 26.370 |
|  | rs10793123 | 0.043 | 0.487 | 0.032 | 0.176 | 0.003 | 20.821 |
|  | rs10889353 | -0.010 | 0.264 | 0.036 | 0.779 | 0.003 | 20.530 |
|  | rs113049169 | -0.226 | 0.032 | 0.090 | 0.013 | 0.003 | 19.680 |
|  | rs113394924 | -0.031 | 0.083 | 0.057 | 0.587 | 0.005 | 39.462 |
|  | rs113913626 | 0.160 | 0.035 | 0.087 | 0.067 | 0.003 | 19.533 |
|  | rs116814948 | 0.114 | 0.010 | 0.159 | 0.471 | 0.003 | 20.447 |
|  | rs147549994 | 0.142 | 0.035 | 0.086 | 0.099 | 0.004 | 26.191 |
|  | rs147797702 | 0.313 | 0.010 | 0.158 | 0.047 | 0.004 | 30.191 |
|  | rs173539 | -0.001 | 0.283 | 0.035 | 0.975 | 0.003 | 21.167 |
|  | rs174527 | 0.023 | 0.452 | 0.032 | 0.473 | 0.012 | 89.070 |
|  | rs174537 | 0.080 | 0.413 | 0.032 | 0.012 | 0.123 | 1006.817 |
|  | rs187429064 | 0.102 | 0.051 | 0.072 | 0.158 | 0.007 | 53.611 |
|  | rs1917701 | -0.012 | 0.945 | 0.070 | 0.859 | 0.003 | 20.013 |
|  | rs2943813 | 0.009 | 0.792 | 0.039 | 0.818 | 0.006 | 40.322 |
|  | rs3018617 | 0.065 | 0.309 | 0.035 | 0.059 | 0.003 | 23.807 |
|  | rs4628166 | -0.017 | 0.631 | 0.033 | 0.600 | 0.003 | 19.699 |
|  | rs4846905 | -0.026 | 0.765 | 0.037 | 0.477 | 0.003 | 23.180 |
|  | rs531117 | 0.030 | 0.231 | 0.037 | 0.419 | 0.005 | 32.924 |
|  | rs6083838 | 0.019 | 0.533 | 0.032 | 0.545 | 0.003 | 19.625 |
|  | rs61978272 | 0.003 | 0.276 | 0.036 | 0.938 | 0.004 | 27.749 |
|  | rs641738 | 0.042 | 0.604 | 0.032 | 0.193 | 0.003 | 24.969 |
|  | rs6850591 | -0.013 | 0.549 | 0.032 | 0.680 | 0.003 | 23.007 |
|  | rs73176681 | 0.088 | 0.047 | 0.075 | 0.239 | 0.003 | 20.807 |
|  | rs73618588 | 0.002 | 0.090 | 0.055 | 0.969 | 0.003 | 20.508 |
|  | rs75976069 | 0.023 | 0.026 | 0.100 | 0.821 | 0.003 | 23.447 |
|  | rs76582138 | -0.054 | 0.009 | 0.172 | 0.752 | 0.003 | 20.340 |
|  | rs79113397 | 0.053 | 0.234 | 0.037 | 0.160 | 0.003 | 24.493 |
|  | rs793864 | -0.070 | 0.818 | 0.041 | 0.088 | 0.003 | 21.873 |
|  | rs8107974 | 0.002 | 0.064 | 0.064 | 0.974 | 0.004 | 30.771 |
| Phosphatidylcholine (O-18:0_20:4) | rs11057853 | -0.081 | 0.451 | 0.032 | 0.010 | 0.003 | 20.476 |
|  | rs117870830 | 0.039 | 0.020 | 0.113 | 0.732 | 0.003 | 20.689 |
|  | rs11954170 | -0.019 | 0.314 | 0.034 | 0.573 | 0.003 | 20.489 |
|  | rs138202451 | 0.059 | 0.009 | 0.174 | 0.735 | 0.004 | 26.787 |
|  | rs143233558 | -0.056 | 0.094 | 0.055 | 0.306 | 0.004 | 27.577 |
|  | rs145755646 | -0.138 | 0.063 | 0.066 | 0.035 | 0.003 | 21.195 |
|  | rs148462060 | 0.146 | 0.024 | 0.103 | 0.156 | 0.003 | 21.660 |
|  | rs148840717 | 0.004 | 0.035 | 0.086 | 0.963 | 0.003 | 19.740 |
|  | rs174536 | 0.080 | 0.414 | 0.032 | 0.013 | 0.045 | 331.183 |
|  | rs1800588 | -0.081 | 0.248 | 0.037 | 0.027 | 0.004 | 27.560 |
|  | rs34382810 | -0.026 | 0.364 | 0.033 | 0.434 | 0.003 | 23.749 |
|  | rs34737685 | -0.041 | 0.529 | 0.032 | 0.197 | 0.003 | 20.634 |
|  | rs3741252 | -0.076 | 0.129 | 0.047 | 0.109 | 0.005 | 36.061 |
|  | rs376516 | -0.395 | 0.000 | 0.833 | 0.636 | 0.003 | 19.830 |
|  | rs4706511 | 0.047 | 0.191 | 0.041 | 0.244 | 0.003 | 19.541 |
|  | rs4782783 | 0.004 | 0.886 | 0.050 | 0.939 | 0.003 | 21.473 |
|  | rs6992951 | -0.011 | 0.394 | 0.032 | 0.733 | 0.003 | 22.579 |
|  | rs73074943 | 0.180 | 0.025 | 0.101 | 0.073 | 0.003 | 20.667 |
|  | rs7412 | 0.006 | 0.053 | 0.071 | 0.934 | 0.004 | 26.340 |
|  | rs78711160 | -0.077 | 0.043 | 0.077 | 0.317 | 0.003 | 21.060 |
|  | rs79856406 | 0.103 | 0.101 | 0.053 | 0.050 | 0.003 | 19.540 |
|  | rs9591400 | 0.052 | 0.115 | 0.050 | 0.296 | 0.003 | 20.623 |
| Phosphatidylethanolamine (O-18:1_20:4) | rs10234951 | 0.027 | 0.082 | 0.058 | 0.642 | 0.003 | 22.946 |
|  | rs10520189 | -0.062 | 0.115 | 0.050 | 0.216 | 0.003 | 20.071 |
|  | rs10756627 | -0.071 | 0.539 | 0.032 | 0.025 | 0.003 | 19.950 |
|  | rs11062287 | -0.162 | 0.033 | 0.088 | 0.067 | 0.004 | 25.529 |
|  | rs113913626 | 0.160 | 0.035 | 0.087 | 0.067 | 0.003 | 21.676 |
|  | rs114325590 | -0.077 | 0.012 | 0.150 | 0.608 | 0.003 | 19.649 |
|  | rs117002537 | 0.111 | 0.016 | 0.126 | 0.379 | 0.003 | 19.840 |
|  | rs11725298 | -0.080 | 0.025 | 0.102 | 0.431 | 0.003 | 20.358 |
|  | rs117565387 | -0.028 | 0.048 | 0.074 | 0.704 | 0.003 | 21.544 |
|  | rs1203148 | -0.044 | 0.247 | 0.037 | 0.231 | 0.003 | 20.124 |
|  | rs13269712 | -0.023 | 0.159 | 0.044 | 0.599 | 0.003 | 20.266 |
|  | rs141622900 | 0.098 | 0.032 | 0.090 | 0.280 | 0.003 | 24.790 |
|  | rs1456608 | -0.013 | 0.758 | 0.037 | 0.727 | 0.003 | 20.534 |
|  | rs145926488 | -0.073 | 0.007 | 0.195 | 0.707 | 0.003 | 19.713 |
|  | rs1513795 | -0.012 | 0.442 | 0.032 | 0.714 | 0.003 | 22.214 |
|  | rs174527 | 0.023 | 0.452 | 0.032 | 0.473 | 0.003 | 21.308 |
|  | rs174536 | 0.080 | 0.414 | 0.032 | 0.013 | 0.030 | 222.971 |
|  | rs17703316 | -0.002 | 0.046 | 0.076 | 0.977 | 0.003 | 21.176 |
|  | rs204468 | -0.003 | 0.595 | 0.032 | 0.924 | 0.004 | 31.242 |
|  | rs2277289 | -0.045 | 0.325 | 0.034 | 0.186 | 0.004 | 26.099 |
|  | rs2705007 | 0.194 | 0.990 | 0.168 | 0.247 | 0.003 | 20.818 |
|  | rs311416 | -0.004 | 0.652 | 0.033 | 0.913 | 0.003 | 20.336 |
|  | rs35427954 | 0.036 | 0.086 | 0.057 | 0.522 | 0.003 | 22.358 |
|  | rs3820438 | -0.033 | 0.288 | 0.035 | 0.342 | 0.003 | 19.862 |
|  | rs41376249 | -0.020 | 0.161 | 0.043 | 0.640 | 0.003 | 22.550 |
|  | rs4525452 | 0.055 | 0.397 | 0.032 | 0.088 | 0.003 | 20.152 |
|  | rs6601694 | -0.023 | 0.319 | 0.034 | 0.505 | 0.003 | 24.197 |
|  | rs71420341 | -0.048 | 0.087 | 0.056 | 0.390 | 0.003 | 24.506 |
|  | rs72635955 | -0.023 | 0.129 | 0.047 | 0.624 | 0.004 | 25.240 |
| Sphingomyelin (d42:2) | rs10252648 | -0.164 | 0.978 | 0.107 | 0.127 | 0.003 | 19.542 |
|  | rs112585713 | -0.069 | 0.030 | 0.094 | 0.460 | 0.005 | 36.193 |
|  | rs114483871 | 0.013 | 0.036 | 0.085 | 0.875 | 0.005 | 38.862 |
|  | rs115350073 | 0.140 | 0.016 | 0.129 | 0.277 | 0.003 | 21.517 |
|  | rs11591147 | 0.081 | 0.036 | 0.085 | 0.338 | 0.008 | 60.504 |
|  | rs11610836 | -0.070 | 0.419 | 0.032 | 0.030 | 0.003 | 22.885 |
|  | rs116893213 | -0.190 | 0.036 | 0.085 | 0.026 | 0.004 | 25.657 |
|  | rs116919723 | 0.303 | 0.008 | 0.180 | 0.093 | 0.003 | 20.069 |
|  | rs118068660 | -0.065 | 0.102 | 0.052 | 0.209 | 0.008 | 60.902 |
|  | rs12530474 | 0.044 | 0.137 | 0.046 | 0.336 | 0.003 | 21.039 |
|  | rs1367117 | -0.011 | 0.280 | 0.035 | 0.749 | 0.004 | 29.151 |
|  | rs139474488 | -0.003 | 0.016 | 0.126 | 0.984 | 0.003 | 20.509 |
|  | rs146331166 | -0.011 | 0.062 | 0.066 | 0.870 | 0.006 | 40.090 |
|  | rs17018185 | 0.012 | 0.271 | 0.036 | 0.744 | 0.003 | 19.894 |
|  | rs17110517 | -0.016 | 0.057 | 0.069 | 0.820 | 0.003 | 22.389 |
|  | rs17145750 | -0.074 | 0.148 | 0.044 | 0.098 | 0.004 | 30.720 |
|  | rs17261772 | -0.031 | 0.308 | 0.035 | 0.364 | 0.004 | 25.763 |
|  | rs17276933 | -0.011 | 0.064 | 0.065 | 0.870 | 0.003 | 22.524 |
|  | rs182695896 | 0.031 | 0.026 | 0.100 | 0.757 | 0.007 | 52.244 |
|  | rs184845520 | 0.234 | 0.005 | 0.222 | 0.291 | 0.003 | 21.069 |
|  | rs2034007 | -0.012 | 0.305 | 0.034 | 0.734 | 0.003 | 20.080 |
|  | rs2177333 | -0.021 | 0.530 | 0.032 | 0.517 | 0.003 | 20.018 |
|  | rs3751934 | -0.049 | 0.423 | 0.032 | 0.130 | 0.003 | 20.746 |
|  | rs3912622 | -0.007 | 0.200 | 0.039 | 0.861 | 0.004 | 27.078 |
|  | rs4100654 | -0.004 | 0.076 | 0.061 | 0.943 | 0.003 | 21.412 |
|  | rs4283208 | -0.049 | 0.834 | 0.043 | 0.253 | 0.003 | 20.344 |
|  | rs515081 | 0.062 | 0.694 | 0.035 | 0.075 | 0.003 | 23.446 |
|  | rs59916403 | 0.033 | 0.410 | 0.032 | 0.299 | 0.003 | 21.364 |
|  | rs62274367 | 0.433 | 0.004 | 0.254 | 0.087 | 0.003 | 21.826 |
|  | rs6680361 | -0.052 | 0.800 | 0.039 | 0.189 | 0.003 | 21.051 |
|  | rs67718253 | -0.005 | 0.173 | 0.042 | 0.900 | 0.003 | 21.468 |
|  | rs6843498 | 0.039 | 0.309 | 0.034 | 0.252 | 0.004 | 25.306 |
|  | rs7104343 | -0.023 | 0.212 | 0.039 | 0.551 | 0.003 | 20.238 |
|  | rs7247433 | -0.006 | 0.392 | 0.032 | 0.848 | 0.003 | 20.702 |
|  | rs73047641 | 0.015 | 0.495 | 0.032 | 0.632 | 0.003 | 22.905 |
|  | rs7412 | 0.006 | 0.053 | 0.071 | 0.934 | 0.004 | 32.181 |
|  | rs75444922 | 0.015 | 0.022 | 0.109 | 0.888 | 0.003 | 19.641 |
|  | rs75679663 | 0.127 | 0.028 | 0.096 | 0.183 | 0.014 | 101.344 |
| Triacylglycerol (52:2) | rs1010313 | 0.000 | 0.199 | 0.040 | 0.994 | 0.003 | 21.593 |
|  | rs10147474 | 0.055 | 0.038 | 0.082 | 0.500 | 0.003 | 23.430 |
|  | rs10169217 | 0.021 | 0.674 | 0.034 | 0.537 | 0.003 | 22.129 |
|  | rs1042034 | -0.035 | 0.732 | 0.036 | 0.330 | 0.003 | 23.485 |
|  | rs11017845 | -0.068 | 0.051 | 0.072 | 0.340 | 0.003 | 19.602 |
|  | rs112122274 | -0.134 | 0.031 | 0.092 | 0.145 | 0.003 | 24.281 |
|  | rs1260326 | -0.146 | 0.650 | 0.032 | 0.000 | 0.007 | 48.984 |
|  | rs144241030 | 0.037 | 0.087 | 0.056 | 0.505 | 0.003 | 21.684 |
|  | rs147987331 | -0.091 | 0.021 | 0.112 | 0.417 | 0.003 | 21.311 |
|  | rs150741556 | 0.131 | 0.025 | 0.101 | 0.195 | 0.003 | 20.635 |
|  | rs15285 | -0.065 | 0.257 | 0.036 | 0.072 | 0.004 | 31.415 |
|  | rs2980888 | 0.006 | 0.741 | 0.036 | 0.859 | 0.003 | 20.742 |
|  | rs34621709 | -0.002 | 0.174 | 0.042 | 0.967 | 0.003 | 24.992 |
|  | rs35162925 | -0.018 | 0.020 | 0.112 | 0.874 | 0.003 | 20.769 |
|  | rs35332062 | -0.100 | 0.129 | 0.047 | 0.034 | 0.004 | 30.725 |
|  | rs3770813 | -0.155 | 0.026 | 0.099 | 0.118 | 0.003 | 21.566 |
|  | rs501474 | -0.012 | 0.434 | 0.032 | 0.716 | 0.003 | 19.776 |
|  | rs62291572 | -0.043 | 0.210 | 0.039 | 0.266 | 0.003 | 20.543 |
|  | rs72753379 | 0.066 | 0.036 | 0.085 | 0.433 | 0.003 | 21.254 |
|  | rs76950187 | 0.006 | 0.078 | 0.059 | 0.916 | 0.003 | 23.127 |
|  | rs78275146 | -0.023 | 0.014 | 0.134 | 0.865 | 0.003 | 21.549 |
|  | rs79934453 | -0.029 | 0.004 | 0.245 | 0.904 | 0.003 | 22.070 |
|  | rs79954170 | 0.042 | 0.009 | 0.165 | 0.800 | 0.003 | 21.366 |
|  | rs964184 | 0.007 | 0.855 | 0.045 | 0.870 | 0.016 | 115.906 |
| Triacylglycerol (52:3) | rs1010313 | 0.000 | 0.199 | 0.040 | 0.994 | 0.003 | 24.665 |
|  | rs10105606 | -0.055 | 0.276 | 0.035 | 0.118 | 0.007 | 48.950 |
|  | rs1042034 | -0.035 | 0.732 | 0.036 | 0.330 | 0.005 | 34.743 |
|  | rs116011373 | 0.012 | 0.049 | 0.073 | 0.867 | 0.003 | 20.143 |
|  | rs116843064 | 0.023 | 0.027 | 0.096 | 0.815 | 0.003 | 22.047 |
|  | rs117950471 | 0.141 | 0.043 | 0.078 | 0.069 | 0.003 | 21.697 |
|  | rs12365864 | 0.015 | 0.159 | 0.043 | 0.730 | 0.003 | 24.733 |
|  | rs1260326 | -0.146 | 0.650 | 0.032 | 0.000 | 0.009 | 61.872 |
|  | rs144072769 | 0.137 | 0.063 | 0.065 | 0.036 | 0.003 | 21.949 |
|  | rs144313866 | -0.095 | 0.074 | 0.061 | 0.120 | 0.003 | 19.779 |
|  | rs147987331 | -0.091 | 0.021 | 0.112 | 0.417 | 0.003 | 21.164 |
|  | rs16913024 | 0.074 | 0.006 | 0.216 | 0.731 | 0.003 | 19.569 |
|  | rs174560 | 0.070 | 0.388 | 0.032 | 0.031 | 0.003 | 19.792 |
|  | rs187429064 | 0.102 | 0.051 | 0.072 | 0.158 | 0.003 | 24.000 |
|  | rs2352723 | -0.026 | 0.765 | 0.037 | 0.489 | 0.005 | 33.928 |
|  | rs2980888 | 0.006 | 0.741 | 0.036 | 0.859 | 0.003 | 21.544 |
|  | rs34621709 | -0.002 | 0.174 | 0.042 | 0.967 | 0.004 | 26.828 |
|  | rs35332062 | -0.100 | 0.129 | 0.047 | 0.034 | 0.006 | 39.703 |
|  | rs35986054 | 0.015 | 0.204 | 0.039 | 0.710 | 0.003 | 19.617 |
|  | rs55724405 | -0.020 | 0.279 | 0.035 | 0.562 | 0.003 | 19.620 |
|  | rs57500102 | -0.081 | 0.008 | 0.181 | 0.654 | 0.003 | 21.780 |
|  | rs61702480 | 0.017 | 0.275 | 0.035 | 0.637 | 0.003 | 19.925 |
|  | rs6554273 | -0.018 | 0.461 | 0.032 | 0.564 | 0.003 | 19.843 |
|  | rs72836628 | -0.044 | 0.290 | 0.035 | 0.202 | 0.003 | 19.712 |
|  | rs75237020 | 0.023 | 0.032 | 0.089 | 0.796 | 0.003 | 20.877 |
|  | rs75573052 | -0.136 | 0.090 | 0.055 | 0.014 | 0.003 | 20.621 |
|  | rs76078494 | 0.130 | 0.035 | 0.087 | 0.133 | 0.003 | 20.291 |
|  | rs79954170 | 0.042 | 0.009 | 0.165 | 0.800 | 0.004 | 28.513 |
|  | rs964184 | 0.007 | 0.855 | 0.045 | 0.870 | 0.023 | 169.659 |
|  | rs9997195 | 0.000 | 0.618 | 0.033 | 0.993 | 0.003 | 22.109 |
| Triacylglycerol (52:4) | rs10105606 | -0.055 | 0.276 | 0.035 | 0.118 | 0.006 | 45.749 |
|  | rs1042034 | -0.035 | 0.732 | 0.036 | 0.330 | 0.005 | 32.424 |
|  | rs10773708 | -0.039 | 0.677 | 0.034 | 0.243 | 0.003 | 20.451 |
|  | rs10779836 | -0.015 | 0.771 | 0.038 | 0.700 | 0.005 | 34.300 |
|  | rs113672231 | 0.069 | 0.083 | 0.058 | 0.231 | 0.003 | 20.093 |
|  | rs116497138 | 0.022 | 0.053 | 0.071 | 0.751 | 0.003 | 21.837 |
|  | rs11783556 | -0.040 | 0.200 | 0.040 | 0.316 | 0.003 | 20.491 |
|  | rs12365864 | 0.015 | 0.159 | 0.043 | 0.730 | 0.003 | 21.701 |
|  | rs1260326 | -0.146 | 0.650 | 0.032 | 0.000 | 0.009 | 63.534 |
|  | rs13259370 | 0.000 | 0.199 | 0.040 | 0.991 | 0.003 | 22.524 |
|  | rs13261274 | 0.019 | 0.535 | 0.032 | 0.557 | 0.003 | 20.002 |
|  | rs142146849 | 0.112 | 0.062 | 0.066 | 0.089 | 0.003 | 19.567 |
|  | rs144072769 | 0.137 | 0.063 | 0.065 | 0.036 | 0.003 | 23.656 |
|  | rs157582 | 0.037 | 0.230 | 0.038 | 0.327 | 0.003 | 22.367 |
|  | rs187429064 | 0.102 | 0.051 | 0.072 | 0.158 | 0.004 | 29.825 |
|  | rs34621709 | -0.002 | 0.174 | 0.042 | 0.967 | 0.004 | 26.087 |
|  | rs56051325 | -0.006 | 0.302 | 0.035 | 0.873 | 0.003 | 22.911 |
|  | rs56376607 | 0.040 | 0.030 | 0.095 | 0.672 | 0.003 | 19.683 |
|  | rs61156392 | 0.038 | 0.130 | 0.047 | 0.413 | 0.003 | 20.264 |
|  | rs61702480 | 0.017 | 0.275 | 0.035 | 0.637 | 0.003 | 19.659 |
|  | rs72900657 | -0.066 | 0.026 | 0.101 | 0.511 | 0.003 | 20.491 |
|  | rs76330538 | -0.155 | 0.018 | 0.120 | 0.196 | 0.003 | 19.580 |
|  | rs79624003 | -0.095 | 0.129 | 0.047 | 0.043 | 0.004 | 30.972 |
|  | rs79954170 | 0.042 | 0.009 | 0.165 | 0.800 | 0.003 | 23.687 |
|  | rs9344829 | 0.001 | 0.384 | 0.033 | 0.972 | 0.003 | 20.767 |
|  | rs964184 | 0.007 | 0.855 | 0.045 | 0.870 | 0.021 | 153.514 |
| Triacylglycerol (52:5) | rs10105606 | -0.055 | 0.276 | 0.035 | 0.118 | 0.005 | 36.521 |
|  | rs1042034 | -0.035 | 0.732 | 0.036 | 0.330 | 0.003 | 23.569 |
|  | rs10779836 | -0.015 | 0.771 | 0.038 | 0.700 | 0.003 | 22.356 |
|  | rs111568723 | -0.017 | 0.025 | 0.101 | 0.866 | 0.003 | 19.532 |
|  | rs113672231 | 0.069 | 0.083 | 0.058 | 0.231 | 0.003 | 20.881 |
|  | rs116497138 | 0.022 | 0.053 | 0.071 | 0.751 | 0.003 | 19.861 |
|  | rs11783556 | -0.040 | 0.200 | 0.040 | 0.316 | 0.003 | 21.812 |
|  | rs1260326 | -0.146 | 0.650 | 0.032 | 0.000 | 0.011 | 78.434 |
|  | rs13259370 | 0.000 | 0.199 | 0.040 | 0.991 | 0.003 | 19.999 |
|  | rs142146849 | 0.112 | 0.062 | 0.066 | 0.089 | 0.003 | 22.229 |
|  | rs143467207 | -0.042 | 0.046 | 0.076 | 0.579 | 0.003 | 20.103 |
|  | rs144072769 | 0.137 | 0.063 | 0.065 | 0.036 | 0.003 | 21.934 |
|  | rs157582 | 0.037 | 0.230 | 0.038 | 0.327 | 0.003 | 21.896 |
|  | rs17596144 | -0.024 | 0.146 | 0.045 | 0.595 | 0.003 | 22.121 |
|  | rs187429064 | 0.102 | 0.051 | 0.072 | 0.158 | 0.006 | 40.819 |
|  | rs190411877 | 0.099 | 0.010 | 0.159 | 0.533 | 0.003 | 23.689 |
|  | rs301555 | 0.074 | 0.337 | 0.034 | 0.028 | 0.003 | 21.208 |
|  | rs33951980 | -0.094 | 0.129 | 0.047 | 0.047 | 0.003 | 19.800 |
|  | rs35986054 | 0.015 | 0.204 | 0.039 | 0.710 | 0.003 | 22.000 |
|  | rs4529433 | 0.020 | 0.276 | 0.035 | 0.567 | 0.003 | 19.729 |
|  | rs61156392 | 0.038 | 0.130 | 0.047 | 0.413 | 0.003 | 22.166 |
|  | rs6753694 | -0.037 | 0.284 | 0.035 | 0.285 | 0.003 | 19.926 |
|  | rs72900657 | -0.066 | 0.026 | 0.101 | 0.511 | 0.003 | 22.436 |
|  | rs75560495 | 0.033 | 0.132 | 0.047 | 0.480 | 0.003 | 20.502 |
|  | rs7996305 | -0.087 | 0.247 | 0.037 | 0.017 | 0.003 | 19.962 |
|  | rs9344829 | 0.001 | 0.384 | 0.033 | 0.972 | 0.003 | 21.595 |
|  | rs964184 | 0.007 | 0.855 | 0.045 | 0.870 | 0.016 | 119.115 |
| Triacylglycerol (53:3) | rs1003966 | -0.136 | 0.090 | 0.055 | 0.014 | 0.003 | 24.133 |
|  | rs10147474 | 0.055 | 0.038 | 0.082 | 0.500 | 0.003 | 22.068 |
|  | rs1042034 | -0.035 | 0.732 | 0.036 | 0.330 | 0.004 | 26.961 |
|  | rs111568723 | -0.017 | 0.025 | 0.101 | 0.866 | 0.003 | 20.993 |
|  | rs11584070 | -0.001 | 0.143 | 0.045 | 0.979 | 0.003 | 21.957 |
|  | rs116011373 | 0.012 | 0.049 | 0.073 | 0.867 | 0.003 | 23.932 |
|  | rs12448223 | 0.010 | 0.446 | 0.032 | 0.764 | 0.003 | 21.404 |
|  | rs1260326 | -0.146 | 0.650 | 0.032 | 0.000 | 0.009 | 61.767 |
|  | rs12635725 | -0.035 | 0.563 | 0.032 | 0.270 | 0.003 | 23.226 |
|  | rs138622921 | -0.246 | 0.018 | 0.119 | 0.038 | 0.003 | 20.257 |
|  | rs139500046 | 0.159 | 0.013 | 0.141 | 0.260 | 0.003 | 23.104 |
|  | rs150741556 | 0.131 | 0.025 | 0.101 | 0.195 | 0.003 | 24.118 |
|  | rs15285 | -0.065 | 0.257 | 0.036 | 0.072 | 0.005 | 34.417 |
|  | rs174560 | 0.070 | 0.388 | 0.032 | 0.031 | 0.005 | 36.095 |
|  | rs1989995 | -0.047 | 0.256 | 0.036 | 0.196 | 0.003 | 20.263 |
|  | rs28450659 | -0.067 | 0.118 | 0.049 | 0.174 | 0.003 | 20.310 |
|  | rs34621709 | -0.002 | 0.174 | 0.042 | 0.967 | 0.005 | 33.014 |
|  | rs35332062 | -0.100 | 0.129 | 0.047 | 0.034 | 0.005 | 33.489 |
|  | rs3829088 | 0.040 | 0.208 | 0.039 | 0.309 | 0.003 | 19.702 |
|  | rs501474 | -0.012 | 0.434 | 0.032 | 0.716 | 0.003 | 20.811 |
|  | rs62291574 | -0.044 | 0.210 | 0.039 | 0.260 | 0.003 | 22.115 |
|  | rs6692319 | -0.021 | 0.740 | 0.036 | 0.554 | 0.004 | 25.545 |
|  | rs76950187 | 0.006 | 0.078 | 0.059 | 0.916 | 0.003 | 21.184 |
|  | rs935833 | -0.042 | 0.633 | 0.033 | 0.204 | 0.003 | 20.165 |
|  | rs964184 | 0.007 | 0.855 | 0.045 | 0.870 | 0.021 | 150.548 |
| Triacylglycerol (54:4) | rs10105606 | -0.055 | 0.276 | 0.035 | 0.118 | 0.006 | 44.902 |
|  | rs1042034 | -0.035 | 0.732 | 0.036 | 0.330 | 0.004 | 30.023 |
|  | rs10773708 | -0.039 | 0.677 | 0.034 | 0.243 | 0.003 | 24.075 |
|  | rs10779833 | -0.009 | 0.659 | 0.033 | 0.792 | 0.004 | 28.046 |
|  | rs115215688 | 0.040 | 0.055 | 0.069 | 0.562 | 0.003 | 21.148 |
|  | rs116843064 | 0.023 | 0.027 | 0.096 | 0.815 | 0.003 | 21.206 |
|  | rs12365864 | 0.015 | 0.159 | 0.043 | 0.730 | 0.004 | 28.906 |
|  | rs1260326 | -0.146 | 0.650 | 0.032 | 0.000 | 0.005 | 36.107 |
|  | rs137871178 | 0.046 | 0.013 | 0.140 | 0.743 | 0.004 | 26.046 |
|  | rs144072769 | 0.137 | 0.063 | 0.065 | 0.036 | 0.003 | 24.730 |
|  | rs17160706 | -0.040 | 0.181 | 0.041 | 0.330 | 0.003 | 20.591 |
|  | rs174604 | 0.046 | 0.382 | 0.033 | 0.161 | 0.004 | 31.865 |
|  | rs189281327 | 0.025 | 0.063 | 0.067 | 0.706 | 0.003 | 19.982 |
|  | rs2642442 | -0.005 | 0.706 | 0.035 | 0.892 | 0.006 | 43.381 |
|  | rs2643912 | 0.057 | 0.446 | 0.032 | 0.072 | 0.003 | 19.648 |
|  | rs2941755 | 0.043 | 0.938 | 0.066 | 0.515 | 0.003 | 21.188 |
|  | rs351596 | -0.246 | 0.977 | 0.105 | 0.019 | 0.003 | 19.640 |
|  | rs3812316 | -0.092 | 0.128 | 0.047 | 0.053 | 0.003 | 23.793 |
|  | rs438811 | 0.034 | 0.235 | 0.037 | 0.366 | 0.003 | 21.462 |
|  | rs56051325 | -0.006 | 0.302 | 0.035 | 0.873 | 0.003 | 21.335 |
|  | rs59017319 | 0.063 | 0.042 | 0.079 | 0.424 | 0.003 | 20.657 |
|  | rs61702480 | 0.017 | 0.275 | 0.035 | 0.637 | 0.003 | 19.819 |
|  | rs62291573 | -0.043 | 0.210 | 0.039 | 0.266 | 0.003 | 21.695 |
|  | rs72836628 | -0.044 | 0.290 | 0.035 | 0.202 | 0.003 | 24.265 |
|  | rs73022136 | 0.009 | 0.044 | 0.079 | 0.912 | 0.003 | 20.877 |
|  | rs78433420 | -0.191 | 0.024 | 0.105 | 0.068 | 0.003 | 20.649 |
|  | rs964184 | 0.007 | 0.855 | 0.045 | 0.870 | 0.023 | 171.554 |
| Triacylglycerol (54:5) | rs10105606 | -0.055 | 0.276 | 0.035 | 0.118 | 0.005 | 38.990 |
|  | rs1042034 | -0.035 | 0.732 | 0.036 | 0.330 | 0.003 | 24.378 |
|  | rs10773708 | -0.039 | 0.677 | 0.034 | 0.243 | 0.003 | 23.275 |
|  | rs115614772 | 0.294 | 0.006 | 0.208 | 0.157 | 0.003 | 20.418 |
|  | rs12365864 | 0.015 | 0.159 | 0.043 | 0.730 | 0.003 | 24.674 |
|  | rs1260326 | -0.146 | 0.650 | 0.032 | 0.000 | 0.007 | 48.687 |
|  | rs13178172 | 0.020 | 0.741 | 0.036 | 0.581 | 0.003 | 23.147 |
|  | rs13246993 | -0.091 | 0.128 | 0.047 | 0.054 | 0.003 | 23.088 |
|  | rs137871178 | 0.046 | 0.013 | 0.140 | 0.743 | 0.003 | 20.310 |
|  | rs139245678 | 0.025 | 0.028 | 0.098 | 0.797 | 0.003 | 19.985 |
|  | rs144072769 | 0.137 | 0.063 | 0.065 | 0.036 | 0.003 | 23.909 |
|  | rs1456674 | 0.100 | 0.008 | 0.178 | 0.575 | 0.003 | 22.534 |
|  | rs147151674 | -0.130 | 0.011 | 0.152 | 0.394 | 0.003 | 20.643 |
|  | rs17160706 | -0.040 | 0.181 | 0.041 | 0.330 | 0.003 | 19.962 |
|  | rs17596144 | -0.024 | 0.146 | 0.045 | 0.595 | 0.004 | 30.869 |
|  | rs2352723 | -0.026 | 0.765 | 0.037 | 0.489 | 0.003 | 24.527 |
|  | rs301555 | 0.074 | 0.337 | 0.034 | 0.028 | 0.003 | 19.738 |
|  | rs351596 | -0.246 | 0.977 | 0.105 | 0.019 | 0.003 | 20.546 |
|  | rs438811 | 0.034 | 0.235 | 0.037 | 0.366 | 0.003 | 22.127 |
|  | rs56051325 | -0.006 | 0.302 | 0.035 | 0.873 | 0.003 | 24.338 |
|  | rs59017319 | 0.063 | 0.042 | 0.079 | 0.424 | 0.003 | 21.198 |
|  | rs664178 | 0.042 | 0.437 | 0.032 | 0.186 | 0.003 | 19.791 |
|  | rs72671922 | 0.078 | 0.061 | 0.066 | 0.240 | 0.003 | 19.617 |
|  | rs74994260 | 0.044 | 0.048 | 0.074 | 0.553 | 0.003 | 23.486 |
|  | rs79380657 | 0.078 | 0.018 | 0.120 | 0.516 | 0.003 | 20.523 |
|  | rs9578336 | 0.022 | 0.172 | 0.042 | 0.604 | 0.003 | 20.089 |
|  | rs964184 | 0.007 | 0.855 | 0.045 | 0.870 | 0.019 | 135.317 |
| Abbreviations: SNPs: single nucleotide polymorphisms; EA: effect allele; OA: other allele; EAF: effect allele frequency; SE: standard error; IBD: inflammatory bowel disease; UC: ulcerative colitis; CD: Crohn's disease. | | | | | | | |

| **Supplementary Table S8. MR analysis results of associations between lipidome and IBD after the final screening.** | | | | | | | | | | | | |
| --- | --- | --- | --- | --- | --- | --- | --- | --- | --- | --- | --- | --- |
| **Plasma lipidome** | **Method** | **OR** | **OR_lci95** | **OR_uci95** | **P** | **Heterogeneity** | | | | **Pleiotropy** | | |
|  |  |  |  |  |  | **MR Egger** | | **IVW** | | **MR-Egger intercep** | | **MR-PRESO** |
|  |  |  |  |  |  | **Q** | **P** | **Q** | **P** | **intercept** | **P** | **P** |
| **IBD** |  |  |  |  |  |  |  |  |  |  |  |  |
| Sterol ester (27:1/22:6) | MR Egger | 0.960 | 0.798 | 1.155 | 0.672 | 28.488 | 0.127 | 29.629 | 0.128 | -0.013 | 0.370 | 0.088 |
|  | Weighted median | 0.963 | 0.866 | 1.071 | 0.486 |  |  |  |  |  |  |  |
|  | IVW | 0.888 | 0.821 | 0.960 | 0.003 |  |  |  |  |  |  |  |
|  | Simple mode | 0.990 | 0.813 | 1.206 | 0.922 |  |  |  |  |  |  |  |
|  | Weighted mode | 0.994 | 0.732 | 1.351 | 0.971 |  |  |  |  |  |  |  |
|  | BWMR | 0.911 | 0.836 | 0.992 | 0.033 |  |  |  |  |  |  |  |
| Phosphatidylcholine (20:4_0:0) | MR Egger | 0.860 | 0.803 | 0.920 | 0.000 | 22.955 | 0.580 | 25.557 | 0.488 | 0.011 | 0.119 | 0.484 |
|  | Weighted median | 0.882 | 0.836 | 0.930 | 0.000 |  |  |  |  |  |  |  |
|  | IVW | 0.898 | 0.860 | 0.937 | 0.000 |  |  |  |  |  |  |  |
|  | Simple mode | 0.994 | 0.854 | 1.157 | 0.943 |  |  |  |  |  |  |  |
|  | Weighted mode | 0.883 | 0.834 | 0.934 | 0.000 |  |  |  |  |  |  |  |
|  | BWMR | 0.894 | 0.853 | 0.938 | 0.000 |  |  |  |  |  |  |  |
| Phosphatidylcholine (16:0_20:4) | MR Egger | 0.879 | 0.823 | 0.938 | 0.001 | 24.428 | 0.325 | 25.456 | 0.327 | 0.007 | 0.346 | 0.390 |
|  | Weighted median | 0.889 | 0.846 | 0.933 | 0.000 |  |  |  |  |  |  |  |
|  | IVW | 0.900 | 0.862 | 0.940 | 0.000 |  |  |  |  |  |  |  |
|  | Simple mode | 0.953 | 0.805 | 1.128 | 0.578 |  |  |  |  |  |  |  |
|  | Weighted mode | 0.888 | 0.844 | 0.934 | 0.000 |  |  |  |  |  |  |  |
|  | BWMR | 0.900 | 0.861 | 0.942 | 0.000 |  |  |  |  |  |  |  |
| Phosphatidylcholine (17:0_18:2) | MR Egger | 1.237 | 1.045 | 1.464 | 0.021 | 27.219 | 0.247 | 28.092 | 0.256 | -0.010 | 0.399 | 0.200 |
|  | Weighted median | 1.217 | 1.107 | 1.338 | 0.000 |  |  |  |  |  |  |  |
|  | IVW | 1.156 | 1.081 | 1.237 | 0.000 |  |  |  |  |  |  |  |
|  | Simple mode | 1.207 | 0.973 | 1.497 | 0.100 |  |  |  |  |  |  |  |
|  | Weighted mode | 1.284 | 1.149 | 1.434 | 0.000 |  |  |  |  |  |  |  |
|  | BWMR | 1.157 | 1.077 | 1.243 | 0.000 |  |  |  |  |  |  |  |
| Sphingomyelin (d36:1) | MR Egger | 0.946 | 0.814 | 1.099 | 0.473 | 42.418 | 0.126 | 42.466 | 0.151 | -0.002 | 0.847 | 0.168 |
|  | Weighted median | 0.951 | 0.875 | 1.032 | 0.228 |  |  |  |  |  |  |  |
|  | IVW | 0.933 | 0.877 | 0.993 | 0.030 |  |  |  |  |  |  |  |
|  | Simple mode | 0.960 | 0.808 | 1.141 | 0.647 |  |  |  |  |  |  |  |
|  | Weighted mode | 0.929 | 0.822 | 1.049 | 0.241 |  |  |  |  |  |  |  |
|  | BWMR | 0.932 | 0.872 | 0.996 | 0.038 |  |  |  |  |  |  |  |
| Sphingomyelin (d38:1) | MR Egger | 0.892 | 0.814 | 0.976 | 0.018 | 26.245 | 0.884 | 26.594 | 0.897 | 0.004 | 0.559 | 0.912 |
|  | Weighted median | 0.903 | 0.841 | 0.970 | 0.005 |  |  |  |  |  |  |  |
|  | IVW | 0.913 | 0.871 | 0.957 | 0.000 |  |  |  |  |  |  |  |
|  | Simple mode | 0.908 | 0.805 | 1.024 | 0.125 |  |  |  |  |  |  |  |
|  | Weighted mode | 0.902 | 0.833 | 0.977 | 0.016 |  |  |  |  |  |  |  |
|  | BWMR | 0.911 | 0.868 | 0.957 | 0.000 |  |  |  |  |  |  |  |
| Sphingomyelin (d42:2) | MR Egger | 0.970 | 0.876 | 1.074 | 0.566 | 35.909 | 0.473 | 36.631 | 0.486 | -0.006 | 0.401 | 0.460 |
|  | Weighted median | 0.899 | 0.831 | 0.972 | 0.007 |  |  |  |  |  |  |  |
|  | IVW | 0.934 | 0.886 | 0.986 | 0.013 |  |  |  |  |  |  |  |
|  | Simple mode | 0.890 | 0.763 | 1.038 | 0.145 |  |  |  |  |  |  |  |
|  | Weighted mode | 0.878 | 0.766 | 1.006 | 0.069 |  |  |  |  |  |  |  |
|  | BWMR | 0.930 | 0.878 | 0.985 | 0.013 |  |  |  |  |  |  |  |
| **UC** |  |  |  |  |  |  |  |  |  |  |  |  |
| Phosphatidylcholine (20:4_0:0) | MR Egger | 0.865 | 0.793 | 0.943 | 0.003 | 26.781 | 0.367 | 27.216 | 0.398 | 0.006 | 0.530 | 0.420 |
|  | Weighted median | 0.872 | 0.815 | 0.933 | 0.000 |  |  |  |  |  |  |  |
|  | IVW | 0.884 | 0.838 | 0.932 | 0.000 |  |  |  |  |  |  |  |
|  | Simple mode | 0.923 | 0.758 | 1.124 | 0.434 |  |  |  |  |  |  |  |
|  | Weighted mode | 0.868 | 0.810 | 0.930 | 0.000 |  |  |  |  |  |  |  |
|  | BWMR | 0.879 | 0.833 | 0.927 | 0.000 |  |  |  |  |  |  |  |
| Phosphatidylethanolamine (18:2_0:0) | MR Egger | 1.210 | 1.063 | 1.378 | 0.008 | 28.743 | 0.275 | 30.724 | 0.239 | -0.013 | 0.201 | 0.241 |
|  | Weighted median | 1.198 | 1.091 | 1.314 | 0.000 |  |  |  |  |  |  |  |
|  | IVW | 1.125 | 1.048 | 1.208 | 0.001 |  |  |  |  |  |  |  |
|  | Simple mode | 1.147 | 0.940 | 1.401 | 0.189 |  |  |  |  |  |  |  |
|  | Weighted mode | 1.187 | 1.080 | 1.305 | 0.002 |  |  |  |  |  |  |  |
|  | BWMR | 1.124 | 1.038 | 1.217 | 0.004 |  |  |  |  |  |  |  |
| Phosphatidylcholine (14:0_16:0) | MR Egger | 0.903 | 0.717 | 1.139 | 0.402 | 13.941 | 0.671 | 13.951 | 0.732 | -0.002 | 0.923 | 0.739 |
|  | Weighted median | 0.949 | 0.832 | 1.082 | 0.432 |  |  |  |  |  |  |  |
|  | IVW | 0.894 | 0.813 | 0.983 | 0.021 |  |  |  |  |  |  |  |
|  | Simple mode | 0.974 | 0.763 | 1.243 | 0.833 |  |  |  |  |  |  |  |
|  | Weighted mode | 0.983 | 0.791 | 1.221 | 0.875 |  |  |  |  |  |  |  |
|  | BWMR | 0.891 | 0.805 | 0.986 | 0.026 |  |  |  |  |  |  |  |
| Phosphatidylcholine (16:0_20:2) | MR Egger | 1.254 | 1.070 | 1.471 | 0.010 | 30.143 | 0.219 | 32.365 | 0.181 | -0.016 | 0.187 | 0.112 |
|  | Weighted median | 1.235 | 1.105 | 1.381 | 0.000 |  |  |  |  |  |  |  |
|  | IVW | 1.140 | 1.052 | 1.236 | 0.001 |  |  |  |  |  |  |  |
|  | Simple mode | 1.295 | 0.991 | 1.693 | 0.070 |  |  |  |  |  |  |  |
|  | Weighted mode | 1.332 | 1.167 | 1.520 | 0.000 |  |  |  |  |  |  |  |
|  | BWMR | 1.121 | 1.030 | 1.220 | 0.008 |  |  |  |  |  |  |  |
| Phosphatidylcholine (16:0_20:4) | MR Egger | 0.850 | 0.788 | 0.918 | 0.000 | 17.166 | 0.754 | 20.876 | 0.589 | 0.017 | 0.067 | 0.526 |
|  | Weighted median | 0.876 | 0.822 | 0.935 | 0.000 |  |  |  |  |  |  |  |
|  | IVW | 0.899 | 0.855 | 0.946 | 0.000 |  |  |  |  |  |  |  |
|  | Simple mode | 0.917 | 0.733 | 1.148 | 0.459 |  |  |  |  |  |  |  |
|  | Weighted mode | 0.876 | 0.823 | 0.931 | 0.000 |  |  |  |  |  |  |  |
|  | BWMR | 0.899 | 0.854 | 0.945 | 0.000 |  |  |  |  |  |  |  |
| Phosphatidylcholine (16:1_18:1) | MR Egger | 1.181 | 0.937 | 1.489 | 0.170 | 36.624 | 0.081 | 36.856 | 0.098 | -0.006 | 0.688 | 0.090 |
|  | Weighted median | 1.160 | 1.026 | 1.312 | 0.018 |  |  |  |  |  |  |  |
|  | IVW | 1.130 | 1.032 | 1.238 | 0.008 |  |  |  |  |  |  |  |
|  | Simple mode | 1.217 | 0.915 | 1.619 | 0.188 |  |  |  |  |  |  |  |
|  | Weighted mode | 1.341 | 1.055 | 1.705 | 0.024 |  |  |  |  |  |  |  |
|  | BWMR | 1.125 | 1.023 | 1.238 | 0.015 |  |  |  |  |  |  |  |
| Phosphatidylcholine (17:0_18:2) | MR Egger | 1.298 | 1.036 | 1.626 | 0.033 | 32.175 | 0.097 | 33.552 | 0.093 | -0.016 | 0.331 | 0.073 |
|  | Weighted median | 1.259 | 1.118 | 1.418 | 0.000 |  |  |  |  |  |  |  |
|  | IVW | 1.169 | 1.068 | 1.280 | 0.001 |  |  |  |  |  |  |  |
|  | Simple mode | 1.287 | 0.958 | 1.728 | 0.107 |  |  |  |  |  |  |  |
|  | Weighted mode | 1.337 | 1.158 | 1.544 | 0.001 |  |  |  |  |  |  |  |
|  | BWMR | 1.169 | 1.065 | 1.282 | 0.001 |  |  |  |  |  |  |  |
| Phosphatidylcholine (18:0_20:4) | MR Egger | 0.872 | 0.808 | 0.941 | 0.002 | 34.757 | 0.093 | 36.564 | 0.082 | 0.009 | 0.265 | 0.142 |
|  | Weighted median | 0.886 | 0.840 | 0.935 | 0.000 |  |  |  |  |  |  |  |
|  | IVW | 0.901 | 0.854 | 0.950 | 0.000 |  |  |  |  |  |  |  |
|  | Simple mode | 0.854 | 0.726 | 1.006 | 0.070 |  |  |  |  |  |  |  |
|  | Weighted mode | 0.886 | 0.838 | 0.936 | 0.000 |  |  |  |  |  |  |  |
|  | BWMR | 0.902 | 0.854 | 0.953 | 0.000 |  |  |  |  |  |  |  |
| Phosphatidylcholine (18:1_20:4) | MR Egger | 0.866 | 0.783 | 0.957 | 0.009 | 35.581 | 0.100 | 37.798 | 0.081 | 0.012 | 0.214 | 0.101 |
|  | Weighted median | 0.859 | 0.801 | 0.923 | 0.000 |  |  |  |  |  |  |  |
|  | IVW | 0.910 | 0.854 | 0.971 | 0.004 |  |  |  |  |  |  |  |
|  | Simple mode | 0.946 | 0.749 | 1.194 | 0.643 |  |  |  |  |  |  |  |
|  | Weighted mode | 0.867 | 0.805 | 0.935 | 0.001 |  |  |  |  |  |  |  |
|  | BWMR | 0.910 | 0.854 | 0.969 | 0.003 |  |  |  |  |  |  |  |
| Phosphatidylcholine (O-16:1_20:4) | MR Egger | 0.823 | 0.723 | 0.937 | 0.008 | 26.286 | 0.196 | 30.877 | 0.099 | 0.022 | 0.069 | 0.069 |
|  | Weighted median | 0.857 | 0.779 | 0.942 | 0.001 |  |  |  |  |  |  |  |
|  | IVW | 0.917 | 0.852 | 0.986 | 0.019 |  |  |  |  |  |  |  |
|  | Simple mode | 0.993 | 0.766 | 1.286 | 0.956 |  |  |  |  |  |  |  |
|  | Weighted mode | 0.827 | 0.754 | 0.906 | 0.001 |  |  |  |  |  |  |  |
|  | BWMR | 0.913 | 0.848 | 0.983 | 0.016 |  |  |  |  |  |  |  |
| Phosphatidylcholine (O-18:1_18:2) | MR Egger | 1.363 | 1.138 | 1.634 | 0.003 | 21.423 | 0.555 | 25.306 | 0.389 | -0.024 | 0.061 | 0.270 |
|  | Weighted median | 1.242 | 1.104 | 1.397 | 0.000 |  |  |  |  |  |  |  |
|  | IVW | 1.158 | 1.067 | 1.256 | 0.000 |  |  |  |  |  |  |  |
|  | Simple mode | 1.284 | 0.988 | 1.667 | 0.073 |  |  |  |  |  |  |  |
|  | Weighted mode | 1.345 | 1.149 | 1.573 | 0.001 |  |  |  |  |  |  |  |
|  | BWMR | 1.163 | 1.061 | 1.275 | 0.001 |  |  |  |  |  |  |  |
| Phosphatidylethanolamine (18:0_18:2) | MR Egger | 1.102 | 0.966 | 1.256 | 0.159 | 23.450 | 0.075 | 24.370 | 0.082 | -0.018 | 0.455 | 0.287 |
|  | Weighted median | 1.083 | 0.984 | 1.192 | 0.102 |  |  |  |  |  |  |  |
|  | IVW | 1.091 | 1.022 | 1.166 | 0.009 |  |  |  |  |  |  |  |
|  | Simple mode | 1.031 | 0.856 | 1.243 | 0.749 |  |  |  |  |  |  |  |
|  | Weighted mode | 1.097 | 0.986 | 1.220 | 0.101 |  |  |  |  |  |  |  |
|  | BWMR | 1.084 | 1.013 | 1.160 | 0.019 |  |  |  |  |  |  |  |
| Sphingomyelin (d36:1) | MR Egger | 0.986 | 0.829 | 1.174 | 0.876 | 37.565 | 0.268 | 38.777 | 0.263 | -0.012 | 0.310 | 0.298 |
|  | Weighted median | 0.873 | 0.786 | 0.969 | 0.011 |  |  |  |  |  |  |  |
|  | IVW | 0.908 | 0.843 | 0.977 | 0.010 |  |  |  |  |  |  |  |
|  | Simple mode | 0.882 | 0.730 | 1.066 | 0.202 |  |  |  |  |  |  |  |
|  | Weighted mode | 0.885 | 0.769 | 1.019 | 0.098 |  |  |  |  |  |  |  |
|  | BWMR | 0.907 | 0.837 | 0.983 | 0.018 |  |  |  |  |  |  |  |
| Sphingomyelin (d38:1) | MR Egger | 0.944 | 0.844 | 1.055 | 0.315 | 31.896 | 0.664 | 32.513 | 0.679 | -0.007 | 0.437 | 0.693 |
|  | Weighted median | 0.916 | 0.841 | 0.997 | 0.043 |  |  |  |  |  |  |  |
|  | IVW | 0.908 | 0.857 | 0.962 | 0.001 |  |  |  |  |  |  |  |
|  | Simple mode | 0.853 | 0.736 | 0.988 | 0.041 |  |  |  |  |  |  |  |
|  | Weighted mode | 0.928 | 0.832 | 1.035 | 0.187 |  |  |  |  |  |  |  |
|  | BWMR | 0.906 | 0.854 | 0.962 | 0.001 |  |  |  |  |  |  |  |
| Sphingomyelin (d42:2) | MR Egger | 0.954 | 0.842 | 1.082 | 0.469 | 30.838 | 0.712 | 31.113 | 0.741 | -0.004 | 0.603 | 0.718 |
|  | Weighted median | 0.884 | 0.805 | 0.972 | 0.010 |  |  |  |  |  |  |  |
|  | IVW | 0.927 | 0.869 | 0.990 | 0.024 |  |  |  |  |  |  |  |
|  | Simple mode | 0.841 | 0.700 | 1.011 | 0.073 |  |  |  |  |  |  |  |
|  | Weighted mode | 0.850 | 0.735 | 0.984 | 0.036 |  |  |  |  |  |  |  |
|  | BWMR | 0.926 | 0.865 | 0.991 | 0.026 |  |  |  |  |  |  |  |
| **CD** |  |  |  |  |  |  |  |  |  |  |  |  |
| Sterol ester (27:1/20:4) | MR Egger | 0.878 | 0.773 | 0.998 | 0.056 | 37.509 | 0.134 | 37.868 | 0.153 | 0.008 | 0.602 | 0.562 |
|  | Weighted median | 0.886 | 0.807 | 0.972 | 0.011 |  |  |  |  |  |  |  |
|  | IVW | 0.901 | 0.827 | 0.981 | 0.017 |  |  |  |  |  |  |  |
|  | Simple mode | 0.838 | 0.649 | 1.081 | 0.184 |  |  |  |  |  |  |  |
|  | Weighted mode | 0.881 | 0.802 | 0.968 | 0.013 |  |  |  |  |  |  |  |
|  | BWMR | 0.903 | 0.824 | 0.989 | 0.028 |  |  |  |  |  |  |  |
| Phosphatidylcholine (20:4_0:0) | MR Egger | 0.835 | 0.724 | 0.962 | 0.020 | 22.100 | 0.630 | 24.164 | 0.567 | 0.020 | 0.163 | 0.541 |
|  | Weighted median | 0.872 | 0.781 | 0.973 | 0.015 |  |  |  |  |  |  |  |
|  | IVW | 0.905 | 0.828 | 0.989 | 0.028 |  |  |  |  |  |  |  |
|  | Simple mode | 0.918 | 0.688 | 1.224 | 0.563 |  |  |  |  |  |  |  |
|  | Weighted mode | 0.865 | 0.771 | 0.971 | 0.021 |  |  |  |  |  |  |  |
|  | BWMR | 0.897 | 0.810 | 0.993 | 0.036 |  |  |  |  |  |  |  |
| Phosphatidylcholine (16:0_20:4) | MR Egger | 0.915 | 0.803 | 1.043 | 0.196 | 21.650 | 0.481 | 22.494 | 0.491 | -0.014 | 0.368 | 0.632 |
|  | Weighted median | 0.877 | 0.792 | 0.971 | 0.012 |  |  |  |  |  |  |  |
|  | IVW | 0.874 | 0.802 | 0.953 | 0.002 |  |  |  |  |  |  |  |
|  | Simple mode | 0.856 | 0.623 | 1.177 | 0.350 |  |  |  |  |  |  |  |
|  | Weighted mode | 0.877 | 0.800 | 0.963 | 0.011 |  |  |  |  |  |  |  |
|  | BWMR | 0.872 | 0.801 | 0.950 | 0.002 |  |  |  |  |  |  |  |
| Phosphatidylcholine (17:0_18:1) | MR Egger | 0.739 | 0.521 | 1.048 | 0.107 | 7.660 | 0.983 | 8.273 | 0.984 | 0.019 | 0.444 | 0.982 |
|  | Weighted median | 0.854 | 0.691 | 1.056 | 0.146 |  |  |  |  |  |  |  |
|  | IVW | 0.836 | 0.708 | 0.986 | 0.034 |  |  |  |  |  |  |  |
|  | Simple mode | 0.857 | 0.583 | 1.259 | 0.440 |  |  |  |  |  |  |  |
|  | Weighted mode | 0.862 | 0.616 | 1.207 | 0.399 |  |  |  |  |  |  |  |
|  | BWMR | 0.835 | 0.701 | 0.994 | 0.043 |  |  |  |  |  |  |  |
| Phosphatidylcholine (17:0_18:2) | MR Egger | 1.414 | 1.001 | 1.998 | 0.062 | 26.040 | 0.299 | 27.718 | 0.272 | -0.029 | 0.236 | 0.239 |
|  | Weighted median | 1.195 | 0.982 | 1.454 | 0.076 |  |  |  |  |  |  |  |
|  | IVW | 1.162 | 1.010 | 1.337 | 0.036 |  |  |  |  |  |  |  |
|  | Simple mode | 1.024 | 0.692 | 1.515 | 0.908 |  |  |  |  |  |  |  |
|  | Weighted mode | 1.314 | 1.042 | 1.657 | 0.030 |  |  |  |  |  |  |  |
|  | BWMR | 1.167 | 1.006 | 1.355 | 0.041 |  |  |  |  |  |  |  |
| Phosphatidylcholine (18:1_20:4) | MR Egger | 0.829 | 0.700 | 0.981 | 0.038 | 34.610 | 0.120 | 34.928 | 0.141 | 0.007 | 0.629 | 0.214 |
|  | Weighted median | 0.856 | 0.761 | 0.963 | 0.010 |  |  |  |  |  |  |  |
|  | IVW | 0.856 | 0.771 | 0.951 | 0.004 |  |  |  |  |  |  |  |
|  | Simple mode | 0.917 | 0.680 | 1.235 | 0.572 |  |  |  |  |  |  |  |
|  | Weighted mode | 0.859 | 0.766 | 0.962 | 0.014 |  |  |  |  |  |  |  |
|  | BWMR | 0.850 | 0.773 | 0.935 | 0.001 |  |  |  |  |  |  |  |
| Phosphatidylethanolamine (O-18:1_20:4) | MR Egger | 0.778 | 0.584 | 1.036 | 0.098 | 24.555 | 0.544 | 24.726 | 0.590 | 0.008 | 0.683 | 0.587 |
|  | Weighted median | 0.750 | 0.613 | 0.919 | 0.005 |  |  |  |  |  |  |  |
|  | IVW | 0.821 | 0.721 | 0.934 | 0.003 |  |  |  |  |  |  |  |
|  | Simple mode | 0.790 | 0.533 | 1.172 | 0.251 |  |  |  |  |  |  |  |
|  | Weighted mode | 0.749 | 0.583 | 0.964 | 0.033 |  |  |  |  |  |  |  |
|  | BWMR | 0.812 | 0.711 | 0.927 | 0.002 |  |  |  |  |  |  |  |
| Abbreviations: IVW: inverse variance weighted; BWMR: Bayesian Weighted Mendelian Randomization; β:beta; OR: odds ratio; IBD: inflammatory bowel disease; UC: ulcerative colitis; CD: Crohn's disease. | | | | | | | | | | | |  |

| **Supplementary Table S9. MR analysis results of associations between lipidome and IBD after further validation analysis** | | | | | | | | | | | | |
| --- | --- | --- | --- | --- | --- | --- | --- | --- | --- | --- | --- | --- |
| **Plasma lipidome** | **Method** | **OR** | **OR_lci95** | **OR_uci95** | **P** | **Heterogeneity** | | | | **Pleiotropy** | | |
|  |  |  |  |  |  | **MR Egger** | | **IVW** | | **MR-Egger intercep** | | **MR-PRESO** |
|  |  |  |  |  |  | **Q** | **P** | **Q** | **P** | **intercept** | **P** | **P** |
| **IBD** |  |  |  |  |  |  |  |  |  |  |  |  |
| Sterol ester (27:1/22:6) | MR Egger | 0.816 | 0.669 | 0.995 | 0.065 | 4.815 | 0.439 | 4.894 | 0.557 | 0.007 | 0.790 | 0.187 |
|  | Weighted median | 0.895 | 0.811 | 0.988 | 0.028 |  |  |  |  |  |  |  |
|  | IVW | 0.897 | 0.833 | 0.965 | 0.003 |  |  |  |  |  |  |  |
|  | Simple mode | 0.916 | 0.777 | 1.079 | 0.310 |  |  |  |  |  |  |  |
|  | Weighted mode | 0.895 | 0.783 | 1.022 | 0.124 |  |  |  |  |  |  |  |
|  | BWMR | 0.915 | 0.861 | 0.992 | 0.023 |  |  |  |  |  |  |  |
| Phosphatidylcholine (20:4_0:0) | MR Egger | 0.808 | 0.484 | 1.348 | 0.451 | 8.989 | 0.110 | 9.111 | 0.167 | 0.010 | 0.805 | 0.643 |
|  | Weighted median | 0.907 | 0.779 | 1.056 | 0.208 |  |  |  |  |  |  |  |
|  | IVW | 0.863 | 0.756 | 0.983 | 0.027 |  |  |  |  |  |  |  |
|  | Simple mode | 0.928 | 0.721 | 1.195 | 0.584 |  |  |  |  |  |  |  |
|  | Weighted mode | 0.922 | 0.740 | 1.148 | 0.493 |  |  |  |  |  |  |  |
|  | BWMR | 0.854 | 0.743 | 0.981 | 0.026 |  |  |  |  |  |  |  |
| Phosphatidylcholine (16:0_20:4) | MR Egger | 0.921 | 0.854 | 0.992 | 0.083 | 5.076 | 0.407 | 7.502 | 0.277 | 0.013 | 0.183 | 0.892 |
|  | Weighted median | 0.887 | 0.844 | 0.932 | 0.000 |  |  |  |  |  |  |  |
|  | IVW | 0.879 | 0.835 | 0.926 | 0.000 |  |  |  |  |  |  |  |
|  | Simple mode | 0.813 | 0.687 | 0.962 | 0.053 |  |  |  |  |  |  |  |
|  | Weighted mode | 0.886 | 0.844 | 0.932 | 0.003 |  |  |  |  |  |  |  |
|  | BWMR | 0.881 | 0.842 | 0.922 | 0.000 |  |  |  |  |  |  |  |
| Phosphatidylcholine (17:0_18:2) | MR Egger | 1.395 | 1.037 | 1.876 | 0.070 | 1.577 | 0.503 | 1.261 | 0.228 | -0.029 | 0.233 | 0.321 |
|  | Weighted median | 1.233 | 1.095 | 1.388 | 0.001 |  |  |  |  |  |  |  |
|  | IVW | 1.162 | 1.022 | 1.321 | 0.022 |  |  |  |  |  |  |  |
|  | Simple mode | 1.212 | 0.897 | 1.639 | 0.251 |  |  |  |  |  |  |  |
|  | Weighted mode | 1.289 | 1.146 | 1.451 | 0.004 |  |  |  |  |  |  |  |
|  | BWMR | 1.142 | 1.095 | 1.310 | 0.006 |  |  |  |  |  |  |  |
| Sphingomyelin (d36:1) | MR Egger | 0.939 | 0.700 | 1.258 | 0.690 | 6.241 | 0.937 | 7.250 | 0.925 | 0.016 | 0.333 | 0.456 |
|  | Weighted median | 1.026 | 0.882 | 1.194 | 0.736 |  |  |  |  |  |  |  |
|  | IVW | 0.975 | 0.865 | 1.100 | 0.684 |  |  |  |  |  |  |  |
|  | Simple mode | 1.017 | 0.829 | 1.248 | 0.878 |  |  |  |  |  |  |  |
|  | Weighted mode | 1.017 | 0.838 | 1.234 | 0.872 |  |  |  |  |  |  |  |
|  | BWMR | 0.914 | 0.834 | 1.002 | 0.056 |  |  |  |  |  |  |  |
| Sphingomyelin (d38:1) | MR Egger | 0.899 | 0.798 | 1.012 | 0.104 | 4.373 | 0.976 | 4.373 | 0.987 | 0.000 | 0.977 | 0.732 |
|  | Weighted median | 0.903 | 0.834 | 0.977 | 0.012 |  |  |  |  |  |  |  |
|  | IVW | 0.900 | 0.849 | 0.955 | 0.000 |  |  |  |  |  |  |  |
|  | Simple mode | 0.906 | 0.802 | 1.022 | 0.133 |  |  |  |  |  |  |  |
|  | Weighted mode | 0.905 | 0.827 | 0.990 | 0.048 |  |  |  |  |  |  |  |
|  | BWMR | 0.900 | 0.848 | 0.955 | 0.001 |  |  |  |  |  |  |  |
| Sphingomyelin (d42:2) | MR Egger | 0.926 | 0.768 | 1.117 | 0.438 | 5.065 | 0.368 | 8.069 | 0.543 | -0.001 | 0.968 | 0.055 |
|  | Weighted median | 0.923 | 0.840 | 1.014 | 0.096 |  |  |  |  |  |  |  |
|  | IVW | 0.885 | 0.798 | 0.982 | 0.022 |  |  |  |  |  |  |  |
|  | Simple mode | 0.890 | 0.755 | 1.050 | 0.190 |  |  |  |  |  |  |  |
|  | Weighted mode | 0.881 | 0.768 | 1.011 | 0.094 |  |  |  |  |  |  |  |
|  | BWMR | 0.896 | 0.831 | 0.965 | 0.004 |  |  |  |  |  |  |  |
| **UC** |  |  |  |  |  |  |  |  |  |  |  |  |
| Phosphatidylcholine (20:4_0:0) | MR Egger | 0.890 | 0.478 | 1.656 | 0.727 | 8.744 | 0.120 | 8.777 | 0.187 | -0.006 | 0.896 | 0.923 |
|  | Weighted median | 0.895 | 0.740 | 1.082 | 0.252 |  |  |  |  |  |  |  |
|  | IVW | 0.853 | 0.728 | 0.992 | 0.033 |  |  |  |  |  |  |  |
|  | Simple mode | 0.945 | 0.704 | 1.269 | 0.720 |  |  |  |  |  |  |  |
|  | Weighted mode | 0.919 | 0.719 | 1.175 | 0.527 |  |  |  |  |  |  |  |
|  | BWMR | 0.849 | 0.721 | 0.999 | 0.042 |  |  |  |  |  |  |  |
| Phosphatidylethanolamine (18:2_0:0) | MR Egger | 1.236 | 1.043 | 1.465 | 0.044 | 8.451 | 0.295 | 10.055 | 0.261 | -0.020 | 0.287 | 0.278 |
|  | Weighted median | 1.174 | 1.070 | 1.289 | 0.001 |  |  |  |  |  |  |  |
|  | IVW | 1.136 | 1.036 | 1.245 | 0.007 |  |  |  |  |  |  |  |
|  | Simple mode | 1.135 | 0.926 | 1.392 | 0.258 |  |  |  |  |  |  |  |
|  | Weighted mode | 1.193 | 1.080 | 1.319 | 0.009 |  |  |  |  |  |  |  |
|  | BWMR | 1.123 | 1.006 | 1.253 | 0.039 |  |  |  |  |  |  |  |
| Phosphatidylcholine (16:0_20:2) | MR Egger | 1.386 | 0.992 | 1.938 | 0.092 | 2.191 | 0.639 | 2.062 | 0.495 | -0.030 | 0.277 | 0.511 |
|  | Weighted median | 1.161 | 0.994 | 1.358 | 0.060 |  |  |  |  |  |  |  |
|  | IVW | 1.293 | 1.127 | 1.484 | 0.000 |  |  |  |  |  |  |  |
|  | Simple mode | 1.306 | 0.925 | 1.843 | 0.164 |  |  |  |  |  |  |  |
|  | Weighted mode | 1.337 | 1.168 | 1.530 | 0.002 |  |  |  |  |  |  |  |
|  | BWMR | 1.337 | 1.168 | 1.530 | 0.002 |  |  |  |  |  |  |  |
| Phosphatidylcholine (16:0_20:4) | MR Egger | 0.895 | 0.817 | 0.980 | 0.062 | 4.866 | 0.432 | 5.553 | 0.475 | -0.013 | 0.445 | 0.689 |
|  | Weighted median | 0.872 | 0.822 | 0.926 | 0.000 |  |  |  |  |  |  |  |
|  | IVW | 0.868 | 0.820 | 0.919 | 0.000 |  |  |  |  |  |  |  |
|  | Simple mode | 0.884 | 0.755 | 1.037 | 0.180 |  |  |  |  |  |  |  |
|  | Weighted mode | 0.874 | 0.825 | 0.925 | 0.004 |  |  |  |  |  |  |  |
|  | BWMR | 0.870 | 0.823 | 0.919 | 0.000 |  |  |  |  |  |  |  |
| Phosphatidylcholine (16:1_18:1) | MR Egger | 1.228 | 0.658 | 2.290 | 0.539 | 2.158 | 0.386 | 2.235 | 0.450 | -0.006 | 0.880 | 0.142 |
|  | Weighted median | 1.171 | 0.968 | 1.418 | 0.104 |  |  |  |  |  |  |  |
|  | IVW | 1.313 | 1.106 | 1.558 | 0.002 |  |  |  |  |  |  |  |
|  | Simple mode | 1.353 | 0.927 | 1.973 | 0.155 |  |  |  |  |  |  |  |
|  | Weighted mode | 1.397 | 1.152 | 1.693 | 0.009 |  |  |  |  |  |  |  |
|  | BWMR | 1.397 | 1.152 | 1.693 | 0.009 |  |  |  |  |  |  |  |
| Phosphatidylcholine (17:0_18:2) | MR Egger | 1.489 | 1.047 | 2.116 | 0.068 | 11.728 | 0.068 | 6.188 | 0.235 | -0.040 | 0.182 | 0.834 |
|  | Weighted median | 1.163 | 0.993 | 1.361 | 0.060 |  |  |  |  |  |  |  |
|  | IVW | 1.324 | 1.143 | 1.534 | 0.000 |  |  |  |  |  |  |  |
|  | Simple mode | 1.213 | 0.826 | 1.782 | 0.357 |  |  |  |  |  |  |  |
|  | Weighted mode | 1.328 | 1.156 | 1.525 | 0.005 |  |  |  |  |  |  |  |
|  | BWMR | 1.328 | 1.156 | 1.525 | 0.005 |  |  |  |  |  |  |  |
| Phosphatidylcholine (18:0_20:4) | MR Egger | 0.898 | 0.837 | 0.962 | 0.009 | 9.845 | 0.707 | 10.537 | 0.722 | -0.008 | 0.421 | 0.399 |
|  | Weighted median | 0.885 | 0.838 | 0.935 | 0.000 |  |  |  |  |  |  |  |
|  | IVW | 0.879 | 0.838 | 0.921 | 0.000 |  |  |  |  |  |  |  |
|  | Simple mode | 0.849 | 0.721 | 1.000 | 0.071 |  |  |  |  |  |  |  |
|  | Weighted mode | 0.885 | 0.837 | 0.935 | 0.001 |  |  |  |  |  |  |  |
|  | BWMR | 0.878 | 0.837 | 0.921 | 0.000 |  |  |  |  |  |  |  |
| Phosphatidylcholine (18:1_20:4) | MR Egger | 0.856 | 0.776 | 0.945 | 0.011 | 10.175 | 0.425 | 10.425 | 0.493 | 0.006 | 0.631 | 0.967 |
|  | Weighted median | 0.859 | 0.798 | 0.925 | 0.000 |  |  |  |  |  |  |  |
|  | IVW | 0.873 | 0.821 | 0.928 | 0.000 |  |  |  |  |  |  |  |
|  | Simple mode | 0.895 | 0.731 | 1.097 | 0.310 |  |  |  |  |  |  |  |
|  | Weighted mode | 0.864 | 0.804 | 0.929 | 0.002 |  |  |  |  |  |  |  |
|  | BWMR | 0.873 | 0.822 | 0.927 | 0.000 |  |  |  |  |  |  |  |
| Phosphatidylcholine (O-16:1_20:4) | MR Egger | 0.776 | 0.604 | 0.997 | 0.118 | 2.048 | 0.170 | 1.365 | 0.386 | 0.039 | 0.255 | 0.205 |
|  | Weighted median | 0.838 | 0.762 | 0.921 | 0.000 |  |  |  |  |  |  |  |
|  | IVW | 0.897 | 0.779 | 1.032 | 0.128 |  |  |  |  |  |  |  |
|  | Simple mode | 0.859 | 0.628 | 1.177 | 0.388 |  |  |  |  |  |  |  |
|  | Weighted mode | 0.826 | 0.748 | 0.912 | 0.013 |  |  |  |  |  |  |  |
|  | BWMR | 0.897 | 0.785 | 1.025 | 0.109 |  |  |  |  |  |  |  |
| Phosphatidylcholine (O-18:1_18:2) | MR Egger | 1.571 | 1.160 | 2.126 | 0.043 | 0.769 | 0.943 | 2.161 | 0.827 | -0.024 | 0.304 | 0.576 |
|  | Weighted median | 1.360 | 1.171 | 1.581 | 0.000 |  |  |  |  |  |  |  |
|  | IVW | 1.329 | 1.178 | 1.500 | 0.000 |  |  |  |  |  |  |  |
|  | Simple mode | 1.327 | 1.043 | 1.690 | 0.070 |  |  |  |  |  |  |  |
|  | Weighted mode | 1.386 | 1.193 | 1.611 | 0.008 |  |  |  |  |  |  |  |
|  | BWMR | 1.333 | 1.174 | 1.513 | 0.000 |  |  |  |  |  |  |  |
| Phosphatidylethanolamine (18:0_18:2) | MR Egger | 1.227 | 1.046 | 1.440 | 0.031 | 14.234 | 0.163 | 17.603 | 0.091 | -0.029 | 0.155 | 0.713 |
|  | Weighted median | 1.085 | 0.983 | 1.197 | 0.105 |  |  |  |  |  |  |  |
|  | IVW | 1.097 | 1.016 | 1.184 | 0.018 |  |  |  |  |  |  |  |
|  | Simple mode | 1.099 | 0.833 | 1.173 | 0.897 |  |  |  |  |  |  |  |
|  | Weighted mode | 1.052 | 0.912 | 1.214 | 0.501 |  |  |  |  |  |  |  |
|  | BWMR | 1.088 | 1.005 | 1.178 | 0.037 |  |  |  |  |  |  |  |
| Sphingomyelin (d36:1) | MR Egger | 0.912 | 0.714 | 1.163 | 0.470 | 6.075 | 0.943 | 6.173 | 0.962 | -0.006 | 0.758 | 0.088 |
|  | Weighted median | 0.870 | 0.768 | 0.984 | 0.027 |  |  |  |  |  |  |  |
|  | IVW | 0.879 | 0.803 | 0.962 | 0.005 |  |  |  |  |  |  |  |
|  | Simple mode | 0.862 | 0.708 | 1.049 | 0.161 |  |  |  |  |  |  |  |
|  | Weighted mode | 0.867 | 0.734 | 1.023 | 0.113 |  |  |  |  |  |  |  |
|  | BWMR | 0.877 | 0.800 | 0.962 | 0.005 |  |  |  |  |  |  |  |
| Sphingomyelin (d38:1) | MR Egger | 0.964 | 0.833 | 1.116 | 0.635 | 6.383 | 0.896 | 7.272 | 0.888 | -0.014 | 0.365 | 0.352 |
|  | Weighted median | 0.938 | 0.853 | 1.031 | 0.186 |  |  |  |  |  |  |  |
|  | IVW | 0.907 | 0.844 | 0.975 | 0.008 |  |  |  |  |  |  |  |
|  | Simple mode | 0.928 | 0.798 | 1.080 | 0.354 |  |  |  |  |  |  |  |
|  | Weighted mode | 0.943 | 0.833 | 1.068 | 0.373 |  |  |  |  |  |  |  |
|  | BWMR | 0.907 | 0.842 | 0.976 | 0.009 |  |  |  |  |  |  |  |
| Sphingomyelin (d42:2) | MR Egger | 0.955 | 0.783 | 1.165 | 0.659 | 16.317 | 0.177 | 16.883 | 0.205 | -0.012 | 0.531 | 0.498 |
|  | Weighted median | 0.903 | 0.816 | 1.000 | 0.050 |  |  |  |  |  |  |  |
|  | IVW | 0.870 | 0.767 | 0.986 | 0.029 |  |  |  |  |  |  |  |
|  | Simple mode | 0.858 | 0.703 | 1.048 | 0.157 |  |  |  |  |  |  |  |
|  | Weighted mode | 0.860 | 0.734 | 1.008 | 0.086 |  |  |  |  |  |  |  |
|  | BWMR | 0.902 | 0.812 | 0.990 | 0.025 |  |  |  |  |  |  |  |
| **CD** |  |  |  |  |  |  |  |  |  |  |  |  |
| Sterol ester (27:1/20:4) | MR Egger | 0.967 | 0.838 | 1.116 | 0.655 | 5.140 | 0.972 | 8.687 | 0.851 | -0.047 | 0.082 | 0.621 |
|  | Weighted median | 0.886 | 0.807 | 0.972 | 0.011 |  |  |  |  |  |  |  |
|  | IVW | 0.864 | 0.796 | 0.938 | 0.000 |  |  |  |  |  |  |  |
|  | Simple mode | 0.750 | 0.578 | 0.974 | 0.049 |  |  |  |  |  |  |  |
|  | Weighted mode | 0.884 | 0.802 | 0.975 | 0.027 |  |  |  |  |  |  |  |
|  | BWMR | 0.863 | 0.795 | 0.938 | 0.000 |  |  |  |  |  |  |  |
| Phosphatidylcholine (20:4_0:0) | MR Egger | 0.890 | 0.402 | 1.975 | 0.787 | 3.823 | 0.575 | 3.859 | 0.696 | -0.011 | 0.857 | 0.779 |
|  | Weighted median | 0.797 | 0.598 | 1.063 | 0.123 |  |  |  |  |  |  |  |
|  | IVW | 0.877 | 0.795 | 0.966 | 0.008 |  |  |  |  |  |  |  |
|  | Simple mode | 0.715 | 0.447 | 1.144 | 0.211 |  |  |  |  |  |  |  |
|  | Weighted mode | 0.745 | 0.486 | 1.140 | 0.224 |  |  |  |  |  |  |  |
|  | BWMR | 0.822 | 0.654 | 0.934 | 0.009 |  |  |  |  |  |  |  |
| Phosphatidylcholine (16:0_20:4) | MR Egger | 0.921 | 0.768 | 1.103 | 0.411 | 6.734 | 0.241 | 7.501 | 0.277 | -0.023 | 0.485 | 0.934 |
|  | Weighted median | 0.827 | 0.662 | 1.032 | 0.093 |  |  |  |  |  |  |  |
|  | IVW | 0.872 | 0.782 | 0.972 | 0.013 |  |  |  |  |  |  |  |
|  | Simple mode | 0.856 | 0.644 | 1.136 | 0.322 |  |  |  |  |  |  |  |
|  | Weighted mode | 0.875 | 0.784 | 0.978 | 0.057 |  |  |  |  |  |  |  |
|  | BWMR | 0.872 | 0.793 | 0.958 | 0.004 |  |  |  |  |  |  |  |
| Phosphatidylcholine (17:0_18:1) | MR Egger | 1.296 | 0.182 | 9.229 | 0.812 | 0.392 | 0.942 | 0.479 | 0.975 | -0.035 | 0.787 | 0.103 |
|  | Weighted median | 0.943 | 0.657 | 1.355 | 0.752 |  |  |  |  |  |  |  |
|  | IVW | 0.967 | 0.717 | 1.305 | 0.827 |  |  |  |  |  |  |  |
|  | Simple mode | 0.905 | 0.580 | 1.413 | 0.683 |  |  |  |  |  |  |  |
|  | Weighted mode | 0.900 | 0.582 | 1.391 | 0.659 |  |  |  |  |  |  |  |
|  | BWMR | 0.966 | 0.710 | 1.314 | 0.826 |  |  |  |  |  |  |  |
| Phosphatidylcholine (17:0_18:2) | MR Egger | 1.596 | 0.865 | 2.943 | 0.185 | 12.197 | 0.058 | 11.323 | 0.066 | -0.047 | 0.346 | 0.267 |
|  | Weighted median | 1.257 | 1.011 | 1.562 | 0.040 |  |  |  |  |  |  |  |
|  | IVW | 1.193 | 0.926 | 1.535 | 0.172 |  |  |  |  |  |  |  |
|  | Simple mode | 1.001 | 0.607 | 1.648 | 0.998 |  |  |  |  |  |  |  |
|  | Weighted mode | 1.328 | 1.050 | 1.679 | 0.050 |  |  |  |  |  |  |  |
|  | BWMR | 1.188 | 0.909 | 1.553 | 0.207 |  |  |  |  |  |  |  |
| Phosphatidylcholine (18:1_20:4) | MR Egger | 0.855 | 0.691 | 1.057 | 0.179 | 16.299 | 0.091 | 16.326 | 0.129 | -0.003 | 0.901 | 0.445 |
|  | Weighted median | 0.853 | 0.755 | 0.965 | 0.011 |  |  |  |  |  |  |  |
|  | IVW | 0.846 | 0.745 | 0.961 | 0.010 |  |  |  |  |  |  |  |
|  | Simple mode | 0.877 | 0.645 | 1.191 | 0.418 |  |  |  |  |  |  |  |
|  | Weighted mode | 0.852 | 0.752 | 0.966 | 0.029 |  |  |  |  |  |  |  |
|  | BWMR | 0.846 | 0.756 | 0.947 | 0.004 |  |  |  |  |  |  |  |
| Phosphatidylethanolamine (O-18:1_20:4) | MR Egger | 0.737 | 0.455 | 1.192 | 0.260 | 7.866 | 0.248 | 8.032 | 0.330 | 0.014 | 0.734 | 0.588 |
|  | Weighted median | 0.742 | 0.589 | 0.936 | 0.012 |  |  |  |  |  |  |  |
|  | IVW | 0.797 | 0.655 | 0.970 | 0.024 |  |  |  |  |  |  |  |
|  | Simple mode | 0.745 | 0.482 | 1.153 | 0.228 |  |  |  |  |  |  |  |
|  | Weighted mode | 0.732 | 0.571 | 0.937 | 0.043 |  |  |  |  |  |  |  |
|  | BWMR | 0.791 | 0.655 | 0.957 | 0.016 |  |  |  |  |  |  |  |
| Abbreviations: IVW: inverse variance weighted; BWMR: Bayesian Weighted Mendelian Randomization; β:beta; OR: odds ratio. | | | | | | | | | | | | |

| **Supplementary Table S10. Association of genome-wide SNPs for IBD with lipidome in reverse causal analysis.** | | | | | | | |
| --- | --- | --- | --- | --- | --- | --- | --- |
| **Plasma lipidome** | **SNP** | **Beta outcome** | **EAF outcome** | **SE outcome** | **P outcome** | **R**^2^ | **F-statistic** |
| **IBD** |  |  |  |  |  |  |  |
| Sterol ester (27:1/22:6) | rs10737481 | -0.002 | 0.529 | 0.017 | 0.894 | 0.000 | 94.481 |
|  | rs10748781 | 0.027 | 0.654 | 0.018 | 0.131 | 0.000 | 117.622 |
|  | rs10761659 | -0.003 | 0.524 | 0.017 | 0.875 | 0.000 | 62.483 |
|  | rs10807943 | 0.040 | 0.934 | 0.034 | 0.242 | 0.000 | 147.701 |
|  | rs10865941 | -0.011 | 0.425 | 0.017 | 0.525 | 0.000 | 33.505 |
|  | rs10931828 | 0.004 | 0.437 | 0.017 | 0.789 | 0.000 | 41.528 |
|  | rs11064881 | -0.003 | 0.090 | 0.030 | 0.906 | 0.000 | 31.282 |
|  | rs11190368 | -0.033 | 0.453 | 0.017 | 0.050 | 0.000 | 43.387 |
|  | rs11614309 | 0.006 | 0.330 | 0.018 | 0.754 | 0.000 | 36.593 |
|  | rs11745587 | -0.017 | 0.402 | 0.017 | 0.327 | 0.000 | 68.309 |
|  | rs11771806 | -0.018 | 0.123 | 0.026 | 0.494 | 0.000 | 34.844 |
|  | rs12132298 | 0.029 | 0.209 | 0.021 | 0.156 | 0.000 | 62.746 |
|  | rs12139150 | -0.028 | 0.436 | 0.017 | 0.102 | 0.000 | 87.137 |
|  | rs12536069 | 0.007 | 0.070 | 0.033 | 0.845 | 0.000 | 78.159 |
|  | rs1265108 | 0.007 | 0.465 | 0.017 | 0.674 | 0.000 | 33.667 |
|  | rs12736494 | 0.034 | 0.255 | 0.019 | 0.076 | 0.000 | 42.224 |
|  | rs12942330 | -0.003 | 0.522 | 0.017 | 0.841 | 0.000 | 51.745 |
|  | rs12967678 | -0.056 | 0.113 | 0.026 | 0.033 | 0.000 | 31.558 |
|  | rs13029501 | 0.010 | 0.572 | 0.017 | 0.545 | 0.000 | 30.832 |
|  | rs13300483 | -0.007 | 0.343 | 0.018 | 0.712 | 0.000 | 40.975 |
|  | rs142695953 | 0.008 | 0.194 | 0.021 | 0.719 | 0.000 | 88.882 |
|  | rs148170789 | -0.026 | 0.047 | 0.039 | 0.505 | 0.000 | 118.457 |
|  | rs17587460 | 0.010 | 0.245 | 0.020 | 0.622 | 0.000 | 31.365 |
|  | rs181316459 | -0.036 | 0.051 | 0.040 | 0.362 | 0.001 | 420.418 |
|  | rs191615076 | 0.007 | 0.047 | 0.042 | 0.859 | 0.000 | 49.546 |
|  | rs1921622 | -0.019 | 0.502 | 0.017 | 0.265 | 0.000 | 35.306 |
|  | rs1990760 | -0.046 | 0.580 | 0.017 | 0.006 | 0.000 | 43.331 |
|  | rs2149560 | 0.008 | 0.551 | 0.017 | 0.644 | 0.000 | 54.876 |
|  | rs2735075 | -0.002 | 0.425 | 0.017 | 0.889 | 0.000 | 73.784 |
|  | rs2836878 | 0.006 | 0.240 | 0.020 | 0.764 | 0.000 | 57.191 |
|  | rs3024505 | -0.023 | 0.161 | 0.023 | 0.303 | 0.000 | 144.681 |
|  | rs3091316 | -0.030 | 0.331 | 0.018 | 0.097 | 0.000 | 30.035 |
|  | rs3104371 | 0.002 | 0.181 | 0.022 | 0.914 | 0.000 | 135.292 |
|  | rs3197999 | 0.014 | 0.381 | 0.017 | 0.411 | 0.000 | 127.464 |
|  | rs3736161 | 0.012 | 0.382 | 0.017 | 0.496 | 0.000 | 43.814 |
|  | rs376365394 | -0.004 | 0.047 | 0.040 | 0.927 | 0.000 | 49.949 |
|  | rs3778753 | -0.018 | 0.425 | 0.017 | 0.285 | 0.000 | 30.933 |
|  | rs3810491 | 0.000 | 0.219 | 0.020 | 0.992 | 0.000 | 56.520 |
|  | rs4676410 | 0.053 | 0.257 | 0.019 | 0.005 | 0.000 | 116.914 |
|  | rs4730276 | 0.002 | 0.441 | 0.017 | 0.909 | 0.000 | 40.517 |
|  | rs62029968 | 0.001 | 0.257 | 0.019 | 0.958 | 0.000 | 34.906 |
|  | rs6545766 | 0.002 | 0.405 | 0.017 | 0.923 | 0.000 | 29.980 |
|  | rs6762648 | 0.027 | 0.241 | 0.019 | 0.172 | 0.000 | 54.037 |
|  | rs7285952 | -0.055 | 0.113 | 0.027 | 0.041 | 0.000 | 47.228 |
|  | rs7608910 | 0.009 | 0.380 | 0.017 | 0.585 | 0.000 | 41.838 |
|  | rs7704367 | 0.010 | 0.287 | 0.018 | 0.584 | 0.000 | 41.313 |
|  | rs79973254 | -0.018 | 0.066 | 0.034 | 0.588 | 0.000 | 32.096 |
|  | rs842359 | 0.027 | 0.485 | 0.017 | 0.109 | 0.000 | 30.051 |
|  | rs9617090 | -0.012 | 0.363 | 0.017 | 0.496 | 0.000 | 58.513 |
| Phosphatidylcholine (20:4_0:0) | rs10737481 | -0.034 | 0.529 | 0.018 | 0.058 | 0.000 | 94.481 |
|  | rs10748781 | 0.005 | 0.654 | 0.019 | 0.787 | 0.000 | 117.622 |
|  | rs10761659 | 0.015 | 0.524 | 0.018 | 0.403 | 0.000 | 62.483 |
|  | rs10807943 | 0.061 | 0.934 | 0.036 | 0.091 | 0.000 | 147.701 |
|  | rs10865941 | -0.007 | 0.425 | 0.018 | 0.706 | 0.000 | 33.505 |
|  | rs10931828 | 0.012 | 0.437 | 0.018 | 0.502 | 0.000 | 41.528 |
|  | rs11064881 | 0.032 | 0.090 | 0.032 | 0.321 | 0.000 | 31.282 |
|  | rs11190368 | 0.029 | 0.453 | 0.018 | 0.109 | 0.000 | 43.387 |
|  | rs11614309 | 0.010 | 0.330 | 0.019 | 0.585 | 0.000 | 36.593 |
|  | rs11745587 | 0.004 | 0.402 | 0.018 | 0.808 | 0.000 | 68.309 |
|  | rs11771806 | -0.016 | 0.123 | 0.028 | 0.562 | 0.000 | 34.844 |
|  | rs12132298 | 0.018 | 0.209 | 0.022 | 0.423 | 0.000 | 62.746 |
|  | rs12139150 | -0.005 | 0.436 | 0.018 | 0.802 | 0.000 | 87.137 |
|  | rs12536069 | -0.049 | 0.070 | 0.035 | 0.165 | 0.000 | 78.159 |
|  | rs1265108 | -0.008 | 0.465 | 0.018 | 0.637 | 0.000 | 33.667 |
|  | rs12736494 | -0.009 | 0.255 | 0.020 | 0.664 | 0.000 | 42.224 |
|  | rs12942330 | 0.018 | 0.522 | 0.018 | 0.311 | 0.000 | 51.745 |
|  | rs12967678 | -0.042 | 0.113 | 0.028 | 0.139 | 0.000 | 31.558 |
|  | rs13029501 | -0.001 | 0.572 | 0.018 | 0.963 | 0.000 | 30.832 |
|  | rs13300483 | -0.009 | 0.343 | 0.019 | 0.655 | 0.000 | 40.975 |
|  | rs142695953 | -0.009 | 0.194 | 0.023 | 0.686 | 0.000 | 88.882 |
|  | rs148170789 | 0.029 | 0.047 | 0.041 | 0.485 | 0.000 | 118.457 |
|  | rs17587460 | 0.022 | 0.245 | 0.021 | 0.296 | 0.000 | 31.365 |
|  | rs181316459 | -0.020 | 0.051 | 0.042 | 0.629 | 0.001 | 420.418 |
|  | rs191615076 | -0.010 | 0.047 | 0.045 | 0.817 | 0.000 | 49.546 |
|  | rs1921622 | -0.016 | 0.502 | 0.018 | 0.388 | 0.000 | 35.306 |
|  | rs1990760 | 0.006 | 0.580 | 0.018 | 0.740 | 0.000 | 43.331 |
|  | rs2149560 | -0.015 | 0.551 | 0.018 | 0.393 | 0.000 | 54.876 |
|  | rs2735075 | 0.009 | 0.425 | 0.018 | 0.610 | 0.000 | 73.784 |
|  | rs2836878 | 0.000 | 0.240 | 0.022 | 0.988 | 0.000 | 57.191 |
|  | rs3024505 | -0.020 | 0.161 | 0.024 | 0.411 | 0.000 | 144.681 |
|  | rs3091316 | -0.006 | 0.331 | 0.019 | 0.745 | 0.000 | 30.035 |
|  | rs3104371 | 0.030 | 0.181 | 0.023 | 0.200 | 0.000 | 135.292 |
|  | rs3197999 | 0.007 | 0.381 | 0.018 | 0.707 | 0.000 | 127.464 |
|  | rs3736161 | -0.005 | 0.382 | 0.019 | 0.793 | 0.000 | 43.814 |
|  | rs376365394 | -0.016 | 0.047 | 0.044 | 0.708 | 0.000 | 49.949 |
|  | rs3778753 | 0.027 | 0.425 | 0.018 | 0.131 | 0.000 | 30.933 |
|  | rs3810491 | -0.003 | 0.219 | 0.022 | 0.883 | 0.000 | 56.520 |
|  | rs4676410 | -0.003 | 0.257 | 0.020 | 0.886 | 0.000 | 116.914 |
|  | rs4730276 | 0.028 | 0.441 | 0.018 | 0.117 | 0.000 | 40.517 |
|  | rs62029968 | -0.029 | 0.257 | 0.020 | 0.148 | 0.000 | 34.906 |
|  | rs6545766 | 0.013 | 0.405 | 0.018 | 0.488 | 0.000 | 29.980 |
|  | rs6762648 | 0.012 | 0.241 | 0.021 | 0.577 | 0.000 | 54.037 |
|  | rs7285952 | 0.015 | 0.113 | 0.028 | 0.601 | 0.000 | 47.228 |
|  | rs7608910 | 0.018 | 0.380 | 0.018 | 0.324 | 0.000 | 41.838 |
|  | rs7704367 | 0.019 | 0.287 | 0.020 | 0.325 | 0.000 | 41.313 |
|  | rs79973254 | 0.002 | 0.066 | 0.036 | 0.962 | 0.000 | 32.096 |
|  | rs842359 | 0.034 | 0.485 | 0.018 | 0.061 | 0.000 | 30.051 |
|  | rs9617090 | 0.029 | 0.363 | 0.019 | 0.123 | 0.000 | 58.513 |
| Phosphatidylcholine (16:0_20:4) | rs10737481 | -0.014 | 0.529 | 0.017 | 0.413 | 0.000 | 94.481 |
|  | rs10748781 | 0.010 | 0.654 | 0.018 | 0.561 | 0.000 | 117.622 |
|  | rs10761659 | 0.006 | 0.524 | 0.017 | 0.715 | 0.000 | 62.483 |
|  | rs10807943 | 0.050 | 0.934 | 0.034 | 0.137 | 0.000 | 147.701 |
|  | rs10865941 | -0.003 | 0.425 | 0.017 | 0.851 | 0.000 | 33.505 |
|  | rs10931828 | 0.021 | 0.437 | 0.017 | 0.208 | 0.000 | 41.528 |
|  | rs11064881 | -0.008 | 0.090 | 0.030 | 0.783 | 0.000 | 31.282 |
|  | rs11190368 | -0.003 | 0.453 | 0.017 | 0.860 | 0.000 | 43.387 |
|  | rs11614309 | 0.030 | 0.330 | 0.018 | 0.093 | 0.000 | 36.593 |
|  | rs11745587 | -0.032 | 0.402 | 0.017 | 0.062 | 0.000 | 68.309 |
|  | rs11771806 | -0.004 | 0.123 | 0.026 | 0.887 | 0.000 | 34.844 |
|  | rs12132298 | -0.002 | 0.209 | 0.021 | 0.929 | 0.000 | 62.746 |
|  | rs12139150 | -0.014 | 0.436 | 0.017 | 0.413 | 0.000 | 87.137 |
|  | rs12536069 | -0.020 | 0.070 | 0.033 | 0.551 | 0.000 | 78.159 |
|  | rs1265108 | 0.011 | 0.465 | 0.017 | 0.501 | 0.000 | 33.667 |
|  | rs12736494 | -0.010 | 0.255 | 0.019 | 0.595 | 0.000 | 42.224 |
|  | rs12942330 | 0.024 | 0.522 | 0.017 | 0.145 | 0.000 | 51.745 |
|  | rs12967678 | -0.064 | 0.113 | 0.026 | 0.015 | 0.000 | 31.558 |
|  | rs13029501 | 0.004 | 0.572 | 0.017 | 0.826 | 0.000 | 30.832 |
|  | rs13300483 | -0.009 | 0.343 | 0.018 | 0.608 | 0.000 | 40.975 |
|  | rs142695953 | -0.034 | 0.194 | 0.021 | 0.106 | 0.000 | 88.882 |
|  | rs148170789 | 0.047 | 0.047 | 0.039 | 0.225 | 0.000 | 118.457 |
|  | rs17587460 | -0.008 | 0.245 | 0.020 | 0.682 | 0.000 | 31.365 |
|  | rs181316459 | -0.021 | 0.051 | 0.039 | 0.592 | 0.001 | 420.418 |
|  | rs191615076 | 0.039 | 0.047 | 0.042 | 0.351 | 0.000 | 49.546 |
|  | rs1921622 | -0.035 | 0.502 | 0.017 | 0.036 | 0.000 | 35.306 |
|  | rs1990760 | -0.034 | 0.580 | 0.017 | 0.042 | 0.000 | 43.331 |
|  | rs2149560 | 0.009 | 0.551 | 0.017 | 0.576 | 0.000 | 54.876 |
|  | rs2735075 | 0.002 | 0.425 | 0.017 | 0.922 | 0.000 | 73.784 |
|  | rs2836878 | 0.000 | 0.240 | 0.020 | 0.993 | 0.000 | 57.191 |
|  | rs3024505 | 0.004 | 0.161 | 0.023 | 0.867 | 0.000 | 144.681 |
|  | rs3091316 | 0.003 | 0.331 | 0.018 | 0.859 | 0.000 | 30.035 |
|  | rs3104371 | 0.039 | 0.181 | 0.022 | 0.074 | 0.000 | 135.292 |
|  | rs3197999 | 0.021 | 0.381 | 0.017 | 0.216 | 0.000 | 127.464 |
|  | rs3736161 | -0.002 | 0.382 | 0.017 | 0.888 | 0.000 | 43.814 |
|  | rs376365394 | 0.036 | 0.047 | 0.040 | 0.374 | 0.000 | 49.949 |
|  | rs3778753 | -0.011 | 0.425 | 0.017 | 0.517 | 0.000 | 30.933 |
|  | rs3810491 | 0.001 | 0.219 | 0.020 | 0.948 | 0.000 | 56.520 |
|  | rs4676410 | 0.002 | 0.257 | 0.019 | 0.920 | 0.000 | 116.914 |
|  | rs4730276 | -0.001 | 0.441 | 0.017 | 0.932 | 0.000 | 40.517 |
|  | rs62029968 | -0.014 | 0.257 | 0.019 | 0.447 | 0.000 | 34.906 |
|  | rs6545766 | 0.009 | 0.405 | 0.017 | 0.614 | 0.000 | 29.980 |
|  | rs6762648 | 0.039 | 0.241 | 0.019 | 0.041 | 0.000 | 54.037 |
|  | rs7285952 | 0.014 | 0.113 | 0.027 | 0.612 | 0.000 | 47.228 |
|  | rs7608910 | 0.018 | 0.380 | 0.017 | 0.310 | 0.000 | 41.838 |
|  | rs7704367 | 0.004 | 0.287 | 0.018 | 0.824 | 0.000 | 41.313 |
|  | rs79973254 | 0.023 | 0.066 | 0.034 | 0.493 | 0.000 | 32.096 |
|  | rs842359 | 0.028 | 0.485 | 0.017 | 0.096 | 0.000 | 30.051 |
|  | rs9617090 | 0.000 | 0.363 | 0.017 | 0.999 | 0.000 | 58.513 |
| Phosphatidylcholine (17:0_18:2) | rs10737481 | -0.022 | 0.529 | 0.017 | 0.205 | 0.000 | 94.481 |
|  | rs10748781 | -0.001 | 0.654 | 0.018 | 0.960 | 0.000 | 117.622 |
|  | rs10761659 | -0.011 | 0.524 | 0.017 | 0.501 | 0.000 | 62.483 |
|  | rs10807943 | -0.017 | 0.934 | 0.034 | 0.610 | 0.000 | 147.701 |
|  | rs10865941 | -0.023 | 0.425 | 0.017 | 0.180 | 0.000 | 33.505 |
|  | rs10931828 | 0.025 | 0.437 | 0.017 | 0.138 | 0.000 | 41.528 |
|  | rs11064881 | -0.036 | 0.090 | 0.030 | 0.220 | 0.000 | 31.282 |
|  | rs11190368 | 0.006 | 0.453 | 0.017 | 0.732 | 0.000 | 43.387 |
|  | rs11614309 | 0.009 | 0.330 | 0.018 | 0.597 | 0.000 | 36.593 |
|  | rs11745587 | 0.020 | 0.402 | 0.017 | 0.235 | 0.000 | 68.309 |
|  | rs11771806 | 0.015 | 0.123 | 0.026 | 0.557 | 0.000 | 34.844 |
|  | rs12132298 | -0.009 | 0.209 | 0.021 | 0.680 | 0.000 | 62.746 |
|  | rs12139150 | 0.016 | 0.436 | 0.017 | 0.346 | 0.000 | 87.137 |
|  | rs12536069 | 0.012 | 0.070 | 0.033 | 0.730 | 0.000 | 78.159 |
|  | rs1265108 | 0.004 | 0.465 | 0.017 | 0.806 | 0.000 | 33.667 |
|  | rs12736494 | -0.025 | 0.255 | 0.019 | 0.194 | 0.000 | 42.224 |
|  | rs12942330 | 0.029 | 0.522 | 0.017 | 0.078 | 0.000 | 51.745 |
|  | rs12967678 | -0.013 | 0.113 | 0.026 | 0.631 | 0.000 | 31.558 |
|  | rs13029501 | -0.015 | 0.572 | 0.017 | 0.374 | 0.000 | 30.832 |
|  | rs13300483 | 0.009 | 0.343 | 0.018 | 0.597 | 0.000 | 40.975 |
|  | rs142695953 | 0.014 | 0.194 | 0.021 | 0.506 | 0.000 | 88.882 |
|  | rs148170789 | -0.031 | 0.047 | 0.039 | 0.429 | 0.000 | 118.457 |
|  | rs17587460 | -0.008 | 0.245 | 0.020 | 0.677 | 0.000 | 31.365 |
|  | rs181316459 | -0.005 | 0.051 | 0.040 | 0.894 | 0.001 | 420.418 |
|  | rs191615076 | 0.035 | 0.047 | 0.042 | 0.404 | 0.000 | 49.546 |
|  | rs1921622 | -0.023 | 0.502 | 0.017 | 0.178 | 0.000 | 35.306 |
|  | rs1990760 | -0.039 | 0.580 | 0.017 | 0.022 | 0.000 | 43.331 |
|  | rs2149560 | 0.010 | 0.551 | 0.017 | 0.546 | 0.000 | 54.876 |
|  | rs2735075 | 0.003 | 0.425 | 0.017 | 0.868 | 0.000 | 73.784 |
|  | rs2836878 | -0.017 | 0.240 | 0.020 | 0.407 | 0.000 | 57.191 |
|  | rs3024505 | -0.019 | 0.161 | 0.023 | 0.392 | 0.000 | 144.681 |
|  | rs3091316 | -0.006 | 0.331 | 0.018 | 0.727 | 0.000 | 30.035 |
|  | rs3104371 | 0.022 | 0.181 | 0.022 | 0.317 | 0.000 | 135.292 |
|  | rs3197999 | -0.012 | 0.381 | 0.017 | 0.479 | 0.000 | 127.464 |
|  | rs3736161 | 0.028 | 0.382 | 0.017 | 0.104 | 0.000 | 43.814 |
|  | rs376365394 | 0.028 | 0.047 | 0.040 | 0.494 | 0.000 | 49.949 |
|  | rs3778753 | 0.004 | 0.425 | 0.017 | 0.826 | 0.000 | 30.933 |
|  | rs3810491 | -0.008 | 0.219 | 0.020 | 0.686 | 0.000 | 56.520 |
|  | rs4676410 | 0.011 | 0.257 | 0.019 | 0.549 | 0.000 | 116.914 |
|  | rs4730276 | 0.010 | 0.441 | 0.017 | 0.566 | 0.000 | 40.517 |
|  | rs62029968 | -0.012 | 0.257 | 0.019 | 0.526 | 0.000 | 34.906 |
|  | rs6545766 | -0.024 | 0.405 | 0.017 | 0.166 | 0.000 | 29.980 |
|  | rs6762648 | 0.012 | 0.241 | 0.019 | 0.523 | 0.000 | 54.037 |
|  | rs7285952 | 0.002 | 0.113 | 0.027 | 0.929 | 0.000 | 47.228 |
|  | rs7608910 | -0.010 | 0.380 | 0.017 | 0.548 | 0.000 | 41.838 |
|  | rs7704367 | 0.029 | 0.287 | 0.018 | 0.115 | 0.000 | 41.313 |
|  | rs79973254 | 0.005 | 0.066 | 0.034 | 0.880 | 0.000 | 32.096 |
|  | rs842359 | -0.003 | 0.485 | 0.017 | 0.875 | 0.000 | 30.051 |
|  | rs9617090 | -0.003 | 0.363 | 0.017 | 0.847 | 0.000 | 58.513 |
| Sphingomyelin (d36:1) | rs10737481 | -0.012 | 0.529 | 0.017 | 0.490 | 0.000 | 94.481 |
|  | rs10748781 | -0.025 | 0.654 | 0.018 | 0.152 | 0.000 | 117.622 |
|  | rs10761659 | -0.005 | 0.524 | 0.017 | 0.785 | 0.000 | 62.483 |
|  | rs10807943 | -0.001 | 0.934 | 0.034 | 0.975 | 0.000 | 147.701 |
|  | rs10865941 | -0.007 | 0.425 | 0.017 | 0.666 | 0.000 | 33.505 |
|  | rs10931828 | 0.001 | 0.437 | 0.017 | 0.939 | 0.000 | 41.528 |
|  | rs11064881 | 0.000 | 0.090 | 0.030 | 0.994 | 0.000 | 31.282 |
|  | rs11190368 | 0.005 | 0.453 | 0.017 | 0.781 | 0.000 | 43.387 |
|  | rs11614309 | 0.001 | 0.330 | 0.018 | 0.938 | 0.000 | 36.593 |
|  | rs11745587 | 0.031 | 0.402 | 0.017 | 0.065 | 0.000 | 68.309 |
|  | rs11771806 | -0.052 | 0.123 | 0.026 | 0.044 | 0.000 | 34.844 |
|  | rs12132298 | 0.015 | 0.209 | 0.021 | 0.471 | 0.000 | 62.746 |
|  | rs12139150 | 0.013 | 0.436 | 0.017 | 0.438 | 0.000 | 87.137 |
|  | rs12536069 | 0.017 | 0.070 | 0.033 | 0.612 | 0.000 | 78.159 |
|  | rs1265108 | 0.023 | 0.465 | 0.017 | 0.167 | 0.000 | 33.667 |
|  | rs12736494 | 0.011 | 0.255 | 0.019 | 0.566 | 0.000 | 42.224 |
|  | rs12942330 | -0.001 | 0.522 | 0.017 | 0.962 | 0.000 | 51.745 |
|  | rs12967678 | -0.037 | 0.113 | 0.026 | 0.160 | 0.000 | 31.558 |
|  | rs13029501 | -0.001 | 0.572 | 0.017 | 0.971 | 0.000 | 30.832 |
|  | rs13300483 | 0.014 | 0.343 | 0.018 | 0.446 | 0.000 | 40.975 |
|  | rs142695953 | 0.000 | 0.194 | 0.021 | 0.995 | 0.000 | 88.882 |
|  | rs148170789 | 0.021 | 0.047 | 0.039 | 0.590 | 0.000 | 118.457 |
|  | rs17587460 | 0.017 | 0.245 | 0.020 | 0.395 | 0.000 | 31.365 |
|  | rs181316459 | -0.015 | 0.051 | 0.040 | 0.702 | 0.001 | 420.418 |
|  | rs191615076 | -0.005 | 0.047 | 0.042 | 0.912 | 0.000 | 49.546 |
|  | rs1921622 | -0.037 | 0.502 | 0.017 | 0.029 | 0.000 | 35.306 |
|  | rs1990760 | -0.010 | 0.580 | 0.017 | 0.537 | 0.000 | 43.331 |
|  | rs2149560 | 0.013 | 0.551 | 0.017 | 0.426 | 0.000 | 54.876 |
|  | rs2735075 | -0.002 | 0.425 | 0.017 | 0.882 | 0.000 | 73.784 |
|  | rs2836878 | -0.027 | 0.240 | 0.020 | 0.182 | 0.000 | 57.191 |
|  | rs3024505 | -0.026 | 0.161 | 0.023 | 0.243 | 0.000 | 144.681 |
|  | rs3091316 | -0.022 | 0.331 | 0.018 | 0.217 | 0.000 | 30.035 |
|  | rs3104371 | 0.056 | 0.181 | 0.022 | 0.011 | 0.000 | 135.292 |
|  | rs3197999 | 0.010 | 0.381 | 0.017 | 0.568 | 0.000 | 127.464 |
|  | rs3736161 | 0.010 | 0.382 | 0.017 | 0.557 | 0.000 | 43.814 |
|  | rs376365394 | -0.008 | 0.047 | 0.040 | 0.846 | 0.000 | 49.949 |
|  | rs3778753 | -0.005 | 0.425 | 0.017 | 0.749 | 0.000 | 30.933 |
|  | rs3810491 | 0.025 | 0.219 | 0.020 | 0.217 | 0.000 | 56.520 |
|  | rs4676410 | -0.003 | 0.257 | 0.019 | 0.891 | 0.000 | 116.914 |
|  | rs4730276 | 0.015 | 0.441 | 0.017 | 0.371 | 0.000 | 40.517 |
|  | rs62029968 | -0.012 | 0.257 | 0.019 | 0.520 | 0.000 | 34.906 |
|  | rs6545766 | 0.006 | 0.405 | 0.017 | 0.743 | 0.000 | 29.980 |
|  | rs6762648 | 0.002 | 0.241 | 0.019 | 0.909 | 0.000 | 54.037 |
|  | rs7285952 | -0.010 | 0.113 | 0.027 | 0.722 | 0.000 | 47.228 |
|  | rs7608910 | -0.008 | 0.380 | 0.017 | 0.661 | 0.000 | 41.838 |
|  | rs7704367 | 0.010 | 0.287 | 0.018 | 0.587 | 0.000 | 41.313 |
|  | rs79973254 | -0.033 | 0.066 | 0.034 | 0.327 | 0.000 | 32.096 |
|  | rs842359 | 0.007 | 0.485 | 0.017 | 0.687 | 0.000 | 30.051 |
|  | rs9617090 | -0.009 | 0.363 | 0.017 | 0.604 | 0.000 | 58.513 |
| Sphingomyelin (d38:1) | rs10737481 | -0.019 | 0.529 | 0.017 | 0.252 | 0.000 | 94.481 |
|  | rs10748781 | -0.010 | 0.654 | 0.018 | 0.578 | 0.000 | 117.622 |
|  | rs10761659 | 0.017 | 0.524 | 0.017 | 0.296 | 0.000 | 62.483 |
|  | rs10807943 | -0.022 | 0.934 | 0.034 | 0.523 | 0.000 | 147.701 |
|  | rs10865941 | 0.003 | 0.425 | 0.017 | 0.840 | 0.000 | 33.505 |
|  | rs10931828 | 0.005 | 0.437 | 0.017 | 0.771 | 0.000 | 41.528 |
|  | rs11064881 | -0.024 | 0.090 | 0.030 | 0.425 | 0.000 | 31.282 |
|  | rs11190368 | -0.004 | 0.453 | 0.017 | 0.788 | 0.000 | 43.387 |
|  | rs11614309 | 0.005 | 0.330 | 0.018 | 0.797 | 0.000 | 36.593 |
|  | rs11745587 | 0.040 | 0.402 | 0.017 | 0.020 | 0.000 | 68.309 |
|  | rs11771806 | -0.014 | 0.123 | 0.026 | 0.580 | 0.000 | 34.844 |
|  | rs12132298 | 0.009 | 0.209 | 0.021 | 0.667 | 0.000 | 62.746 |
|  | rs12139150 | 0.012 | 0.436 | 0.017 | 0.479 | 0.000 | 87.137 |
|  | rs12536069 | 0.008 | 0.070 | 0.033 | 0.802 | 0.000 | 78.159 |
|  | rs1265108 | 0.025 | 0.465 | 0.017 | 0.134 | 0.000 | 33.667 |
|  | rs12736494 | 0.007 | 0.255 | 0.019 | 0.698 | 0.000 | 42.224 |
|  | rs12942330 | -0.002 | 0.522 | 0.017 | 0.915 | 0.000 | 51.745 |
|  | rs12967678 | -0.052 | 0.113 | 0.026 | 0.047 | 0.000 | 31.558 |
|  | rs13029501 | -0.006 | 0.572 | 0.017 | 0.728 | 0.000 | 30.832 |
|  | rs13300483 | 0.032 | 0.343 | 0.018 | 0.073 | 0.000 | 40.975 |
|  | rs142695953 | -0.005 | 0.194 | 0.021 | 0.828 | 0.000 | 88.882 |
|  | rs148170789 | 0.019 | 0.047 | 0.039 | 0.623 | 0.000 | 118.457 |
|  | rs17587460 | 0.015 | 0.245 | 0.020 | 0.454 | 0.000 | 31.365 |
|  | rs181316459 | -0.027 | 0.051 | 0.040 | 0.490 | 0.001 | 420.418 |
|  | rs191615076 | -0.081 | 0.047 | 0.042 | 0.051 | 0.000 | 49.546 |
|  | rs1921622 | -0.043 | 0.502 | 0.017 | 0.011 | 0.000 | 35.306 |
|  | rs1990760 | -0.005 | 0.580 | 0.017 | 0.760 | 0.000 | 43.331 |
|  | rs2149560 | 0.002 | 0.551 | 0.017 | 0.886 | 0.000 | 54.876 |
|  | rs2735075 | 0.011 | 0.425 | 0.017 | 0.512 | 0.000 | 73.784 |
|  | rs2836878 | -0.027 | 0.240 | 0.020 | 0.185 | 0.000 | 57.191 |
|  | rs3024505 | -0.040 | 0.161 | 0.023 | 0.077 | 0.000 | 144.681 |
|  | rs3091316 | -0.016 | 0.331 | 0.018 | 0.374 | 0.000 | 30.035 |
|  | rs3104371 | 0.022 | 0.181 | 0.022 | 0.322 | 0.000 | 135.292 |
|  | rs3197999 | 0.000 | 0.381 | 0.017 | 0.981 | 0.000 | 127.464 |
|  | rs3736161 | 0.027 | 0.382 | 0.017 | 0.117 | 0.000 | 43.814 |
|  | rs376365394 | -0.080 | 0.047 | 0.040 | 0.047 | 0.000 | 49.949 |
|  | rs3778753 | -0.004 | 0.425 | 0.017 | 0.797 | 0.000 | 30.933 |
|  | rs3810491 | 0.031 | 0.219 | 0.020 | 0.131 | 0.000 | 56.520 |
|  | rs4676410 | -0.007 | 0.257 | 0.019 | 0.702 | 0.000 | 116.914 |
|  | rs4730276 | -0.008 | 0.441 | 0.017 | 0.641 | 0.000 | 40.517 |
|  | rs62029968 | 0.011 | 0.257 | 0.019 | 0.564 | 0.000 | 34.906 |
|  | rs6545766 | 0.010 | 0.405 | 0.017 | 0.576 | 0.000 | 29.980 |
|  | rs6762648 | 0.030 | 0.241 | 0.019 | 0.123 | 0.000 | 54.037 |
|  | rs7285952 | 0.002 | 0.113 | 0.027 | 0.948 | 0.000 | 47.228 |
|  | rs7608910 | -0.034 | 0.380 | 0.017 | 0.048 | 0.000 | 41.838 |
|  | rs7704367 | 0.020 | 0.287 | 0.018 | 0.275 | 0.000 | 41.313 |
|  | rs79973254 | -0.030 | 0.066 | 0.034 | 0.369 | 0.000 | 32.096 |
|  | rs842359 | 0.000 | 0.485 | 0.017 | 0.995 | 0.000 | 30.051 |
|  | rs9617090 | 0.009 | 0.363 | 0.017 | 0.617 | 0.000 | 58.513 |
| Sphingomyelin (d42:2) | rs10737481 | -0.003 | 0.529 | 0.017 | 0.873 | 0.000 | 94.481 |
|  | rs10748781 | 0.024 | 0.654 | 0.018 | 0.174 | 0.000 | 117.622 |
|  | rs10761659 | -0.007 | 0.524 | 0.017 | 0.691 | 0.000 | 62.483 |
|  | rs10807943 | -0.016 | 0.934 | 0.034 | 0.637 | 0.000 | 147.701 |
|  | rs10865941 | -0.024 | 0.425 | 0.017 | 0.156 | 0.000 | 33.505 |
|  | rs10931828 | 0.010 | 0.437 | 0.017 | 0.549 | 0.000 | 41.528 |
|  | rs11064881 | -0.018 | 0.090 | 0.030 | 0.549 | 0.000 | 31.282 |
|  | rs11190368 | -0.003 | 0.453 | 0.017 | 0.842 | 0.000 | 43.387 |
|  | rs11614309 | 0.019 | 0.330 | 0.018 | 0.287 | 0.000 | 36.593 |
|  | rs11745587 | 0.071 | 0.402 | 0.017 | 0.000 | 0.000 | 68.309 |
|  | rs11771806 | 0.005 | 0.123 | 0.026 | 0.838 | 0.000 | 34.844 |
|  | rs12132298 | 0.006 | 0.209 | 0.021 | 0.789 | 0.000 | 62.746 |
|  | rs12139150 | 0.005 | 0.436 | 0.017 | 0.764 | 0.000 | 87.137 |
|  | rs12536069 | 0.034 | 0.070 | 0.033 | 0.301 | 0.000 | 78.159 |
|  | rs1265108 | 0.015 | 0.465 | 0.017 | 0.376 | 0.000 | 33.667 |
|  | rs12736494 | 0.021 | 0.255 | 0.019 | 0.259 | 0.000 | 42.224 |
|  | rs12942330 | 0.007 | 0.522 | 0.017 | 0.694 | 0.000 | 51.745 |
|  | rs12967678 | -0.022 | 0.113 | 0.026 | 0.408 | 0.000 | 31.558 |
|  | rs13029501 | 0.003 | 0.572 | 0.017 | 0.879 | 0.000 | 30.832 |
|  | rs13300483 | -0.007 | 0.343 | 0.018 | 0.686 | 0.000 | 40.975 |
|  | rs142695953 | 0.038 | 0.194 | 0.021 | 0.078 | 0.000 | 88.882 |
|  | rs148170789 | 0.020 | 0.047 | 0.039 | 0.609 | 0.000 | 118.457 |
|  | rs17587460 | 0.004 | 0.245 | 0.020 | 0.825 | 0.000 | 31.365 |
|  | rs181316459 | 0.010 | 0.051 | 0.040 | 0.801 | 0.001 | 420.418 |
|  | rs191615076 | -0.036 | 0.047 | 0.042 | 0.388 | 0.000 | 49.546 |
|  | rs1921622 | -0.020 | 0.502 | 0.017 | 0.244 | 0.000 | 35.306 |
|  | rs1990760 | -0.031 | 0.580 | 0.017 | 0.063 | 0.000 | 43.331 |
|  | rs2149560 | 0.016 | 0.551 | 0.017 | 0.341 | 0.000 | 54.876 |
|  | rs2735075 | 0.015 | 0.425 | 0.017 | 0.382 | 0.000 | 73.784 |
|  | rs2836878 | -0.049 | 0.240 | 0.020 | 0.014 | 0.000 | 57.191 |
|  | rs3024505 | -0.029 | 0.161 | 0.023 | 0.206 | 0.000 | 144.681 |
|  | rs3091316 | -0.025 | 0.331 | 0.018 | 0.161 | 0.000 | 30.035 |
|  | rs3104371 | 0.045 | 0.181 | 0.022 | 0.039 | 0.000 | 135.292 |
|  | rs3197999 | -0.027 | 0.381 | 0.017 | 0.117 | 0.000 | 127.464 |
|  | rs3736161 | -0.002 | 0.382 | 0.017 | 0.888 | 0.000 | 43.814 |
|  | rs376365394 | -0.036 | 0.047 | 0.040 | 0.378 | 0.000 | 49.949 |
|  | rs3778753 | 0.008 | 0.425 | 0.017 | 0.615 | 0.000 | 30.933 |
|  | rs3810491 | 0.023 | 0.219 | 0.020 | 0.256 | 0.000 | 56.520 |
|  | rs4676410 | 0.009 | 0.257 | 0.019 | 0.624 | 0.000 | 116.914 |
|  | rs4730276 | 0.001 | 0.441 | 0.017 | 0.952 | 0.000 | 40.517 |
|  | rs62029968 | 0.019 | 0.257 | 0.019 | 0.314 | 0.000 | 34.906 |
|  | rs6545766 | 0.036 | 0.405 | 0.017 | 0.037 | 0.000 | 29.980 |
|  | rs6762648 | 0.046 | 0.241 | 0.019 | 0.018 | 0.000 | 54.037 |
|  | rs7285952 | -0.006 | 0.113 | 0.027 | 0.833 | 0.000 | 47.228 |
|  | rs7608910 | 0.000 | 0.380 | 0.017 | 0.997 | 0.000 | 41.838 |
|  | rs7704367 | -0.003 | 0.287 | 0.018 | 0.857 | 0.000 | 41.313 |
|  | rs79973254 | -0.024 | 0.066 | 0.034 | 0.477 | 0.000 | 32.096 |
|  | rs842359 | 0.021 | 0.485 | 0.017 | 0.221 | 0.000 | 30.051 |
|  | rs9617090 | 0.004 | 0.363 | 0.017 | 0.836 | 0.000 | 58.513 |
| **UC** |  |  |  |  |  |  |  |
| Phosphatidylcholine (20:4_0:0) | rs1042131 | 0.002 | 0.494 | 0.018 | 0.891 | 0.000 | 56.562 |
|  | rs1061537 | 0.002 | 0.388 | 0.018 | 0.916 | 0.000 | 59.252 |
|  | rs10748781 | 0.005 | 0.654 | 0.019 | 0.787 | 0.000 | 77.102 |
|  | rs10761659 | 0.015 | 0.524 | 0.018 | 0.403 | 0.000 | 35.706 |
|  | rs10807943 | 0.061 | 0.934 | 0.036 | 0.091 | 0.000 | 87.734 |
|  | rs10931828 | 0.012 | 0.437 | 0.018 | 0.502 | 0.000 | 33.803 |
|  | rs111432088 | -0.039 | 0.017 | 0.069 | 0.575 | 0.000 | 55.020 |
|  | rs11190368 | 0.029 | 0.453 | 0.018 | 0.109 | 0.000 | 31.466 |
|  | rs113595266 | -0.028 | 0.147 | 0.025 | 0.271 | 0.000 | 36.428 |
|  | rs113827118 | -0.009 | 0.194 | 0.023 | 0.688 | 0.000 | 71.157 |
|  | rs11614178 | 0.015 | 0.342 | 0.019 | 0.437 | 0.000 | 39.329 |
|  | rs11745587 | 0.004 | 0.402 | 0.018 | 0.808 | 0.000 | 45.225 |
|  | rs12132298 | 0.018 | 0.209 | 0.022 | 0.423 | 0.000 | 39.640 |
|  | rs12518457 | 0.016 | 0.285 | 0.020 | 0.423 | 0.000 | 30.509 |
|  | rs12536069 | -0.049 | 0.070 | 0.035 | 0.165 | 0.000 | 49.953 |
|  | rs12736494 | -0.009 | 0.255 | 0.020 | 0.664 | 0.000 | 34.777 |
|  | rs12942330 | 0.018 | 0.522 | 0.018 | 0.311 | 0.000 | 47.274 |
|  | rs148170789 | 0.029 | 0.047 | 0.041 | 0.485 | 0.000 | 56.054 |
|  | rs1801274 | -0.001 | 0.496 | 0.018 | 0.943 | 0.000 | 77.438 |
|  | rs181316459 | -0.020 | 0.051 | 0.042 | 0.629 | 0.001 | 266.342 |
|  | rs1990760 | 0.006 | 0.580 | 0.018 | 0.740 | 0.000 | 31.861 |
|  | rs2836883 | 0.001 | 0.240 | 0.022 | 0.973 | 0.000 | 51.168 |
|  | rs3024495 | -0.022 | 0.161 | 0.024 | 0.364 | 0.000 | 121.946 |
|  | rs3197999 | 0.007 | 0.381 | 0.018 | 0.707 | 0.000 | 106.975 |
|  | rs34145423 | 0.050 | 0.076 | 0.034 | 0.138 | 0.000 | 31.831 |
|  | rs34236350 | -0.006 | 0.254 | 0.020 | 0.768 | 0.000 | 81.068 |
|  | rs3732179 | 0.006 | 0.687 | 0.019 | 0.762 | 0.000 | 30.902 |
|  | rs4587163 | 0.026 | 0.270 | 0.020 | 0.205 | 0.000 | 144.846 |
|  | rs4655159 | -0.004 | 0.633 | 0.019 | 0.849 | 0.000 | 31.347 |
|  | rs4730276 | 0.028 | 0.441 | 0.018 | 0.117 | 0.000 | 68.499 |
|  | rs6089926 | 0.000 | 0.206 | 0.022 | 0.991 | 0.000 | 44.287 |
|  | rs6674040 | 0.037 | 0.468 | 0.018 | 0.040 | 0.000 | 96.537 |
|  | rs7101270 | -0.019 | 0.243 | 0.021 | 0.353 | 0.000 | 31.065 |
|  | rs7761376 | 0.001 | 0.616 | 0.018 | 0.963 | 0.000 | 30.777 |
|  | rs7865719 | -0.017 | 0.552 | 0.018 | 0.327 | 0.000 | 35.166 |
|  | rs79973254 | 0.002 | 0.066 | 0.036 | 0.962 | 0.000 | 30.306 |
|  | rs9607629 | 0.024 | 0.119 | 0.028 | 0.395 | 0.000 | 44.168 |
|  | rs9617090 | 0.029 | 0.363 | 0.019 | 0.123 | 0.000 | 64.731 |
| Phosphatidylethanolamine (18:2_0:0) | rs1042131 | -0.007 | 0.494 | 0.017 | 0.687 | 0.000 | 56.562 |
|  | rs1061537 | -0.016 | 0.388 | 0.017 | 0.361 | 0.000 | 59.252 |
|  | rs10748781 | -0.006 | 0.654 | 0.018 | 0.721 | 0.000 | 77.102 |
|  | rs10761659 | 0.002 | 0.524 | 0.017 | 0.888 | 0.000 | 35.706 |
|  | rs10807943 | -0.006 | 0.934 | 0.035 | 0.863 | 0.000 | 87.734 |
|  | rs10931828 | 0.034 | 0.437 | 0.017 | 0.043 | 0.000 | 33.803 |
|  | rs111432088 | 0.041 | 0.017 | 0.066 | 0.539 | 0.000 | 55.020 |
|  | rs11190368 | 0.033 | 0.453 | 0.017 | 0.048 | 0.000 | 31.466 |
|  | rs113595266 | -0.022 | 0.147 | 0.024 | 0.358 | 0.000 | 36.428 |
|  | rs113827118 | 0.008 | 0.194 | 0.022 | 0.721 | 0.000 | 71.157 |
|  | rs11614178 | -0.007 | 0.342 | 0.018 | 0.717 | 0.000 | 39.329 |
|  | rs11745587 | 0.039 | 0.402 | 0.017 | 0.026 | 0.000 | 45.225 |
|  | rs12132298 | -0.015 | 0.209 | 0.021 | 0.491 | 0.000 | 39.640 |
|  | rs12518457 | 0.004 | 0.285 | 0.019 | 0.811 | 0.000 | 30.509 |
|  | rs12536069 | -0.019 | 0.070 | 0.034 | 0.583 | 0.000 | 49.953 |
|  | rs12736494 | -0.033 | 0.255 | 0.019 | 0.092 | 0.000 | 34.777 |
|  | rs12942330 | 0.011 | 0.522 | 0.017 | 0.538 | 0.000 | 47.274 |
|  | rs148170789 | 0.054 | 0.047 | 0.040 | 0.172 | 0.000 | 56.054 |
|  | rs1801274 | 0.023 | 0.496 | 0.017 | 0.192 | 0.000 | 77.438 |
|  | rs181316459 | -0.045 | 0.051 | 0.040 | 0.271 | 0.001 | 266.342 |
|  | rs1990760 | -0.003 | 0.580 | 0.017 | 0.859 | 0.000 | 31.861 |
|  | rs2836883 | 0.006 | 0.240 | 0.021 | 0.766 | 0.000 | 51.168 |
|  | rs3024495 | -0.026 | 0.161 | 0.023 | 0.259 | 0.000 | 121.946 |
|  | rs3197999 | -0.039 | 0.381 | 0.017 | 0.024 | 0.000 | 106.975 |
|  | rs34145423 | 0.018 | 0.076 | 0.033 | 0.589 | 0.000 | 31.831 |
|  | rs34236350 | -0.028 | 0.254 | 0.019 | 0.151 | 0.000 | 81.068 |
|  | rs3732179 | 0.012 | 0.687 | 0.018 | 0.505 | 0.000 | 30.902 |
|  | rs4587163 | 0.004 | 0.270 | 0.019 | 0.817 | 0.000 | 144.846 |
|  | rs4655159 | -0.020 | 0.633 | 0.018 | 0.270 | 0.000 | 31.347 |
|  | rs4730276 | 0.033 | 0.441 | 0.017 | 0.051 | 0.000 | 68.499 |
|  | rs6089926 | 0.012 | 0.206 | 0.021 | 0.569 | 0.000 | 44.287 |
|  | rs6674040 | 0.013 | 0.468 | 0.017 | 0.463 | 0.000 | 96.537 |
|  | rs7101270 | -0.015 | 0.243 | 0.020 | 0.439 | 0.000 | 31.065 |
|  | rs7761376 | -0.016 | 0.616 | 0.018 | 0.361 | 0.000 | 30.777 |
|  | rs7865719 | 0.008 | 0.552 | 0.017 | 0.622 | 0.000 | 35.166 |
|  | rs79973254 | -0.003 | 0.066 | 0.034 | 0.921 | 0.000 | 30.306 |
|  | rs9607629 | 0.037 | 0.119 | 0.027 | 0.171 | 0.000 | 44.168 |
|  | rs9617090 | 0.020 | 0.363 | 0.018 | 0.269 | 0.000 | 64.731 |
| Phosphatidylcholine (14:0_16:0 | rs1042131 | -0.027 | 0.494 | 0.019 | 0.165 | 0.000 | 56.562 |
|  | rs1061537 | -0.010 | 0.388 | 0.019 | 0.624 | 0.000 | 59.252 |
|  | rs10748781 | 0.019 | 0.654 | 0.020 | 0.350 | 0.000 | 77.102 |
|  | rs10761659 | 0.037 | 0.524 | 0.019 | 0.052 | 0.000 | 35.706 |
|  | rs10807943 | 0.032 | 0.934 | 0.039 | 0.413 | 0.000 | 87.734 |
|  | rs10931828 | 0.025 | 0.437 | 0.019 | 0.184 | 0.000 | 33.803 |
|  | rs111432088 | 0.026 | 0.017 | 0.074 | 0.728 | 0.000 | 55.020 |
|  | rs11190368 | -0.018 | 0.453 | 0.019 | 0.348 | 0.000 | 31.466 |
|  | rs113595266 | 0.001 | 0.147 | 0.027 | 0.965 | 0.000 | 36.428 |
|  | rs113827118 | 0.022 | 0.194 | 0.024 | 0.377 | 0.000 | 71.157 |
|  | rs11614178 | -0.005 | 0.342 | 0.020 | 0.825 | 0.000 | 39.329 |
|  | rs11745587 | -0.015 | 0.402 | 0.020 | 0.437 | 0.000 | 45.225 |
|  | rs12132298 | -0.027 | 0.209 | 0.024 | 0.262 | 0.000 | 39.640 |
|  | rs12518457 | 0.010 | 0.285 | 0.021 | 0.643 | 0.000 | 30.509 |
|  | rs12536069 | -0.043 | 0.070 | 0.038 | 0.256 | 0.000 | 49.953 |
|  | rs12736494 | -0.009 | 0.255 | 0.022 | 0.695 | 0.000 | 34.777 |
|  | rs12942330 | 0.016 | 0.522 | 0.019 | 0.407 | 0.000 | 47.274 |
|  | rs148170789 | 0.054 | 0.047 | 0.044 | 0.226 | 0.000 | 56.054 |
|  | rs1801274 | 0.017 | 0.496 | 0.019 | 0.383 | 0.000 | 77.438 |
|  | rs181316459 | -0.005 | 0.051 | 0.045 | 0.918 | 0.001 | 266.342 |
|  | rs1990760 | -0.022 | 0.580 | 0.019 | 0.258 | 0.000 | 31.861 |
|  | rs2836883 | 0.009 | 0.240 | 0.023 | 0.697 | 0.000 | 51.168 |
|  | rs3024495 | 0.003 | 0.161 | 0.026 | 0.901 | 0.000 | 121.946 |
|  | rs3197999 | -0.015 | 0.381 | 0.019 | 0.443 | 0.000 | 106.975 |
|  | rs34145423 | -0.004 | 0.076 | 0.037 | 0.903 | 0.000 | 31.831 |
|  | rs34236350 | -0.009 | 0.254 | 0.022 | 0.689 | 0.000 | 81.068 |
|  | rs3732179 | 0.034 | 0.687 | 0.021 | 0.093 | 0.000 | 30.902 |
|  | rs4587163 | 0.040 | 0.270 | 0.022 | 0.065 | 0.000 | 144.846 |
|  | rs4655159 | -0.005 | 0.633 | 0.020 | 0.788 | 0.000 | 31.347 |
|  | rs4730276 | -0.021 | 0.441 | 0.019 | 0.271 | 0.000 | 68.499 |
|  | rs6089926 | 0.012 | 0.206 | 0.024 | 0.607 | 0.000 | 44.287 |
|  | rs6674040 | -0.008 | 0.468 | 0.020 | 0.701 | 0.000 | 96.537 |
|  | rs7101270 | 0.004 | 0.243 | 0.022 | 0.859 | 0.000 | 31.065 |
|  | rs7761376 | 0.031 | 0.616 | 0.020 | 0.115 | 0.000 | 30.777 |
|  | rs7865719 | -0.025 | 0.552 | 0.019 | 0.194 | 0.000 | 35.166 |
|  | rs79973254 | 0.028 | 0.066 | 0.039 | 0.471 | 0.000 | 30.306 |
|  | rs9607629 | 0.003 | 0.119 | 0.030 | 0.919 | 0.000 | 44.168 |
|  | rs9617090 | -0.009 | 0.363 | 0.020 | 0.640 | 0.000 | 64.731 |
| Phosphatidylcholine (16:0_20:2) | rs1042131 | 0.004 | 0.494 | 0.017 | 0.788 | 0.000 | 56.562 |
|  | rs1061537 | 0.018 | 0.388 | 0.017 | 0.295 | 0.000 | 59.252 |
|  | rs10748781 | 0.021 | 0.654 | 0.018 | 0.242 | 0.000 | 77.102 |
|  | rs10761659 | -0.008 | 0.524 | 0.017 | 0.631 | 0.000 | 35.706 |
|  | rs10807943 | 0.013 | 0.934 | 0.034 | 0.709 | 0.000 | 87.734 |
|  | rs10931828 | 0.028 | 0.437 | 0.017 | 0.100 | 0.000 | 33.803 |
|  | rs111432088 | 0.054 | 0.017 | 0.065 | 0.404 | 0.000 | 55.020 |
|  | rs11190368 | -0.022 | 0.453 | 0.017 | 0.197 | 0.000 | 31.466 |
|  | rs113595266 | -0.009 | 0.147 | 0.024 | 0.714 | 0.000 | 36.428 |
|  | rs113827118 | 0.013 | 0.194 | 0.021 | 0.554 | 0.000 | 71.157 |
|  | rs11614178 | 0.009 | 0.342 | 0.018 | 0.620 | 0.000 | 39.329 |
|  | rs11745587 | 0.004 | 0.402 | 0.017 | 0.805 | 0.000 | 45.225 |
|  | rs12132298 | -0.028 | 0.209 | 0.021 | 0.174 | 0.000 | 39.640 |
|  | rs12518457 | 0.009 | 0.285 | 0.019 | 0.622 | 0.000 | 30.509 |
|  | rs12536069 | 0.017 | 0.070 | 0.033 | 0.610 | 0.000 | 49.953 |
|  | rs12736494 | 0.004 | 0.255 | 0.019 | 0.850 | 0.000 | 34.777 |
|  | rs12942330 | 0.030 | 0.522 | 0.017 | 0.071 | 0.000 | 47.274 |
|  | rs148170789 | -0.011 | 0.047 | 0.039 | 0.770 | 0.000 | 56.054 |
|  | rs1801274 | 0.005 | 0.496 | 0.017 | 0.771 | 0.000 | 77.438 |
|  | rs181316459 | 0.003 | 0.051 | 0.040 | 0.945 | 0.001 | 266.342 |
|  | rs1990760 | -0.031 | 0.580 | 0.017 | 0.069 | 0.000 | 31.861 |
|  | rs2836883 | -0.002 | 0.240 | 0.020 | 0.934 | 0.000 | 51.168 |
|  | rs3024495 | -0.033 | 0.161 | 0.023 | 0.149 | 0.000 | 121.946 |
|  | rs3197999 | -0.019 | 0.381 | 0.017 | 0.279 | 0.000 | 106.975 |
|  | rs34145423 | 0.044 | 0.076 | 0.032 | 0.172 | 0.000 | 31.831 |
|  | rs34236350 | -0.008 | 0.254 | 0.019 | 0.692 | 0.000 | 81.068 |
|  | rs3732179 | 0.006 | 0.687 | 0.018 | 0.741 | 0.000 | 30.902 |
|  | rs4587163 | 0.030 | 0.270 | 0.019 | 0.111 | 0.000 | 144.846 |
|  | rs4655159 | -0.016 | 0.633 | 0.018 | 0.359 | 0.000 | 31.347 |
|  | rs4730276 | 0.007 | 0.441 | 0.017 | 0.676 | 0.000 | 68.499 |
|  | rs6089926 | 0.010 | 0.206 | 0.021 | 0.630 | 0.000 | 44.287 |
|  | rs6674040 | 0.022 | 0.468 | 0.017 | 0.206 | 0.000 | 96.537 |
|  | rs7101270 | -0.031 | 0.243 | 0.020 | 0.118 | 0.000 | 31.065 |
|  | rs7761376 | 0.011 | 0.616 | 0.017 | 0.543 | 0.000 | 30.777 |
|  | rs7865719 | 0.013 | 0.552 | 0.017 | 0.430 | 0.000 | 35.166 |
|  | rs79973254 | 0.067 | 0.066 | 0.034 | 0.048 | 0.000 | 30.306 |
|  | rs9607629 | -0.030 | 0.119 | 0.026 | 0.253 | 0.000 | 44.168 |
|  | rs9617090 | -0.003 | 0.363 | 0.017 | 0.876 | 0.000 | 64.731 |
| Phosphatidylcholine (16:0_20:4) | rs1042131 | 0.009 | 0.494 | 0.017 | 0.575 | 0.000 | 56.562 |
|  | rs1061537 | 0.006 | 0.388 | 0.017 | 0.740 | 0.000 | 59.252 |
|  | rs10748781 | 0.010 | 0.654 | 0.018 | 0.561 | 0.000 | 77.102 |
|  | rs10761659 | 0.006 | 0.524 | 0.017 | 0.715 | 0.000 | 35.706 |
|  | rs10807943 | 0.050 | 0.934 | 0.034 | 0.137 | 0.000 | 87.734 |
|  | rs10931828 | 0.021 | 0.437 | 0.017 | 0.208 | 0.000 | 33.803 |
|  | rs111432088 | -0.040 | 0.017 | 0.064 | 0.539 | 0.000 | 55.020 |
|  | rs11190368 | -0.003 | 0.453 | 0.017 | 0.860 | 0.000 | 31.466 |
|  | rs113595266 | 0.004 | 0.147 | 0.024 | 0.872 | 0.000 | 36.428 |
|  | rs113827118 | -0.034 | 0.194 | 0.021 | 0.106 | 0.000 | 71.157 |
|  | rs11614178 | 0.037 | 0.342 | 0.018 | 0.034 | 0.000 | 39.329 |
|  | rs11745587 | -0.032 | 0.402 | 0.017 | 0.062 | 0.000 | 45.225 |
|  | rs12132298 | -0.002 | 0.209 | 0.021 | 0.929 | 0.000 | 39.640 |
|  | rs12518457 | 0.004 | 0.285 | 0.018 | 0.825 | 0.000 | 30.509 |
|  | rs12536069 | -0.020 | 0.070 | 0.033 | 0.551 | 0.000 | 49.953 |
|  | rs12736494 | -0.010 | 0.255 | 0.019 | 0.595 | 0.000 | 34.777 |
|  | rs12942330 | 0.024 | 0.522 | 0.017 | 0.145 | 0.000 | 47.274 |
|  | rs148170789 | 0.047 | 0.047 | 0.039 | 0.225 | 0.000 | 56.054 |
|  | rs1801274 | -0.015 | 0.496 | 0.017 | 0.373 | 0.000 | 77.438 |
|  | rs181316459 | -0.021 | 0.051 | 0.039 | 0.592 | 0.001 | 266.342 |
|  | rs1990760 | -0.034 | 0.580 | 0.017 | 0.042 | 0.000 | 31.861 |
|  | rs2836883 | 0.000 | 0.240 | 0.020 | 0.982 | 0.000 | 51.168 |
|  | rs3024495 | 0.003 | 0.161 | 0.023 | 0.885 | 0.000 | 121.946 |
|  | rs3197999 | 0.021 | 0.381 | 0.017 | 0.216 | 0.000 | 106.975 |
|  | rs34145423 | 0.078 | 0.076 | 0.032 | 0.014 | 0.000 | 31.831 |
|  | rs34236350 | 0.000 | 0.254 | 0.019 | 0.996 | 0.000 | 81.068 |
|  | rs3732179 | 0.006 | 0.687 | 0.018 | 0.721 | 0.000 | 30.902 |
|  | rs4587163 | 0.047 | 0.270 | 0.019 | 0.013 | 0.000 | 144.846 |
|  | rs4655159 | 0.019 | 0.633 | 0.018 | 0.288 | 0.000 | 31.347 |
|  | rs4730276 | -0.001 | 0.441 | 0.017 | 0.932 | 0.000 | 68.499 |
|  | rs6089926 | 0.003 | 0.206 | 0.021 | 0.875 | 0.000 | 44.287 |
|  | rs6674040 | 0.016 | 0.468 | 0.017 | 0.351 | 0.000 | 96.537 |
|  | rs7101270 | -0.016 | 0.243 | 0.019 | 0.407 | 0.000 | 31.065 |
|  | rs7761376 | 0.007 | 0.616 | 0.017 | 0.689 | 0.000 | 30.777 |
|  | rs7865719 | 0.009 | 0.552 | 0.017 | 0.586 | 0.000 | 35.166 |
|  | rs79973254 | 0.023 | 0.066 | 0.034 | 0.493 | 0.000 | 30.306 |
|  | rs9607629 | 0.006 | 0.119 | 0.026 | 0.807 | 0.000 | 44.168 |
|  | rs9617090 | 0.000 | 0.363 | 0.017 | 0.999 | 0.000 | 64.731 |
| Phosphatidylcholine (16:1_18:1) | rs1042131 | 0.020 | 0.494 | 0.017 | 0.230 | 0.000 | 56.562 |
|  | rs1061537 | 0.010 | 0.388 | 0.017 | 0.551 | 0.000 | 59.252 |
|  | rs10748781 | 0.040 | 0.654 | 0.018 | 0.024 | 0.000 | 77.102 |
|  | rs10761659 | 0.001 | 0.524 | 0.017 | 0.961 | 0.000 | 35.706 |
|  | rs10807943 | 0.003 | 0.934 | 0.034 | 0.930 | 0.000 | 87.734 |
|  | rs10931828 | 0.040 | 0.437 | 0.017 | 0.017 | 0.000 | 33.803 |
|  | rs111432088 | 0.051 | 0.017 | 0.065 | 0.430 | 0.000 | 55.020 |
|  | rs11190368 | -0.003 | 0.453 | 0.017 | 0.867 | 0.000 | 31.466 |
|  | rs113595266 | 0.012 | 0.147 | 0.024 | 0.606 | 0.000 | 36.428 |
|  | rs113827118 | 0.001 | 0.194 | 0.021 | 0.965 | 0.000 | 71.157 |
|  | rs11614178 | 0.036 | 0.342 | 0.018 | 0.046 | 0.000 | 39.329 |
|  | rs11745587 | 0.051 | 0.402 | 0.017 | 0.003 | 0.000 | 45.225 |
|  | rs12132298 | -0.014 | 0.209 | 0.021 | 0.507 | 0.000 | 39.640 |
|  | rs12518457 | 0.014 | 0.285 | 0.018 | 0.446 | 0.000 | 30.509 |
|  | rs12536069 | -0.035 | 0.070 | 0.033 | 0.294 | 0.000 | 49.953 |
|  | rs12736494 | 0.008 | 0.255 | 0.019 | 0.691 | 0.000 | 34.777 |
|  | rs12942330 | 0.046 | 0.522 | 0.017 | 0.006 | 0.000 | 47.274 |
|  | rs148170789 | -0.025 | 0.047 | 0.039 | 0.517 | 0.000 | 56.054 |
|  | rs1801274 | 0.028 | 0.496 | 0.017 | 0.096 | 0.000 | 77.438 |
|  | rs181316459 | -0.022 | 0.051 | 0.040 | 0.586 | 0.001 | 266.342 |
|  | rs1990760 | -0.016 | 0.580 | 0.017 | 0.341 | 0.000 | 31.861 |
|  | rs2836883 | -0.019 | 0.240 | 0.020 | 0.347 | 0.000 | 51.168 |
|  | rs3024495 | -0.051 | 0.161 | 0.023 | 0.026 | 0.000 | 121.946 |
|  | rs3197999 | -0.020 | 0.381 | 0.017 | 0.242 | 0.000 | 106.975 |
|  | rs34145423 | 0.047 | 0.076 | 0.032 | 0.141 | 0.000 | 31.831 |
|  | rs34236350 | 0.003 | 0.254 | 0.019 | 0.876 | 0.000 | 81.068 |
|  | rs3732179 | 0.009 | 0.687 | 0.018 | 0.611 | 0.000 | 30.902 |
|  | rs4587163 | 0.005 | 0.270 | 0.019 | 0.804 | 0.000 | 144.846 |
|  | rs4655159 | 0.008 | 0.633 | 0.018 | 0.640 | 0.000 | 31.347 |
|  | rs4730276 | 0.003 | 0.441 | 0.017 | 0.874 | 0.000 | 68.499 |
|  | rs6089926 | -0.016 | 0.206 | 0.021 | 0.444 | 0.000 | 44.287 |
|  | rs6674040 | 0.008 | 0.468 | 0.017 | 0.646 | 0.000 | 96.537 |
|  | rs7101270 | -0.015 | 0.243 | 0.020 | 0.444 | 0.000 | 31.065 |
|  | rs7761376 | 0.012 | 0.616 | 0.017 | 0.496 | 0.000 | 30.777 |
|  | rs7865719 | 0.009 | 0.552 | 0.017 | 0.572 | 0.000 | 35.166 |
|  | rs79973254 | 0.010 | 0.066 | 0.034 | 0.771 | 0.000 | 30.306 |
|  | rs9607629 | 0.011 | 0.119 | 0.026 | 0.686 | 0.000 | 44.168 |
|  | rs9617090 | 0.001 | 0.363 | 0.017 | 0.965 | 0.000 | 64.731 |
| Phosphatidylcholine (17:0_18:2) | rs1042131 | 0.011 | 0.494 | 0.017 | 0.515 | 0.000 | 56.562 |
|  | rs1061537 | 0.001 | 0.388 | 0.017 | 0.970 | 0.000 | 59.252 |
|  | rs10748781 | -0.001 | 0.654 | 0.018 | 0.960 | 0.000 | 77.102 |
|  | rs10761659 | -0.011 | 0.524 | 0.017 | 0.501 | 0.000 | 35.706 |
|  | rs10807943 | -0.017 | 0.934 | 0.034 | 0.610 | 0.000 | 87.734 |
|  | rs10931828 | 0.025 | 0.437 | 0.017 | 0.138 | 0.000 | 33.803 |
|  | rs111432088 | -0.006 | 0.017 | 0.065 | 0.930 | 0.000 | 55.020 |
|  | rs11190368 | 0.006 | 0.453 | 0.017 | 0.732 | 0.000 | 31.466 |
|  | rs113595266 | -0.011 | 0.147 | 0.024 | 0.637 | 0.000 | 36.428 |
|  | rs113827118 | 0.014 | 0.194 | 0.021 | 0.504 | 0.000 | 71.157 |
|  | rs11614178 | 0.002 | 0.342 | 0.018 | 0.909 | 0.000 | 39.329 |
|  | rs11745587 | 0.020 | 0.402 | 0.017 | 0.235 | 0.000 | 45.225 |
|  | rs12132298 | -0.009 | 0.209 | 0.021 | 0.680 | 0.000 | 39.640 |
|  | rs12518457 | 0.028 | 0.285 | 0.018 | 0.127 | 0.000 | 30.509 |
|  | rs12536069 | 0.012 | 0.070 | 0.033 | 0.730 | 0.000 | 49.953 |
|  | rs12736494 | -0.025 | 0.255 | 0.019 | 0.194 | 0.000 | 34.777 |
|  | rs12942330 | 0.029 | 0.522 | 0.017 | 0.078 | 0.000 | 47.274 |
|  | rs148170789 | -0.031 | 0.047 | 0.039 | 0.429 | 0.000 | 56.054 |
|  | rs1801274 | 0.003 | 0.496 | 0.017 | 0.866 | 0.000 | 77.438 |
|  | rs181316459 | -0.005 | 0.051 | 0.040 | 0.894 | 0.001 | 266.342 |
|  | rs1990760 | -0.039 | 0.580 | 0.017 | 0.022 | 0.000 | 31.861 |
|  | rs2836883 | -0.017 | 0.240 | 0.020 | 0.387 | 0.000 | 51.168 |
|  | rs3024495 | -0.019 | 0.161 | 0.023 | 0.413 | 0.000 | 121.946 |
|  | rs3197999 | -0.012 | 0.381 | 0.017 | 0.479 | 0.000 | 106.975 |
|  | rs34145423 | 0.025 | 0.076 | 0.032 | 0.428 | 0.000 | 31.831 |
|  | rs34236350 | 0.012 | 0.254 | 0.019 | 0.516 | 0.000 | 81.068 |
|  | rs3732179 | -0.018 | 0.687 | 0.018 | 0.315 | 0.000 | 30.902 |
|  | rs4587163 | 0.027 | 0.270 | 0.019 | 0.160 | 0.000 | 144.846 |
|  | rs4655159 | -0.011 | 0.633 | 0.018 | 0.521 | 0.000 | 31.347 |
|  | rs4730276 | 0.010 | 0.441 | 0.017 | 0.566 | 0.000 | 68.499 |
|  | rs6089926 | -0.024 | 0.206 | 0.021 | 0.253 | 0.000 | 44.287 |
|  | rs6674040 | 0.031 | 0.468 | 0.017 | 0.070 | 0.000 | 96.537 |
|  | rs7101270 | 0.000 | 0.243 | 0.019 | 0.994 | 0.000 | 31.065 |
|  | rs7761376 | 0.012 | 0.616 | 0.017 | 0.475 | 0.000 | 30.777 |
|  | rs7865719 | 0.008 | 0.552 | 0.017 | 0.637 | 0.000 | 35.166 |
|  | rs79973254 | 0.005 | 0.066 | 0.034 | 0.880 | 0.000 | 30.306 |
|  | rs9607629 | 0.007 | 0.119 | 0.026 | 0.795 | 0.000 | 44.168 |
|  | rs9617090 | -0.003 | 0.363 | 0.017 | 0.847 | 0.000 | 64.731 |
| Phosphatidylcholine (18:0_20:4) | rs1042131 | 0.019 | 0.494 | 0.017 | 0.239 | 0.000 | 56.562 |
|  | rs1061537 | -0.005 | 0.388 | 0.017 | 0.785 | 0.000 | 59.252 |
|  | rs10748781 | 0.013 | 0.654 | 0.017 | 0.456 | 0.000 | 77.102 |
|  | rs10761659 | 0.006 | 0.524 | 0.016 | 0.703 | 0.000 | 35.706 |
|  | rs10807943 | 0.050 | 0.934 | 0.034 | 0.140 | 0.000 | 87.734 |
|  | rs10931828 | 0.002 | 0.437 | 0.017 | 0.885 | 0.000 | 33.803 |
|  | rs111432088 | -0.042 | 0.017 | 0.064 | 0.512 | 0.000 | 55.020 |
|  | rs11190368 | 0.013 | 0.453 | 0.017 | 0.436 | 0.000 | 31.466 |
|  | rs113595266 | -0.007 | 0.147 | 0.023 | 0.754 | 0.000 | 36.428 |
|  | rs113827118 | -0.026 | 0.194 | 0.021 | 0.216 | 0.000 | 71.157 |
|  | rs11614178 | 0.037 | 0.342 | 0.018 | 0.034 | 0.000 | 39.329 |
|  | rs11745587 | -0.020 | 0.402 | 0.017 | 0.241 | 0.000 | 45.225 |
|  | rs12132298 | 0.007 | 0.209 | 0.020 | 0.735 | 0.000 | 39.640 |
|  | rs12518457 | 0.005 | 0.285 | 0.018 | 0.780 | 0.000 | 30.509 |
|  | rs12536069 | -0.046 | 0.070 | 0.033 | 0.161 | 0.000 | 49.953 |
|  | rs12736494 | -0.014 | 0.255 | 0.019 | 0.472 | 0.000 | 34.777 |
|  | rs12942330 | 0.029 | 0.522 | 0.017 | 0.081 | 0.000 | 47.274 |
|  | rs148170789 | 0.058 | 0.047 | 0.038 | 0.132 | 0.000 | 56.054 |
|  | rs1801274 | -0.011 | 0.496 | 0.017 | 0.522 | 0.000 | 77.438 |
|  | rs181316459 | -0.030 | 0.051 | 0.039 | 0.449 | 0.001 | 266.342 |
|  | rs1990760 | -0.035 | 0.580 | 0.017 | 0.036 | 0.000 | 31.861 |
|  | rs2836883 | -0.010 | 0.240 | 0.020 | 0.599 | 0.000 | 51.168 |
|  | rs3024495 | -0.011 | 0.161 | 0.023 | 0.624 | 0.000 | 121.946 |
|  | rs3197999 | 0.005 | 0.381 | 0.017 | 0.752 | 0.000 | 106.975 |
|  | rs34145423 | 0.070 | 0.076 | 0.032 | 0.026 | 0.000 | 31.831 |
|  | rs34236350 | 0.009 | 0.254 | 0.019 | 0.634 | 0.000 | 81.068 |
|  | rs3732179 | 0.003 | 0.687 | 0.018 | 0.861 | 0.000 | 30.902 |
|  | rs4587163 | 0.051 | 0.270 | 0.019 | 0.007 | 0.000 | 144.846 |
|  | rs4655159 | 0.004 | 0.633 | 0.017 | 0.799 | 0.000 | 31.347 |
|  | rs4730276 | 0.003 | 0.441 | 0.017 | 0.876 | 0.000 | 68.499 |
|  | rs6089926 | -0.007 | 0.206 | 0.021 | 0.733 | 0.000 | 44.287 |
|  | rs6674040 | 0.014 | 0.468 | 0.017 | 0.423 | 0.000 | 96.537 |
|  | rs7101270 | -0.007 | 0.243 | 0.019 | 0.709 | 0.000 | 31.065 |
|  | rs7761376 | 0.014 | 0.616 | 0.017 | 0.404 | 0.000 | 30.777 |
|  | rs7865719 | -0.004 | 0.552 | 0.016 | 0.816 | 0.000 | 35.166 |
|  | rs79973254 | 0.037 | 0.066 | 0.034 | 0.269 | 0.000 | 30.306 |
|  | rs9607629 | 0.016 | 0.119 | 0.026 | 0.549 | 0.000 | 44.168 |
|  | rs9617090 | -0.009 | 0.363 | 0.017 | 0.595 | 0.000 | 64.731 |
| Phosphatidylcholine (18:1_20:4) | rs1042131 | 0.013 | 0.494 | 0.017 | 0.446 | 0.000 | 56.562 |
|  | rs1061537 | 0.006 | 0.388 | 0.017 | 0.712 | 0.000 | 59.252 |
|  | rs10748781 | 0.024 | 0.654 | 0.018 | 0.175 | 0.000 | 77.102 |
|  | rs10761659 | 0.022 | 0.524 | 0.017 | 0.191 | 0.000 | 35.706 |
|  | rs10807943 | 0.030 | 0.934 | 0.034 | 0.378 | 0.000 | 87.734 |
|  | rs10931828 | 0.018 | 0.437 | 0.017 | 0.273 | 0.000 | 33.803 |
|  | rs111432088 | -0.088 | 0.017 | 0.064 | 0.172 | 0.000 | 55.020 |
|  | rs11190368 | 0.015 | 0.453 | 0.017 | 0.366 | 0.000 | 31.466 |
|  | rs113595266 | -0.002 | 0.147 | 0.024 | 0.920 | 0.000 | 36.428 |
|  | rs113827118 | -0.029 | 0.194 | 0.021 | 0.177 | 0.000 | 71.157 |
|  | rs11614178 | 0.043 | 0.342 | 0.018 | 0.015 | 0.000 | 39.329 |
|  | rs11745587 | -0.001 | 0.402 | 0.017 | 0.973 | 0.000 | 45.225 |
|  | rs12132298 | 0.003 | 0.209 | 0.021 | 0.892 | 0.000 | 39.640 |
|  | rs12518457 | 0.013 | 0.285 | 0.018 | 0.489 | 0.000 | 30.509 |
|  | rs12536069 | -0.057 | 0.070 | 0.033 | 0.085 | 0.000 | 49.953 |
|  | rs12736494 | 0.004 | 0.255 | 0.019 | 0.838 | 0.000 | 34.777 |
|  | rs12942330 | 0.047 | 0.522 | 0.017 | 0.005 | 0.000 | 47.274 |
|  | rs148170789 | 0.029 | 0.047 | 0.039 | 0.460 | 0.000 | 56.054 |
|  | rs1801274 | 0.003 | 0.496 | 0.017 | 0.843 | 0.000 | 77.438 |
|  | rs181316459 | -0.033 | 0.051 | 0.040 | 0.406 | 0.001 | 266.342 |
|  | rs1990760 | -0.045 | 0.580 | 0.017 | 0.007 | 0.000 | 31.861 |
|  | rs2836883 | -0.014 | 0.240 | 0.020 | 0.475 | 0.000 | 51.168 |
|  | rs3024495 | -0.009 | 0.161 | 0.023 | 0.685 | 0.000 | 121.946 |
|  | rs3197999 | -0.010 | 0.381 | 0.017 | 0.546 | 0.000 | 106.975 |
|  | rs34145423 | 0.073 | 0.076 | 0.032 | 0.021 | 0.000 | 31.831 |
|  | rs34236350 | -0.004 | 0.254 | 0.019 | 0.848 | 0.000 | 81.068 |
|  | rs3732179 | -0.001 | 0.687 | 0.018 | 0.946 | 0.000 | 30.902 |
|  | rs4587163 | 0.059 | 0.270 | 0.019 | 0.002 | 0.000 | 144.846 |
|  | rs4655159 | -0.003 | 0.633 | 0.018 | 0.845 | 0.000 | 31.347 |
|  | rs4730276 | 0.000 | 0.441 | 0.017 | 0.988 | 0.000 | 68.499 |
|  | rs6089926 | -0.003 | 0.206 | 0.021 | 0.892 | 0.000 | 44.287 |
|  | rs6674040 | 0.010 | 0.468 | 0.017 | 0.542 | 0.000 | 96.537 |
|  | rs7101270 | -0.016 | 0.243 | 0.019 | 0.421 | 0.000 | 31.065 |
|  | rs7761376 | 0.015 | 0.616 | 0.017 | 0.384 | 0.000 | 30.777 |
|  | rs7865719 | 0.001 | 0.552 | 0.017 | 0.960 | 0.000 | 35.166 |
|  | rs79973254 | -0.002 | 0.066 | 0.034 | 0.954 | 0.000 | 30.306 |
|  | rs9607629 | 0.029 | 0.119 | 0.026 | 0.268 | 0.000 | 44.168 |
|  | rs9617090 | -0.005 | 0.363 | 0.017 | 0.761 | 0.000 | 64.731 |
| Phosphatidylcholine (O-16:1_20:4) | rs1042131 | 0.020 | 0.494 | 0.018 | 0.275 | 0.000 | 56.562 |
|  | rs1061537 | 0.036 | 0.388 | 0.018 | 0.050 | 0.000 | 59.252 |
|  | rs10748781 | 0.006 | 0.654 | 0.019 | 0.738 | 0.000 | 77.102 |
|  | rs10761659 | 0.012 | 0.524 | 0.018 | 0.511 | 0.000 | 35.706 |
|  | rs10807943 | 0.050 | 0.934 | 0.037 | 0.175 | 0.000 | 87.734 |
|  | rs10931828 | -0.004 | 0.437 | 0.018 | 0.847 | 0.000 | 33.803 |
|  | rs111432088 | -0.080 | 0.017 | 0.071 | 0.258 | 0.000 | 55.020 |
|  | rs11190368 | 0.033 | 0.453 | 0.018 | 0.066 | 0.000 | 31.466 |
|  | rs113595266 | 0.012 | 0.147 | 0.026 | 0.636 | 0.000 | 36.428 |
|  | rs113827118 | -0.041 | 0.194 | 0.023 | 0.076 | 0.000 | 71.157 |
|  | rs11614178 | 0.002 | 0.342 | 0.019 | 0.899 | 0.000 | 39.329 |
|  | rs11745587 | -0.008 | 0.402 | 0.019 | 0.665 | 0.000 | 45.225 |
|  | rs12132298 | 0.024 | 0.209 | 0.022 | 0.284 | 0.000 | 39.640 |
|  | rs12518457 | -0.002 | 0.285 | 0.020 | 0.903 | 0.000 | 30.509 |
|  | rs12536069 | -0.024 | 0.070 | 0.036 | 0.504 | 0.000 | 49.953 |
|  | rs12736494 | 0.006 | 0.255 | 0.021 | 0.754 | 0.000 | 34.777 |
|  | rs12942330 | 0.005 | 0.522 | 0.018 | 0.788 | 0.000 | 47.274 |
|  | rs148170789 | 0.002 | 0.047 | 0.042 | 0.970 | 0.000 | 56.054 |
|  | rs1801274 | -0.015 | 0.496 | 0.018 | 0.406 | 0.000 | 77.438 |
|  | rs181316459 | -0.067 | 0.051 | 0.043 | 0.120 | 0.001 | 266.342 |
|  | rs1990760 | -0.033 | 0.580 | 0.018 | 0.076 | 0.000 | 31.861 |
|  | rs2836883 | -0.002 | 0.240 | 0.022 | 0.912 | 0.000 | 51.168 |
|  | rs3024495 | -0.005 | 0.161 | 0.025 | 0.838 | 0.000 | 121.946 |
|  | rs3197999 | -0.003 | 0.381 | 0.019 | 0.886 | 0.000 | 106.975 |
|  | rs34145423 | 0.085 | 0.076 | 0.035 | 0.014 | 0.000 | 31.831 |
|  | rs34236350 | 0.013 | 0.254 | 0.021 | 0.526 | 0.000 | 81.068 |
|  | rs3732179 | 0.035 | 0.687 | 0.020 | 0.074 | 0.000 | 30.902 |
|  | rs4587163 | 0.030 | 0.270 | 0.021 | 0.143 | 0.000 | 144.846 |
|  | rs4655159 | -0.020 | 0.633 | 0.019 | 0.299 | 0.000 | 31.347 |
|  | rs4730276 | 0.004 | 0.441 | 0.018 | 0.840 | 0.000 | 68.499 |
|  | rs6089926 | -0.021 | 0.206 | 0.023 | 0.352 | 0.000 | 44.287 |
|  | rs6674040 | 0.003 | 0.468 | 0.019 | 0.888 | 0.000 | 96.537 |
|  | rs7101270 | -0.014 | 0.243 | 0.021 | 0.500 | 0.000 | 31.065 |
|  | rs7761376 | 0.027 | 0.616 | 0.019 | 0.149 | 0.000 | 30.777 |
|  | rs7865719 | -0.006 | 0.552 | 0.018 | 0.724 | 0.000 | 35.166 |
|  | rs79973254 | -0.043 | 0.066 | 0.037 | 0.247 | 0.000 | 30.306 |
|  | rs9607629 | 0.010 | 0.119 | 0.029 | 0.720 | 0.000 | 44.168 |
|  | rs9617090 | 0.014 | 0.363 | 0.019 | 0.444 | 0.000 | 64.731 |
| Phosphatidylcholine (O-18:1_18:2) | rs1042131 | 0.017 | 0.494 | 0.017 | 0.312 | 0.000 | 56.562 |
|  | rs1061537 | 0.030 | 0.388 | 0.018 | 0.092 | 0.000 | 59.252 |
|  | rs10748781 | -0.017 | 0.654 | 0.018 | 0.339 | 0.000 | 77.102 |
|  | rs10761659 | -0.032 | 0.524 | 0.017 | 0.063 | 0.000 | 35.706 |
|  | rs10807943 | -0.020 | 0.934 | 0.035 | 0.578 | 0.000 | 87.734 |
|  | rs10931828 | 0.011 | 0.437 | 0.017 | 0.510 | 0.000 | 33.803 |
|  | rs111432088 | -0.124 | 0.017 | 0.067 | 0.063 | 0.000 | 55.020 |
|  | rs11190368 | -0.013 | 0.453 | 0.017 | 0.452 | 0.000 | 31.466 |
|  | rs113595266 | -0.038 | 0.147 | 0.024 | 0.119 | 0.000 | 36.428 |
|  | rs113827118 | -0.020 | 0.194 | 0.022 | 0.365 | 0.000 | 71.157 |
|  | rs11614178 | -0.007 | 0.342 | 0.018 | 0.696 | 0.000 | 39.329 |
|  | rs11745587 | 0.012 | 0.402 | 0.018 | 0.479 | 0.000 | 45.225 |
|  | rs12132298 | 0.019 | 0.209 | 0.021 | 0.380 | 0.000 | 39.640 |
|  | rs12518457 | 0.002 | 0.285 | 0.019 | 0.907 | 0.000 | 30.509 |
|  | rs12536069 | -0.040 | 0.070 | 0.034 | 0.245 | 0.000 | 49.953 |
|  | rs12736494 | -0.002 | 0.255 | 0.020 | 0.930 | 0.000 | 34.777 |
|  | rs12942330 | 0.003 | 0.522 | 0.017 | 0.877 | 0.000 | 47.274 |
|  | rs148170789 | 0.001 | 0.047 | 0.040 | 0.974 | 0.000 | 56.054 |
|  | rs1801274 | 0.016 | 0.496 | 0.018 | 0.361 | 0.000 | 77.438 |
|  | rs181316459 | -0.074 | 0.051 | 0.041 | 0.072 | 0.001 | 266.342 |
|  | rs1990760 | -0.027 | 0.580 | 0.017 | 0.126 | 0.000 | 31.861 |
|  | rs2836883 | -0.030 | 0.240 | 0.021 | 0.147 | 0.000 | 51.168 |
|  | rs3024495 | -0.028 | 0.161 | 0.024 | 0.229 | 0.000 | 121.946 |
|  | rs3197999 | -0.034 | 0.381 | 0.018 | 0.051 | 0.000 | 106.975 |
|  | rs34145423 | 0.018 | 0.076 | 0.033 | 0.595 | 0.000 | 31.831 |
|  | rs34236350 | 0.015 | 0.254 | 0.020 | 0.445 | 0.000 | 81.068 |
|  | rs3732179 | 0.002 | 0.687 | 0.019 | 0.913 | 0.000 | 30.902 |
|  | rs4587163 | -0.008 | 0.270 | 0.020 | 0.672 | 0.000 | 144.846 |
|  | rs4655159 | -0.015 | 0.633 | 0.018 | 0.403 | 0.000 | 31.347 |
|  | rs4730276 | 0.015 | 0.441 | 0.017 | 0.400 | 0.000 | 68.499 |
|  | rs6089926 | 0.009 | 0.206 | 0.021 | 0.658 | 0.000 | 44.287 |
|  | rs6674040 | 0.007 | 0.468 | 0.018 | 0.695 | 0.000 | 96.537 |
|  | rs7101270 | 0.021 | 0.243 | 0.020 | 0.304 | 0.000 | 31.065 |
|  | rs7761376 | 0.026 | 0.616 | 0.018 | 0.141 | 0.000 | 30.777 |
|  | rs7865719 | 0.012 | 0.552 | 0.017 | 0.495 | 0.000 | 35.166 |
|  | rs79973254 | -0.043 | 0.066 | 0.035 | 0.220 | 0.000 | 30.306 |
|  | rs9607629 | -0.009 | 0.119 | 0.027 | 0.752 | 0.000 | 44.168 |
|  | rs9617090 | -0.006 | 0.363 | 0.018 | 0.743 | 0.000 | 64.731 |
| Phosphatidylethanolamine (18:0_18:2) | rs1042131 | -0.008 | 0.494 | 0.017 | 0.637 | 0.000 | 56.562 |
|  | rs1061537 | 0.006 | 0.388 | 0.017 | 0.706 | 0.000 | 59.252 |
|  | rs10748781 | 0.039 | 0.654 | 0.018 | 0.027 | 0.000 | 77.102 |
|  | rs10761659 | 0.017 | 0.524 | 0.017 | 0.320 | 0.000 | 35.706 |
|  | rs10807943 | 0.008 | 0.934 | 0.034 | 0.820 | 0.000 | 87.734 |
|  | rs10931828 | 0.015 | 0.437 | 0.017 | 0.371 | 0.000 | 33.803 |
|  | rs111432088 | 0.010 | 0.017 | 0.065 | 0.882 | 0.000 | 55.020 |
|  | rs11190368 | -0.010 | 0.453 | 0.017 | 0.531 | 0.000 | 31.466 |
|  | rs113595266 | 0.004 | 0.147 | 0.024 | 0.856 | 0.000 | 36.428 |
|  | rs113827118 | -0.012 | 0.194 | 0.021 | 0.588 | 0.000 | 71.157 |
|  | rs11614178 | -0.006 | 0.342 | 0.018 | 0.748 | 0.000 | 39.329 |
|  | rs11745587 | -0.006 | 0.402 | 0.017 | 0.742 | 0.000 | 45.225 |
|  | rs12132298 | -0.030 | 0.209 | 0.021 | 0.149 | 0.000 | 39.640 |
|  | rs12518457 | 0.032 | 0.285 | 0.018 | 0.085 | 0.000 | 30.509 |
|  | rs12536069 | -0.028 | 0.070 | 0.033 | 0.394 | 0.000 | 49.953 |
|  | rs12736494 | -0.019 | 0.255 | 0.019 | 0.323 | 0.000 | 34.777 |
|  | rs12942330 | 0.040 | 0.522 | 0.017 | 0.016 | 0.000 | 47.274 |
|  | rs148170789 | 0.031 | 0.047 | 0.039 | 0.424 | 0.000 | 56.054 |
|  | rs1801274 | 0.011 | 0.496 | 0.017 | 0.512 | 0.000 | 77.438 |
|  | rs181316459 | -0.056 | 0.051 | 0.039 | 0.159 | 0.001 | 266.342 |
|  | rs1990760 | -0.023 | 0.580 | 0.017 | 0.176 | 0.000 | 31.861 |
|  | rs2836883 | 0.020 | 0.240 | 0.020 | 0.320 | 0.000 | 51.168 |
|  | rs3024495 | 0.016 | 0.161 | 0.023 | 0.475 | 0.000 | 121.946 |
|  | rs3197999 | 0.000 | 0.381 | 0.017 | 0.999 | 0.000 | 106.975 |
|  | rs34145423 | 0.027 | 0.076 | 0.032 | 0.391 | 0.000 | 31.831 |
|  | rs34236350 | -0.013 | 0.254 | 0.019 | 0.493 | 0.000 | 81.068 |
|  | rs3732179 | -0.013 | 0.687 | 0.018 | 0.475 | 0.000 | 30.902 |
|  | rs4587163 | 0.004 | 0.270 | 0.019 | 0.818 | 0.000 | 144.846 |
|  | rs4655159 | -0.018 | 0.633 | 0.018 | 0.295 | 0.000 | 31.347 |
|  | rs4730276 | 0.004 | 0.441 | 0.017 | 0.803 | 0.000 | 68.499 |
|  | rs6089926 | -0.015 | 0.206 | 0.021 | 0.474 | 0.000 | 44.287 |
|  | rs6674040 | 0.000 | 0.468 | 0.017 | 0.981 | 0.000 | 96.537 |
|  | rs7101270 | -0.040 | 0.243 | 0.019 | 0.038 | 0.000 | 31.065 |
|  | rs7761376 | 0.008 | 0.616 | 0.017 | 0.646 | 0.000 | 30.777 |
|  | rs7865719 | 0.002 | 0.552 | 0.017 | 0.900 | 0.000 | 35.166 |
|  | rs79973254 | 0.090 | 0.066 | 0.034 | 0.008 | 0.000 | 30.306 |
|  | rs9607629 | -0.012 | 0.119 | 0.026 | 0.658 | 0.000 | 44.168 |
|  | rs9617090 | 0.014 | 0.363 | 0.017 | 0.422 | 0.000 | 64.731 |
| Sphingomyelin (d36:1) | rs1042131 | 0.023 | 0.494 | 0.017 | 0.159 | 0.000 | 56.562 |
|  | rs1061537 | 0.001 | 0.388 | 0.017 | 0.939 | 0.000 | 59.252 |
|  | rs10748781 | -0.025 | 0.654 | 0.018 | 0.152 | 0.000 | 77.102 |
|  | rs10761659 | -0.005 | 0.524 | 0.017 | 0.785 | 0.000 | 35.706 |
|  | rs10807943 | -0.001 | 0.934 | 0.034 | 0.975 | 0.000 | 87.734 |
|  | rs10931828 | 0.001 | 0.437 | 0.017 | 0.939 | 0.000 | 33.803 |
|  | rs111432088 | 0.055 | 0.017 | 0.065 | 0.393 | 0.000 | 55.020 |
|  | rs11190368 | 0.005 | 0.453 | 0.017 | 0.781 | 0.000 | 31.466 |
|  | rs113595266 | 0.053 | 0.147 | 0.024 | 0.025 | 0.000 | 36.428 |
|  | rs113827118 | 0.000 | 0.194 | 0.021 | 0.997 | 0.000 | 71.157 |
|  | rs11614178 | -0.001 | 0.342 | 0.018 | 0.941 | 0.000 | 39.329 |
|  | rs11745587 | 0.031 | 0.402 | 0.017 | 0.065 | 0.000 | 45.225 |
|  | rs12132298 | 0.015 | 0.209 | 0.021 | 0.471 | 0.000 | 39.640 |
|  | rs12518457 | 0.012 | 0.285 | 0.018 | 0.522 | 0.000 | 30.509 |
|  | rs12536069 | 0.017 | 0.070 | 0.033 | 0.612 | 0.000 | 49.953 |
|  | rs12736494 | 0.011 | 0.255 | 0.019 | 0.566 | 0.000 | 34.777 |
|  | rs12942330 | -0.001 | 0.522 | 0.017 | 0.962 | 0.000 | 47.274 |
|  | rs148170789 | 0.021 | 0.047 | 0.039 | 0.590 | 0.000 | 56.054 |
|  | rs1801274 | 0.002 | 0.496 | 0.017 | 0.918 | 0.000 | 77.438 |
|  | rs181316459 | -0.015 | 0.051 | 0.040 | 0.702 | 0.001 | 266.342 |
|  | rs1990760 | -0.010 | 0.580 | 0.017 | 0.537 | 0.000 | 31.861 |
|  | rs2836883 | -0.027 | 0.240 | 0.020 | 0.183 | 0.000 | 51.168 |
|  | rs3024495 | -0.025 | 0.161 | 0.023 | 0.272 | 0.000 | 121.946 |
|  | rs3197999 | 0.010 | 0.381 | 0.017 | 0.568 | 0.000 | 106.975 |
|  | rs34145423 | 0.060 | 0.076 | 0.032 | 0.062 | 0.000 | 31.831 |
|  | rs34236350 | -0.002 | 0.254 | 0.019 | 0.922 | 0.000 | 81.068 |
|  | rs3732179 | 0.003 | 0.687 | 0.018 | 0.872 | 0.000 | 30.902 |
|  | rs4587163 | 0.030 | 0.270 | 0.019 | 0.110 | 0.000 | 144.846 |
|  | rs4655159 | 0.011 | 0.633 | 0.018 | 0.530 | 0.000 | 31.347 |
|  | rs4730276 | 0.015 | 0.441 | 0.017 | 0.371 | 0.000 | 68.499 |
|  | rs6089926 | 0.017 | 0.206 | 0.021 | 0.413 | 0.000 | 44.287 |
|  | rs6674040 | 0.019 | 0.468 | 0.017 | 0.265 | 0.000 | 96.537 |
|  | rs7101270 | 0.010 | 0.243 | 0.019 | 0.594 | 0.000 | 31.065 |
|  | rs7761376 | -0.003 | 0.616 | 0.017 | 0.885 | 0.000 | 30.777 |
|  | rs7865719 | 0.011 | 0.552 | 0.017 | 0.508 | 0.000 | 35.166 |
|  | rs79973254 | -0.033 | 0.066 | 0.034 | 0.327 | 0.000 | 30.306 |
|  | rs9607629 | -0.018 | 0.119 | 0.026 | 0.495 | 0.000 | 44.168 |
|  | rs9617090 | -0.009 | 0.363 | 0.017 | 0.604 | 0.000 | 64.731 |
| Sphingomyelin (d38:1) | rs1042131 | 0.005 | 0.494 | 0.017 | 0.782 | 0.000 | 56.562 |
|  | rs1061537 | 0.011 | 0.388 | 0.017 | 0.519 | 0.000 | 59.252 |
|  | rs10748781 | -0.010 | 0.654 | 0.018 | 0.578 | 0.000 | 77.102 |
|  | rs10761659 | 0.017 | 0.524 | 0.017 | 0.296 | 0.000 | 35.706 |
|  | rs10807943 | -0.022 | 0.934 | 0.034 | 0.523 | 0.000 | 87.734 |
|  | rs10931828 | 0.005 | 0.437 | 0.017 | 0.771 | 0.000 | 33.803 |
|  | rs111432088 | 0.018 | 0.017 | 0.064 | 0.784 | 0.000 | 55.020 |
|  | rs11190368 | -0.004 | 0.453 | 0.017 | 0.788 | 0.000 | 31.466 |
|  | rs113595266 | 0.025 | 0.147 | 0.024 | 0.280 | 0.000 | 36.428 |
|  | rs113827118 | -0.005 | 0.194 | 0.021 | 0.825 | 0.000 | 71.157 |
|  | rs11614178 | -0.002 | 0.342 | 0.018 | 0.931 | 0.000 | 39.329 |
|  | rs11745587 | 0.040 | 0.402 | 0.017 | 0.020 | 0.000 | 45.225 |
|  | rs12132298 | 0.009 | 0.209 | 0.021 | 0.667 | 0.000 | 39.640 |
|  | rs12518457 | 0.021 | 0.285 | 0.018 | 0.259 | 0.000 | 30.509 |
|  | rs12536069 | 0.008 | 0.070 | 0.033 | 0.802 | 0.000 | 49.953 |
|  | rs12736494 | 0.007 | 0.255 | 0.019 | 0.698 | 0.000 | 34.777 |
|  | rs12942330 | -0.002 | 0.522 | 0.017 | 0.915 | 0.000 | 47.274 |
|  | rs148170789 | 0.019 | 0.047 | 0.039 | 0.623 | 0.000 | 56.054 |
|  | rs1801274 | 0.017 | 0.496 | 0.017 | 0.321 | 0.000 | 77.438 |
|  | rs181316459 | -0.027 | 0.051 | 0.040 | 0.490 | 0.001 | 266.342 |
|  | rs1990760 | -0.005 | 0.580 | 0.017 | 0.760 | 0.000 | 31.861 |
|  | rs2836883 | -0.027 | 0.240 | 0.020 | 0.182 | 0.000 | 51.168 |
|  | rs3024495 | -0.037 | 0.161 | 0.023 | 0.104 | 0.000 | 121.946 |
|  | rs3197999 | 0.000 | 0.381 | 0.017 | 0.981 | 0.000 | 106.975 |
|  | rs34145423 | 0.012 | 0.076 | 0.032 | 0.714 | 0.000 | 31.831 |
|  | rs34236350 | -0.008 | 0.254 | 0.019 | 0.677 | 0.000 | 81.068 |
|  | rs3732179 | -0.023 | 0.687 | 0.018 | 0.196 | 0.000 | 30.902 |
|  | rs4587163 | 0.027 | 0.270 | 0.019 | 0.159 | 0.000 | 144.846 |
|  | rs4655159 | -0.001 | 0.633 | 0.018 | 0.968 | 0.000 | 31.347 |
|  | rs4730276 | -0.008 | 0.441 | 0.017 | 0.641 | 0.000 | 68.499 |
|  | rs6089926 | 0.020 | 0.206 | 0.021 | 0.334 | 0.000 | 44.287 |
|  | rs6674040 | 0.018 | 0.468 | 0.017 | 0.288 | 0.000 | 96.537 |
|  | rs7101270 | -0.008 | 0.243 | 0.019 | 0.675 | 0.000 | 31.065 |
|  | rs7761376 | 0.009 | 0.616 | 0.017 | 0.586 | 0.000 | 30.777 |
|  | rs7865719 | 0.000 | 0.552 | 0.017 | 0.990 | 0.000 | 35.166 |
|  | rs79973254 | -0.030 | 0.066 | 0.034 | 0.369 | 0.000 | 30.306 |
|  | rs9607629 | 0.006 | 0.119 | 0.026 | 0.818 | 0.000 | 44.168 |
|  | rs9617090 | 0.009 | 0.363 | 0.017 | 0.617 | 0.000 | 64.731 |
| Sphingomyelin (d42:2) | rs1042131 | 0.007 | 0.494 | 0.017 | 0.682 | 0.000 | 56.562 |
|  | rs1061537 | 0.033 | 0.388 | 0.017 | 0.050 | 0.000 | 59.252 |
|  | rs10748781 | 0.024 | 0.654 | 0.018 | 0.174 | 0.000 | 77.102 |
|  | rs10761659 | -0.007 | 0.524 | 0.017 | 0.691 | 0.000 | 35.706 |
|  | rs10807943 | -0.016 | 0.934 | 0.034 | 0.637 | 0.000 | 87.734 |
|  | rs10931828 | 0.010 | 0.437 | 0.017 | 0.549 | 0.000 | 33.803 |
|  | rs111432088 | -0.058 | 0.017 | 0.064 | 0.365 | 0.000 | 55.020 |
|  | rs11190368 | -0.003 | 0.453 | 0.017 | 0.842 | 0.000 | 31.466 |
|  | rs113595266 | 0.025 | 0.147 | 0.024 | 0.298 | 0.000 | 36.428 |
|  | rs113827118 | 0.038 | 0.194 | 0.021 | 0.078 | 0.000 | 71.157 |
|  | rs11614178 | 0.017 | 0.342 | 0.018 | 0.347 | 0.000 | 39.329 |
|  | rs11745587 | 0.071 | 0.402 | 0.017 | 0.000 | 0.000 | 45.225 |
|  | rs12132298 | 0.006 | 0.209 | 0.021 | 0.789 | 0.000 | 39.640 |
|  | rs12518457 | -0.003 | 0.285 | 0.018 | 0.886 | 0.000 | 30.509 |
|  | rs12536069 | 0.034 | 0.070 | 0.033 | 0.301 | 0.000 | 49.953 |
|  | rs12736494 | 0.021 | 0.255 | 0.019 | 0.259 | 0.000 | 34.777 |
|  | rs12942330 | 0.007 | 0.522 | 0.017 | 0.694 | 0.000 | 47.274 |
|  | rs148170789 | 0.020 | 0.047 | 0.039 | 0.609 | 0.000 | 56.054 |
|  | rs1801274 | 0.009 | 0.496 | 0.017 | 0.586 | 0.000 | 77.438 |
|  | rs181316459 | 0.010 | 0.051 | 0.040 | 0.801 | 0.001 | 266.342 |
|  | rs1990760 | -0.031 | 0.580 | 0.017 | 0.063 | 0.000 | 31.861 |
|  | rs2836883 | -0.049 | 0.240 | 0.020 | 0.014 | 0.000 | 51.168 |
|  | rs3024495 | -0.029 | 0.161 | 0.023 | 0.196 | 0.000 | 121.946 |
|  | rs3197999 | -0.027 | 0.381 | 0.017 | 0.117 | 0.000 | 106.975 |
|  | rs34145423 | 0.088 | 0.076 | 0.032 | 0.006 | 0.000 | 31.831 |
|  | rs34236350 | 0.007 | 0.254 | 0.019 | 0.706 | 0.000 | 81.068 |
|  | rs3732179 | 0.031 | 0.687 | 0.018 | 0.081 | 0.000 | 30.902 |
|  | rs4587163 | 0.025 | 0.270 | 0.019 | 0.192 | 0.000 | 144.846 |
|  | rs4655159 | -0.004 | 0.633 | 0.018 | 0.819 | 0.000 | 31.347 |
|  | rs4730276 | 0.001 | 0.441 | 0.017 | 0.952 | 0.000 | 68.499 |
|  | rs6089926 | 0.019 | 0.206 | 0.021 | 0.352 | 0.000 | 44.287 |
|  | rs6674040 | 0.006 | 0.468 | 0.017 | 0.729 | 0.000 | 96.537 |
|  | rs7101270 | 0.009 | 0.243 | 0.019 | 0.656 | 0.000 | 31.065 |
|  | rs7761376 | 0.017 | 0.616 | 0.017 | 0.322 | 0.000 | 30.777 |
|  | rs7865719 | 0.012 | 0.552 | 0.017 | 0.460 | 0.000 | 35.166 |
|  | rs79973254 | -0.024 | 0.066 | 0.034 | 0.477 | 0.000 | 30.306 |
|  | rs9607629 | -0.012 | 0.119 | 0.026 | 0.647 | 0.000 | 44.168 |
|  | rs9617090 | 0.004 | 0.363 | 0.017 | 0.836 | 0.000 | 64.731 |
| **CD** |  |  |  |  |  |  |  |
| Sterol ester (27:1/20:4) | rs10807943 | 0.031 | 0.934 | 0.034 | 0.355 | 0.000 | 57.614 |
|  | rs10889669 | -0.015 | 0.257 | 0.019 | 0.422 | 0.000 | 45.245 |
|  | rs13300483 | -0.005 | 0.343 | 0.018 | 0.770 | 0.000 | 31.442 |
|  | rs181316459 | -0.024 | 0.051 | 0.039 | 0.541 | 0.000 | 117.235 |
|  | rs3130980 | 0.000 | 0.338 | 0.017 | 0.979 | 0.000 | 36.178 |
|  | rs34434863 | 0.005 | 0.333 | 0.017 | 0.791 | 0.000 | 57.224 |
|  | rs76176364 | -0.135 | 0.021 | 0.062 | 0.028 | 0.000 | 49.174 |
|  | rs7856092 | 0.003 | 0.750 | 0.019 | 0.877 | 0.000 | 34.509 |
| Phosphatidylcholine (20:4_0:0) | rs10807943 | 0.061 | 0.934 | 0.036 | 0.091 | 0.000 | 57.614 |
|  | rs10889669 | -0.012 | 0.257 | 0.021 | 0.553 | 0.000 | 45.245 |
|  | rs13300483 | -0.009 | 0.343 | 0.019 | 0.655 | 0.000 | 31.442 |
|  | rs181316459 | -0.020 | 0.051 | 0.042 | 0.629 | 0.000 | 117.235 |
|  | rs3130980 | -0.017 | 0.338 | 0.019 | 0.367 | 0.000 | 36.178 |
|  | rs34434863 | 0.008 | 0.333 | 0.019 | 0.652 | 0.000 | 57.224 |
|  | rs76176364 | 0.022 | 0.021 | 0.066 | 0.739 | 0.000 | 49.174 |
|  | rs7856092 | -0.022 | 0.750 | 0.021 | 0.283 | 0.000 | 34.509 |
| Phosphatidylcholine (16:0_20:4) | rs10807943 | 0.050 | 0.934 | 0.034 | 0.137 | 0.000 | 57.614 |
|  | rs10889669 | 0.001 | 0.257 | 0.019 | 0.974 | 0.000 | 45.245 |
|  | rs13300483 | -0.009 | 0.343 | 0.018 | 0.608 | 0.000 | 31.442 |
|  | rs181316459 | -0.021 | 0.051 | 0.039 | 0.592 | 0.000 | 117.235 |
|  | rs3130980 | 0.015 | 0.338 | 0.018 | 0.404 | 0.000 | 36.178 |
|  | rs34434863 | 0.019 | 0.333 | 0.017 | 0.265 | 0.000 | 57.224 |
|  | rs76176364 | -0.097 | 0.021 | 0.062 | 0.117 | 0.000 | 49.174 |
|  | rs7856092 | -0.007 | 0.750 | 0.019 | 0.712 | 0.000 | 34.509 |
| Phosphatidylcholine (17:0_18:1) | rs10807943 | -0.022 | 0.934 | 0.034 | 0.520 | 0.000 | 57.614 |
|  | rs10889669 | -0.005 | 0.257 | 0.019 | 0.791 | 0.000 | 45.245 |
|  | rs13300483 | 0.018 | 0.343 | 0.018 | 0.328 | 0.000 | 31.442 |
|  | rs181316459 | -0.001 | 0.051 | 0.040 | 0.978 | 0.000 | 117.235 |
|  | rs3130980 | -0.004 | 0.338 | 0.018 | 0.803 | 0.000 | 36.178 |
|  | rs34434863 | 0.034 | 0.333 | 0.018 | 0.056 | 0.000 | 57.224 |
|  | rs76176364 | -0.042 | 0.021 | 0.062 | 0.500 | 0.000 | 49.174 |
|  | rs7856092 | 0.012 | 0.750 | 0.020 | 0.556 | 0.000 | 34.509 |
| Phosphatidylcholine (17:0_18:2) | rs10807943 | -0.017 | 0.934 | 0.034 | 0.610 | 0.000 | 57.614 |
|  | rs10889669 | -0.004 | 0.257 | 0.019 | 0.828 | 0.000 | 45.245 |
|  | rs13300483 | 0.009 | 0.343 | 0.018 | 0.597 | 0.000 | 31.442 |
|  | rs181316459 | -0.005 | 0.051 | 0.040 | 0.894 | 0.000 | 117.235 |
|  | rs3130980 | 0.011 | 0.338 | 0.018 | 0.536 | 0.000 | 36.178 |
|  | rs34434863 | 0.025 | 0.333 | 0.017 | 0.147 | 0.000 | 57.224 |
|  | rs76176364 | -0.005 | 0.021 | 0.062 | 0.931 | 0.000 | 49.174 |
|  | rs7856092 | 0.044 | 0.750 | 0.019 | 0.024 | 0.000 | 34.509 |
| Phosphatidylcholine (18:1_20:4) | rs10807943 | 0.030 | 0.934 | 0.034 | 0.378 | 0.000 | 57.614 |
|  | rs10889669 | -0.019 | 0.257 | 0.019 | 0.315 | 0.000 | 45.245 |
|  | rs13300483 | 0.009 | 0.343 | 0.018 | 0.614 | 0.000 | 31.442 |
|  | rs181316459 | -0.033 | 0.051 | 0.040 | 0.406 | 0.000 | 117.235 |
|  | rs3130980 | 0.017 | 0.338 | 0.018 | 0.332 | 0.000 | 36.178 |
|  | rs34434863 | 0.007 | 0.333 | 0.017 | 0.688 | 0.000 | 57.224 |
|  | rs76176364 | -0.043 | 0.021 | 0.062 | 0.488 | 0.000 | 49.174 |
|  | rs7856092 | 0.001 | 0.750 | 0.019 | 0.949 | 0.000 | 34.509 |
| Phosphatidylethanolamine (O-18:1_20:4) | rs10807943 | 0.018 | 0.934 | 0.034 | 0.606 | 0.000 | 57.614 |
|  | rs10889669 | 0.011 | 0.257 | 0.019 | 0.565 | 0.000 | 45.245 |
|  | rs13300483 | 0.003 | 0.343 | 0.018 | 0.847 | 0.000 | 31.442 |
|  | rs181316459 | -0.054 | 0.051 | 0.040 | 0.173 | 0.000 | 117.235 |
|  | rs3130980 | -0.021 | 0.338 | 0.018 | 0.233 | 0.000 | 36.178 |
|  | rs34434863 | 0.010 | 0.333 | 0.017 | 0.561 | 0.000 | 57.224 |
|  | rs76176364 | -0.060 | 0.021 | 0.062 | 0.338 | 0.000 | 49.174 |
|  | rs7856092 | -0.003 | 0.750 | 0.020 | 0.885 | 0.000 | 34.509 |
| Abbreviations: SNPs: single nucleotide polymorphisms; EA: effect allele; OA: other allele; EAF: effect allele frequency; SE: standard error; IBD: inflammatory bowel disease; UC: ulcerative colitis; CD: Crohn's disease. | | | | | | | |

| **Supplementary Table S11. MR analysis results of associations between IBD with lipidome in reverse causal analysis.** | | | | | | |
| --- | --- | --- | --- | --- | --- | --- |
| **Plasma lipidome** | **Method** | **Nsnp** | **OR** | **OR_lci95** | **OR_uci95** | **P** |
| **IBD** |  |  |  |  |  |  |
| Sterol ester (27:1/22:6) | MR Egger | 46 | 0.971 | 0.880 | 1.070 | 0.551 |
|  | Weighted median | 46 | 0.985 | 0.928 | 1.046 | 0.626 |
|  | IVW | 46 | 0.998 | 0.958 | 1.040 | 0.937 |
|  | Simple mode | 46 | 0.999 | 0.887 | 1.125 | 0.984 |
|  | Weighted mode | 46 | 0.984 | 0.897 | 1.080 | 0.741 |
| Phosphatidylcholine (20:4_0:0) | MR Egger | 46 | 0.883 | 0.800 | 0.976 | 0.019 |
|  | Weighted median | 46 | 0.954 | 0.894 | 1.018 | 0.157 |
|  | IVW | 46 | 0.957 | 0.917 | 0.999 | 0.043 |
|  | Simple mode | 46 | 0.942 | 0.838 | 1.058 | 0.317 |
|  | Weighted mode | 46 | 0.949 | 0.865 | 1.041 | 0.272 |
| Phosphatidylcholine (16:0_20:4) | MR Egger | 46 | 0.907 | 0.826 | 0.996 | 0.046 |
|  | Weighted median | 46 | 0.969 | 0.913 | 1.028 | 0.296 |
|  | IVW | 46 | 0.982 | 0.943 | 1.022 | 0.370 |
|  | Simple mode | 46 | 0.970 | 0.864 | 1.090 | 0.614 |
|  | Weighted mode | 46 | 0.970 | 0.883 | 1.066 | 0.533 |
| Phosphatidylcholine (17:0_18:2) | MR Egger | 46 | 1.008 | 0.918 | 1.107 | 0.869 |
|  | Weighted median | 46 | 1.000 | 0.942 | 1.061 | 0.988 |
|  | IVW | 46 | 1.002 | 0.963 | 1.043 | 0.909 |
|  | Simple mode | 46 | 1.053 | 0.944 | 1.175 | 0.357 |
|  | Weighted mode | 46 | 1.024 | 0.936 | 1.119 | 0.609 |
| Sphingomyelin (d36:1) | MR Egger | 46 | 0.944 | 0.860 | 1.037 | 0.237 |
|  | Weighted median | 46 | 0.981 | 0.925 | 1.041 | 0.533 |
|  | IVW | 46 | 0.979 | 0.940 | 1.018 | 0.285 |
|  | Simple mode | 46 | 0.983 | 0.873 | 1.107 | 0.777 |
|  | Weighted mode | 46 | 0.979 | 0.893 | 1.073 | 0.648 |
| Sphingomyelin (d38:1) | MR Egger | 46 | 0.907 | 0.820 | 1.003 | 0.065 |
|  | Weighted median | 46 | 0.958 | 0.903 | 1.016 | 0.150 |
|  | IVW | 46 | 0.960 | 0.920 | 1.003 | 0.065 |
|  | Simple mode | 46 | 0.982 | 0.877 | 1.100 | 0.757 |
|  | Weighted mode | 46 | 0.965 | 0.886 | 1.051 | 0.419 |
| Sphingomyelin (d42:2) | MR Egger | 46 | 0.937 | 0.835 | 1.051 | 0.273 |
|  | Weighted median | 46 | 0.971 | 0.913 | 1.032 | 0.342 |
|  | IVW | 46 | 0.973 | 0.927 | 1.021 | 0.266 |
|  | Simple mode | 46 | 0.967 | 0.849 | 1.101 | 0.614 |
|  | Weighted mode | 46 | 0.978 | 0.890 | 1.074 | 0.641 |
| **UC** |  |  |  |  |  |  |
| Phosphatidylcholine (20:4_0:0) | MR Egger | 37 | 0.871 | 0.781 | 0.972 | 0.019 |
|  | Weighted median | 37 | 0.966 | 0.910 | 1.025 | 0.256 |
|  | IVW | 37 | 0.946 | 0.909 | 0.986 | 0.008 |
|  | Simple mode | 37 | 0.973 | 0.865 | 1.095 | 0.653 |
|  | Weighted mode | 37 | 0.964 | 0.883 | 1.053 | 0.426 |
| Phosphatidylethanolamine (18:2_0:0) | MR Egger | 37 | 0.891 | 0.802 | 0.990 | 0.038 |
|  | Weighted median | 37 | 0.933 | 0.878 | 0.990 | 0.023 |
|  | IVW | 37 | 0.954 | 0.918 | 0.992 | 0.017 |
|  | Simple mode | 37 | 1.042 | 0.924 | 1.174 | 0.508 |
|  | Weighted mode | 37 | 0.931 | 0.848 | 1.022 | 0.144 |
| Phosphatidylcholine (14:0_16:0 | MR Egger | 37 | 0.904 | 0.804 | 1.017 | 0.102 |
|  | Weighted median | 37 | 0.991 | 0.933 | 1.053 | 0.766 |
|  | IVW | 37 | 0.991 | 0.949 | 1.035 | 0.692 |
|  | Simple mode | 37 | 1.015 | 0.888 | 1.159 | 0.833 |
|  | Weighted mode | 37 | 0.987 | 0.886 | 1.099 | 0.812 |
| Phosphatidylcholine (16:0_20:2) | MR Egger | 37 | 0.926 | 0.835 | 1.027 | 0.154 |
|  | Weighted median | 37 | 0.968 | 0.916 | 1.022 | 0.241 |
|  | IVW | 37 | 0.980 | 0.943 | 1.018 | 0.289 |
|  | Simple mode | 37 | 0.957 | 0.862 | 1.062 | 0.415 |
|  | Weighted mode | 37 | 0.950 | 0.871 | 1.036 | 0.254 |
| Phosphatidylcholine (16:0_20:4) | MR Egger | 37 | 0.853 | 0.770 | 0.945 | 0.005 |
|  | Weighted median | 37 | 0.967 | 0.915 | 1.023 | 0.245 |
|  | IVW | 37 | 0.979 | 0.940 | 1.019 | 0.303 |
|  | Simple mode | 37 | 0.987 | 0.885 | 1.100 | 0.808 |
|  | Weighted mode | 37 | 0.977 | 0.887 | 1.076 | 0.637 |
| Phosphatidylcholine (16:1_18:1) | MR Egger | 37 | 0.938 | 0.830 | 1.061 | 0.316 |
|  | Weighted median | 37 | 0.974 | 0.921 | 1.031 | 0.362 |
|  | IVW | 37 | 0.967 | 0.925 | 1.012 | 0.147 |
|  | Simple mode | 37 | 0.984 | 0.882 | 1.098 | 0.780 |
|  | Weighted mode | 37 | 0.976 | 0.894 | 1.065 | 0.582 |
| Phosphatidylcholine (17:0_18:2) | MR Egger | 37 | 0.960 | 0.866 | 1.064 | 0.438 |
|  | Weighted median | 37 | 0.990 | 0.937 | 1.047 | 0.735 |
|  | IVW | 37 | 0.995 | 0.958 | 1.033 | 0.783 |
|  | Simple mode | 37 | 0.996 | 0.901 | 1.101 | 0.937 |
|  | Weighted mode | 37 | 0.982 | 0.903 | 1.068 | 0.679 |
| Phosphatidylcholine (18:0_20:4) | MR Egger | 37 | 0.826 | 0.746 | 0.915 | 0.001 |
|  | Weighted median | 37 | 0.956 | 0.904 | 1.012 | 0.120 |
|  | IVW | 37 | 0.969 | 0.932 | 1.008 | 0.121 |
|  | Simple mode | 37 | 0.992 | 0.885 | 1.113 | 0.897 |
|  | Weighted mode | 37 | 0.969 | 0.884 | 1.062 | 0.502 |
| Phosphatidylcholine (18:1_20:4) | MR Egger | 37 | 0.811 | 0.728 | 0.903 | 0.001 |
|  | Weighted median | 37 | 0.952 | 0.900 | 1.006 | 0.081 |
|  | IVW | 37 | 0.962 | 0.920 | 1.006 | 0.093 |
|  | Simple mode | 37 | 0.981 | 0.896 | 1.074 | 0.683 |
|  | Weighted mode | 37 | 0.964 | 0.885 | 1.050 | 0.404 |
| Phosphatidylcholine (O-16:1_20:4) | MR Egger | 37 | 0.848 | 0.758 | 0.948 | 0.007 |
|  | Weighted median | 37 | 0.977 | 0.917 | 1.040 | 0.466 |
|  | IVW | 37 | 0.963 | 0.924 | 1.003 | 0.070 |
|  | Simple mode | 37 | 0.985 | 0.876 | 1.109 | 0.808 |
|  | Weighted mode | 37 | 0.961 | 0.874 | 1.057 | 0.415 |
| Phosphatidylcholine (O-18:1_18:2) | MR Egger | 37 | 0.919 | 0.826 | 1.022 | 0.129 |
|  | Weighted median | 37 | 0.919 | 0.867 | 0.974 | 0.004 |
|  | IVW | 37 | 0.952 | 0.915 | 0.990 | 0.014 |
|  | Simple mode | 37 | 0.909 | 0.810 | 1.020 | 0.113 |
|  | Weighted mode | 37 | 0.903 | 0.806 | 1.011 | 0.084 |
| Phosphatidylethanolamine (18:0_18:2) | MR Egger | 37 | 0.876 | 0.789 | 0.972 | 0.018 |
|  | Weighted median | 37 | 0.977 | 0.926 | 1.030 | 0.389 |
|  | IVW | 37 | 0.988 | 0.949 | 1.030 | 0.578 |
|  | Simple mode | 37 | 0.948 | 0.859 | 1.045 | 0.288 |
|  | Weighted mode | 37 | 0.955 | 0.876 | 1.041 | 0.306 |
| Sphingomyelin (d36:1) | MR Egger | 37 | 0.968 | 0.873 | 1.073 | 0.539 |
|  | Weighted median | 37 | 0.989 | 0.937 | 1.044 | 0.683 |
|  | IVW | 37 | 0.983 | 0.946 | 1.021 | 0.376 |
|  | Simple mode | 37 | 0.981 | 0.879 | 1.095 | 0.733 |
|  | Weighted mode | 37 | 0.972 | 0.887 | 1.065 | 0.544 |
| Sphingomyelin (d38:1) | MR Egger | 37 | 0.950 | 0.857 | 1.053 | 0.333 |
|  | Weighted median | 37 | 0.956 | 0.909 | 1.007 | 0.089 |
|  | IVW | 37 | 0.970 | 0.934 | 1.008 | 0.120 |
|  | Simple mode | 37 | 0.961 | 0.871 | 1.061 | 0.436 |
|  | Weighted mode | 37 | 0.953 | 0.877 | 1.035 | 0.261 |
| Sphingomyelin (d42:2) | MR Egger | 37 | 0.961 | 0.839 | 1.099 | 0.563 |
|  | Weighted median | 37 | 0.965 | 0.912 | 1.022 | 0.226 |
|  | IVW | 37 | 0.970 | 0.924 | 1.019 | 0.226 |
|  | Simple mode | 37 | 0.945 | 0.851 | 1.050 | 0.299 |
|  | Weighted mode | 37 | 0.950 | 0.870 | 1.037 | 0.256 |
| **CD** |  |  |  |  |  |  |
| Sterol ester (27:1/20:4) | MR Egger | 8 | 0.892 | 0.778 | 1.023 | 0.154 |
|  | Weighted median | 8 | 0.968 | 0.903 | 1.038 | 0.361 |
|  | IVW | 8 | 0.955 | 0.904 | 1.008 | 0.094 |
|  | Simple mode | 8 | 0.975 | 0.888 | 1.071 | 0.617 |
|  | Weighted mode | 8 | 0.971 | 0.893 | 1.056 | 0.512 |
| Phosphatidylcholine (20:4_0:0) | MR Egger | 8 | 0.974 | 0.841 | 1.128 | 0.737 |
|  | Weighted median | 8 | 0.956 | 0.889 | 1.029 | 0.230 |
|  | IVW | 8 | 0.946 | 0.892 | 1.003 | 0.061 |
|  | Simple mode | 8 | 0.956 | 0.856 | 1.067 | 0.447 |
|  | Weighted mode | 8 | 0.962 | 0.871 | 1.062 | 0.466 |
| Phosphatidylcholine (16:0_20:4) | MR Egger | 8 | 0.889 | 0.775 | 1.021 | 0.146 |
|  | Weighted median | 8 | 0.961 | 0.895 | 1.031 | 0.269 |
|  | IVW | 8 | 0.954 | 0.903 | 1.008 | 0.091 |
|  | Simple mode | 8 | 0.960 | 0.867 | 1.064 | 0.462 |
|  | Weighted mode | 8 | 0.960 | 0.871 | 1.059 | 0.443 |
| Phosphatidylcholine (17:0_18:1) | MR Egger | 8 | 0.975 | 0.848 | 1.121 | 0.732 |
|  | Weighted median | 8 | 0.990 | 0.919 | 1.065 | 0.781 |
|  | IVW | 8 | 0.987 | 0.934 | 1.043 | 0.644 |
|  | Simple mode | 8 | 0.996 | 0.894 | 1.110 | 0.946 |
|  | Weighted mode | 8 | 1.000 | 0.900 | 1.111 | 0.999 |
| Phosphatidylcholine (17:0_18:2) | MR Egger | 8 | 0.969 | 0.830 | 1.130 | 0.699 |
|  | Weighted median | 8 | 0.996 | 0.925 | 1.073 | 0.925 |
|  | IVW | 8 | 1.014 | 0.957 | 1.075 | 0.635 |
|  | Simple mode | 8 | 1.012 | 0.906 | 1.131 | 0.839 |
|  | Weighted mode | 8 | 1.000 | 0.905 | 1.105 | 0.998 |
| Phosphatidylcholine (18:1_20:4) | MR Egger | 8 | 0.897 | 0.782 | 1.030 | 0.174 |
|  | Weighted median | 8 | 0.959 | 0.895 | 1.027 | 0.230 |
|  | IVW | 8 | 0.974 | 0.922 | 1.029 | 0.345 |
|  | Simple mode | 8 | 0.944 | 0.853 | 1.045 | 0.304 |
|  | Weighted mode | 8 | 0.949 | 0.863 | 1.043 | 0.314 |
| Phosphatidylethanolamine (O-18:1_20:4) | MR Egger | 8 | 0.905 | 0.789 | 1.040 | 0.208 |
|  | Weighted median | 8 | 0.961 | 0.900 | 1.026 | 0.229 |
|  | IVW | 8 | 0.959 | 0.908 | 1.013 | 0.134 |
|  | Simple mode | 8 | 0.943 | 0.852 | 1.044 | 0.296 |
|  | Weighted mode | 8 | 0.943 | 0.858 | 1.037 | 0.265 |
| Abbreviations: IVW: inverse variance weighted; β:beta; OR: odds ratio; IBD: inflammatory bowel disease; UC: ulcerative colitis; CD: Crohn's disease. | | | | | | |

| **Supplementary Table S12. MR analysis results of associations between inflammatory proteins and IBD after the initial screening.** | | | | | | | | | | | | | |
| --- | --- | --- | --- | --- | --- | --- | --- | --- | --- | --- | --- | --- | --- |
| **Inflammatory proteins** | **Method** | **Nsnp** | **OR** | **OR_lci95** | **OR_uci95** | **P** | **Heterogeneity** | | | | **Pleiotropy** | | |
|  |  |  |  |  |  |  | **MR Egger** | | **IVW** | | **MR-Egger intercep** | | **MR-PRESO** |
|  |  |  |  |  |  |  | **Q** | **P** | **Q** | **P** | **intercept** | **P** | **P** |
| **IBD** |  |  |  |  |  |  |  |  |  |  |  |  |  |
| Axin-1 | MR Egger | 13 | 1.268 | 0.853 | 1.885 | 0.264 | 13.878 | 0.240 | 13.993 | 0.301 | -0.006 | 0.768 | 0.371 |
|  | Weighted median | 13 | 1.176 | 0.979 | 1.412 | 0.083 |  |  |  |  |  |  |  |
|  | IVW | 13 | 1.199 | 1.036 | 1.387 | 0.015 |  |  |  |  |  |  |  |
|  | Simple mode | 13 | 1.233 | 0.911 | 1.668 | 0.200 |  |  |  |  |  |  |  |
|  | Weighted mode | 13 | 1.211 | 0.911 | 1.612 | 0.212 |  |  |  |  |  |  |  |
|  | BWMR | 13 | 1.206 | 1.045 | 1.391 | 0.010 |  |  |  |  |  |  |  |
| CCL11 | MR Egger | 27 | 0.922 | 0.772 | 1.100 | 0.375 | 34.495 | 0.098 | 34.610 | 0.120 | -0.003 | 0.776 | 0.111 |
|  | Weighted median | 27 | 0.954 | 0.840 | 1.083 | 0.464 |  |  |  |  |  |  |  |
|  | IVW | 27 | 0.902 | 0.823 | 0.988 | 0.027 |  |  |  |  |  |  |  |
|  | Simple mode | 27 | 0.942 | 0.741 | 1.198 | 0.630 |  |  |  |  |  |  |  |
|  | Weighted mode | 27 | 1.022 | 0.856 | 1.221 | 0.808 |  |  |  |  |  |  |  |
|  | BWMR | 27 | 0.908 | 0.822 | 1.002 | 0.055 |  |  |  |  |  |  |  |
| CCL19 | MR Egger | 36 | 1.105 | 0.986 | 1.239 | 0.096 | 46.677 | 0.072 | 46.990 | 0.085 | -0.004 | 0.636 | 0.057 |
|  | Weighted median | 36 | 1.167 | 1.063 | 1.282 | 0.001 |  |  |  |  |  |  |  |
|  | IVW | 36 | 1.082 | 1.007 | 1.162 | 0.032 |  |  |  |  |  |  |  |
|  | Simple mode | 36 | 1.085 | 0.882 | 1.334 | 0.447 |  |  |  |  |  |  |  |
|  | Weighted mode | 36 | 1.152 | 1.042 | 1.274 | 0.009 |  |  |  |  |  |  |  |
|  | BWMR | 36 | 1.101 | 1.018 | 1.191 | 0.016 |  |  |  |  |  |  |  |
| CCL20 | MR Egger | 33 | 1.130 | 0.837 | 1.527 | 0.431 | 50.851 | 0.014 | 50.863 | 0.018 | 0.001 | 0.935 | 0.014 |
|  | Weighted median | 33 | 1.094 | 0.961 | 1.245 | 0.174 |  |  |  |  |  |  |  |
|  | IVW | 33 | 1.144 | 1.023 | 1.279 | 0.019 |  |  |  |  |  |  |  |
|  | Simple mode | 33 | 1.072 | 0.841 | 1.367 | 0.577 |  |  |  |  |  |  |  |
|  | Weighted mode | 33 | 1.067 | 0.858 | 1.327 | 0.563 |  |  |  |  |  |  |  |
|  | BWMR | 33 | 1.144 | 1.026 | 1.276 | 0.015 |  |  |  |  |  |  |  |
| CCL4 | MR Egger | 31 | 1.171 | 1.077 | 1.274 | 0.001 | 43.861 | 0.038 | 48.900 | 0.016 | -0.015 | 0.078 | 0.059 |
|  | Weighted median | 31 | 1.140 | 1.045 | 1.244 | 0.003 |  |  |  |  |  |  |  |
|  | IVW | 31 | 1.109 | 1.042 | 1.180 | 0.001 |  |  |  |  |  |  |  |
|  | Simple mode | 31 | 0.988 | 0.800 | 1.221 | 0.913 |  |  |  |  |  |  |  |
|  | Weighted mode | 31 | 1.125 | 1.052 | 1.202 | 0.002 |  |  |  |  |  |  |  |
|  | BWMR | 31 | 1.119 | 1.047 | 1.196 | 0.001 |  |  |  |  |  |  |  |
| CD6 | MR Egger | 24 | 1.123 | 1.021 | 1.235 | 0.026 | 40.006 | 0.011 | 40.678 | 0.013 | -0.006 | 0.549 | 0.051 |
|  | Weighted median | 24 | 1.114 | 1.049 | 1.183 | 0.000 |  |  |  |  |  |  |  |
|  | IVW | 24 | 1.100 | 1.027 | 1.179 | 0.006 |  |  |  |  |  |  |  |
|  | Simple mode | 24 | 0.967 | 0.765 | 1.222 | 0.779 |  |  |  |  |  |  |  |
|  | Weighted mode | 24 | 1.105 | 1.036 | 1.180 | 0.006 |  |  |  |  |  |  |  |
|  | BWMR | 24 | 1.096 | 1.020 | 1.179 | 0.013 |  |  |  |  |  |  |  |
| CXCL10 | MR Egger | 33 | 0.960 | 0.789 | 1.167 | 0.682 | 56.754 | 0.003 | 63.303 | 0.001 | 0.018 | 0.068 | 0.045 |
|  | Weighted median | 33 | 1.074 | 0.937 | 1.232 | 0.305 |  |  |  |  |  |  |  |
|  | IVW | 33 | 1.125 | 1.007 | 1.256 | 0.037 |  |  |  |  |  |  |  |
|  | Simple mode | 33 | 1.000 | 0.762 | 1.311 | 0.998 |  |  |  |  |  |  |  |
|  | Weighted mode | 33 | 1.049 | 0.855 | 1.288 | 0.648 |  |  |  |  |  |  |  |
|  | BWMR | 33 | 1.084 | 0.976 | 1.203 | 0.132 |  |  |  |  |  |  |  |
| FGF-5 | MR Egger | 32 | 1.048 | 0.970 | 1.131 | 0.245 | 43.255 | 0.056 | 43.823 | 0.063 | 0.005 | 0.535 | 0.110 |
|  | Weighted median | 32 | 1.034 | 0.975 | 1.095 | 0.263 |  |  |  |  |  |  |  |
|  | IVW | 32 | 1.066 | 1.010 | 1.125 | 0.019 |  |  |  |  |  |  |  |
|  | Simple mode | 32 | 0.989 | 0.827 | 1.184 | 0.906 |  |  |  |  |  |  |  |
|  | Weighted mode | 32 | 1.038 | 0.981 | 1.100 | 0.207 |  |  |  |  |  |  |  |
|  | BWMR | 32 | 1.074 | 1.013 | 1.139 | 0.016 |  |  |  |  |  |  |  |
| FIt3L | MR Egger | 45 | 0.961 | 0.838 | 1.101 | 0.567 | 66.625 | 0.012 | 69.360 | 0.009 | -0.010 | 0.191 | 0.007 |
|  | Weighted median | 45 | 0.950 | 0.856 | 1.054 | 0.330 |  |  |  |  |  |  |  |
|  | IVW | 45 | 0.891 | 0.823 | 0.966 | 0.005 |  |  |  |  |  |  |  |
|  | Simple mode | 45 | 0.953 | 0.785 | 1.157 | 0.629 |  |  |  |  |  |  |  |
|  | Weighted mode | 45 | 0.940 | 0.848 | 1.043 | 0.251 |  |  |  |  |  |  |  |
|  | BWMR | 45 | 0.897 | 0.833 | 0.964 | 0.003 |  |  |  |  |  |  |  |
| IL-10 α | MR Egger | 19 | 1.183 | 0.936 | 1.495 | 0.178 | 32.606 | 0.013 | 32.716 | 0.018 | 0.003 | 0.813 | 0.036 |
|  | Weighted median | 19 | 1.185 | 1.026 | 1.368 | 0.021 |  |  |  |  |  |  |  |
|  | IVW | 19 | 1.211 | 1.068 | 1.375 | 0.003 |  |  |  |  |  |  |  |
|  | Simple mode | 19 | 1.047 | 0.800 | 1.371 | 0.741 |  |  |  |  |  |  |  |
|  | Weighted mode | 19 | 1.161 | 0.964 | 1.398 | 0.134 |  |  |  |  |  |  |  |
|  | BWMR | 19 | 1.225 | 1.078 | 1.391 | 0.002 |  |  |  |  |  |  |  |
| IL12 β | MR Egger | 37 | 1.199 | 1.072 | 1.341 | 0.003 | 83.358 | 0.000 | 85.777 | 0.000 | -0.008 | 0.321 | 0.012 |
|  | Weighted median | 37 | 1.218 | 1.132 | 1.310 | 0.000 |  |  |  |  |  |  |  |
|  | IVW | 37 | 1.147 | 1.068 | 1.231 | 0.000 |  |  |  |  |  |  |  |
|  | Simple mode | 37 | 1.040 | 0.853 | 1.267 | 0.700 |  |  |  |  |  |  |  |
|  | Weighted mode | 37 | 1.196 | 1.117 | 1.281 | 0.000 |  |  |  |  |  |  |  |
|  | BWMR | 37 | 1.135 | 1.052 | 1.225 | 0.001 |  |  |  |  |  |  |  |
| TNFSF12 | MR Egger | 43 | 1.089 | 0.944 | 1.256 | 0.251 | 35.007 | 0.733 | 35.007 | 0.769 | 0.000 | 0.988 | 0.771 |
|  | Weighted median | 43 | 1.110 | 1.002 | 1.229 | 0.046 |  |  |  |  |  |  |  |
|  | IVW | 43 | 1.088 | 1.016 | 1.165 | 0.016 |  |  |  |  |  |  |  |
|  | Simple mode | 43 | 1.043 | 0.885 | 1.230 | 0.619 |  |  |  |  |  |  |  |
|  | Weighted mode | 43 | 1.121 | 0.986 | 1.274 | 0.089 |  |  |  |  |  |  |  |
|  | BWMR | 43 | 1.084 | 1.010 | 1.163 | 0.025 |  |  |  |  |  |  |  |
| UC |  |  |  |  |  |  |  |  |  |  |  |  |  |
| CCL11 | MR Egger | 27 | 0.963 | 0.756 | 1.227 | 0.765 | 42.778 | 0.015 | 44.099 | 0.015 | -0.013 | 0.388 | 0.057 |
|  | Weighted median | 27 | 0.925 | 0.788 | 1.086 | 0.342 |  |  |  |  |  |  |  |
|  | IVW | 27 | 0.879 | 0.774 | 0.998 | 0.046 |  |  |  |  |  |  |  |
|  | Simple mode | 27 | 0.909 | 0.657 | 1.258 | 0.570 |  |  |  |  |  |  |  |
|  | Weighted mode | 27 | 1.129 | 0.906 | 1.408 | 0.291 |  |  |  |  |  |  |  |
|  | BWMR | 27 | 0.886 | 0.777 | 1.009 | 0.068 |  |  |  |  |  |  |  |
| CCL4 | MR Egger | 31 | 1.196 | 1.062 | 1.346 | 0.006 | 57.821 | 0.001 | 62.187 | 0.000 | -0.017 | 0.150 | 0.370 |
|  | Weighted median | 31 | 1.295 | 1.162 | 1.444 | 0.000 |  |  |  |  |  |  |  |
|  | IVW | 31 | 1.123 | 1.030 | 1.224 | 0.008 |  |  |  |  |  |  |  |
|  | Simple mode | 31 | 0.858 | 0.623 | 1.183 | 0.358 |  |  |  |  |  |  |  |
|  | Weighted mode | 31 | 1.158 | 1.062 | 1.263 | 0.002 |  |  |  |  |  |  |  |
|  | BWMR | 31 | 1.146 | 1.044 | 1.257 | 0.004 |  |  |  |  |  |  |  |
| FGF-5 | MR Egger | 32 | 1.105 | 1.013 | 1.204 | 0.031 | 35.980 | 0.209 | 37.269 | 0.203 | -0.009 | 0.308 | 0.299 |
|  | Weighted median | 32 | 1.067 | 0.993 | 1.147 | 0.076 |  |  |  |  |  |  |  |
|  | IVW | 32 | 1.070 | 1.006 | 1.137 | 0.031 |  |  |  |  |  |  |  |
|  | Simple mode | 32 | 0.842 | 0.690 | 1.027 | 0.100 |  |  |  |  |  |  |  |
|  | Weighted mode | 32 | 1.074 | 1.002 | 1.151 | 0.051 |  |  |  |  |  |  |  |
|  | BWMR | 32 | 1.071 | 1.007 | 1.139 | 0.029 |  |  |  |  |  |  |  |
| FIt3L | MR Egger | 45 | 1.008 | 0.853 | 1.190 | 0.930 | 65.664 | 0.015 | 69.883 | 0.008 | -0.015 | 0.104 | 0.015 |
|  | Weighted median | 45 | 0.947 | 0.830 | 1.080 | 0.416 |  |  |  |  |  |  |  |
|  | IVW | 45 | 0.898 | 0.814 | 0.992 | 0.034 |  |  |  |  |  |  |  |
|  | Simple mode | 45 | 0.985 | 0.786 | 1.235 | 0.899 |  |  |  |  |  |  |  |
|  | Weighted mode | 45 | 0.953 | 0.834 | 1.089 | 0.483 |  |  |  |  |  |  |  |
|  | BWMR | 45 | 0.915 | 0.834 | 1.003 | 0.059 |  |  |  |  |  |  |  |
| IL12 β | MR Egger | 37 | 1.183 | 1.043 | 1.342 | 0.013 | 69.471 | 0.000 | 70.847 | 0.000 | -0.008 | 0.411 | 0.002 |
|  | Weighted median | 37 | 1.210 | 1.109 | 1.320 | 0.000 |  |  |  |  |  |  |  |
|  | IVW | 37 | 1.136 | 1.049 | 1.230 | 0.002 |  |  |  |  |  |  |  |
|  | Simple mode | 37 | 1.182 | 0.938 | 1.490 | 0.165 |  |  |  |  |  |  |  |
|  | Weighted mode | 37 | 1.182 | 1.093 | 1.280 | 0.000 |  |  |  |  |  |  |  |
|  | BWMR | 37 | 1.132 | 1.039 | 1.234 | 0.005 |  |  |  |  |  |  |  |
| MCP-1 | MR Egger | 30 | 0.964 | 0.777 | 1.196 | 0.741 | 72.067 | 0.000 | 75.549 | 0.000 | -0.016 | 0.255 | 0.022 |
|  | Weighted median | 30 | 0.866 | 0.756 | 0.993 | 0.039 |  |  |  |  |  |  |  |
|  | IVW | 30 | 0.869 | 0.766 | 0.986 | 0.030 |  |  |  |  |  |  |  |
|  | Simple mode | 30 | 0.904 | 0.668 | 1.225 | 0.521 |  |  |  |  |  |  |  |
|  | Weighted mode | 30 | 0.822 | 0.683 | 0.989 | 0.047 |  |  |  |  |  |  |  |
|  | BWMR | 30 | 0.919 | 0.811 | 1.041 | 0.183 |  |  |  |  |  |  |  |
| PD-L1 | MR Egger | 24 | 0.773 | 0.530 | 1.128 | 0.195 | 42.779 | 0.005 | 43.357 | 0.006 | 0.009 | 0.591 | 0.007 |
|  | Weighted median | 24 | 0.853 | 0.705 | 1.032 | 0.102 |  |  |  |  |  |  |  |
|  | IVW | 24 | 0.850 | 0.724 | 0.999 | 0.048 |  |  |  |  |  |  |  |
|  | Simple mode | 24 | 0.864 | 0.620 | 1.204 | 0.396 |  |  |  |  |  |  |  |
|  | Weighted mode | 24 | 0.836 | 0.615 | 1.138 | 0.267 |  |  |  |  |  |  |  |
|  | BWMR | 24 | 0.878 | 0.740 | 1.041 | 0.134 |  |  |  |  |  |  |  |
| CD |  |  |  |  |  |  |  |  |  |  |  |  |  |
| Axin-1 | MR Egger | 13 | 2.215 | 1.060 | 4.626 | 0.058 | 6.742 | 0.820 | 8.456 | 0.749 | -0.045 | 0.217 | 0.768 |
|  | Weighted median | 13 | 1.223 | 0.839 | 1.782 | 0.295 |  |  |  |  |  |  |  |
|  | IVW | 13 | 1.406 | 1.060 | 1.864 | 0.018 |  |  |  |  |  |  |  |
|  | Simple mode | 13 | 1.102 | 0.615 | 1.975 | 0.750 |  |  |  |  |  |  |  |
|  | Weighted mode | 13 | 1.135 | 0.634 | 2.031 | 0.677 |  |  |  |  |  |  |  |
|  | BWMR | 13 | 1.405 | 1.044 | 1.892 | 0.025 |  |  |  |  |  |  |  |
| CCL19 | MR Egger | 36 | 1.278 | 1.019 | 1.602 | 0.041 | 43.425 | 0.129 | 44.138 | 0.138 | -0.011 | 0.460 | 0.086 |
|  | Weighted median | 36 | 1.451 | 1.186 | 1.774 | 0.000 |  |  |  |  |  |  |  |
|  | IVW | 36 | 1.196 | 1.036 | 1.380 | 0.015 |  |  |  |  |  |  |  |
|  | Simple mode | 36 | 1.045 | 0.637 | 1.714 | 0.864 |  |  |  |  |  |  |  |
|  | Weighted mode | 36 | 1.459 | 1.185 | 1.796 | 0.001 |  |  |  |  |  |  |  |
|  | BWMR | 36 | 1.210 | 1.038 | 1.410 | 0.015 |  |  |  |  |  |  |  |
| CCL20 | MR Egger | 33 | 0.720 | 0.386 | 1.343 | 0.310 | 49.733 | 0.018 | 56.164 | 0.005 | 0.054 | 0.054 | 0.004 |
|  | Weighted median | 33 | 0.961 | 0.715 | 1.293 | 0.795 |  |  |  |  |  |  |  |
|  | IVW | 33 | 1.298 | 1.015 | 1.660 | 0.038 |  |  |  |  |  |  |  |
|  | Simple mode | 33 | 0.941 | 0.472 | 1.873 | 0.863 |  |  |  |  |  |  |  |
|  | Weighted mode | 33 | 0.930 | 0.491 | 1.761 | 0.825 |  |  |  |  |  |  |  |
|  | BWMR | 33 | 1.249 | 0.987 | 1.581 | 0.065 |  |  |  |  |  |  |  |
| CD5 | MR Egger | 29 | 1.966 | 1.208 | 3.201 | 0.011 | 36.254 | 0.110 | 39.785 | 0.069 | -0.040 | 0.117 | 0.076 |
|  | Weighted median | 29 | 1.384 | 1.074 | 1.785 | 0.012 |  |  |  |  |  |  |  |
|  | IVW | 29 | 1.359 | 1.112 | 1.661 | 0.003 |  |  |  |  |  |  |  |
|  | Simple mode | 29 | 1.117 | 0.702 | 1.778 | 0.645 |  |  |  |  |  |  |  |
|  | Weighted mode | 29 | 1.533 | 1.081 | 2.173 | 0.023 |  |  |  |  |  |  |  |
|  | BWMR | 29 | 1.360 | 1.117 | 1.656 | 0.002 |  |  |  |  |  |  |  |
| CD6 | MR Egger | 24 | 1.272 | 1.086 | 1.489 | 0.007 | 24.807 | 0.306 | 26.297 | 0.287 | -0.017 | 0.263 | 0.312 |
|  | Weighted median | 24 | 1.255 | 1.106 | 1.424 | 0.000 |  |  |  |  |  |  |  |
|  | IVW | 24 | 1.194 | 1.063 | 1.342 | 0.003 |  |  |  |  |  |  |  |
|  | Simple mode | 24 | 1.381 | 0.896 | 2.128 | 0.157 |  |  |  |  |  |  |  |
|  | Weighted mode | 24 | 1.276 | 1.117 | 1.457 | 0.002 |  |  |  |  |  |  |  |
|  | BWMR | 24 | 1.196 | 1.054 | 1.358 | 0.005 |  |  |  |  |  |  |  |
| FGF-21 | MR Egger | 24 | 1.979 | 1.199 | 3.267 | 0.014 | 31.852 | 0.080 | 36.688 | 0.035 | -0.046 | 0.081 | 0.017 |
|  | Weighted median | 24 | 1.459 | 1.099 | 1.936 | 0.009 |  |  |  |  |  |  |  |
|  | IVW | 24 | 1.298 | 1.035 | 1.627 | 0.024 |  |  |  |  |  |  |  |
|  | Simple mode | 24 | 1.243 | 0.605 | 2.554 | 0.559 |  |  |  |  |  |  |  |
|  | Weighted mode | 24 | 1.609 | 1.116 | 2.319 | 0.018 |  |  |  |  |  |  |  |
|  | BWMR | 24 | 1.262 | 0.990 | 1.608 | 0.061 |  |  |  |  |  |  |  |
| IL-10 α | MR Egger | 19 | 1.281 | 0.899 | 1.824 | 0.188 | 11.051 | 0.854 | 11.202 | 0.886 | 0.007 | 0.702 | 0.909 |
|  | Weighted median | 19 | 1.262 | 0.944 | 1.688 | 0.116 |  |  |  |  |  |  |  |
|  | IVW | 19 | 1.358 | 1.116 | 1.652 | 0.002 |  |  |  |  |  |  |  |
|  | Simple mode | 19 | 1.618 | 1.009 | 2.595 | 0.061 |  |  |  |  |  |  |  |
|  | Weighted mode | 19 | 1.217 | 0.892 | 1.660 | 0.232 |  |  |  |  |  |  |  |
|  | BWMR | 19 | 1.414 | 1.141 | 1.752 | 0.002 |  |  |  |  |  |  |  |
| IL12 β | MR Egger | 37 | 1.349 | 1.117 | 1.629 | 0.004 | 53.638 | 0.023 | 56.471 | 0.016 | -0.019 | 0.183 | 0.020 |
|  | Weighted median | 37 | 1.373 | 1.180 | 1.598 | 0.000 |  |  |  |  |  |  |  |
|  | IVW | 37 | 1.219 | 1.080 | 1.376 | 0.001 |  |  |  |  |  |  |  |
|  | Simple mode | 37 | 1.116 | 0.785 | 1.587 | 0.545 |  |  |  |  |  |  |  |
|  | Weighted mode | 37 | 1.333 | 1.166 | 1.524 | 0.000 |  |  |  |  |  |  |  |
|  | BWMR | 37 | 1.213 | 1.065 | 1.380 | 0.004 |  |  |  |  |  |  |  |
| STAMPB | MR Egger | 20 | 1.252 | 0.692 | 2.265 | 0.468 | 7.418 | 0.986 | 7.420 | 0.992 | 0.001 | 0.965 | 0.988 |
|  | Weighted median | 20 | 1.265 | 0.915 | 1.749 | 0.155 |  |  |  |  |  |  |  |
|  | IVW | 20 | 1.267 | 1.002 | 1.602 | 0.048 |  |  |  |  |  |  |  |
|  | Simple mode | 20 | 1.253 | 0.735 | 2.136 | 0.417 |  |  |  |  |  |  |  |
|  | Weighted mode | 20 | 1.213 | 0.737 | 1.999 | 0.457 |  |  |  |  |  |  |  |
|  | BWMR | 20 | 1.276 | 1.000 | 1.628 | 0.050 |  |  |  |  |  |  |  |
| Abbreviations: IVW: inverse variance weighted; BWMR: Bayesian Weighted Mendelian Randomization; β:beta; OR: odds ratio; IBD: inflammatory bowel disease; UC: ulcerative colitis; CD: Crohn's disease. | | | | | | | | | | | | | |

| **Supplementary Table S13. Association of genome-wide SNPs for inflammatory proteins with IBD** | | | | | | | |
| --- | --- | --- | --- | --- | --- | --- | --- |
| **Inflammatory proteins** | **SNP** | **Beta outcome** | **EAF outcome** | **SE outcome** | **P outcome** | **R^2^** | **F-statistic** |
| **IBD** |  |  |  |  |  |  |  |
| Axin-1 | rs10860902 | 0.011 | 0.736 | 0.017 | 0.515 | 0.002 | 19.558 |
|  | rs11202576 | -0.005 | 0.811 | 0.019 | 0.785 | 0.002 | 19.882 |
|  | rs113946880 | 0.045 | 0.035 | 0.041 | 0.273 | 0.002 | 20.632 |
|  | rs115455827 | 0.043 | 0.926 | 0.029 | 0.136 | 0.002 | 22.504 |
|  | rs117097664 | 0.020 | 0.018 | 0.057 | 0.723 | 0.002 | 21.917 |
|  | rs148317708 | -0.183 | 0.010 | 0.076 | 0.016 | 0.002 | 25.474 |
|  | rs2470622 | 0.012 | 0.922 | 0.028 | 0.673 | 0.002 | 25.787 |
|  | rs2691530 | -0.010 | 0.560 | 0.015 | 0.516 | 0.002 | 25.373 |
|  | rs2712427 | 0.018 | 0.466 | 0.015 | 0.233 | 0.002 | 21.820 |
|  | rs428324 | 0.025 | 0.555 | 0.015 | 0.100 | 0.002 | 21.858 |
|  | rs7171268 | 0.073 | 0.961 | 0.039 | 0.062 | 0.002 | 22.307 |
|  | rs72984228 | 0.000 | 0.957 | 0.037 | 0.990 | 0.002 | 19.786 |
|  | rs9962377 | 0.045 | 0.090 | 0.027 | 0.093 | 0.002 | 20.538 |
| CCL11 | rs10911109 | -0.008 | 0.200 | 0.019 | 0.657 | 0.002 | 19.882 |
|  | rs114744647 | -0.031 | 0.983 | 0.059 | 0.601 | 0.001 | 21.823 |
|  | rs12075 | -0.032 | 0.532 | 0.015 | 0.036 | 0.003 | 43.161 |
|  | rs12453306 | 0.002 | 0.903 | 0.026 | 0.939 | 0.002 | 19.916 |
|  | rs139476527 | -0.074 | 0.002 | 0.199 | 0.708 | 0.002 | 21.744 |
|  | rs139597204 | -0.010 | 0.004 | 0.129 | 0.938 | 0.002 | 22.327 |
|  | rs140965534 | 0.026 | 0.014 | 0.064 | 0.688 | 0.001 | 19.725 |
|  | rs148554043 | -0.106 | 0.029 | 0.046 | 0.021 | 0.002 | 20.267 |
|  | rs151240414 | -0.068 | 0.009 | 0.079 | 0.391 | 0.002 | 20.477 |
|  | rs1564995 | 0.002 | 0.558 | 0.015 | 0.918 | 0.002 | 21.609 |
|  | rs170520 | 0.000 | 0.299 | 0.017 | 0.984 | 0.001 | 19.640 |
|  | rs2112920 | 0.017 | 0.835 | 0.020 | 0.394 | 0.002 | 26.070 |
|  | rs2201150 | -0.071 | 0.383 | 0.016 | 0.000 | 0.003 | 40.726 |
|  | rs2228467 | -0.023 | 0.924 | 0.028 | 0.412 | 0.015 | 217.173 |
|  | rs34536806 | -0.011 | 0.929 | 0.030 | 0.707 | 0.002 | 23.903 |
|  | rs35543857 | 0.006 | 0.906 | 0.026 | 0.819 | 0.002 | 21.749 |
|  | rs55843637 | 0.008 | 0.506 | 0.015 | 0.605 | 0.001 | 21.036 |
|  | rs6894241 | -0.007 | 0.240 | 0.018 | 0.695 | 0.002 | 22.925 |
|  | rs73171545 | 0.010 | 0.115 | 0.024 | 0.684 | 0.001 | 21.196 |
|  | rs73209015 | -0.014 | 0.100 | 0.025 | 0.588 | 0.002 | 23.332 |
|  | rs757973 | -0.023 | 0.069 | 0.030 | 0.441 | 0.006 | 95.359 |
|  | rs79432525 | -0.027 | 0.991 | 0.079 | 0.733 | 0.001 | 20.892 |
|  | rs79684468 | -0.019 | 0.044 | 0.037 | 0.607 | 0.002 | 24.569 |
|  | rs79722574 | 0.036 | 0.140 | 0.022 | 0.100 | 0.004 | 60.652 |
|  | rs8068185 | 0.003 | 0.869 | 0.022 | 0.890 | 0.001 | 20.928 |
|  | rs9303380 | -0.007 | 0.042 | 0.038 | 0.857 | 0.001 | 19.885 |
|  | rs9940390 | -0.016 | 0.685 | 0.016 | 0.325 | 0.003 | 37.822 |
|  | rs9976282 | 0.085 | 0.020 | 0.054 | 0.113 | 0.002 | 23.653 |
| CCL19 | rs10242459 | 0.022 | 0.845 | 0.021 | 0.293 | 0.002 | 22.405 |
|  | rs111317412 | 0.010 | 0.966 | 0.042 | 0.814 | 0.001 | 20.346 |
|  | rs11174279 | -0.003 | 0.201 | 0.019 | 0.874 | 0.001 | 19.788 |
|  | rs113139367 | 0.005 | 0.030 | 0.044 | 0.916 | 0.002 | 23.870 |
|  | rs113539352 | 0.117 | 0.999 | 0.359 | 0.745 | 0.002 | 23.658 |
|  | rs115109204 | -0.065 | 0.005 | 0.115 | 0.572 | 0.002 | 20.193 |
|  | rs11574915 | 0.045 | 0.846 | 0.021 | 0.033 | 0.005 | 74.152 |
|  | rs11877886 | -0.003 | 0.082 | 0.028 | 0.918 | 0.001 | 19.854 |
|  | rs11961362 | -0.034 | 0.083 | 0.027 | 0.218 | 0.002 | 20.145 |
|  | rs12521515 | 0.009 | 0.832 | 0.020 | 0.661 | 0.002 | 20.060 |
|  | rs13010492 | -0.011 | 0.366 | 0.016 | 0.465 | 0.003 | 42.614 |
|  | rs13212719 | 0.004 | 0.853 | 0.021 | 0.843 | 0.001 | 20.008 |
|  | rs1328133 | 0.029 | 0.005 | 0.111 | 0.792 | 0.001 | 20.047 |
|  | rs138108512 | 0.075 | 0.006 | 0.103 | 0.469 | 0.002 | 22.760 |
|  | rs138440733 | 0.024 | 0.063 | 0.032 | 0.461 | 0.002 | 22.930 |
|  | rs138618048 | -0.020 | 0.009 | 0.080 | 0.807 | 0.001 | 20.169 |
|  | rs1415763 | 0.041 | 0.197 | 0.019 | 0.032 | 0.002 | 23.245 |
|  | rs147708384 | -0.037 | 0.006 | 0.098 | 0.706 | 0.002 | 22.238 |
|  | rs2070746 | -0.014 | 0.287 | 0.017 | 0.416 | 0.001 | 20.177 |
|  | rs34791102 | 0.088 | 0.028 | 0.046 | 0.059 | 0.001 | 21.822 |
|  | rs374784992 | 0.001 | 0.924 | 0.029 | 0.975 | 0.002 | 20.066 |
|  | rs3792790 | -0.027 | 0.454 | 0.015 | 0.074 | 0.001 | 21.080 |
|  | rs4356912 | 0.002 | 0.715 | 0.017 | 0.906 | 0.001 | 19.810 |
|  | rs454265 | -0.008 | 0.681 | 0.016 | 0.624 | 0.001 | 20.466 |
|  | rs4554017 | 0.007 | 0.437 | 0.015 | 0.657 | 0.002 | 23.862 |
|  | rs4766578 | -0.057 | 0.570 | 0.015 | 0.000 | 0.003 | 36.817 |
|  | rs4907572 | -0.032 | 0.282 | 0.017 | 0.058 | 0.002 | 23.985 |
|  | rs62292952 | -0.020 | 0.870 | 0.023 | 0.368 | 0.011 | 160.788 |
|  | rs6486995 | -0.005 | 0.244 | 0.018 | 0.789 | 0.002 | 26.653 |
|  | rs6535499 | -0.054 | 0.036 | 0.041 | 0.189 | 0.001 | 19.983 |
|  | rs6870560 | -0.045 | 0.839 | 0.021 | 0.030 | 0.002 | 26.073 |
|  | rs7185085 | 0.425 | 0.999 | 0.248 | 0.086 | 0.002 | 19.630 |
|  | rs73257451 | -0.037 | 0.978 | 0.052 | 0.476 | 0.002 | 23.242 |
|  | rs74764632 | 0.061 | 0.938 | 0.031 | 0.053 | 0.001 | 21.051 |
|  | rs7595241 | 0.010 | 0.651 | 0.016 | 0.546 | 0.002 | 22.377 |
|  | rs76295077 | -0.014 | 0.905 | 0.026 | 0.587 | 0.001 | 19.903 |
|  | rs79086127 | -0.106 | 0.005 | 0.107 | 0.324 | 0.002 | 21.692 |
|  | rs9469127 | -0.085 | 0.903 | 0.025 | 0.001 | 0.021 | 310.837 |
| CCL20 | rs10207134 | -0.081 | 0.229 | 0.018 | 0.000 | 0.004 | 60.539 |
|  | rs1027232 | 0.034 | 0.151 | 0.021 | 0.112 | 0.001 | 20.285 |
|  | rs10837780 | -0.003 | 0.944 | 0.033 | 0.940 | 0.001 | 20.981 |
|  | rs111674022 | -0.016 | 0.091 | 0.026 | 0.536 | 0.001 | 20.989 |
|  | rs11242693 | -0.013 | 0.434 | 0.015 | 0.409 | 0.002 | 22.261 |
|  | rs1129474 | 0.001 | 0.548 | 0.015 | 0.970 | 0.002 | 22.797 |
|  | rs11582772 | -0.124 | 0.996 | 0.116 | 0.285 | 0.002 | 22.248 |
|  | rs117753190 | 0.042 | 0.989 | 0.074 | 0.572 | 0.002 | 26.552 |
|  | rs117900576 | -0.086 | 0.990 | 0.076 | 0.254 | 0.002 | 27.650 |
|  | rs118164045 | 0.052 | 0.959 | 0.038 | 0.175 | 0.001 | 20.138 |
|  | rs13049624 | -0.003 | 0.929 | 0.030 | 0.927 | 0.002 | 22.697 |
|  | rs13185268 | 0.001 | 0.918 | 0.028 | 0.984 | 0.001 | 20.835 |
|  | rs151092565 | -0.145 | 0.025 | 0.051 | 0.004 | 0.001 | 21.247 |
|  | rs2003431 | 0.008 | 0.048 | 0.037 | 0.830 | 0.002 | 21.683 |
|  | rs2796032 | 0.013 | 0.320 | 0.016 | 0.408 | 0.002 | 25.075 |
|  | rs34729167 | 0.015 | 0.198 | 0.019 | 0.442 | 0.001 | 20.022 |
|  | rs348389 | -0.019 | 0.354 | 0.016 | 0.242 | 0.001 | 19.671 |
|  | rs35757792 | -0.006 | 0.498 | 0.015 | 0.677 | 0.001 | 19.833 |
|  | rs35833676 | -0.003 | 0.124 | 0.023 | 0.890 | 0.002 | 23.455 |
|  | rs3798304 | 0.042 | 0.760 | 0.018 | 0.018 | 0.001 | 20.443 |
|  | rs41302923 | 0.028 | 0.973 | 0.047 | 0.551 | 0.001 | 21.933 |
|  | rs4981022 | 0.022 | 0.683 | 0.016 | 0.187 | 0.002 | 24.608 |
|  | rs55891514 | 0.091 | 0.957 | 0.037 | 0.015 | 0.001 | 20.100 |
|  | rs5769672 | 0.078 | 0.980 | 0.055 | 0.154 | 0.001 | 21.222 |
|  | rs61945278 | 0.010 | 0.023 | 0.051 | 0.841 | 0.001 | 19.663 |
|  | rs62456601 | 0.018 | 0.601 | 0.015 | 0.251 | 0.002 | 19.887 |
|  | rs6420556 | -0.007 | 0.178 | 0.020 | 0.735 | 0.002 | 21.696 |
|  | rs6498925 | -0.005 | 0.644 | 0.016 | 0.732 | 0.001 | 20.645 |
|  | rs73072893 | 0.018 | 0.966 | 0.042 | 0.669 | 0.002 | 23.756 |
|  | rs742493 | -0.023 | 0.903 | 0.026 | 0.369 | 0.004 | 62.188 |
|  | rs748503 | -0.011 | 0.288 | 0.017 | 0.528 | 0.001 | 19.860 |
|  | rs7749768 | -0.026 | 0.807 | 0.019 | 0.183 | 0.001 | 20.313 |
|  | rs9274623 | 0.009 | 0.141 | 0.022 | 0.681 | 0.002 | 26.366 |
|  | rs9357723 | 0.004 | 0.461 | 0.015 | 0.807 | 0.001 | 20.247 |
| CCL4 | rs10990301 | 0.033 | 0.891 | 0.024 | 0.178 | 0.001 | 20.881 |
|  | rs111476793 | -0.036 | 0.105 | 0.025 | 0.144 | 0.002 | 20.915 |
|  | rs112315344 | 0.074 | 0.012 | 0.069 | 0.284 | 0.002 | 21.768 |
|  | rs112356872 | -0.096 | 0.977 | 0.050 | 0.056 | 0.001 | 20.512 |
|  | rs112846979 | -0.114 | 0.008 | 0.084 | 0.173 | 0.001 | 20.618 |
|  | rs113010081 | -0.122 | 0.872 | 0.022 | 0.000 | 0.064 | 982.208 |
|  | rs1161092 | 0.023 | 0.630 | 0.016 | 0.143 | 0.002 | 25.803 |
|  | rs116193969 | 0.126 | 0.017 | 0.059 | 0.034 | 0.002 | 22.318 |
|  | rs11619402 | -0.024 | 0.851 | 0.021 | 0.260 | 0.001 | 20.068 |
|  | rs116309267 | -0.046 | 0.042 | 0.038 | 0.224 | 0.002 | 23.322 |
|  | rs13100427 | -0.001 | 0.561 | 0.015 | 0.972 | 0.001 | 19.726 |
|  | rs137896358 | -0.040 | 0.017 | 0.059 | 0.496 | 0.002 | 25.764 |
|  | rs140505982 | 0.115 | 0.010 | 0.079 | 0.146 | 0.002 | 30.551 |
|  | rs143922263 | 0.064 | 0.017 | 0.058 | 0.268 | 0.001 | 19.556 |
|  | rs143960014 | 0.181 | 0.004 | 0.129 | 0.162 | 0.002 | 22.037 |
|  | rs148518028 | 0.027 | 0.023 | 0.050 | 0.586 | 0.001 | 20.432 |
|  | rs1634503 | -0.020 | 0.179 | 0.020 | 0.320 | 0.002 | 29.140 |
|  | rs17692864 | 0.007 | 0.953 | 0.036 | 0.852 | 0.005 | 67.253 |
|  | rs17792183 | -0.007 | 0.876 | 0.023 | 0.756 | 0.001 | 20.141 |
|  | rs183450854 | -0.008 | 0.979 | 0.052 | 0.874 | 0.001 | 20.162 |
|  | rs200664920 | -0.009 | 0.969 | 0.044 | 0.835 | 0.002 | 27.414 |
|  | rs3860989 | -0.001 | 0.623 | 0.016 | 0.941 | 0.002 | 19.743 |
|  | rs42279 | -0.003 | 0.151 | 0.021 | 0.897 | 0.001 | 21.032 |
|  | rs56131468 | 0.004 | 0.176 | 0.020 | 0.841 | 0.002 | 21.897 |
|  | rs5743614 | 0.043 | 0.130 | 0.023 | 0.059 | 0.002 | 25.913 |
|  | rs6100282 | 0.000 | 0.161 | 0.021 | 0.999 | 0.002 | 20.219 |
|  | rs66822987 | -0.012 | 0.235 | 0.018 | 0.488 | 0.001 | 19.696 |
|  | rs72800563 | -0.126 | 0.990 | 0.078 | 0.104 | 0.002 | 22.268 |
|  | rs78044056 | -0.030 | 0.008 | 0.087 | 0.728 | 0.002 | 22.302 |
|  | rs8064426 | -0.001 | 0.217 | 0.018 | 0.949 | 0.038 | 562.680 |
|  | rs9818901 | 0.029 | 0.945 | 0.033 | 0.384 | 0.001 | 19.993 |
| CD6 | rs10792322 | -0.025 | 0.287 | 0.017 | 0.136 | 0.002 | 27.600 |
|  | rs10843957 | 0.020 | 0.584 | 0.015 | 0.183 | 0.001 | 20.440 |
|  | rs11230555 | -0.069 | 0.179 | 0.020 | 0.001 | 0.124 | 2026.060 |
|  | rs116557290 | 0.064 | 0.028 | 0.046 | 0.164 | 0.001 | 19.602 |
|  | rs12074680 | -0.042 | 0.023 | 0.050 | 0.400 | 0.002 | 22.390 |
|  | rs143374617 | 0.062 | 0.037 | 0.040 | 0.123 | 0.002 | 28.809 |
|  | rs144764321 | 0.075 | 0.019 | 0.055 | 0.170 | 0.001 | 20.662 |
|  | rs1483572 | -0.024 | 0.327 | 0.016 | 0.143 | 0.002 | 23.566 |
|  | rs150354514 | 0.012 | 0.967 | 0.042 | 0.776 | 0.002 | 19.965 |
|  | rs200729584 | 0.191 | 0.995 | 0.117 | 0.103 | 0.003 | 26.583 |
|  | rs2237995 | -0.023 | 0.051 | 0.035 | 0.508 | 0.019 | 277.689 |
|  | rs28509311 | -0.047 | 0.990 | 0.075 | 0.531 | 0.002 | 20.542 |
|  | rs35086654 | 0.004 | 0.004 | 0.133 | 0.973 | 0.002 | 21.835 |
|  | rs35634144 | 0.114 | 0.991 | 0.081 | 0.157 | 0.002 | 21.709 |
|  | rs4902621 | -0.036 | 0.195 | 0.019 | 0.061 | 0.001 | 19.782 |
|  | rs56294840 | -0.006 | 0.871 | 0.023 | 0.802 | 0.001 | 20.591 |
|  | rs597808 | 0.053 | 0.413 | 0.015 | 0.001 | 0.007 | 74.926 |
|  | rs60988380 | -0.029 | 0.110 | 0.024 | 0.233 | 0.002 | 24.481 |
|  | rs72969761 | 0.010 | 0.946 | 0.034 | 0.762 | 0.002 | 22.813 |
|  | rs74886615 | -0.013 | 0.655 | 0.016 | 0.423 | 0.002 | 28.440 |
|  | rs78007268 | -0.045 | 0.019 | 0.055 | 0.411 | 0.001 | 19.892 |
|  | rs8051133 | -0.069 | 0.046 | 0.036 | 0.060 | 0.001 | 20.901 |
|  | rs841332 | 0.022 | 0.805 | 0.019 | 0.255 | 0.001 | 21.916 |
|  | rs9691968 | 0.006 | 0.215 | 0.018 | 0.758 | 0.002 | 23.238 |
| CXCL10 | rs10481754 | -0.030 | 0.450 | 0.015 | 0.051 | 0.001 | 21.203 |
|  | rs1077965 | -0.028 | 0.558 | 0.015 | 0.063 | 0.001 | 21.407 |
|  | rs112202639 | -0.043 | 0.956 | 0.037 | 0.237 | 0.002 | 22.928 |
|  | rs115140093 | -0.044 | 0.982 | 0.057 | 0.437 | 0.010 | 152.443 |
|  | rs11645285 | -0.001 | 0.937 | 0.032 | 0.973 | 0.002 | 20.508 |
|  | rs117298099 | 0.054 | 0.017 | 0.059 | 0.359 | 0.001 | 20.068 |
|  | rs11825923 | -0.019 | 0.018 | 0.058 | 0.736 | 0.001 | 20.818 |
|  | rs12155428 | -0.011 | 0.007 | 0.095 | 0.910 | 0.002 | 22.150 |
|  | rs12174864 | -0.010 | 0.598 | 0.015 | 0.524 | 0.002 | 23.132 |
|  | rs12476448 | -0.003 | 0.465 | 0.015 | 0.862 | 0.001 | 21.721 |
|  | rs12629593 | -0.040 | 0.084 | 0.028 | 0.152 | 0.002 | 23.323 |
|  | rs12646113 | 0.043 | 0.250 | 0.018 | 0.014 | 0.003 | 51.600 |
|  | rs143796249 | -0.015 | 0.031 | 0.044 | 0.723 | 0.001 | 21.246 |
|  | rs144219691 | -0.021 | 0.740 | 0.017 | 0.231 | 0.002 | 19.784 |
|  | rs150519056 | 0.018 | 0.967 | 0.043 | 0.679 | 0.002 | 23.970 |
|  | rs1563171 | -0.014 | 0.692 | 0.016 | 0.391 | 0.001 | 21.726 |
|  | rs2164693 | 0.023 | 0.932 | 0.030 | 0.438 | 0.001 | 19.510 |
|  | rs2349689 | -0.009 | 0.210 | 0.020 | 0.639 | 0.002 | 23.142 |
|  | rs2523495 | 0.024 | 0.091 | 0.026 | 0.361 | 0.002 | 31.207 |
|  | rs3184504 | 0.052 | 0.407 | 0.015 | 0.001 | 0.006 | 75.452 |
|  | rs3846056 | -0.048 | 0.277 | 0.017 | 0.005 | 0.001 | 20.320 |
|  | rs4367871 | -0.002 | 0.546 | 0.015 | 0.899 | 0.002 | 22.699 |
|  | rs4804148 | -0.008 | 0.698 | 0.016 | 0.646 | 0.002 | 23.408 |
|  | rs55732300 | -0.021 | 0.964 | 0.041 | 0.615 | 0.001 | 22.087 |
|  | rs59465157 | -0.003 | 0.029 | 0.045 | 0.949 | 0.001 | 20.357 |
|  | rs61811733 | 0.010 | 0.960 | 0.039 | 0.791 | 0.001 | 19.641 |
|  | rs62208309 | -0.019 | 0.041 | 0.039 | 0.629 | 0.001 | 19.683 |
|  | rs72651343 | 0.217 | 0.993 | 0.093 | 0.020 | 0.007 | 96.972 |
|  | rs72682331 | -0.025 | 0.099 | 0.026 | 0.320 | 0.002 | 27.146 |
|  | rs741344 | -0.045 | 0.620 | 0.016 | 0.003 | 0.001 | 21.299 |
|  | rs74956615 | -0.142 | 0.035 | 0.043 | 0.001 | 0.002 | 21.332 |
|  | rs76484468 | 0.131 | 0.992 | 0.086 | 0.126 | 0.001 | 20.229 |
|  | rs820963 | 0.011 | 0.366 | 0.016 | 0.483 | 0.001 | 19.516 |
| FGF-5 | rs10788164 | 0.007 | 0.355 | 0.016 | 0.638 | 0.002 | 19.532 |
|  | rs11120686 | -0.003 | 0.615 | 0.016 | 0.831 | 0.002 | 22.242 |
|  | rs112371527 | -0.029 | 0.836 | 0.020 | 0.153 | 0.003 | 25.902 |
|  | rs112790107 | 0.039 | 0.027 | 0.047 | 0.404 | 0.002 | 22.967 |
|  | rs112819708 | 0.008 | 0.986 | 0.067 | 0.902 | 0.002 | 19.907 |
|  | rs11674185 | -0.047 | 0.352 | 0.016 | 0.003 | 0.002 | 20.956 |
|  | rs117253850 | 0.076 | 0.012 | 0.072 | 0.293 | 0.002 | 25.018 |
|  | rs11742072 | 0.029 | 0.926 | 0.029 | 0.316 | 0.002 | 20.807 |
|  | rs12005489 | -0.054 | 0.047 | 0.036 | 0.132 | 0.002 | 21.958 |
|  | rs12907074 | -0.006 | 0.188 | 0.019 | 0.757 | 0.002 | 20.355 |
|  | rs1412253 | -0.030 | 0.886 | 0.024 | 0.208 | 0.002 | 22.411 |
|  | rs1458038 | 0.016 | 0.346 | 0.016 | 0.301 | 0.125 | 1676.163 |
|  | rs146840556 | 0.061 | 0.036 | 0.041 | 0.141 | 0.002 | 19.834 |
|  | rs148332905 | -0.069 | 0.014 | 0.065 | 0.295 | 0.002 | 22.480 |
|  | rs148797749 | -0.062 | 0.047 | 0.036 | 0.086 | 0.002 | 22.303 |
|  | rs150524893 | 0.000 | 0.983 | 0.058 | 0.994 | 0.002 | 21.404 |
|  | rs188323153 | -0.089 | 0.949 | 0.034 | 0.009 | 0.002 | 20.564 |
|  | rs189846846 | 0.083 | 0.002 | 0.189 | 0.661 | 0.002 | 21.304 |
|  | rs192746810 | 0.054 | 0.039 | 0.039 | 0.174 | 0.002 | 21.742 |
|  | rs200900616 | -0.011 | 0.012 | 0.070 | 0.873 | 0.012 | 93.946 |
|  | rs2767000 | 0.052 | 0.905 | 0.026 | 0.046 | 0.002 | 20.711 |
|  | rs305897 | -0.014 | 0.070 | 0.030 | 0.627 | 0.002 | 22.644 |
|  | rs3796605 | 0.054 | 0.925 | 0.029 | 0.060 | 0.003 | 33.128 |
|  | rs4326078 | -0.027 | 0.214 | 0.018 | 0.137 | 0.002 | 23.551 |
|  | rs4910278 | -0.002 | 0.450 | 0.015 | 0.916 | 0.002 | 20.102 |
|  | rs4970834 | -0.003 | 0.196 | 0.019 | 0.881 | 0.002 | 22.117 |
|  | rs56391958 | -0.003 | 0.085 | 0.027 | 0.917 | 0.002 | 23.105 |
|  | rs62211039 | 0.231 | 0.997 | 0.144 | 0.108 | 0.002 | 19.900 |
|  | rs624605 | -0.004 | 0.439 | 0.015 | 0.769 | 0.002 | 21.657 |
|  | rs75924419 | 0.062 | 0.039 | 0.039 | 0.110 | 0.002 | 19.851 |
|  | rs77210320 | 0.050 | 0.952 | 0.036 | 0.159 | 0.002 | 22.081 |
|  | rs9433760 | -0.018 | 0.083 | 0.027 | 0.507 | 0.002 | 20.854 |
| FIt3L | rs10164255 | -0.013 | 0.449 | 0.015 | 0.414 | 0.001 | 21.112 |
|  | rs11212636 | -0.006 | 0.395 | 0.015 | 0.713 | 0.003 | 43.532 |
|  | rs112538528 | -0.059 | 0.978 | 0.051 | 0.247 | 0.001 | 19.667 |
|  | rs113877570 | 0.111 | 0.022 | 0.051 | 0.031 | 0.001 | 20.587 |
|  | rs117040659 | 0.012 | 0.004 | 0.120 | 0.920 | 0.001 | 20.242 |
|  | rs11961049 | -0.005 | 0.040 | 0.039 | 0.889 | 0.001 | 19.746 |
|  | rs12338549 | 0.037 | 0.148 | 0.021 | 0.079 | 0.001 | 20.930 |
|  | rs12609918 | 0.052 | 0.928 | 0.030 | 0.079 | 0.002 | 20.443 |
|  | rs1277312 | -0.004 | 0.470 | 0.015 | 0.773 | 0.001 | 21.220 |
|  | rs1379304 | 0.011 | 0.384 | 0.016 | 0.472 | 0.002 | 22.541 |
|  | rs141117375 | 0.085 | 0.016 | 0.061 | 0.164 | 0.001 | 21.057 |
|  | rs142870956 | -0.588 | 0.000 | 0.414 | 0.156 | 0.002 | 23.788 |
|  | rs144317085 | -0.024 | 0.943 | 0.033 | 0.467 | 0.003 | 46.351 |
|  | rs17272847 | 0.011 | 0.122 | 0.023 | 0.638 | 0.002 | 28.716 |
|  | rs184038883 | -0.076 | 0.007 | 0.094 | 0.419 | 0.002 | 20.390 |
|  | rs185979634 | 0.097 | 0.984 | 0.060 | 0.107 | 0.002 | 23.484 |
|  | rs1866051 | -0.009 | 0.442 | 0.015 | 0.550 | 0.003 | 42.075 |
|  | rs186975 | -0.048 | 0.357 | 0.016 | 0.003 | 0.001 | 21.670 |
|  | rs192857133 | -0.208 | 0.017 | 0.062 | 0.001 | 0.001 | 22.033 |
|  | rs218265 | 0.000 | 0.835 | 0.020 | 0.985 | 0.002 | 27.101 |
|  | rs2325555 | 0.027 | 0.856 | 0.022 | 0.221 | 0.001 | 20.001 |
|  | rs28533776 | -0.026 | 0.164 | 0.020 | 0.211 | 0.002 | 25.958 |
|  | rs35340580 | 0.044 | 0.028 | 0.046 | 0.342 | 0.002 | 20.830 |
|  | rs35977476 | -0.009 | 0.025 | 0.049 | 0.856 | 0.001 | 20.395 |
|  | rs374309 | -0.002 | 0.901 | 0.025 | 0.948 | 0.001 | 20.403 |
|  | rs4919613 | 0.009 | 0.124 | 0.023 | 0.711 | 0.001 | 20.005 |
|  | rs55633025 | -0.020 | 0.456 | 0.015 | 0.186 | 0.003 | 31.581 |
|  | rs55646869 | 0.011 | 0.559 | 0.015 | 0.450 | 0.001 | 20.794 |
|  | rs57191857 | 0.004 | 0.972 | 0.046 | 0.938 | 0.002 | 22.838 |
|  | rs61792394 | 0.011 | 0.962 | 0.040 | 0.786 | 0.002 | 21.545 |
|  | rs6453962 | -0.004 | 0.522 | 0.015 | 0.817 | 0.002 | 22.824 |
|  | rs67811337 | -0.039 | 0.056 | 0.034 | 0.248 | 0.002 | 20.360 |
|  | rs6925032 | -0.026 | 0.239 | 0.018 | 0.146 | 0.002 | 27.888 |
|  | rs72681653 | 0.099 | 0.965 | 0.041 | 0.016 | 0.002 | 23.955 |
|  | rs73000965 | -0.030 | 0.377 | 0.016 | 0.060 | 0.001 | 22.125 |
|  | rs74722523 | 0.098 | 0.998 | 0.172 | 0.570 | 0.002 | 21.558 |
|  | rs7624160 | 0.051 | 0.614 | 0.016 | 0.001 | 0.005 | 79.484 |
|  | rs7625643 | -0.022 | 0.540 | 0.015 | 0.150 | 0.002 | 22.430 |
|  | rs76428106 | 0.055 | 0.987 | 0.069 | 0.425 | 0.031 | 442.139 |
|  | rs7726159 | 0.016 | 0.325 | 0.016 | 0.320 | 0.006 | 86.565 |
|  | rs77584520 | -0.084 | 0.983 | 0.058 | 0.150 | 0.001 | 20.079 |
|  | rs78154669 | -0.014 | 0.102 | 0.025 | 0.578 | 0.001 | 19.879 |
|  | rs78723322 | -0.009 | 0.045 | 0.037 | 0.803 | 0.001 | 20.425 |
|  | rs871051 | 0.014 | 0.045 | 0.036 | 0.708 | 0.001 | 19.875 |
|  | rs9381118 | -0.021 | 0.226 | 0.018 | 0.240 | 0.002 | 22.350 |
|  | rs9554186 | -0.018 | 0.405 | 0.015 | 0.251 | 0.003 | 41.134 |
| IL-10 α | rs10091841 | 0.029 | 0.407 | 0.016 | 0.065 | 0.002 | 22.471 |
|  | rs12026492 | 0.022 | 0.213 | 0.019 | 0.236 | 0.002 | 20.652 |
|  | rs1406714 | 0.008 | 0.401 | 0.015 | 0.624 | 0.002 | 22.613 |
|  | rs143313574 | -0.032 | 0.026 | 0.048 | 0.509 | 0.002 | 21.149 |
|  | rs17832137 | -0.008 | 0.053 | 0.034 | 0.818 | 0.002 | 23.871 |
|  | rs180893431 | -0.068 | 0.997 | 0.159 | 0.667 | 0.002 | 21.993 |
|  | rs28408877 | -0.003 | 0.787 | 0.019 | 0.862 | 0.002 | 20.335 |
|  | rs286605 | 0.037 | 0.577 | 0.015 | 0.015 | 0.002 | 22.613 |
|  | rs3135932 | -0.060 | 0.798 | 0.019 | 0.001 | 0.002 | 25.234 |
|  | rs3777726 | -0.012 | 0.219 | 0.018 | 0.514 | 0.002 | 20.657 |
|  | rs41272625 | 0.038 | 0.102 | 0.025 | 0.127 | 0.002 | 20.982 |
|  | rs492616 | 0.002 | 0.828 | 0.020 | 0.938 | 0.002 | 19.515 |
|  | rs62262707 | 0.058 | 0.015 | 0.063 | 0.357 | 0.002 | 19.922 |
|  | rs6670799 | -0.080 | 0.097 | 0.026 | 0.002 | 0.002 | 24.482 |
|  | rs74719858 | 0.098 | 0.962 | 0.040 | 0.013 | 0.002 | 22.625 |
|  | rs79202194 | 0.007 | 0.093 | 0.026 | 0.791 | 0.002 | 19.648 |
|  | rs80137559 | 0.071 | 0.975 | 0.049 | 0.143 | 0.002 | 21.092 |
|  | rs9472032 | -0.054 | 0.112 | 0.024 | 0.023 | 0.002 | 20.457 |
|  | rs9548767 | 0.000 | 0.200 | 0.019 | 0.980 | 0.002 | 19.584 |
|  | rs958402 | -0.011 | 0.434 | 0.015 | 0.477 | 0.002 | 20.045 |
| IL12 β | rs10100578 | -0.002 | 0.477 | 0.015 | 0.921 | 0.001 | 19.979 |
|  | rs11039216 | 0.016 | 0.439 | 0.015 | 0.285 | 0.002 | 25.691 |
|  | rs11130215 | 0.015 | 0.729 | 0.017 | 0.374 | 0.003 | 44.645 |
|  | rs117888068 | -0.027 | 0.980 | 0.055 | 0.627 | 0.002 | 21.524 |
|  | rs12588969 | -0.011 | 0.792 | 0.019 | 0.554 | 0.007 | 104.283 |
|  | rs13095341 | 0.004 | 0.025 | 0.050 | 0.932 | 0.001 | 19.695 |
|  | rs1398590 | -0.037 | 0.021 | 0.053 | 0.491 | 0.002 | 29.448 |
|  | rs144411658 | -0.048 | 0.457 | 0.015 | 0.002 | 0.002 | 20.589 |
|  | rs144850787 | -0.017 | 0.992 | 0.086 | 0.839 | 0.002 | 31.858 |
|  | rs148549227 | -0.083 | 0.092 | 0.027 | 0.002 | 0.002 | 22.768 |
|  | rs149993822 | 0.035 | 0.153 | 0.022 | 0.103 | 0.002 | 25.723 |
|  | rs150590615 | 0.461 | 0.000 | 0.411 | 0.261 | 0.002 | 19.795 |
|  | rs17056751 | 0.088 | 0.941 | 0.033 | 0.008 | 0.002 | 27.164 |
|  | rs17500902 | 0.004 | 0.894 | 0.025 | 0.877 | 0.001 | 19.648 |
|  | rs17722688 | -0.021 | 0.722 | 0.017 | 0.204 | 0.003 | 42.792 |
|  | rs1950897 | -0.006 | 0.690 | 0.016 | 0.714 | 0.003 | 38.862 |
|  | rs201503938 | -0.015 | 0.157 | 0.021 | 0.476 | 0.002 | 23.600 |
|  | rs3130510 | 0.061 | 0.151 | 0.021 | 0.004 | 0.005 | 68.053 |
|  | rs3184504 | 0.052 | 0.407 | 0.015 | 0.001 | 0.009 | 103.369 |
|  | rs34169305 | 0.035 | 0.369 | 0.016 | 0.025 | 0.001 | 19.821 |
|  | rs3822030 | -0.020 | 0.631 | 0.016 | 0.198 | 0.003 | 20.462 |
|  | rs4244437 | 0.107 | 0.749 | 0.018 | 0.000 | 0.101 | 1647.733 |
|  | rs4783410 | -0.035 | 0.101 | 0.025 | 0.162 | 0.001 | 19.882 |
|  | rs5014495 | 0.012 | 0.064 | 0.032 | 0.712 | 0.002 | 21.929 |
|  | rs516978 | -0.001 | 0.372 | 0.016 | 0.936 | 0.002 | 34.921 |
|  | rs539598 | -0.002 | 0.215 | 0.018 | 0.914 | 0.002 | 27.133 |
|  | rs6750065 | -0.013 | 0.773 | 0.018 | 0.471 | 0.002 | 21.961 |
|  | rs6968201 | -0.015 | 0.480 | 0.015 | 0.320 | 0.002 | 27.317 |
|  | rs705705 | -0.025 | 0.319 | 0.016 | 0.119 | 0.003 | 34.831 |
|  | rs72993671 | 0.121 | 0.008 | 0.085 | 0.157 | 0.002 | 21.990 |
|  | rs73416208 | -0.034 | 0.088 | 0.027 | 0.206 | 0.002 | 36.044 |
|  | rs76322730 | 0.016 | 0.015 | 0.062 | 0.793 | 0.001 | 20.341 |
|  | rs76428106 | 0.055 | 0.987 | 0.069 | 0.425 | 0.003 | 40.525 |
|  | rs780679 | 0.000 | 0.247 | 0.018 | 0.979 | 0.002 | 24.997 |
|  | rs8026767 | 0.056 | 0.224 | 0.018 | 0.002 | 0.002 | 31.679 |
|  | rs9815073 | 0.011 | 0.329 | 0.016 | 0.516 | 0.033 | 484.271 |
|  | rs9965402 | 0.008 | 0.905 | 0.026 | 0.765 | 0.001 | 20.856 |
| TNFSF12 | rs10010472 | 0.001 | 0.732 | 0.017 | 0.969 | 0.001 | 19.680 |
|  | rs10818768 | 0.006 | 0.454 | 0.015 | 0.675 | 0.001 | 19.567 |
|  | rs10831650 | -0.003 | 0.289 | 0.017 | 0.853 | 0.002 | 30.643 |
|  | rs11136339 | 0.036 | 0.395 | 0.015 | 0.019 | 0.002 | 25.243 |
|  | rs111641307 | -0.037 | 0.995 | 0.110 | 0.736 | 0.002 | 21.192 |
|  | rs11676293 | 0.002 | 0.291 | 0.017 | 0.881 | 0.001 | 20.776 |
|  | rs11738159 | 0.026 | 0.409 | 0.015 | 0.091 | 0.002 | 23.507 |
|  | rs117550445 | -0.061 | 0.970 | 0.044 | 0.163 | 0.002 | 24.207 |
|  | rs117799464 | -0.032 | 0.985 | 0.063 | 0.614 | 0.001 | 21.265 |
|  | rs11934678 | -0.003 | 0.092 | 0.027 | 0.905 | 0.001 | 19.755 |
|  | rs12145644 | -0.003 | 0.259 | 0.017 | 0.883 | 0.002 | 26.581 |
|  | rs12726054 | -0.007 | 0.122 | 0.023 | 0.778 | 0.002 | 22.852 |
|  | rs13107325 | 0.003 | 0.014 | 0.064 | 0.960 | 0.005 | 73.761 |
|  | rs137861803 | 0.020 | 0.733 | 0.017 | 0.238 | 0.002 | 20.581 |
|  | rs144115221 | 0.041 | 0.018 | 0.057 | 0.480 | 0.001 | 20.808 |
|  | rs145667392 | -0.005 | 0.027 | 0.048 | 0.909 | 0.002 | 20.664 |
|  | rs147054593 | 0.000 | 0.681 | 0.016 | 0.975 | 0.002 | 20.790 |
|  | rs1564995 | 0.002 | 0.558 | 0.015 | 0.918 | 0.001 | 20.658 |
|  | rs16879825 | -0.018 | 0.934 | 0.030 | 0.554 | 0.002 | 21.413 |
|  | rs1965399 | -0.004 | 0.973 | 0.047 | 0.939 | 0.001 | 21.953 |
|  | rs2163333 | -0.060 | 0.960 | 0.039 | 0.118 | 0.002 | 26.445 |
|  | rs2220833 | -0.029 | 0.606 | 0.015 | 0.065 | 0.001 | 19.680 |
|  | rs2738752 | -0.021 | 0.645 | 0.016 | 0.190 | 0.002 | 20.986 |
|  | rs32923 | -0.013 | 0.721 | 0.017 | 0.449 | 0.001 | 20.144 |
|  | rs34790908 | 0.031 | 0.237 | 0.018 | 0.084 | 0.018 | 267.502 |
|  | rs3821473 | 0.001 | 0.259 | 0.017 | 0.964 | 0.001 | 20.914 |
|  | rs4882710 | -0.002 | 0.412 | 0.015 | 0.890 | 0.002 | 25.843 |
|  | rs56023600 | -0.017 | 0.845 | 0.021 | 0.417 | 0.001 | 21.177 |
|  | rs57053056 | -0.026 | 0.088 | 0.027 | 0.327 | 0.001 | 20.223 |
|  | rs579459 | 0.011 | 0.776 | 0.018 | 0.555 | 0.004 | 48.903 |
|  | rs7019909 | -0.024 | 0.120 | 0.023 | 0.295 | 0.002 | 21.598 |
|  | rs7149501 | -0.129 | 0.996 | 0.124 | 0.297 | 0.002 | 19.626 |
|  | rs73133996 | -0.011 | 0.477 | 0.015 | 0.482 | 0.003 | 45.781 |
|  | rs73590361 | -0.015 | 0.146 | 0.021 | 0.475 | 0.001 | 21.352 |
|  | rs7503751 | 0.009 | 0.050 | 0.035 | 0.806 | 0.002 | 33.909 |
|  | rs76047473 | 0.081 | 0.046 | 0.036 | 0.027 | 0.001 | 21.234 |
|  | rs76133364 | 0.036 | 0.049 | 0.035 | 0.312 | 0.002 | 26.397 |
|  | rs76188822 | 0.093 | 0.977 | 0.051 | 0.068 | 0.001 | 20.068 |
|  | rs76854434 | 0.047 | 0.012 | 0.070 | 0.503 | 0.001 | 19.867 |
|  | rs78254232 | 0.043 | 0.992 | 0.085 | 0.610 | 0.001 | 21.367 |
|  | rs8116972 | 0.018 | 0.066 | 0.030 | 0.556 | 0.001 | 20.397 |
|  | rs870526 | -0.003 | 0.610 | 0.015 | 0.828 | 0.001 | 19.854 |
|  | rs9470247 | -0.026 | 0.929 | 0.030 | 0.379 | 0.001 | 19.555 |
|  | rs9842051 | 0.014 | 0.815 | 0.020 | 0.493 | 0.005 | 72.483 |
| **UC** |  |  |  |  |  |  |  |
| CCL11 | rs10911109 | 0.013 | 0.200 | 0.023 | 0.581 | 0.002 | 19.882 |
|  | rs114744647 | -0.018 | 0.983 | 0.072 | 0.806 | 0.001 | 21.823 |
|  | rs12075 | -0.051 | 0.532 | 0.019 | 0.006 | 0.003 | 43.161 |
|  | rs12453306 | -0.011 | 0.903 | 0.032 | 0.736 | 0.002 | 19.916 |
|  | rs139476527 | -0.035 | 0.002 | 0.245 | 0.886 | 0.002 | 21.744 |
|  | rs139597204 | 0.037 | 0.004 | 0.159 | 0.818 | 0.002 | 22.327 |
|  | rs140965534 | 0.074 | 0.015 | 0.079 | 0.351 | 0.001 | 19.725 |
|  | rs148554043 | -0.099 | 0.029 | 0.057 | 0.081 | 0.002 | 20.267 |
|  | rs151240414 | -0.051 | 0.009 | 0.099 | 0.608 | 0.002 | 20.477 |
|  | rs1564995 | 0.002 | 0.558 | 0.019 | 0.920 | 0.002 | 21.609 |
|  | rs170520 | 0.000 | 0.299 | 0.020 | 0.996 | 0.001 | 19.640 |
|  | rs2112920 | 0.015 | 0.835 | 0.025 | 0.538 | 0.002 | 26.070 |
|  | rs2201150 | -0.084 | 0.383 | 0.019 | 0.000 | 0.003 | 40.726 |
|  | rs2228467 | -0.058 | 0.924 | 0.035 | 0.098 | 0.015 | 217.173 |
|  | rs34536806 | -0.018 | 0.929 | 0.037 | 0.618 | 0.002 | 23.903 |
|  | rs35543857 | -0.016 | 0.906 | 0.032 | 0.611 | 0.002 | 21.749 |
|  | rs55843637 | 0.012 | 0.506 | 0.019 | 0.517 | 0.001 | 21.036 |
|  | rs6894241 | -0.021 | 0.240 | 0.022 | 0.327 | 0.002 | 22.925 |
|  | rs73171545 | 0.014 | 0.115 | 0.029 | 0.633 | 0.001 | 21.196 |
|  | rs73209015 | 0.006 | 0.100 | 0.031 | 0.840 | 0.002 | 23.332 |
|  | rs757973 | -0.025 | 0.069 | 0.037 | 0.491 | 0.006 | 95.359 |
|  | rs79432525 | -0.083 | 0.991 | 0.097 | 0.392 | 0.001 | 20.892 |
|  | rs79684468 | -0.089 | 0.044 | 0.046 | 0.051 | 0.002 | 24.569 |
|  | rs79722574 | 0.060 | 0.140 | 0.027 | 0.026 | 0.004 | 60.652 |
|  | rs8068185 | 0.009 | 0.869 | 0.028 | 0.741 | 0.001 | 20.928 |
|  | rs9303380 | -0.005 | 0.042 | 0.047 | 0.909 | 0.001 | 19.885 |
|  | rs9940390 | -0.014 | 0.685 | 0.020 | 0.478 | 0.003 | 37.822 |
|  | rs9976282 | 0.137 | 0.020 | 0.066 | 0.039 | 0.002 | 23.653 |
| CCL4 | rs10990301 | 0.055 | 0.891 | 0.030 | 0.068 | 0.001 | 20.881 |
|  | rs111476793 | -0.008 | 0.105 | 0.031 | 0.785 | 0.002 | 20.915 |
|  | rs112315344 | 0.088 | 0.012 | 0.085 | 0.300 | 0.002 | 21.768 |
|  | rs112356872 | -0.137 | 0.977 | 0.062 | 0.026 | 0.001 | 20.512 |
|  | rs112846979 | -0.188 | 0.008 | 0.104 | 0.070 | 0.001 | 20.618 |
|  | rs113010081 | -0.149 | 0.872 | 0.027 | 0.000 | 0.064 | 982.208 |
|  | rs1161092 | 0.014 | 0.630 | 0.019 | 0.469 | 0.002 | 25.803 |
|  | rs116193969 | 0.101 | 0.017 | 0.073 | 0.165 | 0.002 | 22.318 |
|  | rs11619402 | -0.029 | 0.851 | 0.026 | 0.271 | 0.001 | 20.068 |
|  | rs116309267 | -0.058 | 0.041 | 0.047 | 0.214 | 0.002 | 23.322 |
|  | rs13100427 | -0.019 | 0.561 | 0.019 | 0.319 | 0.001 | 19.726 |
|  | rs137896358 | -0.073 | 0.017 | 0.073 | 0.319 | 0.002 | 25.764 |
|  | rs140505982 | 0.138 | 0.010 | 0.096 | 0.151 | 0.002 | 30.551 |
|  | rs143922263 | 0.097 | 0.017 | 0.072 | 0.177 | 0.001 | 19.556 |
|  | rs143960014 | 0.375 | 0.004 | 0.161 | 0.020 | 0.002 | 22.037 |
|  | rs148518028 | 0.061 | 0.023 | 0.062 | 0.323 | 0.001 | 20.432 |
|  | rs1634503 | -0.019 | 0.179 | 0.024 | 0.429 | 0.002 | 29.140 |
|  | rs17692864 | 0.042 | 0.953 | 0.044 | 0.333 | 0.005 | 67.253 |
|  | rs17792183 | 0.029 | 0.876 | 0.028 | 0.313 | 0.001 | 20.141 |
|  | rs183450854 | -0.065 | 0.979 | 0.064 | 0.311 | 0.001 | 20.162 |
|  | rs200664920 | 0.029 | 0.969 | 0.054 | 0.599 | 0.002 | 27.414 |
|  | rs3860989 | -0.002 | 0.623 | 0.019 | 0.926 | 0.002 | 19.743 |
|  | rs42279 | -0.018 | 0.151 | 0.026 | 0.500 | 0.001 | 21.032 |
|  | rs56131468 | 0.005 | 0.176 | 0.024 | 0.826 | 0.002 | 21.897 |
|  | rs5743614 | 0.060 | 0.130 | 0.028 | 0.033 | 0.002 | 25.913 |
|  | rs6100282 | 0.026 | 0.161 | 0.025 | 0.308 | 0.002 | 20.219 |
|  | rs66822987 | -0.025 | 0.235 | 0.022 | 0.262 | 0.001 | 19.696 |
|  | rs72800563 | 0.000 | 0.990 | 0.096 | 0.997 | 0.002 | 22.268 |
|  | rs78044056 | -0.121 | 0.008 | 0.107 | 0.255 | 0.002 | 22.302 |
|  | rs8064426 | 0.018 | 0.217 | 0.023 | 0.419 | 0.038 | 562.680 |
|  | rs9818901 | 0.061 | 0.945 | 0.041 | 0.136 | 0.001 | 19.993 |
| FGF-5 | rs10788164 | 0.025 | 0.355 | 0.019 | 0.192 | 0.002 | 19.532 |
|  | rs11120686 | 0.005 | 0.615 | 0.019 | 0.809 | 0.002 | 22.242 |
|  | rs112371527 | -0.027 | 0.836 | 0.025 | 0.278 | 0.003 | 25.902 |
|  | rs112790107 | 0.076 | 0.027 | 0.057 | 0.184 | 0.002 | 22.967 |
|  | rs112819708 | 0.060 | 0.986 | 0.083 | 0.464 | 0.002 | 19.907 |
|  | rs11674185 | -0.030 | 0.352 | 0.020 | 0.120 | 0.002 | 20.956 |
|  | rs117253850 | 0.109 | 0.012 | 0.089 | 0.219 | 0.002 | 25.018 |
|  | rs11742072 | 0.039 | 0.926 | 0.036 | 0.277 | 0.002 | 20.807 |
|  | rs12005489 | -0.050 | 0.047 | 0.044 | 0.253 | 0.002 | 21.958 |
|  | rs12907074 | -0.005 | 0.188 | 0.024 | 0.829 | 0.002 | 20.355 |
|  | rs1412253 | -0.044 | 0.886 | 0.029 | 0.129 | 0.002 | 22.411 |
|  | rs1458038 | 0.031 | 0.346 | 0.020 | 0.117 | 0.125 | 1676.163 |
|  | rs146840556 | 0.089 | 0.036 | 0.051 | 0.080 | 0.002 | 19.834 |
|  | rs148332905 | -0.123 | 0.014 | 0.081 | 0.128 | 0.002 | 22.480 |
|  | rs148797749 | -0.073 | 0.047 | 0.044 | 0.096 | 0.002 | 22.303 |
|  | rs150524893 | -0.004 | 0.983 | 0.072 | 0.961 | 0.002 | 21.404 |
|  | rs188323153 | -0.095 | 0.949 | 0.043 | 0.028 | 0.002 | 20.564 |
|  | rs189846846 | 0.112 | 0.002 | 0.232 | 0.629 | 0.002 | 21.304 |
|  | rs192746810 | 0.044 | 0.039 | 0.048 | 0.360 | 0.002 | 21.742 |
|  | rs200900616 | -0.010 | 0.012 | 0.086 | 0.908 | 0.012 | 93.946 |
|  | rs2767000 | 0.027 | 0.905 | 0.032 | 0.398 | 0.002 | 20.711 |
|  | rs305897 | -0.018 | 0.070 | 0.036 | 0.619 | 0.002 | 22.644 |
|  | rs3796605 | 0.046 | 0.925 | 0.035 | 0.195 | 0.003 | 33.128 |
|  | rs4326078 | -0.023 | 0.215 | 0.023 | 0.318 | 0.002 | 23.551 |
|  | rs4910278 | 0.008 | 0.450 | 0.019 | 0.679 | 0.002 | 20.102 |
|  | rs4970834 | 0.012 | 0.196 | 0.023 | 0.623 | 0.002 | 22.117 |
|  | rs56391958 | 0.010 | 0.085 | 0.033 | 0.760 | 0.002 | 23.105 |
|  | rs62211039 | 0.167 | 0.997 | 0.179 | 0.350 | 0.002 | 19.900 |
|  | rs624605 | -0.011 | 0.439 | 0.019 | 0.554 | 0.002 | 21.657 |
|  | rs75924419 | 0.032 | 0.039 | 0.048 | 0.507 | 0.002 | 19.851 |
|  | rs77210320 | 0.072 | 0.952 | 0.044 | 0.098 | 0.002 | 22.081 |
|  | rs9433760 | -0.066 | 0.083 | 0.034 | 0.052 | 0.002 | 20.854 |
| FIt3L | rs10164255 | -0.018 | 0.449 | 0.019 | 0.339 | 0.001 | 21.112 |
|  | rs11212636 | 0.011 | 0.395 | 0.019 | 0.577 | 0.003 | 43.532 |
|  | rs112538528 | -0.028 | 0.978 | 0.063 | 0.651 | 0.001 | 19.667 |
|  | rs113877570 | 0.119 | 0.022 | 0.064 | 0.060 | 0.001 | 20.587 |
|  | rs117040659 | -0.001 | 0.004 | 0.148 | 0.997 | 0.001 | 20.242 |
|  | rs11961049 | 0.042 | 0.040 | 0.048 | 0.377 | 0.001 | 19.746 |
|  | rs12338549 | -0.009 | 0.148 | 0.026 | 0.726 | 0.001 | 20.930 |
|  | rs12609918 | 0.011 | 0.928 | 0.036 | 0.752 | 0.002 | 20.443 |
|  | rs1277312 | -0.012 | 0.470 | 0.019 | 0.520 | 0.001 | 21.220 |
|  | rs1379304 | -0.004 | 0.384 | 0.019 | 0.824 | 0.002 | 22.541 |
|  | rs141117375 | 0.067 | 0.016 | 0.075 | 0.376 | 0.001 | 21.057 |
|  | rs142870956 | -0.843 | 0.000 | 0.505 | 0.095 | 0.002 | 23.788 |
|  | rs144317085 | 0.025 | 0.943 | 0.040 | 0.535 | 0.003 | 46.351 |
|  | rs17272847 | -0.010 | 0.122 | 0.029 | 0.720 | 0.002 | 28.716 |
|  | rs184038883 | -0.102 | 0.007 | 0.116 | 0.380 | 0.002 | 20.390 |
|  | rs185979634 | 0.181 | 0.984 | 0.074 | 0.014 | 0.002 | 23.484 |
|  | rs1866051 | -0.024 | 0.442 | 0.019 | 0.200 | 0.003 | 42.075 |
|  | rs186975 | -0.077 | 0.357 | 0.020 | 0.000 | 0.001 | 21.670 |
|  | rs192857133 | -0.168 | 0.017 | 0.072 | 0.019 | 0.001 | 22.033 |
|  | rs218265 | -0.008 | 0.835 | 0.025 | 0.753 | 0.002 | 27.101 |
|  | rs2325555 | 0.018 | 0.856 | 0.027 | 0.496 | 0.001 | 20.001 |
|  | rs28533776 | 0.006 | 0.164 | 0.025 | 0.801 | 0.002 | 25.958 |
|  | rs35340580 | 0.104 | 0.028 | 0.057 | 0.068 | 0.002 | 20.830 |
|  | rs35977476 | 0.047 | 0.025 | 0.061 | 0.436 | 0.001 | 20.395 |
|  | rs374309 | -0.033 | 0.901 | 0.031 | 0.283 | 0.001 | 20.403 |
|  | rs4919613 | 0.020 | 0.124 | 0.028 | 0.476 | 0.001 | 20.005 |
|  | rs55633025 | -0.032 | 0.455 | 0.019 | 0.085 | 0.003 | 31.581 |
|  | rs55646869 | 0.019 | 0.559 | 0.019 | 0.309 | 0.001 | 20.794 |
|  | rs57191857 | -0.029 | 0.972 | 0.057 | 0.603 | 0.002 | 22.838 |
|  | rs61792394 | -0.047 | 0.962 | 0.049 | 0.341 | 0.002 | 21.545 |
|  | rs6453962 | -0.008 | 0.522 | 0.019 | 0.676 | 0.002 | 22.824 |
|  | rs67811337 | -0.037 | 0.056 | 0.041 | 0.370 | 0.002 | 20.360 |
|  | rs6925032 | -0.044 | 0.239 | 0.022 | 0.046 | 0.002 | 27.888 |
|  | rs72681653 | 0.082 | 0.965 | 0.050 | 0.105 | 0.002 | 23.955 |
|  | rs73000965 | -0.017 | 0.377 | 0.019 | 0.379 | 0.001 | 22.125 |
|  | rs74722523 | 0.029 | 0.998 | 0.211 | 0.891 | 0.002 | 21.558 |
|  | rs7624160 | 0.068 | 0.614 | 0.019 | 0.000 | 0.005 | 79.484 |
|  | rs7625643 | 0.001 | 0.540 | 0.019 | 0.943 | 0.002 | 22.430 |
|  | rs76428106 | 0.053 | 0.987 | 0.085 | 0.534 | 0.031 | 442.139 |
|  | rs7726159 | 0.022 | 0.325 | 0.020 | 0.260 | 0.006 | 86.565 |
|  | rs77584520 | -0.062 | 0.983 | 0.072 | 0.385 | 0.001 | 20.079 |
|  | rs78154669 | 0.005 | 0.102 | 0.031 | 0.876 | 0.001 | 19.879 |
|  | rs78723322 | -0.020 | 0.045 | 0.045 | 0.665 | 0.001 | 20.425 |
|  | rs871051 | 0.008 | 0.045 | 0.045 | 0.858 | 0.001 | 19.875 |
|  | rs9381118 | -0.026 | 0.226 | 0.022 | 0.236 | 0.002 | 22.350 |
|  | rs9554186 | -0.019 | 0.405 | 0.019 | 0.306 | 0.003 | 41.134 |
| IL12 β | rs10100578 | -0.003 | 0.477 | 0.019 | 0.872 | 0.001 | 19.979 |
|  | rs11039216 | 0.017 | 0.439 | 0.019 | 0.355 | 0.002 | 25.691 |
|  | rs11130215 | -0.020 | 0.729 | 0.021 | 0.333 | 0.003 | 44.645 |
|  | rs117888068 | -0.031 | 0.980 | 0.067 | 0.650 | 0.002 | 21.524 |
|  | rs12588969 | -0.016 | 0.792 | 0.023 | 0.493 | 0.007 | 104.283 |
|  | rs13095341 | -0.011 | 0.025 | 0.061 | 0.855 | 0.001 | 19.695 |
|  | rs1398590 | -0.051 | 0.021 | 0.065 | 0.436 | 0.002 | 29.448 |
|  | rs144411658 | -0.045 | 0.458 | 0.019 | 0.015 | 0.002 | 20.589 |
|  | rs144850787 | -0.026 | 0.992 | 0.106 | 0.803 | 0.002 | 31.858 |
|  | rs148549227 | -0.113 | 0.092 | 0.033 | 0.001 | 0.002 | 22.768 |
|  | rs149993822 | 0.047 | 0.153 | 0.026 | 0.074 | 0.002 | 25.723 |
|  | rs150590615 | 0.383 | 0.000 | 0.504 | 0.447 | 0.002 | 19.795 |
|  | rs17056751 | 0.127 | 0.941 | 0.041 | 0.002 | 0.002 | 27.164 |
|  | rs17500902 | 0.000 | 0.894 | 0.030 | 0.992 | 0.001 | 19.648 |
|  | rs17722688 | -0.024 | 0.722 | 0.021 | 0.253 | 0.003 | 42.792 |
|  | rs1950897 | 0.006 | 0.690 | 0.020 | 0.754 | 0.003 | 38.862 |
|  | rs201503938 | -0.038 | 0.157 | 0.026 | 0.136 | 0.002 | 23.600 |
|  | rs3130510 | 0.058 | 0.151 | 0.026 | 0.026 | 0.005 | 68.053 |
|  | rs3184504 | 0.047 | 0.408 | 0.019 | 0.014 | 0.009 | 103.369 |
|  | rs34169305 | 0.025 | 0.369 | 0.019 | 0.196 | 0.001 | 19.821 |
|  | rs3822030 | -0.030 | 0.631 | 0.019 | 0.124 | 0.003 | 20.462 |
|  | rs4244437 | 0.098 | 0.749 | 0.022 | 0.000 | 0.101 | 1647.733 |
|  | rs4783410 | -0.026 | 0.101 | 0.031 | 0.393 | 0.001 | 19.882 |
|  | rs5014495 | -0.036 | 0.064 | 0.039 | 0.364 | 0.002 | 21.929 |
|  | rs516978 | -0.018 | 0.372 | 0.019 | 0.353 | 0.002 | 34.921 |
|  | rs539598 | -0.008 | 0.215 | 0.023 | 0.718 | 0.002 | 27.133 |
|  | rs6750065 | -0.005 | 0.773 | 0.022 | 0.829 | 0.002 | 21.961 |
|  | rs6968201 | -0.005 | 0.480 | 0.019 | 0.776 | 0.002 | 27.317 |
|  | rs705705 | -0.013 | 0.319 | 0.020 | 0.504 | 0.003 | 34.831 |
|  | rs72993671 | 0.033 | 0.008 | 0.105 | 0.752 | 0.002 | 21.990 |
|  | rs73416208 | -0.050 | 0.088 | 0.033 | 0.131 | 0.002 | 36.044 |
|  | rs76322730 | 0.034 | 0.015 | 0.076 | 0.661 | 0.001 | 20.341 |
|  | rs76428106 | 0.053 | 0.987 | 0.085 | 0.534 | 0.003 | 40.525 |
|  | rs780679 | 0.041 | 0.247 | 0.022 | 0.055 | 0.002 | 24.997 |
|  | rs8026767 | 0.059 | 0.223 | 0.022 | 0.007 | 0.002 | 31.679 |
|  | rs9815073 | 0.003 | 0.329 | 0.020 | 0.880 | 0.033 | 484.271 |
|  | rs9965402 | 0.017 | 0.905 | 0.032 | 0.601 | 0.001 | 20.856 |
| MCP-1 | rs11214549 | -0.055 | 0.054 | 0.041 | 0.188 | 0.001 | 19.795 |
|  | rs117265389 | -0.035 | 0.030 | 0.055 | 0.518 | 0.001 | 21.152 |
|  | rs117317477 | 0.057 | 0.049 | 0.043 | 0.184 | 0.002 | 21.008 |
|  | rs117510656 | -0.091 | 0.977 | 0.062 | 0.146 | 0.001 | 21.187 |
|  | rs11870164 | -0.092 | 0.289 | 0.021 | 0.000 | 0.002 | 33.368 |
|  | rs12075 | -0.051 | 0.532 | 0.019 | 0.006 | 0.015 | 224.704 |
|  | rs12367122 | -0.015 | 0.174 | 0.025 | 0.540 | 0.002 | 21.599 |
|  | rs142018247 | 0.065 | 0.029 | 0.055 | 0.243 | 0.001 | 19.782 |
|  | rs146186562 | 0.070 | 0.967 | 0.052 | 0.179 | 0.001 | 21.283 |
|  | rs148307902 | 0.005 | 0.012 | 0.089 | 0.953 | 0.002 | 22.515 |
|  | rs149604038 | -0.096 | 0.989 | 0.090 | 0.288 | 0.002 | 25.593 |
|  | rs1788443 | 0.009 | 0.468 | 0.019 | 0.642 | 0.001 | 20.361 |
|  | rs181998213 | -0.153 | 0.954 | 0.043 | 0.000 | 0.002 | 28.692 |
|  | rs1846616 | -0.094 | 0.353 | 0.020 | 0.000 | 0.004 | 51.736 |
|  | rs190108694 | -0.113 | 0.018 | 0.071 | 0.115 | 0.002 | 20.975 |
|  | rs207311 | 0.007 | 0.641 | 0.020 | 0.725 | 0.001 | 21.113 |
|  | rs2544916 | -0.055 | 0.975 | 0.060 | 0.357 | 0.001 | 20.395 |
|  | rs34292320 | 0.009 | 0.261 | 0.021 | 0.672 | 0.002 | 26.052 |
|  | rs352082 | 0.008 | 0.075 | 0.036 | 0.822 | 0.001 | 19.595 |
|  | rs35728689 | -0.027 | 0.079 | 0.035 | 0.433 | 0.015 | 230.150 |
|  | rs4682862 | -0.009 | 0.652 | 0.020 | 0.655 | 0.002 | 22.288 |
|  | rs72730728 | 0.021 | 0.939 | 0.039 | 0.588 | 0.001 | 20.713 |
|  | rs74794857 | 0.003 | 0.376 | 0.019 | 0.869 | 0.002 | 19.509 |
|  | rs7619350 | -0.018 | 0.125 | 0.028 | 0.517 | 0.002 | 24.060 |
|  | rs77128226 | -0.003 | 0.055 | 0.041 | 0.939 | 0.002 | 23.773 |
|  | rs77344300 | -0.049 | 0.045 | 0.045 | 0.277 | 0.002 | 22.212 |
|  | rs79697698 | -0.071 | 0.050 | 0.042 | 0.095 | 0.002 | 28.397 |
|  | rs9324105 | -0.016 | 0.151 | 0.026 | 0.537 | 0.001 | 19.609 |
|  | rs9811380 | 0.043 | 0.722 | 0.021 | 0.038 | 0.001 | 19.871 |
|  | rs9942602 | 0.000 | 0.399 | 0.019 | 0.997 | 0.002 | 27.896 |
| PD-L1 | rs113302946 | 0.013 | 0.002 | 0.230 | 0.955 | 0.001 | 20.240 |
|  | rs12539643 | -0.050 | 0.951 | 0.043 | 0.249 | 0.002 | 22.088 |
|  | rs1280389 | 0.006 | 0.334 | 0.020 | 0.776 | 0.001 | 20.750 |
|  | rs146328951 | -0.036 | 0.002 | 0.237 | 0.878 | 0.003 | 24.604 |
|  | rs151255930 | 0.054 | 0.005 | 0.135 | 0.686 | 0.001 | 20.143 |
|  | rs17256127 | -0.019 | 0.236 | 0.022 | 0.395 | 0.002 | 20.830 |
|  | rs181758547 | -0.057 | 0.009 | 0.099 | 0.567 | 0.002 | 19.806 |
|  | rs2019137 | 0.006 | 0.417 | 0.019 | 0.764 | 0.001 | 19.776 |
|  | rs2283438 | -0.006 | 0.427 | 0.019 | 0.745 | 0.002 | 23.117 |
|  | rs4974444 | 0.005 | 0.863 | 0.027 | 0.843 | 0.002 | 21.493 |
|  | rs60830384 | 0.079 | 0.941 | 0.039 | 0.045 | 0.002 | 28.541 |
|  | rs62373966 | -0.030 | 0.903 | 0.032 | 0.338 | 0.002 | 26.558 |
|  | rs6917605 | -0.046 | 0.452 | 0.019 | 0.013 | 0.002 | 22.806 |
|  | rs7026097 | -0.021 | 0.193 | 0.024 | 0.374 | 0.002 | 27.036 |
|  | rs7125631 | 0.024 | 0.724 | 0.021 | 0.252 | 0.002 | 22.634 |
|  | rs7137828 | -0.044 | 0.586 | 0.019 | 0.019 | 0.002 | 21.868 |
|  | rs72823987 | 0.016 | 0.947 | 0.042 | 0.700 | 0.001 | 20.308 |
|  | rs7304709 | -0.034 | 0.612 | 0.019 | 0.071 | 0.001 | 21.063 |
|  | rs73072534 | -0.034 | 0.045 | 0.045 | 0.451 | 0.001 | 19.730 |
|  | rs75510105 | -0.026 | 0.013 | 0.085 | 0.758 | 0.002 | 25.205 |
|  | rs79045618 | -0.011 | 0.058 | 0.040 | 0.776 | 0.002 | 24.964 |
|  | rs7936674 | -0.039 | 0.562 | 0.019 | 0.039 | 0.001 | 19.702 |
|  | rs7945680 | -0.019 | 0.558 | 0.019 | 0.324 | 0.002 | 28.628 |
|  | rs822335 | 0.086 | 0.353 | 0.019 | 0.000 | 0.008 | 116.444 |
| **CD** |  |  |  |  |  |  |  |
| Axin-1 | rs10860902 | 0.002 | 0.736 | 0.036 | 0.950 | 0.002 | 19.558 |
|  | rs11202576 | -0.016 | 0.811 | 0.040 | 0.694 | 0.002 | 19.882 |
|  | rs113946880 | 0.168 | 0.035 | 0.086 | 0.051 | 0.002 | 20.632 |
|  | rs115455827 | 0.028 | 0.926 | 0.061 | 0.642 | 0.002 | 22.504 |
|  | rs117097664 | 0.253 | 0.018 | 0.118 | 0.033 | 0.002 | 21.917 |
|  | rs148317708 | -0.178 | 0.010 | 0.158 | 0.260 | 0.002 | 25.474 |
|  | rs2470622 | 0.012 | 0.922 | 0.059 | 0.838 | 0.002 | 25.787 |
|  | rs2691530 | -0.039 | 0.560 | 0.032 | 0.219 | 0.002 | 25.373 |
|  | rs2712427 | -0.006 | 0.466 | 0.032 | 0.852 | 0.002 | 21.820 |
|  | rs428324 | 0.021 | 0.555 | 0.032 | 0.519 | 0.002 | 21.858 |
|  | rs7171268 | 0.110 | 0.961 | 0.082 | 0.183 | 0.002 | 22.307 |
|  | rs72984228 | -0.018 | 0.957 | 0.078 | 0.821 | 0.002 | 19.786 |
|  | rs9962377 | 0.024 | 0.090 | 0.055 | 0.664 | 0.002 | 20.538 |
| CCL19 | rs10242459 | 0.094 | 0.845 | 0.044 | 0.031 | 0.002 | 22.405 |
|  | rs111317412 | 0.017 | 0.966 | 0.088 | 0.847 | 0.001 | 20.346 |
|  | rs11174279 | -0.025 | 0.201 | 0.039 | 0.520 | 0.001 | 19.788 |
|  | rs113139367 | -0.112 | 0.030 | 0.093 | 0.229 | 0.002 | 23.870 |
|  | rs113539352 | -0.814 | 0.999 | 0.734 | 0.267 | 0.002 | 23.658 |
|  | rs115109204 | -0.065 | 0.004 | 0.240 | 0.786 | 0.002 | 20.193 |
|  | rs11574915 | 0.086 | 0.846 | 0.044 | 0.050 | 0.005 | 74.152 |
|  | rs11877886 | -0.031 | 0.082 | 0.057 | 0.586 | 0.001 | 19.854 |
|  | rs11961362 | -0.011 | 0.083 | 0.057 | 0.853 | 0.002 | 20.145 |
|  | rs12521515 | 0.018 | 0.832 | 0.042 | 0.669 | 0.002 | 20.060 |
|  | rs13010492 | 0.035 | 0.366 | 0.033 | 0.283 | 0.003 | 42.614 |
|  | rs13212719 | -0.006 | 0.853 | 0.045 | 0.894 | 0.001 | 20.008 |
|  | rs1328133 | -0.102 | 0.005 | 0.233 | 0.662 | 0.001 | 20.047 |
|  | rs138108512 | -0.148 | 0.006 | 0.214 | 0.488 | 0.002 | 22.760 |
|  | rs138440733 | 0.147 | 0.063 | 0.067 | 0.029 | 0.002 | 22.930 |
|  | rs138618048 | -0.057 | 0.009 | 0.168 | 0.733 | 0.001 | 20.169 |
|  | rs1415763 | 0.053 | 0.197 | 0.040 | 0.181 | 0.002 | 23.245 |
|  | rs147708384 | -0.221 | 0.006 | 0.206 | 0.283 | 0.002 | 22.238 |
|  | rs2070746 | -0.008 | 0.287 | 0.035 | 0.810 | 0.001 | 20.177 |
|  | rs34791102 | 0.131 | 0.028 | 0.097 | 0.175 | 0.001 | 21.822 |
|  | rs374784992 | 0.072 | 0.924 | 0.060 | 0.228 | 0.002 | 20.066 |
|  | rs3792790 | -0.026 | 0.455 | 0.032 | 0.415 | 0.001 | 21.080 |
|  | rs4356912 | -0.041 | 0.715 | 0.035 | 0.247 | 0.001 | 19.810 |
|  | rs454265 | 0.009 | 0.681 | 0.034 | 0.783 | 0.001 | 20.466 |
|  | rs4554017 | -0.003 | 0.437 | 0.032 | 0.921 | 0.002 | 23.862 |
|  | rs4766578 | -0.076 | 0.570 | 0.032 | 0.018 | 0.003 | 36.817 |
|  | rs4907572 | -0.020 | 0.282 | 0.035 | 0.561 | 0.002 | 23.985 |
|  | rs62292952 | -0.025 | 0.870 | 0.047 | 0.595 | 0.011 | 160.788 |
|  | rs6486995 | 0.003 | 0.244 | 0.037 | 0.933 | 0.002 | 26.653 |
|  | rs6535499 | 0.021 | 0.036 | 0.085 | 0.802 | 0.001 | 19.983 |
|  | rs6870560 | -0.036 | 0.839 | 0.043 | 0.398 | 0.002 | 26.073 |
|  | rs7185085 | -0.055 | 0.999 | 0.519 | 0.915 | 0.002 | 19.630 |
|  | rs73257451 | 0.023 | 0.978 | 0.109 | 0.831 | 0.002 | 23.242 |
|  | rs74764632 | 0.122 | 0.938 | 0.065 | 0.062 | 0.001 | 21.051 |
|  | rs7595241 | 0.044 | 0.651 | 0.033 | 0.182 | 0.002 | 22.377 |
|  | rs76295077 | -0.102 | 0.905 | 0.054 | 0.059 | 0.001 | 19.903 |
|  | rs79086127 | -0.065 | 0.005 | 0.225 | 0.771 | 0.002 | 21.692 |
|  | rs9469127 | -0.195 | 0.903 | 0.050 | 0.000 | 0.021 | 310.837 |
| CCL20 | rs10207134 | -0.092 | 0.230 | 0.038 | 0.015 | 0.004 | 60.539 |
|  | rs1027232 | 0.069 | 0.152 | 0.044 | 0.117 | 0.001 | 20.285 |
|  | rs10837780 | -0.027 | 0.944 | 0.069 | 0.699 | 0.001 | 20.981 |
|  | rs111674022 | 0.017 | 0.091 | 0.055 | 0.757 | 0.001 | 20.989 |
|  | rs11242693 | -0.007 | 0.434 | 0.032 | 0.833 | 0.002 | 22.261 |
|  | rs1129474 | -0.004 | 0.548 | 0.032 | 0.897 | 0.002 | 22.797 |
|  | rs11582772 | -0.288 | 0.996 | 0.243 | 0.236 | 0.002 | 22.248 |
|  | rs117753190 | 0.223 | 0.989 | 0.156 | 0.152 | 0.002 | 26.552 |
|  | rs117900576 | 0.060 | 0.990 | 0.159 | 0.705 | 0.002 | 27.650 |
|  | rs118164045 | 0.079 | 0.959 | 0.080 | 0.323 | 0.001 | 20.138 |
|  | rs13049624 | -0.057 | 0.929 | 0.062 | 0.353 | 0.002 | 22.697 |
|  | rs13185268 | 0.110 | 0.918 | 0.058 | 0.056 | 0.001 | 20.835 |
|  | rs151092565 | -0.384 | 0.025 | 0.114 | 0.001 | 0.001 | 21.247 |
|  | rs2003431 | -0.029 | 0.048 | 0.077 | 0.707 | 0.002 | 21.683 |
|  | rs2796032 | 0.076 | 0.320 | 0.034 | 0.025 | 0.002 | 25.075 |
|  | rs34729167 | -0.008 | 0.198 | 0.040 | 0.841 | 0.001 | 20.022 |
|  | rs348389 | -0.064 | 0.353 | 0.033 | 0.055 | 0.001 | 19.671 |
|  | rs35757792 | 0.002 | 0.498 | 0.032 | 0.944 | 0.001 | 19.833 |
|  | rs35833676 | 0.044 | 0.124 | 0.048 | 0.355 | 0.002 | 23.455 |
|  | rs3798304 | -0.034 | 0.760 | 0.037 | 0.362 | 0.001 | 20.443 |
|  | rs41302923 | -0.031 | 0.973 | 0.098 | 0.757 | 0.001 | 21.933 |
|  | rs4981022 | 0.051 | 0.683 | 0.034 | 0.133 | 0.002 | 24.608 |
|  | rs55891514 | 0.085 | 0.957 | 0.078 | 0.276 | 0.001 | 20.100 |
|  | rs5769672 | 0.108 | 0.980 | 0.114 | 0.342 | 0.001 | 21.222 |
|  | rs61945278 | 0.009 | 0.023 | 0.107 | 0.933 | 0.001 | 19.663 |
|  | rs62456601 | 0.090 | 0.601 | 0.032 | 0.005 | 0.002 | 19.887 |
|  | rs6420556 | 0.010 | 0.178 | 0.041 | 0.812 | 0.002 | 21.696 |
|  | rs6498925 | -0.045 | 0.644 | 0.033 | 0.172 | 0.001 | 20.645 |
|  | rs73072893 | -0.071 | 0.966 | 0.087 | 0.414 | 0.002 | 23.756 |
|  | rs742493 | -0.100 | 0.903 | 0.054 | 0.064 | 0.004 | 62.188 |
|  | rs748503 | 0.003 | 0.288 | 0.036 | 0.938 | 0.001 | 19.860 |
|  | rs7749768 | -0.065 | 0.807 | 0.040 | 0.108 | 0.001 | 20.313 |
|  | rs9274623 | 0.081 | 0.141 | 0.045 | 0.074 | 0.002 | 26.366 |
|  | rs9357723 | -0.005 | 0.461 | 0.032 | 0.879 | 0.001 | 20.247 |
| CD5 | rs11622090 | -0.005 | 0.258 | 0.036 | 0.894 | 0.002 | 21.099 |
|  | rs117276991 | -0.079 | 0.096 | 0.054 | 0.142 | 0.002 | 24.260 |
|  | rs117516652 | 0.028 | 0.984 | 0.128 | 0.829 | 0.002 | 24.871 |
|  | rs12416520 | -0.019 | 0.784 | 0.038 | 0.616 | 0.002 | 22.704 |
|  | rs1259355 | -0.011 | 0.374 | 0.033 | 0.735 | 0.002 | 22.860 |
|  | rs13010492 | 0.035 | 0.366 | 0.033 | 0.283 | 0.002 | 23.305 |
|  | rs141298297 | -0.046 | 0.845 | 0.044 | 0.293 | 0.002 | 19.860 |
|  | rs16541 | 0.003 | 0.859 | 0.046 | 0.940 | 0.001 | 20.488 |
|  | rs17345759 | -0.144 | 0.934 | 0.065 | 0.026 | 0.001 | 21.421 |
|  | rs17769062 | 0.026 | 0.972 | 0.096 | 0.781 | 0.002 | 24.048 |
|  | rs192582121 | -0.232 | 0.997 | 0.300 | 0.440 | 0.002 | 26.750 |
|  | rs193245191 | 0.047 | 0.001 | 0.487 | 0.923 | 0.002 | 19.950 |
|  | rs2237450 | 0.017 | 0.648 | 0.033 | 0.610 | 0.002 | 25.239 |
|  | rs3104373 | -0.025 | 0.138 | 0.046 | 0.588 | 0.002 | 34.770 |
|  | rs3184504 | 0.071 | 0.408 | 0.032 | 0.027 | 0.010 | 124.267 |
|  | rs367364 | -0.076 | 0.125 | 0.047 | 0.109 | 0.002 | 26.777 |
|  | rs4073745 | -0.005 | 0.702 | 0.035 | 0.879 | 0.002 | 27.179 |
|  | rs4893 | 0.261 | 0.962 | 0.088 | 0.003 | 0.001 | 22.084 |
|  | rs4939490 | 0.011 | 0.715 | 0.035 | 0.761 | 0.005 | 81.440 |
|  | rs60562286 | -0.009 | 0.017 | 0.123 | 0.941 | 0.002 | 22.803 |
|  | rs61747728 | 0.217 | 0.063 | 0.061 | 0.000 | 0.002 | 34.591 |
|  | rs61768480 | -0.049 | 0.319 | 0.035 | 0.160 | 0.002 | 22.350 |
|  | rs6488973 | -0.021 | 0.335 | 0.034 | 0.527 | 0.001 | 20.616 |
|  | rs674379 | 0.061 | 0.674 | 0.034 | 0.070 | 0.007 | 89.634 |
|  | rs7012814 | 0.000 | 0.509 | 0.032 | 0.995 | 0.001 | 20.059 |
|  | rs7242462 | -0.072 | 0.860 | 0.045 | 0.115 | 0.003 | 41.105 |
|  | rs7295476 | 0.101 | 0.033 | 0.089 | 0.253 | 0.001 | 19.825 |
|  | rs7298384 | -0.056 | 0.970 | 0.094 | 0.548 | 0.001 | 19.633 |
|  | rs74796205 | -0.006 | 0.977 | 0.108 | 0.958 | 0.001 | 20.667 |
|  | rs78808574 | 0.143 | 0.950 | 0.073 | 0.050 | 0.001 | 19.825 |
| CD6 | rs10792322 | -0.031 | 0.287 | 0.035 | 0.370 | 0.002 | 27.600 |
|  | rs10843957 | 0.016 | 0.584 | 0.032 | 0.623 | 0.001 | 20.440 |
|  | rs11230555 | -0.137 | 0.179 | 0.042 | 0.001 | 0.124 | 2026.060 |
|  | rs116557290 | 0.074 | 0.028 | 0.097 | 0.448 | 0.001 | 19.602 |
|  | rs12074680 | -0.049 | 0.023 | 0.106 | 0.642 | 0.002 | 22.390 |
|  | rs143374617 | 0.004 | 0.037 | 0.084 | 0.961 | 0.002 | 28.809 |
|  | rs144764321 | 0.099 | 0.019 | 0.115 | 0.391 | 0.001 | 20.662 |
|  | rs1483572 | -0.015 | 0.327 | 0.034 | 0.649 | 0.002 | 23.566 |
|  | rs150354514 | 0.129 | 0.967 | 0.089 | 0.147 | 0.002 | 19.965 |
|  | rs200729584 | 0.268 | 0.995 | 0.244 | 0.272 | 0.003 | 26.583 |
|  | rs2237995 | -0.140 | 0.051 | 0.072 | 0.053 | 0.019 | 277.689 |
|  | rs28509311 | -0.083 | 0.990 | 0.155 | 0.592 | 0.002 | 20.542 |
|  | rs35086654 | 0.181 | 0.004 | 0.274 | 0.508 | 0.002 | 21.835 |
|  | rs35634144 | -0.095 | 0.991 | 0.168 | 0.573 | 0.002 | 21.709 |
|  | rs4902621 | -0.065 | 0.195 | 0.040 | 0.103 | 0.001 | 19.782 |
|  | rs56294840 | 0.020 | 0.871 | 0.047 | 0.674 | 0.001 | 20.591 |
|  | rs597808 | 0.080 | 0.414 | 0.032 | 0.013 | 0.007 | 74.926 |
|  | rs60988380 | -0.085 | 0.110 | 0.051 | 0.094 | 0.002 | 24.481 |
|  | rs72969761 | -0.040 | 0.946 | 0.070 | 0.568 | 0.002 | 22.813 |
|  | rs74886615 | -0.018 | 0.655 | 0.033 | 0.591 | 0.002 | 28.440 |
|  | rs78007268 | 0.056 | 0.019 | 0.115 | 0.629 | 0.001 | 19.892 |
|  | rs8051133 | -0.047 | 0.046 | 0.076 | 0.538 | 0.001 | 20.901 |
|  | rs841332 | 0.052 | 0.805 | 0.040 | 0.191 | 0.001 | 21.916 |
|  | rs9691968 | 0.024 | 0.215 | 0.038 | 0.537 | 0.002 | 23.238 |
| FGF-21 | rs10495032 | 0.071 | 0.072 | 0.061 | 0.246 | 0.002 | 23.970 |
|  | rs12290350 | -0.013 | 0.178 | 0.041 | 0.762 | 0.002 | 22.576 |
|  | rs12532101 | -0.043 | 0.245 | 0.037 | 0.236 | 0.001 | 19.559 |
|  | rs1260326 | 0.146 | 0.350 | 0.032 | 0.000 | 0.008 | 123.585 |
|  | rs13062487 | -0.019 | 0.274 | 0.036 | 0.586 | 0.001 | 20.534 |
|  | rs13229619 | -0.092 | 0.128 | 0.047 | 0.053 | 0.006 | 83.566 |
|  | rs142603673 | -0.046 | 0.008 | 0.173 | 0.791 | 0.002 | 26.717 |
|  | rs145127946 | -0.098 | 0.008 | 0.188 | 0.600 | 0.002 | 23.546 |
|  | rs145319387 | -0.049 | 0.686 | 0.034 | 0.152 | 0.002 | 21.774 |
|  | rs149561599 | -0.088 | 0.994 | 0.203 | 0.665 | 0.001 | 19.844 |
|  | rs17284047 | -0.045 | 0.993 | 0.187 | 0.811 | 0.001 | 20.125 |
|  | rs188758663 | 0.232 | 0.005 | 0.226 | 0.305 | 0.002 | 25.749 |
|  | rs2429473 | 0.001 | 0.808 | 0.040 | 0.971 | 0.002 | 30.508 |
|  | rs35852631 | -0.018 | 0.208 | 0.039 | 0.653 | 0.001 | 20.281 |
|  | rs4700382 | -0.002 | 0.271 | 0.036 | 0.957 | 0.001 | 21.456 |
|  | rs60277384 | 0.067 | 0.850 | 0.044 | 0.131 | 0.001 | 21.497 |
|  | rs7012637 | 0.000 | 0.509 | 0.032 | 0.999 | 0.002 | 25.415 |
|  | rs7083293 | -0.027 | 0.597 | 0.032 | 0.397 | 0.002 | 19.713 |
|  | rs72965996 | -0.124 | 0.021 | 0.113 | 0.273 | 0.002 | 24.884 |
|  | rs7442192 | -0.046 | 0.749 | 0.036 | 0.203 | 0.001 | 20.117 |
|  | rs7610704 | 0.019 | 0.526 | 0.032 | 0.557 | 0.002 | 22.401 |
|  | rs79789860 | -0.013 | 0.028 | 0.096 | 0.896 | 0.001 | 19.987 |
|  | rs8191009 | 0.049 | 0.229 | 0.038 | 0.192 | 0.001 | 20.158 |
|  | rs838131 | 0.076 | 0.430 | 0.032 | 0.017 | 0.011 | 156.613 |
|  | rs9974115 | -0.044 | 0.838 | 0.043 | 0.304 | 0.001 | 20.114 |
| IL-10 α | rs10091841 | -0.003 | 0.407 | 0.033 | 0.934 | 0.002 | 22.471 |
|  | rs12026492 | 0.075 | 0.213 | 0.039 | 0.052 | 0.002 | 20.652 |
|  | rs1406714 | 0.033 | 0.401 | 0.032 | 0.308 | 0.002 | 22.613 |
|  | rs143313574 | -0.060 | 0.026 | 0.101 | 0.550 | 0.002 | 21.149 |
|  | rs17832137 | 0.079 | 0.053 | 0.071 | 0.266 | 0.002 | 23.871 |
|  | rs180893431 | 0.192 | 0.997 | 0.321 | 0.549 | 0.002 | 21.993 |
|  | rs28408877 | -0.010 | 0.787 | 0.039 | 0.793 | 0.002 | 20.335 |
|  | rs286605 | 0.004 | 0.577 | 0.032 | 0.895 | 0.002 | 22.613 |
|  | rs3135932 | -0.057 | 0.798 | 0.040 | 0.152 | 0.002 | 25.234 |
|  | rs3777726 | 0.041 | 0.219 | 0.039 | 0.281 | 0.002 | 20.657 |
|  | rs41272625 | 0.036 | 0.102 | 0.052 | 0.493 | 0.002 | 20.982 |
|  | rs492616 | -0.073 | 0.829 | 0.042 | 0.083 | 0.002 | 19.515 |
|  | rs62262707 | -0.026 | 0.015 | 0.130 | 0.841 | 0.002 | 19.922 |
|  | rs6670799 | -0.100 | 0.096 | 0.054 | 0.061 | 0.002 | 24.482 |
|  | rs74719858 | 0.098 | 0.962 | 0.083 | 0.239 | 0.002 | 22.625 |
|  | rs79202194 | -0.054 | 0.093 | 0.055 | 0.326 | 0.002 | 19.648 |
|  | rs80137559 | 0.004 | 0.975 | 0.101 | 0.968 | 0.002 | 21.092 |
|  | rs9472032 | 0.050 | 0.112 | 0.050 | 0.318 | 0.002 | 20.457 |
|  | rs9548767 | -0.043 | 0.200 | 0.040 | 0.279 | 0.002 | 19.584 |
|  | rs958402 | 0.001 | 0.435 | 0.032 | 0.967 | 0.002 | 20.045 |
| IL12 β | rs10100578 | 0.016 | 0.477 | 0.032 | 0.616 | 0.001 | 19.979 |
|  | rs11039216 | 0.042 | 0.439 | 0.032 | 0.183 | 0.002 | 25.691 |
|  | rs11130215 | 0.086 | 0.729 | 0.036 | 0.016 | 0.003 | 44.645 |
|  | rs117888068 | 0.061 | 0.980 | 0.115 | 0.595 | 0.002 | 21.524 |
|  | rs12588969 | 0.006 | 0.792 | 0.039 | 0.886 | 0.007 | 104.283 |
|  | rs13095341 | 0.011 | 0.025 | 0.104 | 0.918 | 0.001 | 19.695 |
|  | rs1398590 | -0.022 | 0.021 | 0.111 | 0.844 | 0.002 | 29.448 |
|  | rs144411658 | -0.062 | 0.458 | 0.032 | 0.050 | 0.002 | 20.589 |
|  | rs144850787 | 0.072 | 0.992 | 0.176 | 0.682 | 0.002 | 31.858 |
|  | rs148549227 | 0.001 | 0.092 | 0.055 | 0.985 | 0.002 | 22.768 |
|  | rs149993822 | -0.002 | 0.153 | 0.045 | 0.964 | 0.002 | 25.723 |
|  | rs150590615 | 1.109 | 0.000 | 0.796 | 0.163 | 0.002 | 19.795 |
|  | rs17056751 | 0.081 | 0.941 | 0.067 | 0.229 | 0.002 | 27.164 |
|  | rs17500902 | 0.053 | 0.894 | 0.051 | 0.299 | 0.001 | 19.648 |
|  | rs17722688 | -0.052 | 0.722 | 0.035 | 0.138 | 0.003 | 42.792 |
|  | rs1950897 | -0.073 | 0.690 | 0.034 | 0.032 | 0.003 | 38.862 |
|  | rs201503938 | 0.044 | 0.157 | 0.044 | 0.308 | 0.002 | 23.600 |
|  | rs3130510 | 0.100 | 0.151 | 0.044 | 0.023 | 0.005 | 68.053 |
|  | rs3184504 | 0.071 | 0.408 | 0.032 | 0.027 | 0.009 | 103.369 |
|  | rs34169305 | 0.024 | 0.369 | 0.033 | 0.462 | 0.001 | 19.821 |
|  | rs3822030 | -0.007 | 0.631 | 0.033 | 0.820 | 0.003 | 20.462 |
|  | rs4244437 | 0.175 | 0.749 | 0.037 | 0.000 | 0.101 | 1647.733 |
|  | rs4783410 | -0.054 | 0.101 | 0.052 | 0.307 | 0.001 | 19.882 |
|  | rs5014495 | 0.036 | 0.064 | 0.067 | 0.591 | 0.002 | 21.929 |
|  | rs516978 | 0.032 | 0.372 | 0.033 | 0.329 | 0.002 | 34.921 |
|  | rs539598 | 0.002 | 0.215 | 0.038 | 0.961 | 0.002 | 27.133 |
|  | rs6750065 | -0.009 | 0.773 | 0.038 | 0.812 | 0.002 | 21.961 |
|  | rs6968201 | -0.019 | 0.480 | 0.032 | 0.556 | 0.002 | 27.317 |
|  | rs705705 | -0.037 | 0.319 | 0.034 | 0.282 | 0.003 | 34.831 |
|  | rs72993671 | 0.303 | 0.008 | 0.177 | 0.087 | 0.002 | 21.990 |
|  | rs73416208 | -0.030 | 0.088 | 0.056 | 0.593 | 0.002 | 36.044 |
|  | rs76322730 | -0.074 | 0.015 | 0.131 | 0.574 | 0.001 | 20.341 |
|  | rs76428106 | 0.028 | 0.987 | 0.147 | 0.849 | 0.003 | 40.525 |
|  | rs780679 | -0.079 | 0.247 | 0.037 | 0.030 | 0.002 | 24.997 |
|  | rs8026767 | 0.064 | 0.223 | 0.038 | 0.092 | 0.002 | 31.679 |
|  | rs9815073 | 0.018 | 0.329 | 0.034 | 0.591 | 0.033 | 484.271 |
|  | rs9965402 | -0.025 | 0.905 | 0.054 | 0.646 | 0.001 | 20.856 |
| STAMPB | rs1034059 | -0.012 | 0.702 | 0.035 | 0.739 | 0.002 | 22.971 |
|  | rs111581172 | 0.128 | 0.013 | 0.138 | 0.355 | 0.002 | 23.929 |
|  | rs111872246 | 0.052 | 0.023 | 0.107 | 0.628 | 0.002 | 26.670 |
|  | rs116132562 | -0.047 | 0.018 | 0.119 | 0.694 | 0.001 | 20.612 |
|  | rs116198125 | -0.135 | 0.970 | 0.093 | 0.150 | 0.001 | 20.847 |
|  | rs12152184 | 0.014 | 0.886 | 0.050 | 0.782 | 0.002 | 21.343 |
|  | rs12531843 | -0.073 | 0.908 | 0.056 | 0.191 | 0.002 | 26.499 |
|  | rs138897138 | 0.059 | 0.915 | 0.057 | 0.301 | 0.002 | 24.768 |
|  | rs140656901 | 0.030 | 0.095 | 0.054 | 0.576 | 0.002 | 22.420 |
|  | rs16847403 | 0.032 | 0.755 | 0.037 | 0.391 | 0.001 | 19.871 |
|  | rs2280441 | -0.037 | 0.023 | 0.105 | 0.727 | 0.002 | 25.845 |
|  | rs2584528 | 0.029 | 0.688 | 0.034 | 0.392 | 0.001 | 20.726 |
|  | rs547553 | -0.003 | 0.948 | 0.072 | 0.970 | 0.001 | 19.654 |
|  | rs61962729 | -0.001 | 0.893 | 0.052 | 0.992 | 0.001 | 20.309 |
|  | rs6492915 | -0.065 | 0.830 | 0.042 | 0.125 | 0.002 | 20.472 |
|  | rs73327656 | -0.019 | 0.008 | 0.181 | 0.916 | 0.002 | 21.064 |
|  | rs73788059 | -0.017 | 0.105 | 0.051 | 0.737 | 0.001 | 20.336 |
|  | rs7418913 | 0.024 | 0.220 | 0.038 | 0.534 | 0.001 | 20.409 |
|  | rs75610895 | -0.045 | 0.009 | 0.173 | 0.795 | 0.002 | 23.448 |
|  | rs851168 | 0.015 | 0.328 | 0.034 | 0.654 | 0.002 | 20.512 |
| Abbreviations: SNPs: single nucleotide polymorphisms; EA: effect allele; OA: other allele; EAF: effect allele frequency; SE: standard error; IBD: inflammatory bowel disease; UC: ulcerative colitis; CD: Crohn's disease. | | | | | | | |

| **Supplementary Table S14. MR analysis results of associations between inflammatory proteins and IBD after the final screening.** | | | | | | | | | | | | | |
| --- | --- | --- | --- | --- | --- | --- | --- | --- | --- | --- | --- | --- | --- |
| **Inflammatory proteins** | **Method** | **Nsnp** | **OR** | **OR_lci95** | **OR_uci95** | **P** | **Heterogeneity** | | | | **Pleiotropy** | | |
|  |  |  |  |  |  |  | **MR Egger** | | **IVW** | | **MR-Egger intercep** | | **MR-PRESO** |
|  |  |  |  |  |  |  | **Q** | **P** | **Q** | **P** | **intercept** | **P** | **P** |
| **IBD** |  |  |  |  |  |  |  |  |  |  |  |  |  |
| Axin-1 | MR Egger | 13 | 1.268 | 0.853 | 1.885 | 0.264 | 13.878 | 0.240 | 13.993 | 0.301 | -0.006 | 0.768 | 0.371 |
|  | Weighted median | 13 | 1.176 | 0.979 | 1.412 | 0.083 |  |  |  |  |  |  |  |
|  | IVW | 13 | 1.199 | 1.036 | 1.387 | 0.015 |  |  |  |  |  |  |  |
|  | Simple mode | 13 | 1.233 | 0.911 | 1.668 | 0.200 |  |  |  |  |  |  |  |
|  | Weighted mode | 13 | 1.211 | 0.911 | 1.612 | 0.212 |  |  |  |  |  |  |  |
|  | BWMR | 13 | 1.206 | 1.045 | 1.391 | 0.010 |  |  |  |  |  |  |  |
| CCL19 | MR Egger | 36 | 1.105 | 0.986 | 1.239 | 0.096 | 46.677 | 0.072 | 46.990 | 0.085 | -0.004 | 0.636 | 0.057 |
|  | Weighted median | 36 | 1.167 | 1.063 | 1.282 | 0.001 |  |  |  |  |  |  |  |
|  | IVW | 36 | 1.082 | 1.007 | 1.162 | 0.032 |  |  |  |  |  |  |  |
|  | Simple mode | 36 | 1.085 | 0.882 | 1.334 | 0.447 |  |  |  |  |  |  |  |
|  | Weighted mode | 36 | 1.152 | 1.042 | 1.274 | 0.009 |  |  |  |  |  |  |  |
|  | BWMR | 36 | 1.101 | 1.018 | 1.191 | 0.016 |  |  |  |  |  |  |  |
| CCL4 | MR Egger | 31 | 1.171 | 1.077 | 1.274 | 0.001 | 43.861 | 0.038 | 48.900 | 0.016 | -0.015 | 0.078 | 0.059 |
|  | Weighted median | 31 | 1.140 | 1.045 | 1.244 | 0.003 |  |  |  |  |  |  |  |
|  | IVW | 31 | 1.109 | 1.042 | 1.180 | 0.001 |  |  |  |  |  |  |  |
|  | Simple mode | 31 | 0.988 | 0.800 | 1.221 | 0.913 |  |  |  |  |  |  |  |
|  | Weighted mode | 31 | 1.125 | 1.052 | 1.202 | 0.002 |  |  |  |  |  |  |  |
|  | BWMR | 31 | 1.119 | 1.047 | 1.196 | 0.001 |  |  |  |  |  |  |  |
| CD6 | MR Egger | 24 | 1.123 | 1.021 | 1.235 | 0.026 | 40.006 | 0.011 | 40.678 | 0.013 | -0.006 | 0.549 | 0.051 |
|  | Weighted median | 24 | 1.114 | 1.049 | 1.183 | 0.000 |  |  |  |  |  |  |  |
|  | IVW | 24 | 1.100 | 1.027 | 1.179 | 0.006 |  |  |  |  |  |  |  |
|  | Simple mode | 24 | 0.967 | 0.765 | 1.222 | 0.779 |  |  |  |  |  |  |  |
|  | Weighted mode | 24 | 1.105 | 1.036 | 1.180 | 0.006 |  |  |  |  |  |  |  |
|  | BWMR | 24 | 1.096 | 1.020 | 1.179 | 0.013 |  |  |  |  |  |  |  |
| FGF-5 | MR Egger | 32 | 1.048 | 0.970 | 1.131 | 0.245 | 43.255 | 0.056 | 43.823 | 0.063 | 0.005 | 0.535 | 0.110 |
|  | Weighted median | 32 | 1.034 | 0.975 | 1.095 | 0.263 |  |  |  |  |  |  |  |
|  | IVW | 32 | 1.066 | 1.010 | 1.125 | 0.019 |  |  |  |  |  |  |  |
|  | Simple mode | 32 | 0.989 | 0.827 | 1.184 | 0.906 |  |  |  |  |  |  |  |
|  | Weighted mode | 32 | 1.038 | 0.981 | 1.100 | 0.207 |  |  |  |  |  |  |  |
|  | BWMR | 32 | 1.074 | 1.013 | 1.139 | 0.016 |  |  |  |  |  |  |  |
| TNFSF12 | MR Egger | 43 | 1.089 | 0.944 | 1.256 | 0.251 | 35.007 | 0.733 | 35.007 | 0.769 | 0.000 | 0.988 | 0.771 |
|  | Weighted median | 43 | 1.110 | 1.002 | 1.229 | 0.046 |  |  |  |  |  |  |  |
|  | IVW | 43 | 1.088 | 1.016 | 1.165 | 0.016 |  |  |  |  |  |  |  |
|  | Simple mode | 43 | 1.043 | 0.885 | 1.230 | 0.619 |  |  |  |  |  |  |  |
|  | Weighted mode | 43 | 1.121 | 0.986 | 1.274 | 0.089 |  |  |  |  |  |  |  |
|  | BWMR | 43 | 1.084 | 1.010 | 1.163 | 0.025 |  |  |  |  |  |  |  |
| **UC** |  |  |  |  |  |  |  |  |  |  |  |  |  |
| CCL4 | MR Egger | 31 | 1.196 | 1.062 | 1.346 | 0.006 | 57.821 | 0.001 | 62.187 | 0.000 | -0.017 | 0.150 | 0.370 |
|  | Weighted median | 31 | 1.295 | 1.162 | 1.444 | 0.000 |  |  |  |  |  |  |  |
|  | IVW | 31 | 1.123 | 1.030 | 1.224 | 0.008 |  |  |  |  |  |  |  |
|  | Simple mode | 31 | 0.858 | 0.623 | 1.183 | 0.358 |  |  |  |  |  |  |  |
|  | Weighted mode | 31 | 1.158 | 1.062 | 1.263 | 0.002 |  |  |  |  |  |  |  |
|  | BWMR | 31 | 1.146 | 1.044 | 1.257 | 0.004 |  |  |  |  |  |  |  |
| FGF-5 | MR Egger | 32 | 1.105 | 1.013 | 1.204 | 0.031 | 35.980 | 0.209 | 37.269 | 0.203 | -0.009 | 0.308 | 0.299 |
|  | Weighted median | 32 | 1.067 | 0.993 | 1.147 | 0.076 |  |  |  |  |  |  |  |
|  | IVW | 32 | 1.070 | 1.006 | 1.137 | 0.031 |  |  |  |  |  |  |  |
|  | Simple mode | 32 | 0.842 | 0.690 | 1.027 | 0.100 |  |  |  |  |  |  |  |
|  | Weighted mode | 32 | 1.074 | 1.002 | 1.151 | 0.051 |  |  |  |  |  |  |  |
|  | BWMR | 32 | 1.071 | 1.007 | 1.139 | 0.029 |  |  |  |  |  |  |  |
| **CD** |  |  |  |  |  |  |  |  |  |  |  |  |  |
| Axin-1 | MR Egger | 13 | 2.215 | 1.060 | 4.626 | 0.058 | 6.742 | 0.820 | 8.456 | 0.749 | -0.045 | 0.217 | 0.768 |
|  | Weighted median | 13 | 1.223 | 0.839 | 1.782 | 0.295 |  |  |  |  |  |  |  |
|  | IVW | 13 | 1.406 | 1.060 | 1.864 | 0.018 |  |  |  |  |  |  |  |
|  | Simple mode | 13 | 1.102 | 0.615 | 1.975 | 0.750 |  |  |  |  |  |  |  |
|  | Weighted mode | 13 | 1.135 | 0.634 | 2.031 | 0.677 |  |  |  |  |  |  |  |
|  | BWMR | 13 | 1.405 | 1.044 | 1.892 | 0.025 |  |  |  |  |  |  |  |
| CCL19 | MR Egger | 36 | 1.278 | 1.019 | 1.602 | 0.041 | 43.425 | 0.129 | 44.138 | 0.138 | -0.011 | 0.460 | 0.086 |
|  | Weighted median | 36 | 1.451 | 1.186 | 1.774 | 0.000 |  |  |  |  |  |  |  |
|  | IVW | 36 | 1.196 | 1.036 | 1.380 | 0.015 |  |  |  |  |  |  |  |
|  | Simple mode | 36 | 1.045 | 0.637 | 1.714 | 0.864 |  |  |  |  |  |  |  |
|  | Weighted mode | 36 | 1.459 | 1.185 | 1.796 | 0.001 |  |  |  |  |  |  |  |
|  | BWMR | 36 | 1.210 | 1.038 | 1.410 | 0.015 |  |  |  |  |  |  |  |
| CD5 | MR Egger | 29 | 1.966 | 1.208 | 3.201 | 0.011 | 36.254 | 0.110 | 39.785 | 0.069 | -0.040 | 0.117 | 0.076 |
|  | Weighted median | 29 | 1.384 | 1.074 | 1.785 | 0.012 |  |  |  |  |  |  |  |
|  | IVW | 29 | 1.359 | 1.112 | 1.661 | 0.003 |  |  |  |  |  |  |  |
|  | Simple mode | 29 | 1.117 | 0.702 | 1.778 | 0.645 |  |  |  |  |  |  |  |
|  | Weighted mode | 29 | 1.533 | 1.081 | 2.173 | 0.023 |  |  |  |  |  |  |  |
|  | BWMR | 29 | 1.360 | 1.117 | 1.656 | 0.002 |  |  |  |  |  |  |  |
| CD6 | MR Egger | 24 | 1.272 | 1.086 | 1.489 | 0.007 | 24.807 | 0.306 | 26.297 | 0.287 | -0.017 | 0.263 | 0.312 |
|  | Weighted median | 24 | 1.255 | 1.106 | 1.424 | 0.000 |  |  |  |  |  |  |  |
|  | IVW | 24 | 1.194 | 1.063 | 1.342 | 0.003 |  |  |  |  |  |  |  |
|  | Simple mode | 24 | 1.381 | 0.896 | 2.128 | 0.157 |  |  |  |  |  |  |  |
|  | Weighted mode | 24 | 1.276 | 1.117 | 1.457 | 0.002 |  |  |  |  |  |  |  |
|  | BWMR | 24 | 1.196 | 1.054 | 1.358 | 0.005 |  |  |  |  |  |  |  |
| IL-10 α | MR Egger | 19 | 1.281 | 0.899 | 1.824 | 0.188 | 11.051 | 0.854 | 11.202 | 0.886 | 0.007 | 0.702 | 0.909 |
|  | Weighted median | 19 | 1.262 | 0.944 | 1.688 | 0.116 |  |  |  |  |  |  |  |
|  | IVW | 19 | 1.358 | 1.116 | 1.652 | 0.002 |  |  |  |  |  |  |  |
|  | Simple mode | 19 | 1.618 | 1.009 | 2.595 | 0.061 |  |  |  |  |  |  |  |
|  | Weighted mode | 19 | 1.217 | 0.892 | 1.660 | 0.232 |  |  |  |  |  |  |  |
|  | BWMR | 19 | 1.414 | 1.141 | 1.752 | 0.002 |  |  |  |  |  |  |  |
| Abbreviations: IVW: inverse variance weighted; BWMR: Bayesian Weighted Mendelian Randomization; β:beta; OR: odds ratio; IBD: inflammatory bowel disease; UC: ulcerative colitis;  CD: Crohn's disease. | | | | | | | | | | | | | |

| **Supplementary Table S15. MR analysis results of associations between inflammatory proteins and IBD after further validation analysis** | | | | | | | | | | | | |
| --- | --- | --- | --- | --- | --- | --- | --- | --- | --- | --- | --- | --- |
| **Plasma lipidome** | **Method** | **OR** | **OR_lci95** | **OR_uci95** | **P** | **Heterogeneity** | | | | **Pleiotropy** | | |
|  |  |  |  |  |  | **MR Egger** | | **IVW** | | **MR-Egger intercep** | | **MR-PRESO** |
|  |  |  |  |  |  | **Q** | **P** | **Q** | **P** | **intercept** | **P** | **P** |
| **IBD** |  |  |  |  |  |  |  |  |  |  |  |  |
| Axin-1 | MR Egger | 1.610 | 0.311 | 8.349 | 0.671 | 1.202 | 0.404 | 1.821 | 0.766 | -0.029 | 0.767 | 0.129 |
|  | Weighted median | 1.078 | 0.752 | 1.545 | 0.684 |  |  |  |  |  |  |  |
|  | IVW | 1.192 | 0.760 | 1.869 | 0.444 |  |  |  |  |  |  |  |
|  | Simple mode | 1.042 | 0.655 | 1.656 | 0.879 |  |  |  |  |  |  |  |
|  | Weighted mode | 1.042 | 0.725 | 1.497 | 0.846 |  |  |  |  |  |  |  |
|  | BWMR | 1.197 | 0.875 | 1.636 | 0.261 |  |  |  |  |  |  |  |
| CCL19 | MR Egger | 1.166 | 0.891 | 1.526 | 0.326 | 1.152 | 0.337 | 2.143 | 0.219 | -0.022 | 0.474 | 0.374 |
|  | Weighted median | 1.074 | 0.907 | 1.272 | 0.409 |  |  |  |  |  |  |  |
|  | IVW | 1.138 | 1.023 | 1.266 | 0.017 |  |  |  |  |  |  |  |
|  | Simple mode | 1.011 | 0.704 | 1.451 | 0.955 |  |  |  |  |  |  |  |
|  | Weighted mode | 1.138 | 1.019 | 1.270 | 0.070 |  |  |  |  |  |  |  |
|  | BWMR | 1.103 | 1.052 | 1.278 | 0.019 |  |  |  |  |  |  |  |
| CCL4 | MR Egger | 1.161 | 0.989 | 1.362 | 0.117 | 2.784 | 0.572 | 2.245 | 0.603 | -0.015 | 0.558 | 0.527 |
|  | Weighted median | 1.066 | 0.966 | 1.177 | 0.202 |  |  |  |  |  |  |  |
|  | IVW | 1.118 | 1.010 | 1.236 | 0.031 |  |  |  |  |  |  |  |
|  | Simple mode | 1.051 | 0.860 | 1.286 | 0.640 |  |  |  |  |  |  |  |
|  | Weighted mode | 1.129 | 1.052 | 1.212 | 0.012 |  |  |  |  |  |  |  |
|  | BWMR | 1.113 | 1.098 | 1.240 | 0.044 |  |  |  |  |  |  |  |
| CD6 | MR Egger | 1.110 | 0.978 | 1.261 | 0.168 | 1.627 | 0.271 | 1.013 | 0.428 | 0.006 | 0.712 | 0.698 |
|  | Weighted median | 1.117 | 1.049 | 1.189 | 0.001 |  |  |  |  |  |  |  |
|  | IVW | 1.130 | 1.037 | 1.230 | 0.005 |  |  |  |  |  |  |  |
|  | Simple mode | 1.342 | 1.095 | 1.644 | 0.030 |  |  |  |  |  |  |  |
|  | Weighted mode | 1.120 | 1.054 | 1.191 | 0.011 |  |  |  |  |  |  |  |
|  | BWMR | 1.124 | 1.033 | 1.222 | 0.006 |  |  |  |  |  |  |  |
| FGF-5 | MR Egger | 0.888 | 0.744 | 1.058 | 0.411 | 0.132 | 0.716 | 3.855 | 0.146 | 0.078 | 0.304 | 0.843 |
|  | Weighted median | 1.044 | 0.982 | 1.110 | 0.172 |  |  |  |  |  |  |  |
|  | IVW | 1.045 | 0.962 | 1.134 | 0.298 |  |  |  |  |  |  |  |
|  | Simple mode | 1.403 | 0.952 | 2.065 | 0.229 |  |  |  |  |  |  |  |
|  | Weighted mode | 1.032 | 0.970 | 1.098 | 0.420 |  |  |  |  |  |  |  |
|  | BWMR | 1.067 | 0.925 | 1.230 | 0.376 |  |  |  |  |  |  |  |
| TNFSF12 | MR Egger | 1.129 | 0.891 | 1.432 | 0.339 | 10.516 | 0.396 | 10.520 | 0.484 | -0.001 | 0.951 | 0.062 |
|  | Weighted median | 1.141 | 0.997 | 1.305 | 0.054 |  |  |  |  |  |  |  |
|  | IVW | 1.122 | 1.013 | 1.242 | 0.027 |  |  |  |  |  |  |  |
|  | Simple mode | 1.028 | 0.847 | 1.248 | 0.786 |  |  |  |  |  |  |  |
|  | Weighted mode | 1.142 | 0.994 | 1.313 | 0.089 |  |  |  |  |  |  |  |
|  | BWMR | 1.103 | 1.094 | 1.223 | 0.044 |  |  |  |  |  |  |  |
| **UC** |  |  |  |  |  |  |  |  |  |  |  |  |
| CCL4 | MR Egger | 1.159 | 0.934 | 1.438 | 0.228 | 27.441 | 0.000 | 27.773 | 0.000 | -0.009 | 0.797 | 0.239 |
|  | Weighted median | 1.133 | 0.993 | 1.294 | 0.064 |  |  |  |  |  |  |  |
|  | IVW | 1.295 | 1.132 | 1.482 | 0.000 |  |  |  |  |  |  |  |
|  | Simple mode | 1.128 | 0.833 | 1.527 | 0.462 |  |  |  |  |  |  |  |
|  | Weighted mode | 1.164 | 1.055 | 1.284 | 0.019 |  |  |  |  |  |  |  |
|  | BWMR | 1.128 | 1.074 | 1.305 | 0.011 |  |  |  |  |  |  |  |
| FGF-5 | MR Egger | 0.939 | 0.756 | 1.167 | 0.672 | 0.501 | 0.479 | 2.124 | 0.346 | 0.064 | 0.424 | 0.413 |
|  | Weighted median | 1.070 | 0.996 | 1.150 | 0.065 |  |  |  |  |  |  |  |
|  | IVW | 1.072 | 0.995 | 1.156 | 0.069 |  |  |  |  |  |  |  |
|  | Simple mode | 1.354 | 1.001 | 1.830 | 0.188 |  |  |  |  |  |  |  |
|  | Weighted mode | 1.065 | 0.988 | 1.147 | 0.243 |  |  |  |  |  |  |  |
|  | BWMR | 1.075 | 0.988 | 1.171 | 0.094 |  |  |  |  |  |  |  |
| **CD** |  |  |  |  |  |  |  |  |  |  |  |  |
| Axin-1 | MR Egger | 1.107 | 0.148 | 8.298 | 0.937 | 1.445 | 0.229 | 1.542 | 0.463 | 0.024 | 0.839 | 0.675 |
|  | Weighted median | 1.458 | 0.700 | 3.037 | 0.314 |  |  |  |  |  |  |  |
|  | IVW | 1.418 | 0.774 | 2.597 | 0.258 |  |  |  |  |  |  |  |
|  | Simple mode | 2.017 | 0.749 | 5.435 | 0.300 |  |  |  |  |  |  |  |
|  | Weighted mode | 1.870 | 0.656 | 5.333 | 0.362 |  |  |  |  |  |  |  |
|  | BWMR | 1.429 | 0.756 | 2.701 | 0.271 |  |  |  |  |  |  |  |
| CCL19 | MR Egger | 1.515 | 1.026 | 2.238 | 0.105 | 9.648 | 0.047 | 14.808 | 0.011 | -0.059 | 0.217 | 0.901 |
|  | Weighted median | 1.217 | 0.916 | 1.616 | 0.175 |  |  |  |  |  |  |  |
|  | IVW | 1.423 | 1.145 | 1.768 | 0.001 |  |  |  |  |  |  |  |
|  | Simple mode | 0.927 | 0.449 | 1.916 | 0.846 |  |  |  |  |  |  |  |
|  | Weighted mode | 1.455 | 1.168 | 1.812 | 0.020 |  |  |  |  |  |  |  |
|  | BWMR | 1.243 | 1.029 | 1.596 | 0.007 |  |  |  |  |  |  |  |
| CD5 | MR Egger | 2.375 | 1.236 | 4.565 | 0.025 | 9.003 | 0.622 | 10.606 | 0.563 | -0.048 | 0.232 | 0.157 |
|  | Weighted median | 1.649 | 1.218 | 2.231 | 0.001 |  |  |  |  |  |  |  |
|  | IVW | 1.593 | 1.291 | 1.966 | 0.000 |  |  |  |  |  |  |  |
|  | Simple mode | 1.547 | 0.934 | 2.562 | 0.116 |  |  |  |  |  |  |  |
|  | Weighted mode | 1.630 | 1.142 | 2.326 | 0.020 |  |  |  |  |  |  |  |
|  | BWMR | 1.602 | 1.289 | 1.990 | 0.000 |  |  |  |  |  |  |  |
| CD6 | MR Egger | 1.293 | 1.029 | 1.625 | 0.079 | 9.153 | 0.103 | 9.214 | 0.162 | -0.005 | 0.862 | 0.294 |
|  | Weighted median | 1.280 | 1.130 | 1.450 | 0.000 |  |  |  |  |  |  |  |
|  | IVW | 1.274 | 1.095 | 1.482 | 0.002 |  |  |  |  |  |  |  |
|  | Simple mode | 1.443 | 1.019 | 2.043 | 0.085 |  |  |  |  |  |  |  |
|  | Weighted mode | 1.280 | 1.125 | 1.456 | 0.009 |  |  |  |  |  |  |  |
|  | BWMR | 1.272 | 1.093 | 1.479 | 0.002 |  |  |  |  |  |  |  |
| Abbreviations: IVW: inverse variance weighted; BWMR: Bayesian Weighted Mendelian Randomization; β:beta; OR: odds ratio. | | | | | | | | | | | | |

| **Supplementary Table S16. MR analysis results of associations between lipidome and inflammatory proteins after the initial screening.** | | | | | | | | | | | | | | |
| --- | --- | --- | --- | --- | --- | --- | --- | --- | --- | --- | --- | --- | --- | --- |
| **Exposure** | **Outcome** | **Method** | **Nsnp** | **OR** | **OR_lci95** | **OR_uci95** | **P** | **Heterogeneity** | | | | **Pleiotropy** | | |
|  |  |  |  |  |  |  |  | **MR Egger** | | **IVW** | | **MR-Egger intercep** | | **MR-PRESO** |
|  |  |  |  |  |  |  |  | **Q** | **P** | **Q** | **P** | **intercept** | **P** | **P** |
| Sterol ester (27:1/20:4) | CD6 | MR Egger | 30 | 0.963 | 0.922 | 1.006 | 0.099 | 29.250 | 0.400 | 29.291 | 0.450 | 0.001 | 0.844 | 0.557 |
|  |  | Weighted median | 30 | 0.966 | 0.933 | 1.001 | 0.054 |  |  |  |  |  |  |  |
|  |  | IVW | 30 | 0.966 | 0.938 | 0.995 | 0.022 |  |  |  |  |  |  |  |
|  |  | Simple mode | 30 | 0.955 | 0.864 | 1.054 | 0.367 |  |  |  |  |  |  |  |
|  |  | Weighted mode | 30 | 0.966 | 0.932 | 1.000 | 0.062 |  |  |  |  |  |  |  |
|  |  | BWMR | 30 | 0.967 | 0.936 | 0.998 | 0.039 |  |  |  |  |  |  |  |
| Sterol ester (27:1/22:6) | CCL4 | MR Egger | 23 | 0.861 | 0.755 | 0.982 | 0.036 | 23.668 | 0.309 | 24.945 | 0.300 | 0.011 | 0.299 | 0.332 |
|  |  | Weighted median | 23 | 0.923 | 0.850 | 1.002 | 0.057 |  |  |  |  |  |  |  |
|  |  | IVW | 23 | 0.918 | 0.866 | 0.973 | 0.004 |  |  |  |  |  |  |  |
|  |  | Simple mode | 23 | 0.911 | 0.782 | 1.060 | 0.238 |  |  |  |  |  |  |  |
|  |  | Weighted mode | 23 | 0.913 | 0.818 | 1.018 | 0.117 |  |  |  |  |  |  |  |
|  |  | BWMR | 23 | 0.915 | 0.864 | 0.969 | 0.002 |  |  |  |  |  |  |  |
| Sterol ester (27:1/22:6) | CD6 | MR Egger | 23 | 0.858 | 0.759 | 0.970 | 0.023 | 19.689 | 0.541 | 22.577 | 0.426 | 0.016 | 0.104 | 0.426 |
|  |  | Weighted median | 23 | 0.956 | 0.881 | 1.038 | 0.288 |  |  |  |  |  |  |  |
|  |  | IVW | 23 | 0.944 | 0.893 | 0.997 | 0.038 |  |  |  |  |  |  |  |
|  |  | Simple mode | 23 | 0.963 | 0.822 | 1.127 | 0.642 |  |  |  |  |  |  |  |
|  |  | Weighted mode | 23 | 0.957 | 0.835 | 1.096 | 0.528 |  |  |  |  |  |  |  |
|  |  | BWMR | 23 | 0.941 | 0.887 | 0.998 | 0.041 |  |  |  |  |  |  |  |
|  |  |  |  |  |  |  |  |  |  |  |  |  |  |  |
| Phosphatidylcholine (20:4_0:0) | CD6 | MR Egger | 27 | 0.957 | 0.904 | 1.012 | 0.134 | 24.760 | 0.476 | 24.777 | 0.532 | 0.001 | 0.898 | 0.536 |
|  |  | Weighted median | 27 | 0.947 | 0.903 | 0.994 | 0.027 |  |  |  |  |  |  |  |
|  |  | IVW | 27 | 0.959 | 0.926 | 0.994 | 0.021 |  |  |  |  |  |  |  |
|  |  | Simple mode | 27 | 0.981 | 0.878 | 1.096 | 0.736 |  |  |  |  |  |  |  |
|  |  | Weighted mode | 27 | 0.951 | 0.905 | 0.998 | 0.052 |  |  |  |  |  |  |  |
|  |  | BWMR | 27 | 0.953 | 0.915 | 0.992 | 0.020 |  |  |  |  |  |  |  |
| Phosphatidylcholine (16:0_20:4) | CCL4 | MR Egger | 24 | 0.970 | 0.920 | 1.022 | 0.266 | 24.209 | 0.336 | 24.319 | 0.386 | -0.002 | 0.754 | 0.501 |
|  |  | Weighted median | 24 | 0.969 | 0.934 | 1.005 | 0.091 |  |  |  |  |  |  |  |
|  |  | IVW | 24 | 0.964 | 0.931 | 0.998 | 0.036 |  |  |  |  |  |  |  |
|  |  | Simple mode | 24 | 0.968 | 0.849 | 1.103 | 0.629 |  |  |  |  |  |  |  |
|  |  | Weighted mode | 24 | 0.968 | 0.930 | 1.007 | 0.118 |  |  |  |  |  |  |  |
|  |  | BWMR | 24 | 0.963 | 0.932 | 0.996 | 0.028 |  |  |  |  |  |  |  |
| Phosphatidylcholine (16:0_20:4) | CD6 | MR Egger | 24 | 0.966 | 0.909 | 1.027 | 0.284 | 33.808 | 0.051 | 34.651 | 0.056 | -0.005 | 0.467 | 0.121 |
|  |  | Weighted median | 24 | 0.954 | 0.918 | 0.991 | 0.016 |  |  |  |  |  |  |  |
|  |  | IVW | 24 | 0.950 | 0.912 | 0.989 | 0.013 |  |  |  |  |  |  |  |
|  |  | Simple mode | 24 | 1.043 | 0.913 | 1.190 | 0.542 |  |  |  |  |  |  |  |
|  |  | Weighted mode | 24 | 0.956 | 0.917 | 0.997 | 0.048 |  |  |  |  |  |  |  |
|  |  | BWMR | 24 | 0.949 | 0.909 | 0.991 | 0.018 |  |  |  |  |  |  |  |
| Phosphatidylcholine (17:0_18:2) | CCL4 | MR Egger | 25 | 1.080 | 0.957 | 1.218 | 0.223 | 13.824 | 0.932 | 14.060 | 0.945 | -0.004 | 0.632 | 0.946 |
|  |  | Weighted median | 25 | 1.076 | 0.997 | 1.162 | 0.061 |  |  |  |  |  |  |  |
|  |  | IVW | 25 | 1.051 | 1.001 | 1.104 | 0.047 |  |  |  |  |  |  |  |
|  |  | Simple mode | 25 | 1.088 | 0.965 | 1.226 | 0.182 |  |  |  |  |  |  |  |
|  |  | Weighted mode | 25 | 1.080 | 0.989 | 1.179 | 0.100 |  |  |  |  |  |  |  |
|  |  | BWMR | 25 | 1.052 | 1.000 | 1.106 | 0.051 |  |  |  |  |  |  |  |
| Phosphatidylcholine (17:0_18:2) | TNFSF12 | MR Egger | 25 | 0.978 | 0.867 | 1.104 | 0.725 | 24.391 | 0.382 | 24.848 | 0.414 | -0.006 | 0.518 | 0.459 |
|  |  | Weighted median | 25 | 0.940 | 0.874 | 1.011 | 0.098 |  |  |  |  |  |  |  |
|  |  | IVW | 25 | 0.943 | 0.898 | 0.990 | 0.018 |  |  |  |  |  |  |  |
|  |  | Simple mode | 25 | 0.932 | 0.828 | 1.049 | 0.255 |  |  |  |  |  |  |  |
|  |  | Weighted mode | 25 | 0.936 | 0.857 | 1.022 | 0.151 |  |  |  |  |  |  |  |
|  |  | BWMR | 25 | 0.942 | 0.891 | 0.996 | 0.034 |  |  |  |  |  |  |  |
| Phosphatidylcholine (18:1_20:4) levels | CD6 | MR Egger | 27 | 0.982 | 0.913 | 1.055 | 0.620 | 43.749 | 0.012 | 46.576 | 0.008 | -0.008 | 0.215 | 0.014 |
|  |  | Weighted median | 27 | 0.948 | 0.907 | 0.991 | 0.018 |  |  |  |  |  |  |  |
|  |  | IVW | 27 | 0.947 | 0.903 | 0.994 | 0.027 |  |  |  |  |  |  |  |
|  |  | Simple mode | 27 | 0.928 | 0.815 | 1.056 | 0.265 |  |  |  |  |  |  |  |
|  |  | Weighted mode | 27 | 0.949 | 0.907 | 0.993 | 0.033 |  |  |  |  |  |  |  |
|  |  | BWMR | 27 | 0.948 | 0.905 | 0.994 | 0.027 |  |  |  |  |  |  |  |
| Sphingomyelin (d38:1) | CCL4 | MR Egger | 38 | 0.977 | 0.894 | 1.068 | 0.611 | 47.064 | 0.103 | 47.603 | 0.114 | -0.004 | 0.525 | 0.143 |
|  |  | Weighted median | 38 | 0.984 | 0.926 | 1.045 | 0.602 |  |  |  |  |  |  |  |
|  |  | IVW | 38 | 0.953 | 0.911 | 0.996 | 0.034 |  |  |  |  |  |  |  |
|  |  | Simple mode | 38 | 0.980 | 0.882 | 1.089 | 0.711 |  |  |  |  |  |  |  |
|  |  | Weighted mode | 38 | 0.991 | 0.933 | 1.053 | 0.779 |  |  |  |  |  |  |  |
|  |  | BWMR | 38 | 0.951 | 0.912 | 0.992 | 0.018 |  |  |  |  |  |  |  |
| Sphingomyelin (d38:1) | CD6 | MR Egger | 38 | 0.967 | 0.888 | 1.053 | 0.444 | 43.696 | 0.177 | 43.811 | 0.205 | -0.002 | 0.760 | 0.214 |
|  |  | Weighted median | 38 | 0.954 | 0.896 | 1.014 | 0.131 |  |  |  |  |  |  |  |
|  |  | IVW | 38 | 0.956 | 0.916 | 0.998 | 0.038 |  |  |  |  |  |  |  |
|  |  | Simple mode | 38 | 0.931 | 0.823 | 1.055 | 0.269 |  |  |  |  |  |  |  |
|  |  | Weighted mode | 38 | 0.965 | 0.905 | 1.028 | 0.274 |  |  |  |  |  |  |  |
|  |  | BWMR | 38 | 0.955 | 0.917 | 0.995 | 0.026 |  |  |  |  |  |  |  |
| Sphingomyelin (d42:2) | CD6 | MR Egger | 38 | 0.993 | 0.908 | 1.086 | 0.875 | 36.671 | 0.438 | 38.579 | 0.398 | -0.008 | 0.180 | 0.392 |
|  |  | Weighted median | 38 | 0.951 | 0.890 | 1.015 | 0.127 |  |  |  |  |  |  |  |
|  |  | IVW | 38 | 0.941 | 0.899 | 0.984 | 0.008 |  |  |  |  |  |  |  |
|  |  | Simple mode | 38 | 0.930 | 0.808 | 1.069 | 0.314 |  |  |  |  |  |  |  |
|  |  | Weighted mode | 38 | 0.952 | 0.830 | 1.092 | 0.485 |  |  |  |  |  |  |  |
|  |  | BWMR | 38 | 0.935 | 0.891 | 0.983 | 0.008 |  |  |  |  |  |  |  |
| Abbreviations: IVW: inverse variance weighted; BWMR: Bayesian Weighted Mendelian Randomization; β:beta; OR: odds ratio. | | | | | | | | | | | | | | |

| **Supplementary Table S17. Association of genome-wide SNPs for lipidome with inflammatory proteins** | | | | | | | | |
| --- | --- | --- | --- | --- | --- | --- | --- | --- |
| **Exposure** | **Outcome** | **SNP** | **Beta outcome** | **EAF outcome** | **SE outcome** | **P outcome** | **R^2^** | **F-statistic** |
| Sterol ester (27:1/20:4) | CD6 | rs11022403 | 0.029 | 0.242 | 0.015 | 0.047 | 0.003 | 21.220 |
|  |  | rs113248417 | 0.023 | 0.027 | 0.038 | 0.550 | 0.005 | 35.544 |
|  |  | rs1135999 | -0.006 | 0.310 | 0.013 | 0.609 | 0.005 | 32.506 |
|  |  | rs113603971 | -0.017 | 0.037 | 0.036 | 0.645 | 0.003 | 19.555 |
|  |  | rs11591147 | 0.126 | 0.019 | 0.047 | 0.007 | 0.006 | 41.421 |
|  |  | rs117084735 | -0.014 | 0.019 | 0.045 | 0.757 | 0.003 | 20.353 |
|  |  | rs117186386 | 0.013 | 0.053 | 0.028 | 0.653 | 0.004 | 25.459 |
|  |  | rs117825513 | 0.062 | 0.018 | 0.052 | 0.231 | 0.003 | 20.318 |
|  |  | rs138482835 | -0.001 | 0.038 | 0.040 | 0.984 | 0.003 | 22.278 |
|  |  | rs147981159 | 0.010 | 0.040 | 0.032 | 0.754 | 0.008 | 60.908 |
|  |  | rs174528 | 0.022 | 0.378 | 0.012 | 0.064 | 0.202 | 1819.849 |
|  |  | rs1800961 | 0.008 | 0.037 | 0.032 | 0.795 | 0.003 | 20.532 |
|  |  | rs182695896 | -0.092 | 0.009 | 0.071 | 0.196 | 0.004 | 31.741 |
|  |  | rs186913978 | 0.017 | 0.021 | 0.046 | 0.719 | 0.004 | 28.720 |
|  |  | rs187429064 | 0.065 | 0.025 | 0.053 | 0.216 | 0.006 | 46.088 |
|  |  | rs2229738 | 0.023 | 0.105 | 0.021 | 0.284 | 0.006 | 42.633 |
|  |  | rs223841 | -0.012 | 0.180 | 0.015 | 0.449 | 0.003 | 21.183 |
|  |  | rs2469034 | 0.013 | 0.814 | 0.015 | 0.397 | 0.003 | 20.122 |
|  |  | rs3741252 | -0.004 | 0.096 | 0.022 | 0.854 | 0.010 | 74.744 |
|  |  | rs3767941 | -0.012 | 0.182 | 0.016 | 0.445 | 0.003 | 22.617 |
|  |  | rs3820438 | -0.001 | 0.241 | 0.014 | 0.955 | 0.003 | 21.528 |
|  |  | rs4382917 | -0.025 | 0.221 | 0.015 | 0.097 | 0.008 | 55.143 |
|  |  | rs635196 | 0.016 | 0.221 | 0.014 | 0.257 | 0.003 | 23.862 |
|  |  | rs646299 | 0.011 | 0.563 | 0.012 | 0.372 | 0.003 | 19.672 |
|  |  | rs6812370 | -0.089 | 0.016 | 0.052 | 0.084 | 0.004 | 27.573 |
|  |  | rs6860806 | -0.016 | 0.517 | 0.012 | 0.156 | 0.003 | 20.015 |
|  |  | rs7020 | -0.008 | 0.461 | 0.012 | 0.465 | 0.003 | 19.900 |
|  |  | rs7412 | 0.006 | 0.077 | 0.022 | 0.791 | 0.010 | 70.977 |
|  |  | rs74915447 | -0.006 | 0.032 | 0.037 | 0.866 | 0.003 | 20.006 |
|  |  | rs7837272 | -0.044 | 0.014 | 0.074 | 0.549 | 0.003 | 21.080 |
|  |  | rs79136768 | 0.036 | 0.024 | 0.040 | 0.372 | 0.003 | 24.145 |
|  |  | rs9388335 | 0.023 | 0.512 | 0.012 | 0.059 | 0.003 | 21.634 |
|  |  | rs9555673 | 0.008 | 0.413 | 0.012 | 0.539 | 0.003 | 20.745 |
| Sterol ester (27:1/22:6) | CCL4 | rs10150075 | 0.016 | 0.099 | 0.020 | 0.440 | 0.004 | 28.636 |
|  |  | rs111751059 | 0.053 | 0.076 | 0.022 | 0.017 | 0.003 | 20.167 |
|  |  | rs113651041 | -0.016 | 0.392 | 0.012 | 0.180 | 0.003 | 19.538 |
|  |  | rs115647337 | 0.026 | 0.025 | 0.042 | 0.541 | 0.003 | 21.698 |
|  |  | rs11591147 | 0.032 | 0.019 | 0.047 | 0.494 | 0.004 | 25.783 |
|  |  | rs11633878 | 0.006 | 0.692 | 0.013 | 0.644 | 0.003 | 22.651 |
|  |  | rs116794590 | -0.003 | 0.031 | 0.037 | 0.934 | 0.003 | 21.674 |
|  |  | rs11696517 | 0.008 | 0.122 | 0.018 | 0.663 | 0.003 | 19.916 |
|  |  | rs117061362 | -0.034 | 0.031 | 0.041 | 0.413 | 0.003 | 23.519 |
|  |  | rs149229417 | 0.008 | 0.034 | 0.037 | 0.826 | 0.003 | 23.251 |
|  |  | rs17110517 | -0.044 | 0.051 | 0.030 | 0.140 | 0.003 | 24.431 |
|  |  | rs2075083 | -0.006 | 0.517 | 0.012 | 0.629 | 0.003 | 19.647 |
|  |  | rs2229738 | 0.000 | 0.105 | 0.022 | 0.989 | 0.003 | 23.485 |
|  |  | rs35473591 | 0.020 | 0.338 | 0.014 | 0.157 | 0.019 | 141.775 |
|  |  | rs4072727 | -0.013 | 0.316 | 0.013 | 0.303 | 0.004 | 29.085 |
|  |  | rs72657952 | -0.080 | 0.029 | 0.038 | 0.036 | 0.003 | 19.945 |
|  |  | rs72852391 | -0.087 | 0.034 | 0.044 | 0.050 | 0.003 | 22.458 |
|  |  | rs73769662 | -0.003 | 0.126 | 0.018 | 0.849 | 0.003 | 19.666 |
|  |  | rs7412 | 0.046 | 0.078 | 0.023 | 0.043 | 0.007 | 52.155 |
|  |  | rs75101588 | 0.024 | 0.045 | 0.030 | 0.430 | 0.004 | 26.866 |
|  |  | rs77456635 | 0.074 | 0.033 | 0.034 | 0.030 | 0.003 | 25.055 |
|  |  | rs77708260 | -0.051 | 0.015 | 0.059 | 0.381 | 0.003 | 20.104 |
|  |  | rs9559135 | 0.008 | 0.310 | 0.013 | 0.528 | 0.003 | 21.738 |
| Sterol ester (27:1/22:6) | CD6 | rs10150075 | -0.022 | 0.099 | 0.020 | 0.276 | 0.004 | 28.636 |
|  |  | rs111751059 | 0.010 | 0.076 | 0.022 | 0.661 | 0.003 | 20.167 |
|  |  | rs113651041 | 0.016 | 0.392 | 0.012 | 0.186 | 0.003 | 19.538 |
|  |  | rs115647337 | -0.001 | 0.025 | 0.042 | 0.985 | 0.003 | 21.698 |
|  |  | rs11591147 | 0.126 | 0.019 | 0.047 | 0.007 | 0.004 | 25.783 |
|  |  | rs11633878 | -0.012 | 0.692 | 0.013 | 0.368 | 0.003 | 22.651 |
|  |  | rs116794590 | -0.030 | 0.031 | 0.037 | 0.425 | 0.003 | 21.674 |
|  |  | rs11696517 | 0.020 | 0.122 | 0.018 | 0.264 | 0.003 | 19.916 |
|  |  | rs117061362 | -0.008 | 0.031 | 0.041 | 0.838 | 0.003 | 23.519 |
|  |  | rs149229417 | -0.071 | 0.034 | 0.037 | 0.056 | 0.003 | 23.251 |
|  |  | rs17110517 | 0.023 | 0.051 | 0.029 | 0.437 | 0.003 | 24.431 |
|  |  | rs2075083 | 0.001 | 0.517 | 0.012 | 0.939 | 0.003 | 19.647 |
|  |  | rs2229738 | 0.023 | 0.105 | 0.021 | 0.284 | 0.003 | 23.485 |
|  |  | rs35473591 | 0.032 | 0.338 | 0.014 | 0.020 | 0.019 | 141.775 |
|  |  | rs4072727 | 0.004 | 0.316 | 0.013 | 0.751 | 0.004 | 29.085 |
|  |  | rs72657952 | 0.005 | 0.029 | 0.038 | 0.900 | 0.003 | 19.945 |
|  |  | rs72852391 | -0.052 | 0.034 | 0.045 | 0.243 | 0.003 | 22.458 |
|  |  | rs73769662 | 0.005 | 0.126 | 0.018 | 0.782 | 0.003 | 19.666 |
|  |  | rs7412 | 0.006 | 0.077 | 0.022 | 0.791 | 0.007 | 52.155 |
|  |  | rs75101588 | -0.014 | 0.045 | 0.030 | 0.644 | 0.004 | 26.866 |
|  |  | rs77456635 | 0.033 | 0.033 | 0.034 | 0.333 | 0.003 | 25.055 |
|  |  | rs77708260 | -0.023 | 0.015 | 0.058 | 0.697 | 0.003 | 20.104 |
|  |  | rs9559135 | 0.004 | 0.310 | 0.013 | 0.752 | 0.003 | 21.738 |
| Phosphatidylcholine (20:4_0:0) | CD6 | rs10733608 | -0.015 | 0.468 | 0.012 | 0.203 | 0.003 | 21.648 |
|  |  | rs10884272 | -0.009 | 0.482 | 0.012 | 0.470 | 0.003 | 20.372 |
|  |  | rs11669519 | 0.014 | 0.346 | 0.013 | 0.256 | 0.003 | 20.381 |
|  |  | rs12226389 | -0.004 | 0.204 | 0.015 | 0.809 | 0.009 | 54.075 |
|  |  | rs12303 | 0.020 | 0.177 | 0.016 | 0.208 | 0.003 | 21.040 |
|  |  | rs12905466 | -0.022 | 0.230 | 0.014 | 0.114 | 0.003 | 20.862 |
|  |  | rs1363011 | 0.007 | 0.146 | 0.016 | 0.678 | 0.003 | 19.537 |
|  |  | rs139516883 | 0.005 | 0.020 | 0.049 | 0.921 | 0.004 | 24.495 |
|  |  | rs142999489 | 0.031 | 0.030 | 0.036 | 0.386 | 0.004 | 23.796 |
|  |  | rs150158785 | -0.007 | 0.066 | 0.025 | 0.782 | 0.003 | 20.086 |
|  |  | rs182611493 | 0.106 | 0.023 | 0.055 | 0.053 | 0.004 | 26.444 |
|  |  | rs191895023 | -0.062 | 0.009 | 0.099 | 0.535 | 0.003 | 20.639 |
|  |  | rs200932481 | 0.001 | 0.018 | 0.057 | 0.992 | 0.004 | 22.994 |
|  |  | rs35473591 | 0.032 | 0.338 | 0.014 | 0.020 | 0.137 | 991.669 |
|  |  | rs35599386 | -0.034 | 0.062 | 0.028 | 0.212 | 0.003 | 20.136 |
|  |  | rs3741252 | -0.004 | 0.096 | 0.022 | 0.854 | 0.006 | 39.579 |
|  |  | rs4708901 | 0.001 | 0.207 | 0.015 | 0.973 | 0.003 | 20.729 |
|  |  | rs474901 | -0.031 | 0.444 | 0.012 | 0.009 | 0.003 | 19.839 |
|  |  | rs529446 | -0.008 | 0.765 | 0.014 | 0.554 | 0.003 | 20.001 |
|  |  | rs600626 | 0.027 | 0.186 | 0.015 | 0.068 | 0.005 | 32.513 |
|  |  | rs62511275 | -0.007 | 0.057 | 0.029 | 0.820 | 0.004 | 22.556 |
|  |  | rs6564238 | -0.011 | 0.299 | 0.014 | 0.419 | 0.003 | 20.071 |
|  |  | rs7179330 | -0.002 | 0.977 | 0.044 | 0.973 | 0.004 | 23.702 |
|  |  | rs76469829 | -0.029 | 0.038 | 0.033 | 0.390 | 0.003 | 20.617 |
|  |  | rs76513766 | -0.009 | 0.041 | 0.031 | 0.762 | 0.003 | 20.216 |
|  |  | rs7936002 | -0.026 | 0.227 | 0.015 | 0.084 | 0.007 | 40.967 |
|  |  | rs79995028 | -0.081 | 0.014 | 0.057 | 0.151 | 0.003 | 20.562 |
|  |  | rs9967725 | 0.005 | 0.296 | 0.013 | 0.723 | 0.005 | 29.177 |
| Phosphatidylcholine (16:0_20:4) | CCL4 | rs11062107 | 0.004 | 0.178 | 0.016 | 0.810 | 0.003 | 19.709 |
|  |  | rs113394924 | -0.021 | 0.041 | 0.032 | 0.524 | 0.007 | 51.998 |
|  |  | rs116438525 | 0.145 | 0.011 | 0.071 | 0.040 | 0.003 | 21.266 |
|  |  | rs116643980 | -0.003 | 0.023 | 0.045 | 0.941 | 0.003 | 20.924 |
|  |  | rs1251003 | -0.012 | 0.080 | 0.022 | 0.595 | 0.003 | 23.562 |
|  |  | rs12720922 | 0.016 | 0.188 | 0.015 | 0.292 | 0.003 | 19.717 |
|  |  | rs13329893 | -0.009 | 0.055 | 0.028 | 0.739 | 0.003 | 19.700 |
|  |  | rs141391095 | -0.037 | 0.035 | 0.035 | 0.296 | 0.003 | 22.132 |
|  |  | rs147549994 | 0.066 | 0.014 | 0.062 | 0.281 | 0.004 | 29.578 |
|  |  | rs149130695 | 0.007 | 0.071 | 0.026 | 0.772 | 0.003 | 19.659 |
|  |  | rs17017629 | -0.042 | 0.042 | 0.030 | 0.160 | 0.003 | 20.281 |
|  |  | rs17366743 | 0.020 | 0.029 | 0.037 | 0.590 | 0.003 | 19.594 |
|  |  | rs174527 | 0.008 | 0.402 | 0.013 | 0.522 | 0.015 | 111.162 |
|  |  | rs174533 | 0.018 | 0.351 | 0.012 | 0.135 | 0.171 | 1480.882 |
|  |  | rs187429064 | 0.098 | 0.025 | 0.052 | 0.060 | 0.005 | 36.009 |
|  |  | rs2943813 | 0.019 | 0.792 | 0.015 | 0.195 | 0.006 | 46.464 |
|  |  | rs2966427 | 0.028 | 0.895 | 0.019 | 0.146 | 0.003 | 20.014 |
|  |  | rs3018617 | -0.004 | 0.351 | 0.013 | 0.739 | 0.005 | 33.199 |
|  |  | rs3820438 | 0.000 | 0.241 | 0.014 | 0.983 | 0.003 | 23.703 |
|  |  | rs4756323 | 0.027 | 0.472 | 0.012 | 0.026 | 0.003 | 21.709 |
|  |  | rs499974 | 0.006 | 0.186 | 0.015 | 0.716 | 0.004 | 26.845 |
|  |  | rs58160859 | 0.003 | 0.234 | 0.014 | 0.822 | 0.003 | 20.602 |
|  |  | rs6498540 | -0.015 | 0.315 | 0.013 | 0.222 | 0.003 | 19.851 |
|  |  | rs72709529 | 0.030 | 0.079 | 0.023 | 0.184 | 0.003 | 23.734 |
|  |  | rs74754540 | -0.012 | 0.028 | 0.038 | 0.761 | 0.003 | 21.202 |
| Phosphatidylcholine (16:0_20:4) | CD6 | rs11062107 | -0.004 | 0.178 | 0.016 | 0.809 | 0.003 | 19.709 |
|  |  | rs113394924 | 0.024 | 0.040 | 0.032 | 0.453 | 0.007 | 51.998 |
|  |  | rs116438525 | 0.073 | 0.011 | 0.070 | 0.302 | 0.003 | 21.266 |
|  |  | rs116643980 | 0.007 | 0.022 | 0.044 | 0.869 | 0.003 | 20.924 |
|  |  | rs1251003 | 0.024 | 0.080 | 0.022 | 0.267 | 0.003 | 23.562 |
|  |  | rs12720922 | 0.013 | 0.188 | 0.015 | 0.369 | 0.003 | 19.717 |
|  |  | rs13329893 | 0.012 | 0.055 | 0.027 | 0.660 | 0.003 | 19.700 |
|  |  | rs141391095 | -0.017 | 0.035 | 0.035 | 0.627 | 0.003 | 22.132 |
|  |  | rs147549994 | 0.098 | 0.014 | 0.062 | 0.112 | 0.004 | 29.578 |
|  |  | rs149130695 | 0.006 | 0.071 | 0.025 | 0.825 | 0.003 | 19.659 |
|  |  | rs17017629 | -0.053 | 0.041 | 0.030 | 0.078 | 0.003 | 20.281 |
|  |  | rs17366743 | -0.034 | 0.029 | 0.037 | 0.355 | 0.003 | 19.594 |
|  |  | rs174527 | 0.022 | 0.402 | 0.013 | 0.092 | 0.015 | 111.162 |
|  |  | rs174533 | 0.028 | 0.351 | 0.012 | 0.022 | 0.171 | 1480.882 |
|  |  | rs187429064 | 0.065 | 0.025 | 0.053 | 0.216 | 0.005 | 36.009 |
|  |  | rs2943813 | 0.056 | 0.792 | 0.014 | 0.000 | 0.006 | 46.464 |
|  |  | rs2966427 | -0.011 | 0.895 | 0.019 | 0.558 | 0.003 | 20.014 |
|  |  | rs3018617 | 0.024 | 0.352 | 0.013 | 0.072 | 0.005 | 33.199 |
|  |  | rs3820438 | -0.001 | 0.241 | 0.014 | 0.955 | 0.003 | 23.703 |
|  |  | rs4756323 | 0.008 | 0.472 | 0.012 | 0.480 | 0.003 | 21.709 |
|  |  | rs499974 | 0.028 | 0.186 | 0.015 | 0.065 | 0.004 | 26.845 |
|  |  | rs58160859 | 0.001 | 0.234 | 0.014 | 0.927 | 0.003 | 20.602 |
|  |  | rs6498540 | -0.006 | 0.315 | 0.013 | 0.660 | 0.003 | 19.851 |
|  |  | rs72709529 | 0.022 | 0.079 | 0.023 | 0.332 | 0.003 | 23.734 |
|  |  | rs74754540 | 0.061 | 0.028 | 0.038 | 0.107 | 0.003 | 21.202 |
| Phosphatidylcholine (17:0_18:2) | CCL4 | rs11027519 | -0.001 | 0.111 | 0.019 | 0.945 | 0.003 | 19.950 |
|  |  | rs111233850 | 0.022 | 0.034 | 0.034 | 0.513 | 0.003 | 20.199 |
|  |  | rs111314022 | 0.014 | 0.074 | 0.025 | 0.566 | 0.003 | 20.345 |
|  |  | rs115686032 | -0.017 | 0.031 | 0.043 | 0.697 | 0.003 | 21.606 |
|  |  | rs1168104 | 0.014 | 0.674 | 0.012 | 0.276 | 0.003 | 20.877 |
|  |  | rs1253283 | -0.033 | 0.049 | 0.030 | 0.262 | 0.003 | 19.660 |
|  |  | rs12548274 | 0.017 | 0.101 | 0.021 | 0.409 | 0.003 | 22.095 |
|  |  | rs141509040 | -0.001 | 0.057 | 0.027 | 0.970 | 0.003 | 20.086 |
|  |  | rs1609446 | 0.013 | 0.115 | 0.019 | 0.517 | 0.003 | 19.967 |
|  |  | rs17231506 | -0.007 | 0.318 | 0.012 | 0.595 | 0.004 | 27.186 |
|  |  | rs174566 | 0.020 | 0.352 | 0.012 | 0.109 | 0.032 | 237.533 |
|  |  | rs1800588 | -0.010 | 0.229 | 0.014 | 0.488 | 0.005 | 35.068 |
|  |  | rs192537901 | -0.045 | 0.014 | 0.060 | 0.451 | 0.003 | 23.785 |
|  |  | rs2126260 | -0.007 | 0.790 | 0.014 | 0.647 | 0.003 | 22.916 |
|  |  | rs34489224 | 0.004 | 0.266 | 0.014 | 0.781 | 0.003 | 21.413 |
|  |  | rs422137 | 0.010 | 0.444 | 0.012 | 0.409 | 0.004 | 25.903 |
|  |  | rs4478142 | -0.010 | 0.238 | 0.014 | 0.451 | 0.003 | 20.039 |
|  |  | rs4840981 | 0.039 | 0.976 | 0.040 | 0.338 | 0.003 | 21.265 |
|  |  | rs57518551 | 0.012 | 0.112 | 0.019 | 0.544 | 0.004 | 25.838 |
|  |  | rs6498540 | -0.015 | 0.315 | 0.013 | 0.222 | 0.004 | 25.828 |
|  |  | rs72747028 | -0.043 | 0.064 | 0.025 | 0.086 | 0.003 | 21.691 |
|  |  | rs73058910 | -0.002 | 0.047 | 0.034 | 0.960 | 0.003 | 21.390 |
|  |  | rs7437408 | 0.016 | 0.495 | 0.012 | 0.166 | 0.003 | 21.048 |
|  |  | rs78592970 | -0.010 | 0.147 | 0.018 | 0.562 | 0.004 | 25.234 |
|  |  | rs964184 | -0.006 | 0.847 | 0.016 | 0.736 | 0.006 | 44.464 |
| Phosphatidylcholine (17:0_18:2) | TNFSF12 | rs11027519 | 0.015 | 0.111 | 0.019 | 0.429 | 0.003 | 19.950 |
|  |  | rs111233850 | -0.026 | 0.034 | 0.033 | 0.430 | 0.003 | 20.199 |
|  |  | rs111314022 | 0.004 | 0.074 | 0.025 | 0.883 | 0.003 | 20.345 |
|  |  | rs115686032 | 0.048 | 0.031 | 0.043 | 0.269 | 0.003 | 21.606 |
|  |  | rs1168104 | 0.017 | 0.675 | 0.012 | 0.163 | 0.003 | 20.877 |
|  |  | rs1253283 | 0.016 | 0.048 | 0.029 | 0.584 | 0.003 | 19.660 |
|  |  | rs12548274 | -0.041 | 0.101 | 0.021 | 0.047 | 0.003 | 22.095 |
|  |  | rs141509040 | -0.001 | 0.056 | 0.026 | 0.973 | 0.003 | 20.086 |
|  |  | rs1609446 | -0.014 | 0.115 | 0.019 | 0.481 | 0.003 | 19.967 |
|  |  | rs17231506 | -0.032 | 0.318 | 0.012 | 0.008 | 0.004 | 27.186 |
|  |  | rs174566 | -0.018 | 0.352 | 0.012 | 0.142 | 0.032 | 237.533 |
|  |  | rs1800588 | -0.015 | 0.229 | 0.014 | 0.280 | 0.005 | 35.068 |
|  |  | rs192537901 | -0.077 | 0.014 | 0.060 | 0.195 | 0.003 | 23.785 |
|  |  | rs2126260 | -0.028 | 0.789 | 0.014 | 0.044 | 0.003 | 22.916 |
|  |  | rs34489224 | 0.008 | 0.266 | 0.014 | 0.556 | 0.003 | 21.413 |
|  |  | rs422137 | -0.013 | 0.444 | 0.012 | 0.260 | 0.004 | 25.903 |
|  |  | rs4478142 | -0.003 | 0.239 | 0.014 | 0.830 | 0.003 | 20.039 |
|  |  | rs4840981 | -0.030 | 0.976 | 0.040 | 0.450 | 0.003 | 21.265 |
|  |  | rs57518551 | 0.003 | 0.112 | 0.019 | 0.862 | 0.004 | 25.838 |
|  |  | rs6498540 | 0.008 | 0.315 | 0.012 | 0.500 | 0.004 | 25.828 |
|  |  | rs72747028 | 0.003 | 0.064 | 0.025 | 0.891 | 0.003 | 21.691 |
|  |  | rs73058910 | -0.020 | 0.047 | 0.034 | 0.544 | 0.003 | 21.390 |
|  |  | rs7437408 | -0.015 | 0.494 | 0.011 | 0.188 | 0.003 | 21.048 |
|  |  | rs78592970 | 0.003 | 0.147 | 0.018 | 0.883 | 0.004 | 25.234 |
|  |  | rs964184 | -0.011 | 0.847 | 0.016 | 0.496 | 0.006 | 44.464 |
| Phosphatidylcholine (18:1_20:4) | CD6 | rs1077834 | 0.004 | 0.234 | 0.014 | 0.796 | 0.004 | 26.370 |
|  |  | rs10793123 | 0.016 | 0.472 | 0.012 | 0.192 | 0.003 | 20.821 |
|  |  | rs10889353 | 0.000 | 0.322 | 0.012 | 0.981 | 0.003 | 20.530 |
|  |  | rs113049169 | -0.031 | 0.035 | 0.037 | 0.396 | 0.003 | 19.680 |
|  |  | rs113394924 | 0.024 | 0.040 | 0.032 | 0.453 | 0.005 | 39.462 |
|  |  | rs113913626 | -0.029 | 0.019 | 0.049 | 0.548 | 0.003 | 19.533 |
|  |  | rs116814948 | 0.018 | 0.022 | 0.042 | 0.674 | 0.003 | 20.447 |
|  |  | rs147549994 | 0.098 | 0.014 | 0.062 | 0.112 | 0.004 | 26.191 |
|  |  | rs147797702 | -0.135 | 0.013 | 0.060 | 0.025 | 0.004 | 30.191 |
|  |  | rs173539 | -0.023 | 0.328 | 0.012 | 0.058 | 0.003 | 21.167 |
|  |  | rs174527 | 0.022 | 0.402 | 0.013 | 0.092 | 0.012 | 89.070 |
|  |  | rs174537 | 0.027 | 0.348 | 0.012 | 0.024 | 0.123 | 1006.817 |
|  |  | rs187429064 | 0.065 | 0.025 | 0.053 | 0.216 | 0.007 | 53.611 |
|  |  | rs2943813 | 0.056 | 0.792 | 0.014 | 0.000 | 0.006 | 40.322 |
|  |  | rs3018617 | 0.024 | 0.352 | 0.013 | 0.072 | 0.003 | 23.807 |
|  |  | rs4628166 | -0.004 | 0.703 | 0.013 | 0.757 | 0.003 | 19.699 |
|  |  | rs4846905 | -0.009 | 0.809 | 0.015 | 0.557 | 0.003 | 23.180 |
|  |  | rs531117 | 0.027 | 0.186 | 0.015 | 0.074 | 0.005 | 32.924 |
|  |  | rs6083838 | 0.007 | 0.528 | 0.012 | 0.520 | 0.003 | 19.625 |
|  |  | rs61978272 | 0.018 | 0.281 | 0.013 | 0.177 | 0.004 | 27.749 |
|  |  | rs641738 | 0.006 | 0.566 | 0.012 | 0.611 | 0.003 | 24.969 |
|  |  | rs6850591 | 0.017 | 0.536 | 0.012 | 0.156 | 0.003 | 23.007 |
|  |  | rs73176681 | -0.021 | 0.034 | 0.034 | 0.545 | 0.003 | 20.807 |
|  |  | rs73618588 | 0.005 | 0.068 | 0.024 | 0.825 | 0.003 | 20.508 |
|  |  | rs75976069 | -0.059 | 0.021 | 0.050 | 0.235 | 0.003 | 23.447 |
|  |  | rs76582138 | -0.024 | 0.022 | 0.046 | 0.593 | 0.003 | 20.340 |
|  |  | rs79113397 | 0.044 | 0.130 | 0.017 | 0.012 | 0.003 | 24.493 |
|  |  | rs793864 | 0.012 | 0.818 | 0.016 | 0.437 | 0.003 | 21.873 |
|  |  | rs8107974 | -0.007 | 0.081 | 0.022 | 0.750 | 0.004 | 30.771 |
| Sphingomyelin (d38:1) | CCL4 | rs114483871 | 0.041 | 0.027 | 0.043 | 0.337 | 0.005 | 35.878 |
|  |  | rs11591147 | 0.032 | 0.019 | 0.047 | 0.494 | 0.006 | 41.800 |
|  |  | rs117146578 | -0.044 | 0.070 | 0.023 | 0.060 | 0.003 | 21.677 |
|  |  | rs117643293 | 0.110 | 0.029 | 0.041 | 0.008 | 0.007 | 51.391 |
|  |  | rs118070067 | 0.055 | 0.029 | 0.039 | 0.155 | 0.003 | 20.131 |
|  |  | rs12462619 | 0.011 | 0.204 | 0.016 | 0.490 | 0.003 | 24.935 |
|  |  | rs12480916 | -0.003 | 0.408 | 0.012 | 0.821 | 0.003 | 20.671 |
|  |  | rs12675806 | -0.059 | 0.093 | 0.025 | 0.020 | 0.003 | 20.931 |
|  |  | rs13258507 | -0.008 | 0.193 | 0.015 | 0.605 | 0.003 | 22.698 |
|  |  | rs144440646 | -0.018 | 0.016 | 0.062 | 0.769 | 0.003 | 23.130 |
|  |  | rs1452773 | -0.013 | 0.140 | 0.017 | 0.442 | 0.003 | 21.171 |
|  |  | rs146127446 | 0.020 | 0.504 | 0.014 | 0.153 | 0.003 | 19.931 |
|  |  | rs147426822 | -0.085 | 0.030 | 0.040 | 0.033 | 0.003 | 20.872 |
|  |  | rs182695896 | -0.044 | 0.009 | 0.072 | 0.537 | 0.007 | 49.183 |
|  |  | rs186039163 | -0.001 | 0.012 | 0.069 | 0.992 | 0.003 | 20.617 |
|  |  | rs188346987 | 0.035 | 0.036 | 0.036 | 0.330 | 0.003 | 20.311 |
|  |  | rs200949196 | 0.016 | 0.015 | 0.069 | 0.819 | 0.003 | 22.596 |
|  |  | rs2867985 | 0.025 | 0.310 | 0.013 | 0.053 | 0.003 | 20.244 |
|  |  | rs364585 | 0.002 | 0.621 | 0.012 | 0.881 | 0.012 | 88.272 |
|  |  | rs374071816 | -0.064 | 0.085 | 0.025 | 0.011 | 0.004 | 27.479 |
|  |  | rs3741111 | -0.004 | 0.507 | 0.012 | 0.743 | 0.004 | 26.904 |
|  |  | rs715119 | 0.005 | 0.239 | 0.014 | 0.723 | 0.003 | 21.776 |
|  |  | rs7157785 | 0.005 | 0.156 | 0.017 | 0.777 | 0.030 | 223.000 |
|  |  | rs7172051 | -0.009 | 0.447 | 0.012 | 0.438 | 0.003 | 19.669 |
|  |  | rs7247433 | 0.014 | 0.399 | 0.013 | 0.288 | 0.003 | 24.016 |
|  |  | rs7248003 | 0.003 | 0.684 | 0.014 | 0.856 | 0.005 | 32.942 |
|  |  | rs7258249 | -0.004 | 0.464 | 0.012 | 0.751 | 0.022 | 164.594 |
|  |  | rs73073606 | 0.000 | 0.299 | 0.015 | 0.984 | 0.003 | 22.957 |
|  |  | rs73193962 | -0.018 | 0.034 | 0.036 | 0.613 | 0.003 | 20.387 |
|  |  | rs7412 | 0.046 | 0.078 | 0.023 | 0.043 | 0.003 | 22.372 |
|  |  | rs7478167 | 0.020 | 0.657 | 0.013 | 0.115 | 0.003 | 21.626 |
|  |  | rs75679663 | 0.024 | 0.023 | 0.058 | 0.673 | 0.011 | 78.660 |
|  |  | rs75740927 | -0.054 | 0.026 | 0.043 | 0.204 | 0.003 | 20.149 |
|  |  | rs76174849 | -0.005 | 0.021 | 0.045 | 0.905 | 0.003 | 21.582 |
|  |  | rs76596580 | 0.006 | 0.116 | 0.019 | 0.768 | 0.003 | 21.031 |
|  |  | rs77645768 | 0.003 | 0.023 | 0.047 | 0.956 | 0.004 | 32.079 |
|  |  | rs79231873 | -0.027 | 0.030 | 0.039 | 0.488 | 0.003 | 20.118 |
|  |  | rs7970512 | -0.068 | 0.026 | 0.042 | 0.103 | 0.003 | 22.221 |
| Sphingomyelin (d38:1) | CD6 | rs114483871 | 0.034 | 0.027 | 0.043 | 0.425 | 0.005 | 35.878 |
|  |  | rs11591147 | 0.126 | 0.019 | 0.047 | 0.007 | 0.006 | 41.800 |
|  |  | rs117146578 | -0.024 | 0.070 | 0.023 | 0.291 | 0.003 | 21.677 |
|  |  | rs117643293 | 0.031 | 0.029 | 0.041 | 0.454 | 0.007 | 51.391 |
|  |  | rs118070067 | 0.078 | 0.029 | 0.039 | 0.042 | 0.003 | 20.131 |
|  |  | rs12462619 | 0.006 | 0.204 | 0.015 | 0.711 | 0.003 | 24.935 |
|  |  | rs12480916 | -0.015 | 0.409 | 0.012 | 0.195 | 0.003 | 20.671 |
|  |  | rs12675806 | 0.001 | 0.093 | 0.025 | 0.975 | 0.003 | 20.931 |
|  |  | rs13258507 | 0.004 | 0.193 | 0.015 | 0.801 | 0.003 | 22.698 |
|  |  | rs144440646 | 0.020 | 0.016 | 0.063 | 0.756 | 0.003 | 23.130 |
|  |  | rs1452773 | 0.006 | 0.140 | 0.017 | 0.730 | 0.003 | 21.171 |
|  |  | rs146127446 | -0.015 | 0.503 | 0.014 | 0.264 | 0.003 | 19.931 |
|  |  | rs147426822 | -0.059 | 0.030 | 0.040 | 0.139 | 0.003 | 20.872 |
|  |  | rs182695896 | -0.092 | 0.009 | 0.071 | 0.196 | 0.007 | 49.183 |
|  |  | rs186039163 | 0.045 | 0.012 | 0.070 | 0.524 | 0.003 | 20.617 |
|  |  | rs188346987 | 0.059 | 0.036 | 0.036 | 0.102 | 0.003 | 20.311 |
|  |  | rs200949196 | -0.074 | 0.015 | 0.069 | 0.281 | 0.003 | 22.596 |
|  |  | rs2867985 | -0.001 | 0.310 | 0.013 | 0.931 | 0.003 | 20.244 |
|  |  | rs364585 | 0.008 | 0.622 | 0.012 | 0.529 | 0.012 | 88.272 |
|  |  | rs374071816 | -0.042 | 0.084 | 0.025 | 0.095 | 0.004 | 27.479 |
|  |  | rs3741111 | 0.009 | 0.508 | 0.012 | 0.461 | 0.004 | 26.904 |
|  |  | rs715119 | 0.006 | 0.239 | 0.014 | 0.677 | 0.003 | 21.776 |
|  |  | rs7157785 | 0.010 | 0.156 | 0.017 | 0.569 | 0.030 | 223.000 |
|  |  | rs7172051 | -0.012 | 0.447 | 0.012 | 0.334 | 0.003 | 19.669 |
|  |  | rs7247433 | 0.010 | 0.399 | 0.013 | 0.441 | 0.003 | 24.016 |
|  |  | rs7248003 | 0.002 | 0.684 | 0.014 | 0.896 | 0.005 | 32.942 |
|  |  | rs7258249 | -0.016 | 0.463 | 0.012 | 0.190 | 0.022 | 164.594 |
|  |  | rs73073606 | 0.005 | 0.299 | 0.015 | 0.739 | 0.003 | 22.957 |
|  |  | rs73193962 | -0.055 | 0.034 | 0.036 | 0.128 | 0.003 | 20.387 |
|  |  | rs7412 | 0.006 | 0.077 | 0.022 | 0.791 | 0.003 | 22.372 |
|  |  | rs7478167 | 0.027 | 0.657 | 0.013 | 0.034 | 0.003 | 21.626 |
|  |  | rs75679663 | -0.056 | 0.023 | 0.058 | 0.331 | 0.011 | 78.660 |
|  |  | rs75740927 | 0.026 | 0.025 | 0.042 | 0.544 | 0.003 | 20.149 |
|  |  | rs76174849 | -0.050 | 0.021 | 0.044 | 0.264 | 0.003 | 21.582 |
|  |  | rs76596580 | 0.018 | 0.116 | 0.019 | 0.351 | 0.003 | 21.031 |
|  |  | rs77645768 | -0.066 | 0.023 | 0.047 | 0.159 | 0.004 | 32.079 |
|  |  | rs79231873 | -0.068 | 0.030 | 0.039 | 0.079 | 0.003 | 20.118 |
|  |  | rs7970512 | -0.035 | 0.026 | 0.041 | 0.401 | 0.003 | 22.221 |
| Sphingomyelin (d42:2) | CD6 | rs10252648 | -0.062 | 0.962 | 0.032 | 0.051 | 0.003 | 19.542 |
|  |  | rs112585713 | 0.020 | 0.025 | 0.046 | 0.661 | 0.005 | 36.193 |
|  |  | rs114483871 | 0.034 | 0.027 | 0.043 | 0.425 | 0.005 | 38.862 |
|  |  | rs115350073 | 0.001 | 0.016 | 0.053 | 0.980 | 0.003 | 21.517 |
|  |  | rs11591147 | 0.126 | 0.019 | 0.047 | 0.007 | 0.008 | 60.504 |
|  |  | rs11610836 | -0.031 | 0.401 | 0.012 | 0.008 | 0.003 | 22.885 |
|  |  | rs116893213 | -0.020 | 0.033 | 0.038 | 0.607 | 0.004 | 25.657 |
|  |  | rs116919723 | 0.037 | 0.016 | 0.057 | 0.520 | 0.003 | 20.069 |
|  |  | rs118068660 | 0.019 | 0.095 | 0.022 | 0.374 | 0.008 | 60.902 |
|  |  | rs12530474 | -0.014 | 0.109 | 0.019 | 0.460 | 0.003 | 21.039 |
|  |  | rs1367117 | -0.021 | 0.311 | 0.012 | 0.084 | 0.004 | 29.151 |
|  |  | rs139474488 | -0.037 | 0.022 | 0.048 | 0.448 | 0.003 | 20.509 |
|  |  | rs146331166 | 0.030 | 0.025 | 0.042 | 0.465 | 0.006 | 40.090 |
|  |  | rs17018185 | -0.006 | 0.221 | 0.014 | 0.653 | 0.003 | 19.894 |
|  |  | rs17110517 | 0.023 | 0.051 | 0.029 | 0.437 | 0.003 | 22.389 |
|  |  | rs17145750 | 0.004 | 0.155 | 0.016 | 0.829 | 0.004 | 30.720 |
|  |  | rs17261772 | -0.021 | 0.287 | 0.015 | 0.169 | 0.004 | 25.763 |
|  |  | rs17276933 | 0.012 | 0.077 | 0.022 | 0.593 | 0.003 | 22.524 |
|  |  | rs182695896 | -0.092 | 0.009 | 0.071 | 0.196 | 0.007 | 52.244 |
|  |  | rs184845520 | 0.061 | 0.010 | 0.101 | 0.544 | 0.003 | 21.069 |
|  |  | rs2034007 | 0.004 | 0.270 | 0.013 | 0.792 | 0.003 | 20.080 |
|  |  | rs2177333 | 0.017 | 0.579 | 0.012 | 0.144 | 0.003 | 20.018 |
|  |  | rs3751934 | -0.004 | 0.420 | 0.015 | 0.789 | 0.003 | 20.746 |
|  |  | rs3912622 | -0.004 | 0.254 | 0.013 | 0.794 | 0.004 | 27.078 |
|  |  | rs4100654 | -0.006 | 0.093 | 0.020 | 0.757 | 0.003 | 21.412 |
|  |  | rs4283208 | 0.019 | 0.800 | 0.015 | 0.212 | 0.003 | 20.344 |
|  |  | rs515081 | 0.020 | 0.652 | 0.013 | 0.132 | 0.003 | 23.446 |
|  |  | rs59916403 | -0.029 | 0.355 | 0.012 | 0.020 | 0.003 | 21.364 |
|  |  | rs62274367 | -0.053 | 0.019 | 0.053 | 0.324 | 0.003 | 21.826 |
|  |  | rs6680361 | -0.010 | 0.832 | 0.016 | 0.530 | 0.003 | 21.051 |
|  |  | rs67718253 | 0.002 | 0.192 | 0.015 | 0.893 | 0.003 | 21.468 |
|  |  | rs6843498 | -0.010 | 0.344 | 0.013 | 0.423 | 0.004 | 25.306 |
|  |  | rs7104343 | -0.006 | 0.108 | 0.019 | 0.738 | 0.003 | 20.238 |
|  |  | rs7247433 | 0.010 | 0.399 | 0.013 | 0.441 | 0.003 | 20.702 |
|  |  | rs73047641 | -0.005 | 0.467 | 0.012 | 0.692 | 0.003 | 22.905 |
|  |  | rs7412 | 0.006 | 0.077 | 0.022 | 0.791 | 0.004 | 32.181 |
|  |  | rs75444922 | -0.012 | 0.049 | 0.032 | 0.708 | 0.003 | 19.641 |
|  |  | rs75679663 | -0.056 | 0.023 | 0.058 | 0.331 | 0.014 | 101.344 |
| Abbreviations: SNPs: single nucleotide polymorphisms; EA: effect allele; OA: other allele; EAF: effect allele frequency; SE: standard error. | | | | | | | | |

| **Supplementary Table S18. MR analysis results of associations between lipidome and inflammatory proteins after the final screening.** | | | | | | | | | | | | | | |
| --- | --- | --- | --- | --- | --- | --- | --- | --- | --- | --- | --- | --- | --- | --- |
| **Exposure** | **Outcome** | **Method** | **Nsnp** | **OR** | **OR_lci95** | **OR_uci95** | **P** | **Heterogeneity** | | | | **Pleiotropy** | | |
|  |  |  |  |  |  |  |  | **MR Egger** | | **IVW** | | **MR-Egger intercep** | | **MR-PRESO** |
|  |  |  |  |  |  |  |  | **Q** | **P** | **Q** | **P** | **intercept** | **P** | **P** |
| Sterol ester (27:1/20:4) | CD6 | MR Egger | 30 | 0.963 | 0.922 | 1.006 | 0.099 | 29.250 | 0.400 | 29.291 | 0.450 | 0.001 | 0.844 | 0.557 |
|  |  | Weighted median | 30 | 0.966 | 0.933 | 1.001 | 0.054 |  |  |  |  |  |  |  |
|  |  | IVW | 30 | 0.966 | 0.938 | 0.995 | 0.022 |  |  |  |  |  |  |  |
|  |  | Simple mode | 30 | 0.955 | 0.864 | 1.054 | 0.367 |  |  |  |  |  |  |  |
|  |  | Weighted mode | 30 | 0.966 | 0.932 | 1.000 | 0.062 |  |  |  |  |  |  |  |
|  |  | BWMR | 30 | 0.967 | 0.936 | 0.998 | 0.039 |  |  |  |  |  |  |  |
| Sterol ester (27:1/22:6) | CCL4 | MR Egger | 23 | 0.861 | 0.755 | 0.982 | 0.036 | 23.668 | 0.309 | 24.945 | 0.300 | 0.011 | 0.299 | 0.332 |
|  |  | Weighted median | 23 | 0.923 | 0.850 | 1.002 | 0.057 |  |  |  |  |  |  |  |
|  |  | IVW | 23 | 0.918 | 0.866 | 0.973 | 0.004 |  |  |  |  |  |  |  |
|  |  | Simple mode | 23 | 0.911 | 0.782 | 1.060 | 0.238 |  |  |  |  |  |  |  |
|  |  | Weighted mode | 23 | 0.913 | 0.818 | 1.018 | 0.117 |  |  |  |  |  |  |  |
|  |  | BWMR | 23 | 0.915 | 0.864 | 0.969 | 0.002 |  |  |  |  |  |  |  |
| Sterol ester (27:1/22:6) | CD6 | MR Egger | 23 | 0.858 | 0.759 | 0.970 | 0.023 | 19.689 | 0.541 | 22.577 | 0.426 | 0.016 | 0.104 | 0.426 |
|  |  | Weighted median | 23 | 0.956 | 0.881 | 1.038 | 0.288 |  |  |  |  |  |  |  |
|  |  | IVW | 23 | 0.944 | 0.893 | 0.997 | 0.038 |  |  |  |  |  |  |  |
|  |  | Simple mode | 23 | 0.963 | 0.822 | 1.127 | 0.642 |  |  |  |  |  |  |  |
|  |  | Weighted mode | 23 | 0.957 | 0.835 | 1.096 | 0.528 |  |  |  |  |  |  |  |
|  |  | BWMR | 23 | 0.941 | 0.887 | 0.998 | 0.041 |  |  |  |  |  |  |  |
|  |  |  |  |  |  |  |  |  |  |  |  |  |  |  |
| Phosphatidylcholine (20:4_0:0) | CD6 | MR Egger | 27 | 0.957 | 0.904 | 1.012 | 0.134 | 24.760 | 0.476 | 24.777 | 0.532 | 0.001 | 0.898 | 0.536 |
|  |  | Weighted median | 27 | 0.947 | 0.903 | 0.994 | 0.027 |  |  |  |  |  |  |  |
|  |  | IVW | 27 | 0.959 | 0.926 | 0.994 | 0.021 |  |  |  |  |  |  |  |
|  |  | Simple mode | 27 | 0.981 | 0.878 | 1.096 | 0.736 |  |  |  |  |  |  |  |
|  |  | Weighted mode | 27 | 0.951 | 0.905 | 0.998 | 0.052 |  |  |  |  |  |  |  |
|  |  | BWMR | 27 | 0.953 | 0.915 | 0.992 | 0.020 |  |  |  |  |  |  |  |
| Phosphatidylcholine (16:0_20:4) | CCL4 | MR Egger | 24 | 0.970 | 0.920 | 1.022 | 0.266 | 24.209 | 0.336 | 24.319 | 0.386 | -0.002 | 0.754 | 0.501 |
|  |  | Weighted median | 24 | 0.969 | 0.934 | 1.005 | 0.091 |  |  |  |  |  |  |  |
|  |  | IVW | 24 | 0.964 | 0.931 | 0.998 | 0.036 |  |  |  |  |  |  |  |
|  |  | Simple mode | 24 | 0.968 | 0.849 | 1.103 | 0.629 |  |  |  |  |  |  |  |
|  |  | Weighted mode | 24 | 0.968 | 0.930 | 1.007 | 0.118 |  |  |  |  |  |  |  |
|  |  | BWMR | 24 | 0.963 | 0.932 | 0.996 | 0.028 |  |  |  |  |  |  |  |
| Phosphatidylcholine (16:0_20:4) | CD6 | MR Egger | 24 | 0.966 | 0.909 | 1.027 | 0.284 | 33.808 | 0.051 | 34.651 | 0.056 | -0.005 | 0.467 | 0.121 |
|  |  | Weighted median | 24 | 0.954 | 0.918 | 0.991 | 0.016 |  |  |  |  |  |  |  |
|  |  | IVW | 24 | 0.950 | 0.912 | 0.989 | 0.013 |  |  |  |  |  |  |  |
|  |  | Simple mode | 24 | 1.043 | 0.913 | 1.190 | 0.542 |  |  |  |  |  |  |  |
|  |  | Weighted mode | 24 | 0.956 | 0.917 | 0.997 | 0.048 |  |  |  |  |  |  |  |
|  |  | BWMR | 24 | 0.949 | 0.909 | 0.991 | 0.018 |  |  |  |  |  |  |  |
| Phosphatidylcholine (17:0_18:2) | CCL4 | MR Egger | 25 | 1.080 | 0.957 | 1.218 | 0.223 | 13.824 | 0.932 | 14.060 | 0.945 | -0.004 | 0.632 | 0.946 |
|  |  | Weighted median | 25 | 1.076 | 0.997 | 1.162 | 0.061 |  |  |  |  |  |  |  |
|  |  | IVW | 25 | 1.051 | 1.001 | 1.104 | 0.047 |  |  |  |  |  |  |  |
|  |  | Simple mode | 25 | 1.088 | 0.965 | 1.226 | 0.182 |  |  |  |  |  |  |  |
|  |  | Weighted mode | 25 | 1.080 | 0.989 | 1.179 | 0.100 |  |  |  |  |  |  |  |
|  |  | BWMR | 25 | 1.052 | 1.000 | 1.106 | 0.051 |  |  |  |  |  |  |  |
| Phosphatidylcholine (17:0_18:2) | TNFSF12 | MR Egger | 25 | 0.978 | 0.867 | 1.104 | 0.725 | 24.391 | 0.382 | 24.848 | 0.414 | -0.006 | 0.518 | 0.459 |
|  |  | Weighted median | 25 | 0.940 | 0.874 | 1.011 | 0.098 |  |  |  |  |  |  |  |
|  |  | IVW | 25 | 0.943 | 0.898 | 0.990 | 0.018 |  |  |  |  |  |  |  |
|  |  | Simple mode | 25 | 0.932 | 0.828 | 1.049 | 0.255 |  |  |  |  |  |  |  |
|  |  | Weighted mode | 25 | 0.936 | 0.857 | 1.022 | 0.151 |  |  |  |  |  |  |  |
|  |  | BWMR | 25 | 0.942 | 0.891 | 0.996 | 0.034 |  |  |  |  |  |  |  |
| Sphingomyelin (d38:1) | CCL4 | MR Egger | 38 | 0.977 | 0.894 | 1.068 | 0.611 | 47.064 | 0.103 | 47.603 | 0.114 | -0.004 | 0.525 | 0.143 |
|  |  | Weighted median | 38 | 0.984 | 0.926 | 1.045 | 0.602 |  |  |  |  |  |  |  |
|  |  | IVW | 38 | 0.953 | 0.911 | 0.996 | 0.034 |  |  |  |  |  |  |  |
|  |  | Simple mode | 38 | 0.980 | 0.882 | 1.089 | 0.711 |  |  |  |  |  |  |  |
|  |  | Weighted mode | 38 | 0.991 | 0.933 | 1.053 | 0.779 |  |  |  |  |  |  |  |
|  |  | BWMR | 38 | 0.951 | 0.912 | 0.992 | 0.018 |  |  |  |  |  |  |  |
| Sphingomyelin (d38:1) | CD6 | MR Egger | 38 | 0.967 | 0.888 | 1.053 | 0.444 | 43.696 | 0.177 | 43.811 | 0.205 | -0.002 | 0.760 | 0.214 |
|  |  | Weighted median | 38 | 0.954 | 0.896 | 1.014 | 0.131 |  |  |  |  |  |  |  |
|  |  | IVW | 38 | 0.956 | 0.916 | 0.998 | 0.038 |  |  |  |  |  |  |  |
|  |  | Simple mode | 38 | 0.931 | 0.823 | 1.055 | 0.269 |  |  |  |  |  |  |  |
|  |  | Weighted mode | 38 | 0.965 | 0.905 | 1.028 | 0.274 |  |  |  |  |  |  |  |
|  |  | BWMR | 38 | 0.955 | 0.917 | 0.995 | 0.026 |  |  |  |  |  |  |  |
| Sphingomyelin (d42:2) | CD6 | MR Egger | 38 | 0.993 | 0.908 | 1.086 | 0.875 | 36.671 | 0.438 | 38.579 | 0.398 | -0.008 | 0.180 | 0.392 |
|  |  | Weighted median | 38 | 0.951 | 0.890 | 1.015 | 0.127 |  |  |  |  |  |  |  |
|  |  | IVW | 38 | 0.941 | 0.899 | 0.984 | 0.008 |  |  |  |  |  |  |  |
|  |  | Simple mode | 38 | 0.930 | 0.808 | 1.069 | 0.314 |  |  |  |  |  |  |  |
|  |  | Weighted mode | 38 | 0.952 | 0.830 | 1.092 | 0.485 |  |  |  |  |  |  |  |
|  |  | BWMR | 38 | 0.935 | 0.891 | 0.983 | 0.008 |  |  |  |  |  |  |  |
| Abbreviations: IVW: inverse variance weighted; BWMR: Bayesian Weighted Mendelian Randomization; β:beta; OR: odds ratio. | | | | | | | | | | | | | | |

| **Supplementary Table S19. MR analysis results of associations between lipidome and inflammatory proteins after further validation analysis** | | | | | | | | | | | | | |
| --- | --- | --- | --- | --- | --- | --- | --- | --- | --- | --- | --- | --- | --- |
| **Exposure** | **Outcome** | **Method** | **OR** | **OR_lci95** | **OR_uci95** | **P** | **Heterogeneity** | | | | **Pleiotropy** | | |
|  |  |  |  |  |  |  | **MR Egger** | | **IVW** | | **MR-Egger intercep** | | **MR-PRESO** |
|  |  |  |  |  |  |  | **Q** | **P** | **Q** | **P** | **intercept** | **P** | **P** |
| Sterol ester (27:1/20:4) | CD6 | MR Egger | 0.971 | 0.914 | 1.033 | 0.369 | 16.285 | 0.234 | 16.536 | 0.282 | -0.005 | 0.661 | 0.936 |
|  |  | Weighted median | 0.966 | 0.932 | 1.001 | 0.059 |  |  |  |  |  |  |  |
|  |  | IVW | 0.960 | 0.928 | 0.994 | 0.022 |  |  |  |  |  |  |  |
|  |  | Simple mode | 0.953 | 0.868 | 1.047 | 0.331 |  |  |  |  |  |  |  |
|  |  | Weighted mode | 0.966 | 0.932 | 1.002 | 0.084 |  |  |  |  |  |  |  |
|  |  | BWMR | 0.960 | 0.930 | 0.991 | 0.013 |  |  |  |  |  |  |  |
| Sterol ester (27:1/22:6) | CCL4 | MR Egger | 0.845 | 0.676 | 1.056 | 0.199 | 2.178 | 0.824 | 2.199 | 0.901 | 0.003 | 0.890 | 0.109 |
|  |  | Weighted median | 0.864 | 0.772 | 0.966 | 0.010 |  |  |  |  |  |  |  |
|  |  | IVW | 0.858 | 0.785 | 0.937 | 0.001 |  |  |  |  |  |  |  |
|  |  | Simple mode | 0.880 | 0.749 | 1.035 | 0.173 |  |  |  |  |  |  |  |
|  |  | Weighted mode | 0.879 | 0.760 | 1.016 | 0.132 |  |  |  |  |  |  |  |
|  |  | BWMR | 0.855 | 0.778 | 0.940 | 0.001 |  |  |  |  |  |  |  |
| Sterol ester (27:1/22:6) | CD6 | MR Egger | 0.894 | 0.701 | 1.140 | 0.407 | 6.008 | 0.305 | 6.042 | 0.419 | 0.004 | 0.874 | 0.253 |
|  |  | Weighted median | 0.942 | 0.843 | 1.053 | 0.293 |  |  |  |  |  |  |  |
|  |  | IVW | 0.911 | 0.834 | 0.995 | 0.037 |  |  |  |  |  |  |  |
|  |  | Simple mode | 0.921 | 0.804 | 1.056 | 0.283 |  |  |  |  |  |  |  |
|  |  | Weighted mode | 0.940 | 0.828 | 1.068 | 0.380 |  |  |  |  |  |  |  |
|  |  | BWMR | 0.909 | 0.829 | 0.997 | 0.043 |  |  |  |  |  |  |  |
| Phosphatidylcholine (20:4_0:0) | CD6 | MR Egger | 0.997 | 0.723 | 1.403 | 0.968 | 5.492 | 0.359 | 5.936 | 0.430 | -0.016 | 0.553 | 0.572 |
|  |  | Weighted median | 0.974 | 0.864 | 1.097 | 0.662 |  |  |  |  |  |  |  |
|  |  | IVW | 0.909 | 0.830 | 0.994 | 0.037 |  |  |  |  |  |  |  |
|  |  | Simple mode | 0.975 | 0.836 | 1.136 | 0.753 |  |  |  |  |  |  |  |
|  |  | Weighted mode | 0.977 | 0.856 | 1.115 | 0.742 |  |  |  |  |  |  |  |
|  |  | BWMR | 0.906 | 0.824 | 0.995 | 0.039 |  |  |  |  |  |  |  |
| Phosphatidylcholine (16:0_20:4) | CCL4 | MR Egger | 0.982 | 0.924 | 1.042 | 0.572 | 4.072 | 0.539 | 4.932 | 0.553 | -0.010 | 0.396 | 0.148 |
|  |  | Weighted median | 0.969 | 0.932 | 1.007 | 0.107 |  |  |  |  |  |  |  |
|  |  | IVW | 0.960 | 0.924 | 0.998 | 0.038 |  |  |  |  |  |  |  |
|  |  | Simple mode | 0.940 | 0.833 | 1.060 | 0.351 |  |  |  |  |  |  |  |
|  |  | Weighted mode | 0.969 | 0.931 | 1.008 | 0.173 |  |  |  |  |  |  |  |
|  |  | BWMR | 0.960 | 0.925 | 0.996 | 0.030 |  |  |  |  |  |  |  |
| Phosphatidylcholine (16:0_20:4) | CD6 | MR Egger | 0.998 | 0.954 | 1.074 | 0.709 | 4.716 | 0.452 | 7.645 | 0.182 | -0.037 | 0.156 | 0.067 |
|  |  | Weighted median | 0.948 | 0.911 | 0.986 | 0.008 |  |  |  |  |  |  |  |
|  |  | IVW | 0.930 | 0.872 | 0.993 | 0.029 |  |  |  |  |  |  |  |
|  |  | Simple mode | 0.749 | 0.576 | 0.975 | 0.075 |  |  |  |  |  |  |  |
|  |  | Weighted mode | 0.952 | 0.913 | 0.991 | 0.055 |  |  |  |  |  |  |  |
|  |  | BWMR | 0.915 | 0.851 | 0.984 | 0.017 |  |  |  |  |  |  |  |
| Phosphatidylcholine (17:0_18:2) | CCL4 | MR Egger | 1.051 | 0.892 | 1.237 | 0.575 | 0.518 | 0.998 | 0.690 | 0.998 | 0.005 | 0.692 | 0.603 |
|  |  | Weighted median | 1.080 | 0.998 | 1.169 | 0.056 |  |  |  |  |  |  |  |
|  |  | IVW | 1.084 | 1.014 | 1.160 | 0.018 |  |  |  |  |  |  |  |
|  |  | Simple mode | 1.083 | 0.957 | 1.227 | 0.248 |  |  |  |  |  |  |  |
|  |  | Weighted mode | 1.080 | 0.987 | 1.182 | 0.136 |  |  |  |  |  |  |  |
|  |  | BWMR | 1.085 | 1.013 | 1.161 | 0.019 |  |  |  |  |  |  |  |
| Phosphatidylcholine (17:0_18:2) | TNFSF12 | MR Egger | 0.979 | 0.813 | 1.178 | 0.829 | 8.119 | 0.230 | 8.513 | 0.290 | -0.007 | 0.609 | 0.276 |
|  |  | Weighted median | 0.940 | 0.863 | 1.024 | 0.157 |  |  |  |  |  |  |  |
|  |  | IVW | 0.934 | 0.869 | 1.004 | 0.065 |  |  |  |  |  |  |  |
|  |  | Simple mode | 0.958 | 0.825 | 1.112 | 0.590 |  |  |  |  |  |  |  |
|  |  | Weighted mode | 0.938 | 0.856 | 1.027 | 0.208 |  |  |  |  |  |  |  |
|  |  | BWMR | 0.936 | 0.865 | 1.012 | 0.094 |  |  |  |  |  |  |  |
| Sphingomyelin (d38:1) | CCL4 | MR Egger | 0.970 | 0.870 | 1.082 | 0.599 | 12.735 | 0.389 | 13.024 | 0.446 | -0.005 | 0.611 | 0.093 |
|  |  | Weighted median | 0.984 | 0.919 | 1.053 | 0.633 |  |  |  |  |  |  |  |
|  |  | IVW | 0.946 | 0.899 | 0.996 | 0.033 |  |  |  |  |  |  |  |
|  |  | Simple mode | 0.962 | 0.876 | 1.056 | 0.429 |  |  |  |  |  |  |  |
|  |  | Weighted mode | 0.982 | 0.922 | 1.045 | 0.569 |  |  |  |  |  |  |  |
|  |  | BWMR | 0.946 | 0.897 | 0.996 | 0.035 |  |  |  |  |  |  |  |
| Sphingomyelin (d38:1) | CD6 | MR Egger | 0.919 | 0.827 | 1.021 | 0.142 | 9.620 | 0.649 | 9.622 | 0.724 | 0.001 | 0.960 | 0.586 |
|  |  | Weighted median | 0.943 | 0.884 | 1.006 | 0.075 |  |  |  |  |  |  |  |
|  |  | IVW | 0.921 | 0.875 | 0.969 | 0.002 |  |  |  |  |  |  |  |
|  |  | Simple mode | 0.915 | 0.819 | 1.022 | 0.141 |  |  |  |  |  |  |  |
|  |  | Weighted mode | 0.950 | 0.890 | 1.014 | 0.146 |  |  |  |  |  |  |  |
|  |  | BWMR | 0.921 | 0.874 | 0.970 | 0.002 |  |  |  |  |  |  |  |
| Sphingomyelin (d42:2) | CD6 | MR Egger | 0.876 | 0.755 | 1.017 | 0.107 | 6.671 | 0.879 | 6.709 | 0.917 | 0.002 | 0.848 | 0.124 |
|  |  | Weighted median | 0.908 | 0.829 | 0.995 | 0.040 |  |  |  |  |  |  |  |
|  |  | IVW | 0.888 | 0.828 | 0.951 | 0.001 |  |  |  |  |  |  |  |
|  |  | Simple mode | 0.917 | 0.795 | 1.058 | 0.254 |  |  |  |  |  |  |  |
|  |  | Weighted mode | 0.923 | 0.811 | 1.050 | 0.244 |  |  |  |  |  |  |  |
|  |  | BWMR | 0.887 | 0.825 | 0.953 | 0.001 |  |  |  |  |  |  |  |
| Abbreviations: IVW: inverse variance weighted; BWMR: Bayesian Weighted Mendelian Randomization; β:beta; OR: odds ratio. | | | | | | | | | | | | | |

| **Supplementary Table S20: Results of mediation analysis under a more stringent SNP selection threshold (P < 1 × 10⁻⁶)** | | | | | | | | |
| --- | --- | --- | --- | --- | --- | --- | --- | --- |
| **Exposure** | **Mediation** | **Outcome** | **β** | **β1** | **β2** | **Mediation effect** | **Direct effect** | **Mediation** |
|  |  |  |  |  |  |  |  | **effect/β** |
| Sterol ester (27:1/22:6) | CCL4 | IBD | -0.109 | -0.153 | 0.111 | -0.017 | -0.092 | 0.1565 |
| Sterol ester (27:1/22:6) | CD6 | IBD | -0.109 | -0.093 | 0.122 | -0.011 | -0.098 | 0.1043 |
| Phosphatidylcholine (20:4_0:0) | CD6 | IBD | -0.148 | -0.096 | 0.122 | -0.012 | -0.136 | 0.0791 |
| Phosphatidylcholine (16:0_20:4) | CCL4 | IBD | -0.129 | -0.04 | 0.111 | -0.005 | -0.124 | 0.035 |
| Phosphatidylcholine (16:0_20:4) | CD6 | IBD | -0.129 | -0.072 | 0.122 | -0.009 | -0.12 | 0.0685 |
| Phosphatidylcholine (17:0_18:2) | CCL4 | IBD | 0.15 | 0.081 | 0.111 | 0.009 | 0.141 | 0.0601 |
| Sphingomyelin (d38:1) | CCL4 | IBD | -0.105 | -0.056 | 0.111 | -0.006 | -0.099 | 0.0592 |
| Sphingomyelin (d38:1) | CD6 | IBD | -0.105 | -0.082 | 0.122 | -0.01 | -0.095 | 0.0956 |
| Sphingomyelin (d42:2) | CD6 | IBD | -0.122 | -0.119 | 0.122 | -0.015 | -0.108 | 0.1190 |
| Phosphatidylcholine (16:0_20:4) | CCL4 | UC | -0.142 | -0.04 | 0.259 | -0.01 | -0.131 | 0.0738 |
| Phosphatidylcholine (17:0_18:2) | CCL4 | UC | 0.281 | 0.081 | 0.259 | 0.021 | 0.26 | 0.0747 |
| Sphingomyelin (d38:1) | CCL4 | UC | -0.097 | -0.056 | 0.259 | -0.014 | -0.083 | 0.1479 |
| Sterol ester (27:1/20:4) | CD6 | CD | -0.146 | -0.041 | 0.242 | -0.01 | -0.137 | 0.067 |
| Phosphatidylcholine (20:4_0:0) | CD6 | CD | -0.132 | -0.096 | 0.242 | -0.023 | -0.108 | 0.1762 |
| Phosphatidylcholine (16:0_20:4) | CD6 | CD | -0.137 | -0.072 | 0.242 | -0.018 | -0.12 | 0.1277 |
